# Allocation factors for meat coproducts: Dataset to perform life cycle assessment at slaughterhouse

**DOI:** 10.1016/j.dib.2020.106558

**Published:** 2020-11-23

**Authors:** Samuel Le Féon, Joël Aubin, Armelle Gac, Christophe Lapasin, Aurélie Wilfart

**Affiliations:** a Independent Researcher in Environmental Assessment, Pépinière ESS, 23 rue des Chênes, 35630 Langouët, France; b SAS, INRAE, Institut Agro, 35000 Rennes, France; c Institut de l’élevage, 35650 le Rheu, France; d Célene, 17, place des vins de France 75012 Paris, France

**Keywords:** Beef, Calf, Lamb, Life cycle assessment, Biophysical allocation, Mass allocation, Economic allocation

## Abstract

The sharing of total environmental impacts between the different products of a multi-output system is crucial in Life Cycle Assessment. ISO standards recommend subdivision then substitution methods when possible. Sometimes, allocations rules are necessary. They consist of allocating the total impact to the different products in proportion to a value that characterize the products. They can be based on physical parameters (such as mass, protein, dry matter, etc.) or the economic value of coproducts can be used as a proxy. As they are based on various type of parameters, allocation rules can lead to significantly different environmental impact results. Then a consensus is difficult to reach between stakeholders as for example in meat sector. To make the debate going further, Chen et al. (2017) proposed a new allocation method based on biophysical parameters (Chen et al., 2017). Adapted from previous methods, they propose to allocate impacts in proportion to the energy needed for the growth, the maintenance and the activity of each tissue. The method has been judged as scientifically viable but also particularly difficult to apply due to the amount of necessary data and to the complexity of the calculation model. In a recent project, we developed a freeware to easily calculate biophysical allocation factors as well as mass and economic factors to allow a fair comparison: MeatPartTool. We also collected data to create a dataset of mass, economic and biophysical allocation factors for a large range of beef (132 individuals), calf (54 individuals) and lamb (14 individuals) at the slaughterhouse stage. This data paper provides both primary data and calculated allocation factors.

## Specifications Table

**Table:** 

Subject	Environmental Science – Environmental Impact Assessment
Specific subject area	Allocation factors for Life Cycle Assessment of meat coproducts
Type of data	Table (raw and calculated data)
How data were acquired	- Model input data: grey and scientific literature, expert interviews - Allocation factors: calculated by Chen et al. (2017) models with the MeatParlTool freeware [7]
Data format	Raw Calculated
Parameters for data collection	Experts have been solicited to provide and control primary data (i.e. model inputs). Most of them were already validated in other projects. Allocation factors were calculated using a model that have been published and peer-reviewed [1].
Description of data collection	Primary data (i.e. model inputs) were collected during a specific research project. They were provided and discussed by the different partners of the project. Most of them were obtained in the context of previous projects. Some adaptations were necessary to ensure the homogeneity of the present dataset. These modifications are detailed in this paper. Calculated data were obtained by applying the model developed by Chen et al. (2017) for biophysical allocation factors and directly calculated from mass (based on wet-mass) and economic values for mass and economic allocation factors.
Data source location	Institutions: INRAE, IDELE, CELENE, INTERBEV Country: France
Data accessibility	Repository name: Data Inrae Direct URL to raw data: https://doi.org/10.15454/552QFN To access to specific allocation factors: open the raw dataset (https://doi.org/10.15454/552QFN) with MeatPartTool freeware (https://doi.org/10.15454/AIMYFG) Other direct URL to raw data (in French): https://www6.inrae.fr/means/Outils-d-analyze-multicritere/MeatPartTool/Les-bases-de-donnees

## Value of the Data

• These data are useful as they move the debate on allocation factors for LCA of meat forward. There is no consensus between stakeholders on the subject when choosing between allocation methods. The lack of data however makes methods difficult to use and compare. Here is proposed an unprecedented range of mass, economic and biophysical allocation factors for meat coproducts.
• This dataset will benefit to everyone who wants to practice LCA to meat products at slaughter stage. Researchers, industrials, decision-makers are interested to better understand environmental impacts of meat. If they cannot calculate their own allocation factors, they can pick the most appropriate ones in this dataset. Furthermore, by proposing both mass, economic and biophysical allocation factors, the dataset sets political questions aside but offers material to discuss.
• These data can be directly used to allocate environmental impacts between meat coproducts at slaughter stage. The vast range of individuals proposed allows the user to choose appropriate allocation factors instead of generic ones. Furthermore, as both mass, economic and biophysical allocation factors are calculated, the user will be able to easily provide sensitivity analysis when using one or another.
• This dataset offers a large range of mass, economic and biophysical allocation factors that were not available so far in literature. From now, only a few ones existed, mostly generic cases. This is the beginning towards more differentiated datasets appropriated to different realities. The authors think that this dataset should be completed by other individuals, especially from different geographical areas. To help, we developed a freeware that calculates mass, economic and biophysical allocation factors by mixing input data provided by the user and possibly default data if the user miss some.

## 1 Data Description

### 1.1 Input data

Primary data (i.e. all the dataset necessary to calculate allocation factors) have been collected from different sources: literature, previous projects and expert interviews. In total, the dataset comprises 132 beef, 54 calves and 14 lambs (Table 1).

**Table 1:** List of animals in the dataset.

Species	Breeds	Categories	Rearing modes
Beef	Primholstein	Young Bull	Pasture
	Charolaise	Heifer	Stall
	Limousine	Cull Cow	Grazing large area
	Blonde d'Aquitaine	Beef	
	Salers		
	Rouge des prés		
	Charolaise x Rustique		
	Montbéliarde		
	Normande		
	Charolaise x Pie Noire		
	Average		
Calves	Primholstein	Milk-fed veal	Pasture
	Charolaise	Rosé Veal	Stall
	Blonde d'Aquitaine		Grazing large area
	Limousine		
	Normande		
	Aubrac		
	Montbéliarde		
	Cow-calf generic		
	Dairy generic		
	Croisé-lait		
	Croisé-viande		
	Average		
Lambs	Milk-fed heavy lamb		Housed ewes
	Milk-fed hardy lamb		Grazing flat pasture
	Grass-fed heavy lamb		Grazing hilly pasture
	Milk lamb		Housed fattening lambs
	Average		

For each species, the list of coproducts has been drawn up. For a given species, it is considered that every breed comprises the same coproducts. Each coproduct is then classified by:

- Destination:
  ○ Human Food
  ○ Pet Food
  ○ PAP C3 (animal by-products, blood, etc.)
  ○ Gelatin C3 (bones, tendons)
  ○ Skin Tannery C3 (skin, mask)
  ○ Fat and Greaves C3 (fat, tallow)
  ○ C1-C2 for disposal
  ○ Spreading/Compost
- Group of tissues
  ○ Carcass
  ○ GIT (stomach, intestines, etc.)
  ○ Liver
  ○ Others
  ○ Whole Body

To complete, in Europe:

- C1 products are those that presents risks of:
  ○ Spongiform encephalopathy transmission;
  ○ Presence of residues of toxic substances;
  ○ Presence of environmental contaminants.
- C2 products are those coming from digestive system that present health hazards
- C3 products are free of risks and used as intrants for industrial production (for example petfood or fertilizers)

C1 and C2 products are generally discarded.

Lists of coproducts and associated destinations and groups of tissues for bovine, calf and ovine are respectively available in Table 2, Table 3, Table 4. These tables also contain, for each coproduct, the percentages of Water, Dry Matter, Lipids and Proteins. These are the same for every breed of a given species in the present dataset. Those data concerning quantity of coproducts and their physicochemical compositions were compiled by Gac et al. (2012) considering bibliographic references, supplemented by extrapolations and expert estimates when information was lacking.

**Table 2:** Destinations, group of tissues and composition of beef coproducts (from Gac et al. (2012)).

Co-produits	Destination	Group of tissues	Water (%)	DRY MATTER (%)	Lipids (%)	Protéins (%)
Abomasum	Human food	GIT	75	25	5	20
Abomasum fat	Fat and greaves C3	GIT	20	80	75	5
Aponeurosis	Human food	Carcass	75	25	2	23
Bile	PAP C3	Others	90	10	2	8
Blood	PAP C3	Others	80	20	2	18
Blood	Pet food	Others	80	20	2	18
Bones	Gelatin C3	Carcass	60	40	15	15
Bones of head, brain, eyes and teeth	C1-C2 for disposal	Others	62	38	2	30
Cheek	Human food	Others	75	25	3	21
Cheek	Human food	Others	75	25	3	21
Cheek trimmings	Pet food	Others	75	25	3	21
Chops	Pet food	Others	68	32	2	30
Contents of intestines	Spreading/Compost	GIT	85	15	13	2
Contents of therumen	Spreading/Compost	GIT	68	32	29	3
Ears	PAP C3	Others	65	35	10	24
Esophagus	Pet food	Others	75	25	4	20
Fat	Fat and greaves C3	Carcass	10	90	88	2
Fat around heart	Fat and greaves C3	Others	10	90	88	2
Fat in the kidney	Fat and greaves C3	Others	10	90	88	2
Feet (without hooves)	Gelatin C3	Others	69	31	5	20
Floatation fat	Spreading/Compost	Others	15	85	84	1
Forehead	C1-C2 for disposal	Others	68	32	2	30
Forelock	PAP C3	Others	20	80	0	79
Gallbladder	Pet food	Others	75	25	5	20
Head trimmings	Pet food	Others	75	25	3	21
Heart	Human food	Others	75	25	3	20
Heart trimmings	Pet food	Others	75	25	3	21
Hide	Skin tannery C3	Others	68	32	2	30
Hooves	PAP C3	Others	20	80	0	79
Horns	PAP C3	Others	20	80	0	79
Kidney	Human food	Others	75	25	2	21
Large intestine	C1-C2 for disposal	GIT	75	25	5	20
Liver	Human food	Liver	70	30	5	20
Liver trimmings	Pet food	Liver	75	25	3	21
Lower jaw	PAP C3	Others	60	40	15	15
Lungs	Pet food	Others	74	26	1	25
Mask	Skin tannery C3	Others	68	32	2	30
Mesenteric fat	C1-C2 for disposal	GIT	10	90	88	2
Muscle	Human food	Carcass	76	24	5	20
Muzzle	Human food	Others	68	32	2	30
Omasum	Human food	GIT	75	25	5	20
Omasum fat	Fat and greaves C3	GIT	20	80	75	5
Rumen and forestomach	Human food	GIT	75	25	5	20
Rumen fat	Fat and greaves C3	GIT	10	80	75	5
Sanitary seizures	C1-C2 for disposal	Others	66	34	16	17
Screening and sifting wastes	C1-C2 for disposal	Others	15	85	84	1
Small intestine	PAP C3	GIT	75	25	5	20
Spinal cord	C1-C2 for disposal	Others	75	25	10	10
Spinal cord waste	C1-C2 for disposal	Others	75	25	10	10
Spine	C1-C2 for disposal	Carcass	60	40	15	15
Spleen	Pet food	Others	75	25	4	21
Stillborn	PAP C3	GIT	75	25	2	21
Tallow	Fat and greaves C3	Others	10	90	88	2
Tongue	Human food	Others	72	28	10	16
Tonsil	C1-C2 for disposal	Others	75	25	12	10
Trachea	Pet food	Others	65	35	5	29
Udder	Pet food	Others	86	14	5	3
Upper throat	Pet food	Others	70	30	5	20
Water in the rumen	Spreading/Compost	GIT	99	1	0	3

**Table 3:** Destinations, group of tissues and composition of calf coproducts (from Gac et al. (2012)).

Co-produits	Destination	Group of tissues	Water (%)	DRY MATTER (%)	Lipids (%)	Protéins (%)
Abomasum	Human food	Others	75	25	5	20
Aponevrosis (1%)	Human food	Carcass	70	21	4	25
Bile	PAP C3	Others	90	10	2	8
Blood	C1-C2 for disposal	Others	80	20	2	18
Bones (11%)	Gelatin C3	Carcass	65	35	2	25
Dead individuals	C1-C2 for disposal	Others	80	20	4	10
Fat (8%)	Fat and greaves C3	Carcass	10	90	88	2
Fat from breasts and penis	Fat and greaves C3	Others	10	90	88	2
Feet (without hooves)	Human food	Others	69	31	5	20
Floatation fat	C1-C2 for disposal	Others	15	85	84	1
Head	Human food	Others	68	32	5	23
Intestines	C1-C2 for disposal	Others	75	25	5	20
Kidney	Human food	Others	75	25	2	21
Manure	Spreading/Compost	Others	0	0	0	0
Meat	Human food	Carcass	75	25	4	20
Pluck	Human food	Others	72	28	4	22
Rumen and forestomach	Human food	Others	75	25	5	20
SPA C3	PAP C3	Others	99	1	0	1
Screening and sifting wastes	C1-C2 for disposal	Others	15	85	84	1
Skin	Skin tannery C3	Others	68	32	2	30
Sludge	Spreading/Compost	Others	0	0	0	0
Spleen	Pet food	Others	75	25	5	20
Sweetbread	Human food	Others	70	30	5	25

**Table 4:** Destinations, group of tissues and composition of lamb coproducts (from Gac et al. (2012)).

Co-produits	Destination	Group of tissues	Water (%)	DRY MATTER (%)	Lipids (%)	Protéins (%)
Blood	PAP C3	Others	80	20	2	18
Blood	Spreading/Compost	Others	80	20	2	18
Bones	PAP C3	Carcass	60	40	15	15
Brain	Human food	Others	75	25	10	10
Contents of the intestines	Spreading/Compost	Others	85	15	8	7
Dead individuals	C1-C2 for disposal	Others	80	20	4	10
Downgraded skin	PAP C3	Others	68	32	2	30
Fat	PAP C3	Carcass	10	90	88	2
Floatation fat	C1-C2 for disposal	Others	15	85	84	1
Meat	Human food	Carcass	76	24	5	20
Other spa c1	C1-C2 for disposal	Others	68	32	5	23
Other spa c3	PAP C3	Others	68	32	4	25
Pluck (liver, heart, trachea)	Human food	Liver	72	28	4	22
Pluck (liver, heart, trachea)	Pet food	Liver	72	28	4	22
Rumen and reticulum	Human food	GIT	75	25	5	20
Rumen and reticulum	Pet food	GIT	75	25	5	20
Sanitary seizures	C1-C2 for disposal	Others	67	33	15	17
Screening waste	C1-C2 for disposal	Others	15	85	84	1
Sifting waste	C1-C2 for disposal	Others	15	85	84	1
Skin	Skin tannery C3	Others	68	32	2	30
Small intestine	C1-C2 for disposal	GIT	75	25	5	20
Small intestine	Human food	GIT	75	25	5	20
Small intestine	PAP C3	GIT	75	25	5	20
Stercoral matter	Spreading/Compost	Others	85	15	8	7
Thymus	Human food	Others	70	30	5	25
Thymus	Pet food	Others	70	30	5	25
Tongue	Human food	Others	72	28	10	16

For each coproduct, the mass fraction of the total mass is necessary. It has been calculated for each breed of each species. They can be considered as generic data to characterize coproducts. Data from Gac et al. (2012) are used as a reference and adapted to each breed depending on carcass yields [2].

$$BW{\%}_{i,\;j}=BW{\%}_{i,\;generic}*\;\frac{Carcass\;Yield_{j}}{Carcass\;Yield_{generic}}\;for\;carcass\;coproducts$$

$$BW{\%}_{i,\;j}=BW{\%}_{i,\;generic}*\;\frac{Carcass\;Yield_{generic}}{Carcass\;Yield_{j}}\;for\;other\;coproducts$$

$with\;BW{\%}_{i,\;j}\;\left(Empty\;Body\;Weight\right)\;the\;mass\;fraction\;of\;the\;coproduct\;i\;from\;breed\;j$

Carcass Yields are available in Table 5, Table 6, Table 7. They come from Laisse et al. [3]. These table also contains the Empty Body Weight at slaughter age that differs from a breed to another. These data have been obtained on the basis of a census data extraction operated by Institut de l'Elevage (GES Division) from SPIE (the Professional Livestock Information System approved by the French State), which contains data from the BDNI (National Data Base of Identification which register all animal birth and movements), completed by the Normabev database (concerning slaughtering of bovines). This French information system on livestock is described by Delomel and Gibon [4]. When data were not available, mean values have been used.

**Table 5:** Carcass Yields and Empty Body Weights at slaughter age for beef (from Laisse et al. (2018) and Delomel and Gibon (2019)).

Breed	Category	Carcass Yield	Empty body weight at slaughter age
Limousine	Heifer	0,57	633
Limousine	Beef	0,58	755
Limousine	Young Bull	0,61	693
Limousine	Cull Cow	0,55	736
Salers	Heifer	0,52	613
Salers	Beef	0,53	758
Salers	Young Bull	0,55	740
Salers	Cull Cow	0,5	690
Primholstein	Heifer	0,49	586
Primholstein	Beef	0,51	680
Primholstein	Young Bull	0,52	692
Primholstein	Cull Cow	0,48	648
Rouge des Prés	Heifer	0,54	639
Rouge des Prés	Beef	0,55	758
Rouge des Prés	Young Bull	0,57	713
Rouge des Prés	Cull Cow	0,52	704
Blonde d'Aquitaine	Heifer	0,59	761
Blonde d'Aquitaine	Beef	0,6	843
Blonde d'Aquitaine	Young Bull	0,63	727
Blonde d'Aquitaine	Cull Cow	0,52	927
Charolais	Heifer	0,55	718
Charolais	Beef	0,56	839
Charolais	Young Bull	0,58	764
Charolais	Cull Cow	0,53	804
Charolais x Rustique	Heifer	0,54	639
Charolais x Rustique	Beef	0,55	758
Charolais x Rustique	Young Bull	0,57	713
Charolais x Rustique	Cull Cow	0,52	704
Montbéliarde	Heifer	0,52	542
Montbéliarde	Beef	0,53	687
Montbéliarde	Young Bull	0,55	707
Montbéliarde	Cull Cow	0,5	632
Normande	Heifer	0,52	617
Normande	Beef	0,53	743
Normande	Young Bull	0,55	695
Normande	Cull Cow	0,5	698
Charolais x Pie Noire	Heifer	0,52	639
Charolais x Pie Noire	Beef	0,54	758
Charolais x Pie Noire	Young Bull	0,56	713
Charolais x Pie Noire	Cull Cow	0,51	704
Average	Heifer	0,54	660
Average	Beef	0,54	780
Average	Young Bull	0,54	730
Average	Cull Cow	0,55	742

**Table 6:** Carcass Yields and Empty Body Weights at slaughter age for calves (from Laisse et al. (2018) and Delomel and Gibon (2019)).

Breed	Category	Carcass Yield	Empty body weight at slaughter age
Limousine	Milk-fed	0,58	271
Limousine	Rosé	0,58	236
Aubrac	Milk-fed	0,58	262
Aubrac	Rosé	0,58	229
Primholstein	Milk-fed	0,58	236
Primholstein	Rosé	0,58	206
Blonde d'Aquitaine	Milk-fed	0,58	284
Blonde d'Aquitaine	Rosé	0,58	248
Charolais	Milk-fed	0,58	252
Charolais	Rosé	0,58	220
Montbéliarde	Milk-fed	0,58	257
Montbéliarde	Rosé	0,58	228
Normande	Milk-fed	0,58	229
Normande	Rosé	0,58	200
Croisé-lait	Milk-fed	0,58	238
Croisé-viande	Rosé	0,58	257
Average	Milk-fed	0,58	252
Average	Rosé	0,58	219

**Table 7:** Carcass Yields and Empty Body Weights at slaughter age for lambs (from Laisse et al. (2018) and Delomel and Gibon (2019)).

Breed	Category	Carcass Yield	Empty body weight at slaughter age
Generic	Milk-fed hardy lamb	0,48	36
Generic	Milk-fed heavy lamb	0,48	39
Generic	Grass-fed heavy lamb	0,46	41
Generic	Milk lamb	0,48	36
Generic	Average	0,475	38

Next table contains a list of parameters that are identical for each breed of a given species (Table 8). These parameters are used by the model developed by Chen et al. [1] to calculate the allocation factors based on the energy required to maintain and produce body tissues as a function of their chemical (protein and lipid) and physiological properties and growth (biophysical allocation). The parameters are:

- Gompertz Coefficient: initial rate of protein growth [5]
- Empty Body Weight at birth (kg) [expert interviews]
- Empty Body Weight at maturity (kg) [expert interviews]
- Birth Body Fat Percentage (%) [expert interviews]
- Normal mature body Fat Percentage (%) [expert interviews]
- Fat percentage at slaughter age (%) [expert interviews]
- Ratio of Body Weight Water to Protein [expert interviews]
- Protein Energy Content (MJ/kg) [5]
- Lipid Energy Content (MJ/kg) [5]

**Table 8:** Model parameters for beef, calves and lambs.

	Beef	Calves	Lambs
Gompertz Coefficient	0.012	0.012	0.03
Empty Body Weight at birth	50	50	5
Empty Body Weight at maturity	1000	1000	65
Birth Body Fat Percentage	0.06	0.06	0.1
Normal mature body Fat Percentage	0.45	0.45	0.33
Fat percentage at slaughter age	0.30	0.30	0.25
Ratio of Body Weight Water to Protein	3	3.5	3.5
Protein Energy Content (MJ/kg)	49.167	49.167	49.167
Lipid Energy Content (MJ/kg)	55.352	55.352	55.352

**Table 9:** Activity coefficients for beef, calves and lambs.

Species	Rearing mode	Details	Cact
Beef and calves	Stall	Small area (little or no energy)	0
	Pasture	Sufficient forage (modest energy)	0.17
	Grazing large areas	Open range land or hilly terrain (significant energy)	0.36
Lambs	Housed ewes	pregnancy in final trimester (50 d)	0.009
	Grazing flat pasture	walk up to 1 km/day and expend very little energy to acquire feed	0.0107
	Grazing hilly pâsture	walk up to 5 km/day and expend significant energy to acquire feed	0.024
	Housed fattening lambs	animals are housed for fattening	0.0067

**Table 10:** Economic value of coproducts for beef in €/ton (from ACYVIA).

Co-produits	Destination	Group of tissues	Economic value for beef (€/Ton)
Abomasum	Human food	GIT	2470
Abomasum fat	Fat and greaves C3	GIT	300
Aponeurosis	Human food	Carcass	3310
Bile	PAP C3	Others	283
Blood	PAP C3	Others	736
Blood	Pet food	Others	242
Bones	Gelatin C3	Carcass	10
Bones of head, brain, eyes and teeth	C1-C2 for disposal	Others	0
Cheek	Human food	Others	7250
Cheek	Human food	Others	7250
Cheek trimmings	Pet food	Others	242
Chops	Pet food	Others	242
Contents of intestines	Spreading/Compost	GIT	0
Contents of the rumen	Spreading/Compost	GIT	0
Ears	PAP C3	Others	283
Esophagus	Pet food	Others	242
Fat	Fat and greaves C3	Carcass	300
Fat around heart	Fat and greaves C3	Others	300
Fat in the kidney	Fat and greaves C3	Others	300
Feet (without hooves)	Gelatin C3	Others	10
Floatation fat	Spreading/Compost	Others	0
Forehead	C1-C2 for disposal	Others	0
Forelock	PAP C3	Others	283
Gallbladder	Pet food	Others	242
Head trimmings	Pet food	Others	242
Heart	Human food	Others	700
Heart trimmings	Pet food	Others	242
Hide	Skin tannery C3	Others	5500
Hooves	PAP C3	Others	283
Horns	PAP C3	Others	283
Kidney	Human food	Others	1340
Large intestine	C1-C2 for disposal	GIT	0
Liver	Human food	Liver	1600
Liver trimmings	Pet food	Liver	242
Lower jaw	PAP C3	Others	283
Lungs	Pet food	Others	242
Mask	Skin tannery C3	Others	5500
Mesenteric fat	C1-C2 for disposal	GIT	0
Muscle	Human food	Carcass	5510
Muzzle	Human food	Others	3310
Omasum	Human food	GIT	2470
Omasum fat	Fat and greaves C3	GIT	300
Rumen and forestomach	Human food	GIT	2470
Rumen fat	Fat and greaves C3	GIT	300
Sanitary seizures	C1-C2 for disposal	Others	0
Screening and sifting wastes	C1-C2 for disposal	Others	0
Small intestine	PAP C3	GIT	200
Spinal cord	C1-C2 for disposal	Others	0
Spinal cord waste	C1-C2 for disposal	Others	0
Spine	C1-C2 for disposal	Carcass	0
Spleen	Pet food	Others	242
Stillborn	PAP C3	GIT	0
Tallow	Fat and greaves C3	Others	300
Tongue	Human food	Others	5250
Tonsil	C1-C2 for disposal	Others	0
Trachea	Pet food	Others	242
Udder	Pet food	Others	242
Upper throat	Pet food	Others	242
Water in the rumen	Spreading/Compost	GIT	0

**Table 11:** Economic value of coproducts for calves in €/ton (from ACYVIA).

Co-produits	Destination	Group of tissues	Economic value for Calves (€/Ton)
Dead individuals	Human food	GIT	200
Manure	Fat and greaves C3	GIT	3310
Screening and sifting wastes	Human food	Carcass	283
Floatation fat	PAP C3	Others	0
Sludge	PAP C3	Others	10
Blood	Pet food	Others	0
Skin	Gelatin C3	Carcass	300
Bile	C1-C2 for disposal	Others	300
Spleen	Human food	Others	10
Intestines	Human food	Others	0
Fat from breasts and penis	Pet food	Others	7500
Rumen and forestomach	Pet food	Others	0
Abomasum	Spreading/Compost	GIT	3480
Sweetbread	Spreading/Compost	GIT	0
Kidney	PAP C3	Others	6840
Pluck	Pet food	Others	1400
Feet (without hooves)	Fat and greaves C3	Carcass	200
Head	Fat and greaves C3	Others	283
SPA C3	Fat and greaves C3	Others	0
Meat	Spreading/Compost	Others	4000
Fat (8%)	C1-C2 for disposal	Others	0
Aponevrosis (1%)	PAP C3	Others	241.5
Bones (11%)	Pet food	Others	5240

**Table 12:** Economic value of coproducts for lambs in €/ton (from ACYVIA).

Co-produits	Destination	Group of tissues	Economic value for lambs (€/Ton)
Blood	PAP C3	Others	0
Blood	Spreading/Compost	Others	0
Bones	PAP C3	Carcass	0
Brain	Human food	Others	0
Contents of the intestines	Spreading/Compost	Others	0
Dead individuals	C1-C2 for disposal	Others	0
Downgraded skin	PAP C3	Others	0
Fat	PAP C3	Carcass	0
Floatation fat	C1-C2 for disposal	Others	0
Meat	Human food	Carcass	5300
Other spa c1	C1-C2 for disposal	Others	0
Other spa c3	PAP C3	Others	0
Pluck (liver, heart, trachea)	Human food	Liver	3100
Pluck (liver, heart, trachea)	Pet food	Liver	3080
Rumen and reticulum	Human food	GIT	0
Rumen and reticulum	Pet food	GIT	0
Sanitary seizures	C1-C2 for disposal	Others	0
Screening waste	C1-C2 for disposal	Others	0
Sifting waste	C1-C2 for disposal	Others	0
Skin	Skin tannery C3	Others	700
Small intestine	C1-C2 for disposal	GIT	0
Small intestine	Human food	GIT	0
Small intestine	PAP C3	GIT	0
Stercoral matter	Spreading/Compost	Others	0
Thymus	Human food	Others	10,200
Thymus	Pet food	Others	10,200
Tongue	Human food	Others	4500

Finally, a coefficient is used to modulate the energy required for the activity. These coefficients are specific for breeds and depend on the rearing mode. Data from IPCC (2006) are used [6]. Data are available in Table 9.

To calculate economic allocation factors, an economic dataset has been built by compiling data from ACYVIA [7]. The dataset is available respectively for beef, calf and lamb in Table 10, Table 11, Table 12

All these input data are also available in a complete *.csv file (supplementary file 13). This is the formatted database as used by MeatPartTool calculation freeware.

### 1.2 Allocation factors

For each individual, mass (based on wet mass), economic and biophysical allocation factors are given per kg of coproduct. They are respectively available for bovine, calf and ovine in supplementary file 1, supplementary file 4 and supplementary file 7. Then the total weightings by coproduct (i.e. allocation factor per kg multiplied by the mass of coproduct) are also given (respectively available in supplementary files 2, 5 and 8). Finally, an aggregation by destination category (e.g. Human food, PAP C3, etc.) is also available (respectively in supplementary files 3, 6 and 9).

## 2 Experimental Design, Materials and Methods

Mass and economic allocation factors have been calculated by following LCA standards. Biophysical allocation factors calculation was performed using Chen et al. (2017) model. A calculation freeware has been developed in Python. The code section that concerns the calculation are given in supplementary files 10, 11 and 12. A specific code is used for each species. These are Python files readable with any code editor (as Notepad++). They work with extra code, formatting a list from a *csv.file. The complete code is implemented in the MeatPartTool open-source freeware [8].

One at a Time sensitivity analysis is provided for the two variant input parameters. The variation of the share of human food destination coproducts is given when testing different Gompertz Coefficients, Carcass Yields and Rearing methods. Results are summed up in Table 13 and more details are provided in Supplementary Files 14. Results are the most sensitive to Gompertz Coefficient with only 10% of variation between extreme values. Very few information was found about this parameter in the case of the present study. Consequently, the authors think that biophysical allocation would benefit from more research on Gompertz coefficient in the future.

**Table 13:** Sensitivity analysis of the share of human food destination coproducts depending on main input parameters.

Input parameter	Initial value	Tested Values	Human Food destination share	Difference between extreme values
Gompertz Coefficient	0,012	[0,003 ; 0,006 ; 0,009 ; 0,012 ; 0,018 ; 0,024]	From 47% to 57%	10%
Carcass Yield	0,56	[0,50 ; 0,52 ; 0,54 ; 0,56 ; 0,58 ; 0,60]	From 48% to 52%	4%

## Declaration of Competing Interest

This work received financial support from Interbev (SECU 19–20).

## Appendix Supplementary Materials

Image, application 1

Image, application 2

Image, application 3

Image, application 4

Image, application 5

Image, application 6

Image, application 7

Image, application 8

Image, application 9

Image, application 10

Image, application 11

Image, application 12

Image, application 13

Image, application 14
