# Supplementary material for: Allocation factors for meat coproducts: Dataset to perform life cycle assessment at slaughterhouse
Source: Data Brief. 2020 Nov 23;33:106558. doi: 10.1016/j.dib.2020.106558 (PMC7718151; doi:10.1016/j.dib.2020.106558)
Supplement: Supplementary file 1 [file mmc1.docx]

Table 1: Allocation factors for Normande Young Bulls reared in Grazing Large Area

| COPRODUCT | Destination | Normande/young bull/grazing large area | | |
| --- | --- | --- | --- | --- |
|  |  | **Biophysical Allocation Factor** | **Mass Allocation Factor** | **Economic Allocation Factor** |
| Abomasum | Human food | 0.0091 | 0.0016 | 0.0012 |
| Abomasum fat | Fat and greaves C3 | 0.0034 | 0.0016 | 0.0001 |
| Aponeurosis | Human food | 0.0012 | 0.0016 | 0.0016 |
| Bile | PAP C3 | 0.0004 | 0.0016 | 0.0001 |
| Blood | PAP C3 | 0.0009 | 0.0016 | 0.0004 |
| Blood | Pet food | 0.0009 | 0.0016 | 0.0001 |
| Bones | Gelatin C3 | 0.0010 | 0.0016 | 0.0000 |
| Bones of head, brain, eyes and teeth | C1-C2 for disposal | 0.0000 | 0.0000 | 0.0000 |
| Cheek | Human food | 0.0011 | 0.0016 | 0.0035 |
| Cheek | Human food | 0.0011 | 0.0016 | 0.0035 |
| Cheek trimmings | Pet food | 0.0011 | 0.0016 | 0.0001 |
| Chops | Pet food | 0.0015 | 0.0016 | 0.0001 |
| Contents of intestines | Spreading/Compost | 0.0000 | 0.0000 | 0.0000 |
| Contents of the rumen | Spreading/Compost | 0.0000 | 0.0000 | 0.0000 |
| Ears | PAP C3 | 0.0014 | 0.0016 | 0.0001 |
| Esophagus | Pet food | 0.0011 | 0.0016 | 0.0001 |
| Fat | Fat and greaves C3 | 0.0015 | 0.0016 | 0.0001 |
| Fat around heart | Fat and greaves C3 | 0.0015 | 0.0016 | 0.0001 |
| Fat in the kidney | Fat and greaves C3 | 0.0015 | 0.0016 | 0.0001 |
| Feet (without hooves) | Gelatin C3 | 0.0011 | 0.0016 | 0.0000 |
| Floatation fat | Spreading/Compost | 0.0000 | 0.0000 | 0.0000 |
| Forehead | C1-C2 for disposal | 0.0000 | 0.0000 | 0.0000 |
| Forelock | PAP C3 | 0.0039 | 0.0016 | 0.0001 |
| Gallbladder | Pet food | 0.0011 | 0.0016 | 0.0001 |
| Head trimmings | Pet food | 0.0011 | 0.0016 | 0.0001 |
| Heart | Human food | 0.0010 | 0.0016 | 0.0003 |
| Heart trimmings | Pet food | 0.0011 | 0.0016 | 0.0001 |
| Hide | Skin tannery C3 | 0.0015 | 0.0016 | 0.0026 |
| Hooves | PAP C3 | 0.0039 | 0.0016 | 0.0001 |
| Horns | PAP C3 | 0.0039 | 0.0016 | 0.0001 |
| Kidney | Human food | 0.0011 | 0.0016 | 0.0006 |
| Large intestine | C1-C2 for disposal | 0.0000 | 0.0000 | 0.0000 |
| Liver | Human food | 0.0047 | 0.0016 | 0.0008 |
| Liver trimmings | Pet food | 0.0049 | 0.0016 | 0.0001 |
| Lower jaw | PAP C3 | 0.0010 | 0.0016 | 0.0001 |
| Lungs | Pet food | 0.0013 | 0.0016 | 0.0001 |
| Mask | Skin tannery C3 | 0.0015 | 0.0016 | 0.0026 |
| Mesenteric fat | C1-C2 for disposal | 0.0000 | 0.0000 | 0.0000 |
| Muscle | Human food | 0.0011 | 0.0016 | 0.0026 |
| Muzzle | Human food | 0.0015 | 0.0016 | 0.0016 |
| Omasum | Human food | 0.0091 | 0.0016 | 0.0012 |
| Omasum fat | Fat and greaves C3 | 0.0034 | 0.0016 | 0.0001 |
| Rumen and forestomach | Human food | 0.0091 | 0.0016 | 0.0012 |
| Rumen fat | Fat and greaves C3 | 0.0034 | 0.0016 | 0.0001 |
| Sanitary seizures | C1-C2 for disposal | 0.0000 | 0.0000 | 0.0000 |
| Screening and sifting wastes | C1-C2 for disposal | 0.0000 | 0.0000 | 0.0000 |
| Small intestine | PAP C3 | 0.0091 | 0.0016 | 0.0001 |
| Spinal cord | C1-C2 for disposal | 0.0000 | 0.0000 | 0.0000 |
| Spinal cord waste | C1-C2 for disposal | 0.0000 | 0.0000 | 0.0000 |
| Spine | C1-C2 for disposal | 0.0000 | 0.0000 | 0.0000 |
| Spleen | Pet food | 0.0011 | 0.0016 | 0.0001 |
| Stillborn | PAP C3 | 0.0095 | 0.0016 | 0.0000 |
| Tallow | Fat and greaves C3 | 0.0015 | 0.0016 | 0.0001 |
| Tongue | Human food | 0.0010 | 0.0016 | 0.0025 |
| Tonsil | C1-C2 for disposal | 0.0000 | 0.0000 | 0.0000 |
| Trachea | Pet food | 0.0015 | 0.0016 | 0.0001 |
| Udder | Pet food | 0.0002 | 0.0016 | 0.0001 |
| Upper throat | Pet food | 0.0011 | 0.0016 | 0.0001 |
| Water in the rumen | Spreading/Compost | 0.0000 | 0.0000 | 0.0000 |

Table 2: Allocation factors for Normande Heifers reared in Grazing Large Area

| COPRODUCT | Destination | Normande/heifer/grazing large area | | |
| --- | --- | --- | --- | --- |
|  |  | **Biophysical Allocation Factor** | **Mass Allocation Factor** | **Economic Allocation Factor** |
| Abomasum | Human food | 0.0099 | 0.0018 | 0.0014 |
| Abomasum fat | Fat and greaves C3 | 0.0037 | 0.0018 | 0.0002 |
| Aponeurosis | Human food | 0.0014 | 0.0018 | 0.0018 |
| Bile | PAP C3 | 0.0005 | 0.0018 | 0.0002 |
| Blood | PAP C3 | 0.0010 | 0.0018 | 0.0004 |
| Blood | Pet food | 0.0010 | 0.0018 | 0.0001 |
| Bones | Gelatin C3 | 0.0011 | 0.0018 | 0.0000 |
| Bones of head, brain, eyes and teeth | C1-C2 for disposal | 0.0000 | 0.0000 | 0.0000 |
| Cheek | Human food | 0.0012 | 0.0018 | 0.0040 |
| Cheek | Human food | 0.0012 | 0.0018 | 0.0040 |
| Cheek trimmings | Pet food | 0.0012 | 0.0018 | 0.0001 |
| Chops | Pet food | 0.0017 | 0.0018 | 0.0001 |
| Contents of intestines | Spreading/Compost | 0.0000 | 0.0000 | 0.0000 |
| Contents of the rumen | Spreading/Compost | 0.0000 | 0.0000 | 0.0000 |
| Ears | PAP C3 | 0.0015 | 0.0018 | 0.0002 |
| Esophagus | Pet food | 0.0012 | 0.0018 | 0.0001 |
| Fat | Fat and greaves C3 | 0.0016 | 0.0018 | 0.0002 |
| Fat around heart | Fat and greaves C3 | 0.0016 | 0.0018 | 0.0002 |
| Fat in the kidney | Fat and greaves C3 | 0.0016 | 0.0018 | 0.0002 |
| Feet (without hooves) | Gelatin C3 | 0.0012 | 0.0018 | 0.0000 |
| Floatation fat | Spreading/Compost | 0.0000 | 0.0000 | 0.0000 |
| Forehead | C1-C2 for disposal | 0.0000 | 0.0000 | 0.0000 |
| Forelock | PAP C3 | 0.0044 | 0.0018 | 0.0002 |
| Gallbladder | Pet food | 0.0012 | 0.0018 | 0.0001 |
| Head trimmings | Pet food | 0.0012 | 0.0018 | 0.0001 |
| Heart | Human food | 0.0012 | 0.0018 | 0.0004 |
| Heart trimmings | Pet food | 0.0012 | 0.0018 | 0.0001 |
| Hide | Skin tannery C3 | 0.0017 | 0.0018 | 0.0030 |
| Hooves | PAP C3 | 0.0044 | 0.0018 | 0.0002 |
| Horns | PAP C3 | 0.0044 | 0.0018 | 0.0002 |
| Kidney | Human food | 0.0012 | 0.0018 | 0.0007 |
| Large intestine | C1-C2 for disposal | 0.0000 | 0.0000 | 0.0000 |
| Liver | Human food | 0.0051 | 0.0018 | 0.0009 |
| Liver trimmings | Pet food | 0.0054 | 0.0018 | 0.0001 |
| Lower jaw | PAP C3 | 0.0011 | 0.0018 | 0.0002 |
| Lungs | Pet food | 0.0014 | 0.0018 | 0.0001 |
| Mask | Skin tannery C3 | 0.0017 | 0.0018 | 0.0030 |
| Mesenteric fat | C1-C2 for disposal | 0.0000 | 0.0000 | 0.0000 |
| Muscle | Human food | 0.0012 | 0.0018 | 0.0030 |
| Muzzle | Human food | 0.0017 | 0.0018 | 0.0018 |
| Omasum | Human food | 0.0099 | 0.0018 | 0.0014 |
| Omasum fat | Fat and greaves C3 | 0.0037 | 0.0018 | 0.0002 |
| Rumen and forestomach | Human food | 0.0099 | 0.0018 | 0.0014 |
| Rumen fat | Fat and greaves C3 | 0.0037 | 0.0018 | 0.0002 |
| Sanitary seizures | C1-C2 for disposal | 0.0000 | 0.0000 | 0.0000 |
| Screening and sifting wastes | C1-C2 for disposal | 0.0000 | 0.0000 | 0.0000 |
| Small intestine | PAP C3 | 0.0099 | 0.0018 | 0.0001 |
| Spinal cord | C1-C2 for disposal | 0.0000 | 0.0000 | 0.0000 |
| Spinal cord waste | C1-C2 for disposal | 0.0000 | 0.0000 | 0.0000 |
| Spine | C1-C2 for disposal | 0.0000 | 0.0000 | 0.0000 |
| Spleen | Pet food | 0.0012 | 0.0018 | 0.0001 |
| Stillborn | PAP C3 | 0.0103 | 0.0018 | 0.0000 |
| Tallow | Fat and greaves C3 | 0.0016 | 0.0018 | 0.0002 |
| Tongue | Human food | 0.0011 | 0.0018 | 0.0029 |
| Tonsil | C1-C2 for disposal | 0.0000 | 0.0000 | 0.0000 |
| Trachea | Pet food | 0.0017 | 0.0018 | 0.0001 |
| Udder | Pet food | 0.0003 | 0.0018 | 0.0001 |
| Upper throat | Pet food | 0.0012 | 0.0018 | 0.0001 |
| Water in the rumen | Spreading/Compost | 0.0000 | 0.0000 | 0.0000 |

Table 3: Allocation factors for Normande Cull Cows reared in Grazing Large Area

| COPRODUCT | Destination | Normande/Cull cow/grazing large area | | |
| --- | --- | --- | --- | --- |
|  |  | **Biophysical Allocation Factor** | **Mass Allocation Factor** | **Economic Allocation Factor** |
| Abomasum | Human food | 0.0089 | 0.0016 | 0.0012 |
| Abomasum fat | Fat and greaves C3 | 0.0033 | 0.0016 | 0.0001 |
| Aponeurosis | Human food | 0.0012 | 0.0016 | 0.0016 |
| Bile | PAP C3 | 0.0004 | 0.0016 | 0.0001 |
| Blood | PAP C3 | 0.0009 | 0.0016 | 0.0004 |
| Blood | Pet food | 0.0009 | 0.0016 | 0.0001 |
| Bones | Gelatin C3 | 0.0010 | 0.0016 | 0.0000 |
| Bones of head, brain, eyes and teeth | C1-C2 for disposal | 0.0000 | 0.0000 | 0.0000 |
| Cheek | Human food | 0.0011 | 0.0016 | 0.0036 |
| Cheek | Human food | 0.0011 | 0.0016 | 0.0036 |
| Cheek trimmings | Pet food | 0.0011 | 0.0016 | 0.0001 |
| Chops | Pet food | 0.0015 | 0.0016 | 0.0001 |
| Contents of intestines | Spreading/Compost | 0.0000 | 0.0000 | 0.0000 |
| Contents of the rumen | Spreading/Compost | 0.0000 | 0.0000 | 0.0000 |
| Ears | PAP C3 | 0.0013 | 0.0016 | 0.0001 |
| Esophagus | Pet food | 0.0010 | 0.0016 | 0.0001 |
| Fat | Fat and greaves C3 | 0.0014 | 0.0016 | 0.0001 |
| Fat around heart | Fat and greaves C3 | 0.0014 | 0.0016 | 0.0001 |
| Fat in the kidney | Fat and greaves C3 | 0.0014 | 0.0016 | 0.0001 |
| Feet (without hooves) | Gelatin C3 | 0.0010 | 0.0016 | 0.0000 |
| Floatation fat | Spreading/Compost | 0.0000 | 0.0000 | 0.0000 |
| Forehead | C1-C2 for disposal | 0.0000 | 0.0000 | 0.0000 |
| Forelock | PAP C3 | 0.0038 | 0.0016 | 0.0001 |
| Gallbladder | Pet food | 0.0010 | 0.0016 | 0.0001 |
| Head trimmings | Pet food | 0.0011 | 0.0016 | 0.0001 |
| Heart | Human food | 0.0010 | 0.0016 | 0.0003 |
| Heart trimmings | Pet food | 0.0011 | 0.0016 | 0.0001 |
| Hide | Skin tannery C3 | 0.0015 | 0.0016 | 0.0027 |
| Hooves | PAP C3 | 0.0038 | 0.0016 | 0.0001 |
| Horns | PAP C3 | 0.0038 | 0.0016 | 0.0001 |
| Kidney | Human food | 0.0010 | 0.0016 | 0.0007 |
| Large intestine | C1-C2 for disposal | 0.0000 | 0.0000 | 0.0000 |
| Liver | Human food | 0.0046 | 0.0016 | 0.0008 |
| Liver trimmings | Pet food | 0.0048 | 0.0016 | 0.0001 |
| Lower jaw | PAP C3 | 0.0009 | 0.0016 | 0.0001 |
| Lungs | Pet food | 0.0012 | 0.0016 | 0.0001 |
| Mask | Skin tannery C3 | 0.0015 | 0.0016 | 0.0027 |
| Mesenteric fat | C1-C2 for disposal | 0.0000 | 0.0000 | 0.0000 |
| Muscle | Human food | 0.0011 | 0.0016 | 0.0027 |
| Muzzle | Human food | 0.0015 | 0.0016 | 0.0016 |
| Omasum | Human food | 0.0089 | 0.0016 | 0.0012 |
| Omasum fat | Fat and greaves C3 | 0.0033 | 0.0016 | 0.0001 |
| Rumen and forestomach | Human food | 0.0089 | 0.0016 | 0.0012 |
| Rumen fat | Fat and greaves C3 | 0.0033 | 0.0016 | 0.0001 |
| Sanitary seizures | C1-C2 for disposal | 0.0000 | 0.0000 | 0.0000 |
| Screening and sifting wastes | C1-C2 for disposal | 0.0000 | 0.0000 | 0.0000 |
| Small intestine | PAP C3 | 0.0089 | 0.0016 | 0.0001 |
| Spinal cord | C1-C2 for disposal | 0.0000 | 0.0000 | 0.0000 |
| Spinal cord waste | C1-C2 for disposal | 0.0000 | 0.0000 | 0.0000 |
| Spine | C1-C2 for disposal | 0.0000 | 0.0000 | 0.0000 |
| Spleen | Pet food | 0.0011 | 0.0016 | 0.0001 |
| Stillborn | PAP C3 | 0.0093 | 0.0016 | 0.0000 |
| Tallow | Fat and greaves C3 | 0.0014 | 0.0016 | 0.0001 |
| Tongue | Human food | 0.0009 | 0.0016 | 0.0026 |
| Tonsil | C1-C2 for disposal | 0.0000 | 0.0000 | 0.0000 |
| Trachea | Pet food | 0.0015 | 0.0016 | 0.0001 |
| Udder | Pet food | 0.0002 | 0.0016 | 0.0001 |
| Upper throat | Pet food | 0.0010 | 0.0016 | 0.0001 |
| Water in the rumen | Spreading/Compost | 0.0000 | 0.0000 | 0.0000 |

Table 4: Allocation factors for Normande Beef reared in Grazing Large Area

| COPRODUCT | Destination | Normande/beef/grazing large area | | |
| --- | --- | --- | --- | --- |
|  |  | **Biophysical Allocation Factor** | **Mass Allocation Factor** | **Economic Allocation Factor** |
| Abomasum | Human food | 0.0086 | 0.0015 | 0.0011 |
| Abomasum fat | Fat and greaves C3 | 0.0032 | 0.0015 | 0.0001 |
| Aponeurosis | Human food | 0.0011 | 0.0015 | 0.0015 |
| Bile | PAP C3 | 0.0004 | 0.0015 | 0.0001 |
| Blood | PAP C3 | 0.0009 | 0.0015 | 0.0003 |
| Blood | Pet food | 0.0009 | 0.0015 | 0.0001 |
| Bones | Gelatin C3 | 0.0009 | 0.0015 | 0.0000 |
| Bones of head, brain, eyes and teeth | C1-C2 for disposal | 0.0000 | 0.0000 | 0.0000 |
| Cheek | Human food | 0.0010 | 0.0015 | 0.0033 |
| Cheek | Human food | 0.0010 | 0.0015 | 0.0033 |
| Cheek trimmings | Pet food | 0.0010 | 0.0015 | 0.0001 |
| Chops | Pet food | 0.0014 | 0.0015 | 0.0001 |
| Contents of intestines | Spreading/Compost | 0.0000 | 0.0000 | 0.0000 |
| Contents of the rumen | Spreading/Compost | 0.0000 | 0.0000 | 0.0000 |
| Ears | PAP C3 | 0.0012 | 0.0015 | 0.0001 |
| Esophagus | Pet food | 0.0010 | 0.0015 | 0.0001 |
| Fat | Fat and greaves C3 | 0.0013 | 0.0015 | 0.0001 |
| Fat around heart | Fat and greaves C3 | 0.0013 | 0.0015 | 0.0001 |
| Fat in the kidney | Fat and greaves C3 | 0.0013 | 0.0015 | 0.0001 |
| Feet (without hooves) | Gelatin C3 | 0.0010 | 0.0015 | 0.0000 |
| Floatation fat | Spreading/Compost | 0.0000 | 0.0000 | 0.0000 |
| Forehead | C1-C2 for disposal | 0.0000 | 0.0000 | 0.0000 |
| Forelock | PAP C3 | 0.0036 | 0.0015 | 0.0001 |
| Gallbladder | Pet food | 0.0010 | 0.0015 | 0.0001 |
| Head trimmings | Pet food | 0.0010 | 0.0015 | 0.0001 |
| Heart | Human food | 0.0010 | 0.0015 | 0.0003 |
| Heart trimmings | Pet food | 0.0010 | 0.0015 | 0.0001 |
| Hide | Skin tannery C3 | 0.0014 | 0.0015 | 0.0025 |
| Hooves | PAP C3 | 0.0036 | 0.0015 | 0.0001 |
| Horns | PAP C3 | 0.0036 | 0.0015 | 0.0001 |
| Kidney | Human food | 0.0010 | 0.0015 | 0.0006 |
| Large intestine | C1-C2 for disposal | 0.0000 | 0.0000 | 0.0000 |
| Liver | Human food | 0.0044 | 0.0015 | 0.0007 |
| Liver trimmings | Pet food | 0.0046 | 0.0015 | 0.0001 |
| Lower jaw | PAP C3 | 0.0009 | 0.0015 | 0.0001 |
| Lungs | Pet food | 0.0012 | 0.0015 | 0.0001 |
| Mask | Skin tannery C3 | 0.0014 | 0.0015 | 0.0025 |
| Mesenteric fat | C1-C2 for disposal | 0.0000 | 0.0000 | 0.0000 |
| Muscle | Human food | 0.0010 | 0.0015 | 0.0025 |
| Muzzle | Human food | 0.0014 | 0.0015 | 0.0015 |
| Omasum | Human food | 0.0086 | 0.0015 | 0.0011 |
| Omasum fat | Fat and greaves C3 | 0.0032 | 0.0015 | 0.0001 |
| Rumen and forestomach | Human food | 0.0086 | 0.0015 | 0.0011 |
| Rumen fat | Fat and greaves C3 | 0.0032 | 0.0015 | 0.0001 |
| Sanitary seizures | C1-C2 for disposal | 0.0000 | 0.0000 | 0.0000 |
| Screening and sifting wastes | C1-C2 for disposal | 0.0000 | 0.0000 | 0.0000 |
| Small intestine | PAP C3 | 0.0086 | 0.0015 | 0.0001 |
| Spinal cord | C1-C2 for disposal | 0.0000 | 0.0000 | 0.0000 |
| Spinal cord waste | C1-C2 for disposal | 0.0000 | 0.0000 | 0.0000 |
| Spine | C1-C2 for disposal | 0.0000 | 0.0000 | 0.0000 |
| Spleen | Pet food | 0.0010 | 0.0015 | 0.0001 |
| Stillborn | PAP C3 | 0.0090 | 0.0015 | 0.0000 |
| Tallow | Fat and greaves C3 | 0.0013 | 0.0015 | 0.0001 |
| Tongue | Human food | 0.0009 | 0.0015 | 0.0024 |
| Tonsil | C1-C2 for disposal | 0.0000 | 0.0000 | 0.0000 |
| Trachea | Pet food | 0.0014 | 0.0015 | 0.0001 |
| Udder | Pet food | 0.0002 | 0.0015 | 0.0001 |
| Upper throat | Pet food | 0.0010 | 0.0015 | 0.0001 |
| Water in the rumen | Spreading/Compost | 0.0000 | 0.0000 | 0.0000 |

Table 5: Allocation factors for Normande Young Bulls reared in Pasture

| COPRODUCT | Destination | Normande/young bull/pasture | | |
| --- | --- | --- | --- | --- |
|  |  | **Biophysical Allocation Factor** | **Mass Allocation Factor** | **Economic Allocation Factor** |
| Abomasum | Human food | 0.0087 | 0.0016 | 0.0012 |
| Abomasum fat | Fat and greaves C3 | 0.0035 | 0.0016 | 0.0001 |
| Aponeurosis | Human food | 0.0012 | 0.0016 | 0.0016 |
| Bile | PAP C3 | 0.0004 | 0.0016 | 0.0001 |
| Blood | PAP C3 | 0.0009 | 0.0016 | 0.0004 |
| Blood | Pet food | 0.0009 | 0.0016 | 0.0001 |
| Bones | Gelatin C3 | 0.0010 | 0.0016 | 0.0000 |
| Bones of head, brain, eyes and teeth | C1-C2 for disposal | 0.0000 | 0.0000 | 0.0000 |
| Cheek | Human food | 0.0011 | 0.0016 | 0.0035 |
| Cheek | Human food | 0.0011 | 0.0016 | 0.0035 |
| Cheek trimmings | Pet food | 0.0011 | 0.0016 | 0.0001 |
| Chops | Pet food | 0.0015 | 0.0016 | 0.0001 |
| Contents of intestines | Spreading/Compost | 0.0000 | 0.0000 | 0.0000 |
| Contents of the rumen | Spreading/Compost | 0.0000 | 0.0000 | 0.0000 |
| Ears | PAP C3 | 0.0014 | 0.0016 | 0.0001 |
| Esophagus | Pet food | 0.0011 | 0.0016 | 0.0001 |
| Fat | Fat and greaves C3 | 0.0016 | 0.0016 | 0.0001 |
| Fat around heart | Fat and greaves C3 | 0.0016 | 0.0016 | 0.0001 |
| Fat in the kidney | Fat and greaves C3 | 0.0016 | 0.0016 | 0.0001 |
| Feet (without hooves) | Gelatin C3 | 0.0011 | 0.0016 | 0.0000 |
| Floatation fat | Spreading/Compost | 0.0000 | 0.0000 | 0.0000 |
| Forehead | C1-C2 for disposal | 0.0000 | 0.0000 | 0.0000 |
| Forelock | PAP C3 | 0.0039 | 0.0016 | 0.0001 |
| Gallbladder | Pet food | 0.0011 | 0.0016 | 0.0001 |
| Head trimmings | Pet food | 0.0011 | 0.0016 | 0.0001 |
| Heart | Human food | 0.0010 | 0.0016 | 0.0003 |
| Heart trimmings | Pet food | 0.0011 | 0.0016 | 0.0001 |
| Hide | Skin tannery C3 | 0.0015 | 0.0016 | 0.0026 |
| Hooves | PAP C3 | 0.0039 | 0.0016 | 0.0001 |
| Horns | PAP C3 | 0.0039 | 0.0016 | 0.0001 |
| Kidney | Human food | 0.0011 | 0.0016 | 0.0006 |
| Large intestine | C1-C2 for disposal | 0.0000 | 0.0000 | 0.0000 |
| Liver | Human food | 0.0045 | 0.0016 | 0.0008 |
| Liver trimmings | Pet food | 0.0047 | 0.0016 | 0.0001 |
| Lower jaw | PAP C3 | 0.0010 | 0.0016 | 0.0001 |
| Lungs | Pet food | 0.0013 | 0.0016 | 0.0001 |
| Mask | Skin tannery C3 | 0.0015 | 0.0016 | 0.0026 |
| Mesenteric fat | C1-C2 for disposal | 0.0000 | 0.0000 | 0.0000 |
| Muscle | Human food | 0.0011 | 0.0016 | 0.0026 |
| Muzzle | Human food | 0.0015 | 0.0016 | 0.0016 |
| Omasum | Human food | 0.0087 | 0.0016 | 0.0012 |
| Omasum fat | Fat and greaves C3 | 0.0035 | 0.0016 | 0.0001 |
| Rumen and forestomach | Human food | 0.0087 | 0.0016 | 0.0012 |
| Rumen fat | Fat and greaves C3 | 0.0035 | 0.0016 | 0.0001 |
| Sanitary seizures | C1-C2 for disposal | 0.0000 | 0.0000 | 0.0000 |
| Screening and sifting wastes | C1-C2 for disposal | 0.0000 | 0.0000 | 0.0000 |
| Small intestine | PAP C3 | 0.0087 | 0.0016 | 0.0001 |
| Spinal cord | C1-C2 for disposal | 0.0000 | 0.0000 | 0.0000 |
| Spinal cord waste | C1-C2 for disposal | 0.0000 | 0.0000 | 0.0000 |
| Spine | C1-C2 for disposal | 0.0000 | 0.0000 | 0.0000 |
| Spleen | Pet food | 0.0011 | 0.0016 | 0.0001 |
| Stillborn | PAP C3 | 0.0091 | 0.0016 | 0.0000 |
| Tallow | Fat and greaves C3 | 0.0016 | 0.0016 | 0.0001 |
| Tongue | Human food | 0.0010 | 0.0016 | 0.0025 |
| Tonsil | C1-C2 for disposal | 0.0000 | 0.0000 | 0.0000 |
| Trachea | Pet food | 0.0015 | 0.0016 | 0.0001 |
| Udder | Pet food | 0.0002 | 0.0016 | 0.0001 |
| Upper throat | Pet food | 0.0011 | 0.0016 | 0.0001 |
| Water in the rumen | Spreading/Compost | 0.0000 | 0.0000 | 0.0000 |

Table 6: Allocation factors for Normande Heifers reared in Pasture

| COPRODUCT | Destination | Normande/heifer/pasture | | |
| --- | --- | --- | --- | --- |
|  |  | **Biophysical Allocation Factor** | **Mass Allocation Factor** | **Economic Allocation Factor** |
| Abomasum | Human food | 0.0094 | 0.0018 | 0.0014 |
| Abomasum fat | Fat and greaves C3 | 0.0038 | 0.0018 | 0.0002 |
| Aponeurosis | Human food | 0.0013 | 0.0018 | 0.0018 |
| Bile | PAP C3 | 0.0005 | 0.0018 | 0.0002 |
| Blood | PAP C3 | 0.0010 | 0.0018 | 0.0004 |
| Blood | Pet food | 0.0010 | 0.0018 | 0.0001 |
| Bones | Gelatin C3 | 0.0011 | 0.0018 | 0.0000 |
| Bones of head, brain, eyes and teeth | C1-C2 for disposal | 0.0000 | 0.0000 | 0.0000 |
| Cheek | Human food | 0.0012 | 0.0018 | 0.0040 |
| Cheek | Human food | 0.0012 | 0.0018 | 0.0040 |
| Cheek trimmings | Pet food | 0.0012 | 0.0018 | 0.0001 |
| Chops | Pet food | 0.0017 | 0.0018 | 0.0001 |
| Contents of intestines | Spreading/Compost | 0.0000 | 0.0000 | 0.0000 |
| Contents of the rumen | Spreading/Compost | 0.0000 | 0.0000 | 0.0000 |
| Ears | PAP C3 | 0.0015 | 0.0018 | 0.0002 |
| Esophagus | Pet food | 0.0012 | 0.0018 | 0.0001 |
| Fat | Fat and greaves C3 | 0.0018 | 0.0018 | 0.0002 |
| Fat around heart | Fat and greaves C3 | 0.0018 | 0.0018 | 0.0002 |
| Fat in the kidney | Fat and greaves C3 | 0.0018 | 0.0018 | 0.0002 |
| Feet (without hooves) | Gelatin C3 | 0.0012 | 0.0018 | 0.0000 |
| Floatation fat | Spreading/Compost | 0.0000 | 0.0000 | 0.0000 |
| Forehead | C1-C2 for disposal | 0.0000 | 0.0000 | 0.0000 |
| Forelock | PAP C3 | 0.0044 | 0.0018 | 0.0002 |
| Gallbladder | Pet food | 0.0012 | 0.0018 | 0.0001 |
| Head trimmings | Pet food | 0.0012 | 0.0018 | 0.0001 |
| Heart | Human food | 0.0012 | 0.0018 | 0.0004 |
| Heart trimmings | Pet food | 0.0012 | 0.0018 | 0.0001 |
| Hide | Skin tannery C3 | 0.0017 | 0.0018 | 0.0030 |
| Hooves | PAP C3 | 0.0044 | 0.0018 | 0.0002 |
| Horns | PAP C3 | 0.0044 | 0.0018 | 0.0002 |
| Kidney | Human food | 0.0012 | 0.0018 | 0.0007 |
| Large intestine | C1-C2 for disposal | 0.0000 | 0.0000 | 0.0000 |
| Liver | Human food | 0.0049 | 0.0018 | 0.0009 |
| Liver trimmings | Pet food | 0.0051 | 0.0018 | 0.0001 |
| Lower jaw | PAP C3 | 0.0011 | 0.0018 | 0.0002 |
| Lungs | Pet food | 0.0014 | 0.0018 | 0.0001 |
| Mask | Skin tannery C3 | 0.0017 | 0.0018 | 0.0030 |
| Mesenteric fat | C1-C2 for disposal | 0.0000 | 0.0000 | 0.0000 |
| Muscle | Human food | 0.0012 | 0.0018 | 0.0030 |
| Muzzle | Human food | 0.0017 | 0.0018 | 0.0018 |
| Omasum | Human food | 0.0094 | 0.0018 | 0.0014 |
| Omasum fat | Fat and greaves C3 | 0.0038 | 0.0018 | 0.0002 |
| Rumen and forestomach | Human food | 0.0094 | 0.0018 | 0.0014 |
| Rumen fat | Fat and greaves C3 | 0.0038 | 0.0018 | 0.0002 |
| Sanitary seizures | C1-C2 for disposal | 0.0000 | 0.0000 | 0.0000 |
| Screening and sifting wastes | C1-C2 for disposal | 0.0000 | 0.0000 | 0.0000 |
| Small intestine | PAP C3 | 0.0094 | 0.0018 | 0.0001 |
| Spinal cord | C1-C2 for disposal | 0.0000 | 0.0000 | 0.0000 |
| Spinal cord waste | C1-C2 for disposal | 0.0000 | 0.0000 | 0.0000 |
| Spine | C1-C2 for disposal | 0.0000 | 0.0000 | 0.0000 |
| Spleen | Pet food | 0.0012 | 0.0018 | 0.0001 |
| Stillborn | PAP C3 | 0.0098 | 0.0018 | 0.0000 |
| Tallow | Fat and greaves C3 | 0.0018 | 0.0018 | 0.0002 |
| Tongue | Human food | 0.0011 | 0.0018 | 0.0029 |
| Tonsil | C1-C2 for disposal | 0.0000 | 0.0000 | 0.0000 |
| Trachea | Pet food | 0.0017 | 0.0018 | 0.0001 |
| Udder | Pet food | 0.0003 | 0.0018 | 0.0001 |
| Upper throat | Pet food | 0.0012 | 0.0018 | 0.0001 |
| Water in the rumen | Spreading/Compost | 0.0000 | 0.0000 | 0.0000 |

Table 7: Allocation factors for Normande Cull Cows reared in Pasture

| COPRODUCT | Destination | Normande/Cull cow/pasture | | |
| --- | --- | --- | --- | --- |
|  |  | **Biophysical Allocation Factor** | **Mass Allocation Factor** | **Economic Allocation Factor** |
| Abomasum | Human food | 0.0085 | 0.0016 | 0.0012 |
| Abomasum fat | Fat and greaves C3 | 0.0033 | 0.0016 | 0.0001 |
| Aponeurosis | Human food | 0.0012 | 0.0016 | 0.0016 |
| Bile | PAP C3 | 0.0004 | 0.0016 | 0.0001 |
| Blood | PAP C3 | 0.0009 | 0.0016 | 0.0004 |
| Blood | Pet food | 0.0009 | 0.0016 | 0.0001 |
| Bones | Gelatin C3 | 0.0010 | 0.0016 | 0.0000 |
| Bones of head, brain, eyes and teeth | C1-C2 for disposal | 0.0000 | 0.0000 | 0.0000 |
| Cheek | Human food | 0.0011 | 0.0016 | 0.0036 |
| Cheek | Human food | 0.0011 | 0.0016 | 0.0036 |
| Cheek trimmings | Pet food | 0.0011 | 0.0016 | 0.0001 |
| Chops | Pet food | 0.0015 | 0.0016 | 0.0001 |
| Contents of intestines | Spreading/Compost | 0.0000 | 0.0000 | 0.0000 |
| Contents of the rumen | Spreading/Compost | 0.0000 | 0.0000 | 0.0000 |
| Ears | PAP C3 | 0.0013 | 0.0016 | 0.0001 |
| Esophagus | Pet food | 0.0010 | 0.0016 | 0.0001 |
| Fat | Fat and greaves C3 | 0.0015 | 0.0016 | 0.0001 |
| Fat around heart | Fat and greaves C3 | 0.0015 | 0.0016 | 0.0001 |
| Fat in the kidney | Fat and greaves C3 | 0.0015 | 0.0016 | 0.0001 |
| Feet (without hooves) | Gelatin C3 | 0.0010 | 0.0016 | 0.0000 |
| Floatation fat | Spreading/Compost | 0.0000 | 0.0000 | 0.0000 |
| Forehead | C1-C2 for disposal | 0.0000 | 0.0000 | 0.0000 |
| Forelock | PAP C3 | 0.0038 | 0.0016 | 0.0001 |
| Gallbladder | Pet food | 0.0010 | 0.0016 | 0.0001 |
| Head trimmings | Pet food | 0.0011 | 0.0016 | 0.0001 |
| Heart | Human food | 0.0010 | 0.0016 | 0.0003 |
| Heart trimmings | Pet food | 0.0011 | 0.0016 | 0.0001 |
| Hide | Skin tannery C3 | 0.0015 | 0.0016 | 0.0027 |
| Hooves | PAP C3 | 0.0038 | 0.0016 | 0.0001 |
| Horns | PAP C3 | 0.0038 | 0.0016 | 0.0001 |
| Kidney | Human food | 0.0010 | 0.0016 | 0.0007 |
| Large intestine | C1-C2 for disposal | 0.0000 | 0.0000 | 0.0000 |
| Liver | Human food | 0.0044 | 0.0016 | 0.0008 |
| Liver trimmings | Pet food | 0.0046 | 0.0016 | 0.0001 |
| Lower jaw | PAP C3 | 0.0010 | 0.0016 | 0.0001 |
| Lungs | Pet food | 0.0012 | 0.0016 | 0.0001 |
| Mask | Skin tannery C3 | 0.0015 | 0.0016 | 0.0027 |
| Mesenteric fat | C1-C2 for disposal | 0.0000 | 0.0000 | 0.0000 |
| Muscle | Human food | 0.0011 | 0.0016 | 0.0027 |
| Muzzle | Human food | 0.0015 | 0.0016 | 0.0016 |
| Omasum | Human food | 0.0085 | 0.0016 | 0.0012 |
| Omasum fat | Fat and greaves C3 | 0.0033 | 0.0016 | 0.0001 |
| Rumen and forestomach | Human food | 0.0085 | 0.0016 | 0.0012 |
| Rumen fat | Fat and greaves C3 | 0.0033 | 0.0016 | 0.0001 |
| Sanitary seizures | C1-C2 for disposal | 0.0000 | 0.0000 | 0.0000 |
| Screening and sifting wastes | C1-C2 for disposal | 0.0000 | 0.0000 | 0.0000 |
| Small intestine | PAP C3 | 0.0085 | 0.0016 | 0.0001 |
| Spinal cord | C1-C2 for disposal | 0.0000 | 0.0000 | 0.0000 |
| Spinal cord waste | C1-C2 for disposal | 0.0000 | 0.0000 | 0.0000 |
| Spine | C1-C2 for disposal | 0.0000 | 0.0000 | 0.0000 |
| Spleen | Pet food | 0.0011 | 0.0016 | 0.0001 |
| Stillborn | PAP C3 | 0.0089 | 0.0016 | 0.0000 |
| Tallow | Fat and greaves C3 | 0.0015 | 0.0016 | 0.0001 |
| Tongue | Human food | 0.0009 | 0.0016 | 0.0026 |
| Tonsil | C1-C2 for disposal | 0.0000 | 0.0000 | 0.0000 |
| Trachea | Pet food | 0.0015 | 0.0016 | 0.0001 |
| Udder | Pet food | 0.0002 | 0.0016 | 0.0001 |
| Upper throat | Pet food | 0.0010 | 0.0016 | 0.0001 |
| Water in the rumen | Spreading/Compost | 0.0000 | 0.0000 | 0.0000 |

Table 8: Allocation factors for Normande Beef reared in Pasture

| COPRODUCT | Destination | Normande/beef/pasture | | |
| --- | --- | --- | --- | --- |
|  |  | **Biophysical Allocation Factor** | **Mass Allocation Factor** | **Economic Allocation Factor** |
| Abomasum | Human food | 0.0082 | 0.0015 | 0.0011 |
| Abomasum fat | Fat and greaves C3 | 0.0032 | 0.0015 | 0.0001 |
| Aponeurosis | Human food | 0.0011 | 0.0015 | 0.0015 |
| Bile | PAP C3 | 0.0004 | 0.0015 | 0.0001 |
| Blood | PAP C3 | 0.0008 | 0.0015 | 0.0003 |
| Blood | Pet food | 0.0008 | 0.0015 | 0.0001 |
| Bones | Gelatin C3 | 0.0009 | 0.0015 | 0.0000 |
| Bones of head, brain, eyes and teeth | C1-C2 for disposal | 0.0000 | 0.0000 | 0.0000 |
| Cheek | Human food | 0.0010 | 0.0015 | 0.0033 |
| Cheek | Human food | 0.0010 | 0.0015 | 0.0033 |
| Cheek trimmings | Pet food | 0.0010 | 0.0015 | 0.0001 |
| Chops | Pet food | 0.0014 | 0.0015 | 0.0001 |
| Contents of intestines | Spreading/Compost | 0.0000 | 0.0000 | 0.0000 |
| Contents of the rumen | Spreading/Compost | 0.0000 | 0.0000 | 0.0000 |
| Ears | PAP C3 | 0.0012 | 0.0015 | 0.0001 |
| Esophagus | Pet food | 0.0010 | 0.0015 | 0.0001 |
| Fat | Fat and greaves C3 | 0.0015 | 0.0015 | 0.0001 |
| Fat around heart | Fat and greaves C3 | 0.0015 | 0.0015 | 0.0001 |
| Fat in the kidney | Fat and greaves C3 | 0.0015 | 0.0015 | 0.0001 |
| Feet (without hooves) | Gelatin C3 | 0.0010 | 0.0015 | 0.0000 |
| Floatation fat | Spreading/Compost | 0.0000 | 0.0000 | 0.0000 |
| Forehead | C1-C2 for disposal | 0.0000 | 0.0000 | 0.0000 |
| Forelock | PAP C3 | 0.0036 | 0.0015 | 0.0001 |
| Gallbladder | Pet food | 0.0010 | 0.0015 | 0.0001 |
| Head trimmings | Pet food | 0.0010 | 0.0015 | 0.0001 |
| Heart | Human food | 0.0010 | 0.0015 | 0.0003 |
| Heart trimmings | Pet food | 0.0010 | 0.0015 | 0.0001 |
| Hide | Skin tannery C3 | 0.0014 | 0.0015 | 0.0025 |
| Hooves | PAP C3 | 0.0036 | 0.0015 | 0.0001 |
| Horns | PAP C3 | 0.0036 | 0.0015 | 0.0001 |
| Kidney | Human food | 0.0010 | 0.0015 | 0.0006 |
| Large intestine | C1-C2 for disposal | 0.0000 | 0.0000 | 0.0000 |
| Liver | Human food | 0.0043 | 0.0015 | 0.0007 |
| Liver trimmings | Pet food | 0.0045 | 0.0015 | 0.0001 |
| Lower jaw | PAP C3 | 0.0009 | 0.0015 | 0.0001 |
| Lungs | Pet food | 0.0012 | 0.0015 | 0.0001 |
| Mask | Skin tannery C3 | 0.0014 | 0.0015 | 0.0025 |
| Mesenteric fat | C1-C2 for disposal | 0.0000 | 0.0000 | 0.0000 |
| Muscle | Human food | 0.0010 | 0.0015 | 0.0025 |
| Muzzle | Human food | 0.0014 | 0.0015 | 0.0015 |
| Omasum | Human food | 0.0082 | 0.0015 | 0.0011 |
| Omasum fat | Fat and greaves C3 | 0.0032 | 0.0015 | 0.0001 |
| Rumen and forestomach | Human food | 0.0082 | 0.0015 | 0.0011 |
| Rumen fat | Fat and greaves C3 | 0.0032 | 0.0015 | 0.0001 |
| Sanitary seizures | C1-C2 for disposal | 0.0000 | 0.0000 | 0.0000 |
| Screening and sifting wastes | C1-C2 for disposal | 0.0000 | 0.0000 | 0.0000 |
| Small intestine | PAP C3 | 0.0082 | 0.0015 | 0.0001 |
| Spinal cord | C1-C2 for disposal | 0.0000 | 0.0000 | 0.0000 |
| Spinal cord waste | C1-C2 for disposal | 0.0000 | 0.0000 | 0.0000 |
| Spine | C1-C2 for disposal | 0.0000 | 0.0000 | 0.0000 |
| Spleen | Pet food | 0.0010 | 0.0015 | 0.0001 |
| Stillborn | PAP C3 | 0.0086 | 0.0015 | 0.0000 |
| Tallow | Fat and greaves C3 | 0.0015 | 0.0015 | 0.0001 |
| Tongue | Human food | 0.0009 | 0.0015 | 0.0024 |
| Tonsil | C1-C2 for disposal | 0.0000 | 0.0000 | 0.0000 |
| Trachea | Pet food | 0.0014 | 0.0015 | 0.0001 |
| Udder | Pet food | 0.0002 | 0.0015 | 0.0001 |
| Upper throat | Pet food | 0.0010 | 0.0015 | 0.0001 |
| Water in the rumen | Spreading/Compost | 0.0000 | 0.0000 | 0.0000 |

Table 9: Allocation factors for Normande Young Bulls reared in Stall

| COPRODUCT | Destination | Normande/young bull/stall | | |
| --- | --- | --- | --- | --- |
|  |  | **Biophysical Allocation Factor** | **Mass Allocation Factor** | **Economic Allocation Factor** |
| Abomasum | Human food | 0.0083 | 0.0016 | 0.0012 |
| Abomasum fat | Fat and greaves C3 | 0.0035 | 0.0016 | 0.0001 |
| Aponeurosis | Human food | 0.0012 | 0.0016 | 0.0016 |
| Bile | PAP C3 | 0.0004 | 0.0016 | 0.0001 |
| Blood | PAP C3 | 0.0009 | 0.0016 | 0.0004 |
| Blood | Pet food | 0.0009 | 0.0016 | 0.0001 |
| Bones | Gelatin C3 | 0.0010 | 0.0016 | 0.0000 |
| Bones of head, brain, eyes and teeth | C1-C2 for disposal | 0.0000 | 0.0000 | 0.0000 |
| Cheek | Human food | 0.0011 | 0.0016 | 0.0035 |
| Cheek | Human food | 0.0011 | 0.0016 | 0.0035 |
| Cheek trimmings | Pet food | 0.0011 | 0.0016 | 0.0001 |
| Chops | Pet food | 0.0015 | 0.0016 | 0.0001 |
| Contents of intestines | Spreading/Compost | 0.0000 | 0.0000 | 0.0000 |
| Contents of the rumen | Spreading/Compost | 0.0000 | 0.0000 | 0.0000 |
| Ears | PAP C3 | 0.0014 | 0.0016 | 0.0001 |
| Esophagus | Pet food | 0.0011 | 0.0016 | 0.0001 |
| Fat | Fat and greaves C3 | 0.0018 | 0.0016 | 0.0001 |
| Fat around heart | Fat and greaves C3 | 0.0018 | 0.0016 | 0.0001 |
| Fat in the kidney | Fat and greaves C3 | 0.0018 | 0.0016 | 0.0001 |
| Feet (without hooves) | Gelatin C3 | 0.0011 | 0.0016 | 0.0000 |
| Floatation fat | Spreading/Compost | 0.0000 | 0.0000 | 0.0000 |
| Forehead | C1-C2 for disposal | 0.0000 | 0.0000 | 0.0000 |
| Forelock | PAP C3 | 0.0039 | 0.0016 | 0.0001 |
| Gallbladder | Pet food | 0.0011 | 0.0016 | 0.0001 |
| Head trimmings | Pet food | 0.0011 | 0.0016 | 0.0001 |
| Heart | Human food | 0.0010 | 0.0016 | 0.0003 |
| Heart trimmings | Pet food | 0.0011 | 0.0016 | 0.0001 |
| Hide | Skin tannery C3 | 0.0015 | 0.0016 | 0.0026 |
| Hooves | PAP C3 | 0.0039 | 0.0016 | 0.0001 |
| Horns | PAP C3 | 0.0039 | 0.0016 | 0.0001 |
| Kidney | Human food | 0.0011 | 0.0016 | 0.0006 |
| Large intestine | C1-C2 for disposal | 0.0000 | 0.0000 | 0.0000 |
| Liver | Human food | 0.0043 | 0.0016 | 0.0008 |
| Liver trimmings | Pet food | 0.0045 | 0.0016 | 0.0001 |
| Lower jaw | PAP C3 | 0.0010 | 0.0016 | 0.0001 |
| Lungs | Pet food | 0.0012 | 0.0016 | 0.0001 |
| Mask | Skin tannery C3 | 0.0015 | 0.0016 | 0.0026 |
| Mesenteric fat | C1-C2 for disposal | 0.0000 | 0.0000 | 0.0000 |
| Muscle | Human food | 0.0011 | 0.0016 | 0.0026 |
| Muzzle | Human food | 0.0015 | 0.0016 | 0.0016 |
| Omasum | Human food | 0.0083 | 0.0016 | 0.0012 |
| Omasum fat | Fat and greaves C3 | 0.0035 | 0.0016 | 0.0001 |
| Rumen and forestomach | Human food | 0.0083 | 0.0016 | 0.0012 |
| Rumen fat | Fat and greaves C3 | 0.0035 | 0.0016 | 0.0001 |
| Sanitary seizures | C1-C2 for disposal | 0.0000 | 0.0000 | 0.0000 |
| Screening and sifting wastes | C1-C2 for disposal | 0.0000 | 0.0000 | 0.0000 |
| Small intestine | PAP C3 | 0.0083 | 0.0016 | 0.0001 |
| Spinal cord | C1-C2 for disposal | 0.0000 | 0.0000 | 0.0000 |
| Spinal cord waste | C1-C2 for disposal | 0.0000 | 0.0000 | 0.0000 |
| Spine | C1-C2 for disposal | 0.0000 | 0.0000 | 0.0000 |
| Spleen | Pet food | 0.0011 | 0.0016 | 0.0001 |
| Stillborn | PAP C3 | 0.0086 | 0.0016 | 0.0000 |
| Tallow | Fat and greaves C3 | 0.0018 | 0.0016 | 0.0001 |
| Tongue | Human food | 0.0010 | 0.0016 | 0.0025 |
| Tonsil | C1-C2 for disposal | 0.0000 | 0.0000 | 0.0000 |
| Trachea | Pet food | 0.0015 | 0.0016 | 0.0001 |
| Udder | Pet food | 0.0002 | 0.0016 | 0.0001 |
| Upper throat | Pet food | 0.0011 | 0.0016 | 0.0001 |
| Water in the rumen | Spreading/Compost | 0.0000 | 0.0000 | 0.0000 |

Table 10: Allocation factors for Normande Heifers reared in Stall

| COPRODUCT | Destination | Normande/heifer/stall | | |
| --- | --- | --- | --- | --- |
|  |  | **Biophysical Allocation Factor** | **Mass Allocation Factor** | **Economic Allocation Factor** |
| Abomasum | Human food | 0.0089 | 0.0018 | 0.0014 |
| Abomasum fat | Fat and greaves C3 | 0.0038 | 0.0018 | 0.0002 |
| Aponeurosis | Human food | 0.0013 | 0.0018 | 0.0018 |
| Bile | PAP C3 | 0.0005 | 0.0018 | 0.0002 |
| Blood | PAP C3 | 0.0010 | 0.0018 | 0.0004 |
| Blood | Pet food | 0.0010 | 0.0018 | 0.0001 |
| Bones | Gelatin C3 | 0.0012 | 0.0018 | 0.0000 |
| Bones of head, brain, eyes and teeth | C1-C2 for disposal | 0.0000 | 0.0000 | 0.0000 |
| Cheek | Human food | 0.0012 | 0.0018 | 0.0040 |
| Cheek | Human food | 0.0012 | 0.0018 | 0.0040 |
| Cheek trimmings | Pet food | 0.0012 | 0.0018 | 0.0001 |
| Chops | Pet food | 0.0017 | 0.0018 | 0.0001 |
| Contents of intestines | Spreading/Compost | 0.0000 | 0.0000 | 0.0000 |
| Contents of the rumen | Spreading/Compost | 0.0000 | 0.0000 | 0.0000 |
| Ears | PAP C3 | 0.0015 | 0.0018 | 0.0002 |
| Esophagus | Pet food | 0.0012 | 0.0018 | 0.0001 |
| Fat | Fat and greaves C3 | 0.0020 | 0.0018 | 0.0002 |
| Fat around heart | Fat and greaves C3 | 0.0020 | 0.0018 | 0.0002 |
| Fat in the kidney | Fat and greaves C3 | 0.0020 | 0.0018 | 0.0002 |
| Feet (without hooves) | Gelatin C3 | 0.0012 | 0.0018 | 0.0000 |
| Floatation fat | Spreading/Compost | 0.0000 | 0.0000 | 0.0000 |
| Forehead | C1-C2 for disposal | 0.0000 | 0.0000 | 0.0000 |
| Forelock | PAP C3 | 0.0043 | 0.0018 | 0.0002 |
| Gallbladder | Pet food | 0.0012 | 0.0018 | 0.0001 |
| Head trimmings | Pet food | 0.0012 | 0.0018 | 0.0001 |
| Heart | Human food | 0.0012 | 0.0018 | 0.0004 |
| Heart trimmings | Pet food | 0.0012 | 0.0018 | 0.0001 |
| Hide | Skin tannery C3 | 0.0017 | 0.0018 | 0.0030 |
| Hooves | PAP C3 | 0.0043 | 0.0018 | 0.0002 |
| Horns | PAP C3 | 0.0043 | 0.0018 | 0.0002 |
| Kidney | Human food | 0.0012 | 0.0018 | 0.0007 |
| Large intestine | C1-C2 for disposal | 0.0000 | 0.0000 | 0.0000 |
| Liver | Human food | 0.0047 | 0.0018 | 0.0009 |
| Liver trimmings | Pet food | 0.0049 | 0.0018 | 0.0001 |
| Lower jaw | PAP C3 | 0.0011 | 0.0018 | 0.0002 |
| Lungs | Pet food | 0.0014 | 0.0018 | 0.0001 |
| Mask | Skin tannery C3 | 0.0017 | 0.0018 | 0.0030 |
| Mesenteric fat | C1-C2 for disposal | 0.0000 | 0.0000 | 0.0000 |
| Muscle | Human food | 0.0012 | 0.0018 | 0.0030 |
| Muzzle | Human food | 0.0017 | 0.0018 | 0.0018 |
| Omasum | Human food | 0.0089 | 0.0018 | 0.0014 |
| Omasum fat | Fat and greaves C3 | 0.0038 | 0.0018 | 0.0002 |
| Rumen and forestomach | Human food | 0.0089 | 0.0018 | 0.0014 |
| Rumen fat | Fat and greaves C3 | 0.0038 | 0.0018 | 0.0002 |
| Sanitary seizures | C1-C2 for disposal | 0.0000 | 0.0000 | 0.0000 |
| Screening and sifting wastes | C1-C2 for disposal | 0.0000 | 0.0000 | 0.0000 |
| Small intestine | PAP C3 | 0.0089 | 0.0018 | 0.0001 |
| Spinal cord | C1-C2 for disposal | 0.0000 | 0.0000 | 0.0000 |
| Spinal cord waste | C1-C2 for disposal | 0.0000 | 0.0000 | 0.0000 |
| Spine | C1-C2 for disposal | 0.0000 | 0.0000 | 0.0000 |
| Spleen | Pet food | 0.0012 | 0.0018 | 0.0001 |
| Stillborn | PAP C3 | 0.0093 | 0.0018 | 0.0000 |
| Tallow | Fat and greaves C3 | 0.0020 | 0.0018 | 0.0002 |
| Tongue | Human food | 0.0011 | 0.0018 | 0.0029 |
| Tonsil | C1-C2 for disposal | 0.0000 | 0.0000 | 0.0000 |
| Trachea | Pet food | 0.0017 | 0.0018 | 0.0001 |
| Udder | Pet food | 0.0003 | 0.0018 | 0.0001 |
| Upper throat | Pet food | 0.0012 | 0.0018 | 0.0001 |
| Water in the rumen | Spreading/Compost | 0.0000 | 0.0000 | 0.0000 |

Table 11: Allocation factors for Normande Cull Cows reared in Stall

| COPRODUCT | Destination | Normande/Cull cow/stall | | |
| --- | --- | --- | --- | --- |
|  |  | **Biophysical Allocation Factor** | **Mass Allocation Factor** | **Economic Allocation Factor** |
| Abomasum | Human food | 0.0081 | 0.0016 | 0.0012 |
| Abomasum fat | Fat and greaves C3 | 0.0034 | 0.0016 | 0.0001 |
| Aponeurosis | Human food | 0.0011 | 0.0016 | 0.0016 |
| Bile | PAP C3 | 0.0004 | 0.0016 | 0.0001 |
| Blood | PAP C3 | 0.0009 | 0.0016 | 0.0004 |
| Blood | Pet food | 0.0009 | 0.0016 | 0.0001 |
| Bones | Gelatin C3 | 0.0010 | 0.0016 | 0.0000 |
| Bones of head, brain, eyes and teeth | C1-C2 for disposal | 0.0000 | 0.0000 | 0.0000 |
| Cheek | Human food | 0.0010 | 0.0016 | 0.0036 |
| Cheek | Human food | 0.0010 | 0.0016 | 0.0036 |
| Cheek trimmings | Pet food | 0.0010 | 0.0016 | 0.0001 |
| Chops | Pet food | 0.0015 | 0.0016 | 0.0001 |
| Contents of intestines | Spreading/Compost | 0.0000 | 0.0000 | 0.0000 |
| Contents of the rumen | Spreading/Compost | 0.0000 | 0.0000 | 0.0000 |
| Ears | PAP C3 | 0.0013 | 0.0016 | 0.0001 |
| Esophagus | Pet food | 0.0010 | 0.0016 | 0.0001 |
| Fat | Fat and greaves C3 | 0.0017 | 0.0016 | 0.0001 |
| Fat around heart | Fat and greaves C3 | 0.0017 | 0.0016 | 0.0001 |
| Fat in the kidney | Fat and greaves C3 | 0.0017 | 0.0016 | 0.0001 |
| Feet (without hooves) | Gelatin C3 | 0.0010 | 0.0016 | 0.0000 |
| Floatation fat | Spreading/Compost | 0.0000 | 0.0000 | 0.0000 |
| Forehead | C1-C2 for disposal | 0.0000 | 0.0000 | 0.0000 |
| Forelock | PAP C3 | 0.0037 | 0.0016 | 0.0001 |
| Gallbladder | Pet food | 0.0010 | 0.0016 | 0.0001 |
| Head trimmings | Pet food | 0.0010 | 0.0016 | 0.0001 |
| Heart | Human food | 0.0010 | 0.0016 | 0.0003 |
| Heart trimmings | Pet food | 0.0010 | 0.0016 | 0.0001 |
| Hide | Skin tannery C3 | 0.0015 | 0.0016 | 0.0027 |
| Hooves | PAP C3 | 0.0037 | 0.0016 | 0.0001 |
| Horns | PAP C3 | 0.0037 | 0.0016 | 0.0001 |
| Kidney | Human food | 0.0010 | 0.0016 | 0.0007 |
| Large intestine | C1-C2 for disposal | 0.0000 | 0.0000 | 0.0000 |
| Liver | Human food | 0.0042 | 0.0016 | 0.0008 |
| Liver trimmings | Pet food | 0.0044 | 0.0016 | 0.0001 |
| Lower jaw | PAP C3 | 0.0010 | 0.0016 | 0.0001 |
| Lungs | Pet food | 0.0012 | 0.0016 | 0.0001 |
| Mask | Skin tannery C3 | 0.0015 | 0.0016 | 0.0027 |
| Mesenteric fat | C1-C2 for disposal | 0.0000 | 0.0000 | 0.0000 |
| Muscle | Human food | 0.0011 | 0.0016 | 0.0027 |
| Muzzle | Human food | 0.0015 | 0.0016 | 0.0016 |
| Omasum | Human food | 0.0081 | 0.0016 | 0.0012 |
| Omasum fat | Fat and greaves C3 | 0.0034 | 0.0016 | 0.0001 |
| Rumen and forestomach | Human food | 0.0081 | 0.0016 | 0.0012 |
| Rumen fat | Fat and greaves C3 | 0.0034 | 0.0016 | 0.0001 |
| Sanitary seizures | C1-C2 for disposal | 0.0000 | 0.0000 | 0.0000 |
| Screening and sifting wastes | C1-C2 for disposal | 0.0000 | 0.0000 | 0.0000 |
| Small intestine | PAP C3 | 0.0081 | 0.0016 | 0.0001 |
| Spinal cord | C1-C2 for disposal | 0.0000 | 0.0000 | 0.0000 |
| Spinal cord waste | C1-C2 for disposal | 0.0000 | 0.0000 | 0.0000 |
| Spine | C1-C2 for disposal | 0.0000 | 0.0000 | 0.0000 |
| Spleen | Pet food | 0.0011 | 0.0016 | 0.0001 |
| Stillborn | PAP C3 | 0.0084 | 0.0016 | 0.0000 |
| Tallow | Fat and greaves C3 | 0.0017 | 0.0016 | 0.0001 |
| Tongue | Human food | 0.0009 | 0.0016 | 0.0026 |
| Tonsil | C1-C2 for disposal | 0.0000 | 0.0000 | 0.0000 |
| Trachea | Pet food | 0.0015 | 0.0016 | 0.0001 |
| Udder | Pet food | 0.0002 | 0.0016 | 0.0001 |
| Upper throat | Pet food | 0.0010 | 0.0016 | 0.0001 |
| Water in the rumen | Spreading/Compost | 0.0000 | 0.0000 | 0.0000 |

Table 12: Allocation factors for Normande Beef reared in Stall

| COPRODUCT | Destination | Normande/beef/stall | | |
| --- | --- | --- | --- | --- |
|  |  | **Biophysical Allocation Factor** | **Mass Allocation Factor** | **Economic Allocation Factor** |
| Abomasum | Human food | 0.0078 | 0.0015 | 0.0011 |
| Abomasum fat | Fat and greaves C3 | 0.0032 | 0.0015 | 0.0001 |
| Aponeurosis | Human food | 0.0011 | 0.0015 | 0.0015 |
| Bile | PAP C3 | 0.0004 | 0.0015 | 0.0001 |
| Blood | PAP C3 | 0.0008 | 0.0015 | 0.0003 |
| Blood | Pet food | 0.0008 | 0.0015 | 0.0001 |
| Bones | Gelatin C3 | 0.0009 | 0.0015 | 0.0000 |
| Bones of head, brain, eyes and teeth | C1-C2 for disposal | 0.0000 | 0.0000 | 0.0000 |
| Cheek | Human food | 0.0010 | 0.0015 | 0.0033 |
| Cheek | Human food | 0.0010 | 0.0015 | 0.0033 |
| Cheek trimmings | Pet food | 0.0010 | 0.0015 | 0.0001 |
| Chops | Pet food | 0.0014 | 0.0015 | 0.0001 |
| Contents of intestines | Spreading/Compost | 0.0000 | 0.0000 | 0.0000 |
| Contents of the rumen | Spreading/Compost | 0.0000 | 0.0000 | 0.0000 |
| Ears | PAP C3 | 0.0012 | 0.0015 | 0.0001 |
| Esophagus | Pet food | 0.0010 | 0.0015 | 0.0001 |
| Fat | Fat and greaves C3 | 0.0016 | 0.0015 | 0.0001 |
| Fat around heart | Fat and greaves C3 | 0.0016 | 0.0015 | 0.0001 |
| Fat in the kidney | Fat and greaves C3 | 0.0016 | 0.0015 | 0.0001 |
| Feet (without hooves) | Gelatin C3 | 0.0010 | 0.0015 | 0.0000 |
| Floatation fat | Spreading/Compost | 0.0000 | 0.0000 | 0.0000 |
| Forehead | C1-C2 for disposal | 0.0000 | 0.0000 | 0.0000 |
| Forelock | PAP C3 | 0.0035 | 0.0015 | 0.0001 |
| Gallbladder | Pet food | 0.0010 | 0.0015 | 0.0001 |
| Head trimmings | Pet food | 0.0010 | 0.0015 | 0.0001 |
| Heart | Human food | 0.0009 | 0.0015 | 0.0003 |
| Heart trimmings | Pet food | 0.0010 | 0.0015 | 0.0001 |
| Hide | Skin tannery C3 | 0.0014 | 0.0015 | 0.0025 |
| Hooves | PAP C3 | 0.0035 | 0.0015 | 0.0001 |
| Horns | PAP C3 | 0.0035 | 0.0015 | 0.0001 |
| Kidney | Human food | 0.0010 | 0.0015 | 0.0006 |
| Large intestine | C1-C2 for disposal | 0.0000 | 0.0000 | 0.0000 |
| Liver | Human food | 0.0041 | 0.0015 | 0.0007 |
| Liver trimmings | Pet food | 0.0043 | 0.0015 | 0.0001 |
| Lower jaw | PAP C3 | 0.0009 | 0.0015 | 0.0001 |
| Lungs | Pet food | 0.0011 | 0.0015 | 0.0001 |
| Mask | Skin tannery C3 | 0.0014 | 0.0015 | 0.0025 |
| Mesenteric fat | C1-C2 for disposal | 0.0000 | 0.0000 | 0.0000 |
| Muscle | Human food | 0.0010 | 0.0015 | 0.0025 |
| Muzzle | Human food | 0.0014 | 0.0015 | 0.0015 |
| Omasum | Human food | 0.0078 | 0.0015 | 0.0011 |
| Omasum fat | Fat and greaves C3 | 0.0032 | 0.0015 | 0.0001 |
| Rumen and forestomach | Human food | 0.0078 | 0.0015 | 0.0011 |
| Rumen fat | Fat and greaves C3 | 0.0032 | 0.0015 | 0.0001 |
| Sanitary seizures | C1-C2 for disposal | 0.0000 | 0.0000 | 0.0000 |
| Screening and sifting wastes | C1-C2 for disposal | 0.0000 | 0.0000 | 0.0000 |
| Small intestine | PAP C3 | 0.0078 | 0.0015 | 0.0001 |
| Spinal cord | C1-C2 for disposal | 0.0000 | 0.0000 | 0.0000 |
| Spinal cord waste | C1-C2 for disposal | 0.0000 | 0.0000 | 0.0000 |
| Spine | C1-C2 for disposal | 0.0000 | 0.0000 | 0.0000 |
| Spleen | Pet food | 0.0010 | 0.0015 | 0.0001 |
| Stillborn | PAP C3 | 0.0082 | 0.0015 | 0.0000 |
| Tallow | Fat and greaves C3 | 0.0016 | 0.0015 | 0.0001 |
| Tongue | Human food | 0.0009 | 0.0015 | 0.0024 |
| Tonsil | C1-C2 for disposal | 0.0000 | 0.0000 | 0.0000 |
| Trachea | Pet food | 0.0014 | 0.0015 | 0.0001 |
| Udder | Pet food | 0.0002 | 0.0015 | 0.0001 |
| Upper throat | Pet food | 0.0010 | 0.0015 | 0.0001 |
| Water in the rumen | Spreading/Compost | 0.0000 | 0.0000 | 0.0000 |

Table 13: Allocation factors for Charolaise Young Bulls reared in Grazing Large Area

| COPRODUCT | Destination | Charolaise/young bull/grazing large area | | |
| --- | --- | --- | --- | --- |
|  |  | **Biophysical Allocation Factor** | **Mass Allocation Factor** | **Economic Allocation Factor** |
| Abomasum | Human food | 0.0087 | 0.0014 | 0.0011 |
| Abomasum fat | Fat and greaves C3 | 0.0032 | 0.0014 | 0.0001 |
| Aponeurosis | Human food | 0.0011 | 0.0014 | 0.0014 |
| Bile | PAP C3 | 0.0004 | 0.0014 | 0.0001 |
| Blood | PAP C3 | 0.0009 | 0.0014 | 0.0003 |
| Blood | Pet food | 0.0009 | 0.0014 | 0.0001 |
| Bones | Gelatin C3 | 0.0009 | 0.0014 | 0.0000 |
| Bones of head, brain, eyes and teeth | C1-C2 for disposal | 0.0000 | 0.0000 | 0.0000 |
| Cheek | Human food | 0.0010 | 0.0014 | 0.0031 |
| Cheek | Human food | 0.0010 | 0.0014 | 0.0031 |
| Cheek trimmings | Pet food | 0.0010 | 0.0014 | 0.0001 |
| Chops | Pet food | 0.0014 | 0.0014 | 0.0001 |
| Contents of intestines | Spreading/Compost | 0.0000 | 0.0000 | 0.0000 |
| Contents of the rumen | Spreading/Compost | 0.0000 | 0.0000 | 0.0000 |
| Ears | PAP C3 | 0.0012 | 0.0014 | 0.0001 |
| Esophagus | Pet food | 0.0010 | 0.0014 | 0.0001 |
| Fat | Fat and greaves C3 | 0.0013 | 0.0014 | 0.0001 |
| Fat around heart | Fat and greaves C3 | 0.0013 | 0.0014 | 0.0001 |
| Fat in the kidney | Fat and greaves C3 | 0.0013 | 0.0014 | 0.0001 |
| Feet (without hooves) | Gelatin C3 | 0.0010 | 0.0014 | 0.0000 |
| Floatation fat | Spreading/Compost | 0.0000 | 0.0000 | 0.0000 |
| Forehead | C1-C2 for disposal | 0.0000 | 0.0000 | 0.0000 |
| Forelock | PAP C3 | 0.0036 | 0.0014 | 0.0001 |
| Gallbladder | Pet food | 0.0010 | 0.0014 | 0.0001 |
| Head trimmings | Pet food | 0.0010 | 0.0014 | 0.0001 |
| Heart | Human food | 0.0010 | 0.0014 | 0.0003 |
| Heart trimmings | Pet food | 0.0010 | 0.0014 | 0.0001 |
| Hide | Skin tannery C3 | 0.0014 | 0.0014 | 0.0023 |
| Hooves | PAP C3 | 0.0036 | 0.0014 | 0.0001 |
| Horns | PAP C3 | 0.0036 | 0.0014 | 0.0001 |
| Kidney | Human food | 0.0010 | 0.0014 | 0.0006 |
| Large intestine | C1-C2 for disposal | 0.0000 | 0.0000 | 0.0000 |
| Liver | Human food | 0.0045 | 0.0014 | 0.0007 |
| Liver trimmings | Pet food | 0.0047 | 0.0014 | 0.0001 |
| Lower jaw | PAP C3 | 0.0009 | 0.0014 | 0.0001 |
| Lungs | Pet food | 0.0012 | 0.0014 | 0.0001 |
| Mask | Skin tannery C3 | 0.0014 | 0.0014 | 0.0023 |
| Mesenteric fat | C1-C2 for disposal | 0.0000 | 0.0000 | 0.0000 |
| Muscle | Human food | 0.0010 | 0.0014 | 0.0024 |
| Muzzle | Human food | 0.0014 | 0.0014 | 0.0014 |
| Omasum | Human food | 0.0087 | 0.0014 | 0.0011 |
| Omasum fat | Fat and greaves C3 | 0.0032 | 0.0014 | 0.0001 |
| Rumen and forestomach | Human food | 0.0087 | 0.0014 | 0.0011 |
| Rumen fat | Fat and greaves C3 | 0.0032 | 0.0014 | 0.0001 |
| Sanitary seizures | C1-C2 for disposal | 0.0000 | 0.0000 | 0.0000 |
| Screening and sifting wastes | C1-C2 for disposal | 0.0000 | 0.0000 | 0.0000 |
| Small intestine | PAP C3 | 0.0087 | 0.0014 | 0.0001 |
| Spinal cord | C1-C2 for disposal | 0.0000 | 0.0000 | 0.0000 |
| Spinal cord waste | C1-C2 for disposal | 0.0000 | 0.0000 | 0.0000 |
| Spine | C1-C2 for disposal | 0.0000 | 0.0000 | 0.0000 |
| Spleen | Pet food | 0.0010 | 0.0014 | 0.0001 |
| Stillborn | PAP C3 | 0.0091 | 0.0014 | 0.0000 |
| Tallow | Fat and greaves C3 | 0.0013 | 0.0014 | 0.0001 |
| Tongue | Human food | 0.0009 | 0.0014 | 0.0022 |
| Tonsil | C1-C2 for disposal | 0.0000 | 0.0000 | 0.0000 |
| Trachea | Pet food | 0.0014 | 0.0014 | 0.0001 |
| Udder | Pet food | 0.0002 | 0.0014 | 0.0001 |
| Upper throat | Pet food | 0.0010 | 0.0014 | 0.0001 |
| Water in the rumen | Spreading/Compost | 0.0000 | 0.0000 | 0.0000 |

Table 14: Allocation factors for Charolaise Heifers reared in Grazing Large Area

| COPRODUCT | Destination | Charolaise/heifer/grazing large area | | |
| --- | --- | --- | --- | --- |
|  |  | **Biophysical Allocation Factor** | **Mass Allocation Factor** | **Economic Allocation Factor** |
| Abomasum | Human food | 0.0089 | 0.0015 | 0.0011 |
| Abomasum fat | Fat and greaves C3 | 0.0033 | 0.0015 | 0.0001 |
| Aponeurosis | Human food | 0.0012 | 0.0015 | 0.0015 |
| Bile | PAP C3 | 0.0004 | 0.0015 | 0.0001 |
| Blood | PAP C3 | 0.0009 | 0.0015 | 0.0003 |
| Blood | Pet food | 0.0009 | 0.0015 | 0.0001 |
| Bones | Gelatin C3 | 0.0010 | 0.0015 | 0.0000 |
| Bones of head, brain, eyes and teeth | C1-C2 for disposal | 0.0000 | 0.0000 | 0.0000 |
| Cheek | Human food | 0.0011 | 0.0015 | 0.0034 |
| Cheek | Human food | 0.0011 | 0.0015 | 0.0034 |
| Cheek trimmings | Pet food | 0.0011 | 0.0015 | 0.0001 |
| Chops | Pet food | 0.0015 | 0.0015 | 0.0001 |
| Contents of intestines | Spreading/Compost | 0.0000 | 0.0000 | 0.0000 |
| Contents of the rumen | Spreading/Compost | 0.0000 | 0.0000 | 0.0000 |
| Ears | PAP C3 | 0.0013 | 0.0015 | 0.0001 |
| Esophagus | Pet food | 0.0010 | 0.0015 | 0.0001 |
| Fat | Fat and greaves C3 | 0.0014 | 0.0015 | 0.0001 |
| Fat around heart | Fat and greaves C3 | 0.0014 | 0.0015 | 0.0001 |
| Fat in the kidney | Fat and greaves C3 | 0.0014 | 0.0015 | 0.0001 |
| Feet (without hooves) | Gelatin C3 | 0.0010 | 0.0015 | 0.0000 |
| Floatation fat | Spreading/Compost | 0.0000 | 0.0000 | 0.0000 |
| Forehead | C1-C2 for disposal | 0.0000 | 0.0000 | 0.0000 |
| Forelock | PAP C3 | 0.0038 | 0.0015 | 0.0001 |
| Gallbladder | Pet food | 0.0010 | 0.0015 | 0.0001 |
| Head trimmings | Pet food | 0.0011 | 0.0015 | 0.0001 |
| Heart | Human food | 0.0010 | 0.0015 | 0.0003 |
| Heart trimmings | Pet food | 0.0011 | 0.0015 | 0.0001 |
| Hide | Skin tannery C3 | 0.0015 | 0.0015 | 0.0026 |
| Hooves | PAP C3 | 0.0038 | 0.0015 | 0.0001 |
| Horns | PAP C3 | 0.0038 | 0.0015 | 0.0001 |
| Kidney | Human food | 0.0010 | 0.0015 | 0.0006 |
| Large intestine | C1-C2 for disposal | 0.0000 | 0.0000 | 0.0000 |
| Liver | Human food | 0.0046 | 0.0015 | 0.0007 |
| Liver trimmings | Pet food | 0.0048 | 0.0015 | 0.0001 |
| Lower jaw | PAP C3 | 0.0009 | 0.0015 | 0.0001 |
| Lungs | Pet food | 0.0012 | 0.0015 | 0.0001 |
| Mask | Skin tannery C3 | 0.0015 | 0.0015 | 0.0026 |
| Mesenteric fat | C1-C2 for disposal | 0.0000 | 0.0000 | 0.0000 |
| Muscle | Human food | 0.0011 | 0.0015 | 0.0026 |
| Muzzle | Human food | 0.0015 | 0.0015 | 0.0015 |
| Omasum | Human food | 0.0089 | 0.0015 | 0.0011 |
| Omasum fat | Fat and greaves C3 | 0.0033 | 0.0015 | 0.0001 |
| Rumen and forestomach | Human food | 0.0089 | 0.0015 | 0.0011 |
| Rumen fat | Fat and greaves C3 | 0.0033 | 0.0015 | 0.0001 |
| Sanitary seizures | C1-C2 for disposal | 0.0000 | 0.0000 | 0.0000 |
| Screening and sifting wastes | C1-C2 for disposal | 0.0000 | 0.0000 | 0.0000 |
| Small intestine | PAP C3 | 0.0089 | 0.0015 | 0.0001 |
| Spinal cord | C1-C2 for disposal | 0.0000 | 0.0000 | 0.0000 |
| Spinal cord waste | C1-C2 for disposal | 0.0000 | 0.0000 | 0.0000 |
| Spine | C1-C2 for disposal | 0.0000 | 0.0000 | 0.0000 |
| Spleen | Pet food | 0.0011 | 0.0015 | 0.0001 |
| Stillborn | PAP C3 | 0.0093 | 0.0015 | 0.0000 |
| Tallow | Fat and greaves C3 | 0.0014 | 0.0015 | 0.0001 |
| Tongue | Human food | 0.0009 | 0.0015 | 0.0024 |
| Tonsil | C1-C2 for disposal | 0.0000 | 0.0000 | 0.0000 |
| Trachea | Pet food | 0.0015 | 0.0015 | 0.0001 |
| Udder | Pet food | 0.0002 | 0.0015 | 0.0001 |
| Upper throat | Pet food | 0.0010 | 0.0015 | 0.0001 |
| Water in the rumen | Spreading/Compost | 0.0000 | 0.0000 | 0.0000 |

Table 15: Allocation factors for Charolaise Cull Cows reared in Grazing Large Area

| COPRODUCT | Destination | Charolaise/Cull cow/grazing large area | | |
| --- | --- | --- | --- | --- |
|  |  | **Biophysical Allocation Factor** | **Mass Allocation Factor** | **Economic Allocation Factor** |
| Abomasum | Human food | 0.0082 | 0.0014 | 0.0010 |
| Abomasum fat | Fat and greaves C3 | 0.0030 | 0.0014 | 0.0001 |
| Aponeurosis | Human food | 0.0010 | 0.0014 | 0.0014 |
| Bile | PAP C3 | 0.0004 | 0.0014 | 0.0001 |
| Blood | PAP C3 | 0.0008 | 0.0014 | 0.0003 |
| Blood | Pet food | 0.0008 | 0.0014 | 0.0001 |
| Bones | Gelatin C3 | 0.0008 | 0.0014 | 0.0000 |
| Bones of head, brain, eyes and teeth | C1-C2 for disposal | 0.0000 | 0.0000 | 0.0000 |
| Cheek | Human food | 0.0009 | 0.0014 | 0.0030 |
| Cheek | Human food | 0.0009 | 0.0014 | 0.0030 |
| Cheek trimmings | Pet food | 0.0009 | 0.0014 | 0.0001 |
| Chops | Pet food | 0.0013 | 0.0014 | 0.0001 |
| Contents of intestines | Spreading/Compost | 0.0000 | 0.0000 | 0.0000 |
| Contents of the rumen | Spreading/Compost | 0.0000 | 0.0000 | 0.0000 |
| Ears | PAP C3 | 0.0011 | 0.0014 | 0.0001 |
| Esophagus | Pet food | 0.0009 | 0.0014 | 0.0001 |
| Fat | Fat and greaves C3 | 0.0012 | 0.0014 | 0.0001 |
| Fat around heart | Fat and greaves C3 | 0.0012 | 0.0014 | 0.0001 |
| Fat in the kidney | Fat and greaves C3 | 0.0012 | 0.0014 | 0.0001 |
| Feet (without hooves) | Gelatin C3 | 0.0009 | 0.0014 | 0.0000 |
| Floatation fat | Spreading/Compost | 0.0000 | 0.0000 | 0.0000 |
| Forehead | C1-C2 for disposal | 0.0000 | 0.0000 | 0.0000 |
| Forelock | PAP C3 | 0.0033 | 0.0014 | 0.0001 |
| Gallbladder | Pet food | 0.0009 | 0.0014 | 0.0001 |
| Head trimmings | Pet food | 0.0009 | 0.0014 | 0.0001 |
| Heart | Human food | 0.0009 | 0.0014 | 0.0003 |
| Heart trimmings | Pet food | 0.0009 | 0.0014 | 0.0001 |
| Hide | Skin tannery C3 | 0.0013 | 0.0014 | 0.0023 |
| Hooves | PAP C3 | 0.0033 | 0.0014 | 0.0001 |
| Horns | PAP C3 | 0.0033 | 0.0014 | 0.0001 |
| Kidney | Human food | 0.0009 | 0.0014 | 0.0006 |
| Large intestine | C1-C2 for disposal | 0.0000 | 0.0000 | 0.0000 |
| Liver | Human food | 0.0042 | 0.0014 | 0.0007 |
| Liver trimmings | Pet food | 0.0044 | 0.0014 | 0.0001 |
| Lower jaw | PAP C3 | 0.0008 | 0.0014 | 0.0001 |
| Lungs | Pet food | 0.0011 | 0.0014 | 0.0001 |
| Mask | Skin tannery C3 | 0.0013 | 0.0014 | 0.0023 |
| Mesenteric fat | C1-C2 for disposal | 0.0000 | 0.0000 | 0.0000 |
| Muscle | Human food | 0.0009 | 0.0014 | 0.0023 |
| Muzzle | Human food | 0.0013 | 0.0014 | 0.0014 |
| Omasum | Human food | 0.0082 | 0.0014 | 0.0010 |
| Omasum fat | Fat and greaves C3 | 0.0030 | 0.0014 | 0.0001 |
| Rumen and forestomach | Human food | 0.0082 | 0.0014 | 0.0010 |
| Rumen fat | Fat and greaves C3 | 0.0030 | 0.0014 | 0.0001 |
| Sanitary seizures | C1-C2 for disposal | 0.0000 | 0.0000 | 0.0000 |
| Screening and sifting wastes | C1-C2 for disposal | 0.0000 | 0.0000 | 0.0000 |
| Small intestine | PAP C3 | 0.0082 | 0.0014 | 0.0001 |
| Spinal cord | C1-C2 for disposal | 0.0000 | 0.0000 | 0.0000 |
| Spinal cord waste | C1-C2 for disposal | 0.0000 | 0.0000 | 0.0000 |
| Spine | C1-C2 for disposal | 0.0000 | 0.0000 | 0.0000 |
| Spleen | Pet food | 0.0009 | 0.0014 | 0.0001 |
| Stillborn | PAP C3 | 0.0085 | 0.0014 | 0.0000 |
| Tallow | Fat and greaves C3 | 0.0012 | 0.0014 | 0.0001 |
| Tongue | Human food | 0.0008 | 0.0014 | 0.0022 |
| Tonsil | C1-C2 for disposal | 0.0000 | 0.0000 | 0.0000 |
| Trachea | Pet food | 0.0013 | 0.0014 | 0.0001 |
| Udder | Pet food | 0.0002 | 0.0014 | 0.0001 |
| Upper throat | Pet food | 0.0009 | 0.0014 | 0.0001 |
| Water in the rumen | Spreading/Compost | 0.0000 | 0.0000 | 0.0000 |

Table 16: Allocation factors for Charolaise Beef reared in Grazing Large Area

| COPRODUCT | Destination | Charolaise/beef/grazing large area | | |
| --- | --- | --- | --- | --- |
|  |  | **Biophysical Allocation Factor** | **Mass Allocation Factor** | **Economic Allocation Factor** |
| Abomasum | Human food | 0.0081 | 0.0013 | 0.0010 |
| Abomasum fat | Fat and greaves C3 | 0.0029 | 0.0013 | 0.0001 |
| Aponeurosis | Human food | 0.0010 | 0.0013 | 0.0013 |
| Bile | PAP C3 | 0.0004 | 0.0013 | 0.0001 |
| Blood | PAP C3 | 0.0008 | 0.0013 | 0.0003 |
| Blood | Pet food | 0.0008 | 0.0013 | 0.0001 |
| Bones | Gelatin C3 | 0.0008 | 0.0013 | 0.0000 |
| Bones of head, brain, eyes and teeth | C1-C2 for disposal | 0.0000 | 0.0000 | 0.0000 |
| Cheek | Human food | 0.0009 | 0.0013 | 0.0029 |
| Cheek | Human food | 0.0009 | 0.0013 | 0.0029 |
| Cheek trimmings | Pet food | 0.0009 | 0.0013 | 0.0001 |
| Chops | Pet food | 0.0013 | 0.0013 | 0.0001 |
| Contents of intestines | Spreading/Compost | 0.0000 | 0.0000 | 0.0000 |
| Contents of the rumen | Spreading/Compost | 0.0000 | 0.0000 | 0.0000 |
| Ears | PAP C3 | 0.0011 | 0.0013 | 0.0001 |
| Esophagus | Pet food | 0.0009 | 0.0013 | 0.0001 |
| Fat | Fat and greaves C3 | 0.0011 | 0.0013 | 0.0001 |
| Fat around heart | Fat and greaves C3 | 0.0011 | 0.0013 | 0.0001 |
| Fat in the kidney | Fat and greaves C3 | 0.0011 | 0.0013 | 0.0001 |
| Feet (without hooves) | Gelatin C3 | 0.0009 | 0.0013 | 0.0000 |
| Floatation fat | Spreading/Compost | 0.0000 | 0.0000 | 0.0000 |
| Forehead | C1-C2 for disposal | 0.0000 | 0.0000 | 0.0000 |
| Forelock | PAP C3 | 0.0033 | 0.0013 | 0.0001 |
| Gallbladder | Pet food | 0.0009 | 0.0013 | 0.0001 |
| Head trimmings | Pet food | 0.0009 | 0.0013 | 0.0001 |
| Heart | Human food | 0.0009 | 0.0013 | 0.0003 |
| Heart trimmings | Pet food | 0.0009 | 0.0013 | 0.0001 |
| Hide | Skin tannery C3 | 0.0013 | 0.0013 | 0.0022 |
| Hooves | PAP C3 | 0.0033 | 0.0013 | 0.0001 |
| Horns | PAP C3 | 0.0033 | 0.0013 | 0.0001 |
| Kidney | Human food | 0.0009 | 0.0013 | 0.0005 |
| Large intestine | C1-C2 for disposal | 0.0000 | 0.0000 | 0.0000 |
| Liver | Human food | 0.0042 | 0.0013 | 0.0006 |
| Liver trimmings | Pet food | 0.0043 | 0.0013 | 0.0001 |
| Lower jaw | PAP C3 | 0.0008 | 0.0013 | 0.0001 |
| Lungs | Pet food | 0.0010 | 0.0013 | 0.0001 |
| Mask | Skin tannery C3 | 0.0013 | 0.0013 | 0.0022 |
| Mesenteric fat | C1-C2 for disposal | 0.0000 | 0.0000 | 0.0000 |
| Muscle | Human food | 0.0009 | 0.0013 | 0.0022 |
| Muzzle | Human food | 0.0013 | 0.0013 | 0.0013 |
| Omasum | Human food | 0.0081 | 0.0013 | 0.0010 |
| Omasum fat | Fat and greaves C3 | 0.0029 | 0.0013 | 0.0001 |
| Rumen and forestomach | Human food | 0.0081 | 0.0013 | 0.0010 |
| Rumen fat | Fat and greaves C3 | 0.0029 | 0.0013 | 0.0001 |
| Sanitary seizures | C1-C2 for disposal | 0.0000 | 0.0000 | 0.0000 |
| Screening and sifting wastes | C1-C2 for disposal | 0.0000 | 0.0000 | 0.0000 |
| Small intestine | PAP C3 | 0.0081 | 0.0013 | 0.0001 |
| Spinal cord | C1-C2 for disposal | 0.0000 | 0.0000 | 0.0000 |
| Spinal cord waste | C1-C2 for disposal | 0.0000 | 0.0000 | 0.0000 |
| Spine | C1-C2 for disposal | 0.0000 | 0.0000 | 0.0000 |
| Spleen | Pet food | 0.0009 | 0.0013 | 0.0001 |
| Stillborn | PAP C3 | 0.0084 | 0.0013 | 0.0000 |
| Tallow | Fat and greaves C3 | 0.0011 | 0.0013 | 0.0001 |
| Tongue | Human food | 0.0008 | 0.0013 | 0.0021 |
| Tonsil | C1-C2 for disposal | 0.0000 | 0.0000 | 0.0000 |
| Trachea | Pet food | 0.0013 | 0.0013 | 0.0001 |
| Udder | Pet food | 0.0002 | 0.0013 | 0.0001 |
| Upper throat | Pet food | 0.0009 | 0.0013 | 0.0001 |
| Water in the rumen | Spreading/Compost | 0.0000 | 0.0000 | 0.0000 |

Table 17: Allocation factors for Charolaise Young Bulls reared in Pasture

| COPRODUCT | Destination | Charolaise/young bull/pasture | | |
| --- | --- | --- | --- | --- |
|  |  | **Biophysical Allocation Factor** | **Mass Allocation Factor** | **Economic Allocation Factor** |
| Abomasum | Human food | 0.0083 | 0.0014 | 0.0011 |
| Abomasum fat | Fat and greaves C3 | 0.0032 | 0.0014 | 0.0001 |
| Aponeurosis | Human food | 0.0011 | 0.0014 | 0.0014 |
| Bile | PAP C3 | 0.0004 | 0.0014 | 0.0001 |
| Blood | PAP C3 | 0.0008 | 0.0014 | 0.0003 |
| Blood | Pet food | 0.0008 | 0.0014 | 0.0001 |
| Bones | Gelatin C3 | 0.0009 | 0.0014 | 0.0000 |
| Bones of head, brain, eyes and teeth | C1-C2 for disposal | 0.0000 | 0.0000 | 0.0000 |
| Cheek | Human food | 0.0010 | 0.0014 | 0.0031 |
| Cheek | Human food | 0.0010 | 0.0014 | 0.0031 |
| Cheek trimmings | Pet food | 0.0010 | 0.0014 | 0.0001 |
| Chops | Pet food | 0.0014 | 0.0014 | 0.0001 |
| Contents of intestines | Spreading/Compost | 0.0000 | 0.0000 | 0.0000 |
| Contents of the rumen | Spreading/Compost | 0.0000 | 0.0000 | 0.0000 |
| Ears | PAP C3 | 0.0012 | 0.0014 | 0.0001 |
| Esophagus | Pet food | 0.0010 | 0.0014 | 0.0001 |
| Fat | Fat and greaves C3 | 0.0015 | 0.0014 | 0.0001 |
| Fat around heart | Fat and greaves C3 | 0.0015 | 0.0014 | 0.0001 |
| Fat in the kidney | Fat and greaves C3 | 0.0015 | 0.0014 | 0.0001 |
| Feet (without hooves) | Gelatin C3 | 0.0010 | 0.0014 | 0.0000 |
| Floatation fat | Spreading/Compost | 0.0000 | 0.0000 | 0.0000 |
| Forehead | C1-C2 for disposal | 0.0000 | 0.0000 | 0.0000 |
| Forelock | PAP C3 | 0.0036 | 0.0014 | 0.0001 |
| Gallbladder | Pet food | 0.0010 | 0.0014 | 0.0001 |
| Head trimmings | Pet food | 0.0010 | 0.0014 | 0.0001 |
| Heart | Human food | 0.0010 | 0.0014 | 0.0003 |
| Heart trimmings | Pet food | 0.0010 | 0.0014 | 0.0001 |
| Hide | Skin tannery C3 | 0.0014 | 0.0014 | 0.0023 |
| Hooves | PAP C3 | 0.0036 | 0.0014 | 0.0001 |
| Horns | PAP C3 | 0.0036 | 0.0014 | 0.0001 |
| Kidney | Human food | 0.0010 | 0.0014 | 0.0006 |
| Large intestine | C1-C2 for disposal | 0.0000 | 0.0000 | 0.0000 |
| Liver | Human food | 0.0043 | 0.0014 | 0.0007 |
| Liver trimmings | Pet food | 0.0045 | 0.0014 | 0.0001 |
| Lower jaw | PAP C3 | 0.0009 | 0.0014 | 0.0001 |
| Lungs | Pet food | 0.0011 | 0.0014 | 0.0001 |
| Mask | Skin tannery C3 | 0.0014 | 0.0014 | 0.0023 |
| Mesenteric fat | C1-C2 for disposal | 0.0000 | 0.0000 | 0.0000 |
| Muscle | Human food | 0.0010 | 0.0014 | 0.0024 |
| Muzzle | Human food | 0.0014 | 0.0014 | 0.0014 |
| Omasum | Human food | 0.0083 | 0.0014 | 0.0011 |
| Omasum fat | Fat and greaves C3 | 0.0032 | 0.0014 | 0.0001 |
| Rumen and forestomach | Human food | 0.0083 | 0.0014 | 0.0011 |
| Rumen fat | Fat and greaves C3 | 0.0032 | 0.0014 | 0.0001 |
| Sanitary seizures | C1-C2 for disposal | 0.0000 | 0.0000 | 0.0000 |
| Screening and sifting wastes | C1-C2 for disposal | 0.0000 | 0.0000 | 0.0000 |
| Small intestine | PAP C3 | 0.0083 | 0.0014 | 0.0001 |
| Spinal cord | C1-C2 for disposal | 0.0000 | 0.0000 | 0.0000 |
| Spinal cord waste | C1-C2 for disposal | 0.0000 | 0.0000 | 0.0000 |
| Spine | C1-C2 for disposal | 0.0000 | 0.0000 | 0.0000 |
| Spleen | Pet food | 0.0010 | 0.0014 | 0.0001 |
| Stillborn | PAP C3 | 0.0087 | 0.0014 | 0.0000 |
| Tallow | Fat and greaves C3 | 0.0015 | 0.0014 | 0.0001 |
| Tongue | Human food | 0.0009 | 0.0014 | 0.0022 |
| Tonsil | C1-C2 for disposal | 0.0000 | 0.0000 | 0.0000 |
| Trachea | Pet food | 0.0014 | 0.0014 | 0.0001 |
| Udder | Pet food | 0.0002 | 0.0014 | 0.0001 |
| Upper throat | Pet food | 0.0010 | 0.0014 | 0.0001 |
| Water in the rumen | Spreading/Compost | 0.0000 | 0.0000 | 0.0000 |

Table 18: Allocation factors for Charolaise Heifers reared in Pasture

| COPRODUCT | Destination | Charolaise/heifer/pasture | | |
| --- | --- | --- | --- | --- |
|  |  | **Biophysical Allocation Factor** | **Mass Allocation Factor** | **Economic Allocation Factor** |
| Abomasum | Human food | 0.0085 | 0.0015 | 0.0011 |
| Abomasum fat | Fat and greaves C3 | 0.0034 | 0.0015 | 0.0001 |
| Aponeurosis | Human food | 0.0012 | 0.0015 | 0.0015 |
| Bile | PAP C3 | 0.0004 | 0.0015 | 0.0001 |
| Blood | PAP C3 | 0.0009 | 0.0015 | 0.0003 |
| Blood | Pet food | 0.0009 | 0.0015 | 0.0001 |
| Bones | Gelatin C3 | 0.0010 | 0.0015 | 0.0000 |
| Bones of head, brain, eyes and teeth | C1-C2 for disposal | 0.0000 | 0.0000 | 0.0000 |
| Cheek | Human food | 0.0011 | 0.0015 | 0.0034 |
| Cheek | Human food | 0.0011 | 0.0015 | 0.0034 |
| Cheek trimmings | Pet food | 0.0011 | 0.0015 | 0.0001 |
| Chops | Pet food | 0.0015 | 0.0015 | 0.0001 |
| Contents of intestines | Spreading/Compost | 0.0000 | 0.0000 | 0.0000 |
| Contents of the rumen | Spreading/Compost | 0.0000 | 0.0000 | 0.0000 |
| Ears | PAP C3 | 0.0013 | 0.0015 | 0.0001 |
| Esophagus | Pet food | 0.0010 | 0.0015 | 0.0001 |
| Fat | Fat and greaves C3 | 0.0016 | 0.0015 | 0.0001 |
| Fat around heart | Fat and greaves C3 | 0.0016 | 0.0015 | 0.0001 |
| Fat in the kidney | Fat and greaves C3 | 0.0016 | 0.0015 | 0.0001 |
| Feet (without hooves) | Gelatin C3 | 0.0010 | 0.0015 | 0.0000 |
| Floatation fat | Spreading/Compost | 0.0000 | 0.0000 | 0.0000 |
| Forehead | C1-C2 for disposal | 0.0000 | 0.0000 | 0.0000 |
| Forelock | PAP C3 | 0.0038 | 0.0015 | 0.0001 |
| Gallbladder | Pet food | 0.0010 | 0.0015 | 0.0001 |
| Head trimmings | Pet food | 0.0011 | 0.0015 | 0.0001 |
| Heart | Human food | 0.0010 | 0.0015 | 0.0003 |
| Heart trimmings | Pet food | 0.0011 | 0.0015 | 0.0001 |
| Hide | Skin tannery C3 | 0.0015 | 0.0015 | 0.0026 |
| Hooves | PAP C3 | 0.0038 | 0.0015 | 0.0001 |
| Horns | PAP C3 | 0.0038 | 0.0015 | 0.0001 |
| Kidney | Human food | 0.0010 | 0.0015 | 0.0006 |
| Large intestine | C1-C2 for disposal | 0.0000 | 0.0000 | 0.0000 |
| Liver | Human food | 0.0044 | 0.0015 | 0.0007 |
| Liver trimmings | Pet food | 0.0046 | 0.0015 | 0.0001 |
| Lower jaw | PAP C3 | 0.0010 | 0.0015 | 0.0001 |
| Lungs | Pet food | 0.0012 | 0.0015 | 0.0001 |
| Mask | Skin tannery C3 | 0.0015 | 0.0015 | 0.0026 |
| Mesenteric fat | C1-C2 for disposal | 0.0000 | 0.0000 | 0.0000 |
| Muscle | Human food | 0.0011 | 0.0015 | 0.0026 |
| Muzzle | Human food | 0.0015 | 0.0015 | 0.0015 |
| Omasum | Human food | 0.0085 | 0.0015 | 0.0011 |
| Omasum fat | Fat and greaves C3 | 0.0034 | 0.0015 | 0.0001 |
| Rumen and forestomach | Human food | 0.0085 | 0.0015 | 0.0011 |
| Rumen fat | Fat and greaves C3 | 0.0034 | 0.0015 | 0.0001 |
| Sanitary seizures | C1-C2 for disposal | 0.0000 | 0.0000 | 0.0000 |
| Screening and sifting wastes | C1-C2 for disposal | 0.0000 | 0.0000 | 0.0000 |
| Small intestine | PAP C3 | 0.0085 | 0.0015 | 0.0001 |
| Spinal cord | C1-C2 for disposal | 0.0000 | 0.0000 | 0.0000 |
| Spinal cord waste | C1-C2 for disposal | 0.0000 | 0.0000 | 0.0000 |
| Spine | C1-C2 for disposal | 0.0000 | 0.0000 | 0.0000 |
| Spleen | Pet food | 0.0011 | 0.0015 | 0.0001 |
| Stillborn | PAP C3 | 0.0089 | 0.0015 | 0.0000 |
| Tallow | Fat and greaves C3 | 0.0016 | 0.0015 | 0.0001 |
| Tongue | Human food | 0.0009 | 0.0015 | 0.0024 |
| Tonsil | C1-C2 for disposal | 0.0000 | 0.0000 | 0.0000 |
| Trachea | Pet food | 0.0015 | 0.0015 | 0.0001 |
| Udder | Pet food | 0.0002 | 0.0015 | 0.0001 |
| Upper throat | Pet food | 0.0010 | 0.0015 | 0.0001 |
| Water in the rumen | Spreading/Compost | 0.0000 | 0.0000 | 0.0000 |

Table 19: Allocation factors for Charolaise Cull Cows reared in Pasture

| COPRODUCT | Destination | Charolaise/Cull cow/pasture | | |
| --- | --- | --- | --- | --- |
|  |  | **Biophysical Allocation Factor** | **Mass Allocation Factor** | **Economic Allocation Factor** |
| Abomasum | Human food | 0.0078 | 0.0014 | 0.0010 |
| Abomasum fat | Fat and greaves C3 | 0.0030 | 0.0014 | 0.0001 |
| Aponeurosis | Human food | 0.0010 | 0.0014 | 0.0014 |
| Bile | PAP C3 | 0.0004 | 0.0014 | 0.0001 |
| Blood | PAP C3 | 0.0008 | 0.0014 | 0.0003 |
| Blood | Pet food | 0.0008 | 0.0014 | 0.0001 |
| Bones | Gelatin C3 | 0.0009 | 0.0014 | 0.0000 |
| Bones of head, brain, eyes and teeth | C1-C2 for disposal | 0.0000 | 0.0000 | 0.0000 |
| Cheek | Human food | 0.0009 | 0.0014 | 0.0030 |
| Cheek | Human food | 0.0009 | 0.0014 | 0.0030 |
| Cheek trimmings | Pet food | 0.0009 | 0.0014 | 0.0001 |
| Chops | Pet food | 0.0013 | 0.0014 | 0.0001 |
| Contents of intestines | Spreading/Compost | 0.0000 | 0.0000 | 0.0000 |
| Contents of the rumen | Spreading/Compost | 0.0000 | 0.0000 | 0.0000 |
| Ears | PAP C3 | 0.0011 | 0.0014 | 0.0001 |
| Esophagus | Pet food | 0.0009 | 0.0014 | 0.0001 |
| Fat | Fat and greaves C3 | 0.0013 | 0.0014 | 0.0001 |
| Fat around heart | Fat and greaves C3 | 0.0013 | 0.0014 | 0.0001 |
| Fat in the kidney | Fat and greaves C3 | 0.0013 | 0.0014 | 0.0001 |
| Feet (without hooves) | Gelatin C3 | 0.0009 | 0.0014 | 0.0000 |
| Floatation fat | Spreading/Compost | 0.0000 | 0.0000 | 0.0000 |
| Forehead | C1-C2 for disposal | 0.0000 | 0.0000 | 0.0000 |
| Forelock | PAP C3 | 0.0033 | 0.0014 | 0.0001 |
| Gallbladder | Pet food | 0.0009 | 0.0014 | 0.0001 |
| Head trimmings | Pet food | 0.0009 | 0.0014 | 0.0001 |
| Heart | Human food | 0.0009 | 0.0014 | 0.0003 |
| Heart trimmings | Pet food | 0.0009 | 0.0014 | 0.0001 |
| Hide | Skin tannery C3 | 0.0013 | 0.0014 | 0.0023 |
| Hooves | PAP C3 | 0.0033 | 0.0014 | 0.0001 |
| Horns | PAP C3 | 0.0033 | 0.0014 | 0.0001 |
| Kidney | Human food | 0.0009 | 0.0014 | 0.0006 |
| Large intestine | C1-C2 for disposal | 0.0000 | 0.0000 | 0.0000 |
| Liver | Human food | 0.0040 | 0.0014 | 0.0007 |
| Liver trimmings | Pet food | 0.0042 | 0.0014 | 0.0001 |
| Lower jaw | PAP C3 | 0.0008 | 0.0014 | 0.0001 |
| Lungs | Pet food | 0.0011 | 0.0014 | 0.0001 |
| Mask | Skin tannery C3 | 0.0013 | 0.0014 | 0.0023 |
| Mesenteric fat | C1-C2 for disposal | 0.0000 | 0.0000 | 0.0000 |
| Muscle | Human food | 0.0009 | 0.0014 | 0.0023 |
| Muzzle | Human food | 0.0013 | 0.0014 | 0.0014 |
| Omasum | Human food | 0.0078 | 0.0014 | 0.0010 |
| Omasum fat | Fat and greaves C3 | 0.0030 | 0.0014 | 0.0001 |
| Rumen and forestomach | Human food | 0.0078 | 0.0014 | 0.0010 |
| Rumen fat | Fat and greaves C3 | 0.0030 | 0.0014 | 0.0001 |
| Sanitary seizures | C1-C2 for disposal | 0.0000 | 0.0000 | 0.0000 |
| Screening and sifting wastes | C1-C2 for disposal | 0.0000 | 0.0000 | 0.0000 |
| Small intestine | PAP C3 | 0.0078 | 0.0014 | 0.0001 |
| Spinal cord | C1-C2 for disposal | 0.0000 | 0.0000 | 0.0000 |
| Spinal cord waste | C1-C2 for disposal | 0.0000 | 0.0000 | 0.0000 |
| Spine | C1-C2 for disposal | 0.0000 | 0.0000 | 0.0000 |
| Spleen | Pet food | 0.0009 | 0.0014 | 0.0001 |
| Stillborn | PAP C3 | 0.0082 | 0.0014 | 0.0000 |
| Tallow | Fat and greaves C3 | 0.0013 | 0.0014 | 0.0001 |
| Tongue | Human food | 0.0008 | 0.0014 | 0.0022 |
| Tonsil | C1-C2 for disposal | 0.0000 | 0.0000 | 0.0000 |
| Trachea | Pet food | 0.0013 | 0.0014 | 0.0001 |
| Udder | Pet food | 0.0002 | 0.0014 | 0.0001 |
| Upper throat | Pet food | 0.0009 | 0.0014 | 0.0001 |
| Water in the rumen | Spreading/Compost | 0.0000 | 0.0000 | 0.0000 |

Table 20: Allocation factors for Charolaise Beef reared in Pasture

| COPRODUCT | Destination | Charolaise/beef/pasture | | |
| --- | --- | --- | --- | --- |
|  |  | **Biophysical Allocation Factor** | **Mass Allocation Factor** | **Economic Allocation Factor** |
| Abomasum | Human food | 0.0078 | 0.0013 | 0.0010 |
| Abomasum fat | Fat and greaves C3 | 0.0029 | 0.0013 | 0.0001 |
| Aponeurosis | Human food | 0.0010 | 0.0013 | 0.0013 |
| Bile | PAP C3 | 0.0004 | 0.0013 | 0.0001 |
| Blood | PAP C3 | 0.0008 | 0.0013 | 0.0003 |
| Blood | Pet food | 0.0008 | 0.0013 | 0.0001 |
| Bones | Gelatin C3 | 0.0008 | 0.0013 | 0.0000 |
| Bones of head, brain, eyes and teeth | C1-C2 for disposal | 0.0000 | 0.0000 | 0.0000 |
| Cheek | Human food | 0.0009 | 0.0013 | 0.0029 |
| Cheek | Human food | 0.0009 | 0.0013 | 0.0029 |
| Cheek trimmings | Pet food | 0.0009 | 0.0013 | 0.0001 |
| Chops | Pet food | 0.0012 | 0.0013 | 0.0001 |
| Contents of intestines | Spreading/Compost | 0.0000 | 0.0000 | 0.0000 |
| Contents of the rumen | Spreading/Compost | 0.0000 | 0.0000 | 0.0000 |
| Ears | PAP C3 | 0.0011 | 0.0013 | 0.0001 |
| Esophagus | Pet food | 0.0009 | 0.0013 | 0.0001 |
| Fat | Fat and greaves C3 | 0.0013 | 0.0013 | 0.0001 |
| Fat around heart | Fat and greaves C3 | 0.0013 | 0.0013 | 0.0001 |
| Fat in the kidney | Fat and greaves C3 | 0.0013 | 0.0013 | 0.0001 |
| Feet (without hooves) | Gelatin C3 | 0.0009 | 0.0013 | 0.0000 |
| Floatation fat | Spreading/Compost | 0.0000 | 0.0000 | 0.0000 |
| Forehead | C1-C2 for disposal | 0.0000 | 0.0000 | 0.0000 |
| Forelock | PAP C3 | 0.0032 | 0.0013 | 0.0001 |
| Gallbladder | Pet food | 0.0009 | 0.0013 | 0.0001 |
| Head trimmings | Pet food | 0.0009 | 0.0013 | 0.0001 |
| Heart | Human food | 0.0009 | 0.0013 | 0.0003 |
| Heart trimmings | Pet food | 0.0009 | 0.0013 | 0.0001 |
| Hide | Skin tannery C3 | 0.0012 | 0.0013 | 0.0022 |
| Hooves | PAP C3 | 0.0032 | 0.0013 | 0.0001 |
| Horns | PAP C3 | 0.0032 | 0.0013 | 0.0001 |
| Kidney | Human food | 0.0009 | 0.0013 | 0.0005 |
| Large intestine | C1-C2 for disposal | 0.0000 | 0.0000 | 0.0000 |
| Liver | Human food | 0.0040 | 0.0013 | 0.0006 |
| Liver trimmings | Pet food | 0.0042 | 0.0013 | 0.0001 |
| Lower jaw | PAP C3 | 0.0008 | 0.0013 | 0.0001 |
| Lungs | Pet food | 0.0010 | 0.0013 | 0.0001 |
| Mask | Skin tannery C3 | 0.0012 | 0.0013 | 0.0022 |
| Mesenteric fat | C1-C2 for disposal | 0.0000 | 0.0000 | 0.0000 |
| Muscle | Human food | 0.0009 | 0.0013 | 0.0022 |
| Muzzle | Human food | 0.0012 | 0.0013 | 0.0013 |
| Omasum | Human food | 0.0078 | 0.0013 | 0.0010 |
| Omasum fat | Fat and greaves C3 | 0.0029 | 0.0013 | 0.0001 |
| Rumen and forestomach | Human food | 0.0078 | 0.0013 | 0.0010 |
| Rumen fat | Fat and greaves C3 | 0.0029 | 0.0013 | 0.0001 |
| Sanitary seizures | C1-C2 for disposal | 0.0000 | 0.0000 | 0.0000 |
| Screening and sifting wastes | C1-C2 for disposal | 0.0000 | 0.0000 | 0.0000 |
| Small intestine | PAP C3 | 0.0078 | 0.0013 | 0.0001 |
| Spinal cord | C1-C2 for disposal | 0.0000 | 0.0000 | 0.0000 |
| Spinal cord waste | C1-C2 for disposal | 0.0000 | 0.0000 | 0.0000 |
| Spine | C1-C2 for disposal | 0.0000 | 0.0000 | 0.0000 |
| Spleen | Pet food | 0.0009 | 0.0013 | 0.0001 |
| Stillborn | PAP C3 | 0.0081 | 0.0013 | 0.0000 |
| Tallow | Fat and greaves C3 | 0.0013 | 0.0013 | 0.0001 |
| Tongue | Human food | 0.0008 | 0.0013 | 0.0021 |
| Tonsil | C1-C2 for disposal | 0.0000 | 0.0000 | 0.0000 |
| Trachea | Pet food | 0.0012 | 0.0013 | 0.0001 |
| Udder | Pet food | 0.0002 | 0.0013 | 0.0001 |
| Upper throat | Pet food | 0.0009 | 0.0013 | 0.0001 |
| Water in the rumen | Spreading/Compost | 0.0000 | 0.0000 | 0.0000 |

Table 21: Allocation factors for Charolaise Young Bulls reared in Stall

| COPRODUCT | Destination | Charolaise/young bull/stall | | |
| --- | --- | --- | --- | --- |
|  |  | **Biophysical Allocation Factor** | **Mass Allocation Factor** | **Economic Allocation Factor** |
| Abomasum | Human food | 0.0079 | 0.0014 | 0.0011 |
| Abomasum fat | Fat and greaves C3 | 0.0033 | 0.0014 | 0.0001 |
| Aponeurosis | Human food | 0.0011 | 0.0014 | 0.0014 |
| Bile | PAP C3 | 0.0004 | 0.0014 | 0.0001 |
| Blood | PAP C3 | 0.0008 | 0.0014 | 0.0003 |
| Blood | Pet food | 0.0008 | 0.0014 | 0.0001 |
| Bones | Gelatin C3 | 0.0009 | 0.0014 | 0.0000 |
| Bones of head, brain, eyes and teeth | C1-C2 for disposal | 0.0000 | 0.0000 | 0.0000 |
| Cheek | Human food | 0.0010 | 0.0014 | 0.0031 |
| Cheek | Human food | 0.0010 | 0.0014 | 0.0031 |
| Cheek trimmings | Pet food | 0.0010 | 0.0014 | 0.0001 |
| Chops | Pet food | 0.0014 | 0.0014 | 0.0001 |
| Contents of intestines | Spreading/Compost | 0.0000 | 0.0000 | 0.0000 |
| Contents of the rumen | Spreading/Compost | 0.0000 | 0.0000 | 0.0000 |
| Ears | PAP C3 | 0.0012 | 0.0014 | 0.0001 |
| Esophagus | Pet food | 0.0010 | 0.0014 | 0.0001 |
| Fat | Fat and greaves C3 | 0.0016 | 0.0014 | 0.0001 |
| Fat around heart | Fat and greaves C3 | 0.0016 | 0.0014 | 0.0001 |
| Fat in the kidney | Fat and greaves C3 | 0.0016 | 0.0014 | 0.0001 |
| Feet (without hooves) | Gelatin C3 | 0.0010 | 0.0014 | 0.0000 |
| Floatation fat | Spreading/Compost | 0.0000 | 0.0000 | 0.0000 |
| Forehead | C1-C2 for disposal | 0.0000 | 0.0000 | 0.0000 |
| Forelock | PAP C3 | 0.0035 | 0.0014 | 0.0001 |
| Gallbladder | Pet food | 0.0010 | 0.0014 | 0.0001 |
| Head trimmings | Pet food | 0.0010 | 0.0014 | 0.0001 |
| Heart | Human food | 0.0009 | 0.0014 | 0.0003 |
| Heart trimmings | Pet food | 0.0010 | 0.0014 | 0.0001 |
| Hide | Skin tannery C3 | 0.0014 | 0.0014 | 0.0023 |
| Hooves | PAP C3 | 0.0035 | 0.0014 | 0.0001 |
| Horns | PAP C3 | 0.0035 | 0.0014 | 0.0001 |
| Kidney | Human food | 0.0010 | 0.0014 | 0.0006 |
| Large intestine | C1-C2 for disposal | 0.0000 | 0.0000 | 0.0000 |
| Liver | Human food | 0.0041 | 0.0014 | 0.0007 |
| Liver trimmings | Pet food | 0.0043 | 0.0014 | 0.0001 |
| Lower jaw | PAP C3 | 0.0009 | 0.0014 | 0.0001 |
| Lungs | Pet food | 0.0011 | 0.0014 | 0.0001 |
| Mask | Skin tannery C3 | 0.0014 | 0.0014 | 0.0023 |
| Mesenteric fat | C1-C2 for disposal | 0.0000 | 0.0000 | 0.0000 |
| Muscle | Human food | 0.0010 | 0.0014 | 0.0024 |
| Muzzle | Human food | 0.0014 | 0.0014 | 0.0014 |
| Omasum | Human food | 0.0079 | 0.0014 | 0.0011 |
| Omasum fat | Fat and greaves C3 | 0.0033 | 0.0014 | 0.0001 |
| Rumen and forestomach | Human food | 0.0079 | 0.0014 | 0.0011 |
| Rumen fat | Fat and greaves C3 | 0.0033 | 0.0014 | 0.0001 |
| Sanitary seizures | C1-C2 for disposal | 0.0000 | 0.0000 | 0.0000 |
| Screening and sifting wastes | C1-C2 for disposal | 0.0000 | 0.0000 | 0.0000 |
| Small intestine | PAP C3 | 0.0079 | 0.0014 | 0.0001 |
| Spinal cord | C1-C2 for disposal | 0.0000 | 0.0000 | 0.0000 |
| Spinal cord waste | C1-C2 for disposal | 0.0000 | 0.0000 | 0.0000 |
| Spine | C1-C2 for disposal | 0.0000 | 0.0000 | 0.0000 |
| Spleen | Pet food | 0.0010 | 0.0014 | 0.0001 |
| Stillborn | PAP C3 | 0.0082 | 0.0014 | 0.0000 |
| Tallow | Fat and greaves C3 | 0.0016 | 0.0014 | 0.0001 |
| Tongue | Human food | 0.0009 | 0.0014 | 0.0022 |
| Tonsil | C1-C2 for disposal | 0.0000 | 0.0000 | 0.0000 |
| Trachea | Pet food | 0.0014 | 0.0014 | 0.0001 |
| Udder | Pet food | 0.0002 | 0.0014 | 0.0001 |
| Upper throat | Pet food | 0.0010 | 0.0014 | 0.0001 |
| Water in the rumen | Spreading/Compost | 0.0000 | 0.0000 | 0.0000 |

Table 22: Allocation factors for Charolaise Heifers reared in Stall

| COPRODUCT | Destination | Charolaise/heifer/stall | | |
| --- | --- | --- | --- | --- |
|  |  | **Biophysical Allocation Factor** | **Mass Allocation Factor** | **Economic Allocation Factor** |
| Abomasum | Human food | 0.0081 | 0.0015 | 0.0011 |
| Abomasum fat | Fat and greaves C3 | 0.0034 | 0.0015 | 0.0001 |
| Aponeurosis | Human food | 0.0011 | 0.0015 | 0.0015 |
| Bile | PAP C3 | 0.0004 | 0.0015 | 0.0001 |
| Blood | PAP C3 | 0.0009 | 0.0015 | 0.0003 |
| Blood | Pet food | 0.0009 | 0.0015 | 0.0001 |
| Bones | Gelatin C3 | 0.0010 | 0.0015 | 0.0000 |
| Bones of head, brain, eyes and teeth | C1-C2 for disposal | 0.0000 | 0.0000 | 0.0000 |
| Cheek | Human food | 0.0010 | 0.0015 | 0.0034 |
| Cheek | Human food | 0.0010 | 0.0015 | 0.0034 |
| Cheek trimmings | Pet food | 0.0010 | 0.0015 | 0.0001 |
| Chops | Pet food | 0.0014 | 0.0015 | 0.0001 |
| Contents of intestines | Spreading/Compost | 0.0000 | 0.0000 | 0.0000 |
| Contents of the rumen | Spreading/Compost | 0.0000 | 0.0000 | 0.0000 |
| Ears | PAP C3 | 0.0013 | 0.0015 | 0.0001 |
| Esophagus | Pet food | 0.0010 | 0.0015 | 0.0001 |
| Fat | Fat and greaves C3 | 0.0017 | 0.0015 | 0.0001 |
| Fat around heart | Fat and greaves C3 | 0.0017 | 0.0015 | 0.0001 |
| Fat in the kidney | Fat and greaves C3 | 0.0017 | 0.0015 | 0.0001 |
| Feet (without hooves) | Gelatin C3 | 0.0010 | 0.0015 | 0.0000 |
| Floatation fat | Spreading/Compost | 0.0000 | 0.0000 | 0.0000 |
| Forehead | C1-C2 for disposal | 0.0000 | 0.0000 | 0.0000 |
| Forelock | PAP C3 | 0.0037 | 0.0015 | 0.0001 |
| Gallbladder | Pet food | 0.0010 | 0.0015 | 0.0001 |
| Head trimmings | Pet food | 0.0010 | 0.0015 | 0.0001 |
| Heart | Human food | 0.0010 | 0.0015 | 0.0003 |
| Heart trimmings | Pet food | 0.0010 | 0.0015 | 0.0001 |
| Hide | Skin tannery C3 | 0.0014 | 0.0015 | 0.0026 |
| Hooves | PAP C3 | 0.0037 | 0.0015 | 0.0001 |
| Horns | PAP C3 | 0.0037 | 0.0015 | 0.0001 |
| Kidney | Human food | 0.0010 | 0.0015 | 0.0006 |
| Large intestine | C1-C2 for disposal | 0.0000 | 0.0000 | 0.0000 |
| Liver | Human food | 0.0042 | 0.0015 | 0.0007 |
| Liver trimmings | Pet food | 0.0044 | 0.0015 | 0.0001 |
| Lower jaw | PAP C3 | 0.0010 | 0.0015 | 0.0001 |
| Lungs | Pet food | 0.0012 | 0.0015 | 0.0001 |
| Mask | Skin tannery C3 | 0.0014 | 0.0015 | 0.0026 |
| Mesenteric fat | C1-C2 for disposal | 0.0000 | 0.0000 | 0.0000 |
| Muscle | Human food | 0.0011 | 0.0015 | 0.0026 |
| Muzzle | Human food | 0.0014 | 0.0015 | 0.0015 |
| Omasum | Human food | 0.0081 | 0.0015 | 0.0011 |
| Omasum fat | Fat and greaves C3 | 0.0034 | 0.0015 | 0.0001 |
| Rumen and forestomach | Human food | 0.0081 | 0.0015 | 0.0011 |
| Rumen fat | Fat and greaves C3 | 0.0034 | 0.0015 | 0.0001 |
| Sanitary seizures | C1-C2 for disposal | 0.0000 | 0.0000 | 0.0000 |
| Screening and sifting wastes | C1-C2 for disposal | 0.0000 | 0.0000 | 0.0000 |
| Small intestine | PAP C3 | 0.0081 | 0.0015 | 0.0001 |
| Spinal cord | C1-C2 for disposal | 0.0000 | 0.0000 | 0.0000 |
| Spinal cord waste | C1-C2 for disposal | 0.0000 | 0.0000 | 0.0000 |
| Spine | C1-C2 for disposal | 0.0000 | 0.0000 | 0.0000 |
| Spleen | Pet food | 0.0011 | 0.0015 | 0.0001 |
| Stillborn | PAP C3 | 0.0085 | 0.0015 | 0.0000 |
| Tallow | Fat and greaves C3 | 0.0017 | 0.0015 | 0.0001 |
| Tongue | Human food | 0.0009 | 0.0015 | 0.0024 |
| Tonsil | C1-C2 for disposal | 0.0000 | 0.0000 | 0.0000 |
| Trachea | Pet food | 0.0015 | 0.0015 | 0.0001 |
| Udder | Pet food | 0.0002 | 0.0015 | 0.0001 |
| Upper throat | Pet food | 0.0010 | 0.0015 | 0.0001 |
| Water in the rumen | Spreading/Compost | 0.0000 | 0.0000 | 0.0000 |

Table 23: Allocation factors for Charolaise Cull Cows reared in Stall

| COPRODUCT | Destination | Charolaise/Cull cow/stall | | |
| --- | --- | --- | --- | --- |
|  |  | **Biophysical Allocation Factor** | **Mass Allocation Factor** | **Economic Allocation Factor** |
| Abomasum | Human food | 0.0075 | 0.0014 | 0.0010 |
| Abomasum fat | Fat and greaves C3 | 0.0030 | 0.0014 | 0.0001 |
| Aponeurosis | Human food | 0.0010 | 0.0014 | 0.0014 |
| Bile | PAP C3 | 0.0004 | 0.0014 | 0.0001 |
| Blood | PAP C3 | 0.0008 | 0.0014 | 0.0003 |
| Blood | Pet food | 0.0008 | 0.0014 | 0.0001 |
| Bones | Gelatin C3 | 0.0009 | 0.0014 | 0.0000 |
| Bones of head, brain, eyes and teeth | C1-C2 for disposal | 0.0000 | 0.0000 | 0.0000 |
| Cheek | Human food | 0.0009 | 0.0014 | 0.0030 |
| Cheek | Human food | 0.0009 | 0.0014 | 0.0030 |
| Cheek trimmings | Pet food | 0.0009 | 0.0014 | 0.0001 |
| Chops | Pet food | 0.0013 | 0.0014 | 0.0001 |
| Contents of intestines | Spreading/Compost | 0.0000 | 0.0000 | 0.0000 |
| Contents of the rumen | Spreading/Compost | 0.0000 | 0.0000 | 0.0000 |
| Ears | PAP C3 | 0.0011 | 0.0014 | 0.0001 |
| Esophagus | Pet food | 0.0009 | 0.0014 | 0.0001 |
| Fat | Fat and greaves C3 | 0.0014 | 0.0014 | 0.0001 |
| Fat around heart | Fat and greaves C3 | 0.0014 | 0.0014 | 0.0001 |
| Fat in the kidney | Fat and greaves C3 | 0.0014 | 0.0014 | 0.0001 |
| Feet (without hooves) | Gelatin C3 | 0.0009 | 0.0014 | 0.0000 |
| Floatation fat | Spreading/Compost | 0.0000 | 0.0000 | 0.0000 |
| Forehead | C1-C2 for disposal | 0.0000 | 0.0000 | 0.0000 |
| Forelock | PAP C3 | 0.0033 | 0.0014 | 0.0001 |
| Gallbladder | Pet food | 0.0009 | 0.0014 | 0.0001 |
| Head trimmings | Pet food | 0.0009 | 0.0014 | 0.0001 |
| Heart | Human food | 0.0009 | 0.0014 | 0.0003 |
| Heart trimmings | Pet food | 0.0009 | 0.0014 | 0.0001 |
| Hide | Skin tannery C3 | 0.0013 | 0.0014 | 0.0023 |
| Hooves | PAP C3 | 0.0033 | 0.0014 | 0.0001 |
| Horns | PAP C3 | 0.0033 | 0.0014 | 0.0001 |
| Kidney | Human food | 0.0009 | 0.0014 | 0.0006 |
| Large intestine | C1-C2 for disposal | 0.0000 | 0.0000 | 0.0000 |
| Liver | Human food | 0.0039 | 0.0014 | 0.0007 |
| Liver trimmings | Pet food | 0.0040 | 0.0014 | 0.0001 |
| Lower jaw | PAP C3 | 0.0009 | 0.0014 | 0.0001 |
| Lungs | Pet food | 0.0010 | 0.0014 | 0.0001 |
| Mask | Skin tannery C3 | 0.0013 | 0.0014 | 0.0023 |
| Mesenteric fat | C1-C2 for disposal | 0.0000 | 0.0000 | 0.0000 |
| Muscle | Human food | 0.0009 | 0.0014 | 0.0023 |
| Muzzle | Human food | 0.0013 | 0.0014 | 0.0014 |
| Omasum | Human food | 0.0075 | 0.0014 | 0.0010 |
| Omasum fat | Fat and greaves C3 | 0.0030 | 0.0014 | 0.0001 |
| Rumen and forestomach | Human food | 0.0075 | 0.0014 | 0.0010 |
| Rumen fat | Fat and greaves C3 | 0.0030 | 0.0014 | 0.0001 |
| Sanitary seizures | C1-C2 for disposal | 0.0000 | 0.0000 | 0.0000 |
| Screening and sifting wastes | C1-C2 for disposal | 0.0000 | 0.0000 | 0.0000 |
| Small intestine | PAP C3 | 0.0075 | 0.0014 | 0.0001 |
| Spinal cord | C1-C2 for disposal | 0.0000 | 0.0000 | 0.0000 |
| Spinal cord waste | C1-C2 for disposal | 0.0000 | 0.0000 | 0.0000 |
| Spine | C1-C2 for disposal | 0.0000 | 0.0000 | 0.0000 |
| Spleen | Pet food | 0.0009 | 0.0014 | 0.0001 |
| Stillborn | PAP C3 | 0.0078 | 0.0014 | 0.0000 |
| Tallow | Fat and greaves C3 | 0.0014 | 0.0014 | 0.0001 |
| Tongue | Human food | 0.0008 | 0.0014 | 0.0022 |
| Tonsil | C1-C2 for disposal | 0.0000 | 0.0000 | 0.0000 |
| Trachea | Pet food | 0.0013 | 0.0014 | 0.0001 |
| Udder | Pet food | 0.0002 | 0.0014 | 0.0001 |
| Upper throat | Pet food | 0.0009 | 0.0014 | 0.0001 |
| Water in the rumen | Spreading/Compost | 0.0000 | 0.0000 | 0.0000 |

Table 24: Allocation factors for Charolaise Beef reared in Stall

| COPRODUCT | Destination | Charolaise/beef/stall | | |
| --- | --- | --- | --- | --- |
|  |  | **Biophysical Allocation Factor** | **Mass Allocation Factor** | **Economic Allocation Factor** |
| Abomasum | Human food | 0.0074 | 0.0013 | 0.0010 |
| Abomasum fat | Fat and greaves C3 | 0.0029 | 0.0013 | 0.0001 |
| Aponeurosis | Human food | 0.0010 | 0.0013 | 0.0013 |
| Bile | PAP C3 | 0.0004 | 0.0013 | 0.0001 |
| Blood | PAP C3 | 0.0008 | 0.0013 | 0.0003 |
| Blood | Pet food | 0.0008 | 0.0013 | 0.0001 |
| Bones | Gelatin C3 | 0.0008 | 0.0013 | 0.0000 |
| Bones of head, brain, eyes and teeth | C1-C2 for disposal | 0.0000 | 0.0000 | 0.0000 |
| Cheek | Human food | 0.0009 | 0.0013 | 0.0029 |
| Cheek | Human food | 0.0009 | 0.0013 | 0.0029 |
| Cheek trimmings | Pet food | 0.0009 | 0.0013 | 0.0001 |
| Chops | Pet food | 0.0012 | 0.0013 | 0.0001 |
| Contents of intestines | Spreading/Compost | 0.0000 | 0.0000 | 0.0000 |
| Contents of the rumen | Spreading/Compost | 0.0000 | 0.0000 | 0.0000 |
| Ears | PAP C3 | 0.0011 | 0.0013 | 0.0001 |
| Esophagus | Pet food | 0.0009 | 0.0013 | 0.0001 |
| Fat | Fat and greaves C3 | 0.0014 | 0.0013 | 0.0001 |
| Fat around heart | Fat and greaves C3 | 0.0014 | 0.0013 | 0.0001 |
| Fat in the kidney | Fat and greaves C3 | 0.0014 | 0.0013 | 0.0001 |
| Feet (without hooves) | Gelatin C3 | 0.0009 | 0.0013 | 0.0000 |
| Floatation fat | Spreading/Compost | 0.0000 | 0.0000 | 0.0000 |
| Forehead | C1-C2 for disposal | 0.0000 | 0.0000 | 0.0000 |
| Forelock | PAP C3 | 0.0032 | 0.0013 | 0.0001 |
| Gallbladder | Pet food | 0.0009 | 0.0013 | 0.0001 |
| Head trimmings | Pet food | 0.0009 | 0.0013 | 0.0001 |
| Heart | Human food | 0.0008 | 0.0013 | 0.0003 |
| Heart trimmings | Pet food | 0.0009 | 0.0013 | 0.0001 |
| Hide | Skin tannery C3 | 0.0012 | 0.0013 | 0.0022 |
| Hooves | PAP C3 | 0.0032 | 0.0013 | 0.0001 |
| Horns | PAP C3 | 0.0032 | 0.0013 | 0.0001 |
| Kidney | Human food | 0.0009 | 0.0013 | 0.0005 |
| Large intestine | C1-C2 for disposal | 0.0000 | 0.0000 | 0.0000 |
| Liver | Human food | 0.0038 | 0.0013 | 0.0006 |
| Liver trimmings | Pet food | 0.0040 | 0.0013 | 0.0001 |
| Lower jaw | PAP C3 | 0.0008 | 0.0013 | 0.0001 |
| Lungs | Pet food | 0.0010 | 0.0013 | 0.0001 |
| Mask | Skin tannery C3 | 0.0012 | 0.0013 | 0.0022 |
| Mesenteric fat | C1-C2 for disposal | 0.0000 | 0.0000 | 0.0000 |
| Muscle | Human food | 0.0009 | 0.0013 | 0.0022 |
| Muzzle | Human food | 0.0012 | 0.0013 | 0.0013 |
| Omasum | Human food | 0.0074 | 0.0013 | 0.0010 |
| Omasum fat | Fat and greaves C3 | 0.0029 | 0.0013 | 0.0001 |
| Rumen and forestomach | Human food | 0.0074 | 0.0013 | 0.0010 |
| Rumen fat | Fat and greaves C3 | 0.0029 | 0.0013 | 0.0001 |
| Sanitary seizures | C1-C2 for disposal | 0.0000 | 0.0000 | 0.0000 |
| Screening and sifting wastes | C1-C2 for disposal | 0.0000 | 0.0000 | 0.0000 |
| Small intestine | PAP C3 | 0.0074 | 0.0013 | 0.0001 |
| Spinal cord | C1-C2 for disposal | 0.0000 | 0.0000 | 0.0000 |
| Spinal cord waste | C1-C2 for disposal | 0.0000 | 0.0000 | 0.0000 |
| Spine | C1-C2 for disposal | 0.0000 | 0.0000 | 0.0000 |
| Spleen | Pet food | 0.0009 | 0.0013 | 0.0001 |
| Stillborn | PAP C3 | 0.0077 | 0.0013 | 0.0000 |
| Tallow | Fat and greaves C3 | 0.0014 | 0.0013 | 0.0001 |
| Tongue | Human food | 0.0008 | 0.0013 | 0.0021 |
| Tonsil | C1-C2 for disposal | 0.0000 | 0.0000 | 0.0000 |
| Trachea | Pet food | 0.0012 | 0.0013 | 0.0001 |
| Udder | Pet food | 0.0002 | 0.0013 | 0.0001 |
| Upper throat | Pet food | 0.0009 | 0.0013 | 0.0001 |
| Water in the rumen | Spreading/Compost | 0.0000 | 0.0000 | 0.0000 |

Table 25: Allocation factors for Primholstein Young Bulls reared in Grazing Large Area

| COPRODUCT | Destination | Primholstein/young bull/grazing large area | | |
| --- | --- | --- | --- | --- |
|  |  | **Biophysical Allocation Factor** | **Mass Allocation Factor** | **Economic Allocation Factor** |
| Abomasum | Human food | 0.0090 | 0.0016 | 0.0012 |
| Abomasum fat | Fat and greaves C3 | 0.0034 | 0.0016 | 0.0001 |
| Aponeurosis | Human food | 0.0012 | 0.0016 | 0.0016 |
| Bile | PAP C3 | 0.0004 | 0.0016 | 0.0001 |
| Blood | PAP C3 | 0.0009 | 0.0016 | 0.0004 |
| Blood | Pet food | 0.0009 | 0.0016 | 0.0001 |
| Bones | Gelatin C3 | 0.0010 | 0.0016 | 0.0000 |
| Bones of head, brain, eyes and teeth | C1-C2 for disposal | 0.0000 | 0.0000 | 0.0000 |
| Cheek | Human food | 0.0011 | 0.0016 | 0.0036 |
| Cheek | Human food | 0.0011 | 0.0016 | 0.0036 |
| Cheek trimmings | Pet food | 0.0011 | 0.0016 | 0.0001 |
| Chops | Pet food | 0.0015 | 0.0016 | 0.0001 |
| Contents of intestines | Spreading/Compost | 0.0000 | 0.0000 | 0.0000 |
| Contents of the rumen | Spreading/Compost | 0.0000 | 0.0000 | 0.0000 |
| Ears | PAP C3 | 0.0013 | 0.0016 | 0.0001 |
| Esophagus | Pet food | 0.0010 | 0.0016 | 0.0001 |
| Fat | Fat and greaves C3 | 0.0014 | 0.0016 | 0.0001 |
| Fat around heart | Fat and greaves C3 | 0.0014 | 0.0016 | 0.0001 |
| Fat in the kidney | Fat and greaves C3 | 0.0014 | 0.0016 | 0.0001 |
| Feet (without hooves) | Gelatin C3 | 0.0011 | 0.0016 | 0.0000 |
| Floatation fat | Spreading/Compost | 0.0000 | 0.0000 | 0.0000 |
| Forehead | C1-C2 for disposal | 0.0000 | 0.0000 | 0.0000 |
| Forelock | PAP C3 | 0.0039 | 0.0016 | 0.0001 |
| Gallbladder | Pet food | 0.0011 | 0.0016 | 0.0001 |
| Head trimmings | Pet food | 0.0011 | 0.0016 | 0.0001 |
| Heart | Human food | 0.0010 | 0.0016 | 0.0003 |
| Heart trimmings | Pet food | 0.0011 | 0.0016 | 0.0001 |
| Hide | Skin tannery C3 | 0.0015 | 0.0016 | 0.0027 |
| Hooves | PAP C3 | 0.0039 | 0.0016 | 0.0001 |
| Horns | PAP C3 | 0.0039 | 0.0016 | 0.0001 |
| Kidney | Human food | 0.0011 | 0.0016 | 0.0007 |
| Large intestine | C1-C2 for disposal | 0.0000 | 0.0000 | 0.0000 |
| Liver | Human food | 0.0047 | 0.0016 | 0.0008 |
| Liver trimmings | Pet food | 0.0049 | 0.0016 | 0.0001 |
| Lower jaw | PAP C3 | 0.0010 | 0.0016 | 0.0001 |
| Lungs | Pet food | 0.0012 | 0.0016 | 0.0001 |
| Mask | Skin tannery C3 | 0.0015 | 0.0016 | 0.0027 |
| Mesenteric fat | C1-C2 for disposal | 0.0000 | 0.0000 | 0.0000 |
| Muscle | Human food | 0.0011 | 0.0016 | 0.0027 |
| Muzzle | Human food | 0.0015 | 0.0016 | 0.0016 |
| Omasum | Human food | 0.0090 | 0.0016 | 0.0012 |
| Omasum fat | Fat and greaves C3 | 0.0034 | 0.0016 | 0.0001 |
| Rumen and forestomach | Human food | 0.0090 | 0.0016 | 0.0012 |
| Rumen fat | Fat and greaves C3 | 0.0034 | 0.0016 | 0.0001 |
| Sanitary seizures | C1-C2 for disposal | 0.0000 | 0.0000 | 0.0000 |
| Screening and sifting wastes | C1-C2 for disposal | 0.0000 | 0.0000 | 0.0000 |
| Small intestine | PAP C3 | 0.0090 | 0.0016 | 0.0001 |
| Spinal cord | C1-C2 for disposal | 0.0000 | 0.0000 | 0.0000 |
| Spinal cord waste | C1-C2 for disposal | 0.0000 | 0.0000 | 0.0000 |
| Spine | C1-C2 for disposal | 0.0000 | 0.0000 | 0.0000 |
| Spleen | Pet food | 0.0011 | 0.0016 | 0.0001 |
| Stillborn | PAP C3 | 0.0094 | 0.0016 | 0.0000 |
| Tallow | Fat and greaves C3 | 0.0014 | 0.0016 | 0.0001 |
| Tongue | Human food | 0.0009 | 0.0016 | 0.0026 |
| Tonsil | C1-C2 for disposal | 0.0000 | 0.0000 | 0.0000 |
| Trachea | Pet food | 0.0015 | 0.0016 | 0.0001 |
| Udder | Pet food | 0.0002 | 0.0016 | 0.0001 |
| Upper throat | Pet food | 0.0011 | 0.0016 | 0.0001 |
| Water in the rumen | Spreading/Compost | 0.0000 | 0.0000 | 0.0000 |

Table 26: Allocation factors for Primholstein Heifers reared in Grazing Large Area

| COPRODUCT | Destination | Primholstein/heifer/grazing large area | | |
| --- | --- | --- | --- | --- |
|  |  | **Biophysical Allocation Factor** | **Mass Allocation Factor** | **Economic Allocation Factor** |
| Abomasum | Human food | 0.0101 | 0.0019 | 0.0015 |
| Abomasum fat | Fat and greaves C3 | 0.0038 | 0.0019 | 0.0002 |
| Aponeurosis | Human food | 0.0014 | 0.0019 | 0.0020 |
| Bile | PAP C3 | 0.0005 | 0.0019 | 0.0002 |
| Blood | PAP C3 | 0.0011 | 0.0019 | 0.0004 |
| Blood | Pet food | 0.0011 | 0.0019 | 0.0001 |
| Bones | Gelatin C3 | 0.0012 | 0.0019 | 0.0000 |
| Bones of head, brain, eyes and teeth | C1-C2 for disposal | 0.0000 | 0.0000 | 0.0000 |
| Cheek | Human food | 0.0013 | 0.0019 | 0.0043 |
| Cheek | Human food | 0.0013 | 0.0019 | 0.0043 |
| Cheek trimmings | Pet food | 0.0013 | 0.0019 | 0.0001 |
| Chops | Pet food | 0.0018 | 0.0019 | 0.0001 |
| Contents of intestines | Spreading/Compost | 0.0000 | 0.0000 | 0.0000 |
| Contents of the rumen | Spreading/Compost | 0.0000 | 0.0000 | 0.0000 |
| Ears | PAP C3 | 0.0016 | 0.0019 | 0.0002 |
| Esophagus | Pet food | 0.0012 | 0.0019 | 0.0001 |
| Fat | Fat and greaves C3 | 0.0017 | 0.0019 | 0.0002 |
| Fat around heart | Fat and greaves C3 | 0.0017 | 0.0019 | 0.0002 |
| Fat in the kidney | Fat and greaves C3 | 0.0017 | 0.0019 | 0.0002 |
| Feet (without hooves) | Gelatin C3 | 0.0013 | 0.0019 | 0.0000 |
| Floatation fat | Spreading/Compost | 0.0000 | 0.0000 | 0.0000 |
| Forehead | C1-C2 for disposal | 0.0000 | 0.0000 | 0.0000 |
| Forelock | PAP C3 | 0.0046 | 0.0019 | 0.0002 |
| Gallbladder | Pet food | 0.0013 | 0.0019 | 0.0001 |
| Head trimmings | Pet food | 0.0013 | 0.0019 | 0.0001 |
| Heart | Human food | 0.0012 | 0.0019 | 0.0004 |
| Heart trimmings | Pet food | 0.0013 | 0.0019 | 0.0001 |
| Hide | Skin tannery C3 | 0.0018 | 0.0019 | 0.0033 |
| Hooves | PAP C3 | 0.0046 | 0.0019 | 0.0002 |
| Horns | PAP C3 | 0.0046 | 0.0019 | 0.0002 |
| Kidney | Human food | 0.0013 | 0.0019 | 0.0008 |
| Large intestine | C1-C2 for disposal | 0.0000 | 0.0000 | 0.0000 |
| Liver | Human food | 0.0053 | 0.0019 | 0.0010 |
| Liver trimmings | Pet food | 0.0055 | 0.0019 | 0.0001 |
| Lower jaw | PAP C3 | 0.0011 | 0.0019 | 0.0002 |
| Lungs | Pet food | 0.0015 | 0.0019 | 0.0001 |
| Mask | Skin tannery C3 | 0.0018 | 0.0019 | 0.0033 |
| Mesenteric fat | C1-C2 for disposal | 0.0000 | 0.0000 | 0.0000 |
| Muscle | Human food | 0.0013 | 0.0019 | 0.0033 |
| Muzzle | Human food | 0.0018 | 0.0019 | 0.0020 |
| Omasum | Human food | 0.0101 | 0.0019 | 0.0015 |
| Omasum fat | Fat and greaves C3 | 0.0038 | 0.0019 | 0.0002 |
| Rumen and forestomach | Human food | 0.0101 | 0.0019 | 0.0015 |
| Rumen fat | Fat and greaves C3 | 0.0038 | 0.0019 | 0.0002 |
| Sanitary seizures | C1-C2 for disposal | 0.0000 | 0.0000 | 0.0000 |
| Screening and sifting wastes | C1-C2 for disposal | 0.0000 | 0.0000 | 0.0000 |
| Small intestine | PAP C3 | 0.0101 | 0.0019 | 0.0001 |
| Spinal cord | C1-C2 for disposal | 0.0000 | 0.0000 | 0.0000 |
| Spinal cord waste | C1-C2 for disposal | 0.0000 | 0.0000 | 0.0000 |
| Spine | C1-C2 for disposal | 0.0000 | 0.0000 | 0.0000 |
| Spleen | Pet food | 0.0013 | 0.0019 | 0.0001 |
| Stillborn | PAP C3 | 0.0106 | 0.0019 | 0.0000 |
| Tallow | Fat and greaves C3 | 0.0017 | 0.0019 | 0.0002 |
| Tongue | Human food | 0.0011 | 0.0019 | 0.0031 |
| Tonsil | C1-C2 for disposal | 0.0000 | 0.0000 | 0.0000 |
| Trachea | Pet food | 0.0018 | 0.0019 | 0.0001 |
| Udder | Pet food | 0.0003 | 0.0019 | 0.0001 |
| Upper throat | Pet food | 0.0013 | 0.0019 | 0.0001 |
| Water in the rumen | Spreading/Compost | 0.0000 | 0.0000 | 0.0000 |

Table 27: Allocation factors for Primholstein Cull Cows reared in Grazing Large Area

| COPRODUCT | Destination | Primholstein/Cull cow/grazing large area | | |
| --- | --- | --- | --- | --- |
|  |  | **Biophysical Allocation Factor** | **Mass Allocation Factor** | **Economic Allocation Factor** |
| Abomasum | Human food | 0.0093 | 0.0017 | 0.0013 |
| Abomasum fat | Fat and greaves C3 | 0.0035 | 0.0017 | 0.0002 |
| Aponeurosis | Human food | 0.0013 | 0.0017 | 0.0018 |
| Bile | PAP C3 | 0.0004 | 0.0017 | 0.0002 |
| Blood | PAP C3 | 0.0010 | 0.0017 | 0.0004 |
| Blood | Pet food | 0.0010 | 0.0017 | 0.0001 |
| Bones | Gelatin C3 | 0.0010 | 0.0017 | 0.0000 |
| Bones of head, brain, eyes and teeth | C1-C2 for disposal | 0.0000 | 0.0000 | 0.0000 |
| Cheek | Human food | 0.0011 | 0.0017 | 0.0039 |
| Cheek | Human food | 0.0011 | 0.0017 | 0.0039 |
| Cheek trimmings | Pet food | 0.0011 | 0.0017 | 0.0001 |
| Chops | Pet food | 0.0016 | 0.0017 | 0.0001 |
| Contents of intestines | Spreading/Compost | 0.0000 | 0.0000 | 0.0000 |
| Contents of the rumen | Spreading/Compost | 0.0000 | 0.0000 | 0.0000 |
| Ears | PAP C3 | 0.0014 | 0.0017 | 0.0002 |
| Esophagus | Pet food | 0.0011 | 0.0017 | 0.0001 |
| Fat | Fat and greaves C3 | 0.0015 | 0.0017 | 0.0002 |
| Fat around heart | Fat and greaves C3 | 0.0015 | 0.0017 | 0.0002 |
| Fat in the kidney | Fat and greaves C3 | 0.0015 | 0.0017 | 0.0002 |
| Feet (without hooves) | Gelatin C3 | 0.0011 | 0.0017 | 0.0000 |
| Floatation fat | Spreading/Compost | 0.0000 | 0.0000 | 0.0000 |
| Forehead | C1-C2 for disposal | 0.0000 | 0.0000 | 0.0000 |
| Forelock | PAP C3 | 0.0041 | 0.0017 | 0.0002 |
| Gallbladder | Pet food | 0.0011 | 0.0017 | 0.0001 |
| Head trimmings | Pet food | 0.0011 | 0.0017 | 0.0001 |
| Heart | Human food | 0.0011 | 0.0017 | 0.0004 |
| Heart trimmings | Pet food | 0.0011 | 0.0017 | 0.0001 |
| Hide | Skin tannery C3 | 0.0016 | 0.0017 | 0.0030 |
| Hooves | PAP C3 | 0.0041 | 0.0017 | 0.0002 |
| Horns | PAP C3 | 0.0041 | 0.0017 | 0.0002 |
| Kidney | Human food | 0.0011 | 0.0017 | 0.0007 |
| Large intestine | C1-C2 for disposal | 0.0000 | 0.0000 | 0.0000 |
| Liver | Human food | 0.0048 | 0.0017 | 0.0009 |
| Liver trimmings | Pet food | 0.0050 | 0.0017 | 0.0001 |
| Lower jaw | PAP C3 | 0.0010 | 0.0017 | 0.0002 |
| Lungs | Pet food | 0.0013 | 0.0017 | 0.0001 |
| Mask | Skin tannery C3 | 0.0016 | 0.0017 | 0.0030 |
| Mesenteric fat | C1-C2 for disposal | 0.0000 | 0.0000 | 0.0000 |
| Muscle | Human food | 0.0011 | 0.0017 | 0.0030 |
| Muzzle | Human food | 0.0016 | 0.0017 | 0.0018 |
| Omasum | Human food | 0.0093 | 0.0017 | 0.0013 |
| Omasum fat | Fat and greaves C3 | 0.0035 | 0.0017 | 0.0002 |
| Rumen and forestomach | Human food | 0.0093 | 0.0017 | 0.0013 |
| Rumen fat | Fat and greaves C3 | 0.0035 | 0.0017 | 0.0002 |
| Sanitary seizures | C1-C2 for disposal | 0.0000 | 0.0000 | 0.0000 |
| Screening and sifting wastes | C1-C2 for disposal | 0.0000 | 0.0000 | 0.0000 |
| Small intestine | PAP C3 | 0.0093 | 0.0017 | 0.0001 |
| Spinal cord | C1-C2 for disposal | 0.0000 | 0.0000 | 0.0000 |
| Spinal cord waste | C1-C2 for disposal | 0.0000 | 0.0000 | 0.0000 |
| Spine | C1-C2 for disposal | 0.0000 | 0.0000 | 0.0000 |
| Spleen | Pet food | 0.0012 | 0.0017 | 0.0001 |
| Stillborn | PAP C3 | 0.0097 | 0.0017 | 0.0000 |
| Tallow | Fat and greaves C3 | 0.0015 | 0.0017 | 0.0002 |
| Tongue | Human food | 0.0010 | 0.0017 | 0.0028 |
| Tonsil | C1-C2 for disposal | 0.0000 | 0.0000 | 0.0000 |
| Trachea | Pet food | 0.0016 | 0.0017 | 0.0001 |
| Udder | Pet food | 0.0002 | 0.0017 | 0.0001 |
| Upper throat | Pet food | 0.0011 | 0.0017 | 0.0001 |
| Water in the rumen | Spreading/Compost | 0.0000 | 0.0000 | 0.0000 |

Table 28: Allocation factors for Primholstein Beef reared in Grazing Large Area

| COPRODUCT | Destination | Primholstein/beef/grazing large area | | |
| --- | --- | --- | --- | --- |
|  |  | **Biophysical Allocation Factor** | **Mass Allocation Factor** | **Economic Allocation Factor** |
| Abomasum | Human food | 0.0091 | 0.0016 | 0.0012 |
| Abomasum fat | Fat and greaves C3 | 0.0034 | 0.0016 | 0.0002 |
| Aponeurosis | Human food | 0.0012 | 0.0016 | 0.0017 |
| Bile | PAP C3 | 0.0004 | 0.0016 | 0.0001 |
| Blood | PAP C3 | 0.0009 | 0.0016 | 0.0004 |
| Blood | Pet food | 0.0009 | 0.0016 | 0.0001 |
| Bones | Gelatin C3 | 0.0010 | 0.0016 | 0.0000 |
| Bones of head, brain, eyes and teeth | C1-C2 for disposal | 0.0000 | 0.0000 | 0.0000 |
| Cheek | Human food | 0.0011 | 0.0016 | 0.0037 |
| Cheek | Human food | 0.0011 | 0.0016 | 0.0037 |
| Cheek trimmings | Pet food | 0.0011 | 0.0016 | 0.0001 |
| Chops | Pet food | 0.0015 | 0.0016 | 0.0001 |
| Contents of intestines | Spreading/Compost | 0.0000 | 0.0000 | 0.0000 |
| Contents of the rumen | Spreading/Compost | 0.0000 | 0.0000 | 0.0000 |
| Ears | PAP C3 | 0.0014 | 0.0016 | 0.0001 |
| Esophagus | Pet food | 0.0011 | 0.0016 | 0.0001 |
| Fat | Fat and greaves C3 | 0.0015 | 0.0016 | 0.0002 |
| Fat around heart | Fat and greaves C3 | 0.0015 | 0.0016 | 0.0002 |
| Fat in the kidney | Fat and greaves C3 | 0.0015 | 0.0016 | 0.0002 |
| Feet (without hooves) | Gelatin C3 | 0.0011 | 0.0016 | 0.0000 |
| Floatation fat | Spreading/Compost | 0.0000 | 0.0000 | 0.0000 |
| Forehead | C1-C2 for disposal | 0.0000 | 0.0000 | 0.0000 |
| Forelock | PAP C3 | 0.0039 | 0.0016 | 0.0001 |
| Gallbladder | Pet food | 0.0011 | 0.0016 | 0.0001 |
| Head trimmings | Pet food | 0.0011 | 0.0016 | 0.0001 |
| Heart | Human food | 0.0010 | 0.0016 | 0.0004 |
| Heart trimmings | Pet food | 0.0011 | 0.0016 | 0.0001 |
| Hide | Skin tannery C3 | 0.0015 | 0.0016 | 0.0028 |
| Hooves | PAP C3 | 0.0039 | 0.0016 | 0.0001 |
| Horns | PAP C3 | 0.0039 | 0.0016 | 0.0001 |
| Kidney | Human food | 0.0011 | 0.0016 | 0.0007 |
| Large intestine | C1-C2 for disposal | 0.0000 | 0.0000 | 0.0000 |
| Liver | Human food | 0.0047 | 0.0016 | 0.0008 |
| Liver trimmings | Pet food | 0.0049 | 0.0016 | 0.0001 |
| Lower jaw | PAP C3 | 0.0010 | 0.0016 | 0.0001 |
| Lungs | Pet food | 0.0013 | 0.0016 | 0.0001 |
| Mask | Skin tannery C3 | 0.0015 | 0.0016 | 0.0028 |
| Mesenteric fat | C1-C2 for disposal | 0.0000 | 0.0000 | 0.0000 |
| Muscle | Human food | 0.0011 | 0.0016 | 0.0028 |
| Muzzle | Human food | 0.0015 | 0.0016 | 0.0017 |
| Omasum | Human food | 0.0091 | 0.0016 | 0.0012 |
| Omasum fat | Fat and greaves C3 | 0.0034 | 0.0016 | 0.0002 |
| Rumen and forestomach | Human food | 0.0091 | 0.0016 | 0.0012 |
| Rumen fat | Fat and greaves C3 | 0.0034 | 0.0016 | 0.0002 |
| Sanitary seizures | C1-C2 for disposal | 0.0000 | 0.0000 | 0.0000 |
| Screening and sifting wastes | C1-C2 for disposal | 0.0000 | 0.0000 | 0.0000 |
| Small intestine | PAP C3 | 0.0091 | 0.0016 | 0.0001 |
| Spinal cord | C1-C2 for disposal | 0.0000 | 0.0000 | 0.0000 |
| Spinal cord waste | C1-C2 for disposal | 0.0000 | 0.0000 | 0.0000 |
| Spine | C1-C2 for disposal | 0.0000 | 0.0000 | 0.0000 |
| Spleen | Pet food | 0.0011 | 0.0016 | 0.0001 |
| Stillborn | PAP C3 | 0.0095 | 0.0016 | 0.0000 |
| Tallow | Fat and greaves C3 | 0.0015 | 0.0016 | 0.0002 |
| Tongue | Human food | 0.0010 | 0.0016 | 0.0027 |
| Tonsil | C1-C2 for disposal | 0.0000 | 0.0000 | 0.0000 |
| Trachea | Pet food | 0.0015 | 0.0016 | 0.0001 |
| Udder | Pet food | 0.0002 | 0.0016 | 0.0001 |
| Upper throat | Pet food | 0.0011 | 0.0016 | 0.0001 |
| Water in the rumen | Spreading/Compost | 0.0000 | 0.0000 | 0.0000 |

Table 29: Allocation factors for Primholstein Young Bulls reared in Pasture

| COPRODUCT | Destination | Primholstein/young bull/pasture | | |
| --- | --- | --- | --- | --- |
|  |  | **Biophysical Allocation Factor** | **Mass Allocation Factor** | **Economic Allocation Factor** |
| Abomasum | Human food | 0.0086 | 0.0016 | 0.0012 |
| Abomasum fat | Fat and greaves C3 | 0.0034 | 0.0016 | 0.0001 |
| Aponeurosis | Human food | 0.0012 | 0.0016 | 0.0016 |
| Bile | PAP C3 | 0.0004 | 0.0016 | 0.0001 |
| Blood | PAP C3 | 0.0009 | 0.0016 | 0.0004 |
| Blood | Pet food | 0.0009 | 0.0016 | 0.0001 |
| Bones | Gelatin C3 | 0.0010 | 0.0016 | 0.0000 |
| Bones of head, brain, eyes and teeth | C1-C2 for disposal | 0.0000 | 0.0000 | 0.0000 |
| Cheek | Human food | 0.0011 | 0.0016 | 0.0036 |
| Cheek | Human food | 0.0011 | 0.0016 | 0.0036 |
| Cheek trimmings | Pet food | 0.0011 | 0.0016 | 0.0001 |
| Chops | Pet food | 0.0015 | 0.0016 | 0.0001 |
| Contents of intestines | Spreading/Compost | 0.0000 | 0.0000 | 0.0000 |
| Contents of the rumen | Spreading/Compost | 0.0000 | 0.0000 | 0.0000 |
| Ears | PAP C3 | 0.0013 | 0.0016 | 0.0001 |
| Esophagus | Pet food | 0.0010 | 0.0016 | 0.0001 |
| Fat | Fat and greaves C3 | 0.0016 | 0.0016 | 0.0001 |
| Fat around heart | Fat and greaves C3 | 0.0016 | 0.0016 | 0.0001 |
| Fat in the kidney | Fat and greaves C3 | 0.0016 | 0.0016 | 0.0001 |
| Feet (without hooves) | Gelatin C3 | 0.0011 | 0.0016 | 0.0000 |
| Floatation fat | Spreading/Compost | 0.0000 | 0.0000 | 0.0000 |
| Forehead | C1-C2 for disposal | 0.0000 | 0.0000 | 0.0000 |
| Forelock | PAP C3 | 0.0039 | 0.0016 | 0.0001 |
| Gallbladder | Pet food | 0.0011 | 0.0016 | 0.0001 |
| Head trimmings | Pet food | 0.0011 | 0.0016 | 0.0001 |
| Heart | Human food | 0.0010 | 0.0016 | 0.0003 |
| Heart trimmings | Pet food | 0.0011 | 0.0016 | 0.0001 |
| Hide | Skin tannery C3 | 0.0015 | 0.0016 | 0.0027 |
| Hooves | PAP C3 | 0.0039 | 0.0016 | 0.0001 |
| Horns | PAP C3 | 0.0039 | 0.0016 | 0.0001 |
| Kidney | Human food | 0.0011 | 0.0016 | 0.0007 |
| Large intestine | C1-C2 for disposal | 0.0000 | 0.0000 | 0.0000 |
| Liver | Human food | 0.0045 | 0.0016 | 0.0008 |
| Liver trimmings | Pet food | 0.0047 | 0.0016 | 0.0001 |
| Lower jaw | PAP C3 | 0.0010 | 0.0016 | 0.0001 |
| Lungs | Pet food | 0.0012 | 0.0016 | 0.0001 |
| Mask | Skin tannery C3 | 0.0015 | 0.0016 | 0.0027 |
| Mesenteric fat | C1-C2 for disposal | 0.0000 | 0.0000 | 0.0000 |
| Muscle | Human food | 0.0011 | 0.0016 | 0.0027 |
| Muzzle | Human food | 0.0015 | 0.0016 | 0.0016 |
| Omasum | Human food | 0.0086 | 0.0016 | 0.0012 |
| Omasum fat | Fat and greaves C3 | 0.0034 | 0.0016 | 0.0001 |
| Rumen and forestomach | Human food | 0.0086 | 0.0016 | 0.0012 |
| Rumen fat | Fat and greaves C3 | 0.0034 | 0.0016 | 0.0001 |
| Sanitary seizures | C1-C2 for disposal | 0.0000 | 0.0000 | 0.0000 |
| Screening and sifting wastes | C1-C2 for disposal | 0.0000 | 0.0000 | 0.0000 |
| Small intestine | PAP C3 | 0.0086 | 0.0016 | 0.0001 |
| Spinal cord | C1-C2 for disposal | 0.0000 | 0.0000 | 0.0000 |
| Spinal cord waste | C1-C2 for disposal | 0.0000 | 0.0000 | 0.0000 |
| Spine | C1-C2 for disposal | 0.0000 | 0.0000 | 0.0000 |
| Spleen | Pet food | 0.0011 | 0.0016 | 0.0001 |
| Stillborn | PAP C3 | 0.0090 | 0.0016 | 0.0000 |
| Tallow | Fat and greaves C3 | 0.0016 | 0.0016 | 0.0001 |
| Tongue | Human food | 0.0010 | 0.0016 | 0.0026 |
| Tonsil | C1-C2 for disposal | 0.0000 | 0.0000 | 0.0000 |
| Trachea | Pet food | 0.0015 | 0.0016 | 0.0001 |
| Udder | Pet food | 0.0002 | 0.0016 | 0.0001 |
| Upper throat | Pet food | 0.0011 | 0.0016 | 0.0001 |
| Water in the rumen | Spreading/Compost | 0.0000 | 0.0000 | 0.0000 |

Table 30: Allocation factors for Primholstein Heifers reared in Pasture

| COPRODUCT | Destination | Primholstein/heifer/pasture | | |
| --- | --- | --- | --- | --- |
|  |  | **Biophysical Allocation Factor** | **Mass Allocation Factor** | **Economic Allocation Factor** |
| Abomasum | Human food | 0.0097 | 0.0019 | 0.0015 |
| Abomasum fat | Fat and greaves C3 | 0.0039 | 0.0019 | 0.0002 |
| Aponeurosis | Human food | 0.0014 | 0.0019 | 0.0020 |
| Bile | PAP C3 | 0.0005 | 0.0019 | 0.0002 |
| Blood | PAP C3 | 0.0011 | 0.0019 | 0.0004 |
| Blood | Pet food | 0.0011 | 0.0019 | 0.0001 |
| Bones | Gelatin C3 | 0.0012 | 0.0019 | 0.0000 |
| Bones of head, brain, eyes and teeth | C1-C2 for disposal | 0.0000 | 0.0000 | 0.0000 |
| Cheek | Human food | 0.0013 | 0.0019 | 0.0043 |
| Cheek | Human food | 0.0013 | 0.0019 | 0.0043 |
| Cheek trimmings | Pet food | 0.0013 | 0.0019 | 0.0001 |
| Chops | Pet food | 0.0018 | 0.0019 | 0.0001 |
| Contents of intestines | Spreading/Compost | 0.0000 | 0.0000 | 0.0000 |
| Contents of the rumen | Spreading/Compost | 0.0000 | 0.0000 | 0.0000 |
| Ears | PAP C3 | 0.0016 | 0.0019 | 0.0002 |
| Esophagus | Pet food | 0.0012 | 0.0019 | 0.0001 |
| Fat | Fat and greaves C3 | 0.0018 | 0.0019 | 0.0002 |
| Fat around heart | Fat and greaves C3 | 0.0018 | 0.0019 | 0.0002 |
| Fat in the kidney | Fat and greaves C3 | 0.0018 | 0.0019 | 0.0002 |
| Feet (without hooves) | Gelatin C3 | 0.0013 | 0.0019 | 0.0000 |
| Floatation fat | Spreading/Compost | 0.0000 | 0.0000 | 0.0000 |
| Forehead | C1-C2 for disposal | 0.0000 | 0.0000 | 0.0000 |
| Forelock | PAP C3 | 0.0046 | 0.0019 | 0.0002 |
| Gallbladder | Pet food | 0.0013 | 0.0019 | 0.0001 |
| Head trimmings | Pet food | 0.0013 | 0.0019 | 0.0001 |
| Heart | Human food | 0.0012 | 0.0019 | 0.0004 |
| Heart trimmings | Pet food | 0.0013 | 0.0019 | 0.0001 |
| Hide | Skin tannery C3 | 0.0018 | 0.0019 | 0.0033 |
| Hooves | PAP C3 | 0.0046 | 0.0019 | 0.0002 |
| Horns | PAP C3 | 0.0046 | 0.0019 | 0.0002 |
| Kidney | Human food | 0.0013 | 0.0019 | 0.0008 |
| Large intestine | C1-C2 for disposal | 0.0000 | 0.0000 | 0.0000 |
| Liver | Human food | 0.0051 | 0.0019 | 0.0010 |
| Liver trimmings | Pet food | 0.0053 | 0.0019 | 0.0001 |
| Lower jaw | PAP C3 | 0.0012 | 0.0019 | 0.0002 |
| Lungs | Pet food | 0.0015 | 0.0019 | 0.0001 |
| Mask | Skin tannery C3 | 0.0018 | 0.0019 | 0.0033 |
| Mesenteric fat | C1-C2 for disposal | 0.0000 | 0.0000 | 0.0000 |
| Muscle | Human food | 0.0013 | 0.0019 | 0.0033 |
| Muzzle | Human food | 0.0018 | 0.0019 | 0.0020 |
| Omasum | Human food | 0.0097 | 0.0019 | 0.0015 |
| Omasum fat | Fat and greaves C3 | 0.0039 | 0.0019 | 0.0002 |
| Rumen and forestomach | Human food | 0.0097 | 0.0019 | 0.0015 |
| Rumen fat | Fat and greaves C3 | 0.0039 | 0.0019 | 0.0002 |
| Sanitary seizures | C1-C2 for disposal | 0.0000 | 0.0000 | 0.0000 |
| Screening and sifting wastes | C1-C2 for disposal | 0.0000 | 0.0000 | 0.0000 |
| Small intestine | PAP C3 | 0.0097 | 0.0019 | 0.0001 |
| Spinal cord | C1-C2 for disposal | 0.0000 | 0.0000 | 0.0000 |
| Spinal cord waste | C1-C2 for disposal | 0.0000 | 0.0000 | 0.0000 |
| Spine | C1-C2 for disposal | 0.0000 | 0.0000 | 0.0000 |
| Spleen | Pet food | 0.0013 | 0.0019 | 0.0001 |
| Stillborn | PAP C3 | 0.0101 | 0.0019 | 0.0000 |
| Tallow | Fat and greaves C3 | 0.0018 | 0.0019 | 0.0002 |
| Tongue | Human food | 0.0011 | 0.0019 | 0.0031 |
| Tonsil | C1-C2 for disposal | 0.0000 | 0.0000 | 0.0000 |
| Trachea | Pet food | 0.0018 | 0.0019 | 0.0001 |
| Udder | Pet food | 0.0003 | 0.0019 | 0.0001 |
| Upper throat | Pet food | 0.0013 | 0.0019 | 0.0001 |
| Water in the rumen | Spreading/Compost | 0.0000 | 0.0000 | 0.0000 |

Table 31: Allocation factors for Primholstein Cull Cows reared in Pasture

| COPRODUCT | Destination | Primholstein/Cull cow/pasture | | |
| --- | --- | --- | --- | --- |
|  |  | **Biophysical Allocation Factor** | **Mass Allocation Factor** | **Economic Allocation Factor** |
| Abomasum | Human food | 0.0089 | 0.0017 | 0.0013 |
| Abomasum fat | Fat and greaves C3 | 0.0035 | 0.0017 | 0.0002 |
| Aponeurosis | Human food | 0.0012 | 0.0017 | 0.0018 |
| Bile | PAP C3 | 0.0004 | 0.0017 | 0.0002 |
| Blood | PAP C3 | 0.0010 | 0.0017 | 0.0004 |
| Blood | Pet food | 0.0010 | 0.0017 | 0.0001 |
| Bones | Gelatin C3 | 0.0011 | 0.0017 | 0.0000 |
| Bones of head, brain, eyes and teeth | C1-C2 for disposal | 0.0000 | 0.0000 | 0.0000 |
| Cheek | Human food | 0.0011 | 0.0017 | 0.0039 |
| Cheek | Human food | 0.0011 | 0.0017 | 0.0039 |
| Cheek trimmings | Pet food | 0.0011 | 0.0017 | 0.0001 |
| Chops | Pet food | 0.0016 | 0.0017 | 0.0001 |
| Contents of intestines | Spreading/Compost | 0.0000 | 0.0000 | 0.0000 |
| Contents of the rumen | Spreading/Compost | 0.0000 | 0.0000 | 0.0000 |
| Ears | PAP C3 | 0.0014 | 0.0017 | 0.0002 |
| Esophagus | Pet food | 0.0011 | 0.0017 | 0.0001 |
| Fat | Fat and greaves C3 | 0.0017 | 0.0017 | 0.0002 |
| Fat around heart | Fat and greaves C3 | 0.0016 | 0.0017 | 0.0002 |
| Fat in the kidney | Fat and greaves C3 | 0.0016 | 0.0017 | 0.0002 |
| Feet (without hooves) | Gelatin C3 | 0.0011 | 0.0017 | 0.0000 |
| Floatation fat | Spreading/Compost | 0.0000 | 0.0000 | 0.0000 |
| Forehead | C1-C2 for disposal | 0.0000 | 0.0000 | 0.0000 |
| Forelock | PAP C3 | 0.0041 | 0.0017 | 0.0002 |
| Gallbladder | Pet food | 0.0011 | 0.0017 | 0.0001 |
| Head trimmings | Pet food | 0.0011 | 0.0017 | 0.0001 |
| Heart | Human food | 0.0011 | 0.0017 | 0.0004 |
| Heart trimmings | Pet food | 0.0011 | 0.0017 | 0.0001 |
| Hide | Skin tannery C3 | 0.0016 | 0.0017 | 0.0030 |
| Hooves | PAP C3 | 0.0041 | 0.0017 | 0.0002 |
| Horns | PAP C3 | 0.0041 | 0.0017 | 0.0002 |
| Kidney | Human food | 0.0011 | 0.0017 | 0.0007 |
| Large intestine | C1-C2 for disposal | 0.0000 | 0.0000 | 0.0000 |
| Liver | Human food | 0.0046 | 0.0017 | 0.0009 |
| Liver trimmings | Pet food | 0.0048 | 0.0017 | 0.0001 |
| Lower jaw | PAP C3 | 0.0010 | 0.0017 | 0.0002 |
| Lungs | Pet food | 0.0013 | 0.0017 | 0.0001 |
| Mask | Skin tannery C3 | 0.0016 | 0.0017 | 0.0030 |
| Mesenteric fat | C1-C2 for disposal | 0.0000 | 0.0000 | 0.0000 |
| Muscle | Human food | 0.0011 | 0.0017 | 0.0030 |
| Muzzle | Human food | 0.0016 | 0.0017 | 0.0018 |
| Omasum | Human food | 0.0089 | 0.0017 | 0.0013 |
| Omasum fat | Fat and greaves C3 | 0.0035 | 0.0017 | 0.0002 |
| Rumen and forestomach | Human food | 0.0089 | 0.0017 | 0.0013 |
| Rumen fat | Fat and greaves C3 | 0.0035 | 0.0017 | 0.0002 |
| Sanitary seizures | C1-C2 for disposal | 0.0000 | 0.0000 | 0.0000 |
| Screening and sifting wastes | C1-C2 for disposal | 0.0000 | 0.0000 | 0.0000 |
| Small intestine | PAP C3 | 0.0089 | 0.0017 | 0.0001 |
| Spinal cord | C1-C2 for disposal | 0.0000 | 0.0000 | 0.0000 |
| Spinal cord waste | C1-C2 for disposal | 0.0000 | 0.0000 | 0.0000 |
| Spine | C1-C2 for disposal | 0.0000 | 0.0000 | 0.0000 |
| Spleen | Pet food | 0.0012 | 0.0017 | 0.0001 |
| Stillborn | PAP C3 | 0.0093 | 0.0017 | 0.0000 |
| Tallow | Fat and greaves C3 | 0.0016 | 0.0017 | 0.0002 |
| Tongue | Human food | 0.0010 | 0.0017 | 0.0028 |
| Tonsil | C1-C2 for disposal | 0.0000 | 0.0000 | 0.0000 |
| Trachea | Pet food | 0.0016 | 0.0017 | 0.0001 |
| Udder | Pet food | 0.0002 | 0.0017 | 0.0001 |
| Upper throat | Pet food | 0.0011 | 0.0017 | 0.0001 |
| Water in the rumen | Spreading/Compost | 0.0000 | 0.0000 | 0.0000 |

Table 32: Allocation factors for Primholstein Beef reared in Pasture

| COPRODUCT | Destination | Primholstein/beef/pasture | | |
| --- | --- | --- | --- | --- |
|  |  | **Biophysical Allocation Factor** | **Mass Allocation Factor** | **Economic Allocation Factor** |
| Abomasum | Human food | 0.0087 | 0.0016 | 0.0012 |
| Abomasum fat | Fat and greaves C3 | 0.0034 | 0.0016 | 0.0002 |
| Aponeurosis | Human food | 0.0012 | 0.0016 | 0.0017 |
| Bile | PAP C3 | 0.0004 | 0.0016 | 0.0001 |
| Blood | PAP C3 | 0.0009 | 0.0016 | 0.0004 |
| Blood | Pet food | 0.0009 | 0.0016 | 0.0001 |
| Bones | Gelatin C3 | 0.0010 | 0.0016 | 0.0000 |
| Bones of head, brain, eyes and teeth | C1-C2 for disposal | 0.0000 | 0.0000 | 0.0000 |
| Cheek | Human food | 0.0011 | 0.0016 | 0.0037 |
| Cheek | Human food | 0.0011 | 0.0016 | 0.0037 |
| Cheek trimmings | Pet food | 0.0011 | 0.0016 | 0.0001 |
| Chops | Pet food | 0.0015 | 0.0016 | 0.0001 |
| Contents of intestines | Spreading/Compost | 0.0000 | 0.0000 | 0.0000 |
| Contents of the rumen | Spreading/Compost | 0.0000 | 0.0000 | 0.0000 |
| Ears | PAP C3 | 0.0014 | 0.0016 | 0.0001 |
| Esophagus | Pet food | 0.0011 | 0.0016 | 0.0001 |
| Fat | Fat and greaves C3 | 0.0016 | 0.0016 | 0.0002 |
| Fat around heart | Fat and greaves C3 | 0.0016 | 0.0016 | 0.0002 |
| Fat in the kidney | Fat and greaves C3 | 0.0016 | 0.0016 | 0.0002 |
| Feet (without hooves) | Gelatin C3 | 0.0011 | 0.0016 | 0.0000 |
| Floatation fat | Spreading/Compost | 0.0000 | 0.0000 | 0.0000 |
| Forehead | C1-C2 for disposal | 0.0000 | 0.0000 | 0.0000 |
| Forelock | PAP C3 | 0.0039 | 0.0016 | 0.0001 |
| Gallbladder | Pet food | 0.0011 | 0.0016 | 0.0001 |
| Head trimmings | Pet food | 0.0011 | 0.0016 | 0.0001 |
| Heart | Human food | 0.0010 | 0.0016 | 0.0004 |
| Heart trimmings | Pet food | 0.0011 | 0.0016 | 0.0001 |
| Hide | Skin tannery C3 | 0.0015 | 0.0016 | 0.0028 |
| Hooves | PAP C3 | 0.0039 | 0.0016 | 0.0001 |
| Horns | PAP C3 | 0.0039 | 0.0016 | 0.0001 |
| Kidney | Human food | 0.0011 | 0.0016 | 0.0007 |
| Large intestine | C1-C2 for disposal | 0.0000 | 0.0000 | 0.0000 |
| Liver | Human food | 0.0045 | 0.0016 | 0.0008 |
| Liver trimmings | Pet food | 0.0047 | 0.0016 | 0.0001 |
| Lower jaw | PAP C3 | 0.0010 | 0.0016 | 0.0001 |
| Lungs | Pet food | 0.0013 | 0.0016 | 0.0001 |
| Mask | Skin tannery C3 | 0.0015 | 0.0016 | 0.0028 |
| Mesenteric fat | C1-C2 for disposal | 0.0000 | 0.0000 | 0.0000 |
| Muscle | Human food | 0.0011 | 0.0016 | 0.0028 |
| Muzzle | Human food | 0.0015 | 0.0016 | 0.0017 |
| Omasum | Human food | 0.0087 | 0.0016 | 0.0012 |
| Omasum fat | Fat and greaves C3 | 0.0034 | 0.0016 | 0.0002 |
| Rumen and forestomach | Human food | 0.0087 | 0.0016 | 0.0012 |
| Rumen fat | Fat and greaves C3 | 0.0034 | 0.0016 | 0.0002 |
| Sanitary seizures | C1-C2 for disposal | 0.0000 | 0.0000 | 0.0000 |
| Screening and sifting wastes | C1-C2 for disposal | 0.0000 | 0.0000 | 0.0000 |
| Small intestine | PAP C3 | 0.0087 | 0.0016 | 0.0001 |
| Spinal cord | C1-C2 for disposal | 0.0000 | 0.0000 | 0.0000 |
| Spinal cord waste | C1-C2 for disposal | 0.0000 | 0.0000 | 0.0000 |
| Spine | C1-C2 for disposal | 0.0000 | 0.0000 | 0.0000 |
| Spleen | Pet food | 0.0011 | 0.0016 | 0.0001 |
| Stillborn | PAP C3 | 0.0091 | 0.0016 | 0.0000 |
| Tallow | Fat and greaves C3 | 0.0016 | 0.0016 | 0.0002 |
| Tongue | Human food | 0.0010 | 0.0016 | 0.0027 |
| Tonsil | C1-C2 for disposal | 0.0000 | 0.0000 | 0.0000 |
| Trachea | Pet food | 0.0015 | 0.0016 | 0.0001 |
| Udder | Pet food | 0.0002 | 0.0016 | 0.0001 |
| Upper throat | Pet food | 0.0011 | 0.0016 | 0.0001 |
| Water in the rumen | Spreading/Compost | 0.0000 | 0.0000 | 0.0000 |

Table 33: Allocation factors for Primholstein Young Bulls reared in Stall

| COPRODUCT | Destination | Primholstein/young bull/stall | | |
| --- | --- | --- | --- | --- |
|  |  | **Biophysical Allocation Factor** | **Mass Allocation Factor** | **Economic Allocation Factor** |
| Abomasum | Human food | 0.0082 | 0.0016 | 0.0012 |
| Abomasum fat | Fat and greaves C3 | 0.0034 | 0.0016 | 0.0001 |
| Aponeurosis | Human food | 0.0012 | 0.0016 | 0.0016 |
| Bile | PAP C3 | 0.0004 | 0.0016 | 0.0001 |
| Blood | PAP C3 | 0.0009 | 0.0016 | 0.0004 |
| Blood | Pet food | 0.0009 | 0.0016 | 0.0001 |
| Bones | Gelatin C3 | 0.0010 | 0.0016 | 0.0000 |
| Bones of head, brain, eyes and teeth | C1-C2 for disposal | 0.0000 | 0.0000 | 0.0000 |
| Cheek | Human food | 0.0011 | 0.0016 | 0.0036 |
| Cheek | Human food | 0.0011 | 0.0016 | 0.0036 |
| Cheek trimmings | Pet food | 0.0011 | 0.0016 | 0.0001 |
| Chops | Pet food | 0.0015 | 0.0016 | 0.0001 |
| Contents of intestines | Spreading/Compost | 0.0000 | 0.0000 | 0.0000 |
| Contents of the rumen | Spreading/Compost | 0.0000 | 0.0000 | 0.0000 |
| Ears | PAP C3 | 0.0013 | 0.0016 | 0.0001 |
| Esophagus | Pet food | 0.0010 | 0.0016 | 0.0001 |
| Fat | Fat and greaves C3 | 0.0017 | 0.0016 | 0.0001 |
| Fat around heart | Fat and greaves C3 | 0.0017 | 0.0016 | 0.0001 |
| Fat in the kidney | Fat and greaves C3 | 0.0017 | 0.0016 | 0.0001 |
| Feet (without hooves) | Gelatin C3 | 0.0011 | 0.0016 | 0.0000 |
| Floatation fat | Spreading/Compost | 0.0000 | 0.0000 | 0.0000 |
| Forehead | C1-C2 for disposal | 0.0000 | 0.0000 | 0.0000 |
| Forelock | PAP C3 | 0.0038 | 0.0016 | 0.0001 |
| Gallbladder | Pet food | 0.0011 | 0.0016 | 0.0001 |
| Head trimmings | Pet food | 0.0011 | 0.0016 | 0.0001 |
| Heart | Human food | 0.0010 | 0.0016 | 0.0003 |
| Heart trimmings | Pet food | 0.0011 | 0.0016 | 0.0001 |
| Hide | Skin tannery C3 | 0.0015 | 0.0016 | 0.0027 |
| Hooves | PAP C3 | 0.0038 | 0.0016 | 0.0001 |
| Horns | PAP C3 | 0.0038 | 0.0016 | 0.0001 |
| Kidney | Human food | 0.0011 | 0.0016 | 0.0007 |
| Large intestine | C1-C2 for disposal | 0.0000 | 0.0000 | 0.0000 |
| Liver | Human food | 0.0043 | 0.0016 | 0.0008 |
| Liver trimmings | Pet food | 0.0045 | 0.0016 | 0.0001 |
| Lower jaw | PAP C3 | 0.0010 | 0.0016 | 0.0001 |
| Lungs | Pet food | 0.0012 | 0.0016 | 0.0001 |
| Mask | Skin tannery C3 | 0.0015 | 0.0016 | 0.0027 |
| Mesenteric fat | C1-C2 for disposal | 0.0000 | 0.0000 | 0.0000 |
| Muscle | Human food | 0.0011 | 0.0016 | 0.0027 |
| Muzzle | Human food | 0.0015 | 0.0016 | 0.0016 |
| Omasum | Human food | 0.0082 | 0.0016 | 0.0012 |
| Omasum fat | Fat and greaves C3 | 0.0034 | 0.0016 | 0.0001 |
| Rumen and forestomach | Human food | 0.0082 | 0.0016 | 0.0012 |
| Rumen fat | Fat and greaves C3 | 0.0034 | 0.0016 | 0.0001 |
| Sanitary seizures | C1-C2 for disposal | 0.0000 | 0.0000 | 0.0000 |
| Screening and sifting wastes | C1-C2 for disposal | 0.0000 | 0.0000 | 0.0000 |
| Small intestine | PAP C3 | 0.0082 | 0.0016 | 0.0001 |
| Spinal cord | C1-C2 for disposal | 0.0000 | 0.0000 | 0.0000 |
| Spinal cord waste | C1-C2 for disposal | 0.0000 | 0.0000 | 0.0000 |
| Spine | C1-C2 for disposal | 0.0000 | 0.0000 | 0.0000 |
| Spleen | Pet food | 0.0011 | 0.0016 | 0.0001 |
| Stillborn | PAP C3 | 0.0085 | 0.0016 | 0.0000 |
| Tallow | Fat and greaves C3 | 0.0017 | 0.0016 | 0.0001 |
| Tongue | Human food | 0.0010 | 0.0016 | 0.0026 |
| Tonsil | C1-C2 for disposal | 0.0000 | 0.0000 | 0.0000 |
| Trachea | Pet food | 0.0015 | 0.0016 | 0.0001 |
| Udder | Pet food | 0.0002 | 0.0016 | 0.0001 |
| Upper throat | Pet food | 0.0011 | 0.0016 | 0.0001 |
| Water in the rumen | Spreading/Compost | 0.0000 | 0.0000 | 0.0000 |

Table 34: Allocation factors for Primholstein Heifers reared in Stall

| COPRODUCT | Destination | Primholstein/heifer/stall | | |
| --- | --- | --- | --- | --- |
|  |  | **Biophysical Allocation Factor** | **Mass Allocation Factor** | **Economic Allocation Factor** |
| Abomasum | Human food | 0.0092 | 0.0019 | 0.0015 |
| Abomasum fat | Fat and greaves C3 | 0.0039 | 0.0019 | 0.0002 |
| Aponeurosis | Human food | 0.0014 | 0.0019 | 0.0020 |
| Bile | PAP C3 | 0.0005 | 0.0019 | 0.0002 |
| Blood | PAP C3 | 0.0011 | 0.0019 | 0.0004 |
| Blood | Pet food | 0.0011 | 0.0019 | 0.0001 |
| Bones | Gelatin C3 | 0.0012 | 0.0019 | 0.0000 |
| Bones of head, brain, eyes and teeth | C1-C2 for disposal | 0.0000 | 0.0000 | 0.0000 |
| Cheek | Human food | 0.0013 | 0.0019 | 0.0043 |
| Cheek | Human food | 0.0013 | 0.0019 | 0.0043 |
| Cheek trimmings | Pet food | 0.0013 | 0.0019 | 0.0001 |
| Chops | Pet food | 0.0018 | 0.0019 | 0.0001 |
| Contents of intestines | Spreading/Compost | 0.0000 | 0.0000 | 0.0000 |
| Contents of the rumen | Spreading/Compost | 0.0000 | 0.0000 | 0.0000 |
| Ears | PAP C3 | 0.0016 | 0.0019 | 0.0002 |
| Esophagus | Pet food | 0.0012 | 0.0019 | 0.0001 |
| Fat | Fat and greaves C3 | 0.0020 | 0.0019 | 0.0002 |
| Fat around heart | Fat and greaves C3 | 0.0020 | 0.0019 | 0.0002 |
| Fat in the kidney | Fat and greaves C3 | 0.0020 | 0.0019 | 0.0002 |
| Feet (without hooves) | Gelatin C3 | 0.0013 | 0.0019 | 0.0000 |
| Floatation fat | Spreading/Compost | 0.0000 | 0.0000 | 0.0000 |
| Forehead | C1-C2 for disposal | 0.0000 | 0.0000 | 0.0000 |
| Forelock | PAP C3 | 0.0045 | 0.0019 | 0.0002 |
| Gallbladder | Pet food | 0.0013 | 0.0019 | 0.0001 |
| Head trimmings | Pet food | 0.0013 | 0.0019 | 0.0001 |
| Heart | Human food | 0.0012 | 0.0019 | 0.0004 |
| Heart trimmings | Pet food | 0.0013 | 0.0019 | 0.0001 |
| Hide | Skin tannery C3 | 0.0018 | 0.0019 | 0.0033 |
| Hooves | PAP C3 | 0.0045 | 0.0019 | 0.0002 |
| Horns | PAP C3 | 0.0045 | 0.0019 | 0.0002 |
| Kidney | Human food | 0.0012 | 0.0019 | 0.0008 |
| Large intestine | C1-C2 for disposal | 0.0000 | 0.0000 | 0.0000 |
| Liver | Human food | 0.0048 | 0.0019 | 0.0010 |
| Liver trimmings | Pet food | 0.0050 | 0.0019 | 0.0001 |
| Lower jaw | PAP C3 | 0.0012 | 0.0019 | 0.0002 |
| Lungs | Pet food | 0.0015 | 0.0019 | 0.0001 |
| Mask | Skin tannery C3 | 0.0018 | 0.0019 | 0.0033 |
| Mesenteric fat | C1-C2 for disposal | 0.0000 | 0.0000 | 0.0000 |
| Muscle | Human food | 0.0013 | 0.0019 | 0.0033 |
| Muzzle | Human food | 0.0018 | 0.0019 | 0.0020 |
| Omasum | Human food | 0.0092 | 0.0019 | 0.0015 |
| Omasum fat | Fat and greaves C3 | 0.0039 | 0.0019 | 0.0002 |
| Rumen and forestomach | Human food | 0.0092 | 0.0019 | 0.0015 |
| Rumen fat | Fat and greaves C3 | 0.0039 | 0.0019 | 0.0002 |
| Sanitary seizures | C1-C2 for disposal | 0.0000 | 0.0000 | 0.0000 |
| Screening and sifting wastes | C1-C2 for disposal | 0.0000 | 0.0000 | 0.0000 |
| Small intestine | PAP C3 | 0.0092 | 0.0019 | 0.0001 |
| Spinal cord | C1-C2 for disposal | 0.0000 | 0.0000 | 0.0000 |
| Spinal cord waste | C1-C2 for disposal | 0.0000 | 0.0000 | 0.0000 |
| Spine | C1-C2 for disposal | 0.0000 | 0.0000 | 0.0000 |
| Spleen | Pet food | 0.0013 | 0.0019 | 0.0001 |
| Stillborn | PAP C3 | 0.0096 | 0.0019 | 0.0000 |
| Tallow | Fat and greaves C3 | 0.0020 | 0.0019 | 0.0002 |
| Tongue | Human food | 0.0011 | 0.0019 | 0.0031 |
| Tonsil | C1-C2 for disposal | 0.0000 | 0.0000 | 0.0000 |
| Trachea | Pet food | 0.0018 | 0.0019 | 0.0001 |
| Udder | Pet food | 0.0003 | 0.0019 | 0.0001 |
| Upper throat | Pet food | 0.0013 | 0.0019 | 0.0001 |
| Water in the rumen | Spreading/Compost | 0.0000 | 0.0000 | 0.0000 |

Table 35: Allocation factors for Primholstein Cull Cows reared in Stall

| COPRODUCT | Destination | Primholstein/Cull cow/stall | | |
| --- | --- | --- | --- | --- |
|  |  | **Biophysical Allocation Factor** | **Mass Allocation Factor** | **Economic Allocation Factor** |
| Abomasum | Human food | 0.0084 | 0.0017 | 0.0013 |
| Abomasum fat | Fat and greaves C3 | 0.0035 | 0.0017 | 0.0002 |
| Aponeurosis | Human food | 0.0012 | 0.0017 | 0.0018 |
| Bile | PAP C3 | 0.0004 | 0.0017 | 0.0002 |
| Blood | PAP C3 | 0.0010 | 0.0017 | 0.0004 |
| Blood | Pet food | 0.0010 | 0.0017 | 0.0001 |
| Bones | Gelatin C3 | 0.0011 | 0.0017 | 0.0000 |
| Bones of head, brain, eyes and teeth | C1-C2 for disposal | 0.0000 | 0.0000 | 0.0000 |
| Cheek | Human food | 0.0011 | 0.0017 | 0.0039 |
| Cheek | Human food | 0.0011 | 0.0017 | 0.0039 |
| Cheek trimmings | Pet food | 0.0011 | 0.0017 | 0.0001 |
| Chops | Pet food | 0.0016 | 0.0017 | 0.0001 |
| Contents of intestines | Spreading/Compost | 0.0000 | 0.0000 | 0.0000 |
| Contents of the rumen | Spreading/Compost | 0.0000 | 0.0000 | 0.0000 |
| Ears | PAP C3 | 0.0014 | 0.0017 | 0.0002 |
| Esophagus | Pet food | 0.0011 | 0.0017 | 0.0001 |
| Fat | Fat and greaves C3 | 0.0018 | 0.0017 | 0.0002 |
| Fat around heart | Fat and greaves C3 | 0.0018 | 0.0017 | 0.0002 |
| Fat in the kidney | Fat and greaves C3 | 0.0018 | 0.0017 | 0.0002 |
| Feet (without hooves) | Gelatin C3 | 0.0011 | 0.0017 | 0.0000 |
| Floatation fat | Spreading/Compost | 0.0000 | 0.0000 | 0.0000 |
| Forehead | C1-C2 for disposal | 0.0000 | 0.0000 | 0.0000 |
| Forelock | PAP C3 | 0.0040 | 0.0017 | 0.0002 |
| Gallbladder | Pet food | 0.0011 | 0.0017 | 0.0001 |
| Head trimmings | Pet food | 0.0011 | 0.0017 | 0.0001 |
| Heart | Human food | 0.0011 | 0.0017 | 0.0004 |
| Heart trimmings | Pet food | 0.0011 | 0.0017 | 0.0001 |
| Hide | Skin tannery C3 | 0.0016 | 0.0017 | 0.0030 |
| Hooves | PAP C3 | 0.0040 | 0.0017 | 0.0002 |
| Horns | PAP C3 | 0.0040 | 0.0017 | 0.0002 |
| Kidney | Human food | 0.0011 | 0.0017 | 0.0007 |
| Large intestine | C1-C2 for disposal | 0.0000 | 0.0000 | 0.0000 |
| Liver | Human food | 0.0044 | 0.0017 | 0.0009 |
| Liver trimmings | Pet food | 0.0046 | 0.0017 | 0.0001 |
| Lower jaw | PAP C3 | 0.0011 | 0.0017 | 0.0002 |
| Lungs | Pet food | 0.0013 | 0.0017 | 0.0001 |
| Mask | Skin tannery C3 | 0.0016 | 0.0017 | 0.0030 |
| Mesenteric fat | C1-C2 for disposal | 0.0000 | 0.0000 | 0.0000 |
| Muscle | Human food | 0.0011 | 0.0017 | 0.0030 |
| Muzzle | Human food | 0.0016 | 0.0017 | 0.0018 |
| Omasum | Human food | 0.0084 | 0.0017 | 0.0013 |
| Omasum fat | Fat and greaves C3 | 0.0035 | 0.0017 | 0.0002 |
| Rumen and forestomach | Human food | 0.0084 | 0.0017 | 0.0013 |
| Rumen fat | Fat and greaves C3 | 0.0035 | 0.0017 | 0.0002 |
| Sanitary seizures | C1-C2 for disposal | 0.0000 | 0.0000 | 0.0000 |
| Screening and sifting wastes | C1-C2 for disposal | 0.0000 | 0.0000 | 0.0000 |
| Small intestine | PAP C3 | 0.0084 | 0.0017 | 0.0001 |
| Spinal cord | C1-C2 for disposal | 0.0000 | 0.0000 | 0.0000 |
| Spinal cord waste | C1-C2 for disposal | 0.0000 | 0.0000 | 0.0000 |
| Spine | C1-C2 for disposal | 0.0000 | 0.0000 | 0.0000 |
| Spleen | Pet food | 0.0011 | 0.0017 | 0.0001 |
| Stillborn | PAP C3 | 0.0088 | 0.0017 | 0.0000 |
| Tallow | Fat and greaves C3 | 0.0018 | 0.0017 | 0.0002 |
| Tongue | Human food | 0.0010 | 0.0017 | 0.0028 |
| Tonsil | C1-C2 for disposal | 0.0000 | 0.0000 | 0.0000 |
| Trachea | Pet food | 0.0016 | 0.0017 | 0.0001 |
| Udder | Pet food | 0.0002 | 0.0017 | 0.0001 |
| Upper throat | Pet food | 0.0011 | 0.0017 | 0.0001 |
| Water in the rumen | Spreading/Compost | 0.0000 | 0.0000 | 0.0000 |

Table 36: Allocation factors for Primholstein Beef reared in Stall

| COPRODUCT | Destination | Primholstein/beef/stall | | |
| --- | --- | --- | --- | --- |
|  |  | **Biophysical Allocation Factor** | **Mass Allocation Factor** | **Economic Allocation Factor** |
| Abomasum | Human food | 0.0083 | 0.0016 | 0.0012 |
| Abomasum fat | Fat and greaves C3 | 0.0035 | 0.0016 | 0.0002 |
| Aponeurosis | Human food | 0.0012 | 0.0016 | 0.0017 |
| Bile | PAP C3 | 0.0004 | 0.0016 | 0.0001 |
| Blood | PAP C3 | 0.0009 | 0.0016 | 0.0004 |
| Blood | Pet food | 0.0009 | 0.0016 | 0.0001 |
| Bones | Gelatin C3 | 0.0010 | 0.0016 | 0.0000 |
| Bones of head, brain, eyes and teeth | C1-C2 for disposal | 0.0000 | 0.0000 | 0.0000 |
| Cheek | Human food | 0.0011 | 0.0016 | 0.0037 |
| Cheek | Human food | 0.0011 | 0.0016 | 0.0037 |
| Cheek trimmings | Pet food | 0.0011 | 0.0016 | 0.0001 |
| Chops | Pet food | 0.0015 | 0.0016 | 0.0001 |
| Contents of intestines | Spreading/Compost | 0.0000 | 0.0000 | 0.0000 |
| Contents of the rumen | Spreading/Compost | 0.0000 | 0.0000 | 0.0000 |
| Ears | PAP C3 | 0.0014 | 0.0016 | 0.0001 |
| Esophagus | Pet food | 0.0011 | 0.0016 | 0.0001 |
| Fat | Fat and greaves C3 | 0.0018 | 0.0016 | 0.0002 |
| Fat around heart | Fat and greaves C3 | 0.0018 | 0.0016 | 0.0002 |
| Fat in the kidney | Fat and greaves C3 | 0.0018 | 0.0016 | 0.0002 |
| Feet (without hooves) | Gelatin C3 | 0.0011 | 0.0016 | 0.0000 |
| Floatation fat | Spreading/Compost | 0.0000 | 0.0000 | 0.0000 |
| Forehead | C1-C2 for disposal | 0.0000 | 0.0000 | 0.0000 |
| Forelock | PAP C3 | 0.0039 | 0.0016 | 0.0001 |
| Gallbladder | Pet food | 0.0011 | 0.0016 | 0.0001 |
| Head trimmings | Pet food | 0.0011 | 0.0016 | 0.0001 |
| Heart | Human food | 0.0010 | 0.0016 | 0.0004 |
| Heart trimmings | Pet food | 0.0011 | 0.0016 | 0.0001 |
| Hide | Skin tannery C3 | 0.0015 | 0.0016 | 0.0028 |
| Hooves | PAP C3 | 0.0039 | 0.0016 | 0.0001 |
| Horns | PAP C3 | 0.0039 | 0.0016 | 0.0001 |
| Kidney | Human food | 0.0011 | 0.0016 | 0.0007 |
| Large intestine | C1-C2 for disposal | 0.0000 | 0.0000 | 0.0000 |
| Liver | Human food | 0.0043 | 0.0016 | 0.0008 |
| Liver trimmings | Pet food | 0.0045 | 0.0016 | 0.0001 |
| Lower jaw | PAP C3 | 0.0010 | 0.0016 | 0.0001 |
| Lungs | Pet food | 0.0012 | 0.0016 | 0.0001 |
| Mask | Skin tannery C3 | 0.0015 | 0.0016 | 0.0028 |
| Mesenteric fat | C1-C2 for disposal | 0.0000 | 0.0000 | 0.0000 |
| Muscle | Human food | 0.0011 | 0.0016 | 0.0028 |
| Muzzle | Human food | 0.0015 | 0.0016 | 0.0017 |
| Omasum | Human food | 0.0083 | 0.0016 | 0.0012 |
| Omasum fat | Fat and greaves C3 | 0.0035 | 0.0016 | 0.0002 |
| Rumen and forestomach | Human food | 0.0083 | 0.0016 | 0.0012 |
| Rumen fat | Fat and greaves C3 | 0.0035 | 0.0016 | 0.0002 |
| Sanitary seizures | C1-C2 for disposal | 0.0000 | 0.0000 | 0.0000 |
| Screening and sifting wastes | C1-C2 for disposal | 0.0000 | 0.0000 | 0.0000 |
| Small intestine | PAP C3 | 0.0083 | 0.0016 | 0.0001 |
| Spinal cord | C1-C2 for disposal | 0.0000 | 0.0000 | 0.0000 |
| Spinal cord waste | C1-C2 for disposal | 0.0000 | 0.0000 | 0.0000 |
| Spine | C1-C2 for disposal | 0.0000 | 0.0000 | 0.0000 |
| Spleen | Pet food | 0.0011 | 0.0016 | 0.0001 |
| Stillborn | PAP C3 | 0.0086 | 0.0016 | 0.0000 |
| Tallow | Fat and greaves C3 | 0.0018 | 0.0016 | 0.0002 |
| Tongue | Human food | 0.0010 | 0.0016 | 0.0027 |
| Tonsil | C1-C2 for disposal | 0.0000 | 0.0000 | 0.0000 |
| Trachea | Pet food | 0.0015 | 0.0016 | 0.0001 |
| Udder | Pet food | 0.0002 | 0.0016 | 0.0001 |
| Upper throat | Pet food | 0.0011 | 0.0016 | 0.0001 |
| Water in the rumen | Spreading/Compost | 0.0000 | 0.0000 | 0.0000 |

Table 37: Allocation factors for Limousine Young Bulls reared in Grazing Large Area

| COPRODUCT | Destination | Limousine/young bull/grazing large area | | |
| --- | --- | --- | --- | --- |
|  |  | **Biophysical Allocation Factor** | **Mass Allocation Factor** | **Economic Allocation Factor** |
| Abomasum | Human food | 0.0095 | 0.0016 | 0.0011 |
| Abomasum fat | Fat and greaves C3 | 0.0036 | 0.0016 | 0.0001 |
| Aponeurosis | Human food | 0.0013 | 0.0016 | 0.0015 |
| Bile | PAP C3 | 0.0004 | 0.0016 | 0.0001 |
| Blood | PAP C3 | 0.0010 | 0.0016 | 0.0003 |
| Blood | Pet food | 0.0010 | 0.0016 | 0.0001 |
| Bones | Gelatin C3 | 0.0010 | 0.0016 | 0.0000 |
| Bones of head, brain, eyes and teeth | C1-C2 for disposal | 0.0000 | 0.0000 | 0.0000 |
| Cheek | Human food | 0.0011 | 0.0016 | 0.0033 |
| Cheek | Human food | 0.0011 | 0.0016 | 0.0033 |
| Cheek trimmings | Pet food | 0.0011 | 0.0016 | 0.0001 |
| Chops | Pet food | 0.0016 | 0.0016 | 0.0001 |
| Contents of intestines | Spreading/Compost | 0.0000 | 0.0000 | 0.0000 |
| Contents of the rumen | Spreading/Compost | 0.0000 | 0.0000 | 0.0000 |
| Ears | PAP C3 | 0.0014 | 0.0016 | 0.0001 |
| Esophagus | Pet food | 0.0011 | 0.0016 | 0.0001 |
| Fat | Fat and greaves C3 | 0.0016 | 0.0016 | 0.0001 |
| Fat around heart | Fat and greaves C3 | 0.0016 | 0.0016 | 0.0001 |
| Fat in the kidney | Fat and greaves C3 | 0.0016 | 0.0016 | 0.0001 |
| Feet (without hooves) | Gelatin C3 | 0.0011 | 0.0016 | 0.0000 |
| Floatation fat | Spreading/Compost | 0.0000 | 0.0000 | 0.0000 |
| Forehead | C1-C2 for disposal | 0.0000 | 0.0000 | 0.0000 |
| Forelock | PAP C3 | 0.0041 | 0.0016 | 0.0001 |
| Gallbladder | Pet food | 0.0011 | 0.0016 | 0.0001 |
| Head trimmings | Pet food | 0.0011 | 0.0016 | 0.0001 |
| Heart | Human food | 0.0011 | 0.0016 | 0.0003 |
| Heart trimmings | Pet food | 0.0011 | 0.0016 | 0.0001 |
| Hide | Skin tannery C3 | 0.0016 | 0.0016 | 0.0025 |
| Hooves | PAP C3 | 0.0041 | 0.0016 | 0.0001 |
| Horns | PAP C3 | 0.0041 | 0.0016 | 0.0001 |
| Kidney | Human food | 0.0011 | 0.0016 | 0.0006 |
| Large intestine | C1-C2 for disposal | 0.0000 | 0.0000 | 0.0000 |
| Liver | Human food | 0.0049 | 0.0016 | 0.0007 |
| Liver trimmings | Pet food | 0.0051 | 0.0016 | 0.0001 |
| Lower jaw | PAP C3 | 0.0010 | 0.0016 | 0.0001 |
| Lungs | Pet food | 0.0013 | 0.0016 | 0.0001 |
| Mask | Skin tannery C3 | 0.0016 | 0.0016 | 0.0025 |
| Mesenteric fat | C1-C2 for disposal | 0.0000 | 0.0000 | 0.0000 |
| Muscle | Human food | 0.0011 | 0.0016 | 0.0025 |
| Muzzle | Human food | 0.0016 | 0.0016 | 0.0015 |
| Omasum | Human food | 0.0095 | 0.0016 | 0.0011 |
| Omasum fat | Fat and greaves C3 | 0.0036 | 0.0016 | 0.0001 |
| Rumen and forestomach | Human food | 0.0095 | 0.0016 | 0.0011 |
| Rumen fat | Fat and greaves C3 | 0.0036 | 0.0016 | 0.0001 |
| Sanitary seizures | C1-C2 for disposal | 0.0000 | 0.0000 | 0.0000 |
| Screening and sifting wastes | C1-C2 for disposal | 0.0000 | 0.0000 | 0.0000 |
| Small intestine | PAP C3 | 0.0095 | 0.0016 | 0.0001 |
| Spinal cord | C1-C2 for disposal | 0.0000 | 0.0000 | 0.0000 |
| Spinal cord waste | C1-C2 for disposal | 0.0000 | 0.0000 | 0.0000 |
| Spine | C1-C2 for disposal | 0.0000 | 0.0000 | 0.0000 |
| Spleen | Pet food | 0.0012 | 0.0016 | 0.0001 |
| Stillborn | PAP C3 | 0.0099 | 0.0016 | 0.0000 |
| Tallow | Fat and greaves C3 | 0.0016 | 0.0016 | 0.0001 |
| Tongue | Human food | 0.0010 | 0.0016 | 0.0024 |
| Tonsil | C1-C2 for disposal | 0.0000 | 0.0000 | 0.0000 |
| Trachea | Pet food | 0.0016 | 0.0016 | 0.0001 |
| Udder | Pet food | 0.0002 | 0.0016 | 0.0001 |
| Upper throat | Pet food | 0.0011 | 0.0016 | 0.0001 |
| Water in the rumen | Spreading/Compost | 0.0000 | 0.0000 | 0.0000 |

Table 38: Allocation factors for Limousine Heifers reared in Grazing Large Area

| COPRODUCT | Destination | Limousine/heifer/grazing large area | | |
| --- | --- | --- | --- | --- |
|  |  | **Biophysical Allocation Factor** | **Mass Allocation Factor** | **Economic Allocation Factor** |
| Abomasum | Human food | 0.0099 | 0.0017 | 0.0013 |
| Abomasum fat | Fat and greaves C3 | 0.0038 | 0.0017 | 0.0002 |
| Aponeurosis | Human food | 0.0013 | 0.0017 | 0.0017 |
| Bile | PAP C3 | 0.0005 | 0.0017 | 0.0001 |
| Blood | PAP C3 | 0.0010 | 0.0017 | 0.0004 |
| Blood | Pet food | 0.0010 | 0.0017 | 0.0001 |
| Bones | Gelatin C3 | 0.0011 | 0.0017 | 0.0000 |
| Bones of head, brain, eyes and teeth | C1-C2 for disposal | 0.0000 | 0.0000 | 0.0000 |
| Cheek | Human food | 0.0012 | 0.0017 | 0.0038 |
| Cheek | Human food | 0.0012 | 0.0017 | 0.0038 |
| Cheek trimmings | Pet food | 0.0012 | 0.0017 | 0.0001 |
| Chops | Pet food | 0.0017 | 0.0017 | 0.0001 |
| Contents of intestines | Spreading/Compost | 0.0000 | 0.0000 | 0.0000 |
| Contents of the rumen | Spreading/Compost | 0.0000 | 0.0000 | 0.0000 |
| Ears | PAP C3 | 0.0015 | 0.0017 | 0.0001 |
| Esophagus | Pet food | 0.0012 | 0.0017 | 0.0001 |
| Fat | Fat and greaves C3 | 0.0017 | 0.0017 | 0.0002 |
| Fat around heart | Fat and greaves C3 | 0.0017 | 0.0017 | 0.0002 |
| Fat in the kidney | Fat and greaves C3 | 0.0017 | 0.0017 | 0.0002 |
| Feet (without hooves) | Gelatin C3 | 0.0012 | 0.0017 | 0.0000 |
| Floatation fat | Spreading/Compost | 0.0000 | 0.0000 | 0.0000 |
| Forehead | C1-C2 for disposal | 0.0000 | 0.0000 | 0.0000 |
| Forelock | PAP C3 | 0.0044 | 0.0017 | 0.0001 |
| Gallbladder | Pet food | 0.0012 | 0.0017 | 0.0001 |
| Head trimmings | Pet food | 0.0012 | 0.0017 | 0.0001 |
| Heart | Human food | 0.0012 | 0.0017 | 0.0004 |
| Heart trimmings | Pet food | 0.0012 | 0.0017 | 0.0001 |
| Hide | Skin tannery C3 | 0.0017 | 0.0017 | 0.0029 |
| Hooves | PAP C3 | 0.0044 | 0.0017 | 0.0001 |
| Horns | PAP C3 | 0.0044 | 0.0017 | 0.0001 |
| Kidney | Human food | 0.0012 | 0.0017 | 0.0007 |
| Large intestine | C1-C2 for disposal | 0.0000 | 0.0000 | 0.0000 |
| Liver | Human food | 0.0052 | 0.0017 | 0.0008 |
| Liver trimmings | Pet food | 0.0054 | 0.0017 | 0.0001 |
| Lower jaw | PAP C3 | 0.0011 | 0.0017 | 0.0001 |
| Lungs | Pet food | 0.0014 | 0.0017 | 0.0001 |
| Mask | Skin tannery C3 | 0.0017 | 0.0017 | 0.0029 |
| Mesenteric fat | C1-C2 for disposal | 0.0000 | 0.0000 | 0.0000 |
| Muscle | Human food | 0.0012 | 0.0017 | 0.0029 |
| Muzzle | Human food | 0.0017 | 0.0017 | 0.0017 |
| Omasum | Human food | 0.0099 | 0.0017 | 0.0013 |
| Omasum fat | Fat and greaves C3 | 0.0038 | 0.0017 | 0.0002 |
| Rumen and forestomach | Human food | 0.0099 | 0.0017 | 0.0013 |
| Rumen fat | Fat and greaves C3 | 0.0038 | 0.0017 | 0.0002 |
| Sanitary seizures | C1-C2 for disposal | 0.0000 | 0.0000 | 0.0000 |
| Screening and sifting wastes | C1-C2 for disposal | 0.0000 | 0.0000 | 0.0000 |
| Small intestine | PAP C3 | 0.0099 | 0.0017 | 0.0001 |
| Spinal cord | C1-C2 for disposal | 0.0000 | 0.0000 | 0.0000 |
| Spinal cord waste | C1-C2 for disposal | 0.0000 | 0.0000 | 0.0000 |
| Spine | C1-C2 for disposal | 0.0000 | 0.0000 | 0.0000 |
| Spleen | Pet food | 0.0012 | 0.0017 | 0.0001 |
| Stillborn | PAP C3 | 0.0104 | 0.0017 | 0.0000 |
| Tallow | Fat and greaves C3 | 0.0017 | 0.0017 | 0.0002 |
| Tongue | Human food | 0.0011 | 0.0017 | 0.0027 |
| Tonsil | C1-C2 for disposal | 0.0000 | 0.0000 | 0.0000 |
| Trachea | Pet food | 0.0017 | 0.0017 | 0.0001 |
| Udder | Pet food | 0.0003 | 0.0017 | 0.0001 |
| Upper throat | Pet food | 0.0012 | 0.0017 | 0.0001 |
| Water in the rumen | Spreading/Compost | 0.0000 | 0.0000 | 0.0000 |

Table 39: Allocation factors for Limousine Cull Cows reared in Grazing Large Area

| COPRODUCT | Destination | Limousine/Cull cow/grazing large area | | |
| --- | --- | --- | --- | --- |
|  |  | **Biophysical Allocation Factor** | **Mass Allocation Factor** | **Economic Allocation Factor** |
| Abomasum | Human food | 0.0088 | 0.0015 | 0.0011 |
| Abomasum fat | Fat and greaves C3 | 0.0033 | 0.0015 | 0.0001 |
| Aponeurosis | Human food | 0.0011 | 0.0015 | 0.0015 |
| Bile | PAP C3 | 0.0004 | 0.0015 | 0.0001 |
| Blood | PAP C3 | 0.0009 | 0.0015 | 0.0003 |
| Blood | Pet food | 0.0009 | 0.0015 | 0.0001 |
| Bones | Gelatin C3 | 0.0009 | 0.0015 | 0.0000 |
| Bones of head, brain, eyes and teeth | C1-C2 for disposal | 0.0000 | 0.0000 | 0.0000 |
| Cheek | Human food | 0.0010 | 0.0015 | 0.0033 |
| Cheek | Human food | 0.0010 | 0.0015 | 0.0033 |
| Cheek trimmings | Pet food | 0.0010 | 0.0015 | 0.0001 |
| Chops | Pet food | 0.0014 | 0.0015 | 0.0001 |
| Contents of intestines | Spreading/Compost | 0.0000 | 0.0000 | 0.0000 |
| Contents of the rumen | Spreading/Compost | 0.0000 | 0.0000 | 0.0000 |
| Ears | PAP C3 | 0.0013 | 0.0015 | 0.0001 |
| Esophagus | Pet food | 0.0010 | 0.0015 | 0.0001 |
| Fat | Fat and greaves C3 | 0.0014 | 0.0015 | 0.0001 |
| Fat around heart | Fat and greaves C3 | 0.0014 | 0.0015 | 0.0001 |
| Fat in the kidney | Fat and greaves C3 | 0.0014 | 0.0015 | 0.0001 |
| Feet (without hooves) | Gelatin C3 | 0.0010 | 0.0015 | 0.0000 |
| Floatation fat | Spreading/Compost | 0.0000 | 0.0000 | 0.0000 |
| Forehead | C1-C2 for disposal | 0.0000 | 0.0000 | 0.0000 |
| Forelock | PAP C3 | 0.0037 | 0.0015 | 0.0001 |
| Gallbladder | Pet food | 0.0010 | 0.0015 | 0.0001 |
| Head trimmings | Pet food | 0.0010 | 0.0015 | 0.0001 |
| Heart | Human food | 0.0010 | 0.0015 | 0.0003 |
| Heart trimmings | Pet food | 0.0010 | 0.0015 | 0.0001 |
| Hide | Skin tannery C3 | 0.0014 | 0.0015 | 0.0025 |
| Hooves | PAP C3 | 0.0037 | 0.0015 | 0.0001 |
| Horns | PAP C3 | 0.0037 | 0.0015 | 0.0001 |
| Kidney | Human food | 0.0010 | 0.0015 | 0.0006 |
| Large intestine | C1-C2 for disposal | 0.0000 | 0.0000 | 0.0000 |
| Liver | Human food | 0.0045 | 0.0015 | 0.0007 |
| Liver trimmings | Pet food | 0.0047 | 0.0015 | 0.0001 |
| Lower jaw | PAP C3 | 0.0009 | 0.0015 | 0.0001 |
| Lungs | Pet food | 0.0012 | 0.0015 | 0.0001 |
| Mask | Skin tannery C3 | 0.0014 | 0.0015 | 0.0025 |
| Mesenteric fat | C1-C2 for disposal | 0.0000 | 0.0000 | 0.0000 |
| Muscle | Human food | 0.0010 | 0.0015 | 0.0025 |
| Muzzle | Human food | 0.0014 | 0.0015 | 0.0015 |
| Omasum | Human food | 0.0088 | 0.0015 | 0.0011 |
| Omasum fat | Fat and greaves C3 | 0.0033 | 0.0015 | 0.0001 |
| Rumen and forestomach | Human food | 0.0088 | 0.0015 | 0.0011 |
| Rumen fat | Fat and greaves C3 | 0.0033 | 0.0015 | 0.0001 |
| Sanitary seizures | C1-C2 for disposal | 0.0000 | 0.0000 | 0.0000 |
| Screening and sifting wastes | C1-C2 for disposal | 0.0000 | 0.0000 | 0.0000 |
| Small intestine | PAP C3 | 0.0088 | 0.0015 | 0.0001 |
| Spinal cord | C1-C2 for disposal | 0.0000 | 0.0000 | 0.0000 |
| Spinal cord waste | C1-C2 for disposal | 0.0000 | 0.0000 | 0.0000 |
| Spine | C1-C2 for disposal | 0.0000 | 0.0000 | 0.0000 |
| Spleen | Pet food | 0.0010 | 0.0015 | 0.0001 |
| Stillborn | PAP C3 | 0.0091 | 0.0015 | 0.0000 |
| Tallow | Fat and greaves C3 | 0.0014 | 0.0015 | 0.0001 |
| Tongue | Human food | 0.0009 | 0.0015 | 0.0024 |
| Tonsil | C1-C2 for disposal | 0.0000 | 0.0000 | 0.0000 |
| Trachea | Pet food | 0.0014 | 0.0015 | 0.0001 |
| Udder | Pet food | 0.0002 | 0.0015 | 0.0001 |
| Upper throat | Pet food | 0.0010 | 0.0015 | 0.0001 |
| Water in the rumen | Spreading/Compost | 0.0000 | 0.0000 | 0.0000 |

Table 40: Allocation factors for Limousine Beef reared in Grazing Large Area

| COPRODUCT | Destination | Limousine/beef/grazing large area | | |
| --- | --- | --- | --- | --- |
|  |  | **Biophysical Allocation Factor** | **Mass Allocation Factor** | **Economic Allocation Factor** |
| Abomasum | Human food | 0.0087 | 0.0014 | 0.0011 |
| Abomasum fat | Fat and greaves C3 | 0.0032 | 0.0014 | 0.0001 |
| Aponeurosis | Human food | 0.0011 | 0.0014 | 0.0014 |
| Bile | PAP C3 | 0.0004 | 0.0014 | 0.0001 |
| Blood | PAP C3 | 0.0009 | 0.0014 | 0.0003 |
| Blood | Pet food | 0.0009 | 0.0014 | 0.0001 |
| Bones | Gelatin C3 | 0.0009 | 0.0014 | 0.0000 |
| Bones of head, brain, eyes and teeth | C1-C2 for disposal | 0.0000 | 0.0000 | 0.0000 |
| Cheek | Human food | 0.0010 | 0.0014 | 0.0031 |
| Cheek | Human food | 0.0010 | 0.0014 | 0.0031 |
| Cheek trimmings | Pet food | 0.0010 | 0.0014 | 0.0001 |
| Chops | Pet food | 0.0014 | 0.0014 | 0.0001 |
| Contents of intestines | Spreading/Compost | 0.0000 | 0.0000 | 0.0000 |
| Contents of the rumen | Spreading/Compost | 0.0000 | 0.0000 | 0.0000 |
| Ears | PAP C3 | 0.0013 | 0.0014 | 0.0001 |
| Esophagus | Pet food | 0.0010 | 0.0014 | 0.0001 |
| Fat | Fat and greaves C3 | 0.0014 | 0.0014 | 0.0001 |
| Fat around heart | Fat and greaves C3 | 0.0014 | 0.0014 | 0.0001 |
| Fat in the kidney | Fat and greaves C3 | 0.0014 | 0.0014 | 0.0001 |
| Feet (without hooves) | Gelatin C3 | 0.0010 | 0.0014 | 0.0000 |
| Floatation fat | Spreading/Compost | 0.0000 | 0.0000 | 0.0000 |
| Forehead | C1-C2 for disposal | 0.0000 | 0.0000 | 0.0000 |
| Forelock | PAP C3 | 0.0037 | 0.0014 | 0.0001 |
| Gallbladder | Pet food | 0.0010 | 0.0014 | 0.0001 |
| Head trimmings | Pet food | 0.0010 | 0.0014 | 0.0001 |
| Heart | Human food | 0.0010 | 0.0014 | 0.0003 |
| Heart trimmings | Pet food | 0.0010 | 0.0014 | 0.0001 |
| Hide | Skin tannery C3 | 0.0014 | 0.0014 | 0.0024 |
| Hooves | PAP C3 | 0.0037 | 0.0014 | 0.0001 |
| Horns | PAP C3 | 0.0037 | 0.0014 | 0.0001 |
| Kidney | Human food | 0.0010 | 0.0014 | 0.0006 |
| Large intestine | C1-C2 for disposal | 0.0000 | 0.0000 | 0.0000 |
| Liver | Human food | 0.0045 | 0.0014 | 0.0007 |
| Liver trimmings | Pet food | 0.0047 | 0.0014 | 0.0001 |
| Lower jaw | PAP C3 | 0.0009 | 0.0014 | 0.0001 |
| Lungs | Pet food | 0.0012 | 0.0014 | 0.0001 |
| Mask | Skin tannery C3 | 0.0014 | 0.0014 | 0.0024 |
| Mesenteric fat | C1-C2 for disposal | 0.0000 | 0.0000 | 0.0000 |
| Muscle | Human food | 0.0010 | 0.0014 | 0.0024 |
| Muzzle | Human food | 0.0014 | 0.0014 | 0.0014 |
| Omasum | Human food | 0.0087 | 0.0014 | 0.0011 |
| Omasum fat | Fat and greaves C3 | 0.0032 | 0.0014 | 0.0001 |
| Rumen and forestomach | Human food | 0.0087 | 0.0014 | 0.0011 |
| Rumen fat | Fat and greaves C3 | 0.0032 | 0.0014 | 0.0001 |
| Sanitary seizures | C1-C2 for disposal | 0.0000 | 0.0000 | 0.0000 |
| Screening and sifting wastes | C1-C2 for disposal | 0.0000 | 0.0000 | 0.0000 |
| Small intestine | PAP C3 | 0.0087 | 0.0014 | 0.0001 |
| Spinal cord | C1-C2 for disposal | 0.0000 | 0.0000 | 0.0000 |
| Spinal cord waste | C1-C2 for disposal | 0.0000 | 0.0000 | 0.0000 |
| Spine | C1-C2 for disposal | 0.0000 | 0.0000 | 0.0000 |
| Spleen | Pet food | 0.0010 | 0.0014 | 0.0001 |
| Stillborn | PAP C3 | 0.0091 | 0.0014 | 0.0000 |
| Tallow | Fat and greaves C3 | 0.0014 | 0.0014 | 0.0001 |
| Tongue | Human food | 0.0009 | 0.0014 | 0.0023 |
| Tonsil | C1-C2 for disposal | 0.0000 | 0.0000 | 0.0000 |
| Trachea | Pet food | 0.0014 | 0.0014 | 0.0001 |
| Udder | Pet food | 0.0002 | 0.0014 | 0.0001 |
| Upper throat | Pet food | 0.0010 | 0.0014 | 0.0001 |
| Water in the rumen | Spreading/Compost | 0.0000 | 0.0000 | 0.0000 |

Table 41: Allocation factors for Limousine Young Bulls reared in Pasture

| COPRODUCT | Destination | Limousine/young bull/pasture | | |
| --- | --- | --- | --- | --- |
|  |  | **Biophysical Allocation Factor** | **Mass Allocation Factor** | **Economic Allocation Factor** |
| Abomasum | Human food | 0.0090 | 0.0016 | 0.0011 |
| Abomasum fat | Fat and greaves C3 | 0.0036 | 0.0016 | 0.0001 |
| Aponeurosis | Human food | 0.0012 | 0.0016 | 0.0015 |
| Bile | PAP C3 | 0.0004 | 0.0016 | 0.0001 |
| Blood | PAP C3 | 0.0010 | 0.0016 | 0.0003 |
| Blood | Pet food | 0.0010 | 0.0016 | 0.0001 |
| Bones | Gelatin C3 | 0.0011 | 0.0016 | 0.0000 |
| Bones of head, brain, eyes and teeth | C1-C2 for disposal | 0.0000 | 0.0000 | 0.0000 |
| Cheek | Human food | 0.0011 | 0.0016 | 0.0033 |
| Cheek | Human food | 0.0011 | 0.0016 | 0.0033 |
| Cheek trimmings | Pet food | 0.0011 | 0.0016 | 0.0001 |
| Chops | Pet food | 0.0016 | 0.0016 | 0.0001 |
| Contents of intestines | Spreading/Compost | 0.0000 | 0.0000 | 0.0000 |
| Contents of the rumen | Spreading/Compost | 0.0000 | 0.0000 | 0.0000 |
| Ears | PAP C3 | 0.0014 | 0.0016 | 0.0001 |
| Esophagus | Pet food | 0.0011 | 0.0016 | 0.0001 |
| Fat | Fat and greaves C3 | 0.0017 | 0.0016 | 0.0001 |
| Fat around heart | Fat and greaves C3 | 0.0017 | 0.0016 | 0.0001 |
| Fat in the kidney | Fat and greaves C3 | 0.0017 | 0.0016 | 0.0001 |
| Feet (without hooves) | Gelatin C3 | 0.0011 | 0.0016 | 0.0000 |
| Floatation fat | Spreading/Compost | 0.0000 | 0.0000 | 0.0000 |
| Forehead | C1-C2 for disposal | 0.0000 | 0.0000 | 0.0000 |
| Forelock | PAP C3 | 0.0040 | 0.0016 | 0.0001 |
| Gallbladder | Pet food | 0.0011 | 0.0016 | 0.0001 |
| Head trimmings | Pet food | 0.0011 | 0.0016 | 0.0001 |
| Heart | Human food | 0.0011 | 0.0016 | 0.0003 |
| Heart trimmings | Pet food | 0.0011 | 0.0016 | 0.0001 |
| Hide | Skin tannery C3 | 0.0016 | 0.0016 | 0.0025 |
| Hooves | PAP C3 | 0.0040 | 0.0016 | 0.0001 |
| Horns | PAP C3 | 0.0040 | 0.0016 | 0.0001 |
| Kidney | Human food | 0.0011 | 0.0016 | 0.0006 |
| Large intestine | C1-C2 for disposal | 0.0000 | 0.0000 | 0.0000 |
| Liver | Human food | 0.0047 | 0.0016 | 0.0007 |
| Liver trimmings | Pet food | 0.0049 | 0.0016 | 0.0001 |
| Lower jaw | PAP C3 | 0.0010 | 0.0016 | 0.0001 |
| Lungs | Pet food | 0.0013 | 0.0016 | 0.0001 |
| Mask | Skin tannery C3 | 0.0016 | 0.0016 | 0.0025 |
| Mesenteric fat | C1-C2 for disposal | 0.0000 | 0.0000 | 0.0000 |
| Muscle | Human food | 0.0011 | 0.0016 | 0.0025 |
| Muzzle | Human food | 0.0016 | 0.0016 | 0.0015 |
| Omasum | Human food | 0.0090 | 0.0016 | 0.0011 |
| Omasum fat | Fat and greaves C3 | 0.0036 | 0.0016 | 0.0001 |
| Rumen and forestomach | Human food | 0.0090 | 0.0016 | 0.0011 |
| Rumen fat | Fat and greaves C3 | 0.0036 | 0.0016 | 0.0001 |
| Sanitary seizures | C1-C2 for disposal | 0.0000 | 0.0000 | 0.0000 |
| Screening and sifting wastes | C1-C2 for disposal | 0.0000 | 0.0000 | 0.0000 |
| Small intestine | PAP C3 | 0.0090 | 0.0016 | 0.0001 |
| Spinal cord | C1-C2 for disposal | 0.0000 | 0.0000 | 0.0000 |
| Spinal cord waste | C1-C2 for disposal | 0.0000 | 0.0000 | 0.0000 |
| Spine | C1-C2 for disposal | 0.0000 | 0.0000 | 0.0000 |
| Spleen | Pet food | 0.0011 | 0.0016 | 0.0001 |
| Stillborn | PAP C3 | 0.0094 | 0.0016 | 0.0000 |
| Tallow | Fat and greaves C3 | 0.0017 | 0.0016 | 0.0001 |
| Tongue | Human food | 0.0010 | 0.0016 | 0.0024 |
| Tonsil | C1-C2 for disposal | 0.0000 | 0.0000 | 0.0000 |
| Trachea | Pet food | 0.0016 | 0.0016 | 0.0001 |
| Udder | Pet food | 0.0002 | 0.0016 | 0.0001 |
| Upper throat | Pet food | 0.0011 | 0.0016 | 0.0001 |
| Water in the rumen | Spreading/Compost | 0.0000 | 0.0000 | 0.0000 |

Table 42: Allocation factors for Limousine Heifers reared in Pasture

| COPRODUCT | Destination | Limousine/heifer/pasture | | |
| --- | --- | --- | --- | --- |
|  |  | **Biophysical Allocation Factor** | **Mass Allocation Factor** | **Economic Allocation Factor** |
| Abomasum | Human food | 0.0095 | 0.0017 | 0.0013 |
| Abomasum fat | Fat and greaves C3 | 0.0038 | 0.0017 | 0.0002 |
| Aponeurosis | Human food | 0.0013 | 0.0017 | 0.0017 |
| Bile | PAP C3 | 0.0005 | 0.0017 | 0.0001 |
| Blood | PAP C3 | 0.0010 | 0.0017 | 0.0004 |
| Blood | Pet food | 0.0010 | 0.0017 | 0.0001 |
| Bones | Gelatin C3 | 0.0011 | 0.0017 | 0.0000 |
| Bones of head, brain, eyes and teeth | C1-C2 for disposal | 0.0000 | 0.0000 | 0.0000 |
| Cheek | Human food | 0.0012 | 0.0017 | 0.0038 |
| Cheek | Human food | 0.0012 | 0.0017 | 0.0038 |
| Cheek trimmings | Pet food | 0.0012 | 0.0017 | 0.0001 |
| Chops | Pet food | 0.0017 | 0.0017 | 0.0001 |
| Contents of intestines | Spreading/Compost | 0.0000 | 0.0000 | 0.0000 |
| Contents of the rumen | Spreading/Compost | 0.0000 | 0.0000 | 0.0000 |
| Ears | PAP C3 | 0.0015 | 0.0017 | 0.0001 |
| Esophagus | Pet food | 0.0012 | 0.0017 | 0.0001 |
| Fat | Fat and greaves C3 | 0.0018 | 0.0017 | 0.0002 |
| Fat around heart | Fat and greaves C3 | 0.0018 | 0.0017 | 0.0002 |
| Fat in the kidney | Fat and greaves C3 | 0.0018 | 0.0017 | 0.0002 |
| Feet (without hooves) | Gelatin C3 | 0.0012 | 0.0017 | 0.0000 |
| Floatation fat | Spreading/Compost | 0.0000 | 0.0000 | 0.0000 |
| Forehead | C1-C2 for disposal | 0.0000 | 0.0000 | 0.0000 |
| Forelock | PAP C3 | 0.0044 | 0.0017 | 0.0001 |
| Gallbladder | Pet food | 0.0012 | 0.0017 | 0.0001 |
| Head trimmings | Pet food | 0.0012 | 0.0017 | 0.0001 |
| Heart | Human food | 0.0012 | 0.0017 | 0.0004 |
| Heart trimmings | Pet food | 0.0012 | 0.0017 | 0.0001 |
| Hide | Skin tannery C3 | 0.0017 | 0.0017 | 0.0029 |
| Hooves | PAP C3 | 0.0044 | 0.0017 | 0.0001 |
| Horns | PAP C3 | 0.0044 | 0.0017 | 0.0001 |
| Kidney | Human food | 0.0012 | 0.0017 | 0.0007 |
| Large intestine | C1-C2 for disposal | 0.0000 | 0.0000 | 0.0000 |
| Liver | Human food | 0.0050 | 0.0017 | 0.0008 |
| Liver trimmings | Pet food | 0.0052 | 0.0017 | 0.0001 |
| Lower jaw | PAP C3 | 0.0011 | 0.0017 | 0.0001 |
| Lungs | Pet food | 0.0014 | 0.0017 | 0.0001 |
| Mask | Skin tannery C3 | 0.0017 | 0.0017 | 0.0029 |
| Mesenteric fat | C1-C2 for disposal | 0.0000 | 0.0000 | 0.0000 |
| Muscle | Human food | 0.0012 | 0.0017 | 0.0029 |
| Muzzle | Human food | 0.0017 | 0.0017 | 0.0017 |
| Omasum | Human food | 0.0095 | 0.0017 | 0.0013 |
| Omasum fat | Fat and greaves C3 | 0.0038 | 0.0017 | 0.0002 |
| Rumen and forestomach | Human food | 0.0095 | 0.0017 | 0.0013 |
| Rumen fat | Fat and greaves C3 | 0.0038 | 0.0017 | 0.0002 |
| Sanitary seizures | C1-C2 for disposal | 0.0000 | 0.0000 | 0.0000 |
| Screening and sifting wastes | C1-C2 for disposal | 0.0000 | 0.0000 | 0.0000 |
| Small intestine | PAP C3 | 0.0095 | 0.0017 | 0.0001 |
| Spinal cord | C1-C2 for disposal | 0.0000 | 0.0000 | 0.0000 |
| Spinal cord waste | C1-C2 for disposal | 0.0000 | 0.0000 | 0.0000 |
| Spine | C1-C2 for disposal | 0.0000 | 0.0000 | 0.0000 |
| Spleen | Pet food | 0.0012 | 0.0017 | 0.0001 |
| Stillborn | PAP C3 | 0.0099 | 0.0017 | 0.0000 |
| Tallow | Fat and greaves C3 | 0.0018 | 0.0017 | 0.0002 |
| Tongue | Human food | 0.0011 | 0.0017 | 0.0027 |
| Tonsil | C1-C2 for disposal | 0.0000 | 0.0000 | 0.0000 |
| Trachea | Pet food | 0.0017 | 0.0017 | 0.0001 |
| Udder | Pet food | 0.0003 | 0.0017 | 0.0001 |
| Upper throat | Pet food | 0.0012 | 0.0017 | 0.0001 |
| Water in the rumen | Spreading/Compost | 0.0000 | 0.0000 | 0.0000 |

Table 43: Allocation factors for Limousine Cull Cows reared in Pasture

| COPRODUCT | Destination | Limousine/Cull cow/pasture | | |
| --- | --- | --- | --- | --- |
|  |  | **Biophysical Allocation Factor** | **Mass Allocation Factor** | **Economic Allocation Factor** |
| Abomasum | Human food | 0.0084 | 0.0015 | 0.0011 |
| Abomasum fat | Fat and greaves C3 | 0.0033 | 0.0015 | 0.0001 |
| Aponeurosis | Human food | 0.0011 | 0.0015 | 0.0015 |
| Bile | PAP C3 | 0.0004 | 0.0015 | 0.0001 |
| Blood | PAP C3 | 0.0009 | 0.0015 | 0.0003 |
| Blood | Pet food | 0.0009 | 0.0015 | 0.0001 |
| Bones | Gelatin C3 | 0.0010 | 0.0015 | 0.0000 |
| Bones of head, brain, eyes and teeth | C1-C2 for disposal | 0.0000 | 0.0000 | 0.0000 |
| Cheek | Human food | 0.0010 | 0.0015 | 0.0033 |
| Cheek | Human food | 0.0010 | 0.0015 | 0.0033 |
| Cheek trimmings | Pet food | 0.0010 | 0.0015 | 0.0001 |
| Chops | Pet food | 0.0014 | 0.0015 | 0.0001 |
| Contents of intestines | Spreading/Compost | 0.0000 | 0.0000 | 0.0000 |
| Contents of the rumen | Spreading/Compost | 0.0000 | 0.0000 | 0.0000 |
| Ears | PAP C3 | 0.0013 | 0.0015 | 0.0001 |
| Esophagus | Pet food | 0.0010 | 0.0015 | 0.0001 |
| Fat | Fat and greaves C3 | 0.0015 | 0.0015 | 0.0001 |
| Fat around heart | Fat and greaves C3 | 0.0015 | 0.0015 | 0.0001 |
| Fat in the kidney | Fat and greaves C3 | 0.0015 | 0.0015 | 0.0001 |
| Feet (without hooves) | Gelatin C3 | 0.0010 | 0.0015 | 0.0000 |
| Floatation fat | Spreading/Compost | 0.0000 | 0.0000 | 0.0000 |
| Forehead | C1-C2 for disposal | 0.0000 | 0.0000 | 0.0000 |
| Forelock | PAP C3 | 0.0037 | 0.0015 | 0.0001 |
| Gallbladder | Pet food | 0.0010 | 0.0015 | 0.0001 |
| Head trimmings | Pet food | 0.0010 | 0.0015 | 0.0001 |
| Heart | Human food | 0.0010 | 0.0015 | 0.0003 |
| Heart trimmings | Pet food | 0.0010 | 0.0015 | 0.0001 |
| Hide | Skin tannery C3 | 0.0014 | 0.0015 | 0.0025 |
| Hooves | PAP C3 | 0.0037 | 0.0015 | 0.0001 |
| Horns | PAP C3 | 0.0037 | 0.0015 | 0.0001 |
| Kidney | Human food | 0.0010 | 0.0015 | 0.0006 |
| Large intestine | C1-C2 for disposal | 0.0000 | 0.0000 | 0.0000 |
| Liver | Human food | 0.0044 | 0.0015 | 0.0007 |
| Liver trimmings | Pet food | 0.0045 | 0.0015 | 0.0001 |
| Lower jaw | PAP C3 | 0.0009 | 0.0015 | 0.0001 |
| Lungs | Pet food | 0.0012 | 0.0015 | 0.0001 |
| Mask | Skin tannery C3 | 0.0014 | 0.0015 | 0.0025 |
| Mesenteric fat | C1-C2 for disposal | 0.0000 | 0.0000 | 0.0000 |
| Muscle | Human food | 0.0010 | 0.0015 | 0.0025 |
| Muzzle | Human food | 0.0014 | 0.0015 | 0.0015 |
| Omasum | Human food | 0.0084 | 0.0015 | 0.0011 |
| Omasum fat | Fat and greaves C3 | 0.0033 | 0.0015 | 0.0001 |
| Rumen and forestomach | Human food | 0.0084 | 0.0015 | 0.0011 |
| Rumen fat | Fat and greaves C3 | 0.0033 | 0.0015 | 0.0001 |
| Sanitary seizures | C1-C2 for disposal | 0.0000 | 0.0000 | 0.0000 |
| Screening and sifting wastes | C1-C2 for disposal | 0.0000 | 0.0000 | 0.0000 |
| Small intestine | PAP C3 | 0.0084 | 0.0015 | 0.0001 |
| Spinal cord | C1-C2 for disposal | 0.0000 | 0.0000 | 0.0000 |
| Spinal cord waste | C1-C2 for disposal | 0.0000 | 0.0000 | 0.0000 |
| Spine | C1-C2 for disposal | 0.0000 | 0.0000 | 0.0000 |
| Spleen | Pet food | 0.0010 | 0.0015 | 0.0001 |
| Stillborn | PAP C3 | 0.0087 | 0.0015 | 0.0000 |
| Tallow | Fat and greaves C3 | 0.0015 | 0.0015 | 0.0001 |
| Tongue | Human food | 0.0009 | 0.0015 | 0.0024 |
| Tonsil | C1-C2 for disposal | 0.0000 | 0.0000 | 0.0000 |
| Trachea | Pet food | 0.0014 | 0.0015 | 0.0001 |
| Udder | Pet food | 0.0002 | 0.0015 | 0.0001 |
| Upper throat | Pet food | 0.0010 | 0.0015 | 0.0001 |
| Water in the rumen | Spreading/Compost | 0.0000 | 0.0000 | 0.0000 |

Table 44: Allocation factors for Limousine Beef reared in Pasture

| COPRODUCT | Destination | Limousine/beef/pasture | | |
| --- | --- | --- | --- | --- |
|  |  | **Biophysical Allocation Factor** | **Mass Allocation Factor** | **Economic Allocation Factor** |
| Abomasum | Human food | 0.0084 | 0.0014 | 0.0011 |
| Abomasum fat | Fat and greaves C3 | 0.0033 | 0.0014 | 0.0001 |
| Aponeurosis | Human food | 0.0011 | 0.0014 | 0.0014 |
| Bile | PAP C3 | 0.0004 | 0.0014 | 0.0001 |
| Blood | PAP C3 | 0.0009 | 0.0014 | 0.0003 |
| Blood | Pet food | 0.0009 | 0.0014 | 0.0001 |
| Bones | Gelatin C3 | 0.0009 | 0.0014 | 0.0000 |
| Bones of head, brain, eyes and teeth | C1-C2 for disposal | 0.0000 | 0.0000 | 0.0000 |
| Cheek | Human food | 0.0010 | 0.0014 | 0.0031 |
| Cheek | Human food | 0.0010 | 0.0014 | 0.0031 |
| Cheek trimmings | Pet food | 0.0010 | 0.0014 | 0.0001 |
| Chops | Pet food | 0.0014 | 0.0014 | 0.0001 |
| Contents of intestines | Spreading/Compost | 0.0000 | 0.0000 | 0.0000 |
| Contents of the rumen | Spreading/Compost | 0.0000 | 0.0000 | 0.0000 |
| Ears | PAP C3 | 0.0013 | 0.0014 | 0.0001 |
| Esophagus | Pet food | 0.0010 | 0.0014 | 0.0001 |
| Fat | Fat and greaves C3 | 0.0015 | 0.0014 | 0.0001 |
| Fat around heart | Fat and greaves C3 | 0.0015 | 0.0014 | 0.0001 |
| Fat in the kidney | Fat and greaves C3 | 0.0015 | 0.0014 | 0.0001 |
| Feet (without hooves) | Gelatin C3 | 0.0010 | 0.0014 | 0.0000 |
| Floatation fat | Spreading/Compost | 0.0000 | 0.0000 | 0.0000 |
| Forehead | C1-C2 for disposal | 0.0000 | 0.0000 | 0.0000 |
| Forelock | PAP C3 | 0.0036 | 0.0014 | 0.0001 |
| Gallbladder | Pet food | 0.0010 | 0.0014 | 0.0001 |
| Head trimmings | Pet food | 0.0010 | 0.0014 | 0.0001 |
| Heart | Human food | 0.0010 | 0.0014 | 0.0003 |
| Heart trimmings | Pet food | 0.0010 | 0.0014 | 0.0001 |
| Hide | Skin tannery C3 | 0.0014 | 0.0014 | 0.0024 |
| Hooves | PAP C3 | 0.0036 | 0.0014 | 0.0001 |
| Horns | PAP C3 | 0.0036 | 0.0014 | 0.0001 |
| Kidney | Human food | 0.0010 | 0.0014 | 0.0006 |
| Large intestine | C1-C2 for disposal | 0.0000 | 0.0000 | 0.0000 |
| Liver | Human food | 0.0043 | 0.0014 | 0.0007 |
| Liver trimmings | Pet food | 0.0045 | 0.0014 | 0.0001 |
| Lower jaw | PAP C3 | 0.0009 | 0.0014 | 0.0001 |
| Lungs | Pet food | 0.0012 | 0.0014 | 0.0001 |
| Mask | Skin tannery C3 | 0.0014 | 0.0014 | 0.0024 |
| Mesenteric fat | C1-C2 for disposal | 0.0000 | 0.0000 | 0.0000 |
| Muscle | Human food | 0.0010 | 0.0014 | 0.0024 |
| Muzzle | Human food | 0.0014 | 0.0014 | 0.0014 |
| Omasum | Human food | 0.0084 | 0.0014 | 0.0011 |
| Omasum fat | Fat and greaves C3 | 0.0033 | 0.0014 | 0.0001 |
| Rumen and forestomach | Human food | 0.0084 | 0.0014 | 0.0011 |
| Rumen fat | Fat and greaves C3 | 0.0033 | 0.0014 | 0.0001 |
| Sanitary seizures | C1-C2 for disposal | 0.0000 | 0.0000 | 0.0000 |
| Screening and sifting wastes | C1-C2 for disposal | 0.0000 | 0.0000 | 0.0000 |
| Small intestine | PAP C3 | 0.0084 | 0.0014 | 0.0001 |
| Spinal cord | C1-C2 for disposal | 0.0000 | 0.0000 | 0.0000 |
| Spinal cord waste | C1-C2 for disposal | 0.0000 | 0.0000 | 0.0000 |
| Spine | C1-C2 for disposal | 0.0000 | 0.0000 | 0.0000 |
| Spleen | Pet food | 0.0010 | 0.0014 | 0.0001 |
| Stillborn | PAP C3 | 0.0087 | 0.0014 | 0.0000 |
| Tallow | Fat and greaves C3 | 0.0015 | 0.0014 | 0.0001 |
| Tongue | Human food | 0.0009 | 0.0014 | 0.0023 |
| Tonsil | C1-C2 for disposal | 0.0000 | 0.0000 | 0.0000 |
| Trachea | Pet food | 0.0014 | 0.0014 | 0.0001 |
| Udder | Pet food | 0.0002 | 0.0014 | 0.0001 |
| Upper throat | Pet food | 0.0010 | 0.0014 | 0.0001 |
| Water in the rumen | Spreading/Compost | 0.0000 | 0.0000 | 0.0000 |

Table 45: Allocation factors for Limousine Young Bulls reared in Stall

| COPRODUCT | Destination | Limousine/young bull/stall | | |
| --- | --- | --- | --- | --- |
|  |  | **Biophysical Allocation Factor** | **Mass Allocation Factor** | **Economic Allocation Factor** |
| Abomasum | Human food | 0.0086 | 0.0016 | 0.0011 |
| Abomasum fat | Fat and greaves C3 | 0.0036 | 0.0016 | 0.0001 |
| Aponeurosis | Human food | 0.0012 | 0.0016 | 0.0015 |
| Bile | PAP C3 | 0.0004 | 0.0016 | 0.0001 |
| Blood | PAP C3 | 0.0009 | 0.0016 | 0.0003 |
| Blood | Pet food | 0.0009 | 0.0016 | 0.0001 |
| Bones | Gelatin C3 | 0.0011 | 0.0016 | 0.0000 |
| Bones of head, brain, eyes and teeth | C1-C2 for disposal | 0.0000 | 0.0000 | 0.0000 |
| Cheek | Human food | 0.0011 | 0.0016 | 0.0033 |
| Cheek | Human food | 0.0011 | 0.0016 | 0.0033 |
| Cheek trimmings | Pet food | 0.0011 | 0.0016 | 0.0001 |
| Chops | Pet food | 0.0016 | 0.0016 | 0.0001 |
| Contents of intestines | Spreading/Compost | 0.0000 | 0.0000 | 0.0000 |
| Contents of the rumen | Spreading/Compost | 0.0000 | 0.0000 | 0.0000 |
| Ears | PAP C3 | 0.0014 | 0.0016 | 0.0001 |
| Esophagus | Pet food | 0.0011 | 0.0016 | 0.0001 |
| Fat | Fat and greaves C3 | 0.0019 | 0.0016 | 0.0001 |
| Fat around heart | Fat and greaves C3 | 0.0019 | 0.0016 | 0.0001 |
| Fat in the kidney | Fat and greaves C3 | 0.0019 | 0.0016 | 0.0001 |
| Feet (without hooves) | Gelatin C3 | 0.0011 | 0.0016 | 0.0000 |
| Floatation fat | Spreading/Compost | 0.0000 | 0.0000 | 0.0000 |
| Forehead | C1-C2 for disposal | 0.0000 | 0.0000 | 0.0000 |
| Forelock | PAP C3 | 0.0040 | 0.0016 | 0.0001 |
| Gallbladder | Pet food | 0.0011 | 0.0016 | 0.0001 |
| Head trimmings | Pet food | 0.0011 | 0.0016 | 0.0001 |
| Heart | Human food | 0.0011 | 0.0016 | 0.0003 |
| Heart trimmings | Pet food | 0.0011 | 0.0016 | 0.0001 |
| Hide | Skin tannery C3 | 0.0016 | 0.0016 | 0.0025 |
| Hooves | PAP C3 | 0.0040 | 0.0016 | 0.0001 |
| Horns | PAP C3 | 0.0040 | 0.0016 | 0.0001 |
| Kidney | Human food | 0.0011 | 0.0016 | 0.0006 |
| Large intestine | C1-C2 for disposal | 0.0000 | 0.0000 | 0.0000 |
| Liver | Human food | 0.0045 | 0.0016 | 0.0007 |
| Liver trimmings | Pet food | 0.0047 | 0.0016 | 0.0001 |
| Lower jaw | PAP C3 | 0.0011 | 0.0016 | 0.0001 |
| Lungs | Pet food | 0.0013 | 0.0016 | 0.0001 |
| Mask | Skin tannery C3 | 0.0016 | 0.0016 | 0.0025 |
| Mesenteric fat | C1-C2 for disposal | 0.0000 | 0.0000 | 0.0000 |
| Muscle | Human food | 0.0011 | 0.0016 | 0.0025 |
| Muzzle | Human food | 0.0016 | 0.0016 | 0.0015 |
| Omasum | Human food | 0.0086 | 0.0016 | 0.0011 |
| Omasum fat | Fat and greaves C3 | 0.0036 | 0.0016 | 0.0001 |
| Rumen and forestomach | Human food | 0.0086 | 0.0016 | 0.0011 |
| Rumen fat | Fat and greaves C3 | 0.0036 | 0.0016 | 0.0001 |
| Sanitary seizures | C1-C2 for disposal | 0.0000 | 0.0000 | 0.0000 |
| Screening and sifting wastes | C1-C2 for disposal | 0.0000 | 0.0000 | 0.0000 |
| Small intestine | PAP C3 | 0.0086 | 0.0016 | 0.0001 |
| Spinal cord | C1-C2 for disposal | 0.0000 | 0.0000 | 0.0000 |
| Spinal cord waste | C1-C2 for disposal | 0.0000 | 0.0000 | 0.0000 |
| Spine | C1-C2 for disposal | 0.0000 | 0.0000 | 0.0000 |
| Spleen | Pet food | 0.0011 | 0.0016 | 0.0001 |
| Stillborn | PAP C3 | 0.0089 | 0.0016 | 0.0000 |
| Tallow | Fat and greaves C3 | 0.0019 | 0.0016 | 0.0001 |
| Tongue | Human food | 0.0010 | 0.0016 | 0.0024 |
| Tonsil | C1-C2 for disposal | 0.0000 | 0.0000 | 0.0000 |
| Trachea | Pet food | 0.0016 | 0.0016 | 0.0001 |
| Udder | Pet food | 0.0003 | 0.0016 | 0.0001 |
| Upper throat | Pet food | 0.0011 | 0.0016 | 0.0001 |
| Water in the rumen | Spreading/Compost | 0.0000 | 0.0000 | 0.0000 |

Table 46: Allocation factors for Limousine Heifers reared in Stall

| COPRODUCT | Destination | Limousine/heifer/stall | | |
| --- | --- | --- | --- | --- |
|  |  | **Biophysical Allocation Factor** | **Mass Allocation Factor** | **Economic Allocation Factor** |
| Abomasum | Human food | 0.0090 | 0.0017 | 0.0013 |
| Abomasum fat | Fat and greaves C3 | 0.0038 | 0.0017 | 0.0002 |
| Aponeurosis | Human food | 0.0013 | 0.0017 | 0.0017 |
| Bile | PAP C3 | 0.0005 | 0.0017 | 0.0001 |
| Blood | PAP C3 | 0.0010 | 0.0017 | 0.0004 |
| Blood | Pet food | 0.0010 | 0.0017 | 0.0001 |
| Bones | Gelatin C3 | 0.0012 | 0.0017 | 0.0000 |
| Bones of head, brain, eyes and teeth | C1-C2 for disposal | 0.0000 | 0.0000 | 0.0000 |
| Cheek | Human food | 0.0012 | 0.0017 | 0.0038 |
| Cheek | Human food | 0.0012 | 0.0017 | 0.0038 |
| Cheek trimmings | Pet food | 0.0012 | 0.0017 | 0.0001 |
| Chops | Pet food | 0.0017 | 0.0017 | 0.0001 |
| Contents of intestines | Spreading/Compost | 0.0000 | 0.0000 | 0.0000 |
| Contents of the rumen | Spreading/Compost | 0.0000 | 0.0000 | 0.0000 |
| Ears | PAP C3 | 0.0015 | 0.0017 | 0.0001 |
| Esophagus | Pet food | 0.0012 | 0.0017 | 0.0001 |
| Fat | Fat and greaves C3 | 0.0020 | 0.0017 | 0.0002 |
| Fat around heart | Fat and greaves C3 | 0.0020 | 0.0017 | 0.0002 |
| Fat in the kidney | Fat and greaves C3 | 0.0020 | 0.0017 | 0.0002 |
| Feet (without hooves) | Gelatin C3 | 0.0012 | 0.0017 | 0.0000 |
| Floatation fat | Spreading/Compost | 0.0000 | 0.0000 | 0.0000 |
| Forehead | C1-C2 for disposal | 0.0000 | 0.0000 | 0.0000 |
| Forelock | PAP C3 | 0.0043 | 0.0017 | 0.0001 |
| Gallbladder | Pet food | 0.0012 | 0.0017 | 0.0001 |
| Head trimmings | Pet food | 0.0012 | 0.0017 | 0.0001 |
| Heart | Human food | 0.0012 | 0.0017 | 0.0004 |
| Heart trimmings | Pet food | 0.0012 | 0.0017 | 0.0001 |
| Hide | Skin tannery C3 | 0.0017 | 0.0017 | 0.0029 |
| Hooves | PAP C3 | 0.0043 | 0.0017 | 0.0001 |
| Horns | PAP C3 | 0.0043 | 0.0017 | 0.0001 |
| Kidney | Human food | 0.0012 | 0.0017 | 0.0007 |
| Large intestine | C1-C2 for disposal | 0.0000 | 0.0000 | 0.0000 |
| Liver | Human food | 0.0047 | 0.0017 | 0.0008 |
| Liver trimmings | Pet food | 0.0049 | 0.0017 | 0.0001 |
| Lower jaw | PAP C3 | 0.0011 | 0.0017 | 0.0001 |
| Lungs | Pet food | 0.0014 | 0.0017 | 0.0001 |
| Mask | Skin tannery C3 | 0.0017 | 0.0017 | 0.0029 |
| Mesenteric fat | C1-C2 for disposal | 0.0000 | 0.0000 | 0.0000 |
| Muscle | Human food | 0.0012 | 0.0017 | 0.0029 |
| Muzzle | Human food | 0.0017 | 0.0017 | 0.0017 |
| Omasum | Human food | 0.0090 | 0.0017 | 0.0013 |
| Omasum fat | Fat and greaves C3 | 0.0038 | 0.0017 | 0.0002 |
| Rumen and forestomach | Human food | 0.0090 | 0.0017 | 0.0013 |
| Rumen fat | Fat and greaves C3 | 0.0038 | 0.0017 | 0.0002 |
| Sanitary seizures | C1-C2 for disposal | 0.0000 | 0.0000 | 0.0000 |
| Screening and sifting wastes | C1-C2 for disposal | 0.0000 | 0.0000 | 0.0000 |
| Small intestine | PAP C3 | 0.0090 | 0.0017 | 0.0001 |
| Spinal cord | C1-C2 for disposal | 0.0000 | 0.0000 | 0.0000 |
| Spinal cord waste | C1-C2 for disposal | 0.0000 | 0.0000 | 0.0000 |
| Spine | C1-C2 for disposal | 0.0000 | 0.0000 | 0.0000 |
| Spleen | Pet food | 0.0012 | 0.0017 | 0.0001 |
| Stillborn | PAP C3 | 0.0093 | 0.0017 | 0.0000 |
| Tallow | Fat and greaves C3 | 0.0020 | 0.0017 | 0.0002 |
| Tongue | Human food | 0.0011 | 0.0017 | 0.0027 |
| Tonsil | C1-C2 for disposal | 0.0000 | 0.0000 | 0.0000 |
| Trachea | Pet food | 0.0017 | 0.0017 | 0.0001 |
| Udder | Pet food | 0.0003 | 0.0017 | 0.0001 |
| Upper throat | Pet food | 0.0012 | 0.0017 | 0.0001 |
| Water in the rumen | Spreading/Compost | 0.0000 | 0.0000 | 0.0000 |

Table 47: Allocation factors for Limousine Cull Cows reared in Stall

| COPRODUCT | Destination | Limousine/Cull cow/stall | | |
| --- | --- | --- | --- | --- |
|  |  | **Biophysical Allocation Factor** | **Mass Allocation Factor** | **Economic Allocation Factor** |
| Abomasum | Human food | 0.0080 | 0.0015 | 0.0011 |
| Abomasum fat | Fat and greaves C3 | 0.0033 | 0.0015 | 0.0001 |
| Aponeurosis | Human food | 0.0011 | 0.0015 | 0.0015 |
| Bile | PAP C3 | 0.0004 | 0.0015 | 0.0001 |
| Blood | PAP C3 | 0.0009 | 0.0015 | 0.0003 |
| Blood | Pet food | 0.0009 | 0.0015 | 0.0001 |
| Bones | Gelatin C3 | 0.0010 | 0.0015 | 0.0000 |
| Bones of head, brain, eyes and teeth | C1-C2 for disposal | 0.0000 | 0.0000 | 0.0000 |
| Cheek | Human food | 0.0010 | 0.0015 | 0.0033 |
| Cheek | Human food | 0.0010 | 0.0015 | 0.0033 |
| Cheek trimmings | Pet food | 0.0010 | 0.0015 | 0.0001 |
| Chops | Pet food | 0.0014 | 0.0015 | 0.0001 |
| Contents of intestines | Spreading/Compost | 0.0000 | 0.0000 | 0.0000 |
| Contents of the rumen | Spreading/Compost | 0.0000 | 0.0000 | 0.0000 |
| Ears | PAP C3 | 0.0013 | 0.0015 | 0.0001 |
| Esophagus | Pet food | 0.0010 | 0.0015 | 0.0001 |
| Fat | Fat and greaves C3 | 0.0017 | 0.0015 | 0.0001 |
| Fat around heart | Fat and greaves C3 | 0.0017 | 0.0015 | 0.0001 |
| Fat in the kidney | Fat and greaves C3 | 0.0017 | 0.0015 | 0.0001 |
| Feet (without hooves) | Gelatin C3 | 0.0010 | 0.0015 | 0.0000 |
| Floatation fat | Spreading/Compost | 0.0000 | 0.0000 | 0.0000 |
| Forehead | C1-C2 for disposal | 0.0000 | 0.0000 | 0.0000 |
| Forelock | PAP C3 | 0.0036 | 0.0015 | 0.0001 |
| Gallbladder | Pet food | 0.0010 | 0.0015 | 0.0001 |
| Head trimmings | Pet food | 0.0010 | 0.0015 | 0.0001 |
| Heart | Human food | 0.0010 | 0.0015 | 0.0003 |
| Heart trimmings | Pet food | 0.0010 | 0.0015 | 0.0001 |
| Hide | Skin tannery C3 | 0.0014 | 0.0015 | 0.0025 |
| Hooves | PAP C3 | 0.0036 | 0.0015 | 0.0001 |
| Horns | PAP C3 | 0.0036 | 0.0015 | 0.0001 |
| Kidney | Human food | 0.0010 | 0.0015 | 0.0006 |
| Large intestine | C1-C2 for disposal | 0.0000 | 0.0000 | 0.0000 |
| Liver | Human food | 0.0042 | 0.0015 | 0.0007 |
| Liver trimmings | Pet food | 0.0043 | 0.0015 | 0.0001 |
| Lower jaw | PAP C3 | 0.0010 | 0.0015 | 0.0001 |
| Lungs | Pet food | 0.0012 | 0.0015 | 0.0001 |
| Mask | Skin tannery C3 | 0.0014 | 0.0015 | 0.0025 |
| Mesenteric fat | C1-C2 for disposal | 0.0000 | 0.0000 | 0.0000 |
| Muscle | Human food | 0.0010 | 0.0015 | 0.0025 |
| Muzzle | Human food | 0.0014 | 0.0015 | 0.0015 |
| Omasum | Human food | 0.0080 | 0.0015 | 0.0011 |
| Omasum fat | Fat and greaves C3 | 0.0033 | 0.0015 | 0.0001 |
| Rumen and forestomach | Human food | 0.0080 | 0.0015 | 0.0011 |
| Rumen fat | Fat and greaves C3 | 0.0033 | 0.0015 | 0.0001 |
| Sanitary seizures | C1-C2 for disposal | 0.0000 | 0.0000 | 0.0000 |
| Screening and sifting wastes | C1-C2 for disposal | 0.0000 | 0.0000 | 0.0000 |
| Small intestine | PAP C3 | 0.0080 | 0.0015 | 0.0001 |
| Spinal cord | C1-C2 for disposal | 0.0000 | 0.0000 | 0.0000 |
| Spinal cord waste | C1-C2 for disposal | 0.0000 | 0.0000 | 0.0000 |
| Spine | C1-C2 for disposal | 0.0000 | 0.0000 | 0.0000 |
| Spleen | Pet food | 0.0010 | 0.0015 | 0.0001 |
| Stillborn | PAP C3 | 0.0083 | 0.0015 | 0.0000 |
| Tallow | Fat and greaves C3 | 0.0017 | 0.0015 | 0.0001 |
| Tongue | Human food | 0.0009 | 0.0015 | 0.0024 |
| Tonsil | C1-C2 for disposal | 0.0000 | 0.0000 | 0.0000 |
| Trachea | Pet food | 0.0014 | 0.0015 | 0.0001 |
| Udder | Pet food | 0.0002 | 0.0015 | 0.0001 |
| Upper throat | Pet food | 0.0010 | 0.0015 | 0.0001 |
| Water in the rumen | Spreading/Compost | 0.0000 | 0.0000 | 0.0000 |

Table 48: Allocation factors for Limousine Beef reared in Stall

| COPRODUCT | Destination | Limousine/beef/stall | | |
| --- | --- | --- | --- | --- |
|  |  | **Biophysical Allocation Factor** | **Mass Allocation Factor** | **Economic Allocation Factor** |
| Abomasum | Human food | 0.0080 | 0.0014 | 0.0011 |
| Abomasum fat | Fat and greaves C3 | 0.0033 | 0.0014 | 0.0001 |
| Aponeurosis | Human food | 0.0011 | 0.0014 | 0.0014 |
| Bile | PAP C3 | 0.0004 | 0.0014 | 0.0001 |
| Blood | PAP C3 | 0.0008 | 0.0014 | 0.0003 |
| Blood | Pet food | 0.0008 | 0.0014 | 0.0001 |
| Bones | Gelatin C3 | 0.0010 | 0.0014 | 0.0000 |
| Bones of head, brain, eyes and teeth | C1-C2 for disposal | 0.0000 | 0.0000 | 0.0000 |
| Cheek | Human food | 0.0010 | 0.0014 | 0.0031 |
| Cheek | Human food | 0.0010 | 0.0014 | 0.0031 |
| Cheek trimmings | Pet food | 0.0010 | 0.0014 | 0.0001 |
| Chops | Pet food | 0.0014 | 0.0014 | 0.0001 |
| Contents of intestines | Spreading/Compost | 0.0000 | 0.0000 | 0.0000 |
| Contents of the rumen | Spreading/Compost | 0.0000 | 0.0000 | 0.0000 |
| Ears | PAP C3 | 0.0013 | 0.0014 | 0.0001 |
| Esophagus | Pet food | 0.0010 | 0.0014 | 0.0001 |
| Fat | Fat and greaves C3 | 0.0016 | 0.0014 | 0.0001 |
| Fat around heart | Fat and greaves C3 | 0.0016 | 0.0014 | 0.0001 |
| Fat in the kidney | Fat and greaves C3 | 0.0016 | 0.0014 | 0.0001 |
| Feet (without hooves) | Gelatin C3 | 0.0010 | 0.0014 | 0.0000 |
| Floatation fat | Spreading/Compost | 0.0000 | 0.0000 | 0.0000 |
| Forehead | C1-C2 for disposal | 0.0000 | 0.0000 | 0.0000 |
| Forelock | PAP C3 | 0.0036 | 0.0014 | 0.0001 |
| Gallbladder | Pet food | 0.0010 | 0.0014 | 0.0001 |
| Head trimmings | Pet food | 0.0010 | 0.0014 | 0.0001 |
| Heart | Human food | 0.0010 | 0.0014 | 0.0003 |
| Heart trimmings | Pet food | 0.0010 | 0.0014 | 0.0001 |
| Hide | Skin tannery C3 | 0.0014 | 0.0014 | 0.0024 |
| Hooves | PAP C3 | 0.0036 | 0.0014 | 0.0001 |
| Horns | PAP C3 | 0.0036 | 0.0014 | 0.0001 |
| Kidney | Human food | 0.0010 | 0.0014 | 0.0006 |
| Large intestine | C1-C2 for disposal | 0.0000 | 0.0000 | 0.0000 |
| Liver | Human food | 0.0042 | 0.0014 | 0.0007 |
| Liver trimmings | Pet food | 0.0043 | 0.0014 | 0.0001 |
| Lower jaw | PAP C3 | 0.0009 | 0.0014 | 0.0001 |
| Lungs | Pet food | 0.0011 | 0.0014 | 0.0001 |
| Mask | Skin tannery C3 | 0.0014 | 0.0014 | 0.0024 |
| Mesenteric fat | C1-C2 for disposal | 0.0000 | 0.0000 | 0.0000 |
| Muscle | Human food | 0.0010 | 0.0014 | 0.0024 |
| Muzzle | Human food | 0.0014 | 0.0014 | 0.0014 |
| Omasum | Human food | 0.0080 | 0.0014 | 0.0011 |
| Omasum fat | Fat and greaves C3 | 0.0033 | 0.0014 | 0.0001 |
| Rumen and forestomach | Human food | 0.0080 | 0.0014 | 0.0011 |
| Rumen fat | Fat and greaves C3 | 0.0033 | 0.0014 | 0.0001 |
| Sanitary seizures | C1-C2 for disposal | 0.0000 | 0.0000 | 0.0000 |
| Screening and sifting wastes | C1-C2 for disposal | 0.0000 | 0.0000 | 0.0000 |
| Small intestine | PAP C3 | 0.0080 | 0.0014 | 0.0001 |
| Spinal cord | C1-C2 for disposal | 0.0000 | 0.0000 | 0.0000 |
| Spinal cord waste | C1-C2 for disposal | 0.0000 | 0.0000 | 0.0000 |
| Spine | C1-C2 for disposal | 0.0000 | 0.0000 | 0.0000 |
| Spleen | Pet food | 0.0010 | 0.0014 | 0.0001 |
| Stillborn | PAP C3 | 0.0083 | 0.0014 | 0.0000 |
| Tallow | Fat and greaves C3 | 0.0016 | 0.0014 | 0.0001 |
| Tongue | Human food | 0.0009 | 0.0014 | 0.0023 |
| Tonsil | C1-C2 for disposal | 0.0000 | 0.0000 | 0.0000 |
| Trachea | Pet food | 0.0014 | 0.0014 | 0.0001 |
| Udder | Pet food | 0.0002 | 0.0014 | 0.0001 |
| Upper throat | Pet food | 0.0010 | 0.0014 | 0.0001 |
| Water in the rumen | Spreading/Compost | 0.0000 | 0.0000 | 0.0000 |

Table 49: Allocation factors for Blonde d’Aquitaine Young Bulls reared in Grazing Large Area

| COPRODUCT | Destination | Blonde d’Aquitaine/young bull/grazing large area | | |
| --- | --- | --- | --- | --- |
|  |  | **Biophysical Allocation Factor** | **Mass Allocation Factor** | **Economic Allocation Factor** |
| Abomasum | Human food | 0.0092 | 0.0015 | 0.0011 |
| Abomasum fat | Fat and greaves C3 | 0.0035 | 0.0015 | 0.0001 |
| Aponeurosis | Human food | 0.0012 | 0.0015 | 0.0014 |
| Bile | PAP C3 | 0.0004 | 0.0015 | 0.0001 |
| Blood | PAP C3 | 0.0009 | 0.0015 | 0.0003 |
| Blood | Pet food | 0.0009 | 0.0015 | 0.0001 |
| Bones | Gelatin C3 | 0.0010 | 0.0015 | 0.0000 |
| Bones of head, brain, eyes and teeth | C1-C2 for disposal | 0.0000 | 0.0000 | 0.0000 |
| Cheek | Human food | 0.0011 | 0.0015 | 0.0031 |
| Cheek | Human food | 0.0011 | 0.0015 | 0.0031 |
| Cheek trimmings | Pet food | 0.0011 | 0.0015 | 0.0001 |
| Chops | Pet food | 0.0015 | 0.0015 | 0.0001 |
| Contents of intestines | Spreading/Compost | 0.0000 | 0.0000 | 0.0000 |
| Contents of the rumen | Spreading/Compost | 0.0000 | 0.0000 | 0.0000 |
| Ears | PAP C3 | 0.0014 | 0.0015 | 0.0001 |
| Esophagus | Pet food | 0.0011 | 0.0015 | 0.0001 |
| Fat | Fat and greaves C3 | 0.0015 | 0.0015 | 0.0001 |
| Fat around heart | Fat and greaves C3 | 0.0015 | 0.0015 | 0.0001 |
| Fat in the kidney | Fat and greaves C3 | 0.0015 | 0.0015 | 0.0001 |
| Feet (without hooves) | Gelatin C3 | 0.0011 | 0.0015 | 0.0000 |
| Floatation fat | Spreading/Compost | 0.0000 | 0.0000 | 0.0000 |
| Forehead | C1-C2 for disposal | 0.0000 | 0.0000 | 0.0000 |
| Forelock | PAP C3 | 0.0039 | 0.0015 | 0.0001 |
| Gallbladder | Pet food | 0.0011 | 0.0015 | 0.0001 |
| Head trimmings | Pet food | 0.0011 | 0.0015 | 0.0001 |
| Heart | Human food | 0.0010 | 0.0015 | 0.0003 |
| Heart trimmings | Pet food | 0.0011 | 0.0015 | 0.0001 |
| Hide | Skin tannery C3 | 0.0015 | 0.0015 | 0.0024 |
| Hooves | PAP C3 | 0.0039 | 0.0015 | 0.0001 |
| Horns | PAP C3 | 0.0039 | 0.0015 | 0.0001 |
| Kidney | Human food | 0.0011 | 0.0015 | 0.0006 |
| Large intestine | C1-C2 for disposal | 0.0000 | 0.0000 | 0.0000 |
| Liver | Human food | 0.0048 | 0.0015 | 0.0007 |
| Liver trimmings | Pet food | 0.0050 | 0.0015 | 0.0001 |
| Lower jaw | PAP C3 | 0.0010 | 0.0015 | 0.0001 |
| Lungs | Pet food | 0.0013 | 0.0015 | 0.0001 |
| Mask | Skin tannery C3 | 0.0015 | 0.0015 | 0.0024 |
| Mesenteric fat | C1-C2 for disposal | 0.0000 | 0.0000 | 0.0000 |
| Muscle | Human food | 0.0011 | 0.0015 | 0.0024 |
| Muzzle | Human food | 0.0015 | 0.0015 | 0.0014 |
| Omasum | Human food | 0.0092 | 0.0015 | 0.0011 |
| Omasum fat | Fat and greaves C3 | 0.0035 | 0.0015 | 0.0001 |
| Rumen and forestomach | Human food | 0.0092 | 0.0015 | 0.0011 |
| Rumen fat | Fat and greaves C3 | 0.0035 | 0.0015 | 0.0001 |
| Sanitary seizures | C1-C2 for disposal | 0.0000 | 0.0000 | 0.0000 |
| Screening and sifting wastes | C1-C2 for disposal | 0.0000 | 0.0000 | 0.0000 |
| Small intestine | PAP C3 | 0.0092 | 0.0015 | 0.0001 |
| Spinal cord | C1-C2 for disposal | 0.0000 | 0.0000 | 0.0000 |
| Spinal cord waste | C1-C2 for disposal | 0.0000 | 0.0000 | 0.0000 |
| Spine | C1-C2 for disposal | 0.0000 | 0.0000 | 0.0000 |
| Spleen | Pet food | 0.0011 | 0.0015 | 0.0001 |
| Stillborn | PAP C3 | 0.0096 | 0.0015 | 0.0000 |
| Tallow | Fat and greaves C3 | 0.0015 | 0.0015 | 0.0001 |
| Tongue | Human food | 0.0010 | 0.0015 | 0.0023 |
| Tonsil | C1-C2 for disposal | 0.0000 | 0.0000 | 0.0000 |
| Trachea | Pet food | 0.0015 | 0.0015 | 0.0001 |
| Udder | Pet food | 0.0002 | 0.0015 | 0.0001 |
| Upper throat | Pet food | 0.0011 | 0.0015 | 0.0001 |
| Water in the rumen | Spreading/Compost | 0.0000 | 0.0000 | 0.0000 |

Table 50: Allocation factors for Blonde d’Aquitaine Heifers reared in Grazing Large Area

| COPRODUCT | Destination | Blonde d’Aquitaine/heifer/grazing large area | | |
| --- | --- | --- | --- | --- |
|  |  | **Biophysical Allocation Factor** | **Mass Allocation Factor** | **Economic Allocation Factor** |
| Abomasum | Human food | 0.0088 | 0.0014 | 0.0010 |
| Abomasum fat | Fat and greaves C3 | 0.0032 | 0.0014 | 0.0001 |
| Aponeurosis | Human food | 0.0011 | 0.0014 | 0.0014 |
| Bile | PAP C3 | 0.0004 | 0.0014 | 0.0001 |
| Blood | PAP C3 | 0.0009 | 0.0014 | 0.0003 |
| Blood | Pet food | 0.0009 | 0.0014 | 0.0001 |
| Bones | Gelatin C3 | 0.0009 | 0.0014 | 0.0000 |
| Bones of head, brain, eyes and teeth | C1-C2 for disposal | 0.0000 | 0.0000 | 0.0000 |
| Cheek | Human food | 0.0010 | 0.0014 | 0.0031 |
| Cheek | Human food | 0.0010 | 0.0014 | 0.0031 |
| Cheek trimmings | Pet food | 0.0010 | 0.0014 | 0.0001 |
| Chops | Pet food | 0.0014 | 0.0014 | 0.0001 |
| Contents of intestines | Spreading/Compost | 0.0000 | 0.0000 | 0.0000 |
| Contents of the rumen | Spreading/Compost | 0.0000 | 0.0000 | 0.0000 |
| Ears | PAP C3 | 0.0013 | 0.0014 | 0.0001 |
| Esophagus | Pet food | 0.0010 | 0.0014 | 0.0001 |
| Fat | Fat and greaves C3 | 0.0014 | 0.0014 | 0.0001 |
| Fat around heart | Fat and greaves C3 | 0.0014 | 0.0014 | 0.0001 |
| Fat in the kidney | Fat and greaves C3 | 0.0014 | 0.0014 | 0.0001 |
| Feet (without hooves) | Gelatin C3 | 0.0010 | 0.0014 | 0.0000 |
| Floatation fat | Spreading/Compost | 0.0000 | 0.0000 | 0.0000 |
| Forehead | C1-C2 for disposal | 0.0000 | 0.0000 | 0.0000 |
| Forelock | PAP C3 | 0.0037 | 0.0014 | 0.0001 |
| Gallbladder | Pet food | 0.0010 | 0.0014 | 0.0001 |
| Head trimmings | Pet food | 0.0010 | 0.0014 | 0.0001 |
| Heart | Human food | 0.0010 | 0.0014 | 0.0003 |
| Heart trimmings | Pet food | 0.0010 | 0.0014 | 0.0001 |
| Hide | Skin tannery C3 | 0.0014 | 0.0014 | 0.0023 |
| Hooves | PAP C3 | 0.0037 | 0.0014 | 0.0001 |
| Horns | PAP C3 | 0.0037 | 0.0014 | 0.0001 |
| Kidney | Human food | 0.0010 | 0.0014 | 0.0006 |
| Large intestine | C1-C2 for disposal | 0.0000 | 0.0000 | 0.0000 |
| Liver | Human food | 0.0045 | 0.0014 | 0.0007 |
| Liver trimmings | Pet food | 0.0047 | 0.0014 | 0.0001 |
| Lower jaw | PAP C3 | 0.0009 | 0.0014 | 0.0001 |
| Lungs | Pet food | 0.0012 | 0.0014 | 0.0001 |
| Mask | Skin tannery C3 | 0.0014 | 0.0014 | 0.0023 |
| Mesenteric fat | C1-C2 for disposal | 0.0000 | 0.0000 | 0.0000 |
| Muscle | Human food | 0.0010 | 0.0014 | 0.0023 |
| Muzzle | Human food | 0.0014 | 0.0014 | 0.0014 |
| Omasum | Human food | 0.0088 | 0.0014 | 0.0010 |
| Omasum fat | Fat and greaves C3 | 0.0032 | 0.0014 | 0.0001 |
| Rumen and forestomach | Human food | 0.0088 | 0.0014 | 0.0010 |
| Rumen fat | Fat and greaves C3 | 0.0032 | 0.0014 | 0.0001 |
| Sanitary seizures | C1-C2 for disposal | 0.0000 | 0.0000 | 0.0000 |
| Screening and sifting wastes | C1-C2 for disposal | 0.0000 | 0.0000 | 0.0000 |
| Small intestine | PAP C3 | 0.0088 | 0.0014 | 0.0001 |
| Spinal cord | C1-C2 for disposal | 0.0000 | 0.0000 | 0.0000 |
| Spinal cord waste | C1-C2 for disposal | 0.0000 | 0.0000 | 0.0000 |
| Spine | C1-C2 for disposal | 0.0000 | 0.0000 | 0.0000 |
| Spleen | Pet food | 0.0010 | 0.0014 | 0.0001 |
| Stillborn | PAP C3 | 0.0091 | 0.0014 | 0.0000 |
| Tallow | Fat and greaves C3 | 0.0014 | 0.0014 | 0.0001 |
| Tongue | Human food | 0.0009 | 0.0014 | 0.0022 |
| Tonsil | C1-C2 for disposal | 0.0000 | 0.0000 | 0.0000 |
| Trachea | Pet food | 0.0014 | 0.0014 | 0.0001 |
| Udder | Pet food | 0.0002 | 0.0014 | 0.0001 |
| Upper throat | Pet food | 0.0010 | 0.0014 | 0.0001 |
| Water in the rumen | Spreading/Compost | 0.0000 | 0.0000 | 0.0000 |

Table 51: Allocation factors for Blonde d’Aquitaine Cull Cows reared in Grazing Large Area

| COPRODUCT | Destination | Blonde d’Aquitaine/Cull cow/grazing large area | | |
| --- | --- | --- | --- | --- |
|  |  | **Biophysical Allocation Factor** | **Mass Allocation Factor** | **Economic Allocation Factor** |
| Abomasum | Human food | 0.0076 | 0.0012 | 0.0009 |
| Abomasum fat | Fat and greaves C3 | 0.0025 | 0.0012 | 0.0001 |
| Aponeurosis | Human food | 0.0009 | 0.0012 | 0.0012 |
| Bile | PAP C3 | 0.0003 | 0.0012 | 0.0001 |
| Blood | PAP C3 | 0.0007 | 0.0012 | 0.0003 |
| Blood | Pet food | 0.0007 | 0.0012 | 0.0001 |
| Bones | Gelatin C3 | 0.0007 | 0.0012 | 0.0000 |
| Bones of head, brain, eyes and teeth | C1-C2 for disposal | 0.0000 | 0.0000 | 0.0000 |
| Cheek | Human food | 0.0008 | 0.0012 | 0.0027 |
| Cheek | Human food | 0.0008 | 0.0012 | 0.0027 |
| Cheek trimmings | Pet food | 0.0008 | 0.0012 | 0.0001 |
| Chops | Pet food | 0.0011 | 0.0012 | 0.0001 |
| Contents of intestines | Spreading/Compost | 0.0000 | 0.0000 | 0.0000 |
| Contents of the rumen | Spreading/Compost | 0.0000 | 0.0000 | 0.0000 |
| Ears | PAP C3 | 0.0010 | 0.0012 | 0.0001 |
| Esophagus | Pet food | 0.0008 | 0.0012 | 0.0001 |
| Fat | Fat and greaves C3 | 0.0009 | 0.0012 | 0.0001 |
| Fat around heart | Fat and greaves C3 | 0.0009 | 0.0012 | 0.0001 |
| Fat in the kidney | Fat and greaves C3 | 0.0009 | 0.0012 | 0.0001 |
| Feet (without hooves) | Gelatin C3 | 0.0008 | 0.0012 | 0.0000 |
| Floatation fat | Spreading/Compost | 0.0000 | 0.0000 | 0.0000 |
| Forehead | C1-C2 for disposal | 0.0000 | 0.0000 | 0.0000 |
| Forelock | PAP C3 | 0.0029 | 0.0012 | 0.0001 |
| Gallbladder | Pet food | 0.0008 | 0.0012 | 0.0001 |
| Head trimmings | Pet food | 0.0008 | 0.0012 | 0.0001 |
| Heart | Human food | 0.0008 | 0.0012 | 0.0003 |
| Heart trimmings | Pet food | 0.0008 | 0.0012 | 0.0001 |
| Hide | Skin tannery C3 | 0.0011 | 0.0012 | 0.0020 |
| Hooves | PAP C3 | 0.0029 | 0.0012 | 0.0001 |
| Horns | PAP C3 | 0.0029 | 0.0012 | 0.0001 |
| Kidney | Human food | 0.0008 | 0.0012 | 0.0005 |
| Large intestine | C1-C2 for disposal | 0.0000 | 0.0000 | 0.0000 |
| Liver | Human food | 0.0039 | 0.0012 | 0.0006 |
| Liver trimmings | Pet food | 0.0040 | 0.0012 | 0.0001 |
| Lower jaw | PAP C3 | 0.0007 | 0.0012 | 0.0001 |
| Lungs | Pet food | 0.0009 | 0.0012 | 0.0001 |
| Mask | Skin tannery C3 | 0.0011 | 0.0012 | 0.0020 |
| Mesenteric fat | C1-C2 for disposal | 0.0000 | 0.0000 | 0.0000 |
| Muscle | Human food | 0.0008 | 0.0012 | 0.0020 |
| Muzzle | Human food | 0.0011 | 0.0012 | 0.0012 |
| Omasum | Human food | 0.0076 | 0.0012 | 0.0009 |
| Omasum fat | Fat and greaves C3 | 0.0025 | 0.0012 | 0.0001 |
| Rumen and forestomach | Human food | 0.0076 | 0.0012 | 0.0009 |
| Rumen fat | Fat and greaves C3 | 0.0025 | 0.0012 | 0.0001 |
| Sanitary seizures | C1-C2 for disposal | 0.0000 | 0.0000 | 0.0000 |
| Screening and sifting wastes | C1-C2 for disposal | 0.0000 | 0.0000 | 0.0000 |
| Small intestine | PAP C3 | 0.0076 | 0.0012 | 0.0001 |
| Spinal cord | C1-C2 for disposal | 0.0000 | 0.0000 | 0.0000 |
| Spinal cord waste | C1-C2 for disposal | 0.0000 | 0.0000 | 0.0000 |
| Spine | C1-C2 for disposal | 0.0000 | 0.0000 | 0.0000 |
| Spleen | Pet food | 0.0008 | 0.0012 | 0.0001 |
| Stillborn | PAP C3 | 0.0079 | 0.0012 | 0.0000 |
| Tallow | Fat and greaves C3 | 0.0009 | 0.0012 | 0.0001 |
| Tongue | Human food | 0.0007 | 0.0012 | 0.0019 |
| Tonsil | C1-C2 for disposal | 0.0000 | 0.0000 | 0.0000 |
| Trachea | Pet food | 0.0011 | 0.0012 | 0.0001 |
| Udder | Pet food | 0.0002 | 0.0012 | 0.0001 |
| Upper throat | Pet food | 0.0008 | 0.0012 | 0.0001 |
| Water in the rumen | Spreading/Compost | 0.0000 | 0.0000 | 0.0000 |

Table 52: Allocation factors for Blonde d’Aquitaine Beef reared in Grazing Large Area

| COPRODUCT | Destination | Blonde d’Aquitaine/beef/grazing large area | | |
| --- | --- | --- | --- | --- |
|  |  | **Biophysical Allocation Factor** | **Mass Allocation Factor** | **Economic Allocation Factor** |
| Abomasum | Human food | 0.0083 | 0.0013 | 0.0009 |
| Abomasum fat | Fat and greaves C3 | 0.0030 | 0.0013 | 0.0001 |
| Aponeurosis | Human food | 0.0010 | 0.0013 | 0.0013 |
| Bile | PAP C3 | 0.0004 | 0.0013 | 0.0001 |
| Blood | PAP C3 | 0.0008 | 0.0013 | 0.0003 |
| Blood | Pet food | 0.0008 | 0.0013 | 0.0001 |
| Bones | Gelatin C3 | 0.0008 | 0.0013 | 0.0000 |
| Bones of head, brain, eyes and teeth | C1-C2 for disposal | 0.0000 | 0.0000 | 0.0000 |
| Cheek | Human food | 0.0009 | 0.0013 | 0.0028 |
| Cheek | Human food | 0.0009 | 0.0013 | 0.0028 |
| Cheek trimmings | Pet food | 0.0009 | 0.0013 | 0.0001 |
| Chops | Pet food | 0.0013 | 0.0013 | 0.0001 |
| Contents of intestines | Spreading/Compost | 0.0000 | 0.0000 | 0.0000 |
| Contents of the rumen | Spreading/Compost | 0.0000 | 0.0000 | 0.0000 |
| Ears | PAP C3 | 0.0011 | 0.0013 | 0.0001 |
| Esophagus | Pet food | 0.0009 | 0.0013 | 0.0001 |
| Fat | Fat and greaves C3 | 0.0012 | 0.0013 | 0.0001 |
| Fat around heart | Fat and greaves C3 | 0.0012 | 0.0013 | 0.0001 |
| Fat in the kidney | Fat and greaves C3 | 0.0012 | 0.0013 | 0.0001 |
| Feet (without hooves) | Gelatin C3 | 0.0009 | 0.0013 | 0.0000 |
| Floatation fat | Spreading/Compost | 0.0000 | 0.0000 | 0.0000 |
| Forehead | C1-C2 for disposal | 0.0000 | 0.0000 | 0.0000 |
| Forelock | PAP C3 | 0.0033 | 0.0013 | 0.0001 |
| Gallbladder | Pet food | 0.0009 | 0.0013 | 0.0001 |
| Head trimmings | Pet food | 0.0009 | 0.0013 | 0.0001 |
| Heart | Human food | 0.0009 | 0.0013 | 0.0003 |
| Heart trimmings | Pet food | 0.0009 | 0.0013 | 0.0001 |
| Hide | Skin tannery C3 | 0.0013 | 0.0013 | 0.0021 |
| Hooves | PAP C3 | 0.0033 | 0.0013 | 0.0001 |
| Horns | PAP C3 | 0.0033 | 0.0013 | 0.0001 |
| Kidney | Human food | 0.0009 | 0.0013 | 0.0005 |
| Large intestine | C1-C2 for disposal | 0.0000 | 0.0000 | 0.0000 |
| Liver | Human food | 0.0042 | 0.0013 | 0.0006 |
| Liver trimmings | Pet food | 0.0044 | 0.0013 | 0.0001 |
| Lower jaw | PAP C3 | 0.0008 | 0.0013 | 0.0001 |
| Lungs | Pet food | 0.0011 | 0.0013 | 0.0001 |
| Mask | Skin tannery C3 | 0.0013 | 0.0013 | 0.0021 |
| Mesenteric fat | C1-C2 for disposal | 0.0000 | 0.0000 | 0.0000 |
| Muscle | Human food | 0.0009 | 0.0013 | 0.0021 |
| Muzzle | Human food | 0.0013 | 0.0013 | 0.0013 |
| Omasum | Human food | 0.0083 | 0.0013 | 0.0009 |
| Omasum fat | Fat and greaves C3 | 0.0030 | 0.0013 | 0.0001 |
| Rumen and forestomach | Human food | 0.0083 | 0.0013 | 0.0009 |
| Rumen fat | Fat and greaves C3 | 0.0030 | 0.0013 | 0.0001 |
| Sanitary seizures | C1-C2 for disposal | 0.0000 | 0.0000 | 0.0000 |
| Screening and sifting wastes | C1-C2 for disposal | 0.0000 | 0.0000 | 0.0000 |
| Small intestine | PAP C3 | 0.0083 | 0.0013 | 0.0001 |
| Spinal cord | C1-C2 for disposal | 0.0000 | 0.0000 | 0.0000 |
| Spinal cord waste | C1-C2 for disposal | 0.0000 | 0.0000 | 0.0000 |
| Spine | C1-C2 for disposal | 0.0000 | 0.0000 | 0.0000 |
| Spleen | Pet food | 0.0009 | 0.0013 | 0.0001 |
| Stillborn | PAP C3 | 0.0086 | 0.0013 | 0.0000 |
| Tallow | Fat and greaves C3 | 0.0012 | 0.0013 | 0.0001 |
| Tongue | Human food | 0.0008 | 0.0013 | 0.0020 |
| Tonsil | C1-C2 for disposal | 0.0000 | 0.0000 | 0.0000 |
| Trachea | Pet food | 0.0013 | 0.0013 | 0.0001 |
| Udder | Pet food | 0.0002 | 0.0013 | 0.0001 |
| Upper throat | Pet food | 0.0009 | 0.0013 | 0.0001 |
| Water in the rumen | Spreading/Compost | 0.0000 | 0.0000 | 0.0000 |

Table 53: Allocation factors for Blonde d’Aquitaine Young Bulls reared in Pasture

| COPRODUCT | Destination | Blonde d’Aquitaine/young bull/pasture | | |
| --- | --- | --- | --- | --- |
|  |  | **Biophysical Allocation Factor** | **Mass Allocation Factor** | **Economic Allocation Factor** |
| Abomasum | Human food | 0.0088 | 0.0015 | 0.0011 |
| Abomasum fat | Fat and greaves C3 | 0.0035 | 0.0015 | 0.0001 |
| Aponeurosis | Human food | 0.0012 | 0.0015 | 0.0014 |
| Bile | PAP C3 | 0.0004 | 0.0015 | 0.0001 |
| Blood | PAP C3 | 0.0009 | 0.0015 | 0.0003 |
| Blood | Pet food | 0.0009 | 0.0015 | 0.0001 |
| Bones | Gelatin C3 | 0.0010 | 0.0015 | 0.0000 |
| Bones of head, brain, eyes and teeth | C1-C2 for disposal | 0.0000 | 0.0000 | 0.0000 |
| Cheek | Human food | 0.0011 | 0.0015 | 0.0031 |
| Cheek | Human food | 0.0011 | 0.0015 | 0.0031 |
| Cheek trimmings | Pet food | 0.0011 | 0.0015 | 0.0001 |
| Chops | Pet food | 0.0015 | 0.0015 | 0.0001 |
| Contents of intestines | Spreading/Compost | 0.0000 | 0.0000 | 0.0000 |
| Contents of the rumen | Spreading/Compost | 0.0000 | 0.0000 | 0.0000 |
| Ears | PAP C3 | 0.0014 | 0.0015 | 0.0001 |
| Esophagus | Pet food | 0.0010 | 0.0015 | 0.0001 |
| Fat | Fat and greaves C3 | 0.0016 | 0.0015 | 0.0001 |
| Fat around heart | Fat and greaves C3 | 0.0016 | 0.0015 | 0.0001 |
| Fat in the kidney | Fat and greaves C3 | 0.0016 | 0.0015 | 0.0001 |
| Feet (without hooves) | Gelatin C3 | 0.0011 | 0.0015 | 0.0000 |
| Floatation fat | Spreading/Compost | 0.0000 | 0.0000 | 0.0000 |
| Forehead | C1-C2 for disposal | 0.0000 | 0.0000 | 0.0000 |
| Forelock | PAP C3 | 0.0039 | 0.0015 | 0.0001 |
| Gallbladder | Pet food | 0.0011 | 0.0015 | 0.0001 |
| Head trimmings | Pet food | 0.0011 | 0.0015 | 0.0001 |
| Heart | Human food | 0.0010 | 0.0015 | 0.0003 |
| Heart trimmings | Pet food | 0.0011 | 0.0015 | 0.0001 |
| Hide | Skin tannery C3 | 0.0015 | 0.0015 | 0.0024 |
| Hooves | PAP C3 | 0.0039 | 0.0015 | 0.0001 |
| Horns | PAP C3 | 0.0039 | 0.0015 | 0.0001 |
| Kidney | Human food | 0.0011 | 0.0015 | 0.0006 |
| Large intestine | C1-C2 for disposal | 0.0000 | 0.0000 | 0.0000 |
| Liver | Human food | 0.0046 | 0.0015 | 0.0007 |
| Liver trimmings | Pet food | 0.0048 | 0.0015 | 0.0001 |
| Lower jaw | PAP C3 | 0.0010 | 0.0015 | 0.0001 |
| Lungs | Pet food | 0.0012 | 0.0015 | 0.0001 |
| Mask | Skin tannery C3 | 0.0015 | 0.0015 | 0.0024 |
| Mesenteric fat | C1-C2 for disposal | 0.0000 | 0.0000 | 0.0000 |
| Muscle | Human food | 0.0011 | 0.0015 | 0.0024 |
| Muzzle | Human food | 0.0015 | 0.0015 | 0.0014 |
| Omasum | Human food | 0.0088 | 0.0015 | 0.0011 |
| Omasum fat | Fat and greaves C3 | 0.0035 | 0.0015 | 0.0001 |
| Rumen and forestomach | Human food | 0.0088 | 0.0015 | 0.0011 |
| Rumen fat | Fat and greaves C3 | 0.0035 | 0.0015 | 0.0001 |
| Sanitary seizures | C1-C2 for disposal | 0.0000 | 0.0000 | 0.0000 |
| Screening and sifting wastes | C1-C2 for disposal | 0.0000 | 0.0000 | 0.0000 |
| Small intestine | PAP C3 | 0.0088 | 0.0015 | 0.0001 |
| Spinal cord | C1-C2 for disposal | 0.0000 | 0.0000 | 0.0000 |
| Spinal cord waste | C1-C2 for disposal | 0.0000 | 0.0000 | 0.0000 |
| Spine | C1-C2 for disposal | 0.0000 | 0.0000 | 0.0000 |
| Spleen | Pet food | 0.0011 | 0.0015 | 0.0001 |
| Stillborn | PAP C3 | 0.0092 | 0.0015 | 0.0000 |
| Tallow | Fat and greaves C3 | 0.0016 | 0.0015 | 0.0001 |
| Tongue | Human food | 0.0010 | 0.0015 | 0.0023 |
| Tonsil | C1-C2 for disposal | 0.0000 | 0.0000 | 0.0000 |
| Trachea | Pet food | 0.0015 | 0.0015 | 0.0001 |
| Udder | Pet food | 0.0002 | 0.0015 | 0.0001 |
| Upper throat | Pet food | 0.0011 | 0.0015 | 0.0001 |
| Water in the rumen | Spreading/Compost | 0.0000 | 0.0000 | 0.0000 |

Table 54: Allocation factors for Blonde d’Aquitaine Heifers reared in Pasture

| COPRODUCT | Destination | Blonde d’Aquitaine/heifer/pasture | | |
| --- | --- | --- | --- | --- |
|  |  | **Biophysical Allocation Factor** | **Mass Allocation Factor** | **Economic Allocation Factor** |
| Abomasum | Human food | 0.0084 | 0.0014 | 0.0010 |
| Abomasum fat | Fat and greaves C3 | 0.0033 | 0.0014 | 0.0001 |
| Aponeurosis | Human food | 0.0011 | 0.0014 | 0.0014 |
| Bile | PAP C3 | 0.0004 | 0.0014 | 0.0001 |
| Blood | PAP C3 | 0.0009 | 0.0014 | 0.0003 |
| Blood | Pet food | 0.0009 | 0.0014 | 0.0001 |
| Bones | Gelatin C3 | 0.0009 | 0.0014 | 0.0000 |
| Bones of head, brain, eyes and teeth | C1-C2 for disposal | 0.0000 | 0.0000 | 0.0000 |
| Cheek | Human food | 0.0010 | 0.0014 | 0.0031 |
| Cheek | Human food | 0.0010 | 0.0014 | 0.0031 |
| Cheek trimmings | Pet food | 0.0010 | 0.0014 | 0.0001 |
| Chops | Pet food | 0.0014 | 0.0014 | 0.0001 |
| Contents of intestines | Spreading/Compost | 0.0000 | 0.0000 | 0.0000 |
| Contents of the rumen | Spreading/Compost | 0.0000 | 0.0000 | 0.0000 |
| Ears | PAP C3 | 0.0013 | 0.0014 | 0.0001 |
| Esophagus | Pet food | 0.0010 | 0.0014 | 0.0001 |
| Fat | Fat and greaves C3 | 0.0015 | 0.0014 | 0.0001 |
| Fat around heart | Fat and greaves C3 | 0.0015 | 0.0014 | 0.0001 |
| Fat in the kidney | Fat and greaves C3 | 0.0015 | 0.0014 | 0.0001 |
| Feet (without hooves) | Gelatin C3 | 0.0010 | 0.0014 | 0.0000 |
| Floatation fat | Spreading/Compost | 0.0000 | 0.0000 | 0.0000 |
| Forehead | C1-C2 for disposal | 0.0000 | 0.0000 | 0.0000 |
| Forelock | PAP C3 | 0.0036 | 0.0014 | 0.0001 |
| Gallbladder | Pet food | 0.0010 | 0.0014 | 0.0001 |
| Head trimmings | Pet food | 0.0010 | 0.0014 | 0.0001 |
| Heart | Human food | 0.0010 | 0.0014 | 0.0003 |
| Heart trimmings | Pet food | 0.0010 | 0.0014 | 0.0001 |
| Hide | Skin tannery C3 | 0.0014 | 0.0014 | 0.0023 |
| Hooves | PAP C3 | 0.0036 | 0.0014 | 0.0001 |
| Horns | PAP C3 | 0.0036 | 0.0014 | 0.0001 |
| Kidney | Human food | 0.0010 | 0.0014 | 0.0006 |
| Large intestine | C1-C2 for disposal | 0.0000 | 0.0000 | 0.0000 |
| Liver | Human food | 0.0043 | 0.0014 | 0.0007 |
| Liver trimmings | Pet food | 0.0045 | 0.0014 | 0.0001 |
| Lower jaw | PAP C3 | 0.0009 | 0.0014 | 0.0001 |
| Lungs | Pet food | 0.0012 | 0.0014 | 0.0001 |
| Mask | Skin tannery C3 | 0.0014 | 0.0014 | 0.0023 |
| Mesenteric fat | C1-C2 for disposal | 0.0000 | 0.0000 | 0.0000 |
| Muscle | Human food | 0.0010 | 0.0014 | 0.0023 |
| Muzzle | Human food | 0.0014 | 0.0014 | 0.0014 |
| Omasum | Human food | 0.0084 | 0.0014 | 0.0010 |
| Omasum fat | Fat and greaves C3 | 0.0033 | 0.0014 | 0.0001 |
| Rumen and forestomach | Human food | 0.0084 | 0.0014 | 0.0010 |
| Rumen fat | Fat and greaves C3 | 0.0033 | 0.0014 | 0.0001 |
| Sanitary seizures | C1-C2 for disposal | 0.0000 | 0.0000 | 0.0000 |
| Screening and sifting wastes | C1-C2 for disposal | 0.0000 | 0.0000 | 0.0000 |
| Small intestine | PAP C3 | 0.0084 | 0.0014 | 0.0001 |
| Spinal cord | C1-C2 for disposal | 0.0000 | 0.0000 | 0.0000 |
| Spinal cord waste | C1-C2 for disposal | 0.0000 | 0.0000 | 0.0000 |
| Spine | C1-C2 for disposal | 0.0000 | 0.0000 | 0.0000 |
| Spleen | Pet food | 0.0010 | 0.0014 | 0.0001 |
| Stillborn | PAP C3 | 0.0087 | 0.0014 | 0.0000 |
| Tallow | Fat and greaves C3 | 0.0015 | 0.0014 | 0.0001 |
| Tongue | Human food | 0.0009 | 0.0014 | 0.0022 |
| Tonsil | C1-C2 for disposal | 0.0000 | 0.0000 | 0.0000 |
| Trachea | Pet food | 0.0014 | 0.0014 | 0.0001 |
| Udder | Pet food | 0.0002 | 0.0014 | 0.0001 |
| Upper throat | Pet food | 0.0010 | 0.0014 | 0.0001 |
| Water in the rumen | Spreading/Compost | 0.0000 | 0.0000 | 0.0000 |

Table 55: Allocation factors for Blonde d’Aquitaine Cull Cows reared in Pasture

| COPRODUCT | Destination | Blonde d’Aquitaine/Cull cow/pasture | | |
| --- | --- | --- | --- | --- |
|  |  | **Biophysical Allocation Factor** | **Mass Allocation Factor** | **Economic Allocation Factor** |
| Abomasum | Human food | 0.0073 | 0.0012 | 0.0009 |
| Abomasum fat | Fat and greaves C3 | 0.0026 | 0.0012 | 0.0001 |
| Aponeurosis | Human food | 0.0009 | 0.0012 | 0.0012 |
| Bile | PAP C3 | 0.0003 | 0.0012 | 0.0001 |
| Blood | PAP C3 | 0.0007 | 0.0012 | 0.0003 |
| Blood | Pet food | 0.0007 | 0.0012 | 0.0001 |
| Bones | Gelatin C3 | 0.0007 | 0.0012 | 0.0000 |
| Bones of head, brain, eyes and teeth | C1-C2 for disposal | 0.0000 | 0.0000 | 0.0000 |
| Cheek | Human food | 0.0008 | 0.0012 | 0.0027 |
| Cheek | Human food | 0.0008 | 0.0012 | 0.0027 |
| Cheek trimmings | Pet food | 0.0008 | 0.0012 | 0.0001 |
| Chops | Pet food | 0.0011 | 0.0012 | 0.0001 |
| Contents of intestines | Spreading/Compost | 0.0000 | 0.0000 | 0.0000 |
| Contents of the rumen | Spreading/Compost | 0.0000 | 0.0000 | 0.0000 |
| Ears | PAP C3 | 0.0010 | 0.0012 | 0.0001 |
| Esophagus | Pet food | 0.0008 | 0.0012 | 0.0001 |
| Fat | Fat and greaves C3 | 0.0010 | 0.0012 | 0.0001 |
| Fat around heart | Fat and greaves C3 | 0.0010 | 0.0012 | 0.0001 |
| Fat in the kidney | Fat and greaves C3 | 0.0010 | 0.0012 | 0.0001 |
| Feet (without hooves) | Gelatin C3 | 0.0008 | 0.0012 | 0.0000 |
| Floatation fat | Spreading/Compost | 0.0000 | 0.0000 | 0.0000 |
| Forehead | C1-C2 for disposal | 0.0000 | 0.0000 | 0.0000 |
| Forelock | PAP C3 | 0.0029 | 0.0012 | 0.0001 |
| Gallbladder | Pet food | 0.0008 | 0.0012 | 0.0001 |
| Head trimmings | Pet food | 0.0008 | 0.0012 | 0.0001 |
| Heart | Human food | 0.0008 | 0.0012 | 0.0003 |
| Heart trimmings | Pet food | 0.0008 | 0.0012 | 0.0001 |
| Hide | Skin tannery C3 | 0.0011 | 0.0012 | 0.0020 |
| Hooves | PAP C3 | 0.0029 | 0.0012 | 0.0001 |
| Horns | PAP C3 | 0.0029 | 0.0012 | 0.0001 |
| Kidney | Human food | 0.0008 | 0.0012 | 0.0005 |
| Large intestine | C1-C2 for disposal | 0.0000 | 0.0000 | 0.0000 |
| Liver | Human food | 0.0037 | 0.0012 | 0.0006 |
| Liver trimmings | Pet food | 0.0039 | 0.0012 | 0.0001 |
| Lower jaw | PAP C3 | 0.0007 | 0.0012 | 0.0001 |
| Lungs | Pet food | 0.0009 | 0.0012 | 0.0001 |
| Mask | Skin tannery C3 | 0.0011 | 0.0012 | 0.0020 |
| Mesenteric fat | C1-C2 for disposal | 0.0000 | 0.0000 | 0.0000 |
| Muscle | Human food | 0.0008 | 0.0012 | 0.0020 |
| Muzzle | Human food | 0.0011 | 0.0012 | 0.0012 |
| Omasum | Human food | 0.0073 | 0.0012 | 0.0009 |
| Omasum fat | Fat and greaves C3 | 0.0026 | 0.0012 | 0.0001 |
| Rumen and forestomach | Human food | 0.0073 | 0.0012 | 0.0009 |
| Rumen fat | Fat and greaves C3 | 0.0026 | 0.0012 | 0.0001 |
| Sanitary seizures | C1-C2 for disposal | 0.0000 | 0.0000 | 0.0000 |
| Screening and sifting wastes | C1-C2 for disposal | 0.0000 | 0.0000 | 0.0000 |
| Small intestine | PAP C3 | 0.0073 | 0.0012 | 0.0001 |
| Spinal cord | C1-C2 for disposal | 0.0000 | 0.0000 | 0.0000 |
| Spinal cord waste | C1-C2 for disposal | 0.0000 | 0.0000 | 0.0000 |
| Spine | C1-C2 for disposal | 0.0000 | 0.0000 | 0.0000 |
| Spleen | Pet food | 0.0008 | 0.0012 | 0.0001 |
| Stillborn | PAP C3 | 0.0076 | 0.0012 | 0.0000 |
| Tallow | Fat and greaves C3 | 0.0010 | 0.0012 | 0.0001 |
| Tongue | Human food | 0.0007 | 0.0012 | 0.0019 |
| Tonsil | C1-C2 for disposal | 0.0000 | 0.0000 | 0.0000 |
| Trachea | Pet food | 0.0011 | 0.0012 | 0.0001 |
| Udder | Pet food | 0.0002 | 0.0012 | 0.0001 |
| Upper throat | Pet food | 0.0008 | 0.0012 | 0.0001 |
| Water in the rumen | Spreading/Compost | 0.0000 | 0.0000 | 0.0000 |

Table 56: Allocation factors for Blonde d’Aquitaine Beef reared in Pasture

| COPRODUCT | Destination | Blonde d’Aquitaine/beef/pasture | | |
| --- | --- | --- | --- | --- |
|  |  | **Biophysical Allocation Factor** | **Mass Allocation Factor** | **Economic Allocation Factor** |
| Abomasum | Human food | 0.0079 | 0.0013 | 0.0009 |
| Abomasum fat | Fat and greaves C3 | 0.0030 | 0.0013 | 0.0001 |
| Aponeurosis | Human food | 0.0010 | 0.0013 | 0.0013 |
| Bile | PAP C3 | 0.0004 | 0.0013 | 0.0001 |
| Blood | PAP C3 | 0.0008 | 0.0013 | 0.0003 |
| Blood | Pet food | 0.0008 | 0.0013 | 0.0001 |
| Bones | Gelatin C3 | 0.0008 | 0.0013 | 0.0000 |
| Bones of head, brain, eyes and teeth | C1-C2 for disposal | 0.0000 | 0.0000 | 0.0000 |
| Cheek | Human food | 0.0009 | 0.0013 | 0.0028 |
| Cheek | Human food | 0.0009 | 0.0013 | 0.0028 |
| Cheek trimmings | Pet food | 0.0009 | 0.0013 | 0.0001 |
| Chops | Pet food | 0.0013 | 0.0013 | 0.0001 |
| Contents of intestines | Spreading/Compost | 0.0000 | 0.0000 | 0.0000 |
| Contents of the rumen | Spreading/Compost | 0.0000 | 0.0000 | 0.0000 |
| Ears | PAP C3 | 0.0011 | 0.0013 | 0.0001 |
| Esophagus | Pet food | 0.0009 | 0.0013 | 0.0001 |
| Fat | Fat and greaves C3 | 0.0013 | 0.0013 | 0.0001 |
| Fat around heart | Fat and greaves C3 | 0.0013 | 0.0013 | 0.0001 |
| Fat in the kidney | Fat and greaves C3 | 0.0013 | 0.0013 | 0.0001 |
| Feet (without hooves) | Gelatin C3 | 0.0009 | 0.0013 | 0.0000 |
| Floatation fat | Spreading/Compost | 0.0000 | 0.0000 | 0.0000 |
| Forehead | C1-C2 for disposal | 0.0000 | 0.0000 | 0.0000 |
| Forelock | PAP C3 | 0.0033 | 0.0013 | 0.0001 |
| Gallbladder | Pet food | 0.0009 | 0.0013 | 0.0001 |
| Head trimmings | Pet food | 0.0009 | 0.0013 | 0.0001 |
| Heart | Human food | 0.0009 | 0.0013 | 0.0003 |
| Heart trimmings | Pet food | 0.0009 | 0.0013 | 0.0001 |
| Hide | Skin tannery C3 | 0.0013 | 0.0013 | 0.0021 |
| Hooves | PAP C3 | 0.0033 | 0.0013 | 0.0001 |
| Horns | PAP C3 | 0.0033 | 0.0013 | 0.0001 |
| Kidney | Human food | 0.0009 | 0.0013 | 0.0005 |
| Large intestine | C1-C2 for disposal | 0.0000 | 0.0000 | 0.0000 |
| Liver | Human food | 0.0041 | 0.0013 | 0.0006 |
| Liver trimmings | Pet food | 0.0043 | 0.0013 | 0.0001 |
| Lower jaw | PAP C3 | 0.0008 | 0.0013 | 0.0001 |
| Lungs | Pet food | 0.0011 | 0.0013 | 0.0001 |
| Mask | Skin tannery C3 | 0.0013 | 0.0013 | 0.0021 |
| Mesenteric fat | C1-C2 for disposal | 0.0000 | 0.0000 | 0.0000 |
| Muscle | Human food | 0.0009 | 0.0013 | 0.0021 |
| Muzzle | Human food | 0.0013 | 0.0013 | 0.0013 |
| Omasum | Human food | 0.0079 | 0.0013 | 0.0009 |
| Omasum fat | Fat and greaves C3 | 0.0030 | 0.0013 | 0.0001 |
| Rumen and forestomach | Human food | 0.0079 | 0.0013 | 0.0009 |
| Rumen fat | Fat and greaves C3 | 0.0030 | 0.0013 | 0.0001 |
| Sanitary seizures | C1-C2 for disposal | 0.0000 | 0.0000 | 0.0000 |
| Screening and sifting wastes | C1-C2 for disposal | 0.0000 | 0.0000 | 0.0000 |
| Small intestine | PAP C3 | 0.0079 | 0.0013 | 0.0001 |
| Spinal cord | C1-C2 for disposal | 0.0000 | 0.0000 | 0.0000 |
| Spinal cord waste | C1-C2 for disposal | 0.0000 | 0.0000 | 0.0000 |
| Spine | C1-C2 for disposal | 0.0000 | 0.0000 | 0.0000 |
| Spleen | Pet food | 0.0009 | 0.0013 | 0.0001 |
| Stillborn | PAP C3 | 0.0083 | 0.0013 | 0.0000 |
| Tallow | Fat and greaves C3 | 0.0013 | 0.0013 | 0.0001 |
| Tongue | Human food | 0.0008 | 0.0013 | 0.0020 |
| Tonsil | C1-C2 for disposal | 0.0000 | 0.0000 | 0.0000 |
| Trachea | Pet food | 0.0013 | 0.0013 | 0.0001 |
| Udder | Pet food | 0.0002 | 0.0013 | 0.0001 |
| Upper throat | Pet food | 0.0009 | 0.0013 | 0.0001 |
| Water in the rumen | Spreading/Compost | 0.0000 | 0.0000 | 0.0000 |

Table 57: Allocation factors for Blonde d’Aquitaine Young Bulls reared in Stall

| COPRODUCT | Destination | Blonde d’Aquitaine/young bull/stall | | |
| --- | --- | --- | --- | --- |
|  |  | **Biophysical Allocation Factor** | **Mass Allocation Factor** | **Economic Allocation Factor** |
| Abomasum | Human food | 0.0084 | 0.0015 | 0.0011 |
| Abomasum fat | Fat and greaves C3 | 0.0035 | 0.0015 | 0.0001 |
| Aponeurosis | Human food | 0.0012 | 0.0015 | 0.0014 |
| Bile | PAP C3 | 0.0004 | 0.0015 | 0.0001 |
| Blood | PAP C3 | 0.0009 | 0.0015 | 0.0003 |
| Blood | Pet food | 0.0009 | 0.0015 | 0.0001 |
| Bones | Gelatin C3 | 0.0010 | 0.0015 | 0.0000 |
| Bones of head, brain, eyes and teeth | C1-C2 for disposal | 0.0000 | 0.0000 | 0.0000 |
| Cheek | Human food | 0.0011 | 0.0015 | 0.0031 |
| Cheek | Human food | 0.0011 | 0.0015 | 0.0031 |
| Cheek trimmings | Pet food | 0.0011 | 0.0015 | 0.0001 |
| Chops | Pet food | 0.0015 | 0.0015 | 0.0001 |
| Contents of intestines | Spreading/Compost | 0.0000 | 0.0000 | 0.0000 |
| Contents of the rumen | Spreading/Compost | 0.0000 | 0.0000 | 0.0000 |
| Ears | PAP C3 | 0.0013 | 0.0015 | 0.0001 |
| Esophagus | Pet food | 0.0010 | 0.0015 | 0.0001 |
| Fat | Fat and greaves C3 | 0.0018 | 0.0015 | 0.0001 |
| Fat around heart | Fat and greaves C3 | 0.0018 | 0.0015 | 0.0001 |
| Fat in the kidney | Fat and greaves C3 | 0.0018 | 0.0015 | 0.0001 |
| Feet (without hooves) | Gelatin C3 | 0.0011 | 0.0015 | 0.0000 |
| Floatation fat | Spreading/Compost | 0.0000 | 0.0000 | 0.0000 |
| Forehead | C1-C2 for disposal | 0.0000 | 0.0000 | 0.0000 |
| Forelock | PAP C3 | 0.0038 | 0.0015 | 0.0001 |
| Gallbladder | Pet food | 0.0011 | 0.0015 | 0.0001 |
| Head trimmings | Pet food | 0.0011 | 0.0015 | 0.0001 |
| Heart | Human food | 0.0010 | 0.0015 | 0.0003 |
| Heart trimmings | Pet food | 0.0011 | 0.0015 | 0.0001 |
| Hide | Skin tannery C3 | 0.0015 | 0.0015 | 0.0024 |
| Hooves | PAP C3 | 0.0038 | 0.0015 | 0.0001 |
| Horns | PAP C3 | 0.0038 | 0.0015 | 0.0001 |
| Kidney | Human food | 0.0011 | 0.0015 | 0.0006 |
| Large intestine | C1-C2 for disposal | 0.0000 | 0.0000 | 0.0000 |
| Liver | Human food | 0.0044 | 0.0015 | 0.0007 |
| Liver trimmings | Pet food | 0.0045 | 0.0015 | 0.0001 |
| Lower jaw | PAP C3 | 0.0010 | 0.0015 | 0.0001 |
| Lungs | Pet food | 0.0012 | 0.0015 | 0.0001 |
| Mask | Skin tannery C3 | 0.0015 | 0.0015 | 0.0024 |
| Mesenteric fat | C1-C2 for disposal | 0.0000 | 0.0000 | 0.0000 |
| Muscle | Human food | 0.0011 | 0.0015 | 0.0024 |
| Muzzle | Human food | 0.0015 | 0.0015 | 0.0014 |
| Omasum | Human food | 0.0084 | 0.0015 | 0.0011 |
| Omasum fat | Fat and greaves C3 | 0.0035 | 0.0015 | 0.0001 |
| Rumen and forestomach | Human food | 0.0084 | 0.0015 | 0.0011 |
| Rumen fat | Fat and greaves C3 | 0.0035 | 0.0015 | 0.0001 |
| Sanitary seizures | C1-C2 for disposal | 0.0000 | 0.0000 | 0.0000 |
| Screening and sifting wastes | C1-C2 for disposal | 0.0000 | 0.0000 | 0.0000 |
| Small intestine | PAP C3 | 0.0084 | 0.0015 | 0.0001 |
| Spinal cord | C1-C2 for disposal | 0.0000 | 0.0000 | 0.0000 |
| Spinal cord waste | C1-C2 for disposal | 0.0000 | 0.0000 | 0.0000 |
| Spine | C1-C2 for disposal | 0.0000 | 0.0000 | 0.0000 |
| Spleen | Pet food | 0.0011 | 0.0015 | 0.0001 |
| Stillborn | PAP C3 | 0.0087 | 0.0015 | 0.0000 |
| Tallow | Fat and greaves C3 | 0.0018 | 0.0015 | 0.0001 |
| Tongue | Human food | 0.0010 | 0.0015 | 0.0023 |
| Tonsil | C1-C2 for disposal | 0.0000 | 0.0000 | 0.0000 |
| Trachea | Pet food | 0.0015 | 0.0015 | 0.0001 |
| Udder | Pet food | 0.0002 | 0.0015 | 0.0001 |
| Upper throat | Pet food | 0.0011 | 0.0015 | 0.0001 |
| Water in the rumen | Spreading/Compost | 0.0000 | 0.0000 | 0.0000 |

Table 58: Allocation factors for Blonde d’Aquitaine Heifers reared in Stall

| COPRODUCT | Destination | Blonde d’Aquitaine/heifer/stall | | |
| --- | --- | --- | --- | --- |
|  |  | **Biophysical Allocation Factor** | **Mass Allocation Factor** | **Economic Allocation Factor** |
| Abomasum | Human food | 0.0080 | 0.0014 | 0.0010 |
| Abomasum fat | Fat and greaves C3 | 0.0033 | 0.0014 | 0.0001 |
| Aponeurosis | Human food | 0.0011 | 0.0014 | 0.0014 |
| Bile | PAP C3 | 0.0004 | 0.0014 | 0.0001 |
| Blood | PAP C3 | 0.0008 | 0.0014 | 0.0003 |
| Blood | Pet food | 0.0008 | 0.0014 | 0.0001 |
| Bones | Gelatin C3 | 0.0010 | 0.0014 | 0.0000 |
| Bones of head, brain, eyes and teeth | C1-C2 for disposal | 0.0000 | 0.0000 | 0.0000 |
| Cheek | Human food | 0.0010 | 0.0014 | 0.0031 |
| Cheek | Human food | 0.0010 | 0.0014 | 0.0031 |
| Cheek trimmings | Pet food | 0.0010 | 0.0014 | 0.0001 |
| Chops | Pet food | 0.0014 | 0.0014 | 0.0001 |
| Contents of intestines | Spreading/Compost | 0.0000 | 0.0000 | 0.0000 |
| Contents of the rumen | Spreading/Compost | 0.0000 | 0.0000 | 0.0000 |
| Ears | PAP C3 | 0.0013 | 0.0014 | 0.0001 |
| Esophagus | Pet food | 0.0010 | 0.0014 | 0.0001 |
| Fat | Fat and greaves C3 | 0.0016 | 0.0014 | 0.0001 |
| Fat around heart | Fat and greaves C3 | 0.0016 | 0.0014 | 0.0001 |
| Fat in the kidney | Fat and greaves C3 | 0.0016 | 0.0014 | 0.0001 |
| Feet (without hooves) | Gelatin C3 | 0.0010 | 0.0014 | 0.0000 |
| Floatation fat | Spreading/Compost | 0.0000 | 0.0000 | 0.0000 |
| Forehead | C1-C2 for disposal | 0.0000 | 0.0000 | 0.0000 |
| Forelock | PAP C3 | 0.0036 | 0.0014 | 0.0001 |
| Gallbladder | Pet food | 0.0010 | 0.0014 | 0.0001 |
| Head trimmings | Pet food | 0.0010 | 0.0014 | 0.0001 |
| Heart | Human food | 0.0010 | 0.0014 | 0.0003 |
| Heart trimmings | Pet food | 0.0010 | 0.0014 | 0.0001 |
| Hide | Skin tannery C3 | 0.0014 | 0.0014 | 0.0023 |
| Hooves | PAP C3 | 0.0036 | 0.0014 | 0.0001 |
| Horns | PAP C3 | 0.0036 | 0.0014 | 0.0001 |
| Kidney | Human food | 0.0010 | 0.0014 | 0.0006 |
| Large intestine | C1-C2 for disposal | 0.0000 | 0.0000 | 0.0000 |
| Liver | Human food | 0.0042 | 0.0014 | 0.0007 |
| Liver trimmings | Pet food | 0.0043 | 0.0014 | 0.0001 |
| Lower jaw | PAP C3 | 0.0009 | 0.0014 | 0.0001 |
| Lungs | Pet food | 0.0011 | 0.0014 | 0.0001 |
| Mask | Skin tannery C3 | 0.0014 | 0.0014 | 0.0023 |
| Mesenteric fat | C1-C2 for disposal | 0.0000 | 0.0000 | 0.0000 |
| Muscle | Human food | 0.0010 | 0.0014 | 0.0023 |
| Muzzle | Human food | 0.0014 | 0.0014 | 0.0014 |
| Omasum | Human food | 0.0080 | 0.0014 | 0.0010 |
| Omasum fat | Fat and greaves C3 | 0.0033 | 0.0014 | 0.0001 |
| Rumen and forestomach | Human food | 0.0080 | 0.0014 | 0.0010 |
| Rumen fat | Fat and greaves C3 | 0.0033 | 0.0014 | 0.0001 |
| Sanitary seizures | C1-C2 for disposal | 0.0000 | 0.0000 | 0.0000 |
| Screening and sifting wastes | C1-C2 for disposal | 0.0000 | 0.0000 | 0.0000 |
| Small intestine | PAP C3 | 0.0080 | 0.0014 | 0.0001 |
| Spinal cord | C1-C2 for disposal | 0.0000 | 0.0000 | 0.0000 |
| Spinal cord waste | C1-C2 for disposal | 0.0000 | 0.0000 | 0.0000 |
| Spine | C1-C2 for disposal | 0.0000 | 0.0000 | 0.0000 |
| Spleen | Pet food | 0.0010 | 0.0014 | 0.0001 |
| Stillborn | PAP C3 | 0.0083 | 0.0014 | 0.0000 |
| Tallow | Fat and greaves C3 | 0.0016 | 0.0014 | 0.0001 |
| Tongue | Human food | 0.0009 | 0.0014 | 0.0022 |
| Tonsil | C1-C2 for disposal | 0.0000 | 0.0000 | 0.0000 |
| Trachea | Pet food | 0.0014 | 0.0014 | 0.0001 |
| Udder | Pet food | 0.0002 | 0.0014 | 0.0001 |
| Upper throat | Pet food | 0.0010 | 0.0014 | 0.0001 |
| Water in the rumen | Spreading/Compost | 0.0000 | 0.0000 | 0.0000 |

Table 59: Allocation factors for Blonde d’Aquitaine Cull Cows reared in Stall

| COPRODUCT | Destination | Blonde d’Aquitaine/Cull cow/stall | | |
| --- | --- | --- | --- | --- |
|  |  | **Biophysical Allocation Factor** | **Mass Allocation Factor** | **Economic Allocation Factor** |
| Abomasum | Human food | 0.0070 | 0.0012 | 0.0009 |
| Abomasum fat | Fat and greaves C3 | 0.0026 | 0.0012 | 0.0001 |
| Aponeurosis | Human food | 0.0009 | 0.0012 | 0.0012 |
| Bile | PAP C3 | 0.0003 | 0.0012 | 0.0001 |
| Blood | PAP C3 | 0.0007 | 0.0012 | 0.0003 |
| Blood | Pet food | 0.0007 | 0.0012 | 0.0001 |
| Bones | Gelatin C3 | 0.0007 | 0.0012 | 0.0000 |
| Bones of head, brain, eyes and teeth | C1-C2 for disposal | 0.0000 | 0.0000 | 0.0000 |
| Cheek | Human food | 0.0008 | 0.0012 | 0.0027 |
| Cheek | Human food | 0.0008 | 0.0012 | 0.0027 |
| Cheek trimmings | Pet food | 0.0008 | 0.0012 | 0.0001 |
| Chops | Pet food | 0.0011 | 0.0012 | 0.0001 |
| Contents of intestines | Spreading/Compost | 0.0000 | 0.0000 | 0.0000 |
| Contents of the rumen | Spreading/Compost | 0.0000 | 0.0000 | 0.0000 |
| Ears | PAP C3 | 0.0010 | 0.0012 | 0.0001 |
| Esophagus | Pet food | 0.0008 | 0.0012 | 0.0001 |
| Fat | Fat and greaves C3 | 0.0011 | 0.0012 | 0.0001 |
| Fat around heart | Fat and greaves C3 | 0.0011 | 0.0012 | 0.0001 |
| Fat in the kidney | Fat and greaves C3 | 0.0011 | 0.0012 | 0.0001 |
| Feet (without hooves) | Gelatin C3 | 0.0008 | 0.0012 | 0.0000 |
| Floatation fat | Spreading/Compost | 0.0000 | 0.0000 | 0.0000 |
| Forehead | C1-C2 for disposal | 0.0000 | 0.0000 | 0.0000 |
| Forelock | PAP C3 | 0.0028 | 0.0012 | 0.0001 |
| Gallbladder | Pet food | 0.0008 | 0.0012 | 0.0001 |
| Head trimmings | Pet food | 0.0008 | 0.0012 | 0.0001 |
| Heart | Human food | 0.0008 | 0.0012 | 0.0003 |
| Heart trimmings | Pet food | 0.0008 | 0.0012 | 0.0001 |
| Hide | Skin tannery C3 | 0.0011 | 0.0012 | 0.0020 |
| Hooves | PAP C3 | 0.0028 | 0.0012 | 0.0001 |
| Horns | PAP C3 | 0.0028 | 0.0012 | 0.0001 |
| Kidney | Human food | 0.0008 | 0.0012 | 0.0005 |
| Large intestine | C1-C2 for disposal | 0.0000 | 0.0000 | 0.0000 |
| Liver | Human food | 0.0036 | 0.0012 | 0.0006 |
| Liver trimmings | Pet food | 0.0038 | 0.0012 | 0.0001 |
| Lower jaw | PAP C3 | 0.0007 | 0.0012 | 0.0001 |
| Lungs | Pet food | 0.0009 | 0.0012 | 0.0001 |
| Mask | Skin tannery C3 | 0.0011 | 0.0012 | 0.0020 |
| Mesenteric fat | C1-C2 for disposal | 0.0000 | 0.0000 | 0.0000 |
| Muscle | Human food | 0.0008 | 0.0012 | 0.0020 |
| Muzzle | Human food | 0.0011 | 0.0012 | 0.0012 |
| Omasum | Human food | 0.0070 | 0.0012 | 0.0009 |
| Omasum fat | Fat and greaves C3 | 0.0026 | 0.0012 | 0.0001 |
| Rumen and forestomach | Human food | 0.0070 | 0.0012 | 0.0009 |
| Rumen fat | Fat and greaves C3 | 0.0026 | 0.0012 | 0.0001 |
| Sanitary seizures | C1-C2 for disposal | 0.0000 | 0.0000 | 0.0000 |
| Screening and sifting wastes | C1-C2 for disposal | 0.0000 | 0.0000 | 0.0000 |
| Small intestine | PAP C3 | 0.0070 | 0.0012 | 0.0001 |
| Spinal cord | C1-C2 for disposal | 0.0000 | 0.0000 | 0.0000 |
| Spinal cord waste | C1-C2 for disposal | 0.0000 | 0.0000 | 0.0000 |
| Spine | C1-C2 for disposal | 0.0000 | 0.0000 | 0.0000 |
| Spleen | Pet food | 0.0008 | 0.0012 | 0.0001 |
| Stillborn | PAP C3 | 0.0074 | 0.0012 | 0.0000 |
| Tallow | Fat and greaves C3 | 0.0011 | 0.0012 | 0.0001 |
| Tongue | Human food | 0.0007 | 0.0012 | 0.0019 |
| Tonsil | C1-C2 for disposal | 0.0000 | 0.0000 | 0.0000 |
| Trachea | Pet food | 0.0011 | 0.0012 | 0.0001 |
| Udder | Pet food | 0.0002 | 0.0012 | 0.0001 |
| Upper throat | Pet food | 0.0008 | 0.0012 | 0.0001 |
| Water in the rumen | Spreading/Compost | 0.0000 | 0.0000 | 0.0000 |

Table 60: Allocation factors for Blonde d’Aquitaine Beef reared in Stall

| COPRODUCT | Destination | Blonde d’Aquitaine/beef/stall | | |
| --- | --- | --- | --- | --- |
|  |  | **Biophysical Allocation Factor** | **Mass Allocation Factor** | **Economic Allocation Factor** |
| Abomasum | Human food | 0.0076 | 0.0013 | 0.0009 |
| Abomasum fat | Fat and greaves C3 | 0.0030 | 0.0013 | 0.0001 |
| Aponeurosis | Human food | 0.0010 | 0.0013 | 0.0013 |
| Bile | PAP C3 | 0.0004 | 0.0013 | 0.0001 |
| Blood | PAP C3 | 0.0008 | 0.0013 | 0.0003 |
| Blood | Pet food | 0.0008 | 0.0013 | 0.0001 |
| Bones | Gelatin C3 | 0.0009 | 0.0013 | 0.0000 |
| Bones of head, brain, eyes and teeth | C1-C2 for disposal | 0.0000 | 0.0000 | 0.0000 |
| Cheek | Human food | 0.0009 | 0.0013 | 0.0028 |
| Cheek | Human food | 0.0009 | 0.0013 | 0.0028 |
| Cheek trimmings | Pet food | 0.0009 | 0.0013 | 0.0001 |
| Chops | Pet food | 0.0013 | 0.0013 | 0.0001 |
| Contents of intestines | Spreading/Compost | 0.0000 | 0.0000 | 0.0000 |
| Contents of the rumen | Spreading/Compost | 0.0000 | 0.0000 | 0.0000 |
| Ears | PAP C3 | 0.0011 | 0.0013 | 0.0001 |
| Esophagus | Pet food | 0.0009 | 0.0013 | 0.0001 |
| Fat | Fat and greaves C3 | 0.0014 | 0.0013 | 0.0001 |
| Fat around heart | Fat and greaves C3 | 0.0014 | 0.0013 | 0.0001 |
| Fat in the kidney | Fat and greaves C3 | 0.0014 | 0.0013 | 0.0001 |
| Feet (without hooves) | Gelatin C3 | 0.0009 | 0.0013 | 0.0000 |
| Floatation fat | Spreading/Compost | 0.0000 | 0.0000 | 0.0000 |
| Forehead | C1-C2 for disposal | 0.0000 | 0.0000 | 0.0000 |
| Forelock | PAP C3 | 0.0032 | 0.0013 | 0.0001 |
| Gallbladder | Pet food | 0.0009 | 0.0013 | 0.0001 |
| Head trimmings | Pet food | 0.0009 | 0.0013 | 0.0001 |
| Heart | Human food | 0.0009 | 0.0013 | 0.0003 |
| Heart trimmings | Pet food | 0.0009 | 0.0013 | 0.0001 |
| Hide | Skin tannery C3 | 0.0013 | 0.0013 | 0.0021 |
| Hooves | PAP C3 | 0.0032 | 0.0013 | 0.0001 |
| Horns | PAP C3 | 0.0032 | 0.0013 | 0.0001 |
| Kidney | Human food | 0.0009 | 0.0013 | 0.0005 |
| Large intestine | C1-C2 for disposal | 0.0000 | 0.0000 | 0.0000 |
| Liver | Human food | 0.0039 | 0.0013 | 0.0006 |
| Liver trimmings | Pet food | 0.0041 | 0.0013 | 0.0001 |
| Lower jaw | PAP C3 | 0.0008 | 0.0013 | 0.0001 |
| Lungs | Pet food | 0.0010 | 0.0013 | 0.0001 |
| Mask | Skin tannery C3 | 0.0013 | 0.0013 | 0.0021 |
| Mesenteric fat | C1-C2 for disposal | 0.0000 | 0.0000 | 0.0000 |
| Muscle | Human food | 0.0009 | 0.0013 | 0.0021 |
| Muzzle | Human food | 0.0013 | 0.0013 | 0.0013 |
| Omasum | Human food | 0.0076 | 0.0013 | 0.0009 |
| Omasum fat | Fat and greaves C3 | 0.0030 | 0.0013 | 0.0001 |
| Rumen and forestomach | Human food | 0.0076 | 0.0013 | 0.0009 |
| Rumen fat | Fat and greaves C3 | 0.0030 | 0.0013 | 0.0001 |
| Sanitary seizures | C1-C2 for disposal | 0.0000 | 0.0000 | 0.0000 |
| Screening and sifting wastes | C1-C2 for disposal | 0.0000 | 0.0000 | 0.0000 |
| Small intestine | PAP C3 | 0.0076 | 0.0013 | 0.0001 |
| Spinal cord | C1-C2 for disposal | 0.0000 | 0.0000 | 0.0000 |
| Spinal cord waste | C1-C2 for disposal | 0.0000 | 0.0000 | 0.0000 |
| Spine | C1-C2 for disposal | 0.0000 | 0.0000 | 0.0000 |
| Spleen | Pet food | 0.0009 | 0.0013 | 0.0001 |
| Stillborn | PAP C3 | 0.0079 | 0.0013 | 0.0000 |
| Tallow | Fat and greaves C3 | 0.0014 | 0.0013 | 0.0001 |
| Tongue | Human food | 0.0008 | 0.0013 | 0.0020 |
| Tonsil | C1-C2 for disposal | 0.0000 | 0.0000 | 0.0000 |
| Trachea | Pet food | 0.0013 | 0.0013 | 0.0001 |
| Udder | Pet food | 0.0002 | 0.0013 | 0.0001 |
| Upper throat | Pet food | 0.0009 | 0.0013 | 0.0001 |
| Water in the rumen | Spreading/Compost | 0.0000 | 0.0000 | 0.0000 |

Table 61: Allocation factors for Salers Young Bulls reared in Grazing Large Area

| COPRODUCT | Destination | Salers/young bull/grazing large area | | |
| --- | --- | --- | --- | --- |
|  |  | **Biophysical Allocation Factor** | **Mass Allocation Factor** | **Economic Allocation Factor** |
| Abomasum | Human food | 0.0087 | 0.0015 | 0.0011 |
| Abomasum fat | Fat and greaves C3 | 0.0032 | 0.0015 | 0.0001 |
| Aponeurosis | Human food | 0.0011 | 0.0015 | 0.0015 |
| Bile | PAP C3 | 0.0004 | 0.0015 | 0.0001 |
| Blood | PAP C3 | 0.0009 | 0.0015 | 0.0003 |
| Blood | Pet food | 0.0009 | 0.0015 | 0.0001 |
| Bones | Gelatin C3 | 0.0009 | 0.0015 | 0.0000 |
| Bones of head, brain, eyes and teeth | C1-C2 for disposal | 0.0000 | 0.0000 | 0.0000 |
| Cheek | Human food | 0.0010 | 0.0015 | 0.0033 |
| Cheek | Human food | 0.0010 | 0.0015 | 0.0033 |
| Cheek trimmings | Pet food | 0.0010 | 0.0015 | 0.0001 |
| Chops | Pet food | 0.0014 | 0.0015 | 0.0001 |
| Contents of intestines | Spreading/Compost | 0.0000 | 0.0000 | 0.0000 |
| Contents of the rumen | Spreading/Compost | 0.0000 | 0.0000 | 0.0000 |
| Ears | PAP C3 | 0.0013 | 0.0015 | 0.0001 |
| Esophagus | Pet food | 0.0010 | 0.0015 | 0.0001 |
| Fat | Fat and greaves C3 | 0.0014 | 0.0015 | 0.0001 |
| Fat around heart | Fat and greaves C3 | 0.0014 | 0.0015 | 0.0001 |
| Fat in the kidney | Fat and greaves C3 | 0.0014 | 0.0015 | 0.0001 |
| Feet (without hooves) | Gelatin C3 | 0.0010 | 0.0015 | 0.0000 |
| Floatation fat | Spreading/Compost | 0.0000 | 0.0000 | 0.0000 |
| Forehead | C1-C2 for disposal | 0.0000 | 0.0000 | 0.0000 |
| Forelock | PAP C3 | 0.0037 | 0.0015 | 0.0001 |
| Gallbladder | Pet food | 0.0010 | 0.0015 | 0.0001 |
| Head trimmings | Pet food | 0.0010 | 0.0015 | 0.0001 |
| Heart | Human food | 0.0010 | 0.0015 | 0.0003 |
| Heart trimmings | Pet food | 0.0010 | 0.0015 | 0.0001 |
| Hide | Skin tannery C3 | 0.0014 | 0.0015 | 0.0025 |
| Hooves | PAP C3 | 0.0037 | 0.0015 | 0.0001 |
| Horns | PAP C3 | 0.0037 | 0.0015 | 0.0001 |
| Kidney | Human food | 0.0010 | 0.0015 | 0.0006 |
| Large intestine | C1-C2 for disposal | 0.0000 | 0.0000 | 0.0000 |
| Liver | Human food | 0.0045 | 0.0015 | 0.0007 |
| Liver trimmings | Pet food | 0.0047 | 0.0015 | 0.0001 |
| Lower jaw | PAP C3 | 0.0009 | 0.0015 | 0.0001 |
| Lungs | Pet food | 0.0012 | 0.0015 | 0.0001 |
| Mask | Skin tannery C3 | 0.0014 | 0.0015 | 0.0025 |
| Mesenteric fat | C1-C2 for disposal | 0.0000 | 0.0000 | 0.0000 |
| Muscle | Human food | 0.0010 | 0.0015 | 0.0025 |
| Muzzle | Human food | 0.0014 | 0.0015 | 0.0015 |
| Omasum | Human food | 0.0087 | 0.0015 | 0.0011 |
| Omasum fat | Fat and greaves C3 | 0.0032 | 0.0015 | 0.0001 |
| Rumen and forestomach | Human food | 0.0087 | 0.0015 | 0.0011 |
| Rumen fat | Fat and greaves C3 | 0.0032 | 0.0015 | 0.0001 |
| Sanitary seizures | C1-C2 for disposal | 0.0000 | 0.0000 | 0.0000 |
| Screening and sifting wastes | C1-C2 for disposal | 0.0000 | 0.0000 | 0.0000 |
| Small intestine | PAP C3 | 0.0087 | 0.0015 | 0.0001 |
| Spinal cord | C1-C2 for disposal | 0.0000 | 0.0000 | 0.0000 |
| Spinal cord waste | C1-C2 for disposal | 0.0000 | 0.0000 | 0.0000 |
| Spine | C1-C2 for disposal | 0.0000 | 0.0000 | 0.0000 |
| Spleen | Pet food | 0.0010 | 0.0015 | 0.0001 |
| Stillborn | PAP C3 | 0.0091 | 0.0015 | 0.0000 |
| Tallow | Fat and greaves C3 | 0.0014 | 0.0015 | 0.0001 |
| Tongue | Human food | 0.0009 | 0.0015 | 0.0024 |
| Tonsil | C1-C2 for disposal | 0.0000 | 0.0000 | 0.0000 |
| Trachea | Pet food | 0.0014 | 0.0015 | 0.0001 |
| Udder | Pet food | 0.0002 | 0.0015 | 0.0001 |
| Upper throat | Pet food | 0.0010 | 0.0015 | 0.0001 |
| Water in the rumen | Spreading/Compost | 0.0000 | 0.0000 | 0.0000 |

Table 62: Allocation factors for Salers Heifers reared in Grazing Large Area

| COPRODUCT | Destination | Salers/heifer/grazing large area | | |
| --- | --- | --- | --- | --- |
|  |  | **Biophysical Allocation Factor** | **Mass Allocation Factor** | **Economic Allocation Factor** |
| Abomasum | Human food | 0.0099 | 0.0018 | 0.0014 |
| Abomasum fat | Fat and greaves C3 | 0.0038 | 0.0018 | 0.0002 |
| Aponeurosis | Human food | 0.0014 | 0.0018 | 0.0018 |
| Bile | PAP C3 | 0.0005 | 0.0018 | 0.0002 |
| Blood | PAP C3 | 0.0010 | 0.0018 | 0.0004 |
| Blood | Pet food | 0.0010 | 0.0018 | 0.0001 |
| Bones | Gelatin C3 | 0.0011 | 0.0018 | 0.0000 |
| Bones of head, brain, eyes and teeth | C1-C2 for disposal | 0.0000 | 0.0000 | 0.0000 |
| Cheek | Human food | 0.0012 | 0.0018 | 0.0040 |
| Cheek | Human food | 0.0012 | 0.0018 | 0.0040 |
| Cheek trimmings | Pet food | 0.0012 | 0.0018 | 0.0001 |
| Chops | Pet food | 0.0017 | 0.0018 | 0.0001 |
| Contents of intestines | Spreading/Compost | 0.0000 | 0.0000 | 0.0000 |
| Contents of the rumen | Spreading/Compost | 0.0000 | 0.0000 | 0.0000 |
| Ears | PAP C3 | 0.0015 | 0.0018 | 0.0002 |
| Esophagus | Pet food | 0.0012 | 0.0018 | 0.0001 |
| Fat | Fat and greaves C3 | 0.0017 | 0.0018 | 0.0002 |
| Fat around heart | Fat and greaves C3 | 0.0017 | 0.0018 | 0.0002 |
| Fat in the kidney | Fat and greaves C3 | 0.0017 | 0.0018 | 0.0002 |
| Feet (without hooves) | Gelatin C3 | 0.0012 | 0.0018 | 0.0000 |
| Floatation fat | Spreading/Compost | 0.0000 | 0.0000 | 0.0000 |
| Forehead | C1-C2 for disposal | 0.0000 | 0.0000 | 0.0000 |
| Forelock | PAP C3 | 0.0045 | 0.0018 | 0.0002 |
| Gallbladder | Pet food | 0.0012 | 0.0018 | 0.0001 |
| Head trimmings | Pet food | 0.0012 | 0.0018 | 0.0001 |
| Heart | Human food | 0.0012 | 0.0018 | 0.0004 |
| Heart trimmings | Pet food | 0.0012 | 0.0018 | 0.0001 |
| Hide | Skin tannery C3 | 0.0017 | 0.0018 | 0.0031 |
| Hooves | PAP C3 | 0.0045 | 0.0018 | 0.0002 |
| Horns | PAP C3 | 0.0045 | 0.0018 | 0.0002 |
| Kidney | Human food | 0.0012 | 0.0018 | 0.0007 |
| Large intestine | C1-C2 for disposal | 0.0000 | 0.0000 | 0.0000 |
| Liver | Human food | 0.0052 | 0.0018 | 0.0009 |
| Liver trimmings | Pet food | 0.0054 | 0.0018 | 0.0001 |
| Lower jaw | PAP C3 | 0.0011 | 0.0018 | 0.0002 |
| Lungs | Pet food | 0.0014 | 0.0018 | 0.0001 |
| Mask | Skin tannery C3 | 0.0017 | 0.0018 | 0.0031 |
| Mesenteric fat | C1-C2 for disposal | 0.0000 | 0.0000 | 0.0000 |
| Muscle | Human food | 0.0012 | 0.0018 | 0.0031 |
| Muzzle | Human food | 0.0017 | 0.0018 | 0.0018 |
| Omasum | Human food | 0.0099 | 0.0018 | 0.0014 |
| Omasum fat | Fat and greaves C3 | 0.0038 | 0.0018 | 0.0002 |
| Rumen and forestomach | Human food | 0.0099 | 0.0018 | 0.0014 |
| Rumen fat | Fat and greaves C3 | 0.0038 | 0.0018 | 0.0002 |
| Sanitary seizures | C1-C2 for disposal | 0.0000 | 0.0000 | 0.0000 |
| Screening and sifting wastes | C1-C2 for disposal | 0.0000 | 0.0000 | 0.0000 |
| Small intestine | PAP C3 | 0.0099 | 0.0018 | 0.0001 |
| Spinal cord | C1-C2 for disposal | 0.0000 | 0.0000 | 0.0000 |
| Spinal cord waste | C1-C2 for disposal | 0.0000 | 0.0000 | 0.0000 |
| Spine | C1-C2 for disposal | 0.0000 | 0.0000 | 0.0000 |
| Spleen | Pet food | 0.0013 | 0.0018 | 0.0001 |
| Stillborn | PAP C3 | 0.0104 | 0.0018 | 0.0000 |
| Tallow | Fat and greaves C3 | 0.0017 | 0.0018 | 0.0002 |
| Tongue | Human food | 0.0011 | 0.0018 | 0.0029 |
| Tonsil | C1-C2 for disposal | 0.0000 | 0.0000 | 0.0000 |
| Trachea | Pet food | 0.0017 | 0.0018 | 0.0001 |
| Udder | Pet food | 0.0003 | 0.0018 | 0.0001 |
| Upper throat | Pet food | 0.0012 | 0.0018 | 0.0001 |
| Water in the rumen | Spreading/Compost | 0.0000 | 0.0000 | 0.0000 |

Table 63: Allocation factors for Salers Cull Cows reared in Grazing Large Area

| COPRODUCT | Destination | Salers/Cull cow/grazing large area | | |
| --- | --- | --- | --- | --- |
|  |  | **Biophysical Allocation Factor** | **Mass Allocation Factor** | **Economic Allocation Factor** |
| Abomasum | Human food | 0.0089 | 0.0016 | 0.0012 |
| Abomasum fat | Fat and greaves C3 | 0.0033 | 0.0016 | 0.0002 |
| Aponeurosis | Human food | 0.0012 | 0.0016 | 0.0017 |
| Bile | PAP C3 | 0.0004 | 0.0016 | 0.0001 |
| Blood | PAP C3 | 0.0009 | 0.0016 | 0.0004 |
| Blood | Pet food | 0.0009 | 0.0016 | 0.0001 |
| Bones | Gelatin C3 | 0.0010 | 0.0016 | 0.0000 |
| Bones of head, brain, eyes and teeth | C1-C2 for disposal | 0.0000 | 0.0000 | 0.0000 |
| Cheek | Human food | 0.0011 | 0.0016 | 0.0036 |
| Cheek | Human food | 0.0011 | 0.0016 | 0.0036 |
| Cheek trimmings | Pet food | 0.0011 | 0.0016 | 0.0001 |
| Chops | Pet food | 0.0015 | 0.0016 | 0.0001 |
| Contents of intestines | Spreading/Compost | 0.0000 | 0.0000 | 0.0000 |
| Contents of the rumen | Spreading/Compost | 0.0000 | 0.0000 | 0.0000 |
| Ears | PAP C3 | 0.0013 | 0.0016 | 0.0001 |
| Esophagus | Pet food | 0.0010 | 0.0016 | 0.0001 |
| Fat | Fat and greaves C3 | 0.0014 | 0.0016 | 0.0002 |
| Fat around heart | Fat and greaves C3 | 0.0014 | 0.0016 | 0.0002 |
| Fat in the kidney | Fat and greaves C3 | 0.0014 | 0.0016 | 0.0002 |
| Feet (without hooves) | Gelatin C3 | 0.0011 | 0.0016 | 0.0000 |
| Floatation fat | Spreading/Compost | 0.0000 | 0.0000 | 0.0000 |
| Forehead | C1-C2 for disposal | 0.0000 | 0.0000 | 0.0000 |
| Forelock | PAP C3 | 0.0039 | 0.0016 | 0.0001 |
| Gallbladder | Pet food | 0.0011 | 0.0016 | 0.0001 |
| Head trimmings | Pet food | 0.0011 | 0.0016 | 0.0001 |
| Heart | Human food | 0.0010 | 0.0016 | 0.0004 |
| Heart trimmings | Pet food | 0.0011 | 0.0016 | 0.0001 |
| Hide | Skin tannery C3 | 0.0015 | 0.0016 | 0.0028 |
| Hooves | PAP C3 | 0.0039 | 0.0016 | 0.0001 |
| Horns | PAP C3 | 0.0039 | 0.0016 | 0.0001 |
| Kidney | Human food | 0.0011 | 0.0016 | 0.0007 |
| Large intestine | C1-C2 for disposal | 0.0000 | 0.0000 | 0.0000 |
| Liver | Human food | 0.0046 | 0.0016 | 0.0008 |
| Liver trimmings | Pet food | 0.0048 | 0.0016 | 0.0001 |
| Lower jaw | PAP C3 | 0.0010 | 0.0016 | 0.0001 |
| Lungs | Pet food | 0.0012 | 0.0016 | 0.0001 |
| Mask | Skin tannery C3 | 0.0015 | 0.0016 | 0.0028 |
| Mesenteric fat | C1-C2 for disposal | 0.0000 | 0.0000 | 0.0000 |
| Muscle | Human food | 0.0011 | 0.0016 | 0.0028 |
| Muzzle | Human food | 0.0015 | 0.0016 | 0.0017 |
| Omasum | Human food | 0.0089 | 0.0016 | 0.0012 |
| Omasum fat | Fat and greaves C3 | 0.0033 | 0.0016 | 0.0002 |
| Rumen and forestomach | Human food | 0.0089 | 0.0016 | 0.0012 |
| Rumen fat | Fat and greaves C3 | 0.0033 | 0.0016 | 0.0002 |
| Sanitary seizures | C1-C2 for disposal | 0.0000 | 0.0000 | 0.0000 |
| Screening and sifting wastes | C1-C2 for disposal | 0.0000 | 0.0000 | 0.0000 |
| Small intestine | PAP C3 | 0.0089 | 0.0016 | 0.0001 |
| Spinal cord | C1-C2 for disposal | 0.0000 | 0.0000 | 0.0000 |
| Spinal cord waste | C1-C2 for disposal | 0.0000 | 0.0000 | 0.0000 |
| Spine | C1-C2 for disposal | 0.0000 | 0.0000 | 0.0000 |
| Spleen | Pet food | 0.0011 | 0.0016 | 0.0001 |
| Stillborn | PAP C3 | 0.0093 | 0.0016 | 0.0000 |
| Tallow | Fat and greaves C3 | 0.0014 | 0.0016 | 0.0002 |
| Tongue | Human food | 0.0009 | 0.0016 | 0.0026 |
| Tonsil | C1-C2 for disposal | 0.0000 | 0.0000 | 0.0000 |
| Trachea | Pet food | 0.0015 | 0.0016 | 0.0001 |
| Udder | Pet food | 0.0002 | 0.0016 | 0.0001 |
| Upper throat | Pet food | 0.0011 | 0.0016 | 0.0001 |
| Water in the rumen | Spreading/Compost | 0.0000 | 0.0000 | 0.0000 |

Table 64: Allocation factors for Salers Beef reared in Grazing Large Area

| COPRODUCT | Destination | Salers/beef/grazing large area | | |
| --- | --- | --- | --- | --- |
|  |  | **Biophysical Allocation Factor** | **Mass Allocation Factor** | **Economic Allocation Factor** |
| Abomasum | Human food | 0.0083 | 0.0014 | 0.0011 |
| Abomasum fat | Fat and greaves C3 | 0.0030 | 0.0014 | 0.0001 |
| Aponeurosis | Human food | 0.0011 | 0.0014 | 0.0014 |
| Bile | PAP C3 | 0.0004 | 0.0014 | 0.0001 |
| Blood | PAP C3 | 0.0008 | 0.0014 | 0.0003 |
| Blood | Pet food | 0.0008 | 0.0014 | 0.0001 |
| Bones | Gelatin C3 | 0.0009 | 0.0014 | 0.0000 |
| Bones of head, brain, eyes and teeth | C1-C2 for disposal | 0.0000 | 0.0000 | 0.0000 |
| Cheek | Human food | 0.0010 | 0.0014 | 0.0031 |
| Cheek | Human food | 0.0010 | 0.0014 | 0.0031 |
| Cheek trimmings | Pet food | 0.0010 | 0.0014 | 0.0001 |
| Chops | Pet food | 0.0013 | 0.0014 | 0.0001 |
| Contents of intestines | Spreading/Compost | 0.0000 | 0.0000 | 0.0000 |
| Contents of the rumen | Spreading/Compost | 0.0000 | 0.0000 | 0.0000 |
| Ears | PAP C3 | 0.0012 | 0.0014 | 0.0001 |
| Esophagus | Pet food | 0.0009 | 0.0014 | 0.0001 |
| Fat | Fat and greaves C3 | 0.0012 | 0.0014 | 0.0001 |
| Fat around heart | Fat and greaves C3 | 0.0012 | 0.0014 | 0.0001 |
| Fat in the kidney | Fat and greaves C3 | 0.0012 | 0.0014 | 0.0001 |
| Feet (without hooves) | Gelatin C3 | 0.0009 | 0.0014 | 0.0000 |
| Floatation fat | Spreading/Compost | 0.0000 | 0.0000 | 0.0000 |
| Forehead | C1-C2 for disposal | 0.0000 | 0.0000 | 0.0000 |
| Forelock | PAP C3 | 0.0035 | 0.0014 | 0.0001 |
| Gallbladder | Pet food | 0.0009 | 0.0014 | 0.0001 |
| Head trimmings | Pet food | 0.0010 | 0.0014 | 0.0001 |
| Heart | Human food | 0.0009 | 0.0014 | 0.0003 |
| Heart trimmings | Pet food | 0.0010 | 0.0014 | 0.0001 |
| Hide | Skin tannery C3 | 0.0013 | 0.0014 | 0.0024 |
| Hooves | PAP C3 | 0.0035 | 0.0014 | 0.0001 |
| Horns | PAP C3 | 0.0035 | 0.0014 | 0.0001 |
| Kidney | Human food | 0.0009 | 0.0014 | 0.0006 |
| Large intestine | C1-C2 for disposal | 0.0000 | 0.0000 | 0.0000 |
| Liver | Human food | 0.0043 | 0.0014 | 0.0007 |
| Liver trimmings | Pet food | 0.0045 | 0.0014 | 0.0001 |
| Lower jaw | PAP C3 | 0.0009 | 0.0014 | 0.0001 |
| Lungs | Pet food | 0.0011 | 0.0014 | 0.0001 |
| Mask | Skin tannery C3 | 0.0013 | 0.0014 | 0.0024 |
| Mesenteric fat | C1-C2 for disposal | 0.0000 | 0.0000 | 0.0000 |
| Muscle | Human food | 0.0010 | 0.0014 | 0.0024 |
| Muzzle | Human food | 0.0013 | 0.0014 | 0.0014 |
| Omasum | Human food | 0.0083 | 0.0014 | 0.0011 |
| Omasum fat | Fat and greaves C3 | 0.0030 | 0.0014 | 0.0001 |
| Rumen and forestomach | Human food | 0.0083 | 0.0014 | 0.0011 |
| Rumen fat | Fat and greaves C3 | 0.0030 | 0.0014 | 0.0001 |
| Sanitary seizures | C1-C2 for disposal | 0.0000 | 0.0000 | 0.0000 |
| Screening and sifting wastes | C1-C2 for disposal | 0.0000 | 0.0000 | 0.0000 |
| Small intestine | PAP C3 | 0.0083 | 0.0014 | 0.0001 |
| Spinal cord | C1-C2 for disposal | 0.0000 | 0.0000 | 0.0000 |
| Spinal cord waste | C1-C2 for disposal | 0.0000 | 0.0000 | 0.0000 |
| Spine | C1-C2 for disposal | 0.0000 | 0.0000 | 0.0000 |
| Spleen | Pet food | 0.0010 | 0.0014 | 0.0001 |
| Stillborn | PAP C3 | 0.0087 | 0.0014 | 0.0000 |
| Tallow | Fat and greaves C3 | 0.0012 | 0.0014 | 0.0001 |
| Tongue | Human food | 0.0008 | 0.0014 | 0.0023 |
| Tonsil | C1-C2 for disposal | 0.0000 | 0.0000 | 0.0000 |
| Trachea | Pet food | 0.0013 | 0.0014 | 0.0001 |
| Udder | Pet food | 0.0002 | 0.0014 | 0.0001 |
| Upper throat | Pet food | 0.0009 | 0.0014 | 0.0001 |
| Water in the rumen | Spreading/Compost | 0.0000 | 0.0000 | 0.0000 |

Table 65: Allocation factors for Salers Young Bulls reared in Pasture

| COPRODUCT | Destination | Salers/young bull/pasture | | |
| --- | --- | --- | --- | --- |
|  |  | **Biophysical Allocation Factor** | **Mass Allocation Factor** | **Economic Allocation Factor** |
| Abomasum | Human food | 0.0084 | 0.0015 | 0.0011 |
| Abomasum fat | Fat and greaves C3 | 0.0033 | 0.0015 | 0.0001 |
| Aponeurosis | Human food | 0.0011 | 0.0015 | 0.0015 |
| Bile | PAP C3 | 0.0004 | 0.0015 | 0.0001 |
| Blood | PAP C3 | 0.0009 | 0.0015 | 0.0003 |
| Blood | Pet food | 0.0009 | 0.0015 | 0.0001 |
| Bones | Gelatin C3 | 0.0009 | 0.0015 | 0.0000 |
| Bones of head, brain, eyes and teeth | C1-C2 for disposal | 0.0000 | 0.0000 | 0.0000 |
| Cheek | Human food | 0.0010 | 0.0015 | 0.0033 |
| Cheek | Human food | 0.0010 | 0.0015 | 0.0033 |
| Cheek trimmings | Pet food | 0.0010 | 0.0015 | 0.0001 |
| Chops | Pet food | 0.0014 | 0.0015 | 0.0001 |
| Contents of intestines | Spreading/Compost | 0.0000 | 0.0000 | 0.0000 |
| Contents of the rumen | Spreading/Compost | 0.0000 | 0.0000 | 0.0000 |
| Ears | PAP C3 | 0.0013 | 0.0015 | 0.0001 |
| Esophagus | Pet food | 0.0010 | 0.0015 | 0.0001 |
| Fat | Fat and greaves C3 | 0.0015 | 0.0015 | 0.0001 |
| Fat around heart | Fat and greaves C3 | 0.0015 | 0.0015 | 0.0001 |
| Fat in the kidney | Fat and greaves C3 | 0.0015 | 0.0015 | 0.0001 |
| Feet (without hooves) | Gelatin C3 | 0.0010 | 0.0015 | 0.0000 |
| Floatation fat | Spreading/Compost | 0.0000 | 0.0000 | 0.0000 |
| Forehead | C1-C2 for disposal | 0.0000 | 0.0000 | 0.0000 |
| Forelock | PAP C3 | 0.0036 | 0.0015 | 0.0001 |
| Gallbladder | Pet food | 0.0010 | 0.0015 | 0.0001 |
| Head trimmings | Pet food | 0.0010 | 0.0015 | 0.0001 |
| Heart | Human food | 0.0010 | 0.0015 | 0.0003 |
| Heart trimmings | Pet food | 0.0010 | 0.0015 | 0.0001 |
| Hide | Skin tannery C3 | 0.0014 | 0.0015 | 0.0025 |
| Hooves | PAP C3 | 0.0036 | 0.0015 | 0.0001 |
| Horns | PAP C3 | 0.0036 | 0.0015 | 0.0001 |
| Kidney | Human food | 0.0010 | 0.0015 | 0.0006 |
| Large intestine | C1-C2 for disposal | 0.0000 | 0.0000 | 0.0000 |
| Liver | Human food | 0.0043 | 0.0015 | 0.0007 |
| Liver trimmings | Pet food | 0.0045 | 0.0015 | 0.0001 |
| Lower jaw | PAP C3 | 0.0009 | 0.0015 | 0.0001 |
| Lungs | Pet food | 0.0012 | 0.0015 | 0.0001 |
| Mask | Skin tannery C3 | 0.0014 | 0.0015 | 0.0025 |
| Mesenteric fat | C1-C2 for disposal | 0.0000 | 0.0000 | 0.0000 |
| Muscle | Human food | 0.0010 | 0.0015 | 0.0025 |
| Muzzle | Human food | 0.0014 | 0.0015 | 0.0015 |
| Omasum | Human food | 0.0084 | 0.0015 | 0.0011 |
| Omasum fat | Fat and greaves C3 | 0.0033 | 0.0015 | 0.0001 |
| Rumen and forestomach | Human food | 0.0084 | 0.0015 | 0.0011 |
| Rumen fat | Fat and greaves C3 | 0.0033 | 0.0015 | 0.0001 |
| Sanitary seizures | C1-C2 for disposal | 0.0000 | 0.0000 | 0.0000 |
| Screening and sifting wastes | C1-C2 for disposal | 0.0000 | 0.0000 | 0.0000 |
| Small intestine | PAP C3 | 0.0084 | 0.0015 | 0.0001 |
| Spinal cord | C1-C2 for disposal | 0.0000 | 0.0000 | 0.0000 |
| Spinal cord waste | C1-C2 for disposal | 0.0000 | 0.0000 | 0.0000 |
| Spine | C1-C2 for disposal | 0.0000 | 0.0000 | 0.0000 |
| Spleen | Pet food | 0.0010 | 0.0015 | 0.0001 |
| Stillborn | PAP C3 | 0.0087 | 0.0015 | 0.0000 |
| Tallow | Fat and greaves C3 | 0.0015 | 0.0015 | 0.0001 |
| Tongue | Human food | 0.0009 | 0.0015 | 0.0024 |
| Tonsil | C1-C2 for disposal | 0.0000 | 0.0000 | 0.0000 |
| Trachea | Pet food | 0.0014 | 0.0015 | 0.0001 |
| Udder | Pet food | 0.0002 | 0.0015 | 0.0001 |
| Upper throat | Pet food | 0.0010 | 0.0015 | 0.0001 |
| Water in the rumen | Spreading/Compost | 0.0000 | 0.0000 | 0.0000 |

Table 66: Allocation factors for Salers Heifers reared in Pasture

| COPRODUCT | Destination | Salers/heifer/pasture | | |
| --- | --- | --- | --- | --- |
|  |  | **Biophysical Allocation Factor** | **Mass Allocation Factor** | **Economic Allocation Factor** |
| Abomasum | Human food | 0.0095 | 0.0018 | 0.0014 |
| Abomasum fat | Fat and greaves C3 | 0.0038 | 0.0018 | 0.0002 |
| Aponeurosis | Human food | 0.0014 | 0.0018 | 0.0018 |
| Bile | PAP C3 | 0.0005 | 0.0018 | 0.0002 |
| Blood | PAP C3 | 0.0010 | 0.0018 | 0.0004 |
| Blood | Pet food | 0.0010 | 0.0018 | 0.0001 |
| Bones | Gelatin C3 | 0.0011 | 0.0018 | 0.0000 |
| Bones of head, brain, eyes and teeth | C1-C2 for disposal | 0.0000 | 0.0000 | 0.0000 |
| Cheek | Human food | 0.0012 | 0.0018 | 0.0040 |
| Cheek | Human food | 0.0012 | 0.0018 | 0.0040 |
| Cheek trimmings | Pet food | 0.0012 | 0.0018 | 0.0001 |
| Chops | Pet food | 0.0017 | 0.0018 | 0.0001 |
| Contents of intestines | Spreading/Compost | 0.0000 | 0.0000 | 0.0000 |
| Contents of the rumen | Spreading/Compost | 0.0000 | 0.0000 | 0.0000 |
| Ears | PAP C3 | 0.0015 | 0.0018 | 0.0002 |
| Esophagus | Pet food | 0.0012 | 0.0018 | 0.0001 |
| Fat | Fat and greaves C3 | 0.0018 | 0.0018 | 0.0002 |
| Fat around heart | Fat and greaves C3 | 0.0018 | 0.0018 | 0.0002 |
| Fat in the kidney | Fat and greaves C3 | 0.0018 | 0.0018 | 0.0002 |
| Feet (without hooves) | Gelatin C3 | 0.0012 | 0.0018 | 0.0000 |
| Floatation fat | Spreading/Compost | 0.0000 | 0.0000 | 0.0000 |
| Forehead | C1-C2 for disposal | 0.0000 | 0.0000 | 0.0000 |
| Forelock | PAP C3 | 0.0044 | 0.0018 | 0.0002 |
| Gallbladder | Pet food | 0.0012 | 0.0018 | 0.0001 |
| Head trimmings | Pet food | 0.0012 | 0.0018 | 0.0001 |
| Heart | Human food | 0.0012 | 0.0018 | 0.0004 |
| Heart trimmings | Pet food | 0.0012 | 0.0018 | 0.0001 |
| Hide | Skin tannery C3 | 0.0017 | 0.0018 | 0.0031 |
| Hooves | PAP C3 | 0.0044 | 0.0018 | 0.0002 |
| Horns | PAP C3 | 0.0044 | 0.0018 | 0.0002 |
| Kidney | Human food | 0.0012 | 0.0018 | 0.0007 |
| Large intestine | C1-C2 for disposal | 0.0000 | 0.0000 | 0.0000 |
| Liver | Human food | 0.0050 | 0.0018 | 0.0009 |
| Liver trimmings | Pet food | 0.0052 | 0.0018 | 0.0001 |
| Lower jaw | PAP C3 | 0.0011 | 0.0018 | 0.0002 |
| Lungs | Pet food | 0.0014 | 0.0018 | 0.0001 |
| Mask | Skin tannery C3 | 0.0017 | 0.0018 | 0.0031 |
| Mesenteric fat | C1-C2 for disposal | 0.0000 | 0.0000 | 0.0000 |
| Muscle | Human food | 0.0012 | 0.0018 | 0.0031 |
| Muzzle | Human food | 0.0017 | 0.0018 | 0.0018 |
| Omasum | Human food | 0.0095 | 0.0018 | 0.0014 |
| Omasum fat | Fat and greaves C3 | 0.0038 | 0.0018 | 0.0002 |
| Rumen and forestomach | Human food | 0.0095 | 0.0018 | 0.0014 |
| Rumen fat | Fat and greaves C3 | 0.0038 | 0.0018 | 0.0002 |
| Sanitary seizures | C1-C2 for disposal | 0.0000 | 0.0000 | 0.0000 |
| Screening and sifting wastes | C1-C2 for disposal | 0.0000 | 0.0000 | 0.0000 |
| Small intestine | PAP C3 | 0.0095 | 0.0018 | 0.0001 |
| Spinal cord | C1-C2 for disposal | 0.0000 | 0.0000 | 0.0000 |
| Spinal cord waste | C1-C2 for disposal | 0.0000 | 0.0000 | 0.0000 |
| Spine | C1-C2 for disposal | 0.0000 | 0.0000 | 0.0000 |
| Spleen | Pet food | 0.0012 | 0.0018 | 0.0001 |
| Stillborn | PAP C3 | 0.0099 | 0.0018 | 0.0000 |
| Tallow | Fat and greaves C3 | 0.0018 | 0.0018 | 0.0002 |
| Tongue | Human food | 0.0011 | 0.0018 | 0.0029 |
| Tonsil | C1-C2 for disposal | 0.0000 | 0.0000 | 0.0000 |
| Trachea | Pet food | 0.0017 | 0.0018 | 0.0001 |
| Udder | Pet food | 0.0003 | 0.0018 | 0.0001 |
| Upper throat | Pet food | 0.0012 | 0.0018 | 0.0001 |
| Water in the rumen | Spreading/Compost | 0.0000 | 0.0000 | 0.0000 |

Table 67: Allocation factors for Salers Cull Cows reared in Pasture

| COPRODUCT | Destination | Salers/Cull cow/pasture | | |
| --- | --- | --- | --- | --- |
|  |  | **Biophysical Allocation Factor** | **Mass Allocation Factor** | **Economic Allocation Factor** |
| Abomasum | Human food | 0.0086 | 0.0016 | 0.0012 |
| Abomasum fat | Fat and greaves C3 | 0.0034 | 0.0016 | 0.0002 |
| Aponeurosis | Human food | 0.0012 | 0.0016 | 0.0017 |
| Bile | PAP C3 | 0.0004 | 0.0016 | 0.0001 |
| Blood | PAP C3 | 0.0009 | 0.0016 | 0.0004 |
| Blood | Pet food | 0.0009 | 0.0016 | 0.0001 |
| Bones | Gelatin C3 | 0.0010 | 0.0016 | 0.0000 |
| Bones of head, brain, eyes and teeth | C1-C2 for disposal | 0.0000 | 0.0000 | 0.0000 |
| Cheek | Human food | 0.0011 | 0.0016 | 0.0036 |
| Cheek | Human food | 0.0011 | 0.0016 | 0.0036 |
| Cheek trimmings | Pet food | 0.0011 | 0.0016 | 0.0001 |
| Chops | Pet food | 0.0015 | 0.0016 | 0.0001 |
| Contents of intestines | Spreading/Compost | 0.0000 | 0.0000 | 0.0000 |
| Contents of the rumen | Spreading/Compost | 0.0000 | 0.0000 | 0.0000 |
| Ears | PAP C3 | 0.0013 | 0.0016 | 0.0001 |
| Esophagus | Pet food | 0.0010 | 0.0016 | 0.0001 |
| Fat | Fat and greaves C3 | 0.0016 | 0.0016 | 0.0002 |
| Fat around heart | Fat and greaves C3 | 0.0016 | 0.0016 | 0.0002 |
| Fat in the kidney | Fat and greaves C3 | 0.0016 | 0.0016 | 0.0002 |
| Feet (without hooves) | Gelatin C3 | 0.0011 | 0.0016 | 0.0000 |
| Floatation fat | Spreading/Compost | 0.0000 | 0.0000 | 0.0000 |
| Forehead | C1-C2 for disposal | 0.0000 | 0.0000 | 0.0000 |
| Forelock | PAP C3 | 0.0038 | 0.0016 | 0.0001 |
| Gallbladder | Pet food | 0.0011 | 0.0016 | 0.0001 |
| Head trimmings | Pet food | 0.0011 | 0.0016 | 0.0001 |
| Heart | Human food | 0.0010 | 0.0016 | 0.0004 |
| Heart trimmings | Pet food | 0.0011 | 0.0016 | 0.0001 |
| Hide | Skin tannery C3 | 0.0015 | 0.0016 | 0.0028 |
| Hooves | PAP C3 | 0.0038 | 0.0016 | 0.0001 |
| Horns | PAP C3 | 0.0038 | 0.0016 | 0.0001 |
| Kidney | Human food | 0.0011 | 0.0016 | 0.0007 |
| Large intestine | C1-C2 for disposal | 0.0000 | 0.0000 | 0.0000 |
| Liver | Human food | 0.0045 | 0.0016 | 0.0008 |
| Liver trimmings | Pet food | 0.0046 | 0.0016 | 0.0001 |
| Lower jaw | PAP C3 | 0.0010 | 0.0016 | 0.0001 |
| Lungs | Pet food | 0.0012 | 0.0016 | 0.0001 |
| Mask | Skin tannery C3 | 0.0015 | 0.0016 | 0.0028 |
| Mesenteric fat | C1-C2 for disposal | 0.0000 | 0.0000 | 0.0000 |
| Muscle | Human food | 0.0011 | 0.0016 | 0.0028 |
| Muzzle | Human food | 0.0015 | 0.0016 | 0.0017 |
| Omasum | Human food | 0.0086 | 0.0016 | 0.0012 |
| Omasum fat | Fat and greaves C3 | 0.0034 | 0.0016 | 0.0002 |
| Rumen and forestomach | Human food | 0.0086 | 0.0016 | 0.0012 |
| Rumen fat | Fat and greaves C3 | 0.0034 | 0.0016 | 0.0002 |
| Sanitary seizures | C1-C2 for disposal | 0.0000 | 0.0000 | 0.0000 |
| Screening and sifting wastes | C1-C2 for disposal | 0.0000 | 0.0000 | 0.0000 |
| Small intestine | PAP C3 | 0.0086 | 0.0016 | 0.0001 |
| Spinal cord | C1-C2 for disposal | 0.0000 | 0.0000 | 0.0000 |
| Spinal cord waste | C1-C2 for disposal | 0.0000 | 0.0000 | 0.0000 |
| Spine | C1-C2 for disposal | 0.0000 | 0.0000 | 0.0000 |
| Spleen | Pet food | 0.0011 | 0.0016 | 0.0001 |
| Stillborn | PAP C3 | 0.0089 | 0.0016 | 0.0000 |
| Tallow | Fat and greaves C3 | 0.0016 | 0.0016 | 0.0002 |
| Tongue | Human food | 0.0009 | 0.0016 | 0.0026 |
| Tonsil | C1-C2 for disposal | 0.0000 | 0.0000 | 0.0000 |
| Trachea | Pet food | 0.0015 | 0.0016 | 0.0001 |
| Udder | Pet food | 0.0002 | 0.0016 | 0.0001 |
| Upper throat | Pet food | 0.0011 | 0.0016 | 0.0001 |
| Water in the rumen | Spreading/Compost | 0.0000 | 0.0000 | 0.0000 |

Table 68: Allocation factors for Salers Beef reared in Pasture

| COPRODUCT | Destination | Salers/beef/pasture | | |
| --- | --- | --- | --- | --- |
|  |  | **Biophysical Allocation Factor** | **Mass Allocation Factor** | **Economic Allocation Factor** |
| Abomasum | Human food | 0.0080 | 0.0014 | 0.0011 |
| Abomasum fat | Fat and greaves C3 | 0.0031 | 0.0014 | 0.0001 |
| Aponeurosis | Human food | 0.0010 | 0.0014 | 0.0014 |
| Bile | PAP C3 | 0.0004 | 0.0014 | 0.0001 |
| Blood | PAP C3 | 0.0008 | 0.0014 | 0.0003 |
| Blood | Pet food | 0.0008 | 0.0014 | 0.0001 |
| Bones | Gelatin C3 | 0.0009 | 0.0014 | 0.0000 |
| Bones of head, brain, eyes and teeth | C1-C2 for disposal | 0.0000 | 0.0000 | 0.0000 |
| Cheek | Human food | 0.0010 | 0.0014 | 0.0031 |
| Cheek | Human food | 0.0010 | 0.0014 | 0.0031 |
| Cheek trimmings | Pet food | 0.0010 | 0.0014 | 0.0001 |
| Chops | Pet food | 0.0013 | 0.0014 | 0.0001 |
| Contents of intestines | Spreading/Compost | 0.0000 | 0.0000 | 0.0000 |
| Contents of the rumen | Spreading/Compost | 0.0000 | 0.0000 | 0.0000 |
| Ears | PAP C3 | 0.0012 | 0.0014 | 0.0001 |
| Esophagus | Pet food | 0.0009 | 0.0014 | 0.0001 |
| Fat | Fat and greaves C3 | 0.0014 | 0.0014 | 0.0001 |
| Fat around heart | Fat and greaves C3 | 0.0014 | 0.0014 | 0.0001 |
| Fat in the kidney | Fat and greaves C3 | 0.0014 | 0.0014 | 0.0001 |
| Feet (without hooves) | Gelatin C3 | 0.0009 | 0.0014 | 0.0000 |
| Floatation fat | Spreading/Compost | 0.0000 | 0.0000 | 0.0000 |
| Forehead | C1-C2 for disposal | 0.0000 | 0.0000 | 0.0000 |
| Forelock | PAP C3 | 0.0034 | 0.0014 | 0.0001 |
| Gallbladder | Pet food | 0.0009 | 0.0014 | 0.0001 |
| Head trimmings | Pet food | 0.0010 | 0.0014 | 0.0001 |
| Heart | Human food | 0.0009 | 0.0014 | 0.0003 |
| Heart trimmings | Pet food | 0.0010 | 0.0014 | 0.0001 |
| Hide | Skin tannery C3 | 0.0013 | 0.0014 | 0.0024 |
| Hooves | PAP C3 | 0.0034 | 0.0014 | 0.0001 |
| Horns | PAP C3 | 0.0034 | 0.0014 | 0.0001 |
| Kidney | Human food | 0.0009 | 0.0014 | 0.0006 |
| Large intestine | C1-C2 for disposal | 0.0000 | 0.0000 | 0.0000 |
| Liver | Human food | 0.0041 | 0.0014 | 0.0007 |
| Liver trimmings | Pet food | 0.0043 | 0.0014 | 0.0001 |
| Lower jaw | PAP C3 | 0.0009 | 0.0014 | 0.0001 |
| Lungs | Pet food | 0.0011 | 0.0014 | 0.0001 |
| Mask | Skin tannery C3 | 0.0013 | 0.0014 | 0.0024 |
| Mesenteric fat | C1-C2 for disposal | 0.0000 | 0.0000 | 0.0000 |
| Muscle | Human food | 0.0010 | 0.0014 | 0.0024 |
| Muzzle | Human food | 0.0013 | 0.0014 | 0.0014 |
| Omasum | Human food | 0.0080 | 0.0014 | 0.0011 |
| Omasum fat | Fat and greaves C3 | 0.0031 | 0.0014 | 0.0001 |
| Rumen and forestomach | Human food | 0.0080 | 0.0014 | 0.0011 |
| Rumen fat | Fat and greaves C3 | 0.0031 | 0.0014 | 0.0001 |
| Sanitary seizures | C1-C2 for disposal | 0.0000 | 0.0000 | 0.0000 |
| Screening and sifting wastes | C1-C2 for disposal | 0.0000 | 0.0000 | 0.0000 |
| Small intestine | PAP C3 | 0.0080 | 0.0014 | 0.0001 |
| Spinal cord | C1-C2 for disposal | 0.0000 | 0.0000 | 0.0000 |
| Spinal cord waste | C1-C2 for disposal | 0.0000 | 0.0000 | 0.0000 |
| Spine | C1-C2 for disposal | 0.0000 | 0.0000 | 0.0000 |
| Spleen | Pet food | 0.0010 | 0.0014 | 0.0001 |
| Stillborn | PAP C3 | 0.0083 | 0.0014 | 0.0000 |
| Tallow | Fat and greaves C3 | 0.0014 | 0.0014 | 0.0001 |
| Tongue | Human food | 0.0008 | 0.0014 | 0.0023 |
| Tonsil | C1-C2 for disposal | 0.0000 | 0.0000 | 0.0000 |
| Trachea | Pet food | 0.0013 | 0.0014 | 0.0001 |
| Udder | Pet food | 0.0002 | 0.0014 | 0.0001 |
| Upper throat | Pet food | 0.0009 | 0.0014 | 0.0001 |
| Water in the rumen | Spreading/Compost | 0.0000 | 0.0000 | 0.0000 |

Table 69: Allocation factors for Salers Young Bulls reared in Stall

| COPRODUCT | Destination | Salers/young bull/stall | | |
| --- | --- | --- | --- | --- |
|  |  | **Biophysical Allocation Factor** | **Mass Allocation Factor** | **Economic Allocation Factor** |
| Abomasum | Human food | 0.0079 | 0.0015 | 0.0011 |
| Abomasum fat | Fat and greaves C3 | 0.0033 | 0.0015 | 0.0001 |
| Aponeurosis | Human food | 0.0011 | 0.0015 | 0.0015 |
| Bile | PAP C3 | 0.0004 | 0.0015 | 0.0001 |
| Blood | PAP C3 | 0.0009 | 0.0015 | 0.0003 |
| Blood | Pet food | 0.0009 | 0.0015 | 0.0001 |
| Bones | Gelatin C3 | 0.0010 | 0.0015 | 0.0000 |
| Bones of head, brain, eyes and teeth | C1-C2 for disposal | 0.0000 | 0.0000 | 0.0000 |
| Cheek | Human food | 0.0010 | 0.0015 | 0.0033 |
| Cheek | Human food | 0.0010 | 0.0015 | 0.0033 |
| Cheek trimmings | Pet food | 0.0010 | 0.0015 | 0.0001 |
| Chops | Pet food | 0.0014 | 0.0015 | 0.0001 |
| Contents of intestines | Spreading/Compost | 0.0000 | 0.0000 | 0.0000 |
| Contents of the rumen | Spreading/Compost | 0.0000 | 0.0000 | 0.0000 |
| Ears | PAP C3 | 0.0013 | 0.0015 | 0.0001 |
| Esophagus | Pet food | 0.0010 | 0.0015 | 0.0001 |
| Fat | Fat and greaves C3 | 0.0016 | 0.0015 | 0.0001 |
| Fat around heart | Fat and greaves C3 | 0.0016 | 0.0015 | 0.0001 |
| Fat in the kidney | Fat and greaves C3 | 0.0016 | 0.0015 | 0.0001 |
| Feet (without hooves) | Gelatin C3 | 0.0010 | 0.0015 | 0.0000 |
| Floatation fat | Spreading/Compost | 0.0000 | 0.0000 | 0.0000 |
| Forehead | C1-C2 for disposal | 0.0000 | 0.0000 | 0.0000 |
| Forelock | PAP C3 | 0.0036 | 0.0015 | 0.0001 |
| Gallbladder | Pet food | 0.0010 | 0.0015 | 0.0001 |
| Head trimmings | Pet food | 0.0010 | 0.0015 | 0.0001 |
| Heart | Human food | 0.0010 | 0.0015 | 0.0003 |
| Heart trimmings | Pet food | 0.0010 | 0.0015 | 0.0001 |
| Hide | Skin tannery C3 | 0.0014 | 0.0015 | 0.0025 |
| Hooves | PAP C3 | 0.0036 | 0.0015 | 0.0001 |
| Horns | PAP C3 | 0.0036 | 0.0015 | 0.0001 |
| Kidney | Human food | 0.0010 | 0.0015 | 0.0006 |
| Large intestine | C1-C2 for disposal | 0.0000 | 0.0000 | 0.0000 |
| Liver | Human food | 0.0041 | 0.0015 | 0.0007 |
| Liver trimmings | Pet food | 0.0043 | 0.0015 | 0.0001 |
| Lower jaw | PAP C3 | 0.0009 | 0.0015 | 0.0001 |
| Lungs | Pet food | 0.0012 | 0.0015 | 0.0001 |
| Mask | Skin tannery C3 | 0.0014 | 0.0015 | 0.0025 |
| Mesenteric fat | C1-C2 for disposal | 0.0000 | 0.0000 | 0.0000 |
| Muscle | Human food | 0.0010 | 0.0015 | 0.0025 |
| Muzzle | Human food | 0.0014 | 0.0015 | 0.0015 |
| Omasum | Human food | 0.0079 | 0.0015 | 0.0011 |
| Omasum fat | Fat and greaves C3 | 0.0033 | 0.0015 | 0.0001 |
| Rumen and forestomach | Human food | 0.0079 | 0.0015 | 0.0011 |
| Rumen fat | Fat and greaves C3 | 0.0033 | 0.0015 | 0.0001 |
| Sanitary seizures | C1-C2 for disposal | 0.0000 | 0.0000 | 0.0000 |
| Screening and sifting wastes | C1-C2 for disposal | 0.0000 | 0.0000 | 0.0000 |
| Small intestine | PAP C3 | 0.0079 | 0.0015 | 0.0001 |
| Spinal cord | C1-C2 for disposal | 0.0000 | 0.0000 | 0.0000 |
| Spinal cord waste | C1-C2 for disposal | 0.0000 | 0.0000 | 0.0000 |
| Spine | C1-C2 for disposal | 0.0000 | 0.0000 | 0.0000 |
| Spleen | Pet food | 0.0010 | 0.0015 | 0.0001 |
| Stillborn | PAP C3 | 0.0083 | 0.0015 | 0.0000 |
| Tallow | Fat and greaves C3 | 0.0016 | 0.0015 | 0.0001 |
| Tongue | Human food | 0.0009 | 0.0015 | 0.0024 |
| Tonsil | C1-C2 for disposal | 0.0000 | 0.0000 | 0.0000 |
| Trachea | Pet food | 0.0014 | 0.0015 | 0.0001 |
| Udder | Pet food | 0.0002 | 0.0015 | 0.0001 |
| Upper throat | Pet food | 0.0010 | 0.0015 | 0.0001 |
| Water in the rumen | Spreading/Compost | 0.0000 | 0.0000 | 0.0000 |

Table 70: Allocation factors for Salers Heifers reared in Stall

| COPRODUCT | Destination | Salers/heifer/stall | | |
| --- | --- | --- | --- | --- |
|  |  | **Biophysical Allocation Factor** | **Mass Allocation Factor** | **Economic Allocation Factor** |
| Abomasum | Human food | 0.0090 | 0.0018 | 0.0014 |
| Abomasum fat | Fat and greaves C3 | 0.0038 | 0.0018 | 0.0002 |
| Aponeurosis | Human food | 0.0013 | 0.0018 | 0.0018 |
| Bile | PAP C3 | 0.0005 | 0.0018 | 0.0002 |
| Blood | PAP C3 | 0.0010 | 0.0018 | 0.0004 |
| Blood | Pet food | 0.0010 | 0.0018 | 0.0001 |
| Bones | Gelatin C3 | 0.0012 | 0.0018 | 0.0000 |
| Bones of head, brain, eyes and teeth | C1-C2 for disposal | 0.0000 | 0.0000 | 0.0000 |
| Cheek | Human food | 0.0012 | 0.0018 | 0.0040 |
| Cheek | Human food | 0.0012 | 0.0018 | 0.0040 |
| Cheek trimmings | Pet food | 0.0012 | 0.0018 | 0.0001 |
| Chops | Pet food | 0.0017 | 0.0018 | 0.0001 |
| Contents of intestines | Spreading/Compost | 0.0000 | 0.0000 | 0.0000 |
| Contents of the rumen | Spreading/Compost | 0.0000 | 0.0000 | 0.0000 |
| Ears | PAP C3 | 0.0015 | 0.0018 | 0.0002 |
| Esophagus | Pet food | 0.0012 | 0.0018 | 0.0001 |
| Fat | Fat and greaves C3 | 0.0020 | 0.0018 | 0.0002 |
| Fat around heart | Fat and greaves C3 | 0.0020 | 0.0018 | 0.0002 |
| Fat in the kidney | Fat and greaves C3 | 0.0020 | 0.0018 | 0.0002 |
| Feet (without hooves) | Gelatin C3 | 0.0012 | 0.0018 | 0.0000 |
| Floatation fat | Spreading/Compost | 0.0000 | 0.0000 | 0.0000 |
| Forehead | C1-C2 for disposal | 0.0000 | 0.0000 | 0.0000 |
| Forelock | PAP C3 | 0.0044 | 0.0018 | 0.0002 |
| Gallbladder | Pet food | 0.0012 | 0.0018 | 0.0001 |
| Head trimmings | Pet food | 0.0012 | 0.0018 | 0.0001 |
| Heart | Human food | 0.0012 | 0.0018 | 0.0004 |
| Heart trimmings | Pet food | 0.0012 | 0.0018 | 0.0001 |
| Hide | Skin tannery C3 | 0.0017 | 0.0018 | 0.0031 |
| Hooves | PAP C3 | 0.0044 | 0.0018 | 0.0002 |
| Horns | PAP C3 | 0.0044 | 0.0018 | 0.0002 |
| Kidney | Human food | 0.0012 | 0.0018 | 0.0007 |
| Large intestine | C1-C2 for disposal | 0.0000 | 0.0000 | 0.0000 |
| Liver | Human food | 0.0047 | 0.0018 | 0.0009 |
| Liver trimmings | Pet food | 0.0049 | 0.0018 | 0.0001 |
| Lower jaw | PAP C3 | 0.0011 | 0.0018 | 0.0002 |
| Lungs | Pet food | 0.0014 | 0.0018 | 0.0001 |
| Mask | Skin tannery C3 | 0.0017 | 0.0018 | 0.0031 |
| Mesenteric fat | C1-C2 for disposal | 0.0000 | 0.0000 | 0.0000 |
| Muscle | Human food | 0.0012 | 0.0018 | 0.0031 |
| Muzzle | Human food | 0.0017 | 0.0018 | 0.0018 |
| Omasum | Human food | 0.0090 | 0.0018 | 0.0014 |
| Omasum fat | Fat and greaves C3 | 0.0038 | 0.0018 | 0.0002 |
| Rumen and forestomach | Human food | 0.0090 | 0.0018 | 0.0014 |
| Rumen fat | Fat and greaves C3 | 0.0038 | 0.0018 | 0.0002 |
| Sanitary seizures | C1-C2 for disposal | 0.0000 | 0.0000 | 0.0000 |
| Screening and sifting wastes | C1-C2 for disposal | 0.0000 | 0.0000 | 0.0000 |
| Small intestine | PAP C3 | 0.0090 | 0.0018 | 0.0001 |
| Spinal cord | C1-C2 for disposal | 0.0000 | 0.0000 | 0.0000 |
| Spinal cord waste | C1-C2 for disposal | 0.0000 | 0.0000 | 0.0000 |
| Spine | C1-C2 for disposal | 0.0000 | 0.0000 | 0.0000 |
| Spleen | Pet food | 0.0012 | 0.0018 | 0.0001 |
| Stillborn | PAP C3 | 0.0094 | 0.0018 | 0.0000 |
| Tallow | Fat and greaves C3 | 0.0020 | 0.0018 | 0.0002 |
| Tongue | Human food | 0.0011 | 0.0018 | 0.0029 |
| Tonsil | C1-C2 for disposal | 0.0000 | 0.0000 | 0.0000 |
| Trachea | Pet food | 0.0017 | 0.0018 | 0.0001 |
| Udder | Pet food | 0.0003 | 0.0018 | 0.0001 |
| Upper throat | Pet food | 0.0012 | 0.0018 | 0.0001 |
| Water in the rumen | Spreading/Compost | 0.0000 | 0.0000 | 0.0000 |

Table 71: Allocation factors for Salers Cull Cows reared in Stall

| COPRODUCT | Destination | Salers/Cull cow/stall | | |
| --- | --- | --- | --- | --- |
|  |  | **Biophysical Allocation Factor** | **Mass Allocation Factor** | **Economic Allocation Factor** |
| Abomasum | Human food | 0.0081 | 0.0016 | 0.0012 |
| Abomasum fat | Fat and greaves C3 | 0.0034 | 0.0016 | 0.0002 |
| Aponeurosis | Human food | 0.0012 | 0.0016 | 0.0017 |
| Bile | PAP C3 | 0.0004 | 0.0016 | 0.0001 |
| Blood | PAP C3 | 0.0009 | 0.0016 | 0.0004 |
| Blood | Pet food | 0.0009 | 0.0016 | 0.0001 |
| Bones | Gelatin C3 | 0.0010 | 0.0016 | 0.0000 |
| Bones of head, brain, eyes and teeth | C1-C2 for disposal | 0.0000 | 0.0000 | 0.0000 |
| Cheek | Human food | 0.0011 | 0.0016 | 0.0036 |
| Cheek | Human food | 0.0011 | 0.0016 | 0.0036 |
| Cheek trimmings | Pet food | 0.0011 | 0.0016 | 0.0001 |
| Chops | Pet food | 0.0015 | 0.0016 | 0.0001 |
| Contents of intestines | Spreading/Compost | 0.0000 | 0.0000 | 0.0000 |
| Contents of the rumen | Spreading/Compost | 0.0000 | 0.0000 | 0.0000 |
| Ears | PAP C3 | 0.0013 | 0.0016 | 0.0001 |
| Esophagus | Pet food | 0.0010 | 0.0016 | 0.0001 |
| Fat | Fat and greaves C3 | 0.0017 | 0.0016 | 0.0002 |
| Fat around heart | Fat and greaves C3 | 0.0017 | 0.0016 | 0.0002 |
| Fat in the kidney | Fat and greaves C3 | 0.0017 | 0.0016 | 0.0002 |
| Feet (without hooves) | Gelatin C3 | 0.0011 | 0.0016 | 0.0000 |
| Floatation fat | Spreading/Compost | 0.0000 | 0.0000 | 0.0000 |
| Forehead | C1-C2 for disposal | 0.0000 | 0.0000 | 0.0000 |
| Forelock | PAP C3 | 0.0038 | 0.0016 | 0.0001 |
| Gallbladder | Pet food | 0.0011 | 0.0016 | 0.0001 |
| Head trimmings | Pet food | 0.0011 | 0.0016 | 0.0001 |
| Heart | Human food | 0.0010 | 0.0016 | 0.0004 |
| Heart trimmings | Pet food | 0.0011 | 0.0016 | 0.0001 |
| Hide | Skin tannery C3 | 0.0015 | 0.0016 | 0.0028 |
| Hooves | PAP C3 | 0.0038 | 0.0016 | 0.0001 |
| Horns | PAP C3 | 0.0038 | 0.0016 | 0.0001 |
| Kidney | Human food | 0.0010 | 0.0016 | 0.0007 |
| Large intestine | C1-C2 for disposal | 0.0000 | 0.0000 | 0.0000 |
| Liver | Human food | 0.0043 | 0.0016 | 0.0008 |
| Liver trimmings | Pet food | 0.0044 | 0.0016 | 0.0001 |
| Lower jaw | PAP C3 | 0.0010 | 0.0016 | 0.0001 |
| Lungs | Pet food | 0.0012 | 0.0016 | 0.0001 |
| Mask | Skin tannery C3 | 0.0015 | 0.0016 | 0.0028 |
| Mesenteric fat | C1-C2 for disposal | 0.0000 | 0.0000 | 0.0000 |
| Muscle | Human food | 0.0011 | 0.0016 | 0.0028 |
| Muzzle | Human food | 0.0015 | 0.0016 | 0.0017 |
| Omasum | Human food | 0.0081 | 0.0016 | 0.0012 |
| Omasum fat | Fat and greaves C3 | 0.0034 | 0.0016 | 0.0002 |
| Rumen and forestomach | Human food | 0.0081 | 0.0016 | 0.0012 |
| Rumen fat | Fat and greaves C3 | 0.0034 | 0.0016 | 0.0002 |
| Sanitary seizures | C1-C2 for disposal | 0.0000 | 0.0000 | 0.0000 |
| Screening and sifting wastes | C1-C2 for disposal | 0.0000 | 0.0000 | 0.0000 |
| Small intestine | PAP C3 | 0.0081 | 0.0016 | 0.0001 |
| Spinal cord | C1-C2 for disposal | 0.0000 | 0.0000 | 0.0000 |
| Spinal cord waste | C1-C2 for disposal | 0.0000 | 0.0000 | 0.0000 |
| Spine | C1-C2 for disposal | 0.0000 | 0.0000 | 0.0000 |
| Spleen | Pet food | 0.0011 | 0.0016 | 0.0001 |
| Stillborn | PAP C3 | 0.0085 | 0.0016 | 0.0000 |
| Tallow | Fat and greaves C3 | 0.0017 | 0.0016 | 0.0002 |
| Tongue | Human food | 0.0010 | 0.0016 | 0.0026 |
| Tonsil | C1-C2 for disposal | 0.0000 | 0.0000 | 0.0000 |
| Trachea | Pet food | 0.0015 | 0.0016 | 0.0001 |
| Udder | Pet food | 0.0002 | 0.0016 | 0.0001 |
| Upper throat | Pet food | 0.0011 | 0.0016 | 0.0001 |
| Water in the rumen | Spreading/Compost | 0.0000 | 0.0000 | 0.0000 |

Table 72: Allocation factors for Salers Beef reared in Stall

| COPRODUCT | Destination | Salers/beef/stall | | |
| --- | --- | --- | --- | --- |
|  |  | **Biophysical Allocation Factor** | **Mass Allocation Factor** | **Economic Allocation Factor** |
| Abomasum | Human food | 0.0076 | 0.0014 | 0.0011 |
| Abomasum fat | Fat and greaves C3 | 0.0031 | 0.0014 | 0.0001 |
| Aponeurosis | Human food | 0.0010 | 0.0014 | 0.0014 |
| Bile | PAP C3 | 0.0004 | 0.0014 | 0.0001 |
| Blood | PAP C3 | 0.0008 | 0.0014 | 0.0003 |
| Blood | Pet food | 0.0008 | 0.0014 | 0.0001 |
| Bones | Gelatin C3 | 0.0009 | 0.0014 | 0.0000 |
| Bones of head, brain, eyes and teeth | C1-C2 for disposal | 0.0000 | 0.0000 | 0.0000 |
| Cheek | Human food | 0.0009 | 0.0014 | 0.0031 |
| Cheek | Human food | 0.0009 | 0.0014 | 0.0031 |
| Cheek trimmings | Pet food | 0.0009 | 0.0014 | 0.0001 |
| Chops | Pet food | 0.0013 | 0.0014 | 0.0001 |
| Contents of intestines | Spreading/Compost | 0.0000 | 0.0000 | 0.0000 |
| Contents of the rumen | Spreading/Compost | 0.0000 | 0.0000 | 0.0000 |
| Ears | PAP C3 | 0.0012 | 0.0014 | 0.0001 |
| Esophagus | Pet food | 0.0009 | 0.0014 | 0.0001 |
| Fat | Fat and greaves C3 | 0.0015 | 0.0014 | 0.0001 |
| Fat around heart | Fat and greaves C3 | 0.0015 | 0.0014 | 0.0001 |
| Fat in the kidney | Fat and greaves C3 | 0.0015 | 0.0014 | 0.0001 |
| Feet (without hooves) | Gelatin C3 | 0.0009 | 0.0014 | 0.0000 |
| Floatation fat | Spreading/Compost | 0.0000 | 0.0000 | 0.0000 |
| Forehead | C1-C2 for disposal | 0.0000 | 0.0000 | 0.0000 |
| Forelock | PAP C3 | 0.0034 | 0.0014 | 0.0001 |
| Gallbladder | Pet food | 0.0009 | 0.0014 | 0.0001 |
| Head trimmings | Pet food | 0.0009 | 0.0014 | 0.0001 |
| Heart | Human food | 0.0009 | 0.0014 | 0.0003 |
| Heart trimmings | Pet food | 0.0009 | 0.0014 | 0.0001 |
| Hide | Skin tannery C3 | 0.0013 | 0.0014 | 0.0024 |
| Hooves | PAP C3 | 0.0034 | 0.0014 | 0.0001 |
| Horns | PAP C3 | 0.0034 | 0.0014 | 0.0001 |
| Kidney | Human food | 0.0009 | 0.0014 | 0.0006 |
| Large intestine | C1-C2 for disposal | 0.0000 | 0.0000 | 0.0000 |
| Liver | Human food | 0.0040 | 0.0014 | 0.0007 |
| Liver trimmings | Pet food | 0.0041 | 0.0014 | 0.0001 |
| Lower jaw | PAP C3 | 0.0009 | 0.0014 | 0.0001 |
| Lungs | Pet food | 0.0011 | 0.0014 | 0.0001 |
| Mask | Skin tannery C3 | 0.0013 | 0.0014 | 0.0024 |
| Mesenteric fat | C1-C2 for disposal | 0.0000 | 0.0000 | 0.0000 |
| Muscle | Human food | 0.0010 | 0.0014 | 0.0024 |
| Muzzle | Human food | 0.0013 | 0.0014 | 0.0014 |
| Omasum | Human food | 0.0076 | 0.0014 | 0.0011 |
| Omasum fat | Fat and greaves C3 | 0.0031 | 0.0014 | 0.0001 |
| Rumen and forestomach | Human food | 0.0076 | 0.0014 | 0.0011 |
| Rumen fat | Fat and greaves C3 | 0.0031 | 0.0014 | 0.0001 |
| Sanitary seizures | C1-C2 for disposal | 0.0000 | 0.0000 | 0.0000 |
| Screening and sifting wastes | C1-C2 for disposal | 0.0000 | 0.0000 | 0.0000 |
| Small intestine | PAP C3 | 0.0076 | 0.0014 | 0.0001 |
| Spinal cord | C1-C2 for disposal | 0.0000 | 0.0000 | 0.0000 |
| Spinal cord waste | C1-C2 for disposal | 0.0000 | 0.0000 | 0.0000 |
| Spine | C1-C2 for disposal | 0.0000 | 0.0000 | 0.0000 |
| Spleen | Pet food | 0.0010 | 0.0014 | 0.0001 |
| Stillborn | PAP C3 | 0.0079 | 0.0014 | 0.0000 |
| Tallow | Fat and greaves C3 | 0.0015 | 0.0014 | 0.0001 |
| Tongue | Human food | 0.0008 | 0.0014 | 0.0023 |
| Tonsil | C1-C2 for disposal | 0.0000 | 0.0000 | 0.0000 |
| Trachea | Pet food | 0.0013 | 0.0014 | 0.0001 |
| Udder | Pet food | 0.0002 | 0.0014 | 0.0001 |
| Upper throat | Pet food | 0.0009 | 0.0014 | 0.0001 |
| Water in the rumen | Spreading/Compost | 0.0000 | 0.0000 | 0.0000 |

Table 73: Allocation factors for Rouge des Prés Young Bulls reared in Grazing Large Area

| COPRODUCT | Destination | Rouge des Prés/young bull/grazing large area | | |
| --- | --- | --- | --- | --- |
|  |  | **Biophysical Allocation Factor** | **Mass Allocation Factor** | **Economic Allocation Factor** |
| Abomasum | Human food | 0.0085 | 0.0014 | 0.0010 |
| Abomasum fat | Fat and greaves C3 | 0.0031 | 0.0014 | 0.0001 |
| Aponeurosis | Human food | 0.0011 | 0.0014 | 0.0014 |
| Bile | PAP C3 | 0.0004 | 0.0014 | 0.0001 |
| Blood | PAP C3 | 0.0008 | 0.0014 | 0.0003 |
| Blood | Pet food | 0.0008 | 0.0014 | 0.0001 |
| Bones | Gelatin C3 | 0.0009 | 0.0014 | 0.0000 |
| Bones of head, brain, eyes and teeth | C1-C2 for disposal | 0.0000 | 0.0000 | 0.0000 |
| Cheek | Human food | 0.0010 | 0.0014 | 0.0030 |
| Cheek | Human food | 0.0010 | 0.0014 | 0.0030 |
| Cheek trimmings | Pet food | 0.0010 | 0.0014 | 0.0001 |
| Chops | Pet food | 0.0014 | 0.0014 | 0.0001 |
| Contents of intestines | Spreading/Compost | 0.0000 | 0.0000 | 0.0000 |
| Contents of the rumen | Spreading/Compost | 0.0000 | 0.0000 | 0.0000 |
| Ears | PAP C3 | 0.0012 | 0.0014 | 0.0001 |
| Esophagus | Pet food | 0.0009 | 0.0014 | 0.0001 |
| Fat | Fat and greaves C3 | 0.0013 | 0.0014 | 0.0001 |
| Fat around heart | Fat and greaves C3 | 0.0013 | 0.0014 | 0.0001 |
| Fat in the kidney | Fat and greaves C3 | 0.0013 | 0.0014 | 0.0001 |
| Feet (without hooves) | Gelatin C3 | 0.0010 | 0.0014 | 0.0000 |
| Floatation fat | Spreading/Compost | 0.0000 | 0.0000 | 0.0000 |
| Forehead | C1-C2 for disposal | 0.0000 | 0.0000 | 0.0000 |
| Forelock | PAP C3 | 0.0035 | 0.0014 | 0.0001 |
| Gallbladder | Pet food | 0.0010 | 0.0014 | 0.0001 |
| Head trimmings | Pet food | 0.0010 | 0.0014 | 0.0001 |
| Heart | Human food | 0.0009 | 0.0014 | 0.0003 |
| Heart trimmings | Pet food | 0.0010 | 0.0014 | 0.0001 |
| Hide | Skin tannery C3 | 0.0014 | 0.0014 | 0.0023 |
| Hooves | PAP C3 | 0.0035 | 0.0014 | 0.0001 |
| Horns | PAP C3 | 0.0035 | 0.0014 | 0.0001 |
| Kidney | Human food | 0.0010 | 0.0014 | 0.0006 |
| Large intestine | C1-C2 for disposal | 0.0000 | 0.0000 | 0.0000 |
| Liver | Human food | 0.0044 | 0.0014 | 0.0007 |
| Liver trimmings | Pet food | 0.0046 | 0.0014 | 0.0001 |
| Lower jaw | PAP C3 | 0.0009 | 0.0014 | 0.0001 |
| Lungs | Pet food | 0.0011 | 0.0014 | 0.0001 |
| Mask | Skin tannery C3 | 0.0014 | 0.0014 | 0.0023 |
| Mesenteric fat | C1-C2 for disposal | 0.0000 | 0.0000 | 0.0000 |
| Muscle | Human food | 0.0010 | 0.0014 | 0.0023 |
| Muzzle | Human food | 0.0014 | 0.0014 | 0.0014 |
| Omasum | Human food | 0.0085 | 0.0014 | 0.0010 |
| Omasum fat | Fat and greaves C3 | 0.0031 | 0.0014 | 0.0001 |
| Rumen and forestomach | Human food | 0.0085 | 0.0014 | 0.0010 |
| Rumen fat | Fat and greaves C3 | 0.0031 | 0.0014 | 0.0001 |
| Sanitary seizures | C1-C2 for disposal | 0.0000 | 0.0000 | 0.0000 |
| Screening and sifting wastes | C1-C2 for disposal | 0.0000 | 0.0000 | 0.0000 |
| Small intestine | PAP C3 | 0.0085 | 0.0014 | 0.0001 |
| Spinal cord | C1-C2 for disposal | 0.0000 | 0.0000 | 0.0000 |
| Spinal cord waste | C1-C2 for disposal | 0.0000 | 0.0000 | 0.0000 |
| Spine | C1-C2 for disposal | 0.0000 | 0.0000 | 0.0000 |
| Spleen | Pet food | 0.0010 | 0.0014 | 0.0001 |
| Stillborn | PAP C3 | 0.0088 | 0.0014 | 0.0000 |
| Tallow | Fat and greaves C3 | 0.0013 | 0.0014 | 0.0001 |
| Tongue | Human food | 0.0008 | 0.0014 | 0.0022 |
| Tonsil | C1-C2 for disposal | 0.0000 | 0.0000 | 0.0000 |
| Trachea | Pet food | 0.0014 | 0.0014 | 0.0001 |
| Udder | Pet food | 0.0002 | 0.0014 | 0.0001 |
| Upper throat | Pet food | 0.0010 | 0.0014 | 0.0001 |
| Water in the rumen | Spreading/Compost | 0.0000 | 0.0000 | 0.0000 |

Table 74: Allocation factors for Rouge des Prés Heifers reared in Grazing Large Area

| COPRODUCT | Destination | Rouge des Prés/heifer/grazing large area | | |
| --- | --- | --- | --- | --- |
|  |  | **Biophysical Allocation Factor** | **Mass Allocation Factor** | **Economic Allocation Factor** |
| Abomasum | Human food | 0.0084 | 0.0014 | 0.0011 |
| Abomasum fat | Fat and greaves C3 | 0.0031 | 0.0014 | 0.0001 |
| Aponeurosis | Human food | 0.0011 | 0.0014 | 0.0014 |
| Bile | PAP C3 | 0.0004 | 0.0014 | 0.0001 |
| Blood | PAP C3 | 0.0008 | 0.0014 | 0.0003 |
| Blood | Pet food | 0.0008 | 0.0014 | 0.0001 |
| Bones | Gelatin C3 | 0.0009 | 0.0014 | 0.0000 |
| Bones of head, brain, eyes and teeth | C1-C2 for disposal | 0.0000 | 0.0000 | 0.0000 |
| Cheek | Human food | 0.0010 | 0.0014 | 0.0031 |
| Cheek | Human food | 0.0010 | 0.0014 | 0.0031 |
| Cheek trimmings | Pet food | 0.0010 | 0.0014 | 0.0001 |
| Chops | Pet food | 0.0014 | 0.0014 | 0.0001 |
| Contents of intestines | Spreading/Compost | 0.0000 | 0.0000 | 0.0000 |
| Contents of the rumen | Spreading/Compost | 0.0000 | 0.0000 | 0.0000 |
| Ears | PAP C3 | 0.0012 | 0.0014 | 0.0001 |
| Esophagus | Pet food | 0.0009 | 0.0014 | 0.0001 |
| Fat | Fat and greaves C3 | 0.0013 | 0.0014 | 0.0001 |
| Fat around heart | Fat and greaves C3 | 0.0013 | 0.0014 | 0.0001 |
| Fat in the kidney | Fat and greaves C3 | 0.0013 | 0.0014 | 0.0001 |
| Feet (without hooves) | Gelatin C3 | 0.0010 | 0.0014 | 0.0000 |
| Floatation fat | Spreading/Compost | 0.0000 | 0.0000 | 0.0000 |
| Forehead | C1-C2 for disposal | 0.0000 | 0.0000 | 0.0000 |
| Forelock | PAP C3 | 0.0035 | 0.0014 | 0.0001 |
| Gallbladder | Pet food | 0.0010 | 0.0014 | 0.0001 |
| Head trimmings | Pet food | 0.0010 | 0.0014 | 0.0001 |
| Heart | Human food | 0.0009 | 0.0014 | 0.0003 |
| Heart trimmings | Pet food | 0.0010 | 0.0014 | 0.0001 |
| Hide | Skin tannery C3 | 0.0014 | 0.0014 | 0.0024 |
| Hooves | PAP C3 | 0.0035 | 0.0014 | 0.0001 |
| Horns | PAP C3 | 0.0035 | 0.0014 | 0.0001 |
| Kidney | Human food | 0.0010 | 0.0014 | 0.0006 |
| Large intestine | C1-C2 for disposal | 0.0000 | 0.0000 | 0.0000 |
| Liver | Human food | 0.0043 | 0.0014 | 0.0007 |
| Liver trimmings | Pet food | 0.0045 | 0.0014 | 0.0001 |
| Lower jaw | PAP C3 | 0.0009 | 0.0014 | 0.0001 |
| Lungs | Pet food | 0.0011 | 0.0014 | 0.0001 |
| Mask | Skin tannery C3 | 0.0014 | 0.0014 | 0.0024 |
| Mesenteric fat | C1-C2 for disposal | 0.0000 | 0.0000 | 0.0000 |
| Muscle | Human food | 0.0010 | 0.0014 | 0.0024 |
| Muzzle | Human food | 0.0014 | 0.0014 | 0.0014 |
| Omasum | Human food | 0.0084 | 0.0014 | 0.0011 |
| Omasum fat | Fat and greaves C3 | 0.0031 | 0.0014 | 0.0001 |
| Rumen and forestomach | Human food | 0.0084 | 0.0014 | 0.0011 |
| Rumen fat | Fat and greaves C3 | 0.0031 | 0.0014 | 0.0001 |
| Sanitary seizures | C1-C2 for disposal | 0.0000 | 0.0000 | 0.0000 |
| Screening and sifting wastes | C1-C2 for disposal | 0.0000 | 0.0000 | 0.0000 |
| Small intestine | PAP C3 | 0.0084 | 0.0014 | 0.0001 |
| Spinal cord | C1-C2 for disposal | 0.0000 | 0.0000 | 0.0000 |
| Spinal cord waste | C1-C2 for disposal | 0.0000 | 0.0000 | 0.0000 |
| Spine | C1-C2 for disposal | 0.0000 | 0.0000 | 0.0000 |
| Spleen | Pet food | 0.0010 | 0.0014 | 0.0001 |
| Stillborn | PAP C3 | 0.0088 | 0.0014 | 0.0000 |
| Tallow | Fat and greaves C3 | 0.0013 | 0.0014 | 0.0001 |
| Tongue | Human food | 0.0008 | 0.0014 | 0.0023 |
| Tonsil | C1-C2 for disposal | 0.0000 | 0.0000 | 0.0000 |
| Trachea | Pet food | 0.0014 | 0.0014 | 0.0001 |
| Udder | Pet food | 0.0002 | 0.0014 | 0.0001 |
| Upper throat | Pet food | 0.0010 | 0.0014 | 0.0001 |
| Water in the rumen | Spreading/Compost | 0.0000 | 0.0000 | 0.0000 |

Table 75: Allocation factors for Rouge des Prés Cull Cows reared in Grazing Large Area

| COPRODUCT | Destination | Rouge des Prés/Cull cow/grazing large area | | |
| --- | --- | --- | --- | --- |
|  |  | **Biophysical Allocation Factor** | **Mass Allocation Factor** | **Economic Allocation Factor** |
| Abomasum | Human food | 0.0079 | 0.0013 | 0.0010 |
| Abomasum fat | Fat and greaves C3 | 0.0028 | 0.0013 | 0.0001 |
| Aponeurosis | Human food | 0.0010 | 0.0013 | 0.0013 |
| Bile | PAP C3 | 0.0003 | 0.0013 | 0.0001 |
| Blood | PAP C3 | 0.0007 | 0.0013 | 0.0003 |
| Blood | Pet food | 0.0007 | 0.0013 | 0.0001 |
| Bones | Gelatin C3 | 0.0008 | 0.0013 | 0.0000 |
| Bones of head, brain, eyes and teeth | C1-C2 for disposal | 0.0000 | 0.0000 | 0.0000 |
| Cheek | Human food | 0.0009 | 0.0013 | 0.0029 |
| Cheek | Human food | 0.0009 | 0.0013 | 0.0029 |
| Cheek trimmings | Pet food | 0.0009 | 0.0013 | 0.0001 |
| Chops | Pet food | 0.0012 | 0.0013 | 0.0001 |
| Contents of intestines | Spreading/Compost | 0.0000 | 0.0000 | 0.0000 |
| Contents of the rumen | Spreading/Compost | 0.0000 | 0.0000 | 0.0000 |
| Ears | PAP C3 | 0.0011 | 0.0013 | 0.0001 |
| Esophagus | Pet food | 0.0008 | 0.0013 | 0.0001 |
| Fat | Fat and greaves C3 | 0.0011 | 0.0013 | 0.0001 |
| Fat around heart | Fat and greaves C3 | 0.0011 | 0.0013 | 0.0001 |
| Fat in the kidney | Fat and greaves C3 | 0.0011 | 0.0013 | 0.0001 |
| Feet (without hooves) | Gelatin C3 | 0.0009 | 0.0013 | 0.0000 |
| Floatation fat | Spreading/Compost | 0.0000 | 0.0000 | 0.0000 |
| Forehead | C1-C2 for disposal | 0.0000 | 0.0000 | 0.0000 |
| Forelock | PAP C3 | 0.0032 | 0.0013 | 0.0001 |
| Gallbladder | Pet food | 0.0009 | 0.0013 | 0.0001 |
| Head trimmings | Pet food | 0.0009 | 0.0013 | 0.0001 |
| Heart | Human food | 0.0008 | 0.0013 | 0.0003 |
| Heart trimmings | Pet food | 0.0009 | 0.0013 | 0.0001 |
| Hide | Skin tannery C3 | 0.0012 | 0.0013 | 0.0022 |
| Hooves | PAP C3 | 0.0032 | 0.0013 | 0.0001 |
| Horns | PAP C3 | 0.0032 | 0.0013 | 0.0001 |
| Kidney | Human food | 0.0009 | 0.0013 | 0.0005 |
| Large intestine | C1-C2 for disposal | 0.0000 | 0.0000 | 0.0000 |
| Liver | Human food | 0.0040 | 0.0013 | 0.0006 |
| Liver trimmings | Pet food | 0.0042 | 0.0013 | 0.0001 |
| Lower jaw | PAP C3 | 0.0008 | 0.0013 | 0.0001 |
| Lungs | Pet food | 0.0010 | 0.0013 | 0.0001 |
| Mask | Skin tannery C3 | 0.0012 | 0.0013 | 0.0022 |
| Mesenteric fat | C1-C2 for disposal | 0.0000 | 0.0000 | 0.0000 |
| Muscle | Human food | 0.0009 | 0.0013 | 0.0022 |
| Muzzle | Human food | 0.0012 | 0.0013 | 0.0013 |
| Omasum | Human food | 0.0079 | 0.0013 | 0.0010 |
| Omasum fat | Fat and greaves C3 | 0.0028 | 0.0013 | 0.0001 |
| Rumen and forestomach | Human food | 0.0079 | 0.0013 | 0.0010 |
| Rumen fat | Fat and greaves C3 | 0.0028 | 0.0013 | 0.0001 |
| Sanitary seizures | C1-C2 for disposal | 0.0000 | 0.0000 | 0.0000 |
| Screening and sifting wastes | C1-C2 for disposal | 0.0000 | 0.0000 | 0.0000 |
| Small intestine | PAP C3 | 0.0079 | 0.0013 | 0.0001 |
| Spinal cord | C1-C2 for disposal | 0.0000 | 0.0000 | 0.0000 |
| Spinal cord waste | C1-C2 for disposal | 0.0000 | 0.0000 | 0.0000 |
| Spine | C1-C2 for disposal | 0.0000 | 0.0000 | 0.0000 |
| Spleen | Pet food | 0.0009 | 0.0013 | 0.0001 |
| Stillborn | PAP C3 | 0.0082 | 0.0013 | 0.0000 |
| Tallow | Fat and greaves C3 | 0.0011 | 0.0013 | 0.0001 |
| Tongue | Human food | 0.0008 | 0.0013 | 0.0021 |
| Tonsil | C1-C2 for disposal | 0.0000 | 0.0000 | 0.0000 |
| Trachea | Pet food | 0.0012 | 0.0013 | 0.0001 |
| Udder | Pet food | 0.0002 | 0.0013 | 0.0001 |
| Upper throat | Pet food | 0.0009 | 0.0013 | 0.0001 |
| Water in the rumen | Spreading/Compost | 0.0000 | 0.0000 | 0.0000 |

Table 76: Allocation factors for Rouge des Prés Beef reared in Grazing Large Area

| COPRODUCT | Destination | Rouge des Prés/beef/grazing large area | | |
| --- | --- | --- | --- | --- |
|  |  | **Biophysical Allocation Factor** | **Mass Allocation Factor** | **Economic Allocation Factor** |
| Abomasum | Human food | 0.0077 | 0.0012 | 0.0009 |
| Abomasum fat | Fat and greaves C3 | 0.0025 | 0.0012 | 0.0001 |
| Aponeurosis | Human food | 0.0009 | 0.0012 | 0.0012 |
| Bile | PAP C3 | 0.0003 | 0.0012 | 0.0001 |
| Blood | PAP C3 | 0.0007 | 0.0012 | 0.0003 |
| Blood | Pet food | 0.0007 | 0.0012 | 0.0001 |
| Bones | Gelatin C3 | 0.0007 | 0.0012 | 0.0000 |
| Bones of head, brain, eyes and teeth | C1-C2 for disposal | 0.0000 | 0.0000 | 0.0000 |
| Cheek | Human food | 0.0008 | 0.0012 | 0.0025 |
| Cheek | Human food | 0.0008 | 0.0012 | 0.0025 |
| Cheek trimmings | Pet food | 0.0008 | 0.0012 | 0.0001 |
| Chops | Pet food | 0.0011 | 0.0012 | 0.0001 |
| Contents of intestines | Spreading/Compost | 0.0000 | 0.0000 | 0.0000 |
| Contents of the rumen | Spreading/Compost | 0.0000 | 0.0000 | 0.0000 |
| Ears | PAP C3 | 0.0010 | 0.0012 | 0.0001 |
| Esophagus | Pet food | 0.0008 | 0.0012 | 0.0001 |
| Fat | Fat and greaves C3 | 0.0008 | 0.0012 | 0.0001 |
| Fat around heart | Fat and greaves C3 | 0.0008 | 0.0012 | 0.0001 |
| Fat in the kidney | Fat and greaves C3 | 0.0008 | 0.0012 | 0.0001 |
| Feet (without hooves) | Gelatin C3 | 0.0008 | 0.0012 | 0.0000 |
| Floatation fat | Spreading/Compost | 0.0000 | 0.0000 | 0.0000 |
| Forehead | C1-C2 for disposal | 0.0000 | 0.0000 | 0.0000 |
| Forelock | PAP C3 | 0.0029 | 0.0012 | 0.0001 |
| Gallbladder | Pet food | 0.0008 | 0.0012 | 0.0001 |
| Head trimmings | Pet food | 0.0008 | 0.0012 | 0.0001 |
| Heart | Human food | 0.0008 | 0.0012 | 0.0002 |
| Heart trimmings | Pet food | 0.0008 | 0.0012 | 0.0001 |
| Hide | Skin tannery C3 | 0.0011 | 0.0012 | 0.0019 |
| Hooves | PAP C3 | 0.0029 | 0.0012 | 0.0001 |
| Horns | PAP C3 | 0.0029 | 0.0012 | 0.0001 |
| Kidney | Human food | 0.0008 | 0.0012 | 0.0005 |
| Large intestine | C1-C2 for disposal | 0.0000 | 0.0000 | 0.0000 |
| Liver | Human food | 0.0039 | 0.0012 | 0.0006 |
| Liver trimmings | Pet food | 0.0041 | 0.0012 | 0.0001 |
| Lower jaw | PAP C3 | 0.0007 | 0.0012 | 0.0001 |
| Lungs | Pet food | 0.0009 | 0.0012 | 0.0001 |
| Mask | Skin tannery C3 | 0.0011 | 0.0012 | 0.0019 |
| Mesenteric fat | C1-C2 for disposal | 0.0000 | 0.0000 | 0.0000 |
| Muscle | Human food | 0.0008 | 0.0012 | 0.0019 |
| Muzzle | Human food | 0.0011 | 0.0012 | 0.0012 |
| Omasum | Human food | 0.0077 | 0.0012 | 0.0009 |
| Omasum fat | Fat and greaves C3 | 0.0025 | 0.0012 | 0.0001 |
| Rumen and forestomach | Human food | 0.0077 | 0.0012 | 0.0009 |
| Rumen fat | Fat and greaves C3 | 0.0025 | 0.0012 | 0.0001 |
| Sanitary seizures | C1-C2 for disposal | 0.0000 | 0.0000 | 0.0000 |
| Screening and sifting wastes | C1-C2 for disposal | 0.0000 | 0.0000 | 0.0000 |
| Small intestine | PAP C3 | 0.0077 | 0.0012 | 0.0001 |
| Spinal cord | C1-C2 for disposal | 0.0000 | 0.0000 | 0.0000 |
| Spinal cord waste | C1-C2 for disposal | 0.0000 | 0.0000 | 0.0000 |
| Spine | C1-C2 for disposal | 0.0000 | 0.0000 | 0.0000 |
| Spleen | Pet food | 0.0008 | 0.0012 | 0.0001 |
| Stillborn | PAP C3 | 0.0080 | 0.0012 | 0.0000 |
| Tallow | Fat and greaves C3 | 0.0008 | 0.0012 | 0.0001 |
| Tongue | Human food | 0.0007 | 0.0012 | 0.0018 |
| Tonsil | C1-C2 for disposal | 0.0000 | 0.0000 | 0.0000 |
| Trachea | Pet food | 0.0011 | 0.0012 | 0.0001 |
| Udder | Pet food | 0.0002 | 0.0012 | 0.0001 |
| Upper throat | Pet food | 0.0008 | 0.0012 | 0.0001 |
| Water in the rumen | Spreading/Compost | 0.0000 | 0.0000 | 0.0000 |

Table 77: Allocation factors for Rouge des Prés Young Bulls reared in Pasture

| COPRODUCT | Destination | Rouge des Prés/young bull/pasture | | |
| --- | --- | --- | --- | --- |
|  |  | **Biophysical Allocation Factor** | **Mass Allocation Factor** | **Economic Allocation Factor** |
| Abomasum | Human food | 0.0081 | 0.0014 | 0.0010 |
| Abomasum fat | Fat and greaves C3 | 0.0031 | 0.0014 | 0.0001 |
| Aponeurosis | Human food | 0.0011 | 0.0014 | 0.0014 |
| Bile | PAP C3 | 0.0004 | 0.0014 | 0.0001 |
| Blood | PAP C3 | 0.0008 | 0.0014 | 0.0003 |
| Blood | Pet food | 0.0008 | 0.0014 | 0.0001 |
| Bones | Gelatin C3 | 0.0009 | 0.0014 | 0.0000 |
| Bones of head, brain, eyes and teeth | C1-C2 for disposal | 0.0000 | 0.0000 | 0.0000 |
| Cheek | Human food | 0.0010 | 0.0014 | 0.0030 |
| Cheek | Human food | 0.0010 | 0.0014 | 0.0030 |
| Cheek trimmings | Pet food | 0.0010 | 0.0014 | 0.0001 |
| Chops | Pet food | 0.0013 | 0.0014 | 0.0001 |
| Contents of intestines | Spreading/Compost | 0.0000 | 0.0000 | 0.0000 |
| Contents of the rumen | Spreading/Compost | 0.0000 | 0.0000 | 0.0000 |
| Ears | PAP C3 | 0.0012 | 0.0014 | 0.0001 |
| Esophagus | Pet food | 0.0009 | 0.0014 | 0.0001 |
| Fat | Fat and greaves C3 | 0.0014 | 0.0014 | 0.0001 |
| Fat around heart | Fat and greaves C3 | 0.0014 | 0.0014 | 0.0001 |
| Fat in the kidney | Fat and greaves C3 | 0.0014 | 0.0014 | 0.0001 |
| Feet (without hooves) | Gelatin C3 | 0.0010 | 0.0014 | 0.0000 |
| Floatation fat | Spreading/Compost | 0.0000 | 0.0000 | 0.0000 |
| Forehead | C1-C2 for disposal | 0.0000 | 0.0000 | 0.0000 |
| Forelock | PAP C3 | 0.0035 | 0.0014 | 0.0001 |
| Gallbladder | Pet food | 0.0010 | 0.0014 | 0.0001 |
| Head trimmings | Pet food | 0.0010 | 0.0014 | 0.0001 |
| Heart | Human food | 0.0009 | 0.0014 | 0.0003 |
| Heart trimmings | Pet food | 0.0010 | 0.0014 | 0.0001 |
| Hide | Skin tannery C3 | 0.0013 | 0.0014 | 0.0023 |
| Hooves | PAP C3 | 0.0035 | 0.0014 | 0.0001 |
| Horns | PAP C3 | 0.0035 | 0.0014 | 0.0001 |
| Kidney | Human food | 0.0009 | 0.0014 | 0.0006 |
| Large intestine | C1-C2 for disposal | 0.0000 | 0.0000 | 0.0000 |
| Liver | Human food | 0.0042 | 0.0014 | 0.0007 |
| Liver trimmings | Pet food | 0.0044 | 0.0014 | 0.0001 |
| Lower jaw | PAP C3 | 0.0009 | 0.0014 | 0.0001 |
| Lungs | Pet food | 0.0011 | 0.0014 | 0.0001 |
| Mask | Skin tannery C3 | 0.0013 | 0.0014 | 0.0023 |
| Mesenteric fat | C1-C2 for disposal | 0.0000 | 0.0000 | 0.0000 |
| Muscle | Human food | 0.0010 | 0.0014 | 0.0023 |
| Muzzle | Human food | 0.0013 | 0.0014 | 0.0014 |
| Omasum | Human food | 0.0081 | 0.0014 | 0.0010 |
| Omasum fat | Fat and greaves C3 | 0.0031 | 0.0014 | 0.0001 |
| Rumen and forestomach | Human food | 0.0081 | 0.0014 | 0.0010 |
| Rumen fat | Fat and greaves C3 | 0.0031 | 0.0014 | 0.0001 |
| Sanitary seizures | C1-C2 for disposal | 0.0000 | 0.0000 | 0.0000 |
| Screening and sifting wastes | C1-C2 for disposal | 0.0000 | 0.0000 | 0.0000 |
| Small intestine | PAP C3 | 0.0081 | 0.0014 | 0.0001 |
| Spinal cord | C1-C2 for disposal | 0.0000 | 0.0000 | 0.0000 |
| Spinal cord waste | C1-C2 for disposal | 0.0000 | 0.0000 | 0.0000 |
| Spine | C1-C2 for disposal | 0.0000 | 0.0000 | 0.0000 |
| Spleen | Pet food | 0.0010 | 0.0014 | 0.0001 |
| Stillborn | PAP C3 | 0.0085 | 0.0014 | 0.0000 |
| Tallow | Fat and greaves C3 | 0.0014 | 0.0014 | 0.0001 |
| Tongue | Human food | 0.0009 | 0.0014 | 0.0022 |
| Tonsil | C1-C2 for disposal | 0.0000 | 0.0000 | 0.0000 |
| Trachea | Pet food | 0.0013 | 0.0014 | 0.0001 |
| Udder | Pet food | 0.0002 | 0.0014 | 0.0001 |
| Upper throat | Pet food | 0.0010 | 0.0014 | 0.0001 |
| Water in the rumen | Spreading/Compost | 0.0000 | 0.0000 | 0.0000 |

Table 78: Allocation factors for Rouge des Prés Heifers reared in Pasture

| COPRODUCT | Destination | Rouge des Prés/heifer/pasture | | |
| --- | --- | --- | --- | --- |
|  |  | **Biophysical Allocation Factor** | **Mass Allocation Factor** | **Economic Allocation Factor** |
| Abomasum | Human food | 0.0081 | 0.0014 | 0.0011 |
| Abomasum fat | Fat and greaves C3 | 0.0031 | 0.0014 | 0.0001 |
| Aponeurosis | Human food | 0.0011 | 0.0014 | 0.0014 |
| Bile | PAP C3 | 0.0004 | 0.0014 | 0.0001 |
| Blood | PAP C3 | 0.0008 | 0.0014 | 0.0003 |
| Blood | Pet food | 0.0008 | 0.0014 | 0.0001 |
| Bones | Gelatin C3 | 0.0009 | 0.0014 | 0.0000 |
| Bones of head, brain, eyes and teeth | C1-C2 for disposal | 0.0000 | 0.0000 | 0.0000 |
| Cheek | Human food | 0.0010 | 0.0014 | 0.0031 |
| Cheek | Human food | 0.0010 | 0.0014 | 0.0031 |
| Cheek trimmings | Pet food | 0.0010 | 0.0014 | 0.0001 |
| Chops | Pet food | 0.0013 | 0.0014 | 0.0001 |
| Contents of intestines | Spreading/Compost | 0.0000 | 0.0000 | 0.0000 |
| Contents of the rumen | Spreading/Compost | 0.0000 | 0.0000 | 0.0000 |
| Ears | PAP C3 | 0.0012 | 0.0014 | 0.0001 |
| Esophagus | Pet food | 0.0009 | 0.0014 | 0.0001 |
| Fat | Fat and greaves C3 | 0.0014 | 0.0014 | 0.0001 |
| Fat around heart | Fat and greaves C3 | 0.0014 | 0.0014 | 0.0001 |
| Fat in the kidney | Fat and greaves C3 | 0.0014 | 0.0014 | 0.0001 |
| Feet (without hooves) | Gelatin C3 | 0.0010 | 0.0014 | 0.0000 |
| Floatation fat | Spreading/Compost | 0.0000 | 0.0000 | 0.0000 |
| Forehead | C1-C2 for disposal | 0.0000 | 0.0000 | 0.0000 |
| Forelock | PAP C3 | 0.0035 | 0.0014 | 0.0001 |
| Gallbladder | Pet food | 0.0010 | 0.0014 | 0.0001 |
| Head trimmings | Pet food | 0.0010 | 0.0014 | 0.0001 |
| Heart | Human food | 0.0009 | 0.0014 | 0.0003 |
| Heart trimmings | Pet food | 0.0010 | 0.0014 | 0.0001 |
| Hide | Skin tannery C3 | 0.0013 | 0.0014 | 0.0024 |
| Hooves | PAP C3 | 0.0035 | 0.0014 | 0.0001 |
| Horns | PAP C3 | 0.0035 | 0.0014 | 0.0001 |
| Kidney | Human food | 0.0009 | 0.0014 | 0.0006 |
| Large intestine | C1-C2 for disposal | 0.0000 | 0.0000 | 0.0000 |
| Liver | Human food | 0.0042 | 0.0014 | 0.0007 |
| Liver trimmings | Pet food | 0.0044 | 0.0014 | 0.0001 |
| Lower jaw | PAP C3 | 0.0009 | 0.0014 | 0.0001 |
| Lungs | Pet food | 0.0011 | 0.0014 | 0.0001 |
| Mask | Skin tannery C3 | 0.0013 | 0.0014 | 0.0024 |
| Mesenteric fat | C1-C2 for disposal | 0.0000 | 0.0000 | 0.0000 |
| Muscle | Human food | 0.0010 | 0.0014 | 0.0024 |
| Muzzle | Human food | 0.0013 | 0.0014 | 0.0014 |
| Omasum | Human food | 0.0081 | 0.0014 | 0.0011 |
| Omasum fat | Fat and greaves C3 | 0.0031 | 0.0014 | 0.0001 |
| Rumen and forestomach | Human food | 0.0081 | 0.0014 | 0.0011 |
| Rumen fat | Fat and greaves C3 | 0.0031 | 0.0014 | 0.0001 |
| Sanitary seizures | C1-C2 for disposal | 0.0000 | 0.0000 | 0.0000 |
| Screening and sifting wastes | C1-C2 for disposal | 0.0000 | 0.0000 | 0.0000 |
| Small intestine | PAP C3 | 0.0081 | 0.0014 | 0.0001 |
| Spinal cord | C1-C2 for disposal | 0.0000 | 0.0000 | 0.0000 |
| Spinal cord waste | C1-C2 for disposal | 0.0000 | 0.0000 | 0.0000 |
| Spine | C1-C2 for disposal | 0.0000 | 0.0000 | 0.0000 |
| Spleen | Pet food | 0.0010 | 0.0014 | 0.0001 |
| Stillborn | PAP C3 | 0.0084 | 0.0014 | 0.0000 |
| Tallow | Fat and greaves C3 | 0.0014 | 0.0014 | 0.0001 |
| Tongue | Human food | 0.0008 | 0.0014 | 0.0023 |
| Tonsil | C1-C2 for disposal | 0.0000 | 0.0000 | 0.0000 |
| Trachea | Pet food | 0.0013 | 0.0014 | 0.0001 |
| Udder | Pet food | 0.0002 | 0.0014 | 0.0001 |
| Upper throat | Pet food | 0.0010 | 0.0014 | 0.0001 |
| Water in the rumen | Spreading/Compost | 0.0000 | 0.0000 | 0.0000 |

Table 79: Allocation factors for Rouge des Prés Cull Cows reared in Pasture

| COPRODUCT | Destination | Rouge des Prés/Cull cow/pasture | | |
| --- | --- | --- | --- | --- |
|  |  | **Biophysical Allocation Factor** | **Mass Allocation Factor** | **Economic Allocation Factor** |
| Abomasum | Human food | 0.0076 | 0.0013 | 0.0010 |
| Abomasum fat | Fat and greaves C3 | 0.0028 | 0.0013 | 0.0001 |
| Aponeurosis | Human food | 0.0010 | 0.0013 | 0.0013 |
| Bile | PAP C3 | 0.0003 | 0.0013 | 0.0001 |
| Blood | PAP C3 | 0.0007 | 0.0013 | 0.0003 |
| Blood | Pet food | 0.0007 | 0.0013 | 0.0001 |
| Bones | Gelatin C3 | 0.0008 | 0.0013 | 0.0000 |
| Bones of head, brain, eyes and teeth | C1-C2 for disposal | 0.0000 | 0.0000 | 0.0000 |
| Cheek | Human food | 0.0009 | 0.0013 | 0.0029 |
| Cheek | Human food | 0.0009 | 0.0013 | 0.0029 |
| Cheek trimmings | Pet food | 0.0009 | 0.0013 | 0.0001 |
| Chops | Pet food | 0.0012 | 0.0013 | 0.0001 |
| Contents of intestines | Spreading/Compost | 0.0000 | 0.0000 | 0.0000 |
| Contents of the rumen | Spreading/Compost | 0.0000 | 0.0000 | 0.0000 |
| Ears | PAP C3 | 0.0011 | 0.0013 | 0.0001 |
| Esophagus | Pet food | 0.0008 | 0.0013 | 0.0001 |
| Fat | Fat and greaves C3 | 0.0012 | 0.0013 | 0.0001 |
| Fat around heart | Fat and greaves C3 | 0.0012 | 0.0013 | 0.0001 |
| Fat in the kidney | Fat and greaves C3 | 0.0012 | 0.0013 | 0.0001 |
| Feet (without hooves) | Gelatin C3 | 0.0009 | 0.0013 | 0.0000 |
| Floatation fat | Spreading/Compost | 0.0000 | 0.0000 | 0.0000 |
| Forehead | C1-C2 for disposal | 0.0000 | 0.0000 | 0.0000 |
| Forelock | PAP C3 | 0.0031 | 0.0013 | 0.0001 |
| Gallbladder | Pet food | 0.0009 | 0.0013 | 0.0001 |
| Head trimmings | Pet food | 0.0009 | 0.0013 | 0.0001 |
| Heart | Human food | 0.0008 | 0.0013 | 0.0003 |
| Heart trimmings | Pet food | 0.0009 | 0.0013 | 0.0001 |
| Hide | Skin tannery C3 | 0.0012 | 0.0013 | 0.0022 |
| Hooves | PAP C3 | 0.0031 | 0.0013 | 0.0001 |
| Horns | PAP C3 | 0.0031 | 0.0013 | 0.0001 |
| Kidney | Human food | 0.0009 | 0.0013 | 0.0005 |
| Large intestine | C1-C2 for disposal | 0.0000 | 0.0000 | 0.0000 |
| Liver | Human food | 0.0039 | 0.0013 | 0.0006 |
| Liver trimmings | Pet food | 0.0041 | 0.0013 | 0.0001 |
| Lower jaw | PAP C3 | 0.0008 | 0.0013 | 0.0001 |
| Lungs | Pet food | 0.0010 | 0.0013 | 0.0001 |
| Mask | Skin tannery C3 | 0.0012 | 0.0013 | 0.0022 |
| Mesenteric fat | C1-C2 for disposal | 0.0000 | 0.0000 | 0.0000 |
| Muscle | Human food | 0.0009 | 0.0013 | 0.0022 |
| Muzzle | Human food | 0.0012 | 0.0013 | 0.0013 |
| Omasum | Human food | 0.0076 | 0.0013 | 0.0010 |
| Omasum fat | Fat and greaves C3 | 0.0028 | 0.0013 | 0.0001 |
| Rumen and forestomach | Human food | 0.0076 | 0.0013 | 0.0010 |
| Rumen fat | Fat and greaves C3 | 0.0028 | 0.0013 | 0.0001 |
| Sanitary seizures | C1-C2 for disposal | 0.0000 | 0.0000 | 0.0000 |
| Screening and sifting wastes | C1-C2 for disposal | 0.0000 | 0.0000 | 0.0000 |
| Small intestine | PAP C3 | 0.0076 | 0.0013 | 0.0001 |
| Spinal cord | C1-C2 for disposal | 0.0000 | 0.0000 | 0.0000 |
| Spinal cord waste | C1-C2 for disposal | 0.0000 | 0.0000 | 0.0000 |
| Spine | C1-C2 for disposal | 0.0000 | 0.0000 | 0.0000 |
| Spleen | Pet food | 0.0009 | 0.0013 | 0.0001 |
| Stillborn | PAP C3 | 0.0079 | 0.0013 | 0.0000 |
| Tallow | Fat and greaves C3 | 0.0012 | 0.0013 | 0.0001 |
| Tongue | Human food | 0.0008 | 0.0013 | 0.0021 |
| Tonsil | C1-C2 for disposal | 0.0000 | 0.0000 | 0.0000 |
| Trachea | Pet food | 0.0012 | 0.0013 | 0.0001 |
| Udder | Pet food | 0.0002 | 0.0013 | 0.0001 |
| Upper throat | Pet food | 0.0009 | 0.0013 | 0.0001 |
| Water in the rumen | Spreading/Compost | 0.0000 | 0.0000 | 0.0000 |

Table 80: Allocation factors for Rouge des Prés Beef reared in Pasture

| COPRODUCT | Destination | Rouge des Prés/beef/pasture | | |
| --- | --- | --- | --- | --- |
|  |  | **Biophysical Allocation Factor** | **Mass Allocation Factor** | **Economic Allocation Factor** |
| Abomasum | Human food | 0.0074 | 0.0012 | 0.0009 |
| Abomasum fat | Fat and greaves C3 | 0.0026 | 0.0012 | 0.0001 |
| Aponeurosis | Human food | 0.0009 | 0.0012 | 0.0012 |
| Bile | PAP C3 | 0.0003 | 0.0012 | 0.0001 |
| Blood | PAP C3 | 0.0007 | 0.0012 | 0.0003 |
| Blood | Pet food | 0.0007 | 0.0012 | 0.0001 |
| Bones | Gelatin C3 | 0.0007 | 0.0012 | 0.0000 |
| Bones of head, brain, eyes and teeth | C1-C2 for disposal | 0.0000 | 0.0000 | 0.0000 |
| Cheek | Human food | 0.0008 | 0.0012 | 0.0025 |
| Cheek | Human food | 0.0008 | 0.0012 | 0.0025 |
| Cheek trimmings | Pet food | 0.0008 | 0.0012 | 0.0001 |
| Chops | Pet food | 0.0011 | 0.0012 | 0.0001 |
| Contents of intestines | Spreading/Compost | 0.0000 | 0.0000 | 0.0000 |
| Contents of the rumen | Spreading/Compost | 0.0000 | 0.0000 | 0.0000 |
| Ears | PAP C3 | 0.0010 | 0.0012 | 0.0001 |
| Esophagus | Pet food | 0.0008 | 0.0012 | 0.0001 |
| Fat | Fat and greaves C3 | 0.0009 | 0.0012 | 0.0001 |
| Fat around heart | Fat and greaves C3 | 0.0009 | 0.0012 | 0.0001 |
| Fat in the kidney | Fat and greaves C3 | 0.0009 | 0.0012 | 0.0001 |
| Feet (without hooves) | Gelatin C3 | 0.0008 | 0.0012 | 0.0000 |
| Floatation fat | Spreading/Compost | 0.0000 | 0.0000 | 0.0000 |
| Forehead | C1-C2 for disposal | 0.0000 | 0.0000 | 0.0000 |
| Forelock | PAP C3 | 0.0029 | 0.0012 | 0.0001 |
| Gallbladder | Pet food | 0.0008 | 0.0012 | 0.0001 |
| Head trimmings | Pet food | 0.0008 | 0.0012 | 0.0001 |
| Heart | Human food | 0.0008 | 0.0012 | 0.0002 |
| Heart trimmings | Pet food | 0.0008 | 0.0012 | 0.0001 |
| Hide | Skin tannery C3 | 0.0011 | 0.0012 | 0.0019 |
| Hooves | PAP C3 | 0.0029 | 0.0012 | 0.0001 |
| Horns | PAP C3 | 0.0029 | 0.0012 | 0.0001 |
| Kidney | Human food | 0.0008 | 0.0012 | 0.0005 |
| Large intestine | C1-C2 for disposal | 0.0000 | 0.0000 | 0.0000 |
| Liver | Human food | 0.0038 | 0.0012 | 0.0006 |
| Liver trimmings | Pet food | 0.0040 | 0.0012 | 0.0001 |
| Lower jaw | PAP C3 | 0.0007 | 0.0012 | 0.0001 |
| Lungs | Pet food | 0.0009 | 0.0012 | 0.0001 |
| Mask | Skin tannery C3 | 0.0011 | 0.0012 | 0.0019 |
| Mesenteric fat | C1-C2 for disposal | 0.0000 | 0.0000 | 0.0000 |
| Muscle | Human food | 0.0008 | 0.0012 | 0.0019 |
| Muzzle | Human food | 0.0011 | 0.0012 | 0.0012 |
| Omasum | Human food | 0.0074 | 0.0012 | 0.0009 |
| Omasum fat | Fat and greaves C3 | 0.0026 | 0.0012 | 0.0001 |
| Rumen and forestomach | Human food | 0.0074 | 0.0012 | 0.0009 |
| Rumen fat | Fat and greaves C3 | 0.0026 | 0.0012 | 0.0001 |
| Sanitary seizures | C1-C2 for disposal | 0.0000 | 0.0000 | 0.0000 |
| Screening and sifting wastes | C1-C2 for disposal | 0.0000 | 0.0000 | 0.0000 |
| Small intestine | PAP C3 | 0.0074 | 0.0012 | 0.0001 |
| Spinal cord | C1-C2 for disposal | 0.0000 | 0.0000 | 0.0000 |
| Spinal cord waste | C1-C2 for disposal | 0.0000 | 0.0000 | 0.0000 |
| Spine | C1-C2 for disposal | 0.0000 | 0.0000 | 0.0000 |
| Spleen | Pet food | 0.0008 | 0.0012 | 0.0001 |
| Stillborn | PAP C3 | 0.0078 | 0.0012 | 0.0000 |
| Tallow | Fat and greaves C3 | 0.0009 | 0.0012 | 0.0001 |
| Tongue | Human food | 0.0007 | 0.0012 | 0.0018 |
| Tonsil | C1-C2 for disposal | 0.0000 | 0.0000 | 0.0000 |
| Trachea | Pet food | 0.0011 | 0.0012 | 0.0001 |
| Udder | Pet food | 0.0002 | 0.0012 | 0.0001 |
| Upper throat | Pet food | 0.0008 | 0.0012 | 0.0001 |
| Water in the rumen | Spreading/Compost | 0.0000 | 0.0000 | 0.0000 |

Table 81: Allocation factors for Rouge des Prés Young Bulls reared in Stall

| COPRODUCT | Destination | Rouge des Prés/young bull/stall | | |
| --- | --- | --- | --- | --- |
|  |  | **Biophysical Allocation Factor** | **Mass Allocation Factor** | **Economic Allocation Factor** |
| Abomasum | Human food | 0.0077 | 0.0014 | 0.0010 |
| Abomasum fat | Fat and greaves C3 | 0.0032 | 0.0014 | 0.0001 |
| Aponeurosis | Human food | 0.0010 | 0.0014 | 0.0014 |
| Bile | PAP C3 | 0.0004 | 0.0014 | 0.0001 |
| Blood | PAP C3 | 0.0008 | 0.0014 | 0.0003 |
| Blood | Pet food | 0.0008 | 0.0014 | 0.0001 |
| Bones | Gelatin C3 | 0.0009 | 0.0014 | 0.0000 |
| Bones of head, brain, eyes and teeth | C1-C2 for disposal | 0.0000 | 0.0000 | 0.0000 |
| Cheek | Human food | 0.0010 | 0.0014 | 0.0030 |
| Cheek | Human food | 0.0010 | 0.0014 | 0.0030 |
| Cheek trimmings | Pet food | 0.0010 | 0.0014 | 0.0001 |
| Chops | Pet food | 0.0013 | 0.0014 | 0.0001 |
| Contents of intestines | Spreading/Compost | 0.0000 | 0.0000 | 0.0000 |
| Contents of the rumen | Spreading/Compost | 0.0000 | 0.0000 | 0.0000 |
| Ears | PAP C3 | 0.0012 | 0.0014 | 0.0001 |
| Esophagus | Pet food | 0.0009 | 0.0014 | 0.0001 |
| Fat | Fat and greaves C3 | 0.0015 | 0.0014 | 0.0001 |
| Fat around heart | Fat and greaves C3 | 0.0015 | 0.0014 | 0.0001 |
| Fat in the kidney | Fat and greaves C3 | 0.0015 | 0.0014 | 0.0001 |
| Feet (without hooves) | Gelatin C3 | 0.0009 | 0.0014 | 0.0000 |
| Floatation fat | Spreading/Compost | 0.0000 | 0.0000 | 0.0000 |
| Forehead | C1-C2 for disposal | 0.0000 | 0.0000 | 0.0000 |
| Forelock | PAP C3 | 0.0034 | 0.0014 | 0.0001 |
| Gallbladder | Pet food | 0.0009 | 0.0014 | 0.0001 |
| Head trimmings | Pet food | 0.0010 | 0.0014 | 0.0001 |
| Heart | Human food | 0.0009 | 0.0014 | 0.0003 |
| Heart trimmings | Pet food | 0.0010 | 0.0014 | 0.0001 |
| Hide | Skin tannery C3 | 0.0013 | 0.0014 | 0.0023 |
| Hooves | PAP C3 | 0.0034 | 0.0014 | 0.0001 |
| Horns | PAP C3 | 0.0034 | 0.0014 | 0.0001 |
| Kidney | Human food | 0.0009 | 0.0014 | 0.0006 |
| Large intestine | C1-C2 for disposal | 0.0000 | 0.0000 | 0.0000 |
| Liver | Human food | 0.0040 | 0.0014 | 0.0007 |
| Liver trimmings | Pet food | 0.0042 | 0.0014 | 0.0001 |
| Lower jaw | PAP C3 | 0.0009 | 0.0014 | 0.0001 |
| Lungs | Pet food | 0.0011 | 0.0014 | 0.0001 |
| Mask | Skin tannery C3 | 0.0013 | 0.0014 | 0.0023 |
| Mesenteric fat | C1-C2 for disposal | 0.0000 | 0.0000 | 0.0000 |
| Muscle | Human food | 0.0010 | 0.0014 | 0.0023 |
| Muzzle | Human food | 0.0013 | 0.0014 | 0.0014 |
| Omasum | Human food | 0.0077 | 0.0014 | 0.0010 |
| Omasum fat | Fat and greaves C3 | 0.0032 | 0.0014 | 0.0001 |
| Rumen and forestomach | Human food | 0.0077 | 0.0014 | 0.0010 |
| Rumen fat | Fat and greaves C3 | 0.0032 | 0.0014 | 0.0001 |
| Sanitary seizures | C1-C2 for disposal | 0.0000 | 0.0000 | 0.0000 |
| Screening and sifting wastes | C1-C2 for disposal | 0.0000 | 0.0000 | 0.0000 |
| Small intestine | PAP C3 | 0.0077 | 0.0014 | 0.0001 |
| Spinal cord | C1-C2 for disposal | 0.0000 | 0.0000 | 0.0000 |
| Spinal cord waste | C1-C2 for disposal | 0.0000 | 0.0000 | 0.0000 |
| Spine | C1-C2 for disposal | 0.0000 | 0.0000 | 0.0000 |
| Spleen | Pet food | 0.0010 | 0.0014 | 0.0001 |
| Stillborn | PAP C3 | 0.0081 | 0.0014 | 0.0000 |
| Tallow | Fat and greaves C3 | 0.0015 | 0.0014 | 0.0001 |
| Tongue | Human food | 0.0009 | 0.0014 | 0.0022 |
| Tonsil | C1-C2 for disposal | 0.0000 | 0.0000 | 0.0000 |
| Trachea | Pet food | 0.0013 | 0.0014 | 0.0001 |
| Udder | Pet food | 0.0002 | 0.0014 | 0.0001 |
| Upper throat | Pet food | 0.0009 | 0.0014 | 0.0001 |
| Water in the rumen | Spreading/Compost | 0.0000 | 0.0000 | 0.0000 |

Table 82: Allocation factors for Rouge des Prés Heifers reared in Stall

| COPRODUCT | Destination | Rouge des Prés/heifer/stall | | |
| --- | --- | --- | --- | --- |
|  |  | **Biophysical Allocation Factor** | **Mass Allocation Factor** | **Economic Allocation Factor** |
| Abomasum | Human food | 0.0077 | 0.0014 | 0.0011 |
| Abomasum fat | Fat and greaves C3 | 0.0031 | 0.0014 | 0.0001 |
| Aponeurosis | Human food | 0.0010 | 0.0014 | 0.0014 |
| Bile | PAP C3 | 0.0004 | 0.0014 | 0.0001 |
| Blood | PAP C3 | 0.0008 | 0.0014 | 0.0003 |
| Blood | Pet food | 0.0008 | 0.0014 | 0.0001 |
| Bones | Gelatin C3 | 0.0009 | 0.0014 | 0.0000 |
| Bones of head, brain, eyes and teeth | C1-C2 for disposal | 0.0000 | 0.0000 | 0.0000 |
| Cheek | Human food | 0.0010 | 0.0014 | 0.0031 |
| Cheek | Human food | 0.0010 | 0.0014 | 0.0031 |
| Cheek trimmings | Pet food | 0.0010 | 0.0014 | 0.0001 |
| Chops | Pet food | 0.0013 | 0.0014 | 0.0001 |
| Contents of intestines | Spreading/Compost | 0.0000 | 0.0000 | 0.0000 |
| Contents of the rumen | Spreading/Compost | 0.0000 | 0.0000 | 0.0000 |
| Ears | PAP C3 | 0.0012 | 0.0014 | 0.0001 |
| Esophagus | Pet food | 0.0009 | 0.0014 | 0.0001 |
| Fat | Fat and greaves C3 | 0.0015 | 0.0014 | 0.0001 |
| Fat around heart | Fat and greaves C3 | 0.0015 | 0.0014 | 0.0001 |
| Fat in the kidney | Fat and greaves C3 | 0.0015 | 0.0014 | 0.0001 |
| Feet (without hooves) | Gelatin C3 | 0.0009 | 0.0014 | 0.0000 |
| Floatation fat | Spreading/Compost | 0.0000 | 0.0000 | 0.0000 |
| Forehead | C1-C2 for disposal | 0.0000 | 0.0000 | 0.0000 |
| Forelock | PAP C3 | 0.0034 | 0.0014 | 0.0001 |
| Gallbladder | Pet food | 0.0009 | 0.0014 | 0.0001 |
| Head trimmings | Pet food | 0.0010 | 0.0014 | 0.0001 |
| Heart | Human food | 0.0009 | 0.0014 | 0.0003 |
| Heart trimmings | Pet food | 0.0010 | 0.0014 | 0.0001 |
| Hide | Skin tannery C3 | 0.0013 | 0.0014 | 0.0024 |
| Hooves | PAP C3 | 0.0034 | 0.0014 | 0.0001 |
| Horns | PAP C3 | 0.0034 | 0.0014 | 0.0001 |
| Kidney | Human food | 0.0009 | 0.0014 | 0.0006 |
| Large intestine | C1-C2 for disposal | 0.0000 | 0.0000 | 0.0000 |
| Liver | Human food | 0.0040 | 0.0014 | 0.0007 |
| Liver trimmings | Pet food | 0.0042 | 0.0014 | 0.0001 |
| Lower jaw | PAP C3 | 0.0009 | 0.0014 | 0.0001 |
| Lungs | Pet food | 0.0011 | 0.0014 | 0.0001 |
| Mask | Skin tannery C3 | 0.0013 | 0.0014 | 0.0024 |
| Mesenteric fat | C1-C2 for disposal | 0.0000 | 0.0000 | 0.0000 |
| Muscle | Human food | 0.0010 | 0.0014 | 0.0024 |
| Muzzle | Human food | 0.0013 | 0.0014 | 0.0014 |
| Omasum | Human food | 0.0077 | 0.0014 | 0.0011 |
| Omasum fat | Fat and greaves C3 | 0.0031 | 0.0014 | 0.0001 |
| Rumen and forestomach | Human food | 0.0077 | 0.0014 | 0.0011 |
| Rumen fat | Fat and greaves C3 | 0.0031 | 0.0014 | 0.0001 |
| Sanitary seizures | C1-C2 for disposal | 0.0000 | 0.0000 | 0.0000 |
| Screening and sifting wastes | C1-C2 for disposal | 0.0000 | 0.0000 | 0.0000 |
| Small intestine | PAP C3 | 0.0077 | 0.0014 | 0.0001 |
| Spinal cord | C1-C2 for disposal | 0.0000 | 0.0000 | 0.0000 |
| Spinal cord waste | C1-C2 for disposal | 0.0000 | 0.0000 | 0.0000 |
| Spine | C1-C2 for disposal | 0.0000 | 0.0000 | 0.0000 |
| Spleen | Pet food | 0.0010 | 0.0014 | 0.0001 |
| Stillborn | PAP C3 | 0.0080 | 0.0014 | 0.0000 |
| Tallow | Fat and greaves C3 | 0.0015 | 0.0014 | 0.0001 |
| Tongue | Human food | 0.0009 | 0.0014 | 0.0023 |
| Tonsil | C1-C2 for disposal | 0.0000 | 0.0000 | 0.0000 |
| Trachea | Pet food | 0.0013 | 0.0014 | 0.0001 |
| Udder | Pet food | 0.0002 | 0.0014 | 0.0001 |
| Upper throat | Pet food | 0.0009 | 0.0014 | 0.0001 |
| Water in the rumen | Spreading/Compost | 0.0000 | 0.0000 | 0.0000 |

Table 83: Allocation factors for Rouge des Prés Cull Cows reared in Stall

| COPRODUCT | Destination | Rouge des Prés/Cull cow/stall | | |
| --- | --- | --- | --- | --- |
|  |  | **Biophysical Allocation Factor** | **Mass Allocation Factor** | **Economic Allocation Factor** |
| Abomasum | Human food | 0.0073 | 0.0013 | 0.0010 |
| Abomasum fat | Fat and greaves C3 | 0.0029 | 0.0013 | 0.0001 |
| Aponeurosis | Human food | 0.0010 | 0.0013 | 0.0013 |
| Bile | PAP C3 | 0.0003 | 0.0013 | 0.0001 |
| Blood | PAP C3 | 0.0007 | 0.0013 | 0.0003 |
| Blood | Pet food | 0.0007 | 0.0013 | 0.0001 |
| Bones | Gelatin C3 | 0.0008 | 0.0013 | 0.0000 |
| Bones of head, brain, eyes and teeth | C1-C2 for disposal | 0.0000 | 0.0000 | 0.0000 |
| Cheek | Human food | 0.0009 | 0.0013 | 0.0029 |
| Cheek | Human food | 0.0009 | 0.0013 | 0.0029 |
| Cheek trimmings | Pet food | 0.0009 | 0.0013 | 0.0001 |
| Chops | Pet food | 0.0012 | 0.0013 | 0.0001 |
| Contents of intestines | Spreading/Compost | 0.0000 | 0.0000 | 0.0000 |
| Contents of the rumen | Spreading/Compost | 0.0000 | 0.0000 | 0.0000 |
| Ears | PAP C3 | 0.0011 | 0.0013 | 0.0001 |
| Esophagus | Pet food | 0.0008 | 0.0013 | 0.0001 |
| Fat | Fat and greaves C3 | 0.0013 | 0.0013 | 0.0001 |
| Fat around heart | Fat and greaves C3 | 0.0013 | 0.0013 | 0.0001 |
| Fat in the kidney | Fat and greaves C3 | 0.0013 | 0.0013 | 0.0001 |
| Feet (without hooves) | Gelatin C3 | 0.0009 | 0.0013 | 0.0000 |
| Floatation fat | Spreading/Compost | 0.0000 | 0.0000 | 0.0000 |
| Forehead | C1-C2 for disposal | 0.0000 | 0.0000 | 0.0000 |
| Forelock | PAP C3 | 0.0031 | 0.0013 | 0.0001 |
| Gallbladder | Pet food | 0.0009 | 0.0013 | 0.0001 |
| Head trimmings | Pet food | 0.0009 | 0.0013 | 0.0001 |
| Heart | Human food | 0.0008 | 0.0013 | 0.0003 |
| Heart trimmings | Pet food | 0.0009 | 0.0013 | 0.0001 |
| Hide | Skin tannery C3 | 0.0012 | 0.0013 | 0.0022 |
| Hooves | PAP C3 | 0.0031 | 0.0013 | 0.0001 |
| Horns | PAP C3 | 0.0031 | 0.0013 | 0.0001 |
| Kidney | Human food | 0.0008 | 0.0013 | 0.0005 |
| Large intestine | C1-C2 for disposal | 0.0000 | 0.0000 | 0.0000 |
| Liver | Human food | 0.0038 | 0.0013 | 0.0006 |
| Liver trimmings | Pet food | 0.0039 | 0.0013 | 0.0001 |
| Lower jaw | PAP C3 | 0.0008 | 0.0013 | 0.0001 |
| Lungs | Pet food | 0.0010 | 0.0013 | 0.0001 |
| Mask | Skin tannery C3 | 0.0012 | 0.0013 | 0.0022 |
| Mesenteric fat | C1-C2 for disposal | 0.0000 | 0.0000 | 0.0000 |
| Muscle | Human food | 0.0009 | 0.0013 | 0.0022 |
| Muzzle | Human food | 0.0012 | 0.0013 | 0.0013 |
| Omasum | Human food | 0.0073 | 0.0013 | 0.0010 |
| Omasum fat | Fat and greaves C3 | 0.0029 | 0.0013 | 0.0001 |
| Rumen and forestomach | Human food | 0.0073 | 0.0013 | 0.0010 |
| Rumen fat | Fat and greaves C3 | 0.0029 | 0.0013 | 0.0001 |
| Sanitary seizures | C1-C2 for disposal | 0.0000 | 0.0000 | 0.0000 |
| Screening and sifting wastes | C1-C2 for disposal | 0.0000 | 0.0000 | 0.0000 |
| Small intestine | PAP C3 | 0.0073 | 0.0013 | 0.0001 |
| Spinal cord | C1-C2 for disposal | 0.0000 | 0.0000 | 0.0000 |
| Spinal cord waste | C1-C2 for disposal | 0.0000 | 0.0000 | 0.0000 |
| Spine | C1-C2 for disposal | 0.0000 | 0.0000 | 0.0000 |
| Spleen | Pet food | 0.0009 | 0.0013 | 0.0001 |
| Stillborn | PAP C3 | 0.0076 | 0.0013 | 0.0000 |
| Tallow | Fat and greaves C3 | 0.0013 | 0.0013 | 0.0001 |
| Tongue | Human food | 0.0008 | 0.0013 | 0.0021 |
| Tonsil | C1-C2 for disposal | 0.0000 | 0.0000 | 0.0000 |
| Trachea | Pet food | 0.0012 | 0.0013 | 0.0001 |
| Udder | Pet food | 0.0002 | 0.0013 | 0.0001 |
| Upper throat | Pet food | 0.0009 | 0.0013 | 0.0001 |
| Water in the rumen | Spreading/Compost | 0.0000 | 0.0000 | 0.0000 |

Table 84: Allocation factors for Rouge des Prés Beef reared in Stall

| COPRODUCT | Destination | Rouge des Prés/beef/stall | | |
| --- | --- | --- | --- | --- |
|  |  | **Biophysical Allocation Factor** | **Mass Allocation Factor** | **Economic Allocation Factor** |
| Abomasum | Human food | 0.0072 | 0.0012 | 0.0009 |
| Abomasum fat | Fat and greaves C3 | 0.0026 | 0.0012 | 0.0001 |
| Aponeurosis | Human food | 0.0009 | 0.0012 | 0.0012 |
| Bile | PAP C3 | 0.0003 | 0.0012 | 0.0001 |
| Blood | PAP C3 | 0.0007 | 0.0012 | 0.0003 |
| Blood | Pet food | 0.0007 | 0.0012 | 0.0001 |
| Bones | Gelatin C3 | 0.0007 | 0.0012 | 0.0000 |
| Bones of head, brain, eyes and teeth | C1-C2 for disposal | 0.0000 | 0.0000 | 0.0000 |
| Cheek | Human food | 0.0008 | 0.0012 | 0.0025 |
| Cheek | Human food | 0.0008 | 0.0012 | 0.0025 |
| Cheek trimmings | Pet food | 0.0008 | 0.0012 | 0.0001 |
| Chops | Pet food | 0.0011 | 0.0012 | 0.0001 |
| Contents of intestines | Spreading/Compost | 0.0000 | 0.0000 | 0.0000 |
| Contents of the rumen | Spreading/Compost | 0.0000 | 0.0000 | 0.0000 |
| Ears | PAP C3 | 0.0010 | 0.0012 | 0.0001 |
| Esophagus | Pet food | 0.0008 | 0.0012 | 0.0001 |
| Fat | Fat and greaves C3 | 0.0010 | 0.0012 | 0.0001 |
| Fat around heart | Fat and greaves C3 | 0.0010 | 0.0012 | 0.0001 |
| Fat in the kidney | Fat and greaves C3 | 0.0010 | 0.0012 | 0.0001 |
| Feet (without hooves) | Gelatin C3 | 0.0008 | 0.0012 | 0.0000 |
| Floatation fat | Spreading/Compost | 0.0000 | 0.0000 | 0.0000 |
| Forehead | C1-C2 for disposal | 0.0000 | 0.0000 | 0.0000 |
| Forelock | PAP C3 | 0.0028 | 0.0012 | 0.0001 |
| Gallbladder | Pet food | 0.0008 | 0.0012 | 0.0001 |
| Head trimmings | Pet food | 0.0008 | 0.0012 | 0.0001 |
| Heart | Human food | 0.0008 | 0.0012 | 0.0002 |
| Heart trimmings | Pet food | 0.0008 | 0.0012 | 0.0001 |
| Hide | Skin tannery C3 | 0.0011 | 0.0012 | 0.0019 |
| Hooves | PAP C3 | 0.0028 | 0.0012 | 0.0001 |
| Horns | PAP C3 | 0.0028 | 0.0012 | 0.0001 |
| Kidney | Human food | 0.0008 | 0.0012 | 0.0005 |
| Large intestine | C1-C2 for disposal | 0.0000 | 0.0000 | 0.0000 |
| Liver | Human food | 0.0037 | 0.0012 | 0.0006 |
| Liver trimmings | Pet food | 0.0038 | 0.0012 | 0.0001 |
| Lower jaw | PAP C3 | 0.0007 | 0.0012 | 0.0001 |
| Lungs | Pet food | 0.0009 | 0.0012 | 0.0001 |
| Mask | Skin tannery C3 | 0.0011 | 0.0012 | 0.0019 |
| Mesenteric fat | C1-C2 for disposal | 0.0000 | 0.0000 | 0.0000 |
| Muscle | Human food | 0.0008 | 0.0012 | 0.0019 |
| Muzzle | Human food | 0.0011 | 0.0012 | 0.0012 |
| Omasum | Human food | 0.0072 | 0.0012 | 0.0009 |
| Omasum fat | Fat and greaves C3 | 0.0026 | 0.0012 | 0.0001 |
| Rumen and forestomach | Human food | 0.0072 | 0.0012 | 0.0009 |
| Rumen fat | Fat and greaves C3 | 0.0026 | 0.0012 | 0.0001 |
| Sanitary seizures | C1-C2 for disposal | 0.0000 | 0.0000 | 0.0000 |
| Screening and sifting wastes | C1-C2 for disposal | 0.0000 | 0.0000 | 0.0000 |
| Small intestine | PAP C3 | 0.0072 | 0.0012 | 0.0001 |
| Spinal cord | C1-C2 for disposal | 0.0000 | 0.0000 | 0.0000 |
| Spinal cord waste | C1-C2 for disposal | 0.0000 | 0.0000 | 0.0000 |
| Spine | C1-C2 for disposal | 0.0000 | 0.0000 | 0.0000 |
| Spleen | Pet food | 0.0008 | 0.0012 | 0.0001 |
| Stillborn | PAP C3 | 0.0075 | 0.0012 | 0.0000 |
| Tallow | Fat and greaves C3 | 0.0010 | 0.0012 | 0.0001 |
| Tongue | Human food | 0.0007 | 0.0012 | 0.0018 |
| Tonsil | C1-C2 for disposal | 0.0000 | 0.0000 | 0.0000 |
| Trachea | Pet food | 0.0011 | 0.0012 | 0.0001 |
| Udder | Pet food | 0.0002 | 0.0012 | 0.0001 |
| Upper throat | Pet food | 0.0008 | 0.0012 | 0.0001 |
| Water in the rumen | Spreading/Compost | 0.0000 | 0.0000 | 0.0000 |

Table 85: Allocation factors for Charolaise x Rustique Young Bulls reared in Grazing Large Area

| COPRODUCT | Destination | Charolaise x Rustique/young bull/grazing large area | | |
| --- | --- | --- | --- | --- |
|  |  | **Biophysical Allocation Factor** | **Mass Allocation Factor** | **Economic Allocation Factor** |
| Abomasum | Human food | 0.0088 | 0.0015 | 0.0011 |
| Abomasum fat | Fat and greaves C3 | 0.0033 | 0.0015 | 0.0001 |
| Aponeurosis | Human food | 0.0011 | 0.0015 | 0.0015 |
| Bile | PAP C3 | 0.0004 | 0.0015 | 0.0001 |
| Blood | PAP C3 | 0.0009 | 0.0015 | 0.0003 |
| Blood | Pet food | 0.0009 | 0.0015 | 0.0001 |
| Bones | Gelatin C3 | 0.0009 | 0.0015 | 0.0000 |
| Bones of head, brain, eyes and teeth | C1-C2 for disposal | 0.0000 | 0.0000 | 0.0000 |
| Cheek | Human food | 0.0010 | 0.0015 | 0.0032 |
| Cheek | Human food | 0.0010 | 0.0015 | 0.0032 |
| Cheek trimmings | Pet food | 0.0010 | 0.0015 | 0.0001 |
| Chops | Pet food | 0.0014 | 0.0015 | 0.0001 |
| Contents of intestines | Spreading/Compost | 0.0000 | 0.0000 | 0.0000 |
| Contents of the rumen | Spreading/Compost | 0.0000 | 0.0000 | 0.0000 |
| Ears | PAP C3 | 0.0013 | 0.0015 | 0.0001 |
| Esophagus | Pet food | 0.0010 | 0.0015 | 0.0001 |
| Fat | Fat and greaves C3 | 0.0014 | 0.0015 | 0.0001 |
| Fat around heart | Fat and greaves C3 | 0.0014 | 0.0015 | 0.0001 |
| Fat in the kidney | Fat and greaves C3 | 0.0014 | 0.0015 | 0.0001 |
| Feet (without hooves) | Gelatin C3 | 0.0010 | 0.0015 | 0.0000 |
| Floatation fat | Spreading/Compost | 0.0000 | 0.0000 | 0.0000 |
| Forehead | C1-C2 for disposal | 0.0000 | 0.0000 | 0.0000 |
| Forelock | PAP C3 | 0.0037 | 0.0015 | 0.0001 |
| Gallbladder | Pet food | 0.0010 | 0.0015 | 0.0001 |
| Head trimmings | Pet food | 0.0010 | 0.0015 | 0.0001 |
| Heart | Human food | 0.0010 | 0.0015 | 0.0003 |
| Heart trimmings | Pet food | 0.0010 | 0.0015 | 0.0001 |
| Hide | Skin tannery C3 | 0.0014 | 0.0015 | 0.0024 |
| Hooves | PAP C3 | 0.0037 | 0.0015 | 0.0001 |
| Horns | PAP C3 | 0.0037 | 0.0015 | 0.0001 |
| Kidney | Human food | 0.0010 | 0.0015 | 0.0006 |
| Large intestine | C1-C2 for disposal | 0.0000 | 0.0000 | 0.0000 |
| Liver | Human food | 0.0046 | 0.0015 | 0.0007 |
| Liver trimmings | Pet food | 0.0048 | 0.0015 | 0.0001 |
| Lower jaw | PAP C3 | 0.0009 | 0.0015 | 0.0001 |
| Lungs | Pet food | 0.0012 | 0.0015 | 0.0001 |
| Mask | Skin tannery C3 | 0.0014 | 0.0015 | 0.0024 |
| Mesenteric fat | C1-C2 for disposal | 0.0000 | 0.0000 | 0.0000 |
| Muscle | Human food | 0.0010 | 0.0015 | 0.0024 |
| Muzzle | Human food | 0.0014 | 0.0015 | 0.0015 |
| Omasum | Human food | 0.0088 | 0.0015 | 0.0011 |
| Omasum fat | Fat and greaves C3 | 0.0033 | 0.0015 | 0.0001 |
| Rumen and forestomach | Human food | 0.0088 | 0.0015 | 0.0011 |
| Rumen fat | Fat and greaves C3 | 0.0033 | 0.0015 | 0.0001 |
| Sanitary seizures | C1-C2 for disposal | 0.0000 | 0.0000 | 0.0000 |
| Screening and sifting wastes | C1-C2 for disposal | 0.0000 | 0.0000 | 0.0000 |
| Small intestine | PAP C3 | 0.0088 | 0.0015 | 0.0001 |
| Spinal cord | C1-C2 for disposal | 0.0000 | 0.0000 | 0.0000 |
| Spinal cord waste | C1-C2 for disposal | 0.0000 | 0.0000 | 0.0000 |
| Spine | C1-C2 for disposal | 0.0000 | 0.0000 | 0.0000 |
| Spleen | Pet food | 0.0011 | 0.0015 | 0.0001 |
| Stillborn | PAP C3 | 0.0092 | 0.0015 | 0.0000 |
| Tallow | Fat and greaves C3 | 0.0014 | 0.0015 | 0.0001 |
| Tongue | Human food | 0.0009 | 0.0015 | 0.0023 |
| Tonsil | C1-C2 for disposal | 0.0000 | 0.0000 | 0.0000 |
| Trachea | Pet food | 0.0014 | 0.0015 | 0.0001 |
| Udder | Pet food | 0.0002 | 0.0015 | 0.0001 |
| Upper throat | Pet food | 0.0010 | 0.0015 | 0.0001 |
| Water in the rumen | Spreading/Compost | 0.0000 | 0.0000 | 0.0000 |

Table 86: Allocation factors for Charolaise x Rustique Heifers reared in Grazing Large Area

| COPRODUCT | Destination | Charolaise x Rustique/heifer/grazing large area | | |
| --- | --- | --- | --- | --- |
|  |  | **Biophysical Allocation Factor** | **Mass Allocation Factor** | **Economic Allocation Factor** |
| Abomasum | Human food | 0.0094 | 0.0017 | 0.0012 |
| Abomasum fat | Fat and greaves C3 | 0.0036 | 0.0017 | 0.0002 |
| Aponeurosis | Human food | 0.0013 | 0.0017 | 0.0017 |
| Bile | PAP C3 | 0.0004 | 0.0017 | 0.0001 |
| Blood | PAP C3 | 0.0010 | 0.0017 | 0.0004 |
| Blood | Pet food | 0.0010 | 0.0017 | 0.0001 |
| Bones | Gelatin C3 | 0.0010 | 0.0017 | 0.0000 |
| Bones of head, brain, eyes and teeth | C1-C2 for disposal | 0.0000 | 0.0000 | 0.0000 |
| Cheek | Human food | 0.0011 | 0.0017 | 0.0037 |
| Cheek | Human food | 0.0011 | 0.0017 | 0.0037 |
| Cheek trimmings | Pet food | 0.0011 | 0.0017 | 0.0001 |
| Chops | Pet food | 0.0016 | 0.0017 | 0.0001 |
| Contents of intestines | Spreading/Compost | 0.0000 | 0.0000 | 0.0000 |
| Contents of the rumen | Spreading/Compost | 0.0000 | 0.0000 | 0.0000 |
| Ears | PAP C3 | 0.0014 | 0.0017 | 0.0001 |
| Esophagus | Pet food | 0.0011 | 0.0017 | 0.0001 |
| Fat | Fat and greaves C3 | 0.0015 | 0.0017 | 0.0002 |
| Fat around heart | Fat and greaves C3 | 0.0015 | 0.0017 | 0.0002 |
| Fat in the kidney | Fat and greaves C3 | 0.0015 | 0.0017 | 0.0002 |
| Feet (without hooves) | Gelatin C3 | 0.0011 | 0.0017 | 0.0000 |
| Floatation fat | Spreading/Compost | 0.0000 | 0.0000 | 0.0000 |
| Forehead | C1-C2 for disposal | 0.0000 | 0.0000 | 0.0000 |
| Forelock | PAP C3 | 0.0041 | 0.0017 | 0.0001 |
| Gallbladder | Pet food | 0.0011 | 0.0017 | 0.0001 |
| Head trimmings | Pet food | 0.0011 | 0.0017 | 0.0001 |
| Heart | Human food | 0.0011 | 0.0017 | 0.0004 |
| Heart trimmings | Pet food | 0.0011 | 0.0017 | 0.0001 |
| Hide | Skin tannery C3 | 0.0016 | 0.0017 | 0.0028 |
| Hooves | PAP C3 | 0.0041 | 0.0017 | 0.0001 |
| Horns | PAP C3 | 0.0041 | 0.0017 | 0.0001 |
| Kidney | Human food | 0.0011 | 0.0017 | 0.0007 |
| Large intestine | C1-C2 for disposal | 0.0000 | 0.0000 | 0.0000 |
| Liver | Human food | 0.0049 | 0.0017 | 0.0008 |
| Liver trimmings | Pet food | 0.0051 | 0.0017 | 0.0001 |
| Lower jaw | PAP C3 | 0.0010 | 0.0017 | 0.0001 |
| Lungs | Pet food | 0.0013 | 0.0017 | 0.0001 |
| Mask | Skin tannery C3 | 0.0016 | 0.0017 | 0.0028 |
| Mesenteric fat | C1-C2 for disposal | 0.0000 | 0.0000 | 0.0000 |
| Muscle | Human food | 0.0011 | 0.0017 | 0.0028 |
| Muzzle | Human food | 0.0016 | 0.0017 | 0.0017 |
| Omasum | Human food | 0.0094 | 0.0017 | 0.0012 |
| Omasum fat | Fat and greaves C3 | 0.0036 | 0.0017 | 0.0002 |
| Rumen and forestomach | Human food | 0.0094 | 0.0017 | 0.0012 |
| Rumen fat | Fat and greaves C3 | 0.0036 | 0.0017 | 0.0002 |
| Sanitary seizures | C1-C2 for disposal | 0.0000 | 0.0000 | 0.0000 |
| Screening and sifting wastes | C1-C2 for disposal | 0.0000 | 0.0000 | 0.0000 |
| Small intestine | PAP C3 | 0.0094 | 0.0017 | 0.0001 |
| Spinal cord | C1-C2 for disposal | 0.0000 | 0.0000 | 0.0000 |
| Spinal cord waste | C1-C2 for disposal | 0.0000 | 0.0000 | 0.0000 |
| Spine | C1-C2 for disposal | 0.0000 | 0.0000 | 0.0000 |
| Spleen | Pet food | 0.0012 | 0.0017 | 0.0001 |
| Stillborn | PAP C3 | 0.0098 | 0.0017 | 0.0000 |
| Tallow | Fat and greaves C3 | 0.0015 | 0.0017 | 0.0002 |
| Tongue | Human food | 0.0010 | 0.0017 | 0.0026 |
| Tonsil | C1-C2 for disposal | 0.0000 | 0.0000 | 0.0000 |
| Trachea | Pet food | 0.0016 | 0.0017 | 0.0001 |
| Udder | Pet food | 0.0002 | 0.0017 | 0.0001 |
| Upper throat | Pet food | 0.0011 | 0.0017 | 0.0001 |
| Water in the rumen | Spreading/Compost | 0.0000 | 0.0000 | 0.0000 |

Table 87: Allocation factors for Charolaise x Rustique Cull Cows reared in Grazing Large Area

| COPRODUCT | Destination | Charolaise x Rustique/Cull cow/grazing large area | | |
| --- | --- | --- | --- | --- |
|  |  | **Biophysical Allocation Factor** | **Mass Allocation Factor** | **Economic Allocation Factor** |
| Abomasum | Human food | 0.0088 | 0.0015 | 0.0012 |
| Abomasum fat | Fat and greaves C3 | 0.0033 | 0.0015 | 0.0001 |
| Aponeurosis | Human food | 0.0012 | 0.0015 | 0.0016 |
| Bile | PAP C3 | 0.0004 | 0.0015 | 0.0001 |
| Blood | PAP C3 | 0.0009 | 0.0015 | 0.0004 |
| Blood | Pet food | 0.0009 | 0.0015 | 0.0001 |
| Bones | Gelatin C3 | 0.0010 | 0.0015 | 0.0000 |
| Bones of head, brain, eyes and teeth | C1-C2 for disposal | 0.0000 | 0.0000 | 0.0000 |
| Cheek | Human food | 0.0010 | 0.0015 | 0.0035 |
| Cheek | Human food | 0.0010 | 0.0015 | 0.0035 |
| Cheek trimmings | Pet food | 0.0010 | 0.0015 | 0.0001 |
| Chops | Pet food | 0.0015 | 0.0015 | 0.0001 |
| Contents of intestines | Spreading/Compost | 0.0000 | 0.0000 | 0.0000 |
| Contents of the rumen | Spreading/Compost | 0.0000 | 0.0000 | 0.0000 |
| Ears | PAP C3 | 0.0013 | 0.0015 | 0.0001 |
| Esophagus | Pet food | 0.0010 | 0.0015 | 0.0001 |
| Fat | Fat and greaves C3 | 0.0014 | 0.0015 | 0.0001 |
| Fat around heart | Fat and greaves C3 | 0.0014 | 0.0015 | 0.0001 |
| Fat in the kidney | Fat and greaves C3 | 0.0014 | 0.0015 | 0.0001 |
| Feet (without hooves) | Gelatin C3 | 0.0010 | 0.0015 | 0.0000 |
| Floatation fat | Spreading/Compost | 0.0000 | 0.0000 | 0.0000 |
| Forehead | C1-C2 for disposal | 0.0000 | 0.0000 | 0.0000 |
| Forelock | PAP C3 | 0.0038 | 0.0015 | 0.0001 |
| Gallbladder | Pet food | 0.0010 | 0.0015 | 0.0001 |
| Head trimmings | Pet food | 0.0010 | 0.0015 | 0.0001 |
| Heart | Human food | 0.0010 | 0.0015 | 0.0003 |
| Heart trimmings | Pet food | 0.0010 | 0.0015 | 0.0001 |
| Hide | Skin tannery C3 | 0.0015 | 0.0015 | 0.0026 |
| Hooves | PAP C3 | 0.0038 | 0.0015 | 0.0001 |
| Horns | PAP C3 | 0.0038 | 0.0015 | 0.0001 |
| Kidney | Human food | 0.0010 | 0.0015 | 0.0006 |
| Large intestine | C1-C2 for disposal | 0.0000 | 0.0000 | 0.0000 |
| Liver | Human food | 0.0046 | 0.0015 | 0.0008 |
| Liver trimmings | Pet food | 0.0048 | 0.0015 | 0.0001 |
| Lower jaw | PAP C3 | 0.0009 | 0.0015 | 0.0001 |
| Lungs | Pet food | 0.0012 | 0.0015 | 0.0001 |
| Mask | Skin tannery C3 | 0.0015 | 0.0015 | 0.0026 |
| Mesenteric fat | C1-C2 for disposal | 0.0000 | 0.0000 | 0.0000 |
| Muscle | Human food | 0.0011 | 0.0015 | 0.0026 |
| Muzzle | Human food | 0.0015 | 0.0015 | 0.0016 |
| Omasum | Human food | 0.0088 | 0.0015 | 0.0012 |
| Omasum fat | Fat and greaves C3 | 0.0033 | 0.0015 | 0.0001 |
| Rumen and forestomach | Human food | 0.0088 | 0.0015 | 0.0012 |
| Rumen fat | Fat and greaves C3 | 0.0033 | 0.0015 | 0.0001 |
| Sanitary seizures | C1-C2 for disposal | 0.0000 | 0.0000 | 0.0000 |
| Screening and sifting wastes | C1-C2 for disposal | 0.0000 | 0.0000 | 0.0000 |
| Small intestine | PAP C3 | 0.0088 | 0.0015 | 0.0001 |
| Spinal cord | C1-C2 for disposal | 0.0000 | 0.0000 | 0.0000 |
| Spinal cord waste | C1-C2 for disposal | 0.0000 | 0.0000 | 0.0000 |
| Spine | C1-C2 for disposal | 0.0000 | 0.0000 | 0.0000 |
| Spleen | Pet food | 0.0011 | 0.0015 | 0.0001 |
| Stillborn | PAP C3 | 0.0092 | 0.0015 | 0.0000 |
| Tallow | Fat and greaves C3 | 0.0014 | 0.0015 | 0.0001 |
| Tongue | Human food | 0.0009 | 0.0015 | 0.0025 |
| Tonsil | C1-C2 for disposal | 0.0000 | 0.0000 | 0.0000 |
| Trachea | Pet food | 0.0015 | 0.0015 | 0.0001 |
| Udder | Pet food | 0.0002 | 0.0015 | 0.0001 |
| Upper throat | Pet food | 0.0010 | 0.0015 | 0.0001 |
| Water in the rumen | Spreading/Compost | 0.0000 | 0.0000 | 0.0000 |

Table 88: Allocation factors for Charolaise x Rustique Beef reared in Grazing Large Area

| COPRODUCT | Destination | Charolaise x Rustique/beef/grazing large area | | |
| --- | --- | --- | --- | --- |
|  |  | **Biophysical Allocation Factor** | **Mass Allocation Factor** | **Economic Allocation Factor** |
| Abomasum | Human food | 0.0086 | 0.0015 | 0.0011 |
| Abomasum fat | Fat and greaves C3 | 0.0032 | 0.0015 | 0.0001 |
| Aponeurosis | Human food | 0.0011 | 0.0015 | 0.0015 |
| Bile | PAP C3 | 0.0004 | 0.0015 | 0.0001 |
| Blood | PAP C3 | 0.0009 | 0.0015 | 0.0003 |
| Blood | Pet food | 0.0009 | 0.0015 | 0.0001 |
| Bones | Gelatin C3 | 0.0009 | 0.0015 | 0.0000 |
| Bones of head, brain, eyes and teeth | C1-C2 for disposal | 0.0000 | 0.0000 | 0.0000 |
| Cheek | Human food | 0.0010 | 0.0015 | 0.0032 |
| Cheek | Human food | 0.0010 | 0.0015 | 0.0032 |
| Cheek trimmings | Pet food | 0.0010 | 0.0015 | 0.0001 |
| Chops | Pet food | 0.0014 | 0.0015 | 0.0001 |
| Contents of intestines | Spreading/Compost | 0.0000 | 0.0000 | 0.0000 |
| Contents of the rumen | Spreading/Compost | 0.0000 | 0.0000 | 0.0000 |
| Ears | PAP C3 | 0.0012 | 0.0015 | 0.0001 |
| Esophagus | Pet food | 0.0010 | 0.0015 | 0.0001 |
| Fat | Fat and greaves C3 | 0.0013 | 0.0015 | 0.0001 |
| Fat around heart | Fat and greaves C3 | 0.0013 | 0.0015 | 0.0001 |
| Fat in the kidney | Fat and greaves C3 | 0.0013 | 0.0015 | 0.0001 |
| Feet (without hooves) | Gelatin C3 | 0.0010 | 0.0015 | 0.0000 |
| Floatation fat | Spreading/Compost | 0.0000 | 0.0000 | 0.0000 |
| Forehead | C1-C2 for disposal | 0.0000 | 0.0000 | 0.0000 |
| Forelock | PAP C3 | 0.0036 | 0.0015 | 0.0001 |
| Gallbladder | Pet food | 0.0010 | 0.0015 | 0.0001 |
| Head trimmings | Pet food | 0.0010 | 0.0015 | 0.0001 |
| Heart | Human food | 0.0010 | 0.0015 | 0.0003 |
| Heart trimmings | Pet food | 0.0010 | 0.0015 | 0.0001 |
| Hide | Skin tannery C3 | 0.0014 | 0.0015 | 0.0024 |
| Hooves | PAP C3 | 0.0036 | 0.0015 | 0.0001 |
| Horns | PAP C3 | 0.0036 | 0.0015 | 0.0001 |
| Kidney | Human food | 0.0010 | 0.0015 | 0.0006 |
| Large intestine | C1-C2 for disposal | 0.0000 | 0.0000 | 0.0000 |
| Liver | Human food | 0.0044 | 0.0015 | 0.0007 |
| Liver trimmings | Pet food | 0.0046 | 0.0015 | 0.0001 |
| Lower jaw | PAP C3 | 0.0009 | 0.0015 | 0.0001 |
| Lungs | Pet food | 0.0012 | 0.0015 | 0.0001 |
| Mask | Skin tannery C3 | 0.0014 | 0.0015 | 0.0024 |
| Mesenteric fat | C1-C2 for disposal | 0.0000 | 0.0000 | 0.0000 |
| Muscle | Human food | 0.0010 | 0.0015 | 0.0024 |
| Muzzle | Human food | 0.0014 | 0.0015 | 0.0015 |
| Omasum | Human food | 0.0086 | 0.0015 | 0.0011 |
| Omasum fat | Fat and greaves C3 | 0.0032 | 0.0015 | 0.0001 |
| Rumen and forestomach | Human food | 0.0086 | 0.0015 | 0.0011 |
| Rumen fat | Fat and greaves C3 | 0.0032 | 0.0015 | 0.0001 |
| Sanitary seizures | C1-C2 for disposal | 0.0000 | 0.0000 | 0.0000 |
| Screening and sifting wastes | C1-C2 for disposal | 0.0000 | 0.0000 | 0.0000 |
| Small intestine | PAP C3 | 0.0086 | 0.0015 | 0.0001 |
| Spinal cord | C1-C2 for disposal | 0.0000 | 0.0000 | 0.0000 |
| Spinal cord waste | C1-C2 for disposal | 0.0000 | 0.0000 | 0.0000 |
| Spine | C1-C2 for disposal | 0.0000 | 0.0000 | 0.0000 |
| Spleen | Pet food | 0.0010 | 0.0015 | 0.0001 |
| Stillborn | PAP C3 | 0.0090 | 0.0015 | 0.0000 |
| Tallow | Fat and greaves C3 | 0.0013 | 0.0015 | 0.0001 |
| Tongue | Human food | 0.0009 | 0.0015 | 0.0023 |
| Tonsil | C1-C2 for disposal | 0.0000 | 0.0000 | 0.0000 |
| Trachea | Pet food | 0.0014 | 0.0015 | 0.0001 |
| Udder | Pet food | 0.0002 | 0.0015 | 0.0001 |
| Upper throat | Pet food | 0.0010 | 0.0015 | 0.0001 |
| Water in the rumen | Spreading/Compost | 0.0000 | 0.0000 | 0.0000 |

Table 89: Allocation factors for Charolaise x Rustique Young Bulls reared in Pasture

| COPRODUCT | Destination | Charolaise x Rustique/young bull/pasture | | |
| --- | --- | --- | --- | --- |
|  |  | **Biophysical Allocation Factor** | **Mass Allocation Factor** | **Economic Allocation Factor** |
| Abomasum | Human food | 0.0084 | 0.0015 | 0.0011 |
| Abomasum fat | Fat and greaves C3 | 0.0033 | 0.0015 | 0.0001 |
| Aponeurosis | Human food | 0.0011 | 0.0015 | 0.0015 |
| Bile | PAP C3 | 0.0004 | 0.0015 | 0.0001 |
| Blood | PAP C3 | 0.0009 | 0.0015 | 0.0003 |
| Blood | Pet food | 0.0009 | 0.0015 | 0.0001 |
| Bones | Gelatin C3 | 0.0010 | 0.0015 | 0.0000 |
| Bones of head, brain, eyes and teeth | C1-C2 for disposal | 0.0000 | 0.0000 | 0.0000 |
| Cheek | Human food | 0.0010 | 0.0015 | 0.0032 |
| Cheek | Human food | 0.0010 | 0.0015 | 0.0032 |
| Cheek trimmings | Pet food | 0.0010 | 0.0015 | 0.0001 |
| Chops | Pet food | 0.0014 | 0.0015 | 0.0001 |
| Contents of intestines | Spreading/Compost | 0.0000 | 0.0000 | 0.0000 |
| Contents of the rumen | Spreading/Compost | 0.0000 | 0.0000 | 0.0000 |
| Ears | PAP C3 | 0.0013 | 0.0015 | 0.0001 |
| Esophagus | Pet food | 0.0010 | 0.0015 | 0.0001 |
| Fat | Fat and greaves C3 | 0.0015 | 0.0015 | 0.0001 |
| Fat around heart | Fat and greaves C3 | 0.0015 | 0.0015 | 0.0001 |
| Fat in the kidney | Fat and greaves C3 | 0.0015 | 0.0015 | 0.0001 |
| Feet (without hooves) | Gelatin C3 | 0.0010 | 0.0015 | 0.0000 |
| Floatation fat | Spreading/Compost | 0.0000 | 0.0000 | 0.0000 |
| Forehead | C1-C2 for disposal | 0.0000 | 0.0000 | 0.0000 |
| Forelock | PAP C3 | 0.0037 | 0.0015 | 0.0001 |
| Gallbladder | Pet food | 0.0010 | 0.0015 | 0.0001 |
| Head trimmings | Pet food | 0.0010 | 0.0015 | 0.0001 |
| Heart | Human food | 0.0010 | 0.0015 | 0.0003 |
| Heart trimmings | Pet food | 0.0010 | 0.0015 | 0.0001 |
| Hide | Skin tannery C3 | 0.0014 | 0.0015 | 0.0024 |
| Hooves | PAP C3 | 0.0037 | 0.0015 | 0.0001 |
| Horns | PAP C3 | 0.0037 | 0.0015 | 0.0001 |
| Kidney | Human food | 0.0010 | 0.0015 | 0.0006 |
| Large intestine | C1-C2 for disposal | 0.0000 | 0.0000 | 0.0000 |
| Liver | Human food | 0.0044 | 0.0015 | 0.0007 |
| Liver trimmings | Pet food | 0.0046 | 0.0015 | 0.0001 |
| Lower jaw | PAP C3 | 0.0009 | 0.0015 | 0.0001 |
| Lungs | Pet food | 0.0012 | 0.0015 | 0.0001 |
| Mask | Skin tannery C3 | 0.0014 | 0.0015 | 0.0024 |
| Mesenteric fat | C1-C2 for disposal | 0.0000 | 0.0000 | 0.0000 |
| Muscle | Human food | 0.0010 | 0.0015 | 0.0024 |
| Muzzle | Human food | 0.0014 | 0.0015 | 0.0015 |
| Omasum | Human food | 0.0084 | 0.0015 | 0.0011 |
| Omasum fat | Fat and greaves C3 | 0.0033 | 0.0015 | 0.0001 |
| Rumen and forestomach | Human food | 0.0084 | 0.0015 | 0.0011 |
| Rumen fat | Fat and greaves C3 | 0.0033 | 0.0015 | 0.0001 |
| Sanitary seizures | C1-C2 for disposal | 0.0000 | 0.0000 | 0.0000 |
| Screening and sifting wastes | C1-C2 for disposal | 0.0000 | 0.0000 | 0.0000 |
| Small intestine | PAP C3 | 0.0084 | 0.0015 | 0.0001 |
| Spinal cord | C1-C2 for disposal | 0.0000 | 0.0000 | 0.0000 |
| Spinal cord waste | C1-C2 for disposal | 0.0000 | 0.0000 | 0.0000 |
| Spine | C1-C2 for disposal | 0.0000 | 0.0000 | 0.0000 |
| Spleen | Pet food | 0.0010 | 0.0015 | 0.0001 |
| Stillborn | PAP C3 | 0.0088 | 0.0015 | 0.0000 |
| Tallow | Fat and greaves C3 | 0.0015 | 0.0015 | 0.0001 |
| Tongue | Human food | 0.0009 | 0.0015 | 0.0023 |
| Tonsil | C1-C2 for disposal | 0.0000 | 0.0000 | 0.0000 |
| Trachea | Pet food | 0.0014 | 0.0015 | 0.0001 |
| Udder | Pet food | 0.0002 | 0.0015 | 0.0001 |
| Upper throat | Pet food | 0.0010 | 0.0015 | 0.0001 |
| Water in the rumen | Spreading/Compost | 0.0000 | 0.0000 | 0.0000 |

Table 90: Allocation factors for Charolaise x Rustique Heifers reared in Pasture

| COPRODUCT | Destination | Charolaise x Rustique/heifer/pasture | | |
| --- | --- | --- | --- | --- |
|  |  | **Biophysical Allocation Factor** | **Mass Allocation Factor** | **Economic Allocation Factor** |
| Abomasum | Human food | 0.0090 | 0.0017 | 0.0012 |
| Abomasum fat | Fat and greaves C3 | 0.0036 | 0.0017 | 0.0002 |
| Aponeurosis | Human food | 0.0012 | 0.0017 | 0.0017 |
| Bile | PAP C3 | 0.0004 | 0.0017 | 0.0001 |
| Blood | PAP C3 | 0.0010 | 0.0017 | 0.0004 |
| Blood | Pet food | 0.0010 | 0.0017 | 0.0001 |
| Bones | Gelatin C3 | 0.0011 | 0.0017 | 0.0000 |
| Bones of head, brain, eyes and teeth | C1-C2 for disposal | 0.0000 | 0.0000 | 0.0000 |
| Cheek | Human food | 0.0011 | 0.0017 | 0.0037 |
| Cheek | Human food | 0.0011 | 0.0017 | 0.0037 |
| Cheek trimmings | Pet food | 0.0011 | 0.0017 | 0.0001 |
| Chops | Pet food | 0.0016 | 0.0017 | 0.0001 |
| Contents of intestines | Spreading/Compost | 0.0000 | 0.0000 | 0.0000 |
| Contents of the rumen | Spreading/Compost | 0.0000 | 0.0000 | 0.0000 |
| Ears | PAP C3 | 0.0014 | 0.0017 | 0.0001 |
| Esophagus | Pet food | 0.0011 | 0.0017 | 0.0001 |
| Fat | Fat and greaves C3 | 0.0017 | 0.0017 | 0.0002 |
| Fat around heart | Fat and greaves C3 | 0.0017 | 0.0017 | 0.0002 |
| Fat in the kidney | Fat and greaves C3 | 0.0017 | 0.0017 | 0.0002 |
| Feet (without hooves) | Gelatin C3 | 0.0011 | 0.0017 | 0.0000 |
| Floatation fat | Spreading/Compost | 0.0000 | 0.0000 | 0.0000 |
| Forehead | C1-C2 for disposal | 0.0000 | 0.0000 | 0.0000 |
| Forelock | PAP C3 | 0.0041 | 0.0017 | 0.0001 |
| Gallbladder | Pet food | 0.0011 | 0.0017 | 0.0001 |
| Head trimmings | Pet food | 0.0011 | 0.0017 | 0.0001 |
| Heart | Human food | 0.0011 | 0.0017 | 0.0004 |
| Heart trimmings | Pet food | 0.0011 | 0.0017 | 0.0001 |
| Hide | Skin tannery C3 | 0.0016 | 0.0017 | 0.0028 |
| Hooves | PAP C3 | 0.0041 | 0.0017 | 0.0001 |
| Horns | PAP C3 | 0.0041 | 0.0017 | 0.0001 |
| Kidney | Human food | 0.0011 | 0.0017 | 0.0007 |
| Large intestine | C1-C2 for disposal | 0.0000 | 0.0000 | 0.0000 |
| Liver | Human food | 0.0047 | 0.0017 | 0.0008 |
| Liver trimmings | Pet food | 0.0049 | 0.0017 | 0.0001 |
| Lower jaw | PAP C3 | 0.0010 | 0.0017 | 0.0001 |
| Lungs | Pet food | 0.0013 | 0.0017 | 0.0001 |
| Mask | Skin tannery C3 | 0.0016 | 0.0017 | 0.0028 |
| Mesenteric fat | C1-C2 for disposal | 0.0000 | 0.0000 | 0.0000 |
| Muscle | Human food | 0.0011 | 0.0017 | 0.0028 |
| Muzzle | Human food | 0.0016 | 0.0017 | 0.0017 |
| Omasum | Human food | 0.0090 | 0.0017 | 0.0012 |
| Omasum fat | Fat and greaves C3 | 0.0036 | 0.0017 | 0.0002 |
| Rumen and forestomach | Human food | 0.0090 | 0.0017 | 0.0012 |
| Rumen fat | Fat and greaves C3 | 0.0036 | 0.0017 | 0.0002 |
| Sanitary seizures | C1-C2 for disposal | 0.0000 | 0.0000 | 0.0000 |
| Screening and sifting wastes | C1-C2 for disposal | 0.0000 | 0.0000 | 0.0000 |
| Small intestine | PAP C3 | 0.0090 | 0.0017 | 0.0001 |
| Spinal cord | C1-C2 for disposal | 0.0000 | 0.0000 | 0.0000 |
| Spinal cord waste | C1-C2 for disposal | 0.0000 | 0.0000 | 0.0000 |
| Spine | C1-C2 for disposal | 0.0000 | 0.0000 | 0.0000 |
| Spleen | Pet food | 0.0012 | 0.0017 | 0.0001 |
| Stillborn | PAP C3 | 0.0094 | 0.0017 | 0.0000 |
| Tallow | Fat and greaves C3 | 0.0017 | 0.0017 | 0.0002 |
| Tongue | Human food | 0.0010 | 0.0017 | 0.0026 |
| Tonsil | C1-C2 for disposal | 0.0000 | 0.0000 | 0.0000 |
| Trachea | Pet food | 0.0016 | 0.0017 | 0.0001 |
| Udder | Pet food | 0.0002 | 0.0017 | 0.0001 |
| Upper throat | Pet food | 0.0011 | 0.0017 | 0.0001 |
| Water in the rumen | Spreading/Compost | 0.0000 | 0.0000 | 0.0000 |

Table 91: Allocation factors for Charolaise x Rustique Cull Cows reared in Pasture

| COPRODUCT | Destination | Charolaise x Rustique/Cull cow/pasture | | |
| --- | --- | --- | --- | --- |
|  |  | **Biophysical Allocation Factor** | **Mass Allocation Factor** | **Economic Allocation Factor** |
| Abomasum | Human food | 0.0084 | 0.0015 | 0.0012 |
| Abomasum fat | Fat and greaves C3 | 0.0033 | 0.0015 | 0.0001 |
| Aponeurosis | Human food | 0.0011 | 0.0015 | 0.0016 |
| Bile | PAP C3 | 0.0004 | 0.0015 | 0.0001 |
| Blood | PAP C3 | 0.0009 | 0.0015 | 0.0004 |
| Blood | Pet food | 0.0009 | 0.0015 | 0.0001 |
| Bones | Gelatin C3 | 0.0010 | 0.0015 | 0.0000 |
| Bones of head, brain, eyes and teeth | C1-C2 for disposal | 0.0000 | 0.0000 | 0.0000 |
| Cheek | Human food | 0.0010 | 0.0015 | 0.0035 |
| Cheek | Human food | 0.0010 | 0.0015 | 0.0035 |
| Cheek trimmings | Pet food | 0.0010 | 0.0015 | 0.0001 |
| Chops | Pet food | 0.0015 | 0.0015 | 0.0001 |
| Contents of intestines | Spreading/Compost | 0.0000 | 0.0000 | 0.0000 |
| Contents of the rumen | Spreading/Compost | 0.0000 | 0.0000 | 0.0000 |
| Ears | PAP C3 | 0.0013 | 0.0015 | 0.0001 |
| Esophagus | Pet food | 0.0010 | 0.0015 | 0.0001 |
| Fat | Fat and greaves C3 | 0.0015 | 0.0015 | 0.0001 |
| Fat around heart | Fat and greaves C3 | 0.0015 | 0.0015 | 0.0001 |
| Fat in the kidney | Fat and greaves C3 | 0.0015 | 0.0015 | 0.0001 |
| Feet (without hooves) | Gelatin C3 | 0.0010 | 0.0015 | 0.0000 |
| Floatation fat | Spreading/Compost | 0.0000 | 0.0000 | 0.0000 |
| Forehead | C1-C2 for disposal | 0.0000 | 0.0000 | 0.0000 |
| Forelock | PAP C3 | 0.0037 | 0.0015 | 0.0001 |
| Gallbladder | Pet food | 0.0010 | 0.0015 | 0.0001 |
| Head trimmings | Pet food | 0.0010 | 0.0015 | 0.0001 |
| Heart | Human food | 0.0010 | 0.0015 | 0.0003 |
| Heart trimmings | Pet food | 0.0010 | 0.0015 | 0.0001 |
| Hide | Skin tannery C3 | 0.0015 | 0.0015 | 0.0026 |
| Hooves | PAP C3 | 0.0037 | 0.0015 | 0.0001 |
| Horns | PAP C3 | 0.0037 | 0.0015 | 0.0001 |
| Kidney | Human food | 0.0010 | 0.0015 | 0.0006 |
| Large intestine | C1-C2 for disposal | 0.0000 | 0.0000 | 0.0000 |
| Liver | Human food | 0.0044 | 0.0015 | 0.0008 |
| Liver trimmings | Pet food | 0.0046 | 0.0015 | 0.0001 |
| Lower jaw | PAP C3 | 0.0010 | 0.0015 | 0.0001 |
| Lungs | Pet food | 0.0012 | 0.0015 | 0.0001 |
| Mask | Skin tannery C3 | 0.0015 | 0.0015 | 0.0026 |
| Mesenteric fat | C1-C2 for disposal | 0.0000 | 0.0000 | 0.0000 |
| Muscle | Human food | 0.0011 | 0.0015 | 0.0026 |
| Muzzle | Human food | 0.0015 | 0.0015 | 0.0016 |
| Omasum | Human food | 0.0084 | 0.0015 | 0.0012 |
| Omasum fat | Fat and greaves C3 | 0.0033 | 0.0015 | 0.0001 |
| Rumen and forestomach | Human food | 0.0084 | 0.0015 | 0.0012 |
| Rumen fat | Fat and greaves C3 | 0.0033 | 0.0015 | 0.0001 |
| Sanitary seizures | C1-C2 for disposal | 0.0000 | 0.0000 | 0.0000 |
| Screening and sifting wastes | C1-C2 for disposal | 0.0000 | 0.0000 | 0.0000 |
| Small intestine | PAP C3 | 0.0084 | 0.0015 | 0.0001 |
| Spinal cord | C1-C2 for disposal | 0.0000 | 0.0000 | 0.0000 |
| Spinal cord waste | C1-C2 for disposal | 0.0000 | 0.0000 | 0.0000 |
| Spine | C1-C2 for disposal | 0.0000 | 0.0000 | 0.0000 |
| Spleen | Pet food | 0.0011 | 0.0015 | 0.0001 |
| Stillborn | PAP C3 | 0.0088 | 0.0015 | 0.0000 |
| Tallow | Fat and greaves C3 | 0.0015 | 0.0015 | 0.0001 |
| Tongue | Human food | 0.0009 | 0.0015 | 0.0025 |
| Tonsil | C1-C2 for disposal | 0.0000 | 0.0000 | 0.0000 |
| Trachea | Pet food | 0.0015 | 0.0015 | 0.0001 |
| Udder | Pet food | 0.0002 | 0.0015 | 0.0001 |
| Upper throat | Pet food | 0.0010 | 0.0015 | 0.0001 |
| Water in the rumen | Spreading/Compost | 0.0000 | 0.0000 | 0.0000 |

Table 92: Allocation factors for Charolaise x Rustique Beef reared in Pasture

| COPRODUCT | Destination | Charolaise x Rustique/beef/pasture | | |
| --- | --- | --- | --- | --- |
|  |  | **Biophysical Allocation Factor** | **Mass Allocation Factor** | **Economic Allocation Factor** |
| Abomasum | Human food | 0.0082 | 0.0015 | 0.0011 |
| Abomasum fat | Fat and greaves C3 | 0.0032 | 0.0015 | 0.0001 |
| Aponeurosis | Human food | 0.0011 | 0.0015 | 0.0015 |
| Bile | PAP C3 | 0.0004 | 0.0015 | 0.0001 |
| Blood | PAP C3 | 0.0008 | 0.0015 | 0.0003 |
| Blood | Pet food | 0.0008 | 0.0015 | 0.0001 |
| Bones | Gelatin C3 | 0.0009 | 0.0015 | 0.0000 |
| Bones of head, brain, eyes and teeth | C1-C2 for disposal | 0.0000 | 0.0000 | 0.0000 |
| Cheek | Human food | 0.0010 | 0.0015 | 0.0032 |
| Cheek | Human food | 0.0010 | 0.0015 | 0.0032 |
| Cheek trimmings | Pet food | 0.0010 | 0.0015 | 0.0001 |
| Chops | Pet food | 0.0014 | 0.0015 | 0.0001 |
| Contents of intestines | Spreading/Compost | 0.0000 | 0.0000 | 0.0000 |
| Contents of the rumen | Spreading/Compost | 0.0000 | 0.0000 | 0.0000 |
| Ears | PAP C3 | 0.0012 | 0.0015 | 0.0001 |
| Esophagus | Pet food | 0.0010 | 0.0015 | 0.0001 |
| Fat | Fat and greaves C3 | 0.0015 | 0.0015 | 0.0001 |
| Fat around heart | Fat and greaves C3 | 0.0015 | 0.0015 | 0.0001 |
| Fat in the kidney | Fat and greaves C3 | 0.0015 | 0.0015 | 0.0001 |
| Feet (without hooves) | Gelatin C3 | 0.0010 | 0.0015 | 0.0000 |
| Floatation fat | Spreading/Compost | 0.0000 | 0.0000 | 0.0000 |
| Forehead | C1-C2 for disposal | 0.0000 | 0.0000 | 0.0000 |
| Forelock | PAP C3 | 0.0036 | 0.0015 | 0.0001 |
| Gallbladder | Pet food | 0.0010 | 0.0015 | 0.0001 |
| Head trimmings | Pet food | 0.0010 | 0.0015 | 0.0001 |
| Heart | Human food | 0.0010 | 0.0015 | 0.0003 |
| Heart trimmings | Pet food | 0.0010 | 0.0015 | 0.0001 |
| Hide | Skin tannery C3 | 0.0014 | 0.0015 | 0.0024 |
| Hooves | PAP C3 | 0.0036 | 0.0015 | 0.0001 |
| Horns | PAP C3 | 0.0036 | 0.0015 | 0.0001 |
| Kidney | Human food | 0.0010 | 0.0015 | 0.0006 |
| Large intestine | C1-C2 for disposal | 0.0000 | 0.0000 | 0.0000 |
| Liver | Human food | 0.0043 | 0.0015 | 0.0007 |
| Liver trimmings | Pet food | 0.0045 | 0.0015 | 0.0001 |
| Lower jaw | PAP C3 | 0.0009 | 0.0015 | 0.0001 |
| Lungs | Pet food | 0.0011 | 0.0015 | 0.0001 |
| Mask | Skin tannery C3 | 0.0014 | 0.0015 | 0.0024 |
| Mesenteric fat | C1-C2 for disposal | 0.0000 | 0.0000 | 0.0000 |
| Muscle | Human food | 0.0010 | 0.0015 | 0.0024 |
| Muzzle | Human food | 0.0014 | 0.0015 | 0.0015 |
| Omasum | Human food | 0.0082 | 0.0015 | 0.0011 |
| Omasum fat | Fat and greaves C3 | 0.0032 | 0.0015 | 0.0001 |
| Rumen and forestomach | Human food | 0.0082 | 0.0015 | 0.0011 |
| Rumen fat | Fat and greaves C3 | 0.0032 | 0.0015 | 0.0001 |
| Sanitary seizures | C1-C2 for disposal | 0.0000 | 0.0000 | 0.0000 |
| Screening and sifting wastes | C1-C2 for disposal | 0.0000 | 0.0000 | 0.0000 |
| Small intestine | PAP C3 | 0.0082 | 0.0015 | 0.0001 |
| Spinal cord | C1-C2 for disposal | 0.0000 | 0.0000 | 0.0000 |
| Spinal cord waste | C1-C2 for disposal | 0.0000 | 0.0000 | 0.0000 |
| Spine | C1-C2 for disposal | 0.0000 | 0.0000 | 0.0000 |
| Spleen | Pet food | 0.0010 | 0.0015 | 0.0001 |
| Stillborn | PAP C3 | 0.0086 | 0.0015 | 0.0000 |
| Tallow | Fat and greaves C3 | 0.0015 | 0.0015 | 0.0001 |
| Tongue | Human food | 0.0009 | 0.0015 | 0.0023 |
| Tonsil | C1-C2 for disposal | 0.0000 | 0.0000 | 0.0000 |
| Trachea | Pet food | 0.0014 | 0.0015 | 0.0001 |
| Udder | Pet food | 0.0002 | 0.0015 | 0.0001 |
| Upper throat | Pet food | 0.0010 | 0.0015 | 0.0001 |
| Water in the rumen | Spreading/Compost | 0.0000 | 0.0000 | 0.0000 |

Table 93: Allocation factors for Charolaise x Rustique Young Bulls reared in Stall

| COPRODUCT | Destination | Charolaise x Rustique/young bull/stall | | |
| --- | --- | --- | --- | --- |
|  |  | **Biophysical Allocation Factor** | **Mass Allocation Factor** | **Economic Allocation Factor** |
| Abomasum | Human food | 0.0033 | 0.0015 | 0.0001 |
| Abomasum fat | Fat and greaves C3 | 0.0011 | 0.0015 | 0.0015 |
| Aponeurosis | Human food | 0.0004 | 0.0015 | 0.0001 |
| Bile | PAP C3 | 0.0009 | 0.0015 | 0.0003 |
| Blood | PAP C3 | 0.0009 | 0.0015 | 0.0001 |
| Blood | Pet food | 0.0010 | 0.0015 | 0.0000 |
| Bones | Gelatin C3 | 0.0000 | 0.0000 | 0.0000 |
| Bones of head, brain, eyes and teeth | C1-C2 for disposal | 0.0010 | 0.0015 | 0.0032 |
| Cheek | Human food | 0.0010 | 0.0015 | 0.0032 |
| Cheek | Human food | 0.0010 | 0.0015 | 0.0001 |
| Cheek trimmings | Pet food | 0.0014 | 0.0015 | 0.0001 |
| Chops | Pet food | 0.0000 | 0.0000 | 0.0000 |
| Contents of intestines | Spreading/Compost | 0.0000 | 0.0000 | 0.0000 |
| Contents of the rumen | Spreading/Compost | 0.0013 | 0.0015 | 0.0001 |
| Ears | PAP C3 | 0.0010 | 0.0015 | 0.0001 |
| Esophagus | Pet food | 0.0017 | 0.0015 | 0.0001 |
| Fat | Fat and greaves C3 | 0.0017 | 0.0015 | 0.0001 |
| Fat around heart | Fat and greaves C3 | 0.0017 | 0.0015 | 0.0001 |
| Fat in the kidney | Fat and greaves C3 | 0.0010 | 0.0015 | 0.0000 |
| Feet (without hooves) | Gelatin C3 | 0.0000 | 0.0000 | 0.0000 |
| Floatation fat | Spreading/Compost | 0.0000 | 0.0000 | 0.0000 |
| Forehead | C1-C2 for disposal | 0.0036 | 0.0015 | 0.0001 |
| Forelock | PAP C3 | 0.0010 | 0.0015 | 0.0001 |
| Gallbladder | Pet food | 0.0010 | 0.0015 | 0.0001 |
| Head trimmings | Pet food | 0.0010 | 0.0015 | 0.0003 |
| Heart | Human food | 0.0010 | 0.0015 | 0.0001 |
| Heart trimmings | Pet food | 0.0014 | 0.0015 | 0.0024 |
| Hide | Skin tannery C3 | 0.0036 | 0.0015 | 0.0001 |
| Hooves | PAP C3 | 0.0036 | 0.0015 | 0.0001 |
| Horns | PAP C3 | 0.0010 | 0.0015 | 0.0006 |
| Kidney | Human food | 0.0000 | 0.0000 | 0.0000 |
| Large intestine | C1-C2 for disposal | 0.0042 | 0.0015 | 0.0007 |
| Liver | Human food | 0.0044 | 0.0015 | 0.0001 |
| Liver trimmings | Pet food | 0.0010 | 0.0015 | 0.0001 |
| Lower jaw | PAP C3 | 0.0012 | 0.0015 | 0.0001 |
| Lungs | Pet food | 0.0014 | 0.0015 | 0.0024 |
| Mask | Skin tannery C3 | 0.0000 | 0.0000 | 0.0000 |
| Mesenteric fat | C1-C2 for disposal | 0.0010 | 0.0015 | 0.0024 |
| Muscle | Human food | 0.0014 | 0.0015 | 0.0015 |
| Muzzle | Human food | 0.0080 | 0.0015 | 0.0011 |
| Omasum | Human food | 0.0033 | 0.0015 | 0.0001 |
| Omasum fat | Fat and greaves C3 | 0.0080 | 0.0015 | 0.0011 |
| Rumen and forestomach | Human food | 0.0033 | 0.0015 | 0.0001 |
| Rumen fat | Fat and greaves C3 | 0.0000 | 0.0000 | 0.0000 |
| Sanitary seizures | C1-C2 for disposal | 0.0000 | 0.0000 | 0.0000 |
| Screening and sifting wastes | C1-C2 for disposal | 0.0080 | 0.0015 | 0.0001 |
| Small intestine | PAP C3 | 0.0000 | 0.0000 | 0.0000 |
| Spinal cord | C1-C2 for disposal | 0.0000 | 0.0000 | 0.0000 |
| Spinal cord waste | C1-C2 for disposal | 0.0000 | 0.0000 | 0.0000 |
| Spine | C1-C2 for disposal | 0.0010 | 0.0015 | 0.0001 |
| Spleen | Pet food | 0.0084 | 0.0015 | 0.0000 |
| Stillborn | PAP C3 | 0.0017 | 0.0015 | 0.0001 |
| Tallow | Fat and greaves C3 | 0.0009 | 0.0015 | 0.0023 |
| Tongue | Human food | 0.0000 | 0.0000 | 0.0000 |
| Tonsil | C1-C2 for disposal | 0.0014 | 0.0015 | 0.0001 |
| Trachea | Pet food | 0.0002 | 0.0015 | 0.0001 |
| Udder | Pet food | 0.0010 | 0.0015 | 0.0001 |
| Upper throat | Pet food | 0.0000 | 0.0000 | 0.0000 |
| Water in the rumen | Spreading/Compost | 0.0033 | 0.0015 | 0.0001 |

Table 94: Allocation factors for Charolaise x Rustique Heifers reared in Stall

| COPRODUCT | Destination | Charolaise x Rustique/heifer/stall | | |
| --- | --- | --- | --- | --- |
|  |  | **Biophysical Allocation Factor** | **Mass Allocation Factor** | **Economic Allocation Factor** |
| Abomasum | Human food | 0.0085 | 0.0017 | 0.0012 |
| Abomasum fat | Fat and greaves C3 | 0.0036 | 0.0017 | 0.0002 |
| Aponeurosis | Human food | 0.0012 | 0.0017 | 0.0017 |
| Bile | PAP C3 | 0.0004 | 0.0017 | 0.0001 |
| Blood | PAP C3 | 0.0010 | 0.0017 | 0.0004 |
| Blood | Pet food | 0.0010 | 0.0017 | 0.0001 |
| Bones | Gelatin C3 | 0.0011 | 0.0017 | 0.0000 |
| Bones of head, brain, eyes and teeth | C1-C2 for disposal | 0.0000 | 0.0000 | 0.0000 |
| Cheek | Human food | 0.0011 | 0.0017 | 0.0037 |
| Cheek | Human food | 0.0011 | 0.0017 | 0.0037 |
| Cheek trimmings | Pet food | 0.0011 | 0.0017 | 0.0001 |
| Chops | Pet food | 0.0016 | 0.0017 | 0.0001 |
| Contents of intestines | Spreading/Compost | 0.0000 | 0.0000 | 0.0000 |
| Contents of the rumen | Spreading/Compost | 0.0000 | 0.0000 | 0.0000 |
| Ears | PAP C3 | 0.0014 | 0.0017 | 0.0001 |
| Esophagus | Pet food | 0.0011 | 0.0017 | 0.0001 |
| Fat | Fat and greaves C3 | 0.0018 | 0.0017 | 0.0002 |
| Fat around heart | Fat and greaves C3 | 0.0018 | 0.0017 | 0.0002 |
| Fat in the kidney | Fat and greaves C3 | 0.0018 | 0.0017 | 0.0002 |
| Feet (without hooves) | Gelatin C3 | 0.0011 | 0.0017 | 0.0000 |
| Floatation fat | Spreading/Compost | 0.0000 | 0.0000 | 0.0000 |
| Forehead | C1-C2 for disposal | 0.0000 | 0.0000 | 0.0000 |
| Forelock | PAP C3 | 0.0040 | 0.0017 | 0.0001 |
| Gallbladder | Pet food | 0.0011 | 0.0017 | 0.0001 |
| Head trimmings | Pet food | 0.0011 | 0.0017 | 0.0001 |
| Heart | Human food | 0.0011 | 0.0017 | 0.0004 |
| Heart trimmings | Pet food | 0.0011 | 0.0017 | 0.0001 |
| Hide | Skin tannery C3 | 0.0016 | 0.0017 | 0.0028 |
| Hooves | PAP C3 | 0.0040 | 0.0017 | 0.0001 |
| Horns | PAP C3 | 0.0040 | 0.0017 | 0.0001 |
| Kidney | Human food | 0.0011 | 0.0017 | 0.0007 |
| Large intestine | C1-C2 for disposal | 0.0000 | 0.0000 | 0.0000 |
| Liver | Human food | 0.0045 | 0.0017 | 0.0008 |
| Liver trimmings | Pet food | 0.0047 | 0.0017 | 0.0001 |
| Lower jaw | PAP C3 | 0.0011 | 0.0017 | 0.0001 |
| Lungs | Pet food | 0.0013 | 0.0017 | 0.0001 |
| Mask | Skin tannery C3 | 0.0016 | 0.0017 | 0.0028 |
| Mesenteric fat | C1-C2 for disposal | 0.0000 | 0.0000 | 0.0000 |
| Muscle | Human food | 0.0011 | 0.0017 | 0.0028 |
| Muzzle | Human food | 0.0016 | 0.0017 | 0.0017 |
| Omasum | Human food | 0.0085 | 0.0017 | 0.0012 |
| Omasum fat | Fat and greaves C3 | 0.0036 | 0.0017 | 0.0002 |
| Rumen and forestomach | Human food | 0.0085 | 0.0017 | 0.0012 |
| Rumen fat | Fat and greaves C3 | 0.0036 | 0.0017 | 0.0002 |
| Sanitary seizures | C1-C2 for disposal | 0.0000 | 0.0000 | 0.0000 |
| Screening and sifting wastes | C1-C2 for disposal | 0.0000 | 0.0000 | 0.0000 |
| Small intestine | PAP C3 | 0.0085 | 0.0017 | 0.0001 |
| Spinal cord | C1-C2 for disposal | 0.0000 | 0.0000 | 0.0000 |
| Spinal cord waste | C1-C2 for disposal | 0.0000 | 0.0000 | 0.0000 |
| Spine | C1-C2 for disposal | 0.0000 | 0.0000 | 0.0000 |
| Spleen | Pet food | 0.0011 | 0.0017 | 0.0001 |
| Stillborn | PAP C3 | 0.0089 | 0.0017 | 0.0000 |
| Tallow | Fat and greaves C3 | 0.0018 | 0.0017 | 0.0002 |
| Tongue | Human food | 0.0010 | 0.0017 | 0.0026 |
| Tonsil | C1-C2 for disposal | 0.0000 | 0.0000 | 0.0000 |
| Trachea | Pet food | 0.0016 | 0.0017 | 0.0001 |
| Udder | Pet food | 0.0003 | 0.0017 | 0.0001 |
| Upper throat | Pet food | 0.0011 | 0.0017 | 0.0001 |
| Water in the rumen | Spreading/Compost | 0.0000 | 0.0000 | 0.0000 |

Table 95: Allocation factors for Charolaise x Rustique Cull Cows reared in Stall

| COPRODUCT | Destination | Charolaise x Rustique/Cull cow/stall | | |
| --- | --- | --- | --- | --- |
|  |  | **Biophysical Allocation Factor** | **Mass Allocation Factor** | **Economic Allocation Factor** |
| Abomasum | Human food | 0.0080 | 0.0015 | 0.0012 |
| Abomasum fat | Fat and greaves C3 | 0.0033 | 0.0015 | 0.0001 |
| Aponeurosis | Human food | 0.0011 | 0.0015 | 0.0016 |
| Bile | PAP C3 | 0.0004 | 0.0015 | 0.0001 |
| Blood | PAP C3 | 0.0009 | 0.0015 | 0.0004 |
| Blood | Pet food | 0.0009 | 0.0015 | 0.0001 |
| Bones | Gelatin C3 | 0.0010 | 0.0015 | 0.0000 |
| Bones of head, brain, eyes and teeth | C1-C2 for disposal | 0.0000 | 0.0000 | 0.0000 |
| Cheek | Human food | 0.0010 | 0.0015 | 0.0035 |
| Cheek | Human food | 0.0010 | 0.0015 | 0.0035 |
| Cheek trimmings | Pet food | 0.0010 | 0.0015 | 0.0001 |
| Chops | Pet food | 0.0014 | 0.0015 | 0.0001 |
| Contents of intestines | Spreading/Compost | 0.0000 | 0.0000 | 0.0000 |
| Contents of the rumen | Spreading/Compost | 0.0000 | 0.0000 | 0.0000 |
| Ears | PAP C3 | 0.0013 | 0.0015 | 0.0001 |
| Esophagus | Pet food | 0.0010 | 0.0015 | 0.0001 |
| Fat | Fat and greaves C3 | 0.0017 | 0.0015 | 0.0001 |
| Fat around heart | Fat and greaves C3 | 0.0017 | 0.0015 | 0.0001 |
| Fat in the kidney | Fat and greaves C3 | 0.0017 | 0.0015 | 0.0001 |
| Feet (without hooves) | Gelatin C3 | 0.0010 | 0.0015 | 0.0000 |
| Floatation fat | Spreading/Compost | 0.0000 | 0.0000 | 0.0000 |
| Forehead | C1-C2 for disposal | 0.0000 | 0.0000 | 0.0000 |
| Forelock | PAP C3 | 0.0037 | 0.0015 | 0.0001 |
| Gallbladder | Pet food | 0.0010 | 0.0015 | 0.0001 |
| Head trimmings | Pet food | 0.0010 | 0.0015 | 0.0001 |
| Heart | Human food | 0.0010 | 0.0015 | 0.0003 |
| Heart trimmings | Pet food | 0.0010 | 0.0015 | 0.0001 |
| Hide | Skin tannery C3 | 0.0014 | 0.0015 | 0.0026 |
| Hooves | PAP C3 | 0.0037 | 0.0015 | 0.0001 |
| Horns | PAP C3 | 0.0037 | 0.0015 | 0.0001 |
| Kidney | Human food | 0.0010 | 0.0015 | 0.0006 |
| Large intestine | C1-C2 for disposal | 0.0000 | 0.0000 | 0.0000 |
| Liver | Human food | 0.0042 | 0.0015 | 0.0008 |
| Liver trimmings | Pet food | 0.0044 | 0.0015 | 0.0001 |
| Lower jaw | PAP C3 | 0.0010 | 0.0015 | 0.0001 |
| Lungs | Pet food | 0.0012 | 0.0015 | 0.0001 |
| Mask | Skin tannery C3 | 0.0014 | 0.0015 | 0.0026 |
| Mesenteric fat | C1-C2 for disposal | 0.0000 | 0.0000 | 0.0000 |
| Muscle | Human food | 0.0010 | 0.0015 | 0.0026 |
| Muzzle | Human food | 0.0014 | 0.0015 | 0.0016 |
| Omasum | Human food | 0.0080 | 0.0015 | 0.0012 |
| Omasum fat | Fat and greaves C3 | 0.0033 | 0.0015 | 0.0001 |
| Rumen and forestomach | Human food | 0.0080 | 0.0015 | 0.0012 |
| Rumen fat | Fat and greaves C3 | 0.0033 | 0.0015 | 0.0001 |
| Sanitary seizures | C1-C2 for disposal | 0.0000 | 0.0000 | 0.0000 |
| Screening and sifting wastes | C1-C2 for disposal | 0.0000 | 0.0000 | 0.0000 |
| Small intestine | PAP C3 | 0.0080 | 0.0015 | 0.0001 |
| Spinal cord | C1-C2 for disposal | 0.0000 | 0.0000 | 0.0000 |
| Spinal cord waste | C1-C2 for disposal | 0.0000 | 0.0000 | 0.0000 |
| Spine | C1-C2 for disposal | 0.0000 | 0.0000 | 0.0000 |
| Spleen | Pet food | 0.0011 | 0.0015 | 0.0001 |
| Stillborn | PAP C3 | 0.0084 | 0.0015 | 0.0000 |
| Tallow | Fat and greaves C3 | 0.0017 | 0.0015 | 0.0001 |
| Tongue | Human food | 0.0009 | 0.0015 | 0.0025 |
| Tonsil | C1-C2 for disposal | 0.0000 | 0.0000 | 0.0000 |
| Trachea | Pet food | 0.0014 | 0.0015 | 0.0001 |
| Udder | Pet food | 0.0002 | 0.0015 | 0.0001 |
| Upper throat | Pet food | 0.0010 | 0.0015 | 0.0001 |
| Water in the rumen | Spreading/Compost | 0.0000 | 0.0000 | 0.0000 |

Table 96: Allocation factors for Charolaise x Rustique Beef reared in Stall

| COPRODUCT | Destination | Charolaise x Rustique/beef/stall | | |
| --- | --- | --- | --- | --- |
|  |  | **Biophysical Allocation Factor** | **Mass Allocation Factor** | **Economic Allocation Factor** |
| Abomasum | Human food | 0.0078 | 0.0015 | 0.0011 |
| Abomasum fat | Fat and greaves C3 | 0.0032 | 0.0015 | 0.0001 |
| Aponeurosis | Human food | 0.0011 | 0.0015 | 0.0015 |
| Bile | PAP C3 | 0.0004 | 0.0015 | 0.0001 |
| Blood | PAP C3 | 0.0008 | 0.0015 | 0.0003 |
| Blood | Pet food | 0.0008 | 0.0015 | 0.0001 |
| Bones | Gelatin C3 | 0.0009 | 0.0015 | 0.0000 |
| Bones of head, brain, eyes and teeth | C1-C2 for disposal | 0.0000 | 0.0000 | 0.0000 |
| Cheek | Human food | 0.0010 | 0.0015 | 0.0032 |
| Cheek | Human food | 0.0010 | 0.0015 | 0.0032 |
| Cheek trimmings | Pet food | 0.0010 | 0.0015 | 0.0001 |
| Chops | Pet food | 0.0014 | 0.0015 | 0.0001 |
| Contents of intestines | Spreading/Compost | 0.0000 | 0.0000 | 0.0000 |
| Contents of the rumen | Spreading/Compost | 0.0000 | 0.0000 | 0.0000 |
| Ears | PAP C3 | 0.0012 | 0.0015 | 0.0001 |
| Esophagus | Pet food | 0.0010 | 0.0015 | 0.0001 |
| Fat | Fat and greaves C3 | 0.0016 | 0.0015 | 0.0001 |
| Fat around heart | Fat and greaves C3 | 0.0016 | 0.0015 | 0.0001 |
| Fat in the kidney | Fat and greaves C3 | 0.0016 | 0.0015 | 0.0001 |
| Feet (without hooves) | Gelatin C3 | 0.0010 | 0.0015 | 0.0000 |
| Floatation fat | Spreading/Compost | 0.0000 | 0.0000 | 0.0000 |
| Forehead | C1-C2 for disposal | 0.0000 | 0.0000 | 0.0000 |
| Forelock | PAP C3 | 0.0035 | 0.0015 | 0.0001 |
| Gallbladder | Pet food | 0.0010 | 0.0015 | 0.0001 |
| Head trimmings | Pet food | 0.0010 | 0.0015 | 0.0001 |
| Heart | Human food | 0.0009 | 0.0015 | 0.0003 |
| Heart trimmings | Pet food | 0.0010 | 0.0015 | 0.0001 |
| Hide | Skin tannery C3 | 0.0014 | 0.0015 | 0.0024 |
| Hooves | PAP C3 | 0.0035 | 0.0015 | 0.0001 |
| Horns | PAP C3 | 0.0035 | 0.0015 | 0.0001 |
| Kidney | Human food | 0.0010 | 0.0015 | 0.0006 |
| Large intestine | C1-C2 for disposal | 0.0000 | 0.0000 | 0.0000 |
| Liver | Human food | 0.0041 | 0.0015 | 0.0007 |
| Liver trimmings | Pet food | 0.0043 | 0.0015 | 0.0001 |
| Lower jaw | PAP C3 | 0.0009 | 0.0015 | 0.0001 |
| Lungs | Pet food | 0.0011 | 0.0015 | 0.0001 |
| Mask | Skin tannery C3 | 0.0014 | 0.0015 | 0.0024 |
| Mesenteric fat | C1-C2 for disposal | 0.0000 | 0.0000 | 0.0000 |
| Muscle | Human food | 0.0010 | 0.0015 | 0.0024 |
| Muzzle | Human food | 0.0014 | 0.0015 | 0.0015 |
| Omasum | Human food | 0.0078 | 0.0015 | 0.0011 |
| Omasum fat | Fat and greaves C3 | 0.0032 | 0.0015 | 0.0001 |
| Rumen and forestomach | Human food | 0.0078 | 0.0015 | 0.0011 |
| Rumen fat | Fat and greaves C3 | 0.0032 | 0.0015 | 0.0001 |
| Sanitary seizures | C1-C2 for disposal | 0.0000 | 0.0000 | 0.0000 |
| Screening and sifting wastes | C1-C2 for disposal | 0.0000 | 0.0000 | 0.0000 |
| Small intestine | PAP C3 | 0.0078 | 0.0015 | 0.0001 |
| Spinal cord | C1-C2 for disposal | 0.0000 | 0.0000 | 0.0000 |
| Spinal cord waste | C1-C2 for disposal | 0.0000 | 0.0000 | 0.0000 |
| Spine | C1-C2 for disposal | 0.0000 | 0.0000 | 0.0000 |
| Spleen | Pet food | 0.0010 | 0.0015 | 0.0001 |
| Stillborn | PAP C3 | 0.0082 | 0.0015 | 0.0000 |
| Tallow | Fat and greaves C3 | 0.0016 | 0.0015 | 0.0001 |
| Tongue | Human food | 0.0009 | 0.0015 | 0.0023 |
| Tonsil | C1-C2 for disposal | 0.0000 | 0.0000 | 0.0000 |
| Trachea | Pet food | 0.0014 | 0.0015 | 0.0001 |
| Udder | Pet food | 0.0002 | 0.0015 | 0.0001 |
| Upper throat | Pet food | 0.0010 | 0.0015 | 0.0001 |
| Water in the rumen | Spreading/Compost | 0.0000 | 0.0000 | 0.0000 |

Table 97: Allocation factors for Montbéliarde Young Bulls reared in Grazing Large Area

| COPRODUCT | Destination | Montbéliarde/young bull/grazing large area | | |
| --- | --- | --- | --- | --- |
|  |  | **Biophysical Allocation Factor** | **Mass Allocation Factor** | **Economic Allocation Factor** |
| Abomasum | Human food | 0.0090 | 0.0016 | 0.0012 |
| Abomasum fat | Fat and greaves C3 | 0.0034 | 0.0016 | 0.0001 |
| Aponeurosis | Human food | 0.0012 | 0.0016 | 0.0016 |
| Bile | PAP C3 | 0.0004 | 0.0016 | 0.0001 |
| Blood | PAP C3 | 0.0009 | 0.0016 | 0.0003 |
| Blood | Pet food | 0.0009 | 0.0016 | 0.0001 |
| Bones | Gelatin C3 | 0.0010 | 0.0016 | 0.0000 |
| Bones of head, brain, eyes and teeth | C1-C2 for disposal | 0.0000 | 0.0000 | 0.0000 |
| Cheek | Human food | 0.0011 | 0.0016 | 0.0034 |
| Cheek | Human food | 0.0011 | 0.0016 | 0.0034 |
| Cheek trimmings | Pet food | 0.0011 | 0.0016 | 0.0001 |
| Chops | Pet food | 0.0015 | 0.0016 | 0.0001 |
| Contents of intestines | Spreading/Compost | 0.0000 | 0.0000 | 0.0000 |
| Contents of the rumen | Spreading/Compost | 0.0000 | 0.0000 | 0.0000 |
| Ears | PAP C3 | 0.0013 | 0.0016 | 0.0001 |
| Esophagus | Pet food | 0.0010 | 0.0016 | 0.0001 |
| Fat | Fat and greaves C3 | 0.0014 | 0.0016 | 0.0001 |
| Fat around heart | Fat and greaves C3 | 0.0014 | 0.0016 | 0.0001 |
| Fat in the kidney | Fat and greaves C3 | 0.0014 | 0.0016 | 0.0001 |
| Feet (without hooves) | Gelatin C3 | 0.0011 | 0.0016 | 0.0000 |
| Floatation fat | Spreading/Compost | 0.0000 | 0.0000 | 0.0000 |
| Forehead | C1-C2 for disposal | 0.0000 | 0.0000 | 0.0000 |
| Forelock | PAP C3 | 0.0039 | 0.0016 | 0.0001 |
| Gallbladder | Pet food | 0.0011 | 0.0016 | 0.0001 |
| Head trimmings | Pet food | 0.0011 | 0.0016 | 0.0001 |
| Heart | Human food | 0.0010 | 0.0016 | 0.0003 |
| Heart trimmings | Pet food | 0.0011 | 0.0016 | 0.0001 |
| Hide | Skin tannery C3 | 0.0015 | 0.0016 | 0.0026 |
| Hooves | PAP C3 | 0.0039 | 0.0016 | 0.0001 |
| Horns | PAP C3 | 0.0039 | 0.0016 | 0.0001 |
| Kidney | Human food | 0.0011 | 0.0016 | 0.0006 |
| Large intestine | C1-C2 for disposal | 0.0000 | 0.0000 | 0.0000 |
| Liver | Human food | 0.0047 | 0.0016 | 0.0008 |
| Liver trimmings | Pet food | 0.0049 | 0.0016 | 0.0001 |
| Lower jaw | PAP C3 | 0.0010 | 0.0016 | 0.0001 |
| Lungs | Pet food | 0.0012 | 0.0016 | 0.0001 |
| Mask | Skin tannery C3 | 0.0015 | 0.0016 | 0.0026 |
| Mesenteric fat | C1-C2 for disposal | 0.0000 | 0.0000 | 0.0000 |
| Muscle | Human food | 0.0011 | 0.0016 | 0.0026 |
| Muzzle | Human food | 0.0015 | 0.0016 | 0.0016 |
| Omasum | Human food | 0.0090 | 0.0016 | 0.0012 |
| Omasum fat | Fat and greaves C3 | 0.0034 | 0.0016 | 0.0001 |
| Rumen and forestomach | Human food | 0.0090 | 0.0016 | 0.0012 |
| Rumen fat | Fat and greaves C3 | 0.0034 | 0.0016 | 0.0001 |
| Sanitary seizures | C1-C2 for disposal | 0.0000 | 0.0000 | 0.0000 |
| Screening and sifting wastes | C1-C2 for disposal | 0.0000 | 0.0000 | 0.0000 |
| Small intestine | PAP C3 | 0.0090 | 0.0016 | 0.0001 |
| Spinal cord | C1-C2 for disposal | 0.0000 | 0.0000 | 0.0000 |
| Spinal cord waste | C1-C2 for disposal | 0.0000 | 0.0000 | 0.0000 |
| Spine | C1-C2 for disposal | 0.0000 | 0.0000 | 0.0000 |
| Spleen | Pet food | 0.0011 | 0.0016 | 0.0001 |
| Stillborn | PAP C3 | 0.0094 | 0.0016 | 0.0000 |
| Tallow | Fat and greaves C3 | 0.0014 | 0.0016 | 0.0001 |
| Tongue | Human food | 0.0009 | 0.0016 | 0.0025 |
| Tonsil | C1-C2 for disposal | 0.0000 | 0.0000 | 0.0000 |
| Trachea | Pet food | 0.0015 | 0.0016 | 0.0001 |
| Udder | Pet food | 0.0002 | 0.0016 | 0.0001 |
| Upper throat | Pet food | 0.0011 | 0.0016 | 0.0001 |
| Water in the rumen | Spreading/Compost | 0.0000 | 0.0000 | 0.0000 |

Table 98: Allocation factors for Montbéliarde Heifers reared in Grazing Large Area

| COPRODUCT | Destination | Montbéliarde/heifer/grazing large area | | |
| --- | --- | --- | --- | --- |
|  |  | **Biophysical Allocation Factor** | **Mass Allocation Factor** | **Economic Allocation Factor** |
| Abomasum | Human food | 0.0110 | 0.0020 | 0.0016 |
| Abomasum fat | Fat and greaves C3 | 0.0042 | 0.0020 | 0.0002 |
| Aponeurosis | Human food | 0.0016 | 0.0020 | 0.0021 |
| Bile | PAP C3 | 0.0006 | 0.0020 | 0.0002 |
| Blood | PAP C3 | 0.0012 | 0.0020 | 0.0005 |
| Blood | Pet food | 0.0012 | 0.0020 | 0.0002 |
| Bones | Gelatin C3 | 0.0013 | 0.0020 | 0.0000 |
| Bones of head, brain, eyes and teeth | C1-C2 for disposal | 0.0000 | 0.0000 | 0.0000 |
| Cheek | Human food | 0.0014 | 0.0020 | 0.0046 |
| Cheek | Human food | 0.0014 | 0.0020 | 0.0046 |
| Cheek trimmings | Pet food | 0.0014 | 0.0020 | 0.0002 |
| Chops | Pet food | 0.0020 | 0.0020 | 0.0002 |
| Contents of intestines | Spreading/Compost | 0.0000 | 0.0000 | 0.0000 |
| Contents of the rumen | Spreading/Compost | 0.0000 | 0.0000 | 0.0000 |
| Ears | PAP C3 | 0.0017 | 0.0020 | 0.0002 |
| Esophagus | Pet food | 0.0014 | 0.0020 | 0.0002 |
| Fat | Fat and greaves C3 | 0.0019 | 0.0020 | 0.0002 |
| Fat around heart | Fat and greaves C3 | 0.0019 | 0.0020 | 0.0002 |
| Fat in the kidney | Fat and greaves C3 | 0.0019 | 0.0020 | 0.0002 |
| Feet (without hooves) | Gelatin C3 | 0.0014 | 0.0020 | 0.0000 |
| Floatation fat | Spreading/Compost | 0.0000 | 0.0000 | 0.0000 |
| Forehead | C1-C2 for disposal | 0.0000 | 0.0000 | 0.0000 |
| Forelock | PAP C3 | 0.0051 | 0.0020 | 0.0002 |
| Gallbladder | Pet food | 0.0014 | 0.0020 | 0.0002 |
| Head trimmings | Pet food | 0.0014 | 0.0020 | 0.0002 |
| Heart | Human food | 0.0014 | 0.0020 | 0.0004 |
| Heart trimmings | Pet food | 0.0014 | 0.0020 | 0.0002 |
| Hide | Skin tannery C3 | 0.0020 | 0.0020 | 0.0035 |
| Hooves | PAP C3 | 0.0051 | 0.0020 | 0.0002 |
| Horns | PAP C3 | 0.0051 | 0.0020 | 0.0002 |
| Kidney | Human food | 0.0014 | 0.0020 | 0.0008 |
| Large intestine | C1-C2 for disposal | 0.0000 | 0.0000 | 0.0000 |
| Liver | Human food | 0.0057 | 0.0020 | 0.0010 |
| Liver trimmings | Pet food | 0.0060 | 0.0020 | 0.0002 |
| Lower jaw | PAP C3 | 0.0013 | 0.0020 | 0.0002 |
| Lungs | Pet food | 0.0016 | 0.0020 | 0.0002 |
| Mask | Skin tannery C3 | 0.0020 | 0.0020 | 0.0035 |
| Mesenteric fat | C1-C2 for disposal | 0.0000 | 0.0000 | 0.0000 |
| Muscle | Human food | 0.0014 | 0.0020 | 0.0035 |
| Muzzle | Human food | 0.0020 | 0.0020 | 0.0021 |
| Omasum | Human food | 0.0110 | 0.0020 | 0.0016 |
| Omasum fat | Fat and greaves C3 | 0.0042 | 0.0020 | 0.0002 |
| Rumen and forestomach | Human food | 0.0110 | 0.0020 | 0.0016 |
| Rumen fat | Fat and greaves C3 | 0.0042 | 0.0020 | 0.0002 |
| Sanitary seizures | C1-C2 for disposal | 0.0000 | 0.0000 | 0.0000 |
| Screening and sifting wastes | C1-C2 for disposal | 0.0000 | 0.0000 | 0.0000 |
| Small intestine | PAP C3 | 0.0110 | 0.0020 | 0.0001 |
| Spinal cord | C1-C2 for disposal | 0.0000 | 0.0000 | 0.0000 |
| Spinal cord waste | C1-C2 for disposal | 0.0000 | 0.0000 | 0.0000 |
| Spine | C1-C2 for disposal | 0.0000 | 0.0000 | 0.0000 |
| Spleen | Pet food | 0.0014 | 0.0020 | 0.0002 |
| Stillborn | PAP C3 | 0.0115 | 0.0020 | 0.0000 |
| Tallow | Fat and greaves C3 | 0.0019 | 0.0020 | 0.0002 |
| Tongue | Human food | 0.0012 | 0.0020 | 0.0033 |
| Tonsil | C1-C2 for disposal | 0.0000 | 0.0000 | 0.0000 |
| Trachea | Pet food | 0.0020 | 0.0020 | 0.0002 |
| Udder | Pet food | 0.0003 | 0.0020 | 0.0002 |
| Upper throat | Pet food | 0.0014 | 0.0020 | 0.0002 |
| Water in the rumen | Spreading/Compost | 0.0000 | 0.0000 | 0.0000 |

Table 99: Allocation factors for Montbéliarde Cull Cows reared in Grazing Large Area

| COPRODUCT | Destination | Montbéliarde/Cull cow/grazing large area | | |
| --- | --- | --- | --- | --- |
|  |  | **Biophysical Allocation Factor** | **Mass Allocation Factor** | **Economic Allocation Factor** |
| Abomasum | Human food | 0.0096 | 0.0017 | 0.0014 |
| Abomasum fat | Fat and greaves C3 | 0.0036 | 0.0017 | 0.0002 |
| Aponeurosis | Human food | 0.0013 | 0.0017 | 0.0018 |
| Bile | PAP C3 | 0.0005 | 0.0017 | 0.0002 |
| Blood | PAP C3 | 0.0010 | 0.0017 | 0.0004 |
| Blood | Pet food | 0.0010 | 0.0017 | 0.0001 |
| Bones | Gelatin C3 | 0.0011 | 0.0017 | 0.0000 |
| Bones of head, brain, eyes and teeth | C1-C2 for disposal | 0.0000 | 0.0000 | 0.0000 |
| Cheek | Human food | 0.0012 | 0.0017 | 0.0040 |
| Cheek | Human food | 0.0012 | 0.0017 | 0.0040 |
| Cheek trimmings | Pet food | 0.0012 | 0.0017 | 0.0001 |
| Chops | Pet food | 0.0017 | 0.0017 | 0.0001 |
| Contents of intestines | Spreading/Compost | 0.0000 | 0.0000 | 0.0000 |
| Contents of the rumen | Spreading/Compost | 0.0000 | 0.0000 | 0.0000 |
| Ears | PAP C3 | 0.0015 | 0.0017 | 0.0002 |
| Esophagus | Pet food | 0.0011 | 0.0017 | 0.0001 |
| Fat | Fat and greaves C3 | 0.0016 | 0.0017 | 0.0002 |
| Fat around heart | Fat and greaves C3 | 0.0016 | 0.0017 | 0.0002 |
| Fat in the kidney | Fat and greaves C3 | 0.0016 | 0.0017 | 0.0002 |
| Feet (without hooves) | Gelatin C3 | 0.0012 | 0.0017 | 0.0000 |
| Floatation fat | Spreading/Compost | 0.0000 | 0.0000 | 0.0000 |
| Forehead | C1-C2 for disposal | 0.0000 | 0.0000 | 0.0000 |
| Forelock | PAP C3 | 0.0043 | 0.0017 | 0.0002 |
| Gallbladder | Pet food | 0.0012 | 0.0017 | 0.0001 |
| Head trimmings | Pet food | 0.0012 | 0.0017 | 0.0001 |
| Heart | Human food | 0.0011 | 0.0017 | 0.0004 |
| Heart trimmings | Pet food | 0.0012 | 0.0017 | 0.0001 |
| Hide | Skin tannery C3 | 0.0017 | 0.0017 | 0.0030 |
| Hooves | PAP C3 | 0.0043 | 0.0017 | 0.0002 |
| Horns | PAP C3 | 0.0043 | 0.0017 | 0.0002 |
| Kidney | Human food | 0.0012 | 0.0017 | 0.0007 |
| Large intestine | C1-C2 for disposal | 0.0000 | 0.0000 | 0.0000 |
| Liver | Human food | 0.0050 | 0.0017 | 0.0009 |
| Liver trimmings | Pet food | 0.0052 | 0.0017 | 0.0001 |
| Lower jaw | PAP C3 | 0.0011 | 0.0017 | 0.0002 |
| Lungs | Pet food | 0.0014 | 0.0017 | 0.0001 |
| Mask | Skin tannery C3 | 0.0017 | 0.0017 | 0.0030 |
| Mesenteric fat | C1-C2 for disposal | 0.0000 | 0.0000 | 0.0000 |
| Muscle | Human food | 0.0012 | 0.0017 | 0.0030 |
| Muzzle | Human food | 0.0017 | 0.0017 | 0.0018 |
| Omasum | Human food | 0.0096 | 0.0017 | 0.0014 |
| Omasum fat | Fat and greaves C3 | 0.0036 | 0.0017 | 0.0002 |
| Rumen and forestomach | Human food | 0.0096 | 0.0017 | 0.0014 |
| Rumen fat | Fat and greaves C3 | 0.0036 | 0.0017 | 0.0002 |
| Sanitary seizures | C1-C2 for disposal | 0.0000 | 0.0000 | 0.0000 |
| Screening and sifting wastes | C1-C2 for disposal | 0.0000 | 0.0000 | 0.0000 |
| Small intestine | PAP C3 | 0.0096 | 0.0017 | 0.0001 |
| Spinal cord | C1-C2 for disposal | 0.0000 | 0.0000 | 0.0000 |
| Spinal cord waste | C1-C2 for disposal | 0.0000 | 0.0000 | 0.0000 |
| Spine | C1-C2 for disposal | 0.0000 | 0.0000 | 0.0000 |
| Spleen | Pet food | 0.0012 | 0.0017 | 0.0001 |
| Stillborn | PAP C3 | 0.0100 | 0.0017 | 0.0000 |
| Tallow | Fat and greaves C3 | 0.0016 | 0.0017 | 0.0002 |
| Tongue | Human food | 0.0010 | 0.0017 | 0.0029 |
| Tonsil | C1-C2 for disposal | 0.0000 | 0.0000 | 0.0000 |
| Trachea | Pet food | 0.0016 | 0.0017 | 0.0001 |
| Udder | Pet food | 0.0002 | 0.0017 | 0.0001 |
| Upper throat | Pet food | 0.0012 | 0.0017 | 0.0001 |
| Water in the rumen | Spreading/Compost | 0.0000 | 0.0000 | 0.0000 |

Table 100: Allocation factors for Montbéliarde Beef reared in Grazing Large Area

| COPRODUCT | Destination | Montbéliarde/beef/grazing large area | | |
| --- | --- | --- | --- | --- |
|  |  | **Biophysical Allocation Factor** | **Mass Allocation Factor** | **Economic Allocation Factor** |
| Abomasum | Human food | 0.0091 | 0.0016 | 0.0012 |
| Abomasum fat | Fat and greaves C3 | 0.0034 | 0.0016 | 0.0001 |
| Aponeurosis | Human food | 0.0012 | 0.0016 | 0.0016 |
| Bile | PAP C3 | 0.0004 | 0.0016 | 0.0001 |
| Blood | PAP C3 | 0.0009 | 0.0016 | 0.0004 |
| Blood | Pet food | 0.0009 | 0.0016 | 0.0001 |
| Bones | Gelatin C3 | 0.0010 | 0.0016 | 0.0000 |
| Bones of head, brain, eyes and teeth | C1-C2 for disposal | 0.0000 | 0.0000 | 0.0000 |
| Cheek | Human food | 0.0011 | 0.0016 | 0.0036 |
| Cheek | Human food | 0.0011 | 0.0016 | 0.0036 |
| Cheek trimmings | Pet food | 0.0011 | 0.0016 | 0.0001 |
| Chops | Pet food | 0.0015 | 0.0016 | 0.0001 |
| Contents of intestines | Spreading/Compost | 0.0000 | 0.0000 | 0.0000 |
| Contents of the rumen | Spreading/Compost | 0.0000 | 0.0000 | 0.0000 |
| Ears | PAP C3 | 0.0014 | 0.0016 | 0.0001 |
| Esophagus | Pet food | 0.0011 | 0.0016 | 0.0001 |
| Fat | Fat and greaves C3 | 0.0015 | 0.0016 | 0.0001 |
| Fat around heart | Fat and greaves C3 | 0.0015 | 0.0016 | 0.0001 |
| Fat in the kidney | Fat and greaves C3 | 0.0015 | 0.0016 | 0.0001 |
| Feet (without hooves) | Gelatin C3 | 0.0011 | 0.0016 | 0.0000 |
| Floatation fat | Spreading/Compost | 0.0000 | 0.0000 | 0.0000 |
| Forehead | C1-C2 for disposal | 0.0000 | 0.0000 | 0.0000 |
| Forelock | PAP C3 | 0.0040 | 0.0016 | 0.0001 |
| Gallbladder | Pet food | 0.0011 | 0.0016 | 0.0001 |
| Head trimmings | Pet food | 0.0011 | 0.0016 | 0.0001 |
| Heart | Human food | 0.0010 | 0.0016 | 0.0003 |
| Heart trimmings | Pet food | 0.0011 | 0.0016 | 0.0001 |
| Hide | Skin tannery C3 | 0.0015 | 0.0016 | 0.0027 |
| Hooves | PAP C3 | 0.0040 | 0.0016 | 0.0001 |
| Horns | PAP C3 | 0.0040 | 0.0016 | 0.0001 |
| Kidney | Human food | 0.0011 | 0.0016 | 0.0007 |
| Large intestine | C1-C2 for disposal | 0.0000 | 0.0000 | 0.0000 |
| Liver | Human food | 0.0047 | 0.0016 | 0.0008 |
| Liver trimmings | Pet food | 0.0049 | 0.0016 | 0.0001 |
| Lower jaw | PAP C3 | 0.0010 | 0.0016 | 0.0001 |
| Lungs | Pet food | 0.0013 | 0.0016 | 0.0001 |
| Mask | Skin tannery C3 | 0.0015 | 0.0016 | 0.0027 |
| Mesenteric fat | C1-C2 for disposal | 0.0000 | 0.0000 | 0.0000 |
| Muscle | Human food | 0.0011 | 0.0016 | 0.0027 |
| Muzzle | Human food | 0.0015 | 0.0016 | 0.0016 |
| Omasum | Human food | 0.0091 | 0.0016 | 0.0012 |
| Omasum fat | Fat and greaves C3 | 0.0034 | 0.0016 | 0.0001 |
| Rumen and forestomach | Human food | 0.0091 | 0.0016 | 0.0012 |
| Rumen fat | Fat and greaves C3 | 0.0034 | 0.0016 | 0.0001 |
| Sanitary seizures | C1-C2 for disposal | 0.0000 | 0.0000 | 0.0000 |
| Screening and sifting wastes | C1-C2 for disposal | 0.0000 | 0.0000 | 0.0000 |
| Small intestine | PAP C3 | 0.0091 | 0.0016 | 0.0001 |
| Spinal cord | C1-C2 for disposal | 0.0000 | 0.0000 | 0.0000 |
| Spinal cord waste | C1-C2 for disposal | 0.0000 | 0.0000 | 0.0000 |
| Spine | C1-C2 for disposal | 0.0000 | 0.0000 | 0.0000 |
| Spleen | Pet food | 0.0011 | 0.0016 | 0.0001 |
| Stillborn | PAP C3 | 0.0095 | 0.0016 | 0.0000 |
| Tallow | Fat and greaves C3 | 0.0015 | 0.0016 | 0.0001 |
| Tongue | Human food | 0.0010 | 0.0016 | 0.0026 |
| Tonsil | C1-C2 for disposal | 0.0000 | 0.0000 | 0.0000 |
| Trachea | Pet food | 0.0015 | 0.0016 | 0.0001 |
| Udder | Pet food | 0.0002 | 0.0016 | 0.0001 |
| Upper throat | Pet food | 0.0011 | 0.0016 | 0.0001 |
| Water in the rumen | Spreading/Compost | 0.0000 | 0.0000 | 0.0000 |

Table 101: Allocation factors for Montbéliarde Young Bulls reared in Pasture

| COPRODUCT | Destination | Montbéliarde/young bull/pasture | | |
| --- | --- | --- | --- | --- |
|  |  | **Biophysical Allocation Factor** | **Mass Allocation Factor** | **Economic Allocation Factor** |
| Abomasum | Human food | 0.0086 | 0.0016 | 0.0012 |
| Abomasum fat | Fat and greaves C3 | 0.0034 | 0.0016 | 0.0001 |
| Aponeurosis | Human food | 0.0012 | 0.0016 | 0.0016 |
| Bile | PAP C3 | 0.0004 | 0.0016 | 0.0001 |
| Blood | PAP C3 | 0.0009 | 0.0016 | 0.0003 |
| Blood | Pet food | 0.0009 | 0.0016 | 0.0001 |
| Bones | Gelatin C3 | 0.0010 | 0.0016 | 0.0000 |
| Bones of head, brain, eyes and teeth | C1-C2 for disposal | 0.0000 | 0.0000 | 0.0000 |
| Cheek | Human food | 0.0011 | 0.0016 | 0.0034 |
| Cheek | Human food | 0.0011 | 0.0016 | 0.0034 |
| Cheek trimmings | Pet food | 0.0011 | 0.0016 | 0.0001 |
| Chops | Pet food | 0.0015 | 0.0016 | 0.0001 |
| Contents of intestines | Spreading/Compost | 0.0000 | 0.0000 | 0.0000 |
| Contents of the rumen | Spreading/Compost | 0.0000 | 0.0000 | 0.0000 |
| Ears | PAP C3 | 0.0013 | 0.0016 | 0.0001 |
| Esophagus | Pet food | 0.0010 | 0.0016 | 0.0001 |
| Fat | Fat and greaves C3 | 0.0016 | 0.0016 | 0.0001 |
| Fat around heart | Fat and greaves C3 | 0.0016 | 0.0016 | 0.0001 |
| Fat in the kidney | Fat and greaves C3 | 0.0016 | 0.0016 | 0.0001 |
| Feet (without hooves) | Gelatin C3 | 0.0011 | 0.0016 | 0.0000 |
| Floatation fat | Spreading/Compost | 0.0000 | 0.0000 | 0.0000 |
| Forehead | C1-C2 for disposal | 0.0000 | 0.0000 | 0.0000 |
| Forelock | PAP C3 | 0.0038 | 0.0016 | 0.0001 |
| Gallbladder | Pet food | 0.0011 | 0.0016 | 0.0001 |
| Head trimmings | Pet food | 0.0011 | 0.0016 | 0.0001 |
| Heart | Human food | 0.0010 | 0.0016 | 0.0003 |
| Heart trimmings | Pet food | 0.0011 | 0.0016 | 0.0001 |
| Hide | Skin tannery C3 | 0.0015 | 0.0016 | 0.0026 |
| Hooves | PAP C3 | 0.0038 | 0.0016 | 0.0001 |
| Horns | PAP C3 | 0.0038 | 0.0016 | 0.0001 |
| Kidney | Human food | 0.0011 | 0.0016 | 0.0006 |
| Large intestine | C1-C2 for disposal | 0.0000 | 0.0000 | 0.0000 |
| Liver | Human food | 0.0045 | 0.0016 | 0.0008 |
| Liver trimmings | Pet food | 0.0047 | 0.0016 | 0.0001 |
| Lower jaw | PAP C3 | 0.0010 | 0.0016 | 0.0001 |
| Lungs | Pet food | 0.0012 | 0.0016 | 0.0001 |
| Mask | Skin tannery C3 | 0.0015 | 0.0016 | 0.0026 |
| Mesenteric fat | C1-C2 for disposal | 0.0000 | 0.0000 | 0.0000 |
| Muscle | Human food | 0.0011 | 0.0016 | 0.0026 |
| Muzzle | Human food | 0.0015 | 0.0016 | 0.0016 |
| Omasum | Human food | 0.0086 | 0.0016 | 0.0012 |
| Omasum fat | Fat and greaves C3 | 0.0034 | 0.0016 | 0.0001 |
| Rumen and forestomach | Human food | 0.0086 | 0.0016 | 0.0012 |
| Rumen fat | Fat and greaves C3 | 0.0034 | 0.0016 | 0.0001 |
| Sanitary seizures | C1-C2 for disposal | 0.0000 | 0.0000 | 0.0000 |
| Screening and sifting wastes | C1-C2 for disposal | 0.0000 | 0.0000 | 0.0000 |
| Small intestine | PAP C3 | 0.0086 | 0.0016 | 0.0001 |
| Spinal cord | C1-C2 for disposal | 0.0000 | 0.0000 | 0.0000 |
| Spinal cord waste | C1-C2 for disposal | 0.0000 | 0.0000 | 0.0000 |
| Spine | C1-C2 for disposal | 0.0000 | 0.0000 | 0.0000 |
| Spleen | Pet food | 0.0011 | 0.0016 | 0.0001 |
| Stillborn | PAP C3 | 0.0090 | 0.0016 | 0.0000 |
| Tallow | Fat and greaves C3 | 0.0016 | 0.0016 | 0.0001 |
| Tongue | Human food | 0.0009 | 0.0016 | 0.0025 |
| Tonsil | C1-C2 for disposal | 0.0000 | 0.0000 | 0.0000 |
| Trachea | Pet food | 0.0015 | 0.0016 | 0.0001 |
| Udder | Pet food | 0.0002 | 0.0016 | 0.0001 |
| Upper throat | Pet food | 0.0011 | 0.0016 | 0.0001 |
| Water in the rumen | Spreading/Compost | 0.0000 | 0.0000 | 0.0000 |

Table 102: Allocation factors for Montbéliarde Heifers reared in Pasture

| COPRODUCT | Destination | Montbéliarde/heifer/pasture | | |
| --- | --- | --- | --- | --- |
|  |  | **Biophysical Allocation Factor** | **Mass Allocation Factor** | **Economic Allocation Factor** |
| Abomasum | Human food | 0.0105 | 0.0020 | 0.0016 |
| Abomasum fat | Fat and greaves C3 | 0.0042 | 0.0020 | 0.0002 |
| Aponeurosis | Human food | 0.0015 | 0.0020 | 0.0021 |
| Bile | PAP C3 | 0.0006 | 0.0020 | 0.0002 |
| Blood | PAP C3 | 0.0012 | 0.0020 | 0.0005 |
| Blood | Pet food | 0.0012 | 0.0020 | 0.0002 |
| Bones | Gelatin C3 | 0.0013 | 0.0020 | 0.0000 |
| Bones of head, brain, eyes and teeth | C1-C2 for disposal | 0.0000 | 0.0000 | 0.0000 |
| Cheek | Human food | 0.0014 | 0.0020 | 0.0046 |
| Cheek | Human food | 0.0014 | 0.0020 | 0.0046 |
| Cheek trimmings | Pet food | 0.0014 | 0.0020 | 0.0002 |
| Chops | Pet food | 0.0020 | 0.0020 | 0.0002 |
| Contents of intestines | Spreading/Compost | 0.0000 | 0.0000 | 0.0000 |
| Contents of the rumen | Spreading/Compost | 0.0000 | 0.0000 | 0.0000 |
| Ears | PAP C3 | 0.0018 | 0.0020 | 0.0002 |
| Esophagus | Pet food | 0.0014 | 0.0020 | 0.0002 |
| Fat | Fat and greaves C3 | 0.0020 | 0.0020 | 0.0002 |
| Fat around heart | Fat and greaves C3 | 0.0020 | 0.0020 | 0.0002 |
| Fat in the kidney | Fat and greaves C3 | 0.0020 | 0.0020 | 0.0002 |
| Feet (without hooves) | Gelatin C3 | 0.0014 | 0.0020 | 0.0000 |
| Floatation fat | Spreading/Compost | 0.0000 | 0.0000 | 0.0000 |
| Forehead | C1-C2 for disposal | 0.0000 | 0.0000 | 0.0000 |
| Forelock | PAP C3 | 0.0051 | 0.0020 | 0.0002 |
| Gallbladder | Pet food | 0.0014 | 0.0020 | 0.0002 |
| Head trimmings | Pet food | 0.0014 | 0.0020 | 0.0002 |
| Heart | Human food | 0.0013 | 0.0020 | 0.0004 |
| Heart trimmings | Pet food | 0.0014 | 0.0020 | 0.0002 |
| Hide | Skin tannery C3 | 0.0020 | 0.0020 | 0.0035 |
| Hooves | PAP C3 | 0.0051 | 0.0020 | 0.0002 |
| Horns | PAP C3 | 0.0051 | 0.0020 | 0.0002 |
| Kidney | Human food | 0.0014 | 0.0020 | 0.0008 |
| Large intestine | C1-C2 for disposal | 0.0000 | 0.0000 | 0.0000 |
| Liver | Human food | 0.0055 | 0.0020 | 0.0010 |
| Liver trimmings | Pet food | 0.0057 | 0.0020 | 0.0002 |
| Lower jaw | PAP C3 | 0.0013 | 0.0020 | 0.0002 |
| Lungs | Pet food | 0.0016 | 0.0020 | 0.0002 |
| Mask | Skin tannery C3 | 0.0020 | 0.0020 | 0.0035 |
| Mesenteric fat | C1-C2 for disposal | 0.0000 | 0.0000 | 0.0000 |
| Muscle | Human food | 0.0014 | 0.0020 | 0.0035 |
| Muzzle | Human food | 0.0020 | 0.0020 | 0.0021 |
| Omasum | Human food | 0.0105 | 0.0020 | 0.0016 |
| Omasum fat | Fat and greaves C3 | 0.0042 | 0.0020 | 0.0002 |
| Rumen and forestomach | Human food | 0.0105 | 0.0020 | 0.0016 |
| Rumen fat | Fat and greaves C3 | 0.0042 | 0.0020 | 0.0002 |
| Sanitary seizures | C1-C2 for disposal | 0.0000 | 0.0000 | 0.0000 |
| Screening and sifting wastes | C1-C2 for disposal | 0.0000 | 0.0000 | 0.0000 |
| Small intestine | PAP C3 | 0.0105 | 0.0020 | 0.0001 |
| Spinal cord | C1-C2 for disposal | 0.0000 | 0.0000 | 0.0000 |
| Spinal cord waste | C1-C2 for disposal | 0.0000 | 0.0000 | 0.0000 |
| Spine | C1-C2 for disposal | 0.0000 | 0.0000 | 0.0000 |
| Spleen | Pet food | 0.0014 | 0.0020 | 0.0002 |
| Stillborn | PAP C3 | 0.0109 | 0.0020 | 0.0000 |
| Tallow | Fat and greaves C3 | 0.0020 | 0.0020 | 0.0002 |
| Tongue | Human food | 0.0012 | 0.0020 | 0.0033 |
| Tonsil | C1-C2 for disposal | 0.0000 | 0.0000 | 0.0000 |
| Trachea | Pet food | 0.0020 | 0.0020 | 0.0002 |
| Udder | Pet food | 0.0003 | 0.0020 | 0.0002 |
| Upper throat | Pet food | 0.0014 | 0.0020 | 0.0002 |
| Water in the rumen | Spreading/Compost | 0.0000 | 0.0000 | 0.0000 |

Table 103: Allocation factors for Montbéliarde Cull Cows reared in Pasture

| COPRODUCT | Destination | Montbéliarde/Cull cow/pasture | | |
| --- | --- | --- | --- | --- |
|  |  | **Biophysical Allocation Factor** | **Mass Allocation Factor** | **Economic Allocation Factor** |
| Abomasum | Human food | 0.0091 | 0.0017 | 0.0014 |
| Abomasum fat | Fat and greaves C3 | 0.0036 | 0.0017 | 0.0002 |
| Aponeurosis | Human food | 0.0013 | 0.0017 | 0.0018 |
| Bile | PAP C3 | 0.0005 | 0.0017 | 0.0002 |
| Blood | PAP C3 | 0.0010 | 0.0017 | 0.0004 |
| Blood | Pet food | 0.0010 | 0.0017 | 0.0001 |
| Bones | Gelatin C3 | 0.0011 | 0.0017 | 0.0000 |
| Bones of head, brain, eyes and teeth | C1-C2 for disposal | 0.0000 | 0.0000 | 0.0000 |
| Cheek | Human food | 0.0012 | 0.0017 | 0.0040 |
| Cheek | Human food | 0.0012 | 0.0017 | 0.0040 |
| Cheek trimmings | Pet food | 0.0012 | 0.0017 | 0.0001 |
| Chops | Pet food | 0.0016 | 0.0017 | 0.0001 |
| Contents of intestines | Spreading/Compost | 0.0000 | 0.0000 | 0.0000 |
| Contents of the rumen | Spreading/Compost | 0.0000 | 0.0000 | 0.0000 |
| Ears | PAP C3 | 0.0015 | 0.0017 | 0.0002 |
| Esophagus | Pet food | 0.0011 | 0.0017 | 0.0001 |
| Fat | Fat and greaves C3 | 0.0017 | 0.0017 | 0.0002 |
| Fat around heart | Fat and greaves C3 | 0.0017 | 0.0017 | 0.0002 |
| Fat in the kidney | Fat and greaves C3 | 0.0017 | 0.0017 | 0.0002 |
| Feet (without hooves) | Gelatin C3 | 0.0012 | 0.0017 | 0.0000 |
| Floatation fat | Spreading/Compost | 0.0000 | 0.0000 | 0.0000 |
| Forehead | C1-C2 for disposal | 0.0000 | 0.0000 | 0.0000 |
| Forelock | PAP C3 | 0.0042 | 0.0017 | 0.0002 |
| Gallbladder | Pet food | 0.0012 | 0.0017 | 0.0001 |
| Head trimmings | Pet food | 0.0012 | 0.0017 | 0.0001 |
| Heart | Human food | 0.0011 | 0.0017 | 0.0004 |
| Heart trimmings | Pet food | 0.0012 | 0.0017 | 0.0001 |
| Hide | Skin tannery C3 | 0.0016 | 0.0017 | 0.0030 |
| Hooves | PAP C3 | 0.0042 | 0.0017 | 0.0002 |
| Horns | PAP C3 | 0.0042 | 0.0017 | 0.0002 |
| Kidney | Human food | 0.0012 | 0.0017 | 0.0007 |
| Large intestine | C1-C2 for disposal | 0.0000 | 0.0000 | 0.0000 |
| Liver | Human food | 0.0048 | 0.0017 | 0.0009 |
| Liver trimmings | Pet food | 0.0050 | 0.0017 | 0.0001 |
| Lower jaw | PAP C3 | 0.0011 | 0.0017 | 0.0002 |
| Lungs | Pet food | 0.0014 | 0.0017 | 0.0001 |
| Mask | Skin tannery C3 | 0.0016 | 0.0017 | 0.0030 |
| Mesenteric fat | C1-C2 for disposal | 0.0000 | 0.0000 | 0.0000 |
| Muscle | Human food | 0.0012 | 0.0017 | 0.0030 |
| Muzzle | Human food | 0.0016 | 0.0017 | 0.0018 |
| Omasum | Human food | 0.0091 | 0.0017 | 0.0014 |
| Omasum fat | Fat and greaves C3 | 0.0036 | 0.0017 | 0.0002 |
| Rumen and forestomach | Human food | 0.0091 | 0.0017 | 0.0014 |
| Rumen fat | Fat and greaves C3 | 0.0036 | 0.0017 | 0.0002 |
| Sanitary seizures | C1-C2 for disposal | 0.0000 | 0.0000 | 0.0000 |
| Screening and sifting wastes | C1-C2 for disposal | 0.0000 | 0.0000 | 0.0000 |
| Small intestine | PAP C3 | 0.0091 | 0.0017 | 0.0001 |
| Spinal cord | C1-C2 for disposal | 0.0000 | 0.0000 | 0.0000 |
| Spinal cord waste | C1-C2 for disposal | 0.0000 | 0.0000 | 0.0000 |
| Spine | C1-C2 for disposal | 0.0000 | 0.0000 | 0.0000 |
| Spleen | Pet food | 0.0012 | 0.0017 | 0.0001 |
| Stillborn | PAP C3 | 0.0095 | 0.0017 | 0.0000 |
| Tallow | Fat and greaves C3 | 0.0017 | 0.0017 | 0.0002 |
| Tongue | Human food | 0.0010 | 0.0017 | 0.0029 |
| Tonsil | C1-C2 for disposal | 0.0000 | 0.0000 | 0.0000 |
| Trachea | Pet food | 0.0016 | 0.0017 | 0.0001 |
| Udder | Pet food | 0.0003 | 0.0017 | 0.0001 |
| Upper throat | Pet food | 0.0012 | 0.0017 | 0.0001 |
| Water in the rumen | Spreading/Compost | 0.0000 | 0.0000 | 0.0000 |

Table 104: Allocation factors for Montbéliarde Beef reared in Pasture

| COPRODUCT | Destination | Montbéliarde/beef/pasture | | |
| --- | --- | --- | --- | --- |
|  |  | **Biophysical Allocation Factor** | **Mass Allocation Factor** | **Economic Allocation Factor** |
| Abomasum | Human food | 0.0087 | 0.0016 | 0.0012 |
| Abomasum fat | Fat and greaves C3 | 0.0034 | 0.0016 | 0.0001 |
| Aponeurosis | Human food | 0.0012 | 0.0016 | 0.0016 |
| Bile | PAP C3 | 0.0004 | 0.0016 | 0.0001 |
| Blood | PAP C3 | 0.0009 | 0.0016 | 0.0004 |
| Blood | Pet food | 0.0009 | 0.0016 | 0.0001 |
| Bones | Gelatin C3 | 0.0010 | 0.0016 | 0.0000 |
| Bones of head, brain, eyes and teeth | C1-C2 for disposal | 0.0000 | 0.0000 | 0.0000 |
| Cheek | Human food | 0.0011 | 0.0016 | 0.0036 |
| Cheek | Human food | 0.0011 | 0.0016 | 0.0036 |
| Cheek trimmings | Pet food | 0.0011 | 0.0016 | 0.0001 |
| Chops | Pet food | 0.0015 | 0.0016 | 0.0001 |
| Contents of intestines | Spreading/Compost | 0.0000 | 0.0000 | 0.0000 |
| Contents of the rumen | Spreading/Compost | 0.0000 | 0.0000 | 0.0000 |
| Ears | PAP C3 | 0.0014 | 0.0016 | 0.0001 |
| Esophagus | Pet food | 0.0011 | 0.0016 | 0.0001 |
| Fat | Fat and greaves C3 | 0.0016 | 0.0016 | 0.0001 |
| Fat around heart | Fat and greaves C3 | 0.0016 | 0.0016 | 0.0001 |
| Fat in the kidney | Fat and greaves C3 | 0.0016 | 0.0016 | 0.0001 |
| Feet (without hooves) | Gelatin C3 | 0.0011 | 0.0016 | 0.0000 |
| Floatation fat | Spreading/Compost | 0.0000 | 0.0000 | 0.0000 |
| Forehead | C1-C2 for disposal | 0.0000 | 0.0000 | 0.0000 |
| Forelock | PAP C3 | 0.0039 | 0.0016 | 0.0001 |
| Gallbladder | Pet food | 0.0011 | 0.0016 | 0.0001 |
| Head trimmings | Pet food | 0.0011 | 0.0016 | 0.0001 |
| Heart | Human food | 0.0010 | 0.0016 | 0.0003 |
| Heart trimmings | Pet food | 0.0011 | 0.0016 | 0.0001 |
| Hide | Skin tannery C3 | 0.0015 | 0.0016 | 0.0027 |
| Hooves | PAP C3 | 0.0039 | 0.0016 | 0.0001 |
| Horns | PAP C3 | 0.0039 | 0.0016 | 0.0001 |
| Kidney | Human food | 0.0011 | 0.0016 | 0.0007 |
| Large intestine | C1-C2 for disposal | 0.0000 | 0.0000 | 0.0000 |
| Liver | Human food | 0.0045 | 0.0016 | 0.0008 |
| Liver trimmings | Pet food | 0.0047 | 0.0016 | 0.0001 |
| Lower jaw | PAP C3 | 0.0010 | 0.0016 | 0.0001 |
| Lungs | Pet food | 0.0013 | 0.0016 | 0.0001 |
| Mask | Skin tannery C3 | 0.0015 | 0.0016 | 0.0027 |
| Mesenteric fat | C1-C2 for disposal | 0.0000 | 0.0000 | 0.0000 |
| Muscle | Human food | 0.0011 | 0.0016 | 0.0027 |
| Muzzle | Human food | 0.0015 | 0.0016 | 0.0016 |
| Omasum | Human food | 0.0087 | 0.0016 | 0.0012 |
| Omasum fat | Fat and greaves C3 | 0.0034 | 0.0016 | 0.0001 |
| Rumen and forestomach | Human food | 0.0087 | 0.0016 | 0.0012 |
| Rumen fat | Fat and greaves C3 | 0.0034 | 0.0016 | 0.0001 |
| Sanitary seizures | C1-C2 for disposal | 0.0000 | 0.0000 | 0.0000 |
| Screening and sifting wastes | C1-C2 for disposal | 0.0000 | 0.0000 | 0.0000 |
| Small intestine | PAP C3 | 0.0087 | 0.0016 | 0.0001 |
| Spinal cord | C1-C2 for disposal | 0.0000 | 0.0000 | 0.0000 |
| Spinal cord waste | C1-C2 for disposal | 0.0000 | 0.0000 | 0.0000 |
| Spine | C1-C2 for disposal | 0.0000 | 0.0000 | 0.0000 |
| Spleen | Pet food | 0.0011 | 0.0016 | 0.0001 |
| Stillborn | PAP C3 | 0.0091 | 0.0016 | 0.0000 |
| Tallow | Fat and greaves C3 | 0.0016 | 0.0016 | 0.0001 |
| Tongue | Human food | 0.0010 | 0.0016 | 0.0026 |
| Tonsil | C1-C2 for disposal | 0.0000 | 0.0000 | 0.0000 |
| Trachea | Pet food | 0.0015 | 0.0016 | 0.0001 |
| Udder | Pet food | 0.0002 | 0.0016 | 0.0001 |
| Upper throat | Pet food | 0.0011 | 0.0016 | 0.0001 |
| Water in the rumen | Spreading/Compost | 0.0000 | 0.0000 | 0.0000 |

Table 105: Allocation factors for Montbéliarde Young Bulls reared in Stall

| COPRODUCT | Destination | Montbéliarde/young bull/stall | | |
| --- | --- | --- | --- | --- |
|  |  | **Biophysical Allocation Factor** | **Mass Allocation Factor** | **Economic Allocation Factor** |
| Abomasum | Human food | 0.0082 | 0.0016 | 0.0012 |
| Abomasum fat | Fat and greaves C3 | 0.0034 | 0.0016 | 0.0001 |
| Aponeurosis | Human food | 0.0012 | 0.0016 | 0.0016 |
| Bile | PAP C3 | 0.0004 | 0.0016 | 0.0001 |
| Blood | PAP C3 | 0.0009 | 0.0016 | 0.0003 |
| Blood | Pet food | 0.0009 | 0.0016 | 0.0001 |
| Bones | Gelatin C3 | 0.0010 | 0.0016 | 0.0000 |
| Bones of head, brain, eyes and teeth | C1-C2 for disposal | 0.0000 | 0.0000 | 0.0000 |
| Cheek | Human food | 0.0011 | 0.0016 | 0.0034 |
| Cheek | Human food | 0.0011 | 0.0016 | 0.0034 |
| Cheek trimmings | Pet food | 0.0011 | 0.0016 | 0.0001 |
| Chops | Pet food | 0.0015 | 0.0016 | 0.0001 |
| Contents of intestines | Spreading/Compost | 0.0000 | 0.0000 | 0.0000 |
| Contents of the rumen | Spreading/Compost | 0.0000 | 0.0000 | 0.0000 |
| Ears | PAP C3 | 0.0013 | 0.0016 | 0.0001 |
| Esophagus | Pet food | 0.0010 | 0.0016 | 0.0001 |
| Fat | Fat and greaves C3 | 0.0017 | 0.0016 | 0.0001 |
| Fat around heart | Fat and greaves C3 | 0.0017 | 0.0016 | 0.0001 |
| Fat in the kidney | Fat and greaves C3 | 0.0017 | 0.0016 | 0.0001 |
| Feet (without hooves) | Gelatin C3 | 0.0010 | 0.0016 | 0.0000 |
| Floatation fat | Spreading/Compost | 0.0000 | 0.0000 | 0.0000 |
| Forehead | C1-C2 for disposal | 0.0000 | 0.0000 | 0.0000 |
| Forelock | PAP C3 | 0.0038 | 0.0016 | 0.0001 |
| Gallbladder | Pet food | 0.0010 | 0.0016 | 0.0001 |
| Head trimmings | Pet food | 0.0011 | 0.0016 | 0.0001 |
| Heart | Human food | 0.0010 | 0.0016 | 0.0003 |
| Heart trimmings | Pet food | 0.0011 | 0.0016 | 0.0001 |
| Hide | Skin tannery C3 | 0.0015 | 0.0016 | 0.0026 |
| Hooves | PAP C3 | 0.0038 | 0.0016 | 0.0001 |
| Horns | PAP C3 | 0.0038 | 0.0016 | 0.0001 |
| Kidney | Human food | 0.0010 | 0.0016 | 0.0006 |
| Large intestine | C1-C2 for disposal | 0.0000 | 0.0000 | 0.0000 |
| Liver | Human food | 0.0043 | 0.0016 | 0.0008 |
| Liver trimmings | Pet food | 0.0045 | 0.0016 | 0.0001 |
| Lower jaw | PAP C3 | 0.0010 | 0.0016 | 0.0001 |
| Lungs | Pet food | 0.0012 | 0.0016 | 0.0001 |
| Mask | Skin tannery C3 | 0.0015 | 0.0016 | 0.0026 |
| Mesenteric fat | C1-C2 for disposal | 0.0000 | 0.0000 | 0.0000 |
| Muscle | Human food | 0.0011 | 0.0016 | 0.0026 |
| Muzzle | Human food | 0.0015 | 0.0016 | 0.0016 |
| Omasum | Human food | 0.0082 | 0.0016 | 0.0012 |
| Omasum fat | Fat and greaves C3 | 0.0034 | 0.0016 | 0.0001 |
| Rumen and forestomach | Human food | 0.0082 | 0.0016 | 0.0012 |
| Rumen fat | Fat and greaves C3 | 0.0034 | 0.0016 | 0.0001 |
| Sanitary seizures | C1-C2 for disposal | 0.0000 | 0.0000 | 0.0000 |
| Screening and sifting wastes | C1-C2 for disposal | 0.0000 | 0.0000 | 0.0000 |
| Small intestine | PAP C3 | 0.0082 | 0.0016 | 0.0001 |
| Spinal cord | C1-C2 for disposal | 0.0000 | 0.0000 | 0.0000 |
| Spinal cord waste | C1-C2 for disposal | 0.0000 | 0.0000 | 0.0000 |
| Spine | C1-C2 for disposal | 0.0000 | 0.0000 | 0.0000 |
| Spleen | Pet food | 0.0011 | 0.0016 | 0.0001 |
| Stillborn | PAP C3 | 0.0085 | 0.0016 | 0.0000 |
| Tallow | Fat and greaves C3 | 0.0017 | 0.0016 | 0.0001 |
| Tongue | Human food | 0.0010 | 0.0016 | 0.0025 |
| Tonsil | C1-C2 for disposal | 0.0000 | 0.0000 | 0.0000 |
| Trachea | Pet food | 0.0015 | 0.0016 | 0.0001 |
| Udder | Pet food | 0.0002 | 0.0016 | 0.0001 |
| Upper throat | Pet food | 0.0010 | 0.0016 | 0.0001 |
| Water in the rumen | Spreading/Compost | 0.0000 | 0.0000 | 0.0000 |

Table 106: Allocation factors for Montbéliarde Heifers reared in Stall

| COPRODUCT | Destination | Montbéliarde/heifer/stall | | |
| --- | --- | --- | --- | --- |
|  |  | **Biophysical Allocation Factor** | **Mass Allocation Factor** | **Economic Allocation Factor** |
| Abomasum | Human food | 0.0099 | 0.0020 | 0.0016 |
| Abomasum fat | Fat and greaves C3 | 0.0042 | 0.0020 | 0.0002 |
| Aponeurosis | Human food | 0.0015 | 0.0020 | 0.0021 |
| Bile | PAP C3 | 0.0006 | 0.0020 | 0.0002 |
| Blood | PAP C3 | 0.0012 | 0.0020 | 0.0005 |
| Blood | Pet food | 0.0012 | 0.0020 | 0.0002 |
| Bones | Gelatin C3 | 0.0013 | 0.0020 | 0.0000 |
| Bones of head, brain, eyes and teeth | C1-C2 for disposal | 0.0000 | 0.0000 | 0.0000 |
| Cheek | Human food | 0.0014 | 0.0020 | 0.0046 |
| Cheek | Human food | 0.0014 | 0.0020 | 0.0046 |
| Cheek trimmings | Pet food | 0.0014 | 0.0020 | 0.0002 |
| Chops | Pet food | 0.0020 | 0.0020 | 0.0002 |
| Contents of intestines | Spreading/Compost | 0.0000 | 0.0000 | 0.0000 |
| Contents of the rumen | Spreading/Compost | 0.0000 | 0.0000 | 0.0000 |
| Ears | PAP C3 | 0.0018 | 0.0020 | 0.0002 |
| Esophagus | Pet food | 0.0014 | 0.0020 | 0.0002 |
| Fat | Fat and greaves C3 | 0.0022 | 0.0020 | 0.0002 |
| Fat around heart | Fat and greaves C3 | 0.0022 | 0.0020 | 0.0002 |
| Fat in the kidney | Fat and greaves C3 | 0.0022 | 0.0020 | 0.0002 |
| Feet (without hooves) | Gelatin C3 | 0.0014 | 0.0020 | 0.0000 |
| Floatation fat | Spreading/Compost | 0.0000 | 0.0000 | 0.0000 |
| Forehead | C1-C2 for disposal | 0.0000 | 0.0000 | 0.0000 |
| Forelock | PAP C3 | 0.0050 | 0.0020 | 0.0002 |
| Gallbladder | Pet food | 0.0014 | 0.0020 | 0.0002 |
| Head trimmings | Pet food | 0.0014 | 0.0020 | 0.0002 |
| Heart | Human food | 0.0013 | 0.0020 | 0.0004 |
| Heart trimmings | Pet food | 0.0014 | 0.0020 | 0.0002 |
| Hide | Skin tannery C3 | 0.0020 | 0.0020 | 0.0035 |
| Hooves | PAP C3 | 0.0050 | 0.0020 | 0.0002 |
| Horns | PAP C3 | 0.0050 | 0.0020 | 0.0002 |
| Kidney | Human food | 0.0014 | 0.0020 | 0.0008 |
| Large intestine | C1-C2 for disposal | 0.0000 | 0.0000 | 0.0000 |
| Liver | Human food | 0.0053 | 0.0020 | 0.0010 |
| Liver trimmings | Pet food | 0.0055 | 0.0020 | 0.0002 |
| Lower jaw | PAP C3 | 0.0013 | 0.0020 | 0.0002 |
| Lungs | Pet food | 0.0016 | 0.0020 | 0.0002 |
| Mask | Skin tannery C3 | 0.0020 | 0.0020 | 0.0035 |
| Mesenteric fat | C1-C2 for disposal | 0.0000 | 0.0000 | 0.0000 |
| Muscle | Human food | 0.0014 | 0.0020 | 0.0035 |
| Muzzle | Human food | 0.0020 | 0.0020 | 0.0021 |
| Omasum | Human food | 0.0099 | 0.0020 | 0.0016 |
| Omasum fat | Fat and greaves C3 | 0.0042 | 0.0020 | 0.0002 |
| Rumen and forestomach | Human food | 0.0099 | 0.0020 | 0.0016 |
| Rumen fat | Fat and greaves C3 | 0.0042 | 0.0020 | 0.0002 |
| Sanitary seizures | C1-C2 for disposal | 0.0000 | 0.0000 | 0.0000 |
| Screening and sifting wastes | C1-C2 for disposal | 0.0000 | 0.0000 | 0.0000 |
| Small intestine | PAP C3 | 0.0099 | 0.0020 | 0.0001 |
| Spinal cord | C1-C2 for disposal | 0.0000 | 0.0000 | 0.0000 |
| Spinal cord waste | C1-C2 for disposal | 0.0000 | 0.0000 | 0.0000 |
| Spine | C1-C2 for disposal | 0.0000 | 0.0000 | 0.0000 |
| Spleen | Pet food | 0.0014 | 0.0020 | 0.0002 |
| Stillborn | PAP C3 | 0.0103 | 0.0020 | 0.0000 |
| Tallow | Fat and greaves C3 | 0.0022 | 0.0020 | 0.0002 |
| Tongue | Human food | 0.0013 | 0.0020 | 0.0033 |
| Tonsil | C1-C2 for disposal | 0.0000 | 0.0000 | 0.0000 |
| Trachea | Pet food | 0.0020 | 0.0020 | 0.0002 |
| Udder | Pet food | 0.0003 | 0.0020 | 0.0002 |
| Upper throat | Pet food | 0.0014 | 0.0020 | 0.0002 |
| Water in the rumen | Spreading/Compost | 0.0000 | 0.0000 | 0.0000 |

Table 107: Allocation factors for Montbéliarde Cull Cows reared in Stall

| COPRODUCT | Destination | Montbéliarde/Cull cow/stall | | |
| --- | --- | --- | --- | --- |
|  |  | **Biophysical Allocation Factor** | **Mass Allocation Factor** | **Economic Allocation Factor** |
| Abomasum | Human food | 0.0087 | 0.0017 | 0.0014 |
| Abomasum fat | Fat and greaves C3 | 0.0037 | 0.0017 | 0.0002 |
| Aponeurosis | Human food | 0.0013 | 0.0017 | 0.0018 |
| Bile | PAP C3 | 0.0005 | 0.0017 | 0.0002 |
| Blood | PAP C3 | 0.0010 | 0.0017 | 0.0004 |
| Blood | Pet food | 0.0010 | 0.0017 | 0.0001 |
| Bones | Gelatin C3 | 0.0011 | 0.0017 | 0.0000 |
| Bones of head, brain, eyes and teeth | C1-C2 for disposal | 0.0000 | 0.0000 | 0.0000 |
| Cheek | Human food | 0.0012 | 0.0017 | 0.0040 |
| Cheek | Human food | 0.0012 | 0.0017 | 0.0040 |
| Cheek trimmings | Pet food | 0.0012 | 0.0017 | 0.0001 |
| Chops | Pet food | 0.0016 | 0.0017 | 0.0001 |
| Contents of intestines | Spreading/Compost | 0.0000 | 0.0000 | 0.0000 |
| Contents of the rumen | Spreading/Compost | 0.0000 | 0.0000 | 0.0000 |
| Ears | PAP C3 | 0.0015 | 0.0017 | 0.0002 |
| Esophagus | Pet food | 0.0011 | 0.0017 | 0.0001 |
| Fat | Fat and greaves C3 | 0.0019 | 0.0017 | 0.0002 |
| Fat around heart | Fat and greaves C3 | 0.0019 | 0.0017 | 0.0002 |
| Fat in the kidney | Fat and greaves C3 | 0.0019 | 0.0017 | 0.0002 |
| Feet (without hooves) | Gelatin C3 | 0.0012 | 0.0017 | 0.0000 |
| Floatation fat | Spreading/Compost | 0.0000 | 0.0000 | 0.0000 |
| Forehead | C1-C2 for disposal | 0.0000 | 0.0000 | 0.0000 |
| Forelock | PAP C3 | 0.0042 | 0.0017 | 0.0002 |
| Gallbladder | Pet food | 0.0012 | 0.0017 | 0.0001 |
| Head trimmings | Pet food | 0.0012 | 0.0017 | 0.0001 |
| Heart | Human food | 0.0011 | 0.0017 | 0.0004 |
| Heart trimmings | Pet food | 0.0012 | 0.0017 | 0.0001 |
| Hide | Skin tannery C3 | 0.0016 | 0.0017 | 0.0030 |
| Hooves | PAP C3 | 0.0042 | 0.0017 | 0.0002 |
| Horns | PAP C3 | 0.0042 | 0.0017 | 0.0002 |
| Kidney | Human food | 0.0012 | 0.0017 | 0.0007 |
| Large intestine | C1-C2 for disposal | 0.0000 | 0.0000 | 0.0000 |
| Liver | Human food | 0.0046 | 0.0017 | 0.0009 |
| Liver trimmings | Pet food | 0.0048 | 0.0017 | 0.0001 |
| Lower jaw | PAP C3 | 0.0011 | 0.0017 | 0.0002 |
| Lungs | Pet food | 0.0013 | 0.0017 | 0.0001 |
| Mask | Skin tannery C3 | 0.0016 | 0.0017 | 0.0030 |
| Mesenteric fat | C1-C2 for disposal | 0.0000 | 0.0000 | 0.0000 |
| Muscle | Human food | 0.0012 | 0.0017 | 0.0030 |
| Muzzle | Human food | 0.0016 | 0.0017 | 0.0018 |
| Omasum | Human food | 0.0087 | 0.0017 | 0.0014 |
| Omasum fat | Fat and greaves C3 | 0.0037 | 0.0017 | 0.0002 |
| Rumen and forestomach | Human food | 0.0087 | 0.0017 | 0.0014 |
| Rumen fat | Fat and greaves C3 | 0.0037 | 0.0017 | 0.0002 |
| Sanitary seizures | C1-C2 for disposal | 0.0000 | 0.0000 | 0.0000 |
| Screening and sifting wastes | C1-C2 for disposal | 0.0000 | 0.0000 | 0.0000 |
| Small intestine | PAP C3 | 0.0087 | 0.0017 | 0.0001 |
| Spinal cord | C1-C2 for disposal | 0.0000 | 0.0000 | 0.0000 |
| Spinal cord waste | C1-C2 for disposal | 0.0000 | 0.0000 | 0.0000 |
| Spine | C1-C2 for disposal | 0.0000 | 0.0000 | 0.0000 |
| Spleen | Pet food | 0.0012 | 0.0017 | 0.0001 |
| Stillborn | PAP C3 | 0.0091 | 0.0017 | 0.0000 |
| Tallow | Fat and greaves C3 | 0.0019 | 0.0017 | 0.0002 |
| Tongue | Human food | 0.0010 | 0.0017 | 0.0029 |
| Tonsil | C1-C2 for disposal | 0.0000 | 0.0000 | 0.0000 |
| Trachea | Pet food | 0.0016 | 0.0017 | 0.0001 |
| Udder | Pet food | 0.0003 | 0.0017 | 0.0001 |
| Upper throat | Pet food | 0.0012 | 0.0017 | 0.0001 |
| Water in the rumen | Spreading/Compost | 0.0000 | 0.0000 | 0.0000 |

Table 108: Allocation factors for Montbéliarde Beef reared in Stall

| COPRODUCT | Destination | Montbéliarde/beef/stall | | |
| --- | --- | --- | --- | --- |
|  |  | **Biophysical Allocation Factor** | **Mass Allocation Factor** | **Economic Allocation Factor** |
| Abomasum | Human food | 0.0083 | 0.0016 | 0.0012 |
| Abomasum fat | Fat and greaves C3 | 0.0035 | 0.0016 | 0.0001 |
| Aponeurosis | Human food | 0.0012 | 0.0016 | 0.0016 |
| Bile | PAP C3 | 0.0004 | 0.0016 | 0.0001 |
| Blood | PAP C3 | 0.0009 | 0.0016 | 0.0004 |
| Blood | Pet food | 0.0009 | 0.0016 | 0.0001 |
| Bones | Gelatin C3 | 0.0010 | 0.0016 | 0.0000 |
| Bones of head, brain, eyes and teeth | C1-C2 for disposal | 0.0000 | 0.0000 | 0.0000 |
| Cheek | Human food | 0.0011 | 0.0016 | 0.0036 |
| Cheek | Human food | 0.0011 | 0.0016 | 0.0036 |
| Cheek trimmings | Pet food | 0.0011 | 0.0016 | 0.0001 |
| Chops | Pet food | 0.0015 | 0.0016 | 0.0001 |
| Contents of intestines | Spreading/Compost | 0.0000 | 0.0000 | 0.0000 |
| Contents of the rumen | Spreading/Compost | 0.0000 | 0.0000 | 0.0000 |
| Ears | PAP C3 | 0.0014 | 0.0016 | 0.0001 |
| Esophagus | Pet food | 0.0011 | 0.0016 | 0.0001 |
| Fat | Fat and greaves C3 | 0.0018 | 0.0016 | 0.0001 |
| Fat around heart | Fat and greaves C3 | 0.0018 | 0.0016 | 0.0001 |
| Fat in the kidney | Fat and greaves C3 | 0.0018 | 0.0016 | 0.0001 |
| Feet (without hooves) | Gelatin C3 | 0.0011 | 0.0016 | 0.0000 |
| Floatation fat | Spreading/Compost | 0.0000 | 0.0000 | 0.0000 |
| Forehead | C1-C2 for disposal | 0.0000 | 0.0000 | 0.0000 |
| Forelock | PAP C3 | 0.0039 | 0.0016 | 0.0001 |
| Gallbladder | Pet food | 0.0011 | 0.0016 | 0.0001 |
| Head trimmings | Pet food | 0.0011 | 0.0016 | 0.0001 |
| Heart | Human food | 0.0010 | 0.0016 | 0.0003 |
| Heart trimmings | Pet food | 0.0011 | 0.0016 | 0.0001 |
| Hide | Skin tannery C3 | 0.0015 | 0.0016 | 0.0027 |
| Hooves | PAP C3 | 0.0039 | 0.0016 | 0.0001 |
| Horns | PAP C3 | 0.0039 | 0.0016 | 0.0001 |
| Kidney | Human food | 0.0011 | 0.0016 | 0.0007 |
| Large intestine | C1-C2 for disposal | 0.0000 | 0.0000 | 0.0000 |
| Liver | Human food | 0.0043 | 0.0016 | 0.0008 |
| Liver trimmings | Pet food | 0.0045 | 0.0016 | 0.0001 |
| Lower jaw | PAP C3 | 0.0010 | 0.0016 | 0.0001 |
| Lungs | Pet food | 0.0012 | 0.0016 | 0.0001 |
| Mask | Skin tannery C3 | 0.0015 | 0.0016 | 0.0027 |
| Mesenteric fat | C1-C2 for disposal | 0.0000 | 0.0000 | 0.0000 |
| Muscle | Human food | 0.0011 | 0.0016 | 0.0027 |
| Muzzle | Human food | 0.0015 | 0.0016 | 0.0016 |
| Omasum | Human food | 0.0083 | 0.0016 | 0.0012 |
| Omasum fat | Fat and greaves C3 | 0.0035 | 0.0016 | 0.0001 |
| Rumen and forestomach | Human food | 0.0083 | 0.0016 | 0.0012 |
| Rumen fat | Fat and greaves C3 | 0.0035 | 0.0016 | 0.0001 |
| Sanitary seizures | C1-C2 for disposal | 0.0000 | 0.0000 | 0.0000 |
| Screening and sifting wastes | C1-C2 for disposal | 0.0000 | 0.0000 | 0.0000 |
| Small intestine | PAP C3 | 0.0083 | 0.0016 | 0.0001 |
| Spinal cord | C1-C2 for disposal | 0.0000 | 0.0000 | 0.0000 |
| Spinal cord waste | C1-C2 for disposal | 0.0000 | 0.0000 | 0.0000 |
| Spine | C1-C2 for disposal | 0.0000 | 0.0000 | 0.0000 |
| Spleen | Pet food | 0.0011 | 0.0016 | 0.0001 |
| Stillborn | PAP C3 | 0.0086 | 0.0016 | 0.0000 |
| Tallow | Fat and greaves C3 | 0.0018 | 0.0016 | 0.0001 |
| Tongue | Human food | 0.0010 | 0.0016 | 0.0026 |
| Tonsil | C1-C2 for disposal | 0.0000 | 0.0000 | 0.0000 |
| Trachea | Pet food | 0.0015 | 0.0016 | 0.0001 |
| Udder | Pet food | 0.0002 | 0.0016 | 0.0001 |
| Upper throat | Pet food | 0.0011 | 0.0016 | 0.0001 |
| Water in the rumen | Spreading/Compost | 0.0000 | 0.0000 | 0.0000 |

Table 109: Allocation factors for Charolaise x Pie Noire Young Bulls reared in Grazing Large Area

| COPRODUCT | Destination | Charolaise x Pie Noire/young bull/grazing large area | | |
| --- | --- | --- | --- | --- |
|  |  | **Biophysical Allocation Factor** | **Mass Allocation Factor** | **Economic Allocation Factor** |
| Abomasum | Human food | 0.0087 | 0.0015 | 0.0011 |
| Abomasum fat | Fat and greaves C3 | 0.0032 | 0.0015 | 0.0001 |
| Aponeurosis | Human food | 0.0011 | 0.0015 | 0.0015 |
| Bile | PAP C3 | 0.0004 | 0.0015 | 0.0001 |
| Blood | PAP C3 | 0.0009 | 0.0015 | 0.0003 |
| Blood | Pet food | 0.0009 | 0.0015 | 0.0001 |
| Bones | Gelatin C3 | 0.0009 | 0.0015 | 0.0000 |
| Bones of head, brain, eyes and teeth | C1-C2 for disposal | 0.0000 | 0.0000 | 0.0000 |
| Cheek | Human food | 0.0010 | 0.0015 | 0.0032 |
| Cheek | Human food | 0.0010 | 0.0015 | 0.0032 |
| Cheek trimmings | Pet food | 0.0010 | 0.0015 | 0.0001 |
| Chops | Pet food | 0.0014 | 0.0015 | 0.0001 |
| Contents of intestines | Spreading/Compost | 0.0000 | 0.0000 | 0.0000 |
| Contents of the rumen | Spreading/Compost | 0.0000 | 0.0000 | 0.0000 |
| Ears | PAP C3 | 0.0012 | 0.0015 | 0.0001 |
| Esophagus | Pet food | 0.0010 | 0.0015 | 0.0001 |
| Fat | Fat and greaves C3 | 0.0013 | 0.0015 | 0.0001 |
| Fat around heart | Fat and greaves C3 | 0.0013 | 0.0015 | 0.0001 |
| Fat in the kidney | Fat and greaves C3 | 0.0013 | 0.0015 | 0.0001 |
| Feet (without hooves) | Gelatin C3 | 0.0010 | 0.0015 | 0.0000 |
| Floatation fat | Spreading/Compost | 0.0000 | 0.0000 | 0.0000 |
| Forehead | C1-C2 for disposal | 0.0000 | 0.0000 | 0.0000 |
| Forelock | PAP C3 | 0.0036 | 0.0015 | 0.0001 |
| Gallbladder | Pet food | 0.0010 | 0.0015 | 0.0001 |
| Head trimmings | Pet food | 0.0010 | 0.0015 | 0.0001 |
| Heart | Human food | 0.0010 | 0.0015 | 0.0003 |
| Heart trimmings | Pet food | 0.0010 | 0.0015 | 0.0001 |
| Hide | Skin tannery C3 | 0.0014 | 0.0015 | 0.0024 |
| Hooves | PAP C3 | 0.0036 | 0.0015 | 0.0001 |
| Horns | PAP C3 | 0.0036 | 0.0015 | 0.0001 |
| Kidney | Human food | 0.0010 | 0.0015 | 0.0006 |
| Large intestine | C1-C2 for disposal | 0.0000 | 0.0000 | 0.0000 |
| Liver | Human food | 0.0045 | 0.0015 | 0.0007 |
| Liver trimmings | Pet food | 0.0047 | 0.0015 | 0.0001 |
| Lower jaw | PAP C3 | 0.0009 | 0.0015 | 0.0001 |
| Lungs | Pet food | 0.0012 | 0.0015 | 0.0001 |
| Mask | Skin tannery C3 | 0.0014 | 0.0015 | 0.0024 |
| Mesenteric fat | C1-C2 for disposal | 0.0000 | 0.0000 | 0.0000 |
| Muscle | Human food | 0.0010 | 0.0015 | 0.0024 |
| Muzzle | Human food | 0.0014 | 0.0015 | 0.0015 |
| Omasum | Human food | 0.0087 | 0.0015 | 0.0011 |
| Omasum fat | Fat and greaves C3 | 0.0032 | 0.0015 | 0.0001 |
| Rumen and forestomach | Human food | 0.0087 | 0.0015 | 0.0011 |
| Rumen fat | Fat and greaves C3 | 0.0032 | 0.0015 | 0.0001 |
| Sanitary seizures | C1-C2 for disposal | 0.0000 | 0.0000 | 0.0000 |
| Screening and sifting wastes | C1-C2 for disposal | 0.0000 | 0.0000 | 0.0000 |
| Small intestine | PAP C3 | 0.0087 | 0.0015 | 0.0001 |
| Spinal cord | C1-C2 for disposal | 0.0000 | 0.0000 | 0.0000 |
| Spinal cord waste | C1-C2 for disposal | 0.0000 | 0.0000 | 0.0000 |
| Spine | C1-C2 for disposal | 0.0000 | 0.0000 | 0.0000 |
| Spleen | Pet food | 0.0010 | 0.0015 | 0.0001 |
| Stillborn | PAP C3 | 0.0090 | 0.0015 | 0.0000 |
| Tallow | Fat and greaves C3 | 0.0013 | 0.0015 | 0.0001 |
| Tongue | Human food | 0.0009 | 0.0015 | 0.0023 |
| Tonsil | C1-C2 for disposal | 0.0000 | 0.0000 | 0.0000 |
| Trachea | Pet food | 0.0014 | 0.0015 | 0.0001 |
| Udder | Pet food | 0.0002 | 0.0015 | 0.0001 |
| Upper throat | Pet food | 0.0010 | 0.0015 | 0.0001 |
| Water in the rumen | Spreading/Compost | 0.0000 | 0.0000 | 0.0000 |

Table 110: Allocation factors for Charolaise x Pie Noire Heifers reared in Grazing Large Area

| COPRODUCT | Destination | Charolaise x Pie Noire/heifer/grazing large area | | |
| --- | --- | --- | --- | --- |
|  |  | **Biophysical Allocation Factor** | **Mass Allocation Factor** | **Economic Allocation Factor** |
| Abomasum | Human food | 0.0090 | 0.0016 | 0.0012 |
| Abomasum fat | Fat and greaves C3 | 0.0034 | 0.0016 | 0.0001 |
| Aponeurosis | Human food | 0.0012 | 0.0016 | 0.0016 |
| Bile | PAP C3 | 0.0004 | 0.0016 | 0.0001 |
| Blood | PAP C3 | 0.0009 | 0.0016 | 0.0004 |
| Blood | Pet food | 0.0009 | 0.0016 | 0.0001 |
| Bones | Gelatin C3 | 0.0010 | 0.0016 | 0.0000 |
| Bones of head, brain, eyes and teeth | C1-C2 for disposal | 0.0000 | 0.0000 | 0.0000 |
| Cheek | Human food | 0.0011 | 0.0016 | 0.0036 |
| Cheek | Human food | 0.0011 | 0.0016 | 0.0036 |
[truncated: 83,435 more chars]
