# Supplementary material for: Allocation factors for meat coproducts: Dataset to perform life cycle assessment at slaughterhouse
Source: Data Brief. 2020 Nov 23;33:106558. doi: 10.1016/j.dib.2020.106558 (PMC7718151; doi:10.1016/j.dib.2020.106558)
Supplement: Supplementary file 2 [file mmc2.docx]

Table 1: Total weighting by coproducts for Normande Young Bulls reared in Grazing Large Area

| COPRODUCT | Destination | Normande/young bull/grazing large area | | |
| --- | --- | --- | --- | --- |
|  |  | **Biophysical Cumulative share** | **Mass Cumulative share** | **Economic Cumulative share** |
| Abomasum | Human food | 0.0151 | 0.0026 | 0.0020 |
| Abomasum fat | Fat and greaves C3 | 0.0011 | 0.0005 | 0.0000 |
| Aponeurosis | Human food | 0.0261 | 0.0341 | 0.0342 |
| Bile | PAP C3 | 0.0000 | 0.0001 | 0.0000 |
| Blood | PAP C3 | 0.0183 | 0.0311 | 0.0070 |
| Blood | Pet food | 0.0026 | 0.0045 | 0.0003 |
| Bones | Gelatin C3 | 0.0484 | 0.0764 | 0.0002 |
| Bones of head, brain, eyes and teeth | C1-C2 for disposal | 0.0000 | 0.0000 | 0.0000 |
| Cheek | Human food | 0.0011 | 0.0016 | 0.0035 |
| Cheek | Human food | 0.0020 | 0.0029 | 0.0063 |
| Cheek trimmings | Pet food | 0.0005 | 0.0007 | 0.0001 |
| Chops | Pet food | 0.0020 | 0.0021 | 0.0002 |
| Contents of intestines | Spreading/Compost | 0.0000 | 0.0000 | 0.0000 |
| Contents of the rumen | Spreading/Compost | 0.0000 | 0.0000 | 0.0000 |
| Ears | PAP C3 | 0.0009 | 0.0010 | 0.0001 |
| Esophagus | Pet food | 0.0010 | 0.0015 | 0.0001 |
| Fat | Fat and greaves C3 | 0.0765 | 0.0818 | 0.0074 |
| Fat around heart | Fat and greaves C3 | 0.0027 | 0.0029 | 0.0003 |
| Fat in the kidney | Fat and greaves C3 | 0.0003 | 0.0004 | 0.0000 |
| Feet (without hooves) | Gelatin C3 | 0.0128 | 0.0187 | 0.0001 |
| Floatation fat | Spreading/Compost | 0.0000 | 0.0000 | 0.0000 |
| Forehead | C1-C2 for disposal | 0.0000 | 0.0000 | 0.0000 |
| Forelock | PAP C3 | 0.0028 | 0.0011 | 0.0001 |
| Gallbladder | Pet food | 0.0003 | 0.0005 | 0.0000 |
| Head trimmings | Pet food | 0.0028 | 0.0040 | 0.0003 |
| Heart | Human food | 0.0031 | 0.0047 | 0.0010 |
| Heart trimmings | Pet food | 0.0003 | 0.0005 | 0.0000 |
| Hide | Skin tannery C3 | 0.0710 | 0.0733 | 0.1223 |
| Hooves | PAP C3 | 0.0081 | 0.0032 | 0.0003 |
| Horns | PAP C3 | 0.0019 | 0.0007 | 0.0001 |
| Kidney | Human food | 0.0020 | 0.0029 | 0.0012 |
| Large intestine | C1-C2 for disposal | 0.0000 | 0.0000 | 0.0000 |
| Liver | Human food | 0.0569 | 0.0190 | 0.0092 |
| Liver trimmings | Pet food | 0.0066 | 0.0021 | 0.0002 |
| Lower jaw | PAP C3 | 0.0032 | 0.0052 | 0.0004 |
| Lungs | Pet food | 0.0084 | 0.0105 | 0.0008 |
| Mask | Skin tannery C3 | 0.0048 | 0.0050 | 0.0083 |
| Mesenteric fat | C1-C2 for disposal | 0.0000 | 0.0000 | 0.0000 |
| Muscle | Human food | 0.3185 | 0.4569 | 0.7640 |
| Muzzle | Human food | 0.0025 | 0.0026 | 0.0026 |
| Omasum | Human food | 0.0136 | 0.0024 | 0.0018 |
| Omasum fat | Fat and greaves C3 | 0.0035 | 0.0016 | 0.0001 |
| Rumen and forestomach | Human food | 0.0703 | 0.0121 | 0.0091 |
| Rumen fat | Fat and greaves C3 | 0.0051 | 0.0024 | 0.0002 |
| Sanitary seizures | C1-C2 for disposal | 0.0000 | 0.0000 | 0.0000 |
| Screening and sifting wastes | C1-C2 for disposal | 0.0000 | 0.0000 | 0.0000 |
| Small intestine | PAP C3 | 0.0818 | 0.0141 | 0.0009 |
| Spinal cord | C1-C2 for disposal | 0.0000 | 0.0000 | 0.0000 |
| Spinal cord waste | C1-C2 for disposal | 0.0000 | 0.0000 | 0.0000 |
| Spine | C1-C2 for disposal | 0.0000 | 0.0000 | 0.0000 |
| Spleen | Pet food | 0.0020 | 0.0029 | 0.0002 |
| Stillborn | PAP C3 | 0.0427 | 0.0071 | 0.0000 |
| Tallow | Fat and greaves C3 | 0.0678 | 0.0727 | 0.0066 |
| Tongue | Human food | 0.0025 | 0.0042 | 0.0067 |
| Tonsil | C1-C2 for disposal | 0.0000 | 0.0000 | 0.0000 |
| Trachea | Pet food | 0.0018 | 0.0019 | 0.0001 |
| Udder | Pet food | 0.0032 | 0.0222 | 0.0016 |
| Upper throat | Pet food | 0.0011 | 0.0016 | 0.0001 |
| Water in the rumen | Spreading/Compost | 0.0000 | 0.0000 | 0.0000 |

Table 2: Total weighting by coproducts for Normande Heifers reared in Grazing Large Area

| COPRODUCT | Destination | Normande/heifer/grazing large area | | |
| --- | --- | --- | --- | --- |
|  |  | **Biophysical Cumulative share** | **Mass Cumulative share** | **Economic Cumulative share** |
| Abomasum | Human food | 0.0153 | 0.0028 | 0.0021 |
| Abomasum fat | Fat and greaves C3 | 0.0011 | 0.0005 | 0.0000 |
| Aponeurosis | Human food | 0.0247 | 0.0326 | 0.0334 |
| Bile | PAP C3 | 0.0000 | 0.0001 | 0.0000 |
| Blood | PAP C3 | 0.0196 | 0.0335 | 0.0076 |
| Blood | Pet food | 0.0028 | 0.0048 | 0.0004 |
| Bones | Gelatin C3 | 0.0459 | 0.0732 | 0.0002 |
| Bones of head, brain, eyes and teeth | C1-C2 for disposal | 0.0000 | 0.0000 | 0.0000 |
| Cheek | Human food | 0.0011 | 0.0016 | 0.0037 |
| Cheek | Human food | 0.0022 | 0.0031 | 0.0070 |
| Cheek trimmings | Pet food | 0.0006 | 0.0009 | 0.0001 |
| Chops | Pet food | 0.0022 | 0.0023 | 0.0002 |
| Contents of intestines | Spreading/Compost | 0.0000 | 0.0000 | 0.0000 |
| Contents of the rumen | Spreading/Compost | 0.0000 | 0.0000 | 0.0000 |
| Ears | PAP C3 | 0.0010 | 0.0011 | 0.0001 |
| Esophagus | Pet food | 0.0010 | 0.0015 | 0.0001 |
| Fat | Fat and greaves C3 | 0.0721 | 0.0783 | 0.0073 |
| Fat around heart | Fat and greaves C3 | 0.0029 | 0.0031 | 0.0003 |
| Fat in the kidney | Fat and greaves C3 | 0.0003 | 0.0004 | 0.0000 |
| Feet (without hooves) | Gelatin C3 | 0.0137 | 0.0202 | 0.0001 |
| Floatation fat | Spreading/Compost | 0.0000 | 0.0000 | 0.0000 |
| Forehead | C1-C2 for disposal | 0.0000 | 0.0000 | 0.0000 |
| Forelock | PAP C3 | 0.0031 | 0.0013 | 0.0001 |
| Gallbladder | Pet food | 0.0003 | 0.0005 | 0.0000 |
| Head trimmings | Pet food | 0.0029 | 0.0043 | 0.0003 |
| Heart | Human food | 0.0033 | 0.0050 | 0.0011 |
| Heart trimmings | Pet food | 0.0003 | 0.0005 | 0.0000 |
| Hide | Skin tannery C3 | 0.0759 | 0.0791 | 0.1346 |
| Hooves | PAP C3 | 0.0087 | 0.0035 | 0.0003 |
| Horns | PAP C3 | 0.0022 | 0.0009 | 0.0001 |
| Kidney | Human food | 0.0021 | 0.0031 | 0.0013 |
| Large intestine | C1-C2 for disposal | 0.0000 | 0.0000 | 0.0000 |
| Liver | Human food | 0.0589 | 0.0205 | 0.0101 |
| Liver trimmings | Pet food | 0.0068 | 0.0023 | 0.0002 |
| Lower jaw | PAP C3 | 0.0035 | 0.0056 | 0.0005 |
| Lungs | Pet food | 0.0091 | 0.0114 | 0.0009 |
| Mask | Skin tannery C3 | 0.0052 | 0.0054 | 0.0092 |
| Mesenteric fat | C1-C2 for disposal | 0.0000 | 0.0000 | 0.0000 |
| Muscle | Human food | 0.3020 | 0.4374 | 0.7457 |
| Muzzle | Human food | 0.0027 | 0.0028 | 0.0028 |
| Omasum | Human food | 0.0139 | 0.0025 | 0.0019 |
| Omasum fat | Fat and greaves C3 | 0.0034 | 0.0016 | 0.0002 |
| Rumen and forestomach | Human food | 0.0722 | 0.0131 | 0.0100 |
| Rumen fat | Fat and greaves C3 | 0.0053 | 0.0025 | 0.0002 |
| Sanitary seizures | C1-C2 for disposal | 0.0000 | 0.0000 | 0.0000 |
| Screening and sifting wastes | C1-C2 for disposal | 0.0000 | 0.0000 | 0.0000 |
| Small intestine | PAP C3 | 0.0840 | 0.0152 | 0.0009 |
| Spinal cord | C1-C2 for disposal | 0.0000 | 0.0000 | 0.0000 |
| Spinal cord waste | C1-C2 for disposal | 0.0000 | 0.0000 | 0.0000 |
| Spine | C1-C2 for disposal | 0.0000 | 0.0000 | 0.0000 |
| Spleen | Pet food | 0.0022 | 0.0031 | 0.0002 |
| Stillborn | PAP C3 | 0.0442 | 0.0077 | 0.0000 |
| Tallow | Fat and greaves C3 | 0.0721 | 0.0785 | 0.0073 |
| Tongue | Human food | 0.0027 | 0.0045 | 0.0073 |
| Tonsil | C1-C2 for disposal | 0.0000 | 0.0000 | 0.0000 |
| Trachea | Pet food | 0.0019 | 0.0020 | 0.0002 |
| Udder | Pet food | 0.0034 | 0.0240 | 0.0018 |
| Upper throat | Pet food | 0.0011 | 0.0016 | 0.0001 |
| Water in the rumen | Spreading/Compost | 0.0000 | 0.0000 | 0.0000 |

Table 3: Total weighting by coproducts for Normande Cull Cows reared in Grazing Large Area

| COPRODUCT | Destination | Normande/Cull cow/grazing large area | | |
| --- | --- | --- | --- | --- |
|  |  | **Biophysical Cumulative share** | **Mass Cumulative share** | **Economic Cumulative share** |
| Abomasum | Human food | 0.0163 | 0.0029 | 0.0023 |
| Abomasum fat | Fat and greaves C3 | 0.0013 | 0.0006 | 0.0001 |
| Aponeurosis | Human food | 0.0234 | 0.0316 | 0.0328 |
| Bile | PAP C3 | 0.0000 | 0.0001 | 0.0000 |
| Blood | PAP C3 | 0.0200 | 0.0352 | 0.0081 |
| Blood | Pet food | 0.0029 | 0.0051 | 0.0004 |
| Bones | Gelatin C3 | 0.0433 | 0.0709 | 0.0002 |
| Bones of head, brain, eyes and teeth | C1-C2 for disposal | 0.0000 | 0.0000 | 0.0000 |
| Cheek | Human food | 0.0012 | 0.0018 | 0.0040 |
| Cheek | Human food | 0.0022 | 0.0033 | 0.0075 |
| Cheek trimmings | Pet food | 0.0006 | 0.0009 | 0.0001 |
| Chops | Pet food | 0.0023 | 0.0024 | 0.0002 |
| Contents of intestines | Spreading/Compost | 0.0000 | 0.0000 | 0.0000 |
| Contents of the rumen | Spreading/Compost | 0.0000 | 0.0000 | 0.0000 |
| Ears | PAP C3 | 0.0009 | 0.0011 | 0.0001 |
| Esophagus | Pet food | 0.0011 | 0.0016 | 0.0001 |
| Fat | Fat and greaves C3 | 0.0675 | 0.0759 | 0.0071 |
| Fat around heart | Fat and greaves C3 | 0.0029 | 0.0033 | 0.0003 |
| Fat in the kidney | Fat and greaves C3 | 0.0004 | 0.0005 | 0.0000 |
| Feet (without hooves) | Gelatin C3 | 0.0139 | 0.0211 | 0.0001 |
| Floatation fat | Spreading/Compost | 0.0000 | 0.0000 | 0.0000 |
| Forehead | C1-C2 for disposal | 0.0000 | 0.0000 | 0.0000 |
| Forelock | PAP C3 | 0.0034 | 0.0014 | 0.0001 |
| Gallbladder | Pet food | 0.0004 | 0.0006 | 0.0000 |
| Head trimmings | Pet food | 0.0030 | 0.0044 | 0.0003 |
| Heart | Human food | 0.0034 | 0.0053 | 0.0012 |
| Heart trimmings | Pet food | 0.0004 | 0.0006 | 0.0000 |
| Hide | Skin tannery C3 | 0.0777 | 0.0830 | 0.1432 |
| Hooves | PAP C3 | 0.0089 | 0.0037 | 0.0003 |
| Horns | PAP C3 | 0.0021 | 0.0009 | 0.0001 |
| Kidney | Human food | 0.0022 | 0.0033 | 0.0014 |
| Large intestine | C1-C2 for disposal | 0.0000 | 0.0000 | 0.0000 |
| Liver | Human food | 0.0624 | 0.0215 | 0.0108 |
| Liver trimmings | Pet food | 0.0073 | 0.0024 | 0.0002 |
| Lower jaw | PAP C3 | 0.0036 | 0.0059 | 0.0005 |
| Lungs | Pet food | 0.0093 | 0.0120 | 0.0009 |
| Mask | Skin tannery C3 | 0.0052 | 0.0056 | 0.0096 |
| Mesenteric fat | C1-C2 for disposal | 0.0000 | 0.0000 | 0.0000 |
| Muscle | Human food | 0.2855 | 0.4239 | 0.7327 |
| Muzzle | Human food | 0.0027 | 0.0029 | 0.0030 |
| Omasum | Human food | 0.0149 | 0.0027 | 0.0021 |
| Omasum fat | Fat and greaves C3 | 0.0037 | 0.0018 | 0.0002 |
| Rumen and forestomach | Human food | 0.0773 | 0.0138 | 0.0107 |
| Rumen fat | Fat and greaves C3 | 0.0056 | 0.0027 | 0.0003 |
| Sanitary seizures | C1-C2 for disposal | 0.0000 | 0.0000 | 0.0000 |
| Screening and sifting wastes | C1-C2 for disposal | 0.0000 | 0.0000 | 0.0000 |
| Small intestine | PAP C3 | 0.0894 | 0.0159 | 0.0010 |
| Spinal cord | C1-C2 for disposal | 0.0000 | 0.0000 | 0.0000 |
| Spinal cord waste | C1-C2 for disposal | 0.0000 | 0.0000 | 0.0000 |
| Spine | C1-C2 for disposal | 0.0000 | 0.0000 | 0.0000 |
| Spleen | Pet food | 0.0022 | 0.0033 | 0.0002 |
| Stillborn | PAP C3 | 0.0467 | 0.0080 | 0.0000 |
| Tallow | Fat and greaves C3 | 0.0731 | 0.0824 | 0.0078 |
| Tongue | Human food | 0.0027 | 0.0047 | 0.0077 |
| Tonsil | C1-C2 for disposal | 0.0000 | 0.0000 | 0.0000 |
| Trachea | Pet food | 0.0019 | 0.0020 | 0.0002 |
| Udder | Pet food | 0.0035 | 0.0252 | 0.0019 |
| Upper throat | Pet food | 0.0012 | 0.0018 | 0.0001 |
| Water in the rumen | Spreading/Compost | 0.0000 | 0.0000 | 0.0000 |

Table 4: Total weighting by coproducts for Normande Beef reared in Grazing Large Area

| COPRODUCT | Destination | Normande/beef/grazing large area | | |
| --- | --- | --- | --- | --- |
|  |  | **Biophysical Cumulative share** | **Mass Cumulative share** | **Economic Cumulative share** |
| Abomasum | Human food | 0.0160 | 0.0028 | 0.0021 |
| Abomasum fat | Fat and greaves C3 | 0.0011 | 0.0005 | 0.0000 |
| Aponeurosis | Human food | 0.0249 | 0.0331 | 0.0337 |
| Bile | PAP C3 | 0.0000 | 0.0001 | 0.0000 |
| Blood | PAP C3 | 0.0190 | 0.0328 | 0.0074 |
| Blood | Pet food | 0.0027 | 0.0046 | 0.0003 |
| Bones | Gelatin C3 | 0.0461 | 0.0743 | 0.0002 |
| Bones of head, brain, eyes and teeth | C1-C2 for disposal | 0.0000 | 0.0000 | 0.0000 |
| Cheek | Human food | 0.0011 | 0.0016 | 0.0036 |
| Cheek | Human food | 0.0020 | 0.0030 | 0.0067 |
| Cheek trimmings | Pet food | 0.0006 | 0.0009 | 0.0001 |
| Chops | Pet food | 0.0021 | 0.0023 | 0.0002 |
| Contents of intestines | Spreading/Compost | 0.0000 | 0.0000 | 0.0000 |
| Contents of the rumen | Spreading/Compost | 0.0000 | 0.0000 | 0.0000 |
| Ears | PAP C3 | 0.0009 | 0.0011 | 0.0001 |
| Esophagus | Pet food | 0.0010 | 0.0015 | 0.0001 |
| Fat | Fat and greaves C3 | 0.0717 | 0.0795 | 0.0073 |
| Fat around heart | Fat and greaves C3 | 0.0027 | 0.0030 | 0.0003 |
| Fat in the kidney | Fat and greaves C3 | 0.0003 | 0.0004 | 0.0000 |
| Feet (without hooves) | Gelatin C3 | 0.0131 | 0.0196 | 0.0001 |
| Floatation fat | Spreading/Compost | 0.0000 | 0.0000 | 0.0000 |
| Forehead | C1-C2 for disposal | 0.0000 | 0.0000 | 0.0000 |
| Forelock | PAP C3 | 0.0031 | 0.0013 | 0.0001 |
| Gallbladder | Pet food | 0.0003 | 0.0005 | 0.0000 |
| Head trimmings | Pet food | 0.0028 | 0.0041 | 0.0003 |
| Heart | Human food | 0.0033 | 0.0050 | 0.0011 |
| Heart trimmings | Pet food | 0.0003 | 0.0005 | 0.0000 |
| Hide | Skin tannery C3 | 0.0734 | 0.0771 | 0.1304 |
| Hooves | PAP C3 | 0.0083 | 0.0034 | 0.0003 |
| Horns | PAP C3 | 0.0021 | 0.0009 | 0.0001 |
| Kidney | Human food | 0.0020 | 0.0030 | 0.0012 |
| Large intestine | C1-C2 for disposal | 0.0000 | 0.0000 | 0.0000 |
| Liver | Human food | 0.0597 | 0.0199 | 0.0098 |
| Liver trimmings | Pet food | 0.0070 | 0.0023 | 0.0002 |
| Lower jaw | PAP C3 | 0.0034 | 0.0055 | 0.0005 |
| Lungs | Pet food | 0.0088 | 0.0111 | 0.0008 |
| Mask | Skin tannery C3 | 0.0050 | 0.0053 | 0.0089 |
| Mesenteric fat | C1-C2 for disposal | 0.0000 | 0.0000 | 0.0000 |
| Muscle | Human food | 0.3040 | 0.4440 | 0.7520 |
| Muzzle | Human food | 0.0026 | 0.0028 | 0.0028 |
| Omasum | Human food | 0.0145 | 0.0025 | 0.0019 |
| Omasum fat | Fat and greaves C3 | 0.0035 | 0.0016 | 0.0001 |
| Rumen and forestomach | Human food | 0.0741 | 0.0128 | 0.0097 |
| Rumen fat | Fat and greaves C3 | 0.0054 | 0.0025 | 0.0002 |
| Sanitary seizures | C1-C2 for disposal | 0.0000 | 0.0000 | 0.0000 |
| Screening and sifting wastes | C1-C2 for disposal | 0.0000 | 0.0000 | 0.0000 |
| Small intestine | PAP C3 | 0.0865 | 0.0149 | 0.0009 |
| Spinal cord | C1-C2 for disposal | 0.0000 | 0.0000 | 0.0000 |
| Spinal cord waste | C1-C2 for disposal | 0.0000 | 0.0000 | 0.0000 |
| Spine | C1-C2 for disposal | 0.0000 | 0.0000 | 0.0000 |
| Spleen | Pet food | 0.0021 | 0.0030 | 0.0002 |
| Stillborn | PAP C3 | 0.0448 | 0.0074 | 0.0000 |
| Tallow | Fat and greaves C3 | 0.0688 | 0.0765 | 0.0071 |
| Tongue | Human food | 0.0026 | 0.0044 | 0.0071 |
| Tonsil | C1-C2 for disposal | 0.0000 | 0.0000 | 0.0000 |
| Trachea | Pet food | 0.0018 | 0.0019 | 0.0001 |
| Udder | Pet food | 0.0033 | 0.0234 | 0.0017 |
| Upper throat | Pet food | 0.0011 | 0.0016 | 0.0001 |
| Water in the rumen | Spreading/Compost | 0.0000 | 0.0000 | 0.0000 |

Table 5: Total weighting by coproducts for Normande Young Bulls reared in Pasture

| COPRODUCT | Destination | Normande/young bull/pasture | | |
| --- | --- | --- | --- | --- |
|  |  | **Biophysical Cumulative share** | **Mass Cumulative share** | **Economic Cumulative share** |
| Abomasum | Human food | 0.0144 | 0.0026 | 0.0020 |
| Abomasum fat | Fat and greaves C3 | 0.0011 | 0.0005 | 0.0000 |
| Aponeurosis | Human food | 0.0258 | 0.0341 | 0.0342 |
| Bile | PAP C3 | 0.0000 | 0.0001 | 0.0000 |
| Blood | PAP C3 | 0.0182 | 0.0311 | 0.0070 |
| Blood | Pet food | 0.0026 | 0.0045 | 0.0003 |
| Bones | Gelatin C3 | 0.0492 | 0.0764 | 0.0002 |
| Bones of head, brain, eyes and teeth | C1-C2 for disposal | 0.0000 | 0.0000 | 0.0000 |
| Cheek | Human food | 0.0011 | 0.0016 | 0.0035 |
| Cheek | Human food | 0.0020 | 0.0029 | 0.0063 |
| Cheek trimmings | Pet food | 0.0005 | 0.0007 | 0.0001 |
| Chops | Pet food | 0.0020 | 0.0021 | 0.0002 |
| Contents of intestines | Spreading/Compost | 0.0000 | 0.0000 | 0.0000 |
| Contents of the rumen | Spreading/Compost | 0.0000 | 0.0000 | 0.0000 |
| Ears | PAP C3 | 0.0009 | 0.0010 | 0.0001 |
| Esophagus | Pet food | 0.0010 | 0.0015 | 0.0001 |
| Fat | Fat and greaves C3 | 0.0838 | 0.0818 | 0.0074 |
| Fat around heart | Fat and greaves C3 | 0.0029 | 0.0029 | 0.0003 |
| Fat in the kidney | Fat and greaves C3 | 0.0004 | 0.0004 | 0.0000 |
| Feet (without hooves) | Gelatin C3 | 0.0127 | 0.0187 | 0.0001 |
| Floatation fat | Spreading/Compost | 0.0000 | 0.0000 | 0.0000 |
| Forehead | C1-C2 for disposal | 0.0000 | 0.0000 | 0.0000 |
| Forelock | PAP C3 | 0.0028 | 0.0011 | 0.0001 |
| Gallbladder | Pet food | 0.0003 | 0.0005 | 0.0000 |
| Head trimmings | Pet food | 0.0027 | 0.0040 | 0.0003 |
| Heart | Human food | 0.0031 | 0.0047 | 0.0010 |
| Heart trimmings | Pet food | 0.0003 | 0.0005 | 0.0000 |
| Hide | Skin tannery C3 | 0.0703 | 0.0733 | 0.1223 |
| Hooves | PAP C3 | 0.0080 | 0.0032 | 0.0003 |
| Horns | PAP C3 | 0.0018 | 0.0007 | 0.0001 |
| Kidney | Human food | 0.0019 | 0.0029 | 0.0012 |
| Large intestine | C1-C2 for disposal | 0.0000 | 0.0000 | 0.0000 |
| Liver | Human food | 0.0546 | 0.0190 | 0.0092 |
| Liver trimmings | Pet food | 0.0063 | 0.0021 | 0.0002 |
| Lower jaw | PAP C3 | 0.0033 | 0.0052 | 0.0004 |
| Lungs | Pet food | 0.0084 | 0.0105 | 0.0008 |
| Mask | Skin tannery C3 | 0.0048 | 0.0050 | 0.0083 |
| Mesenteric fat | C1-C2 for disposal | 0.0000 | 0.0000 | 0.0000 |
| Muscle | Human food | 0.3172 | 0.4569 | 0.7640 |
| Muzzle | Human food | 0.0025 | 0.0026 | 0.0026 |
| Omasum | Human food | 0.0130 | 0.0024 | 0.0018 |
| Omasum fat | Fat and greaves C3 | 0.0035 | 0.0016 | 0.0001 |
| Rumen and forestomach | Human food | 0.0672 | 0.0121 | 0.0091 |
| Rumen fat | Fat and greaves C3 | 0.0052 | 0.0024 | 0.0002 |
| Sanitary seizures | C1-C2 for disposal | 0.0000 | 0.0000 | 0.0000 |
| Screening and sifting wastes | C1-C2 for disposal | 0.0000 | 0.0000 | 0.0000 |
| Small intestine | PAP C3 | 0.0782 | 0.0141 | 0.0009 |
| Spinal cord | C1-C2 for disposal | 0.0000 | 0.0000 | 0.0000 |
| Spinal cord waste | C1-C2 for disposal | 0.0000 | 0.0000 | 0.0000 |
| Spine | C1-C2 for disposal | 0.0000 | 0.0000 | 0.0000 |
| Spleen | Pet food | 0.0020 | 0.0029 | 0.0002 |
| Stillborn | PAP C3 | 0.0408 | 0.0071 | 0.0000 |
| Tallow | Fat and greaves C3 | 0.0743 | 0.0727 | 0.0066 |
| Tongue | Human food | 0.0026 | 0.0042 | 0.0067 |
| Tonsil | C1-C2 for disposal | 0.0000 | 0.0000 | 0.0000 |
| Trachea | Pet food | 0.0018 | 0.0019 | 0.0001 |
| Udder | Pet food | 0.0033 | 0.0222 | 0.0016 |
| Upper throat | Pet food | 0.0011 | 0.0016 | 0.0001 |
| Water in the rumen | Spreading/Compost | 0.0000 | 0.0000 | 0.0000 |

Table 6: Total weighting by coproducts for Normande Heifers reared in Pasture

| COPRODUCT | Destination | Normande/heifer/pasture | | |
| --- | --- | --- | --- | --- |
|  |  | **Biophysical Cumulative share** | **Mass Cumulative share** | **Economic Cumulative share** |
| Abomasum | Human food | 0.0146 | 0.0028 | 0.0021 |
| Abomasum fat | Fat and greaves C3 | 0.0011 | 0.0005 | 0.0000 |
| Aponeurosis | Human food | 0.0245 | 0.0326 | 0.0334 |
| Bile | PAP C3 | 0.0000 | 0.0001 | 0.0000 |
| Blood | PAP C3 | 0.0195 | 0.0335 | 0.0076 |
| Blood | Pet food | 0.0028 | 0.0048 | 0.0004 |
| Bones | Gelatin C3 | 0.0467 | 0.0732 | 0.0002 |
| Bones of head, brain, eyes and teeth | C1-C2 for disposal | 0.0000 | 0.0000 | 0.0000 |
| Cheek | Human food | 0.0011 | 0.0016 | 0.0037 |
| Cheek | Human food | 0.0021 | 0.0031 | 0.0070 |
| Cheek trimmings | Pet food | 0.0006 | 0.0009 | 0.0001 |
| Chops | Pet food | 0.0022 | 0.0023 | 0.0002 |
| Contents of intestines | Spreading/Compost | 0.0000 | 0.0000 | 0.0000 |
| Contents of the rumen | Spreading/Compost | 0.0000 | 0.0000 | 0.0000 |
| Ears | PAP C3 | 0.0010 | 0.0011 | 0.0001 |
| Esophagus | Pet food | 0.0010 | 0.0015 | 0.0001 |
| Fat | Fat and greaves C3 | 0.0789 | 0.0783 | 0.0073 |
| Fat around heart | Fat and greaves C3 | 0.0032 | 0.0031 | 0.0003 |
| Fat in the kidney | Fat and greaves C3 | 0.0004 | 0.0004 | 0.0000 |
| Feet (without hooves) | Gelatin C3 | 0.0136 | 0.0202 | 0.0001 |
| Floatation fat | Spreading/Compost | 0.0000 | 0.0000 | 0.0000 |
| Forehead | C1-C2 for disposal | 0.0000 | 0.0000 | 0.0000 |
| Forelock | PAP C3 | 0.0031 | 0.0013 | 0.0001 |
| Gallbladder | Pet food | 0.0003 | 0.0005 | 0.0000 |
| Head trimmings | Pet food | 0.0029 | 0.0043 | 0.0003 |
| Heart | Human food | 0.0033 | 0.0050 | 0.0011 |
| Heart trimmings | Pet food | 0.0003 | 0.0005 | 0.0000 |
| Hide | Skin tannery C3 | 0.0754 | 0.0791 | 0.1346 |
| Hooves | PAP C3 | 0.0086 | 0.0035 | 0.0003 |
| Horns | PAP C3 | 0.0022 | 0.0009 | 0.0001 |
| Kidney | Human food | 0.0021 | 0.0031 | 0.0013 |
| Large intestine | C1-C2 for disposal | 0.0000 | 0.0000 | 0.0000 |
| Liver | Human food | 0.0566 | 0.0205 | 0.0101 |
| Liver trimmings | Pet food | 0.0065 | 0.0023 | 0.0002 |
| Lower jaw | PAP C3 | 0.0035 | 0.0056 | 0.0005 |
| Lungs | Pet food | 0.0090 | 0.0114 | 0.0009 |
| Mask | Skin tannery C3 | 0.0051 | 0.0054 | 0.0092 |
| Mesenteric fat | C1-C2 for disposal | 0.0000 | 0.0000 | 0.0000 |
| Muscle | Human food | 0.3014 | 0.4374 | 0.7457 |
| Muzzle | Human food | 0.0026 | 0.0028 | 0.0028 |
| Omasum | Human food | 0.0133 | 0.0025 | 0.0019 |
| Omasum fat | Fat and greaves C3 | 0.0034 | 0.0016 | 0.0002 |
| Rumen and forestomach | Human food | 0.0689 | 0.0131 | 0.0100 |
| Rumen fat | Fat and greaves C3 | 0.0053 | 0.0025 | 0.0002 |
| Sanitary seizures | C1-C2 for disposal | 0.0000 | 0.0000 | 0.0000 |
| Screening and sifting wastes | C1-C2 for disposal | 0.0000 | 0.0000 | 0.0000 |
| Small intestine | PAP C3 | 0.0802 | 0.0152 | 0.0009 |
| Spinal cord | C1-C2 for disposal | 0.0000 | 0.0000 | 0.0000 |
| Spinal cord waste | C1-C2 for disposal | 0.0000 | 0.0000 | 0.0000 |
| Spine | C1-C2 for disposal | 0.0000 | 0.0000 | 0.0000 |
| Spleen | Pet food | 0.0022 | 0.0031 | 0.0002 |
| Stillborn | PAP C3 | 0.0422 | 0.0077 | 0.0000 |
| Tallow | Fat and greaves C3 | 0.0789 | 0.0785 | 0.0073 |
| Tongue | Human food | 0.0027 | 0.0045 | 0.0073 |
| Tonsil | C1-C2 for disposal | 0.0000 | 0.0000 | 0.0000 |
| Trachea | Pet food | 0.0019 | 0.0020 | 0.0002 |
| Udder | Pet food | 0.0035 | 0.0240 | 0.0018 |
| Upper throat | Pet food | 0.0011 | 0.0016 | 0.0001 |
| Water in the rumen | Spreading/Compost | 0.0000 | 0.0000 | 0.0000 |

Table 7: Total weighting by coproducts for Normande Cull Cows reared in Pasture

| COPRODUCT | Destination | Normande/Cull cow/pasture | | |
| --- | --- | --- | --- | --- |
|  |  | **Biophysical Cumulative share** | **Mass Cumulative share** | **Economic Cumulative share** |
| Abomasum | Human food | 0.0156 | 0.0029 | 0.0023 |
| Abomasum fat | Fat and greaves C3 | 0.0013 | 0.0006 | 0.0001 |
| Aponeurosis | Human food | 0.0232 | 0.0316 | 0.0328 |
| Bile | PAP C3 | 0.0000 | 0.0001 | 0.0000 |
| Blood | PAP C3 | 0.0199 | 0.0352 | 0.0081 |
| Blood | Pet food | 0.0029 | 0.0051 | 0.0004 |
| Bones | Gelatin C3 | 0.0440 | 0.0709 | 0.0002 |
| Bones of head, brain, eyes and teeth | C1-C2 for disposal | 0.0000 | 0.0000 | 0.0000 |
| Cheek | Human food | 0.0012 | 0.0018 | 0.0040 |
| Cheek | Human food | 0.0022 | 0.0033 | 0.0075 |
| Cheek trimmings | Pet food | 0.0006 | 0.0009 | 0.0001 |
| Chops | Pet food | 0.0022 | 0.0024 | 0.0002 |
| Contents of intestines | Spreading/Compost | 0.0000 | 0.0000 | 0.0000 |
| Contents of the rumen | Spreading/Compost | 0.0000 | 0.0000 | 0.0000 |
| Ears | PAP C3 | 0.0009 | 0.0011 | 0.0001 |
| Esophagus | Pet food | 0.0011 | 0.0016 | 0.0001 |
| Fat | Fat and greaves C3 | 0.0741 | 0.0759 | 0.0071 |
| Fat around heart | Fat and greaves C3 | 0.0032 | 0.0033 | 0.0003 |
| Fat in the kidney | Fat and greaves C3 | 0.0005 | 0.0005 | 0.0000 |
| Feet (without hooves) | Gelatin C3 | 0.0139 | 0.0211 | 0.0001 |
| Floatation fat | Spreading/Compost | 0.0000 | 0.0000 | 0.0000 |
| Forehead | C1-C2 for disposal | 0.0000 | 0.0000 | 0.0000 |
| Forelock | PAP C3 | 0.0033 | 0.0014 | 0.0001 |
| Gallbladder | Pet food | 0.0004 | 0.0006 | 0.0000 |
| Head trimmings | Pet food | 0.0030 | 0.0044 | 0.0003 |
| Heart | Human food | 0.0034 | 0.0053 | 0.0012 |
| Heart trimmings | Pet food | 0.0004 | 0.0006 | 0.0000 |
| Hide | Skin tannery C3 | 0.0772 | 0.0830 | 0.1432 |
| Hooves | PAP C3 | 0.0088 | 0.0037 | 0.0003 |
| Horns | PAP C3 | 0.0021 | 0.0009 | 0.0001 |
| Kidney | Human food | 0.0022 | 0.0033 | 0.0014 |
| Large intestine | C1-C2 for disposal | 0.0000 | 0.0000 | 0.0000 |
| Liver | Human food | 0.0601 | 0.0215 | 0.0108 |
| Liver trimmings | Pet food | 0.0070 | 0.0024 | 0.0002 |
| Lower jaw | PAP C3 | 0.0036 | 0.0059 | 0.0005 |
| Lungs | Pet food | 0.0092 | 0.0120 | 0.0009 |
| Mask | Skin tannery C3 | 0.0052 | 0.0056 | 0.0096 |
| Mesenteric fat | C1-C2 for disposal | 0.0000 | 0.0000 | 0.0000 |
| Muscle | Human food | 0.2848 | 0.4239 | 0.7327 |
| Muzzle | Human food | 0.0027 | 0.0029 | 0.0030 |
| Omasum | Human food | 0.0143 | 0.0027 | 0.0021 |
| Omasum fat | Fat and greaves C3 | 0.0037 | 0.0018 | 0.0002 |
| Rumen and forestomach | Human food | 0.0740 | 0.0138 | 0.0107 |
| Rumen fat | Fat and greaves C3 | 0.0056 | 0.0027 | 0.0003 |
| Sanitary seizures | C1-C2 for disposal | 0.0000 | 0.0000 | 0.0000 |
| Screening and sifting wastes | C1-C2 for disposal | 0.0000 | 0.0000 | 0.0000 |
| Small intestine | PAP C3 | 0.0855 | 0.0159 | 0.0010 |
| Spinal cord | C1-C2 for disposal | 0.0000 | 0.0000 | 0.0000 |
| Spinal cord waste | C1-C2 for disposal | 0.0000 | 0.0000 | 0.0000 |
| Spine | C1-C2 for disposal | 0.0000 | 0.0000 | 0.0000 |
| Spleen | Pet food | 0.0022 | 0.0033 | 0.0002 |
| Stillborn | PAP C3 | 0.0446 | 0.0080 | 0.0000 |
| Tallow | Fat and greaves C3 | 0.0803 | 0.0824 | 0.0078 |
| Tongue | Human food | 0.0028 | 0.0047 | 0.0077 |
| Tonsil | C1-C2 for disposal | 0.0000 | 0.0000 | 0.0000 |
| Trachea | Pet food | 0.0019 | 0.0020 | 0.0002 |
| Udder | Pet food | 0.0036 | 0.0252 | 0.0019 |
| Upper throat | Pet food | 0.0012 | 0.0018 | 0.0001 |
| Water in the rumen | Spreading/Compost | 0.0000 | 0.0000 | 0.0000 |

Table 8: Total weighting by coproducts for Normande Beef reared in Pasture

| COPRODUCT | Destination | Normande/beef/pasture | | |
| --- | --- | --- | --- | --- |
|  |  | **Biophysical Cumulative share** | **Mass Cumulative share** | **Economic Cumulative share** |
| Abomasum | Human food | 0.0153 | 0.0028 | 0.0021 |
| Abomasum fat | Fat and greaves C3 | 0.0011 | 0.0005 | 0.0000 |
| Aponeurosis | Human food | 0.0247 | 0.0331 | 0.0337 |
| Bile | PAP C3 | 0.0000 | 0.0001 | 0.0000 |
| Blood | PAP C3 | 0.0188 | 0.0328 | 0.0074 |
| Blood | Pet food | 0.0027 | 0.0046 | 0.0003 |
| Bones | Gelatin C3 | 0.0468 | 0.0743 | 0.0002 |
| Bones of head, brain, eyes and teeth | C1-C2 for disposal | 0.0000 | 0.0000 | 0.0000 |
| Cheek | Human food | 0.0011 | 0.0016 | 0.0036 |
| Cheek | Human food | 0.0020 | 0.0030 | 0.0067 |
| Cheek trimmings | Pet food | 0.0006 | 0.0009 | 0.0001 |
| Chops | Pet food | 0.0021 | 0.0023 | 0.0002 |
| Contents of intestines | Spreading/Compost | 0.0000 | 0.0000 | 0.0000 |
| Contents of the rumen | Spreading/Compost | 0.0000 | 0.0000 | 0.0000 |
| Ears | PAP C3 | 0.0009 | 0.0011 | 0.0001 |
| Esophagus | Pet food | 0.0010 | 0.0015 | 0.0001 |
| Fat | Fat and greaves C3 | 0.0788 | 0.0795 | 0.0073 |
| Fat around heart | Fat and greaves C3 | 0.0030 | 0.0030 | 0.0003 |
| Fat in the kidney | Fat and greaves C3 | 0.0004 | 0.0004 | 0.0000 |
| Feet (without hooves) | Gelatin C3 | 0.0131 | 0.0196 | 0.0001 |
| Floatation fat | Spreading/Compost | 0.0000 | 0.0000 | 0.0000 |
| Forehead | C1-C2 for disposal | 0.0000 | 0.0000 | 0.0000 |
| Forelock | PAP C3 | 0.0030 | 0.0013 | 0.0001 |
| Gallbladder | Pet food | 0.0003 | 0.0005 | 0.0000 |
| Head trimmings | Pet food | 0.0028 | 0.0041 | 0.0003 |
| Heart | Human food | 0.0032 | 0.0050 | 0.0011 |
| Heart trimmings | Pet food | 0.0003 | 0.0005 | 0.0000 |
| Hide | Skin tannery C3 | 0.0727 | 0.0771 | 0.1304 |
| Hooves | PAP C3 | 0.0082 | 0.0034 | 0.0003 |
| Horns | PAP C3 | 0.0021 | 0.0009 | 0.0001 |
| Kidney | Human food | 0.0020 | 0.0030 | 0.0012 |
| Large intestine | C1-C2 for disposal | 0.0000 | 0.0000 | 0.0000 |
| Liver | Human food | 0.0575 | 0.0199 | 0.0098 |
| Liver trimmings | Pet food | 0.0068 | 0.0023 | 0.0002 |
| Lower jaw | PAP C3 | 0.0034 | 0.0055 | 0.0005 |
| Lungs | Pet food | 0.0087 | 0.0111 | 0.0008 |
| Mask | Skin tannery C3 | 0.0050 | 0.0053 | 0.0089 |
| Mesenteric fat | C1-C2 for disposal | 0.0000 | 0.0000 | 0.0000 |
| Muscle | Human food | 0.3028 | 0.4440 | 0.7520 |
| Muzzle | Human food | 0.0026 | 0.0028 | 0.0028 |
| Omasum | Human food | 0.0139 | 0.0025 | 0.0019 |
| Omasum fat | Fat and greaves C3 | 0.0035 | 0.0016 | 0.0001 |
| Rumen and forestomach | Human food | 0.0710 | 0.0128 | 0.0097 |
| Rumen fat | Fat and greaves C3 | 0.0054 | 0.0025 | 0.0002 |
| Sanitary seizures | C1-C2 for disposal | 0.0000 | 0.0000 | 0.0000 |
| Screening and sifting wastes | C1-C2 for disposal | 0.0000 | 0.0000 | 0.0000 |
| Small intestine | PAP C3 | 0.0828 | 0.0149 | 0.0009 |
| Spinal cord | C1-C2 for disposal | 0.0000 | 0.0000 | 0.0000 |
| Spinal cord waste | C1-C2 for disposal | 0.0000 | 0.0000 | 0.0000 |
| Spine | C1-C2 for disposal | 0.0000 | 0.0000 | 0.0000 |
| Spleen | Pet food | 0.0021 | 0.0030 | 0.0002 |
| Stillborn | PAP C3 | 0.0429 | 0.0074 | 0.0000 |
| Tallow | Fat and greaves C3 | 0.0757 | 0.0765 | 0.0071 |
| Tongue | Human food | 0.0026 | 0.0044 | 0.0071 |
| Tonsil | C1-C2 for disposal | 0.0000 | 0.0000 | 0.0000 |
| Trachea | Pet food | 0.0018 | 0.0019 | 0.0001 |
| Udder | Pet food | 0.0034 | 0.0234 | 0.0017 |
| Upper throat | Pet food | 0.0011 | 0.0016 | 0.0001 |
| Water in the rumen | Spreading/Compost | 0.0000 | 0.0000 | 0.0000 |

Table 9: Total weighting by coproducts for Normande Young Bulls reared in Stall

| COPRODUCT | Destination | Normande/young bull/stall | | |
| --- | --- | --- | --- | --- |
|  |  | **Biophysical Cumulative share** | **Mass Cumulative share** | **Economic Cumulative share** |
| Abomasum | Human food | 0.0137 | 0.0026 | 0.0020 |
| Abomasum fat | Fat and greaves C3 | 0.0011 | 0.0005 | 0.0000 |
| Aponeurosis | Human food | 0.0256 | 0.0341 | 0.0342 |
| Bile | PAP C3 | 0.0000 | 0.0001 | 0.0000 |
| Blood | PAP C3 | 0.0180 | 0.0311 | 0.0070 |
| Blood | Pet food | 0.0026 | 0.0045 | 0.0003 |
| Bones | Gelatin C3 | 0.0499 | 0.0764 | 0.0002 |
| Bones of head, brain, eyes and teeth | C1-C2 for disposal | 0.0000 | 0.0000 | 0.0000 |
| Cheek | Human food | 0.0011 | 0.0016 | 0.0035 |
| Cheek | Human food | 0.0020 | 0.0029 | 0.0063 |
| Cheek trimmings | Pet food | 0.0005 | 0.0007 | 0.0001 |
| Chops | Pet food | 0.0020 | 0.0021 | 0.0002 |
| Contents of intestines | Spreading/Compost | 0.0000 | 0.0000 | 0.0000 |
| Contents of the rumen | Spreading/Compost | 0.0000 | 0.0000 | 0.0000 |
| Ears | PAP C3 | 0.0009 | 0.0010 | 0.0001 |
| Esophagus | Pet food | 0.0010 | 0.0015 | 0.0001 |
| Fat | Fat and greaves C3 | 0.0919 | 0.0818 | 0.0074 |
| Fat around heart | Fat and greaves C3 | 0.0032 | 0.0029 | 0.0003 |
| Fat in the kidney | Fat and greaves C3 | 0.0004 | 0.0004 | 0.0000 |
| Feet (without hooves) | Gelatin C3 | 0.0127 | 0.0187 | 0.0001 |
| Floatation fat | Spreading/Compost | 0.0000 | 0.0000 | 0.0000 |
| Forehead | C1-C2 for disposal | 0.0000 | 0.0000 | 0.0000 |
| Forelock | PAP C3 | 0.0027 | 0.0011 | 0.0001 |
| Gallbladder | Pet food | 0.0003 | 0.0005 | 0.0000 |
| Head trimmings | Pet food | 0.0027 | 0.0040 | 0.0003 |
| Heart | Human food | 0.0031 | 0.0047 | 0.0010 |
| Heart trimmings | Pet food | 0.0003 | 0.0005 | 0.0000 |
| Hide | Skin tannery C3 | 0.0696 | 0.0733 | 0.1223 |
| Hooves | PAP C3 | 0.0079 | 0.0032 | 0.0003 |
| Horns | PAP C3 | 0.0018 | 0.0007 | 0.0001 |
| Kidney | Human food | 0.0019 | 0.0029 | 0.0012 |
| Large intestine | C1-C2 for disposal | 0.0000 | 0.0000 | 0.0000 |
| Liver | Human food | 0.0522 | 0.0190 | 0.0092 |
| Liver trimmings | Pet food | 0.0060 | 0.0021 | 0.0002 |
| Lower jaw | PAP C3 | 0.0034 | 0.0052 | 0.0004 |
| Lungs | Pet food | 0.0083 | 0.0105 | 0.0008 |
| Mask | Skin tannery C3 | 0.0047 | 0.0050 | 0.0083 |
| Mesenteric fat | C1-C2 for disposal | 0.0000 | 0.0000 | 0.0000 |
| Muscle | Human food | 0.3159 | 0.4569 | 0.7640 |
| Muzzle | Human food | 0.0025 | 0.0026 | 0.0026 |
| Omasum | Human food | 0.0124 | 0.0024 | 0.0018 |
| Omasum fat | Fat and greaves C3 | 0.0035 | 0.0016 | 0.0001 |
| Rumen and forestomach | Human food | 0.0638 | 0.0121 | 0.0091 |
| Rumen fat | Fat and greaves C3 | 0.0052 | 0.0024 | 0.0002 |
| Sanitary seizures | C1-C2 for disposal | 0.0000 | 0.0000 | 0.0000 |
| Screening and sifting wastes | C1-C2 for disposal | 0.0000 | 0.0000 | 0.0000 |
| Small intestine | PAP C3 | 0.0742 | 0.0141 | 0.0009 |
| Spinal cord | C1-C2 for disposal | 0.0000 | 0.0000 | 0.0000 |
| Spinal cord waste | C1-C2 for disposal | 0.0000 | 0.0000 | 0.0000 |
| Spine | C1-C2 for disposal | 0.0000 | 0.0000 | 0.0000 |
| Spleen | Pet food | 0.0020 | 0.0029 | 0.0002 |
| Stillborn | PAP C3 | 0.0387 | 0.0071 | 0.0000 |
| Tallow | Fat and greaves C3 | 0.0815 | 0.0727 | 0.0066 |
| Tongue | Human food | 0.0026 | 0.0042 | 0.0067 |
| Tonsil | C1-C2 for disposal | 0.0000 | 0.0000 | 0.0000 |
| Trachea | Pet food | 0.0018 | 0.0019 | 0.0001 |
| Udder | Pet food | 0.0034 | 0.0222 | 0.0016 |
| Upper throat | Pet food | 0.0011 | 0.0016 | 0.0001 |
| Water in the rumen | Spreading/Compost | 0.0000 | 0.0000 | 0.0000 |

Table 10: Total weighting by coproducts for Normande Heifers reared in Stall

| COPRODUCT | Destination | Normande/heifer/stall | | |
| --- | --- | --- | --- | --- |
|  |  | **Biophysical Cumulative share** | **Mass Cumulative share** | **Economic Cumulative share** |
| Abomasum | Human food | 0.0138 | 0.0028 | 0.0021 |
| Abomasum fat | Fat and greaves C3 | 0.0011 | 0.0005 | 0.0000 |
| Aponeurosis | Human food | 0.0244 | 0.0326 | 0.0334 |
| Bile | PAP C3 | 0.0000 | 0.0001 | 0.0000 |
| Blood | PAP C3 | 0.0194 | 0.0335 | 0.0076 |
| Blood | Pet food | 0.0028 | 0.0048 | 0.0004 |
| Bones | Gelatin C3 | 0.0475 | 0.0732 | 0.0002 |
| Bones of head, brain, eyes and teeth | C1-C2 for disposal | 0.0000 | 0.0000 | 0.0000 |
| Cheek | Human food | 0.0011 | 0.0016 | 0.0037 |
| Cheek | Human food | 0.0021 | 0.0031 | 0.0070 |
| Cheek trimmings | Pet food | 0.0006 | 0.0009 | 0.0001 |
| Chops | Pet food | 0.0021 | 0.0023 | 0.0002 |
| Contents of intestines | Spreading/Compost | 0.0000 | 0.0000 | 0.0000 |
| Contents of the rumen | Spreading/Compost | 0.0000 | 0.0000 | 0.0000 |
| Ears | PAP C3 | 0.0010 | 0.0011 | 0.0001 |
| Esophagus | Pet food | 0.0010 | 0.0015 | 0.0001 |
| Fat | Fat and greaves C3 | 0.0863 | 0.0783 | 0.0073 |
| Fat around heart | Fat and greaves C3 | 0.0035 | 0.0031 | 0.0003 |
| Fat in the kidney | Fat and greaves C3 | 0.0004 | 0.0004 | 0.0000 |
| Feet (without hooves) | Gelatin C3 | 0.0136 | 0.0202 | 0.0001 |
| Floatation fat | Spreading/Compost | 0.0000 | 0.0000 | 0.0000 |
| Forehead | C1-C2 for disposal | 0.0000 | 0.0000 | 0.0000 |
| Forelock | PAP C3 | 0.0031 | 0.0013 | 0.0001 |
| Gallbladder | Pet food | 0.0003 | 0.0005 | 0.0000 |
| Head trimmings | Pet food | 0.0029 | 0.0043 | 0.0003 |
| Heart | Human food | 0.0033 | 0.0050 | 0.0011 |
| Heart trimmings | Pet food | 0.0003 | 0.0005 | 0.0000 |
| Hide | Skin tannery C3 | 0.0749 | 0.0791 | 0.1346 |
| Hooves | PAP C3 | 0.0085 | 0.0035 | 0.0003 |
| Horns | PAP C3 | 0.0021 | 0.0009 | 0.0001 |
| Kidney | Human food | 0.0021 | 0.0031 | 0.0013 |
| Large intestine | C1-C2 for disposal | 0.0000 | 0.0000 | 0.0000 |
| Liver | Human food | 0.0540 | 0.0205 | 0.0101 |
| Liver trimmings | Pet food | 0.0062 | 0.0023 | 0.0002 |
| Lower jaw | PAP C3 | 0.0036 | 0.0056 | 0.0005 |
| Lungs | Pet food | 0.0089 | 0.0114 | 0.0009 |
| Mask | Skin tannery C3 | 0.0051 | 0.0054 | 0.0092 |
| Mesenteric fat | C1-C2 for disposal | 0.0000 | 0.0000 | 0.0000 |
| Muscle | Human food | 0.3007 | 0.4374 | 0.7457 |
| Muzzle | Human food | 0.0026 | 0.0028 | 0.0028 |
| Omasum | Human food | 0.0126 | 0.0025 | 0.0019 |
| Omasum fat | Fat and greaves C3 | 0.0035 | 0.0016 | 0.0002 |
| Rumen and forestomach | Human food | 0.0654 | 0.0131 | 0.0100 |
| Rumen fat | Fat and greaves C3 | 0.0053 | 0.0025 | 0.0002 |
| Sanitary seizures | C1-C2 for disposal | 0.0000 | 0.0000 | 0.0000 |
| Screening and sifting wastes | C1-C2 for disposal | 0.0000 | 0.0000 | 0.0000 |
| Small intestine | PAP C3 | 0.0760 | 0.0152 | 0.0009 |
| Spinal cord | C1-C2 for disposal | 0.0000 | 0.0000 | 0.0000 |
| Spinal cord waste | C1-C2 for disposal | 0.0000 | 0.0000 | 0.0000 |
| Spine | C1-C2 for disposal | 0.0000 | 0.0000 | 0.0000 |
| Spleen | Pet food | 0.0022 | 0.0031 | 0.0002 |
| Stillborn | PAP C3 | 0.0400 | 0.0077 | 0.0000 |
| Tallow | Fat and greaves C3 | 0.0863 | 0.0785 | 0.0073 |
| Tongue | Human food | 0.0028 | 0.0045 | 0.0073 |
| Tonsil | C1-C2 for disposal | 0.0000 | 0.0000 | 0.0000 |
| Trachea | Pet food | 0.0019 | 0.0020 | 0.0002 |
| Udder | Pet food | 0.0036 | 0.0240 | 0.0018 |
| Upper throat | Pet food | 0.0011 | 0.0016 | 0.0001 |
| Water in the rumen | Spreading/Compost | 0.0000 | 0.0000 | 0.0000 |

Table 11: Total weighting by coproducts for Normande Cull Cows reared in Stall

| COPRODUCT | Destination | Normande/Cull cow/stall | | |
| --- | --- | --- | --- | --- |
|  |  | **Biophysical Cumulative share** | **Mass Cumulative share** | **Economic Cumulative share** |
| Abomasum | Human food | 0.0149 | 0.0029 | 0.0023 |
| Abomasum fat | Fat and greaves C3 | 0.0013 | 0.0006 | 0.0001 |
| Aponeurosis | Human food | 0.0230 | 0.0316 | 0.0328 |
| Bile | PAP C3 | 0.0000 | 0.0001 | 0.0000 |
| Blood | PAP C3 | 0.0198 | 0.0352 | 0.0081 |
| Blood | Pet food | 0.0028 | 0.0051 | 0.0004 |
| Bones | Gelatin C3 | 0.0448 | 0.0709 | 0.0002 |
| Bones of head, brain, eyes and teeth | C1-C2 for disposal | 0.0000 | 0.0000 | 0.0000 |
| Cheek | Human food | 0.0012 | 0.0018 | 0.0040 |
| Cheek | Human food | 0.0022 | 0.0033 | 0.0075 |
| Cheek trimmings | Pet food | 0.0006 | 0.0009 | 0.0001 |
| Chops | Pet food | 0.0022 | 0.0024 | 0.0002 |
| Contents of intestines | Spreading/Compost | 0.0000 | 0.0000 | 0.0000 |
| Contents of the rumen | Spreading/Compost | 0.0000 | 0.0000 | 0.0000 |
| Ears | PAP C3 | 0.0009 | 0.0011 | 0.0001 |
| Esophagus | Pet food | 0.0011 | 0.0016 | 0.0001 |
| Fat | Fat and greaves C3 | 0.0814 | 0.0759 | 0.0071 |
| Fat around heart | Fat and greaves C3 | 0.0035 | 0.0033 | 0.0003 |
| Fat in the kidney | Fat and greaves C3 | 0.0005 | 0.0005 | 0.0000 |
| Feet (without hooves) | Gelatin C3 | 0.0139 | 0.0211 | 0.0001 |
| Floatation fat | Spreading/Compost | 0.0000 | 0.0000 | 0.0000 |
| Forehead | C1-C2 for disposal | 0.0000 | 0.0000 | 0.0000 |
| Forelock | PAP C3 | 0.0033 | 0.0014 | 0.0001 |
| Gallbladder | Pet food | 0.0004 | 0.0006 | 0.0000 |
| Head trimmings | Pet food | 0.0029 | 0.0044 | 0.0003 |
| Heart | Human food | 0.0034 | 0.0053 | 0.0012 |
| Heart trimmings | Pet food | 0.0004 | 0.0006 | 0.0000 |
| Hide | Skin tannery C3 | 0.0765 | 0.0830 | 0.1432 |
| Hooves | PAP C3 | 0.0087 | 0.0037 | 0.0003 |
| Horns | PAP C3 | 0.0021 | 0.0009 | 0.0001 |
| Kidney | Human food | 0.0021 | 0.0033 | 0.0014 |
| Large intestine | C1-C2 for disposal | 0.0000 | 0.0000 | 0.0000 |
| Liver | Human food | 0.0575 | 0.0215 | 0.0108 |
| Liver trimmings | Pet food | 0.0067 | 0.0024 | 0.0002 |
| Lower jaw | PAP C3 | 0.0037 | 0.0059 | 0.0005 |
| Lungs | Pet food | 0.0091 | 0.0120 | 0.0009 |
| Mask | Skin tannery C3 | 0.0051 | 0.0056 | 0.0096 |
| Mesenteric fat | C1-C2 for disposal | 0.0000 | 0.0000 | 0.0000 |
| Muscle | Human food | 0.2840 | 0.4239 | 0.7327 |
| Muzzle | Human food | 0.0027 | 0.0029 | 0.0030 |
| Omasum | Human food | 0.0136 | 0.0027 | 0.0021 |
| Omasum fat | Fat and greaves C3 | 0.0038 | 0.0018 | 0.0002 |
| Rumen and forestomach | Human food | 0.0704 | 0.0138 | 0.0107 |
| Rumen fat | Fat and greaves C3 | 0.0056 | 0.0027 | 0.0003 |
| Sanitary seizures | C1-C2 for disposal | 0.0000 | 0.0000 | 0.0000 |
| Screening and sifting wastes | C1-C2 for disposal | 0.0000 | 0.0000 | 0.0000 |
| Small intestine | PAP C3 | 0.0814 | 0.0159 | 0.0010 |
| Spinal cord | C1-C2 for disposal | 0.0000 | 0.0000 | 0.0000 |
| Spinal cord waste | C1-C2 for disposal | 0.0000 | 0.0000 | 0.0000 |
| Spine | C1-C2 for disposal | 0.0000 | 0.0000 | 0.0000 |
| Spleen | Pet food | 0.0022 | 0.0033 | 0.0002 |
| Stillborn | PAP C3 | 0.0424 | 0.0080 | 0.0000 |
| Tallow | Fat and greaves C3 | 0.0882 | 0.0824 | 0.0078 |
| Tongue | Human food | 0.0028 | 0.0047 | 0.0077 |
| Tonsil | C1-C2 for disposal | 0.0000 | 0.0000 | 0.0000 |
| Trachea | Pet food | 0.0019 | 0.0020 | 0.0002 |
| Udder | Pet food | 0.0037 | 0.0252 | 0.0019 |
| Upper throat | Pet food | 0.0012 | 0.0018 | 0.0001 |
| Water in the rumen | Spreading/Compost | 0.0000 | 0.0000 | 0.0000 |

Table 12: Total weighting by coproducts for Normande Beef reared in Stall

| COPRODUCT | Destination | Normande/beef/stall | | |
| --- | --- | --- | --- | --- |
|  |  | **Biophysical Cumulative share** | **Mass Cumulative share** | **Economic Cumulative share** |
| Abomasum | Human food | 0.0146 | 0.0028 | 0.0021 |
| Abomasum fat | Fat and greaves C3 | 0.0011 | 0.0005 | 0.0000 |
| Aponeurosis | Human food | 0.0244 | 0.0331 | 0.0337 |
| Bile | PAP C3 | 0.0000 | 0.0001 | 0.0000 |
| Blood | PAP C3 | 0.0187 | 0.0328 | 0.0074 |
| Blood | Pet food | 0.0026 | 0.0046 | 0.0003 |
| Bones | Gelatin C3 | 0.0476 | 0.0743 | 0.0002 |
| Bones of head, brain, eyes and teeth | C1-C2 for disposal | 0.0000 | 0.0000 | 0.0000 |
| Cheek | Human food | 0.0011 | 0.0016 | 0.0036 |
| Cheek | Human food | 0.0020 | 0.0030 | 0.0067 |
| Cheek trimmings | Pet food | 0.0006 | 0.0009 | 0.0001 |
| Chops | Pet food | 0.0021 | 0.0023 | 0.0002 |
| Contents of intestines | Spreading/Compost | 0.0000 | 0.0000 | 0.0000 |
| Contents of the rumen | Spreading/Compost | 0.0000 | 0.0000 | 0.0000 |
| Ears | PAP C3 | 0.0010 | 0.0011 | 0.0001 |
| Esophagus | Pet food | 0.0010 | 0.0015 | 0.0001 |
| Fat | Fat and greaves C3 | 0.0866 | 0.0795 | 0.0073 |
| Fat around heart | Fat and greaves C3 | 0.0033 | 0.0030 | 0.0003 |
| Fat in the kidney | Fat and greaves C3 | 0.0004 | 0.0004 | 0.0000 |
| Feet (without hooves) | Gelatin C3 | 0.0131 | 0.0196 | 0.0001 |
| Floatation fat | Spreading/Compost | 0.0000 | 0.0000 | 0.0000 |
| Forehead | C1-C2 for disposal | 0.0000 | 0.0000 | 0.0000 |
| Forelock | PAP C3 | 0.0030 | 0.0013 | 0.0001 |
| Gallbladder | Pet food | 0.0003 | 0.0005 | 0.0000 |
| Head trimmings | Pet food | 0.0028 | 0.0041 | 0.0003 |
| Heart | Human food | 0.0032 | 0.0050 | 0.0011 |
| Heart trimmings | Pet food | 0.0003 | 0.0005 | 0.0000 |
| Hide | Skin tannery C3 | 0.0720 | 0.0771 | 0.1304 |
| Hooves | PAP C3 | 0.0081 | 0.0034 | 0.0003 |
| Horns | PAP C3 | 0.0021 | 0.0009 | 0.0001 |
| Kidney | Human food | 0.0020 | 0.0030 | 0.0012 |
| Large intestine | C1-C2 for disposal | 0.0000 | 0.0000 | 0.0000 |
| Liver | Human food | 0.0550 | 0.0199 | 0.0098 |
| Liver trimmings | Pet food | 0.0065 | 0.0023 | 0.0002 |
| Lower jaw | PAP C3 | 0.0035 | 0.0055 | 0.0005 |
| Lungs | Pet food | 0.0086 | 0.0111 | 0.0008 |
| Mask | Skin tannery C3 | 0.0049 | 0.0053 | 0.0089 |
| Mesenteric fat | C1-C2 for disposal | 0.0000 | 0.0000 | 0.0000 |
| Muscle | Human food | 0.3014 | 0.4440 | 0.7520 |
| Muzzle | Human food | 0.0026 | 0.0028 | 0.0028 |
| Omasum | Human food | 0.0133 | 0.0025 | 0.0019 |
| Omasum fat | Fat and greaves C3 | 0.0036 | 0.0016 | 0.0001 |
| Rumen and forestomach | Human food | 0.0676 | 0.0128 | 0.0097 |
| Rumen fat | Fat and greaves C3 | 0.0055 | 0.0025 | 0.0002 |
| Sanitary seizures | C1-C2 for disposal | 0.0000 | 0.0000 | 0.0000 |
| Screening and sifting wastes | C1-C2 for disposal | 0.0000 | 0.0000 | 0.0000 |
| Small intestine | PAP C3 | 0.0789 | 0.0149 | 0.0009 |
| Spinal cord | C1-C2 for disposal | 0.0000 | 0.0000 | 0.0000 |
| Spinal cord waste | C1-C2 for disposal | 0.0000 | 0.0000 | 0.0000 |
| Spine | C1-C2 for disposal | 0.0000 | 0.0000 | 0.0000 |
| Spleen | Pet food | 0.0021 | 0.0030 | 0.0002 |
| Stillborn | PAP C3 | 0.0408 | 0.0074 | 0.0000 |
| Tallow | Fat and greaves C3 | 0.0832 | 0.0765 | 0.0071 |
| Tongue | Human food | 0.0026 | 0.0044 | 0.0071 |
| Tonsil | C1-C2 for disposal | 0.0000 | 0.0000 | 0.0000 |
| Trachea | Pet food | 0.0018 | 0.0019 | 0.0001 |
| Udder | Pet food | 0.0035 | 0.0234 | 0.0017 |
| Upper throat | Pet food | 0.0011 | 0.0016 | 0.0001 |
| Water in the rumen | Spreading/Compost | 0.0000 | 0.0000 | 0.0000 |

Table 13: Total weighting by coproducts for Charolaise Young Bulls reared in Grazing Large Area

| COPRODUCT | Destination | Charolaise/young bull/grazing large area | | |
| --- | --- | --- | --- | --- |
|  |  | **Biophysical Cumulative share** | **Mass Cumulative share** | **Economic Cumulative share** |
| Abomasum | Human food | 0.0148 | 0.0024 | 0.0018 |
| Abomasum fat | Fat and greaves C3 | 0.0011 | 0.0005 | 0.0000 |
| Aponeurosis | Human food | 0.0276 | 0.0355 | 0.0350 |
| Bile | PAP C3 | 0.0000 | 0.0001 | 0.0000 |
| Blood | PAP C3 | 0.0171 | 0.0287 | 0.0063 |
| Blood | Pet food | 0.0024 | 0.0040 | 0.0003 |
| Bones | Gelatin C3 | 0.0511 | 0.0796 | 0.0002 |
| Bones of head, brain, eyes and teeth | C1-C2 for disposal | 0.0000 | 0.0000 | 0.0000 |
| Cheek | Human food | 0.0010 | 0.0015 | 0.0032 |
| Cheek | Human food | 0.0019 | 0.0027 | 0.0058 |
| Cheek trimmings | Pet food | 0.0005 | 0.0007 | 0.0001 |
| Chops | Pet food | 0.0019 | 0.0020 | 0.0001 |
| Contents of intestines | Spreading/Compost | 0.0000 | 0.0000 | 0.0000 |
| Contents of the rumen | Spreading/Compost | 0.0000 | 0.0000 | 0.0000 |
| Ears | PAP C3 | 0.0009 | 0.0010 | 0.0001 |
| Esophagus | Pet food | 0.0009 | 0.0013 | 0.0001 |
| Fat | Fat and greaves C3 | 0.0799 | 0.0852 | 0.0076 |
| Fat around heart | Fat and greaves C3 | 0.0025 | 0.0027 | 0.0002 |
| Fat in the kidney | Fat and greaves C3 | 0.0003 | 0.0004 | 0.0000 |
| Feet (without hooves) | Gelatin C3 | 0.0119 | 0.0173 | 0.0001 |
| Floatation fat | Spreading/Compost | 0.0000 | 0.0000 | 0.0000 |
| Forehead | C1-C2 for disposal | 0.0000 | 0.0000 | 0.0000 |
| Forelock | PAP C3 | 0.0028 | 0.0011 | 0.0001 |
| Gallbladder | Pet food | 0.0003 | 0.0005 | 0.0000 |
| Head trimmings | Pet food | 0.0025 | 0.0036 | 0.0003 |
| Heart | Human food | 0.0029 | 0.0043 | 0.0009 |
| Heart trimmings | Pet food | 0.0003 | 0.0005 | 0.0000 |
| Hide | Skin tannery C3 | 0.0663 | 0.0675 | 0.1106 |
| Hooves | PAP C3 | 0.0078 | 0.0031 | 0.0003 |
| Horns | PAP C3 | 0.0019 | 0.0007 | 0.0001 |
| Kidney | Human food | 0.0019 | 0.0027 | 0.0011 |
| Large intestine | C1-C2 for disposal | 0.0000 | 0.0000 | 0.0000 |
| Liver | Human food | 0.0548 | 0.0175 | 0.0084 |
| Liver trimmings | Pet food | 0.0064 | 0.0020 | 0.0001 |
| Lower jaw | PAP C3 | 0.0030 | 0.0048 | 0.0004 |
| Lungs | Pet food | 0.0080 | 0.0098 | 0.0007 |
| Mask | Skin tannery C3 | 0.0045 | 0.0045 | 0.0074 |
| Mesenteric fat | C1-C2 for disposal | 0.0000 | 0.0000 | 0.0000 |
| Muscle | Human food | 0.3367 | 0.4759 | 0.7815 |
| Muzzle | Human food | 0.0024 | 0.0024 | 0.0024 |
| Omasum | Human food | 0.0134 | 0.0022 | 0.0016 |
| Omasum fat | Fat and greaves C3 | 0.0033 | 0.0015 | 0.0001 |
| Rumen and forestomach | Human food | 0.0675 | 0.0111 | 0.0082 |
| Rumen fat | Fat and greaves C3 | 0.0050 | 0.0022 | 0.0002 |
| Sanitary seizures | C1-C2 for disposal | 0.0000 | 0.0000 | 0.0000 |
| Screening and sifting wastes | C1-C2 for disposal | 0.0000 | 0.0000 | 0.0000 |
| Small intestine | PAP C3 | 0.0787 | 0.0130 | 0.0008 |
| Spinal cord | C1-C2 for disposal | 0.0000 | 0.0000 | 0.0000 |
| Spinal cord waste | C1-C2 for disposal | 0.0000 | 0.0000 | 0.0000 |
| Spine | C1-C2 for disposal | 0.0000 | 0.0000 | 0.0000 |
| Spleen | Pet food | 0.0019 | 0.0027 | 0.0002 |
| Stillborn | PAP C3 | 0.0411 | 0.0065 | 0.0000 |
| Tallow | Fat and greaves C3 | 0.0627 | 0.0670 | 0.0060 |
| Tongue | Human food | 0.0023 | 0.0038 | 0.0059 |
| Tonsil | C1-C2 for disposal | 0.0000 | 0.0000 | 0.0000 |
| Trachea | Pet food | 0.0017 | 0.0017 | 0.0001 |
| Udder | Pet food | 0.0030 | 0.0205 | 0.0015 |
| Upper throat | Pet food | 0.0010 | 0.0015 | 0.0001 |
| Water in the rumen | Spreading/Compost | 0.0000 | 0.0000 | 0.0000 |

Table 14: Total weighting by coproducts for Charolaise Heifers reared in Grazing Large Area

| COPRODUCT | Destination | Charolaise/heifer/grazing large area | | |
| --- | --- | --- | --- | --- |
|  |  | **Biophysical Cumulative share** | **Mass Cumulative share** | **Economic Cumulative share** |
| Abomasum | Human food | 0.0152 | 0.0026 | 0.0020 |
| Abomasum fat | Fat and greaves C3 | 0.0011 | 0.0005 | 0.0000 |
| Aponeurosis | Human food | 0.0260 | 0.0341 | 0.0342 |
| Bile | PAP C3 | 0.0000 | 0.0001 | 0.0000 |
| Blood | PAP C3 | 0.0183 | 0.0311 | 0.0070 |
| Blood | Pet food | 0.0026 | 0.0045 | 0.0003 |
| Bones | Gelatin C3 | 0.0483 | 0.0764 | 0.0002 |
| Bones of head, brain, eyes and teeth | C1-C2 for disposal | 0.0000 | 0.0000 | 0.0000 |
| Cheek | Human food | 0.0011 | 0.0016 | 0.0035 |
| Cheek | Human food | 0.0020 | 0.0029 | 0.0063 |
| Cheek trimmings | Pet food | 0.0005 | 0.0007 | 0.0001 |
| Chops | Pet food | 0.0020 | 0.0021 | 0.0002 |
| Contents of intestines | Spreading/Compost | 0.0000 | 0.0000 | 0.0000 |
| Contents of the rumen | Spreading/Compost | 0.0000 | 0.0000 | 0.0000 |
| Ears | PAP C3 | 0.0008 | 0.0010 | 0.0001 |
| Esophagus | Pet food | 0.0010 | 0.0015 | 0.0001 |
| Fat | Fat and greaves C3 | 0.0759 | 0.0818 | 0.0074 |
| Fat around heart | Fat and greaves C3 | 0.0026 | 0.0029 | 0.0003 |
| Fat in the kidney | Fat and greaves C3 | 0.0003 | 0.0004 | 0.0000 |
| Feet (without hooves) | Gelatin C3 | 0.0127 | 0.0187 | 0.0001 |
| Floatation fat | Spreading/Compost | 0.0000 | 0.0000 | 0.0000 |
| Forehead | C1-C2 for disposal | 0.0000 | 0.0000 | 0.0000 |
| Forelock | PAP C3 | 0.0028 | 0.0011 | 0.0001 |
| Gallbladder | Pet food | 0.0003 | 0.0005 | 0.0000 |
| Head trimmings | Pet food | 0.0027 | 0.0040 | 0.0003 |
| Heart | Human food | 0.0031 | 0.0047 | 0.0010 |
| Heart trimmings | Pet food | 0.0003 | 0.0005 | 0.0000 |
| Hide | Skin tannery C3 | 0.0708 | 0.0733 | 0.1223 |
| Hooves | PAP C3 | 0.0080 | 0.0032 | 0.0003 |
| Horns | PAP C3 | 0.0019 | 0.0007 | 0.0001 |
| Kidney | Human food | 0.0019 | 0.0029 | 0.0012 |
| Large intestine | C1-C2 for disposal | 0.0000 | 0.0000 | 0.0000 |
| Liver | Human food | 0.0573 | 0.0190 | 0.0092 |
| Liver trimmings | Pet food | 0.0066 | 0.0021 | 0.0002 |
| Lower jaw | PAP C3 | 0.0032 | 0.0052 | 0.0004 |
| Lungs | Pet food | 0.0084 | 0.0105 | 0.0008 |
| Mask | Skin tannery C3 | 0.0048 | 0.0050 | 0.0083 |
| Mesenteric fat | C1-C2 for disposal | 0.0000 | 0.0000 | 0.0000 |
| Muscle | Human food | 0.3178 | 0.4569 | 0.7640 |
| Muzzle | Human food | 0.0025 | 0.0026 | 0.0026 |
| Omasum | Human food | 0.0137 | 0.0024 | 0.0018 |
| Omasum fat | Fat and greaves C3 | 0.0035 | 0.0016 | 0.0001 |
| Rumen and forestomach | Human food | 0.0709 | 0.0121 | 0.0091 |
| Rumen fat | Fat and greaves C3 | 0.0051 | 0.0024 | 0.0002 |
| Sanitary seizures | C1-C2 for disposal | 0.0000 | 0.0000 | 0.0000 |
| Screening and sifting wastes | C1-C2 for disposal | 0.0000 | 0.0000 | 0.0000 |
| Small intestine | PAP C3 | 0.0825 | 0.0141 | 0.0009 |
| Spinal cord | C1-C2 for disposal | 0.0000 | 0.0000 | 0.0000 |
| Spinal cord waste | C1-C2 for disposal | 0.0000 | 0.0000 | 0.0000 |
| Spine | C1-C2 for disposal | 0.0000 | 0.0000 | 0.0000 |
| Spleen | Pet food | 0.0020 | 0.0029 | 0.0002 |
| Stillborn | PAP C3 | 0.0431 | 0.0071 | 0.0000 |
| Tallow | Fat and greaves C3 | 0.0673 | 0.0727 | 0.0066 |
| Tongue | Human food | 0.0025 | 0.0042 | 0.0067 |
| Tonsil | C1-C2 for disposal | 0.0000 | 0.0000 | 0.0000 |
| Trachea | Pet food | 0.0018 | 0.0019 | 0.0001 |
| Udder | Pet food | 0.0032 | 0.0222 | 0.0016 |
| Upper throat | Pet food | 0.0011 | 0.0016 | 0.0001 |
| Water in the rumen | Spreading/Compost | 0.0000 | 0.0000 | 0.0000 |

Table 15: Total weighting by coproducts for Charolaise Cull Cows reared in Grazing Large Area

| COPRODUCT | Destination | Charolaise/Cull cow/grazing large area | | |
| --- | --- | --- | --- | --- |
|  |  | **Biophysical Cumulative share** | **Mass Cumulative share** | **Economic Cumulative share** |
| Abomasum | Human food | 0.0164 | 0.0028 | 0.0021 |
| Abomasum fat | Fat and greaves C3 | 0.0011 | 0.0005 | 0.0000 |
| Aponeurosis | Human food | 0.0248 | 0.0331 | 0.0337 |
| Bile | PAP C3 | 0.0000 | 0.0001 | 0.0000 |
| Blood | PAP C3 | 0.0189 | 0.0328 | 0.0074 |
| Blood | Pet food | 0.0027 | 0.0046 | 0.0003 |
| Bones | Gelatin C3 | 0.0456 | 0.0743 | 0.0002 |
| Bones of head, brain, eyes and teeth | C1-C2 for disposal | 0.0000 | 0.0000 | 0.0000 |
| Cheek | Human food | 0.0011 | 0.0016 | 0.0036 |
| Cheek | Human food | 0.0020 | 0.0030 | 0.0067 |
| Cheek trimmings | Pet food | 0.0006 | 0.0009 | 0.0001 |
| Chops | Pet food | 0.0021 | 0.0023 | 0.0002 |
| Contents of intestines | Spreading/Compost | 0.0000 | 0.0000 | 0.0000 |
| Contents of the rumen | Spreading/Compost | 0.0000 | 0.0000 | 0.0000 |
| Ears | PAP C3 | 0.0009 | 0.0011 | 0.0001 |
| Esophagus | Pet food | 0.0010 | 0.0015 | 0.0001 |
| Fat | Fat and greaves C3 | 0.0692 | 0.0795 | 0.0073 |
| Fat around heart | Fat and greaves C3 | 0.0026 | 0.0030 | 0.0003 |
| Fat in the kidney | Fat and greaves C3 | 0.0003 | 0.0004 | 0.0000 |
| Feet (without hooves) | Gelatin C3 | 0.0131 | 0.0196 | 0.0001 |
| Floatation fat | Spreading/Compost | 0.0000 | 0.0000 | 0.0000 |
| Forehead | C1-C2 for disposal | 0.0000 | 0.0000 | 0.0000 |
| Forelock | PAP C3 | 0.0031 | 0.0013 | 0.0001 |
| Gallbladder | Pet food | 0.0003 | 0.0005 | 0.0000 |
| Head trimmings | Pet food | 0.0028 | 0.0041 | 0.0003 |
| Heart | Human food | 0.0032 | 0.0050 | 0.0011 |
| Heart trimmings | Pet food | 0.0003 | 0.0005 | 0.0000 |
| Hide | Skin tannery C3 | 0.0732 | 0.0771 | 0.1304 |
| Hooves | PAP C3 | 0.0083 | 0.0034 | 0.0003 |
| Horns | PAP C3 | 0.0021 | 0.0009 | 0.0001 |
| Kidney | Human food | 0.0020 | 0.0030 | 0.0012 |
| Large intestine | C1-C2 for disposal | 0.0000 | 0.0000 | 0.0000 |
| Liver | Human food | 0.0610 | 0.0199 | 0.0098 |
| Liver trimmings | Pet food | 0.0072 | 0.0023 | 0.0002 |
| Lower jaw | PAP C3 | 0.0033 | 0.0055 | 0.0005 |
| Lungs | Pet food | 0.0087 | 0.0111 | 0.0008 |
| Mask | Skin tannery C3 | 0.0050 | 0.0053 | 0.0089 |
| Mesenteric fat | C1-C2 for disposal | 0.0000 | 0.0000 | 0.0000 |
| Muscle | Human food | 0.3026 | 0.4440 | 0.7520 |
| Muzzle | Human food | 0.0026 | 0.0028 | 0.0028 |
| Omasum | Human food | 0.0149 | 0.0025 | 0.0019 |
| Omasum fat | Fat and greaves C3 | 0.0035 | 0.0016 | 0.0001 |
| Rumen and forestomach | Human food | 0.0760 | 0.0128 | 0.0097 |
| Rumen fat | Fat and greaves C3 | 0.0054 | 0.0025 | 0.0002 |
| Sanitary seizures | C1-C2 for disposal | 0.0000 | 0.0000 | 0.0000 |
| Screening and sifting wastes | C1-C2 for disposal | 0.0000 | 0.0000 | 0.0000 |
| Small intestine | PAP C3 | 0.0887 | 0.0149 | 0.0009 |
| Spinal cord | C1-C2 for disposal | 0.0000 | 0.0000 | 0.0000 |
| Spinal cord waste | C1-C2 for disposal | 0.0000 | 0.0000 | 0.0000 |
| Spine | C1-C2 for disposal | 0.0000 | 0.0000 | 0.0000 |
| Spleen | Pet food | 0.0021 | 0.0030 | 0.0002 |
| Stillborn | PAP C3 | 0.0459 | 0.0074 | 0.0000 |
| Tallow | Fat and greaves C3 | 0.0665 | 0.0765 | 0.0071 |
| Tongue | Human food | 0.0026 | 0.0044 | 0.0071 |
| Tonsil | C1-C2 for disposal | 0.0000 | 0.0000 | 0.0000 |
| Trachea | Pet food | 0.0018 | 0.0019 | 0.0001 |
| Udder | Pet food | 0.0032 | 0.0234 | 0.0017 |
| Upper throat | Pet food | 0.0011 | 0.0016 | 0.0001 |
| Water in the rumen | Spreading/Compost | 0.0000 | 0.0000 | 0.0000 |

Table 16: Total weighting by coproducts for Charolaise Beef reared in Grazing Large Area

| COPRODUCT | Destination | Charolaise/beef/grazing large area | | |
| --- | --- | --- | --- | --- |
|  |  | **Biophysical Cumulative share** | **Mass Cumulative share** | **Economic Cumulative share** |
| Abomasum | Human food | 0.0160 | 0.0026 | 0.0019 |
| Abomasum fat | Fat and greaves C3 | 0.0011 | 0.0005 | 0.0000 |
| Aponeurosis | Human food | 0.0265 | 0.0346 | 0.0345 |
| Bile | PAP C3 | 0.0000 | 0.0001 | 0.0000 |
| Blood | PAP C3 | 0.0178 | 0.0303 | 0.0067 |
| Blood | Pet food | 0.0025 | 0.0043 | 0.0003 |
| Bones | Gelatin C3 | 0.0484 | 0.0776 | 0.0002 |
| Bones of head, brain, eyes and teeth | C1-C2 for disposal | 0.0000 | 0.0000 | 0.0000 |
| Cheek | Human food | 0.0010 | 0.0015 | 0.0032 |
| Cheek | Human food | 0.0020 | 0.0028 | 0.0062 |
| Cheek trimmings | Pet food | 0.0005 | 0.0007 | 0.0001 |
| Chops | Pet food | 0.0019 | 0.0020 | 0.0001 |
| Contents of intestines | Spreading/Compost | 0.0000 | 0.0000 | 0.0000 |
| Contents of the rumen | Spreading/Compost | 0.0000 | 0.0000 | 0.0000 |
| Ears | PAP C3 | 0.0008 | 0.0010 | 0.0001 |
| Esophagus | Pet food | 0.0009 | 0.0014 | 0.0001 |
| Fat | Fat and greaves C3 | 0.0722 | 0.0830 | 0.0075 |
| Fat around heart | Fat and greaves C3 | 0.0025 | 0.0028 | 0.0003 |
| Fat in the kidney | Fat and greaves C3 | 0.0003 | 0.0004 | 0.0000 |
| Feet (without hooves) | Gelatin C3 | 0.0123 | 0.0182 | 0.0001 |
| Floatation fat | Spreading/Compost | 0.0000 | 0.0000 | 0.0000 |
| Forehead | C1-C2 for disposal | 0.0000 | 0.0000 | 0.0000 |
| Forelock | PAP C3 | 0.0028 | 0.0011 | 0.0001 |
| Gallbladder | Pet food | 0.0003 | 0.0005 | 0.0000 |
| Head trimmings | Pet food | 0.0026 | 0.0038 | 0.0003 |
| Heart | Human food | 0.0030 | 0.0046 | 0.0010 |
| Heart trimmings | Pet food | 0.0003 | 0.0005 | 0.0000 |
| Hide | Skin tannery C3 | 0.0691 | 0.0714 | 0.1184 |
| Hooves | PAP C3 | 0.0080 | 0.0032 | 0.0003 |
| Horns | PAP C3 | 0.0019 | 0.0007 | 0.0001 |
| Kidney | Human food | 0.0019 | 0.0028 | 0.0011 |
| Large intestine | C1-C2 for disposal | 0.0000 | 0.0000 | 0.0000 |
| Liver | Human food | 0.0585 | 0.0184 | 0.0089 |
| Liver trimmings | Pet food | 0.0066 | 0.0020 | 0.0001 |
| Lower jaw | PAP C3 | 0.0031 | 0.0051 | 0.0004 |
| Lungs | Pet food | 0.0082 | 0.0103 | 0.0007 |
| Mask | Skin tannery C3 | 0.0047 | 0.0048 | 0.0080 |
| Mesenteric fat | C1-C2 for disposal | 0.0000 | 0.0000 | 0.0000 |
| Muscle | Human food | 0.3221 | 0.4634 | 0.7699 |
| Muzzle | Human food | 0.0025 | 0.0026 | 0.0026 |
| Omasum | Human food | 0.0145 | 0.0023 | 0.0017 |
| Omasum fat | Fat and greaves C3 | 0.0033 | 0.0015 | 0.0001 |
| Rumen and forestomach | Human food | 0.0734 | 0.0119 | 0.0088 |
| Rumen fat | Fat and greaves C3 | 0.0052 | 0.0023 | 0.0002 |
| Sanitary seizures | C1-C2 for disposal | 0.0000 | 0.0000 | 0.0000 |
| Screening and sifting wastes | C1-C2 for disposal | 0.0000 | 0.0000 | 0.0000 |
| Small intestine | PAP C3 | 0.0848 | 0.0137 | 0.0008 |
| Spinal cord | C1-C2 for disposal | 0.0000 | 0.0000 | 0.0000 |
| Spinal cord waste | C1-C2 for disposal | 0.0000 | 0.0000 | 0.0000 |
| Spine | C1-C2 for disposal | 0.0000 | 0.0000 | 0.0000 |
| Spleen | Pet food | 0.0020 | 0.0028 | 0.0002 |
| Stillborn | PAP C3 | 0.0447 | 0.0069 | 0.0000 |
| Tallow | Fat and greaves C3 | 0.0614 | 0.0708 | 0.0064 |
| Tongue | Human food | 0.0024 | 0.0041 | 0.0065 |
| Tonsil | C1-C2 for disposal | 0.0000 | 0.0000 | 0.0000 |
| Trachea | Pet food | 0.0017 | 0.0017 | 0.0001 |
| Udder | Pet food | 0.0030 | 0.0216 | 0.0016 |
| Upper throat | Pet food | 0.0010 | 0.0015 | 0.0001 |
| Water in the rumen | Spreading/Compost | 0.0000 | 0.0000 | 0.0000 |

Table 17: Total weighting by coproducts for Charolaise Young Bulls reared in Pasture

| COPRODUCT | Destination | Charolaise/young bull/pasture | | |
| --- | --- | --- | --- | --- |
|  |  | **Biophysical Cumulative share** | **Mass Cumulative share** | **Economic Cumulative share** |
| Abomasum | Human food | 0.0142 | 0.0024 | 0.0018 |
| Abomasum fat | Fat and greaves C3 | 0.0011 | 0.0005 | 0.0000 |
| Aponeurosis | Human food | 0.0273 | 0.0355 | 0.0350 |
| Bile | PAP C3 | 0.0000 | 0.0001 | 0.0000 |
| Blood | PAP C3 | 0.0170 | 0.0287 | 0.0063 |
| Blood | Pet food | 0.0024 | 0.0040 | 0.0003 |
| Bones | Gelatin C3 | 0.0518 | 0.0796 | 0.0002 |
| Bones of head, brain, eyes and teeth | C1-C2 for disposal | 0.0000 | 0.0000 | 0.0000 |
| Cheek | Human food | 0.0010 | 0.0015 | 0.0032 |
| Cheek | Human food | 0.0019 | 0.0027 | 0.0058 |
| Cheek trimmings | Pet food | 0.0005 | 0.0007 | 0.0001 |
| Chops | Pet food | 0.0019 | 0.0020 | 0.0001 |
| Contents of intestines | Spreading/Compost | 0.0000 | 0.0000 | 0.0000 |
| Contents of the rumen | Spreading/Compost | 0.0000 | 0.0000 | 0.0000 |
| Ears | PAP C3 | 0.0009 | 0.0010 | 0.0001 |
| Esophagus | Pet food | 0.0009 | 0.0013 | 0.0001 |
| Fat | Fat and greaves C3 | 0.0878 | 0.0852 | 0.0076 |
| Fat around heart | Fat and greaves C3 | 0.0028 | 0.0027 | 0.0002 |
| Fat in the kidney | Fat and greaves C3 | 0.0004 | 0.0004 | 0.0000 |
| Feet (without hooves) | Gelatin C3 | 0.0119 | 0.0173 | 0.0001 |
| Floatation fat | Spreading/Compost | 0.0000 | 0.0000 | 0.0000 |
| Forehead | C1-C2 for disposal | 0.0000 | 0.0000 | 0.0000 |
| Forelock | PAP C3 | 0.0028 | 0.0011 | 0.0001 |
| Gallbladder | Pet food | 0.0003 | 0.0005 | 0.0000 |
| Head trimmings | Pet food | 0.0025 | 0.0036 | 0.0003 |
| Heart | Human food | 0.0029 | 0.0043 | 0.0009 |
| Heart trimmings | Pet food | 0.0003 | 0.0005 | 0.0000 |
| Hide | Skin tannery C3 | 0.0656 | 0.0675 | 0.1106 |
| Hooves | PAP C3 | 0.0077 | 0.0031 | 0.0003 |
| Horns | PAP C3 | 0.0018 | 0.0007 | 0.0001 |
| Kidney | Human food | 0.0019 | 0.0027 | 0.0011 |
| Large intestine | C1-C2 for disposal | 0.0000 | 0.0000 | 0.0000 |
| Liver | Human food | 0.0527 | 0.0175 | 0.0084 |
| Liver trimmings | Pet food | 0.0061 | 0.0020 | 0.0001 |
| Lower jaw | PAP C3 | 0.0031 | 0.0048 | 0.0004 |
| Lungs | Pet food | 0.0079 | 0.0098 | 0.0007 |
| Mask | Skin tannery C3 | 0.0044 | 0.0045 | 0.0074 |
| Mesenteric fat | C1-C2 for disposal | 0.0000 | 0.0000 | 0.0000 |
| Muscle | Human food | 0.3348 | 0.4759 | 0.7815 |
| Muzzle | Human food | 0.0024 | 0.0024 | 0.0024 |
| Omasum | Human food | 0.0128 | 0.0022 | 0.0016 |
| Omasum fat | Fat and greaves C3 | 0.0033 | 0.0015 | 0.0001 |
| Rumen and forestomach | Human food | 0.0647 | 0.0111 | 0.0082 |
| Rumen fat | Fat and greaves C3 | 0.0050 | 0.0022 | 0.0002 |
| Sanitary seizures | C1-C2 for disposal | 0.0000 | 0.0000 | 0.0000 |
| Screening and sifting wastes | C1-C2 for disposal | 0.0000 | 0.0000 | 0.0000 |
| Small intestine | PAP C3 | 0.0753 | 0.0130 | 0.0008 |
| Spinal cord | C1-C2 for disposal | 0.0000 | 0.0000 | 0.0000 |
| Spinal cord waste | C1-C2 for disposal | 0.0000 | 0.0000 | 0.0000 |
| Spine | C1-C2 for disposal | 0.0000 | 0.0000 | 0.0000 |
| Spleen | Pet food | 0.0019 | 0.0027 | 0.0002 |
| Stillborn | PAP C3 | 0.0393 | 0.0065 | 0.0000 |
| Tallow | Fat and greaves C3 | 0.0689 | 0.0670 | 0.0060 |
| Tongue | Human food | 0.0023 | 0.0038 | 0.0059 |
| Tonsil | C1-C2 for disposal | 0.0000 | 0.0000 | 0.0000 |
| Trachea | Pet food | 0.0017 | 0.0017 | 0.0001 |
| Udder | Pet food | 0.0031 | 0.0205 | 0.0015 |
| Upper throat | Pet food | 0.0010 | 0.0015 | 0.0001 |
| Water in the rumen | Spreading/Compost | 0.0000 | 0.0000 | 0.0000 |

Table 18: Total weighting by coproducts for Charolaise Heifers reared in Pasture

| COPRODUCT | Destination | Charolaise/heifer/pasture | | |
| --- | --- | --- | --- | --- |
|  |  | **Biophysical Cumulative share** | **Mass Cumulative share** | **Economic Cumulative share** |
| Abomasum | Human food | 0.0145 | 0.0026 | 0.0020 |
| Abomasum fat | Fat and greaves C3 | 0.0011 | 0.0005 | 0.0000 |
| Aponeurosis | Human food | 0.0258 | 0.0341 | 0.0342 |
| Bile | PAP C3 | 0.0000 | 0.0001 | 0.0000 |
| Blood | PAP C3 | 0.0181 | 0.0311 | 0.0070 |
| Blood | Pet food | 0.0026 | 0.0045 | 0.0003 |
| Bones | Gelatin C3 | 0.0490 | 0.0764 | 0.0002 |
| Bones of head, brain, eyes and teeth | C1-C2 for disposal | 0.0000 | 0.0000 | 0.0000 |
| Cheek | Human food | 0.0011 | 0.0016 | 0.0035 |
| Cheek | Human food | 0.0020 | 0.0029 | 0.0063 |
| Cheek trimmings | Pet food | 0.0005 | 0.0007 | 0.0001 |
| Chops | Pet food | 0.0020 | 0.0021 | 0.0002 |
| Contents of intestines | Spreading/Compost | 0.0000 | 0.0000 | 0.0000 |
| Contents of the rumen | Spreading/Compost | 0.0000 | 0.0000 | 0.0000 |
| Ears | PAP C3 | 0.0008 | 0.0010 | 0.0001 |
| Esophagus | Pet food | 0.0010 | 0.0015 | 0.0001 |
| Fat | Fat and greaves C3 | 0.0833 | 0.0818 | 0.0074 |
| Fat around heart | Fat and greaves C3 | 0.0029 | 0.0029 | 0.0003 |
| Fat in the kidney | Fat and greaves C3 | 0.0004 | 0.0004 | 0.0000 |
| Feet (without hooves) | Gelatin C3 | 0.0127 | 0.0187 | 0.0001 |
| Floatation fat | Spreading/Compost | 0.0000 | 0.0000 | 0.0000 |
| Forehead | C1-C2 for disposal | 0.0000 | 0.0000 | 0.0000 |
| Forelock | PAP C3 | 0.0027 | 0.0011 | 0.0001 |
| Gallbladder | Pet food | 0.0003 | 0.0005 | 0.0000 |
| Head trimmings | Pet food | 0.0027 | 0.0040 | 0.0003 |
| Heart | Human food | 0.0031 | 0.0047 | 0.0010 |
| Heart trimmings | Pet food | 0.0003 | 0.0005 | 0.0000 |
| Hide | Skin tannery C3 | 0.0701 | 0.0733 | 0.1223 |
| Hooves | PAP C3 | 0.0079 | 0.0032 | 0.0003 |
| Horns | PAP C3 | 0.0018 | 0.0007 | 0.0001 |
| Kidney | Human food | 0.0019 | 0.0029 | 0.0012 |
| Large intestine | C1-C2 for disposal | 0.0000 | 0.0000 | 0.0000 |
| Liver | Human food | 0.0551 | 0.0190 | 0.0092 |
| Liver trimmings | Pet food | 0.0064 | 0.0021 | 0.0002 |
| Lower jaw | PAP C3 | 0.0033 | 0.0052 | 0.0004 |
| Lungs | Pet food | 0.0083 | 0.0105 | 0.0008 |
| Mask | Skin tannery C3 | 0.0047 | 0.0050 | 0.0083 |
| Mesenteric fat | C1-C2 for disposal | 0.0000 | 0.0000 | 0.0000 |
| Muscle | Human food | 0.3164 | 0.4569 | 0.7640 |
| Muzzle | Human food | 0.0025 | 0.0026 | 0.0026 |
| Omasum | Human food | 0.0131 | 0.0024 | 0.0018 |
| Omasum fat | Fat and greaves C3 | 0.0035 | 0.0016 | 0.0001 |
| Rumen and forestomach | Human food | 0.0678 | 0.0121 | 0.0091 |
| Rumen fat | Fat and greaves C3 | 0.0052 | 0.0024 | 0.0002 |
| Sanitary seizures | C1-C2 for disposal | 0.0000 | 0.0000 | 0.0000 |
| Screening and sifting wastes | C1-C2 for disposal | 0.0000 | 0.0000 | 0.0000 |
| Small intestine | PAP C3 | 0.0789 | 0.0141 | 0.0009 |
| Spinal cord | C1-C2 for disposal | 0.0000 | 0.0000 | 0.0000 |
| Spinal cord waste | C1-C2 for disposal | 0.0000 | 0.0000 | 0.0000 |
| Spine | C1-C2 for disposal | 0.0000 | 0.0000 | 0.0000 |
| Spleen | Pet food | 0.0020 | 0.0029 | 0.0002 |
| Stillborn | PAP C3 | 0.0412 | 0.0071 | 0.0000 |
| Tallow | Fat and greaves C3 | 0.0739 | 0.0727 | 0.0066 |
| Tongue | Human food | 0.0026 | 0.0042 | 0.0067 |
| Tonsil | C1-C2 for disposal | 0.0000 | 0.0000 | 0.0000 |
| Trachea | Pet food | 0.0018 | 0.0019 | 0.0001 |
| Udder | Pet food | 0.0033 | 0.0222 | 0.0016 |
| Upper throat | Pet food | 0.0011 | 0.0016 | 0.0001 |
| Water in the rumen | Spreading/Compost | 0.0000 | 0.0000 | 0.0000 |

Table 19: Total weighting by coproducts for Charolaise Cull Cows reared in Pasture

| COPRODUCT | Destination | Charolaise/Cull cow/pasture | | |
| --- | --- | --- | --- | --- |
|  |  | **Biophysical Cumulative share** | **Mass Cumulative share** | **Economic Cumulative share** |
| Abomasum | Human food | 0.0157 | 0.0028 | 0.0021 |
| Abomasum fat | Fat and greaves C3 | 0.0011 | 0.0005 | 0.0000 |
| Aponeurosis | Human food | 0.0246 | 0.0331 | 0.0337 |
| Bile | PAP C3 | 0.0000 | 0.0001 | 0.0000 |
| Blood | PAP C3 | 0.0187 | 0.0328 | 0.0074 |
| Blood | Pet food | 0.0026 | 0.0046 | 0.0003 |
| Bones | Gelatin C3 | 0.0463 | 0.0743 | 0.0002 |
| Bones of head, brain, eyes and teeth | C1-C2 for disposal | 0.0000 | 0.0000 | 0.0000 |
| Cheek | Human food | 0.0011 | 0.0016 | 0.0036 |
| Cheek | Human food | 0.0020 | 0.0030 | 0.0067 |
| Cheek trimmings | Pet food | 0.0006 | 0.0009 | 0.0001 |
| Chops | Pet food | 0.0021 | 0.0023 | 0.0002 |
| Contents of intestines | Spreading/Compost | 0.0000 | 0.0000 | 0.0000 |
| Contents of the rumen | Spreading/Compost | 0.0000 | 0.0000 | 0.0000 |
| Ears | PAP C3 | 0.0009 | 0.0011 | 0.0001 |
| Esophagus | Pet food | 0.0010 | 0.0015 | 0.0001 |
| Fat | Fat and greaves C3 | 0.0763 | 0.0795 | 0.0073 |
| Fat around heart | Fat and greaves C3 | 0.0029 | 0.0030 | 0.0003 |
| Fat in the kidney | Fat and greaves C3 | 0.0004 | 0.0004 | 0.0000 |
| Feet (without hooves) | Gelatin C3 | 0.0130 | 0.0196 | 0.0001 |
| Floatation fat | Spreading/Compost | 0.0000 | 0.0000 | 0.0000 |
| Forehead | C1-C2 for disposal | 0.0000 | 0.0000 | 0.0000 |
| Forelock | PAP C3 | 0.0030 | 0.0013 | 0.0001 |
| Gallbladder | Pet food | 0.0003 | 0.0005 | 0.0000 |
| Head trimmings | Pet food | 0.0028 | 0.0041 | 0.0003 |
| Heart | Human food | 0.0032 | 0.0050 | 0.0011 |
| Heart trimmings | Pet food | 0.0003 | 0.0005 | 0.0000 |
| Hide | Skin tannery C3 | 0.0724 | 0.0771 | 0.1304 |
| Hooves | PAP C3 | 0.0082 | 0.0034 | 0.0003 |
| Horns | PAP C3 | 0.0021 | 0.0009 | 0.0001 |
| Kidney | Human food | 0.0020 | 0.0030 | 0.0012 |
| Large intestine | C1-C2 for disposal | 0.0000 | 0.0000 | 0.0000 |
| Liver | Human food | 0.0588 | 0.0199 | 0.0098 |
| Liver trimmings | Pet food | 0.0069 | 0.0023 | 0.0002 |
| Lower jaw | PAP C3 | 0.0034 | 0.0055 | 0.0005 |
| Lungs | Pet food | 0.0086 | 0.0111 | 0.0008 |
| Mask | Skin tannery C3 | 0.0049 | 0.0053 | 0.0089 |
| Mesenteric fat | C1-C2 for disposal | 0.0000 | 0.0000 | 0.0000 |
| Muscle | Human food | 0.3012 | 0.4440 | 0.7520 |
| Muzzle | Human food | 0.0026 | 0.0028 | 0.0028 |
| Omasum | Human food | 0.0143 | 0.0025 | 0.0019 |
| Omasum fat | Fat and greaves C3 | 0.0035 | 0.0016 | 0.0001 |
| Rumen and forestomach | Human food | 0.0730 | 0.0128 | 0.0097 |
| Rumen fat | Fat and greaves C3 | 0.0055 | 0.0025 | 0.0002 |
| Sanitary seizures | C1-C2 for disposal | 0.0000 | 0.0000 | 0.0000 |
| Screening and sifting wastes | C1-C2 for disposal | 0.0000 | 0.0000 | 0.0000 |
| Small intestine | PAP C3 | 0.0852 | 0.0149 | 0.0009 |
| Spinal cord | C1-C2 for disposal | 0.0000 | 0.0000 | 0.0000 |
| Spinal cord waste | C1-C2 for disposal | 0.0000 | 0.0000 | 0.0000 |
| Spine | C1-C2 for disposal | 0.0000 | 0.0000 | 0.0000 |
| Spleen | Pet food | 0.0021 | 0.0030 | 0.0002 |
| Stillborn | PAP C3 | 0.0441 | 0.0074 | 0.0000 |
| Tallow | Fat and greaves C3 | 0.0733 | 0.0765 | 0.0071 |
| Tongue | Human food | 0.0026 | 0.0044 | 0.0071 |
| Tonsil | C1-C2 for disposal | 0.0000 | 0.0000 | 0.0000 |
| Trachea | Pet food | 0.0018 | 0.0019 | 0.0001 |
| Udder | Pet food | 0.0033 | 0.0234 | 0.0017 |
| Upper throat | Pet food | 0.0011 | 0.0016 | 0.0001 |
| Water in the rumen | Spreading/Compost | 0.0000 | 0.0000 | 0.0000 |

Table 20: Total weighting by coproducts for Charolaise Beef reared in Pasture

| COPRODUCT | Destination | Charolaise/beef/pasture | | |
| --- | --- | --- | --- | --- |
|  |  | **Biophysical Cumulative share** | **Mass Cumulative share** | **Economic Cumulative share** |
| Abomasum | Human food | 0.0154 | 0.0026 | 0.0019 |
| Abomasum fat | Fat and greaves C3 | 0.0011 | 0.0005 | 0.0000 |
| Aponeurosis | Human food | 0.0262 | 0.0346 | 0.0345 |
| Bile | PAP C3 | 0.0000 | 0.0001 | 0.0000 |
| Blood | PAP C3 | 0.0176 | 0.0303 | 0.0067 |
| Blood | Pet food | 0.0025 | 0.0043 | 0.0003 |
| Bones | Gelatin C3 | 0.0491 | 0.0776 | 0.0002 |
| Bones of head, brain, eyes and teeth | C1-C2 for disposal | 0.0000 | 0.0000 | 0.0000 |
| Cheek | Human food | 0.0010 | 0.0015 | 0.0032 |
| Cheek | Human food | 0.0019 | 0.0028 | 0.0062 |
| Cheek trimmings | Pet food | 0.0005 | 0.0007 | 0.0001 |
| Chops | Pet food | 0.0019 | 0.0020 | 0.0001 |
| Contents of intestines | Spreading/Compost | 0.0000 | 0.0000 | 0.0000 |
| Contents of the rumen | Spreading/Compost | 0.0000 | 0.0000 | 0.0000 |
| Ears | PAP C3 | 0.0008 | 0.0010 | 0.0001 |
| Esophagus | Pet food | 0.0009 | 0.0014 | 0.0001 |
| Fat | Fat and greaves C3 | 0.0796 | 0.0830 | 0.0075 |
| Fat around heart | Fat and greaves C3 | 0.0027 | 0.0028 | 0.0003 |
| Fat in the kidney | Fat and greaves C3 | 0.0004 | 0.0004 | 0.0000 |
| Feet (without hooves) | Gelatin C3 | 0.0123 | 0.0182 | 0.0001 |
| Floatation fat | Spreading/Compost | 0.0000 | 0.0000 | 0.0000 |
| Forehead | C1-C2 for disposal | 0.0000 | 0.0000 | 0.0000 |
| Forelock | PAP C3 | 0.0027 | 0.0011 | 0.0001 |
| Gallbladder | Pet food | 0.0003 | 0.0005 | 0.0000 |
| Head trimmings | Pet food | 0.0026 | 0.0038 | 0.0003 |
| Heart | Human food | 0.0030 | 0.0046 | 0.0010 |
| Heart trimmings | Pet food | 0.0003 | 0.0005 | 0.0000 |
| Hide | Skin tannery C3 | 0.0683 | 0.0714 | 0.1184 |
| Hooves | PAP C3 | 0.0079 | 0.0032 | 0.0003 |
| Horns | PAP C3 | 0.0018 | 0.0007 | 0.0001 |
| Kidney | Human food | 0.0019 | 0.0028 | 0.0011 |
| Large intestine | C1-C2 for disposal | 0.0000 | 0.0000 | 0.0000 |
| Liver | Human food | 0.0565 | 0.0184 | 0.0089 |
| Liver trimmings | Pet food | 0.0063 | 0.0020 | 0.0001 |
| Lower jaw | PAP C3 | 0.0031 | 0.0051 | 0.0004 |
| Lungs | Pet food | 0.0081 | 0.0103 | 0.0007 |
| Mask | Skin tannery C3 | 0.0046 | 0.0048 | 0.0080 |
| Mesenteric fat | C1-C2 for disposal | 0.0000 | 0.0000 | 0.0000 |
| Muscle | Human food | 0.3202 | 0.4634 | 0.7699 |
| Muzzle | Human food | 0.0025 | 0.0026 | 0.0026 |
| Omasum | Human food | 0.0140 | 0.0023 | 0.0017 |
| Omasum fat | Fat and greaves C3 | 0.0033 | 0.0015 | 0.0001 |
| Rumen and forestomach | Human food | 0.0705 | 0.0119 | 0.0088 |
| Rumen fat | Fat and greaves C3 | 0.0053 | 0.0023 | 0.0002 |
| Sanitary seizures | C1-C2 for disposal | 0.0000 | 0.0000 | 0.0000 |
| Screening and sifting wastes | C1-C2 for disposal | 0.0000 | 0.0000 | 0.0000 |
| Small intestine | PAP C3 | 0.0816 | 0.0137 | 0.0008 |
| Spinal cord | C1-C2 for disposal | 0.0000 | 0.0000 | 0.0000 |
| Spinal cord waste | C1-C2 for disposal | 0.0000 | 0.0000 | 0.0000 |
| Spine | C1-C2 for disposal | 0.0000 | 0.0000 | 0.0000 |
| Spleen | Pet food | 0.0020 | 0.0028 | 0.0002 |
| Stillborn | PAP C3 | 0.0430 | 0.0069 | 0.0000 |
| Tallow | Fat and greaves C3 | 0.0678 | 0.0708 | 0.0064 |
| Tongue | Human food | 0.0025 | 0.0041 | 0.0065 |
| Tonsil | C1-C2 for disposal | 0.0000 | 0.0000 | 0.0000 |
| Trachea | Pet food | 0.0017 | 0.0017 | 0.0001 |
| Udder | Pet food | 0.0031 | 0.0216 | 0.0016 |
| Upper throat | Pet food | 0.0010 | 0.0015 | 0.0001 |
| Water in the rumen | Spreading/Compost | 0.0000 | 0.0000 | 0.0000 |

Table 21: Total weighting by coproducts for Charolaise Young Bulls reared in Stall

| COPRODUCT | Destination | Charolaise/young bull/stall | | |
| --- | --- | --- | --- | --- |
|  |  | **Biophysical Cumulative share** | **Mass Cumulative share** | **Economic Cumulative share** |
| Abomasum | Human food | 0.0135 | 0.0024 | 0.0018 |
| Abomasum fat | Fat and greaves C3 | 0.0011 | 0.0005 | 0.0000 |
| Aponeurosis | Human food | 0.0269 | 0.0355 | 0.0350 |
| Bile | PAP C3 | 0.0000 | 0.0001 | 0.0000 |
| Blood | PAP C3 | 0.0168 | 0.0287 | 0.0063 |
| Blood | Pet food | 0.0024 | 0.0040 | 0.0003 |
| Bones | Gelatin C3 | 0.0526 | 0.0796 | 0.0002 |
| Bones of head, brain, eyes and teeth | C1-C2 for disposal | 0.0000 | 0.0000 | 0.0000 |
| Cheek | Human food | 0.0010 | 0.0015 | 0.0032 |
| Cheek | Human food | 0.0019 | 0.0027 | 0.0058 |
| Cheek trimmings | Pet food | 0.0005 | 0.0007 | 0.0001 |
| Chops | Pet food | 0.0019 | 0.0020 | 0.0001 |
| Contents of intestines | Spreading/Compost | 0.0000 | 0.0000 | 0.0000 |
| Contents of the rumen | Spreading/Compost | 0.0000 | 0.0000 | 0.0000 |
| Ears | PAP C3 | 0.0009 | 0.0010 | 0.0001 |
| Esophagus | Pet food | 0.0009 | 0.0013 | 0.0001 |
| Fat | Fat and greaves C3 | 0.0964 | 0.0852 | 0.0076 |
| Fat around heart | Fat and greaves C3 | 0.0030 | 0.0027 | 0.0002 |
| Fat in the kidney | Fat and greaves C3 | 0.0004 | 0.0004 | 0.0000 |
| Feet (without hooves) | Gelatin C3 | 0.0118 | 0.0173 | 0.0001 |
| Floatation fat | Spreading/Compost | 0.0000 | 0.0000 | 0.0000 |
| Forehead | C1-C2 for disposal | 0.0000 | 0.0000 | 0.0000 |
| Forelock | PAP C3 | 0.0027 | 0.0011 | 0.0001 |
| Gallbladder | Pet food | 0.0003 | 0.0005 | 0.0000 |
| Head trimmings | Pet food | 0.0025 | 0.0036 | 0.0003 |
| Heart | Human food | 0.0028 | 0.0043 | 0.0009 |
| Heart trimmings | Pet food | 0.0003 | 0.0005 | 0.0000 |
| Hide | Skin tannery C3 | 0.0648 | 0.0675 | 0.1106 |
| Hooves | PAP C3 | 0.0075 | 0.0031 | 0.0003 |
| Horns | PAP C3 | 0.0018 | 0.0007 | 0.0001 |
| Kidney | Human food | 0.0018 | 0.0027 | 0.0011 |
| Large intestine | C1-C2 for disposal | 0.0000 | 0.0000 | 0.0000 |
| Liver | Human food | 0.0504 | 0.0175 | 0.0084 |
| Liver trimmings | Pet food | 0.0059 | 0.0020 | 0.0001 |
| Lower jaw | PAP C3 | 0.0031 | 0.0048 | 0.0004 |
| Lungs | Pet food | 0.0078 | 0.0098 | 0.0007 |
| Mask | Skin tannery C3 | 0.0044 | 0.0045 | 0.0074 |
| Mesenteric fat | C1-C2 for disposal | 0.0000 | 0.0000 | 0.0000 |
| Muscle | Human food | 0.3327 | 0.4759 | 0.7815 |
| Muzzle | Human food | 0.0024 | 0.0024 | 0.0024 |
| Omasum | Human food | 0.0122 | 0.0022 | 0.0016 |
| Omasum fat | Fat and greaves C3 | 0.0033 | 0.0015 | 0.0001 |
| Rumen and forestomach | Human food | 0.0615 | 0.0111 | 0.0082 |
| Rumen fat | Fat and greaves C3 | 0.0050 | 0.0022 | 0.0002 |
| Sanitary seizures | C1-C2 for disposal | 0.0000 | 0.0000 | 0.0000 |
| Screening and sifting wastes | C1-C2 for disposal | 0.0000 | 0.0000 | 0.0000 |
| Small intestine | PAP C3 | 0.0716 | 0.0130 | 0.0008 |
| Spinal cord | C1-C2 for disposal | 0.0000 | 0.0000 | 0.0000 |
| Spinal cord waste | C1-C2 for disposal | 0.0000 | 0.0000 | 0.0000 |
| Spine | C1-C2 for disposal | 0.0000 | 0.0000 | 0.0000 |
| Spleen | Pet food | 0.0019 | 0.0027 | 0.0002 |
| Stillborn | PAP C3 | 0.0374 | 0.0065 | 0.0000 |
| Tallow | Fat and greaves C3 | 0.0757 | 0.0670 | 0.0060 |
| Tongue | Human food | 0.0024 | 0.0038 | 0.0059 |
| Tonsil | C1-C2 for disposal | 0.0000 | 0.0000 | 0.0000 |
| Trachea | Pet food | 0.0017 | 0.0017 | 0.0001 |
| Udder | Pet food | 0.0032 | 0.0205 | 0.0015 |
| Upper throat | Pet food | 0.0010 | 0.0015 | 0.0001 |
| Water in the rumen | Spreading/Compost | 0.0000 | 0.0000 | 0.0000 |

Table 22: Total weighting by coproducts for Charolaise Heifers reared in Stall

| COPRODUCT | Destination | Charolaise/heifer/stall | | |
| --- | --- | --- | --- | --- |
|  |  | **Biophysical Cumulative share** | **Mass Cumulative share** | **Economic Cumulative share** |
| Abomasum | Human food | 0.0138 | 0.0026 | 0.0020 |
| Abomasum fat | Fat and greaves C3 | 0.0011 | 0.0005 | 0.0000 |
| Aponeurosis | Human food | 0.0255 | 0.0341 | 0.0342 |
| Bile | PAP C3 | 0.0000 | 0.0001 | 0.0000 |
| Blood | PAP C3 | 0.0180 | 0.0311 | 0.0070 |
| Blood | Pet food | 0.0026 | 0.0045 | 0.0003 |
| Bones | Gelatin C3 | 0.0498 | 0.0764 | 0.0002 |
| Bones of head, brain, eyes and teeth | C1-C2 for disposal | 0.0000 | 0.0000 | 0.0000 |
| Cheek | Human food | 0.0011 | 0.0016 | 0.0035 |
| Cheek | Human food | 0.0019 | 0.0029 | 0.0063 |
| Cheek trimmings | Pet food | 0.0005 | 0.0007 | 0.0001 |
| Chops | Pet food | 0.0020 | 0.0021 | 0.0002 |
| Contents of intestines | Spreading/Compost | 0.0000 | 0.0000 | 0.0000 |
| Contents of the rumen | Spreading/Compost | 0.0000 | 0.0000 | 0.0000 |
| Ears | PAP C3 | 0.0009 | 0.0010 | 0.0001 |
| Esophagus | Pet food | 0.0010 | 0.0015 | 0.0001 |
| Fat | Fat and greaves C3 | 0.0914 | 0.0818 | 0.0074 |
| Fat around heart | Fat and greaves C3 | 0.0032 | 0.0029 | 0.0003 |
| Fat in the kidney | Fat and greaves C3 | 0.0004 | 0.0004 | 0.0000 |
| Feet (without hooves) | Gelatin C3 | 0.0126 | 0.0187 | 0.0001 |
| Floatation fat | Spreading/Compost | 0.0000 | 0.0000 | 0.0000 |
| Forehead | C1-C2 for disposal | 0.0000 | 0.0000 | 0.0000 |
| Forelock | PAP C3 | 0.0027 | 0.0011 | 0.0001 |
| Gallbladder | Pet food | 0.0003 | 0.0005 | 0.0000 |
| Head trimmings | Pet food | 0.0027 | 0.0040 | 0.0003 |
| Heart | Human food | 0.0031 | 0.0047 | 0.0010 |
| Heart trimmings | Pet food | 0.0003 | 0.0005 | 0.0000 |
| Hide | Skin tannery C3 | 0.0694 | 0.0733 | 0.1223 |
| Hooves | PAP C3 | 0.0078 | 0.0032 | 0.0003 |
| Horns | PAP C3 | 0.0018 | 0.0007 | 0.0001 |
| Kidney | Human food | 0.0019 | 0.0029 | 0.0012 |
| Large intestine | C1-C2 for disposal | 0.0000 | 0.0000 | 0.0000 |
| Liver | Human food | 0.0527 | 0.0190 | 0.0092 |
| Liver trimmings | Pet food | 0.0061 | 0.0021 | 0.0002 |
| Lower jaw | PAP C3 | 0.0033 | 0.0052 | 0.0004 |
| Lungs | Pet food | 0.0082 | 0.0105 | 0.0008 |
| Mask | Skin tannery C3 | 0.0047 | 0.0050 | 0.0083 |
| Mesenteric fat | C1-C2 for disposal | 0.0000 | 0.0000 | 0.0000 |
| Muscle | Human food | 0.3149 | 0.4569 | 0.7640 |
| Muzzle | Human food | 0.0025 | 0.0026 | 0.0026 |
| Omasum | Human food | 0.0125 | 0.0024 | 0.0018 |
| Omasum fat | Fat and greaves C3 | 0.0036 | 0.0016 | 0.0001 |
| Rumen and forestomach | Human food | 0.0645 | 0.0121 | 0.0091 |
| Rumen fat | Fat and greaves C3 | 0.0052 | 0.0024 | 0.0002 |
| Sanitary seizures | C1-C2 for disposal | 0.0000 | 0.0000 | 0.0000 |
| Screening and sifting wastes | C1-C2 for disposal | 0.0000 | 0.0000 | 0.0000 |
| Small intestine | PAP C3 | 0.0750 | 0.0141 | 0.0009 |
| Spinal cord | C1-C2 for disposal | 0.0000 | 0.0000 | 0.0000 |
| Spinal cord waste | C1-C2 for disposal | 0.0000 | 0.0000 | 0.0000 |
| Spine | C1-C2 for disposal | 0.0000 | 0.0000 | 0.0000 |
| Spleen | Pet food | 0.0020 | 0.0029 | 0.0002 |
| Stillborn | PAP C3 | 0.0391 | 0.0071 | 0.0000 |
| Tallow | Fat and greaves C3 | 0.0811 | 0.0727 | 0.0066 |
| Tongue | Human food | 0.0026 | 0.0042 | 0.0067 |
| Tonsil | C1-C2 for disposal | 0.0000 | 0.0000 | 0.0000 |
| Trachea | Pet food | 0.0018 | 0.0019 | 0.0001 |
| Udder | Pet food | 0.0034 | 0.0222 | 0.0016 |
| Upper throat | Pet food | 0.0011 | 0.0016 | 0.0001 |
| Water in the rumen | Spreading/Compost | 0.0000 | 0.0000 | 0.0000 |

Table 23: Total weighting by coproducts for Charolaise Cull Cows reared in Stall

| COPRODUCT | Destination | Charolaise/Cull cow/stall | | |
| --- | --- | --- | --- | --- |
|  |  | **Biophysical Cumulative share** | **Mass Cumulative share** | **Economic Cumulative share** |
| Abomasum | Human food | 0.0150 | 0.0028 | 0.0021 |
| Abomasum fat | Fat and greaves C3 | 0.0011 | 0.0005 | 0.0000 |
| Aponeurosis | Human food | 0.0243 | 0.0331 | 0.0337 |
| Bile | PAP C3 | 0.0000 | 0.0001 | 0.0000 |
| Blood | PAP C3 | 0.0185 | 0.0328 | 0.0074 |
| Blood | Pet food | 0.0026 | 0.0046 | 0.0003 |
| Bones | Gelatin C3 | 0.0470 | 0.0743 | 0.0002 |
| Bones of head, brain, eyes and teeth | C1-C2 for disposal | 0.0000 | 0.0000 | 0.0000 |
| Cheek | Human food | 0.0011 | 0.0016 | 0.0036 |
| Cheek | Human food | 0.0020 | 0.0030 | 0.0067 |
| Cheek trimmings | Pet food | 0.0006 | 0.0009 | 0.0001 |
| Chops | Pet food | 0.0021 | 0.0023 | 0.0002 |
| Contents of intestines | Spreading/Compost | 0.0000 | 0.0000 | 0.0000 |
| Contents of the rumen | Spreading/Compost | 0.0000 | 0.0000 | 0.0000 |
| Ears | PAP C3 | 0.0009 | 0.0011 | 0.0001 |
| Esophagus | Pet food | 0.0010 | 0.0015 | 0.0001 |
| Fat | Fat and greaves C3 | 0.0841 | 0.0795 | 0.0073 |
| Fat around heart | Fat and greaves C3 | 0.0032 | 0.0030 | 0.0003 |
| Fat in the kidney | Fat and greaves C3 | 0.0004 | 0.0004 | 0.0000 |
| Feet (without hooves) | Gelatin C3 | 0.0130 | 0.0196 | 0.0001 |
| Floatation fat | Spreading/Compost | 0.0000 | 0.0000 | 0.0000 |
| Forehead | C1-C2 for disposal | 0.0000 | 0.0000 | 0.0000 |
| Forelock | PAP C3 | 0.0030 | 0.0013 | 0.0001 |
| Gallbladder | Pet food | 0.0003 | 0.0005 | 0.0000 |
| Head trimmings | Pet food | 0.0028 | 0.0041 | 0.0003 |
| Heart | Human food | 0.0032 | 0.0050 | 0.0011 |
| Heart trimmings | Pet food | 0.0003 | 0.0005 | 0.0000 |
| Hide | Skin tannery C3 | 0.0716 | 0.0771 | 0.1304 |
| Hooves | PAP C3 | 0.0081 | 0.0034 | 0.0003 |
| Horns | PAP C3 | 0.0021 | 0.0009 | 0.0001 |
| Kidney | Human food | 0.0020 | 0.0030 | 0.0012 |
| Large intestine | C1-C2 for disposal | 0.0000 | 0.0000 | 0.0000 |
| Liver | Human food | 0.0565 | 0.0199 | 0.0098 |
| Liver trimmings | Pet food | 0.0067 | 0.0023 | 0.0002 |
| Lower jaw | PAP C3 | 0.0034 | 0.0055 | 0.0005 |
| Lungs | Pet food | 0.0085 | 0.0111 | 0.0008 |
| Mask | Skin tannery C3 | 0.0049 | 0.0053 | 0.0089 |
| Mesenteric fat | C1-C2 for disposal | 0.0000 | 0.0000 | 0.0000 |
| Muscle | Human food | 0.2996 | 0.4440 | 0.7520 |
| Muzzle | Human food | 0.0026 | 0.0028 | 0.0028 |
| Omasum | Human food | 0.0137 | 0.0025 | 0.0019 |
| Omasum fat | Fat and greaves C3 | 0.0036 | 0.0016 | 0.0001 |
| Rumen and forestomach | Human food | 0.0697 | 0.0128 | 0.0097 |
| Rumen fat | Fat and greaves C3 | 0.0055 | 0.0025 | 0.0002 |
| Sanitary seizures | C1-C2 for disposal | 0.0000 | 0.0000 | 0.0000 |
| Screening and sifting wastes | C1-C2 for disposal | 0.0000 | 0.0000 | 0.0000 |
| Small intestine | PAP C3 | 0.0813 | 0.0149 | 0.0009 |
| Spinal cord | C1-C2 for disposal | 0.0000 | 0.0000 | 0.0000 |
| Spinal cord waste | C1-C2 for disposal | 0.0000 | 0.0000 | 0.0000 |
| Spine | C1-C2 for disposal | 0.0000 | 0.0000 | 0.0000 |
| Spleen | Pet food | 0.0020 | 0.0030 | 0.0002 |
| Stillborn | PAP C3 | 0.0421 | 0.0074 | 0.0000 |
| Tallow | Fat and greaves C3 | 0.0808 | 0.0765 | 0.0071 |
| Tongue | Human food | 0.0026 | 0.0044 | 0.0071 |
| Tonsil | C1-C2 for disposal | 0.0000 | 0.0000 | 0.0000 |
| Trachea | Pet food | 0.0017 | 0.0019 | 0.0001 |
| Udder | Pet food | 0.0034 | 0.0234 | 0.0017 |
| Upper throat | Pet food | 0.0011 | 0.0016 | 0.0001 |
| Water in the rumen | Spreading/Compost | 0.0000 | 0.0000 | 0.0000 |

Table 24: Total weighting by coproducts for Charolaise Beef reared in Stall

| COPRODUCT | Destination | Charolaise/beef/stall | | |
| --- | --- | --- | --- | --- |
|  |  | **Biophysical Cumulative share** | **Mass Cumulative share** | **Economic Cumulative share** |
| Abomasum | Human food | 0.0147 | 0.0026 | 0.0019 |
| Abomasum fat | Fat and greaves C3 | 0.0011 | 0.0005 | 0.0000 |
| Aponeurosis | Human food | 0.0258 | 0.0346 | 0.0345 |
| Bile | PAP C3 | 0.0000 | 0.0001 | 0.0000 |
| Blood | PAP C3 | 0.0174 | 0.0303 | 0.0067 |
| Blood | Pet food | 0.0025 | 0.0043 | 0.0003 |
| Bones | Gelatin C3 | 0.0498 | 0.0776 | 0.0002 |
| Bones of head, brain, eyes and teeth | C1-C2 for disposal | 0.0000 | 0.0000 | 0.0000 |
| Cheek | Human food | 0.0010 | 0.0015 | 0.0032 |
| Cheek | Human food | 0.0019 | 0.0028 | 0.0062 |
| Cheek trimmings | Pet food | 0.0005 | 0.0007 | 0.0001 |
| Chops | Pet food | 0.0019 | 0.0020 | 0.0001 |
| Contents of intestines | Spreading/Compost | 0.0000 | 0.0000 | 0.0000 |
| Contents of the rumen | Spreading/Compost | 0.0000 | 0.0000 | 0.0000 |
| Ears | PAP C3 | 0.0008 | 0.0010 | 0.0001 |
| Esophagus | Pet food | 0.0009 | 0.0014 | 0.0001 |
| Fat | Fat and greaves C3 | 0.0879 | 0.0830 | 0.0075 |
| Fat around heart | Fat and greaves C3 | 0.0030 | 0.0028 | 0.0003 |
| Fat in the kidney | Fat and greaves C3 | 0.0004 | 0.0004 | 0.0000 |
| Feet (without hooves) | Gelatin C3 | 0.0122 | 0.0182 | 0.0001 |
| Floatation fat | Spreading/Compost | 0.0000 | 0.0000 | 0.0000 |
| Forehead | C1-C2 for disposal | 0.0000 | 0.0000 | 0.0000 |
| Forelock | PAP C3 | 0.0027 | 0.0011 | 0.0001 |
| Gallbladder | Pet food | 0.0003 | 0.0005 | 0.0000 |
| Head trimmings | Pet food | 0.0026 | 0.0038 | 0.0003 |
| Heart | Human food | 0.0030 | 0.0046 | 0.0010 |
| Heart trimmings | Pet food | 0.0003 | 0.0005 | 0.0000 |
| Hide | Skin tannery C3 | 0.0675 | 0.0714 | 0.1184 |
| Hooves | PAP C3 | 0.0078 | 0.0032 | 0.0003 |
| Horns | PAP C3 | 0.0018 | 0.0007 | 0.0001 |
| Kidney | Human food | 0.0019 | 0.0028 | 0.0011 |
| Large intestine | C1-C2 for disposal | 0.0000 | 0.0000 | 0.0000 |
| Liver | Human food | 0.0542 | 0.0184 | 0.0089 |
| Liver trimmings | Pet food | 0.0061 | 0.0020 | 0.0001 |
| Lower jaw | PAP C3 | 0.0032 | 0.0051 | 0.0004 |
| Lungs | Pet food | 0.0080 | 0.0103 | 0.0007 |
| Mask | Skin tannery C3 | 0.0046 | 0.0048 | 0.0080 |
| Mesenteric fat | C1-C2 for disposal | 0.0000 | 0.0000 | 0.0000 |
| Muscle | Human food | 0.3182 | 0.4634 | 0.7699 |
| Muzzle | Human food | 0.0025 | 0.0026 | 0.0026 |
| Omasum | Human food | 0.0133 | 0.0023 | 0.0017 |
| Omasum fat | Fat and greaves C3 | 0.0033 | 0.0015 | 0.0001 |
| Rumen and forestomach | Human food | 0.0674 | 0.0119 | 0.0088 |
| Rumen fat | Fat and greaves C3 | 0.0053 | 0.0023 | 0.0002 |
| Sanitary seizures | C1-C2 for disposal | 0.0000 | 0.0000 | 0.0000 |
| Screening and sifting wastes | C1-C2 for disposal | 0.0000 | 0.0000 | 0.0000 |
| Small intestine | PAP C3 | 0.0779 | 0.0137 | 0.0008 |
| Spinal cord | C1-C2 for disposal | 0.0000 | 0.0000 | 0.0000 |
| Spinal cord waste | C1-C2 for disposal | 0.0000 | 0.0000 | 0.0000 |
| Spine | C1-C2 for disposal | 0.0000 | 0.0000 | 0.0000 |
| Spleen | Pet food | 0.0020 | 0.0028 | 0.0002 |
| Stillborn | PAP C3 | 0.0410 | 0.0069 | 0.0000 |
| Tallow | Fat and greaves C3 | 0.0748 | 0.0708 | 0.0064 |
| Tongue | Human food | 0.0025 | 0.0041 | 0.0065 |
| Tonsil | C1-C2 for disposal | 0.0000 | 0.0000 | 0.0000 |
| Trachea | Pet food | 0.0016 | 0.0017 | 0.0001 |
| Udder | Pet food | 0.0032 | 0.0216 | 0.0016 |
| Upper throat | Pet food | 0.0010 | 0.0015 | 0.0001 |
| Water in the rumen | Spreading/Compost | 0.0000 | 0.0000 | 0.0000 |

Table 25: Total weighting by coproducts for Primholstein Young Bulls reared in Grazing Large Area

| COPRODUCT | Destination | Primholstein/young bull/grazing large area | | |
| --- | --- | --- | --- | --- |
|  |  | **Biophysical Cumulative share** | **Mass Cumulative share** | **Economic Cumulative share** |
| Abomasum | Human food | 0.0157 | 0.0028 | 0.0021 |
| Abomasum fat | Fat and greaves C3 | 0.0011 | 0.0005 | 0.0000 |
| Aponeurosis | Human food | 0.0245 | 0.0326 | 0.0334 |
| Bile | PAP C3 | 0.0000 | 0.0001 | 0.0000 |
| Blood | PAP C3 | 0.0194 | 0.0335 | 0.0076 |
| Blood | Pet food | 0.0028 | 0.0048 | 0.0004 |
| Bones | Gelatin C3 | 0.0454 | 0.0732 | 0.0002 |
| Bones of head, brain, eyes and teeth | C1-C2 for disposal | 0.0000 | 0.0000 | 0.0000 |
| Cheek | Human food | 0.0011 | 0.0016 | 0.0037 |
| Cheek | Human food | 0.0021 | 0.0031 | 0.0070 |
| Cheek trimmings | Pet food | 0.0006 | 0.0009 | 0.0001 |
| Chops | Pet food | 0.0021 | 0.0023 | 0.0002 |
| Contents of intestines | Spreading/Compost | 0.0000 | 0.0000 | 0.0000 |
| Contents of the rumen | Spreading/Compost | 0.0000 | 0.0000 | 0.0000 |
| Ears | PAP C3 | 0.0010 | 0.0011 | 0.0001 |
| Esophagus | Pet food | 0.0010 | 0.0015 | 0.0001 |
| Fat | Fat and greaves C3 | 0.0713 | 0.0783 | 0.0073 |
| Fat around heart | Fat and greaves C3 | 0.0028 | 0.0031 | 0.0003 |
| Fat in the kidney | Fat and greaves C3 | 0.0003 | 0.0004 | 0.0000 |
| Feet (without hooves) | Gelatin C3 | 0.0135 | 0.0202 | 0.0001 |
| Floatation fat | Spreading/Compost | 0.0000 | 0.0000 | 0.0000 |
| Forehead | C1-C2 for disposal | 0.0000 | 0.0000 | 0.0000 |
| Forelock | PAP C3 | 0.0031 | 0.0013 | 0.0001 |
| Gallbladder | Pet food | 0.0003 | 0.0005 | 0.0000 |
| Head trimmings | Pet food | 0.0029 | 0.0043 | 0.0003 |
| Heart | Human food | 0.0033 | 0.0050 | 0.0011 |
| Heart trimmings | Pet food | 0.0003 | 0.0005 | 0.0000 |
| Hide | Skin tannery C3 | 0.0752 | 0.0791 | 0.1346 |
| Hooves | PAP C3 | 0.0086 | 0.0035 | 0.0003 |
| Horns | PAP C3 | 0.0022 | 0.0009 | 0.0001 |
| Kidney | Human food | 0.0021 | 0.0031 | 0.0013 |
| Large intestine | C1-C2 for disposal | 0.0000 | 0.0000 | 0.0000 |
| Liver | Human food | 0.0601 | 0.0205 | 0.0101 |
| Liver trimmings | Pet food | 0.0069 | 0.0023 | 0.0002 |
| Lower jaw | PAP C3 | 0.0034 | 0.0056 | 0.0005 |
| Lungs | Pet food | 0.0090 | 0.0114 | 0.0009 |
| Mask | Skin tannery C3 | 0.0051 | 0.0054 | 0.0092 |
| Mesenteric fat | C1-C2 for disposal | 0.0000 | 0.0000 | 0.0000 |
| Muscle | Human food | 0.2991 | 0.4374 | 0.7457 |
| Muzzle | Human food | 0.0026 | 0.0028 | 0.0028 |
| Omasum | Human food | 0.0142 | 0.0025 | 0.0019 |
| Omasum fat | Fat and greaves C3 | 0.0035 | 0.0016 | 0.0002 |
| Rumen and forestomach | Human food | 0.0740 | 0.0131 | 0.0100 |
| Rumen fat | Fat and greaves C3 | 0.0053 | 0.0025 | 0.0002 |
| Sanitary seizures | C1-C2 for disposal | 0.0000 | 0.0000 | 0.0000 |
| Screening and sifting wastes | C1-C2 for disposal | 0.0000 | 0.0000 | 0.0000 |
| Small intestine | PAP C3 | 0.0861 | 0.0152 | 0.0009 |
| Spinal cord | C1-C2 for disposal | 0.0000 | 0.0000 | 0.0000 |
| Spinal cord waste | C1-C2 for disposal | 0.0000 | 0.0000 | 0.0000 |
| Spine | C1-C2 for disposal | 0.0000 | 0.0000 | 0.0000 |
| Spleen | Pet food | 0.0022 | 0.0031 | 0.0002 |
| Stillborn | PAP C3 | 0.0454 | 0.0077 | 0.0000 |
| Tallow | Fat and greaves C3 | 0.0712 | 0.0785 | 0.0073 |
| Tongue | Human food | 0.0027 | 0.0045 | 0.0073 |
| Tonsil | C1-C2 for disposal | 0.0000 | 0.0000 | 0.0000 |
| Trachea | Pet food | 0.0019 | 0.0020 | 0.0002 |
| Udder | Pet food | 0.0034 | 0.0240 | 0.0018 |
| Upper throat | Pet food | 0.0011 | 0.0016 | 0.0001 |
| Water in the rumen | Spreading/Compost | 0.0000 | 0.0000 | 0.0000 |

Table 26: Total weighting by coproducts for Primholstein Heifers reared in Grazing Large Area

| COPRODUCT | Destination | Primholstein/heifer/grazing large area | | |
| --- | --- | --- | --- | --- |
|  |  | **Biophysical Cumulative share** | **Mass Cumulative share** | **Economic Cumulative share** |
| Abomasum | Human food | 0.0164 | 0.0030 | 0.0024 |
| Abomasum fat | Fat and greaves C3 | 0.0013 | 0.0006 | 0.0001 |
| Aponeurosis | Human food | 0.0232 | 0.0311 | 0.0325 |
| Bile | PAP C3 | 0.0000 | 0.0001 | 0.0000 |
| Blood | PAP C3 | 0.0208 | 0.0361 | 0.0084 |
| Blood | Pet food | 0.0030 | 0.0052 | 0.0004 |
| Bones | Gelatin C3 | 0.0430 | 0.0697 | 0.0002 |
| Bones of head, brain, eyes and teeth | C1-C2 for disposal | 0.0000 | 0.0000 | 0.0000 |
| Cheek | Human food | 0.0012 | 0.0018 | 0.0041 |
| Cheek | Human food | 0.0022 | 0.0033 | 0.0076 |
| Cheek trimmings | Pet food | 0.0006 | 0.0009 | 0.0001 |
| Chops | Pet food | 0.0023 | 0.0024 | 0.0002 |
| Contents of intestines | Spreading/Compost | 0.0000 | 0.0000 | 0.0000 |
| Contents of the rumen | Spreading/Compost | 0.0000 | 0.0000 | 0.0000 |
| Ears | PAP C3 | 0.0011 | 0.0013 | 0.0001 |
| Esophagus | Pet food | 0.0011 | 0.0017 | 0.0001 |
| Fat | Fat and greaves C3 | 0.0669 | 0.0747 | 0.0071 |
| Fat around heart | Fat and greaves C3 | 0.0030 | 0.0033 | 0.0003 |
| Fat in the kidney | Fat and greaves C3 | 0.0005 | 0.0005 | 0.0000 |
| Feet (without hooves) | Gelatin C3 | 0.0145 | 0.0217 | 0.0001 |
| Floatation fat | Spreading/Compost | 0.0000 | 0.0000 | 0.0000 |
| Forehead | C1-C2 for disposal | 0.0000 | 0.0000 | 0.0000 |
| Forelock | PAP C3 | 0.0034 | 0.0014 | 0.0001 |
| Gallbladder | Pet food | 0.0004 | 0.0006 | 0.0000 |
| Head trimmings | Pet food | 0.0031 | 0.0046 | 0.0003 |
| Heart | Human food | 0.0035 | 0.0055 | 0.0012 |
| Heart trimmings | Pet food | 0.0004 | 0.0006 | 0.0000 |
| Hide | Skin tannery C3 | 0.0806 | 0.0851 | 0.1479 |
| Hooves | PAP C3 | 0.0093 | 0.0038 | 0.0003 |
| Horns | PAP C3 | 0.0022 | 0.0009 | 0.0001 |
| Kidney | Human food | 0.0022 | 0.0033 | 0.0014 |
| Large intestine | C1-C2 for disposal | 0.0000 | 0.0000 | 0.0000 |
| Liver | Human food | 0.0616 | 0.0220 | 0.0111 |
| Liver trimmings | Pet food | 0.0070 | 0.0024 | 0.0002 |
| Lower jaw | PAP C3 | 0.0037 | 0.0061 | 0.0005 |
| Lungs | Pet food | 0.0096 | 0.0123 | 0.0009 |
| Mask | Skin tannery C3 | 0.0054 | 0.0057 | 0.0099 |
| Mesenteric fat | C1-C2 for disposal | 0.0000 | 0.0000 | 0.0000 |
| Muscle | Human food | 0.2835 | 0.4171 | 0.7259 |
| Muzzle | Human food | 0.0029 | 0.0030 | 0.0032 |
| Omasum | Human food | 0.0143 | 0.0027 | 0.0021 |
| Omasum fat | Fat and greaves C3 | 0.0036 | 0.0018 | 0.0002 |
| Rumen and forestomach | Human food | 0.0758 | 0.0141 | 0.0110 |
| Rumen fat | Fat and greaves C3 | 0.0054 | 0.0027 | 0.0003 |
| Sanitary seizures | C1-C2 for disposal | 0.0000 | 0.0000 | 0.0000 |
| Screening and sifting wastes | C1-C2 for disposal | 0.0000 | 0.0000 | 0.0000 |
| Small intestine | PAP C3 | 0.0881 | 0.0164 | 0.0010 |
| Spinal cord | C1-C2 for disposal | 0.0000 | 0.0000 | 0.0000 |
| Spinal cord waste | C1-C2 for disposal | 0.0000 | 0.0000 | 0.0000 |
| Spine | C1-C2 for disposal | 0.0000 | 0.0000 | 0.0000 |
| Spleen | Pet food | 0.0023 | 0.0033 | 0.0003 |
| Stillborn | PAP C3 | 0.0456 | 0.0081 | 0.0000 |
| Tallow | Fat and greaves C3 | 0.0754 | 0.0844 | 0.0080 |
| Tongue | Human food | 0.0028 | 0.0048 | 0.0080 |
| Tonsil | C1-C2 for disposal | 0.0000 | 0.0000 | 0.0000 |
| Trachea | Pet food | 0.0020 | 0.0022 | 0.0002 |
| Udder | Pet food | 0.0036 | 0.0258 | 0.0020 |
| Upper throat | Pet food | 0.0012 | 0.0018 | 0.0001 |
| Water in the rumen | Spreading/Compost | 0.0000 | 0.0000 | 0.0000 |

Table 27: Total weighting by coproducts for Primholstein Cull Cows reared in Grazing Large Area

| COPRODUCT | Destination | Primholstein/Cull cow/grazing large area | | |
| --- | --- | --- | --- | --- |
|  |  | **Biophysical Cumulative share** | **Mass Cumulative share** | **Economic Cumulative share** |
| Abomasum | Human food | 0.0167 | 0.0031 | 0.0024 |
| Abomasum fat | Fat and greaves C3 | 0.0013 | 0.0006 | 0.0001 |
| Aponeurosis | Human food | 0.0225 | 0.0306 | 0.0322 |
| Bile | PAP C3 | 0.0000 | 0.0001 | 0.0000 |
| Blood | PAP C3 | 0.0209 | 0.0370 | 0.0087 |
| Blood | Pet food | 0.0030 | 0.0052 | 0.0004 |
| Bones | Gelatin C3 | 0.0416 | 0.0686 | 0.0002 |
| Bones of head, brain, eyes and teeth | C1-C2 for disposal | 0.0000 | 0.0000 | 0.0000 |
| Cheek | Human food | 0.0013 | 0.0019 | 0.0044 |
| Cheek | Human food | 0.0023 | 0.0034 | 0.0079 |
| Cheek trimmings | Pet food | 0.0006 | 0.0009 | 0.0001 |
| Chops | Pet food | 0.0023 | 0.0024 | 0.0002 |
| Contents of intestines | Spreading/Compost | 0.0000 | 0.0000 | 0.0000 |
| Contents of the rumen | Spreading/Compost | 0.0000 | 0.0000 | 0.0000 |
| Ears | PAP C3 | 0.0010 | 0.0013 | 0.0001 |
| Esophagus | Pet food | 0.0011 | 0.0017 | 0.0001 |
| Fat | Fat and greaves C3 | 0.0648 | 0.0735 | 0.0070 |
| Fat around heart | Fat and greaves C3 | 0.0030 | 0.0034 | 0.0003 |
| Fat in the kidney | Fat and greaves C3 | 0.0004 | 0.0005 | 0.0000 |
| Feet (without hooves) | Gelatin C3 | 0.0145 | 0.0222 | 0.0001 |
| Floatation fat | Spreading/Compost | 0.0000 | 0.0000 | 0.0000 |
| Forehead | C1-C2 for disposal | 0.0000 | 0.0000 | 0.0000 |
| Forelock | PAP C3 | 0.0034 | 0.0014 | 0.0001 |
| Gallbladder | Pet food | 0.0004 | 0.0006 | 0.0000 |
| Head trimmings | Pet food | 0.0031 | 0.0047 | 0.0004 |
| Heart | Human food | 0.0036 | 0.0056 | 0.0012 |
| Heart trimmings | Pet food | 0.0004 | 0.0006 | 0.0000 |
| Hide | Skin tannery C3 | 0.0811 | 0.0871 | 0.1524 |
| Hooves | PAP C3 | 0.0092 | 0.0038 | 0.0003 |
| Horns | PAP C3 | 0.0021 | 0.0009 | 0.0001 |
| Kidney | Human food | 0.0023 | 0.0034 | 0.0015 |
| Large intestine | C1-C2 for disposal | 0.0000 | 0.0000 | 0.0000 |
| Liver | Human food | 0.0635 | 0.0224 | 0.0114 |
| Liver trimmings | Pet food | 0.0071 | 0.0024 | 0.0002 |
| Lower jaw | PAP C3 | 0.0037 | 0.0063 | 0.0006 |
| Lungs | Pet food | 0.0097 | 0.0126 | 0.0010 |
| Mask | Skin tannery C3 | 0.0055 | 0.0059 | 0.0103 |
| Mesenteric fat | C1-C2 for disposal | 0.0000 | 0.0000 | 0.0000 |
| Muscle | Human food | 0.2744 | 0.4102 | 0.7188 |
| Muzzle | Human food | 0.0028 | 0.0031 | 0.0032 |
| Omasum | Human food | 0.0153 | 0.0028 | 0.0022 |
| Omasum fat | Fat and greaves C3 | 0.0039 | 0.0019 | 0.0002 |
| Rumen and forestomach | Human food | 0.0784 | 0.0144 | 0.0113 |
| Rumen fat | Fat and greaves C3 | 0.0057 | 0.0028 | 0.0003 |
| Sanitary seizures | C1-C2 for disposal | 0.0000 | 0.0000 | 0.0000 |
| Screening and sifting wastes | C1-C2 for disposal | 0.0000 | 0.0000 | 0.0000 |
| Small intestine | PAP C3 | 0.0909 | 0.0167 | 0.0011 |
| Spinal cord | C1-C2 for disposal | 0.0000 | 0.0000 | 0.0000 |
| Spinal cord waste | C1-C2 for disposal | 0.0000 | 0.0000 | 0.0000 |
| Spine | C1-C2 for disposal | 0.0000 | 0.0000 | 0.0000 |
| Spleen | Pet food | 0.0023 | 0.0034 | 0.0003 |
| Stillborn | PAP C3 | 0.0479 | 0.0084 | 0.0000 |
| Tallow | Fat and greaves C3 | 0.0760 | 0.0864 | 0.0082 |
| Tongue | Human food | 0.0029 | 0.0050 | 0.0083 |
| Tonsil | C1-C2 for disposal | 0.0000 | 0.0000 | 0.0000 |
| Trachea | Pet food | 0.0020 | 0.0022 | 0.0002 |
| Udder | Pet food | 0.0036 | 0.0264 | 0.0020 |
| Upper throat | Pet food | 0.0013 | 0.0019 | 0.0001 |
| Water in the rumen | Spreading/Compost | 0.0000 | 0.0000 | 0.0000 |

Table 28: Total weighting by coproducts for Primholstein Beef reared in Grazing Large Area

| COPRODUCT | Destination | Primholstein/beef/grazing large area | | |
| --- | --- | --- | --- | --- |
|  |  | **Biophysical Cumulative share** | **Mass Cumulative share** | **Economic Cumulative share** |
| Abomasum | Human food | 0.0162 | 0.0029 | 0.0022 |
| Abomasum fat | Fat and greaves C3 | 0.0013 | 0.0006 | 0.0001 |
| Aponeurosis | Human food | 0.0239 | 0.0321 | 0.0331 |
| Bile | PAP C3 | 0.0000 | 0.0001 | 0.0000 |
| Blood | PAP C3 | 0.0197 | 0.0344 | 0.0079 |
| Blood | Pet food | 0.0028 | 0.0049 | 0.0004 |
| Bones | Gelatin C3 | 0.0444 | 0.0721 | 0.0002 |
| Bones of head, brain, eyes and teeth | C1-C2 for disposal | 0.0000 | 0.0000 | 0.0000 |
| Cheek | Human food | 0.0012 | 0.0018 | 0.0040 |
| Cheek | Human food | 0.0021 | 0.0031 | 0.0071 |
| Cheek trimmings | Pet food | 0.0006 | 0.0009 | 0.0001 |
| Chops | Pet food | 0.0021 | 0.0023 | 0.0002 |
| Contents of intestines | Spreading/Compost | 0.0000 | 0.0000 | 0.0000 |
| Contents of the rumen | Spreading/Compost | 0.0000 | 0.0000 | 0.0000 |
| Ears | PAP C3 | 0.0009 | 0.0011 | 0.0001 |
| Esophagus | Pet food | 0.0011 | 0.0016 | 0.0001 |
| Fat | Fat and greaves C3 | 0.0695 | 0.0771 | 0.0072 |
| Fat around heart | Fat and greaves C3 | 0.0028 | 0.0031 | 0.0003 |
| Fat in the kidney | Fat and greaves C3 | 0.0003 | 0.0004 | 0.0000 |
| Feet (without hooves) | Gelatin C3 | 0.0137 | 0.0207 | 0.0001 |
| Floatation fat | Spreading/Compost | 0.0000 | 0.0000 | 0.0000 |
| Forehead | C1-C2 for disposal | 0.0000 | 0.0000 | 0.0000 |
| Forelock | PAP C3 | 0.0031 | 0.0013 | 0.0001 |
| Gallbladder | Pet food | 0.0004 | 0.0006 | 0.0000 |
| Head trimmings | Pet food | 0.0029 | 0.0043 | 0.0003 |
| Heart | Human food | 0.0033 | 0.0052 | 0.0011 |
| Heart trimmings | Pet food | 0.0004 | 0.0006 | 0.0000 |
| Hide | Skin tannery C3 | 0.0765 | 0.0810 | 0.1388 |
| Hooves | PAP C3 | 0.0089 | 0.0037 | 0.0003 |
| Horns | PAP C3 | 0.0021 | 0.0009 | 0.0001 |
| Kidney | Human food | 0.0021 | 0.0031 | 0.0013 |
| Large intestine | C1-C2 for disposal | 0.0000 | 0.0000 | 0.0000 |
| Liver | Human food | 0.0607 | 0.0209 | 0.0104 |
| Liver trimmings | Pet food | 0.0069 | 0.0023 | 0.0002 |
| Lower jaw | PAP C3 | 0.0035 | 0.0058 | 0.0005 |
| Lungs | Pet food | 0.0091 | 0.0117 | 0.0009 |
| Mask | Skin tannery C3 | 0.0052 | 0.0055 | 0.0095 |
| Mesenteric fat | C1-C2 for disposal | 0.0000 | 0.0000 | 0.0000 |
| Muscle | Human food | 0.2923 | 0.4305 | 0.7390 |
| Muzzle | Human food | 0.0027 | 0.0029 | 0.0030 |
| Omasum | Human food | 0.0148 | 0.0026 | 0.0020 |
| Omasum fat | Fat and greaves C3 | 0.0037 | 0.0018 | 0.0002 |
| Rumen and forestomach | Human food | 0.0755 | 0.0135 | 0.0104 |
| Rumen fat | Fat and greaves C3 | 0.0056 | 0.0026 | 0.0002 |
| Sanitary seizures | C1-C2 for disposal | 0.0000 | 0.0000 | 0.0000 |
| Screening and sifting wastes | C1-C2 for disposal | 0.0000 | 0.0000 | 0.0000 |
| Small intestine | PAP C3 | 0.0875 | 0.0156 | 0.0010 |
| Spinal cord | C1-C2 for disposal | 0.0000 | 0.0000 | 0.0000 |
| Spinal cord waste | C1-C2 for disposal | 0.0000 | 0.0000 | 0.0000 |
| Spine | C1-C2 for disposal | 0.0000 | 0.0000 | 0.0000 |
| Spleen | Pet food | 0.0022 | 0.0031 | 0.0002 |
| Stillborn | PAP C3 | 0.0457 | 0.0078 | 0.0000 |
| Tallow | Fat and greaves C3 | 0.0724 | 0.0804 | 0.0075 |
| Tongue | Human food | 0.0027 | 0.0047 | 0.0076 |
| Tonsil | C1-C2 for disposal | 0.0000 | 0.0000 | 0.0000 |
| Trachea | Pet food | 0.0019 | 0.0020 | 0.0002 |
| Udder | Pet food | 0.0034 | 0.0246 | 0.0019 |
| Upper throat | Pet food | 0.0012 | 0.0018 | 0.0001 |
| Water in the rumen | Spreading/Compost | 0.0000 | 0.0000 | 0.0000 |

Table 29: Total weighting by coproducts for Primholstein Young Bulls reared in Pasture

| COPRODUCT | Destination | Primholstein/young bull/pasture | | |
| --- | --- | --- | --- | --- |
|  |  | **Biophysical Cumulative share** | **Mass Cumulative share** | **Economic Cumulative share** |
| Abomasum | Human food | 0.0150 | 0.0028 | 0.0021 |
| Abomasum fat | Fat and greaves C3 | 0.0011 | 0.0005 | 0.0000 |
| Aponeurosis | Human food | 0.0243 | 0.0326 | 0.0334 |
| Bile | PAP C3 | 0.0000 | 0.0001 | 0.0000 |
| Blood | PAP C3 | 0.0192 | 0.0335 | 0.0076 |
| Blood | Pet food | 0.0027 | 0.0048 | 0.0004 |
| Bones | Gelatin C3 | 0.0462 | 0.0732 | 0.0002 |
| Bones of head, brain, eyes and teeth | C1-C2 for disposal | 0.0000 | 0.0000 | 0.0000 |
| Cheek | Human food | 0.0011 | 0.0016 | 0.0037 |
| Cheek | Human food | 0.0021 | 0.0031 | 0.0070 |
| Cheek trimmings | Pet food | 0.0006 | 0.0009 | 0.0001 |
| Chops | Pet food | 0.0021 | 0.0023 | 0.0002 |
| Contents of intestines | Spreading/Compost | 0.0000 | 0.0000 | 0.0000 |
| Contents of the rumen | Spreading/Compost | 0.0000 | 0.0000 | 0.0000 |
| Ears | PAP C3 | 0.0010 | 0.0011 | 0.0001 |
| Esophagus | Pet food | 0.0010 | 0.0015 | 0.0001 |
| Fat | Fat and greaves C3 | 0.0782 | 0.0783 | 0.0073 |
| Fat around heart | Fat and greaves C3 | 0.0031 | 0.0031 | 0.0003 |
| Fat in the kidney | Fat and greaves C3 | 0.0004 | 0.0004 | 0.0000 |
| Feet (without hooves) | Gelatin C3 | 0.0135 | 0.0202 | 0.0001 |
| Floatation fat | Spreading/Compost | 0.0000 | 0.0000 | 0.0000 |
| Forehead | C1-C2 for disposal | 0.0000 | 0.0000 | 0.0000 |
| Forelock | PAP C3 | 0.0030 | 0.0013 | 0.0001 |
| Gallbladder | Pet food | 0.0003 | 0.0005 | 0.0000 |
| Head trimmings | Pet food | 0.0029 | 0.0043 | 0.0003 |
| Heart | Human food | 0.0032 | 0.0050 | 0.0011 |
| Heart trimmings | Pet food | 0.0003 | 0.0005 | 0.0000 |
| Hide | Skin tannery C3 | 0.0746 | 0.0791 | 0.1346 |
| Hooves | PAP C3 | 0.0085 | 0.0035 | 0.0003 |
| Horns | PAP C3 | 0.0021 | 0.0009 | 0.0001 |
| Kidney | Human food | 0.0021 | 0.0031 | 0.0013 |
| Large intestine | C1-C2 for disposal | 0.0000 | 0.0000 | 0.0000 |
| Liver | Human food | 0.0578 | 0.0205 | 0.0101 |
| Liver trimmings | Pet food | 0.0066 | 0.0023 | 0.0002 |
| Lower jaw | PAP C3 | 0.0035 | 0.0056 | 0.0005 |
| Lungs | Pet food | 0.0089 | 0.0114 | 0.0009 |
| Mask | Skin tannery C3 | 0.0051 | 0.0054 | 0.0092 |
| Mesenteric fat | C1-C2 for disposal | 0.0000 | 0.0000 | 0.0000 |
| Muscle | Human food | 0.2981 | 0.4374 | 0.7457 |
| Muzzle | Human food | 0.0026 | 0.0028 | 0.0028 |
| Omasum | Human food | 0.0136 | 0.0025 | 0.0019 |
| Omasum fat | Fat and greaves C3 | 0.0035 | 0.0016 | 0.0002 |
| Rumen and forestomach | Human food | 0.0708 | 0.0131 | 0.0100 |
| Rumen fat | Fat and greaves C3 | 0.0054 | 0.0025 | 0.0002 |
| Sanitary seizures | C1-C2 for disposal | 0.0000 | 0.0000 | 0.0000 |
| Screening and sifting wastes | C1-C2 for disposal | 0.0000 | 0.0000 | 0.0000 |
| Small intestine | PAP C3 | 0.0824 | 0.0152 | 0.0009 |
| Spinal cord | C1-C2 for disposal | 0.0000 | 0.0000 | 0.0000 |
| Spinal cord waste | C1-C2 for disposal | 0.0000 | 0.0000 | 0.0000 |
| Spine | C1-C2 for disposal | 0.0000 | 0.0000 | 0.0000 |
| Spleen | Pet food | 0.0022 | 0.0031 | 0.0002 |
| Stillborn | PAP C3 | 0.0433 | 0.0077 | 0.0000 |
| Tallow | Fat and greaves C3 | 0.0782 | 0.0785 | 0.0073 |
| Tongue | Human food | 0.0027 | 0.0045 | 0.0073 |
| Tonsil | C1-C2 for disposal | 0.0000 | 0.0000 | 0.0000 |
| Trachea | Pet food | 0.0019 | 0.0020 | 0.0002 |
| Udder | Pet food | 0.0035 | 0.0240 | 0.0018 |
| Upper throat | Pet food | 0.0011 | 0.0016 | 0.0001 |
| Water in the rumen | Spreading/Compost | 0.0000 | 0.0000 | 0.0000 |

Table 30: Total weighting by coproducts for Primholstein Heifers reared in Pasture

| COPRODUCT | Destination | Primholstein/heifer/pasture | | |
| --- | --- | --- | --- | --- |
|  |  | **Biophysical Cumulative share** | **Mass Cumulative share** | **Economic Cumulative share** |
| Abomasum | Human food | 0.0156 | 0.0030 | 0.0024 |
| Abomasum fat | Fat and greaves C3 | 0.0013 | 0.0006 | 0.0001 |
| Aponeurosis | Human food | 0.0231 | 0.0311 | 0.0325 |
| Bile | PAP C3 | 0.0000 | 0.0001 | 0.0000 |
| Blood | PAP C3 | 0.0207 | 0.0361 | 0.0084 |
| Blood | Pet food | 0.0030 | 0.0052 | 0.0004 |
| Bones | Gelatin C3 | 0.0437 | 0.0697 | 0.0002 |
| Bones of head, brain, eyes and teeth | C1-C2 for disposal | 0.0000 | 0.0000 | 0.0000 |
| Cheek | Human food | 0.0012 | 0.0018 | 0.0041 |
| Cheek | Human food | 0.0022 | 0.0033 | 0.0076 |
| Cheek trimmings | Pet food | 0.0006 | 0.0009 | 0.0001 |
| Chops | Pet food | 0.0023 | 0.0024 | 0.0002 |
| Contents of intestines | Spreading/Compost | 0.0000 | 0.0000 | 0.0000 |
| Contents of the rumen | Spreading/Compost | 0.0000 | 0.0000 | 0.0000 |
| Ears | PAP C3 | 0.0011 | 0.0013 | 0.0001 |
| Esophagus | Pet food | 0.0011 | 0.0017 | 0.0001 |
| Fat | Fat and greaves C3 | 0.0732 | 0.0747 | 0.0071 |
| Fat around heart | Fat and greaves C3 | 0.0032 | 0.0033 | 0.0003 |
| Fat in the kidney | Fat and greaves C3 | 0.0005 | 0.0005 | 0.0000 |
| Feet (without hooves) | Gelatin C3 | 0.0145 | 0.0217 | 0.0001 |
| Floatation fat | Spreading/Compost | 0.0000 | 0.0000 | 0.0000 |
| Forehead | C1-C2 for disposal | 0.0000 | 0.0000 | 0.0000 |
| Forelock | PAP C3 | 0.0034 | 0.0014 | 0.0001 |
| Gallbladder | Pet food | 0.0004 | 0.0006 | 0.0000 |
| Head trimmings | Pet food | 0.0031 | 0.0046 | 0.0003 |
| Heart | Human food | 0.0035 | 0.0055 | 0.0012 |
| Heart trimmings | Pet food | 0.0004 | 0.0006 | 0.0000 |
| Hide | Skin tannery C3 | 0.0802 | 0.0851 | 0.1479 |
| Hooves | PAP C3 | 0.0092 | 0.0038 | 0.0003 |
| Horns | PAP C3 | 0.0022 | 0.0009 | 0.0001 |
| Kidney | Human food | 0.0022 | 0.0033 | 0.0014 |
| Large intestine | C1-C2 for disposal | 0.0000 | 0.0000 | 0.0000 |
| Liver | Human food | 0.0591 | 0.0220 | 0.0111 |
| Liver trimmings | Pet food | 0.0068 | 0.0024 | 0.0002 |
| Lower jaw | PAP C3 | 0.0038 | 0.0061 | 0.0005 |
| Lungs | Pet food | 0.0096 | 0.0123 | 0.0009 |
| Mask | Skin tannery C3 | 0.0054 | 0.0057 | 0.0099 |
| Mesenteric fat | C1-C2 for disposal | 0.0000 | 0.0000 | 0.0000 |
| Muscle | Human food | 0.2833 | 0.4171 | 0.7259 |
| Muzzle | Human food | 0.0029 | 0.0030 | 0.0032 |
| Omasum | Human food | 0.0137 | 0.0027 | 0.0021 |
| Omasum fat | Fat and greaves C3 | 0.0036 | 0.0018 | 0.0002 |
| Rumen and forestomach | Human food | 0.0723 | 0.0141 | 0.0110 |
| Rumen fat | Fat and greaves C3 | 0.0055 | 0.0027 | 0.0003 |
| Sanitary seizures | C1-C2 for disposal | 0.0000 | 0.0000 | 0.0000 |
| Screening and sifting wastes | C1-C2 for disposal | 0.0000 | 0.0000 | 0.0000 |
| Small intestine | PAP C3 | 0.0841 | 0.0164 | 0.0010 |
| Spinal cord | C1-C2 for disposal | 0.0000 | 0.0000 | 0.0000 |
| Spinal cord waste | C1-C2 for disposal | 0.0000 | 0.0000 | 0.0000 |
| Spine | C1-C2 for disposal | 0.0000 | 0.0000 | 0.0000 |
| Spleen | Pet food | 0.0023 | 0.0033 | 0.0003 |
| Stillborn | PAP C3 | 0.0435 | 0.0081 | 0.0000 |
| Tallow | Fat and greaves C3 | 0.0825 | 0.0844 | 0.0080 |
| Tongue | Human food | 0.0029 | 0.0048 | 0.0080 |
| Tonsil | C1-C2 for disposal | 0.0000 | 0.0000 | 0.0000 |
| Trachea | Pet food | 0.0020 | 0.0022 | 0.0002 |
| Udder | Pet food | 0.0037 | 0.0258 | 0.0020 |
| Upper throat | Pet food | 0.0012 | 0.0018 | 0.0001 |
| Water in the rumen | Spreading/Compost | 0.0000 | 0.0000 | 0.0000 |

Table 31: Total weighting by coproducts for Primholstein Cull Cows reared in Pasture

| COPRODUCT | Destination | Primholstein/Cull cow/pasture | | |
| --- | --- | --- | --- | --- |
|  |  | **Biophysical Cumulative share** | **Mass Cumulative share** | **Economic Cumulative share** |
| Abomasum | Human food | 0.0159 | 0.0031 | 0.0024 |
| Abomasum fat | Fat and greaves C3 | 0.0013 | 0.0006 | 0.0001 |
| Aponeurosis | Human food | 0.0223 | 0.0306 | 0.0322 |
| Bile | PAP C3 | 0.0000 | 0.0001 | 0.0000 |
| Blood | PAP C3 | 0.0208 | 0.0370 | 0.0087 |
| Blood | Pet food | 0.0029 | 0.0052 | 0.0004 |
| Bones | Gelatin C3 | 0.0423 | 0.0686 | 0.0002 |
| Bones of head, brain, eyes and teeth | C1-C2 for disposal | 0.0000 | 0.0000 | 0.0000 |
| Cheek | Human food | 0.0013 | 0.0019 | 0.0044 |
| Cheek | Human food | 0.0023 | 0.0034 | 0.0079 |
| Cheek trimmings | Pet food | 0.0006 | 0.0009 | 0.0001 |
| Chops | Pet food | 0.0022 | 0.0024 | 0.0002 |
| Contents of intestines | Spreading/Compost | 0.0000 | 0.0000 | 0.0000 |
| Contents of the rumen | Spreading/Compost | 0.0000 | 0.0000 | 0.0000 |
| Ears | PAP C3 | 0.0011 | 0.0013 | 0.0001 |
| Esophagus | Pet food | 0.0011 | 0.0017 | 0.0001 |
| Fat | Fat and greaves C3 | 0.0711 | 0.0735 | 0.0070 |
| Fat around heart | Fat and greaves C3 | 0.0033 | 0.0034 | 0.0003 |
| Fat in the kidney | Fat and greaves C3 | 0.0005 | 0.0005 | 0.0000 |
| Feet (without hooves) | Gelatin C3 | 0.0145 | 0.0222 | 0.0001 |
| Floatation fat | Spreading/Compost | 0.0000 | 0.0000 | 0.0000 |
| Forehead | C1-C2 for disposal | 0.0000 | 0.0000 | 0.0000 |
| Forelock | PAP C3 | 0.0033 | 0.0014 | 0.0001 |
| Gallbladder | Pet food | 0.0004 | 0.0006 | 0.0000 |
| Head trimmings | Pet food | 0.0031 | 0.0047 | 0.0004 |
| Heart | Human food | 0.0036 | 0.0056 | 0.0012 |
| Heart trimmings | Pet food | 0.0004 | 0.0006 | 0.0000 |
| Hide | Skin tannery C3 | 0.0806 | 0.0871 | 0.1524 |
| Hooves | PAP C3 | 0.0091 | 0.0038 | 0.0003 |
| Horns | PAP C3 | 0.0021 | 0.0009 | 0.0001 |
| Kidney | Human food | 0.0023 | 0.0034 | 0.0015 |
| Large intestine | C1-C2 for disposal | 0.0000 | 0.0000 | 0.0000 |
| Liver | Human food | 0.0610 | 0.0224 | 0.0114 |
| Liver trimmings | Pet food | 0.0069 | 0.0024 | 0.0002 |
| Lower jaw | PAP C3 | 0.0038 | 0.0063 | 0.0006 |
| Lungs | Pet food | 0.0096 | 0.0126 | 0.0010 |
| Mask | Skin tannery C3 | 0.0054 | 0.0059 | 0.0103 |
| Mesenteric fat | C1-C2 for disposal | 0.0000 | 0.0000 | 0.0000 |
| Muscle | Human food | 0.2740 | 0.4102 | 0.7188 |
| Muzzle | Human food | 0.0028 | 0.0031 | 0.0032 |
| Omasum | Human food | 0.0146 | 0.0028 | 0.0022 |
| Omasum fat | Fat and greaves C3 | 0.0039 | 0.0019 | 0.0002 |
| Rumen and forestomach | Human food | 0.0750 | 0.0144 | 0.0113 |
| Rumen fat | Fat and greaves C3 | 0.0058 | 0.0028 | 0.0003 |
| Sanitary seizures | C1-C2 for disposal | 0.0000 | 0.0000 | 0.0000 |
| Screening and sifting wastes | C1-C2 for disposal | 0.0000 | 0.0000 | 0.0000 |
| Small intestine | PAP C3 | 0.0870 | 0.0167 | 0.0011 |
| Spinal cord | C1-C2 for disposal | 0.0000 | 0.0000 | 0.0000 |
| Spinal cord waste | C1-C2 for disposal | 0.0000 | 0.0000 | 0.0000 |
| Spine | C1-C2 for disposal | 0.0000 | 0.0000 | 0.0000 |
| Spleen | Pet food | 0.0023 | 0.0034 | 0.0003 |
| Stillborn | PAP C3 | 0.0457 | 0.0084 | 0.0000 |
| Tallow | Fat and greaves C3 | 0.0834 | 0.0864 | 0.0082 |
| Tongue | Human food | 0.0029 | 0.0050 | 0.0083 |
| Tonsil | C1-C2 for disposal | 0.0000 | 0.0000 | 0.0000 |
| Trachea | Pet food | 0.0020 | 0.0022 | 0.0002 |
| Udder | Pet food | 0.0037 | 0.0264 | 0.0020 |
| Upper throat | Pet food | 0.0013 | 0.0019 | 0.0001 |
| Water in the rumen | Spreading/Compost | 0.0000 | 0.0000 | 0.0000 |

Table 32: Total weighting by coproducts for Primholstein Beef reared in Pasture

| COPRODUCT | Destination | Primholstein/beef/pasture | | |
| --- | --- | --- | --- | --- |
|  |  | **Biophysical Cumulative share** | **Mass Cumulative share** | **Economic Cumulative share** |
| Abomasum | Human food | 0.0155 | 0.0029 | 0.0022 |
| Abomasum fat | Fat and greaves C3 | 0.0013 | 0.0006 | 0.0001 |
| Aponeurosis | Human food | 0.0238 | 0.0321 | 0.0331 |
| Bile | PAP C3 | 0.0000 | 0.0001 | 0.0000 |
| Blood | PAP C3 | 0.0196 | 0.0344 | 0.0079 |
| Blood | Pet food | 0.0028 | 0.0049 | 0.0004 |
| Bones | Gelatin C3 | 0.0451 | 0.0721 | 0.0002 |
| Bones of head, brain, eyes and teeth | C1-C2 for disposal | 0.0000 | 0.0000 | 0.0000 |
| Cheek | Human food | 0.0012 | 0.0018 | 0.0040 |
| Cheek | Human food | 0.0021 | 0.0031 | 0.0071 |
| Cheek trimmings | Pet food | 0.0006 | 0.0009 | 0.0001 |
| Chops | Pet food | 0.0021 | 0.0023 | 0.0002 |
| Contents of intestines | Spreading/Compost | 0.0000 | 0.0000 | 0.0000 |
| Contents of the rumen | Spreading/Compost | 0.0000 | 0.0000 | 0.0000 |
| Ears | PAP C3 | 0.0010 | 0.0011 | 0.0001 |
| Esophagus | Pet food | 0.0011 | 0.0016 | 0.0001 |
| Fat | Fat and greaves C3 | 0.0763 | 0.0771 | 0.0072 |
| Fat around heart | Fat and greaves C3 | 0.0031 | 0.0031 | 0.0003 |
| Fat in the kidney | Fat and greaves C3 | 0.0004 | 0.0004 | 0.0000 |
| Feet (without hooves) | Gelatin C3 | 0.0137 | 0.0207 | 0.0001 |
| Floatation fat | Spreading/Compost | 0.0000 | 0.0000 | 0.0000 |
| Forehead | C1-C2 for disposal | 0.0000 | 0.0000 | 0.0000 |
| Forelock | PAP C3 | 0.0030 | 0.0013 | 0.0001 |
| Gallbladder | Pet food | 0.0004 | 0.0006 | 0.0000 |
| Head trimmings | Pet food | 0.0029 | 0.0043 | 0.0003 |
| Heart | Human food | 0.0033 | 0.0052 | 0.0011 |
| Heart trimmings | Pet food | 0.0004 | 0.0006 | 0.0000 |
| Hide | Skin tannery C3 | 0.0759 | 0.0810 | 0.1388 |
| Hooves | PAP C3 | 0.0088 | 0.0037 | 0.0003 |
| Horns | PAP C3 | 0.0021 | 0.0009 | 0.0001 |
| Kidney | Human food | 0.0021 | 0.0031 | 0.0013 |
| Large intestine | C1-C2 for disposal | 0.0000 | 0.0000 | 0.0000 |
| Liver | Human food | 0.0584 | 0.0209 | 0.0104 |
| Liver trimmings | Pet food | 0.0066 | 0.0023 | 0.0002 |
| Lower jaw | PAP C3 | 0.0036 | 0.0058 | 0.0005 |
| Lungs | Pet food | 0.0091 | 0.0117 | 0.0009 |
| Mask | Skin tannery C3 | 0.0052 | 0.0055 | 0.0095 |
| Mesenteric fat | C1-C2 for disposal | 0.0000 | 0.0000 | 0.0000 |
| Muscle | Human food | 0.2916 | 0.4305 | 0.7390 |
| Muzzle | Human food | 0.0027 | 0.0029 | 0.0030 |
| Omasum | Human food | 0.0142 | 0.0026 | 0.0020 |
| Omasum fat | Fat and greaves C3 | 0.0037 | 0.0018 | 0.0002 |
| Rumen and forestomach | Human food | 0.0722 | 0.0135 | 0.0104 |
| Rumen fat | Fat and greaves C3 | 0.0056 | 0.0026 | 0.0002 |
| Sanitary seizures | C1-C2 for disposal | 0.0000 | 0.0000 | 0.0000 |
| Screening and sifting wastes | C1-C2 for disposal | 0.0000 | 0.0000 | 0.0000 |
| Small intestine | PAP C3 | 0.0837 | 0.0156 | 0.0010 |
| Spinal cord | C1-C2 for disposal | 0.0000 | 0.0000 | 0.0000 |
| Spinal cord waste | C1-C2 for disposal | 0.0000 | 0.0000 | 0.0000 |
| Spine | C1-C2 for disposal | 0.0000 | 0.0000 | 0.0000 |
| Spleen | Pet food | 0.0022 | 0.0031 | 0.0002 |
| Stillborn | PAP C3 | 0.0437 | 0.0078 | 0.0000 |
| Tallow | Fat and greaves C3 | 0.0794 | 0.0804 | 0.0075 |
| Tongue | Human food | 0.0028 | 0.0047 | 0.0076 |
| Tonsil | C1-C2 for disposal | 0.0000 | 0.0000 | 0.0000 |
| Trachea | Pet food | 0.0019 | 0.0020 | 0.0002 |
| Udder | Pet food | 0.0035 | 0.0246 | 0.0019 |
| Upper throat | Pet food | 0.0012 | 0.0018 | 0.0001 |
| Water in the rumen | Spreading/Compost | 0.0000 | 0.0000 | 0.0000 |

Table 33: Total weighting by coproducts for Primholstein Young Bulls reared in Stall

| COPRODUCT | Destination | Primholstein/young bull/stall | | |
| --- | --- | --- | --- | --- |
|  |  | **Biophysical Cumulative share** | **Mass Cumulative share** | **Economic Cumulative share** |
| Abomasum | Human food | 0.0142 | 0.0028 | 0.0021 |
| Abomasum fat | Fat and greaves C3 | 0.0011 | 0.0005 | 0.0000 |
| Aponeurosis | Human food | 0.0241 | 0.0326 | 0.0334 |
| Bile | PAP C3 | 0.0000 | 0.0001 | 0.0000 |
| Blood | PAP C3 | 0.0191 | 0.0335 | 0.0076 |
| Blood | Pet food | 0.0027 | 0.0048 | 0.0004 |
| Bones | Gelatin C3 | 0.0470 | 0.0732 | 0.0002 |
| Bones of head, brain, eyes and teeth | C1-C2 for disposal | 0.0000 | 0.0000 | 0.0000 |
| Cheek | Human food | 0.0011 | 0.0016 | 0.0037 |
| Cheek | Human food | 0.0021 | 0.0031 | 0.0070 |
| Cheek trimmings | Pet food | 0.0006 | 0.0009 | 0.0001 |
| Chops | Pet food | 0.0021 | 0.0023 | 0.0002 |
| Contents of intestines | Spreading/Compost | 0.0000 | 0.0000 | 0.0000 |
| Contents of the rumen | Spreading/Compost | 0.0000 | 0.0000 | 0.0000 |
| Ears | PAP C3 | 0.0010 | 0.0011 | 0.0001 |
| Esophagus | Pet food | 0.0010 | 0.0015 | 0.0001 |
| Fat | Fat and greaves C3 | 0.0858 | 0.0783 | 0.0073 |
| Fat around heart | Fat and greaves C3 | 0.0034 | 0.0031 | 0.0003 |
| Fat in the kidney | Fat and greaves C3 | 0.0004 | 0.0004 | 0.0000 |
| Feet (without hooves) | Gelatin C3 | 0.0135 | 0.0202 | 0.0001 |
| Floatation fat | Spreading/Compost | 0.0000 | 0.0000 | 0.0000 |
| Forehead | C1-C2 for disposal | 0.0000 | 0.0000 | 0.0000 |
| Forelock | PAP C3 | 0.0030 | 0.0013 | 0.0001 |
| Gallbladder | Pet food | 0.0003 | 0.0005 | 0.0000 |
| Head trimmings | Pet food | 0.0029 | 0.0043 | 0.0003 |
| Heart | Human food | 0.0032 | 0.0050 | 0.0011 |
| Heart trimmings | Pet food | 0.0003 | 0.0005 | 0.0000 |
| Hide | Skin tannery C3 | 0.0739 | 0.0791 | 0.1346 |
| Hooves | PAP C3 | 0.0084 | 0.0035 | 0.0003 |
| Horns | PAP C3 | 0.0021 | 0.0009 | 0.0001 |
| Kidney | Human food | 0.0021 | 0.0031 | 0.0013 |
| Large intestine | C1-C2 for disposal | 0.0000 | 0.0000 | 0.0000 |
| Liver | Human food | 0.0553 | 0.0205 | 0.0101 |
| Liver trimmings | Pet food | 0.0064 | 0.0023 | 0.0002 |
| Lower jaw | PAP C3 | 0.0036 | 0.0056 | 0.0005 |
| Lungs | Pet food | 0.0088 | 0.0114 | 0.0009 |
| Mask | Skin tannery C3 | 0.0050 | 0.0054 | 0.0092 |
| Mesenteric fat | C1-C2 for disposal | 0.0000 | 0.0000 | 0.0000 |
| Muscle | Human food | 0.2971 | 0.4374 | 0.7457 |
| Muzzle | Human food | 0.0026 | 0.0028 | 0.0028 |
| Omasum | Human food | 0.0129 | 0.0025 | 0.0019 |
| Omasum fat | Fat and greaves C3 | 0.0035 | 0.0016 | 0.0002 |
| Rumen and forestomach | Human food | 0.0673 | 0.0131 | 0.0100 |
| Rumen fat | Fat and greaves C3 | 0.0054 | 0.0025 | 0.0002 |
| Sanitary seizures | C1-C2 for disposal | 0.0000 | 0.0000 | 0.0000 |
| Screening and sifting wastes | C1-C2 for disposal | 0.0000 | 0.0000 | 0.0000 |
| Small intestine | PAP C3 | 0.0783 | 0.0152 | 0.0009 |
| Spinal cord | C1-C2 for disposal | 0.0000 | 0.0000 | 0.0000 |
| Spinal cord waste | C1-C2 for disposal | 0.0000 | 0.0000 | 0.0000 |
| Spine | C1-C2 for disposal | 0.0000 | 0.0000 | 0.0000 |
| Spleen | Pet food | 0.0021 | 0.0031 | 0.0002 |
| Stillborn | PAP C3 | 0.0412 | 0.0077 | 0.0000 |
| Tallow | Fat and greaves C3 | 0.0858 | 0.0785 | 0.0073 |
| Tongue | Human food | 0.0027 | 0.0045 | 0.0073 |
| Tonsil | C1-C2 for disposal | 0.0000 | 0.0000 | 0.0000 |
| Trachea | Pet food | 0.0019 | 0.0020 | 0.0002 |
| Udder | Pet food | 0.0036 | 0.0240 | 0.0018 |
| Upper throat | Pet food | 0.0011 | 0.0016 | 0.0001 |
| Water in the rumen | Spreading/Compost | 0.0000 | 0.0000 | 0.0000 |

Table 34: Total weighting by coproducts for Primholstein Heifers reared in Stall

| COPRODUCT | Destination | Primholstein/heifer/stall | | |
| --- | --- | --- | --- | --- |
|  |  | **Biophysical Cumulative share** | **Mass Cumulative share** | **Economic Cumulative share** |
| Abomasum | Human food | 0.0148 | 0.0030 | 0.0024 |
| Abomasum fat | Fat and greaves C3 | 0.0013 | 0.0006 | 0.0001 |
| Aponeurosis | Human food | 0.0230 | 0.0311 | 0.0325 |
| Bile | PAP C3 | 0.0000 | 0.0001 | 0.0000 |
| Blood | PAP C3 | 0.0206 | 0.0361 | 0.0084 |
| Blood | Pet food | 0.0030 | 0.0052 | 0.0004 |
| Bones | Gelatin C3 | 0.0445 | 0.0697 | 0.0002 |
| Bones of head, brain, eyes and teeth | C1-C2 for disposal | 0.0000 | 0.0000 | 0.0000 |
| Cheek | Human food | 0.0012 | 0.0018 | 0.0041 |
| Cheek | Human food | 0.0022 | 0.0033 | 0.0076 |
| Cheek trimmings | Pet food | 0.0006 | 0.0009 | 0.0001 |
| Chops | Pet food | 0.0023 | 0.0024 | 0.0002 |
| Contents of intestines | Spreading/Compost | 0.0000 | 0.0000 | 0.0000 |
| Contents of the rumen | Spreading/Compost | 0.0000 | 0.0000 | 0.0000 |
| Ears | PAP C3 | 0.0011 | 0.0013 | 0.0001 |
| Esophagus | Pet food | 0.0011 | 0.0017 | 0.0001 |
| Fat | Fat and greaves C3 | 0.0800 | 0.0747 | 0.0071 |
| Fat around heart | Fat and greaves C3 | 0.0035 | 0.0033 | 0.0003 |
| Fat in the kidney | Fat and greaves C3 | 0.0005 | 0.0005 | 0.0000 |
| Feet (without hooves) | Gelatin C3 | 0.0145 | 0.0217 | 0.0001 |
| Floatation fat | Spreading/Compost | 0.0000 | 0.0000 | 0.0000 |
| Forehead | C1-C2 for disposal | 0.0000 | 0.0000 | 0.0000 |
| Forelock | PAP C3 | 0.0034 | 0.0014 | 0.0001 |
| Gallbladder | Pet food | 0.0004 | 0.0006 | 0.0000 |
| Head trimmings | Pet food | 0.0031 | 0.0046 | 0.0003 |
| Heart | Human food | 0.0035 | 0.0055 | 0.0012 |
| Heart trimmings | Pet food | 0.0004 | 0.0006 | 0.0000 |
| Hide | Skin tannery C3 | 0.0797 | 0.0851 | 0.1479 |
| Hooves | PAP C3 | 0.0092 | 0.0038 | 0.0003 |
| Horns | PAP C3 | 0.0021 | 0.0009 | 0.0001 |
| Kidney | Human food | 0.0022 | 0.0033 | 0.0014 |
| Large intestine | C1-C2 for disposal | 0.0000 | 0.0000 | 0.0000 |
| Liver | Human food | 0.0565 | 0.0220 | 0.0111 |
| Liver trimmings | Pet food | 0.0065 | 0.0024 | 0.0002 |
| Lower jaw | PAP C3 | 0.0038 | 0.0061 | 0.0005 |
| Lungs | Pet food | 0.0095 | 0.0123 | 0.0009 |
| Mask | Skin tannery C3 | 0.0054 | 0.0057 | 0.0099 |
| Mesenteric fat | C1-C2 for disposal | 0.0000 | 0.0000 | 0.0000 |
| Muscle | Human food | 0.2831 | 0.4171 | 0.7259 |
| Muzzle | Human food | 0.0029 | 0.0030 | 0.0032 |
| Omasum | Human food | 0.0130 | 0.0027 | 0.0021 |
| Omasum fat | Fat and greaves C3 | 0.0037 | 0.0018 | 0.0002 |
| Rumen and forestomach | Human food | 0.0686 | 0.0141 | 0.0110 |
| Rumen fat | Fat and greaves C3 | 0.0055 | 0.0027 | 0.0003 |
| Sanitary seizures | C1-C2 for disposal | 0.0000 | 0.0000 | 0.0000 |
| Screening and sifting wastes | C1-C2 for disposal | 0.0000 | 0.0000 | 0.0000 |
| Small intestine | PAP C3 | 0.0797 | 0.0164 | 0.0010 |
| Spinal cord | C1-C2 for disposal | 0.0000 | 0.0000 | 0.0000 |
| Spinal cord waste | C1-C2 for disposal | 0.0000 | 0.0000 | 0.0000 |
| Spine | C1-C2 for disposal | 0.0000 | 0.0000 | 0.0000 |
| Spleen | Pet food | 0.0023 | 0.0033 | 0.0003 |
| Stillborn | PAP C3 | 0.0412 | 0.0081 | 0.0000 |
| Tallow | Fat and greaves C3 | 0.0902 | 0.0844 | 0.0080 |
| Tongue | Human food | 0.0029 | 0.0048 | 0.0080 |
| Tonsil | C1-C2 for disposal | 0.0000 | 0.0000 | 0.0000 |
| Trachea | Pet food | 0.0020 | 0.0022 | 0.0002 |
| Udder | Pet food | 0.0038 | 0.0258 | 0.0020 |
| Upper throat | Pet food | 0.0012 | 0.0018 | 0.0001 |
| Water in the rumen | Spreading/Compost | 0.0000 | 0.0000 | 0.0000 |

Table 35: Total weighting by coproducts for Primholstein Cull Cows reared in Stall

| COPRODUCT | Destination | Primholstein/Cull cow/stall | | |
| --- | --- | --- | --- | --- |
|  |  | **Biophysical Cumulative share** | **Mass Cumulative share** | **Economic Cumulative share** |
| Abomasum | Human food | 0.0151 | 0.0031 | 0.0024 |
| Abomasum fat | Fat and greaves C3 | 0.0013 | 0.0006 | 0.0001 |
| Aponeurosis | Human food | 0.0222 | 0.0306 | 0.0322 |
| Bile | PAP C3 | 0.0000 | 0.0001 | 0.0000 |
| Blood | PAP C3 | 0.0207 | 0.0370 | 0.0087 |
| Blood | Pet food | 0.0029 | 0.0052 | 0.0004 |
| Bones | Gelatin C3 | 0.0431 | 0.0686 | 0.0002 |
| Bones of head, brain, eyes and teeth | C1-C2 for disposal | 0.0000 | 0.0000 | 0.0000 |
| Cheek | Human food | 0.0013 | 0.0019 | 0.0044 |
| Cheek | Human food | 0.0023 | 0.0034 | 0.0079 |
| Cheek trimmings | Pet food | 0.0006 | 0.0009 | 0.0001 |
| Chops | Pet food | 0.0022 | 0.0024 | 0.0002 |
| Contents of intestines | Spreading/Compost | 0.0000 | 0.0000 | 0.0000 |
| Contents of the rumen | Spreading/Compost | 0.0000 | 0.0000 | 0.0000 |
| Ears | PAP C3 | 0.0011 | 0.0013 | 0.0001 |
| Esophagus | Pet food | 0.0011 | 0.0017 | 0.0001 |
| Fat | Fat and greaves C3 | 0.0779 | 0.0735 | 0.0070 |
| Fat around heart | Fat and greaves C3 | 0.0036 | 0.0034 | 0.0003 |
| Fat in the kidney | Fat and greaves C3 | 0.0005 | 0.0005 | 0.0000 |
| Feet (without hooves) | Gelatin C3 | 0.0145 | 0.0222 | 0.0001 |
| Floatation fat | Spreading/Compost | 0.0000 | 0.0000 | 0.0000 |
| Forehead | C1-C2 for disposal | 0.0000 | 0.0000 | 0.0000 |
| Forelock | PAP C3 | 0.0033 | 0.0014 | 0.0001 |
| Gallbladder | Pet food | 0.0004 | 0.0006 | 0.0000 |
| Head trimmings | Pet food | 0.0031 | 0.0047 | 0.0004 |
| Heart | Human food | 0.0035 | 0.0056 | 0.0012 |
| Heart trimmings | Pet food | 0.0004 | 0.0006 | 0.0000 |
| Hide | Skin tannery C3 | 0.0801 | 0.0871 | 0.1524 |
| Hooves | PAP C3 | 0.0090 | 0.0038 | 0.0003 |
| Horns | PAP C3 | 0.0021 | 0.0009 | 0.0001 |
| Kidney | Human food | 0.0022 | 0.0034 | 0.0015 |
| Large intestine | C1-C2 for disposal | 0.0000 | 0.0000 | 0.0000 |
| Liver | Human food | 0.0584 | 0.0224 | 0.0114 |
| Liver trimmings | Pet food | 0.0066 | 0.0024 | 0.0002 |
| Lower jaw | PAP C3 | 0.0039 | 0.0063 | 0.0006 |
| Lungs | Pet food | 0.0096 | 0.0126 | 0.0010 |
| Mask | Skin tannery C3 | 0.0054 | 0.0059 | 0.0103 |
| Mesenteric fat | C1-C2 for disposal | 0.0000 | 0.0000 | 0.0000 |
| Muscle | Human food | 0.2737 | 0.4102 | 0.7188 |
| Muzzle | Human food | 0.0028 | 0.0031 | 0.0032 |
| Omasum | Human food | 0.0139 | 0.0028 | 0.0022 |
| Omasum fat | Fat and greaves C3 | 0.0040 | 0.0019 | 0.0002 |
| Rumen and forestomach | Human food | 0.0713 | 0.0144 | 0.0113 |
| Rumen fat | Fat and greaves C3 | 0.0058 | 0.0028 | 0.0003 |
| Sanitary seizures | C1-C2 for disposal | 0.0000 | 0.0000 | 0.0000 |
| Screening and sifting wastes | C1-C2 for disposal | 0.0000 | 0.0000 | 0.0000 |
| Small intestine | PAP C3 | 0.0827 | 0.0167 | 0.0011 |
| Spinal cord | C1-C2 for disposal | 0.0000 | 0.0000 | 0.0000 |
| Spinal cord waste | C1-C2 for disposal | 0.0000 | 0.0000 | 0.0000 |
| Spine | C1-C2 for disposal | 0.0000 | 0.0000 | 0.0000 |
| Spleen | Pet food | 0.0023 | 0.0034 | 0.0003 |
| Stillborn | PAP C3 | 0.0434 | 0.0084 | 0.0000 |
| Tallow | Fat and greaves C3 | 0.0915 | 0.0864 | 0.0082 |
| Tongue | Human food | 0.0029 | 0.0050 | 0.0083 |
| Tonsil | C1-C2 for disposal | 0.0000 | 0.0000 | 0.0000 |
| Trachea | Pet food | 0.0020 | 0.0022 | 0.0002 |
| Udder | Pet food | 0.0039 | 0.0264 | 0.0020 |
| Upper throat | Pet food | 0.0013 | 0.0019 | 0.0001 |
| Water in the rumen | Spreading/Compost | 0.0000 | 0.0000 | 0.0000 |

Table 36: Total weighting by coproducts for Primholstein Beef reared in Stall

| COPRODUCT | Destination | Primholstein/beef/stall | | |
| --- | --- | --- | --- | --- |
|  |  | **Biophysical Cumulative share** | **Mass Cumulative share** | **Economic Cumulative share** |
| Abomasum | Human food | 0.0148 | 0.0029 | 0.0022 |
| Abomasum fat | Fat and greaves C3 | 0.0013 | 0.0006 | 0.0001 |
| Aponeurosis | Human food | 0.0235 | 0.0321 | 0.0331 |
| Bile | PAP C3 | 0.0000 | 0.0001 | 0.0000 |
| Blood | PAP C3 | 0.0195 | 0.0344 | 0.0079 |
| Blood | Pet food | 0.0028 | 0.0049 | 0.0004 |
| Bones | Gelatin C3 | 0.0459 | 0.0721 | 0.0002 |
| Bones of head, brain, eyes and teeth | C1-C2 for disposal | 0.0000 | 0.0000 | 0.0000 |
| Cheek | Human food | 0.0012 | 0.0018 | 0.0040 |
| Cheek | Human food | 0.0021 | 0.0031 | 0.0071 |
| Cheek trimmings | Pet food | 0.0006 | 0.0009 | 0.0001 |
| Chops | Pet food | 0.0021 | 0.0023 | 0.0002 |
| Contents of intestines | Spreading/Compost | 0.0000 | 0.0000 | 0.0000 |
| Contents of the rumen | Spreading/Compost | 0.0000 | 0.0000 | 0.0000 |
| Ears | PAP C3 | 0.0010 | 0.0011 | 0.0001 |
| Esophagus | Pet food | 0.0011 | 0.0016 | 0.0001 |
| Fat | Fat and greaves C3 | 0.0837 | 0.0771 | 0.0072 |
| Fat around heart | Fat and greaves C3 | 0.0034 | 0.0031 | 0.0003 |
| Fat in the kidney | Fat and greaves C3 | 0.0004 | 0.0004 | 0.0000 |
| Feet (without hooves) | Gelatin C3 | 0.0137 | 0.0207 | 0.0001 |
| Floatation fat | Spreading/Compost | 0.0000 | 0.0000 | 0.0000 |
| Forehead | C1-C2 for disposal | 0.0000 | 0.0000 | 0.0000 |
| Forelock | PAP C3 | 0.0030 | 0.0013 | 0.0001 |
| Gallbladder | Pet food | 0.0004 | 0.0006 | 0.0000 |
| Head trimmings | Pet food | 0.0029 | 0.0043 | 0.0003 |
| Heart | Human food | 0.0033 | 0.0052 | 0.0011 |
| Heart trimmings | Pet food | 0.0004 | 0.0006 | 0.0000 |
| Hide | Skin tannery C3 | 0.0753 | 0.0810 | 0.1388 |
| Hooves | PAP C3 | 0.0087 | 0.0037 | 0.0003 |
| Horns | PAP C3 | 0.0021 | 0.0009 | 0.0001 |
| Kidney | Human food | 0.0021 | 0.0031 | 0.0013 |
| Large intestine | C1-C2 for disposal | 0.0000 | 0.0000 | 0.0000 |
| Liver | Human food | 0.0559 | 0.0209 | 0.0104 |
| Liver trimmings | Pet food | 0.0063 | 0.0023 | 0.0002 |
| Lower jaw | PAP C3 | 0.0036 | 0.0058 | 0.0005 |
| Lungs | Pet food | 0.0090 | 0.0117 | 0.0009 |
| Mask | Skin tannery C3 | 0.0052 | 0.0055 | 0.0095 |
| Mesenteric fat | C1-C2 for disposal | 0.0000 | 0.0000 | 0.0000 |
| Muscle | Human food | 0.2907 | 0.4305 | 0.7390 |
| Muzzle | Human food | 0.0027 | 0.0029 | 0.0030 |
| Omasum | Human food | 0.0135 | 0.0026 | 0.0020 |
| Omasum fat | Fat and greaves C3 | 0.0038 | 0.0018 | 0.0002 |
| Rumen and forestomach | Human food | 0.0687 | 0.0135 | 0.0104 |
| Rumen fat | Fat and greaves C3 | 0.0056 | 0.0026 | 0.0002 |
| Sanitary seizures | C1-C2 for disposal | 0.0000 | 0.0000 | 0.0000 |
| Screening and sifting wastes | C1-C2 for disposal | 0.0000 | 0.0000 | 0.0000 |
| Small intestine | PAP C3 | 0.0796 | 0.0156 | 0.0010 |
| Spinal cord | C1-C2 for disposal | 0.0000 | 0.0000 | 0.0000 |
| Spinal cord waste | C1-C2 for disposal | 0.0000 | 0.0000 | 0.0000 |
| Spine | C1-C2 for disposal | 0.0000 | 0.0000 | 0.0000 |
| Spleen | Pet food | 0.0021 | 0.0031 | 0.0002 |
| Stillborn | PAP C3 | 0.0415 | 0.0078 | 0.0000 |
| Tallow | Fat and greaves C3 | 0.0871 | 0.0804 | 0.0075 |
| Tongue | Human food | 0.0028 | 0.0047 | 0.0076 |
| Tonsil | C1-C2 for disposal | 0.0000 | 0.0000 | 0.0000 |
| Trachea | Pet food | 0.0019 | 0.0020 | 0.0002 |
| Udder | Pet food | 0.0037 | 0.0246 | 0.0019 |
| Upper throat | Pet food | 0.0012 | 0.0018 | 0.0001 |
| Water in the rumen | Spreading/Compost | 0.0000 | 0.0000 | 0.0000 |

Table 37: Total weighting by coproducts for Limousine Young Bulls reared in Grazing Large Area

| COPRODUCT | Destination | Limousine/young bull/grazing large area | | |
| --- | --- | --- | --- | --- |
|  |  | **Biophysical Cumulative share** | **Mass Cumulative share** | **Economic Cumulative share** |
| Abomasum | Human food | 0.0131 | 0.0022 | 0.0016 |
| Abomasum fat | Fat and greaves C3 | 0.0011 | 0.0005 | 0.0000 |
| Aponeurosis | Human food | 0.0294 | 0.0369 | 0.0358 |
| Bile | PAP C3 | 0.0000 | 0.0001 | 0.0000 |
| Blood | PAP C3 | 0.0161 | 0.0263 | 0.0057 |
| Blood | Pet food | 0.0023 | 0.0038 | 0.0003 |
| Bones | Gelatin C3 | 0.0549 | 0.0828 | 0.0002 |
| Bones of head, brain, eyes and teeth | C1-C2 for disposal | 0.0000 | 0.0000 | 0.0000 |
| Cheek | Human food | 0.0010 | 0.0013 | 0.0028 |
| Cheek | Human food | 0.0018 | 0.0024 | 0.0051 |
| Cheek trimmings | Pet food | 0.0004 | 0.0006 | 0.0000 |
| Chops | Pet food | 0.0018 | 0.0018 | 0.0001 |
| Contents of intestines | Spreading/Compost | 0.0000 | 0.0000 | 0.0000 |
| Contents of the rumen | Spreading/Compost | 0.0000 | 0.0000 | 0.0000 |
| Ears | PAP C3 | 0.0008 | 0.0008 | 0.0001 |
| Esophagus | Pet food | 0.0008 | 0.0012 | 0.0001 |
| Fat | Fat and greaves C3 | 0.0879 | 0.0886 | 0.0078 |
| Fat around heart | Fat and greaves C3 | 0.0024 | 0.0024 | 0.0002 |
| Fat in the kidney | Fat and greaves C3 | 0.0004 | 0.0004 | 0.0000 |
| Feet (without hooves) | Gelatin C3 | 0.0113 | 0.0159 | 0.0000 |
| Floatation fat | Spreading/Compost | 0.0000 | 0.0000 | 0.0000 |
| Forehead | C1-C2 for disposal | 0.0000 | 0.0000 | 0.0000 |
| Forelock | PAP C3 | 0.0025 | 0.0010 | 0.0001 |
| Gallbladder | Pet food | 0.0003 | 0.0005 | 0.0000 |
| Head trimmings | Pet food | 0.0024 | 0.0033 | 0.0002 |
| Heart | Human food | 0.0028 | 0.0040 | 0.0008 |
| Heart trimmings | Pet food | 0.0004 | 0.0005 | 0.0000 |
| Hide | Skin tannery C3 | 0.0625 | 0.0620 | 0.0998 |
| Hooves | PAP C3 | 0.0072 | 0.0028 | 0.0002 |
| Horns | PAP C3 | 0.0016 | 0.0006 | 0.0001 |
| Kidney | Human food | 0.0017 | 0.0024 | 0.0009 |
| Large intestine | C1-C2 for disposal | 0.0000 | 0.0000 | 0.0000 |
| Liver | Human food | 0.0499 | 0.0160 | 0.0075 |
| Liver trimmings | Pet food | 0.0059 | 0.0018 | 0.0001 |
| Lower jaw | PAP C3 | 0.0029 | 0.0045 | 0.0004 |
| Lungs | Pet food | 0.0075 | 0.0090 | 0.0006 |
| Mask | Skin tannery C3 | 0.0043 | 0.0042 | 0.0068 |
| Mesenteric fat | C1-C2 for disposal | 0.0000 | 0.0000 | 0.0000 |
| Muscle | Human food | 0.3597 | 0.4949 | 0.7980 |
| Muzzle | Human food | 0.0022 | 0.0022 | 0.0021 |
| Omasum | Human food | 0.0117 | 0.0019 | 0.0014 |
| Omasum fat | Fat and greaves C3 | 0.0030 | 0.0013 | 0.0001 |
| Rumen and forestomach | Human food | 0.0620 | 0.0103 | 0.0074 |
| Rumen fat | Fat and greaves C3 | 0.0044 | 0.0019 | 0.0002 |
| Sanitary seizures | C1-C2 for disposal | 0.0000 | 0.0000 | 0.0000 |
| Screening and sifting wastes | C1-C2 for disposal | 0.0000 | 0.0000 | 0.0000 |
| Small intestine | PAP C3 | 0.0722 | 0.0120 | 0.0007 |
| Spinal cord | C1-C2 for disposal | 0.0000 | 0.0000 | 0.0000 |
| Spinal cord waste | C1-C2 for disposal | 0.0000 | 0.0000 | 0.0000 |
| Spine | C1-C2 for disposal | 0.0000 | 0.0000 | 0.0000 |
| Spleen | Pet food | 0.0018 | 0.0024 | 0.0002 |
| Stillborn | PAP C3 | 0.0373 | 0.0059 | 0.0000 |
| Tallow | Fat and greaves C3 | 0.0608 | 0.0614 | 0.0054 |
| Tongue | Human food | 0.0022 | 0.0035 | 0.0054 |
| Tonsil | C1-C2 for disposal | 0.0000 | 0.0000 | 0.0000 |
| Trachea | Pet food | 0.0016 | 0.0016 | 0.0001 |
| Udder | Pet food | 0.0028 | 0.0188 | 0.0013 |
| Upper throat | Pet food | 0.0009 | 0.0013 | 0.0001 |
| Water in the rumen | Spreading/Compost | 0.0000 | 0.0000 | 0.0000 |

Table 38: Total weighting by coproducts for Limousine Heifers reared in Grazing Large Area

| COPRODUCT | Destination | Limousine/heifer/grazing large area | | |
| --- | --- | --- | --- | --- |
|  |  | **Biophysical Cumulative share** | **Mass Cumulative share** | **Economic Cumulative share** |
| Abomasum | Human food | 0.0141 | 0.0025 | 0.0018 |
| Abomasum fat | Fat and greaves C3 | 0.0011 | 0.0005 | 0.0000 |
| Aponeurosis | Human food | 0.0274 | 0.0351 | 0.0348 |
| Bile | PAP C3 | 0.0000 | 0.0001 | 0.0000 |
| Blood | PAP C3 | 0.0178 | 0.0295 | 0.0065 |
| Blood | Pet food | 0.0025 | 0.0042 | 0.0003 |
| Bones | Gelatin C3 | 0.0510 | 0.0786 | 0.0002 |
| Bones of head, brain, eyes and teeth | C1-C2 for disposal | 0.0000 | 0.0000 | 0.0000 |
| Cheek | Human food | 0.0010 | 0.0015 | 0.0032 |
| Cheek | Human food | 0.0019 | 0.0027 | 0.0059 |
| Cheek trimmings | Pet food | 0.0005 | 0.0007 | 0.0001 |
| Chops | Pet food | 0.0019 | 0.0020 | 0.0001 |
| Contents of intestines | Spreading/Compost | 0.0000 | 0.0000 | 0.0000 |
| Contents of the rumen | Spreading/Compost | 0.0000 | 0.0000 | 0.0000 |
| Ears | PAP C3 | 0.0009 | 0.0010 | 0.0001 |
| Esophagus | Pet food | 0.0009 | 0.0014 | 0.0001 |
| Fat | Fat and greaves C3 | 0.0811 | 0.0841 | 0.0076 |
| Fat around heart | Fat and greaves C3 | 0.0026 | 0.0027 | 0.0002 |
| Fat in the kidney | Fat and greaves C3 | 0.0004 | 0.0004 | 0.0000 |
| Feet (without hooves) | Gelatin C3 | 0.0123 | 0.0177 | 0.0001 |
| Floatation fat | Spreading/Compost | 0.0000 | 0.0000 | 0.0000 |
| Forehead | C1-C2 for disposal | 0.0000 | 0.0000 | 0.0000 |
| Forelock | PAP C3 | 0.0028 | 0.0011 | 0.0001 |
| Gallbladder | Pet food | 0.0003 | 0.0005 | 0.0000 |
| Head trimmings | Pet food | 0.0026 | 0.0037 | 0.0003 |
| Heart | Human food | 0.0030 | 0.0044 | 0.0009 |
| Heart trimmings | Pet food | 0.0003 | 0.0005 | 0.0000 |
| Hide | Skin tannery C3 | 0.0687 | 0.0695 | 0.1146 |
| Hooves | PAP C3 | 0.0078 | 0.0031 | 0.0003 |
| Horns | PAP C3 | 0.0019 | 0.0007 | 0.0001 |
| Kidney | Human food | 0.0019 | 0.0027 | 0.0011 |
| Large intestine | C1-C2 for disposal | 0.0000 | 0.0000 | 0.0000 |
| Liver | Human food | 0.0536 | 0.0180 | 0.0086 |
| Liver trimmings | Pet food | 0.0061 | 0.0020 | 0.0001 |
| Lower jaw | PAP C3 | 0.0031 | 0.0049 | 0.0004 |
| Lungs | Pet food | 0.0082 | 0.0101 | 0.0007 |
| Mask | Skin tannery C3 | 0.0046 | 0.0047 | 0.0077 |
| Mesenteric fat | C1-C2 for disposal | 0.0000 | 0.0000 | 0.0000 |
| Muscle | Human food | 0.3344 | 0.4698 | 0.7759 |
| Muzzle | Human food | 0.0024 | 0.0025 | 0.0024 |
| Omasum | Human food | 0.0127 | 0.0022 | 0.0016 |
| Omasum fat | Fat and greaves C3 | 0.0032 | 0.0015 | 0.0001 |
| Rumen and forestomach | Human food | 0.0664 | 0.0116 | 0.0086 |
| Rumen fat | Fat and greaves C3 | 0.0048 | 0.0022 | 0.0002 |
| Sanitary seizures | C1-C2 for disposal | 0.0000 | 0.0000 | 0.0000 |
| Screening and sifting wastes | C1-C2 for disposal | 0.0000 | 0.0000 | 0.0000 |
| Small intestine | PAP C3 | 0.0770 | 0.0134 | 0.0008 |
| Spinal cord | C1-C2 for disposal | 0.0000 | 0.0000 | 0.0000 |
| Spinal cord waste | C1-C2 for disposal | 0.0000 | 0.0000 | 0.0000 |
| Spine | C1-C2 for disposal | 0.0000 | 0.0000 | 0.0000 |
| Spleen | Pet food | 0.0019 | 0.0027 | 0.0002 |
| Stillborn | PAP C3 | 0.0398 | 0.0066 | 0.0000 |
| Tallow | Fat and greaves C3 | 0.0663 | 0.0689 | 0.0062 |
| Tongue | Human food | 0.0024 | 0.0039 | 0.0062 |
| Tonsil | C1-C2 for disposal | 0.0000 | 0.0000 | 0.0000 |
| Trachea | Pet food | 0.0017 | 0.0017 | 0.0001 |
| Udder | Pet food | 0.0031 | 0.0210 | 0.0015 |
| Upper throat | Pet food | 0.0010 | 0.0015 | 0.0001 |
| Water in the rumen | Spreading/Compost | 0.0000 | 0.0000 | 0.0000 |

Table 39: Total weighting by coproducts for Limousine Cull Cows reared in Grazing Large Area

| COPRODUCT | Destination | Limousine/Cull cow/grazing large area | | |
| --- | --- | --- | --- | --- |
|  |  | **Biophysical Cumulative share** | **Mass Cumulative share** | **Economic Cumulative share** |
| Abomasum | Human food | 0.0153 | 0.0026 | 0.0020 |
| Abomasum fat | Fat and greaves C3 | 0.0011 | 0.0005 | 0.0000 |
| Aponeurosis | Human food | 0.0260 | 0.0341 | 0.0342 |
| Bile | PAP C3 | 0.0000 | 0.0001 | 0.0000 |
| Blood | PAP C3 | 0.0183 | 0.0311 | 0.0070 |
| Blood | Pet food | 0.0026 | 0.0045 | 0.0003 |
| Bones | Gelatin C3 | 0.0481 | 0.0764 | 0.0002 |
| Bones of head, brain, eyes and teeth | C1-C2 for disposal | 0.0000 | 0.0000 | 0.0000 |
| Cheek | Human food | 0.0011 | 0.0016 | 0.0035 |
| Cheek | Human food | 0.0020 | 0.0029 | 0.0063 |
| Cheek trimmings | Pet food | 0.0005 | 0.0007 | 0.0001 |
| Chops | Pet food | 0.0020 | 0.0021 | 0.0002 |
| Contents of intestines | Spreading/Compost | 0.0000 | 0.0000 | 0.0000 |
| Contents of the rumen | Spreading/Compost | 0.0000 | 0.0000 | 0.0000 |
| Ears | PAP C3 | 0.0008 | 0.0010 | 0.0001 |
| Esophagus | Pet food | 0.0010 | 0.0015 | 0.0001 |
| Fat | Fat and greaves C3 | 0.0754 | 0.0818 | 0.0074 |
| Fat around heart | Fat and greaves C3 | 0.0026 | 0.0029 | 0.0003 |
| Fat in the kidney | Fat and greaves C3 | 0.0003 | 0.0004 | 0.0000 |
| Feet (without hooves) | Gelatin C3 | 0.0127 | 0.0187 | 0.0001 |
| Floatation fat | Spreading/Compost | 0.0000 | 0.0000 | 0.0000 |
| Forehead | C1-C2 for disposal | 0.0000 | 0.0000 | 0.0000 |
| Forelock | PAP C3 | 0.0028 | 0.0011 | 0.0001 |
| Gallbladder | Pet food | 0.0003 | 0.0005 | 0.0000 |
| Head trimmings | Pet food | 0.0027 | 0.0040 | 0.0003 |
| Heart | Human food | 0.0031 | 0.0047 | 0.0010 |
| Heart trimmings | Pet food | 0.0003 | 0.0005 | 0.0000 |
| Hide | Skin tannery C3 | 0.0707 | 0.0733 | 0.1223 |
| Hooves | PAP C3 | 0.0080 | 0.0032 | 0.0003 |
| Horns | PAP C3 | 0.0019 | 0.0007 | 0.0001 |
| Kidney | Human food | 0.0019 | 0.0029 | 0.0012 |
| Large intestine | C1-C2 for disposal | 0.0000 | 0.0000 | 0.0000 |
| Liver | Human food | 0.0576 | 0.0190 | 0.0092 |
| Liver trimmings | Pet food | 0.0067 | 0.0021 | 0.0002 |
| Lower jaw | PAP C3 | 0.0032 | 0.0052 | 0.0004 |
| Lungs | Pet food | 0.0084 | 0.0105 | 0.0008 |
| Mask | Skin tannery C3 | 0.0048 | 0.0050 | 0.0083 |
| Mesenteric fat | C1-C2 for disposal | 0.0000 | 0.0000 | 0.0000 |
| Muscle | Human food | 0.3173 | 0.4569 | 0.7640 |
| Muzzle | Human food | 0.0025 | 0.0026 | 0.0026 |
| Omasum | Human food | 0.0138 | 0.0024 | 0.0018 |
| Omasum fat | Fat and greaves C3 | 0.0035 | 0.0016 | 0.0001 |
| Rumen and forestomach | Human food | 0.0714 | 0.0121 | 0.0091 |
| Rumen fat | Fat and greaves C3 | 0.0052 | 0.0024 | 0.0002 |
| Sanitary seizures | C1-C2 for disposal | 0.0000 | 0.0000 | 0.0000 |
| Screening and sifting wastes | C1-C2 for disposal | 0.0000 | 0.0000 | 0.0000 |
| Small intestine | PAP C3 | 0.0830 | 0.0141 | 0.0009 |
| Spinal cord | C1-C2 for disposal | 0.0000 | 0.0000 | 0.0000 |
| Spinal cord waste | C1-C2 for disposal | 0.0000 | 0.0000 | 0.0000 |
| Spine | C1-C2 for disposal | 0.0000 | 0.0000 | 0.0000 |
| Spleen | Pet food | 0.0020 | 0.0029 | 0.0002 |
| Stillborn | PAP C3 | 0.0434 | 0.0071 | 0.0000 |
| Tallow | Fat and greaves C3 | 0.0668 | 0.0727 | 0.0066 |
| Tongue | Human food | 0.0025 | 0.0042 | 0.0067 |
| Tonsil | C1-C2 for disposal | 0.0000 | 0.0000 | 0.0000 |
| Trachea | Pet food | 0.0018 | 0.0019 | 0.0001 |
| Udder | Pet food | 0.0032 | 0.0222 | 0.0016 |
| Upper throat | Pet food | 0.0011 | 0.0016 | 0.0001 |
| Water in the rumen | Spreading/Compost | 0.0000 | 0.0000 | 0.0000 |

Table 40: Total weighting by coproducts for Limousine Beef reared in Grazing Large Area

| COPRODUCT | Destination | Limousine/beef/grazing large area | | |
| --- | --- | --- | --- | --- |
|  |  | **Biophysical Cumulative share** | **Mass Cumulative share** | **Economic Cumulative share** |
| Abomasum | Human food | 0.0148 | 0.0024 | 0.0018 |
| Abomasum fat | Fat and greaves C3 | 0.0011 | 0.0005 | 0.0000 |
| Aponeurosis | Human food | 0.0276 | 0.0355 | 0.0350 |
| Bile | PAP C3 | 0.0000 | 0.0001 | 0.0000 |
| Blood | PAP C3 | 0.0171 | 0.0287 | 0.0063 |
| Blood | Pet food | 0.0024 | 0.0040 | 0.0003 |
| Bones | Gelatin C3 | 0.0512 | 0.0796 | 0.0002 |
| Bones of head, brain, eyes and teeth | C1-C2 for disposal | 0.0000 | 0.0000 | 0.0000 |
| Cheek | Human food | 0.0010 | 0.0015 | 0.0032 |
| Cheek | Human food | 0.0019 | 0.0027 | 0.0058 |
| Cheek trimmings | Pet food | 0.0005 | 0.0007 | 0.0001 |
| Chops | Pet food | 0.0019 | 0.0020 | 0.0001 |
| Contents of intestines | Spreading/Compost | 0.0000 | 0.0000 | 0.0000 |
| Contents of the rumen | Spreading/Compost | 0.0000 | 0.0000 | 0.0000 |
| Ears | PAP C3 | 0.0009 | 0.0010 | 0.0001 |
| Esophagus | Pet food | 0.0009 | 0.0013 | 0.0001 |
| Fat | Fat and greaves C3 | 0.0802 | 0.0852 | 0.0076 |
| Fat around heart | Fat and greaves C3 | 0.0025 | 0.0027 | 0.0002 |
| Fat in the kidney | Fat and greaves C3 | 0.0003 | 0.0004 | 0.0000 |
| Feet (without hooves) | Gelatin C3 | 0.0119 | 0.0173 | 0.0001 |
| Floatation fat | Spreading/Compost | 0.0000 | 0.0000 | 0.0000 |
| Forehead | C1-C2 for disposal | 0.0000 | 0.0000 | 0.0000 |
| Forelock | PAP C3 | 0.0028 | 0.0011 | 0.0001 |
| Gallbladder | Pet food | 0.0003 | 0.0005 | 0.0000 |
| Head trimmings | Pet food | 0.0025 | 0.0036 | 0.0003 |
| Heart | Human food | 0.0029 | 0.0043 | 0.0009 |
| Heart trimmings | Pet food | 0.0003 | 0.0005 | 0.0000 |
| Hide | Skin tannery C3 | 0.0663 | 0.0675 | 0.1106 |
| Hooves | PAP C3 | 0.0078 | 0.0031 | 0.0003 |
| Horns | PAP C3 | 0.0019 | 0.0007 | 0.0001 |
| Kidney | Human food | 0.0019 | 0.0027 | 0.0011 |
| Large intestine | C1-C2 for disposal | 0.0000 | 0.0000 | 0.0000 |
| Liver | Human food | 0.0546 | 0.0175 | 0.0084 |
| Liver trimmings | Pet food | 0.0064 | 0.0020 | 0.0001 |
| Lower jaw | PAP C3 | 0.0030 | 0.0048 | 0.0004 |
| Lungs | Pet food | 0.0080 | 0.0098 | 0.0007 |
| Mask | Skin tannery C3 | 0.0045 | 0.0045 | 0.0074 |
| Mesenteric fat | C1-C2 for disposal | 0.0000 | 0.0000 | 0.0000 |
| Muscle | Human food | 0.3369 | 0.4759 | 0.7815 |
| Muzzle | Human food | 0.0024 | 0.0024 | 0.0024 |
| Omasum | Human food | 0.0133 | 0.0022 | 0.0016 |
| Omasum fat | Fat and greaves C3 | 0.0033 | 0.0015 | 0.0001 |
| Rumen and forestomach | Human food | 0.0673 | 0.0111 | 0.0082 |
| Rumen fat | Fat and greaves C3 | 0.0049 | 0.0022 | 0.0002 |
| Sanitary seizures | C1-C2 for disposal | 0.0000 | 0.0000 | 0.0000 |
| Screening and sifting wastes | C1-C2 for disposal | 0.0000 | 0.0000 | 0.0000 |
| Small intestine | PAP C3 | 0.0784 | 0.0130 | 0.0008 |
| Spinal cord | C1-C2 for disposal | 0.0000 | 0.0000 | 0.0000 |
| Spinal cord waste | C1-C2 for disposal | 0.0000 | 0.0000 | 0.0000 |
| Spine | C1-C2 for disposal | 0.0000 | 0.0000 | 0.0000 |
| Spleen | Pet food | 0.0019 | 0.0027 | 0.0002 |
| Stillborn | PAP C3 | 0.0410 | 0.0065 | 0.0000 |
| Tallow | Fat and greaves C3 | 0.0629 | 0.0670 | 0.0060 |
| Tongue | Human food | 0.0023 | 0.0038 | 0.0059 |
| Tonsil | C1-C2 for disposal | 0.0000 | 0.0000 | 0.0000 |
| Trachea | Pet food | 0.0017 | 0.0017 | 0.0001 |
| Udder | Pet food | 0.0030 | 0.0205 | 0.0015 |
| Upper throat | Pet food | 0.0010 | 0.0015 | 0.0001 |
| Water in the rumen | Spreading/Compost | 0.0000 | 0.0000 | 0.0000 |

Table 41: Total weighting by coproducts for Limousine Young Bulls reared in Pasture

| COPRODUCT | Destination | Limousine/young bull/pasture | | |
| --- | --- | --- | --- | --- |
|  |  | **Biophysical Cumulative share** | **Mass Cumulative share** | **Economic Cumulative share** |
| Abomasum | Human food | 0.0125 | 0.0022 | 0.0016 |
| Abomasum fat | Fat and greaves C3 | 0.0011 | 0.0005 | 0.0000 |
| Aponeurosis | Human food | 0.0291 | 0.0369 | 0.0358 |
| Bile | PAP C3 | 0.0000 | 0.0001 | 0.0000 |
| Blood | PAP C3 | 0.0160 | 0.0263 | 0.0057 |
| Blood | Pet food | 0.0023 | 0.0038 | 0.0003 |
| Bones | Gelatin C3 | 0.0557 | 0.0828 | 0.0002 |
| Bones of head, brain, eyes and teeth | C1-C2 for disposal | 0.0000 | 0.0000 | 0.0000 |
| Cheek | Human food | 0.0010 | 0.0013 | 0.0028 |
| Cheek | Human food | 0.0017 | 0.0024 | 0.0051 |
| Cheek trimmings | Pet food | 0.0004 | 0.0006 | 0.0000 |
| Chops | Pet food | 0.0018 | 0.0018 | 0.0001 |
| Contents of intestines | Spreading/Compost | 0.0000 | 0.0000 | 0.0000 |
| Contents of the rumen | Spreading/Compost | 0.0000 | 0.0000 | 0.0000 |
| Ears | PAP C3 | 0.0008 | 0.0008 | 0.0001 |
| Esophagus | Pet food | 0.0008 | 0.0012 | 0.0001 |
| Fat | Fat and greaves C3 | 0.0962 | 0.0886 | 0.0078 |
| Fat around heart | Fat and greaves C3 | 0.0026 | 0.0024 | 0.0002 |
| Fat in the kidney | Fat and greaves C3 | 0.0004 | 0.0004 | 0.0000 |
| Feet (without hooves) | Gelatin C3 | 0.0112 | 0.0159 | 0.0000 |
| Floatation fat | Spreading/Compost | 0.0000 | 0.0000 | 0.0000 |
| Forehead | C1-C2 for disposal | 0.0000 | 0.0000 | 0.0000 |
| Forelock | PAP C3 | 0.0025 | 0.0010 | 0.0001 |
| Gallbladder | Pet food | 0.0003 | 0.0005 | 0.0000 |
| Head trimmings | Pet food | 0.0023 | 0.0033 | 0.0002 |
| Heart | Human food | 0.0027 | 0.0040 | 0.0008 |
| Heart trimmings | Pet food | 0.0003 | 0.0005 | 0.0000 |
| Hide | Skin tannery C3 | 0.0619 | 0.0620 | 0.0998 |
| Hooves | PAP C3 | 0.0071 | 0.0028 | 0.0002 |
| Horns | PAP C3 | 0.0016 | 0.0006 | 0.0001 |
| Kidney | Human food | 0.0017 | 0.0024 | 0.0009 |
| Large intestine | C1-C2 for disposal | 0.0000 | 0.0000 | 0.0000 |
| Liver | Human food | 0.0478 | 0.0160 | 0.0075 |
| Liver trimmings | Pet food | 0.0057 | 0.0018 | 0.0001 |
| Lower jaw | PAP C3 | 0.0030 | 0.0045 | 0.0004 |
| Lungs | Pet food | 0.0074 | 0.0090 | 0.0006 |
| Mask | Skin tannery C3 | 0.0042 | 0.0042 | 0.0068 |
| Mesenteric fat | C1-C2 for disposal | 0.0000 | 0.0000 | 0.0000 |
| Muscle | Human food | 0.3576 | 0.4949 | 0.7980 |
| Muzzle | Human food | 0.0022 | 0.0022 | 0.0021 |
| Omasum | Human food | 0.0111 | 0.0019 | 0.0014 |
| Omasum fat | Fat and greaves C3 | 0.0030 | 0.0013 | 0.0001 |
| Rumen and forestomach | Human food | 0.0591 | 0.0103 | 0.0074 |
| Rumen fat | Fat and greaves C3 | 0.0044 | 0.0019 | 0.0002 |
| Sanitary seizures | C1-C2 for disposal | 0.0000 | 0.0000 | 0.0000 |
| Screening and sifting wastes | C1-C2 for disposal | 0.0000 | 0.0000 | 0.0000 |
| Small intestine | PAP C3 | 0.0688 | 0.0120 | 0.0007 |
| Spinal cord | C1-C2 for disposal | 0.0000 | 0.0000 | 0.0000 |
| Spinal cord waste | C1-C2 for disposal | 0.0000 | 0.0000 | 0.0000 |
| Spine | C1-C2 for disposal | 0.0000 | 0.0000 | 0.0000 |
| Spleen | Pet food | 0.0018 | 0.0024 | 0.0002 |
| Stillborn | PAP C3 | 0.0355 | 0.0059 | 0.0000 |
| Tallow | Fat and greaves C3 | 0.0665 | 0.0614 | 0.0054 |
| Tongue | Human food | 0.0022 | 0.0035 | 0.0054 |
| Tonsil | C1-C2 for disposal | 0.0000 | 0.0000 | 0.0000 |
| Trachea | Pet food | 0.0016 | 0.0016 | 0.0001 |
| Udder | Pet food | 0.0029 | 0.0188 | 0.0013 |
| Upper throat | Pet food | 0.0009 | 0.0013 | 0.0001 |
| Water in the rumen | Spreading/Compost | 0.0000 | 0.0000 | 0.0000 |

Table 42: Total weighting by coproducts for Limousine Heifers reared in Pasture

| COPRODUCT | Destination | Limousine/heifer/pasture | | |
| --- | --- | --- | --- | --- |
|  |  | **Biophysical Cumulative share** | **Mass Cumulative share** | **Economic Cumulative share** |
| Abomasum | Human food | 0.0135 | 0.0025 | 0.0018 |
| Abomasum fat | Fat and greaves C3 | 0.0011 | 0.0005 | 0.0000 |
| Aponeurosis | Human food | 0.0271 | 0.0351 | 0.0348 |
| Bile | PAP C3 | 0.0000 | 0.0001 | 0.0000 |
| Blood | PAP C3 | 0.0176 | 0.0295 | 0.0065 |
| Blood | Pet food | 0.0025 | 0.0042 | 0.0003 |
| Bones | Gelatin C3 | 0.0517 | 0.0786 | 0.0002 |
| Bones of head, brain, eyes and teeth | C1-C2 for disposal | 0.0000 | 0.0000 | 0.0000 |
| Cheek | Human food | 0.0010 | 0.0015 | 0.0032 |
| Cheek | Human food | 0.0019 | 0.0027 | 0.0059 |
| Cheek trimmings | Pet food | 0.0005 | 0.0007 | 0.0001 |
| Chops | Pet food | 0.0019 | 0.0020 | 0.0001 |
| Contents of intestines | Spreading/Compost | 0.0000 | 0.0000 | 0.0000 |
| Contents of the rumen | Spreading/Compost | 0.0000 | 0.0000 | 0.0000 |
| Ears | PAP C3 | 0.0009 | 0.0010 | 0.0001 |
| Esophagus | Pet food | 0.0009 | 0.0014 | 0.0001 |
| Fat | Fat and greaves C3 | 0.0886 | 0.0841 | 0.0076 |
| Fat around heart | Fat and greaves C3 | 0.0028 | 0.0027 | 0.0002 |
| Fat in the kidney | Fat and greaves C3 | 0.0004 | 0.0004 | 0.0000 |
| Feet (without hooves) | Gelatin C3 | 0.0123 | 0.0177 | 0.0001 |
| Floatation fat | Spreading/Compost | 0.0000 | 0.0000 | 0.0000 |
| Forehead | C1-C2 for disposal | 0.0000 | 0.0000 | 0.0000 |
| Forelock | PAP C3 | 0.0028 | 0.0011 | 0.0001 |
| Gallbladder | Pet food | 0.0003 | 0.0005 | 0.0000 |
| Head trimmings | Pet food | 0.0026 | 0.0037 | 0.0003 |
| Heart | Human food | 0.0030 | 0.0044 | 0.0009 |
| Heart trimmings | Pet food | 0.0003 | 0.0005 | 0.0000 |
| Hide | Skin tannery C3 | 0.0682 | 0.0695 | 0.1146 |
| Hooves | PAP C3 | 0.0078 | 0.0031 | 0.0003 |
| Horns | PAP C3 | 0.0019 | 0.0007 | 0.0001 |
| Kidney | Human food | 0.0019 | 0.0027 | 0.0011 |
| Large intestine | C1-C2 for disposal | 0.0000 | 0.0000 | 0.0000 |
| Liver | Human food | 0.0514 | 0.0180 | 0.0086 |
| Liver trimmings | Pet food | 0.0059 | 0.0020 | 0.0001 |
| Lower jaw | PAP C3 | 0.0032 | 0.0049 | 0.0004 |
| Lungs | Pet food | 0.0082 | 0.0101 | 0.0007 |
| Mask | Skin tannery C3 | 0.0046 | 0.0047 | 0.0077 |
| Mesenteric fat | C1-C2 for disposal | 0.0000 | 0.0000 | 0.0000 |
| Muscle | Human food | 0.3332 | 0.4698 | 0.7759 |
| Muzzle | Human food | 0.0024 | 0.0025 | 0.0024 |
| Omasum | Human food | 0.0121 | 0.0022 | 0.0016 |
| Omasum fat | Fat and greaves C3 | 0.0032 | 0.0015 | 0.0001 |
| Rumen and forestomach | Human food | 0.0633 | 0.0116 | 0.0086 |
| Rumen fat | Fat and greaves C3 | 0.0049 | 0.0022 | 0.0002 |
| Sanitary seizures | C1-C2 for disposal | 0.0000 | 0.0000 | 0.0000 |
| Screening and sifting wastes | C1-C2 for disposal | 0.0000 | 0.0000 | 0.0000 |
| Small intestine | PAP C3 | 0.0734 | 0.0134 | 0.0008 |
| Spinal cord | C1-C2 for disposal | 0.0000 | 0.0000 | 0.0000 |
| Spinal cord waste | C1-C2 for disposal | 0.0000 | 0.0000 | 0.0000 |
| Spine | C1-C2 for disposal | 0.0000 | 0.0000 | 0.0000 |
| Spleen | Pet food | 0.0019 | 0.0027 | 0.0002 |
| Stillborn | PAP C3 | 0.0379 | 0.0066 | 0.0000 |
| Tallow | Fat and greaves C3 | 0.0724 | 0.0689 | 0.0062 |
| Tongue | Human food | 0.0025 | 0.0039 | 0.0062 |
| Tonsil | C1-C2 for disposal | 0.0000 | 0.0000 | 0.0000 |
| Trachea | Pet food | 0.0017 | 0.0017 | 0.0001 |
| Udder | Pet food | 0.0032 | 0.0210 | 0.0015 |
| Upper throat | Pet food | 0.0010 | 0.0015 | 0.0001 |
| Water in the rumen | Spreading/Compost | 0.0000 | 0.0000 | 0.0000 |

Table 43: Total weighting by coproducts for Limousine Cull Cows reared in Pasture

| COPRODUCT | Destination | Limousine/Cull cow/pasture | | |
| --- | --- | --- | --- | --- |
|  |  | **Biophysical Cumulative share** | **Mass Cumulative share** | **Economic Cumulative share** |
| Abomasum | Human food | 0.0146 | 0.0026 | 0.0020 |
| Abomasum fat | Fat and greaves C3 | 0.0011 | 0.0005 | 0.0000 |
| Aponeurosis | Human food | 0.0257 | 0.0341 | 0.0342 |
| Bile | PAP C3 | 0.0000 | 0.0001 | 0.0000 |
| Blood | PAP C3 | 0.0181 | 0.0311 | 0.0070 |
| Blood | Pet food | 0.0026 | 0.0045 | 0.0003 |
| Bones | Gelatin C3 | 0.0489 | 0.0764 | 0.0002 |
| Bones of head, brain, eyes and teeth | C1-C2 for disposal | 0.0000 | 0.0000 | 0.0000 |
| Cheek | Human food | 0.0011 | 0.0016 | 0.0035 |
| Cheek | Human food | 0.0020 | 0.0029 | 0.0063 |
| Cheek trimmings | Pet food | 0.0005 | 0.0007 | 0.0001 |
| Chops | Pet food | 0.0020 | 0.0021 | 0.0002 |
| Contents of intestines | Spreading/Compost | 0.0000 | 0.0000 | 0.0000 |
| Contents of the rumen | Spreading/Compost | 0.0000 | 0.0000 | 0.0000 |
| Ears | PAP C3 | 0.0008 | 0.0010 | 0.0001 |
| Esophagus | Pet food | 0.0010 | 0.0015 | 0.0001 |
| Fat | Fat and greaves C3 | 0.0828 | 0.0818 | 0.0074 |
| Fat around heart | Fat and greaves C3 | 0.0029 | 0.0029 | 0.0003 |
| Fat in the kidney | Fat and greaves C3 | 0.0004 | 0.0004 | 0.0000 |
| Feet (without hooves) | Gelatin C3 | 0.0127 | 0.0187 | 0.0001 |
| Floatation fat | Spreading/Compost | 0.0000 | 0.0000 | 0.0000 |
| Forehead | C1-C2 for disposal | 0.0000 | 0.0000 | 0.0000 |
| Forelock | PAP C3 | 0.0027 | 0.0011 | 0.0001 |
| Gallbladder | Pet food | 0.0003 | 0.0005 | 0.0000 |
| Head trimmings | Pet food | 0.0027 | 0.0040 | 0.0003 |
| Heart | Human food | 0.0031 | 0.0047 | 0.0010 |
| Heart trimmings | Pet food | 0.0003 | 0.0005 | 0.0000 |
| Hide | Skin tannery C3 | 0.0700 | 0.0733 | 0.1223 |
| Hooves | PAP C3 | 0.0079 | 0.0032 | 0.0003 |
| Horns | PAP C3 | 0.0018 | 0.0007 | 0.0001 |
| Kidney | Human food | 0.0019 | 0.0029 | 0.0012 |
| Large intestine | C1-C2 for disposal | 0.0000 | 0.0000 | 0.0000 |
| Liver | Human food | 0.0554 | 0.0190 | 0.0092 |
| Liver trimmings | Pet food | 0.0064 | 0.0021 | 0.0002 |
| Lower jaw | PAP C3 | 0.0033 | 0.0052 | 0.0004 |
| Lungs | Pet food | 0.0083 | 0.0105 | 0.0008 |
| Mask | Skin tannery C3 | 0.0047 | 0.0050 | 0.0083 |
| Mesenteric fat | C1-C2 for disposal | 0.0000 | 0.0000 | 0.0000 |
| Muscle | Human food | 0.3158 | 0.4569 | 0.7640 |
| Muzzle | Human food | 0.0025 | 0.0026 | 0.0026 |
| Omasum | Human food | 0.0132 | 0.0024 | 0.0018 |
| Omasum fat | Fat and greaves C3 | 0.0035 | 0.0016 | 0.0001 |
| Rumen and forestomach | Human food | 0.0683 | 0.0121 | 0.0091 |
| Rumen fat | Fat and greaves C3 | 0.0052 | 0.0024 | 0.0002 |
| Sanitary seizures | C1-C2 for disposal | 0.0000 | 0.0000 | 0.0000 |
| Screening and sifting wastes | C1-C2 for disposal | 0.0000 | 0.0000 | 0.0000 |
| Small intestine | PAP C3 | 0.0795 | 0.0141 | 0.0009 |
| Spinal cord | C1-C2 for disposal | 0.0000 | 0.0000 | 0.0000 |
| Spinal cord waste | C1-C2 for disposal | 0.0000 | 0.0000 | 0.0000 |
| Spine | C1-C2 for disposal | 0.0000 | 0.0000 | 0.0000 |
| Spleen | Pet food | 0.0020 | 0.0029 | 0.0002 |
| Stillborn | PAP C3 | 0.0415 | 0.0071 | 0.0000 |
| Tallow | Fat and greaves C3 | 0.0734 | 0.0727 | 0.0066 |
| Tongue | Human food | 0.0026 | 0.0042 | 0.0067 |
| Tonsil | C1-C2 for disposal | 0.0000 | 0.0000 | 0.0000 |
| Trachea | Pet food | 0.0018 | 0.0019 | 0.0001 |
| Udder | Pet food | 0.0033 | 0.0222 | 0.0016 |
| Upper throat | Pet food | 0.0011 | 0.0016 | 0.0001 |
| Water in the rumen | Spreading/Compost | 0.0000 | 0.0000 | 0.0000 |

Table 44: Total weighting by coproducts for Limousine Beef reared in Pasture

| COPRODUCT | Destination | Limousine/beef/pasture | | |
| --- | --- | --- | --- | --- |
|  |  | **Biophysical Cumulative share** | **Mass Cumulative share** | **Economic Cumulative share** |
| Abomasum | Human food | 0.0142 | 0.0024 | 0.0018 |
| Abomasum fat | Fat and greaves C3 | 0.0011 | 0.0005 | 0.0000 |
| Aponeurosis | Human food | 0.0273 | 0.0355 | 0.0350 |
| Bile | PAP C3 | 0.0000 | 0.0001 | 0.0000 |
| Blood | PAP C3 | 0.0170 | 0.0287 | 0.0063 |
| Blood | Pet food | 0.0024 | 0.0040 | 0.0003 |
| Bones | Gelatin C3 | 0.0519 | 0.0796 | 0.0002 |
| Bones of head, brain, eyes and teeth | C1-C2 for disposal | 0.0000 | 0.0000 | 0.0000 |
| Cheek | Human food | 0.0010 | 0.0015 | 0.0032 |
| Cheek | Human food | 0.0019 | 0.0027 | 0.0058 |
| Cheek trimmings | Pet food | 0.0005 | 0.0007 | 0.0001 |
| Chops | Pet food | 0.0019 | 0.0020 | 0.0001 |
| Contents of intestines | Spreading/Compost | 0.0000 | 0.0000 | 0.0000 |
| Contents of the rumen | Spreading/Compost | 0.0000 | 0.0000 | 0.0000 |
| Ears | PAP C3 | 0.0009 | 0.0010 | 0.0001 |
| Esophagus | Pet food | 0.0009 | 0.0013 | 0.0001 |
| Fat | Fat and greaves C3 | 0.0881 | 0.0852 | 0.0076 |
| Fat around heart | Fat and greaves C3 | 0.0028 | 0.0027 | 0.0002 |
| Fat in the kidney | Fat and greaves C3 | 0.0004 | 0.0004 | 0.0000 |
| Feet (without hooves) | Gelatin C3 | 0.0119 | 0.0173 | 0.0001 |
| Floatation fat | Spreading/Compost | 0.0000 | 0.0000 | 0.0000 |
| Forehead | C1-C2 for disposal | 0.0000 | 0.0000 | 0.0000 |
| Forelock | PAP C3 | 0.0028 | 0.0011 | 0.0001 |
| Gallbladder | Pet food | 0.0003 | 0.0005 | 0.0000 |
| Head trimmings | Pet food | 0.0025 | 0.0036 | 0.0003 |
| Heart | Human food | 0.0029 | 0.0043 | 0.0009 |
| Heart trimmings | Pet food | 0.0003 | 0.0005 | 0.0000 |
| Hide | Skin tannery C3 | 0.0656 | 0.0675 | 0.1106 |
| Hooves | PAP C3 | 0.0077 | 0.0031 | 0.0003 |
| Horns | PAP C3 | 0.0018 | 0.0007 | 0.0001 |
| Kidney | Human food | 0.0019 | 0.0027 | 0.0011 |
| Large intestine | C1-C2 for disposal | 0.0000 | 0.0000 | 0.0000 |
| Liver | Human food | 0.0525 | 0.0175 | 0.0084 |
| Liver trimmings | Pet food | 0.0061 | 0.0020 | 0.0001 |
| Lower jaw | PAP C3 | 0.0031 | 0.0048 | 0.0004 |
| Lungs | Pet food | 0.0079 | 0.0098 | 0.0007 |
| Mask | Skin tannery C3 | 0.0044 | 0.0045 | 0.0074 |
| Mesenteric fat | C1-C2 for disposal | 0.0000 | 0.0000 | 0.0000 |
| Muscle | Human food | 0.3350 | 0.4759 | 0.7815 |
| Muzzle | Human food | 0.0024 | 0.0024 | 0.0024 |
| Omasum | Human food | 0.0127 | 0.0022 | 0.0016 |
| Omasum fat | Fat and greaves C3 | 0.0033 | 0.0015 | 0.0001 |
| Rumen and forestomach | Human food | 0.0644 | 0.0111 | 0.0082 |
| Rumen fat | Fat and greaves C3 | 0.0050 | 0.0022 | 0.0002 |
| Sanitary seizures | C1-C2 for disposal | 0.0000 | 0.0000 | 0.0000 |
| Screening and sifting wastes | C1-C2 for disposal | 0.0000 | 0.0000 | 0.0000 |
| Small intestine | PAP C3 | 0.0750 | 0.0130 | 0.0008 |
| Spinal cord | C1-C2 for disposal | 0.0000 | 0.0000 | 0.0000 |
| Spinal cord waste | C1-C2 for disposal | 0.0000 | 0.0000 | 0.0000 |
| Spine | C1-C2 for disposal | 0.0000 | 0.0000 | 0.0000 |
| Spleen | Pet food | 0.0019 | 0.0027 | 0.0002 |
| Stillborn | PAP C3 | 0.0392 | 0.0065 | 0.0000 |
| Tallow | Fat and greaves C3 | 0.0691 | 0.0670 | 0.0060 |
| Tongue | Human food | 0.0023 | 0.0038 | 0.0059 |
| Tonsil | C1-C2 for disposal | 0.0000 | 0.0000 | 0.0000 |
| Trachea | Pet food | 0.0017 | 0.0017 | 0.0001 |
| Udder | Pet food | 0.0031 | 0.0205 | 0.0015 |
| Upper throat | Pet food | 0.0010 | 0.0015 | 0.0001 |
| Water in the rumen | Spreading/Compost | 0.0000 | 0.0000 | 0.0000 |

Table 45: Total weighting by coproducts for Limousine Young Bulls reared in Stall

| COPRODUCT | Destination | Limousine/young bull/stall | | |
| --- | --- | --- | --- | --- |
|  |  | **Biophysical Cumulative share** | **Mass Cumulative share** | **Economic Cumulative share** |
| Abomasum | Human food | 0.0119 | 0.0022 | 0.0016 |
| Abomasum fat | Fat and greaves C3 | 0.0011 | 0.0005 | 0.0000 |
| Aponeurosis | Human food | 0.0287 | 0.0369 | 0.0358 |
| Bile | PAP C3 | 0.0000 | 0.0001 | 0.0000 |
| Blood | PAP C3 | 0.0158 | 0.0263 | 0.0057 |
| Blood | Pet food | 0.0023 | 0.0038 | 0.0003 |
| Bones | Gelatin C3 | 0.0565 | 0.0828 | 0.0002 |
| Bones of head, brain, eyes and teeth | C1-C2 for disposal | 0.0000 | 0.0000 | 0.0000 |
| Cheek | Human food | 0.0009 | 0.0013 | 0.0028 |
| Cheek | Human food | 0.0017 | 0.0024 | 0.0051 |
| Cheek trimmings | Pet food | 0.0004 | 0.0006 | 0.0000 |
| Chops | Pet food | 0.0018 | 0.0018 | 0.0001 |
| Contents of intestines | Spreading/Compost | 0.0000 | 0.0000 | 0.0000 |
| Contents of the rumen | Spreading/Compost | 0.0000 | 0.0000 | 0.0000 |
| Ears | PAP C3 | 0.0008 | 0.0008 | 0.0001 |
| Esophagus | Pet food | 0.0008 | 0.0012 | 0.0001 |
| Fat | Fat and greaves C3 | 0.1052 | 0.0886 | 0.0078 |
| Fat around heart | Fat and greaves C3 | 0.0029 | 0.0024 | 0.0002 |
| Fat in the kidney | Fat and greaves C3 | 0.0004 | 0.0004 | 0.0000 |
| Feet (without hooves) | Gelatin C3 | 0.0112 | 0.0159 | 0.0000 |
| Floatation fat | Spreading/Compost | 0.0000 | 0.0000 | 0.0000 |
| Forehead | C1-C2 for disposal | 0.0000 | 0.0000 | 0.0000 |
| Forelock | PAP C3 | 0.0024 | 0.0010 | 0.0001 |
| Gallbladder | Pet food | 0.0003 | 0.0005 | 0.0000 |
| Head trimmings | Pet food | 0.0023 | 0.0033 | 0.0002 |
| Heart | Human food | 0.0027 | 0.0040 | 0.0008 |
| Heart trimmings | Pet food | 0.0003 | 0.0005 | 0.0000 |
| Hide | Skin tannery C3 | 0.0611 | 0.0620 | 0.0998 |
| Hooves | PAP C3 | 0.0070 | 0.0028 | 0.0002 |
| Horns | PAP C3 | 0.0015 | 0.0006 | 0.0001 |
| Kidney | Human food | 0.0017 | 0.0024 | 0.0009 |
| Large intestine | C1-C2 for disposal | 0.0000 | 0.0000 | 0.0000 |
| Liver | Human food | 0.0456 | 0.0160 | 0.0075 |
| Liver trimmings | Pet food | 0.0054 | 0.0018 | 0.0001 |
| Lower jaw | PAP C3 | 0.0030 | 0.0045 | 0.0004 |
| Lungs | Pet food | 0.0073 | 0.0090 | 0.0006 |
| Mask | Skin tannery C3 | 0.0042 | 0.0042 | 0.0068 |
| Mesenteric fat | C1-C2 for disposal | 0.0000 | 0.0000 | 0.0000 |
| Muscle | Human food | 0.3553 | 0.4949 | 0.7980 |
| Muzzle | Human food | 0.0021 | 0.0022 | 0.0021 |
| Omasum | Human food | 0.0105 | 0.0019 | 0.0014 |
| Omasum fat | Fat and greaves C3 | 0.0031 | 0.0013 | 0.0001 |
| Rumen and forestomach | Human food | 0.0560 | 0.0103 | 0.0074 |
| Rumen fat | Fat and greaves C3 | 0.0045 | 0.0019 | 0.0002 |
| Sanitary seizures | C1-C2 for disposal | 0.0000 | 0.0000 | 0.0000 |
| Screening and sifting wastes | C1-C2 for disposal | 0.0000 | 0.0000 | 0.0000 |
| Small intestine | PAP C3 | 0.0652 | 0.0120 | 0.0007 |
| Spinal cord | C1-C2 for disposal | 0.0000 | 0.0000 | 0.0000 |
| Spinal cord waste | C1-C2 for disposal | 0.0000 | 0.0000 | 0.0000 |
| Spine | C1-C2 for disposal | 0.0000 | 0.0000 | 0.0000 |
| Spleen | Pet food | 0.0018 | 0.0024 | 0.0002 |
| Stillborn | PAP C3 | 0.0336 | 0.0059 | 0.0000 |
| Tallow | Fat and greaves C3 | 0.0728 | 0.0614 | 0.0054 |
| Tongue | Human food | 0.0022 | 0.0035 | 0.0054 |
| Tonsil | C1-C2 for disposal | 0.0000 | 0.0000 | 0.0000 |
| Trachea | Pet food | 0.0016 | 0.0016 | 0.0001 |
| Udder | Pet food | 0.0030 | 0.0188 | 0.0013 |
| Upper throat | Pet food | 0.0009 | 0.0013 | 0.0001 |
| Water in the rumen | Spreading/Compost | 0.0000 | 0.0000 | 0.0000 |

Table 46: Total weighting by coproducts for Limousine Heifers reared in Stall

| COPRODUCT | Destination | Limousine/heifer/stall | | |
| --- | --- | --- | --- | --- |
|  |  | **Biophysical Cumulative share** | **Mass Cumulative share** | **Economic Cumulative share** |
| Abomasum | Human food | 0.0128 | 0.0025 | 0.0018 |
| Abomasum fat | Fat and greaves C3 | 0.0011 | 0.0005 | 0.0000 |
| Aponeurosis | Human food | 0.0269 | 0.0351 | 0.0348 |
| Bile | PAP C3 | 0.0000 | 0.0001 | 0.0000 |
| Blood | PAP C3 | 0.0175 | 0.0295 | 0.0065 |
| Blood | Pet food | 0.0025 | 0.0042 | 0.0003 |
| Bones | Gelatin C3 | 0.0525 | 0.0786 | 0.0002 |
| Bones of head, brain, eyes and teeth | C1-C2 for disposal | 0.0000 | 0.0000 | 0.0000 |
| Cheek | Human food | 0.0010 | 0.0015 | 0.0032 |
| Cheek | Human food | 0.0019 | 0.0027 | 0.0059 |
| Cheek trimmings | Pet food | 0.0005 | 0.0007 | 0.0001 |
| Chops | Pet food | 0.0019 | 0.0020 | 0.0001 |
| Contents of intestines | Spreading/Compost | 0.0000 | 0.0000 | 0.0000 |
| Contents of the rumen | Spreading/Compost | 0.0000 | 0.0000 | 0.0000 |
| Ears | PAP C3 | 0.0009 | 0.0010 | 0.0001 |
| Esophagus | Pet food | 0.0009 | 0.0014 | 0.0001 |
| Fat | Fat and greaves C3 | 0.0968 | 0.0841 | 0.0076 |
| Fat around heart | Fat and greaves C3 | 0.0031 | 0.0027 | 0.0002 |
| Fat in the kidney | Fat and greaves C3 | 0.0004 | 0.0004 | 0.0000 |
| Feet (without hooves) | Gelatin C3 | 0.0123 | 0.0177 | 0.0001 |
| Floatation fat | Spreading/Compost | 0.0000 | 0.0000 | 0.0000 |
| Forehead | C1-C2 for disposal | 0.0000 | 0.0000 | 0.0000 |
| Forelock | PAP C3 | 0.0028 | 0.0011 | 0.0001 |
| Gallbladder | Pet food | 0.0003 | 0.0005 | 0.0000 |
| Head trimmings | Pet food | 0.0026 | 0.0037 | 0.0003 |
| Heart | Human food | 0.0030 | 0.0044 | 0.0009 |
| Heart trimmings | Pet food | 0.0003 | 0.0005 | 0.0000 |
| Hide | Skin tannery C3 | 0.0675 | 0.0695 | 0.1146 |
| Hooves | PAP C3 | 0.0077 | 0.0031 | 0.0003 |
| Horns | PAP C3 | 0.0018 | 0.0007 | 0.0001 |
| Kidney | Human food | 0.0019 | 0.0027 | 0.0011 |
| Large intestine | C1-C2 for disposal | 0.0000 | 0.0000 | 0.0000 |
| Liver | Human food | 0.0490 | 0.0180 | 0.0086 |
| Liver trimmings | Pet food | 0.0056 | 0.0020 | 0.0001 |
| Lower jaw | PAP C3 | 0.0032 | 0.0049 | 0.0004 |
| Lungs | Pet food | 0.0081 | 0.0101 | 0.0007 |
| Mask | Skin tannery C3 | 0.0045 | 0.0047 | 0.0077 |
| Mesenteric fat | C1-C2 for disposal | 0.0000 | 0.0000 | 0.0000 |
| Muscle | Human food | 0.3318 | 0.4698 | 0.7759 |
| Muzzle | Human food | 0.0024 | 0.0025 | 0.0024 |
| Omasum | Human food | 0.0115 | 0.0022 | 0.0016 |
| Omasum fat | Fat and greaves C3 | 0.0033 | 0.0015 | 0.0001 |
| Rumen and forestomach | Human food | 0.0599 | 0.0116 | 0.0086 |
| Rumen fat | Fat and greaves C3 | 0.0049 | 0.0022 | 0.0002 |
| Sanitary seizures | C1-C2 for disposal | 0.0000 | 0.0000 | 0.0000 |
| Screening and sifting wastes | C1-C2 for disposal | 0.0000 | 0.0000 | 0.0000 |
| Small intestine | PAP C3 | 0.0695 | 0.0134 | 0.0008 |
| Spinal cord | C1-C2 for disposal | 0.0000 | 0.0000 | 0.0000 |
| Spinal cord waste | C1-C2 for disposal | 0.0000 | 0.0000 | 0.0000 |
| Spine | C1-C2 for disposal | 0.0000 | 0.0000 | 0.0000 |
| Spleen | Pet food | 0.0019 | 0.0027 | 0.0002 |
| Stillborn | PAP C3 | 0.0359 | 0.0066 | 0.0000 |
| Tallow | Fat and greaves C3 | 0.0792 | 0.0689 | 0.0062 |
| Tongue | Human food | 0.0025 | 0.0039 | 0.0062 |
| Tonsil | C1-C2 for disposal | 0.0000 | 0.0000 | 0.0000 |
| Trachea | Pet food | 0.0017 | 0.0017 | 0.0001 |
| Udder | Pet food | 0.0033 | 0.0210 | 0.0015 |
| Upper throat | Pet food | 0.0010 | 0.0015 | 0.0001 |
| Water in the rumen | Spreading/Compost | 0.0000 | 0.0000 | 0.0000 |

Table 47: Total weighting by coproducts for Limousine Cull Cows reared in Stall

| COPRODUCT | Destination | Limousine/Cull cow/stall | | |
| --- | --- | --- | --- | --- |
|  |  | **Biophysical Cumulative share** | **Mass Cumulative share** | **Economic Cumulative share** |
| Abomasum | Human food | 0.0139 | 0.0026 | 0.0020 |
| Abomasum fat | Fat and greaves C3 | 0.0011 | 0.0005 | 0.0000 |
| Aponeurosis | Human food | 0.0254 | 0.0341 | 0.0342 |
| Bile | PAP C3 | 0.0000 | 0.0001 | 0.0000 |
| Blood | PAP C3 | 0.0179 | 0.0311 | 0.0070 |
| Blood | Pet food | 0.0026 | 0.0045 | 0.0003 |
| Bones | Gelatin C3 | 0.0496 | 0.0764 | 0.0002 |
| Bones of head, brain, eyes and teeth | C1-C2 for disposal | 0.0000 | 0.0000 | 0.0000 |
| Cheek | Human food | 0.0011 | 0.0016 | 0.0035 |
| Cheek | Human food | 0.0019 | 0.0029 | 0.0063 |
| Cheek trimmings | Pet food | 0.0005 | 0.0007 | 0.0001 |
| Chops | Pet food | 0.0020 | 0.0021 | 0.0002 |
| Contents of intestines | Spreading/Compost | 0.0000 | 0.0000 | 0.0000 |
| Contents of the rumen | Spreading/Compost | 0.0000 | 0.0000 | 0.0000 |
| Ears | PAP C3 | 0.0008 | 0.0010 | 0.0001 |
| Esophagus | Pet food | 0.0010 | 0.0015 | 0.0001 |
| Fat | Fat and greaves C3 | 0.0909 | 0.0818 | 0.0074 |
| Fat around heart | Fat and greaves C3 | 0.0032 | 0.0029 | 0.0003 |
| Fat in the kidney | Fat and greaves C3 | 0.0004 | 0.0004 | 0.0000 |
| Feet (without hooves) | Gelatin C3 | 0.0126 | 0.0187 | 0.0001 |
| Floatation fat | Spreading/Compost | 0.0000 | 0.0000 | 0.0000 |
| Forehead | C1-C2 for disposal | 0.0000 | 0.0000 | 0.0000 |
| Forelock | PAP C3 | 0.0027 | 0.0011 | 0.0001 |
| Gallbladder | Pet food | 0.0003 | 0.0005 | 0.0000 |
| Head trimmings | Pet food | 0.0027 | 0.0040 | 0.0003 |
| Heart | Human food | 0.0031 | 0.0047 | 0.0010 |
| Heart trimmings | Pet food | 0.0003 | 0.0005 | 0.0000 |
| Hide | Skin tannery C3 | 0.0693 | 0.0733 | 0.1223 |
| Hooves | PAP C3 | 0.0078 | 0.0032 | 0.0003 |
| Horns | PAP C3 | 0.0018 | 0.0007 | 0.0001 |
| Kidney | Human food | 0.0019 | 0.0029 | 0.0012 |
| Large intestine | C1-C2 for disposal | 0.0000 | 0.0000 | 0.0000 |
| Liver | Human food | 0.0530 | 0.0190 | 0.0092 |
| Liver trimmings | Pet food | 0.0061 | 0.0021 | 0.0002 |
| Lower jaw | PAP C3 | 0.0033 | 0.0052 | 0.0004 |
| Lungs | Pet food | 0.0082 | 0.0105 | 0.0008 |
| Mask | Skin tannery C3 | 0.0047 | 0.0050 | 0.0083 |
| Mesenteric fat | C1-C2 for disposal | 0.0000 | 0.0000 | 0.0000 |
| Muscle | Human food | 0.3143 | 0.4569 | 0.7640 |
| Muzzle | Human food | 0.0025 | 0.0026 | 0.0026 |
| Omasum | Human food | 0.0126 | 0.0024 | 0.0018 |
| Omasum fat | Fat and greaves C3 | 0.0036 | 0.0016 | 0.0001 |
| Rumen and forestomach | Human food | 0.0650 | 0.0121 | 0.0091 |
| Rumen fat | Fat and greaves C3 | 0.0052 | 0.0024 | 0.0002 |
| Sanitary seizures | C1-C2 for disposal | 0.0000 | 0.0000 | 0.0000 |
| Screening and sifting wastes | C1-C2 for disposal | 0.0000 | 0.0000 | 0.0000 |
| Small intestine | PAP C3 | 0.0756 | 0.0141 | 0.0009 |
| Spinal cord | C1-C2 for disposal | 0.0000 | 0.0000 | 0.0000 |
| Spinal cord waste | C1-C2 for disposal | 0.0000 | 0.0000 | 0.0000 |
| Spine | C1-C2 for disposal | 0.0000 | 0.0000 | 0.0000 |
| Spleen | Pet food | 0.0020 | 0.0029 | 0.0002 |
| Stillborn | PAP C3 | 0.0394 | 0.0071 | 0.0000 |
| Tallow | Fat and greaves C3 | 0.0806 | 0.0727 | 0.0066 |
| Tongue | Human food | 0.0026 | 0.0042 | 0.0067 |
| Tonsil | C1-C2 for disposal | 0.0000 | 0.0000 | 0.0000 |
| Trachea | Pet food | 0.0018 | 0.0019 | 0.0001 |
| Udder | Pet food | 0.0034 | 0.0222 | 0.0016 |
| Upper throat | Pet food | 0.0011 | 0.0016 | 0.0001 |
| Water in the rumen | Spreading/Compost | 0.0000 | 0.0000 | 0.0000 |

Table 48: Total weighting by coproducts for Limousine Beef reared in Stall

| COPRODUCT | Destination | Limousine/beef/stall | | |
| --- | --- | --- | --- | --- |
|  |  | **Biophysical Cumulative share** | **Mass Cumulative share** | **Economic Cumulative share** |
| Abomasum | Human food | 0.0135 | 0.0024 | 0.0018 |
| Abomasum fat | Fat and greaves C3 | 0.0011 | 0.0005 | 0.0000 |
| Aponeurosis | Human food | 0.0270 | 0.0355 | 0.0350 |
| Bile | PAP C3 | 0.0000 | 0.0001 | 0.0000 |
| Blood | PAP C3 | 0.0168 | 0.0287 | 0.0063 |
| Blood | Pet food | 0.0024 | 0.0040 | 0.0003 |
| Bones | Gelatin C3 | 0.0527 | 0.0796 | 0.0002 |
| Bones of head, brain, eyes and teeth | C1-C2 for disposal | 0.0000 | 0.0000 | 0.0000 |
| Cheek | Human food | 0.0010 | 0.0015 | 0.0032 |
| Cheek | Human food | 0.0019 | 0.0027 | 0.0058 |
| Cheek trimmings | Pet food | 0.0005 | 0.0007 | 0.0001 |
| Chops | Pet food | 0.0019 | 0.0020 | 0.0001 |
| Contents of intestines | Spreading/Compost | 0.0000 | 0.0000 | 0.0000 |
| Contents of the rumen | Spreading/Compost | 0.0000 | 0.0000 | 0.0000 |
| Ears | PAP C3 | 0.0009 | 0.0010 | 0.0001 |
| Esophagus | Pet food | 0.0009 | 0.0013 | 0.0001 |
| Fat | Fat and greaves C3 | 0.0967 | 0.0852 | 0.0076 |
| Fat around heart | Fat and greaves C3 | 0.0031 | 0.0027 | 0.0002 |
| Fat in the kidney | Fat and greaves C3 | 0.0004 | 0.0004 | 0.0000 |
| Feet (without hooves) | Gelatin C3 | 0.0118 | 0.0173 | 0.0001 |
| Floatation fat | Spreading/Compost | 0.0000 | 0.0000 | 0.0000 |
| Forehead | C1-C2 for disposal | 0.0000 | 0.0000 | 0.0000 |
| Forelock | PAP C3 | 0.0027 | 0.0011 | 0.0001 |
| Gallbladder | Pet food | 0.0003 | 0.0005 | 0.0000 |
| Head trimmings | Pet food | 0.0025 | 0.0036 | 0.0003 |
| Heart | Human food | 0.0028 | 0.0043 | 0.0009 |
| Heart trimmings | Pet food | 0.0003 | 0.0005 | 0.0000 |
| Hide | Skin tannery C3 | 0.0648 | 0.0675 | 0.1106 |
| Hooves | PAP C3 | 0.0076 | 0.0031 | 0.0003 |
| Horns | PAP C3 | 0.0018 | 0.0007 | 0.0001 |
| Kidney | Human food | 0.0018 | 0.0027 | 0.0011 |
| Large intestine | C1-C2 for disposal | 0.0000 | 0.0000 | 0.0000 |
| Liver | Human food | 0.0502 | 0.0175 | 0.0084 |
| Liver trimmings | Pet food | 0.0058 | 0.0020 | 0.0001 |
| Lower jaw | PAP C3 | 0.0031 | 0.0048 | 0.0004 |
| Lungs | Pet food | 0.0078 | 0.0098 | 0.0007 |
| Mask | Skin tannery C3 | 0.0044 | 0.0045 | 0.0074 |
| Mesenteric fat | C1-C2 for disposal | 0.0000 | 0.0000 | 0.0000 |
| Muscle | Human food | 0.3329 | 0.4759 | 0.7815 |
| Muzzle | Human food | 0.0024 | 0.0024 | 0.0024 |
| Omasum | Human food | 0.0121 | 0.0022 | 0.0016 |
| Omasum fat | Fat and greaves C3 | 0.0033 | 0.0015 | 0.0001 |
| Rumen and forestomach | Human food | 0.0612 | 0.0111 | 0.0082 |
| Rumen fat | Fat and greaves C3 | 0.0050 | 0.0022 | 0.0002 |
| Sanitary seizures | C1-C2 for disposal | 0.0000 | 0.0000 | 0.0000 |
| Screening and sifting wastes | C1-C2 for disposal | 0.0000 | 0.0000 | 0.0000 |
| Small intestine | PAP C3 | 0.0713 | 0.0130 | 0.0008 |
| Spinal cord | C1-C2 for disposal | 0.0000 | 0.0000 | 0.0000 |
| Spinal cord waste | C1-C2 for disposal | 0.0000 | 0.0000 | 0.0000 |
| Spine | C1-C2 for disposal | 0.0000 | 0.0000 | 0.0000 |
| Spleen | Pet food | 0.0019 | 0.0027 | 0.0002 |
| Stillborn | PAP C3 | 0.0372 | 0.0065 | 0.0000 |
| Tallow | Fat and greaves C3 | 0.0759 | 0.0670 | 0.0060 |
| Tongue | Human food | 0.0024 | 0.0038 | 0.0059 |
| Tonsil | C1-C2 for disposal | 0.0000 | 0.0000 | 0.0000 |
| Trachea | Pet food | 0.0017 | 0.0017 | 0.0001 |
| Udder | Pet food | 0.0032 | 0.0205 | 0.0015 |
| Upper throat | Pet food | 0.0010 | 0.0015 | 0.0001 |
| Water in the rumen | Spreading/Compost | 0.0000 | 0.0000 | 0.0000 |

Table 49: Total weighting by coproducts for Blonde d’Aquitaine Young Bulls reared in Grazing Large Area

| COPRODUCT | Destination | Blonde d’Aquitaine/young bull/grazing large area | | |
| --- | --- | --- | --- | --- |
|  |  | **Biophysical Cumulative share** | **Mass Cumulative share** | **Economic Cumulative share** |
| Abomasum | Human food | 0.0126 | 0.0020 | 0.0015 |
| Abomasum fat | Fat and greaves C3 | 0.0008 | 0.0004 | 0.0000 |
| Aponeurosis | Human food | 0.0304 | 0.0378 | 0.0362 |
| Bile | PAP C3 | 0.0000 | 0.0001 | 0.0000 |
| Blood | PAP C3 | 0.0153 | 0.0247 | 0.0053 |
| Blood | Pet food | 0.0022 | 0.0035 | 0.0002 |
| Bones | Gelatin C3 | 0.0568 | 0.0848 | 0.0002 |
| Bones of head, brain, eyes and teeth | C1-C2 for disposal | 0.0000 | 0.0000 | 0.0000 |
| Cheek | Human food | 0.0009 | 0.0012 | 0.0025 |
| Cheek | Human food | 0.0017 | 0.0023 | 0.0048 |
| Cheek trimmings | Pet food | 0.0004 | 0.0006 | 0.0000 |
| Chops | Pet food | 0.0017 | 0.0017 | 0.0001 |
| Contents of intestines | Spreading/Compost | 0.0000 | 0.0000 | 0.0000 |
| Contents of the rumen | Spreading/Compost | 0.0000 | 0.0000 | 0.0000 |
| Ears | PAP C3 | 0.0008 | 0.0008 | 0.0001 |
| Esophagus | Pet food | 0.0008 | 0.0012 | 0.0001 |
| Fat | Fat and greaves C3 | 0.0908 | 0.0908 | 0.0079 |
| Fat around heart | Fat and greaves C3 | 0.0023 | 0.0023 | 0.0002 |
| Fat in the kidney | Fat and greaves C3 | 0.0004 | 0.0004 | 0.0000 |
| Feet (without hooves) | Gelatin C3 | 0.0107 | 0.0149 | 0.0000 |
| Floatation fat | Spreading/Compost | 0.0000 | 0.0000 | 0.0000 |
| Forehead | C1-C2 for disposal | 0.0000 | 0.0000 | 0.0000 |
| Forelock | PAP C3 | 0.0025 | 0.0010 | 0.0001 |
| Gallbladder | Pet food | 0.0003 | 0.0004 | 0.0000 |
| Head trimmings | Pet food | 0.0023 | 0.0031 | 0.0002 |
| Heart | Human food | 0.0026 | 0.0037 | 0.0008 |
| Heart trimmings | Pet food | 0.0003 | 0.0004 | 0.0000 |
| Hide | Skin tannery C3 | 0.0594 | 0.0584 | 0.0929 |
| Hooves | PAP C3 | 0.0069 | 0.0026 | 0.0002 |
| Horns | PAP C3 | 0.0016 | 0.0006 | 0.0000 |
| Kidney | Human food | 0.0016 | 0.0023 | 0.0009 |
| Large intestine | C1-C2 for disposal | 0.0000 | 0.0000 | 0.0000 |
| Liver | Human food | 0.0483 | 0.0151 | 0.0070 |
| Liver trimmings | Pet food | 0.0056 | 0.0017 | 0.0001 |
| Lower jaw | PAP C3 | 0.0028 | 0.0042 | 0.0003 |
| Lungs | Pet food | 0.0071 | 0.0084 | 0.0006 |
| Mask | Skin tannery C3 | 0.0040 | 0.0040 | 0.0063 |
| Mesenteric fat | C1-C2 for disposal | 0.0000 | 0.0000 | 0.0000 |
| Muscle | Human food | 0.3722 | 0.5071 | 0.8083 |
| Muzzle | Human food | 0.0021 | 0.0020 | 0.0020 |
| Omasum | Human food | 0.0119 | 0.0019 | 0.0014 |
| Omasum fat | Fat and greaves C3 | 0.0028 | 0.0012 | 0.0001 |
| Rumen and forestomach | Human food | 0.0600 | 0.0097 | 0.0070 |
| Rumen fat | Fat and greaves C3 | 0.0045 | 0.0019 | 0.0002 |
| Sanitary seizures | C1-C2 for disposal | 0.0000 | 0.0000 | 0.0000 |
| Screening and sifting wastes | C1-C2 for disposal | 0.0000 | 0.0000 | 0.0000 |
| Small intestine | PAP C3 | 0.0697 | 0.0113 | 0.0007 |
| Spinal cord | C1-C2 for disposal | 0.0000 | 0.0000 | 0.0000 |
| Spinal cord waste | C1-C2 for disposal | 0.0000 | 0.0000 | 0.0000 |
| Spine | C1-C2 for disposal | 0.0000 | 0.0000 | 0.0000 |
| Spleen | Pet food | 0.0017 | 0.0023 | 0.0002 |
| Stillborn | PAP C3 | 0.0364 | 0.0056 | 0.0000 |
| Tallow | Fat and greaves C3 | 0.0577 | 0.0578 | 0.0050 |
| Tongue | Human food | 0.0021 | 0.0034 | 0.0051 |
| Tonsil | C1-C2 for disposal | 0.0000 | 0.0000 | 0.0000 |
| Trachea | Pet food | 0.0015 | 0.0014 | 0.0001 |
| Udder | Pet food | 0.0027 | 0.0177 | 0.0012 |
| Upper throat | Pet food | 0.0009 | 0.0012 | 0.0001 |
| Water in the rumen | Spreading/Compost | 0.0000 | 0.0000 | 0.0000 |

Table 50: Total weighting by coproducts for Blonde d’Aquitaine Heifers reared in Grazing Large Area

| COPRODUCT | Destination | Blonde d’Aquitaine/heifer/grazing large area | | |
| --- | --- | --- | --- | --- |
|  |  | **Biophysical Cumulative share** | **Mass Cumulative share** | **Economic Cumulative share** |
| Abomasum | Human food | 0.0141 | 0.0023 | 0.0017 |
| Abomasum fat | Fat and greaves C3 | 0.0011 | 0.0005 | 0.0000 |
| Aponeurosis | Human food | 0.0282 | 0.0360 | 0.0353 |
| Bile | PAP C3 | 0.0000 | 0.0001 | 0.0000 |
| Blood | PAP C3 | 0.0168 | 0.0278 | 0.0061 |
| Blood | Pet food | 0.0024 | 0.0040 | 0.0003 |
| Bones | Gelatin C3 | 0.0522 | 0.0807 | 0.0002 |
| Bones of head, brain, eyes and teeth | C1-C2 for disposal | 0.0000 | 0.0000 | 0.0000 |
| Cheek | Human food | 0.0010 | 0.0015 | 0.0031 |
| Cheek | Human food | 0.0018 | 0.0026 | 0.0055 |
| Cheek trimmings | Pet food | 0.0005 | 0.0007 | 0.0001 |
| Chops | Pet food | 0.0018 | 0.0018 | 0.0001 |
| Contents of intestines | Spreading/Compost | 0.0000 | 0.0000 | 0.0000 |
| Contents of the rumen | Spreading/Compost | 0.0000 | 0.0000 | 0.0000 |
| Ears | PAP C3 | 0.0009 | 0.0010 | 0.0001 |
| Esophagus | Pet food | 0.0009 | 0.0013 | 0.0001 |
| Fat | Fat and greaves C3 | 0.0820 | 0.0864 | 0.0077 |
| Fat around heart | Fat and greaves C3 | 0.0024 | 0.0026 | 0.0002 |
| Fat in the kidney | Fat and greaves C3 | 0.0003 | 0.0004 | 0.0000 |
| Feet (without hooves) | Gelatin C3 | 0.0117 | 0.0167 | 0.0000 |
| Floatation fat | Spreading/Compost | 0.0000 | 0.0000 | 0.0000 |
| Forehead | C1-C2 for disposal | 0.0000 | 0.0000 | 0.0000 |
| Forelock | PAP C3 | 0.0028 | 0.0011 | 0.0001 |
| Gallbladder | Pet food | 0.0003 | 0.0005 | 0.0000 |
| Head trimmings | Pet food | 0.0025 | 0.0035 | 0.0003 |
| Heart | Human food | 0.0029 | 0.0043 | 0.0009 |
| Heart trimmings | Pet food | 0.0003 | 0.0005 | 0.0000 |
| Hide | Skin tannery C3 | 0.0650 | 0.0656 | 0.1069 |
| Hooves | PAP C3 | 0.0075 | 0.0029 | 0.0002 |
| Horns | PAP C3 | 0.0019 | 0.0007 | 0.0001 |
| Kidney | Human food | 0.0018 | 0.0026 | 0.0010 |
| Large intestine | C1-C2 for disposal | 0.0000 | 0.0000 | 0.0000 |
| Liver | Human food | 0.0534 | 0.0170 | 0.0080 |
| Liver trimmings | Pet food | 0.0060 | 0.0018 | 0.0001 |
| Lower jaw | PAP C3 | 0.0029 | 0.0046 | 0.0004 |
| Lungs | Pet food | 0.0078 | 0.0095 | 0.0007 |
| Mask | Skin tannery C3 | 0.0044 | 0.0044 | 0.0072 |
| Mesenteric fat | C1-C2 for disposal | 0.0000 | 0.0000 | 0.0000 |
| Muscle | Human food | 0.3440 | 0.4824 | 0.7871 |
| Muzzle | Human food | 0.0023 | 0.0023 | 0.0023 |
| Omasum | Human food | 0.0126 | 0.0021 | 0.0015 |
| Omasum fat | Fat and greaves C3 | 0.0033 | 0.0015 | 0.0001 |
| Rumen and forestomach | Human food | 0.0662 | 0.0109 | 0.0079 |
| Rumen fat | Fat and greaves C3 | 0.0047 | 0.0021 | 0.0002 |
| Sanitary seizures | C1-C2 for disposal | 0.0000 | 0.0000 | 0.0000 |
| Screening and sifting wastes | C1-C2 for disposal | 0.0000 | 0.0000 | 0.0000 |
| Small intestine | PAP C3 | 0.0774 | 0.0127 | 0.0008 |
| Spinal cord | C1-C2 for disposal | 0.0000 | 0.0000 | 0.0000 |
| Spinal cord waste | C1-C2 for disposal | 0.0000 | 0.0000 | 0.0000 |
| Spine | C1-C2 for disposal | 0.0000 | 0.0000 | 0.0000 |
| Spleen | Pet food | 0.0018 | 0.0026 | 0.0002 |
| Stillborn | PAP C3 | 0.0404 | 0.0063 | 0.0000 |
| Tallow | Fat and greaves C3 | 0.0616 | 0.0650 | 0.0058 |
| Tongue | Human food | 0.0023 | 0.0038 | 0.0059 |
| Tonsil | C1-C2 for disposal | 0.0000 | 0.0000 | 0.0000 |
| Trachea | Pet food | 0.0016 | 0.0016 | 0.0001 |
| Udder | Pet food | 0.0029 | 0.0199 | 0.0014 |
| Upper throat | Pet food | 0.0010 | 0.0015 | 0.0001 |
| Water in the rumen | Spreading/Compost | 0.0000 | 0.0000 | 0.0000 |

Table 51: Total weighting by coproducts for Blonde d’Aquitaine Cull Cows reared in Grazing Large Area

| COPRODUCT | Destination | Blonde d’Aquitaine/Cull cow/grazing large area | | |
| --- | --- | --- | --- | --- |
|  |  | **Biophysical Cumulative share** | **Mass Cumulative share** | **Economic Cumulative share** |
| Abomasum | Human food | 0.0176 | 0.0028 | 0.0021 |
| Abomasum fat | Fat and greaves C3 | 0.0011 | 0.0005 | 0.0000 |
| Aponeurosis | Human food | 0.0244 | 0.0326 | 0.0334 |
| Bile | PAP C3 | 0.0000 | 0.0001 | 0.0000 |
| Blood | PAP C3 | 0.0192 | 0.0335 | 0.0076 |
| Blood | Pet food | 0.0027 | 0.0048 | 0.0004 |
| Bones | Gelatin C3 | 0.0433 | 0.0732 | 0.0002 |
| Bones of head, brain, eyes and teeth | C1-C2 for disposal | 0.0000 | 0.0000 | 0.0000 |
| Cheek | Human food | 0.0011 | 0.0016 | 0.0037 |
| Cheek | Human food | 0.0021 | 0.0031 | 0.0070 |
| Cheek trimmings | Pet food | 0.0006 | 0.0009 | 0.0001 |
| Chops | Pet food | 0.0021 | 0.0023 | 0.0002 |
| Contents of intestines | Spreading/Compost | 0.0000 | 0.0000 | 0.0000 |
| Contents of the rumen | Spreading/Compost | 0.0000 | 0.0000 | 0.0000 |
| Ears | PAP C3 | 0.0009 | 0.0011 | 0.0001 |
| Esophagus | Pet food | 0.0010 | 0.0015 | 0.0001 |
| Fat | Fat and greaves C3 | 0.0571 | 0.0783 | 0.0073 |
| Fat around heart | Fat and greaves C3 | 0.0023 | 0.0031 | 0.0003 |
| Fat in the kidney | Fat and greaves C3 | 0.0003 | 0.0004 | 0.0000 |
| Feet (without hooves) | Gelatin C3 | 0.0133 | 0.0202 | 0.0001 |
| Floatation fat | Spreading/Compost | 0.0000 | 0.0000 | 0.0000 |
| Forehead | C1-C2 for disposal | 0.0000 | 0.0000 | 0.0000 |
| Forelock | PAP C3 | 0.0031 | 0.0013 | 0.0001 |
| Gallbladder | Pet food | 0.0003 | 0.0005 | 0.0000 |
| Head trimmings | Pet food | 0.0029 | 0.0043 | 0.0003 |
| Heart | Human food | 0.0032 | 0.0050 | 0.0011 |
| Heart trimmings | Pet food | 0.0003 | 0.0005 | 0.0000 |
| Hide | Skin tannery C3 | 0.0749 | 0.0791 | 0.1346 |
| Hooves | PAP C3 | 0.0086 | 0.0035 | 0.0003 |
| Horns | PAP C3 | 0.0022 | 0.0009 | 0.0001 |
| Kidney | Human food | 0.0021 | 0.0031 | 0.0013 |
| Large intestine | C1-C2 for disposal | 0.0000 | 0.0000 | 0.0000 |
| Liver | Human food | 0.0664 | 0.0205 | 0.0101 |
| Liver trimmings | Pet food | 0.0077 | 0.0023 | 0.0002 |
| Lower jaw | PAP C3 | 0.0033 | 0.0056 | 0.0005 |
| Lungs | Pet food | 0.0090 | 0.0114 | 0.0009 |
| Mask | Skin tannery C3 | 0.0051 | 0.0054 | 0.0092 |
| Mesenteric fat | C1-C2 for disposal | 0.0000 | 0.0000 | 0.0000 |
| Muscle | Human food | 0.2956 | 0.4374 | 0.7457 |
| Muzzle | Human food | 0.0026 | 0.0028 | 0.0028 |
| Omasum | Human food | 0.0160 | 0.0025 | 0.0019 |
| Omasum fat | Fat and greaves C3 | 0.0035 | 0.0016 | 0.0002 |
| Rumen and forestomach | Human food | 0.0831 | 0.0131 | 0.0100 |
| Rumen fat | Fat and greaves C3 | 0.0054 | 0.0025 | 0.0002 |
| Sanitary seizures | C1-C2 for disposal | 0.0000 | 0.0000 | 0.0000 |
| Screening and sifting wastes | C1-C2 for disposal | 0.0000 | 0.0000 | 0.0000 |
| Small intestine | PAP C3 | 0.0966 | 0.0152 | 0.0009 |
| Spinal cord | C1-C2 for disposal | 0.0000 | 0.0000 | 0.0000 |
| Spinal cord waste | C1-C2 for disposal | 0.0000 | 0.0000 | 0.0000 |
| Spine | C1-C2 for disposal | 0.0000 | 0.0000 | 0.0000 |
| Spleen | Pet food | 0.0021 | 0.0031 | 0.0002 |
| Stillborn | PAP C3 | 0.0510 | 0.0077 | 0.0000 |
| Tallow | Fat and greaves C3 | 0.0570 | 0.0785 | 0.0073 |
| Tongue | Human food | 0.0026 | 0.0045 | 0.0073 |
| Tonsil | C1-C2 for disposal | 0.0000 | 0.0000 | 0.0000 |
| Trachea | Pet food | 0.0019 | 0.0020 | 0.0002 |
| Udder | Pet food | 0.0031 | 0.0240 | 0.0018 |
| Upper throat | Pet food | 0.0011 | 0.0016 | 0.0001 |
| Water in the rumen | Spreading/Compost | 0.0000 | 0.0000 | 0.0000 |

Table 52: Total weighting by coproducts for Blonde d’Aquitaine Beef reared in Grazing Large Area

| COPRODUCT | Destination | Blonde d’Aquitaine/beef/grazing large area | | |
| --- | --- | --- | --- | --- |
|  |  | **Biophysical Cumulative share** | **Mass Cumulative share** | **Economic Cumulative share** |
| Abomasum | Human food | 0.0147 | 0.0023 | 0.0017 |
| Abomasum fat | Fat and greaves C3 | 0.0011 | 0.0005 | 0.0000 |
| Aponeurosis | Human food | 0.0287 | 0.0365 | 0.0355 |
| Bile | PAP C3 | 0.0000 | 0.0001 | 0.0000 |
| Blood | PAP C3 | 0.0164 | 0.0271 | 0.0059 |
| Blood | Pet food | 0.0024 | 0.0039 | 0.0003 |
| Bones | Gelatin C3 | 0.0526 | 0.0817 | 0.0002 |
| Bones of head, brain, eyes and teeth | C1-C2 for disposal | 0.0000 | 0.0000 | 0.0000 |
| Cheek | Human food | 0.0010 | 0.0013 | 0.0029 |
| Cheek | Human food | 0.0018 | 0.0026 | 0.0054 |
| Cheek trimmings | Pet food | 0.0005 | 0.0007 | 0.0001 |
| Chops | Pet food | 0.0018 | 0.0018 | 0.0001 |
| Contents of intestines | Spreading/Compost | 0.0000 | 0.0000 | 0.0000 |
| Contents of the rumen | Spreading/Compost | 0.0000 | 0.0000 | 0.0000 |
| Ears | PAP C3 | 0.0007 | 0.0009 | 0.0001 |
| Esophagus | Pet food | 0.0008 | 0.0012 | 0.0001 |
| Fat | Fat and greaves C3 | 0.0790 | 0.0875 | 0.0077 |
| Fat around heart | Fat and greaves C3 | 0.0023 | 0.0026 | 0.0002 |
| Fat in the kidney | Fat and greaves C3 | 0.0003 | 0.0004 | 0.0000 |
| Feet (without hooves) | Gelatin C3 | 0.0114 | 0.0163 | 0.0000 |
| Floatation fat | Spreading/Compost | 0.0000 | 0.0000 | 0.0000 |
| Forehead | C1-C2 for disposal | 0.0000 | 0.0000 | 0.0000 |
| Forelock | PAP C3 | 0.0025 | 0.0010 | 0.0001 |
| Gallbladder | Pet food | 0.0003 | 0.0005 | 0.0000 |
| Head trimmings | Pet food | 0.0024 | 0.0034 | 0.0002 |
| Heart | Human food | 0.0028 | 0.0041 | 0.0009 |
| Heart trimmings | Pet food | 0.0003 | 0.0005 | 0.0000 |
| Hide | Skin tannery C3 | 0.0635 | 0.0638 | 0.1033 |
| Hooves | PAP C3 | 0.0072 | 0.0028 | 0.0002 |
| Horns | PAP C3 | 0.0019 | 0.0007 | 0.0001 |
| Kidney | Human food | 0.0018 | 0.0026 | 0.0010 |
| Large intestine | C1-C2 for disposal | 0.0000 | 0.0000 | 0.0000 |
| Liver | Human food | 0.0542 | 0.0165 | 0.0078 |
| Liver trimmings | Pet food | 0.0062 | 0.0018 | 0.0001 |
| Lower jaw | PAP C3 | 0.0028 | 0.0045 | 0.0004 |
| Lungs | Pet food | 0.0076 | 0.0092 | 0.0007 |
| Mask | Skin tannery C3 | 0.0044 | 0.0044 | 0.0071 |
| Mesenteric fat | C1-C2 for disposal | 0.0000 | 0.0000 | 0.0000 |
| Muscle | Human food | 0.3497 | 0.4885 | 0.7924 |
| Muzzle | Human food | 0.0023 | 0.0023 | 0.0022 |
| Omasum | Human food | 0.0132 | 0.0021 | 0.0015 |
| Omasum fat | Fat and greaves C3 | 0.0031 | 0.0013 | 0.0001 |
| Rumen and forestomach | Human food | 0.0675 | 0.0106 | 0.0077 |
| Rumen fat | Fat and greaves C3 | 0.0047 | 0.0021 | 0.0002 |
| Sanitary seizures | C1-C2 for disposal | 0.0000 | 0.0000 | 0.0000 |
| Screening and sifting wastes | C1-C2 for disposal | 0.0000 | 0.0000 | 0.0000 |
| Small intestine | PAP C3 | 0.0783 | 0.0123 | 0.0007 |
| Spinal cord | C1-C2 for disposal | 0.0000 | 0.0000 | 0.0000 |
| Spinal cord waste | C1-C2 for disposal | 0.0000 | 0.0000 | 0.0000 |
| Spine | C1-C2 for disposal | 0.0000 | 0.0000 | 0.0000 |
| Spleen | Pet food | 0.0018 | 0.0026 | 0.0002 |
| Stillborn | PAP C3 | 0.0413 | 0.0062 | 0.0000 |
| Tallow | Fat and greaves C3 | 0.0569 | 0.0632 | 0.0056 |
| Tongue | Human food | 0.0022 | 0.0036 | 0.0056 |
| Tonsil | C1-C2 for disposal | 0.0000 | 0.0000 | 0.0000 |
| Trachea | Pet food | 0.0016 | 0.0016 | 0.0001 |
| Udder | Pet food | 0.0028 | 0.0193 | 0.0014 |
| Upper throat | Pet food | 0.0009 | 0.0013 | 0.0001 |
| Water in the rumen | Spreading/Compost | 0.0000 | 0.0000 | 0.0000 |

Table 53: Total weighting by coproducts for Blonde d’Aquitaine Young Bulls reared in Pasture

| COPRODUCT | Destination | Blonde d’Aquitaine/young bull/pasture | | |
| --- | --- | --- | --- | --- |
|  |  | **Biophysical Cumulative share** | **Mass Cumulative share** | **Economic Cumulative share** |
| Abomasum | Human food | 0.0120 | 0.0020 | 0.0015 |
| Abomasum fat | Fat and greaves C3 | 0.0008 | 0.0004 | 0.0000 |
| Aponeurosis | Human food | 0.0301 | 0.0378 | 0.0362 |
| Bile | PAP C3 | 0.0000 | 0.0001 | 0.0000 |
| Blood | PAP C3 | 0.0152 | 0.0247 | 0.0053 |
| Blood | Pet food | 0.0021 | 0.0035 | 0.0002 |
| Bones | Gelatin C3 | 0.0575 | 0.0848 | 0.0002 |
| Bones of head, brain, eyes and teeth | C1-C2 for disposal | 0.0000 | 0.0000 | 0.0000 |
| Cheek | Human food | 0.0009 | 0.0012 | 0.0025 |
| Cheek | Human food | 0.0016 | 0.0023 | 0.0048 |
| Cheek trimmings | Pet food | 0.0004 | 0.0006 | 0.0000 |
| Chops | Pet food | 0.0017 | 0.0017 | 0.0001 |
| Contents of intestines | Spreading/Compost | 0.0000 | 0.0000 | 0.0000 |
| Contents of the rumen | Spreading/Compost | 0.0000 | 0.0000 | 0.0000 |
| Ears | PAP C3 | 0.0008 | 0.0008 | 0.0001 |
| Esophagus | Pet food | 0.0008 | 0.0012 | 0.0001 |
| Fat | Fat and greaves C3 | 0.0995 | 0.0908 | 0.0079 |
| Fat around heart | Fat and greaves C3 | 0.0025 | 0.0023 | 0.0002 |
| Fat in the kidney | Fat and greaves C3 | 0.0004 | 0.0004 | 0.0000 |
| Feet (without hooves) | Gelatin C3 | 0.0106 | 0.0149 | 0.0000 |
| Floatation fat | Spreading/Compost | 0.0000 | 0.0000 | 0.0000 |
| Forehead | C1-C2 for disposal | 0.0000 | 0.0000 | 0.0000 |
| Forelock | PAP C3 | 0.0025 | 0.0010 | 0.0001 |
| Gallbladder | Pet food | 0.0003 | 0.0004 | 0.0000 |
| Head trimmings | Pet food | 0.0023 | 0.0031 | 0.0002 |
| Heart | Human food | 0.0026 | 0.0037 | 0.0008 |
| Heart trimmings | Pet food | 0.0003 | 0.0004 | 0.0000 |
| Hide | Skin tannery C3 | 0.0587 | 0.0584 | 0.0929 |
| Hooves | PAP C3 | 0.0068 | 0.0026 | 0.0002 |
| Horns | PAP C3 | 0.0016 | 0.0006 | 0.0000 |
| Kidney | Human food | 0.0016 | 0.0023 | 0.0009 |
| Large intestine | C1-C2 for disposal | 0.0000 | 0.0000 | 0.0000 |
| Liver | Human food | 0.0463 | 0.0151 | 0.0070 |
| Liver trimmings | Pet food | 0.0054 | 0.0017 | 0.0001 |
| Lower jaw | PAP C3 | 0.0028 | 0.0042 | 0.0003 |
| Lungs | Pet food | 0.0070 | 0.0084 | 0.0006 |
| Mask | Skin tannery C3 | 0.0040 | 0.0040 | 0.0063 |
| Mesenteric fat | C1-C2 for disposal | 0.0000 | 0.0000 | 0.0000 |
| Muscle | Human food | 0.3696 | 0.5071 | 0.8083 |
| Muzzle | Human food | 0.0021 | 0.0020 | 0.0020 |
| Omasum | Human food | 0.0113 | 0.0019 | 0.0014 |
| Omasum fat | Fat and greaves C3 | 0.0028 | 0.0012 | 0.0001 |
| Rumen and forestomach | Human food | 0.0573 | 0.0097 | 0.0070 |
| Rumen fat | Fat and greaves C3 | 0.0045 | 0.0019 | 0.0002 |
| Sanitary seizures | C1-C2 for disposal | 0.0000 | 0.0000 | 0.0000 |
| Screening and sifting wastes | C1-C2 for disposal | 0.0000 | 0.0000 | 0.0000 |
| Small intestine | PAP C3 | 0.0665 | 0.0113 | 0.0007 |
| Spinal cord | C1-C2 for disposal | 0.0000 | 0.0000 | 0.0000 |
| Spinal cord waste | C1-C2 for disposal | 0.0000 | 0.0000 | 0.0000 |
| Spine | C1-C2 for disposal | 0.0000 | 0.0000 | 0.0000 |
| Spleen | Pet food | 0.0017 | 0.0023 | 0.0002 |
| Stillborn | PAP C3 | 0.0347 | 0.0056 | 0.0000 |
| Tallow | Fat and greaves C3 | 0.0632 | 0.0578 | 0.0050 |
| Tongue | Human food | 0.0022 | 0.0034 | 0.0051 |
| Tonsil | C1-C2 for disposal | 0.0000 | 0.0000 | 0.0000 |
| Trachea | Pet food | 0.0015 | 0.0014 | 0.0001 |
| Udder | Pet food | 0.0028 | 0.0177 | 0.0012 |
| Upper throat | Pet food | 0.0009 | 0.0012 | 0.0001 |
| Water in the rumen | Spreading/Compost | 0.0000 | 0.0000 | 0.0000 |

Table 54: Total weighting by coproducts for Blonde d’Aquitaine Heifers reared in Pasture

| COPRODUCT | Destination | Blonde d’Aquitaine/heifer/pasture | | |
| --- | --- | --- | --- | --- |
|  |  | **Biophysical Cumulative share** | **Mass Cumulative share** | **Economic Cumulative share** |
| Abomasum | Human food | 0.0135 | 0.0023 | 0.0017 |
| Abomasum fat | Fat and greaves C3 | 0.0011 | 0.0005 | 0.0000 |
| Aponeurosis | Human food | 0.0278 | 0.0360 | 0.0353 |
| Bile | PAP C3 | 0.0000 | 0.0001 | 0.0000 |
| Blood | PAP C3 | 0.0166 | 0.0278 | 0.0061 |
| Blood | Pet food | 0.0024 | 0.0040 | 0.0003 |
| Bones | Gelatin C3 | 0.0529 | 0.0807 | 0.0002 |
| Bones of head, brain, eyes and teeth | C1-C2 for disposal | 0.0000 | 0.0000 | 0.0000 |
| Cheek | Human food | 0.0010 | 0.0015 | 0.0031 |
| Cheek | Human food | 0.0018 | 0.0026 | 0.0055 |
| Cheek trimmings | Pet food | 0.0005 | 0.0007 | 0.0001 |
| Chops | Pet food | 0.0018 | 0.0018 | 0.0001 |
| Contents of intestines | Spreading/Compost | 0.0000 | 0.0000 | 0.0000 |
| Contents of the rumen | Spreading/Compost | 0.0000 | 0.0000 | 0.0000 |
| Ears | PAP C3 | 0.0009 | 0.0010 | 0.0001 |
| Esophagus | Pet food | 0.0009 | 0.0013 | 0.0001 |
| Fat | Fat and greaves C3 | 0.0900 | 0.0864 | 0.0077 |
| Fat around heart | Fat and greaves C3 | 0.0027 | 0.0026 | 0.0002 |
| Fat in the kidney | Fat and greaves C3 | 0.0004 | 0.0004 | 0.0000 |
| Feet (without hooves) | Gelatin C3 | 0.0116 | 0.0167 | 0.0000 |
| Floatation fat | Spreading/Compost | 0.0000 | 0.0000 | 0.0000 |
| Forehead | C1-C2 for disposal | 0.0000 | 0.0000 | 0.0000 |
| Forelock | PAP C3 | 0.0028 | 0.0011 | 0.0001 |
| Gallbladder | Pet food | 0.0003 | 0.0005 | 0.0000 |
| Head trimmings | Pet food | 0.0025 | 0.0035 | 0.0003 |
| Heart | Human food | 0.0029 | 0.0043 | 0.0009 |
| Heart trimmings | Pet food | 0.0003 | 0.0005 | 0.0000 |
| Hide | Skin tannery C3 | 0.0643 | 0.0656 | 0.1069 |
| Hooves | PAP C3 | 0.0074 | 0.0029 | 0.0002 |
| Horns | PAP C3 | 0.0018 | 0.0007 | 0.0001 |
| Kidney | Human food | 0.0018 | 0.0026 | 0.0010 |
| Large intestine | C1-C2 for disposal | 0.0000 | 0.0000 | 0.0000 |
| Liver | Human food | 0.0513 | 0.0170 | 0.0080 |
| Liver trimmings | Pet food | 0.0058 | 0.0018 | 0.0001 |
| Lower jaw | PAP C3 | 0.0030 | 0.0046 | 0.0004 |
| Lungs | Pet food | 0.0077 | 0.0095 | 0.0007 |
| Mask | Skin tannery C3 | 0.0043 | 0.0044 | 0.0072 |
| Mesenteric fat | C1-C2 for disposal | 0.0000 | 0.0000 | 0.0000 |
| Muscle | Human food | 0.3419 | 0.4824 | 0.7871 |
| Muzzle | Human food | 0.0023 | 0.0023 | 0.0023 |
| Omasum | Human food | 0.0121 | 0.0021 | 0.0015 |
| Omasum fat | Fat and greaves C3 | 0.0033 | 0.0015 | 0.0001 |
| Rumen and forestomach | Human food | 0.0634 | 0.0109 | 0.0079 |
| Rumen fat | Fat and greaves C3 | 0.0047 | 0.0021 | 0.0002 |
| Sanitary seizures | C1-C2 for disposal | 0.0000 | 0.0000 | 0.0000 |
| Screening and sifting wastes | C1-C2 for disposal | 0.0000 | 0.0000 | 0.0000 |
| Small intestine | PAP C3 | 0.0740 | 0.0127 | 0.0008 |
| Spinal cord | C1-C2 for disposal | 0.0000 | 0.0000 | 0.0000 |
| Spinal cord waste | C1-C2 for disposal | 0.0000 | 0.0000 | 0.0000 |
| Spine | C1-C2 for disposal | 0.0000 | 0.0000 | 0.0000 |
| Spleen | Pet food | 0.0018 | 0.0026 | 0.0002 |
| Stillborn | PAP C3 | 0.0386 | 0.0063 | 0.0000 |
| Tallow | Fat and greaves C3 | 0.0677 | 0.0650 | 0.0058 |
| Tongue | Human food | 0.0024 | 0.0038 | 0.0059 |
| Tonsil | C1-C2 for disposal | 0.0000 | 0.0000 | 0.0000 |
| Trachea | Pet food | 0.0016 | 0.0016 | 0.0001 |
| Udder | Pet food | 0.0030 | 0.0199 | 0.0014 |
| Upper throat | Pet food | 0.0010 | 0.0015 | 0.0001 |
| Water in the rumen | Spreading/Compost | 0.0000 | 0.0000 | 0.0000 |

Table 55: Total weighting by coproducts for Blonde d’Aquitaine Cull Cows reared in Pasture

| COPRODUCT | Destination | Blonde d’Aquitaine/Cull cow/pasture | | |
| --- | --- | --- | --- | --- |
|  |  | **Biophysical Cumulative share** | **Mass Cumulative share** | **Economic Cumulative share** |
| Abomasum | Human food | 0.0170 | 0.0028 | 0.0021 |
| Abomasum fat | Fat and greaves C3 | 0.0011 | 0.0005 | 0.0000 |
| Aponeurosis | Human food | 0.0242 | 0.0326 | 0.0334 |
| Bile | PAP C3 | 0.0000 | 0.0001 | 0.0000 |
| Blood | PAP C3 | 0.0191 | 0.0335 | 0.0076 |
| Blood | Pet food | 0.0027 | 0.0048 | 0.0004 |
| Bones | Gelatin C3 | 0.0439 | 0.0732 | 0.0002 |
| Bones of head, brain, eyes and teeth | C1-C2 for disposal | 0.0000 | 0.0000 | 0.0000 |
| Cheek | Human food | 0.0011 | 0.0016 | 0.0037 |
| Cheek | Human food | 0.0021 | 0.0031 | 0.0070 |
| Cheek trimmings | Pet food | 0.0006 | 0.0009 | 0.0001 |
| Chops | Pet food | 0.0021 | 0.0023 | 0.0002 |
| Contents of intestines | Spreading/Compost | 0.0000 | 0.0000 | 0.0000 |
| Contents of the rumen | Spreading/Compost | 0.0000 | 0.0000 | 0.0000 |
| Ears | PAP C3 | 0.0009 | 0.0011 | 0.0001 |
| Esophagus | Pet food | 0.0010 | 0.0015 | 0.0001 |
| Fat | Fat and greaves C3 | 0.0634 | 0.0783 | 0.0073 |
| Fat around heart | Fat and greaves C3 | 0.0025 | 0.0031 | 0.0003 |
| Fat in the kidney | Fat and greaves C3 | 0.0003 | 0.0004 | 0.0000 |
| Feet (without hooves) | Gelatin C3 | 0.0133 | 0.0202 | 0.0001 |
| Floatation fat | Spreading/Compost | 0.0000 | 0.0000 | 0.0000 |
| Forehead | C1-C2 for disposal | 0.0000 | 0.0000 | 0.0000 |
| Forelock | PAP C3 | 0.0030 | 0.0013 | 0.0001 |
| Gallbladder | Pet food | 0.0003 | 0.0005 | 0.0000 |
| Head trimmings | Pet food | 0.0029 | 0.0043 | 0.0003 |
| Heart | Human food | 0.0032 | 0.0050 | 0.0011 |
| Heart trimmings | Pet food | 0.0003 | 0.0005 | 0.0000 |
| Hide | Skin tannery C3 | 0.0741 | 0.0791 | 0.1346 |
| Hooves | PAP C3 | 0.0085 | 0.0035 | 0.0003 |
| Horns | PAP C3 | 0.0021 | 0.0009 | 0.0001 |
| Kidney | Human food | 0.0021 | 0.0031 | 0.0013 |
| Large intestine | C1-C2 for disposal | 0.0000 | 0.0000 | 0.0000 |
| Liver | Human food | 0.0645 | 0.0205 | 0.0101 |
| Liver trimmings | Pet food | 0.0074 | 0.0023 | 0.0002 |
| Lower jaw | PAP C3 | 0.0033 | 0.0056 | 0.0005 |
| Lungs | Pet food | 0.0089 | 0.0114 | 0.0009 |
| Mask | Skin tannery C3 | 0.0051 | 0.0054 | 0.0092 |
| Mesenteric fat | C1-C2 for disposal | 0.0000 | 0.0000 | 0.0000 |
| Muscle | Human food | 0.2941 | 0.4374 | 0.7457 |
| Muzzle | Human food | 0.0026 | 0.0028 | 0.0028 |
| Omasum | Human food | 0.0155 | 0.0025 | 0.0019 |
| Omasum fat | Fat and greaves C3 | 0.0035 | 0.0016 | 0.0002 |
| Rumen and forestomach | Human food | 0.0805 | 0.0131 | 0.0100 |
| Rumen fat | Fat and greaves C3 | 0.0054 | 0.0025 | 0.0002 |
| Sanitary seizures | C1-C2 for disposal | 0.0000 | 0.0000 | 0.0000 |
| Screening and sifting wastes | C1-C2 for disposal | 0.0000 | 0.0000 | 0.0000 |
| Small intestine | PAP C3 | 0.0936 | 0.0152 | 0.0009 |
| Spinal cord | C1-C2 for disposal | 0.0000 | 0.0000 | 0.0000 |
| Spinal cord waste | C1-C2 for disposal | 0.0000 | 0.0000 | 0.0000 |
| Spine | C1-C2 for disposal | 0.0000 | 0.0000 | 0.0000 |
| Spleen | Pet food | 0.0021 | 0.0031 | 0.0002 |
| Stillborn | PAP C3 | 0.0493 | 0.0077 | 0.0000 |
| Tallow | Fat and greaves C3 | 0.0634 | 0.0785 | 0.0073 |
| Tongue | Human food | 0.0026 | 0.0045 | 0.0073 |
| Tonsil | C1-C2 for disposal | 0.0000 | 0.0000 | 0.0000 |
| Trachea | Pet food | 0.0019 | 0.0020 | 0.0002 |
| Udder | Pet food | 0.0032 | 0.0240 | 0.0018 |
| Upper throat | Pet food | 0.0011 | 0.0016 | 0.0001 |
| Water in the rumen | Spreading/Compost | 0.0000 | 0.0000 | 0.0000 |

Table 56: Total weighting by coproducts for Blonde d’Aquitaine Beef reared in Pasture

| COPRODUCT | Destination | Blonde d’Aquitaine/beef/pasture | | |
| --- | --- | --- | --- | --- |
|  |  | **Biophysical Cumulative share** | **Mass Cumulative share** | **Economic Cumulative share** |
| Abomasum | Human food | 0.0142 | 0.0023 | 0.0017 |
| Abomasum fat | Fat and greaves C3 | 0.0011 | 0.0005 | 0.0000 |
| Aponeurosis | Human food | 0.0284 | 0.0365 | 0.0355 |
| Bile | PAP C3 | 0.0000 | 0.0001 | 0.0000 |
| Blood | PAP C3 | 0.0162 | 0.0271 | 0.0059 |
| Blood | Pet food | 0.0023 | 0.0039 | 0.0003 |
| Bones | Gelatin C3 | 0.0532 | 0.0817 | 0.0002 |
| Bones of head, brain, eyes and teeth | C1-C2 for disposal | 0.0000 | 0.0000 | 0.0000 |
| Cheek | Human food | 0.0009 | 0.0013 | 0.0029 |
| Cheek | Human food | 0.0018 | 0.0026 | 0.0054 |
| Cheek trimmings | Pet food | 0.0005 | 0.0007 | 0.0001 |
| Chops | Pet food | 0.0018 | 0.0018 | 0.0001 |
| Contents of intestines | Spreading/Compost | 0.0000 | 0.0000 | 0.0000 |
| Contents of the rumen | Spreading/Compost | 0.0000 | 0.0000 | 0.0000 |
| Ears | PAP C3 | 0.0007 | 0.0009 | 0.0001 |
| Esophagus | Pet food | 0.0008 | 0.0012 | 0.0001 |
| Fat | Fat and greaves C3 | 0.0870 | 0.0875 | 0.0077 |
| Fat around heart | Fat and greaves C3 | 0.0025 | 0.0026 | 0.0002 |
| Fat in the kidney | Fat and greaves C3 | 0.0004 | 0.0004 | 0.0000 |
| Feet (without hooves) | Gelatin C3 | 0.0113 | 0.0163 | 0.0000 |
| Floatation fat | Spreading/Compost | 0.0000 | 0.0000 | 0.0000 |
| Forehead | C1-C2 for disposal | 0.0000 | 0.0000 | 0.0000 |
| Forelock | PAP C3 | 0.0025 | 0.0010 | 0.0001 |
| Gallbladder | Pet food | 0.0003 | 0.0005 | 0.0000 |
| Head trimmings | Pet food | 0.0024 | 0.0034 | 0.0002 |
| Heart | Human food | 0.0028 | 0.0041 | 0.0009 |
| Heart trimmings | Pet food | 0.0003 | 0.0005 | 0.0000 |
| Hide | Skin tannery C3 | 0.0628 | 0.0638 | 0.1033 |
| Hooves | PAP C3 | 0.0071 | 0.0028 | 0.0002 |
| Horns | PAP C3 | 0.0018 | 0.0007 | 0.0001 |
| Kidney | Human food | 0.0018 | 0.0026 | 0.0010 |
| Large intestine | C1-C2 for disposal | 0.0000 | 0.0000 | 0.0000 |
| Liver | Human food | 0.0522 | 0.0165 | 0.0078 |
| Liver trimmings | Pet food | 0.0060 | 0.0018 | 0.0001 |
| Lower jaw | PAP C3 | 0.0029 | 0.0045 | 0.0004 |
| Lungs | Pet food | 0.0075 | 0.0092 | 0.0007 |
| Mask | Skin tannery C3 | 0.0043 | 0.0044 | 0.0071 |
| Mesenteric fat | C1-C2 for disposal | 0.0000 | 0.0000 | 0.0000 |
| Muscle | Human food | 0.3472 | 0.4885 | 0.7924 |
| Muzzle | Human food | 0.0023 | 0.0023 | 0.0022 |
| Omasum | Human food | 0.0127 | 0.0021 | 0.0015 |
| Omasum fat | Fat and greaves C3 | 0.0031 | 0.0013 | 0.0001 |
| Rumen and forestomach | Human food | 0.0648 | 0.0106 | 0.0077 |
| Rumen fat | Fat and greaves C3 | 0.0048 | 0.0021 | 0.0002 |
| Sanitary seizures | C1-C2 for disposal | 0.0000 | 0.0000 | 0.0000 |
| Screening and sifting wastes | C1-C2 for disposal | 0.0000 | 0.0000 | 0.0000 |
| Small intestine | PAP C3 | 0.0752 | 0.0123 | 0.0007 |
| Spinal cord | C1-C2 for disposal | 0.0000 | 0.0000 | 0.0000 |
| Spinal cord waste | C1-C2 for disposal | 0.0000 | 0.0000 | 0.0000 |
| Spine | C1-C2 for disposal | 0.0000 | 0.0000 | 0.0000 |
| Spleen | Pet food | 0.0018 | 0.0026 | 0.0002 |
| Stillborn | PAP C3 | 0.0397 | 0.0062 | 0.0000 |
| Tallow | Fat and greaves C3 | 0.0627 | 0.0632 | 0.0056 |
| Tongue | Human food | 0.0023 | 0.0036 | 0.0056 |
| Tonsil | C1-C2 for disposal | 0.0000 | 0.0000 | 0.0000 |
| Trachea | Pet food | 0.0016 | 0.0016 | 0.0001 |
| Udder | Pet food | 0.0029 | 0.0193 | 0.0014 |
| Upper throat | Pet food | 0.0009 | 0.0013 | 0.0001 |
| Water in the rumen | Spreading/Compost | 0.0000 | 0.0000 | 0.0000 |

Table 57: Total weighting by coproducts for Blonde d’Aquitaine Young Bulls reared in Stall

| COPRODUCT | Destination | Blonde d’Aquitaine/young bull/stall | | |
| --- | --- | --- | --- | --- |
|  |  | **Biophysical Cumulative share** | **Mass Cumulative share** | **Economic Cumulative share** |
| Abomasum | Human food | 0.0114 | 0.0020 | 0.0015 |
| Abomasum fat | Fat and greaves C3 | 0.0008 | 0.0004 | 0.0000 |
| Aponeurosis | Human food | 0.0297 | 0.0378 | 0.0362 |
| Bile | PAP C3 | 0.0000 | 0.0001 | 0.0000 |
| Blood | PAP C3 | 0.0150 | 0.0247 | 0.0053 |
| Blood | Pet food | 0.0021 | 0.0035 | 0.0002 |
| Bones | Gelatin C3 | 0.0583 | 0.0848 | 0.0002 |
| Bones of head, brain, eyes and teeth | C1-C2 for disposal | 0.0000 | 0.0000 | 0.0000 |
| Cheek | Human food | 0.0009 | 0.0012 | 0.0025 |
| Cheek | Human food | 0.0016 | 0.0023 | 0.0048 |
| Cheek trimmings | Pet food | 0.0004 | 0.0006 | 0.0000 |
| Chops | Pet food | 0.0017 | 0.0017 | 0.0001 |
| Contents of intestines | Spreading/Compost | 0.0000 | 0.0000 | 0.0000 |
| Contents of the rumen | Spreading/Compost | 0.0000 | 0.0000 | 0.0000 |
| Ears | PAP C3 | 0.0008 | 0.0008 | 0.0001 |
| Esophagus | Pet food | 0.0008 | 0.0012 | 0.0001 |
| Fat | Fat and greaves C3 | 0.1089 | 0.0908 | 0.0079 |
| Fat around heart | Fat and greaves C3 | 0.0027 | 0.0023 | 0.0002 |
| Fat in the kidney | Fat and greaves C3 | 0.0004 | 0.0004 | 0.0000 |
| Feet (without hooves) | Gelatin C3 | 0.0106 | 0.0149 | 0.0000 |
| Floatation fat | Spreading/Compost | 0.0000 | 0.0000 | 0.0000 |
| Forehead | C1-C2 for disposal | 0.0000 | 0.0000 | 0.0000 |
| Forelock | PAP C3 | 0.0024 | 0.0010 | 0.0001 |
| Gallbladder | Pet food | 0.0003 | 0.0004 | 0.0000 |
| Head trimmings | Pet food | 0.0022 | 0.0031 | 0.0002 |
| Heart | Human food | 0.0025 | 0.0037 | 0.0008 |
| Heart trimmings | Pet food | 0.0003 | 0.0004 | 0.0000 |
| Hide | Skin tannery C3 | 0.0580 | 0.0584 | 0.0929 |
| Hooves | PAP C3 | 0.0067 | 0.0026 | 0.0002 |
| Horns | PAP C3 | 0.0015 | 0.0006 | 0.0000 |
| Kidney | Human food | 0.0016 | 0.0023 | 0.0009 |
| Large intestine | C1-C2 for disposal | 0.0000 | 0.0000 | 0.0000 |
| Liver | Human food | 0.0442 | 0.0151 | 0.0070 |
| Liver trimmings | Pet food | 0.0051 | 0.0017 | 0.0001 |
| Lower jaw | PAP C3 | 0.0028 | 0.0042 | 0.0003 |
| Lungs | Pet food | 0.0069 | 0.0084 | 0.0006 |
| Mask | Skin tannery C3 | 0.0039 | 0.0040 | 0.0063 |
| Mesenteric fat | C1-C2 for disposal | 0.0000 | 0.0000 | 0.0000 |
| Muscle | Human food | 0.3669 | 0.5071 | 0.8083 |
| Muzzle | Human food | 0.0020 | 0.0020 | 0.0020 |
| Omasum | Human food | 0.0107 | 0.0019 | 0.0014 |
| Omasum fat | Fat and greaves C3 | 0.0028 | 0.0012 | 0.0001 |
| Rumen and forestomach | Human food | 0.0543 | 0.0097 | 0.0070 |
| Rumen fat | Fat and greaves C3 | 0.0045 | 0.0019 | 0.0002 |
| Sanitary seizures | C1-C2 for disposal | 0.0000 | 0.0000 | 0.0000 |
| Screening and sifting wastes | C1-C2 for disposal | 0.0000 | 0.0000 | 0.0000 |
| Small intestine | PAP C3 | 0.0631 | 0.0113 | 0.0007 |
| Spinal cord | C1-C2 for disposal | 0.0000 | 0.0000 | 0.0000 |
| Spinal cord waste | C1-C2 for disposal | 0.0000 | 0.0000 | 0.0000 |
| Spine | C1-C2 for disposal | 0.0000 | 0.0000 | 0.0000 |
| Spleen | Pet food | 0.0017 | 0.0023 | 0.0002 |
| Stillborn | PAP C3 | 0.0329 | 0.0056 | 0.0000 |
| Tallow | Fat and greaves C3 | 0.0692 | 0.0578 | 0.0050 |
| Tongue | Human food | 0.0022 | 0.0034 | 0.0051 |
| Tonsil | C1-C2 for disposal | 0.0000 | 0.0000 | 0.0000 |
| Trachea | Pet food | 0.0014 | 0.0014 | 0.0001 |
| Udder | Pet food | 0.0028 | 0.0177 | 0.0012 |
| Upper throat | Pet food | 0.0009 | 0.0012 | 0.0001 |
| Water in the rumen | Spreading/Compost | 0.0000 | 0.0000 | 0.0000 |

Table 58: Total weighting by coproducts for Blonde d’Aquitaine Heifers reared in Stall

| COPRODUCT | Destination | Blonde d’Aquitaine/heifer/stall | | |
| --- | --- | --- | --- | --- |
|  |  | **Biophysical Cumulative share** | **Mass Cumulative share** | **Economic Cumulative share** |
| Abomasum | Human food | 0.0129 | 0.0023 | 0.0017 |
| Abomasum fat | Fat and greaves C3 | 0.0011 | 0.0005 | 0.0000 |
| Aponeurosis | Human food | 0.0275 | 0.0360 | 0.0353 |
| Bile | PAP C3 | 0.0000 | 0.0001 | 0.0000 |
| Blood | PAP C3 | 0.0164 | 0.0278 | 0.0061 |
| Blood | Pet food | 0.0024 | 0.0040 | 0.0003 |
| Bones | Gelatin C3 | 0.0537 | 0.0807 | 0.0002 |
| Bones of head, brain, eyes and teeth | C1-C2 for disposal | 0.0000 | 0.0000 | 0.0000 |
| Cheek | Human food | 0.0010 | 0.0015 | 0.0031 |
| Cheek | Human food | 0.0018 | 0.0026 | 0.0055 |
| Cheek trimmings | Pet food | 0.0005 | 0.0007 | 0.0001 |
| Chops | Pet food | 0.0018 | 0.0018 | 0.0001 |
| Contents of intestines | Spreading/Compost | 0.0000 | 0.0000 | 0.0000 |
| Contents of the rumen | Spreading/Compost | 0.0000 | 0.0000 | 0.0000 |
| Ears | PAP C3 | 0.0009 | 0.0010 | 0.0001 |
| Esophagus | Pet food | 0.0009 | 0.0013 | 0.0001 |
| Fat | Fat and greaves C3 | 0.0988 | 0.0864 | 0.0077 |
| Fat around heart | Fat and greaves C3 | 0.0029 | 0.0026 | 0.0002 |
| Fat in the kidney | Fat and greaves C3 | 0.0004 | 0.0004 | 0.0000 |
| Feet (without hooves) | Gelatin C3 | 0.0115 | 0.0167 | 0.0000 |
| Floatation fat | Spreading/Compost | 0.0000 | 0.0000 | 0.0000 |
| Forehead | C1-C2 for disposal | 0.0000 | 0.0000 | 0.0000 |
| Forelock | PAP C3 | 0.0027 | 0.0011 | 0.0001 |
| Gallbladder | Pet food | 0.0003 | 0.0005 | 0.0000 |
| Head trimmings | Pet food | 0.0025 | 0.0035 | 0.0003 |
| Heart | Human food | 0.0028 | 0.0043 | 0.0009 |
| Heart trimmings | Pet food | 0.0003 | 0.0005 | 0.0000 |
| Hide | Skin tannery C3 | 0.0635 | 0.0656 | 0.1069 |
| Hooves | PAP C3 | 0.0073 | 0.0029 | 0.0002 |
| Horns | PAP C3 | 0.0018 | 0.0007 | 0.0001 |
| Kidney | Human food | 0.0018 | 0.0026 | 0.0010 |
| Large intestine | C1-C2 for disposal | 0.0000 | 0.0000 | 0.0000 |
| Liver | Human food | 0.0491 | 0.0170 | 0.0080 |
| Liver trimmings | Pet food | 0.0055 | 0.0018 | 0.0001 |
| Lower jaw | PAP C3 | 0.0030 | 0.0046 | 0.0004 |
| Lungs | Pet food | 0.0076 | 0.0095 | 0.0007 |
| Mask | Skin tannery C3 | 0.0042 | 0.0044 | 0.0072 |
| Mesenteric fat | C1-C2 for disposal | 0.0000 | 0.0000 | 0.0000 |
| Muscle | Human food | 0.3396 | 0.4824 | 0.7871 |
| Muzzle | Human food | 0.0022 | 0.0023 | 0.0023 |
| Omasum | Human food | 0.0115 | 0.0021 | 0.0015 |
| Omasum fat | Fat and greaves C3 | 0.0034 | 0.0015 | 0.0001 |
| Rumen and forestomach | Human food | 0.0602 | 0.0109 | 0.0079 |
| Rumen fat | Fat and greaves C3 | 0.0048 | 0.0021 | 0.0002 |
| Sanitary seizures | C1-C2 for disposal | 0.0000 | 0.0000 | 0.0000 |
| Screening and sifting wastes | C1-C2 for disposal | 0.0000 | 0.0000 | 0.0000 |
| Small intestine | PAP C3 | 0.0704 | 0.0127 | 0.0008 |
| Spinal cord | C1-C2 for disposal | 0.0000 | 0.0000 | 0.0000 |
| Spinal cord waste | C1-C2 for disposal | 0.0000 | 0.0000 | 0.0000 |
| Spine | C1-C2 for disposal | 0.0000 | 0.0000 | 0.0000 |
| Spleen | Pet food | 0.0018 | 0.0026 | 0.0002 |
| Stillborn | PAP C3 | 0.0367 | 0.0063 | 0.0000 |
| Tallow | Fat and greaves C3 | 0.0743 | 0.0650 | 0.0058 |
| Tongue | Human food | 0.0024 | 0.0038 | 0.0059 |
| Tonsil | C1-C2 for disposal | 0.0000 | 0.0000 | 0.0000 |
| Trachea | Pet food | 0.0015 | 0.0016 | 0.0001 |
| Udder | Pet food | 0.0031 | 0.0199 | 0.0014 |
| Upper throat | Pet food | 0.0010 | 0.0015 | 0.0001 |
| Water in the rumen | Spreading/Compost | 0.0000 | 0.0000 | 0.0000 |

Table 59: Total weighting by coproducts for Blonde d’Aquitaine Cull Cows reared in Stall

| COPRODUCT | Destination | Blonde d’Aquitaine/Cull cow/stall | | |
| --- | --- | --- | --- | --- |
|  |  | **Biophysical Cumulative share** | **Mass Cumulative share** | **Economic Cumulative share** |
| Abomasum | Human food | 0.0164 | 0.0028 | 0.0021 |
| Abomasum fat | Fat and greaves C3 | 0.0011 | 0.0005 | 0.0000 |
| Aponeurosis | Human food | 0.0239 | 0.0326 | 0.0334 |
| Bile | PAP C3 | 0.0000 | 0.0001 | 0.0000 |
| Blood | PAP C3 | 0.0189 | 0.0335 | 0.0076 |
| Blood | Pet food | 0.0027 | 0.0048 | 0.0004 |
| Bones | Gelatin C3 | 0.0445 | 0.0732 | 0.0002 |
| Bones of head, brain, eyes and teeth | C1-C2 for disposal | 0.0000 | 0.0000 | 0.0000 |
| Cheek | Human food | 0.0011 | 0.0016 | 0.0037 |
| Cheek | Human food | 0.0021 | 0.0031 | 0.0070 |
| Cheek trimmings | Pet food | 0.0006 | 0.0009 | 0.0001 |
| Chops | Pet food | 0.0021 | 0.0023 | 0.0002 |
| Contents of intestines | Spreading/Compost | 0.0000 | 0.0000 | 0.0000 |
| Contents of the rumen | Spreading/Compost | 0.0000 | 0.0000 | 0.0000 |
| Ears | PAP C3 | 0.0009 | 0.0011 | 0.0001 |
| Esophagus | Pet food | 0.0010 | 0.0015 | 0.0001 |
| Fat | Fat and greaves C3 | 0.0705 | 0.0783 | 0.0073 |
| Fat around heart | Fat and greaves C3 | 0.0028 | 0.0031 | 0.0003 |
| Fat in the kidney | Fat and greaves C3 | 0.0003 | 0.0004 | 0.0000 |
| Feet (without hooves) | Gelatin C3 | 0.0132 | 0.0202 | 0.0001 |
| Floatation fat | Spreading/Compost | 0.0000 | 0.0000 | 0.0000 |
| Forehead | C1-C2 for disposal | 0.0000 | 0.0000 | 0.0000 |
| Forelock | PAP C3 | 0.0030 | 0.0013 | 0.0001 |
| Gallbladder | Pet food | 0.0003 | 0.0005 | 0.0000 |
| Head trimmings | Pet food | 0.0028 | 0.0043 | 0.0003 |
| Heart | Human food | 0.0032 | 0.0050 | 0.0011 |
| Heart trimmings | Pet food | 0.0003 | 0.0005 | 0.0000 |
| Hide | Skin tannery C3 | 0.0733 | 0.0791 | 0.1346 |
| Hooves | PAP C3 | 0.0084 | 0.0035 | 0.0003 |
| Horns | PAP C3 | 0.0021 | 0.0009 | 0.0001 |
| Kidney | Human food | 0.0021 | 0.0031 | 0.0013 |
| Large intestine | C1-C2 for disposal | 0.0000 | 0.0000 | 0.0000 |
| Liver | Human food | 0.0624 | 0.0205 | 0.0101 |
| Liver trimmings | Pet food | 0.0072 | 0.0023 | 0.0002 |
| Lower jaw | PAP C3 | 0.0034 | 0.0056 | 0.0005 |
| Lungs | Pet food | 0.0088 | 0.0114 | 0.0009 |
| Mask | Skin tannery C3 | 0.0050 | 0.0054 | 0.0092 |
| Mesenteric fat | C1-C2 for disposal | 0.0000 | 0.0000 | 0.0000 |
| Muscle | Human food | 0.2924 | 0.4374 | 0.7457 |
| Muzzle | Human food | 0.0026 | 0.0028 | 0.0028 |
| Omasum | Human food | 0.0149 | 0.0025 | 0.0019 |
| Omasum fat | Fat and greaves C3 | 0.0036 | 0.0016 | 0.0002 |
| Rumen and forestomach | Human food | 0.0775 | 0.0131 | 0.0100 |
| Rumen fat | Fat and greaves C3 | 0.0055 | 0.0025 | 0.0002 |
| Sanitary seizures | C1-C2 for disposal | 0.0000 | 0.0000 | 0.0000 |
| Screening and sifting wastes | C1-C2 for disposal | 0.0000 | 0.0000 | 0.0000 |
| Small intestine | PAP C3 | 0.0902 | 0.0152 | 0.0009 |
| Spinal cord | C1-C2 for disposal | 0.0000 | 0.0000 | 0.0000 |
| Spinal cord waste | C1-C2 for disposal | 0.0000 | 0.0000 | 0.0000 |
| Spine | C1-C2 for disposal | 0.0000 | 0.0000 | 0.0000 |
| Spleen | Pet food | 0.0021 | 0.0031 | 0.0002 |
| Stillborn | PAP C3 | 0.0475 | 0.0077 | 0.0000 |
| Tallow | Fat and greaves C3 | 0.0705 | 0.0785 | 0.0073 |
| Tongue | Human food | 0.0026 | 0.0045 | 0.0073 |
| Tonsil | C1-C2 for disposal | 0.0000 | 0.0000 | 0.0000 |
| Trachea | Pet food | 0.0019 | 0.0020 | 0.0002 |
| Udder | Pet food | 0.0033 | 0.0240 | 0.0018 |
| Upper throat | Pet food | 0.0011 | 0.0016 | 0.0001 |
| Water in the rumen | Spreading/Compost | 0.0000 | 0.0000 | 0.0000 |

Table 60: Total weighting by coproducts for Blonde d’Aquitaine Beef reared in Stall

| COPRODUCT | Destination | Blonde d’Aquitaine/beef/stall | | |
| --- | --- | --- | --- | --- |
|  |  | **Biophysical Cumulative share** | **Mass Cumulative share** | **Economic Cumulative share** |
| Abomasum | Human food | 0.0135 | 0.0023 | 0.0017 |
| Abomasum fat | Fat and greaves C3 | 0.0011 | 0.0005 | 0.0000 |
| Aponeurosis | Human food | 0.0280 | 0.0365 | 0.0355 |
| Bile | PAP C3 | 0.0000 | 0.0001 | 0.0000 |
| Blood | PAP C3 | 0.0160 | 0.0271 | 0.0059 |
| Blood | Pet food | 0.0023 | 0.0039 | 0.0003 |
| Bones | Gelatin C3 | 0.0540 | 0.0817 | 0.0002 |
| Bones of head, brain, eyes and teeth | C1-C2 for disposal | 0.0000 | 0.0000 | 0.0000 |
| Cheek | Human food | 0.0009 | 0.0013 | 0.0029 |
| Cheek | Human food | 0.0018 | 0.0026 | 0.0054 |
| Cheek trimmings | Pet food | 0.0005 | 0.0007 | 0.0001 |
| Chops | Pet food | 0.0018 | 0.0018 | 0.0001 |
| Contents of intestines | Spreading/Compost | 0.0000 | 0.0000 | 0.0000 |
| Contents of the rumen | Spreading/Compost | 0.0000 | 0.0000 | 0.0000 |
| Ears | PAP C3 | 0.0007 | 0.0009 | 0.0001 |
| Esophagus | Pet food | 0.0008 | 0.0012 | 0.0001 |
| Fat | Fat and greaves C3 | 0.0959 | 0.0875 | 0.0077 |
| Fat around heart | Fat and greaves C3 | 0.0028 | 0.0026 | 0.0002 |
| Fat in the kidney | Fat and greaves C3 | 0.0004 | 0.0004 | 0.0000 |
| Feet (without hooves) | Gelatin C3 | 0.0112 | 0.0163 | 0.0000 |
| Floatation fat | Spreading/Compost | 0.0000 | 0.0000 | 0.0000 |
| Forehead | C1-C2 for disposal | 0.0000 | 0.0000 | 0.0000 |
| Forelock | PAP C3 | 0.0024 | 0.0010 | 0.0001 |
| Gallbladder | Pet food | 0.0003 | 0.0005 | 0.0000 |
| Head trimmings | Pet food | 0.0024 | 0.0034 | 0.0002 |
| Heart | Human food | 0.0028 | 0.0041 | 0.0009 |
| Heart trimmings | Pet food | 0.0003 | 0.0005 | 0.0000 |
| Hide | Skin tannery C3 | 0.0619 | 0.0638 | 0.1033 |
| Hooves | PAP C3 | 0.0070 | 0.0028 | 0.0002 |
| Horns | PAP C3 | 0.0018 | 0.0007 | 0.0001 |
| Kidney | Human food | 0.0018 | 0.0026 | 0.0010 |
| Large intestine | C1-C2 for disposal | 0.0000 | 0.0000 | 0.0000 |
| Liver | Human food | 0.0501 | 0.0165 | 0.0078 |
| Liver trimmings | Pet food | 0.0058 | 0.0018 | 0.0001 |
| Lower jaw | PAP C3 | 0.0029 | 0.0045 | 0.0004 |
| Lungs | Pet food | 0.0074 | 0.0092 | 0.0007 |
| Mask | Skin tannery C3 | 0.0042 | 0.0044 | 0.0071 |
| Mesenteric fat | C1-C2 for disposal | 0.0000 | 0.0000 | 0.0000 |
| Muscle | Human food | 0.3446 | 0.4885 | 0.7924 |
| Muzzle | Human food | 0.0022 | 0.0023 | 0.0022 |
| Omasum | Human food | 0.0121 | 0.0021 | 0.0015 |
| Omasum fat | Fat and greaves C3 | 0.0031 | 0.0013 | 0.0001 |
| Rumen and forestomach | Human food | 0.0619 | 0.0106 | 0.0077 |
| Rumen fat | Fat and greaves C3 | 0.0048 | 0.0021 | 0.0002 |
| Sanitary seizures | C1-C2 for disposal | 0.0000 | 0.0000 | 0.0000 |
| Screening and sifting wastes | C1-C2 for disposal | 0.0000 | 0.0000 | 0.0000 |
| Small intestine | PAP C3 | 0.0718 | 0.0123 | 0.0007 |
| Spinal cord | C1-C2 for disposal | 0.0000 | 0.0000 | 0.0000 |
| Spinal cord waste | C1-C2 for disposal | 0.0000 | 0.0000 | 0.0000 |
| Spine | C1-C2 for disposal | 0.0000 | 0.0000 | 0.0000 |
| Spleen | Pet food | 0.0018 | 0.0026 | 0.0002 |
| Stillborn | PAP C3 | 0.0378 | 0.0062 | 0.0000 |
| Tallow | Fat and greaves C3 | 0.0692 | 0.0632 | 0.0056 |
| Tongue | Human food | 0.0023 | 0.0036 | 0.0056 |
| Tonsil | C1-C2 for disposal | 0.0000 | 0.0000 | 0.0000 |
| Trachea | Pet food | 0.0015 | 0.0016 | 0.0001 |
| Udder | Pet food | 0.0030 | 0.0193 | 0.0014 |
| Upper throat | Pet food | 0.0009 | 0.0013 | 0.0001 |
| Water in the rumen | Spreading/Compost | 0.0000 | 0.0000 | 0.0000 |

Table 61: Total weighting by coproducts for Salers Young Bulls reared in Grazing Large Area

| COPRODUCT | Destination | Salers/young bull/grazing large area | | |
| --- | --- | --- | --- | --- |
|  |  | **Biophysical Cumulative share** | **Mass Cumulative share** | **Economic Cumulative share** |
| Abomasum | Human food | 0.0153 | 0.0026 | 0.0020 |
| Abomasum fat | Fat and greaves C3 | 0.0011 | 0.0005 | 0.0000 |
| Aponeurosis | Human food | 0.0260 | 0.0341 | 0.0342 |
| Bile | PAP C3 | 0.0000 | 0.0001 | 0.0000 |
| Blood | PAP C3 | 0.0183 | 0.0311 | 0.0070 |
| Blood | Pet food | 0.0026 | 0.0045 | 0.0003 |
| Bones | Gelatin C3 | 0.0481 | 0.0764 | 0.0002 |
| Bones of head, brain, eyes and teeth | C1-C2 for disposal | 0.0000 | 0.0000 | 0.0000 |
| Cheek | Human food | 0.0011 | 0.0016 | 0.0035 |
| Cheek | Human food | 0.0020 | 0.0029 | 0.0063 |
| Cheek trimmings | Pet food | 0.0005 | 0.0007 | 0.0001 |
| Chops | Pet food | 0.0020 | 0.0021 | 0.0002 |
| Contents of intestines | Spreading/Compost | 0.0000 | 0.0000 | 0.0000 |
| Contents of the rumen | Spreading/Compost | 0.0000 | 0.0000 | 0.0000 |
| Ears | PAP C3 | 0.0008 | 0.0010 | 0.0001 |
| Esophagus | Pet food | 0.0010 | 0.0015 | 0.0001 |
| Fat | Fat and greaves C3 | 0.0753 | 0.0818 | 0.0074 |
| Fat around heart | Fat and greaves C3 | 0.0026 | 0.0029 | 0.0003 |
| Fat in the kidney | Fat and greaves C3 | 0.0003 | 0.0004 | 0.0000 |
| Feet (without hooves) | Gelatin C3 | 0.0127 | 0.0187 | 0.0001 |
| Floatation fat | Spreading/Compost | 0.0000 | 0.0000 | 0.0000 |
| Forehead | C1-C2 for disposal | 0.0000 | 0.0000 | 0.0000 |
| Forelock | PAP C3 | 0.0028 | 0.0011 | 0.0001 |
| Gallbladder | Pet food | 0.0003 | 0.0005 | 0.0000 |
| Head trimmings | Pet food | 0.0027 | 0.0040 | 0.0003 |
| Heart | Human food | 0.0031 | 0.0047 | 0.0010 |
| Heart trimmings | Pet food | 0.0003 | 0.0005 | 0.0000 |
| Hide | Skin tannery C3 | 0.0707 | 0.0733 | 0.1223 |
| Hooves | PAP C3 | 0.0080 | 0.0032 | 0.0003 |
| Horns | PAP C3 | 0.0019 | 0.0007 | 0.0001 |
| Kidney | Human food | 0.0019 | 0.0029 | 0.0012 |
| Large intestine | C1-C2 for disposal | 0.0000 | 0.0000 | 0.0000 |
| Liver | Human food | 0.0577 | 0.0190 | 0.0092 |
| Liver trimmings | Pet food | 0.0067 | 0.0021 | 0.0002 |
| Lower jaw | PAP C3 | 0.0032 | 0.0052 | 0.0004 |
| Lungs | Pet food | 0.0084 | 0.0105 | 0.0008 |
| Mask | Skin tannery C3 | 0.0048 | 0.0050 | 0.0083 |
| Mesenteric fat | C1-C2 for disposal | 0.0000 | 0.0000 | 0.0000 |
| Muscle | Human food | 0.3172 | 0.4569 | 0.7640 |
| Muzzle | Human food | 0.0025 | 0.0026 | 0.0026 |
| Omasum | Human food | 0.0139 | 0.0024 | 0.0018 |
| Omasum fat | Fat and greaves C3 | 0.0035 | 0.0016 | 0.0001 |
| Rumen and forestomach | Human food | 0.0715 | 0.0121 | 0.0091 |
| Rumen fat | Fat and greaves C3 | 0.0052 | 0.0024 | 0.0002 |
| Sanitary seizures | C1-C2 for disposal | 0.0000 | 0.0000 | 0.0000 |
| Screening and sifting wastes | C1-C2 for disposal | 0.0000 | 0.0000 | 0.0000 |
| Small intestine | PAP C3 | 0.0832 | 0.0141 | 0.0009 |
| Spinal cord | C1-C2 for disposal | 0.0000 | 0.0000 | 0.0000 |
| Spinal cord waste | C1-C2 for disposal | 0.0000 | 0.0000 | 0.0000 |
| Spine | C1-C2 for disposal | 0.0000 | 0.0000 | 0.0000 |
| Spleen | Pet food | 0.0020 | 0.0029 | 0.0002 |
| Stillborn | PAP C3 | 0.0434 | 0.0071 | 0.0000 |
| Tallow | Fat and greaves C3 | 0.0667 | 0.0727 | 0.0066 |
| Tongue | Human food | 0.0025 | 0.0042 | 0.0067 |
| Tonsil | C1-C2 for disposal | 0.0000 | 0.0000 | 0.0000 |
| Trachea | Pet food | 0.0018 | 0.0019 | 0.0001 |
| Udder | Pet food | 0.0032 | 0.0222 | 0.0016 |
| Upper throat | Pet food | 0.0011 | 0.0016 | 0.0001 |
| Water in the rumen | Spreading/Compost | 0.0000 | 0.0000 | 0.0000 |

Table 62: Total weighting by coproducts for Salers Heifers reared in Grazing Large Area

| COPRODUCT | Destination | Salers/heifer/grazing large area | | |
| --- | --- | --- | --- | --- |
|  |  | **Biophysical Cumulative share** | **Mass Cumulative share** | **Economic Cumulative share** |
| Abomasum | Human food | 0.0153 | 0.0028 | 0.0021 |
| Abomasum fat | Fat and greaves C3 | 0.0011 | 0.0005 | 0.0000 |
| Aponeurosis | Human food | 0.0247 | 0.0326 | 0.0334 |
| Bile | PAP C3 | 0.0000 | 0.0001 | 0.0000 |
| Blood | PAP C3 | 0.0196 | 0.0335 | 0.0076 |
| Blood | Pet food | 0.0028 | 0.0048 | 0.0004 |
| Bones | Gelatin C3 | 0.0459 | 0.0732 | 0.0002 |
| Bones of head, brain, eyes and teeth | C1-C2 for disposal | 0.0000 | 0.0000 | 0.0000 |
| Cheek | Human food | 0.0011 | 0.0016 | 0.0037 |
| Cheek | Human food | 0.0022 | 0.0031 | 0.0070 |
| Cheek trimmings | Pet food | 0.0006 | 0.0009 | 0.0001 |
| Chops | Pet food | 0.0022 | 0.0023 | 0.0002 |
| Contents of intestines | Spreading/Compost | 0.0000 | 0.0000 | 0.0000 |
| Contents of the rumen | Spreading/Compost | 0.0000 | 0.0000 | 0.0000 |
| Ears | PAP C3 | 0.0010 | 0.0011 | 0.0001 |
| Esophagus | Pet food | 0.0010 | 0.0015 | 0.0001 |
| Fat | Fat and greaves C3 | 0.0721 | 0.0783 | 0.0073 |
| Fat around heart | Fat and greaves C3 | 0.0029 | 0.0031 | 0.0003 |
| Fat in the kidney | Fat and greaves C3 | 0.0003 | 0.0004 | 0.0000 |
| Feet (without hooves) | Gelatin C3 | 0.0137 | 0.0202 | 0.0001 |
| Floatation fat | Spreading/Compost | 0.0000 | 0.0000 | 0.0000 |
| Forehead | C1-C2 for disposal | 0.0000 | 0.0000 | 0.0000 |
| Forelock | PAP C3 | 0.0031 | 0.0013 | 0.0001 |
| Gallbladder | Pet food | 0.0003 | 0.0005 | 0.0000 |
| Head trimmings | Pet food | 0.0029 | 0.0043 | 0.0003 |
| Heart | Human food | 0.0033 | 0.0050 | 0.0011 |
| Heart trimmings | Pet food | 0.0003 | 0.0005 | 0.0000 |
| Hide | Skin tannery C3 | 0.0760 | 0.0791 | 0.1346 |
| Hooves | PAP C3 | 0.0087 | 0.0035 | 0.0003 |
| Horns | PAP C3 | 0.0022 | 0.0009 | 0.0001 |
| Kidney | Human food | 0.0021 | 0.0031 | 0.0013 |
| Large intestine | C1-C2 for disposal | 0.0000 | 0.0000 | 0.0000 |
| Liver | Human food | 0.0589 | 0.0205 | 0.0101 |
| Liver trimmings | Pet food | 0.0068 | 0.0023 | 0.0002 |
| Lower jaw | PAP C3 | 0.0035 | 0.0056 | 0.0005 |
| Lungs | Pet food | 0.0091 | 0.0114 | 0.0009 |
| Mask | Skin tannery C3 | 0.0052 | 0.0054 | 0.0092 |
| Mesenteric fat | C1-C2 for disposal | 0.0000 | 0.0000 | 0.0000 |
| Muscle | Human food | 0.3021 | 0.4374 | 0.7457 |
| Muzzle | Human food | 0.0027 | 0.0028 | 0.0028 |
| Omasum | Human food | 0.0139 | 0.0025 | 0.0019 |
| Omasum fat | Fat and greaves C3 | 0.0034 | 0.0016 | 0.0002 |
| Rumen and forestomach | Human food | 0.0721 | 0.0131 | 0.0100 |
| Rumen fat | Fat and greaves C3 | 0.0053 | 0.0025 | 0.0002 |
| Sanitary seizures | C1-C2 for disposal | 0.0000 | 0.0000 | 0.0000 |
| Screening and sifting wastes | C1-C2 for disposal | 0.0000 | 0.0000 | 0.0000 |
| Small intestine | PAP C3 | 0.0839 | 0.0152 | 0.0009 |
| Spinal cord | C1-C2 for disposal | 0.0000 | 0.0000 | 0.0000 |
| Spinal cord waste | C1-C2 for disposal | 0.0000 | 0.0000 | 0.0000 |
| Spine | C1-C2 for disposal | 0.0000 | 0.0000 | 0.0000 |
| Spleen | Pet food | 0.0022 | 0.0031 | 0.0002 |
| Stillborn | PAP C3 | 0.0442 | 0.0077 | 0.0000 |
| Tallow | Fat and greaves C3 | 0.0721 | 0.0785 | 0.0073 |
| Tongue | Human food | 0.0027 | 0.0045 | 0.0073 |
| Tonsil | C1-C2 for disposal | 0.0000 | 0.0000 | 0.0000 |
| Trachea | Pet food | 0.0019 | 0.0020 | 0.0002 |
| Udder | Pet food | 0.0034 | 0.0240 | 0.0018 |
| Upper throat | Pet food | 0.0011 | 0.0016 | 0.0001 |
| Water in the rumen | Spreading/Compost | 0.0000 | 0.0000 | 0.0000 |

Table 63: Total weighting by coproducts for Salers Cull Cows reared in Grazing Large Area

| COPRODUCT | Destination | Salers/Cull cow/grazing large area | | |
| --- | --- | --- | --- | --- |
|  |  | **Biophysical Cumulative share** | **Mass Cumulative share** | **Economic Cumulative share** |
| Abomasum | Human food | 0.0163 | 0.0029 | 0.0023 |
| Abomasum fat | Fat and greaves C3 | 0.0013 | 0.0006 | 0.0001 |
| Aponeurosis | Human food | 0.0234 | 0.0316 | 0.0328 |
| Bile | PAP C3 | 0.0000 | 0.0001 | 0.0000 |
| Blood | PAP C3 | 0.0201 | 0.0352 | 0.0081 |
| Blood | Pet food | 0.0029 | 0.0051 | 0.0004 |
| Bones | Gelatin C3 | 0.0433 | 0.0709 | 0.0002 |
| Bones of head, brain, eyes and teeth | C1-C2 for disposal | 0.0000 | 0.0000 | 0.0000 |
| Cheek | Human food | 0.0012 | 0.0018 | 0.0040 |
| Cheek | Human food | 0.0022 | 0.0033 | 0.0075 |
| Cheek trimmings | Pet food | 0.0006 | 0.0009 | 0.0001 |
| Chops | Pet food | 0.0023 | 0.0024 | 0.0002 |
| Contents of intestines | Spreading/Compost | 0.0000 | 0.0000 | 0.0000 |
| Contents of the rumen | Spreading/Compost | 0.0000 | 0.0000 | 0.0000 |
| Ears | PAP C3 | 0.0009 | 0.0011 | 0.0001 |
| Esophagus | Pet food | 0.0011 | 0.0016 | 0.0001 |
| Fat | Fat and greaves C3 | 0.0677 | 0.0759 | 0.0071 |
| Fat around heart | Fat and greaves C3 | 0.0029 | 0.0033 | 0.0003 |
| Fat in the kidney | Fat and greaves C3 | 0.0005 | 0.0005 | 0.0000 |
| Feet (without hooves) | Gelatin C3 | 0.0139 | 0.0211 | 0.0001 |
| Floatation fat | Spreading/Compost | 0.0000 | 0.0000 | 0.0000 |
| Forehead | C1-C2 for disposal | 0.0000 | 0.0000 | 0.0000 |
| Forelock | PAP C3 | 0.0034 | 0.0014 | 0.0001 |
| Gallbladder | Pet food | 0.0004 | 0.0006 | 0.0000 |
| Head trimmings | Pet food | 0.0030 | 0.0044 | 0.0003 |
| Heart | Human food | 0.0034 | 0.0053 | 0.0012 |
| Heart trimmings | Pet food | 0.0004 | 0.0006 | 0.0000 |
| Hide | Skin tannery C3 | 0.0778 | 0.0830 | 0.1432 |
| Hooves | PAP C3 | 0.0089 | 0.0037 | 0.0003 |
| Horns | PAP C3 | 0.0021 | 0.0009 | 0.0001 |
| Kidney | Human food | 0.0022 | 0.0033 | 0.0014 |
| Large intestine | C1-C2 for disposal | 0.0000 | 0.0000 | 0.0000 |
| Liver | Human food | 0.0623 | 0.0215 | 0.0108 |
| Liver trimmings | Pet food | 0.0073 | 0.0024 | 0.0002 |
| Lower jaw | PAP C3 | 0.0036 | 0.0059 | 0.0005 |
| Lungs | Pet food | 0.0093 | 0.0120 | 0.0009 |
| Mask | Skin tannery C3 | 0.0052 | 0.0056 | 0.0096 |
| Mesenteric fat | C1-C2 for disposal | 0.0000 | 0.0000 | 0.0000 |
| Muscle | Human food | 0.2858 | 0.4239 | 0.7327 |
| Muzzle | Human food | 0.0027 | 0.0029 | 0.0030 |
| Omasum | Human food | 0.0149 | 0.0027 | 0.0021 |
| Omasum fat | Fat and greaves C3 | 0.0037 | 0.0018 | 0.0002 |
| Rumen and forestomach | Human food | 0.0771 | 0.0138 | 0.0107 |
| Rumen fat | Fat and greaves C3 | 0.0056 | 0.0027 | 0.0003 |
| Sanitary seizures | C1-C2 for disposal | 0.0000 | 0.0000 | 0.0000 |
| Screening and sifting wastes | C1-C2 for disposal | 0.0000 | 0.0000 | 0.0000 |
| Small intestine | PAP C3 | 0.0891 | 0.0159 | 0.0010 |
| Spinal cord | C1-C2 for disposal | 0.0000 | 0.0000 | 0.0000 |
| Spinal cord waste | C1-C2 for disposal | 0.0000 | 0.0000 | 0.0000 |
| Spine | C1-C2 for disposal | 0.0000 | 0.0000 | 0.0000 |
| Spleen | Pet food | 0.0022 | 0.0033 | 0.0002 |
| Stillborn | PAP C3 | 0.0465 | 0.0080 | 0.0000 |
| Tallow | Fat and greaves C3 | 0.0733 | 0.0824 | 0.0078 |
| Tongue | Human food | 0.0027 | 0.0047 | 0.0077 |
| Tonsil | C1-C2 for disposal | 0.0000 | 0.0000 | 0.0000 |
| Trachea | Pet food | 0.0019 | 0.0020 | 0.0002 |
| Udder | Pet food | 0.0035 | 0.0252 | 0.0019 |
| Upper throat | Pet food | 0.0012 | 0.0018 | 0.0001 |
| Water in the rumen | Spreading/Compost | 0.0000 | 0.0000 | 0.0000 |

Table 64: Total weighting by coproducts for Salers Beef reared in Grazing Large Area

| COPRODUCT | Destination | Salers/beef/grazing large area | | |
| --- | --- | --- | --- | --- |
|  |  | **Biophysical Cumulative share** | **Mass Cumulative share** | **Economic Cumulative share** |
| Abomasum | Human food | 0.0162 | 0.0028 | 0.0021 |
| Abomasum fat | Fat and greaves C3 | 0.0011 | 0.0005 | 0.0000 |
| Aponeurosis | Human food | 0.0249 | 0.0331 | 0.0337 |
| Bile | PAP C3 | 0.0000 | 0.0001 | 0.0000 |
| Blood | PAP C3 | 0.0189 | 0.0328 | 0.0074 |
| Blood | Pet food | 0.0027 | 0.0046 | 0.0003 |
| Bones | Gelatin C3 | 0.0458 | 0.0743 | 0.0002 |
| Bones of head, brain, eyes and teeth | C1-C2 for disposal | 0.0000 | 0.0000 | 0.0000 |
| Cheek | Human food | 0.0011 | 0.0016 | 0.0036 |
| Cheek | Human food | 0.0020 | 0.0030 | 0.0067 |
| Cheek trimmings | Pet food | 0.0006 | 0.0009 | 0.0001 |
| Chops | Pet food | 0.0021 | 0.0023 | 0.0002 |
| Contents of intestines | Spreading/Compost | 0.0000 | 0.0000 | 0.0000 |
| Contents of the rumen | Spreading/Compost | 0.0000 | 0.0000 | 0.0000 |
| Ears | PAP C3 | 0.0009 | 0.0011 | 0.0001 |
| Esophagus | Pet food | 0.0010 | 0.0015 | 0.0001 |
| Fat | Fat and greaves C3 | 0.0703 | 0.0795 | 0.0073 |
| Fat around heart | Fat and greaves C3 | 0.0026 | 0.0030 | 0.0003 |
| Fat in the kidney | Fat and greaves C3 | 0.0003 | 0.0004 | 0.0000 |
| Feet (without hooves) | Gelatin C3 | 0.0131 | 0.0196 | 0.0001 |
| Floatation fat | Spreading/Compost | 0.0000 | 0.0000 | 0.0000 |
| Forehead | C1-C2 for disposal | 0.0000 | 0.0000 | 0.0000 |
| Forelock | PAP C3 | 0.0031 | 0.0013 | 0.0001 |
| Gallbladder | Pet food | 0.0003 | 0.0005 | 0.0000 |
| Head trimmings | Pet food | 0.0028 | 0.0041 | 0.0003 |
| Heart | Human food | 0.0032 | 0.0050 | 0.0011 |
| Heart trimmings | Pet food | 0.0003 | 0.0005 | 0.0000 |
| Hide | Skin tannery C3 | 0.0732 | 0.0771 | 0.1304 |
| Hooves | PAP C3 | 0.0083 | 0.0034 | 0.0003 |
| Horns | PAP C3 | 0.0021 | 0.0009 | 0.0001 |
| Kidney | Human food | 0.0020 | 0.0030 | 0.0012 |
| Large intestine | C1-C2 for disposal | 0.0000 | 0.0000 | 0.0000 |
| Liver | Human food | 0.0604 | 0.0199 | 0.0098 |
| Liver trimmings | Pet food | 0.0071 | 0.0023 | 0.0002 |
| Lower jaw | PAP C3 | 0.0033 | 0.0055 | 0.0005 |
| Lungs | Pet food | 0.0087 | 0.0111 | 0.0008 |
| Mask | Skin tannery C3 | 0.0050 | 0.0053 | 0.0089 |
| Mesenteric fat | C1-C2 for disposal | 0.0000 | 0.0000 | 0.0000 |
| Muscle | Human food | 0.3031 | 0.4440 | 0.7520 |
| Muzzle | Human food | 0.0026 | 0.0028 | 0.0028 |
| Omasum | Human food | 0.0148 | 0.0025 | 0.0019 |
| Omasum fat | Fat and greaves C3 | 0.0035 | 0.0016 | 0.0001 |
| Rumen and forestomach | Human food | 0.0752 | 0.0128 | 0.0097 |
| Rumen fat | Fat and greaves C3 | 0.0054 | 0.0025 | 0.0002 |
| Sanitary seizures | C1-C2 for disposal | 0.0000 | 0.0000 | 0.0000 |
| Screening and sifting wastes | C1-C2 for disposal | 0.0000 | 0.0000 | 0.0000 |
| Small intestine | PAP C3 | 0.0878 | 0.0149 | 0.0009 |
| Spinal cord | C1-C2 for disposal | 0.0000 | 0.0000 | 0.0000 |
| Spinal cord waste | C1-C2 for disposal | 0.0000 | 0.0000 | 0.0000 |
| Spine | C1-C2 for disposal | 0.0000 | 0.0000 | 0.0000 |
| Spleen | Pet food | 0.0021 | 0.0030 | 0.0002 |
| Stillborn | PAP C3 | 0.0455 | 0.0074 | 0.0000 |
| Tallow | Fat and greaves C3 | 0.0675 | 0.0765 | 0.0071 |
| Tongue | Human food | 0.0026 | 0.0044 | 0.0071 |
| Tonsil | C1-C2 for disposal | 0.0000 | 0.0000 | 0.0000 |
| Trachea | Pet food | 0.0018 | 0.0019 | 0.0001 |
| Udder | Pet food | 0.0033 | 0.0234 | 0.0017 |
| Upper throat | Pet food | 0.0011 | 0.0016 | 0.0001 |
| Water in the rumen | Spreading/Compost | 0.0000 | 0.0000 | 0.0000 |

Table 65: Total weighting by coproducts for Salers Young Bulls reared in Pasture

| COPRODUCT | Destination | Salers/young bull/pasture | | |
| --- | --- | --- | --- | --- |
|  |  | **Biophysical Cumulative share** | **Mass Cumulative share** | **Economic Cumulative share** |
| Abomasum | Human food | 0.0147 | 0.0026 | 0.0020 |
| Abomasum fat | Fat and greaves C3 | 0.0011 | 0.0005 | 0.0000 |
| Aponeurosis | Human food | 0.0257 | 0.0341 | 0.0342 |
| Bile | PAP C3 | 0.0000 | 0.0001 | 0.0000 |
| Blood | PAP C3 | 0.0181 | 0.0311 | 0.0070 |
| Blood | Pet food | 0.0026 | 0.0045 | 0.0003 |
| Bones | Gelatin C3 | 0.0488 | 0.0764 | 0.0002 |
| Bones of head, brain, eyes and teeth | C1-C2 for disposal | 0.0000 | 0.0000 | 0.0000 |
| Cheek | Human food | 0.0011 | 0.0016 | 0.0035 |
| Cheek | Human food | 0.0020 | 0.0029 | 0.0063 |
| Cheek trimmings | Pet food | 0.0005 | 0.0007 | 0.0001 |
| Chops | Pet food | 0.0020 | 0.0021 | 0.0002 |
| Contents of intestines | Spreading/Compost | 0.0000 | 0.0000 | 0.0000 |
| Contents of the rumen | Spreading/Compost | 0.0000 | 0.0000 | 0.0000 |
| Ears | PAP C3 | 0.0008 | 0.0010 | 0.0001 |
| Esophagus | Pet food | 0.0010 | 0.0015 | 0.0001 |
| Fat | Fat and greaves C3 | 0.0827 | 0.0818 | 0.0074 |
| Fat around heart | Fat and greaves C3 | 0.0029 | 0.0029 | 0.0003 |
| Fat in the kidney | Fat and greaves C3 | 0.0004 | 0.0004 | 0.0000 |
| Feet (without hooves) | Gelatin C3 | 0.0127 | 0.0187 | 0.0001 |
| Floatation fat | Spreading/Compost | 0.0000 | 0.0000 | 0.0000 |
| Forehead | C1-C2 for disposal | 0.0000 | 0.0000 | 0.0000 |
| Forelock | PAP C3 | 0.0027 | 0.0011 | 0.0001 |
| Gallbladder | Pet food | 0.0003 | 0.0005 | 0.0000 |
| Head trimmings | Pet food | 0.0027 | 0.0040 | 0.0003 |
| Heart | Human food | 0.0031 | 0.0047 | 0.0010 |
| Heart trimmings | Pet food | 0.0003 | 0.0005 | 0.0000 |
| Hide | Skin tannery C3 | 0.0700 | 0.0733 | 0.1223 |
| Hooves | PAP C3 | 0.0079 | 0.0032 | 0.0003 |
| Horns | PAP C3 | 0.0018 | 0.0007 | 0.0001 |
| Kidney | Human food | 0.0019 | 0.0029 | 0.0012 |
| Large intestine | C1-C2 for disposal | 0.0000 | 0.0000 | 0.0000 |
| Liver | Human food | 0.0555 | 0.0190 | 0.0092 |
| Liver trimmings | Pet food | 0.0064 | 0.0021 | 0.0002 |
| Lower jaw | PAP C3 | 0.0033 | 0.0052 | 0.0004 |
| Lungs | Pet food | 0.0083 | 0.0105 | 0.0008 |
| Mask | Skin tannery C3 | 0.0047 | 0.0050 | 0.0083 |
| Mesenteric fat | C1-C2 for disposal | 0.0000 | 0.0000 | 0.0000 |
| Muscle | Human food | 0.3157 | 0.4569 | 0.7640 |
| Muzzle | Human food | 0.0025 | 0.0026 | 0.0026 |
| Omasum | Human food | 0.0133 | 0.0024 | 0.0018 |
| Omasum fat | Fat and greaves C3 | 0.0035 | 0.0016 | 0.0001 |
| Rumen and forestomach | Human food | 0.0684 | 0.0121 | 0.0091 |
| Rumen fat | Fat and greaves C3 | 0.0052 | 0.0024 | 0.0002 |
| Sanitary seizures | C1-C2 for disposal | 0.0000 | 0.0000 | 0.0000 |
| Screening and sifting wastes | C1-C2 for disposal | 0.0000 | 0.0000 | 0.0000 |
| Small intestine | PAP C3 | 0.0796 | 0.0141 | 0.0009 |
| Spinal cord | C1-C2 for disposal | 0.0000 | 0.0000 | 0.0000 |
| Spinal cord waste | C1-C2 for disposal | 0.0000 | 0.0000 | 0.0000 |
| Spine | C1-C2 for disposal | 0.0000 | 0.0000 | 0.0000 |
| Spleen | Pet food | 0.0020 | 0.0029 | 0.0002 |
| Stillborn | PAP C3 | 0.0415 | 0.0071 | 0.0000 |
| Tallow | Fat and greaves C3 | 0.0733 | 0.0727 | 0.0066 |
| Tongue | Human food | 0.0026 | 0.0042 | 0.0067 |
| Tonsil | C1-C2 for disposal | 0.0000 | 0.0000 | 0.0000 |
| Trachea | Pet food | 0.0018 | 0.0019 | 0.0001 |
| Udder | Pet food | 0.0033 | 0.0222 | 0.0016 |
| Upper throat | Pet food | 0.0011 | 0.0016 | 0.0001 |
| Water in the rumen | Spreading/Compost | 0.0000 | 0.0000 | 0.0000 |

Table 66: Total weighting by coproducts for Salers Heifers reared in Pasture

| COPRODUCT | Destination | Salers/heifer/pasture | | |
| --- | --- | --- | --- | --- |
|  |  | **Biophysical Cumulative share** | **Mass Cumulative share** | **Economic Cumulative share** |
| Abomasum | Human food | 0.0146 | 0.0028 | 0.0021 |
| Abomasum fat | Fat and greaves C3 | 0.0011 | 0.0005 | 0.0000 |
| Aponeurosis | Human food | 0.0246 | 0.0326 | 0.0334 |
| Bile | PAP C3 | 0.0000 | 0.0001 | 0.0000 |
| Blood | PAP C3 | 0.0195 | 0.0335 | 0.0076 |
| Blood | Pet food | 0.0028 | 0.0048 | 0.0004 |
| Bones | Gelatin C3 | 0.0467 | 0.0732 | 0.0002 |
| Bones of head, brain, eyes and teeth | C1-C2 for disposal | 0.0000 | 0.0000 | 0.0000 |
| Cheek | Human food | 0.0011 | 0.0016 | 0.0037 |
| Cheek | Human food | 0.0022 | 0.0031 | 0.0070 |
| Cheek trimmings | Pet food | 0.0006 | 0.0009 | 0.0001 |
| Chops | Pet food | 0.0022 | 0.0023 | 0.0002 |
| Contents of intestines | Spreading/Compost | 0.0000 | 0.0000 | 0.0000 |
| Contents of the rumen | Spreading/Compost | 0.0000 | 0.0000 | 0.0000 |
| Ears | PAP C3 | 0.0010 | 0.0011 | 0.0001 |
| Esophagus | Pet food | 0.0010 | 0.0015 | 0.0001 |
| Fat | Fat and greaves C3 | 0.0789 | 0.0783 | 0.0073 |
| Fat around heart | Fat and greaves C3 | 0.0032 | 0.0031 | 0.0003 |
| Fat in the kidney | Fat and greaves C3 | 0.0004 | 0.0004 | 0.0000 |
| Feet (without hooves) | Gelatin C3 | 0.0137 | 0.0202 | 0.0001 |
| Floatation fat | Spreading/Compost | 0.0000 | 0.0000 | 0.0000 |
| Forehead | C1-C2 for disposal | 0.0000 | 0.0000 | 0.0000 |
| Forelock | PAP C3 | 0.0031 | 0.0013 | 0.0001 |
| Gallbladder | Pet food | 0.0003 | 0.0005 | 0.0000 |
| Head trimmings | Pet food | 0.0029 | 0.0043 | 0.0003 |
| Heart | Human food | 0.0033 | 0.0050 | 0.0011 |
| Heart trimmings | Pet food | 0.0003 | 0.0005 | 0.0000 |
| Hide | Skin tannery C3 | 0.0755 | 0.0791 | 0.1346 |
| Hooves | PAP C3 | 0.0086 | 0.0035 | 0.0003 |
| Horns | PAP C3 | 0.0022 | 0.0009 | 0.0001 |
| Kidney | Human food | 0.0021 | 0.0031 | 0.0013 |
| Large intestine | C1-C2 for disposal | 0.0000 | 0.0000 | 0.0000 |
| Liver | Human food | 0.0565 | 0.0205 | 0.0101 |
| Liver trimmings | Pet food | 0.0065 | 0.0023 | 0.0002 |
| Lower jaw | PAP C3 | 0.0035 | 0.0056 | 0.0005 |
| Lungs | Pet food | 0.0090 | 0.0114 | 0.0009 |
| Mask | Skin tannery C3 | 0.0052 | 0.0054 | 0.0092 |
| Mesenteric fat | C1-C2 for disposal | 0.0000 | 0.0000 | 0.0000 |
| Muscle | Human food | 0.3015 | 0.4374 | 0.7457 |
| Muzzle | Human food | 0.0026 | 0.0028 | 0.0028 |
| Omasum | Human food | 0.0132 | 0.0025 | 0.0019 |
| Omasum fat | Fat and greaves C3 | 0.0034 | 0.0016 | 0.0002 |
| Rumen and forestomach | Human food | 0.0688 | 0.0131 | 0.0100 |
| Rumen fat | Fat and greaves C3 | 0.0053 | 0.0025 | 0.0002 |
| Sanitary seizures | C1-C2 for disposal | 0.0000 | 0.0000 | 0.0000 |
| Screening and sifting wastes | C1-C2 for disposal | 0.0000 | 0.0000 | 0.0000 |
| Small intestine | PAP C3 | 0.0801 | 0.0152 | 0.0009 |
| Spinal cord | C1-C2 for disposal | 0.0000 | 0.0000 | 0.0000 |
| Spinal cord waste | C1-C2 for disposal | 0.0000 | 0.0000 | 0.0000 |
| Spine | C1-C2 for disposal | 0.0000 | 0.0000 | 0.0000 |
| Spleen | Pet food | 0.0022 | 0.0031 | 0.0002 |
| Stillborn | PAP C3 | 0.0421 | 0.0077 | 0.0000 |
| Tallow | Fat and greaves C3 | 0.0790 | 0.0785 | 0.0073 |
| Tongue | Human food | 0.0027 | 0.0045 | 0.0073 |
| Tonsil | C1-C2 for disposal | 0.0000 | 0.0000 | 0.0000 |
| Trachea | Pet food | 0.0019 | 0.0020 | 0.0002 |
| Udder | Pet food | 0.0035 | 0.0240 | 0.0018 |
| Upper throat | Pet food | 0.0011 | 0.0016 | 0.0001 |
| Water in the rumen | Spreading/Compost | 0.0000 | 0.0000 | 0.0000 |

Table 67: Total weighting by coproducts for Salers Cull Cows reared in Pasture

| COPRODUCT | Destination | Salers/Cull cow/pasture | | |
| --- | --- | --- | --- | --- |
|  |  | **Biophysical Cumulative share** | **Mass Cumulative share** | **Economic Cumulative share** |
| Abomasum | Human food | 0.0156 | 0.0029 | 0.0023 |
| Abomasum fat | Fat and greaves C3 | 0.0013 | 0.0006 | 0.0001 |
| Aponeurosis | Human food | 0.0232 | 0.0316 | 0.0328 |
| Bile | PAP C3 | 0.0000 | 0.0001 | 0.0000 |
| Blood | PAP C3 | 0.0199 | 0.0352 | 0.0081 |
| Blood | Pet food | 0.0029 | 0.0051 | 0.0004 |
| Bones | Gelatin C3 | 0.0440 | 0.0709 | 0.0002 |
| Bones of head, brain, eyes and teeth | C1-C2 for disposal | 0.0000 | 0.0000 | 0.0000 |
| Cheek | Human food | 0.0012 | 0.0018 | 0.0040 |
| Cheek | Human food | 0.0022 | 0.0033 | 0.0075 |
| Cheek trimmings | Pet food | 0.0006 | 0.0009 | 0.0001 |
| Chops | Pet food | 0.0022 | 0.0024 | 0.0002 |
| Contents of intestines | Spreading/Compost | 0.0000 | 0.0000 | 0.0000 |
| Contents of the rumen | Spreading/Compost | 0.0000 | 0.0000 | 0.0000 |
| Ears | PAP C3 | 0.0009 | 0.0011 | 0.0001 |
| Esophagus | Pet food | 0.0011 | 0.0016 | 0.0001 |
| Fat | Fat and greaves C3 | 0.0743 | 0.0759 | 0.0071 |
| Fat around heart | Fat and greaves C3 | 0.0032 | 0.0033 | 0.0003 |
| Fat in the kidney | Fat and greaves C3 | 0.0005 | 0.0005 | 0.0000 |
| Feet (without hooves) | Gelatin C3 | 0.0139 | 0.0211 | 0.0001 |
| Floatation fat | Spreading/Compost | 0.0000 | 0.0000 | 0.0000 |
| Forehead | C1-C2 for disposal | 0.0000 | 0.0000 | 0.0000 |
| Forelock | PAP C3 | 0.0033 | 0.0014 | 0.0001 |
| Gallbladder | Pet food | 0.0004 | 0.0006 | 0.0000 |
| Head trimmings | Pet food | 0.0030 | 0.0044 | 0.0003 |
| Heart | Human food | 0.0034 | 0.0053 | 0.0012 |
| Heart trimmings | Pet food | 0.0004 | 0.0006 | 0.0000 |
| Hide | Skin tannery C3 | 0.0772 | 0.0830 | 0.1432 |
| Hooves | PAP C3 | 0.0088 | 0.0037 | 0.0003 |
| Horns | PAP C3 | 0.0021 | 0.0009 | 0.0001 |
| Kidney | Human food | 0.0022 | 0.0033 | 0.0014 |
| Large intestine | C1-C2 for disposal | 0.0000 | 0.0000 | 0.0000 |
| Liver | Human food | 0.0599 | 0.0215 | 0.0108 |
| Liver trimmings | Pet food | 0.0070 | 0.0024 | 0.0002 |
| Lower jaw | PAP C3 | 0.0036 | 0.0059 | 0.0005 |
| Lungs | Pet food | 0.0092 | 0.0120 | 0.0009 |
| Mask | Skin tannery C3 | 0.0052 | 0.0056 | 0.0096 |
| Mesenteric fat | C1-C2 for disposal | 0.0000 | 0.0000 | 0.0000 |
| Muscle | Human food | 0.2851 | 0.4239 | 0.7327 |
| Muzzle | Human food | 0.0027 | 0.0029 | 0.0030 |
| Omasum | Human food | 0.0142 | 0.0027 | 0.0021 |
| Omasum fat | Fat and greaves C3 | 0.0037 | 0.0018 | 0.0002 |
| Rumen and forestomach | Human food | 0.0738 | 0.0138 | 0.0107 |
| Rumen fat | Fat and greaves C3 | 0.0056 | 0.0027 | 0.0003 |
| Sanitary seizures | C1-C2 for disposal | 0.0000 | 0.0000 | 0.0000 |
| Screening and sifting wastes | C1-C2 for disposal | 0.0000 | 0.0000 | 0.0000 |
| Small intestine | PAP C3 | 0.0853 | 0.0159 | 0.0010 |
| Spinal cord | C1-C2 for disposal | 0.0000 | 0.0000 | 0.0000 |
| Spinal cord waste | C1-C2 for disposal | 0.0000 | 0.0000 | 0.0000 |
| Spine | C1-C2 for disposal | 0.0000 | 0.0000 | 0.0000 |
| Spleen | Pet food | 0.0022 | 0.0033 | 0.0002 |
| Stillborn | PAP C3 | 0.0445 | 0.0080 | 0.0000 |
| Tallow | Fat and greaves C3 | 0.0805 | 0.0824 | 0.0078 |
| Tongue | Human food | 0.0028 | 0.0047 | 0.0077 |
| Tonsil | C1-C2 for disposal | 0.0000 | 0.0000 | 0.0000 |
| Trachea | Pet food | 0.0019 | 0.0020 | 0.0002 |
| Udder | Pet food | 0.0036 | 0.0252 | 0.0019 |
| Upper throat | Pet food | 0.0012 | 0.0018 | 0.0001 |
| Water in the rumen | Spreading/Compost | 0.0000 | 0.0000 | 0.0000 |

Table 68: Total weighting by coproducts for Salers Beef reared in Pasture

| COPRODUCT | Destination | Salers/beef/pasture | | |
| --- | --- | --- | --- | --- |
|  |  | **Biophysical Cumulative share** | **Mass Cumulative share** | **Economic Cumulative share** |
| Abomasum | Human food | 0.0156 | 0.0028 | 0.0021 |
| Abomasum fat | Fat and greaves C3 | 0.0011 | 0.0005 | 0.0000 |
| Aponeurosis | Human food | 0.0246 | 0.0331 | 0.0337 |
| Bile | PAP C3 | 0.0000 | 0.0001 | 0.0000 |
| Blood | PAP C3 | 0.0187 | 0.0328 | 0.0074 |
| Blood | Pet food | 0.0026 | 0.0046 | 0.0003 |
| Bones | Gelatin C3 | 0.0465 | 0.0743 | 0.0002 |
| Bones of head, brain, eyes and teeth | C1-C2 for disposal | 0.0000 | 0.0000 | 0.0000 |
| Cheek | Human food | 0.0011 | 0.0016 | 0.0036 |
| Cheek | Human food | 0.0020 | 0.0030 | 0.0067 |
| Cheek trimmings | Pet food | 0.0006 | 0.0009 | 0.0001 |
| Chops | Pet food | 0.0021 | 0.0023 | 0.0002 |
| Contents of intestines | Spreading/Compost | 0.0000 | 0.0000 | 0.0000 |
| Contents of the rumen | Spreading/Compost | 0.0000 | 0.0000 | 0.0000 |
| Ears | PAP C3 | 0.0009 | 0.0011 | 0.0001 |
| Esophagus | Pet food | 0.0010 | 0.0015 | 0.0001 |
| Fat | Fat and greaves C3 | 0.0774 | 0.0795 | 0.0073 |
| Fat around heart | Fat and greaves C3 | 0.0029 | 0.0030 | 0.0003 |
| Fat in the kidney | Fat and greaves C3 | 0.0004 | 0.0004 | 0.0000 |
| Feet (without hooves) | Gelatin C3 | 0.0130 | 0.0196 | 0.0001 |
| Floatation fat | Spreading/Compost | 0.0000 | 0.0000 | 0.0000 |
| Forehead | C1-C2 for disposal | 0.0000 | 0.0000 | 0.0000 |
| Forelock | PAP C3 | 0.0030 | 0.0013 | 0.0001 |
| Gallbladder | Pet food | 0.0003 | 0.0005 | 0.0000 |
| Head trimmings | Pet food | 0.0028 | 0.0041 | 0.0003 |
| Heart | Human food | 0.0032 | 0.0050 | 0.0011 |
| Heart trimmings | Pet food | 0.0003 | 0.0005 | 0.0000 |
| Hide | Skin tannery C3 | 0.0725 | 0.0771 | 0.1304 |
| Hooves | PAP C3 | 0.0082 | 0.0034 | 0.0003 |
| Horns | PAP C3 | 0.0021 | 0.0009 | 0.0001 |
| Kidney | Human food | 0.0020 | 0.0030 | 0.0012 |
| Large intestine | C1-C2 for disposal | 0.0000 | 0.0000 | 0.0000 |
| Liver | Human food | 0.0583 | 0.0199 | 0.0098 |
| Liver trimmings | Pet food | 0.0069 | 0.0023 | 0.0002 |
| Lower jaw | PAP C3 | 0.0034 | 0.0055 | 0.0005 |
| Lungs | Pet food | 0.0086 | 0.0111 | 0.0008 |
| Mask | Skin tannery C3 | 0.0049 | 0.0053 | 0.0089 |
| Mesenteric fat | C1-C2 for disposal | 0.0000 | 0.0000 | 0.0000 |
| Muscle | Human food | 0.3017 | 0.4440 | 0.7520 |
| Muzzle | Human food | 0.0026 | 0.0028 | 0.0028 |
| Omasum | Human food | 0.0142 | 0.0025 | 0.0019 |
| Omasum fat | Fat and greaves C3 | 0.0035 | 0.0016 | 0.0001 |
| Rumen and forestomach | Human food | 0.0722 | 0.0128 | 0.0097 |
| Rumen fat | Fat and greaves C3 | 0.0054 | 0.0025 | 0.0002 |
| Sanitary seizures | C1-C2 for disposal | 0.0000 | 0.0000 | 0.0000 |
| Screening and sifting wastes | C1-C2 for disposal | 0.0000 | 0.0000 | 0.0000 |
| Small intestine | PAP C3 | 0.0842 | 0.0149 | 0.0009 |
| Spinal cord | C1-C2 for disposal | 0.0000 | 0.0000 | 0.0000 |
| Spinal cord waste | C1-C2 for disposal | 0.0000 | 0.0000 | 0.0000 |
| Spine | C1-C2 for disposal | 0.0000 | 0.0000 | 0.0000 |
| Spleen | Pet food | 0.0021 | 0.0030 | 0.0002 |
| Stillborn | PAP C3 | 0.0436 | 0.0074 | 0.0000 |
| Tallow | Fat and greaves C3 | 0.0744 | 0.0765 | 0.0071 |
| Tongue | Human food | 0.0026 | 0.0044 | 0.0071 |
| Tonsil | C1-C2 for disposal | 0.0000 | 0.0000 | 0.0000 |
| Trachea | Pet food | 0.0018 | 0.0019 | 0.0001 |
| Udder | Pet food | 0.0034 | 0.0234 | 0.0017 |
| Upper throat | Pet food | 0.0011 | 0.0016 | 0.0001 |
| Water in the rumen | Spreading/Compost | 0.0000 | 0.0000 | 0.0000 |

Table 69: Total weighting by coproducts for Salers Young Bulls reared in Stall

| COPRODUCT | Destination | Salers/young bull/stall | | |
| --- | --- | --- | --- | --- |
|  |  | **Biophysical Cumulative share** | **Mass Cumulative share** | **Economic Cumulative share** |
| Abomasum | Human food | 0.0139 | 0.0026 | 0.0020 |
| Abomasum fat | Fat and greaves C3 | 0.0011 | 0.0005 | 0.0000 |
| Aponeurosis | Human food | 0.0254 | 0.0341 | 0.0342 |
| Bile | PAP C3 | 0.0000 | 0.0001 | 0.0000 |
| Blood | PAP C3 | 0.0179 | 0.0311 | 0.0070 |
| Blood | Pet food | 0.0026 | 0.0045 | 0.0003 |
| Bones | Gelatin C3 | 0.0496 | 0.0764 | 0.0002 |
| Bones of head, brain, eyes and teeth | C1-C2 for disposal | 0.0000 | 0.0000 | 0.0000 |
| Cheek | Human food | 0.0011 | 0.0016 | 0.0035 |
| Cheek | Human food | 0.0019 | 0.0029 | 0.0063 |
| Cheek trimmings | Pet food | 0.0005 | 0.0007 | 0.0001 |
| Chops | Pet food | 0.0020 | 0.0021 | 0.0002 |
| Contents of intestines | Spreading/Compost | 0.0000 | 0.0000 | 0.0000 |
| Contents of the rumen | Spreading/Compost | 0.0000 | 0.0000 | 0.0000 |
| Ears | PAP C3 | 0.0008 | 0.0010 | 0.0001 |
| Esophagus | Pet food | 0.0010 | 0.0015 | 0.0001 |
| Fat | Fat and greaves C3 | 0.0908 | 0.0818 | 0.0074 |
| Fat around heart | Fat and greaves C3 | 0.0032 | 0.0029 | 0.0003 |
| Fat in the kidney | Fat and greaves C3 | 0.0004 | 0.0004 | 0.0000 |
| Feet (without hooves) | Gelatin C3 | 0.0126 | 0.0187 | 0.0001 |
| Floatation fat | Spreading/Compost | 0.0000 | 0.0000 | 0.0000 |
| Forehead | C1-C2 for disposal | 0.0000 | 0.0000 | 0.0000 |
| Forelock | PAP C3 | 0.0027 | 0.0011 | 0.0001 |
| Gallbladder | Pet food | 0.0003 | 0.0005 | 0.0000 |
| Head trimmings | Pet food | 0.0027 | 0.0040 | 0.0003 |
| Heart | Human food | 0.0031 | 0.0047 | 0.0010 |
| Heart trimmings | Pet food | 0.0003 | 0.0005 | 0.0000 |
| Hide | Skin tannery C3 | 0.0692 | 0.0733 | 0.1223 |
| Hooves | PAP C3 | 0.0078 | 0.0032 | 0.0003 |
| Horns | PAP C3 | 0.0018 | 0.0007 | 0.0001 |
| Kidney | Human food | 0.0019 | 0.0029 | 0.0012 |
| Large intestine | C1-C2 for disposal | 0.0000 | 0.0000 | 0.0000 |
| Liver | Human food | 0.0531 | 0.0190 | 0.0092 |
| Liver trimmings | Pet food | 0.0061 | 0.0021 | 0.0002 |
| Lower jaw | PAP C3 | 0.0033 | 0.0052 | 0.0004 |
| Lungs | Pet food | 0.0082 | 0.0105 | 0.0008 |
| Mask | Skin tannery C3 | 0.0047 | 0.0050 | 0.0083 |
| Mesenteric fat | C1-C2 for disposal | 0.0000 | 0.0000 | 0.0000 |
| Muscle | Human food | 0.3141 | 0.4569 | 0.7640 |
| Muzzle | Human food | 0.0025 | 0.0026 | 0.0026 |
| Omasum | Human food | 0.0126 | 0.0024 | 0.0018 |
| Omasum fat | Fat and greaves C3 | 0.0036 | 0.0016 | 0.0001 |
| Rumen and forestomach | Human food | 0.0651 | 0.0121 | 0.0091 |
| Rumen fat | Fat and greaves C3 | 0.0052 | 0.0024 | 0.0002 |
| Sanitary seizures | C1-C2 for disposal | 0.0000 | 0.0000 | 0.0000 |
| Screening and sifting wastes | C1-C2 for disposal | 0.0000 | 0.0000 | 0.0000 |
| Small intestine | PAP C3 | 0.0757 | 0.0141 | 0.0009 |
| Spinal cord | C1-C2 for disposal | 0.0000 | 0.0000 | 0.0000 |
| Spinal cord waste | C1-C2 for disposal | 0.0000 | 0.0000 | 0.0000 |
| Spine | C1-C2 for disposal | 0.0000 | 0.0000 | 0.0000 |
| Spleen | Pet food | 0.0020 | 0.0029 | 0.0002 |
| Stillborn | PAP C3 | 0.0395 | 0.0071 | 0.0000 |
| Tallow | Fat and greaves C3 | 0.0805 | 0.0727 | 0.0066 |
| Tongue | Human food | 0.0026 | 0.0042 | 0.0067 |
| Tonsil | C1-C2 for disposal | 0.0000 | 0.0000 | 0.0000 |
| Trachea | Pet food | 0.0018 | 0.0019 | 0.0001 |
| Udder | Pet food | 0.0034 | 0.0222 | 0.0016 |
| Upper throat | Pet food | 0.0011 | 0.0016 | 0.0001 |
| Water in the rumen | Spreading/Compost | 0.0000 | 0.0000 | 0.0000 |

Table 70: Total weighting by coproducts for Salers Heifers reared in Stall

| COPRODUCT | Destination | Salers/heifer/stall | | |
| --- | --- | --- | --- | --- |
|  |  | **Biophysical Cumulative share** | **Mass Cumulative share** | **Economic Cumulative share** |
| Abomasum | Human food | 0.0138 | 0.0028 | 0.0021 |
| Abomasum fat | Fat and greaves C3 | 0.0011 | 0.0005 | 0.0000 |
| Aponeurosis | Human food | 0.0244 | 0.0326 | 0.0334 |
| Bile | PAP C3 | 0.0000 | 0.0001 | 0.0000 |
| Blood | PAP C3 | 0.0194 | 0.0335 | 0.0076 |
| Blood | Pet food | 0.0028 | 0.0048 | 0.0004 |
| Bones | Gelatin C3 | 0.0475 | 0.0732 | 0.0002 |
| Bones of head, brain, eyes and teeth | C1-C2 for disposal | 0.0000 | 0.0000 | 0.0000 |
| Cheek | Human food | 0.0011 | 0.0016 | 0.0037 |
| Cheek | Human food | 0.0021 | 0.0031 | 0.0070 |
| Cheek trimmings | Pet food | 0.0006 | 0.0009 | 0.0001 |
| Chops | Pet food | 0.0021 | 0.0023 | 0.0002 |
| Contents of intestines | Spreading/Compost | 0.0000 | 0.0000 | 0.0000 |
| Contents of the rumen | Spreading/Compost | 0.0000 | 0.0000 | 0.0000 |
| Ears | PAP C3 | 0.0010 | 0.0011 | 0.0001 |
| Esophagus | Pet food | 0.0010 | 0.0015 | 0.0001 |
| Fat | Fat and greaves C3 | 0.0863 | 0.0783 | 0.0073 |
| Fat around heart | Fat and greaves C3 | 0.0035 | 0.0031 | 0.0003 |
| Fat in the kidney | Fat and greaves C3 | 0.0004 | 0.0004 | 0.0000 |
| Feet (without hooves) | Gelatin C3 | 0.0136 | 0.0202 | 0.0001 |
| Floatation fat | Spreading/Compost | 0.0000 | 0.0000 | 0.0000 |
| Forehead | C1-C2 for disposal | 0.0000 | 0.0000 | 0.0000 |
| Forelock | PAP C3 | 0.0031 | 0.0013 | 0.0001 |
| Gallbladder | Pet food | 0.0003 | 0.0005 | 0.0000 |
| Head trimmings | Pet food | 0.0029 | 0.0043 | 0.0003 |
| Heart | Human food | 0.0033 | 0.0050 | 0.0011 |
| Heart trimmings | Pet food | 0.0003 | 0.0005 | 0.0000 |
| Hide | Skin tannery C3 | 0.0749 | 0.0791 | 0.1346 |
| Hooves | PAP C3 | 0.0086 | 0.0035 | 0.0003 |
| Horns | PAP C3 | 0.0021 | 0.0009 | 0.0001 |
| Kidney | Human food | 0.0021 | 0.0031 | 0.0013 |
| Large intestine | C1-C2 for disposal | 0.0000 | 0.0000 | 0.0000 |
| Liver | Human food | 0.0540 | 0.0205 | 0.0101 |
| Liver trimmings | Pet food | 0.0062 | 0.0023 | 0.0002 |
| Lower jaw | PAP C3 | 0.0036 | 0.0056 | 0.0005 |
| Lungs | Pet food | 0.0089 | 0.0114 | 0.0009 |
| Mask | Skin tannery C3 | 0.0051 | 0.0054 | 0.0092 |
| Mesenteric fat | C1-C2 for disposal | 0.0000 | 0.0000 | 0.0000 |
| Muscle | Human food | 0.3009 | 0.4374 | 0.7457 |
| Muzzle | Human food | 0.0026 | 0.0028 | 0.0028 |
| Omasum | Human food | 0.0126 | 0.0025 | 0.0019 |
| Omasum fat | Fat and greaves C3 | 0.0035 | 0.0016 | 0.0002 |
| Rumen and forestomach | Human food | 0.0653 | 0.0131 | 0.0100 |
| Rumen fat | Fat and greaves C3 | 0.0053 | 0.0025 | 0.0002 |
| Sanitary seizures | C1-C2 for disposal | 0.0000 | 0.0000 | 0.0000 |
| Screening and sifting wastes | C1-C2 for disposal | 0.0000 | 0.0000 | 0.0000 |
| Small intestine | PAP C3 | 0.0759 | 0.0152 | 0.0009 |
| Spinal cord | C1-C2 for disposal | 0.0000 | 0.0000 | 0.0000 |
| Spinal cord waste | C1-C2 for disposal | 0.0000 | 0.0000 | 0.0000 |
| Spine | C1-C2 for disposal | 0.0000 | 0.0000 | 0.0000 |
| Spleen | Pet food | 0.0022 | 0.0031 | 0.0002 |
| Stillborn | PAP C3 | 0.0399 | 0.0077 | 0.0000 |
| Tallow | Fat and greaves C3 | 0.0863 | 0.0785 | 0.0073 |
| Tongue | Human food | 0.0028 | 0.0045 | 0.0073 |
| Tonsil | C1-C2 for disposal | 0.0000 | 0.0000 | 0.0000 |
| Trachea | Pet food | 0.0019 | 0.0020 | 0.0002 |
| Udder | Pet food | 0.0036 | 0.0240 | 0.0018 |
| Upper throat | Pet food | 0.0011 | 0.0016 | 0.0001 |
| Water in the rumen | Spreading/Compost | 0.0000 | 0.0000 | 0.0000 |

Table 71: Total weighting by coproducts for Salers Cull Cows reared in Stall

| COPRODUCT | Destination | Salers/Cull cow/stall | | |
| --- | --- | --- | --- | --- |
|  |  | **Biophysical Cumulative share** | **Mass Cumulative share** | **Economic Cumulative share** |
| Abomasum | Human food | 0.0148 | 0.0029 | 0.0023 |
| Abomasum fat | Fat and greaves C3 | 0.0013 | 0.0006 | 0.0001 |
| Aponeurosis | Human food | 0.0230 | 0.0316 | 0.0328 |
| Bile | PAP C3 | 0.0000 | 0.0001 | 0.0000 |
| Blood | PAP C3 | 0.0198 | 0.0352 | 0.0081 |
| Blood | Pet food | 0.0028 | 0.0051 | 0.0004 |
| Bones | Gelatin C3 | 0.0448 | 0.0709 | 0.0002 |
| Bones of head, brain, eyes and teeth | C1-C2 for disposal | 0.0000 | 0.0000 | 0.0000 |
| Cheek | Human food | 0.0012 | 0.0018 | 0.0040 |
| Cheek | Human food | 0.0022 | 0.0033 | 0.0075 |
| Cheek trimmings | Pet food | 0.0006 | 0.0009 | 0.0001 |
| Chops | Pet food | 0.0022 | 0.0024 | 0.0002 |
| Contents of intestines | Spreading/Compost | 0.0000 | 0.0000 | 0.0000 |
| Contents of the rumen | Spreading/Compost | 0.0000 | 0.0000 | 0.0000 |
| Ears | PAP C3 | 0.0010 | 0.0011 | 0.0001 |
| Esophagus | Pet food | 0.0011 | 0.0016 | 0.0001 |
| Fat | Fat and greaves C3 | 0.0815 | 0.0759 | 0.0071 |
| Fat around heart | Fat and greaves C3 | 0.0035 | 0.0033 | 0.0003 |
| Fat in the kidney | Fat and greaves C3 | 0.0005 | 0.0005 | 0.0000 |
| Feet (without hooves) | Gelatin C3 | 0.0139 | 0.0211 | 0.0001 |
| Floatation fat | Spreading/Compost | 0.0000 | 0.0000 | 0.0000 |
| Forehead | C1-C2 for disposal | 0.0000 | 0.0000 | 0.0000 |
| Forelock | PAP C3 | 0.0033 | 0.0014 | 0.0001 |
| Gallbladder | Pet food | 0.0004 | 0.0006 | 0.0000 |
| Head trimmings | Pet food | 0.0029 | 0.0044 | 0.0003 |
| Heart | Human food | 0.0034 | 0.0053 | 0.0012 |
| Heart trimmings | Pet food | 0.0004 | 0.0006 | 0.0000 |
| Hide | Skin tannery C3 | 0.0766 | 0.0830 | 0.1432 |
| Hooves | PAP C3 | 0.0087 | 0.0037 | 0.0003 |
| Horns | PAP C3 | 0.0021 | 0.0009 | 0.0001 |
| Kidney | Human food | 0.0021 | 0.0033 | 0.0014 |
| Large intestine | C1-C2 for disposal | 0.0000 | 0.0000 | 0.0000 |
| Liver | Human food | 0.0574 | 0.0215 | 0.0108 |
| Liver trimmings | Pet food | 0.0067 | 0.0024 | 0.0002 |
| Lower jaw | PAP C3 | 0.0037 | 0.0059 | 0.0005 |
| Lungs | Pet food | 0.0092 | 0.0120 | 0.0009 |
| Mask | Skin tannery C3 | 0.0051 | 0.0056 | 0.0096 |
| Mesenteric fat | C1-C2 for disposal | 0.0000 | 0.0000 | 0.0000 |
| Muscle | Human food | 0.2843 | 0.4239 | 0.7327 |
| Muzzle | Human food | 0.0027 | 0.0029 | 0.0030 |
| Omasum | Human food | 0.0135 | 0.0027 | 0.0021 |
| Omasum fat | Fat and greaves C3 | 0.0038 | 0.0018 | 0.0002 |
| Rumen and forestomach | Human food | 0.0702 | 0.0138 | 0.0107 |
| Rumen fat | Fat and greaves C3 | 0.0056 | 0.0027 | 0.0003 |
| Sanitary seizures | C1-C2 for disposal | 0.0000 | 0.0000 | 0.0000 |
| Screening and sifting wastes | C1-C2 for disposal | 0.0000 | 0.0000 | 0.0000 |
| Small intestine | PAP C3 | 0.0811 | 0.0159 | 0.0010 |
| Spinal cord | C1-C2 for disposal | 0.0000 | 0.0000 | 0.0000 |
| Spinal cord waste | C1-C2 for disposal | 0.0000 | 0.0000 | 0.0000 |
| Spine | C1-C2 for disposal | 0.0000 | 0.0000 | 0.0000 |
| Spleen | Pet food | 0.0022 | 0.0033 | 0.0002 |
| Stillborn | PAP C3 | 0.0423 | 0.0080 | 0.0000 |
| Tallow | Fat and greaves C3 | 0.0883 | 0.0824 | 0.0078 |
| Tongue | Human food | 0.0028 | 0.0047 | 0.0077 |
| Tonsil | C1-C2 for disposal | 0.0000 | 0.0000 | 0.0000 |
| Trachea | Pet food | 0.0019 | 0.0020 | 0.0002 |
| Udder | Pet food | 0.0037 | 0.0252 | 0.0019 |
| Upper throat | Pet food | 0.0012 | 0.0018 | 0.0001 |
| Water in the rumen | Spreading/Compost | 0.0000 | 0.0000 | 0.0000 |

Table 72: Total weighting by coproducts for Salers Beef reared in Stall

| COPRODUCT | Destination | Salers/beef/stall | | |
| --- | --- | --- | --- | --- |
|  |  | **Biophysical Cumulative share** | **Mass Cumulative share** | **Economic Cumulative share** |
| Abomasum | Human food | 0.0148 | 0.0028 | 0.0021 |
| Abomasum fat | Fat and greaves C3 | 0.0011 | 0.0005 | 0.0000 |
| Aponeurosis | Human food | 0.0243 | 0.0331 | 0.0337 |
| Bile | PAP C3 | 0.0000 | 0.0001 | 0.0000 |
| Blood | PAP C3 | 0.0186 | 0.0328 | 0.0074 |
| Blood | Pet food | 0.0026 | 0.0046 | 0.0003 |
| Bones | Gelatin C3 | 0.0473 | 0.0743 | 0.0002 |
| Bones of head, brain, eyes and teeth | C1-C2 for disposal | 0.0000 | 0.0000 | 0.0000 |
| Cheek | Human food | 0.0011 | 0.0016 | 0.0036 |
| Cheek | Human food | 0.0020 | 0.0030 | 0.0067 |
| Cheek trimmings | Pet food | 0.0006 | 0.0009 | 0.0001 |
| Chops | Pet food | 0.0021 | 0.0023 | 0.0002 |
| Contents of intestines | Spreading/Compost | 0.0000 | 0.0000 | 0.0000 |
| Contents of the rumen | Spreading/Compost | 0.0000 | 0.0000 | 0.0000 |
| Ears | PAP C3 | 0.0009 | 0.0011 | 0.0001 |
| Esophagus | Pet food | 0.0010 | 0.0015 | 0.0001 |
| Fat | Fat and greaves C3 | 0.0852 | 0.0795 | 0.0073 |
| Fat around heart | Fat and greaves C3 | 0.0032 | 0.0030 | 0.0003 |
| Fat in the kidney | Fat and greaves C3 | 0.0004 | 0.0004 | 0.0000 |
| Feet (without hooves) | Gelatin C3 | 0.0130 | 0.0196 | 0.0001 |
| Floatation fat | Spreading/Compost | 0.0000 | 0.0000 | 0.0000 |
| Forehead | C1-C2 for disposal | 0.0000 | 0.0000 | 0.0000 |
| Forelock | PAP C3 | 0.0030 | 0.0013 | 0.0001 |
| Gallbladder | Pet food | 0.0003 | 0.0005 | 0.0000 |
| Head trimmings | Pet food | 0.0028 | 0.0041 | 0.0003 |
| Heart | Human food | 0.0032 | 0.0050 | 0.0011 |
| Heart trimmings | Pet food | 0.0003 | 0.0005 | 0.0000 |
| Hide | Skin tannery C3 | 0.0717 | 0.0771 | 0.1304 |
| Hooves | PAP C3 | 0.0081 | 0.0034 | 0.0003 |
| Horns | PAP C3 | 0.0021 | 0.0009 | 0.0001 |
| Kidney | Human food | 0.0020 | 0.0030 | 0.0012 |
| Large intestine | C1-C2 for disposal | 0.0000 | 0.0000 | 0.0000 |
| Liver | Human food | 0.0559 | 0.0199 | 0.0098 |
| Liver trimmings | Pet food | 0.0066 | 0.0023 | 0.0002 |
| Lower jaw | PAP C3 | 0.0034 | 0.0055 | 0.0005 |
| Lungs | Pet food | 0.0085 | 0.0111 | 0.0008 |
| Mask | Skin tannery C3 | 0.0049 | 0.0053 | 0.0089 |
| Mesenteric fat | C1-C2 for disposal | 0.0000 | 0.0000 | 0.0000 |
| Muscle | Human food | 0.3002 | 0.4440 | 0.7520 |
| Muzzle | Human food | 0.0026 | 0.0028 | 0.0028 |
| Omasum | Human food | 0.0135 | 0.0025 | 0.0019 |
| Omasum fat | Fat and greaves C3 | 0.0036 | 0.0016 | 0.0001 |
| Rumen and forestomach | Human food | 0.0688 | 0.0128 | 0.0097 |
| Rumen fat | Fat and greaves C3 | 0.0055 | 0.0025 | 0.0002 |
| Sanitary seizures | C1-C2 for disposal | 0.0000 | 0.0000 | 0.0000 |
| Screening and sifting wastes | C1-C2 for disposal | 0.0000 | 0.0000 | 0.0000 |
| Small intestine | PAP C3 | 0.0803 | 0.0149 | 0.0009 |
| Spinal cord | C1-C2 for disposal | 0.0000 | 0.0000 | 0.0000 |
| Spinal cord waste | C1-C2 for disposal | 0.0000 | 0.0000 | 0.0000 |
| Spine | C1-C2 for disposal | 0.0000 | 0.0000 | 0.0000 |
| Spleen | Pet food | 0.0020 | 0.0030 | 0.0002 |
| Stillborn | PAP C3 | 0.0415 | 0.0074 | 0.0000 |
| Tallow | Fat and greaves C3 | 0.0819 | 0.0765 | 0.0071 |
| Tongue | Human food | 0.0026 | 0.0044 | 0.0071 |
| Tonsil | C1-C2 for disposal | 0.0000 | 0.0000 | 0.0000 |
| Trachea | Pet food | 0.0018 | 0.0019 | 0.0001 |
| Udder | Pet food | 0.0035 | 0.0234 | 0.0017 |
| Upper throat | Pet food | 0.0011 | 0.0016 | 0.0001 |
| Water in the rumen | Spreading/Compost | 0.0000 | 0.0000 | 0.0000 |

Table 73: Total weighting by coproducts for Rouge des Prés Young Bulls reared in Grazing Large Area

| COPRODUCT | Destination | Rouge des Prés/young bull/grazing large area | | |
| --- | --- | --- | --- | --- |
|  |  | **Biophysical Cumulative share** | **Mass Cumulative share** | **Economic Cumulative share** |
| Abomasum | Human food | 0.0150 | 0.0025 | 0.0018 |
| Abomasum fat | Fat and greaves C3 | 0.0011 | 0.0005 | 0.0000 |
| Aponeurosis | Human food | 0.0270 | 0.0351 | 0.0348 |
| Bile | PAP C3 | 0.0000 | 0.0001 | 0.0000 |
| Blood | PAP C3 | 0.0175 | 0.0295 | 0.0065 |
| Blood | Pet food | 0.0025 | 0.0042 | 0.0003 |
| Bones | Gelatin C3 | 0.0499 | 0.0786 | 0.0002 |
| Bones of head, brain, eyes and teeth | C1-C2 for disposal | 0.0000 | 0.0000 | 0.0000 |
| Cheek | Human food | 0.0010 | 0.0015 | 0.0032 |
| Cheek | Human food | 0.0019 | 0.0027 | 0.0059 |
| Cheek trimmings | Pet food | 0.0005 | 0.0007 | 0.0001 |
| Chops | Pet food | 0.0019 | 0.0020 | 0.0001 |
| Contents of intestines | Spreading/Compost | 0.0000 | 0.0000 | 0.0000 |
| Contents of the rumen | Spreading/Compost | 0.0000 | 0.0000 | 0.0000 |
| Ears | PAP C3 | 0.0008 | 0.0010 | 0.0001 |
| Esophagus | Pet food | 0.0009 | 0.0014 | 0.0001 |
| Fat | Fat and greaves C3 | 0.0771 | 0.0841 | 0.0076 |
| Fat around heart | Fat and greaves C3 | 0.0025 | 0.0027 | 0.0002 |
| Fat in the kidney | Fat and greaves C3 | 0.0003 | 0.0004 | 0.0000 |
| Feet (without hooves) | Gelatin C3 | 0.0121 | 0.0177 | 0.0001 |
| Floatation fat | Spreading/Compost | 0.0000 | 0.0000 | 0.0000 |
| Forehead | C1-C2 for disposal | 0.0000 | 0.0000 | 0.0000 |
| Forelock | PAP C3 | 0.0028 | 0.0011 | 0.0001 |
| Gallbladder | Pet food | 0.0003 | 0.0005 | 0.0000 |
| Head trimmings | Pet food | 0.0026 | 0.0037 | 0.0003 |
| Heart | Human food | 0.0030 | 0.0044 | 0.0009 |
| Heart trimmings | Pet food | 0.0003 | 0.0005 | 0.0000 |
| Hide | Skin tannery C3 | 0.0678 | 0.0695 | 0.1146 |
| Hooves | PAP C3 | 0.0077 | 0.0031 | 0.0003 |
| Horns | PAP C3 | 0.0019 | 0.0007 | 0.0001 |
| Kidney | Human food | 0.0019 | 0.0027 | 0.0011 |
| Large intestine | C1-C2 for disposal | 0.0000 | 0.0000 | 0.0000 |
| Liver | Human food | 0.0563 | 0.0180 | 0.0086 |
| Liver trimmings | Pet food | 0.0064 | 0.0020 | 0.0001 |
| Lower jaw | PAP C3 | 0.0031 | 0.0049 | 0.0004 |
| Lungs | Pet food | 0.0081 | 0.0101 | 0.0007 |
| Mask | Skin tannery C3 | 0.0046 | 0.0047 | 0.0077 |
| Mesenteric fat | C1-C2 for disposal | 0.0000 | 0.0000 | 0.0000 |
| Muscle | Human food | 0.3298 | 0.4698 | 0.7759 |
| Muzzle | Human food | 0.0024 | 0.0025 | 0.0024 |
| Omasum | Human food | 0.0135 | 0.0022 | 0.0016 |
| Omasum fat | Fat and greaves C3 | 0.0033 | 0.0015 | 0.0001 |
| Rumen and forestomach | Human food | 0.0703 | 0.0116 | 0.0086 |
| Rumen fat | Fat and greaves C3 | 0.0049 | 0.0022 | 0.0002 |
| Sanitary seizures | C1-C2 for disposal | 0.0000 | 0.0000 | 0.0000 |
| Screening and sifting wastes | C1-C2 for disposal | 0.0000 | 0.0000 | 0.0000 |
| Small intestine | PAP C3 | 0.0815 | 0.0134 | 0.0008 |
| Spinal cord | C1-C2 for disposal | 0.0000 | 0.0000 | 0.0000 |
| Spinal cord waste | C1-C2 for disposal | 0.0000 | 0.0000 | 0.0000 |
| Spine | C1-C2 for disposal | 0.0000 | 0.0000 | 0.0000 |
| Spleen | Pet food | 0.0019 | 0.0027 | 0.0002 |
| Stillborn | PAP C3 | 0.0422 | 0.0066 | 0.0000 |
| Tallow | Fat and greaves C3 | 0.0630 | 0.0689 | 0.0062 |
| Tongue | Human food | 0.0024 | 0.0039 | 0.0062 |
| Tonsil | C1-C2 for disposal | 0.0000 | 0.0000 | 0.0000 |
| Trachea | Pet food | 0.0017 | 0.0017 | 0.0001 |
| Udder | Pet food | 0.0030 | 0.0210 | 0.0015 |
| Upper throat | Pet food | 0.0010 | 0.0015 | 0.0001 |
| Water in the rumen | Spreading/Compost | 0.0000 | 0.0000 | 0.0000 |

Table 74: Total weighting by coproducts for Rouge des Prés Heifers reared in Grazing Large Area

| COPRODUCT | Destination | Rouge des Prés/heifer/grazing large area | | |
| --- | --- | --- | --- | --- |
|  |  | **Biophysical Cumulative share** | **Mass Cumulative share** | **Economic Cumulative share** |
| Abomasum | Human food | 0.0162 | 0.0027 | 0.0021 |
| Abomasum fat | Fat and greaves C3 | 0.0011 | 0.0005 | 0.0000 |
| Aponeurosis | Human food | 0.0254 | 0.0336 | 0.0340 |
| Bile | PAP C3 | 0.0000 | 0.0001 | 0.0000 |
| Blood | PAP C3 | 0.0185 | 0.0319 | 0.0072 |
| Blood | Pet food | 0.0027 | 0.0046 | 0.0003 |
| Bones | Gelatin C3 | 0.0469 | 0.0753 | 0.0002 |
| Bones of head, brain, eyes and teeth | C1-C2 for disposal | 0.0000 | 0.0000 | 0.0000 |
| Cheek | Human food | 0.0011 | 0.0016 | 0.0036 |
| Cheek | Human food | 0.0020 | 0.0030 | 0.0066 |
| Cheek trimmings | Pet food | 0.0005 | 0.0007 | 0.0001 |
| Chops | Pet food | 0.0020 | 0.0021 | 0.0002 |
| Contents of intestines | Spreading/Compost | 0.0000 | 0.0000 | 0.0000 |
| Contents of the rumen | Spreading/Compost | 0.0000 | 0.0000 | 0.0000 |
| Ears | PAP C3 | 0.0009 | 0.0011 | 0.0001 |
| Esophagus | Pet food | 0.0010 | 0.0015 | 0.0001 |
| Fat | Fat and greaves C3 | 0.0723 | 0.0807 | 0.0074 |
| Fat around heart | Fat and greaves C3 | 0.0027 | 0.0030 | 0.0003 |
| Fat in the kidney | Fat and greaves C3 | 0.0003 | 0.0004 | 0.0000 |
| Feet (without hooves) | Gelatin C3 | 0.0129 | 0.0192 | 0.0001 |
| Floatation fat | Spreading/Compost | 0.0000 | 0.0000 | 0.0000 |
| Forehead | C1-C2 for disposal | 0.0000 | 0.0000 | 0.0000 |
| Forelock | PAP C3 | 0.0031 | 0.0012 | 0.0001 |
| Gallbladder | Pet food | 0.0003 | 0.0005 | 0.0000 |
| Head trimmings | Pet food | 0.0027 | 0.0040 | 0.0003 |
| Heart | Human food | 0.0032 | 0.0049 | 0.0010 |
| Heart trimmings | Pet food | 0.0003 | 0.0005 | 0.0000 |
| Hide | Skin tannery C3 | 0.0719 | 0.0752 | 0.1263 |
| Hooves | PAP C3 | 0.0083 | 0.0034 | 0.0003 |
| Horns | PAP C3 | 0.0018 | 0.0007 | 0.0001 |
| Kidney | Human food | 0.0020 | 0.0030 | 0.0012 |
| Large intestine | C1-C2 for disposal | 0.0000 | 0.0000 | 0.0000 |
| Liver | Human food | 0.0594 | 0.0194 | 0.0095 |
| Liver trimmings | Pet food | 0.0067 | 0.0021 | 0.0002 |
| Lower jaw | PAP C3 | 0.0033 | 0.0054 | 0.0005 |
| Lungs | Pet food | 0.0086 | 0.0108 | 0.0008 |
| Mask | Skin tannery C3 | 0.0049 | 0.0051 | 0.0086 |
| Mesenteric fat | C1-C2 for disposal | 0.0000 | 0.0000 | 0.0000 |
| Muscle | Human food | 0.3099 | 0.4504 | 0.7579 |
| Muzzle | Human food | 0.0026 | 0.0027 | 0.0028 |
| Omasum | Human food | 0.0140 | 0.0024 | 0.0018 |
| Omasum fat | Fat and greaves C3 | 0.0035 | 0.0016 | 0.0001 |
| Rumen and forestomach | Human food | 0.0738 | 0.0125 | 0.0094 |
| Rumen fat | Fat and greaves C3 | 0.0052 | 0.0024 | 0.0002 |
| Sanitary seizures | C1-C2 for disposal | 0.0000 | 0.0000 | 0.0000 |
| Screening and sifting wastes | C1-C2 for disposal | 0.0000 | 0.0000 | 0.0000 |
| Small intestine | PAP C3 | 0.0856 | 0.0144 | 0.0009 |
| Spinal cord | C1-C2 for disposal | 0.0000 | 0.0000 | 0.0000 |
| Spinal cord waste | C1-C2 for disposal | 0.0000 | 0.0000 | 0.0000 |
| Spine | C1-C2 for disposal | 0.0000 | 0.0000 | 0.0000 |
| Spleen | Pet food | 0.0021 | 0.0030 | 0.0002 |
| Stillborn | PAP C3 | 0.0447 | 0.0072 | 0.0000 |
| Tallow | Fat and greaves C3 | 0.0667 | 0.0746 | 0.0068 |
| Tongue | Human food | 0.0025 | 0.0042 | 0.0068 |
| Tonsil | C1-C2 for disposal | 0.0000 | 0.0000 | 0.0000 |
| Trachea | Pet food | 0.0018 | 0.0019 | 0.0001 |
| Udder | Pet food | 0.0032 | 0.0228 | 0.0017 |
| Upper throat | Pet food | 0.0011 | 0.0016 | 0.0001 |
| Water in the rumen | Spreading/Compost | 0.0000 | 0.0000 | 0.0000 |

Table 75: Total weighting by coproducts for Rouge des Prés Cull Cows reared in Grazing Large Area

| COPRODUCT | Destination | Rouge des Prés/Cull cow/grazing large area | | |
| --- | --- | --- | --- | --- |
|  |  | **Biophysical Cumulative share** | **Mass Cumulative share** | **Economic Cumulative share** |
| Abomasum | Human food | 0.0167 | 0.0028 | 0.0021 |
| Abomasum fat | Fat and greaves C3 | 0.0011 | 0.0005 | 0.0000 |
| Aponeurosis | Human food | 0.0243 | 0.0326 | 0.0334 |
| Bile | PAP C3 | 0.0000 | 0.0001 | 0.0000 |
| Blood | PAP C3 | 0.0192 | 0.0335 | 0.0076 |
| Blood | Pet food | 0.0027 | 0.0048 | 0.0004 |
| Bones | Gelatin C3 | 0.0443 | 0.0732 | 0.0002 |
| Bones of head, brain, eyes and teeth | C1-C2 for disposal | 0.0000 | 0.0000 | 0.0000 |
| Cheek | Human food | 0.0011 | 0.0016 | 0.0037 |
| Cheek | Human food | 0.0021 | 0.0031 | 0.0070 |
| Cheek trimmings | Pet food | 0.0006 | 0.0009 | 0.0001 |
| Chops | Pet food | 0.0021 | 0.0023 | 0.0002 |
| Contents of intestines | Spreading/Compost | 0.0000 | 0.0000 | 0.0000 |
| Contents of the rumen | Spreading/Compost | 0.0000 | 0.0000 | 0.0000 |
| Ears | PAP C3 | 0.0009 | 0.0011 | 0.0001 |
| Esophagus | Pet food | 0.0010 | 0.0015 | 0.0001 |
| Fat | Fat and greaves C3 | 0.0652 | 0.0783 | 0.0073 |
| Fat around heart | Fat and greaves C3 | 0.0026 | 0.0031 | 0.0003 |
| Fat in the kidney | Fat and greaves C3 | 0.0003 | 0.0004 | 0.0000 |
| Feet (without hooves) | Gelatin C3 | 0.0133 | 0.0202 | 0.0001 |
| Floatation fat | Spreading/Compost | 0.0000 | 0.0000 | 0.0000 |
| Forehead | C1-C2 for disposal | 0.0000 | 0.0000 | 0.0000 |
| Forelock | PAP C3 | 0.0031 | 0.0013 | 0.0001 |
| Gallbladder | Pet food | 0.0003 | 0.0005 | 0.0000 |
| Head trimmings | Pet food | 0.0029 | 0.0043 | 0.0003 |
| Heart | Human food | 0.0032 | 0.0050 | 0.0011 |
| Heart trimmings | Pet food | 0.0003 | 0.0005 | 0.0000 |
| Hide | Skin tannery C3 | 0.0745 | 0.0791 | 0.1346 |
| Hooves | PAP C3 | 0.0086 | 0.0035 | 0.0003 |
| Horns | PAP C3 | 0.0021 | 0.0009 | 0.0001 |
| Kidney | Human food | 0.0021 | 0.0031 | 0.0013 |
| Large intestine | C1-C2 for disposal | 0.0000 | 0.0000 | 0.0000 |
| Liver | Human food | 0.0634 | 0.0205 | 0.0101 |
| Liver trimmings | Pet food | 0.0073 | 0.0023 | 0.0002 |
| Lower jaw | PAP C3 | 0.0034 | 0.0056 | 0.0005 |
| Lungs | Pet food | 0.0089 | 0.0114 | 0.0009 |
| Mask | Skin tannery C3 | 0.0051 | 0.0054 | 0.0092 |
| Mesenteric fat | C1-C2 for disposal | 0.0000 | 0.0000 | 0.0000 |
| Muscle | Human food | 0.2956 | 0.4374 | 0.7457 |
| Muzzle | Human food | 0.0026 | 0.0028 | 0.0028 |
| Omasum | Human food | 0.0152 | 0.0025 | 0.0019 |
| Omasum fat | Fat and greaves C3 | 0.0035 | 0.0016 | 0.0002 |
| Rumen and forestomach | Human food | 0.0788 | 0.0131 | 0.0100 |
| Rumen fat | Fat and greaves C3 | 0.0054 | 0.0025 | 0.0002 |
| Sanitary seizures | C1-C2 for disposal | 0.0000 | 0.0000 | 0.0000 |
| Screening and sifting wastes | C1-C2 for disposal | 0.0000 | 0.0000 | 0.0000 |
| Small intestine | PAP C3 | 0.0917 | 0.0152 | 0.0009 |
| Spinal cord | C1-C2 for disposal | 0.0000 | 0.0000 | 0.0000 |
| Spinal cord waste | C1-C2 for disposal | 0.0000 | 0.0000 | 0.0000 |
| Spine | C1-C2 for disposal | 0.0000 | 0.0000 | 0.0000 |
| Spleen | Pet food | 0.0021 | 0.0031 | 0.0002 |
| Stillborn | PAP C3 | 0.0483 | 0.0077 | 0.0000 |
| Tallow | Fat and greaves C3 | 0.0652 | 0.0785 | 0.0073 |
| Tongue | Human food | 0.0026 | 0.0045 | 0.0073 |
| Tonsil | C1-C2 for disposal | 0.0000 | 0.0000 | 0.0000 |
| Trachea | Pet food | 0.0019 | 0.0020 | 0.0002 |
| Udder | Pet food | 0.0033 | 0.0240 | 0.0018 |
| Upper throat | Pet food | 0.0011 | 0.0016 | 0.0001 |
| Water in the rumen | Spreading/Compost | 0.0000 | 0.0000 | 0.0000 |

Table 76: Total weighting by coproducts for Rouge des Prés Beef reared in Grazing Large Area

| COPRODUCT | Destination | Rouge des Prés/beef/grazing large area | | |
| --- | --- | --- | --- | --- |
|  |  | **Biophysical Cumulative share** | **Mass Cumulative share** | **Economic Cumulative share** |
| Abomasum | Human food | 0.0172 | 0.0026 | 0.0020 |
| Abomasum fat | Fat and greaves C3 | 0.0011 | 0.0005 | 0.0000 |
| Aponeurosis | Human food | 0.0262 | 0.0341 | 0.0342 |
| Bile | PAP C3 | 0.0000 | 0.0001 | 0.0000 |
| Blood | PAP C3 | 0.0183 | 0.0311 | 0.0070 |
| Blood | Pet food | 0.0026 | 0.0045 | 0.0003 |
| Bones | Gelatin C3 | 0.0459 | 0.0764 | 0.0002 |
| Bones of head, brain, eyes and teeth | C1-C2 for disposal | 0.0000 | 0.0000 | 0.0000 |
| Cheek | Human food | 0.0011 | 0.0016 | 0.0035 |
| Cheek | Human food | 0.0020 | 0.0029 | 0.0063 |
| Cheek trimmings | Pet food | 0.0005 | 0.0007 | 0.0001 |
| Chops | Pet food | 0.0020 | 0.0021 | 0.0002 |
| Contents of intestines | Spreading/Compost | 0.0000 | 0.0000 | 0.0000 |
| Contents of the rumen | Spreading/Compost | 0.0000 | 0.0000 | 0.0000 |
| Ears | PAP C3 | 0.0008 | 0.0010 | 0.0001 |
| Esophagus | Pet food | 0.0010 | 0.0015 | 0.0001 |
| Fat | Fat and greaves C3 | 0.0580 | 0.0818 | 0.0074 |
| Fat around heart | Fat and greaves C3 | 0.0020 | 0.0029 | 0.0003 |
| Fat in the kidney | Fat and greaves C3 | 0.0003 | 0.0004 | 0.0000 |
| Feet (without hooves) | Gelatin C3 | 0.0126 | 0.0187 | 0.0001 |
| Floatation fat | Spreading/Compost | 0.0000 | 0.0000 | 0.0000 |
| Forehead | C1-C2 for disposal | 0.0000 | 0.0000 | 0.0000 |
| Forelock | PAP C3 | 0.0028 | 0.0011 | 0.0001 |
| Gallbladder | Pet food | 0.0003 | 0.0005 | 0.0000 |
| Head trimmings | Pet food | 0.0027 | 0.0040 | 0.0003 |
| Heart | Human food | 0.0031 | 0.0047 | 0.0010 |
| Heart trimmings | Pet food | 0.0003 | 0.0005 | 0.0000 |
| Hide | Skin tannery C3 | 0.0712 | 0.0733 | 0.1223 |
| Hooves | PAP C3 | 0.0081 | 0.0032 | 0.0003 |
| Horns | PAP C3 | 0.0019 | 0.0007 | 0.0001 |
| Kidney | Human food | 0.0020 | 0.0029 | 0.0012 |
| Large intestine | C1-C2 for disposal | 0.0000 | 0.0000 | 0.0000 |
| Liver | Human food | 0.0640 | 0.0190 | 0.0092 |
| Liver trimmings | Pet food | 0.0074 | 0.0021 | 0.0002 |
| Lower jaw | PAP C3 | 0.0031 | 0.0052 | 0.0004 |
| Lungs | Pet food | 0.0085 | 0.0105 | 0.0008 |
| Mask | Skin tannery C3 | 0.0048 | 0.0050 | 0.0083 |
| Mesenteric fat | C1-C2 for disposal | 0.0000 | 0.0000 | 0.0000 |
| Muscle | Human food | 0.3163 | 0.4569 | 0.7640 |
| Muzzle | Human food | 0.0025 | 0.0026 | 0.0026 |
| Omasum | Human food | 0.0156 | 0.0024 | 0.0018 |
| Omasum fat | Fat and greaves C3 | 0.0035 | 0.0016 | 0.0001 |
| Rumen and forestomach | Human food | 0.0805 | 0.0121 | 0.0091 |
| Rumen fat | Fat and greaves C3 | 0.0052 | 0.0024 | 0.0002 |
| Sanitary seizures | C1-C2 for disposal | 0.0000 | 0.0000 | 0.0000 |
| Screening and sifting wastes | C1-C2 for disposal | 0.0000 | 0.0000 | 0.0000 |
| Small intestine | PAP C3 | 0.0936 | 0.0141 | 0.0009 |
| Spinal cord | C1-C2 for disposal | 0.0000 | 0.0000 | 0.0000 |
| Spinal cord waste | C1-C2 for disposal | 0.0000 | 0.0000 | 0.0000 |
| Spine | C1-C2 for disposal | 0.0000 | 0.0000 | 0.0000 |
| Spleen | Pet food | 0.0020 | 0.0029 | 0.0002 |
| Stillborn | PAP C3 | 0.0490 | 0.0071 | 0.0000 |
| Tallow | Fat and greaves C3 | 0.0514 | 0.0727 | 0.0066 |
| Tongue | Human food | 0.0025 | 0.0042 | 0.0067 |
| Tonsil | C1-C2 for disposal | 0.0000 | 0.0000 | 0.0000 |
| Trachea | Pet food | 0.0018 | 0.0019 | 0.0001 |
| Udder | Pet food | 0.0029 | 0.0222 | 0.0016 |
| Upper throat | Pet food | 0.0011 | 0.0016 | 0.0001 |
| Water in the rumen | Spreading/Compost | 0.0000 | 0.0000 | 0.0000 |

Table 77: Total weighting by coproducts for Rouge des Prés Young Bulls reared in Pasture

| COPRODUCT | Destination | Rouge des Prés/young bull/pasture | | |
| --- | --- | --- | --- | --- |
|  |  | **Biophysical Cumulative share** | **Mass Cumulative share** | **Economic Cumulative share** |
| Abomasum | Human food | 0.0143 | 0.0025 | 0.0018 |
| Abomasum fat | Fat and greaves C3 | 0.0011 | 0.0005 | 0.0000 |
| Aponeurosis | Human food | 0.0267 | 0.0351 | 0.0348 |
| Bile | PAP C3 | 0.0000 | 0.0001 | 0.0000 |
| Blood | PAP C3 | 0.0173 | 0.0295 | 0.0065 |
| Blood | Pet food | 0.0025 | 0.0042 | 0.0003 |
| Bones | Gelatin C3 | 0.0506 | 0.0786 | 0.0002 |
| Bones of head, brain, eyes and teeth | C1-C2 for disposal | 0.0000 | 0.0000 | 0.0000 |
| Cheek | Human food | 0.0010 | 0.0015 | 0.0032 |
| Cheek | Human food | 0.0019 | 0.0027 | 0.0059 |
| Cheek trimmings | Pet food | 0.0005 | 0.0007 | 0.0001 |
| Chops | Pet food | 0.0019 | 0.0020 | 0.0001 |
| Contents of intestines | Spreading/Compost | 0.0000 | 0.0000 | 0.0000 |
| Contents of the rumen | Spreading/Compost | 0.0000 | 0.0000 | 0.0000 |
| Ears | PAP C3 | 0.0008 | 0.0010 | 0.0001 |
| Esophagus | Pet food | 0.0009 | 0.0014 | 0.0001 |
| Fat | Fat and greaves C3 | 0.0848 | 0.0841 | 0.0076 |
| Fat around heart | Fat and greaves C3 | 0.0027 | 0.0027 | 0.0002 |
| Fat in the kidney | Fat and greaves C3 | 0.0004 | 0.0004 | 0.0000 |
| Feet (without hooves) | Gelatin C3 | 0.0121 | 0.0177 | 0.0001 |
| Floatation fat | Spreading/Compost | 0.0000 | 0.0000 | 0.0000 |
| Forehead | C1-C2 for disposal | 0.0000 | 0.0000 | 0.0000 |
| Forelock | PAP C3 | 0.0028 | 0.0011 | 0.0001 |
| Gallbladder | Pet food | 0.0003 | 0.0005 | 0.0000 |
| Head trimmings | Pet food | 0.0026 | 0.0037 | 0.0003 |
| Heart | Human food | 0.0029 | 0.0044 | 0.0009 |
| Heart trimmings | Pet food | 0.0003 | 0.0005 | 0.0000 |
| Hide | Skin tannery C3 | 0.0671 | 0.0695 | 0.1146 |
| Hooves | PAP C3 | 0.0076 | 0.0031 | 0.0003 |
| Horns | PAP C3 | 0.0018 | 0.0007 | 0.0001 |
| Kidney | Human food | 0.0018 | 0.0027 | 0.0011 |
| Large intestine | C1-C2 for disposal | 0.0000 | 0.0000 | 0.0000 |
| Liver | Human food | 0.0542 | 0.0180 | 0.0086 |
| Liver trimmings | Pet food | 0.0062 | 0.0020 | 0.0001 |
| Lower jaw | PAP C3 | 0.0031 | 0.0049 | 0.0004 |
| Lungs | Pet food | 0.0080 | 0.0101 | 0.0007 |
| Mask | Skin tannery C3 | 0.0045 | 0.0047 | 0.0077 |
| Mesenteric fat | C1-C2 for disposal | 0.0000 | 0.0000 | 0.0000 |
| Muscle | Human food | 0.3279 | 0.4698 | 0.7759 |
| Muzzle | Human food | 0.0024 | 0.0025 | 0.0024 |
| Omasum | Human food | 0.0129 | 0.0022 | 0.0016 |
| Omasum fat | Fat and greaves C3 | 0.0033 | 0.0015 | 0.0001 |
| Rumen and forestomach | Human food | 0.0674 | 0.0116 | 0.0086 |
| Rumen fat | Fat and greaves C3 | 0.0050 | 0.0022 | 0.0002 |
| Sanitary seizures | C1-C2 for disposal | 0.0000 | 0.0000 | 0.0000 |
| Screening and sifting wastes | C1-C2 for disposal | 0.0000 | 0.0000 | 0.0000 |
| Small intestine | PAP C3 | 0.0782 | 0.0134 | 0.0008 |
| Spinal cord | C1-C2 for disposal | 0.0000 | 0.0000 | 0.0000 |
| Spinal cord waste | C1-C2 for disposal | 0.0000 | 0.0000 | 0.0000 |
| Spine | C1-C2 for disposal | 0.0000 | 0.0000 | 0.0000 |
| Spleen | Pet food | 0.0019 | 0.0027 | 0.0002 |
| Stillborn | PAP C3 | 0.0404 | 0.0066 | 0.0000 |
| Tallow | Fat and greaves C3 | 0.0694 | 0.0689 | 0.0062 |
| Tongue | Human food | 0.0024 | 0.0039 | 0.0062 |
| Tonsil | C1-C2 for disposal | 0.0000 | 0.0000 | 0.0000 |
| Trachea | Pet food | 0.0017 | 0.0017 | 0.0001 |
| Udder | Pet food | 0.0031 | 0.0210 | 0.0015 |
| Upper throat | Pet food | 0.0010 | 0.0015 | 0.0001 |
| Water in the rumen | Spreading/Compost | 0.0000 | 0.0000 | 0.0000 |

Table 78: Total weighting by coproducts for Rouge des Prés Heifers reared in Pasture

| COPRODUCT | Destination | Rouge des Prés/heifer/pasture | | |
| --- | --- | --- | --- | --- |
|  |  | **Biophysical Cumulative share** | **Mass Cumulative share** | **Economic Cumulative share** |
| Abomasum | Human food | 0.0156 | 0.0027 | 0.0021 |
| Abomasum fat | Fat and greaves C3 | 0.0011 | 0.0005 | 0.0000 |
| Aponeurosis | Human food | 0.0251 | 0.0336 | 0.0340 |
| Bile | PAP C3 | 0.0000 | 0.0001 | 0.0000 |
| Blood | PAP C3 | 0.0184 | 0.0319 | 0.0072 |
| Blood | Pet food | 0.0027 | 0.0046 | 0.0003 |
| Bones | Gelatin C3 | 0.0476 | 0.0753 | 0.0002 |
| Bones of head, brain, eyes and teeth | C1-C2 for disposal | 0.0000 | 0.0000 | 0.0000 |
| Cheek | Human food | 0.0011 | 0.0016 | 0.0036 |
| Cheek | Human food | 0.0020 | 0.0030 | 0.0066 |
| Cheek trimmings | Pet food | 0.0005 | 0.0007 | 0.0001 |
| Chops | Pet food | 0.0020 | 0.0021 | 0.0002 |
| Contents of intestines | Spreading/Compost | 0.0000 | 0.0000 | 0.0000 |
| Contents of the rumen | Spreading/Compost | 0.0000 | 0.0000 | 0.0000 |
| Ears | PAP C3 | 0.0009 | 0.0011 | 0.0001 |
| Esophagus | Pet food | 0.0010 | 0.0015 | 0.0001 |
| Fat | Fat and greaves C3 | 0.0796 | 0.0807 | 0.0074 |
| Fat around heart | Fat and greaves C3 | 0.0029 | 0.0030 | 0.0003 |
| Fat in the kidney | Fat and greaves C3 | 0.0004 | 0.0004 | 0.0000 |
| Feet (without hooves) | Gelatin C3 | 0.0128 | 0.0192 | 0.0001 |
| Floatation fat | Spreading/Compost | 0.0000 | 0.0000 | 0.0000 |
| Forehead | C1-C2 for disposal | 0.0000 | 0.0000 | 0.0000 |
| Forelock | PAP C3 | 0.0030 | 0.0012 | 0.0001 |
| Gallbladder | Pet food | 0.0003 | 0.0005 | 0.0000 |
| Head trimmings | Pet food | 0.0027 | 0.0040 | 0.0003 |
| Heart | Human food | 0.0032 | 0.0049 | 0.0010 |
| Heart trimmings | Pet food | 0.0003 | 0.0005 | 0.0000 |
| Hide | Skin tannery C3 | 0.0712 | 0.0752 | 0.1263 |
| Hooves | PAP C3 | 0.0082 | 0.0034 | 0.0003 |
| Horns | PAP C3 | 0.0018 | 0.0007 | 0.0001 |
| Kidney | Human food | 0.0020 | 0.0030 | 0.0012 |
| Large intestine | C1-C2 for disposal | 0.0000 | 0.0000 | 0.0000 |
| Liver | Human food | 0.0572 | 0.0194 | 0.0095 |
| Liver trimmings | Pet food | 0.0065 | 0.0021 | 0.0002 |
| Lower jaw | PAP C3 | 0.0033 | 0.0054 | 0.0005 |
| Lungs | Pet food | 0.0085 | 0.0108 | 0.0008 |
| Mask | Skin tannery C3 | 0.0048 | 0.0051 | 0.0086 |
| Mesenteric fat | C1-C2 for disposal | 0.0000 | 0.0000 | 0.0000 |
| Muscle | Human food | 0.3084 | 0.4504 | 0.7579 |
| Muzzle | Human food | 0.0026 | 0.0027 | 0.0028 |
| Omasum | Human food | 0.0134 | 0.0024 | 0.0018 |
| Omasum fat | Fat and greaves C3 | 0.0036 | 0.0016 | 0.0001 |
| Rumen and forestomach | Human food | 0.0708 | 0.0125 | 0.0094 |
| Rumen fat | Fat and greaves C3 | 0.0052 | 0.0024 | 0.0002 |
| Sanitary seizures | C1-C2 for disposal | 0.0000 | 0.0000 | 0.0000 |
| Screening and sifting wastes | C1-C2 for disposal | 0.0000 | 0.0000 | 0.0000 |
| Small intestine | PAP C3 | 0.0821 | 0.0144 | 0.0009 |
| Spinal cord | C1-C2 for disposal | 0.0000 | 0.0000 | 0.0000 |
| Spinal cord waste | C1-C2 for disposal | 0.0000 | 0.0000 | 0.0000 |
| Spine | C1-C2 for disposal | 0.0000 | 0.0000 | 0.0000 |
| Spleen | Pet food | 0.0021 | 0.0030 | 0.0002 |
| Stillborn | PAP C3 | 0.0429 | 0.0072 | 0.0000 |
| Tallow | Fat and greaves C3 | 0.0734 | 0.0746 | 0.0068 |
| Tongue | Human food | 0.0025 | 0.0042 | 0.0068 |
| Tonsil | C1-C2 for disposal | 0.0000 | 0.0000 | 0.0000 |
| Trachea | Pet food | 0.0018 | 0.0019 | 0.0001 |
| Udder | Pet food | 0.0033 | 0.0228 | 0.0017 |
| Upper throat | Pet food | 0.0011 | 0.0016 | 0.0001 |
| Water in the rumen | Spreading/Compost | 0.0000 | 0.0000 | 0.0000 |

Table 79: Total weighting by coproducts for Rouge des Prés Cull Cows reared in Pasture

| COPRODUCT | Destination | Rouge des Prés/Cull cow/pasture | | |
| --- | --- | --- | --- | --- |
|  |  | **Biophysical Cumulative share** | **Mass Cumulative share** | **Economic Cumulative share** |
| Abomasum | Human food | 0.0161 | 0.0028 | 0.0021 |
| Abomasum fat | Fat and greaves C3 | 0.0011 | 0.0005 | 0.0000 |
| Aponeurosis | Human food | 0.0241 | 0.0326 | 0.0334 |
| Bile | PAP C3 | 0.0000 | 0.0001 | 0.0000 |
| Blood | PAP C3 | 0.0190 | 0.0335 | 0.0076 |
| Blood | Pet food | 0.0027 | 0.0048 | 0.0004 |
| Bones | Gelatin C3 | 0.0449 | 0.0732 | 0.0002 |
| Bones of head, brain, eyes and teeth | C1-C2 for disposal | 0.0000 | 0.0000 | 0.0000 |
| Cheek | Human food | 0.0011 | 0.0016 | 0.0037 |
| Cheek | Human food | 0.0021 | 0.0031 | 0.0070 |
| Cheek trimmings | Pet food | 0.0006 | 0.0009 | 0.0001 |
| Chops | Pet food | 0.0021 | 0.0023 | 0.0002 |
| Contents of intestines | Spreading/Compost | 0.0000 | 0.0000 | 0.0000 |
| Contents of the rumen | Spreading/Compost | 0.0000 | 0.0000 | 0.0000 |
| Ears | PAP C3 | 0.0009 | 0.0011 | 0.0001 |
| Esophagus | Pet food | 0.0010 | 0.0015 | 0.0001 |
| Fat | Fat and greaves C3 | 0.0721 | 0.0783 | 0.0073 |
| Fat around heart | Fat and greaves C3 | 0.0029 | 0.0031 | 0.0003 |
| Fat in the kidney | Fat and greaves C3 | 0.0003 | 0.0004 | 0.0000 |
| Feet (without hooves) | Gelatin C3 | 0.0133 | 0.0202 | 0.0001 |
| Floatation fat | Spreading/Compost | 0.0000 | 0.0000 | 0.0000 |
| Forehead | C1-C2 for disposal | 0.0000 | 0.0000 | 0.0000 |
| Forelock | PAP C3 | 0.0030 | 0.0013 | 0.0001 |
| Gallbladder | Pet food | 0.0003 | 0.0005 | 0.0000 |
| Head trimmings | Pet food | 0.0029 | 0.0043 | 0.0003 |
| Heart | Human food | 0.0032 | 0.0050 | 0.0011 |
| Heart trimmings | Pet food | 0.0003 | 0.0005 | 0.0000 |
| Hide | Skin tannery C3 | 0.0737 | 0.0791 | 0.1346 |
| Hooves | PAP C3 | 0.0084 | 0.0035 | 0.0003 |
| Horns | PAP C3 | 0.0021 | 0.0009 | 0.0001 |
| Kidney | Human food | 0.0021 | 0.0031 | 0.0013 |
| Large intestine | C1-C2 for disposal | 0.0000 | 0.0000 | 0.0000 |
| Liver | Human food | 0.0613 | 0.0205 | 0.0101 |
| Liver trimmings | Pet food | 0.0071 | 0.0023 | 0.0002 |
| Lower jaw | PAP C3 | 0.0034 | 0.0056 | 0.0005 |
| Lungs | Pet food | 0.0088 | 0.0114 | 0.0009 |
| Mask | Skin tannery C3 | 0.0050 | 0.0054 | 0.0092 |
| Mesenteric fat | C1-C2 for disposal | 0.0000 | 0.0000 | 0.0000 |
| Muscle | Human food | 0.2942 | 0.4374 | 0.7457 |
| Muzzle | Human food | 0.0026 | 0.0028 | 0.0028 |
| Omasum | Human food | 0.0146 | 0.0025 | 0.0019 |
| Omasum fat | Fat and greaves C3 | 0.0035 | 0.0016 | 0.0002 |
| Rumen and forestomach | Human food | 0.0759 | 0.0131 | 0.0100 |
| Rumen fat | Fat and greaves C3 | 0.0055 | 0.0025 | 0.0002 |
| Sanitary seizures | C1-C2 for disposal | 0.0000 | 0.0000 | 0.0000 |
| Screening and sifting wastes | C1-C2 for disposal | 0.0000 | 0.0000 | 0.0000 |
| Small intestine | PAP C3 | 0.0883 | 0.0152 | 0.0009 |
| Spinal cord | C1-C2 for disposal | 0.0000 | 0.0000 | 0.0000 |
| Spinal cord waste | C1-C2 for disposal | 0.0000 | 0.0000 | 0.0000 |
| Spine | C1-C2 for disposal | 0.0000 | 0.0000 | 0.0000 |
| Spleen | Pet food | 0.0021 | 0.0031 | 0.0002 |
| Stillborn | PAP C3 | 0.0465 | 0.0077 | 0.0000 |
| Tallow | Fat and greaves C3 | 0.0720 | 0.0785 | 0.0073 |
| Tongue | Human food | 0.0026 | 0.0045 | 0.0073 |
| Tonsil | C1-C2 for disposal | 0.0000 | 0.0000 | 0.0000 |
| Trachea | Pet food | 0.0019 | 0.0020 | 0.0002 |
| Udder | Pet food | 0.0034 | 0.0240 | 0.0018 |
| Upper throat | Pet food | 0.0011 | 0.0016 | 0.0001 |
| Water in the rumen | Spreading/Compost | 0.0000 | 0.0000 | 0.0000 |

Table 80: Total weighting by coproducts for Rouge des Prés Beef reared in Pasture

| COPRODUCT | Destination | Rouge des Prés/beef/pasture | | |
| --- | --- | --- | --- | --- |
|  |  | **Biophysical Cumulative share** | **Mass Cumulative share** | **Economic Cumulative share** |
| Abomasum | Human food | 0.0167 | 0.0026 | 0.0020 |
| Abomasum fat | Fat and greaves C3 | 0.0011 | 0.0005 | 0.0000 |
| Aponeurosis | Human food | 0.0259 | 0.0341 | 0.0342 |
| Bile | PAP C3 | 0.0000 | 0.0001 | 0.0000 |
| Blood | PAP C3 | 0.0182 | 0.0311 | 0.0070 |
| Blood | Pet food | 0.0026 | 0.0045 | 0.0003 |
| Bones | Gelatin C3 | 0.0465 | 0.0764 | 0.0002 |
| Bones of head, brain, eyes and teeth | C1-C2 for disposal | 0.0000 | 0.0000 | 0.0000 |
| Cheek | Human food | 0.0011 | 0.0016 | 0.0035 |
| Cheek | Human food | 0.0020 | 0.0029 | 0.0063 |
| Cheek trimmings | Pet food | 0.0005 | 0.0007 | 0.0001 |
| Chops | Pet food | 0.0020 | 0.0021 | 0.0002 |
| Contents of intestines | Spreading/Compost | 0.0000 | 0.0000 | 0.0000 |
| Contents of the rumen | Spreading/Compost | 0.0000 | 0.0000 | 0.0000 |
| Ears | PAP C3 | 0.0008 | 0.0010 | 0.0001 |
| Esophagus | Pet food | 0.0010 | 0.0015 | 0.0001 |
| Fat | Fat and greaves C3 | 0.0645 | 0.0818 | 0.0074 |
| Fat around heart | Fat and greaves C3 | 0.0022 | 0.0029 | 0.0003 |
| Fat in the kidney | Fat and greaves C3 | 0.0003 | 0.0004 | 0.0000 |
| Feet (without hooves) | Gelatin C3 | 0.0126 | 0.0187 | 0.0001 |
| Floatation fat | Spreading/Compost | 0.0000 | 0.0000 | 0.0000 |
| Forehead | C1-C2 for disposal | 0.0000 | 0.0000 | 0.0000 |
| Forelock | PAP C3 | 0.0028 | 0.0011 | 0.0001 |
| Gallbladder | Pet food | 0.0003 | 0.0005 | 0.0000 |
| Head trimmings | Pet food | 0.0027 | 0.0040 | 0.0003 |
| Heart | Human food | 0.0031 | 0.0047 | 0.0010 |
| Heart trimmings | Pet food | 0.0003 | 0.0005 | 0.0000 |
| Hide | Skin tannery C3 | 0.0705 | 0.0733 | 0.1223 |
| Hooves | PAP C3 | 0.0080 | 0.0032 | 0.0003 |
| Horns | PAP C3 | 0.0019 | 0.0007 | 0.0001 |
| Kidney | Human food | 0.0019 | 0.0029 | 0.0012 |
| Large intestine | C1-C2 for disposal | 0.0000 | 0.0000 | 0.0000 |
| Liver | Human food | 0.0622 | 0.0190 | 0.0092 |
| Liver trimmings | Pet food | 0.0072 | 0.0021 | 0.0002 |
| Lower jaw | PAP C3 | 0.0031 | 0.0052 | 0.0004 |
| Lungs | Pet food | 0.0084 | 0.0105 | 0.0008 |
| Mask | Skin tannery C3 | 0.0048 | 0.0050 | 0.0083 |
| Mesenteric fat | C1-C2 for disposal | 0.0000 | 0.0000 | 0.0000 |
| Muscle | Human food | 0.3146 | 0.4569 | 0.7640 |
| Muzzle | Human food | 0.0025 | 0.0026 | 0.0026 |
| Omasum | Human food | 0.0151 | 0.0024 | 0.0018 |
| Omasum fat | Fat and greaves C3 | 0.0036 | 0.0016 | 0.0001 |
| Rumen and forestomach | Human food | 0.0780 | 0.0121 | 0.0091 |
| Rumen fat | Fat and greaves C3 | 0.0052 | 0.0024 | 0.0002 |
| Sanitary seizures | C1-C2 for disposal | 0.0000 | 0.0000 | 0.0000 |
| Screening and sifting wastes | C1-C2 for disposal | 0.0000 | 0.0000 | 0.0000 |
| Small intestine | PAP C3 | 0.0908 | 0.0141 | 0.0009 |
| Spinal cord | C1-C2 for disposal | 0.0000 | 0.0000 | 0.0000 |
| Spinal cord waste | C1-C2 for disposal | 0.0000 | 0.0000 | 0.0000 |
| Spine | C1-C2 for disposal | 0.0000 | 0.0000 | 0.0000 |
| Spleen | Pet food | 0.0020 | 0.0029 | 0.0002 |
| Stillborn | PAP C3 | 0.0475 | 0.0071 | 0.0000 |
| Tallow | Fat and greaves C3 | 0.0571 | 0.0727 | 0.0066 |
| Tongue | Human food | 0.0025 | 0.0042 | 0.0067 |
| Tonsil | C1-C2 for disposal | 0.0000 | 0.0000 | 0.0000 |
| Trachea | Pet food | 0.0018 | 0.0019 | 0.0001 |
| Udder | Pet food | 0.0030 | 0.0222 | 0.0016 |
| Upper throat | Pet food | 0.0011 | 0.0016 | 0.0001 |
| Water in the rumen | Spreading/Compost | 0.0000 | 0.0000 | 0.0000 |

Table 81: Total weighting by coproducts for Rouge des Prés Young Bulls reared in Stall

| COPRODUCT | Destination | Rouge des Prés/young bull/stall | | |
| --- | --- | --- | --- | --- |
|  |  | **Biophysical Cumulative share** | **Mass Cumulative share** | **Economic Cumulative share** |
| Abomasum | Human food | 0.0137 | 0.0025 | 0.0018 |
| Abomasum fat | Fat and greaves C3 | 0.0011 | 0.0005 | 0.0000 |
| Aponeurosis | Human food | 0.0264 | 0.0351 | 0.0348 |
| Bile | PAP C3 | 0.0000 | 0.0001 | 0.0000 |
| Blood | PAP C3 | 0.0172 | 0.0295 | 0.0065 |
| Blood | Pet food | 0.0024 | 0.0042 | 0.0003 |
| Bones | Gelatin C3 | 0.0514 | 0.0786 | 0.0002 |
| Bones of head, brain, eyes and teeth | C1-C2 for disposal | 0.0000 | 0.0000 | 0.0000 |
| Cheek | Human food | 0.0010 | 0.0015 | 0.0032 |
| Cheek | Human food | 0.0019 | 0.0027 | 0.0059 |
| Cheek trimmings | Pet food | 0.0005 | 0.0007 | 0.0001 |
| Chops | Pet food | 0.0019 | 0.0020 | 0.0001 |
| Contents of intestines | Spreading/Compost | 0.0000 | 0.0000 | 0.0000 |
| Contents of the rumen | Spreading/Compost | 0.0000 | 0.0000 | 0.0000 |
| Ears | PAP C3 | 0.0008 | 0.0010 | 0.0001 |
| Esophagus | Pet food | 0.0009 | 0.0014 | 0.0001 |
| Fat | Fat and greaves C3 | 0.0933 | 0.0841 | 0.0076 |
| Fat around heart | Fat and greaves C3 | 0.0030 | 0.0027 | 0.0002 |
| Fat in the kidney | Fat and greaves C3 | 0.0004 | 0.0004 | 0.0000 |
| Feet (without hooves) | Gelatin C3 | 0.0120 | 0.0177 | 0.0001 |
| Floatation fat | Spreading/Compost | 0.0000 | 0.0000 | 0.0000 |
| Forehead | C1-C2 for disposal | 0.0000 | 0.0000 | 0.0000 |
| Forelock | PAP C3 | 0.0027 | 0.0011 | 0.0001 |
| Gallbladder | Pet food | 0.0003 | 0.0005 | 0.0000 |
| Head trimmings | Pet food | 0.0025 | 0.0037 | 0.0003 |
| Heart | Human food | 0.0029 | 0.0044 | 0.0009 |
| Heart trimmings | Pet food | 0.0003 | 0.0005 | 0.0000 |
| Hide | Skin tannery C3 | 0.0663 | 0.0695 | 0.1146 |
| Hooves | PAP C3 | 0.0075 | 0.0031 | 0.0003 |
| Horns | PAP C3 | 0.0018 | 0.0007 | 0.0001 |
| Kidney | Human food | 0.0018 | 0.0027 | 0.0011 |
| Large intestine | C1-C2 for disposal | 0.0000 | 0.0000 | 0.0000 |
| Liver | Human food | 0.0519 | 0.0180 | 0.0086 |
| Liver trimmings | Pet food | 0.0059 | 0.0020 | 0.0001 |
| Lower jaw | PAP C3 | 0.0032 | 0.0049 | 0.0004 |
| Lungs | Pet food | 0.0079 | 0.0101 | 0.0007 |
| Mask | Skin tannery C3 | 0.0045 | 0.0047 | 0.0077 |
| Mesenteric fat | C1-C2 for disposal | 0.0000 | 0.0000 | 0.0000 |
| Muscle | Human food | 0.3259 | 0.4698 | 0.7759 |
| Muzzle | Human food | 0.0023 | 0.0025 | 0.0024 |
| Omasum | Human food | 0.0123 | 0.0022 | 0.0016 |
| Omasum fat | Fat and greaves C3 | 0.0033 | 0.0015 | 0.0001 |
| Rumen and forestomach | Human food | 0.0642 | 0.0116 | 0.0086 |
| Rumen fat | Fat and greaves C3 | 0.0050 | 0.0022 | 0.0002 |
| Sanitary seizures | C1-C2 for disposal | 0.0000 | 0.0000 | 0.0000 |
| Screening and sifting wastes | C1-C2 for disposal | 0.0000 | 0.0000 | 0.0000 |
| Small intestine | PAP C3 | 0.0744 | 0.0134 | 0.0008 |
| Spinal cord | C1-C2 for disposal | 0.0000 | 0.0000 | 0.0000 |
| Spinal cord waste | C1-C2 for disposal | 0.0000 | 0.0000 | 0.0000 |
| Spine | C1-C2 for disposal | 0.0000 | 0.0000 | 0.0000 |
| Spleen | Pet food | 0.0019 | 0.0027 | 0.0002 |
| Stillborn | PAP C3 | 0.0385 | 0.0066 | 0.0000 |
| Tallow | Fat and greaves C3 | 0.0763 | 0.0689 | 0.0062 |
| Tongue | Human food | 0.0024 | 0.0039 | 0.0062 |
| Tonsil | C1-C2 for disposal | 0.0000 | 0.0000 | 0.0000 |
| Trachea | Pet food | 0.0016 | 0.0017 | 0.0001 |
| Udder | Pet food | 0.0032 | 0.0210 | 0.0015 |
| Upper throat | Pet food | 0.0010 | 0.0015 | 0.0001 |
| Water in the rumen | Spreading/Compost | 0.0000 | 0.0000 | 0.0000 |

Table 82: Total weighting by coproducts for Rouge des Prés Heifers reared in Stall

| COPRODUCT | Destination | Rouge des Prés/heifer/stall | | |
| --- | --- | --- | --- | --- |
|  |  | **Biophysical Cumulative share** | **Mass Cumulative share** | **Economic Cumulative share** |
| Abomasum | Human food | 0.0148 | 0.0027 | 0.0021 |
| Abomasum fat | Fat and greaves C3 | 0.0011 | 0.0005 | 0.0000 |
| Aponeurosis | Human food | 0.0249 | 0.0336 | 0.0340 |
| Bile | PAP C3 | 0.0000 | 0.0001 | 0.0000 |
| Blood | PAP C3 | 0.0182 | 0.0319 | 0.0072 |
| Blood | Pet food | 0.0026 | 0.0046 | 0.0003 |
| Bones | Gelatin C3 | 0.0483 | 0.0753 | 0.0002 |
| Bones of head, brain, eyes and teeth | C1-C2 for disposal | 0.0000 | 0.0000 | 0.0000 |
| Cheek | Human food | 0.0011 | 0.0016 | 0.0036 |
| Cheek | Human food | 0.0020 | 0.0030 | 0.0066 |
| Cheek trimmings | Pet food | 0.0005 | 0.0007 | 0.0001 |
| Chops | Pet food | 0.0020 | 0.0021 | 0.0002 |
| Contents of intestines | Spreading/Compost | 0.0000 | 0.0000 | 0.0000 |
| Contents of the rumen | Spreading/Compost | 0.0000 | 0.0000 | 0.0000 |
| Ears | PAP C3 | 0.0009 | 0.0011 | 0.0001 |
| Esophagus | Pet food | 0.0010 | 0.0015 | 0.0001 |
| Fat | Fat and greaves C3 | 0.0876 | 0.0807 | 0.0074 |
| Fat around heart | Fat and greaves C3 | 0.0032 | 0.0030 | 0.0003 |
| Fat in the kidney | Fat and greaves C3 | 0.0004 | 0.0004 | 0.0000 |
| Feet (without hooves) | Gelatin C3 | 0.0128 | 0.0192 | 0.0001 |
| Floatation fat | Spreading/Compost | 0.0000 | 0.0000 | 0.0000 |
| Forehead | C1-C2 for disposal | 0.0000 | 0.0000 | 0.0000 |
| Forelock | PAP C3 | 0.0030 | 0.0012 | 0.0001 |
| Gallbladder | Pet food | 0.0003 | 0.0005 | 0.0000 |
| Head trimmings | Pet food | 0.0027 | 0.0040 | 0.0003 |
| Heart | Human food | 0.0031 | 0.0049 | 0.0010 |
| Heart trimmings | Pet food | 0.0003 | 0.0005 | 0.0000 |
| Hide | Skin tannery C3 | 0.0704 | 0.0752 | 0.1263 |
| Hooves | PAP C3 | 0.0081 | 0.0034 | 0.0003 |
| Horns | PAP C3 | 0.0018 | 0.0007 | 0.0001 |
| Kidney | Human food | 0.0020 | 0.0030 | 0.0012 |
| Large intestine | C1-C2 for disposal | 0.0000 | 0.0000 | 0.0000 |
| Liver | Human food | 0.0548 | 0.0194 | 0.0095 |
| Liver trimmings | Pet food | 0.0062 | 0.0021 | 0.0002 |
| Lower jaw | PAP C3 | 0.0034 | 0.0054 | 0.0005 |
| Lungs | Pet food | 0.0084 | 0.0108 | 0.0008 |
| Mask | Skin tannery C3 | 0.0048 | 0.0051 | 0.0086 |
| Mesenteric fat | C1-C2 for disposal | 0.0000 | 0.0000 | 0.0000 |
| Muscle | Human food | 0.3068 | 0.4504 | 0.7579 |
| Muzzle | Human food | 0.0026 | 0.0027 | 0.0028 |
| Omasum | Human food | 0.0128 | 0.0024 | 0.0018 |
| Omasum fat | Fat and greaves C3 | 0.0036 | 0.0016 | 0.0001 |
| Rumen and forestomach | Human food | 0.0674 | 0.0125 | 0.0094 |
| Rumen fat | Fat and greaves C3 | 0.0052 | 0.0024 | 0.0002 |
| Sanitary seizures | C1-C2 for disposal | 0.0000 | 0.0000 | 0.0000 |
| Screening and sifting wastes | C1-C2 for disposal | 0.0000 | 0.0000 | 0.0000 |
| Small intestine | PAP C3 | 0.0782 | 0.0144 | 0.0009 |
| Spinal cord | C1-C2 for disposal | 0.0000 | 0.0000 | 0.0000 |
| Spinal cord waste | C1-C2 for disposal | 0.0000 | 0.0000 | 0.0000 |
| Spine | C1-C2 for disposal | 0.0000 | 0.0000 | 0.0000 |
| Spleen | Pet food | 0.0020 | 0.0030 | 0.0002 |
| Stillborn | PAP C3 | 0.0408 | 0.0072 | 0.0000 |
| Tallow | Fat and greaves C3 | 0.0808 | 0.0746 | 0.0068 |
| Tongue | Human food | 0.0026 | 0.0042 | 0.0068 |
| Tonsil | C1-C2 for disposal | 0.0000 | 0.0000 | 0.0000 |
| Trachea | Pet food | 0.0018 | 0.0019 | 0.0001 |
| Udder | Pet food | 0.0034 | 0.0228 | 0.0017 |
| Upper throat | Pet food | 0.0011 | 0.0016 | 0.0001 |
| Water in the rumen | Spreading/Compost | 0.0000 | 0.0000 | 0.0000 |

Table 83: Total weighting by coproducts for Rouge des Prés Cull Cows reared in Stall

| COPRODUCT | Destination | Rouge des Prés/Cull cow/stall | | |
| --- | --- | --- | --- | --- |
|  |  | **Biophysical Cumulative share** | **Mass Cumulative share** | **Economic Cumulative share** |
| Abomasum | Human food | 0.0154 | 0.0028 | 0.0021 |
| Abomasum fat | Fat and greaves C3 | 0.0011 | 0.0005 | 0.0000 |
| Aponeurosis | Human food | 0.0238 | 0.0326 | 0.0334 |
| Bile | PAP C3 | 0.0000 | 0.0001 | 0.0000 |
| Blood | PAP C3 | 0.0188 | 0.0335 | 0.0076 |
| Blood | Pet food | 0.0027 | 0.0048 | 0.0004 |
| Bones | Gelatin C3 | 0.0457 | 0.0732 | 0.0002 |
| Bones of head, brain, eyes and teeth | C1-C2 for disposal | 0.0000 | 0.0000 | 0.0000 |
| Cheek | Human food | 0.0011 | 0.0016 | 0.0037 |
| Cheek | Human food | 0.0021 | 0.0031 | 0.0070 |
| Cheek trimmings | Pet food | 0.0006 | 0.0009 | 0.0001 |
| Chops | Pet food | 0.0021 | 0.0023 | 0.0002 |
| Contents of intestines | Spreading/Compost | 0.0000 | 0.0000 | 0.0000 |
| Contents of the rumen | Spreading/Compost | 0.0000 | 0.0000 | 0.0000 |
| Ears | PAP C3 | 0.0009 | 0.0011 | 0.0001 |
| Esophagus | Pet food | 0.0010 | 0.0015 | 0.0001 |
| Fat | Fat and greaves C3 | 0.0796 | 0.0783 | 0.0073 |
| Fat around heart | Fat and greaves C3 | 0.0032 | 0.0031 | 0.0003 |
| Fat in the kidney | Fat and greaves C3 | 0.0004 | 0.0004 | 0.0000 |
| Feet (without hooves) | Gelatin C3 | 0.0132 | 0.0202 | 0.0001 |
| Floatation fat | Spreading/Compost | 0.0000 | 0.0000 | 0.0000 |
| Forehead | C1-C2 for disposal | 0.0000 | 0.0000 | 0.0000 |
| Forelock | PAP C3 | 0.0030 | 0.0013 | 0.0001 |
| Gallbladder | Pet food | 0.0003 | 0.0005 | 0.0000 |
| Head trimmings | Pet food | 0.0028 | 0.0043 | 0.0003 |
| Heart | Human food | 0.0032 | 0.0050 | 0.0011 |
| Heart trimmings | Pet food | 0.0003 | 0.0005 | 0.0000 |
| Hide | Skin tannery C3 | 0.0729 | 0.0791 | 0.1346 |
| Hooves | PAP C3 | 0.0083 | 0.0035 | 0.0003 |
| Horns | PAP C3 | 0.0021 | 0.0009 | 0.0001 |
| Kidney | Human food | 0.0020 | 0.0031 | 0.0013 |
| Large intestine | C1-C2 for disposal | 0.0000 | 0.0000 | 0.0000 |
| Liver | Human food | 0.0590 | 0.0205 | 0.0101 |
| Liver trimmings | Pet food | 0.0068 | 0.0023 | 0.0002 |
| Lower jaw | PAP C3 | 0.0035 | 0.0056 | 0.0005 |
| Lungs | Pet food | 0.0087 | 0.0114 | 0.0009 |
| Mask | Skin tannery C3 | 0.0050 | 0.0054 | 0.0092 |
| Mesenteric fat | C1-C2 for disposal | 0.0000 | 0.0000 | 0.0000 |
| Muscle | Human food | 0.2926 | 0.4374 | 0.7457 |
| Muzzle | Human food | 0.0025 | 0.0028 | 0.0028 |
| Omasum | Human food | 0.0140 | 0.0025 | 0.0019 |
| Omasum fat | Fat and greaves C3 | 0.0036 | 0.0016 | 0.0002 |
| Rumen and forestomach | Human food | 0.0726 | 0.0131 | 0.0100 |
| Rumen fat | Fat and greaves C3 | 0.0055 | 0.0025 | 0.0002 |
| Sanitary seizures | C1-C2 for disposal | 0.0000 | 0.0000 | 0.0000 |
| Screening and sifting wastes | C1-C2 for disposal | 0.0000 | 0.0000 | 0.0000 |
| Small intestine | PAP C3 | 0.0845 | 0.0152 | 0.0009 |
| Spinal cord | C1-C2 for disposal | 0.0000 | 0.0000 | 0.0000 |
| Spinal cord waste | C1-C2 for disposal | 0.0000 | 0.0000 | 0.0000 |
| Spine | C1-C2 for disposal | 0.0000 | 0.0000 | 0.0000 |
| Spleen | Pet food | 0.0021 | 0.0031 | 0.0002 |
| Stillborn | PAP C3 | 0.0445 | 0.0077 | 0.0000 |
| Tallow | Fat and greaves C3 | 0.0796 | 0.0785 | 0.0073 |
| Tongue | Human food | 0.0027 | 0.0045 | 0.0073 |
| Tonsil | C1-C2 for disposal | 0.0000 | 0.0000 | 0.0000 |
| Trachea | Pet food | 0.0019 | 0.0020 | 0.0002 |
| Udder | Pet food | 0.0035 | 0.0240 | 0.0018 |
| Upper throat | Pet food | 0.0011 | 0.0016 | 0.0001 |
| Water in the rumen | Spreading/Compost | 0.0000 | 0.0000 | 0.0000 |

Table 84: Total weighting by coproducts for Rouge des Prés Beef reared in Stall

| COPRODUCT | Destination | Rouge des Prés/beef/stall | | |
| --- | --- | --- | --- | --- |
|  |  | **Biophysical Cumulative share** | **Mass Cumulative share** | **Economic Cumulative share** |
| Abomasum | Human food | 0.0161 | 0.0026 | 0.0020 |
| Abomasum fat | Fat and greaves C3 | 0.0011 | 0.0005 | 0.0000 |
| Aponeurosis | Human food | 0.0256 | 0.0341 | 0.0342 |
| Bile | PAP C3 | 0.0000 | 0.0001 | 0.0000 |
| Blood | PAP C3 | 0.0180 | 0.0311 | 0.0070 |
| Blood | Pet food | 0.0026 | 0.0045 | 0.0003 |
| Bones | Gelatin C3 | 0.0471 | 0.0764 | 0.0002 |
| Bones of head, brain, eyes and teeth | C1-C2 for disposal | 0.0000 | 0.0000 | 0.0000 |
| Cheek | Human food | 0.0011 | 0.0016 | 0.0035 |
| Cheek | Human food | 0.0019 | 0.0029 | 0.0063 |
| Cheek trimmings | Pet food | 0.0005 | 0.0007 | 0.0001 |
| Chops | Pet food | 0.0020 | 0.0021 | 0.0002 |
| Contents of intestines | Spreading/Compost | 0.0000 | 0.0000 | 0.0000 |
| Contents of the rumen | Spreading/Compost | 0.0000 | 0.0000 | 0.0000 |
| Ears | PAP C3 | 0.0008 | 0.0010 | 0.0001 |
| Esophagus | Pet food | 0.0010 | 0.0015 | 0.0001 |
| Fat | Fat and greaves C3 | 0.0718 | 0.0818 | 0.0074 |
| Fat around heart | Fat and greaves C3 | 0.0025 | 0.0029 | 0.0003 |
| Fat in the kidney | Fat and greaves C3 | 0.0003 | 0.0004 | 0.0000 |
| Feet (without hooves) | Gelatin C3 | 0.0125 | 0.0187 | 0.0001 |
| Floatation fat | Spreading/Compost | 0.0000 | 0.0000 | 0.0000 |
| Forehead | C1-C2 for disposal | 0.0000 | 0.0000 | 0.0000 |
| Forelock | PAP C3 | 0.0027 | 0.0011 | 0.0001 |
| Gallbladder | Pet food | 0.0003 | 0.0005 | 0.0000 |
| Head trimmings | Pet food | 0.0027 | 0.0040 | 0.0003 |
| Heart | Human food | 0.0031 | 0.0047 | 0.0010 |
| Heart trimmings | Pet food | 0.0003 | 0.0005 | 0.0000 |
| Hide | Skin tannery C3 | 0.0696 | 0.0733 | 0.1223 |
| Hooves | PAP C3 | 0.0079 | 0.0032 | 0.0003 |
| Horns | PAP C3 | 0.0018 | 0.0007 | 0.0001 |
| Kidney | Human food | 0.0019 | 0.0029 | 0.0012 |
| Large intestine | C1-C2 for disposal | 0.0000 | 0.0000 | 0.0000 |
| Liver | Human food | 0.0603 | 0.0190 | 0.0092 |
| Liver trimmings | Pet food | 0.0070 | 0.0021 | 0.0002 |
| Lower jaw | PAP C3 | 0.0031 | 0.0052 | 0.0004 |
| Lungs | Pet food | 0.0083 | 0.0105 | 0.0008 |
| Mask | Skin tannery C3 | 0.0047 | 0.0050 | 0.0083 |
| Mesenteric fat | C1-C2 for disposal | 0.0000 | 0.0000 | 0.0000 |
| Muscle | Human food | 0.3125 | 0.4569 | 0.7640 |
| Muzzle | Human food | 0.0025 | 0.0026 | 0.0026 |
| Omasum | Human food | 0.0146 | 0.0024 | 0.0018 |
| Omasum fat | Fat and greaves C3 | 0.0036 | 0.0016 | 0.0001 |
| Rumen and forestomach | Human food | 0.0753 | 0.0121 | 0.0091 |
| Rumen fat | Fat and greaves C3 | 0.0053 | 0.0024 | 0.0002 |
| Sanitary seizures | C1-C2 for disposal | 0.0000 | 0.0000 | 0.0000 |
| Screening and sifting wastes | C1-C2 for disposal | 0.0000 | 0.0000 | 0.0000 |
| Small intestine | PAP C3 | 0.0876 | 0.0141 | 0.0009 |
| Spinal cord | C1-C2 for disposal | 0.0000 | 0.0000 | 0.0000 |
| Spinal cord waste | C1-C2 for disposal | 0.0000 | 0.0000 | 0.0000 |
| Spine | C1-C2 for disposal | 0.0000 | 0.0000 | 0.0000 |
| Spleen | Pet food | 0.0020 | 0.0029 | 0.0002 |
| Stillborn | PAP C3 | 0.0458 | 0.0071 | 0.0000 |
| Tallow | Fat and greaves C3 | 0.0636 | 0.0727 | 0.0066 |
| Tongue | Human food | 0.0025 | 0.0042 | 0.0067 |
| Tonsil | C1-C2 for disposal | 0.0000 | 0.0000 | 0.0000 |
| Trachea | Pet food | 0.0018 | 0.0019 | 0.0001 |
| Udder | Pet food | 0.0031 | 0.0222 | 0.0016 |
| Upper throat | Pet food | 0.0011 | 0.0016 | 0.0001 |
| Water in the rumen | Spreading/Compost | 0.0000 | 0.0000 | 0.0000 |

Table 85: Total weighting by coproducts for Charolaise x Rustique Young Bulls reared in Grazing Large Area

| COPRODUCT | Destination | Charolaise x Rustique/young bull/grazing large area | | |
| --- | --- | --- | --- | --- |
|  |  | **Biophysical Cumulative share** | **Mass Cumulative share** | **Economic Cumulative share** |
| Abomasum | Human food | 0.0147 | 0.0025 | 0.0018 |
| Abomasum fat | Fat and greaves C3 | 0.0011 | 0.0005 | 0.0000 |
| Aponeurosis | Human food | 0.0271 | 0.0351 | 0.0348 |
| Bile | PAP C3 | 0.0000 | 0.0001 | 0.0000 |
| Blood | PAP C3 | 0.0176 | 0.0295 | 0.0065 |
| Blood | Pet food | 0.0025 | 0.0042 | 0.0003 |
| Bones | Gelatin C3 | 0.0503 | 0.0786 | 0.0002 |
| Bones of head, brain, eyes and teeth | C1-C2 for disposal | 0.0000 | 0.0000 | 0.0000 |
| Cheek | Human food | 0.0010 | 0.0015 | 0.0032 |
| Cheek | Human food | 0.0019 | 0.0027 | 0.0059 |
| Cheek trimmings | Pet food | 0.0005 | 0.0007 | 0.0001 |
| Chops | Pet food | 0.0019 | 0.0020 | 0.0001 |
| Contents of intestines | Spreading/Compost | 0.0000 | 0.0000 | 0.0000 |
| Contents of the rumen | Spreading/Compost | 0.0000 | 0.0000 | 0.0000 |
| Ears | PAP C3 | 0.0009 | 0.0010 | 0.0001 |
| Esophagus | Pet food | 0.0009 | 0.0014 | 0.0001 |
| Fat | Fat and greaves C3 | 0.0790 | 0.0841 | 0.0076 |
| Fat around heart | Fat and greaves C3 | 0.0025 | 0.0027 | 0.0002 |
| Fat in the kidney | Fat and greaves C3 | 0.0003 | 0.0004 | 0.0000 |
| Feet (without hooves) | Gelatin C3 | 0.0122 | 0.0177 | 0.0001 |
| Floatation fat | Spreading/Compost | 0.0000 | 0.0000 | 0.0000 |
| Forehead | C1-C2 for disposal | 0.0000 | 0.0000 | 0.0000 |
| Forelock | PAP C3 | 0.0028 | 0.0011 | 0.0001 |
| Gallbladder | Pet food | 0.0003 | 0.0005 | 0.0000 |
| Head trimmings | Pet food | 0.0026 | 0.0037 | 0.0003 |
| Heart | Human food | 0.0030 | 0.0044 | 0.0009 |
| Heart trimmings | Pet food | 0.0003 | 0.0005 | 0.0000 |
| Hide | Skin tannery C3 | 0.0680 | 0.0695 | 0.1146 |
| Hooves | PAP C3 | 0.0078 | 0.0031 | 0.0003 |
| Horns | PAP C3 | 0.0019 | 0.0007 | 0.0001 |
| Kidney | Human food | 0.0019 | 0.0027 | 0.0011 |
| Large intestine | C1-C2 for disposal | 0.0000 | 0.0000 | 0.0000 |
| Liver | Human food | 0.0554 | 0.0180 | 0.0086 |
| Liver trimmings | Pet food | 0.0063 | 0.0020 | 0.0001 |
| Lower jaw | PAP C3 | 0.0031 | 0.0049 | 0.0004 |
| Lungs | Pet food | 0.0082 | 0.0101 | 0.0007 |
| Mask | Skin tannery C3 | 0.0046 | 0.0047 | 0.0077 |
| Mesenteric fat | C1-C2 for disposal | 0.0000 | 0.0000 | 0.0000 |
| Muscle | Human food | 0.3308 | 0.4698 | 0.7759 |
| Muzzle | Human food | 0.0024 | 0.0025 | 0.0024 |
| Omasum | Human food | 0.0132 | 0.0022 | 0.0016 |
| Omasum fat | Fat and greaves C3 | 0.0033 | 0.0015 | 0.0001 |
| Rumen and forestomach | Human food | 0.0690 | 0.0116 | 0.0086 |
| Rumen fat | Fat and greaves C3 | 0.0049 | 0.0022 | 0.0002 |
| Sanitary seizures | C1-C2 for disposal | 0.0000 | 0.0000 | 0.0000 |
| Screening and sifting wastes | C1-C2 for disposal | 0.0000 | 0.0000 | 0.0000 |
| Small intestine | PAP C3 | 0.0800 | 0.0134 | 0.0008 |
| Spinal cord | C1-C2 for disposal | 0.0000 | 0.0000 | 0.0000 |
| Spinal cord waste | C1-C2 for disposal | 0.0000 | 0.0000 | 0.0000 |
| Spine | C1-C2 for disposal | 0.0000 | 0.0000 | 0.0000 |
| Spleen | Pet food | 0.0019 | 0.0027 | 0.0002 |
| Stillborn | PAP C3 | 0.0414 | 0.0066 | 0.0000 |
| Tallow | Fat and greaves C3 | 0.0646 | 0.0689 | 0.0062 |
| Tongue | Human food | 0.0024 | 0.0039 | 0.0062 |
| Tonsil | C1-C2 for disposal | 0.0000 | 0.0000 | 0.0000 |
| Trachea | Pet food | 0.0017 | 0.0017 | 0.0001 |
| Udder | Pet food | 0.0031 | 0.0210 | 0.0015 |
| Upper throat | Pet food | 0.0010 | 0.0015 | 0.0001 |
| Water in the rumen | Spreading/Compost | 0.0000 | 0.0000 | 0.0000 |

Table 86: Total weighting by coproducts for Charolaise x Rustique Heifers reared in Grazing Large Area

| COPRODUCT | Destination | Charolaise x Rustique/heifer/grazing large area | | |
| --- | --- | --- | --- | --- |
|  |  | **Biophysical Cumulative share** | **Mass Cumulative share** | **Economic Cumulative share** |
| Abomasum | Human food | 0.0156 | 0.0027 | 0.0021 |
| Abomasum fat | Fat and greaves C3 | 0.0011 | 0.0005 | 0.0000 |
| Aponeurosis | Human food | 0.0256 | 0.0336 | 0.0340 |
| Bile | PAP C3 | 0.0000 | 0.0001 | 0.0000 |
| Blood | PAP C3 | 0.0187 | 0.0319 | 0.0072 |
| Blood | Pet food | 0.0027 | 0.0046 | 0.0003 |
| Bones | Gelatin C3 | 0.0476 | 0.0753 | 0.0002 |
| Bones of head, brain, eyes and teeth | C1-C2 for disposal | 0.0000 | 0.0000 | 0.0000 |
| Cheek | Human food | 0.0011 | 0.0016 | 0.0036 |
| Cheek | Human food | 0.0021 | 0.0030 | 0.0066 |
| Cheek trimmings | Pet food | 0.0005 | 0.0007 | 0.0001 |
| Chops | Pet food | 0.0020 | 0.0021 | 0.0002 |
| Contents of intestines | Spreading/Compost | 0.0000 | 0.0000 | 0.0000 |
| Contents of the rumen | Spreading/Compost | 0.0000 | 0.0000 | 0.0000 |
| Ears | PAP C3 | 0.0010 | 0.0011 | 0.0001 |
| Esophagus | Pet food | 0.0010 | 0.0015 | 0.0001 |
| Fat | Fat and greaves C3 | 0.0752 | 0.0807 | 0.0074 |
| Fat around heart | Fat and greaves C3 | 0.0028 | 0.0030 | 0.0003 |
| Fat in the kidney | Fat and greaves C3 | 0.0003 | 0.0004 | 0.0000 |
| Feet (without hooves) | Gelatin C3 | 0.0130 | 0.0192 | 0.0001 |
| Floatation fat | Spreading/Compost | 0.0000 | 0.0000 | 0.0000 |
| Forehead | C1-C2 for disposal | 0.0000 | 0.0000 | 0.0000 |
| Forelock | PAP C3 | 0.0031 | 0.0012 | 0.0001 |
| Gallbladder | Pet food | 0.0003 | 0.0005 | 0.0000 |
| Head trimmings | Pet food | 0.0028 | 0.0040 | 0.0003 |
| Heart | Human food | 0.0032 | 0.0049 | 0.0010 |
| Heart trimmings | Pet food | 0.0003 | 0.0005 | 0.0000 |
| Hide | Skin tannery C3 | 0.0726 | 0.0752 | 0.1263 |
| Hooves | PAP C3 | 0.0084 | 0.0034 | 0.0003 |
| Horns | PAP C3 | 0.0019 | 0.0007 | 0.0001 |
| Kidney | Human food | 0.0020 | 0.0030 | 0.0012 |
| Large intestine | C1-C2 for disposal | 0.0000 | 0.0000 | 0.0000 |
| Liver | Human food | 0.0574 | 0.0194 | 0.0095 |
| Liver trimmings | Pet food | 0.0065 | 0.0021 | 0.0002 |
| Lower jaw | PAP C3 | 0.0033 | 0.0054 | 0.0005 |
| Lungs | Pet food | 0.0086 | 0.0108 | 0.0008 |
| Mask | Skin tannery C3 | 0.0049 | 0.0051 | 0.0086 |
| Mesenteric fat | C1-C2 for disposal | 0.0000 | 0.0000 | 0.0000 |
| Muscle | Human food | 0.3131 | 0.4504 | 0.7579 |
| Muzzle | Human food | 0.0026 | 0.0027 | 0.0028 |
| Omasum | Human food | 0.0135 | 0.0024 | 0.0018 |
| Omasum fat | Fat and greaves C3 | 0.0035 | 0.0016 | 0.0001 |
| Rumen and forestomach | Human food | 0.0709 | 0.0125 | 0.0094 |
| Rumen fat | Fat and greaves C3 | 0.0051 | 0.0024 | 0.0002 |
| Sanitary seizures | C1-C2 for disposal | 0.0000 | 0.0000 | 0.0000 |
| Screening and sifting wastes | C1-C2 for disposal | 0.0000 | 0.0000 | 0.0000 |
| Small intestine | PAP C3 | 0.0822 | 0.0144 | 0.0009 |
| Spinal cord | C1-C2 for disposal | 0.0000 | 0.0000 | 0.0000 |
| Spinal cord waste | C1-C2 for disposal | 0.0000 | 0.0000 | 0.0000 |
| Spine | C1-C2 for disposal | 0.0000 | 0.0000 | 0.0000 |
| Spleen | Pet food | 0.0021 | 0.0030 | 0.0002 |
| Stillborn | PAP C3 | 0.0429 | 0.0072 | 0.0000 |
| Tallow | Fat and greaves C3 | 0.0694 | 0.0746 | 0.0068 |
| Tongue | Human food | 0.0026 | 0.0042 | 0.0068 |
| Tonsil | C1-C2 for disposal | 0.0000 | 0.0000 | 0.0000 |
| Trachea | Pet food | 0.0018 | 0.0019 | 0.0001 |
| Udder | Pet food | 0.0033 | 0.0228 | 0.0017 |
| Upper throat | Pet food | 0.0011 | 0.0016 | 0.0001 |
| Water in the rumen | Spreading/Compost | 0.0000 | 0.0000 | 0.0000 |

Table 87: Total weighting by coproducts for Charolaise x Rustique Cull Cows reared in Grazing Large Area

| COPRODUCT | Destination | Charolaise x Rustique/Cull cow/grazing large area | | |
| --- | --- | --- | --- | --- |
|  |  | **Biophysical Cumulative share** | **Mass Cumulative share** | **Economic Cumulative share** |
| Abomasum | Human food | 0.0158 | 0.0028 | 0.0021 |
| Abomasum fat | Fat and greaves C3 | 0.0011 | 0.0005 | 0.0000 |
| Aponeurosis | Human food | 0.0244 | 0.0326 | 0.0334 |
| Bile | PAP C3 | 0.0000 | 0.0001 | 0.0000 |
| Blood | PAP C3 | 0.0193 | 0.0335 | 0.0076 |
| Blood | Pet food | 0.0028 | 0.0048 | 0.0004 |
| Bones | Gelatin C3 | 0.0453 | 0.0732 | 0.0002 |
| Bones of head, brain, eyes and teeth | C1-C2 for disposal | 0.0000 | 0.0000 | 0.0000 |
| Cheek | Human food | 0.0011 | 0.0016 | 0.0037 |
| Cheek | Human food | 0.0021 | 0.0031 | 0.0070 |
| Cheek trimmings | Pet food | 0.0006 | 0.0009 | 0.0001 |
| Chops | Pet food | 0.0021 | 0.0023 | 0.0002 |
| Contents of intestines | Spreading/Compost | 0.0000 | 0.0000 | 0.0000 |
| Contents of the rumen | Spreading/Compost | 0.0000 | 0.0000 | 0.0000 |
| Ears | PAP C3 | 0.0009 | 0.0011 | 0.0001 |
| Esophagus | Pet food | 0.0010 | 0.0015 | 0.0001 |
| Fat | Fat and greaves C3 | 0.0708 | 0.0783 | 0.0073 |
| Fat around heart | Fat and greaves C3 | 0.0028 | 0.0031 | 0.0003 |
| Fat in the kidney | Fat and greaves C3 | 0.0003 | 0.0004 | 0.0000 |
| Feet (without hooves) | Gelatin C3 | 0.0135 | 0.0202 | 0.0001 |
| Floatation fat | Spreading/Compost | 0.0000 | 0.0000 | 0.0000 |
| Forehead | C1-C2 for disposal | 0.0000 | 0.0000 | 0.0000 |
| Forelock | PAP C3 | 0.0031 | 0.0013 | 0.0001 |
| Gallbladder | Pet food | 0.0003 | 0.0005 | 0.0000 |
| Head trimmings | Pet food | 0.0029 | 0.0043 | 0.0003 |
| Heart | Human food | 0.0033 | 0.0050 | 0.0011 |
| Heart trimmings | Pet food | 0.0003 | 0.0005 | 0.0000 |
| Hide | Skin tannery C3 | 0.0750 | 0.0791 | 0.1346 |
| Hooves | PAP C3 | 0.0086 | 0.0035 | 0.0003 |
| Horns | PAP C3 | 0.0022 | 0.0009 | 0.0001 |
| Kidney | Human food | 0.0021 | 0.0031 | 0.0013 |
| Large intestine | C1-C2 for disposal | 0.0000 | 0.0000 | 0.0000 |
| Liver | Human food | 0.0605 | 0.0205 | 0.0101 |
| Liver trimmings | Pet food | 0.0070 | 0.0023 | 0.0002 |
| Lower jaw | PAP C3 | 0.0034 | 0.0056 | 0.0005 |
| Lungs | Pet food | 0.0090 | 0.0114 | 0.0009 |
| Mask | Skin tannery C3 | 0.0051 | 0.0054 | 0.0092 |
| Mesenteric fat | C1-C2 for disposal | 0.0000 | 0.0000 | 0.0000 |
| Muscle | Human food | 0.2984 | 0.4374 | 0.7457 |
| Muzzle | Human food | 0.0026 | 0.0028 | 0.0028 |
| Omasum | Human food | 0.0143 | 0.0025 | 0.0019 |
| Omasum fat | Fat and greaves C3 | 0.0035 | 0.0016 | 0.0002 |
| Rumen and forestomach | Human food | 0.0746 | 0.0131 | 0.0100 |
| Rumen fat | Fat and greaves C3 | 0.0054 | 0.0025 | 0.0002 |
| Sanitary seizures | C1-C2 for disposal | 0.0000 | 0.0000 | 0.0000 |
| Screening and sifting wastes | C1-C2 for disposal | 0.0000 | 0.0000 | 0.0000 |
| Small intestine | PAP C3 | 0.0867 | 0.0152 | 0.0009 |
| Spinal cord | C1-C2 for disposal | 0.0000 | 0.0000 | 0.0000 |
| Spinal cord waste | C1-C2 for disposal | 0.0000 | 0.0000 | 0.0000 |
| Spine | C1-C2 for disposal | 0.0000 | 0.0000 | 0.0000 |
| Spleen | Pet food | 0.0022 | 0.0031 | 0.0002 |
| Stillborn | PAP C3 | 0.0457 | 0.0077 | 0.0000 |
| Tallow | Fat and greaves C3 | 0.0708 | 0.0785 | 0.0073 |
| Tongue | Human food | 0.0027 | 0.0045 | 0.0073 |
| Tonsil | C1-C2 for disposal | 0.0000 | 0.0000 | 0.0000 |
| Trachea | Pet food | 0.0019 | 0.0020 | 0.0002 |
| Udder | Pet food | 0.0034 | 0.0240 | 0.0018 |
| Upper throat | Pet food | 0.0011 | 0.0016 | 0.0001 |
| Water in the rumen | Spreading/Compost | 0.0000 | 0.0000 | 0.0000 |

Table 88: Total weighting by coproducts for Charolaise x Rustique Beef reared in Grazing Large Area

| COPRODUCT | Destination | Charolaise x Rustique/beef/grazing large area | | |
| --- | --- | --- | --- | --- |
|  |  | **Biophysical Cumulative share** | **Mass Cumulative share** | **Economic Cumulative share** |
| Abomasum | Human food | 0.0154 | 0.0026 | 0.0020 |
| Abomasum fat | Fat and greaves C3 | 0.0011 | 0.0005 | 0.0000 |
| Aponeurosis | Human food | 0.0260 | 0.0341 | 0.0342 |
| Bile | PAP C3 | 0.0000 | 0.0001 | 0.0000 |
| Blood | PAP C3 | 0.0182 | 0.0311 | 0.0070 |
| Blood | Pet food | 0.0026 | 0.0045 | 0.0003 |
| Bones | Gelatin C3 | 0.0480 | 0.0764 | 0.0002 |
| Bones of head, brain, eyes and teeth | C1-C2 for disposal | 0.0000 | 0.0000 | 0.0000 |
| Cheek | Human food | 0.0011 | 0.0016 | 0.0035 |
| Cheek | Human food | 0.0020 | 0.0029 | 0.0063 |
| Cheek trimmings | Pet food | 0.0005 | 0.0007 | 0.0001 |
| Chops | Pet food | 0.0020 | 0.0021 | 0.0002 |
| Contents of intestines | Spreading/Compost | 0.0000 | 0.0000 | 0.0000 |
| Contents of the rumen | Spreading/Compost | 0.0000 | 0.0000 | 0.0000 |
| Ears | PAP C3 | 0.0008 | 0.0010 | 0.0001 |
| Esophagus | Pet food | 0.0010 | 0.0015 | 0.0001 |
| Fat | Fat and greaves C3 | 0.0748 | 0.0818 | 0.0074 |
| Fat around heart | Fat and greaves C3 | 0.0026 | 0.0029 | 0.0003 |
| Fat in the kidney | Fat and greaves C3 | 0.0003 | 0.0004 | 0.0000 |
| Feet (without hooves) | Gelatin C3 | 0.0127 | 0.0187 | 0.0001 |
| Floatation fat | Spreading/Compost | 0.0000 | 0.0000 | 0.0000 |
| Forehead | C1-C2 for disposal | 0.0000 | 0.0000 | 0.0000 |
| Forelock | PAP C3 | 0.0028 | 0.0011 | 0.0001 |
| Gallbladder | Pet food | 0.0003 | 0.0005 | 0.0000 |
| Head trimmings | Pet food | 0.0027 | 0.0040 | 0.0003 |
| Heart | Human food | 0.0031 | 0.0047 | 0.0010 |
| Heart trimmings | Pet food | 0.0003 | 0.0005 | 0.0000 |
| Hide | Skin tannery C3 | 0.0706 | 0.0733 | 0.1223 |
| Hooves | PAP C3 | 0.0080 | 0.0032 | 0.0003 |
| Horns | PAP C3 | 0.0018 | 0.0007 | 0.0001 |
| Kidney | Human food | 0.0019 | 0.0029 | 0.0012 |
| Large intestine | C1-C2 for disposal | 0.0000 | 0.0000 | 0.0000 |
| Liver | Human food | 0.0580 | 0.0190 | 0.0092 |
| Liver trimmings | Pet food | 0.0067 | 0.0021 | 0.0002 |
| Lower jaw | PAP C3 | 0.0032 | 0.0052 | 0.0004 |
| Lungs | Pet food | 0.0084 | 0.0105 | 0.0008 |
| Mask | Skin tannery C3 | 0.0048 | 0.0050 | 0.0083 |
| Mesenteric fat | C1-C2 for disposal | 0.0000 | 0.0000 | 0.0000 |
| Muscle | Human food | 0.3168 | 0.4569 | 0.7640 |
| Muzzle | Human food | 0.0025 | 0.0026 | 0.0026 |
| Omasum | Human food | 0.0139 | 0.0024 | 0.0018 |
| Omasum fat | Fat and greaves C3 | 0.0035 | 0.0016 | 0.0001 |
| Rumen and forestomach | Human food | 0.0719 | 0.0121 | 0.0091 |
| Rumen fat | Fat and greaves C3 | 0.0052 | 0.0024 | 0.0002 |
| Sanitary seizures | C1-C2 for disposal | 0.0000 | 0.0000 | 0.0000 |
| Screening and sifting wastes | C1-C2 for disposal | 0.0000 | 0.0000 | 0.0000 |
| Small intestine | PAP C3 | 0.0837 | 0.0141 | 0.0009 |
| Spinal cord | C1-C2 for disposal | 0.0000 | 0.0000 | 0.0000 |
| Spinal cord waste | C1-C2 for disposal | 0.0000 | 0.0000 | 0.0000 |
| Spine | C1-C2 for disposal | 0.0000 | 0.0000 | 0.0000 |
| Spleen | Pet food | 0.0020 | 0.0029 | 0.0002 |
| Stillborn | PAP C3 | 0.0437 | 0.0071 | 0.0000 |
| Tallow | Fat and greaves C3 | 0.0663 | 0.0727 | 0.0066 |
| Tongue | Human food | 0.0025 | 0.0042 | 0.0067 |
| Tonsil | C1-C2 for disposal | 0.0000 | 0.0000 | 0.0000 |
| Trachea | Pet food | 0.0018 | 0.0019 | 0.0001 |
| Udder | Pet food | 0.0032 | 0.0222 | 0.0016 |
| Upper throat | Pet food | 0.0011 | 0.0016 | 0.0001 |
| Water in the rumen | Spreading/Compost | 0.0000 | 0.0000 | 0.0000 |

Table 89: Total weighting by coproducts for Charolaise x Rustique Young Bulls reared in Pasture

| COPRODUCT | Destination | Charolaise x Rustique/young bull/pasture | | |
| --- | --- | --- | --- | --- |
|  |  | **Biophysical Cumulative share** | **Mass Cumulative share** | **Economic Cumulative share** |
| Abomasum | Human food | 0.0140 | 0.0025 | 0.0018 |
| Abomasum fat | Fat and greaves C3 | 0.0011 | 0.0005 | 0.0000 |
| Aponeurosis | Human food | 0.0268 | 0.0351 | 0.0348 |
| Bile | PAP C3 | 0.0000 | 0.0001 | 0.0000 |
| Blood | PAP C3 | 0.0174 | 0.0295 | 0.0065 |
| Blood | Pet food | 0.0025 | 0.0042 | 0.0003 |
| Bones | Gelatin C3 | 0.0510 | 0.0786 | 0.0002 |
| Bones of head, brain, eyes and teeth | C1-C2 for disposal | 0.0000 | 0.0000 | 0.0000 |
| Cheek | Human food | 0.0010 | 0.0015 | 0.0032 |
| Cheek | Human food | 0.0019 | 0.0027 | 0.0059 |
| Cheek trimmings | Pet food | 0.0005 | 0.0007 | 0.0001 |
| Chops | Pet food | 0.0019 | 0.0020 | 0.0001 |
| Contents of intestines | Spreading/Compost | 0.0000 | 0.0000 | 0.0000 |
| Contents of the rumen | Spreading/Compost | 0.0000 | 0.0000 | 0.0000 |
| Ears | PAP C3 | 0.0009 | 0.0010 | 0.0001 |
| Esophagus | Pet food | 0.0009 | 0.0014 | 0.0001 |
| Fat | Fat and greaves C3 | 0.0867 | 0.0841 | 0.0076 |
| Fat around heart | Fat and greaves C3 | 0.0028 | 0.0027 | 0.0002 |
| Fat in the kidney | Fat and greaves C3 | 0.0004 | 0.0004 | 0.0000 |
| Feet (without hooves) | Gelatin C3 | 0.0121 | 0.0177 | 0.0001 |
| Floatation fat | Spreading/Compost | 0.0000 | 0.0000 | 0.0000 |
| Forehead | C1-C2 for disposal | 0.0000 | 0.0000 | 0.0000 |
| Forelock | PAP C3 | 0.0028 | 0.0011 | 0.0001 |
| Gallbladder | Pet food | 0.0003 | 0.0005 | 0.0000 |
| Head trimmings | Pet food | 0.0026 | 0.0037 | 0.0003 |
| Heart | Human food | 0.0029 | 0.0044 | 0.0009 |
| Heart trimmings | Pet food | 0.0003 | 0.0005 | 0.0000 |
| Hide | Skin tannery C3 | 0.0673 | 0.0695 | 0.1146 |
| Hooves | PAP C3 | 0.0077 | 0.0031 | 0.0003 |
| Horns | PAP C3 | 0.0018 | 0.0007 | 0.0001 |
| Kidney | Human food | 0.0019 | 0.0027 | 0.0011 |
| Large intestine | C1-C2 for disposal | 0.0000 | 0.0000 | 0.0000 |
| Liver | Human food | 0.0532 | 0.0180 | 0.0086 |
| Liver trimmings | Pet food | 0.0061 | 0.0020 | 0.0001 |
| Lower jaw | PAP C3 | 0.0031 | 0.0049 | 0.0004 |
| Lungs | Pet food | 0.0081 | 0.0101 | 0.0007 |
| Mask | Skin tannery C3 | 0.0045 | 0.0047 | 0.0077 |
| Mesenteric fat | C1-C2 for disposal | 0.0000 | 0.0000 | 0.0000 |
| Muscle | Human food | 0.3291 | 0.4698 | 0.7759 |
| Muzzle | Human food | 0.0024 | 0.0025 | 0.0024 |
| Omasum | Human food | 0.0126 | 0.0022 | 0.0016 |
| Omasum fat | Fat and greaves C3 | 0.0033 | 0.0015 | 0.0001 |
| Rumen and forestomach | Human food | 0.0660 | 0.0116 | 0.0086 |
| Rumen fat | Fat and greaves C3 | 0.0050 | 0.0022 | 0.0002 |
| Sanitary seizures | C1-C2 for disposal | 0.0000 | 0.0000 | 0.0000 |
| Screening and sifting wastes | C1-C2 for disposal | 0.0000 | 0.0000 | 0.0000 |
| Small intestine | PAP C3 | 0.0765 | 0.0134 | 0.0008 |
| Spinal cord | C1-C2 for disposal | 0.0000 | 0.0000 | 0.0000 |
| Spinal cord waste | C1-C2 for disposal | 0.0000 | 0.0000 | 0.0000 |
| Spine | C1-C2 for disposal | 0.0000 | 0.0000 | 0.0000 |
| Spleen | Pet food | 0.0019 | 0.0027 | 0.0002 |
| Stillborn | PAP C3 | 0.0396 | 0.0066 | 0.0000 |
| Tallow | Fat and greaves C3 | 0.0709 | 0.0689 | 0.0062 |
| Tongue | Human food | 0.0024 | 0.0039 | 0.0062 |
| Tonsil | C1-C2 for disposal | 0.0000 | 0.0000 | 0.0000 |
| Trachea | Pet food | 0.0017 | 0.0017 | 0.0001 |
| Udder | Pet food | 0.0031 | 0.0210 | 0.0015 |
| Upper throat | Pet food | 0.0010 | 0.0015 | 0.0001 |
| Water in the rumen | Spreading/Compost | 0.0000 | 0.0000 | 0.0000 |

Table 90: Total weighting by coproducts for Charolaise x Rustique Heifers reared in Pasture

| COPRODUCT | Destination | Charolaise x Rustique/heifer/pasture | | |
| --- | --- | --- | --- | --- |
|  |  | **Biophysical Cumulative share** | **Mass Cumulative share** | **Economic Cumulative share** |
| Abomasum | Human food | 0.0149 | 0.0027 | 0.0021 |
| Abomasum fat | Fat and greaves C3 | 0.0011 | 0.0005 | 0.0000 |
| Aponeurosis | Human food | 0.0254 | 0.0336 | 0.0340 |
| Bile | PAP C3 | 0.0000 | 0.0001 | 0.0000 |
| Blood | PAP C3 | 0.0186 | 0.0319 | 0.0072 |
| Blood | Pet food | 0.0027 | 0.0046 | 0.0003 |
| Bones | Gelatin C3 | 0.0484 | 0.0753 | 0.0002 |
| Bones of head, brain, eyes and teeth | C1-C2 for disposal | 0.0000 | 0.0000 | 0.0000 |
| Cheek | Human food | 0.0011 | 0.0016 | 0.0036 |
| Cheek | Human food | 0.0021 | 0.0030 | 0.0066 |
| Cheek trimmings | Pet food | 0.0005 | 0.0007 | 0.0001 |
| Chops | Pet food | 0.0020 | 0.0021 | 0.0002 |
| Contents of intestines | Spreading/Compost | 0.0000 | 0.0000 | 0.0000 |
| Contents of the rumen | Spreading/Compost | 0.0000 | 0.0000 | 0.0000 |
| Ears | PAP C3 | 0.0010 | 0.0011 | 0.0001 |
| Esophagus | Pet food | 0.0010 | 0.0015 | 0.0001 |
| Fat | Fat and greaves C3 | 0.0824 | 0.0807 | 0.0074 |
| Fat around heart | Fat and greaves C3 | 0.0030 | 0.0030 | 0.0003 |
| Fat in the kidney | Fat and greaves C3 | 0.0004 | 0.0004 | 0.0000 |
| Feet (without hooves) | Gelatin C3 | 0.0130 | 0.0192 | 0.0001 |
| Floatation fat | Spreading/Compost | 0.0000 | 0.0000 | 0.0000 |
| Forehead | C1-C2 for disposal | 0.0000 | 0.0000 | 0.0000 |
| Forelock | PAP C3 | 0.0031 | 0.0012 | 0.0001 |
| Gallbladder | Pet food | 0.0003 | 0.0005 | 0.0000 |
| Head trimmings | Pet food | 0.0027 | 0.0040 | 0.0003 |
| Heart | Human food | 0.0032 | 0.0049 | 0.0010 |
| Heart trimmings | Pet food | 0.0003 | 0.0005 | 0.0000 |
| Hide | Skin tannery C3 | 0.0720 | 0.0752 | 0.1263 |
| Hooves | PAP C3 | 0.0083 | 0.0034 | 0.0003 |
| Horns | PAP C3 | 0.0018 | 0.0007 | 0.0001 |
| Kidney | Human food | 0.0020 | 0.0030 | 0.0012 |
| Large intestine | C1-C2 for disposal | 0.0000 | 0.0000 | 0.0000 |
| Liver | Human food | 0.0551 | 0.0194 | 0.0095 |
| Liver trimmings | Pet food | 0.0063 | 0.0021 | 0.0002 |
| Lower jaw | PAP C3 | 0.0034 | 0.0054 | 0.0005 |
| Lungs | Pet food | 0.0086 | 0.0108 | 0.0008 |
| Mask | Skin tannery C3 | 0.0049 | 0.0051 | 0.0086 |
| Mesenteric fat | C1-C2 for disposal | 0.0000 | 0.0000 | 0.0000 |
| Muscle | Human food | 0.3121 | 0.4504 | 0.7579 |
| Muzzle | Human food | 0.0026 | 0.0027 | 0.0028 |
| Omasum | Human food | 0.0129 | 0.0024 | 0.0018 |
| Omasum fat | Fat and greaves C3 | 0.0035 | 0.0016 | 0.0001 |
| Rumen and forestomach | Human food | 0.0677 | 0.0125 | 0.0094 |
| Rumen fat | Fat and greaves C3 | 0.0051 | 0.0024 | 0.0002 |
| Sanitary seizures | C1-C2 for disposal | 0.0000 | 0.0000 | 0.0000 |
| Screening and sifting wastes | C1-C2 for disposal | 0.0000 | 0.0000 | 0.0000 |
| Small intestine | PAP C3 | 0.0785 | 0.0144 | 0.0009 |
| Spinal cord | C1-C2 for disposal | 0.0000 | 0.0000 | 0.0000 |
| Spinal cord waste | C1-C2 for disposal | 0.0000 | 0.0000 | 0.0000 |
| Spine | C1-C2 for disposal | 0.0000 | 0.0000 | 0.0000 |
| Spleen | Pet food | 0.0021 | 0.0030 | 0.0002 |
| Stillborn | PAP C3 | 0.0410 | 0.0072 | 0.0000 |
| Tallow | Fat and greaves C3 | 0.0761 | 0.0746 | 0.0068 |
| Tongue | Human food | 0.0026 | 0.0042 | 0.0068 |
| Tonsil | C1-C2 for disposal | 0.0000 | 0.0000 | 0.0000 |
| Trachea | Pet food | 0.0018 | 0.0019 | 0.0001 |
| Udder | Pet food | 0.0034 | 0.0228 | 0.0017 |
| Upper throat | Pet food | 0.0011 | 0.0016 | 0.0001 |
| Water in the rumen | Spreading/Compost | 0.0000 | 0.0000 | 0.0000 |

Table 91: Total weighting by coproducts for Charolaise x Rustique Cull Cows reared in Pasture

| COPRODUCT | Destination | Charolaise x Rustique/Cull cow/pasture | | |
| --- | --- | --- | --- | --- |
|  |  | **Biophysical Cumulative share** | **Mass Cumulative share** | **Economic Cumulative share** |
| Abomasum | Human food | 0.0151 | 0.0028 | 0.0021 |
| Abomasum fat | Fat and greaves C3 | 0.0011 | 0.0005 | 0.0000 |
| Aponeurosis | Human food | 0.0242 | 0.0326 | 0.0334 |
| Bile | PAP C3 | 0.0000 | 0.0001 | 0.0000 |
| Blood | PAP C3 | 0.0192 | 0.0335 | 0.0076 |
| Blood | Pet food | 0.0027 | 0.0048 | 0.0004 |
| Bones | Gelatin C3 | 0.0460 | 0.0732 | 0.0002 |
| Bones of head, brain, eyes and teeth | C1-C2 for disposal | 0.0000 | 0.0000 | 0.0000 |
| Cheek | Human food | 0.0011 | 0.0016 | 0.0037 |
| Cheek | Human food | 0.0021 | 0.0031 | 0.0070 |
| Cheek trimmings | Pet food | 0.0006 | 0.0009 | 0.0001 |
| Chops | Pet food | 0.0021 | 0.0023 | 0.0002 |
| Contents of intestines | Spreading/Compost | 0.0000 | 0.0000 | 0.0000 |
| Contents of the rumen | Spreading/Compost | 0.0000 | 0.0000 | 0.0000 |
| Ears | PAP C3 | 0.0010 | 0.0011 | 0.0001 |
| Esophagus | Pet food | 0.0010 | 0.0015 | 0.0001 |
| Fat | Fat and greaves C3 | 0.0778 | 0.0783 | 0.0073 |
| Fat around heart | Fat and greaves C3 | 0.0031 | 0.0031 | 0.0003 |
| Fat in the kidney | Fat and greaves C3 | 0.0004 | 0.0004 | 0.0000 |
| Feet (without hooves) | Gelatin C3 | 0.0135 | 0.0202 | 0.0001 |
| Floatation fat | Spreading/Compost | 0.0000 | 0.0000 | 0.0000 |
| Forehead | C1-C2 for disposal | 0.0000 | 0.0000 | 0.0000 |
| Forelock | PAP C3 | 0.0030 | 0.0013 | 0.0001 |
| Gallbladder | Pet food | 0.0003 | 0.0005 | 0.0000 |
| Head trimmings | Pet food | 0.0029 | 0.0043 | 0.0003 |
| Heart | Human food | 0.0032 | 0.0050 | 0.0011 |
| Heart trimmings | Pet food | 0.0003 | 0.0005 | 0.0000 |
| Hide | Skin tannery C3 | 0.0744 | 0.0791 | 0.1346 |
| Hooves | PAP C3 | 0.0085 | 0.0035 | 0.0003 |
| Horns | PAP C3 | 0.0021 | 0.0009 | 0.0001 |
| Kidney | Human food | 0.0021 | 0.0031 | 0.0013 |
| Large intestine | C1-C2 for disposal | 0.0000 | 0.0000 | 0.0000 |
| Liver | Human food | 0.0582 | 0.0205 | 0.0101 |
| Liver trimmings | Pet food | 0.0067 | 0.0023 | 0.0002 |
| Lower jaw | PAP C3 | 0.0035 | 0.0056 | 0.0005 |
| Lungs | Pet food | 0.0089 | 0.0114 | 0.0009 |
| Mask | Skin tannery C3 | 0.0051 | 0.0054 | 0.0092 |
| Mesenteric fat | C1-C2 for disposal | 0.0000 | 0.0000 | 0.0000 |
| Muscle | Human food | 0.2974 | 0.4374 | 0.7457 |
| Muzzle | Human food | 0.0026 | 0.0028 | 0.0028 |
| Omasum | Human food | 0.0137 | 0.0025 | 0.0019 |
| Omasum fat | Fat and greaves C3 | 0.0035 | 0.0016 | 0.0002 |
| Rumen and forestomach | Human food | 0.0714 | 0.0131 | 0.0100 |
| Rumen fat | Fat and greaves C3 | 0.0054 | 0.0025 | 0.0002 |
| Sanitary seizures | C1-C2 for disposal | 0.0000 | 0.0000 | 0.0000 |
| Screening and sifting wastes | C1-C2 for disposal | 0.0000 | 0.0000 | 0.0000 |
| Small intestine | PAP C3 | 0.0830 | 0.0152 | 0.0009 |
| Spinal cord | C1-C2 for disposal | 0.0000 | 0.0000 | 0.0000 |
| Spinal cord waste | C1-C2 for disposal | 0.0000 | 0.0000 | 0.0000 |
| Spine | C1-C2 for disposal | 0.0000 | 0.0000 | 0.0000 |
| Spleen | Pet food | 0.0022 | 0.0031 | 0.0002 |
| Stillborn | PAP C3 | 0.0437 | 0.0077 | 0.0000 |
| Tallow | Fat and greaves C3 | 0.0778 | 0.0785 | 0.0073 |
| Tongue | Human food | 0.0027 | 0.0045 | 0.0073 |
| Tonsil | C1-C2 for disposal | 0.0000 | 0.0000 | 0.0000 |
| Trachea | Pet food | 0.0019 | 0.0020 | 0.0002 |
| Udder | Pet food | 0.0035 | 0.0240 | 0.0018 |
| Upper throat | Pet food | 0.0011 | 0.0016 | 0.0001 |
| Water in the rumen | Spreading/Compost | 0.0000 | 0.0000 | 0.0000 |

Table 92: Total weighting by coproducts for Charolaise x Rustique Beef reared in Pasture

| COPRODUCT | Destination | Charolaise x Rustique/beef/pasture | | |
| --- | --- | --- | --- | --- |
|  |  | **Biophysical Cumulative share** | **Mass Cumulative share** | **Economic Cumulative share** |
| Abomasum | Human food | 0.0148 | 0.0026 | 0.0020 |
| Abomasum fat | Fat and greaves C3 | 0.0011 | 0.0005 | 0.0000 |
| Aponeurosis | Human food | 0.0257 | 0.0341 | 0.0342 |
| Bile | PAP C3 | 0.0000 | 0.0001 | 0.0000 |
| Blood | PAP C3 | 0.0181 | 0.0311 | 0.0070 |
| Blood | Pet food | 0.0026 | 0.0045 | 0.0003 |
| Bones | Gelatin C3 | 0.0487 | 0.0764 | 0.0002 |
| Bones of head, brain, eyes and teeth | C1-C2 for disposal | 0.0000 | 0.0000 | 0.0000 |
| Cheek | Human food | 0.0011 | 0.0016 | 0.0035 |
| Cheek | Human food | 0.0020 | 0.0029 | 0.0063 |
| Cheek trimmings | Pet food | 0.0005 | 0.0007 | 0.0001 |
| Chops | Pet food | 0.0020 | 0.0021 | 0.0002 |
| Contents of intestines | Spreading/Compost | 0.0000 | 0.0000 | 0.0000 |
| Contents of the rumen | Spreading/Compost | 0.0000 | 0.0000 | 0.0000 |
| Ears | PAP C3 | 0.0008 | 0.0010 | 0.0001 |
| Esophagus | Pet food | 0.0010 | 0.0015 | 0.0001 |
| Fat | Fat and greaves C3 | 0.0822 | 0.0818 | 0.0074 |
| Fat around heart | Fat and greaves C3 | 0.0029 | 0.0029 | 0.0003 |
| Fat in the kidney | Fat and greaves C3 | 0.0004 | 0.0004 | 0.0000 |
| Feet (without hooves) | Gelatin C3 | 0.0126 | 0.0187 | 0.0001 |
| Floatation fat | Spreading/Compost | 0.0000 | 0.0000 | 0.0000 |
| Forehead | C1-C2 for disposal | 0.0000 | 0.0000 | 0.0000 |
| Forelock | PAP C3 | 0.0027 | 0.0011 | 0.0001 |
| Gallbladder | Pet food | 0.0003 | 0.0005 | 0.0000 |
| Head trimmings | Pet food | 0.0027 | 0.0040 | 0.0003 |
| Heart | Human food | 0.0031 | 0.0047 | 0.0010 |
| Heart trimmings | Pet food | 0.0003 | 0.0005 | 0.0000 |
| Hide | Skin tannery C3 | 0.0699 | 0.0733 | 0.1223 |
| Hooves | PAP C3 | 0.0079 | 0.0032 | 0.0003 |
| Horns | PAP C3 | 0.0018 | 0.0007 | 0.0001 |
| Kidney | Human food | 0.0019 | 0.0029 | 0.0012 |
| Large intestine | C1-C2 for disposal | 0.0000 | 0.0000 | 0.0000 |
| Liver | Human food | 0.0558 | 0.0190 | 0.0092 |
| Liver trimmings | Pet food | 0.0065 | 0.0021 | 0.0002 |
| Lower jaw | PAP C3 | 0.0033 | 0.0052 | 0.0004 |
| Lungs | Pet food | 0.0083 | 0.0105 | 0.0008 |
| Mask | Skin tannery C3 | 0.0047 | 0.0050 | 0.0083 |
| Mesenteric fat | C1-C2 for disposal | 0.0000 | 0.0000 | 0.0000 |
| Muscle | Human food | 0.3153 | 0.4569 | 0.7640 |
| Muzzle | Human food | 0.0025 | 0.0026 | 0.0026 |
| Omasum | Human food | 0.0134 | 0.0024 | 0.0018 |
| Omasum fat | Fat and greaves C3 | 0.0036 | 0.0016 | 0.0001 |
| Rumen and forestomach | Human food | 0.0689 | 0.0121 | 0.0091 |
| Rumen fat | Fat and greaves C3 | 0.0052 | 0.0024 | 0.0002 |
| Sanitary seizures | C1-C2 for disposal | 0.0000 | 0.0000 | 0.0000 |
| Screening and sifting wastes | C1-C2 for disposal | 0.0000 | 0.0000 | 0.0000 |
| Small intestine | PAP C3 | 0.0801 | 0.0141 | 0.0009 |
| Spinal cord | C1-C2 for disposal | 0.0000 | 0.0000 | 0.0000 |
| Spinal cord waste | C1-C2 for disposal | 0.0000 | 0.0000 | 0.0000 |
| Spine | C1-C2 for disposal | 0.0000 | 0.0000 | 0.0000 |
| Spleen | Pet food | 0.0020 | 0.0029 | 0.0002 |
| Stillborn | PAP C3 | 0.0418 | 0.0071 | 0.0000 |
| Tallow | Fat and greaves C3 | 0.0729 | 0.0727 | 0.0066 |
| Tongue | Human food | 0.0025 | 0.0042 | 0.0067 |
| Tonsil | C1-C2 for disposal | 0.0000 | 0.0000 | 0.0000 |
| Trachea | Pet food | 0.0018 | 0.0019 | 0.0001 |
| Udder | Pet food | 0.0033 | 0.0222 | 0.0016 |
| Upper throat | Pet food | 0.0011 | 0.0016 | 0.0001 |
| Water in the rumen | Spreading/Compost | 0.0000 | 0.0000 | 0.0000 |

Table 93: Total weighting by coproducts for Charolaise x Rustique Young Bulls reared in Stall

| COPRODUCT | Destination | Charolaise x Rustique/young bull/stall | | |
| --- | --- | --- | --- | --- |
|  |  | **Biophysical Cumulative share** | **Mass Cumulative share** | **Economic Cumulative share** |
| Abomasum | Human food | 0.0133 | 0.0025 | 0.0018 |
| Abomasum fat | Fat and greaves C3 | 0.0011 | 0.0005 | 0.0000 |
| Aponeurosis | Human food | 0.0265 | 0.0351 | 0.0348 |
| Bile | PAP C3 | 0.0000 | 0.0001 | 0.0000 |
| Blood | PAP C3 | 0.0172 | 0.0295 | 0.0065 |
| Blood | Pet food | 0.0024 | 0.0042 | 0.0003 |
| Bones | Gelatin C3 | 0.0518 | 0.0786 | 0.0002 |
| Bones of head, brain, eyes and teeth | C1-C2 for disposal | 0.0000 | 0.0000 | 0.0000 |
| Cheek | Human food | 0.0010 | 0.0015 | 0.0032 |
| Cheek | Human food | 0.0019 | 0.0027 | 0.0059 |
| Cheek trimmings | Pet food | 0.0005 | 0.0007 | 0.0001 |
| Chops | Pet food | 0.0019 | 0.0020 | 0.0001 |
| Contents of intestines | Spreading/Compost | 0.0000 | 0.0000 | 0.0000 |
| Contents of the rumen | Spreading/Compost | 0.0000 | 0.0000 | 0.0000 |
| Ears | PAP C3 | 0.0009 | 0.0010 | 0.0001 |
| Esophagus | Pet food | 0.0009 | 0.0014 | 0.0001 |
| Fat | Fat and greaves C3 | 0.0952 | 0.0841 | 0.0076 |
| Fat around heart | Fat and greaves C3 | 0.0031 | 0.0027 | 0.0002 |
| Fat in the kidney | Fat and greaves C3 | 0.0004 | 0.0004 | 0.0000 |
| Feet (without hooves) | Gelatin C3 | 0.0121 | 0.0177 | 0.0001 |
| Floatation fat | Spreading/Compost | 0.0000 | 0.0000 | 0.0000 |
| Forehead | C1-C2 for disposal | 0.0000 | 0.0000 | 0.0000 |
| Forelock | PAP C3 | 0.0027 | 0.0011 | 0.0001 |
| Gallbladder | Pet food | 0.0003 | 0.0005 | 0.0000 |
| Head trimmings | Pet food | 0.0025 | 0.0037 | 0.0003 |
| Heart | Human food | 0.0029 | 0.0044 | 0.0009 |
| Heart trimmings | Pet food | 0.0003 | 0.0005 | 0.0000 |
| Hide | Skin tannery C3 | 0.0665 | 0.0695 | 0.1146 |
| Hooves | PAP C3 | 0.0076 | 0.0031 | 0.0003 |
| Horns | PAP C3 | 0.0018 | 0.0007 | 0.0001 |
| Kidney | Human food | 0.0018 | 0.0027 | 0.0011 |
| Large intestine | C1-C2 for disposal | 0.0000 | 0.0000 | 0.0000 |
| Liver | Human food | 0.0509 | 0.0180 | 0.0086 |
| Liver trimmings | Pet food | 0.0058 | 0.0020 | 0.0001 |
| Lower jaw | PAP C3 | 0.0032 | 0.0049 | 0.0004 |
| Lungs | Pet food | 0.0080 | 0.0101 | 0.0007 |
| Mask | Skin tannery C3 | 0.0045 | 0.0047 | 0.0077 |
| Mesenteric fat | C1-C2 for disposal | 0.0000 | 0.0000 | 0.0000 |
| Muscle | Human food | 0.3272 | 0.4698 | 0.7759 |
| Muzzle | Human food | 0.0024 | 0.0025 | 0.0024 |
| Omasum | Human food | 0.0120 | 0.0022 | 0.0016 |
| Omasum fat | Fat and greaves C3 | 0.0033 | 0.0015 | 0.0001 |
| Rumen and forestomach | Human food | 0.0627 | 0.0116 | 0.0086 |
| Rumen fat | Fat and greaves C3 | 0.0050 | 0.0022 | 0.0002 |
| Sanitary seizures | C1-C2 for disposal | 0.0000 | 0.0000 | 0.0000 |
| Screening and sifting wastes | C1-C2 for disposal | 0.0000 | 0.0000 | 0.0000 |
| Small intestine | PAP C3 | 0.0727 | 0.0134 | 0.0008 |
| Spinal cord | C1-C2 for disposal | 0.0000 | 0.0000 | 0.0000 |
| Spinal cord waste | C1-C2 for disposal | 0.0000 | 0.0000 | 0.0000 |
| Spine | C1-C2 for disposal | 0.0000 | 0.0000 | 0.0000 |
| Spleen | Pet food | 0.0019 | 0.0027 | 0.0002 |
| Stillborn | PAP C3 | 0.0376 | 0.0066 | 0.0000 |
| Tallow | Fat and greaves C3 | 0.0778 | 0.0689 | 0.0062 |
| Tongue | Human food | 0.0024 | 0.0039 | 0.0062 |
| Tonsil | C1-C2 for disposal | 0.0000 | 0.0000 | 0.0000 |
| Trachea | Pet food | 0.0017 | 0.0017 | 0.0001 |
| Udder | Pet food | 0.0032 | 0.0210 | 0.0015 |
| Upper throat | Pet food | 0.0010 | 0.0015 | 0.0001 |
| Water in the rumen | Spreading/Compost | 0.0000 | 0.0000 | 0.0000 |

Table 94: Total weighting by coproducts for Charolaise x Rustique Heifers reared in Stall

| COPRODUCT | Destination | Charolaise x Rustique/heifer/stall | | |
| --- | --- | --- | --- | --- |
|  |  | **Biophysical Cumulative share** | **Mass Cumulative share** | **Economic Cumulative share** |
| Abomasum | Human food | 0.0141 | 0.0027 | 0.0021 |
| Abomasum fat | Fat and greaves C3 | 0.0011 | 0.0005 | 0.0000 |
| Aponeurosis | Human food | 0.0252 | 0.0336 | 0.0340 |
| Bile | PAP C3 | 0.0000 | 0.0001 | 0.0000 |
| Blood | PAP C3 | 0.0185 | 0.0319 | 0.0072 |
| Blood | Pet food | 0.0027 | 0.0046 | 0.0003 |
| Bones | Gelatin C3 | 0.0492 | 0.0753 | 0.0002 |
| Bones of head, brain, eyes and teeth | C1-C2 for disposal | 0.0000 | 0.0000 | 0.0000 |
| Cheek | Human food | 0.0011 | 0.0016 | 0.0036 |
| Cheek | Human food | 0.0020 | 0.0030 | 0.0066 |
| Cheek trimmings | Pet food | 0.0005 | 0.0007 | 0.0001 |
| Chops | Pet food | 0.0020 | 0.0021 | 0.0002 |
| Contents of intestines | Spreading/Compost | 0.0000 | 0.0000 | 0.0000 |
| Contents of the rumen | Spreading/Compost | 0.0000 | 0.0000 | 0.0000 |
| Ears | PAP C3 | 0.0010 | 0.0011 | 0.0001 |
| Esophagus | Pet food | 0.0010 | 0.0015 | 0.0001 |
| Fat | Fat and greaves C3 | 0.0902 | 0.0807 | 0.0074 |
| Fat around heart | Fat and greaves C3 | 0.0033 | 0.0030 | 0.0003 |
| Fat in the kidney | Fat and greaves C3 | 0.0004 | 0.0004 | 0.0000 |
| Feet (without hooves) | Gelatin C3 | 0.0130 | 0.0192 | 0.0001 |
| Floatation fat | Spreading/Compost | 0.0000 | 0.0000 | 0.0000 |
| Forehead | C1-C2 for disposal | 0.0000 | 0.0000 | 0.0000 |
| Forelock | PAP C3 | 0.0030 | 0.0012 | 0.0001 |
| Gallbladder | Pet food | 0.0003 | 0.0005 | 0.0000 |
| Head trimmings | Pet food | 0.0027 | 0.0040 | 0.0003 |
| Heart | Human food | 0.0032 | 0.0049 | 0.0010 |
| Heart trimmings | Pet food | 0.0003 | 0.0005 | 0.0000 |
| Hide | Skin tannery C3 | 0.0714 | 0.0752 | 0.1263 |
| Hooves | PAP C3 | 0.0082 | 0.0034 | 0.0003 |
| Horns | PAP C3 | 0.0018 | 0.0007 | 0.0001 |
| Kidney | Human food | 0.0020 | 0.0030 | 0.0012 |
| Large intestine | C1-C2 for disposal | 0.0000 | 0.0000 | 0.0000 |
| Liver | Human food | 0.0526 | 0.0194 | 0.0095 |
| Liver trimmings | Pet food | 0.0060 | 0.0021 | 0.0002 |
| Lower jaw | PAP C3 | 0.0034 | 0.0054 | 0.0005 |
| Lungs | Pet food | 0.0085 | 0.0108 | 0.0008 |
| Mask | Skin tannery C3 | 0.0048 | 0.0051 | 0.0086 |
| Mesenteric fat | C1-C2 for disposal | 0.0000 | 0.0000 | 0.0000 |
| Muscle | Human food | 0.3110 | 0.4504 | 0.7579 |
| Muzzle | Human food | 0.0026 | 0.0027 | 0.0028 |
| Omasum | Human food | 0.0122 | 0.0024 | 0.0018 |
| Omasum fat | Fat and greaves C3 | 0.0035 | 0.0016 | 0.0001 |
| Rumen and forestomach | Human food | 0.0642 | 0.0125 | 0.0094 |
| Rumen fat | Fat and greaves C3 | 0.0051 | 0.0024 | 0.0002 |
| Sanitary seizures | C1-C2 for disposal | 0.0000 | 0.0000 | 0.0000 |
| Screening and sifting wastes | C1-C2 for disposal | 0.0000 | 0.0000 | 0.0000 |
| Small intestine | PAP C3 | 0.0745 | 0.0144 | 0.0009 |
| Spinal cord | C1-C2 for disposal | 0.0000 | 0.0000 | 0.0000 |
| Spinal cord waste | C1-C2 for disposal | 0.0000 | 0.0000 | 0.0000 |
| Spine | C1-C2 for disposal | 0.0000 | 0.0000 | 0.0000 |
| Spleen | Pet food | 0.0021 | 0.0030 | 0.0002 |
| Stillborn | PAP C3 | 0.0388 | 0.0072 | 0.0000 |
| Tallow | Fat and greaves C3 | 0.0833 | 0.0746 | 0.0068 |
| Tongue | Human food | 0.0026 | 0.0042 | 0.0068 |
| Tonsil | C1-C2 for disposal | 0.0000 | 0.0000 | 0.0000 |
| Trachea | Pet food | 0.0018 | 0.0019 | 0.0001 |
| Udder | Pet food | 0.0035 | 0.0228 | 0.0017 |
| Upper throat | Pet food | 0.0011 | 0.0016 | 0.0001 |
| Water in the rumen | Spreading/Compost | 0.0000 | 0.0000 | 0.0000 |

Table 95: Total weighting by coproducts for Charolaise x Rustique Cull Cows reared in Stall

| COPRODUCT | Destination | Charolaise x Rustique/Cull cow/stall | | |
| --- | --- | --- | --- | --- |
|  |  | **Biophysical Cumulative share** | **Mass Cumulative share** | **Economic Cumulative share** |
| Abomasum | Human food | 0.0144 | 0.0028 | 0.0021 |
| Abomasum fat | Fat and greaves C3 | 0.0011 | 0.0005 | 0.0000 |
| Aponeurosis | Human food | 0.0240 | 0.0326 | 0.0334 |
| Bile | PAP C3 | 0.0000 | 0.0001 | 0.0000 |
| Blood | PAP C3 | 0.0190 | 0.0335 | 0.0076 |
| Blood | Pet food | 0.0027 | 0.0048 | 0.0004 |
| Bones | Gelatin C3 | 0.0468 | 0.0732 | 0.0002 |
| Bones of head, brain, eyes and teeth | C1-C2 for disposal | 0.0000 | 0.0000 | 0.0000 |
| Cheek | Human food | 0.0011 | 0.0016 | 0.0037 |
| Cheek | Human food | 0.0021 | 0.0031 | 0.0070 |
| Cheek trimmings | Pet food | 0.0006 | 0.0009 | 0.0001 |
| Chops | Pet food | 0.0021 | 0.0023 | 0.0002 |
| Contents of intestines | Spreading/Compost | 0.0000 | 0.0000 | 0.0000 |
| Contents of the rumen | Spreading/Compost | 0.0000 | 0.0000 | 0.0000 |
| Ears | PAP C3 | 0.0010 | 0.0011 | 0.0001 |
| Esophagus | Pet food | 0.0010 | 0.0015 | 0.0001 |
| Fat | Fat and greaves C3 | 0.0854 | 0.0783 | 0.0073 |
| Fat around heart | Fat and greaves C3 | 0.0034 | 0.0031 | 0.0003 |
| Fat in the kidney | Fat and greaves C3 | 0.0004 | 0.0004 | 0.0000 |
| Feet (without hooves) | Gelatin C3 | 0.0134 | 0.0202 | 0.0001 |
| Floatation fat | Spreading/Compost | 0.0000 | 0.0000 | 0.0000 |
| Forehead | C1-C2 for disposal | 0.0000 | 0.0000 | 0.0000 |
| Forelock | PAP C3 | 0.0030 | 0.0013 | 0.0001 |
| Gallbladder | Pet food | 0.0003 | 0.0005 | 0.0000 |
| Head trimmings | Pet food | 0.0029 | 0.0043 | 0.0003 |
| Heart | Human food | 0.0032 | 0.0050 | 0.0011 |
| Heart trimmings | Pet food | 0.0003 | 0.0005 | 0.0000 |
| Hide | Skin tannery C3 | 0.0737 | 0.0791 | 0.1346 |
| Hooves | PAP C3 | 0.0084 | 0.0035 | 0.0003 |
| Horns | PAP C3 | 0.0021 | 0.0009 | 0.0001 |
| Kidney | Human food | 0.0021 | 0.0031 | 0.0013 |
| Large intestine | C1-C2 for disposal | 0.0000 | 0.0000 | 0.0000 |
| Liver | Human food | 0.0557 | 0.0205 | 0.0101 |
| Liver trimmings | Pet food | 0.0064 | 0.0023 | 0.0002 |
| Lower jaw | PAP C3 | 0.0036 | 0.0056 | 0.0005 |
| Lungs | Pet food | 0.0088 | 0.0114 | 0.0009 |
| Mask | Skin tannery C3 | 0.0050 | 0.0054 | 0.0092 |
| Mesenteric fat | C1-C2 for disposal | 0.0000 | 0.0000 | 0.0000 |
| Muscle | Human food | 0.2963 | 0.4374 | 0.7457 |
| Muzzle | Human food | 0.0026 | 0.0028 | 0.0028 |
| Omasum | Human food | 0.0131 | 0.0025 | 0.0019 |
| Omasum fat | Fat and greaves C3 | 0.0035 | 0.0016 | 0.0002 |
| Rumen and forestomach | Human food | 0.0679 | 0.0131 | 0.0100 |
| Rumen fat | Fat and greaves C3 | 0.0054 | 0.0025 | 0.0002 |
| Sanitary seizures | C1-C2 for disposal | 0.0000 | 0.0000 | 0.0000 |
| Screening and sifting wastes | C1-C2 for disposal | 0.0000 | 0.0000 | 0.0000 |
| Small intestine | PAP C3 | 0.0790 | 0.0152 | 0.0009 |
| Spinal cord | C1-C2 for disposal | 0.0000 | 0.0000 | 0.0000 |
| Spinal cord waste | C1-C2 for disposal | 0.0000 | 0.0000 | 0.0000 |
| Spine | C1-C2 for disposal | 0.0000 | 0.0000 | 0.0000 |
| Spleen | Pet food | 0.0021 | 0.0031 | 0.0002 |
| Stillborn | PAP C3 | 0.0415 | 0.0077 | 0.0000 |
| Tallow | Fat and greaves C3 | 0.0854 | 0.0785 | 0.0073 |
| Tongue | Human food | 0.0027 | 0.0045 | 0.0073 |
| Tonsil | C1-C2 for disposal | 0.0000 | 0.0000 | 0.0000 |
| Trachea | Pet food | 0.0019 | 0.0020 | 0.0002 |
| Udder | Pet food | 0.0036 | 0.0240 | 0.0018 |
| Upper throat | Pet food | 0.0011 | 0.0016 | 0.0001 |
| Water in the rumen | Spreading/Compost | 0.0000 | 0.0000 | 0.0000 |

Table 96: Total weighting by coproducts for Charolaise x Rustique Beef reared in Stall

| COPRODUCT | Destination | Charolaise x Rustique/beef/stall | | |
| --- | --- | --- | --- | --- |
|  |  | **Biophysical Cumulative share** | **Mass Cumulative share** | **Economic Cumulative share** |
| Abomasum | Human food | 0.0141 | 0.0026 | 0.0020 |
| Abomasum fat | Fat and greaves C3 | 0.0011 | 0.0005 | 0.0000 |
| Aponeurosis | Human food | 0.0254 | 0.0341 | 0.0342 |
| Bile | PAP C3 | 0.0000 | 0.0001 | 0.0000 |
| Blood | PAP C3 | 0.0179 | 0.0311 | 0.0070 |
| Blood | Pet food | 0.0026 | 0.0045 | 0.0003 |
| Bones | Gelatin C3 | 0.0495 | 0.0764 | 0.0002 |
| Bones of head, brain, eyes and teeth | C1-C2 for disposal | 0.0000 | 0.0000 | 0.0000 |
| Cheek | Human food | 0.0011 | 0.0016 | 0.0035 |
| Cheek | Human food | 0.0019 | 0.0029 | 0.0063 |
| Cheek trimmings | Pet food | 0.0005 | 0.0007 | 0.0001 |
| Chops | Pet food | 0.0020 | 0.0021 | 0.0002 |
| Contents of intestines | Spreading/Compost | 0.0000 | 0.0000 | 0.0000 |
| Contents of the rumen | Spreading/Compost | 0.0000 | 0.0000 | 0.0000 |
| Ears | PAP C3 | 0.0008 | 0.0010 | 0.0001 |
| Esophagus | Pet food | 0.0010 | 0.0015 | 0.0001 |
| Fat | Fat and greaves C3 | 0.0903 | 0.0818 | 0.0074 |
| Fat around heart | Fat and greaves C3 | 0.0031 | 0.0029 | 0.0003 |
| Fat in the kidney | Fat and greaves C3 | 0.0004 | 0.0004 | 0.0000 |
| Feet (without hooves) | Gelatin C3 | 0.0126 | 0.0187 | 0.0001 |
| Floatation fat | Spreading/Compost | 0.0000 | 0.0000 | 0.0000 |
| Forehead | C1-C2 for disposal | 0.0000 | 0.0000 | 0.0000 |
| Forelock | PAP C3 | 0.0027 | 0.0011 | 0.0001 |
| Gallbladder | Pet food | 0.0003 | 0.0005 | 0.0000 |
| Head trimmings | Pet food | 0.0027 | 0.0040 | 0.0003 |
| Heart | Human food | 0.0031 | 0.0047 | 0.0010 |
| Heart trimmings | Pet food | 0.0003 | 0.0005 | 0.0000 |
| Hide | Skin tannery C3 | 0.0691 | 0.0733 | 0.1223 |
| Hooves | PAP C3 | 0.0078 | 0.0032 | 0.0003 |
| Horns | PAP C3 | 0.0018 | 0.0007 | 0.0001 |
| Kidney | Human food | 0.0019 | 0.0029 | 0.0012 |
| Large intestine | C1-C2 for disposal | 0.0000 | 0.0000 | 0.0000 |
| Liver | Human food | 0.0534 | 0.0190 | 0.0092 |
| Liver trimmings | Pet food | 0.0062 | 0.0021 | 0.0002 |
| Lower jaw | PAP C3 | 0.0033 | 0.0052 | 0.0004 |
| Lungs | Pet food | 0.0082 | 0.0105 | 0.0008 |
| Mask | Skin tannery C3 | 0.0047 | 0.0050 | 0.0083 |
| Mesenteric fat | C1-C2 for disposal | 0.0000 | 0.0000 | 0.0000 |
| Muscle | Human food | 0.3136 | 0.4569 | 0.7640 |
| Muzzle | Human food | 0.0025 | 0.0026 | 0.0026 |
| Omasum | Human food | 0.0127 | 0.0024 | 0.0018 |
| Omasum fat | Fat and greaves C3 | 0.0036 | 0.0016 | 0.0001 |
| Rumen and forestomach | Human food | 0.0656 | 0.0121 | 0.0091 |
| Rumen fat | Fat and greaves C3 | 0.0052 | 0.0024 | 0.0002 |
| Sanitary seizures | C1-C2 for disposal | 0.0000 | 0.0000 | 0.0000 |
| Screening and sifting wastes | C1-C2 for disposal | 0.0000 | 0.0000 | 0.0000 |
| Small intestine | PAP C3 | 0.0763 | 0.0141 | 0.0009 |
| Spinal cord | C1-C2 for disposal | 0.0000 | 0.0000 | 0.0000 |
| Spinal cord waste | C1-C2 for disposal | 0.0000 | 0.0000 | 0.0000 |
| Spine | C1-C2 for disposal | 0.0000 | 0.0000 | 0.0000 |
| Spleen | Pet food | 0.0020 | 0.0029 | 0.0002 |
| Stillborn | PAP C3 | 0.0398 | 0.0071 | 0.0000 |
| Tallow | Fat and greaves C3 | 0.0801 | 0.0727 | 0.0066 |
| Tongue | Human food | 0.0026 | 0.0042 | 0.0067 |
| Tonsil | C1-C2 for disposal | 0.0000 | 0.0000 | 0.0000 |
| Trachea | Pet food | 0.0018 | 0.0019 | 0.0001 |
| Udder | Pet food | 0.0034 | 0.0222 | 0.0016 |
| Upper throat | Pet food | 0.0011 | 0.0016 | 0.0001 |
| Water in the rumen | Spreading/Compost | 0.0000 | 0.0000 | 0.0000 |

Table 97: Total weighting by coproducts for Montbéliarde Young Bulls reared in Grazing Large Area

| COPRODUCT | Destination | Montbéliarde/young bull/grazing large area | | |
| --- | --- | --- | --- | --- |
|  |  | **Biophysical Cumulative share** | **Mass Cumulative share** | **Economic Cumulative share** |
| Abomasum | Human food | 0.0151 | 0.0026 | 0.0020 |
| Abomasum fat | Fat and greaves C3 | 0.0011 | 0.0005 | 0.0000 |
| Aponeurosis | Human food | 0.0260 | 0.0341 | 0.0342 |
| Bile | PAP C3 | 0.0000 | 0.0001 | 0.0000 |
| Blood | PAP C3 | 0.0183 | 0.0311 | 0.0070 |
| Blood | Pet food | 0.0026 | 0.0045 | 0.0003 |
| Bones | Gelatin C3 | 0.0483 | 0.0764 | 0.0002 |
| Bones of head, brain, eyes and teeth | C1-C2 for disposal | 0.0000 | 0.0000 | 0.0000 |
| Cheek | Human food | 0.0011 | 0.0016 | 0.0035 |
| Cheek | Human food | 0.0020 | 0.0029 | 0.0063 |
| Cheek trimmings | Pet food | 0.0005 | 0.0007 | 0.0001 |
| Chops | Pet food | 0.0020 | 0.0021 | 0.0002 |
| Contents of intestines | Spreading/Compost | 0.0000 | 0.0000 | 0.0000 |
| Contents of the rumen | Spreading/Compost | 0.0000 | 0.0000 | 0.0000 |
| Ears | PAP C3 | 0.0008 | 0.0010 | 0.0001 |
| Esophagus | Pet food | 0.0010 | 0.0015 | 0.0001 |
| Fat | Fat and greaves C3 | 0.0762 | 0.0818 | 0.0074 |
| Fat around heart | Fat and greaves C3 | 0.0027 | 0.0029 | 0.0003 |
| Fat in the kidney | Fat and greaves C3 | 0.0003 | 0.0004 | 0.0000 |
| Feet (without hooves) | Gelatin C3 | 0.0127 | 0.0187 | 0.0001 |
| Floatation fat | Spreading/Compost | 0.0000 | 0.0000 | 0.0000 |
| Forehead | C1-C2 for disposal | 0.0000 | 0.0000 | 0.0000 |
| Forelock | PAP C3 | 0.0028 | 0.0011 | 0.0001 |
| Gallbladder | Pet food | 0.0003 | 0.0005 | 0.0000 |
| Head trimmings | Pet food | 0.0027 | 0.0040 | 0.0003 |
| Heart | Human food | 0.0031 | 0.0047 | 0.0010 |
| Heart trimmings | Pet food | 0.0003 | 0.0005 | 0.0000 |
| Hide | Skin tannery C3 | 0.0709 | 0.0733 | 0.1223 |
| Hooves | PAP C3 | 0.0080 | 0.0032 | 0.0003 |
| Horns | PAP C3 | 0.0019 | 0.0007 | 0.0001 |
| Kidney | Human food | 0.0019 | 0.0029 | 0.0012 |
| Large intestine | C1-C2 for disposal | 0.0000 | 0.0000 | 0.0000 |
| Liver | Human food | 0.0571 | 0.0190 | 0.0092 |
| Liver trimmings | Pet food | 0.0066 | 0.0021 | 0.0002 |
| Lower jaw | PAP C3 | 0.0032 | 0.0052 | 0.0004 |
| Lungs | Pet food | 0.0084 | 0.0105 | 0.0008 |
| Mask | Skin tannery C3 | 0.0048 | 0.0050 | 0.0083 |
| Mesenteric fat | C1-C2 for disposal | 0.0000 | 0.0000 | 0.0000 |
| Muscle | Human food | 0.3181 | 0.4569 | 0.7640 |
| Muzzle | Human food | 0.0025 | 0.0026 | 0.0026 |
| Omasum | Human food | 0.0137 | 0.0024 | 0.0018 |
| Omasum fat | Fat and greaves C3 | 0.0035 | 0.0016 | 0.0001 |
| Rumen and forestomach | Human food | 0.0706 | 0.0121 | 0.0091 |
| Rumen fat | Fat and greaves C3 | 0.0051 | 0.0024 | 0.0002 |
| Sanitary seizures | C1-C2 for disposal | 0.0000 | 0.0000 | 0.0000 |
| Screening and sifting wastes | C1-C2 for disposal | 0.0000 | 0.0000 | 0.0000 |
| Small intestine | PAP C3 | 0.0821 | 0.0141 | 0.0009 |
| Spinal cord | C1-C2 for disposal | 0.0000 | 0.0000 | 0.0000 |
| Spinal cord waste | C1-C2 for disposal | 0.0000 | 0.0000 | 0.0000 |
| Spine | C1-C2 for disposal | 0.0000 | 0.0000 | 0.0000 |
| Spleen | Pet food | 0.0020 | 0.0029 | 0.0002 |
| Stillborn | PAP C3 | 0.0429 | 0.0071 | 0.0000 |
| Tallow | Fat and greaves C3 | 0.0675 | 0.0727 | 0.0066 |
| Tongue | Human food | 0.0025 | 0.0042 | 0.0067 |
| Tonsil | C1-C2 for disposal | 0.0000 | 0.0000 | 0.0000 |
| Trachea | Pet food | 0.0018 | 0.0019 | 0.0001 |
| Udder | Pet food | 0.0032 | 0.0222 | 0.0016 |
| Upper throat | Pet food | 0.0011 | 0.0016 | 0.0001 |
| Water in the rumen | Spreading/Compost | 0.0000 | 0.0000 | 0.0000 |

Table 98: Total weighting by coproducts for Montbéliarde Heifers reared in Grazing Large Area

| COPRODUCT | Destination | Montbéliarde/heifer/grazing large area | | |
| --- | --- | --- | --- | --- |
|  |  | **Biophysical Cumulative share** | **Mass Cumulative share** | **Economic Cumulative share** |
| Abomasum | Human food | 0.0149 | 0.0028 | 0.0021 |
| Abomasum fat | Fat and greaves C3 | 0.0010 | 0.0005 | 0.0000 |
| Aponeurosis | Human food | 0.0250 | 0.0326 | 0.0334 |
| Bile | PAP C3 | 0.0000 | 0.0001 | 0.0000 |
| Blood | PAP C3 | 0.0198 | 0.0335 | 0.0076 |
| Blood | Pet food | 0.0028 | 0.0048 | 0.0004 |
| Bones | Gelatin C3 | 0.0463 | 0.0732 | 0.0002 |
| Bones of head, brain, eyes and teeth | C1-C2 for disposal | 0.0000 | 0.0000 | 0.0000 |
| Cheek | Human food | 0.0011 | 0.0016 | 0.0037 |
| Cheek | Human food | 0.0022 | 0.0031 | 0.0070 |
| Cheek trimmings | Pet food | 0.0006 | 0.0009 | 0.0001 |
| Chops | Pet food | 0.0022 | 0.0023 | 0.0002 |
| Contents of intestines | Spreading/Compost | 0.0000 | 0.0000 | 0.0000 |
| Contents of the rumen | Spreading/Compost | 0.0000 | 0.0000 | 0.0000 |
| Ears | PAP C3 | 0.0010 | 0.0011 | 0.0001 |
| Esophagus | Pet food | 0.0010 | 0.0015 | 0.0001 |
| Fat | Fat and greaves C3 | 0.0720 | 0.0783 | 0.0073 |
| Fat around heart | Fat and greaves C3 | 0.0029 | 0.0031 | 0.0003 |
| Fat in the kidney | Fat and greaves C3 | 0.0003 | 0.0004 | 0.0000 |
| Feet (without hooves) | Gelatin C3 | 0.0138 | 0.0202 | 0.0001 |
| Floatation fat | Spreading/Compost | 0.0000 | 0.0000 | 0.0000 |
| Forehead | C1-C2 for disposal | 0.0000 | 0.0000 | 0.0000 |
| Forelock | PAP C3 | 0.0032 | 0.0013 | 0.0001 |
| Gallbladder | Pet food | 0.0003 | 0.0005 | 0.0000 |
| Head trimmings | Pet food | 0.0030 | 0.0043 | 0.0003 |
| Heart | Human food | 0.0033 | 0.0050 | 0.0011 |
| Heart trimmings | Pet food | 0.0003 | 0.0005 | 0.0000 |
| Hide | Skin tannery C3 | 0.0770 | 0.0791 | 0.1346 |
| Hooves | PAP C3 | 0.0088 | 0.0035 | 0.0003 |
| Horns | PAP C3 | 0.0022 | 0.0009 | 0.0001 |
| Kidney | Human food | 0.0022 | 0.0031 | 0.0013 |
| Large intestine | C1-C2 for disposal | 0.0000 | 0.0000 | 0.0000 |
| Liver | Human food | 0.0579 | 0.0205 | 0.0101 |
| Liver trimmings | Pet food | 0.0067 | 0.0023 | 0.0002 |
| Lower jaw | PAP C3 | 0.0035 | 0.0056 | 0.0005 |
| Lungs | Pet food | 0.0092 | 0.0114 | 0.0009 |
| Mask | Skin tannery C3 | 0.0053 | 0.0054 | 0.0092 |
| Mesenteric fat | C1-C2 for disposal | 0.0000 | 0.0000 | 0.0000 |
| Muscle | Human food | 0.3055 | 0.4374 | 0.7457 |
| Muzzle | Human food | 0.0027 | 0.0028 | 0.0028 |
| Omasum | Human food | 0.0136 | 0.0025 | 0.0019 |
| Omasum fat | Fat and greaves C3 | 0.0034 | 0.0016 | 0.0002 |
| Rumen and forestomach | Human food | 0.0706 | 0.0131 | 0.0100 |
| Rumen fat | Fat and greaves C3 | 0.0052 | 0.0025 | 0.0002 |
| Sanitary seizures | C1-C2 for disposal | 0.0000 | 0.0000 | 0.0000 |
| Screening and sifting wastes | C1-C2 for disposal | 0.0000 | 0.0000 | 0.0000 |
| Small intestine | PAP C3 | 0.0822 | 0.0152 | 0.0009 |
| Spinal cord | C1-C2 for disposal | 0.0000 | 0.0000 | 0.0000 |
| Spinal cord waste | C1-C2 for disposal | 0.0000 | 0.0000 | 0.0000 |
| Spine | C1-C2 for disposal | 0.0000 | 0.0000 | 0.0000 |
| Spleen | Pet food | 0.0022 | 0.0031 | 0.0002 |
| Stillborn | PAP C3 | 0.0433 | 0.0077 | 0.0000 |
| Tallow | Fat and greaves C3 | 0.0720 | 0.0785 | 0.0073 |
| Tongue | Human food | 0.0027 | 0.0045 | 0.0073 |
| Tonsil | C1-C2 for disposal | 0.0000 | 0.0000 | 0.0000 |
| Trachea | Pet food | 0.0019 | 0.0020 | 0.0002 |
| Udder | Pet food | 0.0035 | 0.0240 | 0.0018 |
| Upper throat | Pet food | 0.0011 | 0.0016 | 0.0001 |
| Water in the rumen | Spreading/Compost | 0.0000 | 0.0000 | 0.0000 |

Table 99: Total weighting by coproducts for Montbéliarde Cull Cows reared in Grazing Large Area

| COPRODUCT | Destination | Montbéliarde/Cull cow/grazing large area | | |
| --- | --- | --- | --- | --- |
|  |  | **Biophysical Cumulative share** | **Mass Cumulative share** | **Economic Cumulative share** |
| Abomasum | Human food | 0.0160 | 0.0029 | 0.0023 |
| Abomasum fat | Fat and greaves C3 | 0.0013 | 0.0006 | 0.0001 |
| Aponeurosis | Human food | 0.0236 | 0.0316 | 0.0328 |
| Bile | PAP C3 | 0.0000 | 0.0001 | 0.0000 |
| Blood | PAP C3 | 0.0202 | 0.0352 | 0.0081 |
| Blood | Pet food | 0.0029 | 0.0051 | 0.0004 |
| Bones | Gelatin C3 | 0.0437 | 0.0709 | 0.0002 |
| Bones of head, brain, eyes and teeth | C1-C2 for disposal | 0.0000 | 0.0000 | 0.0000 |
| Cheek | Human food | 0.0012 | 0.0018 | 0.0040 |
| Cheek | Human food | 0.0022 | 0.0033 | 0.0075 |
| Cheek trimmings | Pet food | 0.0006 | 0.0009 | 0.0001 |
| Chops | Pet food | 0.0023 | 0.0024 | 0.0002 |
| Contents of intestines | Spreading/Compost | 0.0000 | 0.0000 | 0.0000 |
| Contents of the rumen | Spreading/Compost | 0.0000 | 0.0000 | 0.0000 |
| Ears | PAP C3 | 0.0010 | 0.0011 | 0.0001 |
| Esophagus | Pet food | 0.0011 | 0.0016 | 0.0001 |
| Fat | Fat and greaves C3 | 0.0684 | 0.0759 | 0.0071 |
| Fat around heart | Fat and greaves C3 | 0.0030 | 0.0033 | 0.0003 |
| Fat in the kidney | Fat and greaves C3 | 0.0005 | 0.0005 | 0.0000 |
| Feet (without hooves) | Gelatin C3 | 0.0141 | 0.0211 | 0.0001 |
| Floatation fat | Spreading/Compost | 0.0000 | 0.0000 | 0.0000 |
| Forehead | C1-C2 for disposal | 0.0000 | 0.0000 | 0.0000 |
| Forelock | PAP C3 | 0.0034 | 0.0014 | 0.0001 |
| Gallbladder | Pet food | 0.0004 | 0.0006 | 0.0000 |
| Head trimmings | Pet food | 0.0030 | 0.0044 | 0.0003 |
| Heart | Human food | 0.0034 | 0.0053 | 0.0012 |
| Heart trimmings | Pet food | 0.0004 | 0.0006 | 0.0000 |
| Hide | Skin tannery C3 | 0.0785 | 0.0830 | 0.1432 |
| Hooves | PAP C3 | 0.0089 | 0.0037 | 0.0003 |
| Horns | PAP C3 | 0.0022 | 0.0009 | 0.0001 |
| Kidney | Human food | 0.0022 | 0.0033 | 0.0014 |
| Large intestine | C1-C2 for disposal | 0.0000 | 0.0000 | 0.0000 |
| Liver | Human food | 0.0613 | 0.0215 | 0.0108 |
| Liver trimmings | Pet food | 0.0071 | 0.0024 | 0.0002 |
| Lower jaw | PAP C3 | 0.0036 | 0.0059 | 0.0005 |
| Lungs | Pet food | 0.0094 | 0.0120 | 0.0009 |
| Mask | Skin tannery C3 | 0.0053 | 0.0056 | 0.0096 |
| Mesenteric fat | C1-C2 for disposal | 0.0000 | 0.0000 | 0.0000 |
| Muscle | Human food | 0.2880 | 0.4239 | 0.7327 |
| Muzzle | Human food | 0.0028 | 0.0029 | 0.0030 |
| Omasum | Human food | 0.0146 | 0.0027 | 0.0021 |
| Omasum fat | Fat and greaves C3 | 0.0037 | 0.0018 | 0.0002 |
| Rumen and forestomach | Human food | 0.0756 | 0.0138 | 0.0107 |
| Rumen fat | Fat and greaves C3 | 0.0055 | 0.0027 | 0.0003 |
| Sanitary seizures | C1-C2 for disposal | 0.0000 | 0.0000 | 0.0000 |
| Screening and sifting wastes | C1-C2 for disposal | 0.0000 | 0.0000 | 0.0000 |
| Small intestine | PAP C3 | 0.0874 | 0.0159 | 0.0010 |
| Spinal cord | C1-C2 for disposal | 0.0000 | 0.0000 | 0.0000 |
| Spinal cord waste | C1-C2 for disposal | 0.0000 | 0.0000 | 0.0000 |
| Spine | C1-C2 for disposal | 0.0000 | 0.0000 | 0.0000 |
| Spleen | Pet food | 0.0023 | 0.0033 | 0.0002 |
| Stillborn | PAP C3 | 0.0457 | 0.0080 | 0.0000 |
| Tallow | Fat and greaves C3 | 0.0741 | 0.0824 | 0.0078 |
| Tongue | Human food | 0.0028 | 0.0047 | 0.0077 |
| Tonsil | C1-C2 for disposal | 0.0000 | 0.0000 | 0.0000 |
| Trachea | Pet food | 0.0019 | 0.0020 | 0.0002 |
| Udder | Pet food | 0.0035 | 0.0252 | 0.0019 |
| Upper throat | Pet food | 0.0012 | 0.0018 | 0.0001 |
| Water in the rumen | Spreading/Compost | 0.0000 | 0.0000 | 0.0000 |

Table 100: Total weighting by coproducts for Montbéliarde Beef reared in Grazing Large Area

| COPRODUCT | Destination | Montbéliarde/beef/grazing large area | | |
| --- | --- | --- | --- | --- |
|  |  | **Biophysical Cumulative share** | **Mass Cumulative share** | **Economic Cumulative share** |
| Abomasum | Human food | 0.0157 | 0.0028 | 0.0021 |
| Abomasum fat | Fat and greaves C3 | 0.0011 | 0.0005 | 0.0000 |
| Aponeurosis | Human food | 0.0250 | 0.0331 | 0.0337 |
| Bile | PAP C3 | 0.0000 | 0.0001 | 0.0000 |
| Blood | PAP C3 | 0.0191 | 0.0328 | 0.0074 |
| Blood | Pet food | 0.0027 | 0.0046 | 0.0003 |
| Bones | Gelatin C3 | 0.0465 | 0.0743 | 0.0002 |
| Bones of head, brain, eyes and teeth | C1-C2 for disposal | 0.0000 | 0.0000 | 0.0000 |
| Cheek | Human food | 0.0011 | 0.0016 | 0.0036 |
| Cheek | Human food | 0.0021 | 0.0030 | 0.0067 |
| Cheek trimmings | Pet food | 0.0006 | 0.0009 | 0.0001 |
| Chops | Pet food | 0.0022 | 0.0023 | 0.0002 |
| Contents of intestines | Spreading/Compost | 0.0000 | 0.0000 | 0.0000 |
| Contents of the rumen | Spreading/Compost | 0.0000 | 0.0000 | 0.0000 |
| Ears | PAP C3 | 0.0010 | 0.0011 | 0.0001 |
| Esophagus | Pet food | 0.0010 | 0.0015 | 0.0001 |
| Fat | Fat and greaves C3 | 0.0730 | 0.0795 | 0.0073 |
| Fat around heart | Fat and greaves C3 | 0.0028 | 0.0030 | 0.0003 |
| Fat in the kidney | Fat and greaves C3 | 0.0003 | 0.0004 | 0.0000 |
| Feet (without hooves) | Gelatin C3 | 0.0132 | 0.0196 | 0.0001 |
| Floatation fat | Spreading/Compost | 0.0000 | 0.0000 | 0.0000 |
| Forehead | C1-C2 for disposal | 0.0000 | 0.0000 | 0.0000 |
| Forelock | PAP C3 | 0.0031 | 0.0013 | 0.0001 |
| Gallbladder | Pet food | 0.0003 | 0.0005 | 0.0000 |
| Head trimmings | Pet food | 0.0028 | 0.0041 | 0.0003 |
| Heart | Human food | 0.0033 | 0.0050 | 0.0011 |
| Heart trimmings | Pet food | 0.0003 | 0.0005 | 0.0000 |
| Hide | Skin tannery C3 | 0.0738 | 0.0771 | 0.1304 |
| Hooves | PAP C3 | 0.0083 | 0.0034 | 0.0003 |
| Horns | PAP C3 | 0.0022 | 0.0009 | 0.0001 |
| Kidney | Human food | 0.0020 | 0.0030 | 0.0012 |
| Large intestine | C1-C2 for disposal | 0.0000 | 0.0000 | 0.0000 |
| Liver | Human food | 0.0587 | 0.0199 | 0.0098 |
| Liver trimmings | Pet food | 0.0069 | 0.0023 | 0.0002 |
| Lower jaw | PAP C3 | 0.0034 | 0.0055 | 0.0005 |
| Lungs | Pet food | 0.0088 | 0.0111 | 0.0008 |
| Mask | Skin tannery C3 | 0.0050 | 0.0053 | 0.0089 |
| Mesenteric fat | C1-C2 for disposal | 0.0000 | 0.0000 | 0.0000 |
| Muscle | Human food | 0.3057 | 0.4440 | 0.7520 |
| Muzzle | Human food | 0.0026 | 0.0028 | 0.0028 |
| Omasum | Human food | 0.0142 | 0.0025 | 0.0019 |
| Omasum fat | Fat and greaves C3 | 0.0035 | 0.0016 | 0.0001 |
| Rumen and forestomach | Human food | 0.0726 | 0.0128 | 0.0097 |
| Rumen fat | Fat and greaves C3 | 0.0054 | 0.0025 | 0.0002 |
| Sanitary seizures | C1-C2 for disposal | 0.0000 | 0.0000 | 0.0000 |
| Screening and sifting wastes | C1-C2 for disposal | 0.0000 | 0.0000 | 0.0000 |
| Small intestine | PAP C3 | 0.0847 | 0.0149 | 0.0009 |
| Spinal cord | C1-C2 for disposal | 0.0000 | 0.0000 | 0.0000 |
| Spinal cord waste | C1-C2 for disposal | 0.0000 | 0.0000 | 0.0000 |
| Spine | C1-C2 for disposal | 0.0000 | 0.0000 | 0.0000 |
| Spleen | Pet food | 0.0021 | 0.0030 | 0.0002 |
| Stillborn | PAP C3 | 0.0439 | 0.0074 | 0.0000 |
| Tallow | Fat and greaves C3 | 0.0702 | 0.0765 | 0.0071 |
| Tongue | Human food | 0.0026 | 0.0044 | 0.0071 |
| Tonsil | C1-C2 for disposal | 0.0000 | 0.0000 | 0.0000 |
| Trachea | Pet food | 0.0018 | 0.0019 | 0.0001 |
| Udder | Pet food | 0.0033 | 0.0234 | 0.0017 |
| Upper throat | Pet food | 0.0011 | 0.0016 | 0.0001 |
| Water in the rumen | Spreading/Compost | 0.0000 | 0.0000 | 0.0000 |

Table 101: Total weighting by coproducts for Montbéliarde Young Bulls reared in Pasture

| COPRODUCT | Destination | Montbéliarde/young bull/pasture | | |
| --- | --- | --- | --- | --- |
|  |  | **Biophysical Cumulative share** | **Mass Cumulative share** | **Economic Cumulative share** |
| Abomasum | Human food | 0.0145 | 0.0026 | 0.0020 |
| Abomasum fat | Fat and greaves C3 | 0.0011 | 0.0005 | 0.0000 |
| Aponeurosis | Human food | 0.0258 | 0.0341 | 0.0342 |
| Bile | PAP C3 | 0.0000 | 0.0001 | 0.0000 |
| Blood | PAP C3 | 0.0182 | 0.0311 | 0.0070 |
| Blood | Pet food | 0.0026 | 0.0045 | 0.0003 |
| Bones | Gelatin C3 | 0.0491 | 0.0764 | 0.0002 |
| Bones of head, brain, eyes and teeth | C1-C2 for disposal | 0.0000 | 0.0000 | 0.0000 |
| Cheek | Human food | 0.0011 | 0.0016 | 0.0035 |
| Cheek | Human food | 0.0020 | 0.0029 | 0.0063 |
| Cheek trimmings | Pet food | 0.0005 | 0.0007 | 0.0001 |
| Chops | Pet food | 0.0020 | 0.0021 | 0.0002 |
| Contents of intestines | Spreading/Compost | 0.0000 | 0.0000 | 0.0000 |
| Contents of the rumen | Spreading/Compost | 0.0000 | 0.0000 | 0.0000 |
| Ears | PAP C3 | 0.0009 | 0.0010 | 0.0001 |
| Esophagus | Pet food | 0.0010 | 0.0015 | 0.0001 |
| Fat | Fat and greaves C3 | 0.0836 | 0.0818 | 0.0074 |
| Fat around heart | Fat and greaves C3 | 0.0029 | 0.0029 | 0.0003 |
| Fat in the kidney | Fat and greaves C3 | 0.0004 | 0.0004 | 0.0000 |
| Feet (without hooves) | Gelatin C3 | 0.0127 | 0.0187 | 0.0001 |
| Floatation fat | Spreading/Compost | 0.0000 | 0.0000 | 0.0000 |
| Forehead | C1-C2 for disposal | 0.0000 | 0.0000 | 0.0000 |
| Forelock | PAP C3 | 0.0028 | 0.0011 | 0.0001 |
| Gallbladder | Pet food | 0.0003 | 0.0005 | 0.0000 |
| Head trimmings | Pet food | 0.0027 | 0.0040 | 0.0003 |
| Heart | Human food | 0.0031 | 0.0047 | 0.0010 |
| Heart trimmings | Pet food | 0.0003 | 0.0005 | 0.0000 |
| Hide | Skin tannery C3 | 0.0702 | 0.0733 | 0.1223 |
| Hooves | PAP C3 | 0.0080 | 0.0032 | 0.0003 |
| Horns | PAP C3 | 0.0018 | 0.0007 | 0.0001 |
| Kidney | Human food | 0.0019 | 0.0029 | 0.0012 |
| Large intestine | C1-C2 for disposal | 0.0000 | 0.0000 | 0.0000 |
| Liver | Human food | 0.0549 | 0.0190 | 0.0092 |
| Liver trimmings | Pet food | 0.0063 | 0.0021 | 0.0002 |
| Lower jaw | PAP C3 | 0.0033 | 0.0052 | 0.0004 |
| Lungs | Pet food | 0.0083 | 0.0105 | 0.0008 |
| Mask | Skin tannery C3 | 0.0048 | 0.0050 | 0.0083 |
| Mesenteric fat | C1-C2 for disposal | 0.0000 | 0.0000 | 0.0000 |
| Muscle | Human food | 0.3168 | 0.4569 | 0.7640 |
| Muzzle | Human food | 0.0025 | 0.0026 | 0.0026 |
| Omasum | Human food | 0.0131 | 0.0024 | 0.0018 |
| Omasum fat | Fat and greaves C3 | 0.0035 | 0.0016 | 0.0001 |
| Rumen and forestomach | Human food | 0.0675 | 0.0121 | 0.0091 |
| Rumen fat | Fat and greaves C3 | 0.0052 | 0.0024 | 0.0002 |
| Sanitary seizures | C1-C2 for disposal | 0.0000 | 0.0000 | 0.0000 |
| Screening and sifting wastes | C1-C2 for disposal | 0.0000 | 0.0000 | 0.0000 |
| Small intestine | PAP C3 | 0.0785 | 0.0141 | 0.0009 |
| Spinal cord | C1-C2 for disposal | 0.0000 | 0.0000 | 0.0000 |
| Spinal cord waste | C1-C2 for disposal | 0.0000 | 0.0000 | 0.0000 |
| Spine | C1-C2 for disposal | 0.0000 | 0.0000 | 0.0000 |
| Spleen | Pet food | 0.0020 | 0.0029 | 0.0002 |
| Stillborn | PAP C3 | 0.0410 | 0.0071 | 0.0000 |
| Tallow | Fat and greaves C3 | 0.0741 | 0.0727 | 0.0066 |
| Tongue | Human food | 0.0026 | 0.0042 | 0.0067 |
| Tonsil | C1-C2 for disposal | 0.0000 | 0.0000 | 0.0000 |
| Trachea | Pet food | 0.0018 | 0.0019 | 0.0001 |
| Udder | Pet food | 0.0033 | 0.0222 | 0.0016 |
| Upper throat | Pet food | 0.0011 | 0.0016 | 0.0001 |
| Water in the rumen | Spreading/Compost | 0.0000 | 0.0000 | 0.0000 |

Table 102: Total weighting by coproducts for Montbéliarde Heifers reared in Pasture

| COPRODUCT | Destination | Montbéliarde/heifer/pasture | | |
| --- | --- | --- | --- | --- |
|  |  | **Biophysical Cumulative share** | **Mass Cumulative share** | **Economic Cumulative share** |
| Abomasum | Human food | 0.0142 | 0.0028 | 0.0021 |
| Abomasum fat | Fat and greaves C3 | 0.0010 | 0.0005 | 0.0000 |
| Aponeurosis | Human food | 0.0249 | 0.0326 | 0.0334 |
| Bile | PAP C3 | 0.0000 | 0.0001 | 0.0000 |
| Blood | PAP C3 | 0.0198 | 0.0335 | 0.0076 |
| Blood | Pet food | 0.0028 | 0.0048 | 0.0004 |
| Bones | Gelatin C3 | 0.0471 | 0.0732 | 0.0002 |
| Bones of head, brain, eyes and teeth | C1-C2 for disposal | 0.0000 | 0.0000 | 0.0000 |
| Cheek | Human food | 0.0011 | 0.0016 | 0.0037 |
| Cheek | Human food | 0.0022 | 0.0031 | 0.0070 |
| Cheek trimmings | Pet food | 0.0006 | 0.0009 | 0.0001 |
| Chops | Pet food | 0.0022 | 0.0023 | 0.0002 |
| Contents of intestines | Spreading/Compost | 0.0000 | 0.0000 | 0.0000 |
| Contents of the rumen | Spreading/Compost | 0.0000 | 0.0000 | 0.0000 |
| Ears | PAP C3 | 0.0010 | 0.0011 | 0.0001 |
| Esophagus | Pet food | 0.0010 | 0.0015 | 0.0001 |
| Fat | Fat and greaves C3 | 0.0786 | 0.0783 | 0.0073 |
| Fat around heart | Fat and greaves C3 | 0.0031 | 0.0031 | 0.0003 |
| Fat in the kidney | Fat and greaves C3 | 0.0004 | 0.0004 | 0.0000 |
| Feet (without hooves) | Gelatin C3 | 0.0138 | 0.0202 | 0.0001 |
| Floatation fat | Spreading/Compost | 0.0000 | 0.0000 | 0.0000 |
| Forehead | C1-C2 for disposal | 0.0000 | 0.0000 | 0.0000 |
| Forelock | PAP C3 | 0.0031 | 0.0013 | 0.0001 |
| Gallbladder | Pet food | 0.0003 | 0.0005 | 0.0000 |
| Head trimmings | Pet food | 0.0030 | 0.0043 | 0.0003 |
| Heart | Human food | 0.0033 | 0.0050 | 0.0011 |
| Heart trimmings | Pet food | 0.0003 | 0.0005 | 0.0000 |
| Hide | Skin tannery C3 | 0.0766 | 0.0791 | 0.1346 |
| Hooves | PAP C3 | 0.0088 | 0.0035 | 0.0003 |
| Horns | PAP C3 | 0.0022 | 0.0009 | 0.0001 |
| Kidney | Human food | 0.0021 | 0.0031 | 0.0013 |
| Large intestine | C1-C2 for disposal | 0.0000 | 0.0000 | 0.0000 |
| Liver | Human food | 0.0555 | 0.0205 | 0.0101 |
| Liver trimmings | Pet food | 0.0064 | 0.0023 | 0.0002 |
| Lower jaw | PAP C3 | 0.0036 | 0.0056 | 0.0005 |
| Lungs | Pet food | 0.0091 | 0.0114 | 0.0009 |
| Mask | Skin tannery C3 | 0.0052 | 0.0054 | 0.0092 |
| Mesenteric fat | C1-C2 for disposal | 0.0000 | 0.0000 | 0.0000 |
| Muscle | Human food | 0.3053 | 0.4374 | 0.7457 |
| Muzzle | Human food | 0.0027 | 0.0028 | 0.0028 |
| Omasum | Human food | 0.0129 | 0.0025 | 0.0019 |
| Omasum fat | Fat and greaves C3 | 0.0034 | 0.0016 | 0.0002 |
| Rumen and forestomach | Human food | 0.0673 | 0.0131 | 0.0100 |
| Rumen fat | Fat and greaves C3 | 0.0052 | 0.0025 | 0.0002 |
| Sanitary seizures | C1-C2 for disposal | 0.0000 | 0.0000 | 0.0000 |
| Screening and sifting wastes | C1-C2 for disposal | 0.0000 | 0.0000 | 0.0000 |
| Small intestine | PAP C3 | 0.0783 | 0.0152 | 0.0009 |
| Spinal cord | C1-C2 for disposal | 0.0000 | 0.0000 | 0.0000 |
| Spinal cord waste | C1-C2 for disposal | 0.0000 | 0.0000 | 0.0000 |
| Spine | C1-C2 for disposal | 0.0000 | 0.0000 | 0.0000 |
| Spleen | Pet food | 0.0022 | 0.0031 | 0.0002 |
| Stillborn | PAP C3 | 0.0412 | 0.0077 | 0.0000 |
| Tallow | Fat and greaves C3 | 0.0786 | 0.0785 | 0.0073 |
| Tongue | Human food | 0.0028 | 0.0045 | 0.0073 |
| Tonsil | C1-C2 for disposal | 0.0000 | 0.0000 | 0.0000 |
| Trachea | Pet food | 0.0019 | 0.0020 | 0.0002 |
| Udder | Pet food | 0.0035 | 0.0240 | 0.0018 |
| Upper throat | Pet food | 0.0011 | 0.0016 | 0.0001 |
| Water in the rumen | Spreading/Compost | 0.0000 | 0.0000 | 0.0000 |

Table 103: Total weighting by coproducts for Montbéliarde Cull Cows reared in Pasture

| COPRODUCT | Destination | Montbéliarde/Cull cow/pasture | | |
| --- | --- | --- | --- | --- |
|  |  | **Biophysical Cumulative share** | **Mass Cumulative share** | **Economic Cumulative share** |
| Abomasum | Human food | 0.0153 | 0.0029 | 0.0023 |
| Abomasum fat | Fat and greaves C3 | 0.0013 | 0.0006 | 0.0001 |
| Aponeurosis | Human food | 0.0234 | 0.0316 | 0.0328 |
| Bile | PAP C3 | 0.0000 | 0.0001 | 0.0000 |
| Blood | PAP C3 | 0.0201 | 0.0352 | 0.0081 |
| Blood | Pet food | 0.0029 | 0.0051 | 0.0004 |
| Bones | Gelatin C3 | 0.0444 | 0.0709 | 0.0002 |
| Bones of head, brain, eyes and teeth | C1-C2 for disposal | 0.0000 | 0.0000 | 0.0000 |
| Cheek | Human food | 0.0012 | 0.0018 | 0.0040 |
| Cheek | Human food | 0.0022 | 0.0033 | 0.0075 |
| Cheek trimmings | Pet food | 0.0006 | 0.0009 | 0.0001 |
| Chops | Pet food | 0.0023 | 0.0024 | 0.0002 |
| Contents of intestines | Spreading/Compost | 0.0000 | 0.0000 | 0.0000 |
| Contents of the rumen | Spreading/Compost | 0.0000 | 0.0000 | 0.0000 |
| Ears | PAP C3 | 0.0010 | 0.0011 | 0.0001 |
| Esophagus | Pet food | 0.0011 | 0.0016 | 0.0001 |
| Fat | Fat and greaves C3 | 0.0749 | 0.0759 | 0.0071 |
| Fat around heart | Fat and greaves C3 | 0.0032 | 0.0033 | 0.0003 |
| Fat in the kidney | Fat and greaves C3 | 0.0005 | 0.0005 | 0.0000 |
| Feet (without hooves) | Gelatin C3 | 0.0140 | 0.0211 | 0.0001 |
| Floatation fat | Spreading/Compost | 0.0000 | 0.0000 | 0.0000 |
| Forehead | C1-C2 for disposal | 0.0000 | 0.0000 | 0.0000 |
| Forelock | PAP C3 | 0.0034 | 0.0014 | 0.0001 |
| Gallbladder | Pet food | 0.0004 | 0.0006 | 0.0000 |
| Head trimmings | Pet food | 0.0030 | 0.0044 | 0.0003 |
| Heart | Human food | 0.0034 | 0.0053 | 0.0012 |
| Heart trimmings | Pet food | 0.0004 | 0.0006 | 0.0000 |
| Hide | Skin tannery C3 | 0.0780 | 0.0830 | 0.1432 |
| Hooves | PAP C3 | 0.0089 | 0.0037 | 0.0003 |
| Horns | PAP C3 | 0.0021 | 0.0009 | 0.0001 |
| Kidney | Human food | 0.0022 | 0.0033 | 0.0014 |
| Large intestine | C1-C2 for disposal | 0.0000 | 0.0000 | 0.0000 |
| Liver | Human food | 0.0590 | 0.0215 | 0.0108 |
| Liver trimmings | Pet food | 0.0069 | 0.0024 | 0.0002 |
| Lower jaw | PAP C3 | 0.0037 | 0.0059 | 0.0005 |
| Lungs | Pet food | 0.0093 | 0.0120 | 0.0009 |
| Mask | Skin tannery C3 | 0.0052 | 0.0056 | 0.0096 |
| Mesenteric fat | C1-C2 for disposal | 0.0000 | 0.0000 | 0.0000 |
| Muscle | Human food | 0.2875 | 0.4239 | 0.7327 |
| Muzzle | Human food | 0.0027 | 0.0029 | 0.0030 |
| Omasum | Human food | 0.0139 | 0.0027 | 0.0021 |
| Omasum fat | Fat and greaves C3 | 0.0037 | 0.0018 | 0.0002 |
| Rumen and forestomach | Human food | 0.0723 | 0.0138 | 0.0107 |
| Rumen fat | Fat and greaves C3 | 0.0055 | 0.0027 | 0.0003 |
| Sanitary seizures | C1-C2 for disposal | 0.0000 | 0.0000 | 0.0000 |
| Screening and sifting wastes | C1-C2 for disposal | 0.0000 | 0.0000 | 0.0000 |
| Small intestine | PAP C3 | 0.0836 | 0.0159 | 0.0010 |
| Spinal cord | C1-C2 for disposal | 0.0000 | 0.0000 | 0.0000 |
| Spinal cord waste | C1-C2 for disposal | 0.0000 | 0.0000 | 0.0000 |
| Spine | C1-C2 for disposal | 0.0000 | 0.0000 | 0.0000 |
| Spleen | Pet food | 0.0023 | 0.0033 | 0.0002 |
| Stillborn | PAP C3 | 0.0436 | 0.0080 | 0.0000 |
| Tallow | Fat and greaves C3 | 0.0812 | 0.0824 | 0.0078 |
| Tongue | Human food | 0.0028 | 0.0047 | 0.0077 |
| Tonsil | C1-C2 for disposal | 0.0000 | 0.0000 | 0.0000 |
| Trachea | Pet food | 0.0019 | 0.0020 | 0.0002 |
| Udder | Pet food | 0.0036 | 0.0252 | 0.0019 |
| Upper throat | Pet food | 0.0012 | 0.0018 | 0.0001 |
| Water in the rumen | Spreading/Compost | 0.0000 | 0.0000 | 0.0000 |

Table 104: Total weighting by coproducts for Montbéliarde Beef reared in Pasture

| COPRODUCT | Destination | Montbéliarde/beef/pasture | | |
| --- | --- | --- | --- | --- |
|  |  | **Biophysical Cumulative share** | **Mass Cumulative share** | **Economic Cumulative share** |
| Abomasum | Human food | 0.0150 | 0.0028 | 0.0021 |
| Abomasum fat | Fat and greaves C3 | 0.0011 | 0.0005 | 0.0000 |
| Aponeurosis | Human food | 0.0248 | 0.0331 | 0.0337 |
| Bile | PAP C3 | 0.0000 | 0.0001 | 0.0000 |
| Blood | PAP C3 | 0.0189 | 0.0328 | 0.0074 |
| Blood | Pet food | 0.0027 | 0.0046 | 0.0003 |
| Bones | Gelatin C3 | 0.0472 | 0.0743 | 0.0002 |
| Bones of head, brain, eyes and teeth | C1-C2 for disposal | 0.0000 | 0.0000 | 0.0000 |
| Cheek | Human food | 0.0011 | 0.0016 | 0.0036 |
| Cheek | Human food | 0.0020 | 0.0030 | 0.0067 |
| Cheek trimmings | Pet food | 0.0006 | 0.0009 | 0.0001 |
| Chops | Pet food | 0.0021 | 0.0023 | 0.0002 |
| Contents of intestines | Spreading/Compost | 0.0000 | 0.0000 | 0.0000 |
| Contents of the rumen | Spreading/Compost | 0.0000 | 0.0000 | 0.0000 |
| Ears | PAP C3 | 0.0010 | 0.0011 | 0.0001 |
| Esophagus | Pet food | 0.0010 | 0.0015 | 0.0001 |
| Fat | Fat and greaves C3 | 0.0801 | 0.0795 | 0.0073 |
| Fat around heart | Fat and greaves C3 | 0.0030 | 0.0030 | 0.0003 |
| Fat in the kidney | Fat and greaves C3 | 0.0004 | 0.0004 | 0.0000 |
| Feet (without hooves) | Gelatin C3 | 0.0132 | 0.0196 | 0.0001 |
| Floatation fat | Spreading/Compost | 0.0000 | 0.0000 | 0.0000 |
| Forehead | C1-C2 for disposal | 0.0000 | 0.0000 | 0.0000 |
| Forelock | PAP C3 | 0.0031 | 0.0013 | 0.0001 |
| Gallbladder | Pet food | 0.0003 | 0.0005 | 0.0000 |
| Head trimmings | Pet food | 0.0028 | 0.0041 | 0.0003 |
| Heart | Human food | 0.0033 | 0.0050 | 0.0011 |
| Heart trimmings | Pet food | 0.0003 | 0.0005 | 0.0000 |
| Hide | Skin tannery C3 | 0.0732 | 0.0771 | 0.1304 |
| Hooves | PAP C3 | 0.0082 | 0.0034 | 0.0003 |
| Horns | PAP C3 | 0.0021 | 0.0009 | 0.0001 |
| Kidney | Human food | 0.0020 | 0.0030 | 0.0012 |
| Large intestine | C1-C2 for disposal | 0.0000 | 0.0000 | 0.0000 |
| Liver | Human food | 0.0564 | 0.0199 | 0.0098 |
| Liver trimmings | Pet food | 0.0067 | 0.0023 | 0.0002 |
| Lower jaw | PAP C3 | 0.0034 | 0.0055 | 0.0005 |
| Lungs | Pet food | 0.0087 | 0.0111 | 0.0008 |
| Mask | Skin tannery C3 | 0.0050 | 0.0053 | 0.0089 |
| Mesenteric fat | C1-C2 for disposal | 0.0000 | 0.0000 | 0.0000 |
| Muscle | Human food | 0.3047 | 0.4440 | 0.7520 |
| Muzzle | Human food | 0.0026 | 0.0028 | 0.0028 |
| Omasum | Human food | 0.0136 | 0.0025 | 0.0019 |
| Omasum fat | Fat and greaves C3 | 0.0035 | 0.0016 | 0.0001 |
| Rumen and forestomach | Human food | 0.0694 | 0.0128 | 0.0097 |
| Rumen fat | Fat and greaves C3 | 0.0054 | 0.0025 | 0.0002 |
| Sanitary seizures | C1-C2 for disposal | 0.0000 | 0.0000 | 0.0000 |
| Screening and sifting wastes | C1-C2 for disposal | 0.0000 | 0.0000 | 0.0000 |
| Small intestine | PAP C3 | 0.0810 | 0.0149 | 0.0009 |
| Spinal cord | C1-C2 for disposal | 0.0000 | 0.0000 | 0.0000 |
| Spinal cord waste | C1-C2 for disposal | 0.0000 | 0.0000 | 0.0000 |
| Spine | C1-C2 for disposal | 0.0000 | 0.0000 | 0.0000 |
| Spleen | Pet food | 0.0021 | 0.0030 | 0.0002 |
| Stillborn | PAP C3 | 0.0419 | 0.0074 | 0.0000 |
| Tallow | Fat and greaves C3 | 0.0770 | 0.0765 | 0.0071 |
| Tongue | Human food | 0.0026 | 0.0044 | 0.0071 |
| Tonsil | C1-C2 for disposal | 0.0000 | 0.0000 | 0.0000 |
| Trachea | Pet food | 0.0018 | 0.0019 | 0.0001 |
| Udder | Pet food | 0.0034 | 0.0234 | 0.0017 |
| Upper throat | Pet food | 0.0011 | 0.0016 | 0.0001 |
| Water in the rumen | Spreading/Compost | 0.0000 | 0.0000 | 0.0000 |

Table 105: Total weighting by coproducts for Montbéliarde Young Bulls reared in Stall

| COPRODUCT | Destination | Montbéliarde/young bull/stall | | |
| --- | --- | --- | --- | --- |
|  |  | **Biophysical Cumulative share** | **Mass Cumulative share** | **Economic Cumulative share** |
| Abomasum | Human food | 0.0137 | 0.0026 | 0.0020 |
| Abomasum fat | Fat and greaves C3 | 0.0011 | 0.0005 | 0.0000 |
| Aponeurosis | Human food | 0.0255 | 0.0341 | 0.0342 |
| Bile | PAP C3 | 0.0000 | 0.0001 | 0.0000 |
| Blood | PAP C3 | 0.0180 | 0.0311 | 0.0070 |
| Blood | Pet food | 0.0026 | 0.0045 | 0.0003 |
| Bones | Gelatin C3 | 0.0499 | 0.0764 | 0.0002 |
| Bones of head, brain, eyes and teeth | C1-C2 for disposal | 0.0000 | 0.0000 | 0.0000 |
| Cheek | Human food | 0.0011 | 0.0016 | 0.0035 |
| Cheek | Human food | 0.0019 | 0.0029 | 0.0063 |
| Cheek trimmings | Pet food | 0.0005 | 0.0007 | 0.0001 |
| Chops | Pet food | 0.0020 | 0.0021 | 0.0002 |
| Contents of intestines | Spreading/Compost | 0.0000 | 0.0000 | 0.0000 |
| Contents of the rumen | Spreading/Compost | 0.0000 | 0.0000 | 0.0000 |
| Ears | PAP C3 | 0.0009 | 0.0010 | 0.0001 |
| Esophagus | Pet food | 0.0010 | 0.0015 | 0.0001 |
| Fat | Fat and greaves C3 | 0.0916 | 0.0818 | 0.0074 |
| Fat around heart | Fat and greaves C3 | 0.0032 | 0.0029 | 0.0003 |
| Fat in the kidney | Fat and greaves C3 | 0.0004 | 0.0004 | 0.0000 |
| Feet (without hooves) | Gelatin C3 | 0.0127 | 0.0187 | 0.0001 |
| Floatation fat | Spreading/Compost | 0.0000 | 0.0000 | 0.0000 |
| Forehead | C1-C2 for disposal | 0.0000 | 0.0000 | 0.0000 |
| Forelock | PAP C3 | 0.0027 | 0.0011 | 0.0001 |
| Gallbladder | Pet food | 0.0003 | 0.0005 | 0.0000 |
| Head trimmings | Pet food | 0.0027 | 0.0040 | 0.0003 |
| Heart | Human food | 0.0031 | 0.0047 | 0.0010 |
| Heart trimmings | Pet food | 0.0003 | 0.0005 | 0.0000 |
| Hide | Skin tannery C3 | 0.0695 | 0.0733 | 0.1223 |
| Hooves | PAP C3 | 0.0078 | 0.0032 | 0.0003 |
| Horns | PAP C3 | 0.0018 | 0.0007 | 0.0001 |
| Kidney | Human food | 0.0019 | 0.0029 | 0.0012 |
| Large intestine | C1-C2 for disposal | 0.0000 | 0.0000 | 0.0000 |
| Liver | Human food | 0.0524 | 0.0190 | 0.0092 |
| Liver trimmings | Pet food | 0.0061 | 0.0021 | 0.0002 |
| Lower jaw | PAP C3 | 0.0033 | 0.0052 | 0.0004 |
| Lungs | Pet food | 0.0082 | 0.0105 | 0.0008 |
| Mask | Skin tannery C3 | 0.0047 | 0.0050 | 0.0083 |
| Mesenteric fat | C1-C2 for disposal | 0.0000 | 0.0000 | 0.0000 |
| Muscle | Human food | 0.3154 | 0.4569 | 0.7640 |
| Muzzle | Human food | 0.0025 | 0.0026 | 0.0026 |
| Omasum | Human food | 0.0124 | 0.0024 | 0.0018 |
| Omasum fat | Fat and greaves C3 | 0.0036 | 0.0016 | 0.0001 |
| Rumen and forestomach | Human food | 0.0641 | 0.0121 | 0.0091 |
| Rumen fat | Fat and greaves C3 | 0.0052 | 0.0024 | 0.0002 |
| Sanitary seizures | C1-C2 for disposal | 0.0000 | 0.0000 | 0.0000 |
| Screening and sifting wastes | C1-C2 for disposal | 0.0000 | 0.0000 | 0.0000 |
| Small intestine | PAP C3 | 0.0746 | 0.0141 | 0.0009 |
| Spinal cord | C1-C2 for disposal | 0.0000 | 0.0000 | 0.0000 |
| Spinal cord waste | C1-C2 for disposal | 0.0000 | 0.0000 | 0.0000 |
| Spine | C1-C2 for disposal | 0.0000 | 0.0000 | 0.0000 |
| Spleen | Pet food | 0.0020 | 0.0029 | 0.0002 |
| Stillborn | PAP C3 | 0.0389 | 0.0071 | 0.0000 |
| Tallow | Fat and greaves C3 | 0.0813 | 0.0727 | 0.0066 |
| Tongue | Human food | 0.0026 | 0.0042 | 0.0067 |
| Tonsil | C1-C2 for disposal | 0.0000 | 0.0000 | 0.0000 |
| Trachea | Pet food | 0.0018 | 0.0019 | 0.0001 |
| Udder | Pet food | 0.0034 | 0.0222 | 0.0016 |
| Upper throat | Pet food | 0.0011 | 0.0016 | 0.0001 |
| Water in the rumen | Spreading/Compost | 0.0000 | 0.0000 | 0.0000 |

Table 106: Total weighting by coproducts for Montbéliarde Heifers reared in Stall

| COPRODUCT | Destination | Montbéliarde/heifer/stall | | |
| --- | --- | --- | --- | --- |
|  |  | **Biophysical Cumulative share** | **Mass Cumulative share** | **Economic Cumulative share** |
| Abomasum | Human food | 0.0135 | 0.0028 | 0.0021 |
| Abomasum fat | Fat and greaves C3 | 0.0010 | 0.0005 | 0.0000 |
| Aponeurosis | Human food | 0.0247 | 0.0326 | 0.0334 |
| Bile | PAP C3 | 0.0000 | 0.0001 | 0.0000 |
| Blood | PAP C3 | 0.0197 | 0.0335 | 0.0076 |
| Blood | Pet food | 0.0028 | 0.0048 | 0.0004 |
| Bones | Gelatin C3 | 0.0479 | 0.0732 | 0.0002 |
| Bones of head, brain, eyes and teeth | C1-C2 for disposal | 0.0000 | 0.0000 | 0.0000 |
| Cheek | Human food | 0.0011 | 0.0016 | 0.0037 |
| Cheek | Human food | 0.0022 | 0.0031 | 0.0070 |
| Cheek trimmings | Pet food | 0.0006 | 0.0009 | 0.0001 |
| Chops | Pet food | 0.0022 | 0.0023 | 0.0002 |
| Contents of intestines | Spreading/Compost | 0.0000 | 0.0000 | 0.0000 |
| Contents of the rumen | Spreading/Compost | 0.0000 | 0.0000 | 0.0000 |
| Ears | PAP C3 | 0.0010 | 0.0011 | 0.0001 |
| Esophagus | Pet food | 0.0010 | 0.0015 | 0.0001 |
| Fat | Fat and greaves C3 | 0.0857 | 0.0783 | 0.0073 |
| Fat around heart | Fat and greaves C3 | 0.0034 | 0.0031 | 0.0003 |
| Fat in the kidney | Fat and greaves C3 | 0.0004 | 0.0004 | 0.0000 |
| Feet (without hooves) | Gelatin C3 | 0.0138 | 0.0202 | 0.0001 |
| Floatation fat | Spreading/Compost | 0.0000 | 0.0000 | 0.0000 |
| Forehead | C1-C2 for disposal | 0.0000 | 0.0000 | 0.0000 |
| Forelock | PAP C3 | 0.0031 | 0.0013 | 0.0001 |
| Gallbladder | Pet food | 0.0003 | 0.0005 | 0.0000 |
| Head trimmings | Pet food | 0.0030 | 0.0043 | 0.0003 |
| Heart | Human food | 0.0033 | 0.0050 | 0.0011 |
| Heart trimmings | Pet food | 0.0003 | 0.0005 | 0.0000 |
| Hide | Skin tannery C3 | 0.0762 | 0.0791 | 0.1346 |
| Hooves | PAP C3 | 0.0087 | 0.0035 | 0.0003 |
| Horns | PAP C3 | 0.0022 | 0.0009 | 0.0001 |
| Kidney | Human food | 0.0021 | 0.0031 | 0.0013 |
| Large intestine | C1-C2 for disposal | 0.0000 | 0.0000 | 0.0000 |
| Liver | Human food | 0.0529 | 0.0205 | 0.0101 |
| Liver trimmings | Pet food | 0.0061 | 0.0023 | 0.0002 |
| Lower jaw | PAP C3 | 0.0036 | 0.0056 | 0.0005 |
| Lungs | Pet food | 0.0091 | 0.0114 | 0.0009 |
| Mask | Skin tannery C3 | 0.0052 | 0.0054 | 0.0092 |
| Mesenteric fat | C1-C2 for disposal | 0.0000 | 0.0000 | 0.0000 |
| Muscle | Human food | 0.3050 | 0.4374 | 0.7457 |
| Muzzle | Human food | 0.0027 | 0.0028 | 0.0028 |
| Omasum | Human food | 0.0122 | 0.0025 | 0.0019 |
| Omasum fat | Fat and greaves C3 | 0.0034 | 0.0016 | 0.0002 |
| Rumen and forestomach | Human food | 0.0636 | 0.0131 | 0.0100 |
| Rumen fat | Fat and greaves C3 | 0.0052 | 0.0025 | 0.0002 |
| Sanitary seizures | C1-C2 for disposal | 0.0000 | 0.0000 | 0.0000 |
| Screening and sifting wastes | C1-C2 for disposal | 0.0000 | 0.0000 | 0.0000 |
| Small intestine | PAP C3 | 0.0741 | 0.0152 | 0.0009 |
| Spinal cord | C1-C2 for disposal | 0.0000 | 0.0000 | 0.0000 |
| Spinal cord waste | C1-C2 for disposal | 0.0000 | 0.0000 | 0.0000 |
| Spine | C1-C2 for disposal | 0.0000 | 0.0000 | 0.0000 |
| Spleen | Pet food | 0.0022 | 0.0031 | 0.0002 |
| Stillborn | PAP C3 | 0.0389 | 0.0077 | 0.0000 |
| Tallow | Fat and greaves C3 | 0.0858 | 0.0785 | 0.0073 |
| Tongue | Human food | 0.0028 | 0.0045 | 0.0073 |
| Tonsil | C1-C2 for disposal | 0.0000 | 0.0000 | 0.0000 |
| Trachea | Pet food | 0.0019 | 0.0020 | 0.0002 |
| Udder | Pet food | 0.0037 | 0.0240 | 0.0018 |
| Upper throat | Pet food | 0.0011 | 0.0016 | 0.0001 |
| Water in the rumen | Spreading/Compost | 0.0000 | 0.0000 | 0.0000 |

Table 107: Total weighting by coproducts for Montbéliarde Cull Cows reared in Stall

| COPRODUCT | Destination | Montbéliarde/Cull cow/stall | | |
| --- | --- | --- | --- | --- |
|  |  | **Biophysical Cumulative share** | **Mass Cumulative share** | **Economic Cumulative share** |
| Abomasum | Human food | 0.0145 | 0.0029 | 0.0023 |
| Abomasum fat | Fat and greaves C3 | 0.0013 | 0.0006 | 0.0001 |
| Aponeurosis | Human food | 0.0233 | 0.0316 | 0.0328 |
| Bile | PAP C3 | 0.0000 | 0.0001 | 0.0000 |
| Blood | PAP C3 | 0.0200 | 0.0352 | 0.0081 |
| Blood | Pet food | 0.0029 | 0.0051 | 0.0004 |
| Bones | Gelatin C3 | 0.0452 | 0.0709 | 0.0002 |
| Bones of head, brain, eyes and teeth | C1-C2 for disposal | 0.0000 | 0.0000 | 0.0000 |
| Cheek | Human food | 0.0012 | 0.0018 | 0.0040 |
| Cheek | Human food | 0.0022 | 0.0033 | 0.0075 |
| Cheek trimmings | Pet food | 0.0006 | 0.0009 | 0.0001 |
| Chops | Pet food | 0.0022 | 0.0024 | 0.0002 |
| Contents of intestines | Spreading/Compost | 0.0000 | 0.0000 | 0.0000 |
| Contents of the rumen | Spreading/Compost | 0.0000 | 0.0000 | 0.0000 |
| Ears | PAP C3 | 0.0010 | 0.0011 | 0.0001 |
| Esophagus | Pet food | 0.0011 | 0.0016 | 0.0001 |
| Fat | Fat and greaves C3 | 0.0820 | 0.0759 | 0.0071 |
| Fat around heart | Fat and greaves C3 | 0.0036 | 0.0033 | 0.0003 |
| Fat in the kidney | Fat and greaves C3 | 0.0005 | 0.0005 | 0.0000 |
| Feet (without hooves) | Gelatin C3 | 0.0140 | 0.0211 | 0.0001 |
| Floatation fat | Spreading/Compost | 0.0000 | 0.0000 | 0.0000 |
| Forehead | C1-C2 for disposal | 0.0000 | 0.0000 | 0.0000 |
| Forelock | PAP C3 | 0.0033 | 0.0014 | 0.0001 |
| Gallbladder | Pet food | 0.0004 | 0.0006 | 0.0000 |
| Head trimmings | Pet food | 0.0030 | 0.0044 | 0.0003 |
| Heart | Human food | 0.0034 | 0.0053 | 0.0012 |
| Heart trimmings | Pet food | 0.0004 | 0.0006 | 0.0000 |
| Hide | Skin tannery C3 | 0.0774 | 0.0830 | 0.1432 |
| Hooves | PAP C3 | 0.0088 | 0.0037 | 0.0003 |
| Horns | PAP C3 | 0.0021 | 0.0009 | 0.0001 |
| Kidney | Human food | 0.0022 | 0.0033 | 0.0014 |
| Large intestine | C1-C2 for disposal | 0.0000 | 0.0000 | 0.0000 |
| Liver | Human food | 0.0564 | 0.0215 | 0.0108 |
| Liver trimmings | Pet food | 0.0066 | 0.0024 | 0.0002 |
| Lower jaw | PAP C3 | 0.0037 | 0.0059 | 0.0005 |
| Lungs | Pet food | 0.0092 | 0.0120 | 0.0009 |
| Mask | Skin tannery C3 | 0.0052 | 0.0056 | 0.0096 |
| Mesenteric fat | C1-C2 for disposal | 0.0000 | 0.0000 | 0.0000 |
| Muscle | Human food | 0.2870 | 0.4239 | 0.7327 |
| Muzzle | Human food | 0.0027 | 0.0029 | 0.0030 |
| Omasum | Human food | 0.0132 | 0.0027 | 0.0021 |
| Omasum fat | Fat and greaves C3 | 0.0037 | 0.0018 | 0.0002 |
| Rumen and forestomach | Human food | 0.0686 | 0.0138 | 0.0107 |
| Rumen fat | Fat and greaves C3 | 0.0056 | 0.0027 | 0.0003 |
| Sanitary seizures | C1-C2 for disposal | 0.0000 | 0.0000 | 0.0000 |
| Screening and sifting wastes | C1-C2 for disposal | 0.0000 | 0.0000 | 0.0000 |
| Small intestine | PAP C3 | 0.0793 | 0.0159 | 0.0010 |
| Spinal cord | C1-C2 for disposal | 0.0000 | 0.0000 | 0.0000 |
| Spinal cord waste | C1-C2 for disposal | 0.0000 | 0.0000 | 0.0000 |
| Spine | C1-C2 for disposal | 0.0000 | 0.0000 | 0.0000 |
| Spleen | Pet food | 0.0022 | 0.0033 | 0.0002 |
| Stillborn | PAP C3 | 0.0413 | 0.0080 | 0.0000 |
| Tallow | Fat and greaves C3 | 0.0889 | 0.0824 | 0.0078 |
| Tongue | Human food | 0.0028 | 0.0047 | 0.0077 |
| Tonsil | C1-C2 for disposal | 0.0000 | 0.0000 | 0.0000 |
| Trachea | Pet food | 0.0019 | 0.0020 | 0.0002 |
| Udder | Pet food | 0.0037 | 0.0252 | 0.0019 |
| Upper throat | Pet food | 0.0012 | 0.0018 | 0.0001 |
| Water in the rumen | Spreading/Compost | 0.0000 | 0.0000 | 0.0000 |

Table 108: Total weighting by coproducts for Montbéliarde Beef reared in Stall

| COPRODUCT | Destination | Montbéliarde/beef/stall | | |
| --- | --- | --- | --- | --- |
|  |  | **Biophysical Cumulative share** | **Mass Cumulative share** | **Economic Cumulative share** |
| Abomasum | Human food | 0.0142 | 0.0028 | 0.0021 |
| Abomasum fat | Fat and greaves C3 | 0.0011 | 0.0005 | 0.0000 |
| Aponeurosis | Human food | 0.0246 | 0.0331 | 0.0337 |
| Bile | PAP C3 | 0.0000 | 0.0001 | 0.0000 |
| Blood | PAP C3 | 0.0188 | 0.0328 | 0.0074 |
| Blood | Pet food | 0.0027 | 0.0046 | 0.0003 |
| Bones | Gelatin C3 | 0.0480 | 0.0743 | 0.0002 |
| Bones of head, brain, eyes and teeth | C1-C2 for disposal | 0.0000 | 0.0000 | 0.0000 |
| Cheek | Human food | 0.0011 | 0.0016 | 0.0036 |
| Cheek | Human food | 0.0020 | 0.0030 | 0.0067 |
| Cheek trimmings | Pet food | 0.0006 | 0.0009 | 0.0001 |
| Chops | Pet food | 0.0021 | 0.0023 | 0.0002 |
| Contents of intestines | Spreading/Compost | 0.0000 | 0.0000 | 0.0000 |
| Contents of the rumen | Spreading/Compost | 0.0000 | 0.0000 | 0.0000 |
| Ears | PAP C3 | 0.0010 | 0.0011 | 0.0001 |
| Esophagus | Pet food | 0.0010 | 0.0015 | 0.0001 |
| Fat | Fat and greaves C3 | 0.0878 | 0.0795 | 0.0073 |
| Fat around heart | Fat and greaves C3 | 0.0033 | 0.0030 | 0.0003 |
| Fat in the kidney | Fat and greaves C3 | 0.0004 | 0.0004 | 0.0000 |
| Feet (without hooves) | Gelatin C3 | 0.0132 | 0.0196 | 0.0001 |
| Floatation fat | Spreading/Compost | 0.0000 | 0.0000 | 0.0000 |
| Forehead | C1-C2 for disposal | 0.0000 | 0.0000 | 0.0000 |
| Forelock | PAP C3 | 0.0030 | 0.0013 | 0.0001 |
| Gallbladder | Pet food | 0.0003 | 0.0005 | 0.0000 |
| Head trimmings | Pet food | 0.0028 | 0.0041 | 0.0003 |
| Heart | Human food | 0.0032 | 0.0050 | 0.0011 |
| Heart trimmings | Pet food | 0.0003 | 0.0005 | 0.0000 |
| Hide | Skin tannery C3 | 0.0725 | 0.0771 | 0.1304 |
| Hooves | PAP C3 | 0.0081 | 0.0034 | 0.0003 |
| Horns | PAP C3 | 0.0021 | 0.0009 | 0.0001 |
| Kidney | Human food | 0.0020 | 0.0030 | 0.0012 |
| Large intestine | C1-C2 for disposal | 0.0000 | 0.0000 | 0.0000 |
| Liver | Human food | 0.0539 | 0.0199 | 0.0098 |
| Liver trimmings | Pet food | 0.0064 | 0.0023 | 0.0002 |
| Lower jaw | PAP C3 | 0.0035 | 0.0055 | 0.0005 |
| Lungs | Pet food | 0.0086 | 0.0111 | 0.0008 |
| Mask | Skin tannery C3 | 0.0049 | 0.0053 | 0.0089 |
| Mesenteric fat | C1-C2 for disposal | 0.0000 | 0.0000 | 0.0000 |
| Muscle | Human food | 0.3036 | 0.4440 | 0.7520 |
| Muzzle | Human food | 0.0026 | 0.0028 | 0.0028 |
| Omasum | Human food | 0.0129 | 0.0025 | 0.0019 |
| Omasum fat | Fat and greaves C3 | 0.0035 | 0.0016 | 0.0001 |
| Rumen and forestomach | Human food | 0.0660 | 0.0128 | 0.0097 |
| Rumen fat | Fat and greaves C3 | 0.0054 | 0.0025 | 0.0002 |
| Sanitary seizures | C1-C2 for disposal | 0.0000 | 0.0000 | 0.0000 |
| Screening and sifting wastes | C1-C2 for disposal | 0.0000 | 0.0000 | 0.0000 |
| Small intestine | PAP C3 | 0.0770 | 0.0149 | 0.0009 |
| Spinal cord | C1-C2 for disposal | 0.0000 | 0.0000 | 0.0000 |
| Spinal cord waste | C1-C2 for disposal | 0.0000 | 0.0000 | 0.0000 |
| Spine | C1-C2 for disposal | 0.0000 | 0.0000 | 0.0000 |
| Spleen | Pet food | 0.0021 | 0.0030 | 0.0002 |
| Stillborn | PAP C3 | 0.0398 | 0.0074 | 0.0000 |
| Tallow | Fat and greaves C3 | 0.0844 | 0.0765 | 0.0071 |
| Tongue | Human food | 0.0027 | 0.0044 | 0.0071 |
| Tonsil | C1-C2 for disposal | 0.0000 | 0.0000 | 0.0000 |
| Trachea | Pet food | 0.0018 | 0.0019 | 0.0001 |
| Udder | Pet food | 0.0035 | 0.0234 | 0.0017 |
| Upper throat | Pet food | 0.0011 | 0.0016 | 0.0001 |
| Water in the rumen | Spreading/Compost | 0.0000 | 0.0000 | 0.0000 |

Table 109: Total weighting by coproducts for Charolaise x Pie Noire Young Bulls reared in Grazing Large Area

| COPRODUCT | Destination | Charolaise x Pie Noire/young bull/grazing large area | | |
| --- | --- | --- | --- | --- |
|  |  | **Biophysical Cumulative share** | **Mass Cumulative share** | **Economic Cumulative share** |
| Abomasum | Human food | 0.0154 | 0.0026 | 0.0019 |
| Abomasum fat | Fat and greaves C3 | 0.0011 | 0.0005 | 0.0000 |
| Aponeurosis | Human food | 0.0265 | 0.0346 | 0.0345 |
| Bile | PAP C3 | 0.0000 | 0.0001 | 0.0000 |
| Blood | PAP C3 | 0.0178 | 0.0303 | 0.0067 |
| Blood | Pet food | 0.0025 | 0.0043 | 0.0003 |
| Bones | Gelatin C3 | 0.0491 | 0.0776 | 0.0002 |
| Bones of head, brain, eyes and teeth | C1-C2 for disposal | 0.0000 | 0.0000 | 0.0000 |
| Cheek | Human food | 0.0010 | 0.0015 | 0.0032 |
| Cheek | Human food | 0.0020 | 0.0028 | 0.0062 |
| Cheek trimmings | Pet food | 0.0005 | 0.0007 | 0.0001 |
| Chops | Pet food | 0.0019 | 0.0020 | 0.0001 |
| Contents of intestines | Spreading/Compost | 0.0000 | 0.0000 | 0.0000 |
| Contents of the rumen | Spreading/Compost | 0.0000 | 0.0000 | 0.0000 |
| Ears | PAP C3 | 0.0008 | 0.0010 | 0.0001 |
| Esophagus | Pet food | 0.0009 | 0.0014 | 0.0001 |
| Fat | Fat and greaves C3 | 0.0766 | 0.0830 | 0.0075 |
| Fat around heart | Fat and greaves C3 | 0.0026 | 0.0028 | 0.0003 |
| Fat in the kidney | Fat and greaves C3 | 0.0003 | 0.0004 | 0.0000 |
| Feet (without hooves) | Gelatin C3 | 0.0124 | 0.0182 | 0.0001 |
| Floatation fat | Spreading/Compost | 0.0000 | 0.0000 | 0.0000 |
| Forehead | C1-C2 for disposal | 0.0000 | 0.0000 | 0.0000 |
| Forelock | PAP C3 | 0.0028 | 0.0011 | 0.0001 |
| Gallbladder | Pet food | 0.0003 | 0.0005 | 0.0000 |
| Head trimmings | Pet food | 0.0027 | 0.0038 | 0.0003 |
| Heart | Human food | 0.0030 | 0.0046 | 0.0010 |
| Heart trimmings | Pet food | 0.0003 | 0.0005 | 0.0000 |
| Hide | Skin tannery C3 | 0.0692 | 0.0714 | 0.1184 |
| Hooves | PAP C3 | 0.0080 | 0.0032 | 0.0003 |
| Horns | PAP C3 | 0.0019 | 0.0007 | 0.0001 |
| Kidney | Human food | 0.0019 | 0.0028 | 0.0011 |
| Large intestine | C1-C2 for disposal | 0.0000 | 0.0000 | 0.0000 |
| Liver | Human food | 0.0566 | 0.0184 | 0.0089 |
| Liver trimmings | Pet food | 0.0063 | 0.0020 | 0.0001 |
| Lower jaw | PAP C3 | 0.0031 | 0.0051 | 0.0004 |
| Lungs | Pet food | 0.0082 | 0.0103 | 0.0007 |
| Mask | Skin tannery C3 | 0.0047 | 0.0048 | 0.0080 |
| Mesenteric fat | C1-C2 for disposal | 0.0000 | 0.0000 | 0.0000 |
| Muscle | Human food | 0.3235 | 0.4634 | 0.7699 |
| Muzzle | Human food | 0.0025 | 0.0026 | 0.0026 |
| Omasum | Human food | 0.0140 | 0.0023 | 0.0017 |
| Omasum fat | Fat and greaves C3 | 0.0033 | 0.0015 | 0.0001 |
| Rumen and forestomach | Human food | 0.0706 | 0.0119 | 0.0088 |
| Rumen fat | Fat and greaves C3 | 0.0052 | 0.0023 | 0.0002 |
| Sanitary seizures | C1-C2 for disposal | 0.0000 | 0.0000 | 0.0000 |
| Screening and sifting wastes | C1-C2 for disposal | 0.0000 | 0.0000 | 0.0000 |
| Small intestine | PAP C3 | 0.0816 | 0.0137 | 0.0008 |
| Spinal cord | C1-C2 for disposal | 0.0000 | 0.0000 | 0.0000 |
| Spinal cord waste | C1-C2 for disposal | 0.0000 | 0.0000 | 0.0000 |
| Spine | C1-C2 for disposal | 0.0000 | 0.0000 | 0.0000 |
| Spleen | Pet food | 0.0020 | 0.0028 | 0.0002 |
| Stillborn | PAP C3 | 0.0430 | 0.0069 | 0.0000 |
| Tallow | Fat and greaves C3 | 0.0652 | 0.0708 | 0.0064 |
| Tongue | Human food | 0.0025 | 0.0041 | 0.0065 |
| Tonsil | C1-C2 for disposal | 0.0000 | 0.0000 | 0.0000 |
| Trachea | Pet food | 0.0017 | 0.0017 | 0.0001 |
| Udder | Pet food | 0.0031 | 0.0216 | 0.0016 |
| Upper throat | Pet food | 0.0010 | 0.0015 | 0.0001 |
| Water in the rumen | Spreading/Compost | 0.0000 | 0.0000 | 0.0000 |
[truncated: 84,481 more chars]
