# Supplementary material for: Allocation factors for meat coproducts: Dataset to perform life cycle assessment at slaughterhouse
Source: Data Brief. 2020 Nov 23;33:106558. doi: 10.1016/j.dib.2020.106558 (PMC7718151; doi:10.1016/j.dib.2020.106558)
Supplement: Supplementary file 3 [file mmc3.docx]

Table 1: Total weighting by destination category for Normande Young Bulls reared in grazing large area

| destination | Normande/YOUNG BULL/grazing large area | | |
| --- | --- | --- | --- |
|  | **Biophysical Partition** | **Mass Partition** | **Economic Partition** |
| Pet Food | 0,0326 | 0,055 | 0,004 |
| PAP C3 | 0,1597 | 0,0636 | 0,0089 |
| Gelatin C3 | 0,0612 | 0,0951 | 0,0003 |
| C1-C2 for disposal | 0 | 0 | 0 |
| Skin tannery C3 | 0,0758 | 0,0783 | 0,1306 |
| Human food | 0,5137 | 0,546 | 0,8416 |
| Fat and greaves C3 | 0,157 | 0,1623 | 0,0146 |
| Spreading/Compost | 0 | 0 | 0 |
|  |  |  |  |

Table 2: Total weighting by destination category for Normande Heifers reared in grazing large area

| destination | Normande/HEIFER/grazing large area | | |
| --- | --- | --- | --- |
|  | **Biophysical Partition** | **Mass Partition** | **Economic Partition** |
| Pet Food | 0,0346 | 0,0592 | 0,0045 |
| PAP C3 | 0,1663 | 0,0689 | 0,0096 |
| Gelatin C3 | 0,0596 | 0,0934 | 0,0003 |
| C1-C2 for disposal | 0 | 0 | 0 |
| Skin tannery C3 | 0,0811 | 0,0845 | 0,1438 |
| Human food | 0,5011 | 0,529 | 0,8264 |
| Fat and greaves C3 | 0,1572 | 0,1649 | 0,0153 |
| Spreading/Compost | 0 | 0 | 0 |
|  |  |  |  |

Table 3: Total weighting by destination category for Normande Cull Cows reared in grazing large area

| destination | Normande/Cull Cows/grazing large area | | |
| --- | --- | --- | --- |
|  | **Biophysical Partition** | **Mass Partition** | **Economic Partition** |
| Pet Food | 0,0361 | 0,0623 | 0,0046 |
| PAP C3 | 0,175 | 0,0722 | 0,0102 |
| Gelatin C3 | 0,0572 | 0,092 | 0,0003 |
| C1-C2 for disposal | 0 | 0 | 0 |
| Skin tannery C3 | 0,0829 | 0,0886 | 0,1528 |
| Human food | 0,4942 | 0,5177 | 0,8162 |
| Fat and greaves C3 | 0,1545 | 0,1672 | 0,0158 |
| Spreading/Compost | 0 | 0 | 0 |

Table 4: Total weighting by destination category for Normande Beef reared in grazing large area

| destination | Normande/Beef/grazing large area | | |
| --- | --- | --- | --- |
|  | **Biophysical Partition** | **Mass Partition** | **Economic Partition** |
| Pet Food | 0,0339 | 0,0577 | 0,0041 |
| PAP C3 | 0,1681 | 0,0674 | 0,0094 |
| Gelatin C3 | 0,0592 | 0,0939 | 0,0003 |
| C1-C2 for disposal | 0 | 0 | 0 |
| Skin tannery C3 | 0,0784 | 0,0824 | 0,1393 |
| Human food | 0,5068 | 0,5349 | 0,8317 |
| Fat and greaves C3 | 0,1535 | 0,164 | 0,015 |
| Spreading/Compost | 0 | 0 | 0 |

Table 5: Total weighting by destination category for Normande Young Bulls reared in pasture

| destination | Normande/YOUNG BULL/pasture | | |
| --- | --- | --- | --- |
|  | **Biophysical Partition** | **Mass Partition** | **Economic Partition** |
| Pet Food | 0,0323 | 0,055 | 0,004 |
| PAP C3 | 0,154 | 0,0636 | 0,0089 |
| Gelatin C3 | 0,0619 | 0,0951 | 0,0003 |
| C1-C2 for disposal | 0 | 0 | 0 |
| Skin tannery C3 | 0,0751 | 0,0783 | 0,1306 |
| Human food | 0,5054 | 0,546 | 0,8416 |
| Fat and greaves C3 | 0,1712 | 0,1623 | 0,0146 |
| Spreading/Compost | 0 | 0 | 0 |

Table 6: Total weighting by destination category for Normande Heifers reared in pasture

| destination | Normande/HEIFER/pasture | | |
| --- | --- | --- | --- |
|  | **Biophysical Partition** | **Mass Partition** | **Economic Partition** |
| Pet Food | 0,0343 | 0,0592 | 0,0045 |
| PAP C3 | 0,1603 | 0,0689 | 0,0096 |
| Gelatin C3 | 0,0603 | 0,0934 | 0,0003 |
| C1-C2 for disposal | 0 | 0 | 0 |
| Skin tannery C3 | 0,0805 | 0,0845 | 0,1438 |
| Human food | 0,4932 | 0,529 | 0,8264 |
| Fat and greaves C3 | 0,1712 | 0,1649 | 0,0153 |
| Spreading/Compost | 0 | 0 | 0 |

Table 7: Total weighting by destination category for Normande Cull Cows reared in pasture

| destination | Normande/Cull Cows/pasture | | |
| --- | --- | --- | --- |
|  | **Biophysical Partition** | **Mass Partition** | **Economic Partition** |
| Pet Food | 0,0357 | 0,0623 | 0,0046 |
| PAP C3 | 0,1687 | 0,0722 | 0,0102 |
| Gelatin C3 | 0,0579 | 0,092 | 0,0003 |
| C1-C2 for disposal | 0 | 0 | 0 |
| Skin tannery C3 | 0,0824 | 0,0886 | 0,1528 |
| Human food | 0,4865 | 0,5177 | 0,8162 |
| Fat and greaves C3 | 0,1687 | 0,1672 | 0,0158 |
| Spreading/Compost | 0 | 0 | 0 |

Table 8: Total weighting by destination category for Normande Beef reared in pasture

| destination | Normande/Beef/pasture | | |
| --- | --- | --- | --- |
|  | **Biophysical Partition** | **Mass Partition** | **Economic Partition** |
| Pet Food | 0,0337 | 0,0577 | 0,0041 |
| PAP C3 | 0,1621 | 0,0674 | 0,0094 |
| Gelatin C3 | 0,0599 | 0,0939 | 0,0003 |
| C1-C2 for disposal | 0 | 0 | 0 |
| Skin tannery C3 | 0,0777 | 0,0824 | 0,1393 |
| Human food | 0,4987 | 0,5349 | 0,8317 |
| Fat and greaves C3 | 0,1679 | 0,164 | 0,015 |
| Spreading/Compost | 0 | 0 | 0 |

Table 9: Total weighting by destination category for Normande Young Bulls reared in stall

| destination | Normande/YOUNG BULL/stall | | |
| --- | --- | --- | --- |
|  | **Biophysical Partition** | **Mass Partition** | **Economic Partition** |
| Pet Food | 0,032 | 0,055 | 0,004 |
| PAP C3 | 0,1476 | 0,0636 | 0,0089 |
| Gelatin C3 | 0,0626 | 0,0951 | 0,0003 |
| C1-C2 for disposal | 0 | 0 | 0 |
| Skin tannery C3 | 0,0743 | 0,0783 | 0,1306 |
| Human food | 0,4968 | 0,546 | 0,8416 |
| Fat and greaves C3 | 0,1868 | 0,1623 | 0,0146 |
| Spreading/Compost | 0 | 0 | 0 |

Table 10: Total weighting by destination category for Normande Heifers reared in stall

| destination | Normande/HEIFER/stall | | |
| --- | --- | --- | --- |
|  | **Biophysical Partition** | **Mass Partition** | **Economic Partition** |
| Pet Food | 0,0339 | 0,0592 | 0,0045 |
| PAP C3 | 0,1537 | 0,0689 | 0,0096 |
| Gelatin C3 | 0,0611 | 0,0934 | 0,0003 |
| C1-C2 for disposal | 0 | 0 | 0 |
| Skin tannery C3 | 0,08 | 0,0845 | 0,1438 |
| Human food | 0,4849 | 0,529 | 0,8264 |
| Fat and greaves C3 | 0,1864 | 0,1649 | 0,0153 |
| Spreading/Compost | 0 | 0 | 0 |

Table 11: Total weighting by destination category for Normande Cull Cows reared in stall

| destination | Normande/Cull Cows/stall | | |
| --- | --- | --- | --- |
|  | **Biophysical Partition** | **Mass Partition** | **Economic Partition** |
| Pet Food | 0,0352 | 0,0623 | 0,0046 |
| PAP C3 | 0,1623 | 0,0722 | 0,0102 |
| Gelatin C3 | 0,0587 | 0,092 | 0,0003 |
| C1-C2 for disposal | 0 | 0 | 0 |
| Skin tannery C3 | 0,0816 | 0,0886 | 0,1528 |
| Human food | 0,4778 | 0,5177 | 0,8162 |
| Fat and greaves C3 | 0,1843 | 0,1672 | 0,0158 |
| Spreading/Compost | 0 | 0 | 0 |

Table 12: Total weighting by destination category for Normande Beef reared in stall

| destination | Normande/Beef/stall | | |
| --- | --- | --- | --- |
|  | **Biophysical Partition** | **Mass Partition** | **Economic Partition** |
| Pet Food | 0,0333 | 0,0577 | 0,0041 |
| PAP C3 | 0,1561 | 0,0674 | 0,0094 |
| Gelatin C3 | 0,0607 | 0,0939 | 0,0003 |
| C1-C2 for disposal | 0 | 0 | 0 |
| Skin tannery C3 | 0,0769 | 0,0824 | 0,1393 |
| Human food | 0,4898 | 0,5349 | 0,8317 |
| Fat and greaves C3 | 0,1837 | 0,164 | 0,015 |
| Spreading/Compost | 0 | 0 | 0 |

Table 13: Total weighting by destination category for Charolaise Young Bulls reared in grazing large area

| destination | Charolaise/YOUNG BULL/grazing large area | | |
| --- | --- | --- | --- |
|  | **Biophysical Partition** | **Mass Partition** | **Economic Partition** |
| Pet Food | 0,0308 | 0,0508 | 0,0036 |
| PAP C3 | 0,1533 | 0,059 | 0,0081 |
| Gelatin C3 | 0,063 | 0,0969 | 0,0003 |
| C1-C2 for disposal | 0 | 0 | 0 |
| Skin tannery C3 | 0,0708 | 0,072 | 0,118 |
| Human food | 0,5272 | 0,562 | 0,8558 |
| Fat and greaves C3 | 0,1548 | 0,1595 | 0,0141 |
| Spreading/Compost | 0 | 0 | 0 |

Table 14: Total weighting by destination category for Charolaise Heifers reared in grazing large area

| destination | Charolaise/HEIFER/grazing large area | | |
| --- | --- | --- | --- |
|  | **Biophysical Partition** | **Mass Partition** | **Economic Partition** |
| Pet Food | 0,0325 | 0,055 | 0,004 |
| PAP C3 | 0,1606 | 0,0636 | 0,0089 |
| Gelatin C3 | 0,061 | 0,0951 | 0,0003 |
| C1-C2 for disposal | 0 | 0 | 0 |
| Skin tannery C3 | 0,0756 | 0,0783 | 0,1306 |
| Human food | 0,514 | 0,546 | 0,8416 |
| Fat and greaves C3 | 0,1558 | 0,1623 | 0,0146 |
| Spreading/Compost | 0 | 0 | 0 |

Table 15: Total weighting by destination category for Charolaise Cull Cows reared in grazing large area

| destination | Charolaise/Cull Cows/grazing large area | | |
| --- | --- | --- | --- |
|  | **Biophysical Partition** | **Mass Partition** | **Economic Partition** |
| Pet Food | 0,0339 | 0,0577 | 0,0041 |
| PAP C3 | 0,1712 | 0,0674 | 0,0094 |
| Gelatin C3 | 0,0587 | 0,0939 | 0,0003 |
| C1-C2 for disposal | 0 | 0 | 0 |
| Skin tannery C3 | 0,0782 | 0,0824 | 0,1393 |
| Human food | 0,5092 | 0,5349 | 0,8317 |
| Fat and greaves C3 | 0,1486 | 0,164 | 0,015 |
| Spreading/Compost | 0 | 0 | 0 |

Table 16: Total weighting by destination category for Charolaise Beef reared in grazing large area

| destination | Charolaise/Beef/grazing large area | | |
| --- | --- | --- | --- |
|  | **Biophysical Partition** | **Mass Partition** | **Economic Partition** |
| Pet Food | 0,0315 | 0,0531 | 0,0037 |
| PAP C3 | 0,1639 | 0,0621 | 0,0085 |
| Gelatin C3 | 0,0607 | 0,0958 | 0,0003 |
| C1-C2 for disposal | 0 | 0 | 0 |
| Skin tannery C3 | 0,0738 | 0,0762 | 0,1264 |
| Human food | 0,5238 | 0,5516 | 0,8463 |
| Fat and greaves C3 | 0,146 | 0,1613 | 0,0145 |
| Spreading/Compost | 0 | 0 | 0 |

Table 17: Total weighting by destination category for Charolaise Young Bulls reared in pasture

| destination | Charolaise/YOUNG BULL/pasture | | |
| --- | --- | --- | --- |
|  | **Biophysical Partition** | **Mass Partition** | **Economic Partition** |
| Pet Food | 0,0305 | 0,0508 | 0,0036 |
| PAP C3 | 0,1479 | 0,059 | 0,0081 |
| Gelatin C3 | 0,0637 | 0,0969 | 0,0003 |
| C1-C2 for disposal | 0 | 0 | 0 |
| Skin tannery C3 | 0,07 | 0,072 | 0,118 |
| Human food | 0,5189 | 0,562 | 0,8558 |
| Fat and greaves C3 | 0,1693 | 0,1595 | 0,0141 |
| Spreading/Compost | 0 | 0 | 0 |

Table 18: Total weighting by destination category for Charolaise Heifers reared in pasture

| destination | Charolaise/HEIFER/pasture | | |
| --- | --- | --- | --- |
|  | **Biophysical Partition** | **Mass Partition** | **Economic Partition** |
| Pet Food | 0,0323 | 0,055 | 0,004 |
| PAP C3 | 0,1547 | 0,0636 | 0,0089 |
| Gelatin C3 | 0,0617 | 0,0951 | 0,0003 |
| C1-C2 for disposal | 0 | 0 | 0 |
| Skin tannery C3 | 0,0748 | 0,0783 | 0,1306 |
| Human food | 0,5059 | 0,546 | 0,8416 |
| Fat and greaves C3 | 0,1703 | 0,1623 | 0,0146 |
| Spreading/Compost | 0 | 0 | 0 |

Table 19: Total weighting by destination category for Charolaise Cull Cows reared in pasture

| destination | Charolaise/Cull Cows/pasture | | |
| --- | --- | --- | --- |
|  | **Biophysical Partition** | **Mass Partition** | **Economic Partition** |
| Pet Food | 0,0335 | 0,0577 | 0,0041 |
| PAP C3 | 0,1656 | 0,0674 | 0,0094 |
| Gelatin C3 | 0,0593 | 0,0939 | 0,0003 |
| C1-C2 for disposal | 0 | 0 | 0 |
| Skin tannery C3 | 0,0773 | 0,0824 | 0,1393 |
| Human food | 0,5011 | 0,5349 | 0,8317 |
| Fat and greaves C3 | 0,163 | 0,164 | 0,015 |
| Spreading/Compost | 0 | 0 | 0 |

Table 20: Total weighting by destination category for Charolaise Beef reared in pasture

| destination | Charolaise/Beef/pasture | | |
| --- | --- | --- | --- |
|  | **Biophysical Partition** | **Mass Partition** | **Economic Partition** |
| Pet Food | 0,0312 | 0,0531 | 0,0037 |
| PAP C3 | 0,1585 | 0,0621 | 0,0085 |
| Gelatin C3 | 0,0614 | 0,0958 | 0,0003 |
| C1-C2 for disposal | 0 | 0 | 0 |
| Skin tannery C3 | 0,0729 | 0,0762 | 0,1264 |
| Human food | 0,5156 | 0,5516 | 0,8463 |
| Fat and greaves C3 | 0,1602 | 0,1613 | 0,0145 |
| Spreading/Compost | 0 | 0 | 0 |

Table 21: Total weighting by destination category for Charolaise Young Bulls reared in stall

| destination | Charolaise/YOUNG BULL/stall | | |
| --- | --- | --- | --- |
|  | **Biophysical Partition** | **Mass Partition** | **Economic Partition** |
| Pet Food | 0,0303 | 0,0508 | 0,0036 |
| PAP C3 | 0,1418 | 0,059 | 0,0081 |
| Gelatin C3 | 0,0644 | 0,0969 | 0,0003 |
| C1-C2 for disposal | 0 | 0 | 0 |
| Skin tannery C3 | 0,0692 | 0,072 | 0,118 |
| Human food | 0,5095 | 0,562 | 0,8558 |
| Fat and greaves C3 | 0,1849 | 0,1595 | 0,0141 |
| Spreading/Compost | 0 | 0 | 0 |

Table 22: Total weighting by destination category for Charolaise Heifers reared in stall

| destination | Charolaise/HEIFER/stall | | |
| --- | --- | --- | --- |
|  | **Biophysical Partition** | **Mass Partition** | **Economic Partition** |
| Pet Food | 0,032 | 0,055 | 0,004 |
| PAP C3 | 0,1486 | 0,0636 | 0,0089 |
| Gelatin C3 | 0,0624 | 0,0951 | 0,0003 |
| C1-C2 for disposal | 0 | 0 | 0 |
| Skin tannery C3 | 0,0741 | 0,0783 | 0,1306 |
| Human food | 0,497 | 0,546 | 0,8416 |
| Fat and greaves C3 | 0,186 | 0,1623 | 0,0146 |
| Spreading/Compost | 0 | 0 | 0 |

Table 23: Total weighting by destination category for Charolaise Cull Cows reared in stall

| destination | Charolaise/Cull Cows/stall | | |
| --- | --- | --- | --- |
|  | **Biophysical Partition** | **Mass Partition** | **Economic Partition** |
| Pet Food | 0,0331 | 0,0577 | 0,0041 |
| PAP C3 | 0,1594 | 0,0674 | 0,0094 |
| Gelatin C3 | 0,06 | 0,0939 | 0,0003 |
| C1-C2 for disposal | 0 | 0 | 0 |
| Skin tannery C3 | 0,0765 | 0,0824 | 0,1393 |
| Human food | 0,4923 | 0,5349 | 0,8317 |
| Fat and greaves C3 | 0,1787 | 0,164 | 0,015 |
| Spreading/Compost | 0 | 0 | 0 |

Table 24: Total weighting by destination category for Charolaise Beef reared in stall

| destination | Charolaise/Beef/stall | | |
| --- | --- | --- | --- |
|  | **Biophysical Partition** | **Mass Partition** | **Economic Partition** |
| Pet Food | 0,0309 | 0,0531 | 0,0037 |
| PAP C3 | 0,1526 | 0,0621 | 0,0085 |
| Gelatin C3 | 0,062 | 0,0958 | 0,0003 |
| C1-C2 for disposal | 0 | 0 | 0 |
| Skin tannery C3 | 0,0721 | 0,0762 | 0,1264 |
| Human food | 0,5064 | 0,5516 | 0,8463 |
| Fat and greaves C3 | 0,1758 | 0,1613 | 0,0145 |
| Spreading/Compost | 0 | 0 | 0 |

Table 25: Total weighting by destination category for Primholstein Young Bulls reared in grazing large area

| destination | Primholstein/YOUNG BULL/grazing large area | | |
| --- | --- | --- | --- |
|  | **Biophysical Partition** | **Mass Partition** | **Economic Partition** |
| Pet Food | 0,0345 | 0,0592 | 0,0045 |
| PAP C3 | 0,1692 | 0,0689 | 0,0096 |
| Gelatin C3 | 0,0589 | 0,0934 | 0,0003 |
| C1-C2 for disposal | 0 | 0 | 0 |
| Skin tannery C3 | 0,0803 | 0,0845 | 0,1438 |
| Human food | 0,5015 | 0,529 | 0,8264 |
| Fat and greaves C3 | 0,1555 | 0,1649 | 0,0153 |
| Spreading/Compost | 0 | 0 | 0 |

Table 26: Total weighting by destination category for Primholstein Heifers reared in grazing large area

| destination | Primholstein/HEIFER/grazing large area | | |
| --- | --- | --- | --- |
|  | **Biophysical Partition** | **Mass Partition** | **Economic Partition** |
| Pet Food | 0,0366 | 0,0638 | 0,0048 |
| PAP C3 | 0,1742 | 0,0742 | 0,0105 |
| Gelatin C3 | 0,0575 | 0,0914 | 0,0003 |
| C1-C2 for disposal | 0 | 0 | 0 |
| Skin tannery C3 | 0,086 | 0,0908 | 0,1578 |
| Human food | 0,4896 | 0,5117 | 0,8105 |
| Fat and greaves C3 | 0,1561 | 0,168 | 0,016 |
| Spreading/Compost | 0 | 0 | 0 |

Table 27: Total weighting by destination category for Primholstein Cull Cows reared in grazing large area

| destination | Primholstein/Cull Cows/grazing large area | | |
| --- | --- | --- | --- |
|  | **Biophysical Partition** | **Mass Partition** | **Economic Partition** |
| Pet Food | 0,0369 | 0,065 | 0,005 |
| PAP C3 | 0,1791 | 0,0759 | 0,011 |
| Gelatin C3 | 0,0561 | 0,0908 | 0,0003 |
| C1-C2 for disposal | 0 | 0 | 0 |
| Skin tannery C3 | 0,0866 | 0,093 | 0,1627 |
| Human food | 0,486 | 0,5059 | 0,8048 |
| Fat and greaves C3 | 0,1551 | 0,1691 | 0,0161 |
| Spreading/Compost | 0 | 0 | 0 |

Table 28: Total weighting by destination category for Primholstein Beef reared in grazing large area

| destination | Primholstein/Beef/grazing large area | | |
| --- | --- | --- | --- |
|  | **Biophysical Partition** | **Mass Partition** | **Economic Partition** |
| Pet Food | 0,035 | 0,0607 | 0,0046 |
| PAP C3 | 0,1714 | 0,0707 | 0,01 |
| Gelatin C3 | 0,0581 | 0,0928 | 0,0003 |
| C1-C2 for disposal | 0 | 0 | 0 |
| Skin tannery C3 | 0,0817 | 0,0865 | 0,1483 |
| Human food | 0,4975 | 0,5233 | 0,8212 |
| Fat and greaves C3 | 0,1556 | 0,166 | 0,0155 |
| Spreading/Compost | 0 | 0 | 0 |

Table 29: Total weighting by destination category for Primholstein Young Bulls reared in pasture

| destination | Primholstein/YOUNG BULL/pasture | | |
| --- | --- | --- | --- |
|  | **Biophysical Partition** | **Mass Partition** | **Economic Partition** |
| Pet Food | 0,0341 | 0,0592 | 0,0045 |
| PAP C3 | 0,163 | 0,0689 | 0,0096 |
| Gelatin C3 | 0,0597 | 0,0934 | 0,0003 |
| C1-C2 for disposal | 0 | 0 | 0 |
| Skin tannery C3 | 0,0797 | 0,0845 | 0,1438 |
| Human food | 0,4934 | 0,529 | 0,8264 |
| Fat and greaves C3 | 0,1699 | 0,1649 | 0,0153 |
| Spreading/Compost | 0 | 0 | 0 |

Table 30: Total weighting by destination category for Primholstein Heifers reared in pasture

| destination | Primholstein/HEIFER/pasture | | |
| --- | --- | --- | --- |
|  | **Biophysical Partition** | **Mass Partition** | **Economic Partition** |
| Pet Food | 0,0365 | 0,0638 | 0,0048 |
| PAP C3 | 0,168 | 0,0742 | 0,0105 |
| Gelatin C3 | 0,0582 | 0,0914 | 0,0003 |
| C1-C2 for disposal | 0 | 0 | 0 |
| Skin tannery C3 | 0,0856 | 0,0908 | 0,1578 |
| Human food | 0,482 | 0,5117 | 0,8105 |
| Fat and greaves C3 | 0,1698 | 0,168 | 0,016 |
| Spreading/Compost | 0 | 0 | 0 |

Table 31: Total weighting by destination category for Primholstein Cull Cows reared in pasture

| destination | Primholstein/Cull Cows/pasture | | |
| --- | --- | --- | --- |
|  | **Biophysical Partition** | **Mass Partition** | **Economic Partition** |
| Pet Food | 0,0365 | 0,065 | 0,005 |
| PAP C3 | 0,1729 | 0,0759 | 0,011 |
| Gelatin C3 | 0,0568 | 0,0908 | 0,0003 |
| C1-C2 for disposal | 0 | 0 | 0 |
| Skin tannery C3 | 0,086 | 0,093 | 0,1627 |
| Human food | 0,478 | 0,5059 | 0,8048 |
| Fat and greaves C3 | 0,1693 | 0,1691 | 0,0161 |
| Spreading/Compost | 0 | 0 | 0 |

Table 32: Total weighting by destination category for Primholstein Beef reared in pasture

| destination | Primholstein/Beef/pasture | | |
| --- | --- | --- | --- |
|  | **Biophysical Partition** | **Mass Partition** | **Economic Partition** |
| Pet Food | 0,0348 | 0,0607 | 0,0046 |
| PAP C3 | 0,1655 | 0,0707 | 0,01 |
| Gelatin C3 | 0,0588 | 0,0928 | 0,0003 |
| C1-C2 for disposal | 0 | 0 | 0 |
| Skin tannery C3 | 0,0811 | 0,0865 | 0,1483 |
| Human food | 0,4899 | 0,5233 | 0,8212 |
| Fat and greaves C3 | 0,1698 | 0,166 | 0,0155 |
| Spreading/Compost | 0 | 0 | 0 |

Table 33: Total weighting by destination category for Primholstein Young Bulls reared in stall

| destination | Primholstein/YOUNG BULL/stall | | |
| --- | --- | --- | --- |
|  | **Biophysical Partition** | **Mass Partition** | **Economic Partition** |
| Pet Food | 0,0338 | 0,0592 | 0,0045 |
| PAP C3 | 0,1567 | 0,0689 | 0,0096 |
| Gelatin C3 | 0,0605 | 0,0934 | 0,0003 |
| C1-C2 for disposal | 0 | 0 | 0 |
| Skin tannery C3 | 0,0789 | 0,0845 | 0,1438 |
| Human food | 0,4847 | 0,529 | 0,8264 |
| Fat and greaves C3 | 0,1854 | 0,1649 | 0,0153 |
| Spreading/Compost | 0 | 0 | 0 |

Table 34: Total weighting by destination category for Primholstein Heifers reared in stall

| destination | Primholstein/HEIFER/stall | | |
| --- | --- | --- | --- |
|  | **Biophysical Partition** | **Mass Partition** | **Economic Partition** |
| Pet Food | 0,0362 | 0,0638 | 0,0048 |
| PAP C3 | 0,1611 | 0,0742 | 0,0105 |
| Gelatin C3 | 0,059 | 0,0914 | 0,0003 |
| C1-C2 for disposal | 0 | 0 | 0 |
| Skin tannery C3 | 0,0851 | 0,0908 | 0,1578 |
| Human food | 0,4739 | 0,5117 | 0,8105 |
| Fat and greaves C3 | 0,1847 | 0,168 | 0,016 |
| Spreading/Compost | 0 | 0 | 0 |

Table 35: Total weighting by destination category for Primholstein Cull Cows reared in stall

| destination | Primholstein/Cull Cows/stall | | |
| --- | --- | --- | --- |
|  | **Biophysical Partition** | **Mass Partition** | **Economic Partition** |
| Pet Food | 0,0364 | 0,065 | 0,005 |
| PAP C3 | 0,1662 | 0,0759 | 0,011 |
| Gelatin C3 | 0,0576 | 0,0908 | 0,0003 |
| C1-C2 for disposal | 0 | 0 | 0 |
| Skin tannery C3 | 0,0855 | 0,093 | 0,1627 |
| Human food | 0,4696 | 0,5059 | 0,8048 |
| Fat and greaves C3 | 0,1846 | 0,1691 | 0,0161 |
| Spreading/Compost | 0 | 0 | 0 |

Table 36: Total weighting by destination category for Primholstein Beef reared in stall

| destination | Primholstein/Beef/stall | | |
| --- | --- | --- | --- |
|  | **Biophysical Partition** | **Mass Partition** | **Economic Partition** |
| Pet Food | 0,0345 | 0,0607 | 0,0046 |
| PAP C3 | 0,159 | 0,0707 | 0,01 |
| Gelatin C3 | 0,0596 | 0,0928 | 0,0003 |
| C1-C2 for disposal | 0 | 0 | 0 |
| Skin tannery C3 | 0,0805 | 0,0865 | 0,1483 |
| Human food | 0,4813 | 0,5233 | 0,8212 |
| Fat and greaves C3 | 0,1853 | 0,166 | 0,0155 |
| Spreading/Compost | 0 | 0 | 0 |

Table 37: Total weighting by destination category for Limousine Young Bulls reared in grazing large area

| destination | Limousine/YOUNG BULL/grazing large area | | |
| --- | --- | --- | --- |
|  | **Biophysical Partition** | **Mass Partition** | **Economic Partition** |
| Pet Food | 0,0289 | 0,0466 | 0,0031 |
| PAP C3 | 0,1406 | 0,054 | 0,0073 |
| Gelatin C3 | 0,0662 | 0,0987 | 0,0002 |
| C1-C2 for disposal | 0 | 0 | 0 |
| Skin tannery C3 | 0,0668 | 0,0662 | 0,1066 |
| Human food | 0,5375 | 0,578 | 0,8688 |
| Fat and greaves C3 | 0,16 | 0,1565 | 0,0137 |
| Spreading/Compost | 0 | 0 | 0 |

Table 38: Total weighting by destination category for Limousine Heifers reared in grazing large area

| destination | Limousine/HEIFER/grazing large area | | |
| --- | --- | --- | --- |
|  | **Biophysical Partition** | **Mass Partition** | **Economic Partition** |
| Pet Food | 0,031 | 0,052 | 0,0036 |
| PAP C3 | 0,1511 | 0,0604 | 0,0083 |
| Gelatin C3 | 0,0633 | 0,0963 | 0,0003 |
| C1-C2 for disposal | 0 | 0 | 0 |
| Skin tannery C3 | 0,0733 | 0,0742 | 0,1223 |
| Human food | 0,5212 | 0,5569 | 0,851 |
| Fat and greaves C3 | 0,1595 | 0,1603 | 0,0143 |
| Spreading/Compost | 0 | 0 | 0 |

Table 39: Total weighting by destination category for Limousine Cull Cows reared in grazing large area

| destination | Limousine/Cull Cows/grazing large area | | |
| --- | --- | --- | --- |
|  | **Biophysical Partition** | **Mass Partition** | **Economic Partition** |
| Pet Food | 0,0326 | 0,055 | 0,004 |
| PAP C3 | 0,1614 | 0,0636 | 0,0089 |
| Gelatin C3 | 0,0608 | 0,0951 | 0,0003 |
| C1-C2 for disposal | 0 | 0 | 0 |
| Skin tannery C3 | 0,0755 | 0,0783 | 0,1306 |
| Human food | 0,5145 | 0,546 | 0,8416 |
| Fat and greaves C3 | 0,1549 | 0,1623 | 0,0146 |
| Spreading/Compost | 0 | 0 | 0 |

Table 40: Total weighting by destination category for Limousine Beef reared in grazing large area

| destination | Limousine/Beef/grazing large area | | |
| --- | --- | --- | --- |
|  | **Biophysical Partition** | **Mass Partition** | **Economic Partition** |
| Pet Food | 0,0308 | 0,0508 | 0,0036 |
| PAP C3 | 0,1529 | 0,059 | 0,0081 |
| Gelatin C3 | 0,0631 | 0,0969 | 0,0003 |
| C1-C2 for disposal | 0 | 0 | 0 |
| Skin tannery C3 | 0,0708 | 0,072 | 0,118 |
| Human food | 0,5269 | 0,562 | 0,8558 |
| Fat and greaves C3 | 0,1552 | 0,1595 | 0,0141 |
| Spreading/Compost | 0 | 0 | 0 |

Table 41: Total weighting by destination category for Limousine Young Bulls reared in pasture

| destination | Limousine/YOUNG BULL/pasture | | |
| --- | --- | --- | --- |
|  | **Biophysical Partition** | **Mass Partition** | **Economic Partition** |
| Pet Food | 0,0285 | 0,0466 | 0,0031 |
| PAP C3 | 0,1353 | 0,054 | 0,0073 |
| Gelatin C3 | 0,0669 | 0,0987 | 0,0002 |
| C1-C2 for disposal | 0 | 0 | 0 |
| Skin tannery C3 | 0,0661 | 0,0662 | 0,1066 |
| Human food | 0,5287 | 0,578 | 0,8688 |
| Fat and greaves C3 | 0,1742 | 0,1565 | 0,0137 |
| Spreading/Compost | 0 | 0 | 0 |

Table 42: Total weighting by destination category for Limousine Heifers reared in pasture

| destination | Limousine/HEIFER/pasture | | |
| --- | --- | --- | --- |
|  | **Biophysical Partition** | **Mass Partition** | **Economic Partition** |
| Pet Food | 0,0309 | 0,052 | 0,0036 |
| PAP C3 | 0,1455 | 0,0604 | 0,0083 |
| Gelatin C3 | 0,064 | 0,0963 | 0,0003 |
| C1-C2 for disposal | 0 | 0 | 0 |
| Skin tannery C3 | 0,0728 | 0,0742 | 0,1223 |
| Human food | 0,5133 | 0,5569 | 0,851 |
| Fat and greaves C3 | 0,1734 | 0,1603 | 0,0143 |
| Spreading/Compost | 0 | 0 | 0 |

Table 43: Total weighting by destination category for Limousine Cull Cows reared in pasture

| destination | Limousine/Cull Cows/pasture | | |
| --- | --- | --- | --- |
|  | **Biophysical Partition** | **Mass Partition** | **Economic Partition** |
| Pet Food | 0,0323 | 0,055 | 0,004 |
| PAP C3 | 0,1556 | 0,0636 | 0,0089 |
| Gelatin C3 | 0,0616 | 0,0951 | 0,0003 |
| C1-C2 for disposal | 0 | 0 | 0 |
| Skin tannery C3 | 0,0747 | 0,0783 | 0,1306 |
| Human food | 0,5062 | 0,546 | 0,8416 |
| Fat and greaves C3 | 0,1693 | 0,1623 | 0,0146 |
| Spreading/Compost | 0 | 0 | 0 |

Table 44: Total weighting by destination category for Limousine Beef reared in pasture

| destination | Limousine/Beef/pasture | | |
| --- | --- | --- | --- |
|  | **Biophysical Partition** | **Mass Partition** | **Economic Partition** |
| Pet Food | 0,0305 | 0,0508 | 0,0036 |
| PAP C3 | 0,1475 | 0,059 | 0,0081 |
| Gelatin C3 | 0,0638 | 0,0969 | 0,0003 |
| C1-C2 for disposal | 0 | 0 | 0 |
| Skin tannery C3 | 0,07 | 0,072 | 0,118 |
| Human food | 0,5185 | 0,562 | 0,8558 |
| Fat and greaves C3 | 0,1698 | 0,1595 | 0,0141 |
| Spreading/Compost | 0 | 0 | 0 |

Table 45: Total weighting by destination category for Limousine Young Bulls reared in stall

| destination | Limousine/YOUNG BULL/stall | | |
| --- | --- | --- | --- |
|  | **Biophysical Partition** | **Mass Partition** | **Economic Partition** |
| Pet Food | 0,0282 | 0,0466 | 0,0031 |
| PAP C3 | 0,1293 | 0,054 | 0,0073 |
| Gelatin C3 | 0,0677 | 0,0987 | 0,0002 |
| C1-C2 for disposal | 0 | 0 | 0 |
| Skin tannery C3 | 0,0653 | 0,0662 | 0,1066 |
| Human food | 0,5193 | 0,578 | 0,8688 |
| Fat and greaves C3 | 0,19 | 0,1565 | 0,0137 |
| Spreading/Compost | 0 | 0 | 0 |

Table 46: Total weighting by destination category for Limousine Heifers reared in stall

| destination | Limousine/HEIFER/stall | | |
| --- | --- | --- | --- |
|  | **Biophysical Partition** | **Mass Partition** | **Economic Partition** |
| Pet Food | 0,0306 | 0,052 | 0,0036 |
| PAP C3 | 0,1393 | 0,0604 | 0,0083 |
| Gelatin C3 | 0,0648 | 0,0963 | 0,0003 |
| C1-C2 for disposal | 0 | 0 | 0 |
| Skin tannery C3 | 0,072 | 0,0742 | 0,1223 |
| Human food | 0,5046 | 0,5569 | 0,851 |
| Fat and greaves C3 | 0,1888 | 0,1603 | 0,0143 |
| Spreading/Compost | 0 | 0 | 0 |

Table 47: Total weighting by destination category for Limousine Cull Cows reared in stall

| destination | Limousine/Cull Cows/stall | | |
| --- | --- | --- | --- |
|  | **Biophysical Partition** | **Mass Partition** | **Economic Partition** |
| Pet Food | 0,032 | 0,055 | 0,004 |
| PAP C3 | 0,1493 | 0,0636 | 0,0089 |
| Gelatin C3 | 0,0622 | 0,0951 | 0,0003 |
| C1-C2 for disposal | 0 | 0 | 0 |
| Skin tannery C3 | 0,074 | 0,0783 | 0,1306 |
| Human food | 0,4973 | 0,546 | 0,8416 |
| Fat and greaves C3 | 0,185 | 0,1623 | 0,0146 |
| Spreading/Compost | 0 | 0 | 0 |

Table 48: Total weighting by destination category for Limousine Beef reared in stall

| destination | Limousine/Beef/stall | | |
| --- | --- | --- | --- |
|  | **Biophysical Partition** | **Mass Partition** | **Economic Partition** |
| Pet Food | 0,0302 | 0,0508 | 0,0036 |
| PAP C3 | 0,1414 | 0,059 | 0,0081 |
| Gelatin C3 | 0,0645 | 0,0969 | 0,0003 |
| C1-C2 for disposal | 0 | 0 | 0 |
| Skin tannery C3 | 0,0692 | 0,072 | 0,118 |
| Human food | 0,5092 | 0,562 | 0,8558 |
| Fat and greaves C3 | 0,1855 | 0,1595 | 0,0141 |
| Spreading/Compost | 0 | 0 | 0 |

Table 49: Total weighting by destination category for Blonde d’Aquitaine Young Bulls reared in grazing large area

| destination | Blonde d’Aquitaine/YOUNG BULL/grazing large area | | |
| --- | --- | --- | --- |
|  | **Biophysical Partition** | **Mass Partition** | **Economic Partition** |
| Pet Food | 0,0275 | 0,0436 | 0,0029 |
| PAP C3 | 0,136 | 0,0509 | 0,0067 |
| Gelatin C3 | 0,0675 | 0,0997 | 0,0002 |
| C1-C2 for disposal | 0 | 0 | 0 |
| Skin tannery C3 | 0,0634 | 0,0624 | 0,0992 |
| Human food | 0,5464 | 0,5885 | 0,8775 |
| Fat and greaves C3 | 0,1593 | 0,1548 | 0,0134 |
| Spreading/Compost | 0 | 0 | 0 |

Table 50: Total weighting by destination category for Blonde d’Aquitaine Heifers reared in grazing large area

| destination | Blonde d’Aquitaine/HEIFER/grazing large area | | |
| --- | --- | --- | --- |
|  | **Biophysical Partition** | **Mass Partition** | **Economic Partition** |
| Pet Food | 0,0298 | 0,0492 | 0,0035 |
| PAP C3 | 0,1506 | 0,0572 | 0,0078 |
| Gelatin C3 | 0,0639 | 0,0974 | 0,0002 |
| C1-C2 for disposal | 0 | 0 | 0 |
| Skin tannery C3 | 0,0694 | 0,07 | 0,1141 |
| Human food | 0,5306 | 0,5678 | 0,8602 |
| Fat and greaves C3 | 0,1554 | 0,1585 | 0,014 |
| Spreading/Compost | 0 | 0 | 0 |

Table 51: Total weighting by destination category for Blonde d’Aquitaine Cull Cows reared in grazing large area

| destination | Blonde d’Aquitaine/Cull Cows/grazing large area | | |
| --- | --- | --- | --- |
|  | **Biophysical Partition** | **Mass Partition** | **Economic Partition** |
| Pet Food | 0,0348 | 0,0592 | 0,0045 |
| PAP C3 | 0,1849 | 0,0689 | 0,0096 |
| Gelatin C3 | 0,0566 | 0,0934 | 0,0003 |
| C1-C2 for disposal | 0 | 0 | 0 |
| Skin tannery C3 | 0,08 | 0,0845 | 0,1438 |
| Human food | 0,5168 | 0,529 | 0,8264 |
| Fat and greaves C3 | 0,1267 | 0,1649 | 0,0153 |
| Spreading/Compost | 0 | 0 | 0 |

Table 52: Total weighting by destination category for Blonde d’Aquitaine Beef reared in grazing large area

| destination | Blonde d’Aquitaine/Beef/grazing large area | | |
| --- | --- | --- | --- |
|  | **Biophysical Partition** | **Mass Partition** | **Economic Partition** |
| Pet Food | 0,0294 | 0,0478 | 0,0034 |
| PAP C3 | 0,1511 | 0,0556 | 0,0075 |
| Gelatin C3 | 0,064 | 0,098 | 0,0002 |
| C1-C2 for disposal | 0 | 0 | 0 |
| Skin tannery C3 | 0,0679 | 0,0682 | 0,1104 |
| Human food | 0,5399 | 0,573 | 0,8646 |
| Fat and greaves C3 | 0,1474 | 0,1576 | 0,0138 |
| Spreading/Compost | 0 | 0 | 0 |

Table 53: Total weighting by destination category for Blonde d’Aquitaine Young Bulls reared in pasture

| destination | Blonde d’Aquitaine/YOUNG BULL/pasture | | |
| --- | --- | --- | --- |
|  | **Biophysical Partition** | **Mass Partition** | **Economic Partition** |
| Pet Food | 0,0272 | 0,0436 | 0,0029 |
| PAP C3 | 0,1309 | 0,0509 | 0,0067 |
| Gelatin C3 | 0,0681 | 0,0997 | 0,0002 |
| C1-C2 for disposal | 0 | 0 | 0 |
| Skin tannery C3 | 0,0627 | 0,0624 | 0,0992 |
| Human food | 0,5376 | 0,5885 | 0,8775 |
| Fat and greaves C3 | 0,1737 | 0,1548 | 0,0134 |
| Spreading/Compost | 0 | 0 | 0 |

Table 54: Total weighting by destination category for Blonde d’Aquitaine Heifers reared in pasture

| destination | Blonde d’Aquitaine/HEIFER/pasture | | |
| --- | --- | --- | --- |
|  | **Biophysical Partition** | **Mass Partition** | **Economic Partition** |
| Pet Food | 0,0296 | 0,0492 | 0,0035 |
| PAP C3 | 0,1451 | 0,0572 | 0,0078 |
| Gelatin C3 | 0,0645 | 0,0974 | 0,0002 |
| C1-C2 for disposal | 0 | 0 | 0 |
| Skin tannery C3 | 0,0686 | 0,07 | 0,1141 |
| Human food | 0,5222 | 0,5678 | 0,8602 |
| Fat and greaves C3 | 0,1699 | 0,1585 | 0,014 |
| Spreading/Compost | 0 | 0 | 0 |

Table 55: Total weighting by destination category for Blonde d’Aquitaine Cull Cows reared in pasture

| destination | Blonde d’Aquitaine/Cull Cows/pasture | | |
| --- | --- | --- | --- |
|  | **Biophysical Partition** | **Mass Partition** | **Economic Partition** |
| Pet Food | 0,0345 | 0,0592 | 0,0045 |
| PAP C3 | 0,1798 | 0,0689 | 0,0096 |
| Gelatin C3 | 0,0572 | 0,0934 | 0,0003 |
| C1-C2 for disposal | 0 | 0 | 0 |
| Skin tannery C3 | 0,0792 | 0,0845 | 0,1438 |
| Human food | 0,5095 | 0,529 | 0,8264 |
| Fat and greaves C3 | 0,1396 | 0,1649 | 0,0153 |
| Spreading/Compost | 0 | 0 | 0 |

Table 56: Total weighting by destination category for Blonde d’Aquitaine Beef reared in pasture

| destination | Blonde d’Aquitaine/Beef/pasture | | |
| --- | --- | --- | --- |
|  | **Biophysical Partition** | **Mass Partition** | **Economic Partition** |
| Pet Food | 0,0291 | 0,0478 | 0,0034 |
| PAP C3 | 0,1461 | 0,0556 | 0,0075 |
| Gelatin C3 | 0,0645 | 0,098 | 0,0002 |
| C1-C2 for disposal | 0 | 0 | 0 |
| Skin tannery C3 | 0,0671 | 0,0682 | 0,1104 |
| Human food | 0,5314 | 0,573 | 0,8646 |
| Fat and greaves C3 | 0,1616 | 0,1576 | 0,0138 |
| Spreading/Compost | 0 | 0 | 0 |

Table 57: Total weighting by destination category for Blonde d’Aquitaine Young Bulls reared in stall

| destination | Blonde d’Aquitaine/YOUNG BULL/stall | | |
| --- | --- | --- | --- |
|  | **Biophysical Partition** | **Mass Partition** | **Economic Partition** |
| Pet Food | 0,0266 | 0,0436 | 0,0029 |
| PAP C3 | 0,1252 | 0,0509 | 0,0067 |
| Gelatin C3 | 0,0689 | 0,0997 | 0,0002 |
| C1-C2 for disposal | 0 | 0 | 0 |
| Skin tannery C3 | 0,0619 | 0,0624 | 0,0992 |
| Human food | 0,528 | 0,5885 | 0,8775 |
| Fat and greaves C3 | 0,1893 | 0,1548 | 0,0134 |
| Spreading/Compost | 0 | 0 | 0 |

Table 58: Total weighting by destination category for Blonde d’Aquitaine Heifers reared in stall

| destination | Blonde d’Aquitaine/HEIFER/stall | | |
| --- | --- | --- | --- |
|  | **Biophysical Partition** | **Mass Partition** | **Economic Partition** |
| Pet Food | 0,0292 | 0,0492 | 0,0035 |
| PAP C3 | 0,1392 | 0,0572 | 0,0078 |
| Gelatin C3 | 0,0652 | 0,0974 | 0,0002 |
| C1-C2 for disposal | 0 | 0 | 0 |
| Skin tannery C3 | 0,0677 | 0,07 | 0,1141 |
| Human food | 0,5128 | 0,5678 | 0,8602 |
| Fat and greaves C3 | 0,1857 | 0,1585 | 0,014 |
| Spreading/Compost | 0 | 0 | 0 |

Table 59: Total weighting by destination category for Blonde d’Aquitaine Cull Cows reared in stall

| destination | Blonde d’Aquitaine/Cull Cows/stall | | |
| --- | --- | --- | --- |
|  | **Biophysical Partition** | **Mass Partition** | **Economic Partition** |
| Pet Food | 0,0342 | 0,0592 | 0,0045 |
| PAP C3 | 0,1744 | 0,0689 | 0,0096 |
| Gelatin C3 | 0,0577 | 0,0934 | 0,0003 |
| C1-C2 for disposal | 0 | 0 | 0 |
| Skin tannery C3 | 0,0783 | 0,0845 | 0,1438 |
| Human food | 0,5012 | 0,529 | 0,8264 |
| Fat and greaves C3 | 0,1543 | 0,1649 | 0,0153 |
| Spreading/Compost | 0 | 0 | 0 |

Table 60: Total weighting by destination category for Blonde d’Aquitaine Beef reared in stall

| destination | Blonde d’Aquitaine/Beef/stall | | |
| --- | --- | --- | --- |
|  | **Biophysical Partition** | **Mass Partition** | **Economic Partition** |
| Pet Food | 0,0288 | 0,0478 | 0,0034 |
| PAP C3 | 0,1404 | 0,0556 | 0,0075 |
| Gelatin C3 | 0,0652 | 0,098 | 0,0002 |
| C1-C2 for disposal | 0 | 0 | 0 |
| Skin tannery C3 | 0,0661 | 0,0682 | 0,1104 |
| Human food | 0,522 | 0,573 | 0,8646 |
| Fat and greaves C3 | 0,1773 | 0,1576 | 0,0138 |
| Spreading/Compost | 0 | 0 | 0 |

Table 61: Total weighting by destination category for Salers Young Bulls reared in grazing large area

| destination | Salers/YOUNG BULL/grazing large area | | |
| --- | --- | --- | --- |
|  | **Biophysical Partition** | **Mass Partition** | **Economic Partition** |
| Pet Food | 0,0326 | 0,055 | 0,004 |
| PAP C3 | 0,1616 | 0,0636 | 0,0089 |
| Gelatin C3 | 0,0608 | 0,0951 | 0,0003 |
| C1-C2 for disposal | 0 | 0 | 0 |
| Skin tannery C3 | 0,0755 | 0,0783 | 0,1306 |
| Human food | 0,5147 | 0,546 | 0,8416 |
| Fat and greaves C3 | 0,1547 | 0,1623 | 0,0146 |
| Spreading/Compost | 0 | 0 | 0 |

Table 62: Total weighting by destination category for Salers Heifers reared in grazing large area

| destination | Salers/HEIFER/grazing large area | | |
| --- | --- | --- | --- |
|  | **Biophysical Partition** | **Mass Partition** | **Economic Partition** |
| Pet Food | 0,0346 | 0,0592 | 0,0045 |
| PAP C3 | 0,1662 | 0,0689 | 0,0096 |
| Gelatin C3 | 0,0596 | 0,0934 | 0,0003 |
| C1-C2 for disposal | 0 | 0 | 0 |
| Skin tannery C3 | 0,0812 | 0,0845 | 0,1438 |
| Human food | 0,5011 | 0,529 | 0,8264 |
| Fat and greaves C3 | 0,1572 | 0,1649 | 0,0153 |
| Spreading/Compost | 0 | 0 | 0 |

Table 63: Total weighting by destination category for Salers Cull Cows reared in grazing large area

| destination | Salers/Cull Cows/grazing large area | | |
| --- | --- | --- | --- |
|  | **Biophysical Partition** | **Mass Partition** | **Economic Partition** |
| Pet Food | 0,0361 | 0,0623 | 0,0046 |
| PAP C3 | 0,1746 | 0,0722 | 0,0102 |
| Gelatin C3 | 0,0572 | 0,092 | 0,0003 |
| C1-C2 for disposal | 0 | 0 | 0 |
| Skin tannery C3 | 0,083 | 0,0886 | 0,1528 |
| Human food | 0,4942 | 0,5177 | 0,8162 |
| Fat and greaves C3 | 0,155 | 0,1672 | 0,0158 |
| Spreading/Compost | 0 | 0 | 0 |

Table 64: Total weighting by destination category for Salers Beef reared in grazing large area

| destination | Salers/Beef/grazing large area | | |
| --- | --- | --- | --- |
|  | **Biophysical Partition** | **Mass Partition** | **Economic Partition** |
| Pet Food | 0,0339 | 0,0577 | 0,0041 |
| PAP C3 | 0,1699 | 0,0674 | 0,0094 |
| Gelatin C3 | 0,0589 | 0,0939 | 0,0003 |
| C1-C2 for disposal | 0 | 0 | 0 |
| Skin tannery C3 | 0,0782 | 0,0824 | 0,1393 |
| Human food | 0,5081 | 0,5349 | 0,8317 |
| Fat and greaves C3 | 0,1507 | 0,164 | 0,015 |
| Spreading/Compost | 0 | 0 | 0 |

Table 65: Total weighting by destination category for Salers Young Bulls reared in pasture

| destination | Salers/YOUNG BULL/pasture | | |
| --- | --- | --- | --- |
|  | **Biophysical Partition** | **Mass Partition** | **Economic Partition** |
| Pet Food | 0,0323 | 0,055 | 0,004 |
| PAP C3 | 0,1557 | 0,0636 | 0,0089 |
| Gelatin C3 | 0,0615 | 0,0951 | 0,0003 |
| C1-C2 for disposal | 0 | 0 | 0 |
| Skin tannery C3 | 0,0747 | 0,0783 | 0,1306 |
| Human food | 0,5065 | 0,546 | 0,8416 |
| Fat and greaves C3 | 0,1691 | 0,1623 | 0,0146 |
| Spreading/Compost | 0 | 0 | 0 |

Table 66: Total weighting by destination category for Salers Heifers reared in pasture

| destination | Salers/HEIFER/pasture | | |
| --- | --- | --- | --- |
|  | **Biophysical Partition** | **Mass Partition** | **Economic Partition** |
| Pet Food | 0,0343 | 0,0592 | 0,0045 |
| PAP C3 | 0,1601 | 0,0689 | 0,0096 |
| Gelatin C3 | 0,0604 | 0,0934 | 0,0003 |
| C1-C2 for disposal | 0 | 0 | 0 |
| Skin tannery C3 | 0,0807 | 0,0845 | 0,1438 |
| Human food | 0,4932 | 0,529 | 0,8264 |
| Fat and greaves C3 | 0,1713 | 0,1649 | 0,0153 |
| Spreading/Compost | 0 | 0 | 0 |

Table 67: Total weighting by destination category for Salers Cull Cows reared in pasture

| destination | Salers/Cull Cows/pasture | | |
| --- | --- | --- | --- |
|  | **Biophysical Partition** | **Mass Partition** | **Economic Partition** |
| Pet Food | 0,0357 | 0,0623 | 0,0046 |
| PAP C3 | 0,1684 | 0,0722 | 0,0102 |
| Gelatin C3 | 0,0579 | 0,092 | 0,0003 |
| C1-C2 for disposal | 0 | 0 | 0 |
| Skin tannery C3 | 0,0824 | 0,0886 | 0,1528 |
| Human food | 0,4863 | 0,5177 | 0,8162 |
| Fat and greaves C3 | 0,1691 | 0,1672 | 0,0158 |
| Spreading/Compost | 0 | 0 | 0 |

Table 68: Total weighting by destination category for Salers Beef reared in pasture

| destination | Salers/Beef/pasture | | |
| --- | --- | --- | --- |
|  | **Biophysical Partition** | **Mass Partition** | **Economic Partition** |
| Pet Food | 0,0336 | 0,0577 | 0,0041 |
| PAP C3 | 0,1641 | 0,0674 | 0,0094 |
| Gelatin C3 | 0,0595 | 0,0939 | 0,0003 |
| C1-C2 for disposal | 0 | 0 | 0 |
| Skin tannery C3 | 0,0774 | 0,0824 | 0,1393 |
| Human food | 0,5001 | 0,5349 | 0,8317 |
| Fat and greaves C3 | 0,1651 | 0,164 | 0,015 |
| Spreading/Compost | 0 | 0 | 0 |

Table 69: Total weighting by destination category for Salers Young Bulls reared in stall

| destination | Salers/YOUNG BULL/stall | | |
| --- | --- | --- | --- |
|  | **Biophysical Partition** | **Mass Partition** | **Economic Partition** |
| Pet Food | 0,032 | 0,055 | 0,004 |
| PAP C3 | 0,1495 | 0,0636 | 0,0089 |
| Gelatin C3 | 0,0622 | 0,0951 | 0,0003 |
| C1-C2 for disposal | 0 | 0 | 0 |
| Skin tannery C3 | 0,0739 | 0,0783 | 0,1306 |
| Human food | 0,4973 | 0,546 | 0,8416 |
| Fat and greaves C3 | 0,1848 | 0,1623 | 0,0146 |
| Spreading/Compost | 0 | 0 | 0 |

Table 70: Total weighting by destination category for Salers Heifers reared in stall

| destination | Salers/HEIFER/stall | | |
| --- | --- | --- | --- |
|  | **Biophysical Partition** | **Mass Partition** | **Economic Partition** |
| Pet Food | 0,0339 | 0,0592 | 0,0045 |
| PAP C3 | 0,1536 | 0,0689 | 0,0096 |
| Gelatin C3 | 0,0611 | 0,0934 | 0,0003 |
| C1-C2 for disposal | 0 | 0 | 0 |
| Skin tannery C3 | 0,08 | 0,0845 | 0,1438 |
| Human food | 0,485 | 0,529 | 0,8264 |
| Fat and greaves C3 | 0,1864 | 0,1649 | 0,0153 |
| Spreading/Compost | 0 | 0 | 0 |

Table 71: Total weighting by destination category for Salers Cull Cows reared in stall

| destination | Salers/Cull Cows/stall | | |
| --- | --- | --- | --- |
|  | **Biophysical Partition** | **Mass Partition** | **Economic Partition** |
| Pet Food | 0,0353 | 0,0623 | 0,0046 |
| PAP C3 | 0,162 | 0,0722 | 0,0102 |
| Gelatin C3 | 0,0587 | 0,092 | 0,0003 |
| C1-C2 for disposal | 0 | 0 | 0 |
| Skin tannery C3 | 0,0817 | 0,0886 | 0,1528 |
| Human food | 0,4776 | 0,5177 | 0,8162 |
| Fat and greaves C3 | 0,1845 | 0,1672 | 0,0158 |
| Spreading/Compost | 0 | 0 | 0 |

Table 72: Total weighting by destination category for Salers Beef reared in stall

| destination | Salers/Beef/stall | | |
| --- | --- | --- | --- |
|  | **Biophysical Partition** | **Mass Partition** | **Economic Partition** |
| Pet Food | 0,0332 | 0,0577 | 0,0041 |
| PAP C3 | 0,1579 | 0,0674 | 0,0094 |
| Gelatin C3 | 0,0603 | 0,0939 | 0,0003 |
| C1-C2 for disposal | 0 | 0 | 0 |
| Skin tannery C3 | 0,0766 | 0,0824 | 0,1393 |
| Human food | 0,491 | 0,5349 | 0,8317 |
| Fat and greaves C3 | 0,1809 | 0,164 | 0,015 |
| Spreading/Compost | 0 | 0 | 0 |

Table 73: Total weighting by destination category for Rouge des Prés Young Bulls reared in grazing large area

| destination | Limousine/YOUNG BULL/grazing large area | | |
| --- | --- | --- | --- |
|  | **Biophysical Partition** | **Mass Partition** | **Economic Partition** |
| Pet Food | 0,0311 | 0,052 | 0,0036 |
| PAP C3 | 0,1575 | 0,0604 | 0,0083 |
| Gelatin C3 | 0,062 | 0,0963 | 0,0003 |
| C1-C2 for disposal | 0 | 0 | 0 |
| Skin tannery C3 | 0,0724 | 0,0742 | 0,1223 |
| Human food | 0,5245 | 0,5569 | 0,851 |
| Fat and greaves C3 | 0,1522 | 0,1603 | 0,0143 |
| Spreading/Compost | 0 | 0 | 0 |

Table 74: Total weighting by destination category for Rouge des Prés Heifers reared in grazing large area

| destination | Rouge des Prés/HEIFER/grazing large area | | |
| --- | --- | --- | --- |
|  | **Biophysical Partition** | **Mass Partition** | **Economic Partition** |
| Pet Food | 0,033 | 0,0561 | 0,0041 |
| PAP C3 | 0,1662 | 0,0654 | 0,0092 |
| Gelatin C3 | 0,0598 | 0,0945 | 0,0003 |
| C1-C2 for disposal | 0 | 0 | 0 |
| Skin tannery C3 | 0,0768 | 0,0803 | 0,1349 |
| Human food | 0,5121 | 0,5404 | 0,8367 |
| Fat and greaves C3 | 0,1518 | 0,1632 | 0,0148 |
| Spreading/Compost | 0 | 0 | 0 |

Table 75: Total weighting by destination category for Rouge des Prés Cull Cows reared in grazing large area

| destination | Rouge des Prés/Cull Cows/grazing large area | | |
| --- | --- | --- | --- |
|  | **Biophysical Partition** | **Mass Partition** | **Economic Partition** |
| Pet Food | 0,0345 | 0,0592 | 0,0045 |
| PAP C3 | 0,1773 | 0,0689 | 0,0096 |
| Gelatin C3 | 0,0576 | 0,0934 | 0,0003 |
| C1-C2 for disposal | 0 | 0 | 0 |
| Skin tannery C3 | 0,0796 | 0,0845 | 0,1438 |
| Human food | 0,5077 | 0,529 | 0,8264 |
| Fat and greaves C3 | 0,1433 | 0,1649 | 0,0153 |
| Spreading/Compost | 0 | 0 | 0 |

Table 76: Total weighting by destination category for Rouge des Prés Beef reared in grazing large area

| destination | Rouge des Prés/Beef/grazing large area | | |
| --- | --- | --- | --- |
|  | **Biophysical Partition** | **Mass Partition** | **Economic Partition** |
| Pet Food | 0,0331 | 0,055 | 0,004 |
| PAP C3 | 0,1776 | 0,0636 | 0,0089 |
| Gelatin C3 | 0,0585 | 0,0951 | 0,0003 |
| C1-C2 for disposal | 0 | 0 | 0 |
| Skin tannery C3 | 0,076 | 0,0783 | 0,1306 |
| Human food | 0,533 | 0,546 | 0,8416 |
| Fat and greaves C3 | 0,1215 | 0,1623 | 0,0146 |
| Spreading/Compost | 0 | 0 | 0 |

Table 77: Total weighting by destination category for Rouge des Prés Young Bulls reared in pasture

| destination | Rouge des Prés/YOUNG BULL/pasture | | |
| --- | --- | --- | --- |
|  | **Biophysical Partition** | **Mass Partition** | **Economic Partition** |
| Pet Food | 0,0309 | 0,052 | 0,0036 |
| PAP C3 | 0,152 | 0,0604 | 0,0083 |
| Gelatin C3 | 0,0627 | 0,0963 | 0,0003 |
| C1-C2 for disposal | 0 | 0 | 0 |
| Skin tannery C3 | 0,0716 | 0,0742 | 0,1223 |
| Human food | 0,5158 | 0,5569 | 0,851 |
| Fat and greaves C3 | 0,1667 | 0,1603 | 0,0143 |
| Spreading/Compost | 0 | 0 | 0 |

Table 78: Total weighting by destination category for Rouge des Prés Heifers reared in pasture

| destination | Rouge des Prés/HEIFER/pasture | | |
| --- | --- | --- | --- |
|  | **Biophysical Partition** | **Mass Partition** | **Economic Partition** |
| Pet Food | 0,0328 | 0,0561 | 0,0041 |
| PAP C3 | 0,1606 | 0,0654 | 0,0092 |
| Gelatin C3 | 0,0604 | 0,0945 | 0,0003 |
| C1-C2 for disposal | 0 | 0 | 0 |
| Skin tannery C3 | 0,076 | 0,0803 | 0,1349 |
| Human food | 0,5039 | 0,5404 | 0,8367 |
| Fat and greaves C3 | 0,1662 | 0,1632 | 0,0148 |
| Spreading/Compost | 0 | 0 | 0 |

Table 79: Total weighting by destination category for Rouge des Prés Cull Cows reared in pasture

| destination | Rouge des Prés/Cull Cows/pasture | | |
| --- | --- | --- | --- |
|  | **Biophysical Partition** | **Mass Partition** | **Economic Partition** |
| Pet Food | 0,0343 | 0,0592 | 0,0045 |
| PAP C3 | 0,1716 | 0,0689 | 0,0096 |
| Gelatin C3 | 0,0582 | 0,0934 | 0,0003 |
| C1-C2 for disposal | 0 | 0 | 0 |
| Skin tannery C3 | 0,0787 | 0,0845 | 0,1438 |
| Human food | 0,4999 | 0,529 | 0,8264 |
| Fat and greaves C3 | 0,1574 | 0,1649 | 0,0153 |
| Spreading/Compost | 0 | 0 | 0 |

Table 80: Total weighting by destination category for Rouge des Prés Beef reared in pasture

| destination | Rouge des Prés/Beef/pasture | | |
| --- | --- | --- | --- |
|  | **Biophysical Partition** | **Mass Partition** | **Economic Partition** |
| Pet Food | 0,0329 | 0,055 | 0,004 |
| PAP C3 | 0,1731 | 0,0636 | 0,0089 |
| Gelatin C3 | 0,0591 | 0,0951 | 0,0003 |
| C1-C2 for disposal | 0 | 0 | 0 |
| Skin tannery C3 | 0,0753 | 0,0783 | 0,1306 |
| Human food | 0,5256 | 0,546 | 0,8416 |
| Fat and greaves C3 | 0,134 | 0,1623 | 0,0146 |
| Spreading/Compost | 0 | 0 | 0 |

Table 81: Total weighting by destination category for Rouge des Prés Young Bulls reared in stall

| destination | Rouge des Prés/YOUNG BULL/stall | | |
| --- | --- | --- | --- |
|  | **Biophysical Partition** | **Mass Partition** | **Economic Partition** |
| Pet Food | 0,0303 | 0,052 | 0,0036 |
| PAP C3 | 0,1461 | 0,0604 | 0,0083 |
| Gelatin C3 | 0,0634 | 0,0963 | 0,0003 |
| C1-C2 for disposal | 0 | 0 | 0 |
| Skin tannery C3 | 0,0708 | 0,0742 | 0,1223 |
| Human food | 0,5067 | 0,5569 | 0,851 |
| Fat and greaves C3 | 0,1824 | 0,1603 | 0,0143 |
| Spreading/Compost | 0 | 0 | 0 |

Table 82: Total weighting by destination category for Rouge des Prés Heifers reared in stall

| destination | Rouge des Prés/HEIFER/stall | | |
| --- | --- | --- | --- |
|  | **Biophysical Partition** | **Mass Partition** | **Economic Partition** |
| Pet Food | 0,0323 | 0,0561 | 0,0041 |
| PAP C3 | 0,1544 | 0,0654 | 0,0092 |
| Gelatin C3 | 0,0611 | 0,0945 | 0,0003 |
| C1-C2 for disposal | 0 | 0 | 0 |
| Skin tannery C3 | 0,0752 | 0,0803 | 0,1349 |
| Human food | 0,4949 | 0,5404 | 0,8367 |
| Fat and greaves C3 | 0,1819 | 0,1632 | 0,0148 |
| Spreading/Compost | 0 | 0 | 0 |

Table 83: Total weighting by destination category for Rouge des Prés Cull Cows reared in stall

| destination | Rouge des Prés/Cull Cows/stall | | |
| --- | --- | --- | --- |
|  | **Biophysical Partition** | **Mass Partition** | **Economic Partition** |
| Pet Food | 0,0339 | 0,0592 | 0,0045 |
| PAP C3 | 0,1656 | 0,0689 | 0,0096 |
| Gelatin C3 | 0,0589 | 0,0934 | 0,0003 |
| C1-C2 for disposal | 0 | 0 | 0 |
| Skin tannery C3 | 0,0779 | 0,0845 | 0,1438 |
| Human food | 0,491 | 0,529 | 0,8264 |
| Fat and greaves C3 | 0,173 | 0,1649 | 0,0153 |
| Spreading/Compost | 0 | 0 | 0 |

Table 84: Total weighting by destination category for Rouge des Prés Beef reared in stall

| destination | Rouge des Prés/Beef/stall | | |
| --- | --- | --- | --- |
|  | **Biophysical Partition** | **Mass Partition** | **Economic Partition** |
| Pet Food | 0,0327 | 0,055 | 0,004 |
| PAP C3 | 0,1677 | 0,0636 | 0,0089 |
| Gelatin C3 | 0,0596 | 0,0951 | 0,0003 |
| C1-C2 for disposal | 0 | 0 | 0 |
| Skin tannery C3 | 0,0743 | 0,0783 | 0,1306 |
| Human food | 0,5174 | 0,546 | 0,8416 |
| Fat and greaves C3 | 0,1482 | 0,1623 | 0,0146 |
| Spreading/Compost | 0 | 0 | 0 |

Table 85: Total weighting by destination category for Charolaise x Rustique Young Bulls reared in grazing large area

| destination | Charolaise x Rustique/YOUNG BULL/grazing large area | | |
| --- | --- | --- | --- |
|  | **Biophysical Partition** | **Mass Partition** | **Economic Partition** |
| Pet Food | 0,0312 | 0,052 | 0,0036 |
| PAP C3 | 0,1555 | 0,0604 | 0,0083 |
| Gelatin C3 | 0,0625 | 0,0963 | 0,0003 |
| C1-C2 for disposal | 0 | 0 | 0 |
| Skin tannery C3 | 0,0726 | 0,0742 | 0,1223 |
| Human food | 0,5228 | 0,5569 | 0,851 |
| Fat and greaves C3 | 0,1557 | 0,1603 | 0,0143 |
| Spreading/Compost | 0 | 0 | 0 |

Table 86: Total weighting by destination category for Charolaise x Rustique Heifers reared in grazing large area

| destination | Charolaise x Rustique/HEIFER/grazing large area | | |
| --- | --- | --- | --- |
|  | **Biophysical Partition** | **Mass Partition** | **Economic Partition** |
| Pet Food | 0,033 | 0,0561 | 0,0041 |
| PAP C3 | 0,1615 | 0,0654 | 0,0092 |
| Gelatin C3 | 0,0606 | 0,0945 | 0,0003 |
| C1-C2 for disposal | 0 | 0 | 0 |
| Skin tannery C3 | 0,0775 | 0,0803 | 0,1349 |
| Human food | 0,5097 | 0,5404 | 0,8367 |
| Fat and greaves C3 | 0,1574 | 0,1632 | 0,0148 |
| Spreading/Compost | 0 | 0 | 0 |

Table 87: Total weighting by destination category for Charolaise x Rustique Cull Cows reared in grazing large area

| destination | Charolaise x Rustique/Cull Cows/grazing large area | | |
| --- | --- | --- | --- |
|  | **Biophysical Partition** | **Mass Partition** | **Economic Partition** |
| Pet Food | 0,0346 | 0,0592 | 0,0045 |
| PAP C3 | 0,1699 | 0,0689 | 0,0096 |
| Gelatin C3 | 0,0588 | 0,0934 | 0,0003 |
| C1-C2 for disposal | 0 | 0 | 0 |
| Skin tannery C3 | 0,0801 | 0,0845 | 0,1438 |
| Human food | 0,5019 | 0,529 | 0,8264 |
| Fat and greaves C3 | 0,1547 | 0,1649 | 0,0153 |
| Spreading/Compost | 0 | 0 | 0 |

Table 88: Total weighting by destination category for Charolaise x Rustique Beef reared in grazing large area

| destination | Charolaise x Rustique/Beef/grazing large area | | |
| --- | --- | --- | --- |
|  | **Biophysical Partition** | **Mass Partition** | **Economic Partition** |
| Pet Food | 0,0326 | 0,055 | 0,004 |
| PAP C3 | 0,1622 | 0,0636 | 0,0089 |
| Gelatin C3 | 0,0607 | 0,0951 | 0,0003 |
| C1-C2 for disposal | 0 | 0 | 0 |
| Skin tannery C3 | 0,0754 | 0,0783 | 0,1306 |
| Human food | 0,5151 | 0,546 | 0,8416 |
| Fat and greaves C3 | 0,1538 | 0,1623 | 0,0146 |
| Spreading/Compost | 0 | 0 | 0 |

Table 89: Total weighting by destination category for Charolaise x Rustique Young Bulls reared in pasture

| destination | Charolaise x Rustique/YOUNG BULL/pasture | | |
| --- | --- | --- | --- |
|  | **Biophysical Partition** | **Mass Partition** | **Economic Partition** |
| Pet Food | 0,0309 | 0,052 | 0,0036 |
| PAP C3 | 0,1498 | 0,0604 | 0,0083 |
| Gelatin C3 | 0,0631 | 0,0963 | 0,0003 |
| C1-C2 for disposal | 0 | 0 | 0 |
| Skin tannery C3 | 0,0718 | 0,0742 | 0,1223 |
| Human food | 0,5142 | 0,5569 | 0,851 |
| Fat and greaves C3 | 0,1702 | 0,1603 | 0,0143 |
| Spreading/Compost | 0 | 0 | 0 |

Table 90: Total weighting by destination category for Charolaise x Rustique Heifers reared in pasture

| destination | Charolaise x Rustique/HEIFER/pasture | | |
| --- | --- | --- | --- |
|  | **Biophysical Partition** | **Mass Partition** | **Economic Partition** |
| Pet Food | 0,0328 | 0,0561 | 0,0041 |
| PAP C3 | 0,1557 | 0,0654 | 0,0092 |
| Gelatin C3 | 0,0614 | 0,0945 | 0,0003 |
| C1-C2 for disposal | 0 | 0 | 0 |
| Skin tannery C3 | 0,0769 | 0,0803 | 0,1349 |
| Human food | 0,5017 | 0,5404 | 0,8367 |
| Fat and greaves C3 | 0,1716 | 0,1632 | 0,0148 |
| Spreading/Compost | 0 | 0 | 0 |

Table 91: Total weighting by destination category for Charolaise x Rustique Cull Cows reared in pasture

| destination | Charolaise x Rustique/Cull Cows/pasture | | |
| --- | --- | --- | --- |
|  | **Biophysical Partition** | **Mass Partition** | **Economic Partition** |
| Pet Food | 0,0342 | 0,0592 | 0,0045 |
| PAP C3 | 0,164 | 0,0689 | 0,0096 |
| Gelatin C3 | 0,0595 | 0,0934 | 0,0003 |
| C1-C2 for disposal | 0 | 0 | 0 |
| Skin tannery C3 | 0,0795 | 0,0845 | 0,1438 |
| Human food | 0,4938 | 0,529 | 0,8264 |
| Fat and greaves C3 | 0,1691 | 0,1649 | 0,0153 |
| Spreading/Compost | 0 | 0 | 0 |

Table 92: Total weighting by destination category for Charolaise x Rustique Beef reared in pasture

| destination | Charolaise x Rustique/Beef/pasture | | |
| --- | --- | --- | --- |
|  | **Biophysical Partition** | **Mass Partition** | **Economic Partition** |
| Pet Food | 0,0324 | 0,055 | 0,004 |
| PAP C3 | 0,1565 | 0,0636 | 0,0089 |
| Gelatin C3 | 0,0613 | 0,0951 | 0,0003 |
| C1-C2 for disposal | 0 | 0 | 0 |
| Skin tannery C3 | 0,0746 | 0,0783 | 0,1306 |
| Human food | 0,507 | 0,546 | 0,8416 |
| Fat and greaves C3 | 0,1683 | 0,1623 | 0,0146 |
| Spreading/Compost | 0 | 0 | 0 |

Table 93: Total weighting by destination category for Charolaise x Rustique Young Bulls reared in stall

| destination | Charolaise x Rustique/YOUNG BULL/stall | | |
| --- | --- | --- | --- |
|  | **Biophysical Partition** | **Mass Partition** | **Economic Partition** |
| Pet Food | 0,0304 | 0,052 | 0,0036 |
| PAP C3 | 0,1437 | 0,0604 | 0,0083 |
| Gelatin C3 | 0,0639 | 0,0963 | 0,0003 |
| C1-C2 for disposal | 0 | 0 | 0 |
| Skin tannery C3 | 0,071 | 0,0742 | 0,1223 |
| Human food | 0,505 | 0,5569 | 0,851 |
| Fat and greaves C3 | 0,1859 | 0,1603 | 0,0143 |
| Spreading/Compost | 0 | 0 | 0 |

Table 94: Total weighting by destination category for Charolaise x Rustique Heifers reared in stall

| destination | Charolaise x Rustique/HEIFER/stall | | |
| --- | --- | --- | --- |
|  | **Biophysical Partition** | **Mass Partition** | **Economic Partition** |
| Pet Food | 0,0325 | 0,0561 | 0,0041 |
| PAP C3 | 0,1492 | 0,0654 | 0,0092 |
| Gelatin C3 | 0,0622 | 0,0945 | 0,0003 |
| C1-C2 for disposal | 0 | 0 | 0 |
| Skin tannery C3 | 0,0762 | 0,0803 | 0,1349 |
| Human food | 0,4928 | 0,5404 | 0,8367 |
| Fat and greaves C3 | 0,1869 | 0,1632 | 0,0148 |
| Spreading/Compost | 0 | 0 | 0 |

Table 95: Total weighting by destination category for Charolaise x Rustique Cull Cows reared in stall

| destination | Charolaise x Rustique/Cull Cows/stall | | |
| --- | --- | --- | --- |
|  | **Biophysical Partition** | **Mass Partition** | **Economic Partition** |
| Pet Food | 0,0338 | 0,0592 | 0,0045 |
| PAP C3 | 0,1576 | 0,0689 | 0,0096 |
| Gelatin C3 | 0,0602 | 0,0934 | 0,0003 |
| C1-C2 for disposal | 0 | 0 | 0 |
| Skin tannery C3 | 0,0787 | 0,0845 | 0,1438 |
| Human food | 0,4852 | 0,529 | 0,8264 |
| Fat and greaves C3 | 0,1846 | 0,1649 | 0,0153 |
| Spreading/Compost | 0 | 0 | 0 |

Table 96: Total weighting by destination category for Charolaise x Rustique Beef reared in stall

| destination | Charolaise x Rustique/Beef/stall | | |
| --- | --- | --- | --- |
|  | **Biophysical Partition** | **Mass Partition** | **Economic Partition** |
| Pet Food | 0,0321 | 0,055 | 0,004 |
| PAP C3 | 0,1504 | 0,0636 | 0,0089 |
| Gelatin C3 | 0,0621 | 0,0951 | 0,0003 |
| C1-C2 for disposal | 0 | 0 | 0 |
| Skin tannery C3 | 0,0738 | 0,0783 | 0,1306 |
| Human food | 0,4979 | 0,546 | 0,8416 |
| Fat and greaves C3 | 0,1838 | 0,1623 | 0,0146 |
| Spreading/Compost | 0 | 0 | 0 |

Table 97: Total weighting by destination category for Montbéliarde Young Bulls reared in grazing large area

| destination | Montbéliarde/YOUNG BULL/grazing large area | | |
| --- | --- | --- | --- |
|  | **Biophysical Partition** | **Mass Partition** | **Economic Partition** |
| Pet Food | 0,0325 | 0,055 | 0,004 |
| PAP C3 | 0,16 | 0,0636 | 0,0089 |
| Gelatin C3 | 0,061 | 0,0951 | 0,0003 |
| C1-C2 for disposal | 0 | 0 | 0 |
| Skin tannery C3 | 0,0757 | 0,0783 | 0,1306 |
| Human food | 0,5137 | 0,546 | 0,8416 |
| Fat and greaves C3 | 0,1564 | 0,1623 | 0,0146 |
| Spreading/Compost | 0 | 0 | 0 |

Table 98: Total weighting by destination category for Montbéliarde Heifers reared in grazing large area

| destination | Montbéliarde/HEIFER/grazing large area | | |
| --- | --- | --- | --- |
|  | **Biophysical Partition** | **Mass Partition** | **Economic Partition** |
| Pet Food | 0,0348 | 0,0592 | 0,0045 |
| PAP C3 | 0,164 | 0,0689 | 0,0096 |
| Gelatin C3 | 0,0601 | 0,0934 | 0,0003 |
| C1-C2 for disposal | 0 | 0 | 0 |
| Skin tannery C3 | 0,0823 | 0,0845 | 0,1438 |
| Human food | 0,5017 | 0,529 | 0,8264 |
| Fat and greaves C3 | 0,1568 | 0,1649 | 0,0153 |
| Spreading/Compost | 0 | 0 | 0 |

Table 99: Total weighting by destination category for Montbéliarde Cull Cows reared in grazing large area

| destination | Montbéliarde/Cull Cows/grazing large area | | |
| --- | --- | --- | --- |
|  | **Biophysical Partition** | **Mass Partition** | **Economic Partition** |
| Pet Food | 0,0361 | 0,0623 | 0,0046 |
| PAP C3 | 0,1724 | 0,0722 | 0,0102 |
| Gelatin C3 | 0,0578 | 0,092 | 0,0003 |
| C1-C2 for disposal | 0 | 0 | 0 |
| Skin tannery C3 | 0,0838 | 0,0886 | 0,1528 |
| Human food | 0,4937 | 0,5177 | 0,8162 |
| Fat and greaves C3 | 0,1565 | 0,1672 | 0,0158 |
| Spreading/Compost | 0 | 0 | 0 |

Table 100: Total weighting by destination category for Montbéliarde Beef reared in grazing large area

| destination | Montbéliarde/Beef/grazing large area | | |
| --- | --- | --- | --- |
|  | **Biophysical Partition** | **Mass Partition** | **Economic Partition** |
| Pet Food | 0,0339 | 0,0577 | 0,0041 |
| PAP C3 | 0,1657 | 0,0674 | 0,0094 |
| Gelatin C3 | 0,0597 | 0,0939 | 0,0003 |
| C1-C2 for disposal | 0 | 0 | 0 |
| Skin tannery C3 | 0,0788 | 0,0824 | 0,1393 |
| Human food | 0,5056 | 0,5349 | 0,8317 |
| Fat and greaves C3 | 0,1563 | 0,164 | 0,015 |
| Spreading/Compost | 0 | 0 | 0 |

Table 101: Total weighting by destination category for Montbéliarde Young Bulls reared in pasture

| destination | Montbéliarde/YOUNG BULL/pasture | | |
| --- | --- | --- | --- |
|  | **Biophysical Partition** | **Mass Partition** | **Economic Partition** |
| Pet Food | 0,0322 | 0,055 | 0,004 |
| PAP C3 | 0,1545 | 0,0636 | 0,0089 |
| Gelatin C3 | 0,0618 | 0,0951 | 0,0003 |
| C1-C2 for disposal | 0 | 0 | 0 |
| Skin tannery C3 | 0,075 | 0,0783 | 0,1306 |
| Human food | 0,5058 | 0,546 | 0,8416 |
| Fat and greaves C3 | 0,1708 | 0,1623 | 0,0146 |
| Spreading/Compost | 0 | 0 | 0 |

Table 102: Total weighting by destination category for Montbéliarde Heifers reared in pasture

| destination | Montbéliarde/HEIFER/pasture | | |
| --- | --- | --- | --- |
|  | **Biophysical Partition** | **Mass Partition** | **Economic Partition** |
| Pet Food | 0,0344 | 0,0592 | 0,0045 |
| PAP C3 | 0,158 | 0,0689 | 0,0096 |
| Gelatin C3 | 0,0609 | 0,0934 | 0,0003 |
| C1-C2 for disposal | 0 | 0 | 0 |
| Skin tannery C3 | 0,0818 | 0,0845 | 0,1438 |
| Human food | 0,4943 | 0,529 | 0,8264 |
| Fat and greaves C3 | 0,1703 | 0,1649 | 0,0153 |
| Spreading/Compost | 0 | 0 | 0 |

Table 103: Total weighting by destination category for Montbéliarde Cull Cows reared in pasture

| destination | Montbéliarde/Cull Cows/pasture | | |
| --- | --- | --- | --- |
|  | **Biophysical Partition** | **Mass Partition** | **Economic Partition** |
| Pet Food | 0,0359 | 0,0623 | 0,0046 |
| PAP C3 | 0,1664 | 0,0722 | 0,0102 |
| Gelatin C3 | 0,0584 | 0,092 | 0,0003 |
| C1-C2 for disposal | 0 | 0 | 0 |
| Skin tannery C3 | 0,0832 | 0,0886 | 0,1528 |
| Human food | 0,4859 | 0,5177 | 0,8162 |
| Fat and greaves C3 | 0,1703 | 0,1672 | 0,0158 |
| Spreading/Compost | 0 | 0 | 0 |

Table 104: Total weighting by destination category for Montbéliarde Beef reared in pasture

| destination | Montbéliarde/Beef/pasture | | |
| --- | --- | --- | --- |
|  | **Biophysical Partition** | **Mass Partition** | **Economic Partition** |
| Pet Food | 0,0336 | 0,0577 | 0,0041 |
| PAP C3 | 0,1596 | 0,0674 | 0,0094 |
| Gelatin C3 | 0,0604 | 0,0939 | 0,0003 |
| C1-C2 for disposal | 0 | 0 | 0 |
| Skin tannery C3 | 0,0782 | 0,0824 | 0,1393 |
| Human food | 0,4975 | 0,5349 | 0,8317 |
| Fat and greaves C3 | 0,1705 | 0,164 | 0,015 |
| Spreading/Compost | 0 | 0 | 0 |

Table 105: Total weighting by destination category for Montbéliarde Young Bulls reared in stall

| destination | Montbéliarde/YOUNG BULL/stall | | |
| --- | --- | --- | --- |
|  | **Biophysical Partition** | **Mass Partition** | **Economic Partition** |
| Pet Food | 0,032 | 0,055 | 0,004 |
| PAP C3 | 0,148 | 0,0636 | 0,0089 |
| Gelatin C3 | 0,0626 | 0,0951 | 0,0003 |
| C1-C2 for disposal | 0 | 0 | 0 |
| Skin tannery C3 | 0,0742 | 0,0783 | 0,1306 |
| Human food | 0,4966 | 0,546 | 0,8416 |
| Fat and greaves C3 | 0,1864 | 0,1623 | 0,0146 |
| Spreading/Compost | 0 | 0 | 0 |

Table 106: Total weighting by destination category for Montbéliarde Heifers reared in stall

| destination | Montbéliarde/HEIFER/stall | | |
| --- | --- | --- | --- |
|  | **Biophysical Partition** | **Mass Partition** | **Economic Partition** |
| Pet Food | 0,0343 | 0,0592 | 0,0045 |
| PAP C3 | 0,1513 | 0,0689 | 0,0096 |
| Gelatin C3 | 0,0617 | 0,0934 | 0,0003 |
| C1-C2 for disposal | 0 | 0 | 0 |
| Skin tannery C3 | 0,0814 | 0,0845 | 0,1438 |
| Human food | 0,4861 | 0,529 | 0,8264 |
| Fat and greaves C3 | 0,1849 | 0,1649 | 0,0153 |
| Spreading/Compost | 0 | 0 | 0 |

Table 107: Total weighting by destination category for Montbéliarde Cull Cows reared in stall

| destination | Montbéliarde/Cull Cows/stall | | |
| --- | --- | --- | --- |
|  | **Biophysical Partition** | **Mass Partition** | **Economic Partition** |
| Pet Food | 0,0354 | 0,0623 | 0,0046 |
| PAP C3 | 0,1595 | 0,0722 | 0,0102 |
| Gelatin C3 | 0,0592 | 0,092 | 0,0003 |
| C1-C2 for disposal | 0 | 0 | 0 |
| Skin tannery C3 | 0,0826 | 0,0886 | 0,1528 |
| Human food | 0,4775 | 0,5177 | 0,8162 |
| Fat and greaves C3 | 0,1856 | 0,1672 | 0,0158 |
| Spreading/Compost | 0 | 0 | 0 |

Table 108: Total weighting by destination category for Montbéliarde Beef reared in stall

| destination | Montbéliarde/Beef/stall | | |
| --- | --- | --- | --- |
|  | **Biophysical Partition** | **Mass Partition** | **Economic Partition** |
| Pet Food | 0,0333 | 0,0577 | 0,0041 |
| PAP C3 | 0,1533 | 0,0674 | 0,0094 |
| Gelatin C3 | 0,0612 | 0,0939 | 0,0003 |
| C1-C2 for disposal | 0 | 0 | 0 |
| Skin tannery C3 | 0,0774 | 0,0824 | 0,1393 |
| Human food | 0,4888 | 0,5349 | 0,8317 |
| Fat and greaves C3 | 0,1859 | 0,164 | 0,015 |
| Spreading/Compost | 0 | 0 | 0 |

Table 109: Total weighting by destination category for Charolaise x Pie Noire Young Bulls reared in grazing large area

| destination | Charolaise x Pie Noire/YOUNG BULL/grazing large area | | |
| --- | --- | --- | --- |
|  | **Biophysical Partition** | **Mass Partition** | **Economic Partition** |
| Pet Food | 0,0314 | 0,0531 | 0,0037 |
| PAP C3 | 0,159 | 0,0621 | 0,0085 |
| Gelatin C3 | 0,0615 | 0,0958 | 0,0003 |
| C1-C2 for disposal | 0 | 0 | 0 |
| Skin tannery C3 | 0,0739 | 0,0762 | 0,1264 |
| Human food | 0,5195 | 0,5516 | 0,8463 |
| Fat and greaves C3 | 0,1543 | 0,1613 | 0,0145 |
| Spreading/Compost | 0 | 0 | 0 |

Table 110: Total weighting by destination category for Charolaise x Pie Noire Young Heifers reared in grazing large area

| destination | Charolaise x Pie Noire/HEIFER/grazing large area | | |
| --- | --- | --- | --- |
|  | **Biophysical Partition** | **Mass Partition** | **Economic Partition** |
| Pet Food | 0,0345 | 0,0592 | 0,0045 |
| PAP C3 | 0,1691 | 0,0689 | 0,0096 |
| Gelatin C3 | 0,059 | 0,0934 | 0,0003 |
| C1-C2 for disposal | 0 | 0 | 0 |
| Skin tannery C3 | 0,0803 | 0,0845 | 0,1438 |
| Human food | 0,5014 | 0,529 | 0,8264 |
| Fat and greaves C3 | 0,1557 | 0,1649 | 0,0153 |
| Spreading/Compost | 0 | 0 | 0 |

Table 111: Total weighting by destination category for Charolaise x Pie Noire Cull Cows reared in grazing large area

| destination | Charolaise x Pie Noire/Cull Cows/grazing large area | | |
| --- | --- | --- | --- |
|  | **Biophysical Partition** | **Mass Partition** | **Economic Partition** |
| Pet Food | 0,035 | 0,0607 | 0,0046 |
| PAP C3 | 0,1734 | 0,0707 | 0,01 |
| Gelatin C3 | 0,0578 | 0,0928 | 0,0003 |
| C1-C2 for disposal | 0 | 0 | 0 |
| Skin tannery C3 | 0,0813 | 0,0865 | 0,1483 |
| Human food | 0,4986 | 0,5233 | 0,8212 |
| Fat and greaves C3 | 0,1537 | 0,166 | 0,0155 |
| Spreading/Compost | 0 | 0 | 0 |

Table 112: Total weighting by destination category for Charolaise x Pie Noire Beef reared in grazing large area

| destination | Charolaise x Pie Noire/Beef/grazing large area | | |
| --- | --- | --- | --- |
|  | **Biophysical Partition** | **Mass Partition** | **Economic Partition** |
| Pet Food | 0,033 | 0,0561 | 0,0041 |
| PAP C3 | 0,1659 | 0,0654 | 0,0092 |
| Gelatin C3 | 0,0598 | 0,0945 | 0,0003 |
| C1-C2 for disposal | 0 | 0 | 0 |
| Skin tannery C3 | 0,0768 | 0,0803 | 0,1349 |
| Human food | 0,5119 | 0,5404 | 0,8367 |
| Fat and greaves C3 | 0,1522 | 0,1632 | 0,0148 |
| Spreading/Compost | 0 | 0 | 0 |

Table 113: Total weighting by destination category for Charolaise x Pie Noire Young Bulls reared in pasture

| destination | Charolaise x Pie Noire/YOUNG BULL/pasture | | |
| --- | --- | --- | --- |
|  | **Biophysical Partition** | **Mass Partition** | **Economic Partition** |
| Pet Food | 0,0311 | 0,0531 | 0,0037 |
| PAP C3 | 0,1534 | 0,0621 | 0,0085 |
| Gelatin C3 | 0,0621 | 0,0958 | 0,0003 |
| C1-C2 for disposal | 0 | 0 | 0 |
| Skin tannery C3 | 0,0731 | 0,0762 | 0,1264 |
| Human food | 0,5112 | 0,5516 | 0,8463 |
| Fat and greaves C3 | 0,1688 | 0,1613 | 0,0145 |
| Spreading/Compost | 0 | 0 | 0 |

Table 114: Total weighting by destination category for Charolaise x Pie Noire Young Heifers reared in pasture

| destination | Charolaise x Pie Noire/HEIFER/pasture | | |
| --- | --- | --- | --- |
|  | **Biophysical Partition** | **Mass Partition** | **Economic Partition** |
| Pet Food | 0,0341 | 0,0592 | 0,0045 |
| PAP C3 | 0,1629 | 0,0689 | 0,0096 |
| Gelatin C3 | 0,0597 | 0,0934 | 0,0003 |
| C1-C2 for disposal | 0 | 0 | 0 |
| Skin tannery C3 | 0,0797 | 0,0845 | 0,1438 |
| Human food | 0,4935 | 0,529 | 0,8264 |
| Fat and greaves C3 | 0,1699 | 0,1649 | 0,0153 |
| Spreading/Compost | 0 | 0 | 0 |

Table 115: Total weighting by destination category for Charolaise x Pie Noire Cull Cows reared in pasture

| destination | Charolaise x Pie Noire/Cull Cows/pasture | | |
| --- | --- | --- | --- |
|  | **Biophysical Partition** | **Mass Partition** | **Economic Partition** |
| Pet Food | 0,0347 | 0,0607 | 0,0046 |
| PAP C3 | 0,1674 | 0,0707 | 0,01 |
| Gelatin C3 | 0,0584 | 0,0928 | 0,0003 |
| C1-C2 for disposal | 0 | 0 | 0 |
| Skin tannery C3 | 0,0807 | 0,0865 | 0,1483 |
| Human food | 0,4906 | 0,5233 | 0,8212 |
| Fat and greaves C3 | 0,1681 | 0,166 | 0,0155 |
| Spreading/Compost | 0 | 0 | 0 |

Table 116: Total weighting by destination category for Charolaise x Pie Noire Beef reared in pasture

| destination | Charolaise x Pie Noire/Beef/pasture | | |
| --- | --- | --- | --- |
|  | **Biophysical Partition** | **Mass Partition** | **Economic Partition** |
| Pet Food | 0,0328 | 0,0561 | 0,0041 |
| PAP C3 | 0,1603 | 0,0654 | 0,0092 |
| Gelatin C3 | 0,0604 | 0,0945 | 0,0003 |
| C1-C2 for disposal | 0 | 0 | 0 |
| Skin tannery C3 | 0,076 | 0,0803 | 0,1349 |
| Human food | 0,5038 | 0,5404 | 0,8367 |
| Fat and greaves C3 | 0,1667 | 0,1632 | 0,0148 |
| Spreading/Compost | 0 | 0 | 0 |

Table 117: Total weighting by destination category for Charolaise x Pie Noire Young Bulls reared in stall

| destination | Charolaise x Pie Noire/YOUNG BULL/stall | | |
| --- | --- | --- | --- |
|  | **Biophysical Partition** | **Mass Partition** | **Economic Partition** |
| Pet Food | 0,0307 | 0,0531 | 0,0037 |
| PAP C3 | 0,1473 | 0,0621 | 0,0085 |
| Gelatin C3 | 0,0629 | 0,0958 | 0,0003 |
| C1-C2 for disposal | 0 | 0 | 0 |
| Skin tannery C3 | 0,0724 | 0,0762 | 0,1264 |
| Human food | 0,502 | 0,5516 | 0,8463 |
| Fat and greaves C3 | 0,1845 | 0,1613 | 0,0145 |
| Spreading/Compost | 0 | 0 | 0 |

Table 118: Total weighting by destination category for Charolaise x Pie Noire Heifers reared in stall

| destination | Charolaise x Pie Noire/HEIFER/stall | | |
| --- | --- | --- | --- |
|  | **Biophysical Partition** | **Mass Partition** | **Economic Partition** |
| Pet Food | 0,0337 | 0,0592 | 0,0045 |
| PAP C3 | 0,1565 | 0,0689 | 0,0096 |
| Gelatin C3 | 0,0605 | 0,0934 | 0,0003 |
| C1-C2 for disposal | 0 | 0 | 0 |
| Skin tannery C3 | 0,0789 | 0,0845 | 0,1438 |
| Human food | 0,4847 | 0,529 | 0,8264 |
| Fat and greaves C3 | 0,1854 | 0,1649 | 0,0153 |
| Spreading/Compost | 0 | 0 | 0 |

Table 119: Total weighting by destination category for Charolaise x Pie Noire Cull Cows reared in stall

| destination | Charolaise x Pie Noire/Cull Cows/stall | | |
| --- | --- | --- | --- |
|  | **Biophysical Partition** | **Mass Partition** | **Economic Partition** |
| Pet Food | 0,0343 | 0,0607 | 0,0046 |
| PAP C3 | 0,1611 | 0,0707 | 0,01 |
| Gelatin C3 | 0,0592 | 0,0928 | 0,0003 |
| C1-C2 for disposal | 0 | 0 | 0 |
| Skin tannery C3 | 0,0799 | 0,0865 | 0,1483 |
| Human food | 0,482 | 0,5233 | 0,8212 |
| Fat and greaves C3 | 0,1839 | 0,166 | 0,0155 |
| Spreading/Compost | 0 | 0 | 0 |

Table 120: Total weighting by destination category for Charolaise x Pie Noire Beef reared in stall

| destination | Charolaise x Pie Noire/Beef/stall | | |
| --- | --- | --- | --- |
|  | **Biophysical Partition** | **Mass Partition** | **Economic Partition** |
| Pet Food | 0,0324 | 0,0561 | 0,0041 |
| PAP C3 | 0,1541 | 0,0654 | 0,0092 |
| Gelatin C3 | 0,0612 | 0,0945 | 0,0003 |
| C1-C2 for disposal | 0 | 0 | 0 |
| Skin tannery C3 | 0,0753 | 0,0803 | 0,1349 |
| Human food | 0,4949 | 0,5404 | 0,8367 |
| Fat and greaves C3 | 0,1823 | 0,1632 | 0,0148 |
| Spreading/Compost | 0 | 0 | 0 |

Table 121: Total weighting by destination category for Average Young Bulls reared in grazing large area

| destination | Average/YOUNG BULL/grazing large area | | |
| --- | --- | --- | --- |
|  | **Biophysical Partition** | **Mass Partition** | **Economic Partition** |
| Pet Food | 0,0312 | 0,0521 | 0,0036 |
| PAP C3 | 0,1551 | 0,0605 | 0,0083 |
| Gelatin C3 | 0,0625 | 0,0963 | 0,0003 |
| C1-C2 for disposal | 0 | 0 | 0 |
| Skin tannery C3 | 0,0728 | 0,0744 | 0,1226 |
| Human food | 0,5221 | 0,5565 | 0,8505 |
| Fat and greaves C3 | 0,1562 | 0,1604 | 0,0143 |
| Spreading/Compost | 0 | 0 | 0 |

Table 122: Total weighting by destination category for Average Heifers reared in grazing large area

| destination | Average/HEIFER/grazing large area | | |
| --- | --- | --- | --- |
|  | **Biophysical Partition** | **Mass Partition** | **Economic Partition** |
| Pet Food | 0,0332 | 0,0569 | 0,0041 |
| PAP C3 | 0,1629 | 0,0663 | 0,0093 |
| Gelatin C3 | 0,0603 | 0,0942 | 0,0003 |
| C1-C2 for disposal | 0 | 0 | 0 |
| Skin tannery C3 | 0,0781 | 0,081 | 0,1365 |
| Human food | 0,5078 | 0,5382 | 0,8347 |
| Fat and greaves C3 | 0,1574 | 0,1635 | 0,0149 |
| Spreading/Compost | 0 | 0 | 0 |

Table 123: Total weighting by destination category for Average Cull Cows reared in grazing large area

| destination | Average/Cull Cows/grazing large area | | |
| --- | --- | --- | --- |
|  | **Biophysical Partition** | **Mass Partition** | **Economic Partition** |
| Pet Food | 0,0349 | 0,0604 | 0,0045 |
| PAP C3 | 0,1732 | 0,0701 | 0,0099 |
| Gelatin C3 | 0,0579 | 0,0929 | 0,0003 |
| C1-C2 for disposal | 0 | 0 | 0 |
| Skin tannery C3 | 0,0807 | 0,0858 | 0,1468 |
| Human food | 0,5003 | 0,525 | 0,823 |
| Fat and greaves C3 | 0,1529 | 0,1657 | 0,0154 |
| Spreading/Compost | 0 | 0 | 0 |

Table 124: Total weighting by destination category for Average Beef reared in grazing large area

| destination | Average/Beef/grazing large area | | |
| --- | --- | --- | --- |
|  | **Biophysical Partition** | **Mass Partition** | **Economic Partition** |
| Pet Food | 0,0315 | 0,0531 | 0,0037 |
| PAP C3 | 0,1639 | 0,0621 | 0,0085 |
| Gelatin C3 | 0,0607 | 0,0958 | 0,0003 |
| C1-C2 for disposal | 0 | 0 | 0 |
| Skin tannery C3 | 0,0738 | 0,0762 | 0,1264 |
| Human food | 0,5238 | 0,5516 | 0,8463 |
| Fat and greaves C3 | 0,146 | 0,1613 | 0,0145 |
| Spreading/Compost | 0 | 0 | 0 |

Table 125: Total weighting by destination category for Average Young Bulls reared in pasture

| destination | Average/YOUNG BULL/pasture | | |
| --- | --- | --- | --- |
|  | **Biophysical Partition** | **Mass Partition** | **Economic Partition** |
| Pet Food | 0,031 | 0,0521 | 0,0036 |
| PAP C3 | 0,1493 | 0,0605 | 0,0083 |
| Gelatin C3 | 0,0632 | 0,0963 | 0,0003 |
| C1-C2 for disposal | 0 | 0 | 0 |
| Skin tannery C3 | 0,072 | 0,0744 | 0,1226 |
| Human food | 0,5137 | 0,5565 | 0,8505 |
| Fat and greaves C3 | 0,1706 | 0,1604 | 0,0143 |
| Spreading/Compost | 0 | 0 | 0 |

Table 126: Total weighting by destination category for Average Heifers reared in pasture

| destination | Average/HEIFER/pasture | | |
| --- | --- | --- | --- |
|  | **Biophysical Partition** | **Mass Partition** | **Economic Partition** |
| Pet Food | 0,033 | 0,0569 | 0,0041 |
| PAP C3 | 0,157 | 0,0663 | 0,0093 |
| Gelatin C3 | 0,0611 | 0,0942 | 0,0003 |
| C1-C2 for disposal | 0 | 0 | 0 |
| Skin tannery C3 | 0,0775 | 0,081 | 0,1365 |
| Human food | 0,4999 | 0,5382 | 0,8347 |
| Fat and greaves C3 | 0,1715 | 0,1635 | 0,0149 |
| Spreading/Compost | 0 | 0 | 0 |

Table 127: Total weighting by destination category for Average Cull Cows reared in pasture

| destination | Average/Cull Cows/pasture | | |
| --- | --- | --- | --- |
|  | **Biophysical Partition** | **Mass Partition** | **Economic Partition** |
| Pet Food | 0,0346 | 0,0604 | 0,0045 |
| PAP C3 | 0,1672 | 0,0701 | 0,0099 |
| Gelatin C3 | 0,0585 | 0,0929 | 0,0003 |
| C1-C2 for disposal | 0 | 0 | 0 |
| Skin tannery C3 | 0,0799 | 0,0858 | 0,1468 |
| Human food | 0,4924 | 0,525 | 0,823 |
| Fat and greaves C3 | 0,1673 | 0,1657 | 0,0154 |
| Spreading/Compost | 0 | 0 | 0 |

Table 128: Total weighting by destination category for Average Beef reared in pasture

| destination | Average/Beef/pasture | | |
| --- | --- | --- | --- |
|  | **Biophysical Partition** | **Mass Partition** | **Economic Partition** |
| Pet Food | 0,0312 | 0,0531 | 0,0037 |
| PAP C3 | 0,1585 | 0,0621 | 0,0085 |
| Gelatin C3 | 0,0614 | 0,0958 | 0,0003 |
| C1-C2 for disposal | 0 | 0 | 0 |
| Skin tannery C3 | 0,0729 | 0,0762 | 0,1264 |
| Human food | 0,5156 | 0,5516 | 0,8463 |
| Fat and greaves C3 | 0,1602 | 0,1613 | 0,0145 |
| Spreading/Compost | 0 | 0 | 0 |

Table 129: Total weighting by destination category for Average Young Bulls reared in stall

| destination | Average/YOUNG BULL/stall | | |
| --- | --- | --- | --- |
|  | **Biophysical Partition** | **Mass Partition** | **Economic Partition** |
| Pet Food | 0,0304 | 0,0521 | 0,0036 |
| PAP C3 | 0,1433 | 0,0605 | 0,0083 |
| Gelatin C3 | 0,0639 | 0,0963 | 0,0003 |
| C1-C2 for disposal | 0 | 0 | 0 |
| Skin tannery C3 | 0,0712 | 0,0744 | 0,1226 |
| Human food | 0,5046 | 0,5565 | 0,8505 |
| Fat and greaves C3 | 0,1863 | 0,1604 | 0,0143 |
| Spreading/Compost | 0 | 0 | 0 |

Table 130: Total weighting by destination category for Average Heifers reared in stall

| destination | Average/HEIFER/stall | | |
| --- | --- | --- | --- |
|  | **Biophysical Partition** | **Mass Partition** | **Economic Partition** |
| Pet Food | 0,0327 | 0,0569 | 0,0041 |
| PAP C3 | 0,1506 | 0,0663 | 0,0093 |
| Gelatin C3 | 0,0619 | 0,0942 | 0,0003 |
| C1-C2 for disposal | 0 | 0 | 0 |
| Skin tannery C3 | 0,0767 | 0,081 | 0,1365 |
| Human food | 0,4912 | 0,5382 | 0,8347 |
| Fat and greaves C3 | 0,1868 | 0,1635 | 0,0149 |
| Spreading/Compost | 0 | 0 | 0 |

Table 131: Total weighting by destination category for Average Cull Cows reared in stall

| destination | Average/Cull Cows/stall | | |
| --- | --- | --- | --- |
|  | **Biophysical Partition** | **Mass Partition** | **Economic Partition** |
| Pet Food | 0,0342 | 0,0604 | 0,0045 |
| PAP C3 | 0,1609 | 0,0701 | 0,0099 |
| Gelatin C3 | 0,0593 | 0,0929 | 0,0003 |
| C1-C2 for disposal | 0 | 0 | 0 |
| Skin tannery C3 | 0,0792 | 0,0858 | 0,1468 |
| Human food | 0,4836 | 0,525 | 0,823 |
| Fat and greaves C3 | 0,183 | 0,1657 | 0,0154 |
| Spreading/Compost | 0 | 0 | 0 |

Table 132: Total weighting by destination category for Average Beef reared in stall

| destination | Average/Beef/stall | | |
| --- | --- | --- | --- |
|  | **Biophysical Partition** | **Mass Partition** | **Economic Partition** |
| Pet Food | 0,0309 | 0,0531 | 0,0037 |
| PAP C3 | 0,1526 | 0,0621 | 0,0085 |
| Gelatin C3 | 0,062 | 0,0958 | 0,0003 |
| C1-C2 for disposal | 0 | 0 | 0 |
| Skin tannery C3 | 0,0721 | 0,0762 | 0,1264 |
| Human food | 0,5064 | 0,5516 | 0,8463 |
| Fat and greaves C3 | 0,1758 | 0,1613 | 0,0145 |
| Spreading/Compost | 0 | 0 | 0 |
