# Supplementary material for: Allocation factors for meat coproducts: Dataset to perform life cycle assessment at slaughterhouse
Source: Data Brief. 2020 Nov 23;33:106558. doi: 10.1016/j.dib.2020.106558 (PMC7718151; doi:10.1016/j.dib.2020.106558)
Supplement: Supplementary file 4 [file mmc4.docx]

Table 1: Allocation factors for Average Milk-fed Calf reared in Grazing Large Area

| COPRODUCT | Destination | Average/milk-fed calf/grazing large area | | |
| --- | --- | --- | --- | --- |
|  |  | **Biophysical Allocation Factor** | **Mass Allocation Factor** | **Economic Allocation Factor** |
| Abomasum | Human food | 0.0040 | 0.0044 | 0.0002 |
| Aponevrosis (1%) | Human food | 0.0048 | 0.0044 | 0.0031 |
| Bile | PAP C3 | 0.0016 | 0.0044 | 0.0003 |
| Blood | C1-C2 for disposal | 0.0000 | 0.0000 | 0.0000 |
| Bones (11%) | Gelatin C3 | 0.0046 | 0.0044 | 0.0000 |
| Dead individuals | C1-C2 for disposal | 0.0000 | 0.0000 | 0.0000 |
| Fat (8%) | Fat and greaves C3 | 0.0111 | 0.0044 | 0.0003 |
| Fat from breasts and penis | Fat and greaves C3 | 0.0111 | 0.0044 | 0.0003 |
| Feet (without hooves) | Human food | 0.0040 | 0.0044 | 0.0000 |
| Floatation fat | C1-C2 for disposal | 0.0000 | 0.0000 | 0.0000 |
| Head | Human food | 0.0045 | 0.0044 | 0.0071 |
| Intestines | C1-C2 for disposal | 0.0000 | 0.0000 | 0.0000 |
| Kidney | Human food | 0.0039 | 0.0044 | 0.0033 |
| Manure | Spreading/Compost | 0.0000 | 0.0000 | 0.0000 |
| Meat | Human food | 0.0039 | 0.0044 | 0.0064 |
| Pluck | Human food | 0.0043 | 0.0044 | 0.0013 |
| Rumen and forestomach | Human food | 0.0040 | 0.0044 | 0.0002 |
| SPA C3 | PAP C3 | 0.0002 | 0.0044 | 0.0003 |
| Screening and sifting wastes | C1-C2 for disposal | 0.0000 | 0.0000 | 0.0000 |
| Skin | Skin tannery C3 | 0.0055 | 0.0044 | 0.0038 |
| Sludge | Spreading/Compost | 0.0000 | 0.0000 | 0.0000 |
| Spleen | Pet food | 0.0040 | 0.0044 | 0.0002 |
| Sweetbread | Human food | 0.0049 | 0.0044 | 0.0049 |

Table 2: Allocation factors for Average Milk-fed Calf reared in Pasture

| COPRODUCT | Destination | Average/milk-fed calf/PASTURE | | |
| --- | --- | --- | --- | --- |
|  |  | **Biophysical Allocation Factor** | **Mass Allocation Factor** | **Economic Allocation Factor** |
| Abomasum | Human food | 0.0040 | 0.0044 | 0.0002 |
| Aponevrosis (1%) | Human food | 0.0048 | 0.0044 | 0.0031 |
| Bile | PAP C3 | 0.0016 | 0.0044 | 0.0003 |
| Blood | C1-C2 for disposal | 0.0000 | 0.0000 | 0.0000 |
| Bones (11%) | Gelatin C3 | 0.0045 | 0.0044 | 0.0000 |
| Dead individuals | C1-C2 for disposal | 0.0000 | 0.0000 | 0.0000 |
| Fat (8%) | Fat and greaves C3 | 0.0116 | 0.0044 | 0.0003 |
| Fat from breasts and penis | Fat and greaves C3 | 0.0116 | 0.0044 | 0.0003 |
| Feet (without hooves) | Human food | 0.0040 | 0.0044 | 0.0000 |
| Floatation fat | C1-C2 for disposal | 0.0000 | 0.0000 | 0.0000 |
| Head | Human food | 0.0045 | 0.0044 | 0.0071 |
| Intestines | C1-C2 for disposal | 0.0000 | 0.0000 | 0.0000 |
| Kidney | Human food | 0.0039 | 0.0044 | 0.0033 |
| Manure | Spreading/Compost | 0.0000 | 0.0000 | 0.0000 |
| Meat | Human food | 0.0039 | 0.0044 | 0.0064 |
| Pluck | Human food | 0.0042 | 0.0044 | 0.0013 |
| Rumen and forestomach | Human food | 0.0040 | 0.0044 | 0.0002 |
| SPA C3 | PAP C3 | 0.0002 | 0.0044 | 0.0003 |
| Screening and sifting wastes | C1-C2 for disposal | 0.0000 | 0.0000 | 0.0000 |
| Skin | Skin tannery C3 | 0.0054 | 0.0044 | 0.0038 |
| Sludge | Spreading/Compost | 0.0000 | 0.0000 | 0.0000 |
| Spleen | Pet food | 0.0040 | 0.0044 | 0.0002 |
| Sweetbread | Human food | 0.0049 | 0.0044 | 0.0049 |

Table 3: Allocation factors for Average Milk-fed Calf reared in Stall

| COPRODUCT | Destination | Average/Milk-fed calf/Stall | | |
| --- | --- | --- | --- | --- |
|  |  | **Biophysical Allocation Factor** | **Mass Allocation Factor** | **Economic Allocation Factor** |
| Abomasum | Human food | 0.0040 | 0.0044 | 0.0002 |
| Aponevrosis (1%) | Human food | 0.0047 | 0.0044 | 0.0031 |
| Bile | PAP C3 | 0.0016 | 0.0044 | 0.0003 |
| Blood | C1-C2 for disposal | 0.0000 | 0.0000 | 0.0000 |
| Bones (11%) | Gelatin C3 | 0.0045 | 0.0044 | 0.0000 |
| Dead individuals | C1-C2 for disposal | 0.0000 | 0.0000 | 0.0000 |
| Fat (8%) | Fat and greaves C3 | 0.0120 | 0.0044 | 0.0003 |
| Fat from breasts and penis | Fat and greaves C3 | 0.0120 | 0.0044 | 0.0003 |
| Feet (without hooves) | Human food | 0.0040 | 0.0044 | 0.0000 |
| Floatation fat | C1-C2 for disposal | 0.0000 | 0.0000 | 0.0000 |
| Head | Human food | 0.0045 | 0.0044 | 0.0071 |
| Intestines | C1-C2 for disposal | 0.0000 | 0.0000 | 0.0000 |
| Kidney | Human food | 0.0038 | 0.0044 | 0.0033 |
| Manure | Spreading/Compost | 0.0000 | 0.0000 | 0.0000 |
| Meat | Human food | 0.0039 | 0.0044 | 0.0064 |
| Pluck | Human food | 0.0042 | 0.0044 | 0.0013 |
| Rumen and forestomach | Human food | 0.0040 | 0.0044 | 0.0002 |
| SPA C3 | PAP C3 | 0.0002 | 0.0044 | 0.0003 |
| Screening and sifting wastes | C1-C2 for disposal | 0.0000 | 0.0000 | 0.0000 |
| Skin | Skin tannery C3 | 0.0053 | 0.0044 | 0.0038 |
| Sludge | Spreading/Compost | 0.0000 | 0.0000 | 0.0000 |
| Spleen | Pet food | 0.0040 | 0.0044 | 0.0002 |
| Sweetbread | Human food | 0.0048 | 0.0044 | 0.0049 |

Table 4: Allocation factors for Average Rosé Calf reared in Grazing Large Area

| COPRODUCT | Destination | Average/rosé calf/grazing large area | | |
| --- | --- | --- | --- | --- |
|  |  | **Biophysical Allocation Factor** | **Mass Allocation Factor** | **Economic Allocation Factor** |
| Abomasum | Human food | 0.0046 | 0.0050 | 0.0002 |
| Aponevrosis (1%) | Human food | 0.0055 | 0.0050 | 0.0036 |
| Bile | PAP C3 | 0.0018 | 0.0050 | 0.0003 |
| Blood | C1-C2 for disposal | 0.0000 | 0.0000 | 0.0000 |
| Bones (11%) | Gelatin C3 | 0.0053 | 0.0050 | 0.0000 |
| Dead individuals | C1-C2 for disposal | 0.0000 | 0.0000 | 0.0000 |
| Fat (8%) | Fat and greaves C3 | 0.0124 | 0.0050 | 0.0003 |
| Fat from breasts and penis | Fat and greaves C3 | 0.0124 | 0.0050 | 0.0003 |
| Feet (without hooves) | Human food | 0.0046 | 0.0050 | 0.0000 |
| Floatation fat | C1-C2 for disposal | 0.0000 | 0.0000 | 0.0000 |
| Head | Human food | 0.0052 | 0.0050 | 0.0081 |
| Intestines | C1-C2 for disposal | 0.0000 | 0.0000 | 0.0000 |
| Kidney | Human food | 0.0045 | 0.0050 | 0.0038 |
| Manure | Spreading/Compost | 0.0000 | 0.0000 | 0.0000 |
| Meat | Human food | 0.0045 | 0.0050 | 0.0074 |
| Pluck | Human food | 0.0049 | 0.0050 | 0.0015 |
| Rumen and forestomach | Human food | 0.0046 | 0.0050 | 0.0002 |
| SPA C3 | PAP C3 | 0.0002 | 0.0050 | 0.0003 |
| Screening and sifting wastes | C1-C2 for disposal | 0.0000 | 0.0000 | 0.0000 |
| Skin | Skin tannery C3 | 0.0063 | 0.0050 | 0.0043 |
| Sludge | Spreading/Compost | 0.0000 | 0.0000 | 0.0000 |
| Spleen | Pet food | 0.0046 | 0.0050 | 0.0003 |
| Sweetbread | Human food | 0.0056 | 0.0050 | 0.0057 |

Table 5: Allocation factors for Average Rosé Calf reared in Pasture

| COPRODUCT | Destination | Average/Rosé calf/Pasture | | |
| --- | --- | --- | --- | --- |
|  |  | **Biophysical Allocation Factor** | **Mass Allocation Factor** | **Economic Allocation Factor** |
| Abomasum | Human food | 0.0046 | 0.0050 | 0.0002 |
| Aponevrosis (1%) | Human food | 0.0055 | 0.0050 | 0.0036 |
| Bile | PAP C3 | 0.0018 | 0.0050 | 0.0003 |
| Blood | C1-C2 for disposal | 0.0000 | 0.0000 | 0.0000 |
| Bones (11%) | Gelatin C3 | 0.0053 | 0.0050 | 0.0000 |
| Dead individuals | C1-C2 for disposal | 0.0000 | 0.0000 | 0.0000 |
| Fat (8%) | Fat and greaves C3 | 0.0129 | 0.0050 | 0.0003 |
| Fat from breasts and penis | Fat and greaves C3 | 0.0129 | 0.0050 | 0.0003 |
| Feet (without hooves) | Human food | 0.0046 | 0.0050 | 0.0000 |
| Floatation fat | C1-C2 for disposal | 0.0000 | 0.0000 | 0.0000 |
| Head | Human food | 0.0052 | 0.0050 | 0.0081 |
| Intestines | C1-C2 for disposal | 0.0000 | 0.0000 | 0.0000 |
| Kidney | Human food | 0.0044 | 0.0050 | 0.0038 |
| Manure | Spreading/Compost | 0.0000 | 0.0000 | 0.0000 |
| Meat | Human food | 0.0045 | 0.0050 | 0.0074 |
| Pluck | Human food | 0.0049 | 0.0050 | 0.0015 |
| Rumen and forestomach | Human food | 0.0046 | 0.0050 | 0.0002 |
| SPA C3 | PAP C3 | 0.0002 | 0.0050 | 0.0003 |
| Screening and sifting wastes | C1-C2 for disposal | 0.0000 | 0.0000 | 0.0000 |
| Skin | Skin tannery C3 | 0.0063 | 0.0050 | 0.0043 |
| Sludge | Spreading/Compost | 0.0000 | 0.0000 | 0.0000 |
| Spleen | Pet food | 0.0046 | 0.0050 | 0.0003 |
| Sweetbread | Human food | 0.0056 | 0.0050 | 0.0057 |

Table 6: Allocation factors for Average Rosé Calf reared in Stall

| COPRODUCT | Destination | Average/Rosé calf/Stall | | |
| --- | --- | --- | --- | --- |
|  |  | **Biophysical Allocation Factor** | **Mass Allocation Factor** | **Economic Allocation Factor** |
| Abomasum | Human food | 0.0046 | 0.0050 | 0.0002 |
| Aponevrosis (1%) | Human food | 0.0054 | 0.0050 | 0.0036 |
| Bile | PAP C3 | 0.0018 | 0.0050 | 0.0003 |
| Blood | C1-C2 for disposal | 0.0000 | 0.0000 | 0.0000 |
| Bones (11%) | Gelatin C3 | 0.0052 | 0.0050 | 0.0000 |
| Dead individuals | C1-C2 for disposal | 0.0000 | 0.0000 | 0.0000 |
| Fat (8%) | Fat and greaves C3 | 0.0133 | 0.0050 | 0.0003 |
| Fat from breasts and penis | Fat and greaves C3 | 0.0133 | 0.0050 | 0.0003 |
| Feet (without hooves) | Human food | 0.0046 | 0.0050 | 0.0000 |
| Floatation fat | C1-C2 for disposal | 0.0000 | 0.0000 | 0.0000 |
| Head | Human food | 0.0052 | 0.0050 | 0.0081 |
| Intestines | C1-C2 for disposal | 0.0000 | 0.0000 | 0.0000 |
| Kidney | Human food | 0.0044 | 0.0050 | 0.0038 |
| Manure | Spreading/Compost | 0.0000 | 0.0000 | 0.0000 |
| Meat | Human food | 0.0044 | 0.0050 | 0.0074 |
| Pluck | Human food | 0.0048 | 0.0050 | 0.0015 |
| Rumen and forestomach | Human food | 0.0046 | 0.0050 | 0.0002 |
| SPA C3 | PAP C3 | 0.0002 | 0.0050 | 0.0003 |
| Screening and sifting wastes | C1-C2 for disposal | 0.0000 | 0.0000 | 0.0000 |
| Skin | Skin tannery C3 | 0.0062 | 0.0050 | 0.0043 |
| Sludge | Spreading/Compost | 0.0000 | 0.0000 | 0.0000 |
| Spleen | Pet food | 0.0046 | 0.0050 | 0.0003 |
| Sweetbread | Human food | 0.0056 | 0.0050 | 0.0057 |

Table 7: Allocation factors for Aubrac Milk-fed Calf reared in Grazing Large Area

| COPRODUCT | Destination | Aubrac /milk-fed calf/grazing large area | | |
| --- | --- | --- | --- | --- |
|  |  | **Biophysical Allocation Factor** | **Mass Allocation Factor** | **Economic Allocation Factor** |
| Abomasum | Human food | 0.0039 | 0.0042 | 0.0002 |
| Aponevrosis (1%) | Human food | 0.0046 | 0.0042 | 0.0030 |
| Bile | PAP C3 | 0.0015 | 0.0042 | 0.0003 |
| Blood | C1-C2 for disposal | 0.0000 | 0.0000 | 0.0000 |
| Bones (11%) | Gelatin C3 | 0.0044 | 0.0042 | 0.0000 |
| Dead individuals | C1-C2 for disposal | 0.0000 | 0.0000 | 0.0000 |
| Fat (8%) | Fat and greaves C3 | 0.0108 | 0.0042 | 0.0003 |
| Fat from breasts and penis | Fat and greaves C3 | 0.0108 | 0.0042 | 0.0003 |
| Feet (without hooves) | Human food | 0.0039 | 0.0042 | 0.0000 |
| Floatation fat | C1-C2 for disposal | 0.0000 | 0.0000 | 0.0000 |
| Head | Human food | 0.0044 | 0.0042 | 0.0068 |
| Intestines | C1-C2 for disposal | 0.0000 | 0.0000 | 0.0000 |
| Kidney | Human food | 0.0037 | 0.0042 | 0.0032 |
| Manure | Spreading/Compost | 0.0000 | 0.0000 | 0.0000 |
| Meat | Human food | 0.0038 | 0.0042 | 0.0062 |
| Pluck | Human food | 0.0041 | 0.0042 | 0.0013 |
| Rumen and forestomach | Human food | 0.0039 | 0.0042 | 0.0002 |
| SPA C3 | PAP C3 | 0.0002 | 0.0042 | 0.0003 |
| Screening and sifting wastes | C1-C2 for disposal | 0.0000 | 0.0000 | 0.0000 |
| Skin | Skin tannery C3 | 0.0053 | 0.0042 | 0.0036 |
| Sludge | Spreading/Compost | 0.0000 | 0.0000 | 0.0000 |
| Spleen | Pet food | 0.0039 | 0.0042 | 0.0002 |
| Sweetbread | Human food | 0.0047 | 0.0042 | 0.0047 |

Table 8: Allocation factors for Aubrac Milk-fed Calf reared in Pasture

| COPRODUCT | Destination | Aubrac /milk-fed calf/PASTURE | | |
| --- | --- | --- | --- | --- |
|  |  | **Biophysical Allocation Factor** | **Mass Allocation Factor** | **Economic Allocation Factor** |
| Abomasum | Human food | 0.0038 | 0.0042 | 0.0002 |
| Aponevrosis (1%) | Human food | 0.0046 | 0.0042 | 0.0030 |
| Bile | PAP C3 | 0.0015 | 0.0042 | 0.0003 |
| Blood | C1-C2 for disposal | 0.0000 | 0.0000 | 0.0000 |
| Bones (11%) | Gelatin C3 | 0.0044 | 0.0042 | 0.0000 |
| Dead individuals | C1-C2 for disposal | 0.0000 | 0.0000 | 0.0000 |
| Fat (8%) | Fat and greaves C3 | 0.0112 | 0.0042 | 0.0003 |
| Fat from breasts and penis | Fat and greaves C3 | 0.0112 | 0.0042 | 0.0003 |
| Feet (without hooves) | Human food | 0.0038 | 0.0042 | 0.0000 |
| Floatation fat | C1-C2 for disposal | 0.0000 | 0.0000 | 0.0000 |
| Head | Human food | 0.0043 | 0.0042 | 0.0068 |
| Intestines | C1-C2 for disposal | 0.0000 | 0.0000 | 0.0000 |
| Kidney | Human food | 0.0037 | 0.0042 | 0.0032 |
| Manure | Spreading/Compost | 0.0000 | 0.0000 | 0.0000 |
| Meat | Human food | 0.0037 | 0.0042 | 0.0062 |
| Pluck | Human food | 0.0041 | 0.0042 | 0.0013 |
| Rumen and forestomach | Human food | 0.0038 | 0.0042 | 0.0002 |
| SPA C3 | PAP C3 | 0.0002 | 0.0042 | 0.0003 |
| Screening and sifting wastes | C1-C2 for disposal | 0.0000 | 0.0000 | 0.0000 |
| Skin | Skin tannery C3 | 0.0052 | 0.0042 | 0.0036 |
| Sludge | Spreading/Compost | 0.0000 | 0.0000 | 0.0000 |
| Spleen | Pet food | 0.0038 | 0.0042 | 0.0002 |
| Sweetbread | Human food | 0.0047 | 0.0042 | 0.0047 |

Table 9: Allocation factors for Aubrac Milk-fed Calf reared in Stall

| COPRODUCT | Destination | Aubrac /Milk-fed calf/Stall | | |
| --- | --- | --- | --- | --- |
|  |  | **Biophysical Allocation Factor** | **Mass Allocation Factor** | **Economic Allocation Factor** |
| Abomasum | Human food | 0.0038 | 0.0042 | 0.0002 |
| Aponevrosis (1%) | Human food | 0.0045 | 0.0042 | 0.0030 |
| Bile | PAP C3 | 0.0015 | 0.0042 | 0.0003 |
| Blood | C1-C2 for disposal | 0.0000 | 0.0000 | 0.0000 |
| Bones (11%) | Gelatin C3 | 0.0043 | 0.0042 | 0.0000 |
| Dead individuals | C1-C2 for disposal | 0.0000 | 0.0000 | 0.0000 |
| Fat (8%) | Fat and greaves C3 | 0.0116 | 0.0042 | 0.0003 |
| Fat from breasts and penis | Fat and greaves C3 | 0.0116 | 0.0042 | 0.0003 |
| Feet (without hooves) | Human food | 0.0038 | 0.0042 | 0.0000 |
| Floatation fat | C1-C2 for disposal | 0.0000 | 0.0000 | 0.0000 |
| Head | Human food | 0.0043 | 0.0042 | 0.0068 |
| Intestines | C1-C2 for disposal | 0.0000 | 0.0000 | 0.0000 |
| Kidney | Human food | 0.0037 | 0.0042 | 0.0032 |
| Manure | Spreading/Compost | 0.0000 | 0.0000 | 0.0000 |
| Meat | Human food | 0.0037 | 0.0042 | 0.0062 |
| Pluck | Human food | 0.0040 | 0.0042 | 0.0013 |
| Rumen and forestomach | Human food | 0.0038 | 0.0042 | 0.0002 |
| SPA C3 | PAP C3 | 0.0002 | 0.0042 | 0.0003 |
| Screening and sifting wastes | C1-C2 for disposal | 0.0000 | 0.0000 | 0.0000 |
| Skin | Skin tannery C3 | 0.0051 | 0.0042 | 0.0036 |
| Sludge | Spreading/Compost | 0.0000 | 0.0000 | 0.0000 |
| Spleen | Pet food | 0.0038 | 0.0042 | 0.0002 |
| Sweetbread | Human food | 0.0046 | 0.0042 | 0.0047 |

Table 10: Allocation factors for Aubrac Rosé Calf reared in Grazing Large Area

| COPRODUCT | Destination | Aubrac /rosé calf/grazing large area | | |
| --- | --- | --- | --- | --- |
|  |  | **Biophysical Allocation Factor** | **Mass Allocation Factor** | **Economic Allocation Factor** |
| Abomasum | Human food | 0.0044 | 0.0048 | 0.0002 |
| Aponevrosis (1%) | Human food | 0.0053 | 0.0048 | 0.0034 |
| Bile | PAP C3 | 0.0018 | 0.0048 | 0.0003 |
| Blood | C1-C2 for disposal | 0.0000 | 0.0000 | 0.0000 |
| Bones (11%) | Gelatin C3 | 0.0051 | 0.0048 | 0.0000 |
| Dead individuals | C1-C2 for disposal | 0.0000 | 0.0000 | 0.0000 |
| Fat (8%) | Fat and greaves C3 | 0.0120 | 0.0048 | 0.0003 |
| Fat from breasts and penis | Fat and greaves C3 | 0.0120 | 0.0048 | 0.0003 |
| Feet (without hooves) | Human food | 0.0044 | 0.0048 | 0.0000 |
| Floatation fat | C1-C2 for disposal | 0.0000 | 0.0000 | 0.0000 |
| Head | Human food | 0.0050 | 0.0048 | 0.0078 |
| Intestines | C1-C2 for disposal | 0.0000 | 0.0000 | 0.0000 |
| Kidney | Human food | 0.0043 | 0.0048 | 0.0036 |
| Manure | Spreading/Compost | 0.0000 | 0.0000 | 0.0000 |
| Meat | Human food | 0.0043 | 0.0048 | 0.0071 |
| Pluck | Human food | 0.0047 | 0.0048 | 0.0015 |
| Rumen and forestomach | Human food | 0.0044 | 0.0048 | 0.0002 |
| SPA C3 | PAP C3 | 0.0002 | 0.0048 | 0.0003 |
| Screening and sifting wastes | C1-C2 for disposal | 0.0000 | 0.0000 | 0.0000 |
| Skin | Skin tannery C3 | 0.0061 | 0.0048 | 0.0041 |
| Sludge | Spreading/Compost | 0.0000 | 0.0000 | 0.0000 |
| Spleen | Pet food | 0.0044 | 0.0048 | 0.0003 |
| Sweetbread | Human food | 0.0054 | 0.0048 | 0.0054 |

Table 11: Allocation factors for Aubrac Rosé Calf reared in Pasture

| COPRODUCT | Destination | Aubrac /Rosé calf/Pasture | | |
| --- | --- | --- | --- | --- |
|  |  | **Biophysical Allocation Factor** | **Mass Allocation Factor** | **Economic Allocation Factor** |
| Abomasum | Human food | 0.0044 | 0.0048 | 0.0002 |
| Aponevrosis (1%) | Human food | 0.0053 | 0.0048 | 0.0034 |
| Bile | PAP C3 | 0.0018 | 0.0048 | 0.0003 |
| Blood | C1-C2 for disposal | 0.0000 | 0.0000 | 0.0000 |
| Bones (11%) | Gelatin C3 | 0.0050 | 0.0048 | 0.0000 |
| Dead individuals | C1-C2 for disposal | 0.0000 | 0.0000 | 0.0000 |
| Fat (8%) | Fat and greaves C3 | 0.0125 | 0.0048 | 0.0003 |
| Fat from breasts and penis | Fat and greaves C3 | 0.0125 | 0.0048 | 0.0003 |
| Feet (without hooves) | Human food | 0.0044 | 0.0048 | 0.0000 |
| Floatation fat | C1-C2 for disposal | 0.0000 | 0.0000 | 0.0000 |
| Head | Human food | 0.0050 | 0.0048 | 0.0078 |
| Intestines | C1-C2 for disposal | 0.0000 | 0.0000 | 0.0000 |
| Kidney | Human food | 0.0043 | 0.0048 | 0.0036 |
| Manure | Spreading/Compost | 0.0000 | 0.0000 | 0.0000 |
| Meat | Human food | 0.0043 | 0.0048 | 0.0071 |
| Pluck | Human food | 0.0047 | 0.0048 | 0.0015 |
| Rumen and forestomach | Human food | 0.0044 | 0.0048 | 0.0002 |
| SPA C3 | PAP C3 | 0.0002 | 0.0048 | 0.0003 |
| Screening and sifting wastes | C1-C2 for disposal | 0.0000 | 0.0000 | 0.0000 |
| Skin | Skin tannery C3 | 0.0060 | 0.0048 | 0.0041 |
| Sludge | Spreading/Compost | 0.0000 | 0.0000 | 0.0000 |
| Spleen | Pet food | 0.0044 | 0.0048 | 0.0003 |
| Sweetbread | Human food | 0.0054 | 0.0048 | 0.0054 |

Table 12: Allocation factors for Aubrac Rosé Calf reared in Stall

| COPRODUCT | Destination | Aubrac /Rosé calf/Stall | | |
| --- | --- | --- | --- | --- |
|  |  | **Biophysical Allocation Factor** | **Mass Allocation Factor** | **Economic Allocation Factor** |
| Abomasum | Human food | 0.0044 | 0.0048 | 0.0002 |
| Aponevrosis (1%) | Human food | 0.0052 | 0.0048 | 0.0034 |
| Bile | PAP C3 | 0.0018 | 0.0048 | 0.0003 |
| Blood | C1-C2 for disposal | 0.0000 | 0.0000 | 0.0000 |
| Bones (11%) | Gelatin C3 | 0.0050 | 0.0048 | 0.0000 |
| Dead individuals | C1-C2 for disposal | 0.0000 | 0.0000 | 0.0000 |
| Fat (8%) | Fat and greaves C3 | 0.0129 | 0.0048 | 0.0003 |
| Fat from breasts and penis | Fat and greaves C3 | 0.0129 | 0.0048 | 0.0003 |
| Feet (without hooves) | Human food | 0.0044 | 0.0048 | 0.0000 |
| Floatation fat | C1-C2 for disposal | 0.0000 | 0.0000 | 0.0000 |
| Head | Human food | 0.0050 | 0.0048 | 0.0078 |
| Intestines | C1-C2 for disposal | 0.0000 | 0.0000 | 0.0000 |
| Kidney | Human food | 0.0042 | 0.0048 | 0.0036 |
| Manure | Spreading/Compost | 0.0000 | 0.0000 | 0.0000 |
| Meat | Human food | 0.0043 | 0.0048 | 0.0071 |
| Pluck | Human food | 0.0046 | 0.0048 | 0.0015 |
| Rumen and forestomach | Human food | 0.0044 | 0.0048 | 0.0002 |
| SPA C3 | PAP C3 | 0.0002 | 0.0048 | 0.0003 |
| Screening and sifting wastes | C1-C2 for disposal | 0.0000 | 0.0000 | 0.0000 |
| Skin | Skin tannery C3 | 0.0059 | 0.0048 | 0.0041 |
| Sludge | Spreading/Compost | 0.0000 | 0.0000 | 0.0000 |
| Spleen | Pet food | 0.0044 | 0.0048 | 0.0003 |
| Sweetbread | Human food | 0.0053 | 0.0048 | 0.0054 |

Table 13: Allocation factors for Blonde d’Aquitaine Milk-fed Calf reared in Grazing Large Area

| COPRODUCT | Destination | Blonde d’Aquitaine /milk-fed calf/grazing large area | | |
| --- | --- | --- | --- | --- |
|  |  | **Biophysical Allocation Factor** | **Mass Allocation Factor** | **Economic Allocation Factor** |
| Abomasum | Human food | 0.0035 | 0.0039 | 0.0002 |
| Aponevrosis (1%) | Human food | 0.0042 | 0.0039 | 0.0028 |
| Bile | PAP C3 | 0.0014 | 0.0039 | 0.0002 |
| Blood | C1-C2 for disposal | 0.0000 | 0.0000 | 0.0000 |
| Bones (11%) | Gelatin C3 | 0.0041 | 0.0039 | 0.0000 |
| Dead individuals | C1-C2 for disposal | 0.0000 | 0.0000 | 0.0000 |
| Fat (8%) | Fat and greaves C3 | 0.0101 | 0.0039 | 0.0003 |
| Fat from breasts and penis | Fat and greaves C3 | 0.0101 | 0.0039 | 0.0003 |
| Feet (without hooves) | Human food | 0.0035 | 0.0039 | 0.0000 |
| Floatation fat | C1-C2 for disposal | 0.0000 | 0.0000 | 0.0000 |
| Head | Human food | 0.0040 | 0.0039 | 0.0063 |
| Intestines | C1-C2 for disposal | 0.0000 | 0.0000 | 0.0000 |
| Kidney | Human food | 0.0034 | 0.0039 | 0.0029 |
| Manure | Spreading/Compost | 0.0000 | 0.0000 | 0.0000 |
| Meat | Human food | 0.0035 | 0.0039 | 0.0057 |
| Pluck | Human food | 0.0038 | 0.0039 | 0.0012 |
| Rumen and forestomach | Human food | 0.0035 | 0.0039 | 0.0002 |
| SPA C3 | PAP C3 | 0.0002 | 0.0039 | 0.0002 |
| Screening and sifting wastes | C1-C2 for disposal | 0.0000 | 0.0000 | 0.0000 |
| Skin | Skin tannery C3 | 0.0048 | 0.0039 | 0.0033 |
| Sludge | Spreading/Compost | 0.0000 | 0.0000 | 0.0000 |
| Spleen | Pet food | 0.0035 | 0.0039 | 0.0002 |
| Sweetbread | Human food | 0.0043 | 0.0039 | 0.0044 |

Table 14: Allocation factors for Blonde d’Aquitaine Milk-fed Calf reared in Pasture

| COPRODUCT | Destination | Blonde d’Aquitaine /milk-fed calf/PASTURE | | |
| --- | --- | --- | --- | --- |
|  |  | **Biophysical Allocation Factor** | **Mass Allocation Factor** | **Economic Allocation Factor** |
| Abomasum | Human food | 0.0035 | 0.0039 | 0.0002 |
| Aponevrosis (1%) | Human food | 0.0042 | 0.0039 | 0.0028 |
| Bile | PAP C3 | 0.0014 | 0.0039 | 0.0002 |
| Blood | C1-C2 for disposal | 0.0000 | 0.0000 | 0.0000 |
| Bones (11%) | Gelatin C3 | 0.0040 | 0.0039 | 0.0000 |
| Dead individuals | C1-C2 for disposal | 0.0000 | 0.0000 | 0.0000 |
| Fat (8%) | Fat and greaves C3 | 0.0105 | 0.0039 | 0.0003 |
| Fat from breasts and penis | Fat and greaves C3 | 0.0105 | 0.0039 | 0.0003 |
| Feet (without hooves) | Human food | 0.0035 | 0.0039 | 0.0000 |
| Floatation fat | C1-C2 for disposal | 0.0000 | 0.0000 | 0.0000 |
| Head | Human food | 0.0040 | 0.0039 | 0.0063 |
| Intestines | C1-C2 for disposal | 0.0000 | 0.0000 | 0.0000 |
| Kidney | Human food | 0.0034 | 0.0039 | 0.0029 |
| Manure | Spreading/Compost | 0.0000 | 0.0000 | 0.0000 |
| Meat | Human food | 0.0034 | 0.0039 | 0.0057 |
| Pluck | Human food | 0.0037 | 0.0039 | 0.0012 |
| Rumen and forestomach | Human food | 0.0035 | 0.0039 | 0.0002 |
| SPA C3 | PAP C3 | 0.0002 | 0.0039 | 0.0002 |
| Screening and sifting wastes | C1-C2 for disposal | 0.0000 | 0.0000 | 0.0000 |
| Skin | Skin tannery C3 | 0.0048 | 0.0039 | 0.0033 |
| Sludge | Spreading/Compost | 0.0000 | 0.0000 | 0.0000 |
| Spleen | Pet food | 0.0035 | 0.0039 | 0.0002 |
| Sweetbread | Human food | 0.0043 | 0.0039 | 0.0044 |

Table 15: Allocation factors for Blonde d’Aquitaine Milk-fed Calf reared in Stall

| COPRODUCT | Destination | Blonde d’Aquitaine /Milk-fed calf/Stall | | |
| --- | --- | --- | --- | --- |
|  |  | **Biophysical Allocation Factor** | **Mass Allocation Factor** | **Economic Allocation Factor** |
| Abomasum | Human food | 0.0035 | 0.0039 | 0.0002 |
| Aponevrosis (1%) | Human food | 0.0042 | 0.0039 | 0.0028 |
| Bile | PAP C3 | 0.0014 | 0.0039 | 0.0002 |
| Blood | C1-C2 for disposal | 0.0000 | 0.0000 | 0.0000 |
| Bones (11%) | Gelatin C3 | 0.0039 | 0.0039 | 0.0000 |
| Dead individuals | C1-C2 for disposal | 0.0000 | 0.0000 | 0.0000 |
| Fat (8%) | Fat and greaves C3 | 0.0109 | 0.0039 | 0.0003 |
| Fat from breasts and penis | Fat and greaves C3 | 0.0109 | 0.0039 | 0.0003 |
| Feet (without hooves) | Human food | 0.0035 | 0.0039 | 0.0000 |
| Floatation fat | C1-C2 for disposal | 0.0000 | 0.0000 | 0.0000 |
| Head | Human food | 0.0040 | 0.0039 | 0.0063 |
| Intestines | C1-C2 for disposal | 0.0000 | 0.0000 | 0.0000 |
| Kidney | Human food | 0.0033 | 0.0039 | 0.0029 |
| Manure | Spreading/Compost | 0.0000 | 0.0000 | 0.0000 |
| Meat | Human food | 0.0034 | 0.0039 | 0.0057 |
| Pluck | Human food | 0.0037 | 0.0039 | 0.0012 |
| Rumen and forestomach | Human food | 0.0035 | 0.0039 | 0.0002 |
| SPA C3 | PAP C3 | 0.0001 | 0.0039 | 0.0002 |
| Screening and sifting wastes | C1-C2 for disposal | 0.0000 | 0.0000 | 0.0000 |
| Skin | Skin tannery C3 | 0.0047 | 0.0039 | 0.0033 |
| Sludge | Spreading/Compost | 0.0000 | 0.0000 | 0.0000 |
| Spleen | Pet food | 0.0035 | 0.0039 | 0.0002 |
| Sweetbread | Human food | 0.0043 | 0.0039 | 0.0044 |

Table 16: Allocation factors for Blonde d’Aquitaine Rosé Calf reared in Grazing Large Area

| COPRODUCT | Destination | Blonde d’Aquitaine /rosé calf/grazing large area | | |
| --- | --- | --- | --- | --- |
|  |  | **Biophysical Allocation Factor** | **Mass Allocation Factor** | **Economic Allocation Factor** |
| Abomasum | Human food | 0.0041 | 0.0045 | 0.0002 |
| Aponevrosis (1%) | Human food | 0.0049 | 0.0045 | 0.0032 |
| Bile | PAP C3 | 0.0016 | 0.0045 | 0.0003 |
| Blood | C1-C2 for disposal | 0.0000 | 0.0000 | 0.0000 |
| Bones (11%) | Gelatin C3 | 0.0047 | 0.0045 | 0.0000 |
| Dead individuals | C1-C2 for disposal | 0.0000 | 0.0000 | 0.0000 |
| Fat (8%) | Fat and greaves C3 | 0.0113 | 0.0045 | 0.0003 |
| Fat from breasts and penis | Fat and greaves C3 | 0.0113 | 0.0045 | 0.0003 |
| Feet (without hooves) | Human food | 0.0041 | 0.0045 | 0.0000 |
| Floatation fat | C1-C2 for disposal | 0.0000 | 0.0000 | 0.0000 |
| Head | Human food | 0.0046 | 0.0045 | 0.0072 |
| Intestines | C1-C2 for disposal | 0.0000 | 0.0000 | 0.0000 |
| Kidney | Human food | 0.0040 | 0.0045 | 0.0033 |
| Manure | Spreading/Compost | 0.0000 | 0.0000 | 0.0000 |
| Meat | Human food | 0.0040 | 0.0045 | 0.0065 |
| Pluck | Human food | 0.0043 | 0.0045 | 0.0013 |
| Rumen and forestomach | Human food | 0.0041 | 0.0045 | 0.0002 |
| SPA C3 | PAP C3 | 0.0002 | 0.0045 | 0.0003 |
| Screening and sifting wastes | C1-C2 for disposal | 0.0000 | 0.0000 | 0.0000 |
| Skin | Skin tannery C3 | 0.0056 | 0.0045 | 0.0038 |
| Sludge | Spreading/Compost | 0.0000 | 0.0000 | 0.0000 |
| Spleen | Pet food | 0.0041 | 0.0045 | 0.0002 |
| Sweetbread | Human food | 0.0050 | 0.0045 | 0.0050 |

Table 17: Allocation factors for Blonde d’Aquitaine Rosé Calf reared in Pasture

| COPRODUCT | Destination | Blonde d’Aquitaine /Rosé calf/Pasture | | |
| --- | --- | --- | --- | --- |
|  |  | **Biophysical Allocation Factor** | **Mass Allocation Factor** | **Economic Allocation Factor** |
| Abomasum | Human food | 0.0041 | 0.0045 | 0.0002 |
| Aponevrosis (1%) | Human food | 0.0048 | 0.0045 | 0.0032 |
| Bile | PAP C3 | 0.0016 | 0.0045 | 0.0003 |
| Blood | C1-C2 for disposal | 0.0000 | 0.0000 | 0.0000 |
| Bones (11%) | Gelatin C3 | 0.0046 | 0.0045 | 0.0000 |
| Dead individuals | C1-C2 for disposal | 0.0000 | 0.0000 | 0.0000 |
| Fat (8%) | Fat and greaves C3 | 0.0117 | 0.0045 | 0.0003 |
| Fat from breasts and penis | Fat and greaves C3 | 0.0117 | 0.0045 | 0.0003 |
| Feet (without hooves) | Human food | 0.0041 | 0.0045 | 0.0000 |
| Floatation fat | C1-C2 for disposal | 0.0000 | 0.0000 | 0.0000 |
| Head | Human food | 0.0046 | 0.0045 | 0.0072 |
| Intestines | C1-C2 for disposal | 0.0000 | 0.0000 | 0.0000 |
| Kidney | Human food | 0.0039 | 0.0045 | 0.0033 |
| Manure | Spreading/Compost | 0.0000 | 0.0000 | 0.0000 |
| Meat | Human food | 0.0040 | 0.0045 | 0.0065 |
| Pluck | Human food | 0.0043 | 0.0045 | 0.0013 |
| Rumen and forestomach | Human food | 0.0041 | 0.0045 | 0.0002 |
| SPA C3 | PAP C3 | 0.0002 | 0.0045 | 0.0003 |
| Screening and sifting wastes | C1-C2 for disposal | 0.0000 | 0.0000 | 0.0000 |
| Skin | Skin tannery C3 | 0.0055 | 0.0045 | 0.0038 |
| Sludge | Spreading/Compost | 0.0000 | 0.0000 | 0.0000 |
| Spleen | Pet food | 0.0041 | 0.0045 | 0.0002 |
| Sweetbread | Human food | 0.0049 | 0.0045 | 0.0050 |

Table 18: Allocation factors for Blonde d’Aquitaine Rosé Calf reared in Stall

| COPRODUCT | Destination | Blonde d’Aquitaine /Rosé calf/Stall | | |
| --- | --- | --- | --- | --- |
|  |  | **Biophysical Allocation Factor** | **Mass Allocation Factor** | **Economic Allocation Factor** |
| Abomasum | Human food | 0.0040 | 0.0045 | 0.0002 |
| Aponevrosis (1%) | Human food | 0.0048 | 0.0045 | 0.0032 |
| Bile | PAP C3 | 0.0016 | 0.0045 | 0.0003 |
| Blood | C1-C2 for disposal | 0.0000 | 0.0000 | 0.0000 |
| Bones (11%) | Gelatin C3 | 0.0046 | 0.0045 | 0.0000 |
| Dead individuals | C1-C2 for disposal | 0.0000 | 0.0000 | 0.0000 |
| Fat (8%) | Fat and greaves C3 | 0.0121 | 0.0045 | 0.0003 |
| Fat from breasts and penis | Fat and greaves C3 | 0.0121 | 0.0045 | 0.0003 |
| Feet (without hooves) | Human food | 0.0040 | 0.0045 | 0.0000 |
| Floatation fat | C1-C2 for disposal | 0.0000 | 0.0000 | 0.0000 |
| Head | Human food | 0.0046 | 0.0045 | 0.0072 |
| Intestines | C1-C2 for disposal | 0.0000 | 0.0000 | 0.0000 |
| Kidney | Human food | 0.0039 | 0.0045 | 0.0033 |
| Manure | Spreading/Compost | 0.0000 | 0.0000 | 0.0000 |
| Meat | Human food | 0.0039 | 0.0045 | 0.0065 |
| Pluck | Human food | 0.0043 | 0.0045 | 0.0013 |
| Rumen and forestomach | Human food | 0.0040 | 0.0045 | 0.0002 |
| SPA C3 | PAP C3 | 0.0002 | 0.0045 | 0.0003 |
| Screening and sifting wastes | C1-C2 for disposal | 0.0000 | 0.0000 | 0.0000 |
| Skin | Skin tannery C3 | 0.0054 | 0.0045 | 0.0038 |
| Sludge | Spreading/Compost | 0.0000 | 0.0000 | 0.0000 |
| Spleen | Pet food | 0.0040 | 0.0045 | 0.0002 |
| Sweetbread | Human food | 0.0049 | 0.0045 | 0.0050 |

Table 19: Allocation factors for Charolais Milk-fed Calf reared in Grazing Large Area

| COPRODUCT | Destination | Charolais /milk-fed calf/grazing large area | | |
| --- | --- | --- | --- | --- |
|  |  | **Biophysical Allocation Factor** | **Mass Allocation Factor** | **Economic Allocation Factor** |
| Abomasum | Human food | 0.0040 | 0.0044 | 0.0002 |
| Aponevrosis (1%) | Human food | 0.0048 | 0.0044 | 0.0031 |
| Bile | PAP C3 | 0.0016 | 0.0044 | 0.0003 |
| Blood | C1-C2 for disposal | 0.0000 | 0.0000 | 0.0000 |
| Bones (11%) | Gelatin C3 | 0.0046 | 0.0044 | 0.0000 |
| Dead individuals | C1-C2 for disposal | 0.0000 | 0.0000 | 0.0000 |
| Fat (8%) | Fat and greaves C3 | 0.0111 | 0.0044 | 0.0003 |
| Fat from breasts and penis | Fat and greaves C3 | 0.0111 | 0.0044 | 0.0003 |
| Feet (without hooves) | Human food | 0.0040 | 0.0044 | 0.0000 |
| Floatation fat | C1-C2 for disposal | 0.0000 | 0.0000 | 0.0000 |
| Head | Human food | 0.0045 | 0.0044 | 0.0071 |
| Intestines | C1-C2 for disposal | 0.0000 | 0.0000 | 0.0000 |
| Kidney | Human food | 0.0039 | 0.0044 | 0.0033 |
| Manure | Spreading/Compost | 0.0000 | 0.0000 | 0.0000 |
| Meat | Human food | 0.0039 | 0.0044 | 0.0064 |
| Pluck | Human food | 0.0043 | 0.0044 | 0.0013 |
| Rumen and forestomach | Human food | 0.0040 | 0.0044 | 0.0002 |
| SPA C3 | PAP C3 | 0.0002 | 0.0044 | 0.0003 |
| Screening and sifting wastes | C1-C2 for disposal | 0.0000 | 0.0000 | 0.0000 |
| Skin | Skin tannery C3 | 0.0055 | 0.0044 | 0.0038 |
| Sludge | Spreading/Compost | 0.0000 | 0.0000 | 0.0000 |
| Spleen | Pet food | 0.0040 | 0.0044 | 0.0002 |
| Sweetbread | Human food | 0.0049 | 0.0044 | 0.0049 |

Table 20: Allocation factors for Charolais Milk-fed Calf reared in Pasture

| COPRODUCT | Destination | Charolais /milk-fed calf/PASTURE | | |
| --- | --- | --- | --- | --- |
|  |  | **Biophysical Allocation Factor** | **Mass Allocation Factor** | **Economic Allocation Factor** |
| Abomasum | Human food | 0.0040 | 0.0044 | 0.0002 |
| Aponevrosis (1%) | Human food | 0.0048 | 0.0044 | 0.0031 |
| Bile | PAP C3 | 0.0016 | 0.0044 | 0.0003 |
| Blood | C1-C2 for disposal | 0.0000 | 0.0000 | 0.0000 |
| Bones (11%) | Gelatin C3 | 0.0045 | 0.0044 | 0.0000 |
| Dead individuals | C1-C2 for disposal | 0.0000 | 0.0000 | 0.0000 |
| Fat (8%) | Fat and greaves C3 | 0.0116 | 0.0044 | 0.0003 |
| Fat from breasts and penis | Fat and greaves C3 | 0.0116 | 0.0044 | 0.0003 |
| Feet (without hooves) | Human food | 0.0040 | 0.0044 | 0.0000 |
| Floatation fat | C1-C2 for disposal | 0.0000 | 0.0000 | 0.0000 |
| Head | Human food | 0.0045 | 0.0044 | 0.0071 |
| Intestines | C1-C2 for disposal | 0.0000 | 0.0000 | 0.0000 |
| Kidney | Human food | 0.0039 | 0.0044 | 0.0033 |
| Manure | Spreading/Compost | 0.0000 | 0.0000 | 0.0000 |
| Meat | Human food | 0.0039 | 0.0044 | 0.0064 |
| Pluck | Human food | 0.0042 | 0.0044 | 0.0013 |
| Rumen and forestomach | Human food | 0.0040 | 0.0044 | 0.0002 |
| SPA C3 | PAP C3 | 0.0002 | 0.0044 | 0.0003 |
| Screening and sifting wastes | C1-C2 for disposal | 0.0000 | 0.0000 | 0.0000 |
| Skin | Skin tannery C3 | 0.0054 | 0.0044 | 0.0038 |
| Sludge | Spreading/Compost | 0.0000 | 0.0000 | 0.0000 |
| Spleen | Pet food | 0.0040 | 0.0044 | 0.0002 |
| Sweetbread | Human food | 0.0049 | 0.0044 | 0.0049 |

Table 21: Allocation factors for Charolais Milk-fed Calf reared in Stall

| COPRODUCT | Destination | Charolais /Milk-fed calf/Stall | | |
| --- | --- | --- | --- | --- |
|  |  | **Biophysical Allocation Factor** | **Mass Allocation Factor** | **Economic Allocation Factor** |
| Abomasum | Human food | 0.0040 | 0.0044 | 0.0002 |
| Aponevrosis (1%) | Human food | 0.0047 | 0.0044 | 0.0031 |
| Bile | PAP C3 | 0.0016 | 0.0044 | 0.0003 |
| Blood | C1-C2 for disposal | 0.0000 | 0.0000 | 0.0000 |
| Bones (11%) | Gelatin C3 | 0.0045 | 0.0044 | 0.0000 |
| Dead individuals | C1-C2 for disposal | 0.0000 | 0.0000 | 0.0000 |
| Fat (8%) | Fat and greaves C3 | 0.0120 | 0.0044 | 0.0003 |
| Fat from breasts and penis | Fat and greaves C3 | 0.0120 | 0.0044 | 0.0003 |
| Feet (without hooves) | Human food | 0.0040 | 0.0044 | 0.0000 |
| Floatation fat | C1-C2 for disposal | 0.0000 | 0.0000 | 0.0000 |
| Head | Human food | 0.0045 | 0.0044 | 0.0071 |
| Intestines | C1-C2 for disposal | 0.0000 | 0.0000 | 0.0000 |
| Kidney | Human food | 0.0038 | 0.0044 | 0.0033 |
| Manure | Spreading/Compost | 0.0000 | 0.0000 | 0.0000 |
| Meat | Human food | 0.0039 | 0.0044 | 0.0064 |
| Pluck | Human food | 0.0042 | 0.0044 | 0.0013 |
| Rumen and forestomach | Human food | 0.0040 | 0.0044 | 0.0002 |
| SPA C3 | PAP C3 | 0.0002 | 0.0044 | 0.0003 |
| Screening and sifting wastes | C1-C2 for disposal | 0.0000 | 0.0000 | 0.0000 |
| Skin | Skin tannery C3 | 0.0053 | 0.0044 | 0.0038 |
| Sludge | Spreading/Compost | 0.0000 | 0.0000 | 0.0000 |
| Spleen | Pet food | 0.0040 | 0.0044 | 0.0002 |
| Sweetbread | Human food | 0.0048 | 0.0044 | 0.0049 |

Table 22: Allocation factors for Charolais Rosé Calf reared in Grazing Large Area

| COPRODUCT | Destination | Charolais /rosé calf/grazing large area | | |
| --- | --- | --- | --- | --- |
|  |  | **Biophysical Allocation Factor** | **Mass Allocation Factor** | **Economic Allocation Factor** |
| Abomasum | Human food | 0.0046 | 0.0050 | 0.0002 |
| Aponevrosis (1%) | Human food | 0.0055 | 0.0050 | 0.0036 |
| Bile | PAP C3 | 0.0018 | 0.0050 | 0.0003 |
| Blood | C1-C2 for disposal | 0.0000 | 0.0000 | 0.0000 |
| Bones (11%) | Gelatin C3 | 0.0053 | 0.0050 | 0.0000 |
| Dead individuals | C1-C2 for disposal | 0.0000 | 0.0000 | 0.0000 |
| Fat (8%) | Fat and greaves C3 | 0.0124 | 0.0050 | 0.0003 |
| Fat from breasts and penis | Fat and greaves C3 | 0.0124 | 0.0050 | 0.0003 |
| Feet (without hooves) | Human food | 0.0046 | 0.0050 | 0.0000 |
| Floatation fat | C1-C2 for disposal | 0.0000 | 0.0000 | 0.0000 |
| Head | Human food | 0.0052 | 0.0050 | 0.0081 |
| Intestines | C1-C2 for disposal | 0.0000 | 0.0000 | 0.0000 |
| Kidney | Human food | 0.0045 | 0.0050 | 0.0038 |
| Manure | Spreading/Compost | 0.0000 | 0.0000 | 0.0000 |
| Meat | Human food | 0.0045 | 0.0050 | 0.0074 |
| Pluck | Human food | 0.0049 | 0.0050 | 0.0015 |
| Rumen and forestomach | Human food | 0.0046 | 0.0050 | 0.0002 |
| SPA C3 | PAP C3 | 0.0002 | 0.0050 | 0.0003 |
| Screening and sifting wastes | C1-C2 for disposal | 0.0000 | 0.0000 | 0.0000 |
| Skin | Skin tannery C3 | 0.0063 | 0.0050 | 0.0043 |
| Sludge | Spreading/Compost | 0.0000 | 0.0000 | 0.0000 |
| Spleen | Pet food | 0.0046 | 0.0050 | 0.0003 |
| Sweetbread | Human food | 0.0056 | 0.0050 | 0.0057 |

Table 23: Allocation factors for Charolais Rosé Calf reared in Pasture

| COPRODUCT | Destination | Charolais /Rosé calf/Pasture | | |
| --- | --- | --- | --- | --- |
|  |  | **Biophysical Allocation Factor** | **Mass Allocation Factor** | **Economic Allocation Factor** |
| Abomasum | Human food | 0.0046 | 0.0050 | 0.0002 |
| Aponevrosis (1%) | Human food | 0.0055 | 0.0050 | 0.0036 |
| Bile | PAP C3 | 0.0018 | 0.0050 | 0.0003 |
| Blood | C1-C2 for disposal | 0.0000 | 0.0000 | 0.0000 |
| Bones (11%) | Gelatin C3 | 0.0053 | 0.0050 | 0.0000 |
| Dead individuals | C1-C2 for disposal | 0.0000 | 0.0000 | 0.0000 |
| Fat (8%) | Fat and greaves C3 | 0.0129 | 0.0050 | 0.0003 |
| Fat from breasts and penis | Fat and greaves C3 | 0.0129 | 0.0050 | 0.0003 |
| Feet (without hooves) | Human food | 0.0046 | 0.0050 | 0.0000 |
| Floatation fat | C1-C2 for disposal | 0.0000 | 0.0000 | 0.0000 |
| Head | Human food | 0.0052 | 0.0050 | 0.0081 |
| Intestines | C1-C2 for disposal | 0.0000 | 0.0000 | 0.0000 |
| Kidney | Human food | 0.0044 | 0.0050 | 0.0038 |
| Manure | Spreading/Compost | 0.0000 | 0.0000 | 0.0000 |
| Meat | Human food | 0.0045 | 0.0050 | 0.0074 |
| Pluck | Human food | 0.0049 | 0.0050 | 0.0015 |
| Rumen and forestomach | Human food | 0.0046 | 0.0050 | 0.0002 |
| SPA C3 | PAP C3 | 0.0002 | 0.0050 | 0.0003 |
| Screening and sifting wastes | C1-C2 for disposal | 0.0000 | 0.0000 | 0.0000 |
| Skin | Skin tannery C3 | 0.0063 | 0.0050 | 0.0043 |
| Sludge | Spreading/Compost | 0.0000 | 0.0000 | 0.0000 |
| Spleen | Pet food | 0.0046 | 0.0050 | 0.0003 |
| Sweetbread | Human food | 0.0056 | 0.0050 | 0.0057 |

Table 24: Allocation factors for Charolais Rosé Calf reared in Stall

| COPRODUCT | Destination | Charolais /Rosé calf/Stall | | |
| --- | --- | --- | --- | --- |
|  |  | **Biophysical Allocation Factor** | **Mass Allocation Factor** | **Economic Allocation Factor** |
| Abomasum | Human food | 0.0046 | 0.0050 | 0.0002 |
| Aponevrosis (1%) | Human food | 0.0054 | 0.0050 | 0.0036 |
| Bile | PAP C3 | 0.0018 | 0.0050 | 0.0003 |
| Blood | C1-C2 for disposal | 0.0000 | 0.0000 | 0.0000 |
| Bones (11%) | Gelatin C3 | 0.0052 | 0.0050 | 0.0000 |
| Dead individuals | C1-C2 for disposal | 0.0000 | 0.0000 | 0.0000 |
| Fat (8%) | Fat and greaves C3 | 0.0133 | 0.0050 | 0.0003 |
| Fat from breasts and penis | Fat and greaves C3 | 0.0133 | 0.0050 | 0.0003 |
| Feet (without hooves) | Human food | 0.0046 | 0.0050 | 0.0000 |
| Floatation fat | C1-C2 for disposal | 0.0000 | 0.0000 | 0.0000 |
| Head | Human food | 0.0052 | 0.0050 | 0.0081 |
| Intestines | C1-C2 for disposal | 0.0000 | 0.0000 | 0.0000 |
| Kidney | Human food | 0.0044 | 0.0050 | 0.0038 |
| Manure | Spreading/Compost | 0.0000 | 0.0000 | 0.0000 |
| Meat | Human food | 0.0044 | 0.0050 | 0.0074 |
| Pluck | Human food | 0.0048 | 0.0050 | 0.0015 |
| Rumen and forestomach | Human food | 0.0046 | 0.0050 | 0.0002 |
| SPA C3 | PAP C3 | 0.0002 | 0.0050 | 0.0003 |
| Screening and sifting wastes | C1-C2 for disposal | 0.0000 | 0.0000 | 0.0000 |
| Skin | Skin tannery C3 | 0.0062 | 0.0050 | 0.0043 |
| Sludge | Spreading/Compost | 0.0000 | 0.0000 | 0.0000 |
| Spleen | Pet food | 0.0046 | 0.0050 | 0.0003 |
| Sweetbread | Human food | 0.0056 | 0.0050 | 0.0057 |

Table 25: Allocation factors for Limousine Milk-fed Calf reared in Grazing Large Area

| COPRODUCT | Destination | Limousine /milk-fed calf/grazing large area | | |
| --- | --- | --- | --- | --- |
|  |  | **Biophysical Allocation Factor** | **Mass Allocation Factor** | **Economic Allocation Factor** |
| Abomasum | Human food | 0.0037 | 0.0041 | 0.0002 |
| Aponevrosis (1%) | Human food | 0.0044 | 0.0041 | 0.0029 |
| Bile | PAP C3 | 0.0015 | 0.0041 | 0.0002 |
| Blood | C1-C2 for disposal | 0.0000 | 0.0000 | 0.0000 |
| Bones (11%) | Gelatin C3 | 0.0043 | 0.0041 | 0.0000 |
| Dead individuals | C1-C2 for disposal | 0.0000 | 0.0000 | 0.0000 |
| Fat (8%) | Fat and greaves C3 | 0.0105 | 0.0041 | 0.0003 |
| Fat from breasts and penis | Fat and greaves C3 | 0.0105 | 0.0041 | 0.0003 |
| Feet (without hooves) | Human food | 0.0037 | 0.0041 | 0.0000 |
| Floatation fat | C1-C2 for disposal | 0.0000 | 0.0000 | 0.0000 |
| Head | Human food | 0.0042 | 0.0041 | 0.0066 |
| Intestines | C1-C2 for disposal | 0.0000 | 0.0000 | 0.0000 |
| Kidney | Human food | 0.0036 | 0.0041 | 0.0030 |
| Manure | Spreading/Compost | 0.0000 | 0.0000 | 0.0000 |
| Meat | Human food | 0.0036 | 0.0041 | 0.0060 |
| Pluck | Human food | 0.0040 | 0.0041 | 0.0012 |
| Rumen and forestomach | Human food | 0.0037 | 0.0041 | 0.0002 |
| SPA C3 | PAP C3 | 0.0002 | 0.0041 | 0.0002 |
| Screening and sifting wastes | C1-C2 for disposal | 0.0000 | 0.0000 | 0.0000 |
| Skin | Skin tannery C3 | 0.0051 | 0.0041 | 0.0035 |
| Sludge | Spreading/Compost | 0.0000 | 0.0000 | 0.0000 |
| Spleen | Pet food | 0.0037 | 0.0041 | 0.0002 |
| Sweetbread | Human food | 0.0045 | 0.0041 | 0.0046 |

Table 26: Allocation factors for Limousine Milk-fed Calf reared in Pasture

| COPRODUCT | Destination | Limousine /milk-fed calf/PASTURE | | |
| --- | --- | --- | --- | --- |
|  |  | **Biophysical Allocation Factor** | **Mass Allocation Factor** | **Economic Allocation Factor** |
| Abomasum | Human food | 0.0037 | 0.0041 | 0.0002 |
| Aponevrosis (1%) | Human food | 0.0044 | 0.0041 | 0.0029 |
| Bile | PAP C3 | 0.0015 | 0.0041 | 0.0002 |
| Blood | C1-C2 for disposal | 0.0000 | 0.0000 | 0.0000 |
| Bones (11%) | Gelatin C3 | 0.0042 | 0.0041 | 0.0000 |
| Dead individuals | C1-C2 for disposal | 0.0000 | 0.0000 | 0.0000 |
| Fat (8%) | Fat and greaves C3 | 0.0109 | 0.0041 | 0.0003 |
| Fat from breasts and penis | Fat and greaves C3 | 0.0109 | 0.0041 | 0.0003 |
| Feet (without hooves) | Human food | 0.0037 | 0.0041 | 0.0000 |
| Floatation fat | C1-C2 for disposal | 0.0000 | 0.0000 | 0.0000 |
| Head | Human food | 0.0042 | 0.0041 | 0.0066 |
| Intestines | C1-C2 for disposal | 0.0000 | 0.0000 | 0.0000 |
| Kidney | Human food | 0.0036 | 0.0041 | 0.0030 |
| Manure | Spreading/Compost | 0.0000 | 0.0000 | 0.0000 |
| Meat | Human food | 0.0036 | 0.0041 | 0.0060 |
| Pluck | Human food | 0.0039 | 0.0041 | 0.0012 |
| Rumen and forestomach | Human food | 0.0037 | 0.0041 | 0.0002 |
| SPA C3 | PAP C3 | 0.0002 | 0.0041 | 0.0002 |
| Screening and sifting wastes | C1-C2 for disposal | 0.0000 | 0.0000 | 0.0000 |
| Skin | Skin tannery C3 | 0.0050 | 0.0041 | 0.0035 |
| Sludge | Spreading/Compost | 0.0000 | 0.0000 | 0.0000 |
| Spleen | Pet food | 0.0037 | 0.0041 | 0.0002 |
| Sweetbread | Human food | 0.0045 | 0.0041 | 0.0046 |

Table 27: Allocation factors for Limousine Milk-fed Calf reared in Stall

| COPRODUCT | Destination | Limousine /Milk-fed calf/Stall | | |
| --- | --- | --- | --- | --- |
|  |  | **Biophysical Allocation Factor** | **Mass Allocation Factor** | **Economic Allocation Factor** |
| Abomasum | Human food | 0.0037 | 0.0041 | 0.0002 |
| Aponevrosis (1%) | Human food | 0.0044 | 0.0041 | 0.0029 |
| Bile | PAP C3 | 0.0015 | 0.0041 | 0.0002 |
| Blood | C1-C2 for disposal | 0.0000 | 0.0000 | 0.0000 |
| Bones (11%) | Gelatin C3 | 0.0042 | 0.0041 | 0.0000 |
| Dead individuals | C1-C2 for disposal | 0.0000 | 0.0000 | 0.0000 |
| Fat (8%) | Fat and greaves C3 | 0.0113 | 0.0041 | 0.0003 |
| Fat from breasts and penis | Fat and greaves C3 | 0.0113 | 0.0041 | 0.0003 |
| Feet (without hooves) | Human food | 0.0037 | 0.0041 | 0.0000 |
| Floatation fat | C1-C2 for disposal | 0.0000 | 0.0000 | 0.0000 |
| Head | Human food | 0.0042 | 0.0041 | 0.0066 |
| Intestines | C1-C2 for disposal | 0.0000 | 0.0000 | 0.0000 |
| Kidney | Human food | 0.0035 | 0.0041 | 0.0030 |
| Manure | Spreading/Compost | 0.0000 | 0.0000 | 0.0000 |
| Meat | Human food | 0.0036 | 0.0041 | 0.0060 |
| Pluck | Human food | 0.0039 | 0.0041 | 0.0012 |
| Rumen and forestomach | Human food | 0.0037 | 0.0041 | 0.0002 |
| SPA C3 | PAP C3 | 0.0002 | 0.0041 | 0.0002 |
| Screening and sifting wastes | C1-C2 for disposal | 0.0000 | 0.0000 | 0.0000 |
| Skin | Skin tannery C3 | 0.0049 | 0.0041 | 0.0035 |
| Sludge | Spreading/Compost | 0.0000 | 0.0000 | 0.0000 |
| Spleen | Pet food | 0.0037 | 0.0041 | 0.0002 |
| Sweetbread | Human food | 0.0045 | 0.0041 | 0.0046 |

Table 28: Allocation factors for Limousine Rosé Calf reared in Grazing Large Area

| COPRODUCT | Destination | Limousine /rosé calf/grazing large area | | |
| --- | --- | --- | --- | --- |
|  |  | **Biophysical Allocation Factor** | **Mass Allocation Factor** | **Economic Allocation Factor** |
| Abomasum | Human food | 0.0043 | 0.0047 | 0.0002 |
| Aponevrosis (1%) | Human food | 0.0051 | 0.0047 | 0.0033 |
| Bile | PAP C3 | 0.0017 | 0.0047 | 0.0003 |
| Blood | C1-C2 for disposal | 0.0000 | 0.0000 | 0.0000 |
| Bones (11%) | Gelatin C3 | 0.0049 | 0.0047 | 0.0000 |
| Dead individuals | C1-C2 for disposal | 0.0000 | 0.0000 | 0.0000 |
| Fat (8%) | Fat and greaves C3 | 0.0117 | 0.0047 | 0.0003 |
| Fat from breasts and penis | Fat and greaves C3 | 0.0117 | 0.0047 | 0.0003 |
| Feet (without hooves) | Human food | 0.0043 | 0.0047 | 0.0000 |
| Floatation fat | C1-C2 for disposal | 0.0000 | 0.0000 | 0.0000 |
| Head | Human food | 0.0049 | 0.0047 | 0.0075 |
| Intestines | C1-C2 for disposal | 0.0000 | 0.0000 | 0.0000 |
| Kidney | Human food | 0.0042 | 0.0047 | 0.0035 |
| Manure | Spreading/Compost | 0.0000 | 0.0000 | 0.0000 |
| Meat | Human food | 0.0042 | 0.0047 | 0.0069 |
| Pluck | Human food | 0.0046 | 0.0047 | 0.0014 |
| Rumen and forestomach | Human food | 0.0043 | 0.0047 | 0.0002 |
| SPA C3 | PAP C3 | 0.0002 | 0.0047 | 0.0003 |
| Screening and sifting wastes | C1-C2 for disposal | 0.0000 | 0.0000 | 0.0000 |
| Skin | Skin tannery C3 | 0.0059 | 0.0047 | 0.0040 |
| Sludge | Spreading/Compost | 0.0000 | 0.0000 | 0.0000 |
| Spleen | Pet food | 0.0043 | 0.0047 | 0.0002 |
| Sweetbread | Human food | 0.0052 | 0.0047 | 0.0053 |

Table 29: Allocation factors for Limousine Rosé Calf reared in Pasture

| COPRODUCT | Destination | Limousine /Rosé calf/Pasture | | |
| --- | --- | --- | --- | --- |
|  |  | **Biophysical Allocation Factor** | **Mass Allocation Factor** | **Economic Allocation Factor** |
| Abomasum | Human food | 0.0043 | 0.0047 | 0.0002 |
| Aponevrosis (1%) | Human food | 0.0051 | 0.0047 | 0.0033 |
| Bile | PAP C3 | 0.0017 | 0.0047 | 0.0003 |
| Blood | C1-C2 for disposal | 0.0000 | 0.0000 | 0.0000 |
| Bones (11%) | Gelatin C3 | 0.0049 | 0.0047 | 0.0000 |
| Dead individuals | C1-C2 for disposal | 0.0000 | 0.0000 | 0.0000 |
| Fat (8%) | Fat and greaves C3 | 0.0122 | 0.0047 | 0.0003 |
| Fat from breasts and penis | Fat and greaves C3 | 0.0122 | 0.0047 | 0.0003 |
| Feet (without hooves) | Human food | 0.0043 | 0.0047 | 0.0000 |
| Floatation fat | C1-C2 for disposal | 0.0000 | 0.0000 | 0.0000 |
| Head | Human food | 0.0048 | 0.0047 | 0.0075 |
| Intestines | C1-C2 for disposal | 0.0000 | 0.0000 | 0.0000 |
| Kidney | Human food | 0.0041 | 0.0047 | 0.0035 |
| Manure | Spreading/Compost | 0.0000 | 0.0000 | 0.0000 |
| Meat | Human food | 0.0042 | 0.0047 | 0.0069 |
| Pluck | Human food | 0.0045 | 0.0047 | 0.0014 |
| Rumen and forestomach | Human food | 0.0043 | 0.0047 | 0.0002 |
| SPA C3 | PAP C3 | 0.0002 | 0.0047 | 0.0003 |
| Screening and sifting wastes | C1-C2 for disposal | 0.0000 | 0.0000 | 0.0000 |
| Skin | Skin tannery C3 | 0.0058 | 0.0047 | 0.0040 |
| Sludge | Spreading/Compost | 0.0000 | 0.0000 | 0.0000 |
| Spleen | Pet food | 0.0043 | 0.0047 | 0.0002 |
| Sweetbread | Human food | 0.0052 | 0.0047 | 0.0053 |

Table 30: Allocation factors for Limousine Rosé Calf reared in Stall

| COPRODUCT | Destination | Limousine /Rosé calf/Stall | | |
| --- | --- | --- | --- | --- |
|  |  | **Biophysical Allocation Factor** | **Mass Allocation Factor** | **Economic Allocation Factor** |
| Abomasum | Human food | 0.0043 | 0.0047 | 0.0002 |
| Aponevrosis (1%) | Human food | 0.0051 | 0.0047 | 0.0033 |
| Bile | PAP C3 | 0.0017 | 0.0047 | 0.0003 |
| Blood | C1-C2 for disposal | 0.0000 | 0.0000 | 0.0000 |
| Bones (11%) | Gelatin C3 | 0.0048 | 0.0047 | 0.0000 |
| Dead individuals | C1-C2 for disposal | 0.0000 | 0.0000 | 0.0000 |
| Fat (8%) | Fat and greaves C3 | 0.0126 | 0.0047 | 0.0003 |
| Fat from breasts and penis | Fat and greaves C3 | 0.0126 | 0.0047 | 0.0003 |
| Feet (without hooves) | Human food | 0.0043 | 0.0047 | 0.0000 |
| Floatation fat | C1-C2 for disposal | 0.0000 | 0.0000 | 0.0000 |
| Head | Human food | 0.0048 | 0.0047 | 0.0075 |
| Intestines | C1-C2 for disposal | 0.0000 | 0.0000 | 0.0000 |
| Kidney | Human food | 0.0041 | 0.0047 | 0.0035 |
| Manure | Spreading/Compost | 0.0000 | 0.0000 | 0.0000 |
| Meat | Human food | 0.0041 | 0.0047 | 0.0069 |
| Pluck | Human food | 0.0045 | 0.0047 | 0.0014 |
| Rumen and forestomach | Human food | 0.0043 | 0.0047 | 0.0002 |
| SPA C3 | PAP C3 | 0.0002 | 0.0047 | 0.0003 |
| Screening and sifting wastes | C1-C2 for disposal | 0.0000 | 0.0000 | 0.0000 |
| Skin | Skin tannery C3 | 0.0057 | 0.0047 | 0.0040 |
| Sludge | Spreading/Compost | 0.0000 | 0.0000 | 0.0000 |
| Spleen | Pet food | 0.0043 | 0.0047 | 0.0002 |
| Sweetbread | Human food | 0.0052 | 0.0047 | 0.0053 |

Table 31: Allocation factors for Montbéliarde Milk-fed Calf reared in Grazing Large Area

| COPRODUCT | Destination | Montbéliarde /milk-fed calf/grazing large area | | |
| --- | --- | --- | --- | --- |
|  |  | **Biophysical Allocation Factor** | **Mass Allocation Factor** | **Economic Allocation Factor** |
| Abomasum | Human food | 0.0045 | 0.0049 | 0.0002 |
| Aponevrosis (1%) | Human food | 0.0053 | 0.0049 | 0.0034 |
| Bile | PAP C3 | 0.0018 | 0.0049 | 0.0003 |
| Blood | C1-C2 for disposal | 0.0000 | 0.0000 | 0.0000 |
| Bones (11%) | Gelatin C3 | 0.0051 | 0.0049 | 0.0000 |
| Dead individuals | C1-C2 for disposal | 0.0000 | 0.0000 | 0.0000 |
| Fat (8%) | Fat and greaves C3 | 0.0121 | 0.0049 | 0.0003 |
| Fat from breasts and penis | Fat and greaves C3 | 0.0121 | 0.0049 | 0.0003 |
| Feet (without hooves) | Human food | 0.0045 | 0.0049 | 0.0000 |
| Floatation fat | C1-C2 for disposal | 0.0000 | 0.0000 | 0.0000 |
| Head | Human food | 0.0050 | 0.0049 | 0.0078 |
| Intestines | C1-C2 for disposal | 0.0000 | 0.0000 | 0.0000 |
| Kidney | Human food | 0.0043 | 0.0049 | 0.0036 |
| Manure | Spreading/Compost | 0.0000 | 0.0000 | 0.0000 |
| Meat | Human food | 0.0043 | 0.0049 | 0.0071 |
| Pluck | Human food | 0.0047 | 0.0049 | 0.0015 |
| Rumen and forestomach | Human food | 0.0045 | 0.0049 | 0.0002 |
| SPA C3 | PAP C3 | 0.0002 | 0.0049 | 0.0003 |
| Screening and sifting wastes | C1-C2 for disposal | 0.0000 | 0.0000 | 0.0000 |
| Skin | Skin tannery C3 | 0.0061 | 0.0049 | 0.0042 |
| Sludge | Spreading/Compost | 0.0000 | 0.0000 | 0.0000 |
| Spleen | Pet food | 0.0045 | 0.0049 | 0.0003 |
| Sweetbread | Human food | 0.0054 | 0.0049 | 0.0055 |

Table 32: Allocation factors for Montbéliarde Milk-fed Calf reared in Pasture

| COPRODUCT | Destination | Montbéliarde /milk-fed calf/PASTURE | | |
| --- | --- | --- | --- | --- |
|  |  | **Biophysical Allocation Factor** | **Mass Allocation Factor** | **Economic Allocation Factor** |
| Abomasum | Human food | 0.0044 | 0.0049 | 0.0002 |
| Aponevrosis (1%) | Human food | 0.0053 | 0.0049 | 0.0034 |
| Bile | PAP C3 | 0.0018 | 0.0049 | 0.0003 |
| Blood | C1-C2 for disposal | 0.0000 | 0.0000 | 0.0000 |
| Bones (11%) | Gelatin C3 | 0.0051 | 0.0049 | 0.0000 |
| Dead individuals | C1-C2 for disposal | 0.0000 | 0.0000 | 0.0000 |
| Fat (8%) | Fat and greaves C3 | 0.0125 | 0.0049 | 0.0003 |
| Fat from breasts and penis | Fat and greaves C3 | 0.0125 | 0.0049 | 0.0003 |
| Feet (without hooves) | Human food | 0.0044 | 0.0049 | 0.0000 |
| Floatation fat | C1-C2 for disposal | 0.0000 | 0.0000 | 0.0000 |
| Head | Human food | 0.0050 | 0.0049 | 0.0078 |
| Intestines | C1-C2 for disposal | 0.0000 | 0.0000 | 0.0000 |
| Kidney | Human food | 0.0043 | 0.0049 | 0.0036 |
| Manure | Spreading/Compost | 0.0000 | 0.0000 | 0.0000 |
| Meat | Human food | 0.0043 | 0.0049 | 0.0071 |
| Pluck | Human food | 0.0047 | 0.0049 | 0.0015 |
| Rumen and forestomach | Human food | 0.0044 | 0.0049 | 0.0002 |
| SPA C3 | PAP C3 | 0.0002 | 0.0049 | 0.0003 |
| Screening and sifting wastes | C1-C2 for disposal | 0.0000 | 0.0000 | 0.0000 |
| Skin | Skin tannery C3 | 0.0060 | 0.0049 | 0.0042 |
| Sludge | Spreading/Compost | 0.0000 | 0.0000 | 0.0000 |
| Spleen | Pet food | 0.0044 | 0.0049 | 0.0003 |
| Sweetbread | Human food | 0.0054 | 0.0049 | 0.0055 |

Table 33: Allocation factors for Montbéliarde Milk-fed Calf reared in Stall

| COPRODUCT | Destination | Montbéliarde /Milk-fed calf/Stall | | |
| --- | --- | --- | --- | --- |
|  |  | **Biophysical Allocation Factor** | **Mass Allocation Factor** | **Economic Allocation Factor** |
| Abomasum | Human food | 0.0044 | 0.0049 | 0.0002 |
| Aponevrosis (1%) | Human food | 0.0052 | 0.0049 | 0.0034 |
| Bile | PAP C3 | 0.0018 | 0.0049 | 0.0003 |
| Blood | C1-C2 for disposal | 0.0000 | 0.0000 | 0.0000 |
| Bones (11%) | Gelatin C3 | 0.0050 | 0.0049 | 0.0000 |
| Dead individuals | C1-C2 for disposal | 0.0000 | 0.0000 | 0.0000 |
| Fat (8%) | Fat and greaves C3 | 0.0130 | 0.0049 | 0.0003 |
| Fat from breasts and penis | Fat and greaves C3 | 0.0130 | 0.0049 | 0.0003 |
| Feet (without hooves) | Human food | 0.0044 | 0.0049 | 0.0000 |
| Floatation fat | C1-C2 for disposal | 0.0000 | 0.0000 | 0.0000 |
| Head | Human food | 0.0050 | 0.0049 | 0.0078 |
| Intestines | C1-C2 for disposal | 0.0000 | 0.0000 | 0.0000 |
| Kidney | Human food | 0.0042 | 0.0049 | 0.0036 |
| Manure | Spreading/Compost | 0.0000 | 0.0000 | 0.0000 |
| Meat | Human food | 0.0043 | 0.0049 | 0.0071 |
| Pluck | Human food | 0.0047 | 0.0049 | 0.0015 |
| Rumen and forestomach | Human food | 0.0044 | 0.0049 | 0.0002 |
| SPA C3 | PAP C3 | 0.0002 | 0.0049 | 0.0003 |
| Screening and sifting wastes | C1-C2 for disposal | 0.0000 | 0.0000 | 0.0000 |
| Skin | Skin tannery C3 | 0.0059 | 0.0049 | 0.0042 |
| Sludge | Spreading/Compost | 0.0000 | 0.0000 | 0.0000 |
| Spleen | Pet food | 0.0044 | 0.0049 | 0.0003 |
| Sweetbread | Human food | 0.0054 | 0.0049 | 0.0055 |

Table 34: Allocation factors for Montbéliarde Rosé Calf reared in Grazing Large Area

| COPRODUCT | Destination | Montbéliarde /rosé calf/grazing large area | | |
| --- | --- | --- | --- | --- |
|  |  | **Biophysical Allocation Factor** | **Mass Allocation Factor** | **Economic Allocation Factor** |
| Abomasum | Human food | 0.0051 | 0.0056 | 0.0002 |
| Aponevrosis (1%) | Human food | 0.0061 | 0.0056 | 0.0039 |
| Bile | PAP C3 | 0.0020 | 0.0056 | 0.0003 |
| Blood | C1-C2 for disposal | 0.0000 | 0.0000 | 0.0000 |
| Bones (11%) | Gelatin C3 | 0.0059 | 0.0056 | 0.0000 |
| Dead individuals | C1-C2 for disposal | 0.0000 | 0.0000 | 0.0000 |
| Fat (8%) | Fat and greaves C3 | 0.0135 | 0.0056 | 0.0004 |
| Fat from breasts and penis | Fat and greaves C3 | 0.0135 | 0.0056 | 0.0004 |
| Feet (without hooves) | Human food | 0.0051 | 0.0056 | 0.0000 |
| Floatation fat | C1-C2 for disposal | 0.0000 | 0.0000 | 0.0000 |
| Head | Human food | 0.0058 | 0.0056 | 0.0089 |
| Intestines | C1-C2 for disposal | 0.0000 | 0.0000 | 0.0000 |
| Kidney | Human food | 0.0050 | 0.0056 | 0.0042 |
| Manure | Spreading/Compost | 0.0000 | 0.0000 | 0.0000 |
| Meat | Human food | 0.0050 | 0.0056 | 0.0082 |
| Pluck | Human food | 0.0055 | 0.0056 | 0.0017 |
| Rumen and forestomach | Human food | 0.0051 | 0.0056 | 0.0002 |
| SPA C3 | PAP C3 | 0.0002 | 0.0056 | 0.0003 |
| Screening and sifting wastes | C1-C2 for disposal | 0.0000 | 0.0000 | 0.0000 |
| Skin | Skin tannery C3 | 0.0070 | 0.0056 | 0.0048 |
| Sludge | Spreading/Compost | 0.0000 | 0.0000 | 0.0000 |
| Spleen | Pet food | 0.0051 | 0.0056 | 0.0003 |
| Sweetbread | Human food | 0.0063 | 0.0056 | 0.0063 |

Table 35: Allocation factors for Montbéliarde Rosé Calf reared in Pasture

| COPRODUCT | Destination | Montbéliarde /Rosé calf/Pasture | | |
| --- | --- | --- | --- | --- |
|  |  | **Biophysical Allocation Factor** | **Mass Allocation Factor** | **Economic Allocation Factor** |
| Abomasum | Human food | 0.0051 | 0.0056 | 0.0002 |
| Aponevrosis (1%) | Human food | 0.0061 | 0.0056 | 0.0039 |
| Bile | PAP C3 | 0.0020 | 0.0056 | 0.0003 |
| Blood | C1-C2 for disposal | 0.0000 | 0.0000 | 0.0000 |
| Bones (11%) | Gelatin C3 | 0.0058 | 0.0056 | 0.0000 |
| Dead individuals | C1-C2 for disposal | 0.0000 | 0.0000 | 0.0000 |
| Fat (8%) | Fat and greaves C3 | 0.0139 | 0.0056 | 0.0004 |
| Fat from breasts and penis | Fat and greaves C3 | 0.0139 | 0.0056 | 0.0004 |
| Feet (without hooves) | Human food | 0.0051 | 0.0056 | 0.0000 |
| Floatation fat | C1-C2 for disposal | 0.0000 | 0.0000 | 0.0000 |
| Head | Human food | 0.0058 | 0.0056 | 0.0089 |
| Intestines | C1-C2 for disposal | 0.0000 | 0.0000 | 0.0000 |
| Kidney | Human food | 0.0049 | 0.0056 | 0.0042 |
| Manure | Spreading/Compost | 0.0000 | 0.0000 | 0.0000 |
| Meat | Human food | 0.0050 | 0.0056 | 0.0082 |
| Pluck | Human food | 0.0054 | 0.0056 | 0.0017 |
| Rumen and forestomach | Human food | 0.0051 | 0.0056 | 0.0002 |
| SPA C3 | PAP C3 | 0.0002 | 0.0056 | 0.0003 |
| Screening and sifting wastes | C1-C2 for disposal | 0.0000 | 0.0000 | 0.0000 |
| Skin | Skin tannery C3 | 0.0070 | 0.0056 | 0.0048 |
| Sludge | Spreading/Compost | 0.0000 | 0.0000 | 0.0000 |
| Spleen | Pet food | 0.0051 | 0.0056 | 0.0003 |
| Sweetbread | Human food | 0.0062 | 0.0056 | 0.0063 |

Table 36: Allocation factors for Montbéliarde Rosé Calf reared in Stall

| COPRODUCT | Destination | Montbéliarde /Rosé calf/Stall | | |
| --- | --- | --- | --- | --- |
|  |  | **Biophysical Allocation Factor** | **Mass Allocation Factor** | **Economic Allocation Factor** |
| Abomasum | Human food | 0.0051 | 0.0056 | 0.0002 |
| Aponevrosis (1%) | Human food | 0.0060 | 0.0056 | 0.0039 |
| Bile | PAP C3 | 0.0020 | 0.0056 | 0.0003 |
| Blood | C1-C2 for disposal | 0.0000 | 0.0000 | 0.0000 |
| Bones (11%) | Gelatin C3 | 0.0058 | 0.0056 | 0.0000 |
| Dead individuals | C1-C2 for disposal | 0.0000 | 0.0000 | 0.0000 |
| Fat (8%) | Fat and greaves C3 | 0.0144 | 0.0056 | 0.0004 |
| Fat from breasts and penis | Fat and greaves C3 | 0.0144 | 0.0056 | 0.0004 |
| Feet (without hooves) | Human food | 0.0051 | 0.0056 | 0.0000 |
| Floatation fat | C1-C2 for disposal | 0.0000 | 0.0000 | 0.0000 |
| Head | Human food | 0.0057 | 0.0056 | 0.0089 |
| Intestines | C1-C2 for disposal | 0.0000 | 0.0000 | 0.0000 |
| Kidney | Human food | 0.0049 | 0.0056 | 0.0042 |
| Manure | Spreading/Compost | 0.0000 | 0.0000 | 0.0000 |
| Meat | Human food | 0.0049 | 0.0056 | 0.0082 |
| Pluck | Human food | 0.0054 | 0.0056 | 0.0017 |
| Rumen and forestomach | Human food | 0.0051 | 0.0056 | 0.0002 |
| SPA C3 | PAP C3 | 0.0002 | 0.0056 | 0.0003 |
| Screening and sifting wastes | C1-C2 for disposal | 0.0000 | 0.0000 | 0.0000 |
| Skin | Skin tannery C3 | 0.0069 | 0.0056 | 0.0048 |
| Sludge | Spreading/Compost | 0.0000 | 0.0000 | 0.0000 |
| Spleen | Pet food | 0.0051 | 0.0056 | 0.0003 |
| Sweetbread | Human food | 0.0062 | 0.0056 | 0.0063 |

Table 37: Allocation factors for Normande Milk-fed Calf reared in Grazing Large Area

| COPRODUCT | Destination | Normande /milk-fed calf/grazing large area | | |
| --- | --- | --- | --- | --- |
|  |  | **Biophysical Allocation Factor** | **Mass Allocation Factor** | **Economic Allocation Factor** |
| Abomasum | Human food | 0.0044 | 0.0048 | 0.0002 |
| Aponevrosis (1%) | Human food | 0.0053 | 0.0048 | 0.0034 |
| Bile | PAP C3 | 0.0018 | 0.0048 | 0.0003 |
| Blood | C1-C2 for disposal | 0.0000 | 0.0000 | 0.0000 |
| Bones (11%) | Gelatin C3 | 0.0051 | 0.0048 | 0.0000 |
| Dead individuals | C1-C2 for disposal | 0.0000 | 0.0000 | 0.0000 |
| Fat (8%) | Fat and greaves C3 | 0.0120 | 0.0048 | 0.0003 |
| Fat from breasts and penis | Fat and greaves C3 | 0.0120 | 0.0048 | 0.0003 |
| Feet (without hooves) | Human food | 0.0044 | 0.0048 | 0.0000 |
| Floatation fat | C1-C2 for disposal | 0.0000 | 0.0000 | 0.0000 |
| Head | Human food | 0.0050 | 0.0048 | 0.0078 |
| Intestines | C1-C2 for disposal | 0.0000 | 0.0000 | 0.0000 |
| Kidney | Human food | 0.0043 | 0.0048 | 0.0036 |
| Manure | Spreading/Compost | 0.0000 | 0.0000 | 0.0000 |
| Meat | Human food | 0.0043 | 0.0048 | 0.0071 |
| Pluck | Human food | 0.0047 | 0.0048 | 0.0015 |
| Rumen and forestomach | Human food | 0.0044 | 0.0048 | 0.0002 |
| SPA C3 | PAP C3 | 0.0002 | 0.0048 | 0.0003 |
| Screening and sifting wastes | C1-C2 for disposal | 0.0000 | 0.0000 | 0.0000 |
| Skin | Skin tannery C3 | 0.0061 | 0.0048 | 0.0041 |
| Sludge | Spreading/Compost | 0.0000 | 0.0000 | 0.0000 |
| Spleen | Pet food | 0.0044 | 0.0048 | 0.0003 |
| Sweetbread | Human food | 0.0054 | 0.0048 | 0.0054 |

Table 38: Allocation factors for Normande Milk-fed Calf reared in Pasture

| COPRODUCT | Destination | Normande /milk-fed calf/PASTURE | | |
| --- | --- | --- | --- | --- |
|  |  | **Biophysical Allocation Factor** | **Mass Allocation Factor** | **Economic Allocation Factor** |
| Abomasum | Human food | 0.0044 | 0.0048 | 0.0002 |
| Aponevrosis (1%) | Human food | 0.0053 | 0.0048 | 0.0034 |
| Bile | PAP C3 | 0.0018 | 0.0048 | 0.0003 |
| Blood | C1-C2 for disposal | 0.0000 | 0.0000 | 0.0000 |
| Bones (11%) | Gelatin C3 | 0.0050 | 0.0048 | 0.0000 |
| Dead individuals | C1-C2 for disposal | 0.0000 | 0.0000 | 0.0000 |
| Fat (8%) | Fat and greaves C3 | 0.0125 | 0.0048 | 0.0003 |
| Fat from breasts and penis | Fat and greaves C3 | 0.0125 | 0.0048 | 0.0003 |
| Feet (without hooves) | Human food | 0.0044 | 0.0048 | 0.0000 |
| Floatation fat | C1-C2 for disposal | 0.0000 | 0.0000 | 0.0000 |
| Head | Human food | 0.0050 | 0.0048 | 0.0078 |
| Intestines | C1-C2 for disposal | 0.0000 | 0.0000 | 0.0000 |
| Kidney | Human food | 0.0043 | 0.0048 | 0.0036 |
| Manure | Spreading/Compost | 0.0000 | 0.0000 | 0.0000 |
| Meat | Human food | 0.0043 | 0.0048 | 0.0071 |
| Pluck | Human food | 0.0047 | 0.0048 | 0.0015 |
| Rumen and forestomach | Human food | 0.0044 | 0.0048 | 0.0002 |
| SPA C3 | PAP C3 | 0.0002 | 0.0048 | 0.0003 |
| Screening and sifting wastes | C1-C2 for disposal | 0.0000 | 0.0000 | 0.0000 |
| Skin | Skin tannery C3 | 0.0060 | 0.0048 | 0.0041 |
| Sludge | Spreading/Compost | 0.0000 | 0.0000 | 0.0000 |
| Spleen | Pet food | 0.0044 | 0.0048 | 0.0003 |
| Sweetbread | Human food | 0.0054 | 0.0048 | 0.0054 |

Table 39: Allocation factors for Normande Milk-fed Calf reared in Stall

| COPRODUCT | Destination | Normande /Milk-fed calf/Stall | | |
| --- | --- | --- | --- | --- |
|  |  | **Biophysical Allocation Factor** | **Mass Allocation Factor** | **Economic Allocation Factor** |
| Abomasum | Human food | 0.0044 | 0.0048 | 0.0002 |
| Aponevrosis (1%) | Human food | 0.0052 | 0.0048 | 0.0034 |
| Bile | PAP C3 | 0.0018 | 0.0048 | 0.0003 |
| Blood | C1-C2 for disposal | 0.0000 | 0.0000 | 0.0000 |
| Bones (11%) | Gelatin C3 | 0.0050 | 0.0048 | 0.0000 |
| Dead individuals | C1-C2 for disposal | 0.0000 | 0.0000 | 0.0000 |
| Fat (8%) | Fat and greaves C3 | 0.0129 | 0.0048 | 0.0003 |
| Fat from breasts and penis | Fat and greaves C3 | 0.0129 | 0.0048 | 0.0003 |
| Feet (without hooves) | Human food | 0.0044 | 0.0048 | 0.0000 |
| Floatation fat | C1-C2 for disposal | 0.0000 | 0.0000 | 0.0000 |
| Head | Human food | 0.0050 | 0.0048 | 0.0078 |
| Intestines | C1-C2 for disposal | 0.0000 | 0.0000 | 0.0000 |
| Kidney | Human food | 0.0042 | 0.0048 | 0.0036 |
| Manure | Spreading/Compost | 0.0000 | 0.0000 | 0.0000 |
| Meat | Human food | 0.0043 | 0.0048 | 0.0071 |
| Pluck | Human food | 0.0046 | 0.0048 | 0.0015 |
| Rumen and forestomach | Human food | 0.0044 | 0.0048 | 0.0002 |
| SPA C3 | PAP C3 | 0.0002 | 0.0048 | 0.0003 |
| Screening and sifting wastes | C1-C2 for disposal | 0.0000 | 0.0000 | 0.0000 |
| Skin | Skin tannery C3 | 0.0059 | 0.0048 | 0.0041 |
| Sludge | Spreading/Compost | 0.0000 | 0.0000 | 0.0000 |
| Spleen | Pet food | 0.0044 | 0.0048 | 0.0003 |
| Sweetbread | Human food | 0.0053 | 0.0048 | 0.0054 |

Table 40: Allocation factors for Normande Rosé Calf reared in Grazing Large Area

| COPRODUCT | Destination | Normande /rosé calf/grazing large area | | |
| --- | --- | --- | --- | --- |
|  |  | **Biophysical Allocation Factor** | **Mass Allocation Factor** | **Economic Allocation Factor** |
| Abomasum | Human food | 0.0051 | 0.0055 | 0.0002 |
| Aponevrosis (1%) | Human food | 0.0061 | 0.0055 | 0.0039 |
| Bile | PAP C3 | 0.0020 | 0.0055 | 0.0003 |
| Blood | C1-C2 for disposal | 0.0000 | 0.0000 | 0.0000 |
| Bones (11%) | Gelatin C3 | 0.0059 | 0.0055 | 0.0000 |
| Dead individuals | C1-C2 for disposal | 0.0000 | 0.0000 | 0.0000 |
| Fat (8%) | Fat and greaves C3 | 0.0134 | 0.0055 | 0.0004 |
| Fat from breasts and penis | Fat and greaves C3 | 0.0134 | 0.0055 | 0.0004 |
| Feet (without hooves) | Human food | 0.0051 | 0.0055 | 0.0000 |
| Floatation fat | C1-C2 for disposal | 0.0000 | 0.0000 | 0.0000 |
| Head | Human food | 0.0058 | 0.0055 | 0.0089 |
| Intestines | C1-C2 for disposal | 0.0000 | 0.0000 | 0.0000 |
| Kidney | Human food | 0.0050 | 0.0055 | 0.0041 |
| Manure | Spreading/Compost | 0.0000 | 0.0000 | 0.0000 |
| Meat | Human food | 0.0050 | 0.0055 | 0.0081 |
| Pluck | Human food | 0.0054 | 0.0055 | 0.0017 |
| Rumen and forestomach | Human food | 0.0051 | 0.0055 | 0.0002 |
| SPA C3 | PAP C3 | 0.0002 | 0.0055 | 0.0003 |
| Screening and sifting wastes | C1-C2 for disposal | 0.0000 | 0.0000 | 0.0000 |
| Skin | Skin tannery C3 | 0.0070 | 0.0055 | 0.0047 |
| Sludge | Spreading/Compost | 0.0000 | 0.0000 | 0.0000 |
| Spleen | Pet food | 0.0051 | 0.0055 | 0.0003 |
| Sweetbread | Human food | 0.0062 | 0.0055 | 0.0062 |

Table 41: Allocation factors for Normande Rosé Calf reared in Pasture

| COPRODUCT | Destination | Normande /Rosé calf/Pasture | | |
| --- | --- | --- | --- | --- |
|  |  | **Biophysical Allocation Factor** | **Mass Allocation Factor** | **Economic Allocation Factor** |
| Abomasum | Human food | 0.0051 | 0.0055 | 0.0002 |
| Aponevrosis (1%) | Human food | 0.0061 | 0.0055 | 0.0039 |
| Bile | PAP C3 | 0.0020 | 0.0055 | 0.0003 |
| Blood | C1-C2 for disposal | 0.0000 | 0.0000 | 0.0000 |
| Bones (11%) | Gelatin C3 | 0.0058 | 0.0055 | 0.0000 |
| Dead individuals | C1-C2 for disposal | 0.0000 | 0.0000 | 0.0000 |
| Fat (8%) | Fat and greaves C3 | 0.0139 | 0.0055 | 0.0004 |
| Fat from breasts and penis | Fat and greaves C3 | 0.0139 | 0.0055 | 0.0004 |
| Feet (without hooves) | Human food | 0.0051 | 0.0055 | 0.0000 |
| Floatation fat | C1-C2 for disposal | 0.0000 | 0.0000 | 0.0000 |
| Head | Human food | 0.0057 | 0.0055 | 0.0089 |
| Intestines | C1-C2 for disposal | 0.0000 | 0.0000 | 0.0000 |
| Kidney | Human food | 0.0049 | 0.0055 | 0.0041 |
| Manure | Spreading/Compost | 0.0000 | 0.0000 | 0.0000 |
| Meat | Human food | 0.0049 | 0.0055 | 0.0081 |
| Pluck | Human food | 0.0054 | 0.0055 | 0.0017 |
| Rumen and forestomach | Human food | 0.0051 | 0.0055 | 0.0002 |
| SPA C3 | PAP C3 | 0.0002 | 0.0055 | 0.0003 |
| Screening and sifting wastes | C1-C2 for disposal | 0.0000 | 0.0000 | 0.0000 |
| Skin | Skin tannery C3 | 0.0069 | 0.0055 | 0.0047 |
| Sludge | Spreading/Compost | 0.0000 | 0.0000 | 0.0000 |
| Spleen | Pet food | 0.0051 | 0.0055 | 0.0003 |
| Sweetbread | Human food | 0.0062 | 0.0055 | 0.0062 |

Table 42: Allocation factors for Normande Rosé Calf reared in Stall

| COPRODUCT | Destination | Normande /Rosé calf/Stall | | |
| --- | --- | --- | --- | --- |
|  |  | **Biophysical Allocation Factor** | **Mass Allocation Factor** | **Economic Allocation Factor** |
| Abomasum | Human food | 0.0050 | 0.0055 | 0.0002 |
| Aponevrosis (1%) | Human food | 0.0060 | 0.0055 | 0.0039 |
| Bile | PAP C3 | 0.0020 | 0.0055 | 0.0003 |
| Blood | C1-C2 for disposal | 0.0000 | 0.0000 | 0.0000 |
| Bones (11%) | Gelatin C3 | 0.0057 | 0.0055 | 0.0000 |
| Dead individuals | C1-C2 for disposal | 0.0000 | 0.0000 | 0.0000 |
| Fat (8%) | Fat and greaves C3 | 0.0144 | 0.0055 | 0.0004 |
| Fat from breasts and penis | Fat and greaves C3 | 0.0144 | 0.0055 | 0.0004 |
| Feet (without hooves) | Human food | 0.0050 | 0.0055 | 0.0000 |
| Floatation fat | C1-C2 for disposal | 0.0000 | 0.0000 | 0.0000 |
| Head | Human food | 0.0057 | 0.0055 | 0.0089 |
| Intestines | C1-C2 for disposal | 0.0000 | 0.0000 | 0.0000 |
| Kidney | Human food | 0.0049 | 0.0055 | 0.0041 |
| Manure | Spreading/Compost | 0.0000 | 0.0000 | 0.0000 |
| Meat | Human food | 0.0049 | 0.0055 | 0.0081 |
| Pluck | Human food | 0.0054 | 0.0055 | 0.0017 |
| Rumen and forestomach | Human food | 0.0050 | 0.0055 | 0.0002 |
| SPA C3 | PAP C3 | 0.0002 | 0.0055 | 0.0003 |
| Screening and sifting wastes | C1-C2 for disposal | 0.0000 | 0.0000 | 0.0000 |
| Skin | Skin tannery C3 | 0.0068 | 0.0055 | 0.0047 |
| Sludge | Spreading/Compost | 0.0000 | 0.0000 | 0.0000 |
| Spleen | Pet food | 0.0050 | 0.0055 | 0.0003 |
| Sweetbread | Human food | 0.0061 | 0.0055 | 0.0062 |

Table 43: Allocation factors for Primholstein Milk-fed Calf reared in Grazing Large Area

| COPRODUCT | Destination | Primholstein /milk-fed calf/grazing large area | | |
| --- | --- | --- | --- | --- |
|  |  | **Biophysical Allocation Factor** | **Mass Allocation Factor** | **Economic Allocation Factor** |
| Abomasum | Human food | 0.0043 | 0.0047 | 0.0002 |
| Aponevrosis (1%) | Human food | 0.0051 | 0.0047 | 0.0033 |
| Bile | PAP C3 | 0.0017 | 0.0047 | 0.0003 |
| Blood | C1-C2 for disposal | 0.0000 | 0.0000 | 0.0000 |
| Bones (11%) | Gelatin C3 | 0.0049 | 0.0047 | 0.0000 |
| Dead individuals | C1-C2 for disposal | 0.0000 | 0.0000 | 0.0000 |
| Fat (8%) | Fat and greaves C3 | 0.0117 | 0.0047 | 0.0003 |
| Fat from breasts and penis | Fat and greaves C3 | 0.0117 | 0.0047 | 0.0003 |
| Feet (without hooves) | Human food | 0.0043 | 0.0047 | 0.0000 |
| Floatation fat | C1-C2 for disposal | 0.0000 | 0.0000 | 0.0000 |
| Head | Human food | 0.0049 | 0.0047 | 0.0075 |
| Intestines | C1-C2 for disposal | 0.0000 | 0.0000 | 0.0000 |
| Kidney | Human food | 0.0042 | 0.0047 | 0.0035 |
| Manure | Spreading/Compost | 0.0000 | 0.0000 | 0.0000 |
| Meat | Human food | 0.0042 | 0.0047 | 0.0069 |
| Pluck | Human food | 0.0046 | 0.0047 | 0.0014 |
| Rumen and forestomach | Human food | 0.0043 | 0.0047 | 0.0002 |
| SPA C3 | PAP C3 | 0.0002 | 0.0047 | 0.0003 |
| Screening and sifting wastes | C1-C2 for disposal | 0.0000 | 0.0000 | 0.0000 |
| Skin | Skin tannery C3 | 0.0059 | 0.0047 | 0.0040 |
| Sludge | Spreading/Compost | 0.0000 | 0.0000 | 0.0000 |
| Spleen | Pet food | 0.0043 | 0.0047 | 0.0002 |
| Sweetbread | Human food | 0.0052 | 0.0047 | 0.0053 |

Table 44: Allocation factors for Primholstein Milk-fed Calf reared in Pasture

| COPRODUCT | Destination | Primholstein /milk-fed calf/PASTURE | | |
| --- | --- | --- | --- | --- |
|  |  | **Biophysical Allocation Factor** | **Mass Allocation Factor** | **Economic Allocation Factor** |
| Abomasum | Human food | 0.0043 | 0.0047 | 0.0002 |
| Aponevrosis (1%) | Human food | 0.0051 | 0.0047 | 0.0033 |
| Bile | PAP C3 | 0.0017 | 0.0047 | 0.0003 |
| Blood | C1-C2 for disposal | 0.0000 | 0.0000 | 0.0000 |
| Bones (11%) | Gelatin C3 | 0.0049 | 0.0047 | 0.0000 |
| Dead individuals | C1-C2 for disposal | 0.0000 | 0.0000 | 0.0000 |
| Fat (8%) | Fat and greaves C3 | 0.0122 | 0.0047 | 0.0003 |
| Fat from breasts and penis | Fat and greaves C3 | 0.0122 | 0.0047 | 0.0003 |
| Feet (without hooves) | Human food | 0.0043 | 0.0047 | 0.0000 |
| Floatation fat | C1-C2 for disposal | 0.0000 | 0.0000 | 0.0000 |
| Head | Human food | 0.0048 | 0.0047 | 0.0075 |
| Intestines | C1-C2 for disposal | 0.0000 | 0.0000 | 0.0000 |
| Kidney | Human food | 0.0041 | 0.0047 | 0.0035 |
| Manure | Spreading/Compost | 0.0000 | 0.0000 | 0.0000 |
| Meat | Human food | 0.0042 | 0.0047 | 0.0069 |
| Pluck | Human food | 0.0045 | 0.0047 | 0.0014 |
| Rumen and forestomach | Human food | 0.0043 | 0.0047 | 0.0002 |
| SPA C3 | PAP C3 | 0.0002 | 0.0047 | 0.0003 |
| Screening and sifting wastes | C1-C2 for disposal | 0.0000 | 0.0000 | 0.0000 |
| Skin | Skin tannery C3 | 0.0058 | 0.0047 | 0.0040 |
| Sludge | Spreading/Compost | 0.0000 | 0.0000 | 0.0000 |
| Spleen | Pet food | 0.0043 | 0.0047 | 0.0002 |
| Sweetbread | Human food | 0.0052 | 0.0047 | 0.0053 |

Table 45: Allocation factors for Primholstein Milk-fed Calf reared in Stall

| COPRODUCT | Destination | Primholstein /Milk-fed calf/Stall | | |
| --- | --- | --- | --- | --- |
|  |  | **Biophysical Allocation Factor** | **Mass Allocation Factor** | **Economic Allocation Factor** |
| Abomasum | Human food | 0.0043 | 0.0047 | 0.0002 |
| Aponevrosis (1%) | Human food | 0.0051 | 0.0047 | 0.0033 |
| Bile | PAP C3 | 0.0017 | 0.0047 | 0.0003 |
| Blood | C1-C2 for disposal | 0.0000 | 0.0000 | 0.0000 |
| Bones (11%) | Gelatin C3 | 0.0048 | 0.0047 | 0.0000 |
| Dead individuals | C1-C2 for disposal | 0.0000 | 0.0000 | 0.0000 |
| Fat (8%) | Fat and greaves C3 | 0.0126 | 0.0047 | 0.0003 |
| Fat from breasts and penis | Fat and greaves C3 | 0.0126 | 0.0047 | 0.0003 |
| Feet (without hooves) | Human food | 0.0043 | 0.0047 | 0.0000 |
| Floatation fat | C1-C2 for disposal | 0.0000 | 0.0000 | 0.0000 |
| Head | Human food | 0.0048 | 0.0047 | 0.0075 |
| Intestines | C1-C2 for disposal | 0.0000 | 0.0000 | 0.0000 |
| Kidney | Human food | 0.0041 | 0.0047 | 0.0035 |
| Manure | Spreading/Compost | 0.0000 | 0.0000 | 0.0000 |
| Meat | Human food | 0.0041 | 0.0047 | 0.0069 |
| Pluck | Human food | 0.0045 | 0.0047 | 0.0014 |
| Rumen and forestomach | Human food | 0.0043 | 0.0047 | 0.0002 |
| SPA C3 | PAP C3 | 0.0002 | 0.0047 | 0.0003 |
| Screening and sifting wastes | C1-C2 for disposal | 0.0000 | 0.0000 | 0.0000 |
| Skin | Skin tannery C3 | 0.0057 | 0.0047 | 0.0040 |
| Sludge | Spreading/Compost | 0.0000 | 0.0000 | 0.0000 |
| Spleen | Pet food | 0.0043 | 0.0047 | 0.0002 |
| Sweetbread | Human food | 0.0052 | 0.0047 | 0.0053 |

Table 46: Allocation factors for Primholstein Rosé Calf reared in Grazing Large Area

| COPRODUCT | Destination | Primholstein /rosé calf/grazing large area | | |
| --- | --- | --- | --- | --- |
|  |  | **Biophysical Allocation Factor** | **Mass Allocation Factor** | **Economic Allocation Factor** |
| Abomasum | Human food | 0.0049 | 0.0054 | 0.0002 |
| Aponevrosis (1%) | Human food | 0.0059 | 0.0054 | 0.0038 |
| Bile | PAP C3 | 0.0020 | 0.0054 | 0.0003 |
| Blood | C1-C2 for disposal | 0.0000 | 0.0000 | 0.0000 |
| Bones (11%) | Gelatin C3 | 0.0057 | 0.0054 | 0.0000 |
| Dead individuals | C1-C2 for disposal | 0.0000 | 0.0000 | 0.0000 |
| Fat (8%) | Fat and greaves C3 | 0.0131 | 0.0054 | 0.0003 |
| Fat from breasts and penis | Fat and greaves C3 | 0.0131 | 0.0054 | 0.0003 |
| Feet (without hooves) | Human food | 0.0049 | 0.0054 | 0.0000 |
| Floatation fat | C1-C2 for disposal | 0.0000 | 0.0000 | 0.0000 |
| Head | Human food | 0.0056 | 0.0054 | 0.0086 |
| Intestines | C1-C2 for disposal | 0.0000 | 0.0000 | 0.0000 |
| Kidney | Human food | 0.0048 | 0.0054 | 0.0040 |
| Manure | Spreading/Compost | 0.0000 | 0.0000 | 0.0000 |
| Meat | Human food | 0.0048 | 0.0054 | 0.0079 |
| Pluck | Human food | 0.0053 | 0.0054 | 0.0016 |
| Rumen and forestomach | Human food | 0.0049 | 0.0054 | 0.0002 |
| SPA C3 | PAP C3 | 0.0002 | 0.0054 | 0.0003 |
| Screening and sifting wastes | C1-C2 for disposal | 0.0000 | 0.0000 | 0.0000 |
| Skin | Skin tannery C3 | 0.0068 | 0.0054 | 0.0046 |
| Sludge | Spreading/Compost | 0.0000 | 0.0000 | 0.0000 |
| Spleen | Pet food | 0.0049 | 0.0054 | 0.0003 |
| Sweetbread | Human food | 0.0060 | 0.0054 | 0.0060 |

Table 47: Allocation factors for Primholstein Rosé Calf reared in Pasture

| COPRODUCT | Destination | Primholstein /Rosé calf/Pasture | | |
| --- | --- | --- | --- | --- |
|  |  | **Biophysical Allocation Factor** | **Mass Allocation Factor** | **Economic Allocation Factor** |
| Abomasum | Human food | 0.0049 | 0.0054 | 0.0002 |
| Aponevrosis (1%) | Human food | 0.0059 | 0.0054 | 0.0038 |
| Bile | PAP C3 | 0.0020 | 0.0054 | 0.0003 |
| Blood | C1-C2 for disposal | 0.0000 | 0.0000 | 0.0000 |
| Bones (11%) | Gelatin C3 | 0.0056 | 0.0054 | 0.0000 |
| Dead individuals | C1-C2 for disposal | 0.0000 | 0.0000 | 0.0000 |
| Fat (8%) | Fat and greaves C3 | 0.0135 | 0.0054 | 0.0003 |
| Fat from breasts and penis | Fat and greaves C3 | 0.0135 | 0.0054 | 0.0003 |
| Feet (without hooves) | Human food | 0.0049 | 0.0054 | 0.0000 |
| Floatation fat | C1-C2 for disposal | 0.0000 | 0.0000 | 0.0000 |
| Head | Human food | 0.0056 | 0.0054 | 0.0086 |
| Intestines | C1-C2 for disposal | 0.0000 | 0.0000 | 0.0000 |
| Kidney | Human food | 0.0048 | 0.0054 | 0.0040 |
| Manure | Spreading/Compost | 0.0000 | 0.0000 | 0.0000 |
| Meat | Human food | 0.0048 | 0.0054 | 0.0079 |
| Pluck | Human food | 0.0052 | 0.0054 | 0.0016 |
| Rumen and forestomach | Human food | 0.0049 | 0.0054 | 0.0002 |
| SPA C3 | PAP C3 | 0.0002 | 0.0054 | 0.0003 |
| Screening and sifting wastes | C1-C2 for disposal | 0.0000 | 0.0000 | 0.0000 |
| Skin | Skin tannery C3 | 0.0067 | 0.0054 | 0.0046 |
| Sludge | Spreading/Compost | 0.0000 | 0.0000 | 0.0000 |
| Spleen | Pet food | 0.0049 | 0.0054 | 0.0003 |
| Sweetbread | Human food | 0.0060 | 0.0054 | 0.0060 |

Table 48: Allocation factors for Primholstein Rosé Calf reared in Stall

| COPRODUCT | Destination | Primholstein /Rosé calf/Stall | | |
| --- | --- | --- | --- | --- |
|  |  | **Biophysical Allocation Factor** | **Mass Allocation Factor** | **Economic Allocation Factor** |
| Abomasum | Human food | 0.0049 | 0.0054 | 0.0002 |
| Aponevrosis (1%) | Human food | 0.0058 | 0.0054 | 0.0038 |
| Bile | PAP C3 | 0.0020 | 0.0054 | 0.0003 |
| Blood | C1-C2 for disposal | 0.0000 | 0.0000 | 0.0000 |
| Bones (11%) | Gelatin C3 | 0.0056 | 0.0054 | 0.0000 |
| Dead individuals | C1-C2 for disposal | 0.0000 | 0.0000 | 0.0000 |
| Fat (8%) | Fat and greaves C3 | 0.0140 | 0.0054 | 0.0003 |
| Fat from breasts and penis | Fat and greaves C3 | 0.0140 | 0.0054 | 0.0003 |
| Feet (without hooves) | Human food | 0.0049 | 0.0054 | 0.0000 |
| Floatation fat | C1-C2 for disposal | 0.0000 | 0.0000 | 0.0000 |
| Head | Human food | 0.0055 | 0.0054 | 0.0086 |
| Intestines | C1-C2 for disposal | 0.0000 | 0.0000 | 0.0000 |
| Kidney | Human food | 0.0047 | 0.0054 | 0.0040 |
| Manure | Spreading/Compost | 0.0000 | 0.0000 | 0.0000 |
| Meat | Human food | 0.0048 | 0.0054 | 0.0079 |
| Pluck | Human food | 0.0052 | 0.0054 | 0.0016 |
| Rumen and forestomach | Human food | 0.0049 | 0.0054 | 0.0002 |
| SPA C3 | PAP C3 | 0.0002 | 0.0054 | 0.0003 |
| Screening and sifting wastes | C1-C2 for disposal | 0.0000 | 0.0000 | 0.0000 |
| Skin | Skin tannery C3 | 0.0066 | 0.0054 | 0.0046 |
| Sludge | Spreading/Compost | 0.0000 | 0.0000 | 0.0000 |
| Spleen | Pet food | 0.0049 | 0.0054 | 0.0003 |
| Sweetbread | Human food | 0.0060 | 0.0054 | 0.0060 |

Table 49: Allocation factors for Croisé-lait Milk-fed Calf reared in Grazing Large Area

| COPRODUCT | Destination | Croisé-lait /milk-fed calf/grazing large area | | |
| --- | --- | --- | --- | --- |
|  |  | **Biophysical Allocation Factor** | **Mass Allocation Factor** | **Economic Allocation Factor** |
| Abomasum | Human food | 0.0043 | 0.0047 | 0.0002 |
| Aponevrosis (1%) | Human food | 0.0051 | 0.0047 | 0.0033 |
| Bile | PAP C3 | 0.0017 | 0.0047 | 0.0003 |
| Blood | C1-C2 for disposal | 0.0000 | 0.0000 | 0.0000 |
| Bones (11%) | Gelatin C3 | 0.0049 | 0.0047 | 0.0000 |
| Dead individuals | C1-C2 for disposal | 0.0000 | 0.0000 | 0.0000 |
| Fat (8%) | Fat and greaves C3 | 0.0117 | 0.0047 | 0.0003 |
| Fat from breasts and penis | Fat and greaves C3 | 0.0117 | 0.0047 | 0.0003 |
| Feet (without hooves) | Human food | 0.0043 | 0.0047 | 0.0000 |
| Floatation fat | C1-C2 for disposal | 0.0000 | 0.0000 | 0.0000 |
| Head | Human food | 0.0048 | 0.0047 | 0.0075 |
| Intestines | C1-C2 for disposal | 0.0000 | 0.0000 | 0.0000 |
| Kidney | Human food | 0.0041 | 0.0047 | 0.0035 |
| Manure | Spreading/Compost | 0.0000 | 0.0000 | 0.0000 |
| Meat | Human food | 0.0042 | 0.0047 | 0.0068 |
| Pluck | Human food | 0.0045 | 0.0047 | 0.0014 |
| Rumen and forestomach | Human food | 0.0043 | 0.0047 | 0.0002 |
| SPA C3 | PAP C3 | 0.0002 | 0.0047 | 0.0003 |
| Screening and sifting wastes | C1-C2 for disposal | 0.0000 | 0.0000 | 0.0000 |
| Skin | Skin tannery C3 | 0.0058 | 0.0047 | 0.0040 |
| Sludge | Spreading/Compost | 0.0000 | 0.0000 | 0.0000 |
| Spleen | Pet food | 0.0043 | 0.0047 | 0.0002 |
| Sweetbread | Human food | 0.0052 | 0.0047 | 0.0052 |

Table 50: Allocation factors for Croisé-lait Milk-fed Calf reared in Pasture

| COPRODUCT | Destination | Croisé-lait /milk-fed calf/PASTURE | | |
| --- | --- | --- | --- | --- |
|  |  | **Biophysical Allocation Factor** | **Mass Allocation Factor** | **Economic Allocation Factor** |
| Abomasum | Human food | 0.0042 | 0.0047 | 0.0002 |
| Aponevrosis (1%) | Human food | 0.0050 | 0.0047 | 0.0033 |
| Bile | PAP C3 | 0.0017 | 0.0047 | 0.0003 |
| Blood | C1-C2 for disposal | 0.0000 | 0.0000 | 0.0000 |
| Bones (11%) | Gelatin C3 | 0.0048 | 0.0047 | 0.0000 |
| Dead individuals | C1-C2 for disposal | 0.0000 | 0.0000 | 0.0000 |
| Fat (8%) | Fat and greaves C3 | 0.0121 | 0.0047 | 0.0003 |
| Fat from breasts and penis | Fat and greaves C3 | 0.0121 | 0.0047 | 0.0003 |
| Feet (without hooves) | Human food | 0.0042 | 0.0047 | 0.0000 |
| Floatation fat | C1-C2 for disposal | 0.0000 | 0.0000 | 0.0000 |
| Head | Human food | 0.0048 | 0.0047 | 0.0075 |
| Intestines | C1-C2 for disposal | 0.0000 | 0.0000 | 0.0000 |
| Kidney | Human food | 0.0041 | 0.0047 | 0.0035 |
| Manure | Spreading/Compost | 0.0000 | 0.0000 | 0.0000 |
| Meat | Human food | 0.0041 | 0.0047 | 0.0068 |
| Pluck | Human food | 0.0045 | 0.0047 | 0.0014 |
| Rumen and forestomach | Human food | 0.0042 | 0.0047 | 0.0002 |
| SPA C3 | PAP C3 | 0.0002 | 0.0047 | 0.0003 |
| Screening and sifting wastes | C1-C2 for disposal | 0.0000 | 0.0000 | 0.0000 |
| Skin | Skin tannery C3 | 0.0058 | 0.0047 | 0.0040 |
| Sludge | Spreading/Compost | 0.0000 | 0.0000 | 0.0000 |
| Spleen | Pet food | 0.0042 | 0.0047 | 0.0002 |
| Sweetbread | Human food | 0.0052 | 0.0047 | 0.0052 |

Table 51: Allocation factors for Croisé-lait Milk-fed Calf reared in Stall

| COPRODUCT | Destination | Croisé-lait /Milk-fed calf/Stall | | |
| --- | --- | --- | --- | --- |
|  |  | **Biophysical Allocation Factor** | **Mass Allocation Factor** | **Economic Allocation Factor** |
| Abomasum | Human food | 0.0042 | 0.0047 | 0.0002 |
| Aponevrosis (1%) | Human food | 0.0050 | 0.0047 | 0.0033 |
| Bile | PAP C3 | 0.0017 | 0.0047 | 0.0003 |
| Blood | C1-C2 for disposal | 0.0000 | 0.0000 | 0.0000 |
| Bones (11%) | Gelatin C3 | 0.0048 | 0.0047 | 0.0000 |
| Dead individuals | C1-C2 for disposal | 0.0000 | 0.0000 | 0.0000 |
| Fat (8%) | Fat and greaves C3 | 0.0125 | 0.0047 | 0.0003 |
| Fat from breasts and penis | Fat and greaves C3 | 0.0125 | 0.0047 | 0.0003 |
| Feet (without hooves) | Human food | 0.0042 | 0.0047 | 0.0000 |
| Floatation fat | C1-C2 for disposal | 0.0000 | 0.0000 | 0.0000 |
| Head | Human food | 0.0048 | 0.0047 | 0.0075 |
| Intestines | C1-C2 for disposal | 0.0000 | 0.0000 | 0.0000 |
| Kidney | Human food | 0.0040 | 0.0047 | 0.0035 |
| Manure | Spreading/Compost | 0.0000 | 0.0000 | 0.0000 |
| Meat | Human food | 0.0041 | 0.0047 | 0.0068 |
| Pluck | Human food | 0.0045 | 0.0047 | 0.0014 |
| Rumen and forestomach | Human food | 0.0042 | 0.0047 | 0.0002 |
| SPA C3 | PAP C3 | 0.0002 | 0.0047 | 0.0003 |
| Screening and sifting wastes | C1-C2 for disposal | 0.0000 | 0.0000 | 0.0000 |
| Skin | Skin tannery C3 | 0.0057 | 0.0047 | 0.0040 |
| Sludge | Spreading/Compost | 0.0000 | 0.0000 | 0.0000 |
| Spleen | Pet food | 0.0042 | 0.0047 | 0.0002 |
| Sweetbread | Human food | 0.0051 | 0.0047 | 0.0052 |

Table 52: Allocation factors for Croisé-viande Rosé Calf reared in Grazing Large Area

| COPRODUCT | Destination | Croisé-lait /rosé calf/grazing large area | | |
| --- | --- | --- | --- | --- |
|  |  | **Biophysical Allocation Factor** | **Mass Allocation Factor** | **Economic Allocation Factor** |
| Abomasum | Human food | 0.0039 | 0.0043 | 0.0002 |
| Aponevrosis (1%) | Human food | 0.0047 | 0.0043 | 0.0031 |
| Bile | PAP C3 | 0.0016 | 0.0043 | 0.0003 |
| Blood | C1-C2 for disposal | 0.0000 | 0.0000 | 0.0000 |
| Bones (11%) | Gelatin C3 | 0.0045 | 0.0043 | 0.0000 |
| Dead individuals | C1-C2 for disposal | 0.0000 | 0.0000 | 0.0000 |
| Fat (8%) | Fat and greaves C3 | 0.0110 | 0.0043 | 0.0003 |
| Fat from breasts and penis | Fat and greaves C3 | 0.0110 | 0.0043 | 0.0003 |
| Feet (without hooves) | Human food | 0.0039 | 0.0043 | 0.0000 |
| Floatation fat | C1-C2 for disposal | 0.0000 | 0.0000 | 0.0000 |
| Head | Human food | 0.0045 | 0.0043 | 0.0069 |
| Intestines | C1-C2 for disposal | 0.0000 | 0.0000 | 0.0000 |
| Kidney | Human food | 0.0038 | 0.0043 | 0.0032 |
| Manure | Spreading/Compost | 0.0000 | 0.0000 | 0.0000 |
| Meat | Human food | 0.0038 | 0.0043 | 0.0063 |
| Pluck | Human food | 0.0042 | 0.0043 | 0.0013 |
| Rumen and forestomach | Human food | 0.0039 | 0.0043 | 0.0002 |
| SPA C3 | PAP C3 | 0.0002 | 0.0043 | 0.0003 |
| Screening and sifting wastes | C1-C2 for disposal | 0.0000 | 0.0000 | 0.0000 |
| Skin | Skin tannery C3 | 0.0054 | 0.0043 | 0.0037 |
| Sludge | Spreading/Compost | 0.0000 | 0.0000 | 0.0000 |
| Spleen | Pet food | 0.0039 | 0.0043 | 0.0002 |
| Sweetbread | Human food | 0.0048 | 0.0043 | 0.0048 |

Table 53: Allocation factors for Croisé-viande Rosé Calf reared in Pasture

| COPRODUCT | Destination | Croisé-lait /Rosé calf/Pasture | | |
| --- | --- | --- | --- | --- |
|  |  | **Biophysical Allocation Factor** | **Mass Allocation Factor** | **Economic Allocation Factor** |
| Abomasum | Human food | 0.0039 | 0.0043 | 0.0002 |
| Aponevrosis (1%) | Human food | 0.0047 | 0.0043 | 0.0031 |
| Bile | PAP C3 | 0.0016 | 0.0043 | 0.0003 |
| Blood | C1-C2 for disposal | 0.0000 | 0.0000 | 0.0000 |
| Bones (11%) | Gelatin C3 | 0.0045 | 0.0043 | 0.0000 |
| Dead individuals | C1-C2 for disposal | 0.0000 | 0.0000 | 0.0000 |
| Fat (8%) | Fat and greaves C3 | 0.0114 | 0.0043 | 0.0003 |
| Fat from breasts and penis | Fat and greaves C3 | 0.0114 | 0.0043 | 0.0003 |
| Feet (without hooves) | Human food | 0.0039 | 0.0043 | 0.0000 |
| Floatation fat | C1-C2 for disposal | 0.0000 | 0.0000 | 0.0000 |
| Head | Human food | 0.0044 | 0.0043 | 0.0069 |
| Intestines | C1-C2 for disposal | 0.0000 | 0.0000 | 0.0000 |
| Kidney | Human food | 0.0038 | 0.0043 | 0.0032 |
| Manure | Spreading/Compost | 0.0000 | 0.0000 | 0.0000 |
| Meat | Human food | 0.0038 | 0.0043 | 0.0063 |
| Pluck | Human food | 0.0041 | 0.0043 | 0.0013 |
| Rumen and forestomach | Human food | 0.0039 | 0.0043 | 0.0002 |
| SPA C3 | PAP C3 | 0.0002 | 0.0043 | 0.0003 |
| Screening and sifting wastes | C1-C2 for disposal | 0.0000 | 0.0000 | 0.0000 |
| Skin | Skin tannery C3 | 0.0053 | 0.0043 | 0.0037 |
| Sludge | Spreading/Compost | 0.0000 | 0.0000 | 0.0000 |
| Spleen | Pet food | 0.0039 | 0.0043 | 0.0002 |
| Sweetbread | Human food | 0.0048 | 0.0043 | 0.0048 |

Table 54: Allocation factors for Croisé-viande Rosé Calf reared in Stall

| COPRODUCT | Destination | Croisé-lait /Rosé calf/Stall | | |
| --- | --- | --- | --- | --- |
|  |  | **Biophysical Allocation Factor** | **Mass Allocation Factor** | **Economic Allocation Factor** |
| Abomasum | Human food | 0.0039 | 0.0043 | 0.0002 |
| Aponevrosis (1%) | Human food | 0.0046 | 0.0043 | 0.0031 |
| Bile | PAP C3 | 0.0016 | 0.0043 | 0.0003 |
| Blood | C1-C2 for disposal | 0.0000 | 0.0000 | 0.0000 |
| Bones (11%) | Gelatin C3 | 0.0044 | 0.0043 | 0.0000 |
| Dead individuals | C1-C2 for disposal | 0.0000 | 0.0000 | 0.0000 |
| Fat (8%) | Fat and greaves C3 | 0.0118 | 0.0043 | 0.0003 |
| Fat from breasts and penis | Fat and greaves C3 | 0.0118 | 0.0043 | 0.0003 |
| Feet (without hooves) | Human food | 0.0039 | 0.0043 | 0.0000 |
| Floatation fat | C1-C2 for disposal | 0.0000 | 0.0000 | 0.0000 |
| Head | Human food | 0.0044 | 0.0043 | 0.0069 |
| Intestines | C1-C2 for disposal | 0.0000 | 0.0000 | 0.0000 |
| Kidney | Human food | 0.0037 | 0.0043 | 0.0032 |
| Manure | Spreading/Compost | 0.0000 | 0.0000 | 0.0000 |
| Meat | Human food | 0.0038 | 0.0043 | 0.0063 |
| Pluck | Human food | 0.0041 | 0.0043 | 0.0013 |
| Rumen and forestomach | Human food | 0.0039 | 0.0043 | 0.0002 |
| SPA C3 | PAP C3 | 0.0002 | 0.0043 | 0.0003 |
| Screening and sifting wastes | C1-C2 for disposal | 0.0000 | 0.0000 | 0.0000 |
| Skin | Skin tannery C3 | 0.0052 | 0.0043 | 0.0037 |
| Sludge | Spreading/Compost | 0.0000 | 0.0000 | 0.0000 |
| Spleen | Pet food | 0.0039 | 0.0043 | 0.0002 |
| Sweetbread | Human food | 0.0047 | 0.0043 | 0.0048 |
