# Supplementary material for: Allocation factors for meat coproducts: Dataset to perform life cycle assessment at slaughterhouse
Source: Data Brief. 2020 Nov 23;33:106558. doi: 10.1016/j.dib.2020.106558 (PMC7718151; doi:10.1016/j.dib.2020.106558)
Supplement: Supplementary file 5 [file mmc5.docx]

Table 1: Total weighting by coproducts for Average Milk-fed Calf reared in Grazing Large Area

| COPRODUCT | Destination | Average/milk-fed calf/grazing large area | | |
| --- | --- | --- | --- | --- |
|  |  | **Biophysical Cumulative Share** | **Mass Cumulative Share** | **Economic Cumulative Share** |
| Abomasum | Human food | 0.0047 | 0.0051 | 0.0002 |
| Aponevrosis (1%) | Human food | 0.0077 | 0.0071 | 0.0050 |
| Bile | PAP C3 | 0.0001 | 0.0004 | 0.0000 |
| Blood | C1-C2 for disposal | 0.0000 | 0.0000 | 0.0000 |
| Bones (11%) | Gelatin C3 | 0.0812 | 0.0777 | 0.0002 |
| Dead individuals | C1-C2 for disposal | 0.0000 | 0.0000 | 0.0000 |
| Fat (8%) | Fat and greaves C3 | 0.1431 | 0.0565 | 0.0036 |
| Fat from breasts and penis | Fat and greaves C3 | 0.0385 | 0.0152 | 0.0010 |
| Feet (without hooves) | Human food | 0.0185 | 0.0203 | 0.0000 |
| Floatation fat | C1-C2 for disposal | 0.0000 | 0.0000 | 0.0000 |
| Head | Human food | 0.0461 | 0.0447 | 0.0718 |
| Intestines | C1-C2 for disposal | 0.0000 | 0.0000 | 0.0000 |
| Kidney | Human food | 0.0030 | 0.0034 | 0.0025 |
| Manure | Spreading/Compost | 0.0000 | 0.0000 | 0.0000 |
| Meat | Human food | 0.5027 | 0.5654 | 0.8273 |
| Pluck | Human food | 0.0438 | 0.0452 | 0.0135 |
| Rumen and forestomach | Human food | 0.0043 | 0.0048 | 0.0002 |
| SPA C3 | PAP C3 | 0.0028 | 0.0703 | 0.0043 |
| Screening and sifting wastes | C1-C2 for disposal | 0.0000 | 0.0000 | 0.0000 |
| Skin | Skin tannery C3 | 0.0995 | 0.0799 | 0.0684 |
| Sludge | Spreading/Compost | 0.0000 | 0.0000 | 0.0000 |
| Spleen | Pet food | 0.0021 | 0.0023 | 0.0001 |
| Sweetbread | Human food | 0.0018 | 0.0016 | 0.0018 |

Table 2: Total weighting by coproducts for Average Milk-fed Calf reared in Pasture

| COPRODUCT | Destination | Average/milk-fed calf/PASTURE | | |
| --- | --- | --- | --- | --- |
|  |  | **Biophysical Cumulative Share** | **Mass Cumulative Share** | **Economic Cumulative Share** |
| Abomasum | Human food | 0.0046 | 0.0051 | 0.0002 |
| Aponevrosis (1%) | Human food | 0.0076 | 0.0071 | 0.0050 |
| Bile | PAP C3 | 0.0001 | 0.0004 | 0.0000 |
| Blood | C1-C2 for disposal | 0.0000 | 0.0000 | 0.0000 |
| Bones (11%) | Gelatin C3 | 0.0802 | 0.0777 | 0.0002 |
| Dead individuals | C1-C2 for disposal | 0.0000 | 0.0000 | 0.0000 |
| Fat (8%) | Fat and greaves C3 | 0.1485 | 0.0565 | 0.0036 |
| Fat from breasts and penis | Fat and greaves C3 | 0.0400 | 0.0152 | 0.0010 |
| Feet (without hooves) | Human food | 0.0184 | 0.0203 | 0.0000 |
| Floatation fat | C1-C2 for disposal | 0.0000 | 0.0000 | 0.0000 |
| Head | Human food | 0.0458 | 0.0447 | 0.0718 |
| Intestines | C1-C2 for disposal | 0.0000 | 0.0000 | 0.0000 |
| Kidney | Human food | 0.0030 | 0.0034 | 0.0025 |
| Manure | Spreading/Compost | 0.0000 | 0.0000 | 0.0000 |
| Meat | Human food | 0.4991 | 0.5654 | 0.8273 |
| Pluck | Human food | 0.0435 | 0.0452 | 0.0135 |
| Rumen and forestomach | Human food | 0.0043 | 0.0048 | 0.0002 |
| SPA C3 | PAP C3 | 0.0028 | 0.0703 | 0.0043 |
| Screening and sifting wastes | C1-C2 for disposal | 0.0000 | 0.0000 | 0.0000 |
| Skin | Skin tannery C3 | 0.0982 | 0.0799 | 0.0684 |
| Sludge | Spreading/Compost | 0.0000 | 0.0000 | 0.0000 |
| Spleen | Pet food | 0.0021 | 0.0023 | 0.0001 |
| Sweetbread | Human food | 0.0017 | 0.0016 | 0.0018 |

Table 3: Total weighting by coproducts for Average Milk-fed Calf reared in Stall

| COPRODUCT | Destination | Average/Milk-fed calf/Stall | | |
| --- | --- | --- | --- | --- |
|  |  | **Biophysical Cumulative Share** | **Mass Cumulative Share** | **Economic Cumulative Share** |
| Abomasum | Human food | 0.0046 | 0.0051 | 0.0002 |
| Aponevrosis (1%) | Human food | 0.0076 | 0.0071 | 0.0050 |
| Bile | PAP C3 | 0.0001 | 0.0004 | 0.0000 |
| Blood | C1-C2 for disposal | 0.0000 | 0.0000 | 0.0000 |
| Bones (11%) | Gelatin C3 | 0.0792 | 0.0777 | 0.0002 |
| Dead individuals | C1-C2 for disposal | 0.0000 | 0.0000 | 0.0000 |
| Fat (8%) | Fat and greaves C3 | 0.1540 | 0.0565 | 0.0036 |
| Fat from breasts and penis | Fat and greaves C3 | 0.0415 | 0.0152 | 0.0010 |
| Feet (without hooves) | Human food | 0.0183 | 0.0203 | 0.0000 |
| Floatation fat | C1-C2 for disposal | 0.0000 | 0.0000 | 0.0000 |
| Head | Human food | 0.0455 | 0.0447 | 0.0718 |
| Intestines | C1-C2 for disposal | 0.0000 | 0.0000 | 0.0000 |
| Kidney | Human food | 0.0029 | 0.0034 | 0.0025 |
| Manure | Spreading/Compost | 0.0000 | 0.0000 | 0.0000 |
| Meat | Human food | 0.4954 | 0.5654 | 0.8273 |
| Pluck | Human food | 0.0431 | 0.0452 | 0.0135 |
| Rumen and forestomach | Human food | 0.0043 | 0.0048 | 0.0002 |
| SPA C3 | PAP C3 | 0.0027 | 0.0703 | 0.0043 |
| Screening and sifting wastes | C1-C2 for disposal | 0.0000 | 0.0000 | 0.0000 |
| Skin | Skin tannery C3 | 0.0969 | 0.0799 | 0.0684 |
| Sludge | Spreading/Compost | 0.0000 | 0.0000 | 0.0000 |
| Spleen | Pet food | 0.0021 | 0.0023 | 0.0001 |
| Sweetbread | Human food | 0.0017 | 0.0016 | 0.0018 |

Table 4: Total weighting by coproducts for Average Rosé Calf reared in Grazing Large Area

| COPRODUCT | Destination | Average/rosé calf/grazing large area | | |
| --- | --- | --- | --- | --- |
|  |  | **Biophysical Cumulative Share** | **Mass Cumulative Share** | **Economic Cumulative Share** |
| Abomasum | Human food | 0.0047 | 0.0051 | 0.0002 |
| Aponevrosis (1%) | Human food | 0.0077 | 0.0071 | 0.0050 |
| Bile | PAP C3 | 0.0001 | 0.0004 | 0.0000 |
| Blood | C1-C2 for disposal | 0.0000 | 0.0000 | 0.0000 |
| Bones (11%) | Gelatin C3 | 0.0818 | 0.0777 | 0.0002 |
| Dead individuals | C1-C2 for disposal | 0.0000 | 0.0000 | 0.0000 |
| Fat (8%) | Fat and greaves C3 | 0.1392 | 0.0565 | 0.0036 |
| Fat from breasts and penis | Fat and greaves C3 | 0.0375 | 0.0152 | 0.0010 |
| Feet (without hooves) | Human food | 0.0186 | 0.0203 | 0.0000 |
| Floatation fat | C1-C2 for disposal | 0.0000 | 0.0000 | 0.0000 |
| Head | Human food | 0.0464 | 0.0447 | 0.0718 |
| Intestines | C1-C2 for disposal | 0.0000 | 0.0000 | 0.0000 |
| Kidney | Human food | 0.0030 | 0.0034 | 0.0025 |
| Manure | Spreading/Compost | 0.0000 | 0.0000 | 0.0000 |
| Meat | Human food | 0.5055 | 0.5654 | 0.8273 |
| Pluck | Human food | 0.0441 | 0.0452 | 0.0135 |
| Rumen and forestomach | Human food | 0.0044 | 0.0048 | 0.0002 |
| SPA C3 | PAP C3 | 0.0028 | 0.0703 | 0.0043 |
| Screening and sifting wastes | C1-C2 for disposal | 0.0000 | 0.0000 | 0.0000 |
| Skin | Skin tannery C3 | 0.1003 | 0.0799 | 0.0684 |
| Sludge | Spreading/Compost | 0.0000 | 0.0000 | 0.0000 |
| Spleen | Pet food | 0.0021 | 0.0023 | 0.0001 |
| Sweetbread | Human food | 0.0018 | 0.0016 | 0.0018 |

Table 5: Total weighting by coproducts for Average Rosé Calf reared in Pasture

| COPRODUCT | Destination | Average/Rosé calf/Pasture | | |
| --- | --- | --- | --- | --- |
|  |  | **Biophysical Cumulative Share** | **Mass Cumulative Share** | **Economic Cumulative Share** |
| Abomasum | Human food | 0.0047 | 0.0051 | 0.0002 |
| Aponevrosis (1%) | Human food | 0.0077 | 0.0071 | 0.0050 |
| Bile | PAP C3 | 0.0001 | 0.0004 | 0.0000 |
| Blood | C1-C2 for disposal | 0.0000 | 0.0000 | 0.0000 |
| Bones (11%) | Gelatin C3 | 0.0809 | 0.0777 | 0.0002 |
| Dead individuals | C1-C2 for disposal | 0.0000 | 0.0000 | 0.0000 |
| Fat (8%) | Fat and greaves C3 | 0.1442 | 0.0565 | 0.0036 |
| Fat from breasts and penis | Fat and greaves C3 | 0.0388 | 0.0152 | 0.0010 |
| Feet (without hooves) | Human food | 0.0185 | 0.0203 | 0.0000 |
| Floatation fat | C1-C2 for disposal | 0.0000 | 0.0000 | 0.0000 |
| Head | Human food | 0.0461 | 0.0447 | 0.0718 |
| Intestines | C1-C2 for disposal | 0.0000 | 0.0000 | 0.0000 |
| Kidney | Human food | 0.0030 | 0.0034 | 0.0025 |
| Manure | Spreading/Compost | 0.0000 | 0.0000 | 0.0000 |
| Meat | Human food | 0.5021 | 0.5654 | 0.8273 |
| Pluck | Human food | 0.0437 | 0.0452 | 0.0135 |
| Rumen and forestomach | Human food | 0.0043 | 0.0048 | 0.0002 |
| SPA C3 | PAP C3 | 0.0028 | 0.0703 | 0.0043 |
| Screening and sifting wastes | C1-C2 for disposal | 0.0000 | 0.0000 | 0.0000 |
| Skin | Skin tannery C3 | 0.0991 | 0.0799 | 0.0684 |
| Sludge | Spreading/Compost | 0.0000 | 0.0000 | 0.0000 |
| Spleen | Pet food | 0.0021 | 0.0023 | 0.0001 |
| Sweetbread | Human food | 0.0018 | 0.0016 | 0.0018 |

Table 6: Total weighting by coproducts for Rosé Calf reared in Stall

| COPRODUCT | Destination | Average/Rosé calf/Stall | | |
| --- | --- | --- | --- | --- |
|  |  | **Biophysical Cumulative Share** | **Mass Cumulative Share** | **Economic Cumulative Share** |
| Abomasum | Human food | 0.0046 | 0.0051 | 0.0002 |
| Aponevrosis (1%) | Human food | 0.0076 | 0.0071 | 0.0050 |
| Bile | PAP C3 | 0.0001 | 0.0004 | 0.0000 |
| Blood | C1-C2 for disposal | 0.0000 | 0.0000 | 0.0000 |
| Bones (11%) | Gelatin C3 | 0.0800 | 0.0777 | 0.0002 |
| Dead individuals | C1-C2 for disposal | 0.0000 | 0.0000 | 0.0000 |
| Fat (8%) | Fat and greaves C3 | 0.1493 | 0.0565 | 0.0036 |
| Fat from breasts and penis | Fat and greaves C3 | 0.0402 | 0.0152 | 0.0010 |
| Feet (without hooves) | Human food | 0.0184 | 0.0203 | 0.0000 |
| Floatation fat | C1-C2 for disposal | 0.0000 | 0.0000 | 0.0000 |
| Head | Human food | 0.0458 | 0.0447 | 0.0718 |
| Intestines | C1-C2 for disposal | 0.0000 | 0.0000 | 0.0000 |
| Kidney | Human food | 0.0030 | 0.0034 | 0.0025 |
| Manure | Spreading/Compost | 0.0000 | 0.0000 | 0.0000 |
| Meat | Human food | 0.4987 | 0.5654 | 0.8273 |
| Pluck | Human food | 0.0434 | 0.0452 | 0.0135 |
| Rumen and forestomach | Human food | 0.0043 | 0.0048 | 0.0002 |
| SPA C3 | PAP C3 | 0.0028 | 0.0703 | 0.0043 |
| Screening and sifting wastes | C1-C2 for disposal | 0.0000 | 0.0000 | 0.0000 |
| Skin | Skin tannery C3 | 0.0979 | 0.0799 | 0.0684 |
| Sludge | Spreading/Compost | 0.0000 | 0.0000 | 0.0000 |
| Spleen | Pet food | 0.0021 | 0.0023 | 0.0001 |
| Sweetbread | Human food | 0.0017 | 0.0016 | 0.0018 |

Table 7: Total weighting by coproducts for Aubrac Milk-fed Calf reared in Grazing Large Area

| COPRODUCT | Destination | Aubrac /milk-fed calf/grazing large area | | |
| --- | --- | --- | --- | --- |
|  |  | **Biophysical Cumulative Share** | **Mass Cumulative Share** | **Economic Cumulative Share** |
| Abomasum | Human food | 0.0047 | 0.0051 | 0.0002 |
| Aponevrosis (1%) | Human food | 0.0077 | 0.0071 | 0.0050 |
| Bile | PAP C3 | 0.0001 | 0.0004 | 0.0000 |
| Blood | C1-C2 for disposal | 0.0000 | 0.0000 | 0.0000 |
| Bones (11%) | Gelatin C3 | 0.0810 | 0.0777 | 0.0002 |
| Dead individuals | C1-C2 for disposal | 0.0000 | 0.0000 | 0.0000 |
| Fat (8%) | Fat and greaves C3 | 0.1442 | 0.0565 | 0.0036 |
| Fat from breasts and penis | Fat and greaves C3 | 0.0388 | 0.0152 | 0.0010 |
| Feet (without hooves) | Human food | 0.0185 | 0.0203 | 0.0000 |
| Floatation fat | C1-C2 for disposal | 0.0000 | 0.0000 | 0.0000 |
| Head | Human food | 0.0461 | 0.0447 | 0.0718 |
| Intestines | C1-C2 for disposal | 0.0000 | 0.0000 | 0.0000 |
| Kidney | Human food | 0.0030 | 0.0034 | 0.0025 |
| Manure | Spreading/Compost | 0.0000 | 0.0000 | 0.0000 |
| Meat | Human food | 0.5019 | 0.5654 | 0.8273 |
| Pluck | Human food | 0.0437 | 0.0452 | 0.0135 |
| Rumen and forestomach | Human food | 0.0043 | 0.0048 | 0.0002 |
| SPA C3 | PAP C3 | 0.0028 | 0.0703 | 0.0043 |
| Screening and sifting wastes | C1-C2 for disposal | 0.0000 | 0.0000 | 0.0000 |
| Skin | Skin tannery C3 | 0.0992 | 0.0799 | 0.0684 |
| Sludge | Spreading/Compost | 0.0000 | 0.0000 | 0.0000 |
| Spleen | Pet food | 0.0021 | 0.0023 | 0.0001 |
| Sweetbread | Human food | 0.0018 | 0.0016 | 0.0018 |

Table 8: Total weighting by coproducts for Aubrac Milk-fed Calf reared in Pasture

| COPRODUCT | Destination | Aubrac /milk-fed calf/PASTURE | | |
| --- | --- | --- | --- | --- |
|  |  | **Biophysical Cumulative Share** | **Mass Cumulative Share** | **Economic Cumulative Share** |
| Abomasum | Human food | 0.0046 | 0.0051 | 0.0002 |
| Aponevrosis (1%) | Human food | 0.0076 | 0.0071 | 0.0050 |
| Bile | PAP C3 | 0.0001 | 0.0004 | 0.0000 |
| Blood | C1-C2 for disposal | 0.0000 | 0.0000 | 0.0000 |
| Bones (11%) | Gelatin C3 | 0.0800 | 0.0777 | 0.0002 |
| Dead individuals | C1-C2 for disposal | 0.0000 | 0.0000 | 0.0000 |
| Fat (8%) | Fat and greaves C3 | 0.1497 | 0.0565 | 0.0036 |
| Fat from breasts and penis | Fat and greaves C3 | 0.0403 | 0.0152 | 0.0010 |
| Feet (without hooves) | Human food | 0.0184 | 0.0203 | 0.0000 |
| Floatation fat | C1-C2 for disposal | 0.0000 | 0.0000 | 0.0000 |
| Head | Human food | 0.0457 | 0.0447 | 0.0718 |
| Intestines | C1-C2 for disposal | 0.0000 | 0.0000 | 0.0000 |
| Kidney | Human food | 0.0030 | 0.0034 | 0.0025 |
| Manure | Spreading/Compost | 0.0000 | 0.0000 | 0.0000 |
| Meat | Human food | 0.4982 | 0.5654 | 0.8273 |
| Pluck | Human food | 0.0434 | 0.0452 | 0.0135 |
| Rumen and forestomach | Human food | 0.0043 | 0.0048 | 0.0002 |
| SPA C3 | PAP C3 | 0.0028 | 0.0703 | 0.0043 |
| Screening and sifting wastes | C1-C2 for disposal | 0.0000 | 0.0000 | 0.0000 |
| Skin | Skin tannery C3 | 0.0980 | 0.0799 | 0.0684 |
| Sludge | Spreading/Compost | 0.0000 | 0.0000 | 0.0000 |
| Spleen | Pet food | 0.0021 | 0.0023 | 0.0001 |
| Sweetbread | Human food | 0.0017 | 0.0016 | 0.0018 |

Table 9: Total weighting by coproducts for Aubrac Milk-fed Calf reared in Stall

| COPRODUCT | Destination | Aubrac /Milk-fed calf/Stall | | |
| --- | --- | --- | --- | --- |
|  |  | **Biophysical Cumulative Share** | **Mass Cumulative Share** | **Economic Cumulative Share** |
| Abomasum | Human food | 0.0046 | 0.0051 | 0.0002 |
| Aponevrosis (1%) | Human food | 0.0075 | 0.0071 | 0.0050 |
| Bile | PAP C3 | 0.0001 | 0.0004 | 0.0000 |
| Blood | C1-C2 for disposal | 0.0000 | 0.0000 | 0.0000 |
| Bones (11%) | Gelatin C3 | 0.0790 | 0.0777 | 0.0002 |
| Dead individuals | C1-C2 for disposal | 0.0000 | 0.0000 | 0.0000 |
| Fat (8%) | Fat and greaves C3 | 0.1553 | 0.0565 | 0.0036 |
| Fat from breasts and penis | Fat and greaves C3 | 0.0418 | 0.0152 | 0.0010 |
| Feet (without hooves) | Human food | 0.0183 | 0.0203 | 0.0000 |
| Floatation fat | C1-C2 for disposal | 0.0000 | 0.0000 | 0.0000 |
| Head | Human food | 0.0454 | 0.0447 | 0.0718 |
| Intestines | C1-C2 for disposal | 0.0000 | 0.0000 | 0.0000 |
| Kidney | Human food | 0.0029 | 0.0034 | 0.0025 |
| Manure | Spreading/Compost | 0.0000 | 0.0000 | 0.0000 |
| Meat | Human food | 0.4944 | 0.5654 | 0.8273 |
| Pluck | Human food | 0.0430 | 0.0452 | 0.0135 |
| Rumen and forestomach | Human food | 0.0043 | 0.0048 | 0.0002 |
| SPA C3 | PAP C3 | 0.0027 | 0.0703 | 0.0043 |
| Screening and sifting wastes | C1-C2 for disposal | 0.0000 | 0.0000 | 0.0000 |
| Skin | Skin tannery C3 | 0.0966 | 0.0799 | 0.0684 |
| Sludge | Spreading/Compost | 0.0000 | 0.0000 | 0.0000 |
| Spleen | Pet food | 0.0021 | 0.0023 | 0.0001 |
| Sweetbread | Human food | 0.0017 | 0.0016 | 0.0018 |

Table 10: Total weighting by coproducts for Aubrac Rosé Calf reared in Grazing Large Area

| COPRODUCT | Destination | Aubrac /rosé calf/grazing large area | | |
| --- | --- | --- | --- | --- |
|  |  | **Biophysical Cumulative Share** | **Mass Cumulative Share** | **Economic Cumulative Share** |
| Abomasum | Human food | 0.0047 | 0.0051 | 0.0002 |
| Aponevrosis (1%) | Human food | 0.0077 | 0.0071 | 0.0050 |
| Bile | PAP C3 | 0.0001 | 0.0004 | 0.0000 |
| Blood | C1-C2 for disposal | 0.0000 | 0.0000 | 0.0000 |
| Bones (11%) | Gelatin C3 | 0.0817 | 0.0777 | 0.0002 |
| Dead individuals | C1-C2 for disposal | 0.0000 | 0.0000 | 0.0000 |
| Fat (8%) | Fat and greaves C3 | 0.1403 | 0.0565 | 0.0036 |
| Fat from breasts and penis | Fat and greaves C3 | 0.0378 | 0.0152 | 0.0010 |
| Feet (without hooves) | Human food | 0.0186 | 0.0203 | 0.0000 |
| Floatation fat | C1-C2 for disposal | 0.0000 | 0.0000 | 0.0000 |
| Head | Human food | 0.0463 | 0.0447 | 0.0718 |
| Intestines | C1-C2 for disposal | 0.0000 | 0.0000 | 0.0000 |
| Kidney | Human food | 0.0030 | 0.0034 | 0.0025 |
| Manure | Spreading/Compost | 0.0000 | 0.0000 | 0.0000 |
| Meat | Human food | 0.5047 | 0.5654 | 0.8273 |
| Pluck | Human food | 0.0440 | 0.0452 | 0.0135 |
| Rumen and forestomach | Human food | 0.0043 | 0.0048 | 0.0002 |
| SPA C3 | PAP C3 | 0.0028 | 0.0703 | 0.0043 |
| Screening and sifting wastes | C1-C2 for disposal | 0.0000 | 0.0000 | 0.0000 |
| Skin | Skin tannery C3 | 0.1000 | 0.0799 | 0.0684 |
| Sludge | Spreading/Compost | 0.0000 | 0.0000 | 0.0000 |
| Spleen | Pet food | 0.0021 | 0.0023 | 0.0001 |
| Sweetbread | Human food | 0.0018 | 0.0016 | 0.0018 |

Table 11: Total weighting by coproducts for Aubrac Rosé Calf reared in Pasture

| COPRODUCT | Destination | Aubrac /Rosé calf/Pasture | | |
| --- | --- | --- | --- | --- |
|  |  | **Biophysical Cumulative Share** | **Mass Cumulative Share** | **Economic Cumulative Share** |
| Abomasum | Human food | 0.0047 | 0.0051 | 0.0002 |
| Aponevrosis (1%) | Human food | 0.0077 | 0.0071 | 0.0050 |
| Bile | PAP C3 | 0.0001 | 0.0004 | 0.0000 |
| Blood | C1-C2 for disposal | 0.0000 | 0.0000 | 0.0000 |
| Bones (11%) | Gelatin C3 | 0.0807 | 0.0777 | 0.0002 |
| Dead individuals | C1-C2 for disposal | 0.0000 | 0.0000 | 0.0000 |
| Fat (8%) | Fat and greaves C3 | 0.1454 | 0.0565 | 0.0036 |
| Fat from breasts and penis | Fat and greaves C3 | 0.0392 | 0.0152 | 0.0010 |
| Feet (without hooves) | Human food | 0.0185 | 0.0203 | 0.0000 |
| Floatation fat | C1-C2 for disposal | 0.0000 | 0.0000 | 0.0000 |
| Head | Human food | 0.0460 | 0.0447 | 0.0718 |
| Intestines | C1-C2 for disposal | 0.0000 | 0.0000 | 0.0000 |
| Kidney | Human food | 0.0030 | 0.0034 | 0.0025 |
| Manure | Spreading/Compost | 0.0000 | 0.0000 | 0.0000 |
| Meat | Human food | 0.5012 | 0.5654 | 0.8273 |
| Pluck | Human food | 0.0437 | 0.0452 | 0.0135 |
| Rumen and forestomach | Human food | 0.0043 | 0.0048 | 0.0002 |
| SPA C3 | PAP C3 | 0.0028 | 0.0703 | 0.0043 |
| Screening and sifting wastes | C1-C2 for disposal | 0.0000 | 0.0000 | 0.0000 |
| Skin | Skin tannery C3 | 0.0988 | 0.0799 | 0.0684 |
| Sludge | Spreading/Compost | 0.0000 | 0.0000 | 0.0000 |
| Spleen | Pet food | 0.0021 | 0.0023 | 0.0001 |
| Sweetbread | Human food | 0.0018 | 0.0016 | 0.0018 |

Table 12: Total weighting by coproducts for Aubrac Rosé Calf reared in Stall

| COPRODUCT | Destination | Aubrac /Rosé calf/Stall | | |
| --- | --- | --- | --- | --- |
|  |  | **Biophysical Cumulative Share** | **Mass Cumulative Share** | **Economic Cumulative Share** |
| Abomasum | Human food | 0.0046 | 0.0051 | 0.0002 |
| Aponevrosis (1%) | Human food | 0.0076 | 0.0071 | 0.0050 |
| Bile | PAP C3 | 0.0001 | 0.0004 | 0.0000 |
| Blood | C1-C2 for disposal | 0.0000 | 0.0000 | 0.0000 |
| Bones (11%) | Gelatin C3 | 0.0798 | 0.0777 | 0.0002 |
| Dead individuals | C1-C2 for disposal | 0.0000 | 0.0000 | 0.0000 |
| Fat (8%) | Fat and greaves C3 | 0.1507 | 0.0565 | 0.0036 |
| Fat from breasts and penis | Fat and greaves C3 | 0.0406 | 0.0152 | 0.0010 |
| Feet (without hooves) | Human food | 0.0184 | 0.0203 | 0.0000 |
| Floatation fat | C1-C2 for disposal | 0.0000 | 0.0000 | 0.0000 |
| Head | Human food | 0.0457 | 0.0447 | 0.0718 |
| Intestines | C1-C2 for disposal | 0.0000 | 0.0000 | 0.0000 |
| Kidney | Human food | 0.0030 | 0.0034 | 0.0025 |
| Manure | Spreading/Compost | 0.0000 | 0.0000 | 0.0000 |
| Meat | Human food | 0.4977 | 0.5654 | 0.8273 |
| Pluck | Human food | 0.0433 | 0.0452 | 0.0135 |
| Rumen and forestomach | Human food | 0.0043 | 0.0048 | 0.0002 |
| SPA C3 | PAP C3 | 0.0027 | 0.0703 | 0.0043 |
| Screening and sifting wastes | C1-C2 for disposal | 0.0000 | 0.0000 | 0.0000 |
| Skin | Skin tannery C3 | 0.0976 | 0.0799 | 0.0684 |
| Sludge | Spreading/Compost | 0.0000 | 0.0000 | 0.0000 |
| Spleen | Pet food | 0.0021 | 0.0023 | 0.0001 |
| Sweetbread | Human food | 0.0017 | 0.0016 | 0.0018 |

Table 13: Total weighting by coproducts for Blonde d’Aquitaine Milk-fed Calf reared in Grazing Large Area

| COPRODUCT | Destination | Blonde d’Aquitaine /milk-fed calf/grazing large area | | |
| --- | --- | --- | --- | --- |
|  |  | **Biophysical Cumulative Share** | **Mass Cumulative Share** | **Economic Cumulative Share** |
| Abomasum | Human food | 0.0046 | 0.0051 | 0.0002 |
| Aponevrosis (1%) | Human food | 0.0077 | 0.0071 | 0.0050 |
| Bile | PAP C3 | 0.0001 | 0.0004 | 0.0000 |
| Blood | C1-C2 for disposal | 0.0000 | 0.0000 | 0.0000 |
| Bones (11%) | Gelatin C3 | 0.0806 | 0.0777 | 0.0002 |
| Dead individuals | C1-C2 for disposal | 0.0000 | 0.0000 | 0.0000 |
| Fat (8%) | Fat and greaves C3 | 0.1467 | 0.0565 | 0.0036 |
| Fat from breasts and penis | Fat and greaves C3 | 0.0395 | 0.0152 | 0.0010 |
| Feet (without hooves) | Human food | 0.0185 | 0.0203 | 0.0000 |
| Floatation fat | C1-C2 for disposal | 0.0000 | 0.0000 | 0.0000 |
| Head | Human food | 0.0459 | 0.0447 | 0.0718 |
| Intestines | C1-C2 for disposal | 0.0000 | 0.0000 | 0.0000 |
| Kidney | Human food | 0.0030 | 0.0034 | 0.0025 |
| Manure | Spreading/Compost | 0.0000 | 0.0000 | 0.0000 |
| Meat | Human food | 0.5002 | 0.5654 | 0.8273 |
| Pluck | Human food | 0.0436 | 0.0452 | 0.0135 |
| Rumen and forestomach | Human food | 0.0043 | 0.0048 | 0.0002 |
| SPA C3 | PAP C3 | 0.0028 | 0.0703 | 0.0043 |
| Screening and sifting wastes | C1-C2 for disposal | 0.0000 | 0.0000 | 0.0000 |
| Skin | Skin tannery C3 | 0.0987 | 0.0799 | 0.0684 |
| Sludge | Spreading/Compost | 0.0000 | 0.0000 | 0.0000 |
| Spleen | Pet food | 0.0021 | 0.0023 | 0.0001 |
| Sweetbread | Human food | 0.0018 | 0.0016 | 0.0018 |

Table 14: Total weighting by coproducts for Blonde d’Aquitaine Milk-fed Calf reared in Pasture

| COPRODUCT | Destination | Blonde d’Aquitaine /milk-fed calf/PASTURE | | |
| --- | --- | --- | --- | --- |
|  |  | **Biophysical Cumulative Share** | **Mass Cumulative Share** | **Economic Cumulative Share** |
| Abomasum | Human food | 0.0046 | 0.0051 | 0.0002 |
| Aponevrosis (1%) | Human food | 0.0076 | 0.0071 | 0.0050 |
| Bile | PAP C3 | 0.0001 | 0.0004 | 0.0000 |
| Blood | C1-C2 for disposal | 0.0000 | 0.0000 | 0.0000 |
| Bones (11%) | Gelatin C3 | 0.0796 | 0.0777 | 0.0002 |
| Dead individuals | C1-C2 for disposal | 0.0000 | 0.0000 | 0.0000 |
| Fat (8%) | Fat and greaves C3 | 0.1524 | 0.0565 | 0.0036 |
| Fat from breasts and penis | Fat and greaves C3 | 0.0411 | 0.0152 | 0.0010 |
| Feet (without hooves) | Human food | 0.0184 | 0.0203 | 0.0000 |
| Floatation fat | C1-C2 for disposal | 0.0000 | 0.0000 | 0.0000 |
| Head | Human food | 0.0456 | 0.0447 | 0.0718 |
| Intestines | C1-C2 for disposal | 0.0000 | 0.0000 | 0.0000 |
| Kidney | Human food | 0.0030 | 0.0034 | 0.0025 |
| Manure | Spreading/Compost | 0.0000 | 0.0000 | 0.0000 |
| Meat | Human food | 0.4963 | 0.5654 | 0.8273 |
| Pluck | Human food | 0.0432 | 0.0452 | 0.0135 |
| Rumen and forestomach | Human food | 0.0043 | 0.0048 | 0.0002 |
| SPA C3 | PAP C3 | 0.0027 | 0.0703 | 0.0043 |
| Screening and sifting wastes | C1-C2 for disposal | 0.0000 | 0.0000 | 0.0000 |
| Skin | Skin tannery C3 | 0.0974 | 0.0799 | 0.0684 |
| Sludge | Spreading/Compost | 0.0000 | 0.0000 | 0.0000 |
| Spleen | Pet food | 0.0021 | 0.0023 | 0.0001 |
| Sweetbread | Human food | 0.0017 | 0.0016 | 0.0018 |

Table 15: Total weighting by coproducts for Blonde d’Aquitaine Milk-fed Calf reared in Stall

| COPRODUCT | Destination | Blonde d’Aquitaine /Milk-fed calf/Stall | | |
| --- | --- | --- | --- | --- |
|  |  | **Biophysical Cumulative Share** | **Mass Cumulative Share** | **Economic Cumulative Share** |
| Abomasum | Human food | 0.0046 | 0.0051 | 0.0002 |
| Aponevrosis (1%) | Human food | 0.0075 | 0.0071 | 0.0050 |
| Bile | PAP C3 | 0.0001 | 0.0004 | 0.0000 |
| Blood | C1-C2 for disposal | 0.0000 | 0.0000 | 0.0000 |
| Bones (11%) | Gelatin C3 | 0.0785 | 0.0777 | 0.0002 |
| Dead individuals | C1-C2 for disposal | 0.0000 | 0.0000 | 0.0000 |
| Fat (8%) | Fat and greaves C3 | 0.1583 | 0.0565 | 0.0036 |
| Fat from breasts and penis | Fat and greaves C3 | 0.0426 | 0.0152 | 0.0010 |
| Feet (without hooves) | Human food | 0.0183 | 0.0203 | 0.0000 |
| Floatation fat | C1-C2 for disposal | 0.0000 | 0.0000 | 0.0000 |
| Head | Human food | 0.0452 | 0.0447 | 0.0718 |
| Intestines | C1-C2 for disposal | 0.0000 | 0.0000 | 0.0000 |
| Kidney | Human food | 0.0029 | 0.0034 | 0.0025 |
| Manure | Spreading/Compost | 0.0000 | 0.0000 | 0.0000 |
| Meat | Human food | 0.4923 | 0.5654 | 0.8273 |
| Pluck | Human food | 0.0428 | 0.0452 | 0.0135 |
| Rumen and forestomach | Human food | 0.0043 | 0.0048 | 0.0002 |
| SPA C3 | PAP C3 | 0.0027 | 0.0703 | 0.0043 |
| Screening and sifting wastes | C1-C2 for disposal | 0.0000 | 0.0000 | 0.0000 |
| Skin | Skin tannery C3 | 0.0960 | 0.0799 | 0.0684 |
| Sludge | Spreading/Compost | 0.0000 | 0.0000 | 0.0000 |
| Spleen | Pet food | 0.0021 | 0.0023 | 0.0001 |
| Sweetbread | Human food | 0.0017 | 0.0016 | 0.0018 |

Table 16: Total weighting by coproducts for Blonde d’Aquitaine Rosé Calf reared in Grazing Large Area

| COPRODUCT | Destination | Blonde d’Aquitaine /rosé calf/grazing large area | | |
| --- | --- | --- | --- | --- |
|  |  | **Biophysical Cumulative Share** | **Mass Cumulative Share** | **Economic Cumulative Share** |
| Abomasum | Human food | 0.0047 | 0.0051 | 0.0002 |
| Aponevrosis (1%) | Human food | 0.0077 | 0.0071 | 0.0050 |
| Bile | PAP C3 | 0.0001 | 0.0004 | 0.0000 |
| Blood | C1-C2 for disposal | 0.0000 | 0.0000 | 0.0000 |
| Bones (11%) | Gelatin C3 | 0.0813 | 0.0777 | 0.0002 |
| Dead individuals | C1-C2 for disposal | 0.0000 | 0.0000 | 0.0000 |
| Fat (8%) | Fat and greaves C3 | 0.1426 | 0.0565 | 0.0036 |
| Fat from breasts and penis | Fat and greaves C3 | 0.0384 | 0.0152 | 0.0010 |
| Feet (without hooves) | Human food | 0.0186 | 0.0203 | 0.0000 |
| Floatation fat | C1-C2 for disposal | 0.0000 | 0.0000 | 0.0000 |
| Head | Human food | 0.0462 | 0.0447 | 0.0718 |
| Intestines | C1-C2 for disposal | 0.0000 | 0.0000 | 0.0000 |
| Kidney | Human food | 0.0030 | 0.0034 | 0.0025 |
| Manure | Spreading/Compost | 0.0000 | 0.0000 | 0.0000 |
| Meat | Human food | 0.5030 | 0.5654 | 0.8273 |
| Pluck | Human food | 0.0438 | 0.0452 | 0.0135 |
| Rumen and forestomach | Human food | 0.0043 | 0.0048 | 0.0002 |
| SPA C3 | PAP C3 | 0.0028 | 0.0703 | 0.0043 |
| Screening and sifting wastes | C1-C2 for disposal | 0.0000 | 0.0000 | 0.0000 |
| Skin | Skin tannery C3 | 0.0996 | 0.0799 | 0.0684 |
| Sludge | Spreading/Compost | 0.0000 | 0.0000 | 0.0000 |
| Spleen | Pet food | 0.0021 | 0.0023 | 0.0001 |
| Sweetbread | Human food | 0.0018 | 0.0016 | 0.0018 |

Table 17: Total weighting by coproducts for Blonde d’Aquitaine Rosé Calf reared in Pasture

| COPRODUCT | Destination | Blonde d’Aquitaine /Rosé calf/Pasture | | |
| --- | --- | --- | --- | --- |
|  |  | **Biophysical Cumulative Share** | **Mass Cumulative Share** | **Economic Cumulative Share** |
| Abomasum | Human food | 0.0046 | 0.0051 | 0.0002 |
| Aponevrosis (1%) | Human food | 0.0076 | 0.0071 | 0.0050 |
| Bile | PAP C3 | 0.0001 | 0.0004 | 0.0000 |
| Blood | C1-C2 for disposal | 0.0000 | 0.0000 | 0.0000 |
| Bones (11%) | Gelatin C3 | 0.0803 | 0.0777 | 0.0002 |
| Dead individuals | C1-C2 for disposal | 0.0000 | 0.0000 | 0.0000 |
| Fat (8%) | Fat and greaves C3 | 0.1479 | 0.0565 | 0.0036 |
| Fat from breasts and penis | Fat and greaves C3 | 0.0399 | 0.0152 | 0.0010 |
| Feet (without hooves) | Human food | 0.0185 | 0.0203 | 0.0000 |
| Floatation fat | C1-C2 for disposal | 0.0000 | 0.0000 | 0.0000 |
| Head | Human food | 0.0459 | 0.0447 | 0.0718 |
| Intestines | C1-C2 for disposal | 0.0000 | 0.0000 | 0.0000 |
| Kidney | Human food | 0.0030 | 0.0034 | 0.0025 |
| Manure | Spreading/Compost | 0.0000 | 0.0000 | 0.0000 |
| Meat | Human food | 0.4994 | 0.5654 | 0.8273 |
| Pluck | Human food | 0.0435 | 0.0452 | 0.0135 |
| Rumen and forestomach | Human food | 0.0043 | 0.0048 | 0.0002 |
| SPA C3 | PAP C3 | 0.0028 | 0.0703 | 0.0043 |
| Screening and sifting wastes | C1-C2 for disposal | 0.0000 | 0.0000 | 0.0000 |
| Skin | Skin tannery C3 | 0.0983 | 0.0799 | 0.0684 |
| Sludge | Spreading/Compost | 0.0000 | 0.0000 | 0.0000 |
| Spleen | Pet food | 0.0021 | 0.0023 | 0.0001 |
| Sweetbread | Human food | 0.0017 | 0.0016 | 0.0018 |

Table 18: Total weighting by coproducts for Blonde d’Aquitaine Rosé Calf reared in Stall

| COPRODUCT | Destination | Blonde d’Aquitaine /Rosé calf/Stall | | |
| --- | --- | --- | --- | --- |
|  |  | **Biophysical Cumulative Share** | **Mass Cumulative Share** | **Economic Cumulative Share** |
| Abomasum | Human food | 0.0046 | 0.0051 | 0.0002 |
| Aponevrosis (1%) | Human food | 0.0076 | 0.0071 | 0.0050 |
| Bile | PAP C3 | 0.0001 | 0.0004 | 0.0000 |
| Blood | C1-C2 for disposal | 0.0000 | 0.0000 | 0.0000 |
| Bones (11%) | Gelatin C3 | 0.0793 | 0.0777 | 0.0002 |
| Dead individuals | C1-C2 for disposal | 0.0000 | 0.0000 | 0.0000 |
| Fat (8%) | Fat and greaves C3 | 0.1534 | 0.0565 | 0.0036 |
| Fat from breasts and penis | Fat and greaves C3 | 0.0413 | 0.0152 | 0.0010 |
| Feet (without hooves) | Human food | 0.0184 | 0.0203 | 0.0000 |
| Floatation fat | C1-C2 for disposal | 0.0000 | 0.0000 | 0.0000 |
| Head | Human food | 0.0455 | 0.0447 | 0.0718 |
| Intestines | C1-C2 for disposal | 0.0000 | 0.0000 | 0.0000 |
| Kidney | Human food | 0.0030 | 0.0034 | 0.0025 |
| Manure | Spreading/Compost | 0.0000 | 0.0000 | 0.0000 |
| Meat | Human food | 0.4958 | 0.5654 | 0.8273 |
| Pluck | Human food | 0.0431 | 0.0452 | 0.0135 |
| Rumen and forestomach | Human food | 0.0043 | 0.0048 | 0.0002 |
| SPA C3 | PAP C3 | 0.0027 | 0.0703 | 0.0043 |
| Screening and sifting wastes | C1-C2 for disposal | 0.0000 | 0.0000 | 0.0000 |
| Skin | Skin tannery C3 | 0.0970 | 0.0799 | 0.0684 |
| Sludge | Spreading/Compost | 0.0000 | 0.0000 | 0.0000 |
| Spleen | Pet food | 0.0021 | 0.0023 | 0.0001 |
| Sweetbread | Human food | 0.0017 | 0.0016 | 0.0018 |

Table 19: Total weighting by coproducts for Charolais Milk-fed Calf reared in Grazing Large Area

| COPRODUCT | Destination | Charolais /milk-fed calf/grazing large area | | |
| --- | --- | --- | --- | --- |
|  |  | **Biophysical Cumulative Share** | **Mass Cumulative Share** | **Economic Cumulative Share** |
| Abomasum | Human food | 0.0047 | 0.0051 | 0.0002 |
| Aponevrosis (1%) | Human food | 0.0077 | 0.0071 | 0.0050 |
| Bile | PAP C3 | 0.0001 | 0.0004 | 0.0000 |
| Blood | C1-C2 for disposal | 0.0000 | 0.0000 | 0.0000 |
| Bones (11%) | Gelatin C3 | 0.0812 | 0.0777 | 0.0002 |
| Dead individuals | C1-C2 for disposal | 0.0000 | 0.0000 | 0.0000 |
| Fat (8%) | Fat and greaves C3 | 0.1431 | 0.0565 | 0.0036 |
| Fat from breasts and penis | Fat and greaves C3 | 0.0385 | 0.0152 | 0.0010 |
| Feet (without hooves) | Human food | 0.0185 | 0.0203 | 0.0000 |
| Floatation fat | C1-C2 for disposal | 0.0000 | 0.0000 | 0.0000 |
| Head | Human food | 0.0461 | 0.0447 | 0.0718 |
| Intestines | C1-C2 for disposal | 0.0000 | 0.0000 | 0.0000 |
| Kidney | Human food | 0.0030 | 0.0034 | 0.0025 |
| Manure | Spreading/Compost | 0.0000 | 0.0000 | 0.0000 |
| Meat | Human food | 0.5027 | 0.5654 | 0.8273 |
| Pluck | Human food | 0.0438 | 0.0452 | 0.0135 |
| Rumen and forestomach | Human food | 0.0043 | 0.0048 | 0.0002 |
| SPA C3 | PAP C3 | 0.0028 | 0.0703 | 0.0043 |
| Screening and sifting wastes | C1-C2 for disposal | 0.0000 | 0.0000 | 0.0000 |
| Skin | Skin tannery C3 | 0.0995 | 0.0799 | 0.0684 |
| Sludge | Spreading/Compost | 0.0000 | 0.0000 | 0.0000 |
| Spleen | Pet food | 0.0021 | 0.0023 | 0.0001 |
| Sweetbread | Human food | 0.0018 | 0.0016 | 0.0018 |

Table 20: Total weighting by coproducts for Charolais Milk-fed Calf reared in Pasture

| COPRODUCT | Destination | Charolais /milk-fed calf/PASTURE | | |
| --- | --- | --- | --- | --- |
|  |  | **Biophysical Cumulative Share** | **Mass Cumulative Share** | **Economic Cumulative Share** |
| Abomasum | Human food | 0.0046 | 0.0051 | 0.0002 |
| Aponevrosis (1%) | Human food | 0.0076 | 0.0071 | 0.0050 |
| Bile | PAP C3 | 0.0001 | 0.0004 | 0.0000 |
| Blood | C1-C2 for disposal | 0.0000 | 0.0000 | 0.0000 |
| Bones (11%) | Gelatin C3 | 0.0802 | 0.0777 | 0.0002 |
| Dead individuals | C1-C2 for disposal | 0.0000 | 0.0000 | 0.0000 |
| Fat (8%) | Fat and greaves C3 | 0.1485 | 0.0565 | 0.0036 |
| Fat from breasts and penis | Fat and greaves C3 | 0.0400 | 0.0152 | 0.0010 |
| Feet (without hooves) | Human food | 0.0184 | 0.0203 | 0.0000 |
| Floatation fat | C1-C2 for disposal | 0.0000 | 0.0000 | 0.0000 |
| Head | Human food | 0.0458 | 0.0447 | 0.0718 |
| Intestines | C1-C2 for disposal | 0.0000 | 0.0000 | 0.0000 |
| Kidney | Human food | 0.0030 | 0.0034 | 0.0025 |
| Manure | Spreading/Compost | 0.0000 | 0.0000 | 0.0000 |
| Meat | Human food | 0.4991 | 0.5654 | 0.8273 |
| Pluck | Human food | 0.0435 | 0.0452 | 0.0135 |
| Rumen and forestomach | Human food | 0.0043 | 0.0048 | 0.0002 |
| SPA C3 | PAP C3 | 0.0028 | 0.0703 | 0.0043 |
| Screening and sifting wastes | C1-C2 for disposal | 0.0000 | 0.0000 | 0.0000 |
| Skin | Skin tannery C3 | 0.0982 | 0.0799 | 0.0684 |
| Sludge | Spreading/Compost | 0.0000 | 0.0000 | 0.0000 |
| Spleen | Pet food | 0.0021 | 0.0023 | 0.0001 |
| Sweetbread | Human food | 0.0017 | 0.0016 | 0.0018 |

Table 21: Total weighting by coproducts for Charolais Milk-fed Calf reared in Stall

| COPRODUCT | Destination | Charolais /Milk-fed calf/Stall | | |
| --- | --- | --- | --- | --- |
|  |  | **Biophysical Cumulative Share** | **Mass Cumulative Share** | **Economic Cumulative Share** |
| Abomasum | Human food | 0.0046 | 0.0051 | 0.0002 |
| Aponevrosis (1%) | Human food | 0.0076 | 0.0071 | 0.0050 |
| Bile | PAP C3 | 0.0001 | 0.0004 | 0.0000 |
| Blood | C1-C2 for disposal | 0.0000 | 0.0000 | 0.0000 |
| Bones (11%) | Gelatin C3 | 0.0792 | 0.0777 | 0.0002 |
| Dead individuals | C1-C2 for disposal | 0.0000 | 0.0000 | 0.0000 |
| Fat (8%) | Fat and greaves C3 | 0.1540 | 0.0565 | 0.0036 |
| Fat from breasts and penis | Fat and greaves C3 | 0.0415 | 0.0152 | 0.0010 |
| Feet (without hooves) | Human food | 0.0183 | 0.0203 | 0.0000 |
| Floatation fat | C1-C2 for disposal | 0.0000 | 0.0000 | 0.0000 |
| Head | Human food | 0.0455 | 0.0447 | 0.0718 |
| Intestines | C1-C2 for disposal | 0.0000 | 0.0000 | 0.0000 |
| Kidney | Human food | 0.0029 | 0.0034 | 0.0025 |
| Manure | Spreading/Compost | 0.0000 | 0.0000 | 0.0000 |
| Meat | Human food | 0.4954 | 0.5654 | 0.8273 |
| Pluck | Human food | 0.0431 | 0.0452 | 0.0135 |
| Rumen and forestomach | Human food | 0.0043 | 0.0048 | 0.0002 |
| SPA C3 | PAP C3 | 0.0027 | 0.0703 | 0.0043 |
| Screening and sifting wastes | C1-C2 for disposal | 0.0000 | 0.0000 | 0.0000 |
| Skin | Skin tannery C3 | 0.0969 | 0.0799 | 0.0684 |
| Sludge | Spreading/Compost | 0.0000 | 0.0000 | 0.0000 |
| Spleen | Pet food | 0.0021 | 0.0023 | 0.0001 |
| Sweetbread | Human food | 0.0017 | 0.0016 | 0.0018 |

Table 22: Total weighting by coproducts for Charolais Rosé Calf reared in Grazing Large Area

| COPRODUCT | Destination | Charolais /rosé calf/grazing large area | | |
| --- | --- | --- | --- | --- |
|  |  | **Biophysical Cumulative Share** | **Mass Cumulative Share** | **Economic Cumulative Share** |
| Abomasum | Human food | 0.0047 | 0.0051 | 0.0002 |
| Aponevrosis (1%) | Human food | 0.0077 | 0.0071 | 0.0050 |
| Bile | PAP C3 | 0.0001 | 0.0004 | 0.0000 |
| Blood | C1-C2 for disposal | 0.0000 | 0.0000 | 0.0000 |
| Bones (11%) | Gelatin C3 | 0.0818 | 0.0777 | 0.0002 |
| Dead individuals | C1-C2 for disposal | 0.0000 | 0.0000 | 0.0000 |
| Fat (8%) | Fat and greaves C3 | 0.1392 | 0.0565 | 0.0036 |
| Fat from breasts and penis | Fat and greaves C3 | 0.0375 | 0.0152 | 0.0010 |
| Feet (without hooves) | Human food | 0.0186 | 0.0203 | 0.0000 |
| Floatation fat | C1-C2 for disposal | 0.0000 | 0.0000 | 0.0000 |
| Head | Human food | 0.0464 | 0.0447 | 0.0718 |
| Intestines | C1-C2 for disposal | 0.0000 | 0.0000 | 0.0000 |
| Kidney | Human food | 0.0030 | 0.0034 | 0.0025 |
| Manure | Spreading/Compost | 0.0000 | 0.0000 | 0.0000 |
| Meat | Human food | 0.5055 | 0.5654 | 0.8273 |
| Pluck | Human food | 0.0441 | 0.0452 | 0.0135 |
| Rumen and forestomach | Human food | 0.0044 | 0.0048 | 0.0002 |
| SPA C3 | PAP C3 | 0.0028 | 0.0703 | 0.0043 |
| Screening and sifting wastes | C1-C2 for disposal | 0.0000 | 0.0000 | 0.0000 |
| Skin | Skin tannery C3 | 0.1003 | 0.0799 | 0.0684 |
| Sludge | Spreading/Compost | 0.0000 | 0.0000 | 0.0000 |
| Spleen | Pet food | 0.0021 | 0.0023 | 0.0001 |
| Sweetbread | Human food | 0.0018 | 0.0016 | 0.0018 |

Table 23: Total weighting by coproducts for Charolais Rosé Calf reared in Pasture

| COPRODUCT | Destination | Charolais /Rosé calf/Pasture | | |
| --- | --- | --- | --- | --- |
|  |  | **Biophysical Cumulative Share** | **Mass Cumulative Share** | **Economic Cumulative Share** |
| Abomasum | Human food | 0.0047 | 0.0051 | 0.0002 |
| Aponevrosis (1%) | Human food | 0.0077 | 0.0071 | 0.0050 |
| Bile | PAP C3 | 0.0001 | 0.0004 | 0.0000 |
| Blood | C1-C2 for disposal | 0.0000 | 0.0000 | 0.0000 |
| Bones (11%) | Gelatin C3 | 0.0809 | 0.0777 | 0.0002 |
| Dead individuals | C1-C2 for disposal | 0.0000 | 0.0000 | 0.0000 |
| Fat (8%) | Fat and greaves C3 | 0.1442 | 0.0565 | 0.0036 |
| Fat from breasts and penis | Fat and greaves C3 | 0.0388 | 0.0152 | 0.0010 |
| Feet (without hooves) | Human food | 0.0185 | 0.0203 | 0.0000 |
| Floatation fat | C1-C2 for disposal | 0.0000 | 0.0000 | 0.0000 |
| Head | Human food | 0.0461 | 0.0447 | 0.0718 |
| Intestines | C1-C2 for disposal | 0.0000 | 0.0000 | 0.0000 |
| Kidney | Human food | 0.0030 | 0.0034 | 0.0025 |
| Manure | Spreading/Compost | 0.0000 | 0.0000 | 0.0000 |
| Meat | Human food | 0.5021 | 0.5654 | 0.8273 |
| Pluck | Human food | 0.0437 | 0.0452 | 0.0135 |
| Rumen and forestomach | Human food | 0.0043 | 0.0048 | 0.0002 |
| SPA C3 | PAP C3 | 0.0028 | 0.0703 | 0.0043 |
| Screening and sifting wastes | C1-C2 for disposal | 0.0000 | 0.0000 | 0.0000 |
| Skin | Skin tannery C3 | 0.0991 | 0.0799 | 0.0684 |
| Sludge | Spreading/Compost | 0.0000 | 0.0000 | 0.0000 |
| Spleen | Pet food | 0.0021 | 0.0023 | 0.0001 |
| Sweetbread | Human food | 0.0018 | 0.0016 | 0.0018 |

Table 24: Total weighting by coproducts for Charolais Rosé Calf reared in Stall

| COPRODUCT | Destination | Charolais /Rosé calf/Stall | | |
| --- | --- | --- | --- | --- |
|  |  | **Biophysical Cumulative Share** | **Mass Cumulative Share** | **Economic Cumulative Share** |
| Abomasum | Human food | 0.0046 | 0.0051 | 0.0002 |
| Aponevrosis (1%) | Human food | 0.0076 | 0.0071 | 0.0050 |
| Bile | PAP C3 | 0.0001 | 0.0004 | 0.0000 |
| Blood | C1-C2 for disposal | 0.0000 | 0.0000 | 0.0000 |
| Bones (11%) | Gelatin C3 | 0.0800 | 0.0777 | 0.0002 |
| Dead individuals | C1-C2 for disposal | 0.0000 | 0.0000 | 0.0000 |
| Fat (8%) | Fat and greaves C3 | 0.1493 | 0.0565 | 0.0036 |
| Fat from breasts and penis | Fat and greaves C3 | 0.0402 | 0.0152 | 0.0010 |
| Feet (without hooves) | Human food | 0.0184 | 0.0203 | 0.0000 |
| Floatation fat | C1-C2 for disposal | 0.0000 | 0.0000 | 0.0000 |
| Head | Human food | 0.0458 | 0.0447 | 0.0718 |
| Intestines | C1-C2 for disposal | 0.0000 | 0.0000 | 0.0000 |
| Kidney | Human food | 0.0030 | 0.0034 | 0.0025 |
| Manure | Spreading/Compost | 0.0000 | 0.0000 | 0.0000 |
| Meat | Human food | 0.4987 | 0.5654 | 0.8273 |
| Pluck | Human food | 0.0434 | 0.0452 | 0.0135 |
| Rumen and forestomach | Human food | 0.0043 | 0.0048 | 0.0002 |
| SPA C3 | PAP C3 | 0.0028 | 0.0703 | 0.0043 |
| Screening and sifting wastes | C1-C2 for disposal | 0.0000 | 0.0000 | 0.0000 |
| Skin | Skin tannery C3 | 0.0979 | 0.0799 | 0.0684 |
| Sludge | Spreading/Compost | 0.0000 | 0.0000 | 0.0000 |
| Spleen | Pet food | 0.0021 | 0.0023 | 0.0001 |
| Sweetbread | Human food | 0.0017 | 0.0016 | 0.0018 |

Table 25: Total weighting by coproducts for Limousine Milk-fed Calf reared in Grazing Large Area

| COPRODUCT | Destination | Limousine /milk-fed calf/grazing large area | | |
| --- | --- | --- | --- | --- |
|  |  | **Biophysical Cumulative Share** | **Mass Cumulative Share** | **Economic Cumulative Share** |
| Abomasum | Human food | 0.0047 | 0.0051 | 0.0002 |
| Aponevrosis (1%) | Human food | 0.0077 | 0.0071 | 0.0050 |
| Bile | PAP C3 | 0.0001 | 0.0004 | 0.0000 |
| Blood | C1-C2 for disposal | 0.0000 | 0.0000 | 0.0000 |
| Bones (11%) | Gelatin C3 | 0.0809 | 0.0777 | 0.0002 |
| Dead individuals | C1-C2 for disposal | 0.0000 | 0.0000 | 0.0000 |
| Fat (8%) | Fat and greaves C3 | 0.1451 | 0.0565 | 0.0036 |
| Fat from breasts and penis | Fat and greaves C3 | 0.0391 | 0.0152 | 0.0010 |
| Feet (without hooves) | Human food | 0.0185 | 0.0203 | 0.0000 |
| Floatation fat | C1-C2 for disposal | 0.0000 | 0.0000 | 0.0000 |
| Head | Human food | 0.0460 | 0.0447 | 0.0718 |
| Intestines | C1-C2 for disposal | 0.0000 | 0.0000 | 0.0000 |
| Kidney | Human food | 0.0030 | 0.0034 | 0.0025 |
| Manure | Spreading/Compost | 0.0000 | 0.0000 | 0.0000 |
| Meat | Human food | 0.5012 | 0.5654 | 0.8273 |
| Pluck | Human food | 0.0437 | 0.0452 | 0.0135 |
| Rumen and forestomach | Human food | 0.0043 | 0.0048 | 0.0002 |
| SPA C3 | PAP C3 | 0.0028 | 0.0703 | 0.0043 |
| Screening and sifting wastes | C1-C2 for disposal | 0.0000 | 0.0000 | 0.0000 |
| Skin | Skin tannery C3 | 0.0991 | 0.0799 | 0.0684 |
| Sludge | Spreading/Compost | 0.0000 | 0.0000 | 0.0000 |
| Spleen | Pet food | 0.0021 | 0.0023 | 0.0001 |
| Sweetbread | Human food | 0.0018 | 0.0016 | 0.0018 |

Table 26: Total weighting by coproducts for Limousine Milk-fed Calf reared in Pasture

| COPRODUCT | Destination | Limousine /milk-fed calf/PASTURE | | |
| --- | --- | --- | --- | --- |
|  |  | **Biophysical Cumulative Share** | **Mass Cumulative Share** | **Economic Cumulative Share** |
| Abomasum | Human food | 0.0046 | 0.0051 | 0.0002 |
| Aponevrosis (1%) | Human food | 0.0076 | 0.0071 | 0.0050 |
| Bile | PAP C3 | 0.0001 | 0.0004 | 0.0000 |
| Blood | C1-C2 for disposal | 0.0000 | 0.0000 | 0.0000 |
| Bones (11%) | Gelatin C3 | 0.0798 | 0.0777 | 0.0002 |
| Dead individuals | C1-C2 for disposal | 0.0000 | 0.0000 | 0.0000 |
| Fat (8%) | Fat and greaves C3 | 0.1507 | 0.0565 | 0.0036 |
| Fat from breasts and penis | Fat and greaves C3 | 0.0406 | 0.0152 | 0.0010 |
| Feet (without hooves) | Human food | 0.0184 | 0.0203 | 0.0000 |
| Floatation fat | C1-C2 for disposal | 0.0000 | 0.0000 | 0.0000 |
| Head | Human food | 0.0457 | 0.0447 | 0.0718 |
| Intestines | C1-C2 for disposal | 0.0000 | 0.0000 | 0.0000 |
| Kidney | Human food | 0.0030 | 0.0034 | 0.0025 |
| Manure | Spreading/Compost | 0.0000 | 0.0000 | 0.0000 |
| Meat | Human food | 0.4975 | 0.5654 | 0.8273 |
| Pluck | Human food | 0.0433 | 0.0452 | 0.0135 |
| Rumen and forestomach | Human food | 0.0043 | 0.0048 | 0.0002 |
| SPA C3 | PAP C3 | 0.0028 | 0.0703 | 0.0043 |
| Screening and sifting wastes | C1-C2 for disposal | 0.0000 | 0.0000 | 0.0000 |
| Skin | Skin tannery C3 | 0.0977 | 0.0799 | 0.0684 |
| Sludge | Spreading/Compost | 0.0000 | 0.0000 | 0.0000 |
| Spleen | Pet food | 0.0021 | 0.0023 | 0.0001 |
| Sweetbread | Human food | 0.0017 | 0.0016 | 0.0018 |

Table 27: Total weighting by coproducts for Limousine Milk-fed Calf reared in Stall

| COPRODUCT | Destination | Limousine /Milk-fed calf/Stall | | |
| --- | --- | --- | --- | --- |
|  |  | **Biophysical Cumulative Share** | **Mass Cumulative Share** | **Economic Cumulative Share** |
| Abomasum | Human food | 0.0046 | 0.0051 | 0.0002 |
| Aponevrosis (1%) | Human food | 0.0075 | 0.0071 | 0.0050 |
| Bile | PAP C3 | 0.0001 | 0.0004 | 0.0000 |
| Blood | C1-C2 for disposal | 0.0000 | 0.0000 | 0.0000 |
| Bones (11%) | Gelatin C3 | 0.0788 | 0.0777 | 0.0002 |
| Dead individuals | C1-C2 for disposal | 0.0000 | 0.0000 | 0.0000 |
| Fat (8%) | Fat and greaves C3 | 0.1564 | 0.0565 | 0.0036 |
| Fat from breasts and penis | Fat and greaves C3 | 0.0421 | 0.0152 | 0.0010 |
| Feet (without hooves) | Human food | 0.0183 | 0.0203 | 0.0000 |
| Floatation fat | C1-C2 for disposal | 0.0000 | 0.0000 | 0.0000 |
| Head | Human food | 0.0454 | 0.0447 | 0.0718 |
| Intestines | C1-C2 for disposal | 0.0000 | 0.0000 | 0.0000 |
| Kidney | Human food | 0.0029 | 0.0034 | 0.0025 |
| Manure | Spreading/Compost | 0.0000 | 0.0000 | 0.0000 |
| Meat | Human food | 0.4936 | 0.5654 | 0.8273 |
| Pluck | Human food | 0.0429 | 0.0452 | 0.0135 |
| Rumen and forestomach | Human food | 0.0043 | 0.0048 | 0.0002 |
| SPA C3 | PAP C3 | 0.0027 | 0.0703 | 0.0043 |
| Screening and sifting wastes | C1-C2 for disposal | 0.0000 | 0.0000 | 0.0000 |
| Skin | Skin tannery C3 | 0.0964 | 0.0799 | 0.0684 |
| Sludge | Spreading/Compost | 0.0000 | 0.0000 | 0.0000 |
| Spleen | Pet food | 0.0021 | 0.0023 | 0.0001 |
| Sweetbread | Human food | 0.0017 | 0.0016 | 0.0018 |

Table 28: Total weighting by coproducts for Limousine Rosé Calf reared in Grazing Large Area

| COPRODUCT | Destination | Limousine /rosé calf/grazing large area | | |
| --- | --- | --- | --- | --- |
|  |  | **Biophysical Cumulative Share** | **Mass Cumulative Share** | **Economic Cumulative Share** |
| Abomasum | Human food | 0.0047 | 0.0051 | 0.0002 |
| Aponevrosis (1%) | Human food | 0.0077 | 0.0071 | 0.0050 |
| Bile | PAP C3 | 0.0001 | 0.0004 | 0.0000 |
| Blood | C1-C2 for disposal | 0.0000 | 0.0000 | 0.0000 |
| Bones (11%) | Gelatin C3 | 0.0815 | 0.0777 | 0.0002 |
| Dead individuals | C1-C2 for disposal | 0.0000 | 0.0000 | 0.0000 |
| Fat (8%) | Fat and greaves C3 | 0.1411 | 0.0565 | 0.0036 |
| Fat from breasts and penis | Fat and greaves C3 | 0.0380 | 0.0152 | 0.0010 |
| Feet (without hooves) | Human food | 0.0186 | 0.0203 | 0.0000 |
| Floatation fat | C1-C2 for disposal | 0.0000 | 0.0000 | 0.0000 |
| Head | Human food | 0.0462 | 0.0447 | 0.0718 |
| Intestines | C1-C2 for disposal | 0.0000 | 0.0000 | 0.0000 |
| Kidney | Human food | 0.0030 | 0.0034 | 0.0025 |
| Manure | Spreading/Compost | 0.0000 | 0.0000 | 0.0000 |
| Meat | Human food | 0.5041 | 0.5654 | 0.8273 |
| Pluck | Human food | 0.0439 | 0.0452 | 0.0135 |
| Rumen and forestomach | Human food | 0.0043 | 0.0048 | 0.0002 |
| SPA C3 | PAP C3 | 0.0028 | 0.0703 | 0.0043 |
| Screening and sifting wastes | C1-C2 for disposal | 0.0000 | 0.0000 | 0.0000 |
| Skin | Skin tannery C3 | 0.0999 | 0.0799 | 0.0684 |
| Sludge | Spreading/Compost | 0.0000 | 0.0000 | 0.0000 |
| Spleen | Pet food | 0.0021 | 0.0023 | 0.0001 |
| Sweetbread | Human food | 0.0018 | 0.0016 | 0.0018 |

Table 29: Total weighting by coproducts for Limousine Rosé Calf reared in Pasture

| COPRODUCT | Destination | Limousine /Rosé calf/Pasture | | |
| --- | --- | --- | --- | --- |
|  |  | **Biophysical Cumulative Share** | **Mass Cumulative Share** | **Economic Cumulative Share** |
| Abomasum | Human food | 0.0047 | 0.0051 | 0.0002 |
| Aponevrosis (1%) | Human food | 0.0077 | 0.0071 | 0.0050 |
| Bile | PAP C3 | 0.0001 | 0.0004 | 0.0000 |
| Blood | C1-C2 for disposal | 0.0000 | 0.0000 | 0.0000 |
| Bones (11%) | Gelatin C3 | 0.0806 | 0.0777 | 0.0002 |
| Dead individuals | C1-C2 for disposal | 0.0000 | 0.0000 | 0.0000 |
| Fat (8%) | Fat and greaves C3 | 0.1463 | 0.0565 | 0.0036 |
| Fat from breasts and penis | Fat and greaves C3 | 0.0394 | 0.0152 | 0.0010 |
| Feet (without hooves) | Human food | 0.0185 | 0.0203 | 0.0000 |
| Floatation fat | C1-C2 for disposal | 0.0000 | 0.0000 | 0.0000 |
| Head | Human food | 0.0460 | 0.0447 | 0.0718 |
| Intestines | C1-C2 for disposal | 0.0000 | 0.0000 | 0.0000 |
| Kidney | Human food | 0.0030 | 0.0034 | 0.0025 |
| Manure | Spreading/Compost | 0.0000 | 0.0000 | 0.0000 |
| Meat | Human food | 0.5006 | 0.5654 | 0.8273 |
| Pluck | Human food | 0.0436 | 0.0452 | 0.0135 |
| Rumen and forestomach | Human food | 0.0043 | 0.0048 | 0.0002 |
| SPA C3 | PAP C3 | 0.0028 | 0.0703 | 0.0043 |
| Screening and sifting wastes | C1-C2 for disposal | 0.0000 | 0.0000 | 0.0000 |
| Skin | Skin tannery C3 | 0.0987 | 0.0799 | 0.0684 |
| Sludge | Spreading/Compost | 0.0000 | 0.0000 | 0.0000 |
| Spleen | Pet food | 0.0021 | 0.0023 | 0.0001 |
| Sweetbread | Human food | 0.0018 | 0.0016 | 0.0018 |

Table 30: Total weighting by coproducts for Limousine Rosé Calf reared in Stall

| COPRODUCT | Destination | Limousine /Rosé calf/Stall | | |
| --- | --- | --- | --- | --- |
|  |  | **Biophysical Cumulative Share** | **Mass Cumulative Share** | **Economic Cumulative Share** |
| Abomasum | Human food | 0.0046 | 0.0051 | 0.0002 |
| Aponevrosis (1%) | Human food | 0.0076 | 0.0071 | 0.0050 |
| Bile | PAP C3 | 0.0001 | 0.0004 | 0.0000 |
| Blood | C1-C2 for disposal | 0.0000 | 0.0000 | 0.0000 |
| Bones (11%) | Gelatin C3 | 0.0796 | 0.0777 | 0.0002 |
| Dead individuals | C1-C2 for disposal | 0.0000 | 0.0000 | 0.0000 |
| Fat (8%) | Fat and greaves C3 | 0.1517 | 0.0565 | 0.0036 |
| Fat from breasts and penis | Fat and greaves C3 | 0.0409 | 0.0152 | 0.0010 |
| Feet (without hooves) | Human food | 0.0184 | 0.0203 | 0.0000 |
| Floatation fat | C1-C2 for disposal | 0.0000 | 0.0000 | 0.0000 |
| Head | Human food | 0.0457 | 0.0447 | 0.0718 |
| Intestines | C1-C2 for disposal | 0.0000 | 0.0000 | 0.0000 |
| Kidney | Human food | 0.0030 | 0.0034 | 0.0025 |
| Manure | Spreading/Compost | 0.0000 | 0.0000 | 0.0000 |
| Meat | Human food | 0.4970 | 0.5654 | 0.8273 |
| Pluck | Human food | 0.0433 | 0.0452 | 0.0135 |
| Rumen and forestomach | Human food | 0.0043 | 0.0048 | 0.0002 |
| SPA C3 | PAP C3 | 0.0027 | 0.0703 | 0.0043 |
| Screening and sifting wastes | C1-C2 for disposal | 0.0000 | 0.0000 | 0.0000 |
| Skin | Skin tannery C3 | 0.0974 | 0.0799 | 0.0684 |
| Sludge | Spreading/Compost | 0.0000 | 0.0000 | 0.0000 |
| Spleen | Pet food | 0.0021 | 0.0023 | 0.0001 |
| Sweetbread | Human food | 0.0017 | 0.0016 | 0.0018 |

Table 31: Total weighting by coproducts for Montbéliarde Milk-fed Calf reared in Grazing Large Area

| COPRODUCT | Destination | Montbéliarde /milk-fed calf/grazing large area | | |
| --- | --- | --- | --- | --- |
|  |  | **Biophysical Cumulative Share** | **Mass Cumulative Share** | **Economic Cumulative Share** |
| Abomasum | Human food | 0.0047 | 0.0051 | 0.0002 |
| Aponevrosis (1%) | Human food | 0.0077 | 0.0071 | 0.0050 |
| Bile | PAP C3 | 0.0001 | 0.0004 | 0.0000 |
| Blood | C1-C2 for disposal | 0.0000 | 0.0000 | 0.0000 |
| Bones (11%) | Gelatin C3 | 0.0817 | 0.0777 | 0.0002 |
| Dead individuals | C1-C2 for disposal | 0.0000 | 0.0000 | 0.0000 |
| Fat (8%) | Fat and greaves C3 | 0.1402 | 0.0565 | 0.0036 |
| Fat from breasts and penis | Fat and greaves C3 | 0.0378 | 0.0152 | 0.0010 |
| Feet (without hooves) | Human food | 0.0186 | 0.0203 | 0.0000 |
| Floatation fat | C1-C2 for disposal | 0.0000 | 0.0000 | 0.0000 |
| Head | Human food | 0.0463 | 0.0447 | 0.0718 |
| Intestines | C1-C2 for disposal | 0.0000 | 0.0000 | 0.0000 |
| Kidney | Human food | 0.0030 | 0.0034 | 0.0025 |
| Manure | Spreading/Compost | 0.0000 | 0.0000 | 0.0000 |
| Meat | Human food | 0.5048 | 0.5654 | 0.8273 |
| Pluck | Human food | 0.0440 | 0.0452 | 0.0135 |
| Rumen and forestomach | Human food | 0.0043 | 0.0048 | 0.0002 |
| SPA C3 | PAP C3 | 0.0028 | 0.0703 | 0.0043 |
| Screening and sifting wastes | C1-C2 for disposal | 0.0000 | 0.0000 | 0.0000 |
| Skin | Skin tannery C3 | 0.1001 | 0.0799 | 0.0684 |
| Sludge | Spreading/Compost | 0.0000 | 0.0000 | 0.0000 |
| Spleen | Pet food | 0.0021 | 0.0023 | 0.0001 |
| Sweetbread | Human food | 0.0018 | 0.0016 | 0.0018 |

Table 32: Total weighting by coproducts for Montbéliarde Milk-fed Calf reared in Pasture

| COPRODUCT | Destination | Montbéliarde /milk-fed calf/PASTURE | | |
| --- | --- | --- | --- | --- |
|  |  | **Biophysical Cumulative Share** | **Mass Cumulative Share** | **Economic Cumulative Share** |
| Abomasum | Human food | 0.0047 | 0.0051 | 0.0002 |
| Aponevrosis (1%) | Human food | 0.0077 | 0.0071 | 0.0050 |
| Bile | PAP C3 | 0.0001 | 0.0004 | 0.0000 |
| Blood | C1-C2 for disposal | 0.0000 | 0.0000 | 0.0000 |
| Bones (11%) | Gelatin C3 | 0.0807 | 0.0777 | 0.0002 |
| Dead individuals | C1-C2 for disposal | 0.0000 | 0.0000 | 0.0000 |
| Fat (8%) | Fat and greaves C3 | 0.1453 | 0.0565 | 0.0036 |
| Fat from breasts and penis | Fat and greaves C3 | 0.0391 | 0.0152 | 0.0010 |
| Feet (without hooves) | Human food | 0.0185 | 0.0203 | 0.0000 |
| Floatation fat | C1-C2 for disposal | 0.0000 | 0.0000 | 0.0000 |
| Head | Human food | 0.0460 | 0.0447 | 0.0718 |
| Intestines | C1-C2 for disposal | 0.0000 | 0.0000 | 0.0000 |
| Kidney | Human food | 0.0030 | 0.0034 | 0.0025 |
| Manure | Spreading/Compost | 0.0000 | 0.0000 | 0.0000 |
| Meat | Human food | 0.5013 | 0.5654 | 0.8273 |
| Pluck | Human food | 0.0437 | 0.0452 | 0.0135 |
| Rumen and forestomach | Human food | 0.0043 | 0.0048 | 0.0002 |
| SPA C3 | PAP C3 | 0.0028 | 0.0703 | 0.0043 |
| Screening and sifting wastes | C1-C2 for disposal | 0.0000 | 0.0000 | 0.0000 |
| Skin | Skin tannery C3 | 0.0989 | 0.0799 | 0.0684 |
| Sludge | Spreading/Compost | 0.0000 | 0.0000 | 0.0000 |
| Spleen | Pet food | 0.0021 | 0.0023 | 0.0001 |
| Sweetbread | Human food | 0.0018 | 0.0016 | 0.0018 |

Table 33: Total weighting by coproducts for Montbéliarde Milk-fed Calf reared in Stall

| COPRODUCT | Destination | Montbéliarde /Milk-fed calf/Stall | | |
| --- | --- | --- | --- | --- |
|  |  | **Biophysical Cumulative Share** | **Mass Cumulative Share** | **Economic Cumulative Share** |
| Abomasum | Human food | 0.0046 | 0.0051 | 0.0002 |
| Aponevrosis (1%) | Human food | 0.0076 | 0.0071 | 0.0050 |
| Bile | PAP C3 | 0.0001 | 0.0004 | 0.0000 |
| Blood | C1-C2 for disposal | 0.0000 | 0.0000 | 0.0000 |
| Bones (11%) | Gelatin C3 | 0.0798 | 0.0777 | 0.0002 |
| Dead individuals | C1-C2 for disposal | 0.0000 | 0.0000 | 0.0000 |
| Fat (8%) | Fat and greaves C3 | 0.1505 | 0.0565 | 0.0036 |
| Fat from breasts and penis | Fat and greaves C3 | 0.0405 | 0.0152 | 0.0010 |
| Feet (without hooves) | Human food | 0.0184 | 0.0203 | 0.0000 |
| Floatation fat | C1-C2 for disposal | 0.0000 | 0.0000 | 0.0000 |
| Head | Human food | 0.0457 | 0.0447 | 0.0718 |
| Intestines | C1-C2 for disposal | 0.0000 | 0.0000 | 0.0000 |
| Kidney | Human food | 0.0030 | 0.0034 | 0.0025 |
| Manure | Spreading/Compost | 0.0000 | 0.0000 | 0.0000 |
| Meat | Human food | 0.4978 | 0.5654 | 0.8273 |
| Pluck | Human food | 0.0433 | 0.0452 | 0.0135 |
| Rumen and forestomach | Human food | 0.0043 | 0.0048 | 0.0002 |
| SPA C3 | PAP C3 | 0.0027 | 0.0703 | 0.0043 |
| Screening and sifting wastes | C1-C2 for disposal | 0.0000 | 0.0000 | 0.0000 |
| Skin | Skin tannery C3 | 0.0977 | 0.0799 | 0.0684 |
| Sludge | Spreading/Compost | 0.0000 | 0.0000 | 0.0000 |
| Spleen | Pet food | 0.0021 | 0.0023 | 0.0001 |
| Sweetbread | Human food | 0.0017 | 0.0016 | 0.0018 |

Table 34: Total weighting by coproducts for Montbéliarde Rosé Calf reared in Grazing Large Area

| COPRODUCT | Destination | Montbéliarde /rosé calf/grazing large area | | |
| --- | --- | --- | --- | --- |
|  |  | **Biophysical Cumulative Share** | **Mass Cumulative Share** | **Economic Cumulative Share** |
| Abomasum | Human food | 0.0047 | 0.0051 | 0.0002 |
| Aponevrosis (1%) | Human food | 0.0078 | 0.0071 | 0.0050 |
| Bile | PAP C3 | 0.0001 | 0.0004 | 0.0000 |
| Blood | C1-C2 for disposal | 0.0000 | 0.0000 | 0.0000 |
| Bones (11%) | Gelatin C3 | 0.0823 | 0.0777 | 0.0002 |
| Dead individuals | C1-C2 for disposal | 0.0000 | 0.0000 | 0.0000 |
| Fat (8%) | Fat and greaves C3 | 0.1365 | 0.0565 | 0.0036 |
| Fat from breasts and penis | Fat and greaves C3 | 0.0368 | 0.0152 | 0.0010 |
| Feet (without hooves) | Human food | 0.0187 | 0.0203 | 0.0000 |
| Floatation fat | C1-C2 for disposal | 0.0000 | 0.0000 | 0.0000 |
| Head | Human food | 0.0465 | 0.0447 | 0.0718 |
| Intestines | C1-C2 for disposal | 0.0000 | 0.0000 | 0.0000 |
| Kidney | Human food | 0.0031 | 0.0034 | 0.0025 |
| Manure | Spreading/Compost | 0.0000 | 0.0000 | 0.0000 |
| Meat | Human food | 0.5074 | 0.5654 | 0.8273 |
| Pluck | Human food | 0.0442 | 0.0452 | 0.0135 |
| Rumen and forestomach | Human food | 0.0044 | 0.0048 | 0.0002 |
| SPA C3 | PAP C3 | 0.0029 | 0.0703 | 0.0043 |
| Screening and sifting wastes | C1-C2 for disposal | 0.0000 | 0.0000 | 0.0000 |
| Skin | Skin tannery C3 | 0.1008 | 0.0799 | 0.0684 |
| Sludge | Spreading/Compost | 0.0000 | 0.0000 | 0.0000 |
| Spleen | Pet food | 0.0021 | 0.0023 | 0.0001 |
| Sweetbread | Human food | 0.0018 | 0.0016 | 0.0018 |

Table 35: Total weighting by coproducts for Montbéliarde Rosé Calf reared in Pasture

| COPRODUCT | Destination | Montbéliarde /Rosé calf/Pasture | | |
| --- | --- | --- | --- | --- |
|  |  | **Biophysical Cumulative Share** | **Mass Cumulative Share** | **Economic Cumulative Share** |
| Abomasum | Human food | 0.0047 | 0.0051 | 0.0002 |
| Aponevrosis (1%) | Human food | 0.0077 | 0.0071 | 0.0050 |
| Bile | PAP C3 | 0.0001 | 0.0004 | 0.0000 |
| Blood | C1-C2 for disposal | 0.0000 | 0.0000 | 0.0000 |
| Bones (11%) | Gelatin C3 | 0.0814 | 0.0777 | 0.0002 |
| Dead individuals | C1-C2 for disposal | 0.0000 | 0.0000 | 0.0000 |
| Fat (8%) | Fat and greaves C3 | 0.1413 | 0.0565 | 0.0036 |
| Fat from breasts and penis | Fat and greaves C3 | 0.0381 | 0.0152 | 0.0010 |
| Feet (without hooves) | Human food | 0.0186 | 0.0203 | 0.0000 |
| Floatation fat | C1-C2 for disposal | 0.0000 | 0.0000 | 0.0000 |
| Head | Human food | 0.0463 | 0.0447 | 0.0718 |
| Intestines | C1-C2 for disposal | 0.0000 | 0.0000 | 0.0000 |
| Kidney | Human food | 0.0030 | 0.0034 | 0.0025 |
| Manure | Spreading/Compost | 0.0000 | 0.0000 | 0.0000 |
| Meat | Human food | 0.5042 | 0.5654 | 0.8273 |
| Pluck | Human food | 0.0439 | 0.0452 | 0.0135 |
| Rumen and forestomach | Human food | 0.0043 | 0.0048 | 0.0002 |
| SPA C3 | PAP C3 | 0.0028 | 0.0703 | 0.0043 |
| Screening and sifting wastes | C1-C2 for disposal | 0.0000 | 0.0000 | 0.0000 |
| Skin | Skin tannery C3 | 0.0997 | 0.0799 | 0.0684 |
| Sludge | Spreading/Compost | 0.0000 | 0.0000 | 0.0000 |
| Spleen | Pet food | 0.0021 | 0.0023 | 0.0001 |
| Sweetbread | Human food | 0.0018 | 0.0016 | 0.0018 |

Table 36: Total weighting by coproducts for Montbéliarde Rosé Calf reared in Stall

| COPRODUCT | Destination | Montbéliarde /Rosé calf/Stall | | |
| --- | --- | --- | --- | --- |
|  |  | **Biophysical Cumulative Share** | **Mass Cumulative Share** | **Economic Cumulative Share** |
| Abomasum | Human food | 0.0047 | 0.0051 | 0.0002 |
| Aponevrosis (1%) | Human food | 0.0077 | 0.0071 | 0.0050 |
| Bile | PAP C3 | 0.0001 | 0.0004 | 0.0000 |
| Blood | C1-C2 for disposal | 0.0000 | 0.0000 | 0.0000 |
| Bones (11%) | Gelatin C3 | 0.0805 | 0.0777 | 0.0002 |
| Dead individuals | C1-C2 for disposal | 0.0000 | 0.0000 | 0.0000 |
| Fat (8%) | Fat and greaves C3 | 0.1461 | 0.0565 | 0.0036 |
| Fat from breasts and penis | Fat and greaves C3 | 0.0394 | 0.0152 | 0.0010 |
| Feet (without hooves) | Human food | 0.0185 | 0.0203 | 0.0000 |
| Floatation fat | C1-C2 for disposal | 0.0000 | 0.0000 | 0.0000 |
| Head | Human food | 0.0460 | 0.0447 | 0.0718 |
| Intestines | C1-C2 for disposal | 0.0000 | 0.0000 | 0.0000 |
| Kidney | Human food | 0.0030 | 0.0034 | 0.0025 |
| Manure | Spreading/Compost | 0.0000 | 0.0000 | 0.0000 |
| Meat | Human food | 0.5009 | 0.5654 | 0.8273 |
| Pluck | Human food | 0.0436 | 0.0452 | 0.0135 |
| Rumen and forestomach | Human food | 0.0043 | 0.0048 | 0.0002 |
| SPA C3 | PAP C3 | 0.0028 | 0.0703 | 0.0043 |
| Screening and sifting wastes | C1-C2 for disposal | 0.0000 | 0.0000 | 0.0000 |
| Skin | Skin tannery C3 | 0.0986 | 0.0799 | 0.0684 |
| Sludge | Spreading/Compost | 0.0000 | 0.0000 | 0.0000 |
| Spleen | Pet food | 0.0021 | 0.0023 | 0.0001 |
| Sweetbread | Human food | 0.0018 | 0.0016 | 0.0018 |

Table 37: Total weighting by coproducts for Normande Milk-fed Calf reared in Grazing Large Area

| COPRODUCT | Destination | Normande /milk-fed calf/grazing large area | | |
| --- | --- | --- | --- | --- |
|  |  | **Biophysical Cumulative Share** | **Mass Cumulative Share** | **Economic Cumulative Share** |
| Abomasum | Human food | 0.0047 | 0.0051 | 0.0002 |
| Aponevrosis (1%) | Human food | 0.0077 | 0.0071 | 0.0050 |
| Bile | PAP C3 | 0.0001 | 0.0004 | 0.0000 |
| Blood | C1-C2 for disposal | 0.0000 | 0.0000 | 0.0000 |
| Bones (11%) | Gelatin C3 | 0.0817 | 0.0777 | 0.0002 |
| Dead individuals | C1-C2 for disposal | 0.0000 | 0.0000 | 0.0000 |
| Fat (8%) | Fat and greaves C3 | 0.1403 | 0.0565 | 0.0036 |
| Fat from breasts and penis | Fat and greaves C3 | 0.0378 | 0.0152 | 0.0010 |
| Feet (without hooves) | Human food | 0.0186 | 0.0203 | 0.0000 |
| Floatation fat | C1-C2 for disposal | 0.0000 | 0.0000 | 0.0000 |
| Head | Human food | 0.0463 | 0.0447 | 0.0718 |
| Intestines | C1-C2 for disposal | 0.0000 | 0.0000 | 0.0000 |
| Kidney | Human food | 0.0030 | 0.0034 | 0.0025 |
| Manure | Spreading/Compost | 0.0000 | 0.0000 | 0.0000 |
| Meat | Human food | 0.5047 | 0.5654 | 0.8273 |
| Pluck | Human food | 0.0440 | 0.0452 | 0.0135 |
| Rumen and forestomach | Human food | 0.0043 | 0.0048 | 0.0002 |
| SPA C3 | PAP C3 | 0.0028 | 0.0703 | 0.0043 |
| Screening and sifting wastes | C1-C2 for disposal | 0.0000 | 0.0000 | 0.0000 |
| Skin | Skin tannery C3 | 0.1000 | 0.0799 | 0.0684 |
| Sludge | Spreading/Compost | 0.0000 | 0.0000 | 0.0000 |
| Spleen | Pet food | 0.0021 | 0.0023 | 0.0001 |
| Sweetbread | Human food | 0.0018 | 0.0016 | 0.0018 |

Table 38: Total weighting by coproducts for Normande Milk-fed Calf reared in Pasture

| COPRODUCT | Destination | Normande /milk-fed calf/PASTURE | | |
| --- | --- | --- | --- | --- |
|  |  | **Biophysical Cumulative Share** | **Mass Cumulative Share** | **Economic Cumulative Share** |
| Abomasum | Human food | 0.0047 | 0.0051 | 0.0002 |
| Aponevrosis (1%) | Human food | 0.0077 | 0.0071 | 0.0050 |
| Bile | PAP C3 | 0.0001 | 0.0004 | 0.0000 |
| Blood | C1-C2 for disposal | 0.0000 | 0.0000 | 0.0000 |
| Bones (11%) | Gelatin C3 | 0.0807 | 0.0777 | 0.0002 |
| Dead individuals | C1-C2 for disposal | 0.0000 | 0.0000 | 0.0000 |
| Fat (8%) | Fat and greaves C3 | 0.1454 | 0.0565 | 0.0036 |
| Fat from breasts and penis | Fat and greaves C3 | 0.0392 | 0.0152 | 0.0010 |
| Feet (without hooves) | Human food | 0.0185 | 0.0203 | 0.0000 |
| Floatation fat | C1-C2 for disposal | 0.0000 | 0.0000 | 0.0000 |
| Head | Human food | 0.0460 | 0.0447 | 0.0718 |
| Intestines | C1-C2 for disposal | 0.0000 | 0.0000 | 0.0000 |
| Kidney | Human food | 0.0030 | 0.0034 | 0.0025 |
| Manure | Spreading/Compost | 0.0000 | 0.0000 | 0.0000 |
| Meat | Human food | 0.5012 | 0.5654 | 0.8273 |
| Pluck | Human food | 0.0437 | 0.0452 | 0.0135 |
| Rumen and forestomach | Human food | 0.0043 | 0.0048 | 0.0002 |
| SPA C3 | PAP C3 | 0.0028 | 0.0703 | 0.0043 |
| Screening and sifting wastes | C1-C2 for disposal | 0.0000 | 0.0000 | 0.0000 |
| Skin | Skin tannery C3 | 0.0988 | 0.0799 | 0.0684 |
| Sludge | Spreading/Compost | 0.0000 | 0.0000 | 0.0000 |
| Spleen | Pet food | 0.0021 | 0.0023 | 0.0001 |
| Sweetbread | Human food | 0.0018 | 0.0016 | 0.0018 |

Table 39: Total weighting by coproducts for Normande Milk-fed Calf reared in Stall

| COPRODUCT | Destination | Normande /Milk-fed calf/Stall | | |
| --- | --- | --- | --- | --- |
|  |  | **Biophysical Cumulative Share** | **Mass Cumulative Share** | **Economic Cumulative Share** |
| Abomasum | Human food | 0.0046 | 0.0051 | 0.0002 |
| Aponevrosis (1%) | Human food | 0.0076 | 0.0071 | 0.0050 |
| Bile | PAP C3 | 0.0001 | 0.0004 | 0.0000 |
| Blood | C1-C2 for disposal | 0.0000 | 0.0000 | 0.0000 |
| Bones (11%) | Gelatin C3 | 0.0798 | 0.0777 | 0.0002 |
| Dead individuals | C1-C2 for disposal | 0.0000 | 0.0000 | 0.0000 |
| Fat (8%) | Fat and greaves C3 | 0.1507 | 0.0565 | 0.0036 |
| Fat from breasts and penis | Fat and greaves C3 | 0.0406 | 0.0152 | 0.0010 |
| Feet (without hooves) | Human food | 0.0184 | 0.0203 | 0.0000 |
| Floatation fat | C1-C2 for disposal | 0.0000 | 0.0000 | 0.0000 |
| Head | Human food | 0.0457 | 0.0447 | 0.0718 |
| Intestines | C1-C2 for disposal | 0.0000 | 0.0000 | 0.0000 |
| Kidney | Human food | 0.0030 | 0.0034 | 0.0025 |
| Manure | Spreading/Compost | 0.0000 | 0.0000 | 0.0000 |
| Meat | Human food | 0.4977 | 0.5654 | 0.8273 |
| Pluck | Human food | 0.0433 | 0.0452 | 0.0135 |
| Rumen and forestomach | Human food | 0.0043 | 0.0048 | 0.0002 |
| SPA C3 | PAP C3 | 0.0027 | 0.0703 | 0.0043 |
| Screening and sifting wastes | C1-C2 for disposal | 0.0000 | 0.0000 | 0.0000 |
| Skin | Skin tannery C3 | 0.0976 | 0.0799 | 0.0684 |
| Sludge | Spreading/Compost | 0.0000 | 0.0000 | 0.0000 |
| Spleen | Pet food | 0.0021 | 0.0023 | 0.0001 |
| Sweetbread | Human food | 0.0017 | 0.0016 | 0.0018 |

Table 40: Total weighting by coproducts for Normande Rosé Calf reared in Grazing Large Area

| COPRODUCT | Destination | Normande /rosé calf/grazing large area | | |
| --- | --- | --- | --- | --- |
|  |  | **Biophysical Cumulative Share** | **Mass Cumulative Share** | **Economic Cumulative Share** |
| Abomasum | Human food | 0.0047 | 0.0051 | 0.0002 |
| Aponevrosis (1%) | Human food | 0.0078 | 0.0071 | 0.0050 |
| Bile | PAP C3 | 0.0001 | 0.0004 | 0.0000 |
| Blood | C1-C2 for disposal | 0.0000 | 0.0000 | 0.0000 |
| Bones (11%) | Gelatin C3 | 0.0822 | 0.0777 | 0.0002 |
| Dead individuals | C1-C2 for disposal | 0.0000 | 0.0000 | 0.0000 |
| Fat (8%) | Fat and greaves C3 | 0.1367 | 0.0565 | 0.0036 |
| Fat from breasts and penis | Fat and greaves C3 | 0.0368 | 0.0152 | 0.0010 |
| Feet (without hooves) | Human food | 0.0187 | 0.0203 | 0.0000 |
| Floatation fat | C1-C2 for disposal | 0.0000 | 0.0000 | 0.0000 |
| Head | Human food | 0.0465 | 0.0447 | 0.0718 |
| Intestines | C1-C2 for disposal | 0.0000 | 0.0000 | 0.0000 |
| Kidney | Human food | 0.0031 | 0.0034 | 0.0025 |
| Manure | Spreading/Compost | 0.0000 | 0.0000 | 0.0000 |
| Meat | Human food | 0.5073 | 0.5654 | 0.8273 |
| Pluck | Human food | 0.0442 | 0.0452 | 0.0135 |
| Rumen and forestomach | Human food | 0.0044 | 0.0048 | 0.0002 |
| SPA C3 | PAP C3 | 0.0029 | 0.0703 | 0.0043 |
| Screening and sifting wastes | C1-C2 for disposal | 0.0000 | 0.0000 | 0.0000 |
| Skin | Skin tannery C3 | 0.1008 | 0.0799 | 0.0684 |
| Sludge | Spreading/Compost | 0.0000 | 0.0000 | 0.0000 |
| Spleen | Pet food | 0.0021 | 0.0023 | 0.0001 |
| Sweetbread | Human food | 0.0018 | 0.0016 | 0.0018 |

Table 41: Total weighting by coproducts for Normande Rosé Calf reared in Pasture

| COPRODUCT | Destination | Normande /Rosé calf/Pasture | | |
| --- | --- | --- | --- | --- |
|  |  | **Biophysical Cumulative Share** | **Mass Cumulative Share** | **Economic Cumulative Share** |
| Abomasum | Human food | 0.0047 | 0.0051 | 0.0002 |
| Aponevrosis (1%) | Human food | 0.0077 | 0.0071 | 0.0050 |
| Bile | PAP C3 | 0.0001 | 0.0004 | 0.0000 |
| Blood | C1-C2 for disposal | 0.0000 | 0.0000 | 0.0000 |
| Bones (11%) | Gelatin C3 | 0.0814 | 0.0777 | 0.0002 |
| Dead individuals | C1-C2 for disposal | 0.0000 | 0.0000 | 0.0000 |
| Fat (8%) | Fat and greaves C3 | 0.1414 | 0.0565 | 0.0036 |
| Fat from breasts and penis | Fat and greaves C3 | 0.0381 | 0.0152 | 0.0010 |
| Feet (without hooves) | Human food | 0.0186 | 0.0203 | 0.0000 |
| Floatation fat | C1-C2 for disposal | 0.0000 | 0.0000 | 0.0000 |
| Head | Human food | 0.0463 | 0.0447 | 0.0718 |
| Intestines | C1-C2 for disposal | 0.0000 | 0.0000 | 0.0000 |
| Kidney | Human food | 0.0030 | 0.0034 | 0.0025 |
| Manure | Spreading/Compost | 0.0000 | 0.0000 | 0.0000 |
| Meat | Human food | 0.5040 | 0.5654 | 0.8273 |
| Pluck | Human food | 0.0439 | 0.0452 | 0.0135 |
| Rumen and forestomach | Human food | 0.0043 | 0.0048 | 0.0002 |
| SPA C3 | PAP C3 | 0.0028 | 0.0703 | 0.0043 |
| Screening and sifting wastes | C1-C2 for disposal | 0.0000 | 0.0000 | 0.0000 |
| Skin | Skin tannery C3 | 0.0997 | 0.0799 | 0.0684 |
| Sludge | Spreading/Compost | 0.0000 | 0.0000 | 0.0000 |
| Spleen | Pet food | 0.0021 | 0.0023 | 0.0001 |
| Sweetbread | Human food | 0.0018 | 0.0016 | 0.0018 |

Table 42: Total weighting by coproducts for Normande Rosé Calf reared in Stall

| COPRODUCT | Destination | Normande /Rosé calf/Stall | | |
| --- | --- | --- | --- | --- |
|  |  | **Biophysical Cumulative Share** | **Mass Cumulative Share** | **Economic Cumulative Share** |
| Abomasum | Human food | 0.0047 | 0.0051 | 0.0002 |
| Aponevrosis (1%) | Human food | 0.0077 | 0.0071 | 0.0050 |
| Bile | PAP C3 | 0.0001 | 0.0004 | 0.0000 |
| Blood | C1-C2 for disposal | 0.0000 | 0.0000 | 0.0000 |
| Bones (11%) | Gelatin C3 | 0.0805 | 0.0777 | 0.0002 |
| Dead individuals | C1-C2 for disposal | 0.0000 | 0.0000 | 0.0000 |
| Fat (8%) | Fat and greaves C3 | 0.1463 | 0.0565 | 0.0036 |
| Fat from breasts and penis | Fat and greaves C3 | 0.0394 | 0.0152 | 0.0010 |
| Feet (without hooves) | Human food | 0.0185 | 0.0203 | 0.0000 |
| Floatation fat | C1-C2 for disposal | 0.0000 | 0.0000 | 0.0000 |
| Head | Human food | 0.0460 | 0.0447 | 0.0718 |
| Intestines | C1-C2 for disposal | 0.0000 | 0.0000 | 0.0000 |
| Kidney | Human food | 0.0030 | 0.0034 | 0.0025 |
| Manure | Spreading/Compost | 0.0000 | 0.0000 | 0.0000 |
| Meat | Human food | 0.5008 | 0.5654 | 0.8273 |
| Pluck | Human food | 0.0436 | 0.0452 | 0.0135 |
| Rumen and forestomach | Human food | 0.0043 | 0.0048 | 0.0002 |
| SPA C3 | PAP C3 | 0.0028 | 0.0703 | 0.0043 |
| Screening and sifting wastes | C1-C2 for disposal | 0.0000 | 0.0000 | 0.0000 |
| Skin | Skin tannery C3 | 0.0985 | 0.0799 | 0.0684 |
| Sludge | Spreading/Compost | 0.0000 | 0.0000 | 0.0000 |
| Spleen | Pet food | 0.0021 | 0.0023 | 0.0001 |
| Sweetbread | Human food | 0.0018 | 0.0016 | 0.0018 |

Table 43: Total weighting by coproducts for Primholstein Milk-fed Calf reared in Grazing Large Area

| COPRODUCT | Destination | Primholstein /milk-fed calf/grazing large area | | |
| --- | --- | --- | --- | --- |
|  |  | **Biophysical Cumulative Share** | **Mass Cumulative Share** | **Economic Cumulative Share** |
| Abomasum | Human food | 0.0047 | 0.0051 | 0.0002 |
| Aponevrosis (1%) | Human food | 0.0077 | 0.0071 | 0.0050 |
| Bile | PAP C3 | 0.0001 | 0.0004 | 0.0000 |
| Blood | C1-C2 for disposal | 0.0000 | 0.0000 | 0.0000 |
| Bones (11%) | Gelatin C3 | 0.0815 | 0.0777 | 0.0002 |
| Dead individuals | C1-C2 for disposal | 0.0000 | 0.0000 | 0.0000 |
| Fat (8%) | Fat and greaves C3 | 0.1411 | 0.0565 | 0.0036 |
| Fat from breasts and penis | Fat and greaves C3 | 0.0380 | 0.0152 | 0.0010 |
| Feet (without hooves) | Human food | 0.0186 | 0.0203 | 0.0000 |
| Floatation fat | C1-C2 for disposal | 0.0000 | 0.0000 | 0.0000 |
| Head | Human food | 0.0462 | 0.0447 | 0.0718 |
| Intestines | C1-C2 for disposal | 0.0000 | 0.0000 | 0.0000 |
| Kidney | Human food | 0.0030 | 0.0034 | 0.0025 |
| Manure | Spreading/Compost | 0.0000 | 0.0000 | 0.0000 |
| Meat | Human food | 0.5041 | 0.5654 | 0.8273 |
| Pluck | Human food | 0.0439 | 0.0452 | 0.0135 |
| Rumen and forestomach | Human food | 0.0043 | 0.0048 | 0.0002 |
| SPA C3 | PAP C3 | 0.0028 | 0.0703 | 0.0043 |
| Screening and sifting wastes | C1-C2 for disposal | 0.0000 | 0.0000 | 0.0000 |
| Skin | Skin tannery C3 | 0.0999 | 0.0799 | 0.0684 |
| Sludge | Spreading/Compost | 0.0000 | 0.0000 | 0.0000 |
| Spleen | Pet food | 0.0021 | 0.0023 | 0.0001 |
| Sweetbread | Human food | 0.0018 | 0.0016 | 0.0018 |

Table 44: Total weighting by coproducts for Primholstein Milk-fed Calf reared in Pasture

| COPRODUCT | Destination | Primholstein /milk-fed calf/PASTURE | | |
| --- | --- | --- | --- | --- |
|  |  | **Biophysical Cumulative Share** | **Mass Cumulative Share** | **Economic Cumulative Share** |
| Abomasum | Human food | 0.0047 | 0.0051 | 0.0002 |
| Aponevrosis (1%) | Human food | 0.0077 | 0.0071 | 0.0050 |
| Bile | PAP C3 | 0.0001 | 0.0004 | 0.0000 |
| Blood | C1-C2 for disposal | 0.0000 | 0.0000 | 0.0000 |
| Bones (11%) | Gelatin C3 | 0.0806 | 0.0777 | 0.0002 |
| Dead individuals | C1-C2 for disposal | 0.0000 | 0.0000 | 0.0000 |
| Fat (8%) | Fat and greaves C3 | 0.1463 | 0.0565 | 0.0036 |
| Fat from breasts and penis | Fat and greaves C3 | 0.0394 | 0.0152 | 0.0010 |
| Feet (without hooves) | Human food | 0.0185 | 0.0203 | 0.0000 |
| Floatation fat | C1-C2 for disposal | 0.0000 | 0.0000 | 0.0000 |
| Head | Human food | 0.0460 | 0.0447 | 0.0718 |
| Intestines | C1-C2 for disposal | 0.0000 | 0.0000 | 0.0000 |
| Kidney | Human food | 0.0030 | 0.0034 | 0.0025 |
| Manure | Spreading/Compost | 0.0000 | 0.0000 | 0.0000 |
| Meat | Human food | 0.5006 | 0.5654 | 0.8273 |
| Pluck | Human food | 0.0436 | 0.0452 | 0.0135 |
| Rumen and forestomach | Human food | 0.0043 | 0.0048 | 0.0002 |
| SPA C3 | PAP C3 | 0.0028 | 0.0703 | 0.0043 |
| Screening and sifting wastes | C1-C2 for disposal | 0.0000 | 0.0000 | 0.0000 |
| Skin | Skin tannery C3 | 0.0987 | 0.0799 | 0.0684 |
| Sludge | Spreading/Compost | 0.0000 | 0.0000 | 0.0000 |
| Spleen | Pet food | 0.0021 | 0.0023 | 0.0001 |
| Sweetbread | Human food | 0.0018 | 0.0016 | 0.0018 |

Table 45: Total weighting by coproducts for Primholstein Milk-fed Calf reared in Stall

| COPRODUCT | Destination | Primholstein /Milk-fed calf/Stall | | |
| --- | --- | --- | --- | --- |
|  |  | **Biophysical Cumulative Share** | **Mass Cumulative Share** | **Economic Cumulative Share** |
| Abomasum | Human food | 0.0046 | 0.0051 | 0.0002 |
| Aponevrosis (1%) | Human food | 0.0076 | 0.0071 | 0.0050 |
| Bile | PAP C3 | 0.0001 | 0.0004 | 0.0000 |
| Blood | C1-C2 for disposal | 0.0000 | 0.0000 | 0.0000 |
| Bones (11%) | Gelatin C3 | 0.0796 | 0.0777 | 0.0002 |
| Dead individuals | C1-C2 for disposal | 0.0000 | 0.0000 | 0.0000 |
| Fat (8%) | Fat and greaves C3 | 0.1517 | 0.0565 | 0.0036 |
| Fat from breasts and penis | Fat and greaves C3 | 0.0409 | 0.0152 | 0.0010 |
| Feet (without hooves) | Human food | 0.0184 | 0.0203 | 0.0000 |
| Floatation fat | C1-C2 for disposal | 0.0000 | 0.0000 | 0.0000 |
| Head | Human food | 0.0457 | 0.0447 | 0.0718 |
| Intestines | C1-C2 for disposal | 0.0000 | 0.0000 | 0.0000 |
| Kidney | Human food | 0.0030 | 0.0034 | 0.0025 |
| Manure | Spreading/Compost | 0.0000 | 0.0000 | 0.0000 |
| Meat | Human food | 0.4970 | 0.5654 | 0.8273 |
| Pluck | Human food | 0.0433 | 0.0452 | 0.0135 |
| Rumen and forestomach | Human food | 0.0043 | 0.0048 | 0.0002 |
| SPA C3 | PAP C3 | 0.0027 | 0.0703 | 0.0043 |
| Screening and sifting wastes | C1-C2 for disposal | 0.0000 | 0.0000 | 0.0000 |
| Skin | Skin tannery C3 | 0.0974 | 0.0799 | 0.0684 |
| Sludge | Spreading/Compost | 0.0000 | 0.0000 | 0.0000 |
| Spleen | Pet food | 0.0021 | 0.0023 | 0.0001 |
| Sweetbread | Human food | 0.0017 | 0.0016 | 0.0018 |

Table 46: Total weighting by coproducts for Primholstein Rosé Calf reared in Grazing Large Area

| COPRODUCT | Destination | Primholstein /rosé calf/grazing large area | | |
| --- | --- | --- | --- | --- |
|  |  | **Biophysical Cumulative Share** | **Mass Cumulative Share** | **Economic Cumulative Share** |
| Abomasum | Human food | 0.0047 | 0.0051 | 0.0002 |
| Aponevrosis (1%) | Human food | 0.0078 | 0.0071 | 0.0050 |
| Bile | PAP C3 | 0.0001 | 0.0004 | 0.0000 |
| Blood | C1-C2 for disposal | 0.0000 | 0.0000 | 0.0000 |
| Bones (11%) | Gelatin C3 | 0.0821 | 0.0777 | 0.0002 |
| Dead individuals | C1-C2 for disposal | 0.0000 | 0.0000 | 0.0000 |
| Fat (8%) | Fat and greaves C3 | 0.1373 | 0.0565 | 0.0036 |
| Fat from breasts and penis | Fat and greaves C3 | 0.0370 | 0.0152 | 0.0010 |
| Feet (without hooves) | Human food | 0.0187 | 0.0203 | 0.0000 |
| Floatation fat | C1-C2 for disposal | 0.0000 | 0.0000 | 0.0000 |
| Head | Human food | 0.0465 | 0.0447 | 0.0718 |
| Intestines | C1-C2 for disposal | 0.0000 | 0.0000 | 0.0000 |
| Kidney | Human food | 0.0031 | 0.0034 | 0.0025 |
| Manure | Spreading/Compost | 0.0000 | 0.0000 | 0.0000 |
| Meat | Human food | 0.5068 | 0.5654 | 0.8273 |
| Pluck | Human food | 0.0442 | 0.0452 | 0.0135 |
| Rumen and forestomach | Human food | 0.0044 | 0.0048 | 0.0002 |
| SPA C3 | PAP C3 | 0.0029 | 0.0703 | 0.0043 |
| Screening and sifting wastes | C1-C2 for disposal | 0.0000 | 0.0000 | 0.0000 |
| Skin | Skin tannery C3 | 0.1007 | 0.0799 | 0.0684 |
| Sludge | Spreading/Compost | 0.0000 | 0.0000 | 0.0000 |
| Spleen | Pet food | 0.0021 | 0.0023 | 0.0001 |
| Sweetbread | Human food | 0.0018 | 0.0016 | 0.0018 |

Table 47: Total weighting by coproducts for Primholstein Rosé Calf reared in Pasture

| COPRODUCT | Destination | Primholstein /Rosé calf/Pasture | | |
| --- | --- | --- | --- | --- |
|  |  | **Biophysical Cumulative Share** | **Mass Cumulative Share** | **Economic Cumulative Share** |
| Abomasum | Human food | 0.0047 | 0.0051 | 0.0002 |
| Aponevrosis (1%) | Human food | 0.0077 | 0.0071 | 0.0050 |
| Bile | PAP C3 | 0.0001 | 0.0004 | 0.0000 |
| Blood | C1-C2 for disposal | 0.0000 | 0.0000 | 0.0000 |
| Bones (11%) | Gelatin C3 | 0.0812 | 0.0777 | 0.0002 |
| Dead individuals | C1-C2 for disposal | 0.0000 | 0.0000 | 0.0000 |
| Fat (8%) | Fat and greaves C3 | 0.1422 | 0.0565 | 0.0036 |
| Fat from breasts and penis | Fat and greaves C3 | 0.0383 | 0.0152 | 0.0010 |
| Feet (without hooves) | Human food | 0.0186 | 0.0203 | 0.0000 |
| Floatation fat | C1-C2 for disposal | 0.0000 | 0.0000 | 0.0000 |
| Head | Human food | 0.0462 | 0.0447 | 0.0718 |
| Intestines | C1-C2 for disposal | 0.0000 | 0.0000 | 0.0000 |
| Kidney | Human food | 0.0030 | 0.0034 | 0.0025 |
| Manure | Spreading/Compost | 0.0000 | 0.0000 | 0.0000 |
| Meat | Human food | 0.5035 | 0.5654 | 0.8273 |
| Pluck | Human food | 0.0439 | 0.0452 | 0.0135 |
| Rumen and forestomach | Human food | 0.0043 | 0.0048 | 0.0002 |
| SPA C3 | PAP C3 | 0.0028 | 0.0703 | 0.0043 |
| Screening and sifting wastes | C1-C2 for disposal | 0.0000 | 0.0000 | 0.0000 |
| Skin | Skin tannery C3 | 0.0995 | 0.0799 | 0.0684 |
| Sludge | Spreading/Compost | 0.0000 | 0.0000 | 0.0000 |
| Spleen | Pet food | 0.0021 | 0.0023 | 0.0001 |
| Sweetbread | Human food | 0.0018 | 0.0016 | 0.0018 |

Table 48: Total weighting by coproducts for Primholstein Rosé Calf reared in Stall

| COPRODUCT | Destination | Primholstein /Rosé calf/Stall | | |
| --- | --- | --- | --- | --- |
|  |  | **Biophysical Cumulative Share** | **Mass Cumulative Share** | **Economic Cumulative Share** |
| Abomasum | Human food | 0.0047 | 0.0051 | 0.0002 |
| Aponevrosis (1%) | Human food | 0.0076 | 0.0071 | 0.0050 |
| Bile | PAP C3 | 0.0001 | 0.0004 | 0.0000 |
| Blood | C1-C2 for disposal | 0.0000 | 0.0000 | 0.0000 |
| Bones (11%) | Gelatin C3 | 0.0803 | 0.0777 | 0.0002 |
| Dead individuals | C1-C2 for disposal | 0.0000 | 0.0000 | 0.0000 |
| Fat (8%) | Fat and greaves C3 | 0.1471 | 0.0565 | 0.0036 |
| Fat from breasts and penis | Fat and greaves C3 | 0.0396 | 0.0152 | 0.0010 |
| Feet (without hooves) | Human food | 0.0185 | 0.0203 | 0.0000 |
| Floatation fat | C1-C2 for disposal | 0.0000 | 0.0000 | 0.0000 |
| Head | Human food | 0.0459 | 0.0447 | 0.0718 |
| Intestines | C1-C2 for disposal | 0.0000 | 0.0000 | 0.0000 |
| Kidney | Human food | 0.0030 | 0.0034 | 0.0025 |
| Manure | Spreading/Compost | 0.0000 | 0.0000 | 0.0000 |
| Meat | Human food | 0.5002 | 0.5654 | 0.8273 |
| Pluck | Human food | 0.0436 | 0.0452 | 0.0135 |
| Rumen and forestomach | Human food | 0.0043 | 0.0048 | 0.0002 |
| SPA C3 | PAP C3 | 0.0028 | 0.0703 | 0.0043 |
| Screening and sifting wastes | C1-C2 for disposal | 0.0000 | 0.0000 | 0.0000 |
| Skin | Skin tannery C3 | 0.0984 | 0.0799 | 0.0684 |
| Sludge | Spreading/Compost | 0.0000 | 0.0000 | 0.0000 |
| Spleen | Pet food | 0.0021 | 0.0023 | 0.0001 |
| Sweetbread | Human food | 0.0018 | 0.0016 | 0.0018 |

Table 49: Total weighting by coproducts for Croisé-lait Milk-fed Calf reared in Grazing Large Area

| COPRODUCT | Destination | Croisé-lait /milk-fed calf/grazing large area | | |
| --- | --- | --- | --- | --- |
|  |  | **Biophysical Cumulative Share** | **Mass Cumulative Share** | **Economic Cumulative Share** |
| Abomasum | Human food | 0.0047 | 0.0051 | 0.0002 |
| Aponevrosis (1%) | Human food | 0.0077 | 0.0071 | 0.0050 |
| Bile | PAP C3 | 0.0001 | 0.0004 | 0.0000 |
| Blood | C1-C2 for disposal | 0.0000 | 0.0000 | 0.0000 |
| Bones (11%) | Gelatin C3 | 0.0815 | 0.0777 | 0.0002 |
| Dead individuals | C1-C2 for disposal | 0.0000 | 0.0000 | 0.0000 |
| Fat (8%) | Fat and greaves C3 | 0.1415 | 0.0565 | 0.0036 |
| Fat from breasts and penis | Fat and greaves C3 | 0.0381 | 0.0152 | 0.0010 |
| Feet (without hooves) | Human food | 0.0186 | 0.0203 | 0.0000 |
| Floatation fat | C1-C2 for disposal | 0.0000 | 0.0000 | 0.0000 |
| Head | Human food | 0.0462 | 0.0447 | 0.0718 |
| Intestines | C1-C2 for disposal | 0.0000 | 0.0000 | 0.0000 |
| Kidney | Human food | 0.0030 | 0.0034 | 0.0025 |
| Manure | Spreading/Compost | 0.0000 | 0.0000 | 0.0000 |
| Meat | Human food | 0.5038 | 0.5654 | 0.8273 |
| Pluck | Human food | 0.0439 | 0.0452 | 0.0135 |
| Rumen and forestomach | Human food | 0.0043 | 0.0048 | 0.0002 |
| SPA C3 | PAP C3 | 0.0028 | 0.0703 | 0.0043 |
| Screening and sifting wastes | C1-C2 for disposal | 0.0000 | 0.0000 | 0.0000 |
| Skin | Skin tannery C3 | 0.0998 | 0.0799 | 0.0684 |
| Sludge | Spreading/Compost | 0.0000 | 0.0000 | 0.0000 |
| Spleen | Pet food | 0.0021 | 0.0023 | 0.0001 |
| Sweetbread | Human food | 0.0018 | 0.0016 | 0.0018 |

Table 50: Total weighting by coproducts for Croisé-lait Milk-fed Calf reared in Pasture

| COPRODUCT | Destination | Croisé-lait /milk-fed calf/PASTURE | | |
| --- | --- | --- | --- | --- |
|  |  | **Biophysical Cumulative Share** | **Mass Cumulative Share** | **Economic Cumulative Share** |
| Abomasum | Human food | 0.0046 | 0.0051 | 0.0002 |
| Aponevrosis (1%) | Human food | 0.0077 | 0.0071 | 0.0050 |
| Bile | PAP C3 | 0.0001 | 0.0004 | 0.0000 |
| Blood | C1-C2 for disposal | 0.0000 | 0.0000 | 0.0000 |
| Bones (11%) | Gelatin C3 | 0.0805 | 0.0777 | 0.0002 |
| Dead individuals | C1-C2 for disposal | 0.0000 | 0.0000 | 0.0000 |
| Fat (8%) | Fat and greaves C3 | 0.1467 | 0.0565 | 0.0036 |
| Fat from breasts and penis | Fat and greaves C3 | 0.0395 | 0.0152 | 0.0010 |
| Feet (without hooves) | Human food | 0.0185 | 0.0203 | 0.0000 |
| Floatation fat | C1-C2 for disposal | 0.0000 | 0.0000 | 0.0000 |
| Head | Human food | 0.0459 | 0.0447 | 0.0718 |
| Intestines | C1-C2 for disposal | 0.0000 | 0.0000 | 0.0000 |
| Kidney | Human food | 0.0030 | 0.0034 | 0.0025 |
| Manure | Spreading/Compost | 0.0000 | 0.0000 | 0.0000 |
| Meat | Human food | 0.5003 | 0.5654 | 0.8273 |
| Pluck | Human food | 0.0436 | 0.0452 | 0.0135 |
| Rumen and forestomach | Human food | 0.0043 | 0.0048 | 0.0002 |
| SPA C3 | PAP C3 | 0.0028 | 0.0703 | 0.0043 |
| Screening and sifting wastes | C1-C2 for disposal | 0.0000 | 0.0000 | 0.0000 |
| Skin | Skin tannery C3 | 0.0986 | 0.0799 | 0.0684 |
| Sludge | Spreading/Compost | 0.0000 | 0.0000 | 0.0000 |
| Spleen | Pet food | 0.0021 | 0.0023 | 0.0001 |
| Sweetbread | Human food | 0.0018 | 0.0016 | 0.0018 |

Table 51: Total weighting by coproducts for Croisé-lait Milk-fed Calf reared in Stall

| COPRODUCT | Destination | Croisé-lait /Milk-fed calf/Stall | | |
| --- | --- | --- | --- | --- |
|  |  | **Biophysical Cumulative Share** | **Mass Cumulative Share** | **Economic Cumulative Share** |
| Abomasum | Human food | 0.0046 | 0.0051 | 0.0002 |
| Aponevrosis (1%) | Human food | 0.0076 | 0.0071 | 0.0050 |
| Bile | PAP C3 | 0.0001 | 0.0004 | 0.0000 |
| Blood | C1-C2 for disposal | 0.0000 | 0.0000 | 0.0000 |
| Bones (11%) | Gelatin C3 | 0.0795 | 0.0777 | 0.0002 |
| Dead individuals | C1-C2 for disposal | 0.0000 | 0.0000 | 0.0000 |
| Fat (8%) | Fat and greaves C3 | 0.1520 | 0.0565 | 0.0036 |
| Fat from breasts and penis | Fat and greaves C3 | 0.0410 | 0.0152 | 0.0010 |
| Feet (without hooves) | Human food | 0.0184 | 0.0203 | 0.0000 |
| Floatation fat | C1-C2 for disposal | 0.0000 | 0.0000 | 0.0000 |
| Head | Human food | 0.0456 | 0.0447 | 0.0718 |
| Intestines | C1-C2 for disposal | 0.0000 | 0.0000 | 0.0000 |
| Kidney | Human food | 0.0030 | 0.0034 | 0.0025 |
| Manure | Spreading/Compost | 0.0000 | 0.0000 | 0.0000 |
| Meat | Human food | 0.4967 | 0.5654 | 0.8273 |
| Pluck | Human food | 0.0432 | 0.0452 | 0.0135 |
| Rumen and forestomach | Human food | 0.0043 | 0.0048 | 0.0002 |
| SPA C3 | PAP C3 | 0.0027 | 0.0703 | 0.0043 |
| Screening and sifting wastes | C1-C2 for disposal | 0.0000 | 0.0000 | 0.0000 |
| Skin | Skin tannery C3 | 0.0973 | 0.0799 | 0.0684 |
| Sludge | Spreading/Compost | 0.0000 | 0.0000 | 0.0000 |
| Spleen | Pet food | 0.0021 | 0.0023 | 0.0001 |
| Sweetbread | Human food | 0.0017 | 0.0016 | 0.0018 |

Table 52: Total weighting by coproducts for Croisé-viande Rosé Calf reared in Grazing Large Area

| COPRODUCT | Destination | Croisé-lait /rosé calf/grazing large area | | |
| --- | --- | --- | --- | --- |
|  |  | **Biophysical Cumulative Share** | **Mass Cumulative Share** | **Economic Cumulative Share** |
| Abomasum | Human food | 0.0047 | 0.0051 | 0.0002 |
| Aponevrosis (1%) | Human food | 0.0077 | 0.0071 | 0.0050 |
| Bile | PAP C3 | 0.0001 | 0.0004 | 0.0000 |
| Blood | C1-C2 for disposal | 0.0000 | 0.0000 | 0.0000 |
| Bones (11%) | Gelatin C3 | 0.0811 | 0.0777 | 0.0002 |
| Dead individuals | C1-C2 for disposal | 0.0000 | 0.0000 | 0.0000 |
| Fat (8%) | Fat and greaves C3 | 0.1435 | 0.0565 | 0.0036 |
| Fat from breasts and penis | Fat and greaves C3 | 0.0387 | 0.0152 | 0.0010 |
| Feet (without hooves) | Human food | 0.0185 | 0.0203 | 0.0000 |
| Floatation fat | C1-C2 for disposal | 0.0000 | 0.0000 | 0.0000 |
| Head | Human food | 0.0461 | 0.0447 | 0.0718 |
| Intestines | C1-C2 for disposal | 0.0000 | 0.0000 | 0.0000 |
| Kidney | Human food | 0.0030 | 0.0034 | 0.0025 |
| Manure | Spreading/Compost | 0.0000 | 0.0000 | 0.0000 |
| Meat | Human food | 0.5024 | 0.5654 | 0.8273 |
| Pluck | Human food | 0.0438 | 0.0452 | 0.0135 |
| Rumen and forestomach | Human food | 0.0043 | 0.0048 | 0.0002 |
| SPA C3 | PAP C3 | 0.0028 | 0.0703 | 0.0043 |
| Screening and sifting wastes | C1-C2 for disposal | 0.0000 | 0.0000 | 0.0000 |
| Skin | Skin tannery C3 | 0.0994 | 0.0799 | 0.0684 |
| Sludge | Spreading/Compost | 0.0000 | 0.0000 | 0.0000 |
| Spleen | Pet food | 0.0021 | 0.0023 | 0.0001 |
| Sweetbread | Human food | 0.0018 | 0.0016 | 0.0018 |

Table 53: Total weighting by coproducts for Croisé-viande Rosé Calf reared in Pasture

| COPRODUCT | Destination | Croisé-lait /Rosé calf/Pasture | | |
| --- | --- | --- | --- | --- |
|  |  | **Biophysical Cumulative Share** | **Mass Cumulative Share** | **Economic Cumulative Share** |
| Abomasum | Human food | 0.0046 | 0.0051 | 0.0002 |
| Aponevrosis (1%) | Human food | 0.0076 | 0.0071 | 0.0050 |
| Bile | PAP C3 | 0.0001 | 0.0004 | 0.0000 |
| Blood | C1-C2 for disposal | 0.0000 | 0.0000 | 0.0000 |
| Bones (11%) | Gelatin C3 | 0.0801 | 0.0777 | 0.0002 |
| Dead individuals | C1-C2 for disposal | 0.0000 | 0.0000 | 0.0000 |
| Fat (8%) | Fat and greaves C3 | 0.1490 | 0.0565 | 0.0036 |
| Fat from breasts and penis | Fat and greaves C3 | 0.0401 | 0.0152 | 0.0010 |
| Feet (without hooves) | Human food | 0.0184 | 0.0203 | 0.0000 |
| Floatation fat | C1-C2 for disposal | 0.0000 | 0.0000 | 0.0000 |
| Head | Human food | 0.0458 | 0.0447 | 0.0718 |
| Intestines | C1-C2 for disposal | 0.0000 | 0.0000 | 0.0000 |
| Kidney | Human food | 0.0030 | 0.0034 | 0.0025 |
| Manure | Spreading/Compost | 0.0000 | 0.0000 | 0.0000 |
| Meat | Human food | 0.4987 | 0.5654 | 0.8273 |
| Pluck | Human food | 0.0434 | 0.0452 | 0.0135 |
| Rumen and forestomach | Human food | 0.0043 | 0.0048 | 0.0002 |
| SPA C3 | PAP C3 | 0.0028 | 0.0703 | 0.0043 |
| Screening and sifting wastes | C1-C2 for disposal | 0.0000 | 0.0000 | 0.0000 |
| Skin | Skin tannery C3 | 0.0981 | 0.0799 | 0.0684 |
| Sludge | Spreading/Compost | 0.0000 | 0.0000 | 0.0000 |
| Spleen | Pet food | 0.0021 | 0.0023 | 0.0001 |
| Sweetbread | Human food | 0.0017 | 0.0016 | 0.0018 |

Table 54: Total weighting by coproducts for Croisé-viande Rosé Calf reared in Stall

| COPRODUCT | Destination | Croisé-lait /Rosé calf/Stall | | |
| --- | --- | --- | --- | --- |
|  |  | **Biophysical Cumulative Share** | **Mass Cumulative Share** | **Economic Cumulative Share** |
| Abomasum | Human food | 0.0046 | 0.0051 | 0.0002 |
| Aponevrosis (1%) | Human food | 0.0076 | 0.0071 | 0.0050 |
| Bile | PAP C3 | 0.0001 | 0.0004 | 0.0000 |
| Blood | C1-C2 for disposal | 0.0000 | 0.0000 | 0.0000 |
| Bones (11%) | Gelatin C3 | 0.0791 | 0.0777 | 0.0002 |
| Dead individuals | C1-C2 for disposal | 0.0000 | 0.0000 | 0.0000 |
| Fat (8%) | Fat and greaves C3 | 0.1546 | 0.0565 | 0.0036 |
| Fat from breasts and penis | Fat and greaves C3 | 0.0416 | 0.0152 | 0.0010 |
| Feet (without hooves) | Human food | 0.0183 | 0.0203 | 0.0000 |
| Floatation fat | C1-C2 for disposal | 0.0000 | 0.0000 | 0.0000 |
| Head | Human food | 0.0455 | 0.0447 | 0.0718 |
| Intestines | C1-C2 for disposal | 0.0000 | 0.0000 | 0.0000 |
| Kidney | Human food | 0.0029 | 0.0034 | 0.0025 |
| Manure | Spreading/Compost | 0.0000 | 0.0000 | 0.0000 |
| Meat | Human food | 0.4949 | 0.5654 | 0.8273 |
| Pluck | Human food | 0.0431 | 0.0452 | 0.0135 |
| Rumen and forestomach | Human food | 0.0043 | 0.0048 | 0.0002 |
| SPA C3 | PAP C3 | 0.0027 | 0.0703 | 0.0043 |
| Screening and sifting wastes | C1-C2 for disposal | 0.0000 | 0.0000 | 0.0000 |
| Skin | Skin tannery C3 | 0.0968 | 0.0799 | 0.0684 |
| Sludge | Spreading/Compost | 0.0000 | 0.0000 | 0.0000 |
| Spleen | Pet food | 0.0021 | 0.0023 | 0.0001 |
| Sweetbread | Human food | 0.0017 | 0.0016 | 0.0018 |
