# Supplementary material for: Allocation factors for meat coproducts: Dataset to perform life cycle assessment at slaughterhouse
Source: Data Brief. 2020 Nov 23;33:106558. doi: 10.1016/j.dib.2020.106558 (PMC7718151; doi:10.1016/j.dib.2020.106558)
Supplement: Supplementary file 6 [file mmc6.docx]

Table 1: Total weighting by destination category for Average Milk-fed Calf reared in Grazing Large Area

| destination | Average/milk-fed calf/grazing large area | | |
| --- | --- | --- | --- |
|  | **Biophysical Partition** | **Mass Partition** | **Economic Partition** |
| Pet Food | 0,0021 | 0,0023 | 0,0001 |
| PAP C3 | 0,0029 | 0,0707 | 0,0043 |
| Gelatin C3 | 0,0812 | 0,0777 | 0,0002 |
| C1-C2 for disposal | 0 | 0 | 0 |
| Skin tannery C3 | 0,0995 | 0,0799 | 0,0684 |
| Human food | 0,6326 | 0,6976 | 0,9223 |
| Fat and greaves C3 | 0,1816 | 0,0717 | 0,0046 |
| Spreading/Compost | 0 | 0 | 0 |

Table 2: Total weighting by destination category for Average Milk-fed Calf reared in Pasture

| destination | Average/milk-fed calf/Pasture | | |
| --- | --- | --- | --- |
|  | **Biophysical Partition** | **Mass Partition** | **Economic Partition** |
| Pet Food | 0,0021 | 0,0023 | 0,0001 |
| PAP C3 | 0,0029 | 0,0707 | 0,0043 |
| Gelatin C3 | 0,0802 | 0,0777 | 0,0002 |
| C1-C2 for disposal | 0 | 0 | 0 |
| Skin tannery C3 | 0,0982 | 0,0799 | 0,0684 |
| Human food | 0,628 | 0,6976 | 0,9223 |
| Fat and greaves C3 | 0,1885 | 0,0717 | 0,0046 |
| Spreading/Compost | 0 | 0 | 0 |

Table 3: Total weighting by destination category for Average Milk-fed Calf reared in Stall

| destination | Average/milk-fed calf/Stall | | |
| --- | --- | --- | --- |
|  | **Biophysical Partition** | **Mass Partition** | **Economic Partition** |
| Pet Food | 0,0021 | 0,0023 | 0,0001 |
| PAP C3 | 0,0028 | 0,0707 | 0,0043 |
| Gelatin C3 | 0,0792 | 0,0777 | 0,0002 |
| C1-C2 for disposal | 0 | 0 | 0 |
| Skin tannery C3 | 0,0969 | 0,0799 | 0,0684 |
| Human food | 0,6234 | 0,6976 | 0,9223 |
| Fat and greaves C3 | 0,1955 | 0,0717 | 0,0046 |
| Spreading/Compost | 0 | 0 | 0 |

Table 4: Total weighting by destination category for Average Rosé Calf reared in Grazing Large Area

| destination | Average/Rosé /grazing large area | | |
| --- | --- | --- | --- |
|  | **Biophysical Partition** | **Mass Partition** | **Economic Partition** |
| Pet Food | 0,0021 | 0,0023 | 0,0001 |
| PAP C3 | 0,0029 | 0,0707 | 0,0043 |
| Gelatin C3 | 0,0818 | 0,0777 | 0,0002 |
| C1-C2 for disposal | 0 | 0 | 0 |
| Skin tannery C3 | 0,1003 | 0,0799 | 0,0684 |
| Human food | 0,6362 | 0,6976 | 0,9223 |
| Fat and greaves C3 | 0,1767 | 0,0717 | 0,0046 |
| Spreading/Compost | 0 | 0 | 0 |

Table 5: Total weighting by destination category for Average Rosé Calf reared in Pasture

| destination | Average/Rosé /Pasture | | |
| --- | --- | --- | --- |
|  | **Biophysical Partition** | **Mass Partition** | **Economic Partition** |
| Pet Food | 0,0021 | 0,0023 | 0,0001 |
| PAP C3 | 0,0029 | 0,0707 | 0,0043 |
| Gelatin C3 | 0,0809 | 0,0777 | 0,0002 |
| C1-C2 for disposal | 0 | 0 | 0 |
| Skin tannery C3 | 0,0991 | 0,0799 | 0,0684 |
| Human food | 0,6319 | 0,6976 | 0,9223 |
| Fat and greaves C3 | 0,183 | 0,0717 | 0,0046 |
| Spreading/Compost | 0 | 0 | 0 |

Table 6: Total weighting by destination category for Average Rosé Calf reared in Stall

| destination | Average/Rosé calf/Stall | | |
| --- | --- | --- | --- |
|  | **Biophysical Partition** | **Mass Partition** | **Economic Partition** |
| Pet Food | 0,0021 | 0,0023 | 0,0001 |
| PAP C3 | 0,0029 | 0,0707 | 0,0043 |
| Gelatin C3 | 0,08 | 0,0777 | 0,0002 |
| C1-C2 for disposal | 0 | 0 | 0 |
| Skin tannery C3 | 0,0979 | 0,0799 | 0,0684 |
| Human food | 0,6275 | 0,6976 | 0,9223 |
| Fat and greaves C3 | 0,1895 | 0,0717 | 0,0046 |
| Spreading/Compost | 0 | 0 | 0 |

Table 7: Total weighting by destination category for Aubrac Milk-fed Calf reared in Grazing Large Area

| destination | Aubrac/milk-fed calf/grazing large area | | |
| --- | --- | --- | --- |
|  | **Biophysical Partition** | **Mass Partition** | **Economic Partition** |
| Pet Food | 0,0021 | 0,0023 | 0,0001 |
| PAP C3 | 0,0029 | 0,0707 | 0,0043 |
| Gelatin C3 | 0,081 | 0,0777 | 0,0002 |
| C1-C2 for disposal | 0 | 0 | 0 |
| Skin tannery C3 | 0,0992 | 0,0799 | 0,0684 |
| Human food | 0,6317 | 0,6976 | 0,9223 |
| Fat and greaves C3 | 0,183 | 0,0717 | 0,0046 |
| Spreading/Compost | 0 | 0 | 0 |

Table 8: Total weighting by destination category for Aubrac Milk-fed Calf reared in Pasture

| destination | Aubrac/milk-fed calf/Pasture | | |
| --- | --- | --- | --- |
|  | **Biophysical Partition** | **Mass Partition** | **Economic Partition** |
| Pet Food | 0,0021 | 0,0023 | 0,0001 |
| PAP C3 | 0,0029 | 0,0707 | 0,0043 |
| Gelatin C3 | 0,08 | 0,0777 | 0,0002 |
| C1-C2 for disposal | 0 | 0 | 0 |
| Skin tannery C3 | 0,098 | 0,0799 | 0,0684 |
| Human food | 0,6269 | 0,6976 | 0,9223 |
| Fat and greaves C3 | 0,19 | 0,0717 | 0,0046 |
| Spreading/Compost | 0 | 0 | 0 |

Table 9: Total weighting by destination category for Aubrac Milk-fed Calf reared in Stall

| destination | Aubrac/milk-fed calf/Stall | | |
| --- | --- | --- | --- |
|  | **Biophysical Partition** | **Mass Partition** | **Economic Partition** |
| Pet Food | 0,0021 | 0,0023 | 0,0001 |
| PAP C3 | 0,0028 | 0,0707 | 0,0043 |
| Gelatin C3 | 0,079 | 0,0777 | 0,0002 |
| C1-C2 for disposal | 0 | 0 | 0 |
| Skin tannery C3 | 0,0966 | 0,0799 | 0,0684 |
| Human food | 0,6221 | 0,6976 | 0,9223 |
| Fat and greaves C3 | 0,1971 | 0,0717 | 0,0046 |
| Spreading/Compost | 0 | 0 | 0 |

Table 10: Total weighting by destination category for Aubrac Rosé Calf reared in Grazing Large Area

| destination | Aubrac/Rosé /grazing large area | | |
| --- | --- | --- | --- |
|  | **Biophysical Partition** | **Mass Partition** | **Economic Partition** |
| Pet Food | 0,0021 | 0,0023 | 0,0001 |
| PAP C3 | 0,0029 | 0,0707 | 0,0043 |
| Gelatin C3 | 0,0817 | 0,0777 | 0,0002 |
| C1-C2 for disposal | 0 | 0 | 0 |
| Skin tannery C3 | 0,1 | 0,0799 | 0,0684 |
| Human food | 0,6351 | 0,6976 | 0,9223 |
| Fat and greaves C3 | 0,1781 | 0,0717 | 0,0046 |
| Spreading/Compost | 0 | 0 | 0 |

Table 11: Total weighting by destination category for Aubrac Rosé Calf reared in Pasture

| destination | Aubrac/Rosé /Pasture | | |
| --- | --- | --- | --- |
|  | **Biophysical Partition** | **Mass Partition** | **Economic Partition** |
| Pet Food | 0,0021 | 0,0023 | 0,0001 |
| PAP C3 | 0,0029 | 0,0707 | 0,0043 |
| Gelatin C3 | 0,0807 | 0,0777 | 0,0002 |
| C1-C2 for disposal | 0 | 0 | 0 |
| Skin tannery C3 | 0,0988 | 0,0799 | 0,0684 |
| Human food | 0,6309 | 0,6976 | 0,9223 |
| Fat and greaves C3 | 0,1846 | 0,0717 | 0,0046 |
| Spreading/Compost | 0 | 0 | 0 |

Table 12: Total weighting by destination category for Aubrac Rosé Calf reared in Stall

| destination | Aubrac/Rosé calf/Stall | | |
| --- | --- | --- | --- |
|  | **Biophysical Partition** | **Mass Partition** | **Economic Partition** |
| Pet Food | 0,0021 | 0,0023 | 0,0001 |
| PAP C3 | 0,0028 | 0,0707 | 0,0043 |
| Gelatin C3 | 0,0798 | 0,0777 | 0,0002 |
| C1-C2 for disposal | 0 | 0 | 0 |
| Skin tannery C3 | 0,0976 | 0,0799 | 0,0684 |
| Human food | 0,6263 | 0,6976 | 0,9223 |
| Fat and greaves C3 | 0,1913 | 0,0717 | 0,0046 |
| Spreading/Compost | 0 | 0 | 0 |

Table 13: Total weighting by destination category for Blonde d’Aquitaine Milk-fed Calf reared in Grazing Large Area

| destination | Blonde d’Aquitaine/milk-fed calf/grazing large area | | |
| --- | --- | --- | --- |
|  | **Biophysical Partition** | **Mass Partition** | **Economic Partition** |
| Pet Food | 0,0021 | 0,0023 | 0,0001 |
| PAP C3 | 0,0029 | 0,0707 | 0,0043 |
| Gelatin C3 | 0,0806 | 0,0777 | 0,0002 |
| C1-C2 for disposal | 0 | 0 | 0 |
| Skin tannery C3 | 0,0987 | 0,0799 | 0,0684 |
| Human food | 0,6296 | 0,6976 | 0,9223 |
| Fat and greaves C3 | 0,1862 | 0,0717 | 0,0046 |
| Spreading/Compost | 0 | 0 | 0 |

Table 14: Total weighting by destination category for Blonde d’Aquitaine Milk-fed Calf reared in Pasture

| destination | Blonde d’Aquitaine/milk-fed calf/Pasture | | |
| --- | --- | --- | --- |
|  | **Biophysical Partition** | **Mass Partition** | **Economic Partition** |
| Pet Food | 0,0021 | 0,0023 | 0,0001 |
| PAP C3 | 0,0028 | 0,0707 | 0,0043 |
| Gelatin C3 | 0,0796 | 0,0777 | 0,0002 |
| C1-C2 for disposal | 0 | 0 | 0 |
| Skin tannery C3 | 0,0974 | 0,0799 | 0,0684 |
| Human food | 0,6247 | 0,6976 | 0,9223 |
| Fat and greaves C3 | 0,1935 | 0,0717 | 0,0046 |
| Spreading/Compost | 0 | 0 | 0 |

Table 15: Total weighting by destination category for Blonde d’Aquitaine Milk-fed Calf reared in Stall

| destination | Blonde d’Aquitaine/milk-fed calf/Stall | | |
| --- | --- | --- | --- |
|  | **Biophysical Partition** | **Mass Partition** | **Economic Partition** |
| Pet Food | 0,0021 | 0,0023 | 0,0001 |
| PAP C3 | 0,0028 | 0,0707 | 0,0043 |
| Gelatin C3 | 0,0785 | 0,0777 | 0,0002 |
| C1-C2 for disposal | 0 | 0 | 0 |
| Skin tannery C3 | 0,096 | 0,0799 | 0,0684 |
| Human food | 0,6196 | 0,6976 | 0,9223 |
| Fat and greaves C3 | 0,2009 | 0,0717 | 0,0046 |
| Spreading/Compost | 0 | 0 | 0 |

Table 16: Total weighting by destination category for Blonde d’Aquitaine Rosé Calf reared in Grazing Large Area

| destination | Blonde d’Aquitaine/Rosé /grazing large area | | |
| --- | --- | --- | --- |
|  | **Biophysical Partition** | **Mass Partition** | **Economic Partition** |
| Pet Food | 0,0021 | 0,0023 | 0,0001 |
| PAP C3 | 0,0029 | 0,0707 | 0,0043 |
| Gelatin C3 | 0,0813 | 0,0777 | 0,0002 |
| C1-C2 for disposal | 0 | 0 | 0 |
| Skin tannery C3 | 0,0996 | 0,0799 | 0,0684 |
| Human food | 0,6331 | 0,6976 | 0,9223 |
| Fat and greaves C3 | 0,181 | 0,0717 | 0,0046 |
| Spreading/Compost | 0 | 0 | 0 |

Table 17: Total weighting by destination category for Blonde d’Aquitaine Rosé Calf reared in Pasture

| destination | Blonde d’Aquitaine/Rosé /Pasture | | |
| --- | --- | --- | --- |
|  | **Biophysical Partition** | **Mass Partition** | **Economic Partition** |
| Pet Food | 0,0021 | 0,0023 | 0,0001 |
| PAP C3 | 0,0029 | 0,0707 | 0,0043 |
| Gelatin C3 | 0,0803 | 0,0777 | 0,0002 |
| C1-C2 for disposal | 0 | 0 | 0 |
| Skin tannery C3 | 0,0983 | 0,0799 | 0,0684 |
| Human food | 0,6285 | 0,6976 | 0,9223 |
| Fat and greaves C3 | 0,1878 | 0,0717 | 0,0046 |
| Spreading/Compost | 0 | 0 | 0 |

Table 18: Total weighting by destination category for Blonde d’Aquitaine Rosé Calf reared in Stall

| destination | Blonde d’Aquitaine/Rosé calf/Stall | | |
| --- | --- | --- | --- |
|  | **Biophysical Partition** | **Mass Partition** | **Economic Partition** |
| Pet Food | 0,0021 | 0,0023 | 0,0001 |
| PAP C3 | 0,0028 | 0,0707 | 0,0043 |
| Gelatin C3 | 0,0793 | 0,0777 | 0,0002 |
| C1-C2 for disposal | 0 | 0 | 0 |
| Skin tannery C3 | 0,097 | 0,0799 | 0,0684 |
| Human food | 0,624 | 0,6976 | 0,9223 |
| Fat and greaves C3 | 0,1947 | 0,0717 | 0,0046 |
| Spreading/Compost | 0 | 0 | 0 |

Table 19: Total weighting by destination category for Charolais Milk-fed Calf reared in Grazing Large Area

| destination | Charolais/milk-fed calf/grazing large area | | |
| --- | --- | --- | --- |
|  | **Biophysical Partition** | **Mass Partition** | **Economic Partition** |
| Pet Food | 0,0021 | 0,0023 | 0,0001 |
| PAP C3 | 0,0029 | 0,0707 | 0,0043 |
| Gelatin C3 | 0,0812 | 0,0777 | 0,0002 |
| C1-C2 for disposal | 0 | 0 | 0 |
| Skin tannery C3 | 0,0995 | 0,0799 | 0,0684 |
| Human food | 0,6326 | 0,6976 | 0,9223 |
| Fat and greaves C3 | 0,1816 | 0,0717 | 0,0046 |
| Spreading/Compost | 0 | 0 | 0 |

Table 20: Total weighting by destination category for Charolais Milk-fed Calf reared in Pasture

| destination | Charolais/milk-fed calf/Pasture | | |
| --- | --- | --- | --- |
|  | **Biophysical Partition** | **Mass Partition** | **Economic Partition** |
| Pet Food | 0,0021 | 0,0023 | 0,0001 |
| PAP C3 | 0,0029 | 0,0707 | 0,0043 |
| Gelatin C3 | 0,0802 | 0,0777 | 0,0002 |
| C1-C2 for disposal | 0 | 0 | 0 |
| Skin tannery C3 | 0,0982 | 0,0799 | 0,0684 |
| Human food | 0,628 | 0,6976 | 0,9223 |
| Fat and greaves C3 | 0,1885 | 0,0717 | 0,0046 |
| Spreading/Compost | 0 | 0 | 0 |

Table 21: Total weighting by destination category for Charolais Milk-fed Calf reared in Stall

| destination | Charolais/milk-fed calf/Stall | | |
| --- | --- | --- | --- |
|  | **Biophysical Partition** | **Mass Partition** | **Economic Partition** |
| Pet Food | 0,0021 | 0,0023 | 0,0001 |
| PAP C3 | 0,0028 | 0,0707 | 0,0043 |
| Gelatin C3 | 0,0792 | 0,0777 | 0,0002 |
| C1-C2 for disposal | 0 | 0 | 0 |
| Skin tannery C3 | 0,0969 | 0,0799 | 0,0684 |
| Human food | 0,6234 | 0,6976 | 0,9223 |
| Fat and greaves C3 | 0,1955 | 0,0717 | 0,0046 |
| Spreading/Compost | 0 | 0 | 0 |

Table 22: Total weighting by destination category for Charolais Rosé Calf reared in Grazing Large Area

| destination | Charolais/Rosé /grazing large area | | |
| --- | --- | --- | --- |
|  | **Biophysical Partition** | **Mass Partition** | **Economic Partition** |
| Pet Food | 0,0021 | 0,0023 | 0,0001 |
| PAP C3 | 0,0029 | 0,0707 | 0,0043 |
| Gelatin C3 | 0,0818 | 0,0777 | 0,0002 |
| C1-C2 for disposal | 0 | 0 | 0 |
| Skin tannery C3 | 0,1003 | 0,0799 | 0,0684 |
| Human food | 0,6362 | 0,6976 | 0,9223 |
| Fat and greaves C3 | 0,1767 | 0,0717 | 0,0046 |
| Spreading/Compost | 0 | 0 | 0 |

Table 23: Total weighting by destination category for Charolais Rosé Calf reared in Pasture

| destination | Charolais/Rosé /Pasture | | |
| --- | --- | --- | --- |
|  | **Biophysical Partition** | **Mass Partition** | **Economic Partition** |
| Pet Food | 0,0021 | 0,0023 | 0,0001 |
| PAP C3 | 0,0029 | 0,0707 | 0,0043 |
| Gelatin C3 | 0,0809 | 0,0777 | 0,0002 |
| C1-C2 for disposal | 0 | 0 | 0 |
| Skin tannery C3 | 0,0991 | 0,0799 | 0,0684 |
| Human food | 0,6319 | 0,6976 | 0,9223 |
| Fat and greaves C3 | 0,183 | 0,0717 | 0,0046 |
| Spreading/Compost | 0 | 0 | 0 |

Table 24: Total weighting by destination category for Charolais Rosé Calf reared in Stall

| destination | Charolais/Rosé calf/Stall | | |
| --- | --- | --- | --- |
|  | **Biophysical Partition** | **Mass Partition** | **Economic Partition** |
| Pet Food | 0,0021 | 0,0023 | 0,0001 |
| PAP C3 | 0,0029 | 0,0707 | 0,0043 |
| Gelatin C3 | 0,08 | 0,0777 | 0,0002 |
| C1-C2 for disposal | 0 | 0 | 0 |
| Skin tannery C3 | 0,0979 | 0,0799 | 0,0684 |
| Human food | 0,6275 | 0,6976 | 0,9223 |
| Fat and greaves C3 | 0,1895 | 0,0717 | 0,0046 |
| Spreading/Compost | 0 | 0 | 0 |

Table 25: Total weighting by destination category for Limousine Milk-fed Calf reared in Grazing Large Area

| destination | Limousine/milk-fed calf/grazing large area | | |
| --- | --- | --- | --- |
|  | **Biophysical Partition** | **Mass Partition** | **Economic Partition** |
| Pet Food | 0,0021 | 0,0023 | 0,0001 |
| PAP C3 | 0,0029 | 0,0707 | 0,0043 |
| Gelatin C3 | 0,0809 | 0,0777 | 0,0002 |
| C1-C2 for disposal | 0 | 0 | 0 |
| Skin tannery C3 | 0,0991 | 0,0799 | 0,0684 |
| Human food | 0,6309 | 0,6976 | 0,9223 |
| Fat and greaves C3 | 0,1842 | 0,0717 | 0,0046 |
| Spreading/Compost | 0 | 0 | 0 |

Table 26: Total weighting by destination category for Limousine Milk-fed Calf reared in Pasture

| destination | Limousine/milk-fed calf/Pasture | | |
| --- | --- | --- | --- |
|  | **Biophysical Partition** | **Mass Partition** | **Economic Partition** |
| Pet Food | 0,0021 | 0,0023 | 0,0001 |
| PAP C3 | 0,0029 | 0,0707 | 0,0043 |
| Gelatin C3 | 0,0798 | 0,0777 | 0,0002 |
| C1-C2 for disposal | 0 | 0 | 0 |
| Skin tannery C3 | 0,0977 | 0,0799 | 0,0684 |
| Human food | 0,6261 | 0,6976 | 0,9223 |
| Fat and greaves C3 | 0,1913 | 0,0717 | 0,0046 |
| Spreading/Compost | 0 | 0 | 0 |

Table 27: Total weighting by destination category for Limousine Milk-fed Calf reared in Stall

| destination | Limousine/milk-fed calf/Stall | | |
| --- | --- | --- | --- |
|  | **Biophysical Partition** | **Mass Partition** | **Economic Partition** |
| Pet Food | 0,0021 | 0,0023 | 0,0001 |
| PAP C3 | 0,0028 | 0,0707 | 0,0043 |
| Gelatin C3 | 0,0788 | 0,0777 | 0,0002 |
| C1-C2 for disposal | 0 | 0 | 0 |
| Skin tannery C3 | 0,0964 | 0,0799 | 0,0684 |
| Human food | 0,6212 | 0,6976 | 0,9223 |
| Fat and greaves C3 | 0,1985 | 0,0717 | 0,0046 |
| Spreading/Compost | 0 | 0 | 0 |

Table 28: Total weighting by destination category for Limousine Rosé Calf reared in Grazing Large Area

| destination | Limousine/Rosé /grazing large area | | |
| --- | --- | --- | --- |
|  | **Biophysical Partition** | **Mass Partition** | **Economic Partition** |
| Pet Food | 0,0021 | 0,0023 | 0,0001 |
| PAP C3 | 0,0029 | 0,0707 | 0,0043 |
| Gelatin C3 | 0,0815 | 0,0777 | 0,0002 |
| C1-C2 for disposal | 0 | 0 | 0 |
| Skin tannery C3 | 0,0999 | 0,0799 | 0,0684 |
| Human food | 0,6343 | 0,6976 | 0,9223 |
| Fat and greaves C3 | 0,1791 | 0,0717 | 0,0046 |
| Spreading/Compost | 0 | 0 | 0 |

Table 29: Total weighting by destination category for Limousine Rosé Calf reared in Pasture

| destination | Limousine/Rosé /Pasture | | |
| --- | --- | --- | --- |
|  | **Biophysical Partition** | **Mass Partition** | **Economic Partition** |
| Pet Food | 0,0021 | 0,0023 | 0,0001 |
| PAP C3 | 0,0029 | 0,0707 | 0,0043 |
| Gelatin C3 | 0,0806 | 0,0777 | 0,0002 |
| C1-C2 for disposal | 0 | 0 | 0 |
| Skin tannery C3 | 0,0987 | 0,0799 | 0,0684 |
| Human food | 0,6302 | 0,6976 | 0,9223 |
| Fat and greaves C3 | 0,1857 | 0,0717 | 0,0046 |
| Spreading/Compost | 0 | 0 | 0 |

Table 30: Total weighting by destination category for Limousine Rosé Calf reared in Stall

| destination | Limousine/Rosé calf/Stall | | |
| --- | --- | --- | --- |
|  | **Biophysical Partition** | **Mass Partition** | **Economic Partition** |
| Pet Food | 0,0021 | 0,0023 | 0,0001 |
| PAP C3 | 0,0028 | 0,0707 | 0,0043 |
| Gelatin C3 | 0,0796 | 0,0777 | 0,0002 |
| C1-C2 for disposal | 0 | 0 | 0 |
| Skin tannery C3 | 0,0974 | 0,0799 | 0,0684 |
| Human food | 0,6256 | 0,6976 | 0,9223 |
| Fat and greaves C3 | 0,1926 | 0,0717 | 0,0046 |
| Spreading/Compost | 0 | 0 | 0 |

Table 31: Total weighting by destination category for Montbéliarde Milk-fed Calf reared in Grazing Large Area

| destination | Montbéliarde/milk-fed calf/grazing large area | | |
| --- | --- | --- | --- |
|  | **Biophysical Partition** | **Mass Partition** | **Economic Partition** |
| Pet Food | 0,0021 | 0,0023 | 0,0001 |
| PAP C3 | 0,0029 | 0,0707 | 0,0043 |
| Gelatin C3 | 0,0817 | 0,0777 | 0,0002 |
| C1-C2 for disposal | 0 | 0 | 0 |
| Skin tannery C3 | 0,1001 | 0,0799 | 0,0684 |
| Human food | 0,6352 | 0,6976 | 0,9223 |
| Fat and greaves C3 | 0,178 | 0,0717 | 0,0046 |
| Spreading/Compost | 0 | 0 | 0 |

Table 32: Total weighting by destination category for Montbéliarde Milk-fed Calf reared in Pasture

| destination | Montbéliarde/milk-fed calf/Pasture | | |
| --- | --- | --- | --- |
|  | **Biophysical Partition** | **Mass Partition** | **Economic Partition** |
| Pet Food | 0,0021 | 0,0023 | 0,0001 |
| PAP C3 | 0,0029 | 0,0707 | 0,0043 |
| Gelatin C3 | 0,0807 | 0,0777 | 0,0002 |
| C1-C2 for disposal | 0 | 0 | 0 |
| Skin tannery C3 | 0,0989 | 0,0799 | 0,0684 |
| Human food | 0,631 | 0,6976 | 0,9223 |
| Fat and greaves C3 | 0,1844 | 0,0717 | 0,0046 |
| Spreading/Compost | 0 | 0 | 0 |

Table 33: Total weighting by destination category for Montbéliarde Milk-fed Calf reared in Stall

| destination | Montbéliarde/milk-fed calf/Stall | | |
| --- | --- | --- | --- |
|  | **Biophysical Partition** | **Mass Partition** | **Economic Partition** |
| Pet Food | 0,0021 | 0,0023 | 0,0001 |
| PAP C3 | 0,0028 | 0,0707 | 0,0043 |
| Gelatin C3 | 0,0798 | 0,0777 | 0,0002 |
| C1-C2 for disposal | 0 | 0 | 0 |
| Skin tannery C3 | 0,0977 | 0,0799 | 0,0684 |
| Human food | 0,6264 | 0,6976 | 0,9223 |
| Fat and greaves C3 | 0,191 | 0,0717 | 0,0046 |
| Spreading/Compost | 0 | 0 | 0 |

Table 34: Total weighting by destination category for Montbéliarde Rosé Calf reared in Grazing Large Area

| destination | Montbéliarde/Rosé /grazing large area | | |
| --- | --- | --- | --- |
|  | **Biophysical Partition** | **Mass Partition** | **Economic Partition** |
| Pet Food | 0,0021 | 0,0023 | 0,0001 |
| PAP C3 | 0,003 | 0,0707 | 0,0043 |
| Gelatin C3 | 0,0823 | 0,0777 | 0,0002 |
| C1-C2 for disposal | 0 | 0 | 0 |
| Skin tannery C3 | 0,1008 | 0,0799 | 0,0684 |
| Human food | 0,6386 | 0,6976 | 0,9223 |
| Fat and greaves C3 | 0,1733 | 0,0717 | 0,0046 |
| Spreading/Compost | 0 | 0 | 0 |

Table 35: Total weighting by destination category for Montbéliarde Rosé Calf reared in Pasture

| destination | Montbéliarde/Rosé /Pasture | | |
| --- | --- | --- | --- |
|  | **Biophysical Partition** | **Mass Partition** | **Economic Partition** |
| Pet Food | 0,0021 | 0,0023 | 0,0001 |
| PAP C3 | 0,0029 | 0,0707 | 0,0043 |
| Gelatin C3 | 0,0814 | 0,0777 | 0,0002 |
| C1-C2 for disposal | 0 | 0 | 0 |
| Skin tannery C3 | 0,0997 | 0,0799 | 0,0684 |
| Human food | 0,6345 | 0,6976 | 0,9223 |
| Fat and greaves C3 | 0,1794 | 0,0717 | 0,0046 |
| Spreading/Compost | 0 | 0 | 0 |

Table 36: Total weighting by destination category for Montbéliarde Rosé Calf reared in Stall

| destination | Montbéliarde/Rosé calf/Stall | | |
| --- | --- | --- | --- |
|  | **Biophysical Partition** | **Mass Partition** | **Economic Partition** |
| Pet Food | 0,0021 | 0,0023 | 0,0001 |
| PAP C3 | 0,0029 | 0,0707 | 0,0043 |
| Gelatin C3 | 0,0805 | 0,0777 | 0,0002 |
| C1-C2 for disposal | 0 | 0 | 0 |
| Skin tannery C3 | 0,0986 | 0,0799 | 0,0684 |
| Human food | 0,6305 | 0,6976 | 0,9223 |
| Fat and greaves C3 | 0,1855 | 0,0717 | 0,0046 |
| Spreading/Compost | 0 | 0 | 0 |

Table 37: Total weighting by destination category for Normande Milk-fed Calf reared in Grazing Large Area

| destination | Normande/milk-fed calf/grazing large area | | |
| --- | --- | --- | --- |
|  | **Biophysical Partition** | **Mass Partition** | **Economic Partition** |
| Pet Food | 0,0021 | 0,0023 | 0,0001 |
| PAP C3 | 0,0029 | 0,0707 | 0,0043 |
| Gelatin C3 | 0,0817 | 0,0777 | 0,0002 |
| C1-C2 for disposal | 0 | 0 | 0 |
| Skin tannery C3 | 0,1 | 0,0799 | 0,0684 |
| Human food | 0,6351 | 0,6976 | 0,9223 |
| Fat and greaves C3 | 0,1781 | 0,0717 | 0,0046 |
| Spreading/Compost | 0 | 0 | 0 |

Table 38: Total weighting by destination category for Normande Milk-fed Calf reared in Pasture

| destination | Normande/milk-fed calf/Pasture | | |
| --- | --- | --- | --- |
|  | **Biophysical Partition** | **Mass Partition** | **Economic Partition** |
| Pet Food | 0,0021 | 0,0023 | 0,0001 |
| PAP C3 | 0,0029 | 0,0707 | 0,0043 |
| Gelatin C3 | 0,0807 | 0,0777 | 0,0002 |
| C1-C2 for disposal | 0 | 0 | 0 |
| Skin tannery C3 | 0,0988 | 0,0799 | 0,0684 |
| Human food | 0,6309 | 0,6976 | 0,9223 |
| Fat and greaves C3 | 0,1846 | 0,0717 | 0,0046 |
| Spreading/Compost | 0 | 0 | 0 |

Table 39: Total weighting by destination category for Normande Milk-fed Calf reared in Stall

| destination | Normande/milk-fed calf/Stall | | |
| --- | --- | --- | --- |
|  | **Biophysical Partition** | **Mass Partition** | **Economic Partition** |
| Pet Food | 0,0021 | 0,0023 | 0,0001 |
| PAP C3 | 0,0028 | 0,0707 | 0,0043 |
| Gelatin C3 | 0,0798 | 0,0777 | 0,0002 |
| C1-C2 for disposal | 0 | 0 | 0 |
| Skin tannery C3 | 0,0976 | 0,0799 | 0,0684 |
| Human food | 0,6263 | 0,6976 | 0,9223 |
| Fat and greaves C3 | 0,1913 | 0,0717 | 0,0046 |
| Spreading/Compost | 0 | 0 | 0 |

Table 40: Total weighting by destination category for Normande Rosé Calf reared in Grazing Large Area

| destination | Normande/Rosé /grazing large area | | |
| --- | --- | --- | --- |
|  | **Biophysical Partition** | **Mass Partition** | **Economic Partition** |
| Pet Food | 0,0021 | 0,0023 | 0,0001 |
| PAP C3 | 0,003 | 0,0707 | 0,0043 |
| Gelatin C3 | 0,0822 | 0,0777 | 0,0002 |
| C1-C2 for disposal | 0 | 0 | 0 |
| Skin tannery C3 | 0,1008 | 0,0799 | 0,0684 |
| Human food | 0,6385 | 0,6976 | 0,9223 |
| Fat and greaves C3 | 0,1735 | 0,0717 | 0,0046 |
| Spreading/Compost | 0 | 0 | 0 |

Table 41: Total weighting by destination category for Normande Rosé Calf reared in Pasture

| destination | Normande/Rosé /Pasture | | |
| --- | --- | --- | --- |
|  | **Biophysical Partition** | **Mass Partition** | **Economic Partition** |
| Pet Food | 0,0021 | 0,0023 | 0,0001 |
| PAP C3 | 0,0029 | 0,0707 | 0,0043 |
| Gelatin C3 | 0,0814 | 0,0777 | 0,0002 |
| C1-C2 for disposal | 0 | 0 | 0 |
| Skin tannery C3 | 0,0997 | 0,0799 | 0,0684 |
| Human food | 0,6343 | 0,6976 | 0,9223 |
| Fat and greaves C3 | 0,1795 | 0,0717 | 0,0046 |
| Spreading/Compost | 0 | 0 | 0 |

Table 42: Total weighting by destination category for Normande Rosé Calf reared in Stall

| destination | Normande/Rosé calf/Stall | | |
| --- | --- | --- | --- |
|  | **Biophysical Partition** | **Mass Partition** | **Economic Partition** |
| Pet Food | 0,0021 | 0,0023 | 0,0001 |
| PAP C3 | 0,0029 | 0,0707 | 0,0043 |
| Gelatin C3 | 0,0805 | 0,0777 | 0,0002 |
| C1-C2 for disposal | 0 | 0 | 0 |
| Skin tannery C3 | 0,0985 | 0,0799 | 0,0684 |
| Human food | 0,6304 | 0,6976 | 0,9223 |
| Fat and greaves C3 | 0,1857 | 0,0717 | 0,0046 |
| Spreading/Compost | 0 | 0 | 0 |

Table 43: Total weighting by destination category for Primholstein Milk-fed Calf reared in Grazing Large Area

| destination | Primholstein/milk-fed calf/grazing large area | | |
| --- | --- | --- | --- |
|  | **Biophysical Partition** | **Mass Partition** | **Economic Partition** |
| Pet Food | 0,0021 | 0,0023 | 0,0001 |
| PAP C3 | 0,0029 | 0,0707 | 0,0043 |
| Gelatin C3 | 0,0815 | 0,0777 | 0,0002 |
| C1-C2 for disposal | 0 | 0 | 0 |
| Skin tannery C3 | 0,0999 | 0,0799 | 0,0684 |
| Human food | 0,6343 | 0,6976 | 0,9223 |
| Fat and greaves C3 | 0,1791 | 0,0717 | 0,0046 |
| Spreading/Compost | 0 | 0 | 0 |

Table 44: Total weighting by destination category for Primholstein Milk-fed Calf reared in Pasture

| destination | Primholstein/milk-fed calf/Pasture | | |
| --- | --- | --- | --- |
|  | **Biophysical Partition** | **Mass Partition** | **Economic Partition** |
| Pet Food | 0,0021 | 0,0023 | 0,0001 |
| PAP C3 | 0,0029 | 0,0707 | 0,0043 |
| Gelatin C3 | 0,0806 | 0,0777 | 0,0002 |
| C1-C2 for disposal | 0 | 0 | 0 |
| Skin tannery C3 | 0,0987 | 0,0799 | 0,0684 |
| Human food | 0,6302 | 0,6976 | 0,9223 |
| Fat and greaves C3 | 0,1857 | 0,0717 | 0,0046 |
| Spreading/Compost | 0 | 0 | 0 |

Table 45: Total weighting by destination category for Primholstein Milk-fed Calf reared in Stall

| destination | Primholstein/milk-fed calf/Stall | | |
| --- | --- | --- | --- |
|  | **Biophysical Partition** | **Mass Partition** | **Economic Partition** |
| Pet Food | 0,0021 | 0,0023 | 0,0001 |
| PAP C3 | 0,0028 | 0,0707 | 0,0043 |
| Gelatin C3 | 0,0796 | 0,0777 | 0,0002 |
| C1-C2 for disposal | 0 | 0 | 0 |
| Skin tannery C3 | 0,0974 | 0,0799 | 0,0684 |
| Human food | 0,6256 | 0,6976 | 0,9223 |
| Fat and greaves C3 | 0,1926 | 0,0717 | 0,0046 |
| Spreading/Compost | 0 | 0 | 0 |

Table 46: Total weighting by destination category for Primholstein Rosé Calf reared in Grazing Large Area

| destination | Primholstein/Rosé /grazing large area | | |
| --- | --- | --- | --- |
|  | **Biophysical Partition** | **Mass Partition** | **Economic Partition** |
| Pet Food | 0,0021 | 0,0023 | 0,0001 |
| PAP C3 | 0,003 | 0,0707 | 0,0043 |
| Gelatin C3 | 0,0821 | 0,0777 | 0,0002 |
| C1-C2 for disposal | 0 | 0 | 0 |
| Skin tannery C3 | 0,1007 | 0,0799 | 0,0684 |
| Human food | 0,638 | 0,6976 | 0,9223 |
| Fat and greaves C3 | 0,1743 | 0,0717 | 0,0046 |
| Spreading/Compost | 0 | 0 | 0 |

Table 47: Total weighting by destination category for Primholstein Rosé Calf reared in Pasture

| destination | Primholstein/Rosé /Pasture | | |
| --- | --- | --- | --- |
|  | **Biophysical Partition** | **Mass Partition** | **Economic Partition** |
| Pet Food | 0,0021 | 0,0023 | 0,0001 |
| PAP C3 | 0,0029 | 0,0707 | 0,0043 |
| Gelatin C3 | 0,0812 | 0,0777 | 0,0002 |
| C1-C2 for disposal | 0 | 0 | 0 |
| Skin tannery C3 | 0,0995 | 0,0799 | 0,0684 |
| Human food | 0,6337 | 0,6976 | 0,9223 |
| Fat and greaves C3 | 0,1805 | 0,0717 | 0,0046 |
| Spreading/Compost | 0 | 0 | 0 |

Table 48: Total weighting by destination category for Primholstein Rosé Calf reared in Stall

| destination | Primholstein/Rosé calf/Stall | | |
| --- | --- | --- | --- |
|  | **Biophysical Partition** | **Mass Partition** | **Economic Partition** |
| Pet Food | 0,0021 | 0,0023 | 0,0001 |
| PAP C3 | 0,0029 | 0,0707 | 0,0043 |
| Gelatin C3 | 0,0803 | 0,0777 | 0,0002 |
| C1-C2 for disposal | 0 | 0 | 0 |
| Skin tannery C3 | 0,0984 | 0,0799 | 0,0684 |
| Human food | 0,6296 | 0,6976 | 0,9223 |
| Fat and greaves C3 | 0,1867 | 0,0717 | 0,0046 |
| Spreading/Compost | 0 | 0 | 0 |

Table 49: Total weighting by destination category for Croisé-lait Milk-fed Calf reared in Grazing Large Area

| destination | Croisé-lait/milk-fed calf/grazing large area | | |
| --- | --- | --- | --- |
|  | **Biophysical Partition** | **Mass Partition** | **Economic Partition** |
| Pet Food | 0,0021 | 0,0023 | 0,0001 |
| PAP C3 | 0,0029 | 0,0707 | 0,0043 |
| Gelatin C3 | 0,0815 | 0,0777 | 0,0002 |
| C1-C2 for disposal | 0 | 0 | 0 |
| Skin tannery C3 | 0,0998 | 0,0799 | 0,0684 |
| Human food | 0,634 | 0,6976 | 0,9223 |
| Fat and greaves C3 | 0,1796 | 0,0717 | 0,0046 |
| Spreading/Compost | 0 | 0 | 0 |

Table 50: Total weighting by destination category for Croisé-lait Milk-fed Calf reared in Pasture

| destination | Croisé-lait/milk-fed calf/Pasture | | |
| --- | --- | --- | --- |
|  | **Biophysical Partition** | **Mass Partition** | **Economic Partition** |
| Pet Food | 0,0021 | 0,0023 | 0,0001 |
| PAP C3 | 0,0029 | 0,0707 | 0,0043 |
| Gelatin C3 | 0,0805 | 0,0777 | 0,0002 |
| C1-C2 for disposal | 0 | 0 | 0 |
| Skin tannery C3 | 0,0986 | 0,0799 | 0,0684 |
| Human food | 0,6297 | 0,6976 | 0,9223 |
| Fat and greaves C3 | 0,1862 | 0,0717 | 0,0046 |
| Spreading/Compost | 0 | 0 | 0 |

Table 51: Total weighting by destination category for Croisé-lait Milk-fed Calf reared in Stall

| destination | Croisé-lait/milk-fed calf/Stall | | |
| --- | --- | --- | --- |
|  | **Biophysical Partition** | **Mass Partition** | **Economic Partition** |
| Pet Food | 0,0021 | 0,0023 | 0,0001 |
| PAP C3 | 0,0028 | 0,0707 | 0,0043 |
| Gelatin C3 | 0,0795 | 0,0777 | 0,0002 |
| C1-C2 for disposal | 0 | 0 | 0 |
| Skin tannery C3 | 0,0973 | 0,0799 | 0,0684 |
| Human food | 0,6251 | 0,6976 | 0,9223 |
| Fat and greaves C3 | 0,193 | 0,0717 | 0,0046 |
| Spreading/Compost | 0 | 0 | 0 |

Table 52: Total weighting by destination category for Croisé-viande Rosé Calf reared in Grazing Large Area

| destination | Croisé-viande/Rosé /grazing large area | | |
| --- | --- | --- | --- |
|  | **Biophysical Partition** | **Mass Partition** | **Economic Partition** |
| Pet Food | 0,0021 | 0,0023 | 0,0001 |
| PAP C3 | 0,0029 | 0,0707 | 0,0043 |
| Gelatin C3 | 0,0811 | 0,0777 | 0,0002 |
| C1-C2 for disposal | 0 | 0 | 0 |
| Skin tannery C3 | 0,0994 | 0,0799 | 0,0684 |
| Human food | 0,6323 | 0,6976 | 0,9223 |
| Fat and greaves C3 | 0,1822 | 0,0717 | 0,0046 |
| Spreading/Compost | 0 | 0 | 0 |

Table 53: Total weighting by destination category for Croisé-viande Rosé Calf reared in Pasture

| destination | Croisé-viande/Rosé /Pasture | | |
| --- | --- | --- | --- |
|  | **Biophysical Partition** | **Mass Partition** | **Economic Partition** |
| Pet Food | 0,0021 | 0,0023 | 0,0001 |
| PAP C3 | 0,0029 | 0,0707 | 0,0043 |
| Gelatin C3 | 0,0801 | 0,0777 | 0,0002 |
| C1-C2 for disposal | 0 | 0 | 0 |
| Skin tannery C3 | 0,0981 | 0,0799 | 0,0684 |
| Human food | 0,6275 | 0,6976 | 0,9223 |
| Fat and greaves C3 | 0,1891 | 0,0717 | 0,0046 |
| Spreading/Compost | 0 | 0 | 0 |

Table 54: Total weighting by destination category for Croisé-viande Rosé Calf reared in Stall

| destination | Croisé-viande/Rosé calf/Stall | | |
| --- | --- | --- | --- |
|  | **Biophysical Partition** | **Mass Partition** | **Economic Partition** |
| Pet Food | 0,0021 | 0,0023 | 0,0001 |
| PAP C3 | 0,0028 | 0,0707 | 0,0043 |
| Gelatin C3 | 0,0791 | 0,0777 | 0,0002 |
| C1-C2 for disposal | 0 | 0 | 0 |
| Skin tannery C3 | 0,0968 | 0,0799 | 0,0684 |
| Human food | 0,6229 | 0,6976 | 0,9223 |
| Fat and greaves C3 | 0,1962 | 0,0717 | 0,0046 |
| Spreading/Compost | 0 | 0 | 0 |
