# Supplementary material for: Allocation factors for meat coproducts: Dataset to perform life cycle assessment at slaughterhouse
Source: Data Brief. 2020 Nov 23;33:106558. doi: 10.1016/j.dib.2020.106558 (PMC7718151; doi:10.1016/j.dib.2020.106558)
Supplement: Supplementary file 7 [file mmc7.docx]

Table 1: Allocation factors for Average Ovine reared in Grazing Flat Pasture

| COPRODUCT | Destination | Average/Ovine/grazing Flat Pasture | | |
| --- | --- | --- | --- | --- |
|  |  | **Biophysical Allocation Factor** | **Mass Allocation Factor** | **Economic Allocation Factor** |
| Blood | PAP C3 | 0.0131 | 0.0268 | 0.0000 |
| Blood | Spreading/Compost | 0.0000 | 0.0000 | 0.0000 |
| Bones | PAP C3 | 0.0128 | 0.0268 | 0.0000 |
| Brain | Human food | 0.0087 | 0.0268 | 0.0000 |
| Contents of the intestines | Spreading/Compost | 0.0000 | 0.0000 | 0.0000 |
| Dead individuals | C1-C2 for disposal | 0.0000 | 0.0000 | 0.0000 |
| Downgraded skin | PAP C3 | 0.0249 | 0.0268 | 0.0000 |
| Fat | PAP C3 | 0.0109 | 0.0268 | 0.0000 |
| Floatation fat | C1-C2 for disposal | 0.0000 | 0.0000 | 0.0000 |
| Meat | Human food | 0.0181 | 0.0268 | 0.0511 |
| Other spa c1 | C1-C2 for disposal | 0.0000 | 0.0000 | 0.0000 |
| Other spa c3 | PAP C3 | 0.0234 | 0.0268 | 0.0000 |
| Pluck (liver, heart, trachea) | Human food | 0.1304 | 0.0268 | 0.0299 |
| Pluck (liver, heart, trachea) | Pet food | 0.1334 | 0.0268 | 0.0297 |
| Rumen and reticulum | Human food | 0.1246 | 0.0268 | 0.0000 |
| Rumen and reticulum | Pet food | 0.1274 | 0.0268 | 0.0000 |
| Sanitary seizures | C1-C2 for disposal | 0.0000 | 0.0000 | 0.0000 |
| Screening waste | C1-C2 for disposal | 0.0000 | 0.0000 | 0.0000 |
| Sifting waste | C1-C2 for disposal | 0.0000 | 0.0000 | 0.0000 |
| Skin | Skin tannery C3 | 0.0329 | 0.0268 | 0.0067 |
| Small intestine | C1-C2 for disposal | 0.0000 | 0.0000 | 0.0000 |
| Small intestine | Human food | 0.1450 | 0.0268 | 0.0000 |
| Small intestine | PAP C3 | 0.1481 | 0.0268 | 0.0000 |
| Stercoral matter | Spreading/Compost | 0.0000 | 0.0000 | 0.0000 |
| Thymus | Human food | 0.0307 | 0.0268 | 0.0984 |
| Thymus | Pet food | 0.0313 | 0.0268 | 0.0984 |
| Tongue | Human food | 0.0211 | 0.0268 | 0.0434 |

Table 2: Allocation factors for Average Ovine reared in Grazing Hilly Pasture

| COPRODUCT | Destination | Average/Ovine/grazing Hilly Pasture | | |
| --- | --- | --- | --- | --- |
|  |  | **Biophysical Allocation Factor** | **Mass Allocation Factor** | **Economic Allocation Factor** |
| Blood | PAP C3 | 0.0127 | 0.0261 | 0.0000 |
| Blood | Spreading/Compost | 0.0000 | 0.0000 | 0.0000 |
| Bones | PAP C3 | 0.0125 | 0.0261 | 0.0000 |
| Brain | Human food | 0.0085 | 0.0261 | 0.0000 |
| Contents of the intestines | Spreading/Compost | 0.0000 | 0.0000 | 0.0000 |
| Dead individuals | C1-C2 for disposal | 0.0000 | 0.0000 | 0.0000 |
| Downgraded skin | PAP C3 | 0.0242 | 0.0261 | 0.0000 |
| Fat | PAP C3 | 0.0106 | 0.0261 | 0.0000 |
| Floatation fat | C1-C2 for disposal | 0.0000 | 0.0000 | 0.0000 |
| Meat | Human food | 0.0176 | 0.0261 | 0.0498 |
| Other spa c1 | C1-C2 for disposal | 0.0000 | 0.0000 | 0.0000 |
| Other spa c3 | PAP C3 | 0.0228 | 0.0261 | 0.0000 |
| Pluck (liver, heart, trachea) | Human food | 0.1272 | 0.0261 | 0.0291 |
| Pluck (liver, heart, trachea) | Pet food | 0.1301 | 0.0261 | 0.0289 |
| Rumen and reticulum | Human food | 0.1215 | 0.0261 | 0.0000 |
| Rumen and reticulum | Pet food | 0.1242 | 0.0261 | 0.0000 |
| Sanitary seizures | C1-C2 for disposal | 0.0000 | 0.0000 | 0.0000 |
| Screening waste | C1-C2 for disposal | 0.0000 | 0.0000 | 0.0000 |
| Sifting waste | C1-C2 for disposal | 0.0000 | 0.0000 | 0.0000 |
| Skin | Skin tannery C3 | 0.0321 | 0.0261 | 0.0066 |
| Small intestine | C1-C2 for disposal | 0.0000 | 0.0000 | 0.0000 |
| Small intestine | Human food | 0.1414 | 0.0261 | 0.0000 |
| Small intestine | PAP C3 | 0.1444 | 0.0261 | 0.0000 |
| Stercoral matter | Spreading/Compost | 0.0000 | 0.0000 | 0.0000 |
| Thymus | Human food | 0.0299 | 0.0261 | 0.0958 |
| Thymus | Pet food | 0.0305 | 0.0261 | 0.0958 |
| Tongue | Human food | 0.0206 | 0.0261 | 0.0423 |

Table 3: Allocation factors for Average Ovine reared in Housed Ewes

| COPRODUCT | Destination | Average/Ovine/Housed Ewes | | |
| --- | --- | --- | --- | --- |
|  |  | **Biophysical Allocation Factor** | **Mass Allocation Factor** | **Economic Allocation Factor** |
| Blood | PAP C3 | 0.0127 | 0.0261 | 0.0000 |
| Blood | Spreading/Compost | 0.0000 | 0.0000 | 0.0000 |
| Bones | PAP C3 | 0.0125 | 0.0261 | 0.0000 |
| Brain | Human food | 0.0085 | 0.0261 | 0.0000 |
| Contents of the intestines | Spreading/Compost | 0.0000 | 0.0000 | 0.0000 |
| Dead individuals | C1-C2 for disposal | 0.0000 | 0.0000 | 0.0000 |
| Downgraded skin | PAP C3 | 0.0242 | 0.0261 | 0.0000 |
| Fat | PAP C3 | 0.0106 | 0.0261 | 0.0000 |
| Floatation fat | C1-C2 for disposal | 0.0000 | 0.0000 | 0.0000 |
| Meat | Human food | 0.0176 | 0.0261 | 0.0498 |
| Other spa c1 | C1-C2 for disposal | 0.0000 | 0.0000 | 0.0000 |
| Other spa c3 | PAP C3 | 0.0228 | 0.0261 | 0.0000 |
| Pluck (liver, heart, trachea) | Human food | 0.1272 | 0.0261 | 0.0291 |
| Pluck (liver, heart, trachea) | Pet food | 0.1301 | 0.0261 | 0.0289 |
| Rumen and reticulum | Human food | 0.1215 | 0.0261 | 0.0000 |
| Rumen and reticulum | Pet food | 0.1242 | 0.0261 | 0.0000 |
| Sanitary seizures | C1-C2 for disposal | 0.0000 | 0.0000 | 0.0000 |
| Screening waste | C1-C2 for disposal | 0.0000 | 0.0000 | 0.0000 |
| Sifting waste | C1-C2 for disposal | 0.0000 | 0.0000 | 0.0000 |
| Skin | Skin tannery C3 | 0.0321 | 0.0261 | 0.0066 |
| Small intestine | C1-C2 for disposal | 0.0000 | 0.0000 | 0.0000 |
| Small intestine | Human food | 0.1414 | 0.0261 | 0.0000 |
| Small intestine | PAP C3 | 0.1444 | 0.0261 | 0.0000 |
| Stercoral matter | Spreading/Compost | 0.0000 | 0.0000 | 0.0000 |
| Thymus | Human food | 0.0299 | 0.0261 | 0.0958 |
| Thymus | Pet food | 0.0305 | 0.0261 | 0.0958 |
| Tongue | Human food | 0.0206 | 0.0261 | 0.0423 |

Table 4: Allocation factors for Average Ovine reared in House Fattening

| COPRODUCT | Destination | Average/Ovine/House Fattening | | |
| --- | --- | --- | --- | --- |
|  |  | **Biophysical Allocation Factor** | **Mass Allocation Factor** | **Economic Allocation Factor** |
| Blood | PAP C3 | 0.0134 | 0.0275 | 0.0000 |
| Blood | Spreading/Compost | 0.0000 | 0.0000 | 0.0000 |
| Bones | PAP C3 | 0.0132 | 0.0275 | 0.0000 |
| Brain | Human food | 0.0090 | 0.0275 | 0.0000 |
| Contents of the intestines | Spreading/Compost | 0.0000 | 0.0000 | 0.0000 |
| Dead individuals | C1-C2 for disposal | 0.0000 | 0.0000 | 0.0000 |
| Downgraded skin | PAP C3 | 0.0256 | 0.0275 | 0.0000 |
| Fat | PAP C3 | 0.0112 | 0.0275 | 0.0000 |
| Floatation fat | C1-C2 for disposal | 0.0000 | 0.0000 | 0.0000 |
| Meat | Human food | 0.0186 | 0.0275 | 0.0525 |
| Other spa c1 | C1-C2 for disposal | 0.0000 | 0.0000 | 0.0000 |
| Other spa c3 | PAP C3 | 0.0240 | 0.0275 | 0.0000 |
| Pluck (liver, heart, trachea) | Human food | 0.1338 | 0.0275 | 0.0307 |
| Pluck (liver, heart, trachea) | Pet food | 0.1369 | 0.0275 | 0.0305 |
| Rumen and reticulum | Human food | 0.1278 | 0.0275 | 0.0000 |
| Rumen and reticulum | Pet food | 0.1307 | 0.0275 | 0.0000 |
| Sanitary seizures | C1-C2 for disposal | 0.0000 | 0.0000 | 0.0000 |
| Screening waste | C1-C2 for disposal | 0.0000 | 0.0000 | 0.0000 |
| Sifting waste | C1-C2 for disposal | 0.0000 | 0.0000 | 0.0000 |
| Skin | Skin tannery C3 | 0.0338 | 0.0275 | 0.0069 |
| Small intestine | C1-C2 for disposal | 0.0000 | 0.0000 | 0.0000 |
| Small intestine | Human food | 0.1488 | 0.0275 | 0.0000 |
| Small intestine | PAP C3 | 0.1519 | 0.0275 | 0.0000 |
| Stercoral matter | Spreading/Compost | 0.0000 | 0.0000 | 0.0000 |
| Thymus | Human food | 0.0315 | 0.0275 | 0.1010 |
| Thymus | Pet food | 0.0321 | 0.0275 | 0.1010 |
| Tongue | Human food | 0.0217 | 0.0275 | 0.0446 |

Table 5: Allocation factors for Grass-fed Heavy Lamb reared in Grazing Flat Pasture

| COPRODUCT | Destination | Grass-fed Heavy Lamb/grazing Flat Pasture | | |
| --- | --- | --- | --- | --- |
|  |  | **Biophysical Allocation Factor** | **Mass Allocation Factor** | **Economic Allocation Factor** |
| Blood | PAP C3 | 0.0121 | 0.0248 | 0.0000 |
| Blood | Spreading/Compost | 0.0000 | 0.0000 | 0.0000 |
| Bones | PAP C3 | 0.0119 | 0.0248 | 0.0000 |
| Brain | Human food | 0.0081 | 0.0248 | 0.0000 |
| Contents of the intestines | Spreading/Compost | 0.0000 | 0.0000 | 0.0000 |
| Dead individuals | C1-C2 for disposal | 0.0000 | 0.0000 | 0.0000 |
| Downgraded skin | PAP C3 | 0.0230 | 0.0248 | 0.0000 |
| Fat | PAP C3 | 0.0101 | 0.0248 | 0.0000 |
| Floatation fat | C1-C2 for disposal | 0.0000 | 0.0000 | 0.0000 |
| Meat | Human food | 0.0168 | 0.0248 | 0.0474 |
| Other spa c1 | C1-C2 for disposal | 0.0000 | 0.0000 | 0.0000 |
| Other spa c3 | PAP C3 | 0.0217 | 0.0248 | 0.0000 |
| Pluck (liver, heart, trachea) | Human food | 0.1212 | 0.0248 | 0.0277 |
| Pluck (liver, heart, trachea) | Pet food | 0.1240 | 0.0248 | 0.0275 |
| Rumen and reticulum | Human food | 0.1158 | 0.0248 | 0.0000 |
| Rumen and reticulum | Pet food | 0.1184 | 0.0248 | 0.0000 |
| Sanitary seizures | C1-C2 for disposal | 0.0000 | 0.0000 | 0.0000 |
| Screening waste | C1-C2 for disposal | 0.0000 | 0.0000 | 0.0000 |
| Sifting waste | C1-C2 for disposal | 0.0000 | 0.0000 | 0.0000 |
| Skin | Skin tannery C3 | 0.0305 | 0.0248 | 0.0063 |
| Small intestine | C1-C2 for disposal | 0.0000 | 0.0000 | 0.0000 |
| Small intestine | Human food | 0.1348 | 0.0248 | 0.0000 |
| Small intestine | PAP C3 | 0.1376 | 0.0248 | 0.0000 |
| Stercoral matter | Spreading/Compost | 0.0000 | 0.0000 | 0.0000 |
| Thymus | Human food | 0.0284 | 0.0248 | 0.0912 |
| Thymus | Pet food | 0.0290 | 0.0248 | 0.0912 |
| Tongue | Human food | 0.0196 | 0.0248 | 0.0402 |

Table 6: Allocation factors for Grass-fed Heavy Lamb reared in Grazing Hilly Pasture

| COPRODUCT | Destination | Grass-fed Heavy Lamb/grazing Hilly Pasture | | |
| --- | --- | --- | --- | --- |
|  |  | **Biophysical Allocation Factor** | **Mass Allocation Factor** | **Economic Allocation Factor** |
| Blood | PAP C3 | 0.0121 | 0.0248 | 0.0000 |
| Blood | Spreading/Compost | 0.0000 | 0.0000 | 0.0000 |
| Bones | PAP C3 | 0.0119 | 0.0248 | 0.0000 |
| Brain | Human food | 0.0081 | 0.0248 | 0.0000 |
| Contents of the intestines | Spreading/Compost | 0.0000 | 0.0000 | 0.0000 |
| Dead individuals | C1-C2 for disposal | 0.0000 | 0.0000 | 0.0000 |
| Downgraded skin | PAP C3 | 0.0230 | 0.0248 | 0.0000 |
| Fat | PAP C3 | 0.0101 | 0.0248 | 0.0000 |
| Floatation fat | C1-C2 for disposal | 0.0000 | 0.0000 | 0.0000 |
| Meat | Human food | 0.0168 | 0.0248 | 0.0474 |
| Other spa c1 | C1-C2 for disposal | 0.0000 | 0.0000 | 0.0000 |
| Other spa c3 | PAP C3 | 0.0217 | 0.0248 | 0.0000 |
| Pluck (liver, heart, trachea) | Human food | 0.1212 | 0.0248 | 0.0277 |
| Pluck (liver, heart, trachea) | Pet food | 0.1240 | 0.0248 | 0.0275 |
| Rumen and reticulum | Human food | 0.1158 | 0.0248 | 0.0000 |
| Rumen and reticulum | Pet food | 0.1184 | 0.0248 | 0.0000 |
| Sanitary seizures | C1-C2 for disposal | 0.0000 | 0.0000 | 0.0000 |
| Screening waste | C1-C2 for disposal | 0.0000 | 0.0000 | 0.0000 |
| Sifting waste | C1-C2 for disposal | 0.0000 | 0.0000 | 0.0000 |
| Skin | Skin tannery C3 | 0.0305 | 0.0248 | 0.0063 |
| Small intestine | C1-C2 for disposal | 0.0000 | 0.0000 | 0.0000 |
| Small intestine | Human food | 0.1348 | 0.0248 | 0.0000 |
| Small intestine | PAP C3 | 0.1376 | 0.0248 | 0.0000 |
| Stercoral matter | Spreading/Compost | 0.0000 | 0.0000 | 0.0000 |
| Thymus | Human food | 0.0284 | 0.0248 | 0.0912 |
| Thymus | Pet food | 0.0290 | 0.0248 | 0.0912 |
| Tongue | Human food | 0.0196 | 0.0248 | 0.0402 |

Table 7: Allocation factors for Grass-fed Heavy Lamb reared in Housed Ewes

| COPRODUCT | Destination | Grass-fed Heavy Lamb/Housed ewes | | |
| --- | --- | --- | --- | --- |
|  |  | **Biophysical Allocation Factor** | **Mass Allocation Factor** | **Economic Allocation Factor** |
| Blood | PAP C3 | 0.0121 | 0.0248 | 0.0000 |
| Blood | Spreading/Compost | 0.0000 | 0.0000 | 0.0000 |
| Bones | PAP C3 | 0.0119 | 0.0248 | 0.0000 |
| Brain | Human food | 0.0081 | 0.0248 | 0.0000 |
| Contents of the intestines | Spreading/Compost | 0.0000 | 0.0000 | 0.0000 |
| Dead individuals | C1-C2 for disposal | 0.0000 | 0.0000 | 0.0000 |
| Downgraded skin | PAP C3 | 0.0230 | 0.0248 | 0.0000 |
| Fat | PAP C3 | 0.0101 | 0.0248 | 0.0000 |
| Floatation fat | C1-C2 for disposal | 0.0000 | 0.0000 | 0.0000 |
| Meat | Human food | 0.0168 | 0.0248 | 0.0474 |
| Other spa c1 | C1-C2 for disposal | 0.0000 | 0.0000 | 0.0000 |
| Other spa c3 | PAP C3 | 0.0217 | 0.0248 | 0.0000 |
| Pluck (liver, heart, trachea) | Human food | 0.1212 | 0.0248 | 0.0277 |
| Pluck (liver, heart, trachea) | Pet food | 0.1240 | 0.0248 | 0.0275 |
| Rumen and reticulum | Human food | 0.1158 | 0.0248 | 0.0000 |
| Rumen and reticulum | Pet food | 0.1184 | 0.0248 | 0.0000 |
| Sanitary seizures | C1-C2 for disposal | 0.0000 | 0.0000 | 0.0000 |
| Screening waste | C1-C2 for disposal | 0.0000 | 0.0000 | 0.0000 |
| Sifting waste | C1-C2 for disposal | 0.0000 | 0.0000 | 0.0000 |
| Skin | Skin tannery C3 | 0.0305 | 0.0248 | 0.0063 |
| Small intestine | C1-C2 for disposal | 0.0000 | 0.0000 | 0.0000 |
| Small intestine | Human food | 0.1348 | 0.0248 | 0.0000 |
| Small intestine | PAP C3 | 0.1376 | 0.0248 | 0.0000 |
| Stercoral matter | Spreading/Compost | 0.0000 | 0.0000 | 0.0000 |
| Thymus | Human food | 0.0284 | 0.0248 | 0.0912 |
| Thymus | Pet food | 0.0290 | 0.0248 | 0.0912 |
| Tongue | Human food | 0.0196 | 0.0248 | 0.0402 |

Table 8: Allocation factors for Milk-fed Hardy Lamb reared in Grazing Flat Pasture

| COPRODUCT | Destination | Milk-fed Hardy Lamb/grazing Flat Pasture | | |
| --- | --- | --- | --- | --- |
|  |  | **Biophysical Allocation Factor** | **Mass Allocation Factor** | **Economic Allocation Factor** |
| Blood | PAP C3 | 0.0138 | 0.0283 | 0.0000 |
| Blood | Spreading/Compost | 0.0000 | 0.0000 | 0.0000 |
| Bones | PAP C3 | 0.0135 | 0.0283 | 0.0000 |
| Brain | Human food | 0.0092 | 0.0283 | 0.0000 |
| Contents of the intestines | Spreading/Compost | 0.0000 | 0.0000 | 0.0000 |
| Dead individuals | C1-C2 for disposal | 0.0000 | 0.0000 | 0.0000 |
| Downgraded skin | PAP C3 | 0.0263 | 0.0283 | 0.0000 |
| Fat | PAP C3 | 0.0115 | 0.0283 | 0.0000 |
| Floatation fat | C1-C2 for disposal | 0.0000 | 0.0000 | 0.0000 |
| Meat | Human food | 0.0191 | 0.0283 | 0.0539 |
| Other spa c1 | C1-C2 for disposal | 0.0000 | 0.0000 | 0.0000 |
| Other spa c3 | PAP C3 | 0.0247 | 0.0283 | 0.0000 |
| Pluck (liver, heart, trachea) | Human food | 0.1374 | 0.0283 | 0.0316 |
| Pluck (liver, heart, trachea) | Pet food | 0.1406 | 0.0283 | 0.0313 |
| Rumen and reticulum | Human food | 0.1313 | 0.0283 | 0.0000 |
| Rumen and reticulum | Pet food | 0.1342 | 0.0283 | 0.0000 |
| Sanitary seizures | C1-C2 for disposal | 0.0000 | 0.0000 | 0.0000 |
| Screening waste | C1-C2 for disposal | 0.0000 | 0.0000 | 0.0000 |
| Sifting waste | C1-C2 for disposal | 0.0000 | 0.0000 | 0.0000 |
| Skin | Skin tannery C3 | 0.0348 | 0.0283 | 0.0071 |
| Small intestine | C1-C2 for disposal | 0.0000 | 0.0000 | 0.0000 |
| Small intestine | Human food | 0.1528 | 0.0283 | 0.0000 |
| Small intestine | PAP C3 | 0.1560 | 0.0283 | 0.0000 |
| Stercoral matter | Spreading/Compost | 0.0000 | 0.0000 | 0.0000 |
| Thymus | Human food | 0.0324 | 0.0283 | 0.1038 |
| Thymus | Pet food | 0.0330 | 0.0283 | 0.1038 |
| Tongue | Human food | 0.0223 | 0.0283 | 0.0458 |

Table 9: Allocation factors for Milk-fed Hardy Lamb reared in Housed Ewes

| COPRODUCT | Destination | Milk-fed Hardy Lamb/Housed Ewes | | |
| --- | --- | --- | --- | --- |
|  |  | **Biophysical Allocation Factor** | **Mass Allocation Factor** | **Economic Allocation Factor** |
| Blood | PAP C3 | 0.0138 | 0.0283 | 0.0000 |
| Blood | Spreading/Compost | 0.0000 | 0.0000 | 0.0000 |
| Bones | PAP C3 | 0.0135 | 0.0283 | 0.0000 |
| Brain | Human food | 0.0092 | 0.0283 | 0.0000 |
| Contents of the intestines | Spreading/Compost | 0.0000 | 0.0000 | 0.0000 |
| Dead individuals | C1-C2 for disposal | 0.0000 | 0.0000 | 0.0000 |
| Downgraded skin | PAP C3 | 0.0263 | 0.0283 | 0.0000 |
| Fat | PAP C3 | 0.0115 | 0.0283 | 0.0000 |
| Floatation fat | C1-C2 for disposal | 0.0000 | 0.0000 | 0.0000 |
| Meat | Human food | 0.0191 | 0.0283 | 0.0539 |
| Other spa c1 | C1-C2 for disposal | 0.0000 | 0.0000 | 0.0000 |
| Other spa c3 | PAP C3 | 0.0247 | 0.0283 | 0.0000 |
| Pluck (liver, heart, trachea) | Human food | 0.1374 | 0.0283 | 0.0316 |
| Pluck (liver, heart, trachea) | Pet food | 0.1406 | 0.0283 | 0.0313 |
| Rumen and reticulum | Human food | 0.1313 | 0.0283 | 0.0000 |
| Rumen and reticulum | Pet food | 0.1342 | 0.0283 | 0.0000 |
| Sanitary seizures | C1-C2 for disposal | 0.0000 | 0.0000 | 0.0000 |
| Screening waste | C1-C2 for disposal | 0.0000 | 0.0000 | 0.0000 |
| Sifting waste | C1-C2 for disposal | 0.0000 | 0.0000 | 0.0000 |
| Skin | Skin tannery C3 | 0.0348 | 0.0283 | 0.0071 |
| Small intestine | C1-C2 for disposal | 0.0000 | 0.0000 | 0.0000 |
| Small intestine | Human food | 0.1528 | 0.0283 | 0.0000 |
| Small intestine | PAP C3 | 0.1560 | 0.0283 | 0.0000 |
| Stercoral matter | Spreading/Compost | 0.0000 | 0.0000 | 0.0000 |
| Thymus | Human food | 0.0324 | 0.0283 | 0.1038 |
| Thymus | Pet food | 0.0330 | 0.0283 | 0.1038 |
| Tongue | Human food | 0.0223 | 0.0283 | 0.0458 |

Table 10: Allocation factors for Milk-fed Hardy Lamb reared in House Fattening

| COPRODUCT | Destination | Milk-fed Hardy Lamb/House Fattening | | |
| --- | --- | --- | --- | --- |
|  |  | **Biophysical Allocation Factor** | **Mass Allocation Factor** | **Economic Allocation Factor** |
| Blood | PAP C3 | 0.0138 | 0.0283 | 0.0000 |
| Blood | Spreading/Compost | 0.0000 | 0.0000 | 0.0000 |
| Bones | PAP C3 | 0.0135 | 0.0283 | 0.0000 |
| Brain | Human food | 0.0092 | 0.0283 | 0.0000 |
| Contents of the intestines | Spreading/Compost | 0.0000 | 0.0000 | 0.0000 |
| Dead individuals | C1-C2 for disposal | 0.0000 | 0.0000 | 0.0000 |
| Downgraded skin | PAP C3 | 0.0263 | 0.0283 | 0.0000 |
| Fat | PAP C3 | 0.0115 | 0.0283 | 0.0000 |
| Floatation fat | C1-C2 for disposal | 0.0000 | 0.0000 | 0.0000 |
| Meat | Human food | 0.0191 | 0.0283 | 0.0539 |
| Other spa c1 | C1-C2 for disposal | 0.0000 | 0.0000 | 0.0000 |
| Other spa c3 | PAP C3 | 0.0247 | 0.0283 | 0.0000 |
| Pluck (liver, heart, trachea) | Human food | 0.1374 | 0.0283 | 0.0316 |
| Pluck (liver, heart, trachea) | Pet food | 0.1406 | 0.0283 | 0.0313 |
| Rumen and reticulum | Human food | 0.1313 | 0.0283 | 0.0000 |
| Rumen and reticulum | Pet food | 0.1342 | 0.0283 | 0.0000 |
| Sanitary seizures | C1-C2 for disposal | 0.0000 | 0.0000 | 0.0000 |
| Screening waste | C1-C2 for disposal | 0.0000 | 0.0000 | 0.0000 |
| Sifting waste | C1-C2 for disposal | 0.0000 | 0.0000 | 0.0000 |
| Skin | Skin tannery C3 | 0.0348 | 0.0283 | 0.0071 |
| Small intestine | C1-C2 for disposal | 0.0000 | 0.0000 | 0.0000 |
| Small intestine | Human food | 0.1528 | 0.0283 | 0.0000 |
| Small intestine | PAP C3 | 0.1560 | 0.0283 | 0.0000 |
| Stercoral matter | Spreading/Compost | 0.0000 | 0.0000 | 0.0000 |
| Thymus | Human food | 0.0324 | 0.0283 | 0.1038 |
| Thymus | Pet food | 0.0330 | 0.0283 | 0.1038 |
| Tongue | Human food | 0.0223 | 0.0283 | 0.0458 |

Table 11: Allocation factors for Milk-fed Heavy Lamb reared in Grazing Flat Pasture

| COPRODUCT | Destination | Milk-fed Heavy Lamb/grazing Flat Pasture | | |
| --- | --- | --- | --- | --- |
|  |  | **Biophysical Allocation Factor** | **Mass Allocation Factor** | **Economic Allocation Factor** |
| Blood | PAP C3 | 0.0127 | 0.0261 | 0.0000 |
| Blood | Spreading/Compost | 0.0000 | 0.0000 | 0.0000 |
| Bones | PAP C3 | 0.0125 | 0.0261 | 0.0000 |
| Brain | Human food | 0.0085 | 0.0261 | 0.0000 |
| Contents of the intestines | Spreading/Compost | 0.0000 | 0.0000 | 0.0000 |
| Dead individuals | C1-C2 for disposal | 0.0000 | 0.0000 | 0.0000 |
| Downgraded skin | PAP C3 | 0.0242 | 0.0261 | 0.0000 |
| Fat | PAP C3 | 0.0106 | 0.0261 | 0.0000 |
| Floatation fat | C1-C2 for disposal | 0.0000 | 0.0000 | 0.0000 |
| Meat | Human food | 0.0176 | 0.0261 | 0.0498 |
| Other spa c1 | C1-C2 for disposal | 0.0000 | 0.0000 | 0.0000 |
| Other spa c3 | PAP C3 | 0.0228 | 0.0261 | 0.0000 |
| Pluck (liver, heart, trachea) | Human food | 0.1272 | 0.0261 | 0.0291 |
| Pluck (liver, heart, trachea) | Pet food | 0.1301 | 0.0261 | 0.0289 |
| Rumen and reticulum | Human food | 0.1215 | 0.0261 | 0.0000 |
| Rumen and reticulum | Pet food | 0.1242 | 0.0261 | 0.0000 |
| Sanitary seizures | C1-C2 for disposal | 0.0000 | 0.0000 | 0.0000 |
| Screening waste | C1-C2 for disposal | 0.0000 | 0.0000 | 0.0000 |
| Sifting waste | C1-C2 for disposal | 0.0000 | 0.0000 | 0.0000 |
| Skin | Skin tannery C3 | 0.0321 | 0.0261 | 0.0066 |
| Small intestine | C1-C2 for disposal | 0.0000 | 0.0000 | 0.0000 |
| Small intestine | Human food | 0.1414 | 0.0261 | 0.0000 |
| Small intestine | PAP C3 | 0.1444 | 0.0261 | 0.0000 |
| Stercoral matter | Spreading/Compost | 0.0000 | 0.0000 | 0.0000 |
| Thymus | Human food | 0.0299 | 0.0261 | 0.0958 |
| Thymus | Pet food | 0.0305 | 0.0261 | 0.0958 |
| Tongue | Human food | 0.0206 | 0.0261 | 0.0423 |

Table 12: Allocation factors for Milk-fed Heavy Lamb reared in Housed Ewes

| COPRODUCT | Destination | Milk-fed Heavy Lamb/Housed Ewes | | |
| --- | --- | --- | --- | --- |
|  |  | **Biophysical Allocation Factor** | **Mass Allocation Factor** | **Economic Allocation Factor** |
| Blood | PAP C3 | 0.0127 | 0.0261 | 0.0000 |
| Blood | Spreading/Compost | 0.0000 | 0.0000 | 0.0000 |
| Bones | PAP C3 | 0.0125 | 0.0261 | 0.0000 |
| Brain | Human food | 0.0085 | 0.0261 | 0.0000 |
| Contents of the intestines | Spreading/Compost | 0.0000 | 0.0000 | 0.0000 |
| Dead individuals | C1-C2 for disposal | 0.0000 | 0.0000 | 0.0000 |
| Downgraded skin | PAP C3 | 0.0242 | 0.0261 | 0.0000 |
| Fat | PAP C3 | 0.0106 | 0.0261 | 0.0000 |
| Floatation fat | C1-C2 for disposal | 0.0000 | 0.0000 | 0.0000 |
| Meat | Human food | 0.0176 | 0.0261 | 0.0498 |
| Other spa c1 | C1-C2 for disposal | 0.0000 | 0.0000 | 0.0000 |
| Other spa c3 | PAP C3 | 0.0228 | 0.0261 | 0.0000 |
| Pluck (liver, heart, trachea) | Human food | 0.1272 | 0.0261 | 0.0291 |
| Pluck (liver, heart, trachea) | Pet food | 0.1301 | 0.0261 | 0.0289 |
| Rumen and reticulum | Human food | 0.1215 | 0.0261 | 0.0000 |
| Rumen and reticulum | Pet food | 0.1242 | 0.0261 | 0.0000 |
| Sanitary seizures | C1-C2 for disposal | 0.0000 | 0.0000 | 0.0000 |
| Screening waste | C1-C2 for disposal | 0.0000 | 0.0000 | 0.0000 |
| Sifting waste | C1-C2 for disposal | 0.0000 | 0.0000 | 0.0000 |
| Skin | Skin tannery C3 | 0.0321 | 0.0261 | 0.0066 |
| Small intestine | C1-C2 for disposal | 0.0000 | 0.0000 | 0.0000 |
| Small intestine | Human food | 0.1414 | 0.0261 | 0.0000 |
| Small intestine | PAP C3 | 0.1444 | 0.0261 | 0.0000 |
| Stercoral matter | Spreading/Compost | 0.0000 | 0.0000 | 0.0000 |
| Thymus | Human food | 0.0299 | 0.0261 | 0.0958 |
| Thymus | Pet food | 0.0305 | 0.0261 | 0.0958 |
| Tongue | Human food | 0.0206 | 0.0261 | 0.0423 |

Table 13: Allocation factors for Milk-fed Heavy Lamb in House Fattening

| COPRODUCT | Destination | Milk-fed Heavy Lamb/House Fattening | | |
| --- | --- | --- | --- | --- |
|  |  | **Biophysical Allocation Factor** | **Mass Allocation Factor** | **Economic Allocation Factor** |
| Blood | PAP C3 | 0.0127 | 0.0261 | 0.0000 |
| Blood | Spreading/Compost | 0.0000 | 0.0000 | 0.0000 |
| Bones | PAP C3 | 0.0125 | 0.0261 | 0.0000 |
| Brain | Human food | 0.0085 | 0.0261 | 0.0000 |
| Contents of the intestines | Spreading/Compost | 0.0000 | 0.0000 | 0.0000 |
| Dead individuals | C1-C2 for disposal | 0.0000 | 0.0000 | 0.0000 |
| Downgraded skin | PAP C3 | 0.0242 | 0.0261 | 0.0000 |
| Fat | PAP C3 | 0.0106 | 0.0261 | 0.0000 |
| Floatation fat | C1-C2 for disposal | 0.0000 | 0.0000 | 0.0000 |
| Meat | Human food | 0.0176 | 0.0261 | 0.0498 |
| Other spa c1 | C1-C2 for disposal | 0.0000 | 0.0000 | 0.0000 |
| Other spa c3 | PAP C3 | 0.0228 | 0.0261 | 0.0000 |
| Pluck (liver, heart, trachea) | Human food | 0.1272 | 0.0261 | 0.0291 |
| Pluck (liver, heart, trachea) | Pet food | 0.1301 | 0.0261 | 0.0289 |
| Rumen and reticulum | Human food | 0.1215 | 0.0261 | 0.0000 |
| Rumen and reticulum | Pet food | 0.1242 | 0.0261 | 0.0000 |
| Sanitary seizures | C1-C2 for disposal | 0.0000 | 0.0000 | 0.0000 |
| Screening waste | C1-C2 for disposal | 0.0000 | 0.0000 | 0.0000 |
| Sifting waste | C1-C2 for disposal | 0.0000 | 0.0000 | 0.0000 |
| Skin | Skin tannery C3 | 0.0321 | 0.0261 | 0.0066 |
| Small intestine | C1-C2 for disposal | 0.0000 | 0.0000 | 0.0000 |
| Small intestine | Human food | 0.1414 | 0.0261 | 0.0000 |
| Small intestine | PAP C3 | 0.1444 | 0.0261 | 0.0000 |
| Stercoral matter | Spreading/Compost | 0.0000 | 0.0000 | 0.0000 |
| Thymus | Human food | 0.0299 | 0.0261 | 0.0958 |
| Thymus | Pet food | 0.0305 | 0.0261 | 0.0958 |
| Tongue | Human food | 0.0206 | 0.0261 | 0.0423 |

Table 14: Allocation factors for Milk Lamb reared in House Fattening

| COPRODUCT | Destination | Milk Lamb/House Fattening | | |
| --- | --- | --- | --- | --- |
|  |  | **Biophysical Allocation Factor** | **Mass Allocation Factor** | **Economic Allocation Factor** |
| Blood | PAP C3 | 0.0127 | 0.0261 | 0.0000 |
| Blood | Spreading/Compost | 0.0000 | 0.0000 | 0.0000 |
| Bones | PAP C3 | 0.0125 | 0.0261 | 0.0000 |
| Brain | Human food | 0.0085 | 0.0261 | 0.0000 |
| Contents of the intestines | Spreading/Compost | 0.0000 | 0.0000 | 0.0000 |
| Dead individuals | C1-C2 for disposal | 0.0000 | 0.0000 | 0.0000 |
| Downgraded skin | PAP C3 | 0.0242 | 0.0261 | 0.0000 |
| Fat | PAP C3 | 0.0106 | 0.0261 | 0.0000 |
| Floatation fat | C1-C2 for disposal | 0.0000 | 0.0000 | 0.0000 |
| Meat | Human food | 0.0176 | 0.0261 | 0.0498 |
| Other spa c1 | C1-C2 for disposal | 0.0000 | 0.0000 | 0.0000 |
| Other spa c3 | PAP C3 | 0.0228 | 0.0261 | 0.0000 |
| Pluck (liver, heart, trachea) | Human food | 0.1272 | 0.0261 | 0.0291 |
| Pluck (liver, heart, trachea) | Pet food | 0.1301 | 0.0261 | 0.0289 |
| Rumen and reticulum | Human food | 0.1215 | 0.0261 | 0.0000 |
| Rumen and reticulum | Pet food | 0.1242 | 0.0261 | 0.0000 |
| Sanitary seizures | C1-C2 for disposal | 0.0000 | 0.0000 | 0.0000 |
| Screening waste | C1-C2 for disposal | 0.0000 | 0.0000 | 0.0000 |
| Sifting waste | C1-C2 for disposal | 0.0000 | 0.0000 | 0.0000 |
| Skin | Skin tannery C3 | 0.0321 | 0.0261 | 0.0066 |
| Small intestine | C1-C2 for disposal | 0.0000 | 0.0000 | 0.0000 |
| Small intestine | Human food | 0.1414 | 0.0261 | 0.0000 |
| Small intestine | PAP C3 | 0.1444 | 0.0261 | 0.0000 |
| Stercoral matter | Spreading/Compost | 0.0000 | 0.0000 | 0.0000 |
| Thymus | Human food | 0.0299 | 0.0261 | 0.0958 |
| Thymus | Pet food | 0.0305 | 0.0261 | 0.0958 |
| Tongue | Human food | 0.0206 | 0.0261 | 0.0423 |
