# Supplementary material for: Allocation factors for meat coproducts: Dataset to perform life cycle assessment at slaughterhouse
Source: Data Brief. 2020 Nov 23;33:106558. doi: 10.1016/j.dib.2020.106558 (PMC7718151; doi:10.1016/j.dib.2020.106558)
Supplement: Supplementary file 8 [file mmc8.docx]

Table 1: Total Weighting per coproducts for Average Ovine reared in Grazing Flat Pasture

| COPRODUCT | Destination | Average/Ovine/grazing Flat Pasture | | |
| --- | --- | --- | --- | --- |
|  |  | **Biophysical Cumulative Share** | **Mass Cumulative Share** | **Economic Cumulative Share** |
| Blood | PAP C3 | 0.0032 | 0.0066 | 0.0000 |
| Blood | Spreading/Compost | 0.0000 | 0.0000 | 0.0000 |
| Bones | PAP C3 | 0.0195 | 0.0409 | 0.0000 |
| Brain | Human food | 0.0004 | 0.0011 | 0.0000 |
| Contents of the intestines | Spreading/Compost | 0.0000 | 0.0000 | 0.0000 |
| Dead individuals | C1-C2 for disposal | 0.0000 | 0.0000 | 0.0000 |
| Downgraded skin | PAP C3 | 0.0251 | 0.0270 | 0.0000 |
| Fat | PAP C3 | 0.0273 | 0.0670 | 0.0000 |
| Floatation fat | C1-C2 for disposal | 0.0000 | 0.0000 | 0.0000 |
| Meat | Human food | 0.3270 | 0.4838 | 0.9225 |
| Other spa c1 | C1-C2 for disposal | 0.0000 | 0.0000 | 0.0000 |
| Other spa c3 | PAP C3 | 0.1188 | 0.1361 | 0.0000 |
| Pluck (liver, heart, trachea) | Human food | 0.0582 | 0.0120 | 0.0133 |
| Pluck (liver, heart, trachea) | Pet food | 0.0068 | 0.0014 | 0.0015 |
| Rumen and reticulum | Human food | 0.0012 | 0.0002 | 0.0000 |
| Rumen and reticulum | Pet food | 0.0261 | 0.0055 | 0.0000 |
| Sanitary seizures | C1-C2 for disposal | 0.0000 | 0.0000 | 0.0000 |
| Screening waste | C1-C2 for disposal | 0.0000 | 0.0000 | 0.0000 |
| Sifting waste | C1-C2 for disposal | 0.0000 | 0.0000 | 0.0000 |
| Skin | Skin tannery C3 | 0.2255 | 0.1835 | 0.0462 |
| Small intestine | C1-C2 for disposal | 0.0000 | 0.0000 | 0.0000 |
| Small intestine | Human food | 0.1321 | 0.0244 | 0.0000 |
| Small intestine | PAP C3 | 0.0227 | 0.0041 | 0.0000 |
| Stercoral matter | Spreading/Compost | 0.0000 | 0.0000 | 0.0000 |
| Thymus | Human food | 0.0019 | 0.0016 | 0.0059 |
| Thymus | Pet food | 0.0016 | 0.0014 | 0.0050 |
| Tongue | Human food | 0.0027 | 0.0034 | 0.0054 |

Table 2: Total Weighting per coproducts for Average Ovine reared in Grazing Hilly Pasture

| COPRODUCT | Destination | Average/Ovine/grazing Hilly Pasture | | |
| --- | --- | --- | --- | --- |
|  |  | **Biophysical Cumulative Share** | **Mass Cumulative Share** | **Economic Cumulative Share** |
| Blood | PAP C3 | 0.0032 | 0.0066 | 0.0000 |
| Blood | Spreading/Compost | 0.0000 | 0.0000 | 0.0000 |
| Bones | PAP C3 | 0.0195 | 0.0409 | 0.0000 |
| Brain | Human food | 0.0004 | 0.0011 | 0.0000 |
| Contents of the intestines | Spreading/Compost | 0.0000 | 0.0000 | 0.0000 |
| Dead individuals | C1-C2 for disposal | 0.0000 | 0.0000 | 0.0000 |
| Downgraded skin | PAP C3 | 0.0251 | 0.0270 | 0.0000 |
| Fat | PAP C3 | 0.0273 | 0.0670 | 0.0000 |
| Floatation fat | C1-C2 for disposal | 0.0000 | 0.0000 | 0.0000 |
| Meat | Human food | 0.3269 | 0.4838 | 0.9225 |
| Other spa c1 | C1-C2 for disposal | 0.0000 | 0.0000 | 0.0000 |
| Other spa c3 | PAP C3 | 0.1188 | 0.1361 | 0.0000 |
| Pluck (liver, heart, trachea) | Human food | 0.0582 | 0.0120 | 0.0133 |
| Pluck (liver, heart, trachea) | Pet food | 0.0068 | 0.0014 | 0.0015 |
| Rumen and reticulum | Human food | 0.0012 | 0.0002 | 0.0000 |
| Rumen and reticulum | Pet food | 0.0261 | 0.0055 | 0.0000 |
| Sanitary seizures | C1-C2 for disposal | 0.0000 | 0.0000 | 0.0000 |
| Screening waste | C1-C2 for disposal | 0.0000 | 0.0000 | 0.0000 |
| Sifting waste | C1-C2 for disposal | 0.0000 | 0.0000 | 0.0000 |
| Skin | Skin tannery C3 | 0.2255 | 0.1835 | 0.0462 |
| Small intestine | C1-C2 for disposal | 0.0000 | 0.0000 | 0.0000 |
| Small intestine | Human food | 0.1322 | 0.0244 | 0.0000 |
| Small intestine | PAP C3 | 0.0227 | 0.0041 | 0.0000 |
| Stercoral matter | Spreading/Compost | 0.0000 | 0.0000 | 0.0000 |
| Thymus | Human food | 0.0019 | 0.0016 | 0.0059 |
| Thymus | Pet food | 0.0016 | 0.0014 | 0.0050 |
| Tongue | Human food | 0.0027 | 0.0034 | 0.0054 |

Table 3: Total Weighting per coproducts for Average Ovine reared in Housed Ewes

| COPRODUCT | Destination | Average/Ovine/Housed Ewes | | |
| --- | --- | --- | --- | --- |
|  |  | **Biophysical Cumulative Share** | **Mass Cumulative Share** | **Economic Cumulative Share** |
| Blood | PAP C3 | 0.0032 | 0.0066 | 0.0000 |
| Blood | Spreading/Compost | 0.0000 | 0.0000 | 0.0000 |
| Bones | PAP C3 | 0.0195 | 0.0409 | 0.0000 |
| Brain | Human food | 0.0004 | 0.0011 | 0.0000 |
| Contents of the intestines | Spreading/Compost | 0.0000 | 0.0000 | 0.0000 |
| Dead individuals | C1-C2 for disposal | 0.0000 | 0.0000 | 0.0000 |
| Downgraded skin | PAP C3 | 0.0251 | 0.0270 | 0.0000 |
| Fat | PAP C3 | 0.0273 | 0.0670 | 0.0000 |
| Floatation fat | C1-C2 for disposal | 0.0000 | 0.0000 | 0.0000 |
| Meat | Human food | 0.3269 | 0.4838 | 0.9225 |
| Other spa c1 | C1-C2 for disposal | 0.0000 | 0.0000 | 0.0000 |
| Other spa c3 | PAP C3 | 0.1188 | 0.1361 | 0.0000 |
| Pluck (liver, heart, trachea) | Human food | 0.0582 | 0.0120 | 0.0133 |
| Pluck (liver, heart, trachea) | Pet food | 0.0068 | 0.0014 | 0.0015 |
| Rumen and reticulum | Human food | 0.0012 | 0.0002 | 0.0000 |
| Rumen and reticulum | Pet food | 0.0261 | 0.0055 | 0.0000 |
| Sanitary seizures | C1-C2 for disposal | 0.0000 | 0.0000 | 0.0000 |
| Screening waste | C1-C2 for disposal | 0.0000 | 0.0000 | 0.0000 |
| Sifting waste | C1-C2 for disposal | 0.0000 | 0.0000 | 0.0000 |
| Skin | Skin tannery C3 | 0.2255 | 0.1835 | 0.0462 |
| Small intestine | C1-C2 for disposal | 0.0000 | 0.0000 | 0.0000 |
| Small intestine | Human food | 0.1322 | 0.0244 | 0.0000 |
| Small intestine | PAP C3 | 0.0227 | 0.0041 | 0.0000 |
| Stercoral matter | Spreading/Compost | 0.0000 | 0.0000 | 0.0000 |
| Thymus | Human food | 0.0019 | 0.0016 | 0.0059 |
| Thymus | Pet food | 0.0016 | 0.0014 | 0.0050 |
| Tongue | Human food | 0.0027 | 0.0034 | 0.0054 |

Table 4: Total Weighting per coproducts for Average Ovine reared in House Fattening

| COPRODUCT | Destination | Average/Ovine/House Fattening | | |
| --- | --- | --- | --- | --- |
|  |  | **Biophysical Cumulative Share** | **Mass Cumulative Share** | **Economic Cumulative Share** |
| Blood | PAP C3 | 0.0032 | 0.0066 | 0.0000 |
| Blood | Spreading/Compost | 0.0000 | 0.0000 | 0.0000 |
| Bones | PAP C3 | 0.0195 | 0.0409 | 0.0000 |
| Brain | Human food | 0.0004 | 0.0011 | 0.0000 |
| Contents of the intestines | Spreading/Compost | 0.0000 | 0.0000 | 0.0000 |
| Dead individuals | C1-C2 for disposal | 0.0000 | 0.0000 | 0.0000 |
| Downgraded skin | PAP C3 | 0.0251 | 0.0270 | 0.0000 |
| Fat | PAP C3 | 0.0272 | 0.0670 | 0.0000 |
| Floatation fat | C1-C2 for disposal | 0.0000 | 0.0000 | 0.0000 |
| Meat | Human food | 0.3272 | 0.4838 | 0.9225 |
| Other spa c1 | C1-C2 for disposal | 0.0000 | 0.0000 | 0.0000 |
| Other spa c3 | PAP C3 | 0.1189 | 0.1361 | 0.0000 |
| Pluck (liver, heart, trachea) | Human food | 0.0581 | 0.0120 | 0.0133 |
| Pluck (liver, heart, trachea) | Pet food | 0.0068 | 0.0014 | 0.0015 |
| Rumen and reticulum | Human food | 0.0012 | 0.0002 | 0.0000 |
| Rumen and reticulum | Pet food | 0.0260 | 0.0055 | 0.0000 |
| Sanitary seizures | C1-C2 for disposal | 0.0000 | 0.0000 | 0.0000 |
| Screening waste | C1-C2 for disposal | 0.0000 | 0.0000 | 0.0000 |
| Sifting waste | C1-C2 for disposal | 0.0000 | 0.0000 | 0.0000 |
| Skin | Skin tannery C3 | 0.2255 | 0.1835 | 0.0462 |
| Small intestine | C1-C2 for disposal | 0.0000 | 0.0000 | 0.0000 |
| Small intestine | Human food | 0.1320 | 0.0244 | 0.0000 |
| Small intestine | PAP C3 | 0.0227 | 0.0041 | 0.0000 |
| Stercoral matter | Spreading/Compost | 0.0000 | 0.0000 | 0.0000 |
| Thymus | Human food | 0.0019 | 0.0016 | 0.0059 |
| Thymus | Pet food | 0.0016 | 0.0014 | 0.0050 |
| Tongue | Human food | 0.0027 | 0.0034 | 0.0054 |

Table 5: Total Weighting per coproducts for Grass-fed Heavy Lamb reared in Grazing Flat Pasture

| COPRODUCT | Destination | Grass-fed Heavy Lamb/grazing Flat Pasture | | |
| --- | --- | --- | --- | --- |
|  |  | **Biophysical Cumulative Share** | **Mass Cumulative Share** | **Economic Cumulative Share** |
| Blood | PAP C3 | 0.0032 | 0.0066 | 0.0000 |
| Blood | Spreading/Compost | 0.0000 | 0.0000 | 0.0000 |
| Bones | PAP C3 | 0.0195 | 0.0409 | 0.0000 |
| Brain | Human food | 0.0004 | 0.0011 | 0.0000 |
| Contents of the intestines | Spreading/Compost | 0.0000 | 0.0000 | 0.0000 |
| Dead individuals | C1-C2 for disposal | 0.0000 | 0.0000 | 0.0000 |
| Downgraded skin | PAP C3 | 0.0250 | 0.0270 | 0.0000 |
| Fat | PAP C3 | 0.0273 | 0.0670 | 0.0000 |
| Floatation fat | C1-C2 for disposal | 0.0000 | 0.0000 | 0.0000 |
| Meat | Human food | 0.3266 | 0.4838 | 0.9225 |
| Other spa c1 | C1-C2 for disposal | 0.0000 | 0.0000 | 0.0000 |
| Other spa c3 | PAP C3 | 0.1187 | 0.1361 | 0.0000 |
| Pluck (liver, heart, trachea) | Human food | 0.0584 | 0.0120 | 0.0133 |
| Pluck (liver, heart, trachea) | Pet food | 0.0068 | 0.0014 | 0.0015 |
| Rumen and reticulum | Human food | 0.0012 | 0.0002 | 0.0000 |
| Rumen and reticulum | Pet food | 0.0261 | 0.0055 | 0.0000 |
| Sanitary seizures | C1-C2 for disposal | 0.0000 | 0.0000 | 0.0000 |
| Screening waste | C1-C2 for disposal | 0.0000 | 0.0000 | 0.0000 |
| Sifting waste | C1-C2 for disposal | 0.0000 | 0.0000 | 0.0000 |
| Skin | Skin tannery C3 | 0.2254 | 0.1835 | 0.0462 |
| Small intestine | C1-C2 for disposal | 0.0000 | 0.0000 | 0.0000 |
| Small intestine | Human food | 0.1325 | 0.0244 | 0.0000 |
| Small intestine | PAP C3 | 0.0228 | 0.0041 | 0.0000 |
| Stercoral matter | Spreading/Compost | 0.0000 | 0.0000 | 0.0000 |
| Thymus | Human food | 0.0019 | 0.0016 | 0.0059 |
| Thymus | Pet food | 0.0016 | 0.0014 | 0.0050 |
| Tongue | Human food | 0.0027 | 0.0034 | 0.0054 |

Table 6: Total Weighting per coproducts for Grass-fed Heavy Lamb reared in Grazing Hilly Pasture

| COPRODUCT | Destination | Grass-fed Heavy Lamb/grazing Hilly Pasture | | |
| --- | --- | --- | --- | --- |
|  |  | **Biophysical Cumulative Share** | **Mass Cumulative Share** | **Economic Cumulative Share** |
| Blood | PAP C3 | 0.0032 | 0.0066 | 0.0000 |
| Blood | Spreading/Compost | 0.0000 | 0.0000 | 0.0000 |
| Bones | PAP C3 | 0.0195 | 0.0409 | 0.0000 |
| Brain | Human food | 0.0004 | 0.0011 | 0.0000 |
| Contents of the intestines | Spreading/Compost | 0.0000 | 0.0000 | 0.0000 |
| Dead individuals | C1-C2 for disposal | 0.0000 | 0.0000 | 0.0000 |
| Downgraded skin | PAP C3 | 0.0250 | 0.0270 | 0.0000 |
| Fat | PAP C3 | 0.0273 | 0.0670 | 0.0000 |
| Floatation fat | C1-C2 for disposal | 0.0000 | 0.0000 | 0.0000 |
| Meat | Human food | 0.3266 | 0.4838 | 0.9225 |
| Other spa c1 | C1-C2 for disposal | 0.0000 | 0.0000 | 0.0000 |
| Other spa c3 | PAP C3 | 0.1187 | 0.1361 | 0.0000 |
| Pluck (liver, heart, trachea) | Human food | 0.0584 | 0.0120 | 0.0133 |
| Pluck (liver, heart, trachea) | Pet food | 0.0068 | 0.0014 | 0.0015 |
| Rumen and reticulum | Human food | 0.0012 | 0.0002 | 0.0000 |
| Rumen and reticulum | Pet food | 0.0261 | 0.0055 | 0.0000 |
| Sanitary seizures | C1-C2 for disposal | 0.0000 | 0.0000 | 0.0000 |
| Screening waste | C1-C2 for disposal | 0.0000 | 0.0000 | 0.0000 |
| Sifting waste | C1-C2 for disposal | 0.0000 | 0.0000 | 0.0000 |
| Skin | Skin tannery C3 | 0.2254 | 0.1835 | 0.0462 |
| Small intestine | C1-C2 for disposal | 0.0000 | 0.0000 | 0.0000 |
| Small intestine | Human food | 0.1325 | 0.0244 | 0.0000 |
| Small intestine | PAP C3 | 0.0228 | 0.0041 | 0.0000 |
| Stercoral matter | Spreading/Compost | 0.0000 | 0.0000 | 0.0000 |
| Thymus | Human food | 0.0019 | 0.0016 | 0.0059 |
| Thymus | Pet food | 0.0016 | 0.0014 | 0.0050 |
| Tongue | Human food | 0.0027 | 0.0034 | 0.0054 |

Table 7: Total Weighting per coproducts for Grass-fed Heavy Lamb reared in Housed Ewes

| COPRODUCT | Destination | Grass-fed Heavy Lamb/Housed ewes | | |
| --- | --- | --- | --- | --- |
|  |  | **Biophysical Cumulative Share** | **Mass Cumulative Share** | **Economic Cumulative Share** |
| Blood | PAP C3 | 0.0032 | 0.0066 | 0.0000 |
| Blood | Spreading/Compost | 0.0000 | 0.0000 | 0.0000 |
| Bones | PAP C3 | 0.0195 | 0.0409 | 0.0000 |
| Brain | Human food | 0.0004 | 0.0011 | 0.0000 |
| Contents of the intestines | Spreading/Compost | 0.0000 | 0.0000 | 0.0000 |
| Dead individuals | C1-C2 for disposal | 0.0000 | 0.0000 | 0.0000 |
| Downgraded skin | PAP C3 | 0.0250 | 0.0270 | 0.0000 |
| Fat | PAP C3 | 0.0273 | 0.0670 | 0.0000 |
| Floatation fat | C1-C2 for disposal | 0.0000 | 0.0000 | 0.0000 |
| Meat | Human food | 0.3266 | 0.4838 | 0.9225 |
| Other spa c1 | C1-C2 for disposal | 0.0000 | 0.0000 | 0.0000 |
| Other spa c3 | PAP C3 | 0.1187 | 0.1361 | 0.0000 |
| Pluck (liver, heart, trachea) | Human food | 0.0584 | 0.0120 | 0.0133 |
| Pluck (liver, heart, trachea) | Pet food | 0.0068 | 0.0014 | 0.0015 |
| Rumen and reticulum | Human food | 0.0012 | 0.0002 | 0.0000 |
| Rumen and reticulum | Pet food | 0.0261 | 0.0055 | 0.0000 |
| Sanitary seizures | C1-C2 for disposal | 0.0000 | 0.0000 | 0.0000 |
| Screening waste | C1-C2 for disposal | 0.0000 | 0.0000 | 0.0000 |
| Sifting waste | C1-C2 for disposal | 0.0000 | 0.0000 | 0.0000 |
| Skin | Skin tannery C3 | 0.2254 | 0.1835 | 0.0462 |
| Small intestine | C1-C2 for disposal | 0.0000 | 0.0000 | 0.0000 |
| Small intestine | Human food | 0.1325 | 0.0244 | 0.0000 |
| Small intestine | PAP C3 | 0.0228 | 0.0041 | 0.0000 |
| Stercoral matter | Spreading/Compost | 0.0000 | 0.0000 | 0.0000 |
| Thymus | Human food | 0.0019 | 0.0016 | 0.0059 |
| Thymus | Pet food | 0.0016 | 0.0014 | 0.0050 |
| Tongue | Human food | 0.0027 | 0.0034 | 0.0054 |

Table 8: Total Weighting per coproducts for Milk-fed Hardy Lamb reared in Grazing Flat Pasture

| COPRODUCT | Destination | Milk-fed Hardy Lamb/grazing Flat Pasture | | |
| --- | --- | --- | --- | --- |
|  |  | **Biophysical Cumulative Share** | **Mass Cumulative Share** | **Economic Cumulative Share** |
| Blood | PAP C3 | 0.0032 | 0.0066 | 0.0000 |
| Blood | Spreading/Compost | 0.0000 | 0.0000 | 0.0000 |
| Bones | PAP C3 | 0.0196 | 0.0409 | 0.0000 |
| Brain | Human food | 0.0004 | 0.0011 | 0.0000 |
| Contents of the intestines | Spreading/Compost | 0.0000 | 0.0000 | 0.0000 |
| Dead individuals | C1-C2 for disposal | 0.0000 | 0.0000 | 0.0000 |
| Downgraded skin | PAP C3 | 0.0251 | 0.0270 | 0.0000 |
| Fat | PAP C3 | 0.0272 | 0.0670 | 0.0000 |
| Floatation fat | C1-C2 for disposal | 0.0000 | 0.0000 | 0.0000 |
| Meat | Human food | 0.3273 | 0.4838 | 0.9225 |
| Other spa c1 | C1-C2 for disposal | 0.0000 | 0.0000 | 0.0000 |
| Other spa c3 | PAP C3 | 0.1189 | 0.1361 | 0.0000 |
| Pluck (liver, heart, trachea) | Human food | 0.0581 | 0.0120 | 0.0133 |
| Pluck (liver, heart, trachea) | Pet food | 0.0068 | 0.0014 | 0.0015 |
| Rumen and reticulum | Human food | 0.0012 | 0.0002 | 0.0000 |
| Rumen and reticulum | Pet food | 0.0260 | 0.0055 | 0.0000 |
| Sanitary seizures | C1-C2 for disposal | 0.0000 | 0.0000 | 0.0000 |
| Screening waste | C1-C2 for disposal | 0.0000 | 0.0000 | 0.0000 |
| Sifting waste | C1-C2 for disposal | 0.0000 | 0.0000 | 0.0000 |
| Skin | Skin tannery C3 | 0.2256 | 0.1835 | 0.0462 |
| Small intestine | C1-C2 for disposal | 0.0000 | 0.0000 | 0.0000 |
| Small intestine | Human food | 0.1319 | 0.0244 | 0.0000 |
| Small intestine | PAP C3 | 0.0227 | 0.0041 | 0.0000 |
| Stercoral matter | Spreading/Compost | 0.0000 | 0.0000 | 0.0000 |
| Thymus | Human food | 0.0019 | 0.0016 | 0.0059 |
| Thymus | Pet food | 0.0016 | 0.0014 | 0.0050 |
| Tongue | Human food | 0.0027 | 0.0034 | 0.0054 |

Table 9: Total Weighting per coproducts for Milk-fed Hardy Lamb reared in Housed Ewes

| COPRODUCT | Destination | Milk-fed Hardy Lamb/Housed Ewes | | |
| --- | --- | --- | --- | --- |
|  |  | **Biophysical Cumulative Share** | **Mass Cumulative Share** | **Economic Cumulative Share** |
| Blood | PAP C3 | 0.0032 | 0.0066 | 0.0000 |
| Blood | Spreading/Compost | 0.0000 | 0.0000 | 0.0000 |
| Bones | PAP C3 | 0.0196 | 0.0409 | 0.0000 |
| Brain | Human food | 0.0004 | 0.0011 | 0.0000 |
| Contents of the intestines | Spreading/Compost | 0.0000 | 0.0000 | 0.0000 |
| Dead individuals | C1-C2 for disposal | 0.0000 | 0.0000 | 0.0000 |
| Downgraded skin | PAP C3 | 0.0251 | 0.0270 | 0.0000 |
| Fat | PAP C3 | 0.0272 | 0.0670 | 0.0000 |
| Floatation fat | C1-C2 for disposal | 0.0000 | 0.0000 | 0.0000 |
| Meat | Human food | 0.3273 | 0.4838 | 0.9225 |
| Other spa c1 | C1-C2 for disposal | 0.0000 | 0.0000 | 0.0000 |
| Other spa c3 | PAP C3 | 0.1189 | 0.1361 | 0.0000 |
| Pluck (liver, heart, trachea) | Human food | 0.0581 | 0.0120 | 0.0133 |
| Pluck (liver, heart, trachea) | Pet food | 0.0068 | 0.0014 | 0.0015 |
| Rumen and reticulum | Human food | 0.0012 | 0.0002 | 0.0000 |
| Rumen and reticulum | Pet food | 0.0260 | 0.0055 | 0.0000 |
| Sanitary seizures | C1-C2 for disposal | 0.0000 | 0.0000 | 0.0000 |
| Screening waste | C1-C2 for disposal | 0.0000 | 0.0000 | 0.0000 |
| Sifting waste | C1-C2 for disposal | 0.0000 | 0.0000 | 0.0000 |
| Skin | Skin tannery C3 | 0.2256 | 0.1835 | 0.0462 |
| Small intestine | C1-C2 for disposal | 0.0000 | 0.0000 | 0.0000 |
| Small intestine | Human food | 0.1319 | 0.0244 | 0.0000 |
| Small intestine | PAP C3 | 0.0227 | 0.0041 | 0.0000 |
| Stercoral matter | Spreading/Compost | 0.0000 | 0.0000 | 0.0000 |
| Thymus | Human food | 0.0019 | 0.0016 | 0.0059 |
| Thymus | Pet food | 0.0016 | 0.0014 | 0.0050 |
| Tongue | Human food | 0.0027 | 0.0034 | 0.0054 |

Table 10: Total Weighting per coproducts for Milk-fed Hardy Lamb reared in House Fattening

| COPRODUCT | Destination | Milk-fed Hardy Lamb/House Fattening | | |
| --- | --- | --- | --- | --- |
|  |  | **Biophysical Cumulative Share** | **Mass Cumulative Share** | **Economic Cumulative Share** |
| Blood | PAP C3 | 0.0032 | 0.0066 | 0.0000 |
| Blood | Spreading/Compost | 0.0000 | 0.0000 | 0.0000 |
| Bones | PAP C3 | 0.0196 | 0.0409 | 0.0000 |
| Brain | Human food | 0.0004 | 0.0011 | 0.0000 |
| Contents of the intestines | Spreading/Compost | 0.0000 | 0.0000 | 0.0000 |
| Dead individuals | C1-C2 for disposal | 0.0000 | 0.0000 | 0.0000 |
| Downgraded skin | PAP C3 | 0.0251 | 0.0270 | 0.0000 |
| Fat | PAP C3 | 0.0272 | 0.0670 | 0.0000 |
| Floatation fat | C1-C2 for disposal | 0.0000 | 0.0000 | 0.0000 |
| Meat | Human food | 0.3273 | 0.4838 | 0.9225 |
| Other spa c1 | C1-C2 for disposal | 0.0000 | 0.0000 | 0.0000 |
| Other spa c3 | PAP C3 | 0.1189 | 0.1361 | 0.0000 |
| Pluck (liver, heart, trachea) | Human food | 0.0581 | 0.0120 | 0.0133 |
| Pluck (liver, heart, trachea) | Pet food | 0.0068 | 0.0014 | 0.0015 |
| Rumen and reticulum | Human food | 0.0012 | 0.0002 | 0.0000 |
| Rumen and reticulum | Pet food | 0.0260 | 0.0055 | 0.0000 |
| Sanitary seizures | C1-C2 for disposal | 0.0000 | 0.0000 | 0.0000 |
| Screening waste | C1-C2 for disposal | 0.0000 | 0.0000 | 0.0000 |
| Sifting waste | C1-C2 for disposal | 0.0000 | 0.0000 | 0.0000 |
| Skin | Skin tannery C3 | 0.2256 | 0.1835 | 0.0462 |
| Small intestine | C1-C2 for disposal | 0.0000 | 0.0000 | 0.0000 |
| Small intestine | Human food | 0.1319 | 0.0244 | 0.0000 |
| Small intestine | PAP C3 | 0.0227 | 0.0041 | 0.0000 |
| Stercoral matter | Spreading/Compost | 0.0000 | 0.0000 | 0.0000 |
| Thymus | Human food | 0.0019 | 0.0016 | 0.0059 |
| Thymus | Pet food | 0.0016 | 0.0014 | 0.0050 |
| Tongue | Human food | 0.0027 | 0.0034 | 0.0054 |

Table 11: Total Weighting per coproducts for Milk-fed Heavy Lamb reared in Grazing Flat Pasture

| COPRODUCT | Destination | Milk-fed Heavy Lamb/grazing Flat Pasture | | |
| --- | --- | --- | --- | --- |
|  |  | **Biophysical Cumulative Share** | **Mass Cumulative Share** | **Economic Cumulative Share** |
| Blood | PAP C3 | 0.0032 | 0.0066 | 0.0000 |
| Blood | Spreading/Compost | 0.0000 | 0.0000 | 0.0000 |
| Bones | PAP C3 | 0.0195 | 0.0409 | 0.0000 |
| Brain | Human food | 0.0004 | 0.0011 | 0.0000 |
| Contents of the intestines | Spreading/Compost | 0.0000 | 0.0000 | 0.0000 |
| Dead individuals | C1-C2 for disposal | 0.0000 | 0.0000 | 0.0000 |
| Downgraded skin | PAP C3 | 0.0251 | 0.0270 | 0.0000 |
| Fat | PAP C3 | 0.0273 | 0.0670 | 0.0000 |
| Floatation fat | C1-C2 for disposal | 0.0000 | 0.0000 | 0.0000 |
| Meat | Human food | 0.3269 | 0.4838 | 0.9225 |
| Other spa c1 | C1-C2 for disposal | 0.0000 | 0.0000 | 0.0000 |
| Other spa c3 | PAP C3 | 0.1188 | 0.1361 | 0.0000 |
| Pluck (liver, heart, trachea) | Human food | 0.0582 | 0.0120 | 0.0133 |
| Pluck (liver, heart, trachea) | Pet food | 0.0068 | 0.0014 | 0.0015 |
| Rumen and reticulum | Human food | 0.0012 | 0.0002 | 0.0000 |
| Rumen and reticulum | Pet food | 0.0261 | 0.0055 | 0.0000 |
| Sanitary seizures | C1-C2 for disposal | 0.0000 | 0.0000 | 0.0000 |
| Screening waste | C1-C2 for disposal | 0.0000 | 0.0000 | 0.0000 |
| Sifting waste | C1-C2 for disposal | 0.0000 | 0.0000 | 0.0000 |
| Skin | Skin tannery C3 | 0.2255 | 0.1835 | 0.0462 |
| Small intestine | C1-C2 for disposal | 0.0000 | 0.0000 | 0.0000 |
| Small intestine | Human food | 0.1322 | 0.0244 | 0.0000 |
| Small intestine | PAP C3 | 0.0227 | 0.0041 | 0.0000 |
| Stercoral matter | Spreading/Compost | 0.0000 | 0.0000 | 0.0000 |
| Thymus | Human food | 0.0019 | 0.0016 | 0.0059 |
| Thymus | Pet food | 0.0016 | 0.0014 | 0.0050 |
| Tongue | Human food | 0.0027 | 0.0034 | 0.0054 |

Table 12: Total Weighting per coproducts for Milk-fed Heavy Lamb reared in Housed Ewes

| COPRODUCT | Destination | Milk-fed Heavy Lamb/Housed Ewes | | |
| --- | --- | --- | --- | --- |
|  |  | **Biophysical Cumulative Share** | **Mass Cumulative Share** | **Economic Cumulative Share** |
| Blood | PAP C3 | 0.0032 | 0.0066 | 0.0000 |
| Blood | Spreading/Compost | 0.0000 | 0.0000 | 0.0000 |
| Bones | PAP C3 | 0.0195 | 0.0409 | 0.0000 |
| Brain | Human food | 0.0004 | 0.0011 | 0.0000 |
| Contents of the intestines | Spreading/Compost | 0.0000 | 0.0000 | 0.0000 |
| Dead individuals | C1-C2 for disposal | 0.0000 | 0.0000 | 0.0000 |
| Downgraded skin | PAP C3 | 0.0251 | 0.0270 | 0.0000 |
| Fat | PAP C3 | 0.0273 | 0.0670 | 0.0000 |
| Floatation fat | C1-C2 for disposal | 0.0000 | 0.0000 | 0.0000 |
| Meat | Human food | 0.3269 | 0.4838 | 0.9225 |
| Other spa c1 | C1-C2 for disposal | 0.0000 | 0.0000 | 0.0000 |
| Other spa c3 | PAP C3 | 0.1188 | 0.1361 | 0.0000 |
| Pluck (liver, heart, trachea) | Human food | 0.0582 | 0.0120 | 0.0133 |
| Pluck (liver, heart, trachea) | Pet food | 0.0068 | 0.0014 | 0.0015 |
| Rumen and reticulum | Human food | 0.0012 | 0.0002 | 0.0000 |
| Rumen and reticulum | Pet food | 0.0261 | 0.0055 | 0.0000 |
| Sanitary seizures | C1-C2 for disposal | 0.0000 | 0.0000 | 0.0000 |
| Screening waste | C1-C2 for disposal | 0.0000 | 0.0000 | 0.0000 |
| Sifting waste | C1-C2 for disposal | 0.0000 | 0.0000 | 0.0000 |
| Skin | Skin tannery C3 | 0.2255 | 0.1835 | 0.0462 |
| Small intestine | C1-C2 for disposal | 0.0000 | 0.0000 | 0.0000 |
| Small intestine | Human food | 0.1322 | 0.0244 | 0.0000 |
| Small intestine | PAP C3 | 0.0227 | 0.0041 | 0.0000 |
| Stercoral matter | Spreading/Compost | 0.0000 | 0.0000 | 0.0000 |
| Thymus | Human food | 0.0019 | 0.0016 | 0.0059 |
| Thymus | Pet food | 0.0016 | 0.0014 | 0.0050 |
| Tongue | Human food | 0.0027 | 0.0034 | 0.0054 |

Table 13: Total Weighting per coproducts for Milk-fed Heavy Lamb in House Fattening

| COPRODUCT | Destination | Milk-fed Heavy Lamb/House Fattening | | |
| --- | --- | --- | --- | --- |
|  |  | **Biophysical Cumulative Share** | **Mass Cumulative Share** | **Economic Cumulative Share** |
| Blood | PAP C3 | 0.0032 | 0.0066 | 0.0000 |
| Blood | Spreading/Compost | 0.0000 | 0.0000 | 0.0000 |
| Bones | PAP C3 | 0.0195 | 0.0409 | 0.0000 |
| Brain | Human food | 0.0004 | 0.0011 | 0.0000 |
| Contents of the intestines | Spreading/Compost | 0.0000 | 0.0000 | 0.0000 |
| Dead individuals | C1-C2 for disposal | 0.0000 | 0.0000 | 0.0000 |
| Downgraded skin | PAP C3 | 0.0251 | 0.0270 | 0.0000 |
| Fat | PAP C3 | 0.0273 | 0.0670 | 0.0000 |
| Floatation fat | C1-C2 for disposal | 0.0000 | 0.0000 | 0.0000 |
| Meat | Human food | 0.3269 | 0.4838 | 0.9225 |
| Other spa c1 | C1-C2 for disposal | 0.0000 | 0.0000 | 0.0000 |
| Other spa c3 | PAP C3 | 0.1188 | 0.1361 | 0.0000 |
| Pluck (liver, heart, trachea) | Human food | 0.0582 | 0.0120 | 0.0133 |
| Pluck (liver, heart, trachea) | Pet food | 0.0068 | 0.0014 | 0.0015 |
| Rumen and reticulum | Human food | 0.0012 | 0.0002 | 0.0000 |
| Rumen and reticulum | Pet food | 0.0261 | 0.0055 | 0.0000 |
| Sanitary seizures | C1-C2 for disposal | 0.0000 | 0.0000 | 0.0000 |
| Screening waste | C1-C2 for disposal | 0.0000 | 0.0000 | 0.0000 |
| Sifting waste | C1-C2 for disposal | 0.0000 | 0.0000 | 0.0000 |
| Skin | Skin tannery C3 | 0.2255 | 0.1835 | 0.0462 |
| Small intestine | C1-C2 for disposal | 0.0000 | 0.0000 | 0.0000 |
| Small intestine | Human food | 0.1322 | 0.0244 | 0.0000 |
| Small intestine | PAP C3 | 0.0227 | 0.0041 | 0.0000 |
| Stercoral matter | Spreading/Compost | 0.0000 | 0.0000 | 0.0000 |
| Thymus | Human food | 0.0019 | 0.0016 | 0.0059 |
| Thymus | Pet food | 0.0016 | 0.0014 | 0.0050 |
| Tongue | Human food | 0.0027 | 0.0034 | 0.0054 |

Table 14: Total Weighting per coproducts for Milk Lamb reared in House Fattening

| COPRODUCT | Destination | Milk Lamb/House Fattening | | |
| --- | --- | --- | --- | --- |
|  |  | **Biophysical Cumulative Share** | **Mass Cumulative Share** | **Economic Cumulative Share** |
| Blood | PAP C3 | 0.0032 | 0.0066 | 0.0000 |
| Blood | Spreading/Compost | 0.0000 | 0.0000 | 0.0000 |
| Bones | PAP C3 | 0.0195 | 0.0409 | 0.0000 |
| Brain | Human food | 0.0004 | 0.0011 | 0.0000 |
| Contents of the intestines | Spreading/Compost | 0.0000 | 0.0000 | 0.0000 |
| Dead individuals | C1-C2 for disposal | 0.0000 | 0.0000 | 0.0000 |
| Downgraded skin | PAP C3 | 0.0251 | 0.0270 | 0.0000 |
| Fat | PAP C3 | 0.0273 | 0.0670 | 0.0000 |
| Floatation fat | C1-C2 for disposal | 0.0000 | 0.0000 | 0.0000 |
| Meat | Human food | 0.3269 | 0.4838 | 0.9225 |
| Other spa c1 | C1-C2 for disposal | 0.0000 | 0.0000 | 0.0000 |
| Other spa c3 | PAP C3 | 0.1188 | 0.1361 | 0.0000 |
| Pluck (liver, heart, trachea) | Human food | 0.0582 | 0.0120 | 0.0133 |
| Pluck (liver, heart, trachea) | Pet food | 0.0068 | 0.0014 | 0.0015 |
| Rumen and reticulum | Human food | 0.0012 | 0.0002 | 0.0000 |
| Rumen and reticulum | Pet food | 0.0261 | 0.0055 | 0.0000 |
| Sanitary seizures | C1-C2 for disposal | 0.0000 | 0.0000 | 0.0000 |
| Screening waste | C1-C2 for disposal | 0.0000 | 0.0000 | 0.0000 |
| Sifting waste | C1-C2 for disposal | 0.0000 | 0.0000 | 0.0000 |
| Skin | Skin tannery C3 | 0.2255 | 0.1835 | 0.0462 |
| Small intestine | C1-C2 for disposal | 0.0000 | 0.0000 | 0.0000 |
| Small intestine | Human food | 0.1322 | 0.0244 | 0.0000 |
| Small intestine | PAP C3 | 0.0227 | 0.0041 | 0.0000 |
| Stercoral matter | Spreading/Compost | 0.0000 | 0.0000 | 0.0000 |
| Thymus | Human food | 0.0019 | 0.0016 | 0.0059 |
| Thymus | Pet food | 0.0016 | 0.0014 | 0.0050 |
| Tongue | Human food | 0.0027 | 0.0034 | 0.0054 |
