# Supplementary material for: Allocation factors for meat coproducts: Dataset to perform life cycle assessment at slaughterhouse
Source: Data Brief. 2020 Nov 23;33:106558. doi: 10.1016/j.dib.2020.106558 (PMC7718151; doi:10.1016/j.dib.2020.106558)
Supplement: Supplementary file 9 [file mmc9.docx]

Table 1: Total weighting per Destination for Average Ovine reared in Grazing Flat Pasture

| destination | Average/Ovine/grazing Flat Pasture | | |
| --- | --- | --- | --- |
|  | **Biophysical Partition** | **Mass Partition** | **Economic Partition** |
| Pet Food | 0,0345 | 0,0083 | 0,0065 |
| PAP C3 | 0,2166 | 0,2817 | 0 |
| Gelatin C3 | 0 | 0 | 0 |
| C1-C2 for disposal | 0 | 0 | 0 |
| Skin tannery C3 | 0,2255 | 0,1835 | 0,0462 |
| Human food | 0,5235 | 0,5265 | 0,9471 |
| Fat and greaves C3 | 0 | 0 | 0 |
| Spreading/Compost | 0 | 0 | 0 |

Table 2: Total weighting per Destination for Average Ovine reared in Grazing Hilly Pasture

| destination | Average/Ovine/grazing Hilly Pasture | | |
| --- | --- | --- | --- |
|  | **Biophysical Partition** | **Mass Partition** | **Economic Partition** |
| Pet Food | 0,0345 | 0,0083 | 0,0065 |
| PAP C3 | 0,2166 | 0,2817 | 0 |
| Gelatin C3 | 0 | 0 | 0 |
| C1-C2 for disposal | 0 | 0 | 0 |
| Skin tannery C3 | 0,2255 | 0,1835 | 0,0462 |
| Human food | 0,5235 | 0,5265 | 0,9471 |
| Fat and greaves C3 | 0 | 0 | 0 |
| Spreading/Compost | 0 | 0 | 0 |

Table 3: Total weighting per Destination for Average Ovine reared in Housed Ewes

| destination | Average/Ovine/grazing Flat Pasture | | | o Ewes |
| --- | --- | --- | --- | --- |
|  | **Biophysical Partition** | **Mass Partition** | **Economic Partition** |  |
| Pet Food | 0,0345 | 0,0083 | 0,0065 |  |
| PAP C3 | 0,2166 | 0,2817 | 0 |  |
| Gelatin C3 | 0 | 0 | 0 |  |
| C1-C2 for disposal | 0 | 0 | 0 |  |
| Skin tannery C3 | 0,2255 | 0,1835 | 0,0462 |  |
| Human food | 0,5235 | 0,5265 | 0,9471 |  |
| Fat and greaves C3 | 0 | 0 | 0 |  |
| Spreading/Compost | 0 | 0 | 0 |  |

Table 4: Total weighting per Destination for Average Ovine reared in House Fattening

| destination | Average/Ovine/House Fattening | | |
| --- | --- | --- | --- |
|  | **Biophysical Partition** | **Mass Partition** | **Economic Partition** |
| Pet Food | 0,0344 | 0,0083 | 0,0065 |
| PAP C3 | 0,2166 | 0,2817 | 0 |
| Gelatin C3 | 0 | 0 | 0 |
| C1-C2 for disposal | 0 | 0 | 0 |
| Skin tannery C3 | 0,2255 | 0,1835 | 0,0462 |
| Human food | 0,5235 | 0,5265 | 0,9471 |
| Fat and greaves C3 | 0 | 0 | 0 |
| Spreading/Compost | 0 | 0 | 0 |

Table 5: Total weighting per Destination for Grass-fed Heavy Lamb reared in Grazing Flat Pasture

| destination | Grass-fed Heavy Lamb/grazing Flat Pasture | | |
| --- | --- | --- | --- |
|  | **Biophysical Partition** | **Mass Partition** | **Economic Partition** |
| Pet Food | 0,0345 | 0,0083 | 0,0065 |
| PAP C3 | 0,2165 | 0,2817 | 0 |
| Gelatin C3 | 0 | 0 | 0 |
| C1-C2 for disposal | 0 | 0 | 0 |
| Skin tannery C3 | 0,2254 | 0,1835 | 0,0462 |
| Human food | 0,5237 | 0,5265 | 0,9471 |
| Fat and greaves C3 | 0 | 0 | 0 |
| Spreading/Compost | 0 | 0 | 0 |

Table 6: Total weighting per Destination for Grass-fed Heavy Lamb reared in Grazing Hilly Pasture

| destination | Grass-fed Heavy Lamb/grazing Hilly Pasture | | |
| --- | --- | --- | --- |
|  | **Biophysical Partition** | **Mass Partition** | **Economic Partition** |
| Pet Food | 0,0345 | 0,0083 | 0,0065 |
| PAP C3 | 0,2165 | 0,2817 | 0 |
| Gelatin C3 | 0 | 0 | 0 |
| C1-C2 for disposal | 0 | 0 | 0 |
| Skin tannery C3 | 0,2254 | 0,1835 | 0,0462 |
| Human food | 0,5237 | 0,5265 | 0,9471 |
| Fat and greaves C3 | 0 | 0 | 0 |
| Spreading/Compost | 0 | 0 | 0 |

Table 7: Total weighting per Destination for Grass-fed Heavy Lamb reared in Housed Ewes

| destination | Grass-fed Heavy Lamb/Housed ewes | | |
| --- | --- | --- | --- |
|  | **Biophysical Partition** | **Mass Partition** | **Economic Partition** |
| Pet Food | 0,0345 | 0,0083 | 0,0065 |
| PAP C3 | 0,2165 | 0,2817 | 0 |
| Gelatin C3 | 0 | 0 | 0 |
| C1-C2 for disposal | 0 | 0 | 0 |
| Skin tannery C3 | 0,2254 | 0,1835 | 0,0462 |
| Human food | 0,5237 | 0,5265 | 0,9471 |
| Fat and greaves C3 | 0 | 0 | 0 |
| Spreading/Compost | 0 | 0 | 0 |

Table 8: Total weighting per Destination for Milk-fed Hardy Lamb reared in Grazing Flat Pasture

| destination | Milk-fed Hardy Lamb/grazing Flat Pasture | | |
| --- | --- | --- | --- |
|  | **Biophysical Partition** | **Mass Partition** | **Economic Partition** |
| Pet Food | 0,0344 | 0,0083 | 0,0065 |
| PAP C3 | 0,2167 | 0,2817 | 0 |
| Gelatin C3 | 0 | 0 | 0 |
| C1-C2 for disposal | 0 | 0 | 0 |
| Skin tannery C3 | 0,2256 | 0,1835 | 0,0462 |
| Human food | 0,5235 | 0,5265 | 0,9471 |
| Fat and greaves C3 | 0 | 0 | 0 |
| Spreading/Compost | 0 | 0 | 0 |

Table 9: Total weighting per Destination for Milk-fed Hardy Lamb reared in Housed Ewes

| destination | Milk-fed Hardy Lamb/Housed Ewes | | |
| --- | --- | --- | --- |
|  | **Biophysical Partition** | **Mass Partition** | **Economic Partition** |
| Pet Food | 0,0344 | 0,0083 | 0,0065 |
| PAP C3 | 0,2167 | 0,2817 | 0 |
| Gelatin C3 | 0 | 0 | 0 |
| C1-C2 for disposal | 0 | 0 | 0 |
| Skin tannery C3 | 0,2256 | 0,1835 | 0,0462 |
| Human food | 0,5235 | 0,5265 | 0,9471 |
| Fat and greaves C3 | 0 | 0 | 0 |
| Spreading/Compost | 0 | 0 | 0 |

Table 10: Total weighting per Destination for Milk-fed Hardy Lamb reared in House Fattening

| destination | Milk-fed Hardy Lamb/House Fattening | | |
| --- | --- | --- | --- |
|  | **Biophysical Partition** | **Mass Partition** | **Economic Partition** |
| Pet Food | 0,0344 | 0,0083 | 0,0065 |
| PAP C3 | 0,2167 | 0,2817 | 0 |
| Gelatin C3 | 0 | 0 | 0 |
| C1-C2 for disposal | 0 | 0 | 0 |
| Skin tannery C3 | 0,2256 | 0,1835 | 0,0462 |
| Human food | 0,5235 | 0,5265 | 0,9471 |
| Fat and greaves C3 | 0 | 0 | 0 |
| Spreading/Compost | 0 | 0 | 0 |

Table 11: Total weighting per Destination for Milk-fed Heavy Lamb reared in Grazing Flat Pasture

| destination | Milk-fed Heavy Lamb/grazing Flat Pasture | | |
| --- | --- | --- | --- |
|  | **Biophysical Partition** | **Mass Partition** | **Economic Partition** |
| Pet Food | 0,0345 | 0,0083 | 0,0065 |
| PAP C3 | 0,2166 | 0,2817 | 0 |
| Gelatin C3 | 0 | 0 | 0 |
| C1-C2 for disposal | 0 | 0 | 0 |
| Skin tannery C3 | 0,2255 | 0,1835 | 0,0462 |
| Human food | 0,5235 | 0,5265 | 0,9471 |
| Fat and greaves C3 | 0 | 0 | 0 |
| Spreading/Compost | 0 | 0 | 0 |

Table 12: Total weighting per Destination for Milk-fed Heavy Lamb reared in Housed Ewes

| destination | Milk-fed Heavy Lamb/Housed Ewes | | |
| --- | --- | --- | --- |
|  | **Biophysical Partition** | **Mass Partition** | **Economic Partition** |
| Pet Food | 0,0345 | 0,0083 | 0,0065 |
| PAP C3 | 0,2166 | 0,2817 | 0 |
| Gelatin C3 | 0 | 0 | 0 |
| C1-C2 for disposal | 0 | 0 | 0 |
| Skin tannery C3 | 0,2255 | 0,1835 | 0,0462 |
| Human food | 0,5235 | 0,5265 | 0,9471 |
| Fat and greaves C3 | 0 | 0 | 0 |
| Spreading/Compost | 0 | 0 | 0 |

Table 13: Total weighting per Destination for Milk-fed Heavy Lamb in House Fattening

| destination | Milk-fed Heavy Lamb/House Fattening | | | Milk-fed Heavy Lamb/House Fattening |
| --- | --- | --- | --- | --- |
|  | **Biophysical Partition** | **Mass Partition** | **Economic Partition** |  |
| Pet Food | 0,0345 | 0,0083 | 0,0065 |  |
| PAP C3 | 0,2166 | 0,2817 | 0 |  |
| Gelatin C3 | 0 | 0 | 0 |  |
| C1-C2 for disposal | 0 | 0 | 0 |  |
| Skin tannery C3 | 0,2255 | 0,1835 | 0,0462 |  |
| Human food | 0,5235 | 0,5265 | 0,9471 |  |
| Fat and greaves C3 | 0 | 0 | 0 |  |
| Spreading/Compost | 0 | 0 | 0 |  |

Table 14: Total weighting per Destination for Milk Lamb reared in House Fattening

| destination | Milk Lamb/House Fattening | | | M Lamb/House Fattening |
| --- | --- | --- | --- | --- |
|  | **Biophysical Partition** | **Mass Partition** | **Economic Partition** |  |
| Pet Food | 0,0345 | 0,0083 | 0,0065 |  |
| PAP C3 | 0,2166 | 0,2817 | 0 |  |
| Gelatin C3 | 0 | 0 | 0 |  |
| C1-C2 for disposal | 0 | 0 | 0 |  |
| Skin tannery C3 | 0,2255 | 0,1835 | 0,0462 |  |
| Human food | 0,5235 | 0,5265 | 0,9471 |  |
| Fat and greaves C3 | 0 | 0 | 0 |  |
| Spreading/Compost | 0 | 0 | 0 |  |
