# Supplementary material for: Allocation factors for meat coproducts: Dataset to perform life cycle assessment at slaughterhouse
Source: Data Brief. 2020 Nov 23;33:106558. doi: 10.1016/j.dib.2020.106558 (PMC7718151; doi:10.1016/j.dib.2020.106558)
Supplement: Supplementary file 14 [file mmc14.docx]

Table 1: Sensitivity analysis of coproducts total weightings depending on Gompertz Coefficient

|  |  | Gompertz coefficient | | | | | | |
| --- | --- | --- | --- | --- | --- | --- | --- | --- |
| Co-product | **Destination** | **0,003** | **0,006** | **0,009** | **0,012** | **0,018** | | **0,024** |
| Abomasum | Human food | 0,0182 | 0,0165 | 0,0151 | 0,0140 | 0,0121 | 0,0108 | |
| Abomasum fat | Fat and greaves C3 | 0,0011 | 0,0011 | 0,0011 | 0,0011 | 0,0012 | | 0,0012 |
| Aponeurosis | Human food | 0,0289 | 0,0281 | 0,0274 | 0,0269 | 0,0260 | | 0,0254 |
| Bile | PAP C3 | 0,0000 | 0,0000 | 0,0000 | 0,0000 | 0,0000 | | 0,0000 |
| Blood | PAP C3 | 0,0025 | 0,0024 | 0,0024 | 0,0024 | 0,0023 | | 0,0022 |
| Blood | Pet food | 0,0178 | 0,0174 | 0,0170 | 0,0167 | 0,0163 | | 0,0159 |
| Bones | Gelatin C3 | 0,0468 | 0,0492 | 0,0511 | 0,0527 | 0,0552 | | 0,0571 |
| Bones of head, brain, eyes and teeth | C1-C2 for disposal | 0,0000 | 0,0000 | 0,0000 | 0,0000 | 0,0000 | | 0,0000 |
| Cheek | Human food | 0,0010 | 0,0010 | 0,0009 | 0,0009 | 0,0009 | | 0,0009 |
| Cheek | Human food | 0,0020 | 0,0019 | 0,0019 | 0,0019 | 0,0018 | | 0,0018 |
| Cheek trimmings | Pet food | 0,0005 | 0,0005 | 0,0005 | 0,0005 | 0,0005 | | 0,0005 |
| Chops | Pet food | 0,0020 | 0,0020 | 0,0019 | 0,0019 | 0,0018 | | 0,0018 |
| Contents of intestines | Spreading/Compost | 0,0000 | 0,0000 | 0,0000 | 0,0000 | 0,0000 | | 0,0000 |
| Contents of the rumen | Spreading/Compost | 0,0000 | 0,0000 | 0,0000 | 0,0000 | 0,0000 | | 0,0000 |
| Ears | PAP C3 | 0,0008 | 0,0008 | 0,0008 | 0,0009 | 0,0009 | | 0,0009 |
| Esophagus | Pet food | 0,0009 | 0,0009 | 0,0009 | 0,0009 | 0,0009 | | 0,0009 |
| Fat | Fat and greaves C3 | 0,0415 | 0,0621 | 0,0787 | 0,0926 | 0,1141 | | 0,1302 |
| Fat around heart | Fat and greaves C3 | 0,0012 | 0,0019 | 0,0024 | 0,0028 | 0,0034 | | 0,0039 |
| Fat in the kidney | Fat and greaves C3 | 0,0322 | 0,0484 | 0,0615 | 0,0724 | 0,0893 | | 0,1020 |
| Feet (without hooves) | Gelatin C3 | 0,0121 | 0,0120 | 0,0119 | 0,0118 | 0,0117 | | 0,0116 |
| Floatation fat | Spreading/Compost | 0,0000 | 0,0000 | 0,0000 | 0,0000 | 0,0000 | | 0,0000 |
| Forehead | C1-C2 for disposal | 0,0000 | 0,0000 | 0,0000 | 0,0000 | 0,0000 | | 0,0000 |
| Forelock | PAP C3 | 0,0030 | 0,0029 | 0,0028 | 0,0027 | 0,0026 | | 0,0025 |
| Gallbladder | Pet food | 0,0003 | 0,0003 | 0,0003 | 0,0003 | 0,0003 | | 0,0003 |
| Head trimmings | Pet food | 0,0027 | 0,0026 | 0,0026 | 0,0025 | 0,0025 | | 0,0024 |
| Heart | Human food | 0,0030 | 0,0029 | 0,0029 | 0,0028 | 0,0028 | | 0,0027 |
| Heart trimmings | Pet food | 0,0004 | 0,0003 | 0,0003 | 0,0003 | 0,0003 | | 0,0003 |
| Hide | Skin tannery C3 | 0,0695 | 0,0675 | 0,0660 | 0,0646 | 0,0626 | | 0,0610 |
| Hooves | PAP C3 | 0,0082 | 0,0079 | 0,0077 | 0,0075 | 0,0072 | | 0,0070 |
| Horns | PAP C3 | 0,0020 | 0,0019 | 0,0018 | 0,0018 | 0,0017 | | 0,0017 |
| Kidney | Human food | 0,0019 | 0,0019 | 0,0019 | 0,0018 | 0,0018 | | 0,0017 |
| Large intestine | C1-C2 for disposal | 0,0000 | 0,0000 | 0,0000 | 0,0000 | 0,0000 | | 0,0000 |
| Liver | Human food | 0,0658 | 0,0602 | 0,0556 | 0,0518 | 0,0459 | | 0,0415 |
| Liver trimmings | Pet food | 0,0077 | 0,0070 | 0,0065 | 0,0060 | 0,0053 | | 0,0048 |
| Lower jaw | PAP C3 | 0,0028 | 0,0030 | 0,0031 | 0,0032 | 0,0034 | | 0,0035 |
| Lungs | Pet food | 0,0083 | 0,0080 | 0,0078 | 0,0076 | 0,0074 | | 0,0072 |
| Mask | Skin tannery C3 | 0,0047 | 0,0045 | 0,0044 | 0,0043 | 0,0042 | | 0,0041 |
| Mesenteric fat | C1-C2 for disposal | 0,0000 | 0,0000 | 0,0000 | 0,0000 | 0,0000 | | 0,0000 |
| Muscle | Human food | 0,3430 | 0,3386 | 0,3350 | 0,3321 | 0,3274 | | 0,3239 |
| Muzzle | Human food | 0,0024 | 0,0023 | 0,0023 | 0,0022 | 0,0022 | | 0,0021 |
| Omasum | Human food | 0,0164 | 0,0149 | 0,0136 | 0,0126 | 0,0109 | | 0,0097 |
| Omasum fat | Fat and greaves C3 | 0,0029 | 0,0030 | 0,0031 | 0,0031 | 0,0032 | | 0,0033 |
| Rumen and forestomach | Human food | 0,0838 | 0,0759 | 0,0695 | 0,0642 | 0,0559 | | 0,0497 |
| Rumen fat | Fat and greaves C3 | 0,0048 | 0,0049 | 0,0050 | 0,0051 | 0,0053 | | 0,0054 |
| Sanitary seizures | C1-C2 for disposal | 0,0000 | 0,0000 | 0,0000 | 0,0000 | 0,0000 | | 0,0000 |
| Screening and sifting wastes | C1-C2 for disposal | 0,0000 | 0,0000 | 0,0000 | 0,0000 | 0,0000 | | 0,0000 |
| Small intestine | PAP C3 | 0,0966 | 0,0875 | 0,0801 | 0,0740 | 0,0644 | | 0,0573 |
| Spinal cord | C1-C2 for disposal | 0,0000 | 0,0000 | 0,0000 | 0,0000 | 0,0000 | | 0,0000 |
| Spinal cord waste | C1-C2 for disposal | 0,0000 | 0,0000 | 0,0000 | 0,0000 | 0,0000 | | 0,0000 |
| Spine | C1-C2 for disposal | 0,0000 | 0,0000 | 0,0000 | 0,0000 | 0,0000 | | 0,0000 |
| Spleen | Pet food | 0,0020 | 0,0019 | 0,0019 | 0,0019 | 0,0019 | | 0,0018 |
| Stillborn | PAP C3 | 0,0506 | 0,0458 | 0,0418 | 0,0386 | 0,0335 | | 0,0297 |
| Tallow | Fat and greaves C3 | 0,0002 | 0,0003 | 0,0003 | 0,0004 | 0,0005 | | 0,0006 |
| Tongue | Human food | 0,0023 | 0,0023 | 0,0024 | 0,0024 | 0,0025 | | 0,0025 |
| Tonsil | C1-C2 for disposal | 0,0000 | 0,0000 | 0,0000 | 0,0000 | 0,0000 | | 0,0000 |
| Trachea | Pet food | 0,0017 | 0,0017 | 0,0017 | 0,0016 | 0,0016 | | 0,0016 |
| Udder | Pet food | 0,0025 | 0,0028 | 0,0030 | 0,0032 | 0,0034 | | 0,0037 |
| Upper throat | Pet food | 0,0009 | 0,0009 | 0,0009 | 0,0009 | 0,0009 | | 0,0009 |
| Water in the rumen | Spreading/Compost | 0,0000 | 0,0000 | 0,0000 | 0,0000 | 0,0000 | | 0,0000 |

Table 2: Sensitivity analysis of destination total weightings depending on Gompertz Coefficient

|  | GOMPERTZ COEFFICIENT | | | | | |
| --- | --- | --- | --- | --- | --- | --- |
| Destination | **0.003** | **0.006** | **0.009** | **0.012** | **0.018** | **0.024** |
| Pet Food | 0,0477 | 0,0463 | 0,0453 | 0,0443 | 0,0431 | 0,0421 |
| PAP C3 | 0,1665 | 0,1522 | 0,1405 | 0,1311 | 0,116 | 0,1048 |
| Gelatin C3 | 0,0589 | 0,0612 | 0,063 | 0,0645 | 0,0669 | 0,0687 |
| C1-C2 for disposal | 0 | 0 | 0 | 0 | 0 | 0 |
| Skin tannery C3 | 0,0742 | 0,072 | 0,0704 | 0,0689 | 0,0668 | 0,0651 |
| Human food | 0,5687 | 0,5465 | 0,5285 | 0,5136 | 0,4902 | 0,4727 |
| Fat and greaves C3 | 0,0839 | 0,1217 | 0,1521 | 0,1775 | 0,217 | 0,2466 |
| Spreading/Compost | 0 | 0 | 0 | 0 | 0 | 0 |

Table 3: Sensitivity analysis of coproducts total weightings depending on Carcass Yield

|  |  | Carcass Yield | | | | | |
| --- | --- | --- | --- | --- | --- | --- | --- |
| Co-product | **Destination** | **0,50** | **0,52** | **0,54** | **0,56** | **0,58** | **0,60** |
| Abomasum | Human food | 0,0163 | 0,0157 | 0,0151 | 0,0145 | 0,0140 | 0,0135 |
| Abomasum fat | Fat and greaves C3 | 0,0013 | 0,0013 | 0,0012 | 0,0012 | 0,0011 | 0,0011 |
| Aponeurosis | Human food | 0,0227 | 0,0238 | 0,0249 | 0,0259 | 0,0269 | 0,0277 |
| Bile | PAP C3 | 0,0000 | 0,0000 | 0,0000 | 0,0000 | 0,0000 | 0,0000 |
| Blood | PAP C3 | 0,0027 | 0,0026 | 0,0025 | 0,0024 | 0,0024 | 0,0023 |
| Blood | Pet food | 0,0195 | 0,0188 | 0,0180 | 0,0173 | 0,0167 | 0,0161 |
| Bones | Gelatin C3 | 0,0443 | 0,0465 | 0,0487 | 0,0509 | 0,0527 | 0,0545 |
| Bones of head, brain, eyes and teeth | C1-C2 for disposal | 0,0000 | 0,0000 | 0,0000 | 0,0000 | 0,0000 | 0,0000 |
| Cheek | Human food | 0,0011 | 0,0010 | 0,0010 | 0,0010 | 0,0009 | 0,0009 |
| Cheek | Human food | 0,0022 | 0,0021 | 0,0020 | 0,0019 | 0,0019 | 0,0018 |
| Cheek trimmings | Pet food | 0,0006 | 0,0006 | 0,0005 | 0,0005 | 0,0005 | 0,0005 |
| Chops | Pet food | 0,0022 | 0,0021 | 0,0020 | 0,0019 | 0,0019 | 0,0018 |
| Contents of intestines | Spreading/Compost | 0,0000 | 0,0000 | 0,0000 | 0,0000 | 0,0000 | 0,0000 |
| Contents of the rumen | Spreading/Compost | 0,0000 | 0,0000 | 0,0000 | 0,0000 | 0,0000 | 0,0000 |
| Ears | PAP C3 | 0,0010 | 0,0010 | 0,0009 | 0,0009 | 0,0009 | 0,0008 |
| Esophagus | Pet food | 0,0011 | 0,0010 | 0,0010 | 0,0009 | 0,0009 | 0,0009 |
| Fat | Fat and greaves C3 | 0,0768 | 0,0808 | 0,0850 | 0,0891 | 0,0926 | 0,0960 |
| Fat around heart | Fat and greaves C3 | 0,0032 | 0,0031 | 0,0030 | 0,0029 | 0,0028 | 0,0027 |
| Fat in the kidney | Fat and greaves C3 | 0,0827 | 0,0801 | 0,0774 | 0,0747 | 0,0724 | 0,0701 |
| Feet (without hooves) | Gelatin C3 | 0,0137 | 0,0132 | 0,0127 | 0,0122 | 0,0118 | 0,0114 |
| Floatation fat | Spreading/Compost | 0,0000 | 0,0000 | 0,0000 | 0,0000 | 0,0000 | 0,0000 |
| Forehead | C1-C2 for disposal | 0,0000 | 0,0000 | 0,0000 | 0,0000 | 0,0000 | 0,0000 |
| Forelock | PAP C3 | 0,0032 | 0,0030 | 0,0029 | 0,0028 | 0,0027 | 0,0026 |
| Gallbladder | Pet food | 0,0004 | 0,0004 | 0,0004 | 0,0003 | 0,0003 | 0,0003 |
| Head trimmings | Pet food | 0,0029 | 0,0028 | 0,0027 | 0,0026 | 0,0025 | 0,0024 |
| Heart | Human food | 0,0033 | 0,0032 | 0,0030 | 0,0029 | 0,0028 | 0,0027 |
| Heart trimmings | Pet food | 0,0004 | 0,0004 | 0,0004 | 0,0003 | 0,0003 | 0,0003 |
| Hide | Skin tannery C3 | 0,0753 | 0,0725 | 0,0697 | 0,0670 | 0,0646 | 0,0624 |
| Hooves | PAP C3 | 0,0088 | 0,0084 | 0,0081 | 0,0078 | 0,0075 | 0,0072 |
| Horns | PAP C3 | 0,0021 | 0,0020 | 0,0019 | 0,0019 | 0,0018 | 0,0017 |
| Kidney | Human food | 0,0021 | 0,0020 | 0,0020 | 0,0019 | 0,0018 | 0,0018 |
| Large intestine | C1-C2 for disposal | 0,0000 | 0,0000 | 0,0000 | 0,0000 | 0,0000 | 0,0000 |
| Liver | Human food | 0,0603 | 0,0581 | 0,0559 | 0,0537 | 0,0518 | 0,0500 |
| Liver trimmings | Pet food | 0,0070 | 0,0068 | 0,0065 | 0,0062 | 0,0060 | 0,0058 |
| Lower jaw | PAP C3 | 0,0037 | 0,0036 | 0,0034 | 0,0033 | 0,0032 | 0,0031 |
| Lungs | Pet food | 0,0089 | 0,0086 | 0,0082 | 0,0079 | 0,0076 | 0,0074 |
| Mask | Skin tannery C3 | 0,0050 | 0,0049 | 0,0047 | 0,0045 | 0,0043 | 0,0042 |
| Mesenteric fat | C1-C2 for disposal | 0,0000 | 0,0000 | 0,0000 | 0,0000 | 0,0000 | 0,0000 |
| Muscle | Human food | 0,2804 | 0,2938 | 0,3074 | 0,3208 | 0,3321 | 0,3431 |
| Muzzle | Human food | 0,0026 | 0,0025 | 0,0024 | 0,0023 | 0,0022 | 0,0021 |
| Omasum | Human food | 0,0146 | 0,0141 | 0,0136 | 0,0130 | 0,0126 | 0,0121 |
| Omasum fat | Fat and greaves C3 | 0,0036 | 0,0035 | 0,0034 | 0,0033 | 0,0031 | 0,0030 |
| Rumen and forestomach | Human food | 0,0748 | 0,0720 | 0,0693 | 0,0665 | 0,0642 | 0,0619 |
| Rumen fat | Fat and greaves C3 | 0,0060 | 0,0057 | 0,0055 | 0,0053 | 0,0051 | 0,0050 |
| Sanitary seizures | C1-C2 for disposal | 0,0000 | 0,0000 | 0,0000 | 0,0000 | 0,0000 | 0,0000 |
| Screening and sifting wastes | C1-C2 for disposal | 0,0000 | 0,0000 | 0,0000 | 0,0000 | 0,0000 | 0,0000 |
| Small intestine | PAP C3 | 0,0862 | 0,0830 | 0,0798 | 0,0766 | 0,0740 | 0,0713 |
| Spinal cord | C1-C2 for disposal | 0,0000 | 0,0000 | 0,0000 | 0,0000 | 0,0000 | 0,0000 |
| Spinal cord waste | C1-C2 for disposal | 0,0000 | 0,0000 | 0,0000 | 0,0000 | 0,0000 | 0,0000 |
| Spine | C1-C2 for disposal | 0,0000 | 0,0000 | 0,0000 | 0,0000 | 0,0000 | 0,0000 |
| Spleen | Pet food | 0,0022 | 0,0021 | 0,0020 | 0,0020 | 0,0019 | 0,0018 |
| Stillborn | PAP C3 | 0,0449 | 0,0433 | 0,0416 | 0,0400 | 0,0386 | 0,0372 |
| Tallow | Fat and greaves C3 | 0,0005 | 0,0004 | 0,0004 | 0,0004 | 0,0004 | 0,0004 |
| Tongue | Human food | 0,0028 | 0,0027 | 0,0026 | 0,0025 | 0,0024 | 0,0023 |
| Tonsil | C1-C2 for disposal | 0,0000 | 0,0000 | 0,0000 | 0,0000 | 0,0000 | 0,0000 |
| Trachea | Pet food | 0,0019 | 0,0019 | 0,0018 | 0,0017 | 0,0016 | 0,0016 |
| Udder | Pet food | 0,0037 | 0,0035 | 0,0034 | 0,0033 | 0,0032 | 0,0031 |
| Upper throat | Pet food | 0,0011 | 0,0010 | 0,0010 | 0,0010 | 0,0009 | 0,0009 |
| Water in the rumen | Spreading/Compost | 0,0000 | 0,0000 | 0,0000 | 0,0000 | 0,0000 | 0,0000 |

Table 4: Sensitivity analysis of destination total weightings depending on Carcass Yield

|  | carcass yield | | | | | |
| --- | --- | --- | --- | --- | --- | --- |
| Destination | **0.50** | **0.52** | **0.54** | **0.56** | **0.58** | **0.60** |
| Pet Food | 0,0519 | 0,05 | 0,0479 | 0,0459 | 0,0443 | 0,0429 |
| PAP C3 | 0,1526 | 0,1469 | 0,1411 | 0,1357 | 0,1311 | 0,1262 |
| Gelatin C3 | 0,058 | 0,0597 | 0,0614 | 0,0631 | 0,0645 | 0,0659 |
| C1-C2 for disposal | 0 | 0 | 0 | 0 | 0 | 0 |
| Skin tannery C3 | 0,0803 | 0,0774 | 0,0744 | 0,0715 | 0,0689 | 0,0666 |
| Human food | 0,4832 | 0,491 | 0,4992 | 0,5069 | 0,5136 | 0,5199 |
| Fat and greaves C3 | 0,1741 | 0,1749 | 0,1759 | 0,1769 | 0,1775 | 0,1783 |
| Spreading/Compost | 0 | 0 | 0 | 0 | 0 | 0 |
